# Quantitative proteomic profiling of primary cancer-associated fibroblasts in oesophageal adenocarcinoma

**DOI:** 10.1038/s41416-018-0042-9

**Published:** 2018-03-29

**Authors:** Antigoni Manousopoulou, Annette Hayden, Massimiliano Mellone, Diana J. Garay-Baquero, Cory H. White, Fergus Noble, Monette Lopez, Gareth J. Thomas, Timothy J. Underwood, Spiros D. Garbis

**Affiliations:** 1 Institute for Life Sciences, University of Southampton, Southampton, UK; 2 Cancer Sciences Unit, Faculty of Medicine, University of Southampton, Southampton, UK; 3 Clinical and Experimental Sciences Unit, Faculty of Medicine, University of Southampton, Southampton, UK; 4 University Hospital Southampton NHS Foundation Trust, Southampton, UK; 5 Present Address: Merck Exploratory Science Center, Cambridge, MA USA

**Keywords:** Medical research, Oncology

## Abstract

**Background:**

Cancer-associated fibroblasts (CAFs) form the major stromal component of the tumour microenvironment (TME). The present study aimed to examine the proteomic profiles of CAFs vs. normal fibroblasts (NOFs) from patients with oesophageal adenocarcinoma to gain insight into their pro-oncogenic phenotype.

**Methods:**

CAFs/NOFs from four patients were sub-cultured and analysed using quantitative proteomics. Differentially expressed proteins (DEPs) were subjected to bioinformatics and compared with published proteomics and transcriptomics  datasets.

**Results:**

Principal component analysis of all profiled proteins showed that CAFs had high heterogeneity and clustered separately from NOFs. Bioinformatics interrogation of the DEPs demonstrated inhibition of adhesion of epithelial cells, adhesion of connective tissue cells and cell death of fibroblast cell lines in CAFs vs. NOFs (*p* < 0.0001). KEGG pathway analysis showed a significant enrichment of the insulin-signalling pathway (*p* = 0.03). Gene ontology terms related with myofibroblast phenotype, metabolism, cell adhesion/migration, hypoxia/oxidative stress, angiogenesis, immune/inflammatory response were enriched in CAFs vs. NOFs. Nestin, a stem-cell marker up-regulated in CAFs vs. NOFs, was confirmed to be expressed in the TME with immunohistochemistry.

**Conclusions:**

The identified pathways and participating proteins may provide novel insight on the tumour-promoting properties of CAFs and unravel novel adjuvant therapeutic targets in the TME.

## Introduction

Oesophageal cancer represents a significant global health burden with 395,000 deaths in 2010, an increase of nearly 15% from 1990.^1^ Oesophageal adenocarcinoma (OAC) is the predominant histological subtype in western countries and age-standardised incidence rates are rising by 40% every 5 years.^2^ The United Kingdom has the highest incidence of OAC in the world, and outcomes are poor because 60–70% of patients present with late-stage disease too advanced for treatment with curative intent.^3^

Using whole genome sequencing the OCCAMS consortium has identified new mutational signatures of OAC disease types that might be suitable for targeted treatments.^4–6^ However, findings from the OCCAMS cohorts require pre-clinical validation prior to implementation in trials, and studies are needed to understand the extent to which the genomic distinction is maintained downstream, at the level of the transcriptome and proteome.^7^ Moreover, although mutationally corrupted cancer cells are recognised as the driving force of tumour development and progression, a key knowledge gap hindering the prediction of which patients will benefit from treatment is that the contribution of the tumour microenvironment (TME) is not considered.^8^

Our group’s work has focused on the relationship between tumour cells and cancer-associated fibroblasts (CAFs), which form the major cellular component of the TME.^9^ The in vivo “education” or “reprogramming” of fibroblasts by tumour cells is an established mechanism by which cancer cells exploit the plastic nature of reactive cell populations to generate a tumour-supportive microenvironment.^10^ The accumulation of CAFs in tumours correlates with poor prognosis across cancer types, including OAC, where we have shown that the presence of CAFs is more predictive of poor outcome than T, N or M stage.^11,12^ CAFs are most commonly characterised by the acquisition of an “activated”, alpha-smooth muscle actin (α-SMA) positive, myofibroblast phenotype,^11^ which regulates a number of tumour promoting processes.^12,13^ Additionally, CAFs may be implicated in the development of drug resistance during chemotherapy treatment of cancer patients.^14,15^ Along these lines, anti-cancer drugs have been found to become ineffective against cancer cells co-cultured with various types of stromal cells .^16^

Shotgun proteomics, supported by recent technological advances in liquid chromatography with mass spectrometry (LC-MS), is gradually becoming an indispensible analytical tool in cancer research since the unbiased protein expression profiling of tumours or their microenvironment can provide novel biological insight but also help identify novel diagnostic, prognostic and therapeutic targets that can eventually influence clinical practice.^13,17–20^ There are only a limited number of studies that have examined the global proteomic portrait of primary CAFs derived from human cancer patients.^21–23^

We have previously reported the shotgun proteomic analysis of primary, patient-matched, CAF/NOF pairs (*n* = 4) from patients with OAC.^13^ The focus of this study by Hanley et al. was to examine the relative expression levels of extracellular matrix proteins in primary patient-matched  CAF/NOF pairs (*n* = 4). The LC-MS analysis resulted in the profiling of 3579 unique proteins, of which 172 were up- and 368 down-regulated in CAFs vs. NOFs.

**Fig. 1:**
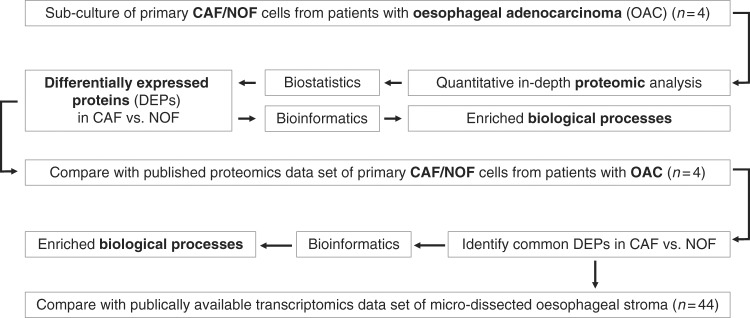
Study workflow

The aim of the present study was to apply a more in-depth proteomics methodology in combination with comprehensive bioinformatics analysis to an additional cohort of primary patient-matched CAF/NOF pairs (*n* = 4) derived from patients with OAC in order to gain insight into the pro-oncogenic features of the myofibroblast phenotype. An additional aim was to identify novel therapeutic targets relevant to the TME. An overview of the study workflow is presented in Fig. 1.

## Materials and methods

### Primary cell culture

Experimental protocols received ethical approval by the Southampton and South West Hampshire Research Ethics Committee (09/H0504/66). All participants signed an informed consent form. Fibroblasts were derived from four patients with OAC and sub-cultured as previously described.^12^ Normal fibroblasts (NOFs) were taken from the proximal resection margin (at least 10 cm distant from the cancer) of each patient. Cell culture passage number was consistently under four.

### Quantitative proteomics sample processing

Cell pellets were snap frozen at −80 °C. These were dissolved in 0.5 M triethylammonium bicarbonate, 0.05% sodium dodecyl sulphate and subjected to pulsed probe sonication (Misonix, Farmingdale, NY, USA). Lysates were centrifuged (16,000*g*, 10 min, 4^o^C) and supernatants were measured for protein content using infrared spectroscopy (Merck Millipore, Darmstadt, Germany). Lysates were then reduced, alkylated and subjected to trypsin proteolysis. Peptides were labelled using the eight-plex iTRAQ reagent kit  with the following reporter ion assignment: 113 = NOF patient 1, 114 = NOF patient 2, 115 = NOF patient 3, 116 = NOF patient 4, 117 = CAF patient 1, 118 = CAF patient 2, 119 = CAF patient 3, and 121 = CAF patient 4.  The labelled peptides were then subjected to  multi-dimensional liquid chromatography and tandem mass spectrometry as described below.

### Two-dimensional LC-MS proteomic analysis

To enhance peptide separation efficiency and subsequent mass spectrometry analysis, the initial offline peptide fractionation was conducted with  alkaline C4 Reverse Phase chromatography (Kromasil 150 × 2.1 mm, 3.5 μm particle, 100 Å pore size, Merck KGaA, Darmstadt, Germany) using gradient mobile phase conditions as previously reported by the authors.^24^ All other method details were as reported by the authors.^24,25^

### Database searching

Unprocessed raw files were submitted to Proteome Discoverer 1.4 for target decoy searching against the UniProtKB homo sapiens database comprised of 20,159 entries (release date January 2015), allowing for up to two missed cleavages, a precursor mass tolerance of 10ppm, a minimum peptide length of six and a maximum of two variable (one equal) modifications of; iTRAQ 8-plex (Y), oxidation (M), deamidation (N, Q), or phosphorylation (S, T, Y). Methylthio (C) and iTRAQ (K, Y and N-terminus) were set as fixed modifications. FDR at the peptide level was set at < 0.05. Percent co-isolation excluding peptides from quantitation was set at 50. Reporter ion ratios from unique peptides only were taken into consideration for the quantitation of the respective protein. Raw iTRAQ intensity values of unique peptides were median-normalised and log_2_ transformed. A Student’s *T*-Test using the normalised raw iTRAQ intensity was performed to identify differentially expressed unique peptides between CAFs and NOFs. Significance was set at *p* ≤ 0.05. A protein was considered to be differentially expressed in CAFs vs. NOFs when it had at least one differentially expressed unique peptide and a mean iTRAQ reporter ion log_2_ -ratio of ≥±0.2. In adherence to the Paris Publication Guidelines for the analysis and documentation of peptide and protein identifications (http://www.mcponline.org/site/misc/ParisReport_Final.xhtml), only proteins identified with at least two unique peptides were further subjected to bioinformatics. All mass spectrometry data have been deposited to the ProteomeXchange Consortium via PRIDE with the data set identifier PXD005444.

### Bioinformatics analysis

Principal component analysis (PCA) using the log_2_ratio of each sample over the mean of all samples was performed using the online tool ClustVis (http://biit.cs.ut.ee/clustvis/). DAVID (https://david.ncifcrf.gov/) was applied to differentially expressed proteins in order to identify over-represented gene ontology terms and KEGG pathways. Fisher exact corrected *p*-values ≤ 0.05 were considered significant. Subcellular localisation of top up- and down-regulated proteins in CAF vs. NOF was manually assessed using ExPASy (www.expasy.org). The diseases and functions module of  Ingenuity Pathway Analysis (IPA)  (Qiagen, Hilden, Germany) was used to predict upstream biological processes activated or inhibited based on a combination of up-regulated and down-regulated proteins observed. Biological processes with a Fisher’s exact *p*-value <0.05 and a false discovery rate score (*z*-score) of ≥2 or ≤−2 were considered significantly activated or inhibited, respectively.^26,27^

### Comparison of DEPs with published proteomics and transcriptomics data sets

DEPs were compared with our previously published proteomics dataset  t of primary CAFs/NOFs from patients with OAC (*n* = 4).^13^ To define DEPs in this previous  dataset , the exact same criteria as described above for the present study were used. Common DEPs in the two proteomics experiments were compared with a publically available transcriptomics  dataset  of laser-capture micro-dissected oesophageal stroma (*n* = 44; 17 with intestinal metaplasia, 16 with dysplasia and 11 with adenocarcinoma) (NCBI/NIH; GEO; dataset  ID: GSE19632).

### In silico evaluation of the prognostic value of DEPs in OAC

Proteins identified to be differentially expressed in CAFs vs. NOFs in both proteomics experiments were *in silico* evaluated for their prognostic value in OAC using PrognoScan (http://www.abren.net/PrognoScan/), a database of published cancer microarray experiments linking gene expression to patient prognosis.^28^

### Immunohistochemical validation of key findings

Immunohistochemical staining was performed in sections derived from a cohort of 183 OAC patients as previously described.^12^ Briefly, sections of thickness 5μm were taken from the recipient paraffin block for IHC staining. Primary antibody dilution for polyclonal rabbit anti-human Nestin was 1:100 (DAKO no. M3515). Slides were de-paraffinised with xylene and rehydrated with alcohol. Incubation in 3% H_2_O_2_ (in deionised water) for 10 min was used to suppress endogenous peroxidase activity. Slides were incubated in 1 mM ethylenediaminetetraacetic acid for 15 min at 98 °C and pH 8.0, allowing antigen retrieval. Tissue was sequentially incubated in avidin, biotin, primary and biotinylated secondary antibody (at appropriate dilutions), streptabidin biotin-peroxidase complexes and DAB (3-3′-diaminobenzidine). Cores were counter-stained with Mayers Haematoxylin, dehydrated and mounted with DPX. The automated immunostainer DAKO^®^ Autostainer Link 48 (Cambridge, UK) was used in a CPA-accredited cellular pathology department with the use of antibodies optimised to national diagnostic standards (NEQAS).

## Results

### Proteomic profiling of primary oesophageal fibroblasts

**Fig. 2:**
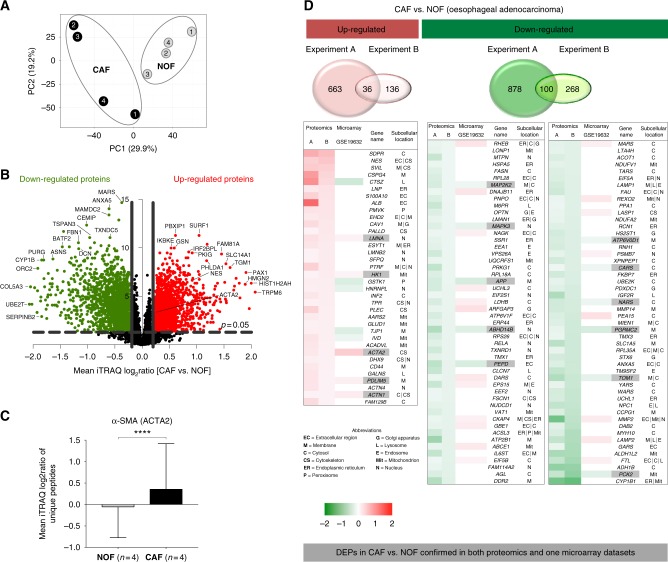
**a** Principal component analysis using the reporter ion log_2_ratios of all analysed proteins showed that CAFs had  a distinct proteomic profile and higher heterogeneity compared to NOFs. **b** Volcano plot highlighting the differentially expressed proteins in CAFs vs. NOFs (red = up-regulated proteins; green = down-regulated proteins). **c** Alpha smooth muscle actin (*ACTA2*) was found to be significantly up-regulated in CAFs vs. NOFs (Mean log_2_ratio (SD) = 0.2 (0.9); *p*-value <0.0001 at the peptide level) **d** In total, 136 DEPs were also analysed with the same trend of modulation in a previously published proteomics  dataset of primary CAFs/NOFs from patients with OAC. Of these, five up-regulated and 11 down-regulated proteins were confirmed in the microarray dataset  (highlighted in grey)

We compared the global proteomic profiles of matched pairs of primary CAFs and NOFs taken from oesophageal resections of four OAC patients in order to identify proteins and pathways that may be responsible for the pro-oncogenic CAF phenotype and the poor patient prognosis associated with the accumulation of CAFs in OAC. Proteomic analysis resulted in the profiling of 7718 unique protein groups (peptide FDR *p*-value <0.05) (Supplementary Table 1), a substantial improvement of more than double the number of profiled unique proteins compared to our previously published proteomics  dataset. PCA of all profiled proteins demonstrated that NOFs had a more homogeneous proteomic profile and clustered separately form the more heterogeneous CAFs. (Fig. 2a).

The differentially expressed proteome (DEP) comprised 699 up-regulated and 987 down-regulated proteins in CAFs compared to NOFs (Supplementary Table 2). A volcano plot representation of the mean iTRAQ reporter ion log_2_ -ratio of proteins in CAF vs. NOF plotted against the minus log10 (*p*-value) is presented in Fig. 2b. Alpha-SMA expression was found to be variable but with a mean log_2 _ratio of 0.2 ± 0.9 (*p*-value < 0.0001 at the peptide level) across all CAFs vs. NOFs examined (Fig. 2c).

### Comparison of DEPs with published proteomics and transcriptomics data sets

Of the DEPs, 136 proteins were also identified with the same trend of modulation in our previously published proteomic analysis of   primary CAF/NOF pairs from an independent cohort of patients with OAC^13^ and the expression trend of five up-regulated and 11 down-regulated proteins was confirmed in the publically available microarray dataset  of OAC micro-dissected stromal cells. These proteins are presented in heatmap format in Fig. 2d. Proteins identified in both proteomic experiments and confirmed with the same trend of modulation in the microarray data set are highlighted in grey (Fig. 2d). Among the proteins identified in both proteomics and confirmed at the transcriptomics data set to be up-regulated in CAFs vs. NOFs were α-SMA, lamin A (*LMNA*) and actin-1 (*ACTN1*).

### Bioinformatics Analysis

**Fig. 3:**
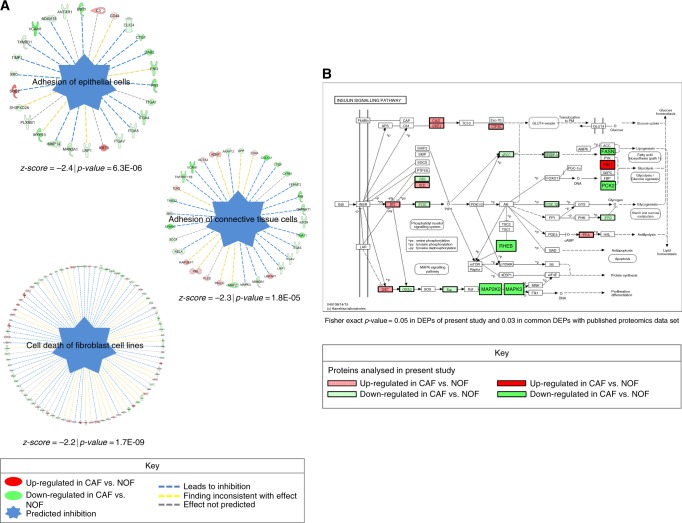
**a** The diseases and functions module of IPA predicted the significant inhibition of adhesion of epithelial cells (*z*-score = −2.4 | *p* = 6.3E-06), adhesion of connective tissue cells (*z*-score = −2.3 | *p* = 1.8E-05) and cell death of fibroblast cell lines (*z*-score = −2.2 | *p* = 1.7E-09) in CAFs vs. NOFs. **b** KEGG pathway analysis using DAVID showed a significant enrichment of the insulin-signalling pathway (Fisher exact *p*-value = 0.03 for the common proteins between the two proteomics experiments and 0.05 for the DEPs analysed in the present study).

The diseases and functions module of IPA predicted, based on the downstream up-regulated and down-regulated proteins, that adhesion of epithelial cells (*z*-score = −2.4 | *p* = 6.3E-06), adhesion of connective tissue cells (*z*-score = −2.3 | *p* = 1.8E−05) and cell death of fibroblast cell lines (*z*-score = −2.2 | *p* = 1.7E−09) were significantly inhibited in CAFs vs. NOFs (Fig. 3a). KEGG pathway analysis using DAVID showed a significant enrichment of the insulin-signalling pathway (Fisher exact *p*-value = 0.03 for the common proteins between the two proteomics experiments and 0.05 for the DEPs analysed in the present study) (Fig. 3b).

**Fig. 4:**
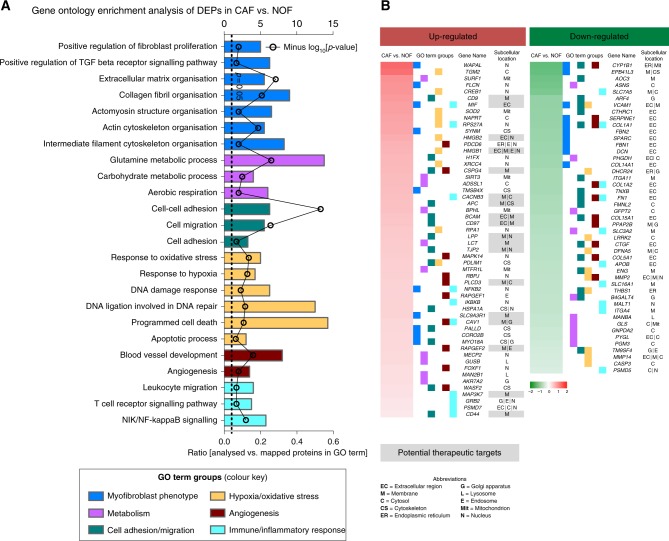
**a** DAVID gene ontology analysis showed that gene ontology terms related with myofibroblast phenotype, metabolism, cell adhesion/migration, hypoxia/oxidative stress, angiogenesis, immune/inflammatory response were significantly over-represented in the DEPs. **b** Heatmap of top 10 up- and top 10 down-regulated proteins mapping to each gene ontology terms  group . The subcellular location of each protein is also presented and up-regulated proteins that are either secreted or membrane are highlighted as potential therapeutic targets.

DAVID gene ontology analysis, accounting for both up-regulated and down-regulated proteins constituting the DEP, demonstrated that processes related with myofibroblast phenotype, metabolism, cell adhesion/migration, hypoxia/oxidative stress, angiogenesis, and immune/inflammatory response were over-represented (Fig. 4a). The top ten up-regulated and down-regulated proteins mapping to each GO term group are presented in heatmap format in Fig. 4b. The sub-cellular localisation of these proteins is also presented in the heatmap. Top up-regulated proteins that are either secreted or localised in the membrane are highlighted in the heatmap as potential therapeutic targets in CAFs (gene names of the respective proteins are: *CD9, MIF, HMGB2, HMGB1, CSPG4, CACNB3, APC, BCAM, CD97, LPP, LCT, TJP2, PLCD3, SLC9A3R1, CAV1, RAPGEF2, MAP3K7* and *CD44*) (Fig. 4b).

### *In silico* evaluation of the prognostic value of Nestin in OAC

**Fig. 5:**
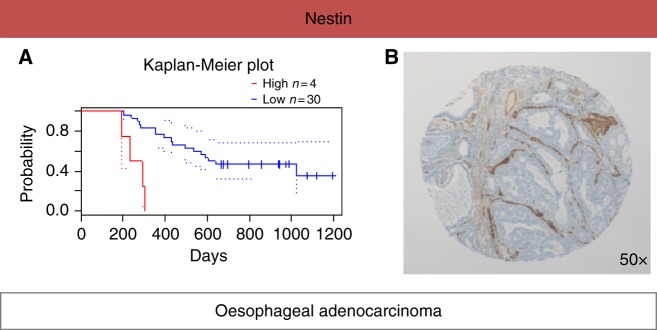
**a** Using the *in silico* PrognoScan meta-analysis microarray database, higher expression levels of nestin were  found to correlate with poor patient prognosis [COX *p*-value = 0.003; HR (95% CI) = 78.0 (4.3–1409.8)]. **b** Immunohistochemical staining of nestin in OAC showed a conserved expression pattern in the tumour microenvironment, with expression being confined to CAFs, blood vessels and smooth muscle cells.

Using the *in silico* PrognoScan meta-analysis microarray database for the common DEPs in both proteomics experiments, increased levels of nestin was found to be associated with poor OAC patient prognosis [COX *p*-value = 0.003; HR (95% CI) = 78.0 (4.3 to 1409.8)] (Fig. 5a). Immunohistochemical staining of nestin was performed in a well-described cohort of 183 oesophageal tumours where the presence of α-SMA positive CAFs correlated strongly with poor overall survival.^12^ The patient clinico-pathological characteristics of this cohort have been reported before.^12^ Nestin showed a conserved expression pattern in the TME of OAC, being confined to CAFs, blood vessels and smooth muscle cells. Example staining is shown in Fig. 5b.

## Discussion

The seminal work of Stephen Paget over a century ago proposed that cancer cells constitute the “seeds” that colonise a favourable stromal microenvironment as the receptive “soil”.^29,30^ A key “soil” constituent is the NOF that acquires a cancerous phenotype by the “seed” cancer cell to facilitate its proliferation, invasion, and metastasis.^31^ However, the proteomic characterisation of such cancer fibroblasts remains limited.

To address this need, our study made use of a comprehensive quantitative proteomics approach (Fig. 1) and reports the most extensive proteome coverage to date of primary CAF/NOF pairs derived from patients with OAC. PCA against the reporter ion ratios of the 7718 unique protein analysed across all samples showed that CAFs had a distict proteomic profile relative to NOFs.  (Fig. 2a). In keeping with previous findings,^12,32^ PCA analysis showed marked heterogeneity in proteome expression between the CAFs  relative to the more homogeneous proteome expression between the NOFs Significant differential expression was observed for 699 up-regulated and 987 down-regulated proteins across all CAFs relative to all NOFs, as highlighted in the volcano plot of Fig. 2b (log_2_ -ratio ≥ 0.2, *p* ≤ 0.05, *t*-test). Alpha-SMA (*ACTA2*) was analysed to be marginally up-regulated in CAFs vs. NOFs (as illustrated in the volcano plot of Fig. 2b) (log_2_ratio = 0.2 ± 0.9; *p*-value <0.0001 at the peptide level) (Fig. 2c). By contrast, our quantitative proteome revealed a large spectrum of novel proteins exhibiting a higher and more consistent level of differential expression that may constitute more robust candidate markers of the CAF phenotype (Fig. 2b,c, Supplementary Table 2). Consistent protein differential expression of CAF canonical markers was observed between the current quantitative proteome, a proteomics dataset  reported by the authors^13^ and a publically available transcriptomics microarray dataset  (Fig. 2d). Notable surrogate markers  consistently observed in the  CAF phenotype include the up-regulated proteins lamin A (*LMNA*) and actin-1 (*ACTN1*). *LMNA* has been implicated in the modulation of TGF-β1 on collagen production and mesenchymal differentiation,^33^ and *ACTN1* up-regulation has been described in stromal fibroblasts derived from oral cancers.^34^

The diseases and functions module of IPA predicted the inhibition of adhesion of epithelial cells (*z*-score = −2.4 | *p* = 6.3E-06) and adhesion of connective tissue cells (*z*-score = −2.3 | *p* = 1.8E–05) (Fig. 3a). The inhibition of these processes suggests the involvement of CAFs on increasing the tumour’s metastatic potential. These findings confirm and extend the current knowledge of the CAF phenotype also affecting cell adhesion/cell migration processes.^12,13^

Of relevance, given the endoergic character of increased cellular proliferation and pro-metastatic phenotypes observed, the insulin-signalling pathway was significantly enriched in the DEPs of the present study as well as the commonly observed  proteins with our previously published proteomics dataset  (Fig. 3b). Increased expression of the insulin-like growth factor 1 (IGF-I) and its receptor (IGF–IR) has been found to be associated with tumour progression and poor prognosis in different cancer types including gastrointestinal tumours.^35,36^ The tumour promoting properties of the IGF–IR are  interlinked with  the activation of the down-stream insulin receptor substrates (IRS).^37,38^ IGF-I also plays a key role in the autocrine and paracrine induction of CAF “activation”.^14^ A recent study showed that NT157, an inhibitor of the IGF–IR–IRS signalling pathway, resulted in inhibition of CAF “activation”, as well as reduced expression of pro-oncogenic chemokines, cytokines and growth factors, including several interleukins (IL-6, IL-11, IL-23) and TGFβ.^39^ The de-regulation of the insulin signalling pathway in CAFs could also be linked to the “Reverse Warburg effect”, a model describing the metabolic coupling between stromal and cancer cells.^40^ One interesting protein mapping to  the insulin-signalling pathway was hexokinase-1 (HK1), which  was consistently upregulated in both proteomic experiments and further confirmed at the microarray dataset  (Figs. 2d and 3b). HK1 catalyses the first obligatory and rate-limiting step involving the phosphorylation of glucose  to G6P.^41^ Furthermore, HK1 has been suggested to regulate cell death, a process associated with abnormal proliferation and tumourigenesis.^42^ HK1 has also been found to be upregulated in different cancer types, including kidney and breast carcinomas.^43,44^ Additionally , a recent study showed that HK1 over-expression was  associated with poor patient prognosis in colorectal cancer.^45^ HK1 expression in CAFs and its implication with tumour aggressiveness warrants further investigation.

DAVID GO analysis identified terms related to the  myofibroblast phenotype, metabolism, cell adhesion/migration, hypoxia/oxidative stress (including DNA damage response), angiogenesis, and immune/inflammatory response processes to be over-represented in the DEPs (Fig. 4a). The gene names of the top-10 differentially expressed proteins observed for each of these processes, including those classified as secreted or membrane associated, constitute novel observations and may reveal candidate therapeutic targets (Fig. 4b).

Hypoxia, oxidative stress and DNA damage response were significantly enriched GO terms. Oxidation–reduction is an established process in CAFs.^31,46^ CAFs have been shown to overproduce reactive oxygen species (ROS), leading to oxidative stress, inflammation and significant cellular damage, which could in turn affect DNA damage response.^31,47^ The over-production of ROS by CAFs can induce oxidative stress in NOFs that further triggers CAF activation, thus leading to a positive feedback loop between ROS production and CAF activation.^48,49^ Moreover , the  cell death of fibroblasts was found to be inhibited (*z*-score = −2.2 | *p* = 1.7E–09), showing that CAFs may evade apoptosis possibly as a result of their enhanced DNA damage response.

Immune and inflammatory responses were also significantly over-represented terms in CAFs vs. NOFs (Fig. 4a). Previous studies have reported on the immunomodulatory effects of CAFs.^50–52^ Specific pathways and their participatory proteins responsible for the interplay between CAFs and the host immune response may be of relevance to a number of current clinical trials using immune checkpoint inhibitors in unselected patients with OAC. The success rate of these therapies may not depend entirely on the immune system, but also implicate CAF-induced alterations of the TME in preventing immune cell entry. This may necessitate  the combined use of immunotherapy and CAF permeability modifiers.^53^ At the same token, CAFs have been reported to promote angiogenesis through different mechanisms, including ECM remodelling, recruitment of epithelial progenitor cells, and increased leucocyte infiltration through chemokine secretion, that in turn produce angiogenic factors.^54^

An up-regulated protein identified in both proteomic experiments was nestin. Nestin was further investigated as it was found to correlate with decreased overall survival in patients with oesophageal cancer  when using  the *in silico* microarray meta-analysis tool PrognoScan (Fig. 5a), suggesting  its  important role in OAC biology. Nestin is an intermediate filament protein originally detected in neuronal stem cells during development.^55^ Nestin has been detected in various types of solid tumours, including mesenchymal tumours and cancers (e.g., breast, lung, ovarian and gastrointestinal).^56^ Nestin has been suggested as a stem-cell marker indicating an undifferentiated and thus more invasive phenotype of transformed cells.^57^ Immunohistochemical staining showed that nestin protein expression was confined to the TME of OAC (Fig. 5b). A recent study showed that nestin suppression reduced the metastatic potential of endometrial cancer cells by inhibiting the TGFβ signalling cascade,^58^ the main pathway promoting aberrant CAF “activation”.^59^

The main study limitation is that only four matched pairs of fibroblasts were used to generate the proteomic expression profiles. This is partly compensated, however, by the evaluation of the analysed proteins using our previously published proteomics dataset  (*n* = 4) and an independent microarray dataset  (*n* = 44).

In conclusion, this study reports the proteomic profiling of primary CAFs from patients with OAC, a cancer with a vast unmet clinical need. The biological pathways and networks observed for the primary CAFs examined were found to emulate all the intrinsic hallmarks of cancer, as expected given the strong functional cross-talk between fibroblasts and cancer cells.  Consequently, the participating proteins to these biological processes may constitute novel adjuvant therapeutic targets for OAC in the TME as part of precision medicine protocols.

## Electronic supplementary material

Supplementary Table 1. Analysed proteins (peptide FDR < 0.05)

Supplementary Table 2. Differentially expressed proteins in CAFs vs. NOFs
