# Supplementary material for: Quantitative proteomic profiling of primary cancer-associated fibroblasts in oesophageal adenocarcinoma
Source: Br J Cancer. 2018 Mar 29;118(9):1200–7. doi: 10.1038/s41416-018-0042-9 (PMC5943522; doi:10.1038/s41416-018-0042-9)
Supplement: Supplementary file 2 — Supplementary Table 2. Differentially expressed proteins in CAFs vs. NOFs [file 41416_2018_42_MOESM2_ESM.pdf]

Supplementary Table 2. Differentially expressed proteins in CAFs vs. NOFs

Accession	Description	Mean log2ratio [CAF vs. NOF]
P05204	Non-histone chromosomal protein HMG-17 OS=Homo sapiens GN=HMGN2 PE=1 SV=3 - [HMGN2_HUMAN]	1.7
Q9BRX8	Redox-regulatory protein FAM213A OS=Homo sapiens GN=FAM213A PE=1 SV=3 - [F213A_HUMAN]	1.6
Q8TBF8	Protein FAM81A OS=Homo sapiens GN=FAM81A PE=2 SV=3 - [FA81A_HUMAN]	1.4
P52943	Cysteine-rich protein 2 OS=Homo sapiens GN=CRIP2 PE=1 SV=1 - [CRIP2_HUMAN]	1.3
P16403	Histone H1.2 OS=Homo sapiens GN=HIST1H1C PE=1 SV=2 - [H12_HUMAN]	1.3
Q7Z5K2	Wings apart-like protein homolog OS=Homo sapiens GN=WAPAL PE=1 SV=1 - [WAPL_HUMAN]	1.3
Q9UBF8	Phosphatidylinositol 4-kinase beta OS=Homo sapiens GN=PI4KB PE=1 SV=1 - [PI4KB_HUMAN]	1.2
P21980	Protein-glutamine gamma-glutamyltransferase 2 OS=Homo sapiens GN=TGM2 PE=1 SV=2 - [TGM2_HUMAN]	1.2
P37059	Estradiol 17-beta-dehydrogenase 2 OS=Homo sapiens GN=HSD17B2 PE=1 SV=1 - [DHB2_HUMAN]	1.1
O15211	Ral guanine nucleotide dissociation stimulator-like 2 OS=Homo sapiens GN=RGL2 PE=1 SV=1 - [RGL2_HUMAN]	1.0
O14879	Interferon-induced protein with tetratricopeptide repeats 3 OS=Homo sapiens GN=IFIT3 PE=1 SV=1 - [IFIT3_HUMAN]	1.0
Q8IZR5	CKLF-like MARVEL transmembrane domain-containing protein 4 OS=Homo sapiens GN=CMTM4 PE=1 SV=1 - [CKLF4_HUMAN]	1.0
O95810	Serum deprivation-response protein OS=Homo sapiens GN=SDPR PE=1 SV=3 - [SDPR_HUMAN]	1.0
Q15413	Ryanodine receptor 3 OS=Homo sapiens GN=RYR3 PE=1 SV=3 - [RYR3_HUMAN]	0.9
Q15526	Surfeit locus protein 1 OS=Homo sapiens GN=SURF1 PE=1 SV=1 - [SURF1_HUMAN]	0.9
O00194	Ras-related protein Rab-27B OS=Homo sapiens GN=RAB27B PE=1 SV=4 - [RB27B_HUMAN]	0.9
P07305	Histone H1.0 OS=Homo sapiens GN=H1F0 PE=1 SV=3 - [H10_HUMAN]	0.9
Q8NFG4	Folliculin OS=Homo sapiens GN=FLCN PE=1 SV=1 - [FLCN_HUMAN]	0.9
P31323	cAMP-dependent protein kinase type II-beta regulatory subunit OS=Homo sapiens GN=PRKAR2B PE=1 SV=3 - [KAP3_HUMAN]	0.9
P16220	Cyclic AMP-responsive element-binding protein 1 OS=Homo sapiens GN=CREB1 PE=1 SV=2 - [CREB1_HUMAN]	0.9
Q9P0V9	Septin-10 OS=Homo sapiens GN=SEPT10 PE=1 SV=2 - [SEP10_HUMAN]	0.9
P21926	CD9 antigen OS=Homo sapiens GN=CD9 PE=1 SV=4 - [CD9_HUMAN]	0.9
Q9C005	Protein dpy-30 homolog OS=Homo sapiens GN=DPY30 PE=1 SV=1 - [DPY30_HUMAN]	0.8
Q9NPH2	Inositol-3-phosphate synthase 1 OS=Homo sapiens GN=ISYNA1 PE=1 SV=1 - [INO1_HUMAN]	0.8
Q6ZUT6	Uncharacterized protein C15orf52 OS=Homo sapiens GN=C15orf52 PE=1 SV=1 - [CO052_HUMAN]	0.8
P48681	Nestin OS=Homo sapiens GN=NES PE=1 SV=2 - [NEST_HUMAN]	0.8
P14174	Macrophage migration inhibitory factor OS=Homo sapiens GN=MIF PE=1 SV=4 - [MIF_HUMAN]	0.8
P04179	Superoxide dismutase [Mn], mitochondrial OS=Homo sapiens GN=SOD2 PE=1 SV=2 - [SODM_HUMAN]	0.8
Q9Y294	Histone chaperone ASF1A OS=Homo sapiens GN=ASF1A PE=1 SV=1 - [ASF1A_HUMAN]	0.8
Q8TDX9	Polycystic kidney disease protein 1-like 1 OS=Homo sapiens GN=PKD1L1 PE=1 SV=1 - [PK1L1_HUMAN]	0.8
Q9Y3Z3	Deoxynucleoside triphosphate triphosphohydrolase SAMHD1 OS=Homo sapiens GN=SAMHD1 PE=1 SV=2 - [SAMH1_HUMAN]	0.8
Q6XQN6	Nicotinate phosphoribosyltransferase OS=Homo sapiens GN=NAPRT PE=1 SV=2 - [PNCB_HUMAN]	0.8
Q9H1B7	Interferon regulatory factor 2-binding protein-like OS=Homo sapiens GN=IRF2BPL PE=1 SV=1 - [I2BPL_HUMAN]	0.8
Q00978	Interferon regulatory factor 9 OS=Homo sapiens GN=IRF9 PE=1 SV=1 - [IRF9_HUMAN]	0.8
P62979	Ubiquitin-40S ribosomal protein S27a OS=Homo sapiens GN=RPS27A PE=1 SV=2 - [RS27A_HUMAN]	0.8
O15061	Synemin OS=Homo sapiens GN=SYNM PE=1 SV=2 - [SYNEM_HUMAN]	0.8
O75762	Transient receptor potential cation channel subfamily A member 1 OS=Homo sapiens GN=TRPA1 PE=1 SV=3 - [TRPA1_HUMAN]	0.8
O15091	Mitochondrial ribonuclease P protein 3 OS=Homo sapiens GN=KIAA0391 PE=1 SV=2 - [MRRP3_HUMAN]	0.7
P0C0S5	Histone H2A.Z OS=Homo sapiens GN=H2AFZ PE=1 SV=2 - [H2AZ_HUMAN]	0.7
P51965	Ubiquitin-conjugating enzyme E2 E1 OS=Homo sapiens GN=UBE2E1 PE=1 SV=1 - [UB2E1_HUMAN]	0.7
Q14141	Septin-6 OS=Homo sapiens GN=SEPT6 PE=1 SV=4 - [SEPT6_HUMAN]	0.7
P16104	Histone H2AX OS=Homo sapiens GN=H2AFX PE=1 SV=2 - [H2AX_HUMAN]	0.7
Q8N283	Ankyrin repeat domain-containing protein 35 OS=Homo sapiens GN=ANKRD35 PE=2 SV=2 - [ANR35_HUMAN]	0.7
Q9UJ14	Gamma-glutamyltransferase 7 OS=Homo sapiens GN=GGT7 PE=1 SV=2 - [GGT7_HUMAN]	0.7
P26583	High mobility group protein B2 OS=Homo sapiens GN=HMGB2 PE=1 SV=2 - [HMGB2_HUMAN]	0.7
O60488	Long-chain-fatty-acid--CoA ligase 4 OS=Homo sapiens GN=ACSL4 PE=1 SV=2 - [ACSL4_HUMAN]	0.7
O75167	Phosphatase and actin regulator 2 OS=Homo sapiens GN=PHACTR2 PE=1 SV=2 - [PHAR2_HUMAN]	0.7
P05787	Keratin, type II cytoskeletal 8 OS=Homo sapiens GN=KRT8 PE=1 SV=7 - [K2C8_HUMAN]	0.7
Q9NZJ6	Hexaprenyldihydroxybenzoate methyltransferase, mitochondrial OS=Homo sapiens GN=COQ3 PE=1 SV=3 - [COQ3_HUMAN]	0.7

O95425	Supervillin OS=Homo sapiens GN=SVIL PE=1 SV=2 - [SVIL_HUMAN]	0.7
P52434	DNA-directed RNA polymerases I, II, and III subunit RPABC3 OS=Homo sapiens GN=POLR2H PE=1 SV=4 - [RPAB3_HUMAN]	0.7
Q8N111	Cell cycle exit and neuronal differentiation protein 1 OS=Homo sapiens GN=CEND1 PE=2 SV=1 - [CEND_HUMAN]	0.7
Q96RT7	Gamma-tubulin complex component 6 OS=Homo sapiens GN=TUBGCP6 PE=1 SV=3 - [GCP6_HUMAN]	0.7
O60658	High affinity cAMP-specific and IBMX-insensitive 3',5'-cyclic phosphodiesterase 8A OS=Homo sapiens GN=PDE8A PE=1 SV=2 - [PDE8A_HUMAN]	0.7
Q9UKL0	REST corepressor 1 OS=Homo sapiens GN=RCOR1 PE=1 SV=1 - [RCOR1_HUMAN]	0.7
Q8TDW0	Volume-regulated anion channel subunit LRRC8C OS=Homo sapiens GN=LRRC8C PE=1 SV=2 - [LRC8C_HUMAN]	0.7
O75340	Programmed cell death protein 6 OS=Homo sapiens GN=PDCD6 PE=1 SV=1 - [PDCD6_HUMAN]	0.6
P78337	Pituitary homeobox 1 OS=Homo sapiens GN=PITX1 PE=1 SV=2 - [PITX1_HUMAN]	0.6
P54652	Heat shock-related 70 kDa protein 2 OS=Homo sapiens GN=HSPA2 PE=1 SV=1 - [HSP72_HUMAN]	0.6
P09429	High mobility group protein B1 OS=Homo sapiens GN=HMGB1 PE=1 SV=3 - [HMGB1_HUMAN]	0.6
O00479	High mobility group nucleosome-binding domain-containing protein 4 OS=Homo sapiens GN=HMGN4 PE=1 SV=3 - [HMGN4_HUMAN]	0.6
Q9H6E4	Coiled-coil domain-containing protein 134 OS=Homo sapiens GN=CCDC134 PE=1 SV=1 - [CC134_HUMAN]	0.6
Q9P2G1	Ankyrin repeat and IBR domain-containing protein 1 OS=Homo sapiens GN=ANKIB1 PE=1 SV=3 - [AKIB1_HUMAN]	0.6
Q92522	Histone H1x OS=Homo sapiens GN=H1FX PE=1 SV=1 - [H1X_HUMAN]	0.6
O95786	Probable ATP-dependent RNA helicase DDX58 OS=Homo sapiens GN=DDX58 PE=1 SV=2 - [DDX58_HUMAN]	0.6
Q9UKS6	Protein kinase C and casein kinase substrate in neurons protein 3 OS=Homo sapiens GN=PACSIN3 PE=1 SV=2 - [PACN3_HUMAN]	0.6
Q13426	DNA repair protein XRCC4 OS=Homo sapiens GN=XRCC4 PE=1 SV=2 - [XRCC4_HUMAN]	0.6
Q8TB37	Iron-sulfur protein NUBPL OS=Homo sapiens GN=NUBPL PE=1 SV=3 - [NUBPL_HUMAN]	0.6
Q6UVK1	Chondroitin sulfate proteoglycan 4 OS=Homo sapiens GN=CSPG4 PE=1 SV=2 - [CSPG4_HUMAN]	0.6
P82909	28S ribosomal protein S36, mitochondrial OS=Homo sapiens GN=MRPS36 PE=1 SV=2 - [RT36_HUMAN]	0.6
O14874	[3-methyl-2-oxobutanoate dehydrogenase [lipoamide]] kinase, mitochondrial OS=Homo sapiens GN=BCKDK PE=1 SV=2 - [BCKD_HUMAN]	0.6
P57679	Ellis-van Creveld syndrome protein OS=Homo sapiens GN=EVC PE=1 SV=1 - [EVC_HUMAN]	0.6
Q9NUT2	ATP-binding cassette sub-family B member 8, mitochondrial OS=Homo sapiens GN=ABCB8 PE=1 SV=3 - [ABCB8_HUMAN]	0.6
Q9NTG7	NAD-dependent protein deacetylase sirtuin-3, mitochondrial OS=Homo sapiens GN=SIRT3 PE=1 SV=2 - [SIR3_HUMAN]	0.6
Q8N142	Adenylosuccinate synthetase isozyme 1 OS=Homo sapiens GN=ADSSL1 PE=2 SV=1 - [PURA1_HUMAN]	0.6
Q9P016	Thymocyte nuclear protein 1 OS=Homo sapiens GN=THYN1 PE=1 SV=1 - [THYN1_HUMAN]	0.6
P62328	Thymosin beta-4 OS=Homo sapiens GN=TMSB4X PE=1 SV=2 - [TYB4_HUMAN]	0.6
Q96JG8	Melanoma-associated antigen D4 OS=Homo sapiens GN=MAGED4 PE=1 SV=3 - [MAGD4_HUMAN]	0.6
Q96HJ9	UPF0562 protein C7orf55 OS=Homo sapiens GN=C7orf55 PE=1 SV=2 - [CG055_HUMAN]	0.6
Q8N2S1	Latent-transforming growth factor beta-binding protein 4 OS=Homo sapiens GN=LTBP4 PE=1 SV=2 - [LTBP4_HUMAN]	0.5
Q9BSQ5	Malcavernin OS=Homo sapiens GN=CCM2 PE=1 SV=1 - [CCM2_HUMAN]	0.5
P05161	Ubiquitin-like protein ISG15 OS=Homo sapiens GN=ISG15 PE=1 SV=5 - [ISG15_HUMAN]	0.5
Q562E7	WD repeat-containing protein 81 OS=Homo sapiens GN=WDR81 PE=1 SV=2 - [WDR81_HUMAN]	0.5
P54284	Voltage-dependent L-type calcium channel subunit beta-3 OS=Homo sapiens GN=CACNB3 PE=1 SV=1 - [CACB3_HUMAN]	0.5
Q9P2K5	Myelin expression factor 2 OS=Homo sapiens GN=MYEF2 PE=1 SV=3 - [MYEF2_HUMAN]	0.5
Q9UBR2	Cathepsin Z OS=Homo sapiens GN=CTSZ PE=1 SV=1 - [CATZ_HUMAN]	0.5
P25054	Adenomatous polyposis coli protein OS=Homo sapiens GN=APC PE=1 SV=2 - [APC_HUMAN]	0.5
Q86WA6	Valacyclovir hydrolase OS=Homo sapiens GN=BPHL PE=1 SV=1 - [BPHL_HUMAN]	0.5
P23527	Histone H2B type 1-O OS=Homo sapiens GN=HIST1H2BO PE=1 SV=3 - [H2B1O_HUMAN]	0.5
P21399	Cytoplasmic aconitate hydratase OS=Homo sapiens GN=ACO1 PE=1 SV=3 - [ACOC_HUMAN]	0.5
Q86U44	N6-adenosine-methyltransferase 70 kDa subunit OS=Homo sapiens GN=METTL3 PE=1 SV=2 - [MTA70_HUMAN]	0.5
P35568	Insulin receptor substrate 1 OS=Homo sapiens GN=IRS1 PE=1 SV=1 - [IRS1_HUMAN]	0.5
Q9UBL3	Set1/Ash2 histone methyltransferase complex subunit ASH2 OS=Homo sapiens GN=ASH2L PE=1 SV=1 - [ASH2L_HUMAN]	0.5
Q9Y5J7	Mitochondrial import inner membrane translocase subunit Tim9 OS=Homo sapiens GN=TIMM9 PE=1 SV=1 - [TIM9_HUMAN]	0.5
Q96AQ6	Pre-B-cell leukemia transcription factor-interacting protein 1 OS=Homo sapiens GN=PBXIP1 PE=1 SV=1 - [PBIP1_HUMAN]	0.5
P62805	Histone H4 OS=Homo sapiens GN=HIST1H4A PE=1 SV=2 - [H4_HUMAN]	0.5
Q14980	Nuclear mitotic apparatus protein 1 OS=Homo sapiens GN=NUMA1 PE=1 SV=2 - [NUMA1_HUMAN]	0.5
P38159	RNA-binding motif protein, X chromosome OS=Homo sapiens GN=RBMX PE=1 SV=3 - [RBMX_HUMAN]	0.5
Q96CN7	Isochorismatase domain-containing protein 1 OS=Homo sapiens GN=ISOC1 PE=1 SV=3 - [ISOC1_HUMAN]	0.5
P09104	Gamma-enolase OS=Homo sapiens GN=ENO2 PE=1 SV=3 - [ENOG_HUMAN]	0.5
Q9UKC9	F-box/LRR-repeat protein 2 OS=Homo sapiens GN=FBXL2 PE=1 SV=3 - [FBXL2_HUMAN]	0.5

Q5VUA4	Zinc finger protein 318 OS=Homo sapiens GN=ZNF318 PE=1 SV=2 - [ZN318_HUMAN]	0.5
Q12996	Cleavage stimulation factor subunit 3 OS=Homo sapiens GN=CSTF3 PE=1 SV=1 - [CSTF3_HUMAN]	0.5
O75828	Carbonyl reductase [NADPH] 3 OS=Homo sapiens GN=CBR3 PE=1 SV=3 - [CBR3_HUMAN]	0.5
Q96FJ2	Dynein light chain 2, cytoplasmic OS=Homo sapiens GN=DYNLL2 PE=1 SV=1 - [DYL2_HUMAN]	0.5
Q16352	Alpha-internexin OS=Homo sapiens GN=INA PE=1 SV=2 - [AINX_HUMAN]	0.5
P06396	Gelsolin OS=Homo sapiens GN=GSN PE=1 SV=1 - [GELS_HUMAN]	0.5
P20962	Parathymosin OS=Homo sapiens GN=PTMS PE=1 SV=2 - [PTMS_HUMAN]	0.5
Q8TDR0	TRAF3-interacting protein 1 OS=Homo sapiens GN=TRAF3IP1 PE=1 SV=1 - [MIPT3_HUMAN]	0.5
Q9BTC0	Death-inducer obliterator 1 OS=Homo sapiens GN=DIDO1 PE=1 SV=5 - [DIDO1_HUMAN]	0.5
Q9BX40	Protein LSM14 homolog B OS=Homo sapiens GN=LSM14B PE=1 SV=1 - [LS14B_HUMAN]	0.5
P50895	Basal cell adhesion molecule OS=Homo sapiens GN=BCAM PE=1 SV=2 - [BCAM_HUMAN]	0.5
Q5BJF6	Outer dense fiber protein 2 OS=Homo sapiens GN=ODF2 PE=1 SV=1 - [ODFP2_HUMAN]	0.5
Q92796	Disks large homolog 3 OS=Homo sapiens GN=DLG3 PE=1 SV=2 - [DLG3_HUMAN]	0.5
P48960	CD97 antigen OS=Homo sapiens GN=CD97 PE=1 SV=4 - [CD97_HUMAN]	0.5
O14795	Protein unc-13 homolog B OS=Homo sapiens GN=UNC13B PE=1 SV=2 - [UN13B_HUMAN]	0.5
P01024	Complement C3 OS=Homo sapiens GN=C3 PE=1 SV=2 - [CO3_HUMAN]	0.5
O00268	Transcription initiation factor TFIID subunit 4 OS=Homo sapiens GN=TAF4 PE=1 SV=2 - [TAF4_HUMAN]	0.5
P17612	cAMP-dependent protein kinase catalytic subunit alpha OS=Homo sapiens GN=PRKACA PE=1 SV=2 - [KAPCA_HUMAN]	0.5
P48740	Mannan-binding lectin serine protease 1 OS=Homo sapiens GN=MASP1 PE=1 SV=3 - [MASP1_HUMAN]	0.5
Q9UH99	SUN domain-containing protein 2 OS=Homo sapiens GN=SUN2 PE=1 SV=3 - [SUN2_HUMAN]	0.5
Q8IVS2	Malonyl-CoA-acyl carrier protein transacylase, mitochondrial OS=Homo sapiens GN=MCAT PE=1 SV=2 - [FABD_HUMAN]	0.5
P27694	Replication protein A 70 kDa DNA-binding subunit OS=Homo sapiens GN=RPA1 PE=1 SV=2 - [RFA1_HUMAN]	0.5
Q93052	Lipoma-preferred partner OS=Homo sapiens GN=LPP PE=1 SV=1 - [LPP_HUMAN]	0.5
Q9Y3T6	R3H and coiled-coil domain-containing protein 1 OS=Homo sapiens GN=R3HCC1 PE=1 SV=2 - [R3HC1_HUMAN]	0.5
O95104	Splicing factor, arginine/serine-rich 15 OS=Homo sapiens GN=SCAF4 PE=1 SV=3 - [SFR15_HUMAN]	0.5
O14939	Phospholipase D2 OS=Homo sapiens GN=PLD2 PE=1 SV=2 - [PLD2_HUMAN]	0.5
Q9NUL5	UPF0515 protein C19orf66 OS=Homo sapiens GN=C19orf66 PE=1 SV=2 - [CS066_HUMAN]	0.5
Q8IY33	MICAL-like protein 2 OS=Homo sapiens GN=MICALL2 PE=1 SV=1 - [MILK2_HUMAN]	0.5
Q99685	Monoglyceride lipase OS=Homo sapiens GN=MGLL PE=1 SV=2 - [MGLL_HUMAN]	0.5
Q9UKU7	Isobutyryl-CoA dehydrogenase, mitochondrial OS=Homo sapiens GN=ACAD8 PE=1 SV=1 - [ACAD8_HUMAN]	0.5
Q15345	Leucine-rich repeat-containing protein 41 OS=Homo sapiens GN=LRRC41 PE=1 SV=3 - [LRC41_HUMAN]	0.5
O60284	Suppression of tumorigenicity 18 protein OS=Homo sapiens GN=ST18 PE=1 SV=1 - [ST18_HUMAN]	0.4
P11234	Ras-related protein Ral-B OS=Homo sapiens GN=RALB PE=1 SV=1 - [RALB_HUMAN]	0.4
Q2TAZ0	Autophagy-related protein 2 homolog A OS=Homo sapiens GN=ATG2A PE=1 SV=3 - [ATG2A_HUMAN]	0.4
Q8TAQ2	SWI/SNF complex subunit SMARCC2 OS=Homo sapiens GN=SMARCC2 PE=1 SV=1 - [SMRC2_HUMAN]	0.4
Q9NXV6	CDKN2A-interacting protein OS=Homo sapiens GN=CDKN2AIP PE=1 SV=3 - [CARF_HUMAN]	0.4
Q6UW68	Transmembrane protein 205 OS=Homo sapiens GN=TMEM205 PE=1 SV=1 - [TM205_HUMAN]	0.4
Q9H0E9	Bromodomain-containing protein 8 OS=Homo sapiens GN=BRD8 PE=1 SV=2 - [BRD8_HUMAN]	0.4
O15116	U6 snRNA-associated Sm-like protein LSm1 OS=Homo sapiens GN=LSM1 PE=1 SV=1 - [LSM1_HUMAN]	0.4
Q96JI7	Spatacsin OS=Homo sapiens GN=SPG11 PE=1 SV=3 - [SPTCS_HUMAN]	0.4
Q8TDB6	E3 ubiquitin-protein ligase DTX3L OS=Homo sapiens GN=DTX3L PE=1 SV=1 - [DTX3L_HUMAN]	0.4
P53814	Smoothelin OS=Homo sapiens GN=SMTN PE=1 SV=7 - [SMTN_HUMAN]	0.4
Q9ULE3	DENN domain-containing protein 2A OS=Homo sapiens GN=DENND2A PE=2 SV=4 - [DEN2A_HUMAN]	0.4
P09848	Lactase-phlorizin hydrolase OS=Homo sapiens GN=LCT PE=1 SV=3 - [LPH_HUMAN]	0.4
Q7LBR1	Charged multivesicular body protein 1b OS=Homo sapiens GN=CHMP1B PE=1 SV=1 - [CHM1B_HUMAN]	0.4
O43823	A-kinase anchor protein 8 OS=Homo sapiens GN=AKAP8 PE=1 SV=1 - [AKAP8_HUMAN]	0.4
P13984	General transcription factor IIF subunit 2 OS=Homo sapiens GN=GTF2F2 PE=1 SV=2 - [T2FB_HUMAN]	0.4
Q9BUL5	PHD finger protein 23 OS=Homo sapiens GN=PHF23 PE=1 SV=1 - [PHF23_HUMAN]	0.4
Q9NQT8	Kinesin-like protein KIF13B OS=Homo sapiens GN=KIF13B PE=1 SV=2 - [KI13B_HUMAN]	0.4
P48454	Serine/threonine-protein phosphatase 2B catalytic subunit gamma isoform OS=Homo sapiens GN=PPP3CC PE=1 SV=3 - [PP2BC_HUMAN]	0.4
Q9UDY2	Tight junction protein ZO-2 OS=Homo sapiens GN=TJP2 PE=1 SV=2 - [ZO2_HUMAN]	0.4
Q86WV6	Stimulator of interferon genes protein OS=Homo sapiens GN=TMEM173 PE=1 SV=1 - [STING_HUMAN]	0.4
O60885	Bromodomain-containing protein 4 OS=Homo sapiens GN=BRD4 PE=1 SV=2 - [BRD4_HUMAN]	0.4

Q9H3P2	Negative elongation factor A OS=Homo sapiens GN=NELFA PE=1 SV=3 - [NELFA_HUMAN]	0.4
P49407	Beta-arrestin-1 OS=Homo sapiens GN=ARRB1 PE=1 SV=2 - [ARRB1_HUMAN]	0.4
Q96ME7	Zinc finger protein 512 OS=Homo sapiens GN=ZNF512 PE=1 SV=2 - [ZN512_HUMAN]	0.4
Q7RTV0	PHD finger-like domain-containing protein 5A OS=Homo sapiens GN=PHF5A PE=1 SV=1 - [PHF5A_HUMAN]	0.4
P05166	Propionyl-CoA carboxylase beta chain, mitochondrial OS=Homo sapiens GN=PCCB PE=1 SV=3 - [PCCB_HUMAN]	0.4
P33316	Deoxyuridine 5'-triphosphate nucleotidohydrolase, mitochondrial OS=Homo sapiens GN=DUT PE=1 SV=4 - [DUT_HUMAN]	0.4
Q9NRA8	Eukaryotic translation initiation factor 4E transporter OS=Homo sapiens GN=EIF4ENIF1 PE=1 SV=2 - [4ET_HUMAN]	0.4
Q16539	Mitogen-activated protein kinase 14 OS=Homo sapiens GN=MAPK14 PE=1 SV=3 - [MK14_HUMAN]	0.4
Q9C0J8	pre-mRNA 3' end processing protein WDR33 OS=Homo sapiens GN=WDR33 PE=1 SV=2 - [WDR33_HUMAN]	0.4
Q01658	Protein Dr1 OS=Homo sapiens GN=DR1 PE=1 SV=1 - [NC2B_HUMAN]	0.4
Q13185	Chromobox protein homolog 3 OS=Homo sapiens GN=CBX3 PE=1 SV=4 - [CBX3_HUMAN]	0.4
A6NKD9	Coiled-coil domain-containing protein 85C OS=Homo sapiens GN=CCDC85C PE=1 SV=1 - [CC85C_HUMAN]	0.4
Q12857	Nuclear factor 1 A-type OS=Homo sapiens GN=NFIA PE=1 SV=2 - [NFIA_HUMAN]	0.4
Q6P587	Acylpyruvase FAHD1, mitochondrial OS=Homo sapiens GN=FAHD1 PE=1 SV=2 - [FAHD1_HUMAN]	0.4
P30533	Alpha-2-macroglobulin receptor-associated protein OS=Homo sapiens GN=LRPAP1 PE=1 SV=1 - [AMRP_HUMAN]	0.4
Q96GY0	Zinc finger C2HC domain-containing protein 1A OS=Homo sapiens GN=ZC2HC1A PE=1 SV=2 - [ZC21A_HUMAN]	0.4
Q63ZY3	KN motif and ankyrin repeat domain-containing protein 2 OS=Homo sapiens GN=KANK2 PE=1 SV=1 - [KANK2_HUMAN]	0.4
Q9P206	Uncharacterized protein KIAA1522 OS=Homo sapiens GN=KIAA1522 PE=1 SV=2 - [K1522_HUMAN]	0.4
O00154	Cytosolic acyl coenzyme A thioester hydrolase OS=Homo sapiens GN=ACOT7 PE=1 SV=3 - [BACH_HUMAN]	0.4
Q8IW45	ATP-dependent (S)-NAD(P)H-hydrate dehydratase OS=Homo sapiens GN=CARKD PE=1 SV=1 - [NNRD_HUMAN]	0.4
Q9C0E8	Protein lunapark OS=Homo sapiens GN=LNP PE=1 SV=2 - [LNP_HUMAN]	0.4
Q9NRS6	Sorting nexin-15 OS=Homo sapiens GN=SNX15 PE=1 SV=1 - [SNX15_HUMAN]	0.4
O00151	PDZ and LIM domain protein 1 OS=Homo sapiens GN=PDLIM1 PE=1 SV=4 - [PDLI1_HUMAN]	0.4
P29466	Caspase-1 OS=Homo sapiens GN=CASP1 PE=1 SV=1 - [CASP1_HUMAN]	0.4
Q96MU7	YTH domain-containing protein 1 OS=Homo sapiens GN=YTHDC1 PE=1 SV=3 - [YTDC1_HUMAN]	0.4
Q86U90	YrdC domain-containing protein, mitochondrial OS=Homo sapiens GN=YRDC PE=1 SV=1 - [YRDC_HUMAN]	0.4
Q9H019	Mitochondrial fission regulator 1-like OS=Homo sapiens GN=MTFR1L PE=1 SV=2 - [MFR1L_HUMAN]	0.4
O95400	CD2 antigen cytoplasmic tail-binding protein 2 OS=Homo sapiens GN=CD2BP2 PE=1 SV=1 - [CD2B2_HUMAN]	0.4
Q2M2I5	Keratin, type I cytoskeletal 24 OS=Homo sapiens GN=KRT24 PE=1 SV=1 - [K1C24_HUMAN]	0.4
P78316	Nucleolar protein 14 OS=Homo sapiens GN=NOP14 PE=1 SV=3 - [NOP14_HUMAN]	0.4
P98179	Putative RNA-binding protein 3 OS=Homo sapiens GN=RBM3 PE=1 SV=1 - [RBM3_HUMAN]	0.4
Q06330	Recombining binding protein suppressor of hairless OS=Homo sapiens GN=RBPJ PE=1 SV=3 - [SUH_HUMAN]	0.4
Q15172	Serine/threonine-protein phosphatase 2A 56 kDa regulatory subunit alpha isoform OS=Homo sapiens GN=PPP2R5A PE=1 SV=1 - [2A5A_HUMAN]	0.4
Q9Y530	O-acetyl-ADP-ribose deacetylase 1 OS=Homo sapiens GN=OARD1 PE=1 SV=2 - [OARD1_HUMAN]	0.4
O75151	Lysine-specific demethylase PHF2 OS=Homo sapiens GN=PHF2 PE=1 SV=4 - [PHF2_HUMAN]	0.4
P35249	Replication factor C subunit 4 OS=Homo sapiens GN=RFC4 PE=1 SV=2 - [RFC4_HUMAN]	0.4
P84103	Serine/arginine-rich splicing factor 3 OS=Homo sapiens GN=SRSF3 PE=1 SV=1 - [SRSF3_HUMAN]	0.4
P37275	Zinc finger E-box-binding homeobox 1 OS=Homo sapiens GN=ZEB1 PE=1 SV=2 - [ZEB1_HUMAN]	0.4
O75475	PC4 and SFRS1-interacting protein OS=Homo sapiens GN=PSIP1 PE=1 SV=1 - [PSIP1_HUMAN]	0.4
Q04725	Transducin-like enhancer protein 2 OS=Homo sapiens GN=TLE2 PE=1 SV=2 - [TLE2_HUMAN]	0.4
Q9UPT8	Zinc finger CCCH domain-containing protein 4 OS=Homo sapiens GN=ZC3H4 PE=1 SV=3 - [ZC3H4_HUMAN]	0.4
P60903	Protein S100-A10 OS=Homo sapiens GN=S100A10 PE=1 SV=2 - [S10AA_HUMAN]	0.4
P55081	Microfibrillar-associated protein 1 OS=Homo sapiens GN=MFAP1 PE=1 SV=2 - [MFAP1_HUMAN]	0.4
O75208	Ubiquinone biosynthesis protein COQ9, mitochondrial OS=Homo sapiens GN=COQ9 PE=1 SV=1 - [COQ9_HUMAN]	0.4
Q8NFV4	Alpha/beta hydrolase domain-containing protein 11 OS=Homo sapiens GN=ABHD11 PE=2 SV=1 - [ABHDB_HUMAN]	0.4
O75746	Calcium-binding mitochondrial carrier protein Aralar1 OS=Homo sapiens GN=SLC25A12 PE=1 SV=2 - [CMC1_HUMAN]	0.4
Q460N5	Poly [ADP-ribose] polymerase 14 OS=Homo sapiens GN=PARP14 PE=1 SV=3 - [PAR14_HUMAN]	0.4
P50613	Cyclin-dependent kinase 7 OS=Homo sapiens GN=CDK7 PE=1 SV=1 - [CDK7_HUMAN]	0.4
O95757	Heat shock 70 kDa protein 4L OS=Homo sapiens GN=HSPA4L PE=1 SV=3 - [HS74L_HUMAN]	0.4
Q8N5M4	Tetratricopeptide repeat protein 9C OS=Homo sapiens GN=TTC9C PE=1 SV=1 - [TTC9C_HUMAN]	0.4
P62081	40S ribosomal protein S7 OS=Homo sapiens GN=RPS7 PE=1 SV=1 - [RS7_HUMAN]	0.4
Q13625	Apoptosis-stimulating of p53 protein 2 OS=Homo sapiens GN=TP53BP2 PE=1 SV=2 - [ASPP2_HUMAN]	0.4
Q1KMD3	Heterogeneous nuclear ribonucleoprotein U-like protein 2 OS=Homo sapiens GN=HNRNPUL2 PE=1 SV=1 - [HNRL2_HUMAN]	0.4

Q8N3E9	1-phosphatidylinositol 4,5-bisphosphate phosphodiesterase delta-3 OS=Homo sapiens GN=PLCD3 PE=1 SV=3 - [PLCD3_HUMAN]	0.4
Q13555	Calcium/calmodulin-dependent protein kinase type II subunit gamma OS=Homo sapiens GN=CAMK2G PE=1 SV=3 - [KCC2G_HUMAN]	0.4
O14773	Tripeptidyl-peptidase 1 OS=Homo sapiens GN=TPP1 PE=1 SV=2 - [TPP1_HUMAN]	0.4
P50336	Protoporphyrinogen oxidase OS=Homo sapiens GN=PPOX PE=1 SV=1 - [PPOX_HUMAN]	0.4
Q9BTE3	Mini-chromosome maintenance complex-binding protein OS=Homo sapiens GN=MCMBP PE=1 SV=2 - [MCMBP_HUMAN]	0.4
Q7Z7K0	COX assembly mitochondrial protein homolog OS=Homo sapiens GN=CMC1 PE=1 SV=1 - [COXM1_HUMAN]	0.4
Q13228	Selenium-binding protein 1 OS=Homo sapiens GN=SELENBP1 PE=1 SV=2 - [SBP1_HUMAN]	0.4
Q7Z7A4	PX domain-containing protein kinase-like protein OS=Homo sapiens GN=PXK PE=1 SV=1 - [PXK_HUMAN]	0.4
Q00653	Nuclear factor NF-kappa-B p100 subunit OS=Homo sapiens GN=NFKB2 PE=1 SV=4 - [NFKB2_HUMAN]	0.4
P02768	Serum albumin OS=Homo sapiens GN=ALB PE=1 SV=2 - [ALBU_HUMAN]	0.4
Q13905	Rap guanine nucleotide exchange factor 1 OS=Homo sapiens GN=RAPGEF1 PE=1 SV=3 - [RPGF1_HUMAN]	0.4
Q7Z6K5	Arpin OS=Homo sapiens GN=ARPIN PE=1 SV=1 - [ARPIN_HUMAN]	0.4
P16401	Histone H1.5 OS=Homo sapiens GN=HIST1H1B PE=1 SV=3 - [H15_HUMAN]	0.4
Q9NP92	28S ribosomal protein S30, mitochondrial OS=Homo sapiens GN=MRPS30 PE=1 SV=2 - [RT30_HUMAN]	0.4
P43304	Glycerol-3-phosphate dehydrogenase, mitochondrial OS=Homo sapiens GN=GPD2 PE=1 SV=3 - [GPDM_HUMAN]	0.4
Q15126	Phosphomevalonate kinase OS=Homo sapiens GN=PMVK PE=1 SV=3 - [PMVK_HUMAN]	0.4
O14920	Inhibitor of nuclear factor kappa-B kinase subunit beta OS=Homo sapiens GN=IKBKB PE=1 SV=1 - [IKKB_HUMAN]	0.4
Q8N6S5	ADP-ribosylation factor-like protein 6-interacting protein 6 OS=Homo sapiens GN=ARL6IP6 PE=1 SV=1 - [AR6P6_HUMAN]	0.4
O95571	Persulfide dioxygenase ETHE1, mitochondrial OS=Homo sapiens GN=ETHE1 PE=1 SV=2 - [ETHE1_HUMAN]	0.4
P08107	Heat shock 70 kDa protein 1A/1B OS=Homo sapiens GN=HSPA1A PE=1 SV=5 - [HSP71_HUMAN]	0.4
O14745	Na(+)/H(+) exchange regulatory cofactor NHE-RF1 OS=Homo sapiens GN=SLC9A3R1 PE=1 SV=4 - [NHRF1_HUMAN]	0.4
Q06787	Fragile X mental retardation protein 1 OS=Homo sapiens GN=FMR1 PE=1 SV=1 - [FMR1_HUMAN]	0.4
Q15262	Receptor-type tyrosine-protein phosphatase kappa OS=Homo sapiens GN=PTPRK PE=1 SV=2 - [PTPRK_HUMAN]	0.4
Q9NZN4	EH domain-containing protein 2 OS=Homo sapiens GN=EHD2 PE=1 SV=2 - [EHD2_HUMAN]	0.4
Q8N163	Cell cycle and apoptosis regulator protein 2 OS=Homo sapiens GN=CCAR2 PE=1 SV=2 - [CCAR2_HUMAN]	0.4
O75382	Tripartite motif-containing protein 3 OS=Homo sapiens GN=TRIM3 PE=1 SV=2 - [TRIM3_HUMAN]	0.4
Q8IXS6	Paralemmin-2 OS=Homo sapiens GN=PALM2 PE=2 SV=3 - [PALM2_HUMAN]	0.4
Q96CB8	Integrator complex subunit 12 OS=Homo sapiens GN=INTS12 PE=1 SV=1 - [INT12_HUMAN]	0.4
P09874	Poly [ADP-ribose] polymerase 1 OS=Homo sapiens GN=PARP1 PE=1 SV=4 - [PARP1_HUMAN]	0.4
P51610	Host cell factor 1 OS=Homo sapiens GN=HCFC1 PE=1 SV=2 - [HCFC1_HUMAN]	0.4
Q96E11	Ribosome-recycling factor, mitochondrial OS=Homo sapiens GN=MRRF PE=1 SV=1 - [RRFM_HUMAN]	0.4
P55854	Small ubiquitin-related modifier 3 OS=Homo sapiens GN=SUMO3 PE=1 SV=2 - [SUMO3_HUMAN]	0.4
Q16762	Thiosulfate sulfurtransferase OS=Homo sapiens GN=TST PE=1 SV=4 - [THTR_HUMAN]	0.3
Q9NTJ4	Alpha-mannosidase 2C1 OS=Homo sapiens GN=MAN2C1 PE=1 SV=1 - [MA2C1_HUMAN]	0.3
Q96T60	Bifunctional polynucleotide phosphatase/kinase OS=Homo sapiens GN=PNKP PE=1 SV=1 - [PNKP_HUMAN]	0.3
Q53FA7	Quinone oxidoreductase PIG3 OS=Homo sapiens GN=TP53I3 PE=1 SV=2 - [QORX_HUMAN]	0.3
Q9NYB9	Abl interactor 2 OS=Homo sapiens GN=ABI2 PE=1 SV=1 - [ABI2_HUMAN]	0.3
P78357	Contactin-associated protein 1 OS=Homo sapiens GN=CNTNAP1 PE=1 SV=1 - [CNTP1_HUMAN]	0.3
O00635	E3 ubiquitin-protein ligase TRIM38 OS=Homo sapiens GN=TRIM38 PE=1 SV=1 - [TRI38_HUMAN]	0.3
O15121	Sphingolipid delta(4)-desaturase DES1 OS=Homo sapiens GN=DEGS1 PE=1 SV=1 - [DEGS1_HUMAN]	0.3
Q9UKV3	Apoptotic chromatin condensation inducer in the nucleus OS=Homo sapiens GN=ACIN1 PE=1 SV=2 - [ACINU_HUMAN]	0.3
P33992	DNA replication licensing factor MCM5 OS=Homo sapiens GN=MCM5 PE=1 SV=5 - [MCM5_HUMAN]	0.3
Q9Y3E2	BolA-like protein 1 OS=Homo sapiens GN=BOLA1 PE=1 SV=1 - [BOLA1_HUMAN]	0.3
P54259	Atrophin-1 OS=Homo sapiens GN=ATN1 PE=1 SV=3 - [ATN1_HUMAN]	0.3
O75367	Core histone macro-H2A.1 OS=Homo sapiens GN=H2AFY PE=1 SV=4 - [H2AY_HUMAN]	0.3
P48506	Glutamate--cysteine ligase catalytic subunit OS=Homo sapiens GN=GCLC PE=1 SV=2 - [GSH1_HUMAN]	0.3
O75676	Ribosomal protein S6 kinase alpha-4 OS=Homo sapiens GN=RPS6KA4 PE=1 SV=1 - [KS6A4_HUMAN]	0.3
P50747	Biotin--protein ligase OS=Homo sapiens GN=HLCS PE=1 SV=1 - [BPL1_HUMAN]	0.3
P10914	Interferon regulatory factor 1 OS=Homo sapiens GN=IRF1 PE=1 SV=2 - [IRF1_HUMAN]	0.3
P23497	Nuclear autoantigen Sp-100 OS=Homo sapiens GN=SP100 PE=1 SV=3 - [SP100_HUMAN]	0.3
P49750	YLP motif-containing protein 1 OS=Homo sapiens GN=YLPM1 PE=1 SV=3 - [YLPM1_HUMAN]	0.3
Q9BVL4	Selenoprotein O OS=Homo sapiens GN=SELO PE=2 SV=3 - [SELO_HUMAN]	0.3

Q7L4I2	Arginine/serine-rich coiled-coil protein 2 OS=Homo sapiens GN=RSRC2 PE=1 SV=1 - [RSRC2_HUMAN]	0.3
P62917	60S ribosomal protein L8 OS=Homo sapiens GN=RPL8 PE=1 SV=2 - [RL8_HUMAN]	0.3
O60879	Protein diaphanous homolog 2 OS=Homo sapiens GN=DIAPH2 PE=1 SV=1 - [DIAP2_HUMAN]	0.3
Q9NNW7	Thioredoxin reductase 2, mitochondrial OS=Homo sapiens GN=TXNRD2 PE=1 SV=3 - [TRXR2_HUMAN]	0.3
Q9HAU0	Pleckstrin homology domain-containing family A member 5 OS=Homo sapiens GN=PLEKHA5 PE=1 SV=1 - [PKHA5_HUMAN]	0.3
Q9H147	Deoxynucleotidyltransferase terminal-interacting protein 1 OS=Homo sapiens GN=DNTTIP1 PE=1 SV=2 - [TDIF1_HUMAN]	0.3
Q92466	DNA damage-binding protein 2 OS=Homo sapiens GN=DDB2 PE=1 SV=1 - [DDB2_HUMAN]	0.3
Q15746	Myosin light chain kinase, smooth muscle OS=Homo sapiens GN=MYLK PE=1 SV=4 - [MYLK_HUMAN]	0.3
P16949	Stathmin OS=Homo sapiens GN=STMN1 PE=1 SV=3 - [STMN1_HUMAN]	0.3
P39748	Flap endonuclease 1 OS=Homo sapiens GN=FEN1 PE=1 SV=1 - [FEN1_HUMAN]	0.3
P49321	Nuclear autoantigenic sperm protein OS=Homo sapiens GN=NASP PE=1 SV=2 - [NASP_HUMAN]	0.3
Q5T200	Zinc finger CCCH domain-containing protein 13 OS=Homo sapiens GN=ZC3H13 PE=1 SV=1 - [ZC3HD_HUMAN]	0.3
Q03135	Caveolin-1 OS=Homo sapiens GN=CAV1 PE=1 SV=4 - [CAV1_HUMAN]	0.3
Q8WWL7	G2/mitotic-specific cyclin-B3 OS=Homo sapiens GN=CCNB3 PE=1 SV=2 - [CCNB3_HUMAN]	0.3
Q8WX93	Palladin OS=Homo sapiens GN=PALLD PE=1 SV=3 - [PALLD_HUMAN]	0.3
Q10570	Cleavage and polyadenylation specificity factor subunit 1 OS=Homo sapiens GN=CPSF1 PE=1 SV=2 - [CPSF1_HUMAN]	0.3
P07910	Heterogeneous nuclear ribonucleoproteins C1/C2 OS=Homo sapiens GN=HNRNPC PE=1 SV=4 - [HNRPC_HUMAN]	0.3
Q14683	Structural maintenance of chromosomes protein 1A OS=Homo sapiens GN=SMC1A PE=1 SV=2 - [SMC1A_HUMAN]	0.3
P31937	3-hydroxyisobutyrate dehydrogenase, mitochondrial OS=Homo sapiens GN=HIBADH PE=1 SV=2 - [3HIDH_HUMAN]	0.3
Q92841	Probable ATP-dependent RNA helicase DDX17 OS=Homo sapiens GN=DDX17 PE=1 SV=2 - [DDX17_HUMAN]	0.3
Q96P47	Arf-GAP with GTPase, ANK repeat and PH domain-containing protein 3 OS=Homo sapiens GN=AGAP3 PE=1 SV=2 - [AGAP3_HUMAN]	0.3
Q9P2N5	RNA-binding protein 27 OS=Homo sapiens GN=RBM27 PE=1 SV=2 - [RBM27_HUMAN]	0.3
Q7Z6B0	Coiled-coil domain-containing protein 91 OS=Homo sapiens GN=CCDC91 PE=1 SV=2 - [CCD91_HUMAN]	0.3
Q9UQ03	Coronin-2B OS=Homo sapiens GN=CORO2B PE=2 SV=4 - [COR2B_HUMAN]	0.3
Q8NFD5	AT-rich interactive domain-containing protein 1B OS=Homo sapiens GN=ARID1B PE=1 SV=2 - [ARI1B_HUMAN]	0.3
Q92614	Unconventional myosin-XVIIIa OS=Homo sapiens GN=MYO18A PE=1 SV=3 - [MY18A_HUMAN]	0.3
Q8WUA2	Peptidyl-prolyl cis-trans isomerase-like 4 OS=Homo sapiens GN=PPIL4 PE=1 SV=1 - [PPIL4_HUMAN]	0.3
Q13315	Serine-protein kinase ATM OS=Homo sapiens GN=ATM PE=1 SV=4 - [ATM_HUMAN]	0.3
Q9NRX1	RNA-binding protein PNO1 OS=Homo sapiens GN=PNO1 PE=1 SV=1 - [PNO1_HUMAN]	0.3
P63279	SUMO-conjugating enzyme UBC9 OS=Homo sapiens GN=UBE2I PE=1 SV=1 - [UBC9_HUMAN]	0.3
P50897	Palmitoyl-protein thioesterase 1 OS=Homo sapiens GN=PPT1 PE=1 SV=1 - [PPT1_HUMAN]	0.3
Q86TB9	Protein PAT1 homolog 1 OS=Homo sapiens GN=PATL1 PE=1 SV=2 - [PATL1_HUMAN]	0.3
P35270	Sepiapterin reductase OS=Homo sapiens GN=SPR PE=1 SV=1 - [SPRE_HUMAN]	0.3
O75410	Transforming acidic coiled-coil-containing protein 1 OS=Homo sapiens GN=TACC1 PE=1 SV=2 - [TACC1_HUMAN]	0.3
P43490	Nicotinamide phosphoribosyltransferase OS=Homo sapiens GN=NAMPT PE=1 SV=1 - [NAMPT_HUMAN]	0.3
Q9Y4G8	Rap guanine nucleotide exchange factor 2 OS=Homo sapiens GN=RAPGEF2 PE=1 SV=1 - [RPGF2_HUMAN]	0.3
P22570	NADPH:adrenodoxin oxidoreductase, mitochondrial OS=Homo sapiens GN=FDXR PE=1 SV=3 - [ADRO_HUMAN]	0.3
P37198	Nuclear pore glycoprotein p62 OS=Homo sapiens GN=NUP62 PE=1 SV=3 - [NUP62_HUMAN]	0.3
Q5VTR2	E3 ubiquitin-protein ligase BRE1A OS=Homo sapiens GN=RNF20 PE=1 SV=2 - [BRE1A_HUMAN]	0.3
Q8IZA0	Dyslexia-associated protein KIAA0319-like protein OS=Homo sapiens GN=KIAA0319L PE=1 SV=2 - [K319L_HUMAN]	0.3
P51608	Methyl-CpG-binding protein 2 OS=Homo sapiens GN=MECP2 PE=1 SV=1 - [MECP2_HUMAN]	0.3
Q86V81	THO complex subunit 4 OS=Homo sapiens GN=ALYREF PE=1 SV=3 - [THOC4_HUMAN]	0.3
Q96HP0	Dedicator of cytokinesis protein 6 OS=Homo sapiens GN=DOCK6 PE=1 SV=3 - [DOCK6_HUMAN]	0.3
P08236	Beta-glucuronidase OS=Homo sapiens GN=GUSB PE=1 SV=2 - [BGLR_HUMAN]	0.3
Q5TFE4	5'-nucleotidase domain-containing protein 1 OS=Homo sapiens GN=NT5DC1 PE=1 SV=1 - [NT5D1_HUMAN]	0.3
P49137	MAP kinase-activated protein kinase 2 OS=Homo sapiens GN=MAPKAPK2 PE=1 SV=1 - [MAPK2_HUMAN]	0.3
Q14151	Scaffold attachment factor B2 OS=Homo sapiens GN=SAFB2 PE=1 SV=1 - [SAFB2_HUMAN]	0.3
Q9H2P0	Activity-dependent neuroprotector homeobox protein OS=Homo sapiens GN=ADNP PE=1 SV=1 - [ADNP_HUMAN]	0.3
Q9NZ32	Actin-related protein 10 OS=Homo sapiens GN=ACTR10 PE=1 SV=1 - [ARP10_HUMAN]	0.3
Q92917	G patch domain and KOW motifs-containing protein OS=Homo sapiens GN=GPKOW PE=1 SV=2 - [GPKOW_HUMAN]	0.3
Q15637	Splicing factor 1 OS=Homo sapiens GN=SF1 PE=1 SV=4 - [SF01_HUMAN]	0.3
Q14103	Heterogeneous nuclear ribonucleoprotein D0 OS=Homo sapiens GN=HNRNPD PE=1 SV=1 - [HNRPD_HUMAN]	0.3
Q9H0D6	5'-3' exoribonuclease 2 OS=Homo sapiens GN=XRN2 PE=1 SV=1 - [XRN2_HUMAN]	0.3

Q13123	Protein Red OS=Homo sapiens GN=IK PE=1 SV=3 - [RED_HUMAN]	0.3
Q13573	SNW domain-containing protein 1 OS=Homo sapiens GN=SNW1 PE=1 SV=1 - [SNW1_HUMAN]	0.3
Q5BKZ1	DBIRD complex subunit ZNF326 OS=Homo sapiens GN=ZNF326 PE=1 SV=2 - [ZN326_HUMAN]	0.3
P36551	Oxygen-dependent coproporphyrinogen-III oxidase, mitochondrial OS=Homo sapiens GN=CPOX PE=1 SV=3 - [HEM6_HUMAN]	0.3
P42167	Lamina-associated polypeptide 2, isoforms beta/gamma OS=Homo sapiens GN=TMPO PE=1 SV=2 - [LAP2B_HUMAN]	0.3
P19474	E3 ubiquitin-protein ligase TRIM21 OS=Homo sapiens GN=TRIM21 PE=1 SV=1 - [RO52_HUMAN]	0.3
Q9Y4F1	FERM, RhoGEF and pleckstrin domain-containing protein 1 OS=Homo sapiens GN=FARP1 PE=1 SV=1 - [FARP1_HUMAN]	0.3
Q5VT52	Regulation of nuclear pre-mRNA domain-containing protein 2 OS=Homo sapiens GN=RPRD2 PE=1 SV=1 - [RPRD2_HUMAN]	0.3
P14678	Small nuclear ribonucleoprotein-associated proteins B and B' OS=Homo sapiens GN=SNRPB PE=1 SV=2 - [RSMB_HUMAN]	0.3
Q8N5G2	Macoilin OS=Homo sapiens GN=TMEM57 PE=1 SV=1 - [MACOI_HUMAN]	0.3
P05165	Propionyl-CoA carboxylase alpha chain, mitochondrial OS=Homo sapiens GN=PCCA PE=1 SV=4 - [PCCA_HUMAN]	0.3
P52597	Heterogeneous nuclear ribonucleoprotein F OS=Homo sapiens GN=HNRNPF PE=1 SV=3 - [HNRPF_HUMAN]	0.3
P02545	Prelamin-A/C OS=Homo sapiens GN=LMNA PE=1 SV=1 - [LMNA_HUMAN]	0.3
Q9UIG0	Tyrosine-protein kinase BAZ1B OS=Homo sapiens GN=BAZ1B PE=1 SV=2 - [BAZ1B_HUMAN]	0.3
O60264	SWI/SNF-related matrix-associated actin-dependent regulator of chromatin subfamily A member 5 OS=Homo sapiens GN=SMARCA5 PE=1 SV=1 - [SMCA5_HUMAN]	0.3
P29590	Protein PML OS=Homo sapiens GN=PML PE=1 SV=3 - [PML_HUMAN]	0.3
P61604	10 kDa heat shock protein, mitochondrial OS=Homo sapiens GN=HSPE1 PE=1 SV=2 - [CH10_HUMAN]	0.3
O14578	Citron Rho-interacting kinase OS=Homo sapiens GN=CIT PE=1 SV=2 - [CTRO_HUMAN]	0.3
Q03519	Antigen peptide transporter 2 OS=Homo sapiens GN=TAP2 PE=1 SV=1 - [TAP2_HUMAN]	0.3
P00352	Retinal dehydrogenase 1 OS=Homo sapiens GN=ALDH1A1 PE=1 SV=2 - [AL1A1_HUMAN]	0.3
Q01844	RNA-binding protein EWS OS=Homo sapiens GN=EWSR1 PE=1 SV=1 - [EWS_HUMAN]	0.3
Q8N392	Rho GTPase-activating protein 18 OS=Homo sapiens GN=ARHGAP18 PE=1 SV=3 - [RHG18_HUMAN]	0.3
Q6PIU2	Neutral cholesterol ester hydrolase 1 OS=Homo sapiens GN=NCEH1 PE=1 SV=3 - [NCEH1_HUMAN]	0.3
P41229	Lysine-specific demethylase 5C OS=Homo sapiens GN=KDM5C PE=1 SV=2 - [KDM5C_HUMAN]	0.3
Q9UK61	Protein FAM208A OS=Homo sapiens GN=FAM208A PE=1 SV=3 - [F208A_HUMAN]	0.3
P49590	Probable histidine--tRNA ligase, mitochondrial OS=Homo sapiens GN=HARS2 PE=1 SV=1 - [SYHM_HUMAN]	0.3
Q96F46	Interleukin-17 receptor A OS=Homo sapiens GN=IL17RA PE=1 SV=2 - [I17RA_HUMAN]	0.3
Q5T9L3	Protein wntless homolog OS=Homo sapiens GN=WLS PE=1 SV=2 - [WLS_HUMAN]	0.3
P31943	Heterogeneous nuclear ribonucleoprotein H OS=Homo sapiens GN=HNRNPH1 PE=1 SV=4 - [HNRH1_HUMAN]	0.3
Q03518	Antigen peptide transporter 1 OS=Homo sapiens GN=TAP1 PE=1 SV=2 - [TAP1_HUMAN]	0.3
Q96EP5	DAZ-associated protein 1 OS=Homo sapiens GN=DAZAP1 PE=1 SV=1 - [DAZP1_HUMAN]	0.3
Q13393	Phospholipase D1 OS=Homo sapiens GN=PLD1 PE=1 SV=1 - [PLD1_HUMAN]	0.3
Q86V88	Magnesium-dependent phosphatase 1 OS=Homo sapiens GN=MDP1 PE=1 SV=1 - [MGDP1_HUMAN]	0.3
Q14966	Zinc finger protein 638 OS=Homo sapiens GN=ZNF638 PE=1 SV=2 - [ZN638_HUMAN]	0.3
Q96A32	Myosin regulatory light chain 2, skeletal muscle isoform OS=Homo sapiens GN=MYLPF PE=2 SV=1 - [MLRS_HUMAN]	0.3
P29401	Transketolase OS=Homo sapiens GN=TKT PE=1 SV=3 - [TKT_HUMAN]	0.3
Q9Y383	Putative RNA-binding protein Luc7-like 2 OS=Homo sapiens GN=LUC7L2 PE=1 SV=2 - [LC7L2_HUMAN]	0.3
Q5SYE7	NHS-like protein 1 OS=Homo sapiens GN=NHSL1 PE=1 SV=2 - [NHSL1_HUMAN]	0.3
Q9NWH9	SAFB-like transcription modulator OS=Homo sapiens GN=SLTM PE=1 SV=2 - [SLTM_HUMAN]	0.3
Q6P3W7	SCY1-like protein 2 OS=Homo sapiens GN=SCYL2 PE=1 SV=1 - [SCYL2_HUMAN]	0.3
Q15599	Na(+)/H(+) exchange regulatory cofactor NHE-RF2 OS=Homo sapiens GN=SLC9A3R2 PE=1 SV=2 - [NHRF2_HUMAN]	0.3
P61962	DDB1- and CUL4-associated factor 7 OS=Homo sapiens GN=DCAF7 PE=1 SV=1 - [DCAF7_HUMAN]	0.3
Q8IU81	Interferon regulatory factor 2-binding protein 1 OS=Homo sapiens GN=IRF2BP1 PE=1 SV=1 - [I2BP1_HUMAN]	0.3
Q9BUH6	Uncharacterized protein C9orf142 OS=Homo sapiens GN=C9orf142 PE=1 SV=2 - [CI142_HUMAN]	0.3
P57772	Selenocysteine-specific elongation factor OS=Homo sapiens GN=EEFSEC PE=1 SV=4 - [SELB_HUMAN]	0.3
Q12946	Forkhead box protein F1 OS=Homo sapiens GN=FOXF1 PE=1 SV=2 - [FOXF1_HUMAN]	0.3
Q13011	Delta(3,5)-Delta(2,4)-dienoyl-CoA isomerase, mitochondrial OS=Homo sapiens GN=ECH1 PE=1 SV=2 - [ECH1_HUMAN]	0.3
Q9UKM9	RNA-binding protein Raly OS=Homo sapiens GN=RALY PE=1 SV=1 - [RALY_HUMAN]	0.3
Q9Y2I1	Nischarin OS=Homo sapiens GN=NISCH PE=1 SV=3 - [NISCH_HUMAN]	0.3
P12694	2-oxoisovalerate dehydrogenase subunit alpha, mitochondrial OS=Homo sapiens GN=BCKDHA PE=1 SV=2 - [ODBA_HUMAN]	0.3
P48651	Phosphatidylserine synthase 1 OS=Homo sapiens GN=PTDSS1 PE=1 SV=1 - [PTSS1_HUMAN]	0.3
Q9NX46	Poly(ADP-ribose) glycohydrolase ARH3 OS=Homo sapiens GN=ADPRHL2 PE=1 SV=1 - [ARHL2_HUMAN]	0.3

O00754	Lysosomal alpha-mannosidase OS=Homo sapiens GN=MAN2B1 PE=1 SV=3 - [MA2B1_HUMAN]	0.3
Q96BP2	Coiled-coil-helix-coiled-coil-helix domain-containing protein 1 OS=Homo sapiens GN=CHCHD1 PE=1 SV=1 - [CHCH1_HUMAN]	0.3
O43488	Aflatoxin B1 aldehyde reductase member 2 OS=Homo sapiens GN=AKR7A2 PE=1 SV=3 - [ARK72_HUMAN]	0.3
Q7Z2K6	Endoplasmic reticulum metallopeptidase 1 OS=Homo sapiens GN=ERMP1 PE=1 SV=2 - [ERMP1_HUMAN]	0.3
Q16740	ATP-dependent Clp protease proteolytic subunit, mitochondrial OS=Homo sapiens GN=CLPP PE=1 SV=1 - [CLPP_HUMAN]	0.3
Q92817	Envoplakin OS=Homo sapiens GN=EVPL PE=1 SV=3 - [EVPL_HUMAN]	0.3
Q4G0J3	La-related protein 7 OS=Homo sapiens GN=LARP7 PE=1 SV=1 - [LARP7_HUMAN]	0.3
O75390	Citrate synthase, mitochondrial OS=Homo sapiens GN=CS PE=1 SV=2 - [CISY_HUMAN]	0.3
Q15334	Lethal(2) giant larvae protein homolog 1 OS=Homo sapiens GN=LLGL1 PE=1 SV=3 - [L2GL1_HUMAN]	0.3
Q92945	Far upstream element-binding protein 2 OS=Homo sapiens GN=KHSRP PE=1 SV=4 - [FUBP2_HUMAN]	0.3
P52815	39S ribosomal protein L12, mitochondrial OS=Homo sapiens GN=MRPL12 PE=1 SV=2 - [RM12_HUMAN]	0.3
Q9BSJ8	Extended synaptotagmin-1 OS=Homo sapiens GN=ESYT1 PE=1 SV=1 - [ESYT1_HUMAN]	0.3
P22087	rRNA 2'-O-methyltransferase fibrillarin OS=Homo sapiens GN=FBL PE=1 SV=2 - [FBRL_HUMAN]	0.3
Q9BZI7	Regulator of nonsense transcripts 3B OS=Homo sapiens GN=UPF3B PE=1 SV=1 - [REN3B_HUMAN]	0.3
P11441	Ubiquitin-like protein 4A OS=Homo sapiens GN=UBL4A PE=1 SV=1 - [UBL4A_HUMAN]	0.3
Q5RKV6	Exosome complex component MTR3 OS=Homo sapiens GN=EXOSC6 PE=1 SV=1 - [EXOS6_HUMAN]	0.3
Q6UVY6	DBH-like monooxygenase protein 1 OS=Homo sapiens GN=MOXD1 PE=2 SV=1 - [MOXD1_HUMAN]	0.3
Q9NR46	Endophilin-B2 OS=Homo sapiens GN=SH3GLB2 PE=1 SV=1 - [SHLB2_HUMAN]	0.3
Q9BUJ2	Heterogeneous nuclear ribonucleoprotein U-like protein 1 OS=Homo sapiens GN=HNRNPUL1 PE=1 SV=2 - [HNRL1_HUMAN]	0.3
P23610	Factor VIII intron 22 protein OS=Homo sapiens GN=F8A1 PE=1 SV=2 - [F8I2_HUMAN]	0.3
P55795	Heterogeneous nuclear ribonucleoprotein H2 OS=Homo sapiens GN=HNRNPH2 PE=1 SV=1 - [HNRH2_HUMAN]	0.3
Q96MK2	Protein FAM65C OS=Homo sapiens GN=FAM65C PE=2 SV=4 - [FA65C_HUMAN]	0.3
Q14031	Collagen alpha-6(IV) chain OS=Homo sapiens GN=COL4A6 PE=1 SV=3 - [CO4A6_HUMAN]	0.3
Q9Y3Y2	Chromatin target of PRMT1 protein OS=Homo sapiens GN=CHTOP PE=1 SV=2 - [CHTOP_HUMAN]	0.3
Q00403	Transcription initiation factor IIB OS=Homo sapiens GN=GTF2B PE=1 SV=1 - [TF2B_HUMAN]	0.3
P11498	Pyruvate carboxylase, mitochondrial OS=Homo sapiens GN=PC PE=1 SV=2 - [PYC_HUMAN]	0.3
Q9P015	39S ribosomal protein L15, mitochondrial OS=Homo sapiens GN=MRPL15 PE=1 SV=1 - [RM15_HUMAN]	0.3
P19387	DNA-directed RNA polymerase II subunit RPB3 OS=Homo sapiens GN=POLR2C PE=1 SV=2 - [RPB3_HUMAN]	0.3
Q03252	Lamin-B2 OS=Homo sapiens GN=LMNB2 PE=1 SV=3 - [LMNB2_HUMAN]	0.3
Q86VM9	Zinc finger CCCH domain-containing protein 18 OS=Homo sapiens GN=ZC3H18 PE=1 SV=2 - [ZCH18_HUMAN]	0.3
O43464	Serine protease HTRA2, mitochondrial OS=Homo sapiens GN=HTRA2 PE=1 SV=2 - [HTRA2_HUMAN]	0.3
Q00688	Peptidyl-prolyl cis-trans isomerase FKBP3 OS=Homo sapiens GN=FKBP3 PE=1 SV=1 - [FKBP3_HUMAN]	0.3
Q15233	Non-POU domain-containing octamer-binding protein OS=Homo sapiens GN=NONO PE=1 SV=4 - [NONO_HUMAN]	0.3
P13716	Delta-aminolevulinic acid dehydratase OS=Homo sapiens GN=ALAD PE=1 SV=1 - [HEM2_HUMAN]	0.3
O14936	Peripheral plasma membrane protein CASK OS=Homo sapiens GN=CASK PE=1 SV=3 - [CSKP_HUMAN]	0.3
P18887	DNA repair protein XRCC1 OS=Homo sapiens GN=XRCC1 PE=1 SV=2 - [XRCC1_HUMAN]	0.3
Q02218	2-oxoglutarate dehydrogenase, mitochondrial OS=Homo sapiens GN=OGDH PE=1 SV=3 - [ODO1_HUMAN]	0.3
Q14814	Myocyte-specific enhancer factor 2D OS=Homo sapiens GN=MEF2D PE=1 SV=1 - [MEF2D_HUMAN]	0.3
P23246	Splicing factor, proline- and glutamine-rich OS=Homo sapiens GN=SFPQ PE=1 SV=2 - [SFPQ_HUMAN]	0.3
P28838	Cytosol aminopeptidase OS=Homo sapiens GN=LAP3 PE=1 SV=3 - [AMPL_HUMAN]	0.3
Q6NZI2	Polymerase I and transcript release factor OS=Homo sapiens GN=PTRF PE=1 SV=1 - [PTRF_HUMAN]	0.3
Q9BZZ5	Apoptosis inhibitor 5 OS=Homo sapiens GN=API5 PE=1 SV=3 - [API5_HUMAN]	0.3
Q16629	Serine/arginine-rich splicing factor 7 OS=Homo sapiens GN=SRSF7 PE=1 SV=1 - [SRSF7_HUMAN]	0.3
Q969G3	SWI/SNF-related matrix-associated actin-dependent regulator of chromatin subfamily E member 1 OS=Homo sapiens GN=SMARCE1 PE=1 SV=2 - [SMCE1_HUMAN]	0.3
Q9HB07	UPF0160 protein MYG1, mitochondrial OS=Homo sapiens GN=C12orf10 PE=1 SV=2 - [MYG1_HUMAN]	0.3
P49411	Elongation factor Tu, mitochondrial OS=Homo sapiens GN=TUFM PE=1 SV=2 - [EFTU_HUMAN]	0.3
Q9Y2W2	WW domain-binding protein 11 OS=Homo sapiens GN=WBP11 PE=1 SV=1 - [WBP11_HUMAN]	0.3
Q9UGP4	LIM domain-containing protein 1 OS=Homo sapiens GN=LIMD1 PE=1 SV=1 - [LIMD1_HUMAN]	0.3
Q9Y4G6	Talin-2 OS=Homo sapiens GN=TLN2 PE=1 SV=4 - [TLN2_HUMAN]	0.3
Q13144	Translation initiation factor eIF-2B subunit epsilon OS=Homo sapiens GN=EIF2B5 PE=1 SV=3 - [EI2BE_HUMAN]	0.3
Q9Y6D9	Mitotic spindle assembly checkpoint protein MAD1 OS=Homo sapiens GN=MAD1L1 PE=1 SV=2 - [MD1L1_HUMAN]	0.3
Q9ULR0	Pre-mRNA-splicing factor ISY1 homolog OS=Homo sapiens GN=ISY1 PE=1 SV=3 - [ISY1_HUMAN]	0.3
P49790	Nuclear pore complex protein Nup153 OS=Homo sapiens GN=NUP153 PE=1 SV=2 - [NU153_HUMAN]	0.3

Q9P0J1	[Pyruvate dehydrogenase [acetyl-transferring]]-phosphatase 1, mitochondrial OS=Homo sapiens GN=PDP1 PE=1 SV=3 - [PDP1_HUMAN]	0.3
P52701	DNA mismatch repair protein Msh6 OS=Homo sapiens GN=MSH6 PE=1 SV=2 - [MSH6_HUMAN]	0.3
O75525	KH domain-containing, RNA-binding, signal transduction-associated protein 3 OS=Homo sapiens GN=KHDRBS3 PE=1 SV=1 - [KHDR3_HUMAN]	0.3
Q9NW64	Pre-mRNA-splicing factor RBM22 OS=Homo sapiens GN=RBM22 PE=1 SV=1 - [RBM22_HUMAN]	0.3
Q8WVV9	Heterogeneous nuclear ribonucleoprotein L-like OS=Homo sapiens GN=HNRNPLL PE=1 SV=1 - [HNRLL_HUMAN]	0.3
Q96AE4	Far upstream element-binding protein 1 OS=Homo sapiens GN=FUBP1 PE=1 SV=3 - [FUBP1_HUMAN]	0.3
P68371	Tubulin beta-4B chain OS=Homo sapiens GN=TUBB4B PE=1 SV=1 - [TBB4B_HUMAN]	0.3
Q8N1G2	Cap-specific mRNA (nucleoside-2'-O-)-methyltransferase 1 OS=Homo sapiens GN=CMTR1 PE=1 SV=1 - [CMTR1_HUMAN]	0.3
Q9Y6W5	Wiskott-Aldrich syndrome protein family member 2 OS=Homo sapiens GN=WASF2 PE=1 SV=3 - [WASF2_HUMAN]	0.3
Q13435	Splicing factor 3B subunit 2 OS=Homo sapiens GN=SF3B2 PE=1 SV=2 - [SF3B2_HUMAN]	0.2
Q6ZSZ5	Rho guanine nucleotide exchange factor 18 OS=Homo sapiens GN=ARHGEF18 PE=1 SV=3 - [ARHGI_HUMAN]	0.2
Q13084	39S ribosomal protein L28, mitochondrial OS=Homo sapiens GN=MRPL28 PE=1 SV=4 - [RM28_HUMAN]	0.2
P49454	Centromere protein F OS=Homo sapiens GN=CENPF PE=1 SV=2 - [CENPF_HUMAN]	0.2
P31689	DnaJ homolog subfamily A member 1 OS=Homo sapiens GN=DNAJA1 PE=1 SV=2 - [DNJA1_HUMAN]	0.2
Q4L180	Filamin A-interacting protein 1-like OS=Homo sapiens GN=FILIP1L PE=1 SV=2 - [FIL1L_HUMAN]	0.2
P49756	RNA-binding protein 25 OS=Homo sapiens GN=RBM25 PE=1 SV=3 - [RBM25_HUMAN]	0.2
P48634	Protein PRRC2A OS=Homo sapiens GN=PRRC2A PE=1 SV=3 - [PRC2A_HUMAN]	0.2
Q9Y679	Ancient ubiquitous protein 1 OS=Homo sapiens GN=AUP1 PE=1 SV=1 - [AUP1_HUMAN]	0.2
Q92997	Segment polarity protein dishevelled homolog DVL-3 OS=Homo sapiens GN=DVL3 PE=1 SV=2 - [DVL3_HUMAN]	0.2
P56381	ATP synthase subunit epsilon, mitochondrial OS=Homo sapiens GN=ATP5E PE=1 SV=2 - [ATP5E_HUMAN]	0.2
Q15427	Splicing factor 3B subunit 4 OS=Homo sapiens GN=SF3B4 PE=1 SV=1 - [SF3B4_HUMAN]	0.2
P63313	Thymosin beta-10 OS=Homo sapiens GN=TMSB10 PE=1 SV=2 - [TYB10_HUMAN]	0.2
P23219	Prostaglandin G/H synthase 1 OS=Homo sapiens GN=PTGS1 PE=1 SV=2 - [PGH1_HUMAN]	0.2
Q7L775	EPM2A-interacting protein 1 OS=Homo sapiens GN=EPM2AIP1 PE=1 SV=1 - [EPMIP_HUMAN]	0.2
Q9P0M6	Core histone macro-H2A.2 OS=Homo sapiens GN=H2AFY2 PE=1 SV=3 - [H2AW_HUMAN]	0.2
Q15650	Activating signal cointegrator 1 OS=Homo sapiens GN=TRIP4 PE=1 SV=4 - [TRIP4_HUMAN]	0.2
P19367	Hexokinase-1 OS=Homo sapiens GN=HK1 PE=1 SV=3 - [HXK1_HUMAN]	0.2
Q9BRT2	Ubiquinol-cytochrome-c reductase complex assembly factor 2 OS=Homo sapiens GN=UQCC2 PE=1 SV=1 - [UQCC2_HUMAN]	0.2
Q9NZM3	Intersectin-2 OS=Homo sapiens GN=ITSN2 PE=1 SV=3 - [ITSN2_HUMAN]	0.2
Q96PE3	Type I inositol 3,4-bisphosphate 4-phosphatase OS=Homo sapiens GN=INPP4A PE=1 SV=1 - [INP4A_HUMAN]	0.2
Q92599	Septin-8 OS=Homo sapiens GN=SEPT8 PE=1 SV=4 - [SEPT8_HUMAN]	0.2
Q9BZL4	Protein phosphatase 1 regulatory subunit 12C OS=Homo sapiens GN=PPP1R12C PE=1 SV=1 - [PP12C_HUMAN]	0.2
Q9UQE7	Structural maintenance of chromosomes protein 3 OS=Homo sapiens GN=SMC3 PE=1 SV=2 - [SMC3_HUMAN]	0.2
Q96PK6	RNA-binding protein 14 OS=Homo sapiens GN=RBM14 PE=1 SV=2 - [RBM14_HUMAN]	0.2
O60518	Ran-binding protein 6 OS=Homo sapiens GN=RANBP6 PE=1 SV=2 - [RNBP6_HUMAN]	0.2
Q08170	Serine/arginine-rich splicing factor 4 OS=Homo sapiens GN=SRSF4 PE=1 SV=2 - [SRSF4_HUMAN]	0.2
O43318	Mitogen-activated protein kinase kinase kinase 7 OS=Homo sapiens GN=MAP3K7 PE=1 SV=1 - [M3K7_HUMAN]	0.2
P09110	3-ketoacyl-CoA thiolase, peroxisomal OS=Homo sapiens GN=ACAA1 PE=1 SV=2 - [THIK_HUMAN]	0.2
Q9Y2Q3	Glutathione S-transferase kappa 1 OS=Homo sapiens GN=GSTK1 PE=1 SV=3 - [GSTK1_HUMAN]	0.2
Q8TAF3	WD repeat-containing protein 48 OS=Homo sapiens GN=WDR48 PE=1 SV=1 - [WDR48_HUMAN]	0.2
P46439	Glutathione S-transferase Mu 5 OS=Homo sapiens GN=GSTM5 PE=1 SV=3 - [GSTM5_HUMAN]	0.2
P30711	Glutathione S-transferase theta-1 OS=Homo sapiens GN=GSTT1 PE=1 SV=4 - [GSTT1_HUMAN]	0.2
P14866	Heterogeneous nuclear ribonucleoprotein L OS=Homo sapiens GN=HNRNPL PE=1 SV=2 - [HNRPL_HUMAN]	0.2
Q3KQV9	UDP-N-acetylhexosamine pyrophosphorylase-like protein 1 OS=Homo sapiens GN=UAP1L1 PE=1 SV=2 - [UAP1L_HUMAN]	0.2
P27707	Deoxycytidine kinase OS=Homo sapiens GN=DCK PE=1 SV=1 - [DCK_HUMAN]	0.2
Q9BWJ5	Splicing factor 3B subunit 5 OS=Homo sapiens GN=SF3B5 PE=1 SV=1 - [SF3B5_HUMAN]	0.2
P37108	Signal recognition particle 14 kDa protein OS=Homo sapiens GN=SRP14 PE=1 SV=2 - [SRP14_HUMAN]	0.2
Q13137	Calcium-binding and coiled-coil domain-containing protein 2 OS=Homo sapiens GN=CALCOCO2 PE=1 SV=1 - [CACO2_HUMAN]	0.2
Q99459	Cell division cycle 5-like protein OS=Homo sapiens GN=CDC5L PE=1 SV=2 - [CDC5L_HUMAN]	0.2
P55265	Double-stranded RNA-specific adenosine deaminase OS=Homo sapiens GN=ADAR PE=1 SV=4 - [DSRAD_HUMAN]	0.2
P43243	Matrin-3 OS=Homo sapiens GN=MATR3 PE=1 SV=2 - [MATR3_HUMAN]	0.2
Q9NQG5	Regulation of nuclear pre-mRNA domain-containing protein 1B OS=Homo sapiens GN=RPRD1B PE=1 SV=1 - [RPR1B_HUMAN]	0.2

Q27J81	Inverted formin-2 OS=Homo sapiens GN=INF2 PE=1 SV=2 - [INF2_HUMAN]	0.2
P36957	Dihydrolipoyllysine-residue succinyltransferase component of 2-oxoglutarate dehydrogenase complex, mitochondrial OS=Homo sapiens GN=DLST PE=1 SV=4 - [ODO2_HUMAN]	0.2
Q8WXE0	Caskin-2 OS=Homo sapiens GN=CASKIN2 PE=1 SV=2 - [CSKI2_HUMAN]	0.2
Q8TC41	Probable E3 ubiquitin-protein ligase RNF217 OS=Homo sapiens GN=RNF217 PE=2 SV=4 - [RN217_HUMAN]	0.2
Q15717	ELAV-like protein 1 OS=Homo sapiens GN=ELAVL1 PE=1 SV=2 - [ELAV1_HUMAN]	0.2
A6NHR9	Structural maintenance of chromosomes flexible hinge domain-containing protein 1 OS=Homo sapiens GN=SMCHD1 PE=1 SV=2 - [SMHD1_HUMAN]	0.2
Q6NYC8	Phostensin OS=Homo sapiens GN=PPP1R18 PE=1 SV=1 - [PPR18_HUMAN]	0.2
P12270	Nucleoprotein TPR OS=Homo sapiens GN=TPR PE=1 SV=3 - [TPR_HUMAN]	0.2
Q9Y314	Nitric oxide synthase-interacting protein OS=Homo sapiens GN=NOSIP PE=1 SV=1 - [NOSIP_HUMAN]	0.2
Q8WYP5	Protein ELYS OS=Homo sapiens GN=AHCTF1 PE=1 SV=3 - [ELYS_HUMAN]	0.2
Q13148	TAR DNA-binding protein 43 OS=Homo sapiens GN=TARDBP PE=1 SV=1 - [TADBP_HUMAN]	0.2
Q8IXM2	Chromatin complexes subunit BAP18 OS=Homo sapiens GN=BAP18 PE=1 SV=1 - [BAP18_HUMAN]	0.2
P06753	Tropomyosin alpha-3 chain OS=Homo sapiens GN=TPM3 PE=1 SV=2 - [TPM3_HUMAN]	0.2
Q9BWM7	Sideroflexin-3 OS=Homo sapiens GN=SFXN3 PE=1 SV=2 - [SFXN3_HUMAN]	0.2
P53597	Succinyl-CoA ligase [ADP/GDP-forming] subunit alpha, mitochondrial OS=Homo sapiens GN=SUCLG1 PE=1 SV=4 - [SUCA_HUMAN]	0.2
Q7Z3B4	Nucleoporin p54 OS=Homo sapiens GN=NUP54 PE=1 SV=2 - [NUP54_HUMAN]	0.2
Q15149	Plectin OS=Homo sapiens GN=PLEC PE=1 SV=3 - [PLEC_HUMAN]	0.2
Q99733	Nucleosome assembly protein 1-like 4 OS=Homo sapiens GN=NAP1L4 PE=1 SV=1 - [NP1L4_HUMAN]	0.2
Q9BX68	Histidine triad nucleotide-binding protein 2, mitochondrial OS=Homo sapiens GN=HINT2 PE=1 SV=1 - [HINT2_HUMAN]	0.2
Q6PJT7	Zinc finger CCCH domain-containing protein 14 OS=Homo sapiens GN=ZC3H14 PE=1 SV=1 - [ZC3HE_HUMAN]	0.2
P51532	Transcription activator BRG1 OS=Homo sapiens GN=SMARCA4 PE=1 SV=2 - [SMCA4_HUMAN]	0.2
Q9Y4E1	WASH complex subunit FAM21C OS=Homo sapiens GN=FAM21C PE=1 SV=3 - [FA21C_HUMAN]	0.2
Q14498	RNA-binding protein 39 OS=Homo sapiens GN=RBM39 PE=1 SV=2 - [RBM39_HUMAN]	0.2
P49792	E3 SUMO-protein ligase RanBP2 OS=Homo sapiens GN=RANBP2 PE=1 SV=2 - [RBP2_HUMAN]	0.2
Q13595	Transformer-2 protein homolog alpha OS=Homo sapiens GN=TRA2A PE=1 SV=1 - [TRA2A_HUMAN]	0.2
Q9BYN8	28S ribosomal protein S26, mitochondrial OS=Homo sapiens GN=MRPS26 PE=1 SV=1 - [RT26_HUMAN]	0.2
P51153	Ras-related protein Rab-13 OS=Homo sapiens GN=RAB13 PE=1 SV=1 - [RAB13_HUMAN]	0.2
Q9NTZ6	RNA-binding protein 12 OS=Homo sapiens GN=RBM12 PE=1 SV=1 - [RBM12_HUMAN]	0.2
Q14444	Caprin-1 OS=Homo sapiens GN=CAPRIN1 PE=1 SV=2 - [CAPR1_HUMAN]	0.2
Q7Z3E5	LisH domain-containing protein ARMC9 OS=Homo sapiens GN=ARMC9 PE=1 SV=2 - [ARMC9_HUMAN]	0.2
Q96FV9	THO complex subunit 1 OS=Homo sapiens GN=THOC1 PE=1 SV=1 - [THOC1_HUMAN]	0.2
Q6L8Q7	2',5'-phosphodiesterase 12 OS=Homo sapiens GN=PDE12 PE=1 SV=2 - [PDE12_HUMAN]	0.2
Q5JTZ9	Alanine--tRNA ligase, mitochondrial OS=Homo sapiens GN=AARS2 PE=1 SV=1 - [SYAM_HUMAN]	0.2
Q96F85	CB1 cannabinoid receptor-interacting protein 1 OS=Homo sapiens GN=CNRIP1 PE=1 SV=1 - [CNRP1_HUMAN]	0.2
Q9Y2X7	ARF GTPase-activating protein GIT1 OS=Homo sapiens GN=GIT1 PE=1 SV=2 - [GIT1_HUMAN]	0.2
Q9H3Q1	Cdc42 effector protein 4 OS=Homo sapiens GN=CDC42EP4 PE=1 SV=1 - [BORG4_HUMAN]	0.2
Q92925	SWI/SNF-related matrix-associated actin-dependent regulator of chromatin subfamily D member 2 OS=Homo sapiens GN=SMARCD2 PE=1 SV=3 - [SMRD2_HUMAN]	0.2
P62875	DNA-directed RNA polymerases I, II, and III subunit RPABC5 OS=Homo sapiens GN=POLR2L PE=1 SV=1 - [RPAB5_HUMAN]	0.2
Q6PML9	Zinc transporter 9 OS=Homo sapiens GN=SLC30A9 PE=1 SV=1 - [ZNT9_HUMAN]	0.2
Q12824	SWI/SNF-related matrix-associated actin-dependent regulator of chromatin subfamily B member 1 OS=Homo sapiens GN=SMARCB1 PE=1 SV=2 - [SNF5_HUMAN]	0.2
Q04837	Single-stranded DNA-binding protein, mitochondrial OS=Homo sapiens GN=SSBP1 PE=1 SV=1 - [SSBP_HUMAN]	0.2
O75970	Multiple PDZ domain protein OS=Homo sapiens GN=MPDZ PE=1 SV=2 - [MPDZ_HUMAN]	0.2
P30084	Enoyl-CoA hydratase, mitochondrial OS=Homo sapiens GN=ECHS1 PE=1 SV=4 - [ECHM_HUMAN]	0.2
Q9H0L4	Cleavage stimulation factor subunit 2 tau variant OS=Homo sapiens GN=CSTF2T PE=1 SV=1 - [CSTFT_HUMAN]	0.2
Q8WXF1	Paraspeckle component 1 OS=Homo sapiens GN=PSPC1 PE=1 SV=1 - [PSPC1_HUMAN]	0.2
P18754	Regulator of chromosome condensation OS=Homo sapiens GN=RCC1 PE=1 SV=1 - [RCC1_HUMAN]	0.2
P46109	Crk-like protein OS=Homo sapiens GN=CRKL PE=1 SV=1 - [CRKL_HUMAN]	0.2
Q8N684	Cleavage and polyadenylation specificity factor subunit 7 OS=Homo sapiens GN=CPSF7 PE=1 SV=1 - [CPSF7_HUMAN]	0.2
Q9Y4F5	Centrosomal protein of 170 kDa protein B OS=Homo sapiens GN=CEP170B PE=1 SV=4 - [C170B_HUMAN]	0.2
P00367	Glutamate dehydrogenase 1, mitochondrial OS=Homo sapiens GN=GLUD1 PE=1 SV=2 - [DHE3_HUMAN]	0.2
Q9NZL9	Methionine adenosyltransferase 2 subunit beta OS=Homo sapiens GN=MAT2B PE=1 SV=1 - [MAT2B_HUMAN]	0.2

P48047	ATP synthase subunit O, mitochondrial OS=Homo sapiens GN=ATP5O PE=1 SV=1 - [ATPO_HUMAN]	0.2
Q12846	Syntaxin-4 OS=Homo sapiens GN=STX4 PE=1 SV=2 - [STX4_HUMAN]	0.2
O43396	Thioredoxin-like protein 1 OS=Homo sapiens GN=TXNL1 PE=1 SV=3 - [TXNL1_HUMAN]	0.2
P48735	Isocitrate dehydrogenase [NADP], mitochondrial OS=Homo sapiens GN=IDH2 PE=1 SV=2 - [IDHP_HUMAN]	0.2
Q07157	Tight junction protein ZO-1 OS=Homo sapiens GN=TJP1 PE=1 SV=3 - [ZO1_HUMAN]	0.2
Q9Y496	Kinesin-like protein KIF3A OS=Homo sapiens GN=KIF3A PE=1 SV=4 - [KIF3A_HUMAN]	0.2
Q96L93	Kinesin-like protein KIF16B OS=Homo sapiens GN=KIF16B PE=1 SV=2 - [KI16B_HUMAN]	0.2
P62993	Growth factor receptor-bound protein 2 OS=Homo sapiens GN=GRB2 PE=1 SV=1 - [GRB2_HUMAN]	0.2
O14828	Secretory carrier-associated membrane protein 3 OS=Homo sapiens GN=SCAMP3 PE=1 SV=3 - [SCAM3_HUMAN]	0.2
Q9NVT9	Armadillo repeat-containing protein 1 OS=Homo sapiens GN=ARMC1 PE=1 SV=1 - [ARMC1_HUMAN]	0.2
P80723	Brain acid soluble protein 1 OS=Homo sapiens GN=BASP1 PE=1 SV=2 - [BASP1_HUMAN]	0.2
P04150	Glucocorticoid receptor OS=Homo sapiens GN=NR3C1 PE=1 SV=1 - [GCR_HUMAN]	0.2
P20700	Lamin-B1 OS=Homo sapiens GN=LMNB1 PE=1 SV=2 - [LMNB1_HUMAN]	0.2
Q96PE2	Rho guanine nucleotide exchange factor 17 OS=Homo sapiens GN=ARHGEF17 PE=1 SV=1 - [ARHGH_HUMAN]	0.2
P25325	3-mercaptopyruvate sulfurtransferase OS=Homo sapiens GN=MPST PE=1 SV=3 - [THTM_HUMAN]	0.2
Q6KC79	Nipped-B-like protein OS=Homo sapiens GN=NIPBL PE=1 SV=2 - [NIPBL_HUMAN]	0.2
Q7Z591	AT-hook-containing transcription factor OS=Homo sapiens GN=AKNA PE=1 SV=2 - [AKNA_HUMAN]	0.2
Q9Y2W1	Thyroid hormone receptor-associated protein 3 OS=Homo sapiens GN=THRAP3 PE=1 SV=2 - [TR150_HUMAN]	0.2
Q9Y394	Dehydrogenase/reductase SDR family member 7 OS=Homo sapiens GN=DHRS7 PE=1 SV=1 - [DHRS7_HUMAN]	0.2
Q9H9S4	Calcium-binding protein 39-like OS=Homo sapiens GN=CAB39L PE=1 SV=3 - [CB39L_HUMAN]	0.2
O95372	Acyl-protein thioesterase 2 OS=Homo sapiens GN=LYPLA2 PE=1 SV=1 - [LYPA2_HUMAN]	0.2
Q96DI7	U5 small nuclear ribonucleoprotein 40 kDa protein OS=Homo sapiens GN=SNRNP40 PE=1 SV=1 - [SNR40_HUMAN]	0.2
P51665	26S proteasome non-ATPase regulatory subunit 7 OS=Homo sapiens GN=PSMD7 PE=1 SV=2 - [PSMD7_HUMAN]	0.2
Q9Y6N5	Sulfide:quinone oxidoreductase, mitochondrial OS=Homo sapiens GN=SQRDL PE=1 SV=1 - [SQRD_HUMAN]	0.2
Q05048	Cleavage stimulation factor subunit 1 OS=Homo sapiens GN=CSTF1 PE=1 SV=1 - [CSTF1_HUMAN]	0.2
P27348	14-3-3 protein theta OS=Homo sapiens GN=YWHAQ PE=1 SV=1 - [1433T_HUMAN]	0.2
P82979	SAP domain-containing ribonucleoprotein OS=Homo sapiens GN=SARNP PE=1 SV=3 - [SARNP_HUMAN]	0.2
P52789	Hexokinase-2 OS=Homo sapiens GN=HK2 PE=1 SV=2 - [HXK2_HUMAN]	0.2
P26440	Isovaleryl-CoA dehydrogenase, mitochondrial OS=Homo sapiens GN=IVD PE=1 SV=1 - [IVD_HUMAN]	0.2
Q9NYF8	Bcl-2-associated transcription factor 1 OS=Homo sapiens GN=BCLAF1 PE=1 SV=2 - [BCLF1_HUMAN]	0.2
Q9H7N4	Splicing factor, arginine/serine-rich 19 OS=Homo sapiens GN=SCAF1 PE=1 SV=3 - [SFR19_HUMAN]	0.2
Q9Y2L5	Trafficking protein particle complex subunit 8 OS=Homo sapiens GN=TRAPPC8 PE=1 SV=2 - [TPPC8_HUMAN]	0.2
Q99836	Myeloid differentiation primary response protein MyD88 OS=Homo sapiens GN=MYD88 PE=1 SV=1 - [MYD88_HUMAN]	0.2
Q9BU02	Thiamine-triphosphatase OS=Homo sapiens GN=THTPA PE=1 SV=3 - [THTPA_HUMAN]	0.2
Q8TBF2	Prostamide/prostaglandin F synthase OS=Homo sapiens GN=FAM213B PE=2 SV=1 - [PGFS_HUMAN]	0.2
Q15057	Arf-GAP with coiled-coil, ANK repeat and PH domain-containing protein 2 OS=Homo sapiens GN=ACAP2 PE=1 SV=3 - [ACAP2_HUMAN]	0.2
Q96C86	m7GpppX diphosphatase OS=Homo sapiens GN=DCPS PE=1 SV=2 - [DCPS_HUMAN]	0.2
P49748	Very long-chain specific acyl-CoA dehydrogenase, mitochondrial OS=Homo sapiens GN=ACADVL PE=1 SV=1 - [ACADV_HUMAN]	0.2
A0JLT2	Mediator of RNA polymerase II transcription subunit 19 OS=Homo sapiens GN=MED19 PE=1 SV=2 - [MED19_HUMAN]	0.2
P61978	Heterogeneous nuclear ribonucleoprotein K OS=Homo sapiens GN=HNRNPK PE=1 SV=1 - [HNRPK_HUMAN]	0.2
P52735	Guanine nucleotide exchange factor VAV2 OS=Homo sapiens GN=VAV2 PE=1 SV=2 - [VAV2_HUMAN]	0.2
Q8ND30	Liprin-beta-2 OS=Homo sapiens GN=PPFIBP2 PE=1 SV=3 - [LIPB2_HUMAN]	0.2
P33897	ATP-binding cassette sub-family D member 1 OS=Homo sapiens GN=ABCD1 PE=1 SV=2 - [ABCD1_HUMAN]	0.2
O75937	DnaJ homolog subfamily C member 8 OS=Homo sapiens GN=DNAJC8 PE=1 SV=2 - [DNJC8_HUMAN]	0.2
O75323	Protein NipSnap homolog 2 OS=Homo sapiens GN=GBAS PE=1 SV=1 - [NIPS2_HUMAN]	0.2
P09543	2',3'-cyclic-nucleotide 3'-phosphodiesterase OS=Homo sapiens GN=CNP PE=1 SV=2 - [CN37_HUMAN]	0.2
P45974	Ubiquitin carboxyl-terminal hydrolase 5 OS=Homo sapiens GN=USP5 PE=1 SV=2 - [UBP5_HUMAN]	0.2
Q10713	Mitochondrial-processing peptidase subunit alpha OS=Homo sapiens GN=PMPCA PE=1 SV=2 - [MPPA_HUMAN]	0.2
O95602	DNA-directed RNA polymerase I subunit RPA1 OS=Homo sapiens GN=POLR1A PE=1 SV=2 - [RPA1_HUMAN]	0.2
Q9UIJ7	GTP:AMP phosphotransferase AK3, mitochondrial OS=Homo sapiens GN=AK3 PE=1 SV=4 - [KAD3_HUMAN]	0.2
Q7L2J0	7SK snRNA methylphosphate capping enzyme OS=Homo sapiens GN=MEPCE PE=1 SV=1 - [MEPCE_HUMAN]	0.2
O75152	Zinc finger CCCH domain-containing protein 11A OS=Homo sapiens GN=ZC3H11A PE=1 SV=3 - [ZC11A_HUMAN]	0.2

P98175	RNA-binding protein 10 OS=Homo sapiens GN=RBM10 PE=1 SV=3 - [RBM10_HUMAN]	0.2
Q9Y2L1	Exosome complex exonuclease RRP44 OS=Homo sapiens GN=DIS3 PE=1 SV=2 - [RRP44_HUMAN]	0.2
Q8N3R9	MAGUK p55 subfamily member 5 OS=Homo sapiens GN=MPP5 PE=1 SV=3 - [MPP5_HUMAN]	0.2
P18583	Protein SON OS=Homo sapiens GN=SON PE=1 SV=4 - [SON_HUMAN]	0.2
Q7Z2Z2	Elongation factor Tu GTP-binding domain-containing protein 1 OS=Homo sapiens GN=EFTUD1 PE=1 SV=2 - [ETUD1_HUMAN]	0.2
Q07666	KH domain-containing, RNA-binding, signal transduction-associated protein 1 OS=Homo sapiens GN=KHDRBS1 PE=1 SV=1 - [KHDR1_HUMAN]	0.2
Q86X10	Ral GTPase-activating protein subunit beta OS=Homo sapiens GN=RALGAPB PE=1 SV=1 - [RLGPB_HUMAN]	0.2
Q9BV79	Trans-2-enoyl-CoA reductase, mitochondrial OS=Homo sapiens GN=MECR PE=1 SV=2 - [MECR_HUMAN]	0.2
Q6Y7W6	PERQ amino acid-rich with GYF domain-containing protein 2 OS=Homo sapiens GN=GIGYF2 PE=1 SV=1 - [PERQ2_HUMAN]	0.2
Q9P270	SLAIN motif-containing protein 2 OS=Homo sapiens GN=SLAIN2 PE=1 SV=2 - [SLAI2_HUMAN]	0.2
Q9NR28	Diablo homolog, mitochondrial OS=Homo sapiens GN=DIABLO PE=1 SV=1 - [DBLOH_HUMAN]	0.2
Q8WWY3	U4/U6 small nuclear ribonucleoprotein Prp31 OS=Homo sapiens GN=PRPF31 PE=1 SV=2 - [PRP31_HUMAN]	0.2
Q9H0U4	Ras-related protein Rab-1B OS=Homo sapiens GN=RAB1B PE=1 SV=1 - [RAB1B_HUMAN]	0.2
P22626	Heterogeneous nuclear ribonucleoproteins A2/B1 OS=Homo sapiens GN=HNRNPA2B1 PE=1 SV=2 - [ROA2_HUMAN]	0.2
Q29RF7	Sister chromatid cohesion protein PDS5 homolog A OS=Homo sapiens GN=PDS5A PE=1 SV=1 - [PDS5A_HUMAN]	0.2
P37802	Transgelin-2 OS=Homo sapiens GN=TAGLN2 PE=1 SV=3 - [TAGL2_HUMAN]	0.2
Q8NBF2	NHL repeat-containing protein 2 OS=Homo sapiens GN=NHLRC2 PE=1 SV=1 - [NHLC2_HUMAN]	0.2
P35658	Nuclear pore complex protein Nup214 OS=Homo sapiens GN=NUP214 PE=1 SV=2 - [NU214_HUMAN]	0.2
O43583	Density-regulated protein OS=Homo sapiens GN=DENR PE=1 SV=2 - [DENR_HUMAN]	0.2
Q8NHQ8	Ras association domain-containing protein 8 OS=Homo sapiens GN=RASSF8 PE=1 SV=2 - [RASF8_HUMAN]	0.2
Q7Z5L9	Interferon regulatory factor 2-binding protein 2 OS=Homo sapiens GN=IRF2BP2 PE=1 SV=2 - [I2BP2_HUMAN]	0.2
Q63HN8	E3 ubiquitin-protein ligase RNF213 OS=Homo sapiens GN=RNF213 PE=1 SV=3 - [RN213_HUMAN]	0.2
P62736	Actin, aortic smooth muscle OS=Homo sapiens GN=ACTA2 PE=1 SV=1 - [ACTA_HUMAN]	0.2
P0DI81	Trafficking protein particle complex subunit 2 OS=Homo sapiens GN=TRAPPC2 PE=1 SV=1 - [TPC2A_HUMAN]	0.2
Q08211	ATP-dependent RNA helicase A OS=Homo sapiens GN=DHX9 PE=1 SV=4 - [DHX9_HUMAN]	0.2
Q9BW91	ADP-ribose pyrophosphatase, mitochondrial OS=Homo sapiens GN=NUDT9 PE=1 SV=1 - [NUDT9_HUMAN]	0.2
P52272	Heterogeneous nuclear ribonucleoprotein M OS=Homo sapiens GN=HNRNPM PE=1 SV=3 - [HNRPM_HUMAN]	0.2
P31942	Heterogeneous nuclear ribonucleoprotein H3 OS=Homo sapiens GN=HNRNPH3 PE=1 SV=2 - [HNRH3_HUMAN]	0.2
P08133	Annexin A6 OS=Homo sapiens GN=ANXA6 PE=1 SV=3 - [ANXA6_HUMAN]	0.2
Q9NVA1	Ubiquinol-cytochrome-c reductase complex assembly factor 1 OS=Homo sapiens GN=UQCC1 PE=1 SV=3 - [UQCC1_HUMAN]	0.2
Q8WX92	Negative elongation factor B OS=Homo sapiens GN=NELFB PE=1 SV=1 - [NELFB_HUMAN]	0.2
Q07955	Serine/arginine-rich splicing factor 1 OS=Homo sapiens GN=SRSF1 PE=1 SV=2 - [SRSF1_HUMAN]	0.2
P16070	CD44 antigen OS=Homo sapiens GN=CD44 PE=1 SV=3 - [CD44_HUMAN]	0.2
Q8NC56	LEM domain-containing protein 2 OS=Homo sapiens GN=LEMD2 PE=1 SV=1 - [LEMD2_HUMAN]	0.2
Q8NC51	Plasminogen activator inhibitor 1 RNA-binding protein OS=Homo sapiens GN=SERBP1 PE=1 SV=2 - [PAIRB_HUMAN]	0.2
P13647	Keratin, type II cytoskeletal 5 OS=Homo sapiens GN=KRT5 PE=1 SV=3 - [K2C5_HUMAN]	0.2
Q9BZL6	Serine/threonine-protein kinase D2 OS=Homo sapiens GN=PRKD2 PE=1 SV=2 - [KPCD2_HUMAN]	0.2
P14618	Pyruvate kinase PKM OS=Homo sapiens GN=PKM PE=1 SV=4 - [KPYM_HUMAN]	0.2
O60869	Endothelial differentiation-related factor 1 OS=Homo sapiens GN=EDF1 PE=1 SV=1 - [EDF1_HUMAN]	0.2
P06576	ATP synthase subunit beta, mitochondrial OS=Homo sapiens GN=ATP5B PE=1 SV=3 - [ATPB_HUMAN]	0.2
P13611	Versican core protein OS=Homo sapiens GN=VCAN PE=1 SV=3 - [CSPG2_HUMAN]	0.2
Q99567	Nuclear pore complex protein Nup88 OS=Homo sapiens GN=NUP88 PE=1 SV=2 - [NUP88_HUMAN]	0.2
Q4V328	GRIP1-associated protein 1 OS=Homo sapiens GN=GRIPAP1 PE=1 SV=1 - [GRAP1_HUMAN]	0.2
Q13405	39S ribosomal protein L49, mitochondrial OS=Homo sapiens GN=MRPL49 PE=1 SV=1 - [RM49_HUMAN]	0.2
Q13263	Transcription intermediary factor 1-beta OS=Homo sapiens GN=TRIM28 PE=1 SV=5 - [TIF1B_HUMAN]	0.2
Q9HCE1	Putative helicase MOV-10 OS=Homo sapiens GN=MOV10 PE=1 SV=2 - [MOV10_HUMAN]	0.2
Q13509	Tubulin beta-3 chain OS=Homo sapiens GN=TUBB3 PE=1 SV=2 - [TBB3_HUMAN]	0.2
P34059	N-acetylgalactosamine-6-sulfatase OS=Homo sapiens GN=GALNS PE=1 SV=1 - [GALNS_HUMAN]	0.2
Q96C19	EF-hand domain-containing protein D2 OS=Homo sapiens GN=EFHD2 PE=1 SV=1 - [EFHD2_HUMAN]	0.2
P15121	Aldose reductase OS=Homo sapiens GN=AKR1B1 PE=1 SV=3 - [ALDR_HUMAN]	0.2
O43290	U4/U6.U5 tri-snRNP-associated protein 1 OS=Homo sapiens GN=SART1 PE=1 SV=1 - [SNUT1_HUMAN]	0.2
Q08945	FACT complex subunit SSRP1 OS=Homo sapiens GN=SSRP1 PE=1 SV=1 - [SSRP1_HUMAN]	0.2
P63096	Guanine nucleotide-binding protein G(i) subunit alpha-1 OS=Homo sapiens GN=GNAI1 PE=1 SV=2 - [GNAI1_HUMAN]	0.2

O43390	Heterogeneous nuclear ribonucleoprotein R OS=Homo sapiens GN=HNRNPR PE=1 SV=1 - [HNRPR_HUMAN]	0.2
O75113	NEDD4-binding protein 1 OS=Homo sapiens GN=N4BP1 PE=1 SV=4 - [N4BP1_HUMAN]	0.2
Q05209	Tyrosine-protein phosphatase non-receptor type 12 OS=Homo sapiens GN=PTPN12 PE=1 SV=3 - [PTN12_HUMAN]	0.2
Q96I99	Succinyl-CoA ligase [GDP-forming] subunit beta, mitochondrial OS=Homo sapiens GN=SUCLG2 PE=1 SV=2 - [SUCB2_HUMAN]	0.2
O00330	Pyruvate dehydrogenase protein X component, mitochondrial OS=Homo sapiens GN=PDHX PE=1 SV=3 - [ODPX_HUMAN]	0.2
P51858	Hepatoma-derived growth factor OS=Homo sapiens GN=HDGF PE=1 SV=1 - [HDGF_HUMAN]	0.2
O94906	Pre-mRNA-processing factor 6 OS=Homo sapiens GN=PRPF6 PE=1 SV=1 - [PRP6_HUMAN]	0.2
Q9Y697	Cysteine desulfurase, mitochondrial OS=Homo sapiens GN=NFS1 PE=1 SV=3 - [NFS1_HUMAN]	0.2
Q7L014	Probable ATP-dependent RNA helicase DDX46 OS=Homo sapiens GN=DDX46 PE=1 SV=2 - [DDX46_HUMAN]	0.2
P99999	Cytochrome c OS=Homo sapiens GN=CYCS PE=1 SV=2 - [CYC_HUMAN]	0.2
P13798	Acylamino-acid-releasing enzyme OS=Homo sapiens GN=APEH PE=1 SV=4 - [ACPH_HUMAN]	0.2
P28370	Probable global transcription activator SNF2L1 OS=Homo sapiens GN=SMARCA1 PE=1 SV=2 - [SMCA1_HUMAN]	0.2
Q9H008	Phospholysine phosphohistidine inorganic pyrophosphate phosphatase OS=Homo sapiens GN=LHPP PE=1 SV=2 - [LHPP_HUMAN]	0.2
Q9NVH2	Integrator complex subunit 7 OS=Homo sapiens GN=INTS7 PE=1 SV=1 - [INT7_HUMAN]	0.2
Q15642	Cdc42-interacting protein 4 OS=Homo sapiens GN=TRIP10 PE=1 SV=3 - [CIP4_HUMAN]	0.2
Q96HC4	PDZ and LIM domain protein 5 OS=Homo sapiens GN=PDLIM5 PE=1 SV=5 - [PDLI5_HUMAN]	0.2
Q9ULK4	Mediator of RNA polymerase II transcription subunit 23 OS=Homo sapiens GN=MED23 PE=1 SV=2 - [MED23_HUMAN]	0.2
Q00839	Heterogeneous nuclear ribonucleoprotein U OS=Homo sapiens GN=HNRNPU PE=1 SV=6 - [HNRPU_HUMAN]	0.2
Q01130	Serine/arginine-rich splicing factor 2 OS=Homo sapiens GN=SRSF2 PE=1 SV=4 - [SRSF2_HUMAN]	0.2
Q9ULH1	Arf-GAP with SH3 domain, ANK repeat and PH domain-containing protein 1 OS=Homo sapiens GN=ASAP1 PE=1 SV=4 - [ASAP1_HUMAN]	0.2
P61956	Small ubiquitin-related modifier 2 OS=Homo sapiens GN=SUMO2 PE=1 SV=3 - [SUMO2_HUMAN]	0.2
Q96RU3	Formin-binding protein 1 OS=Homo sapiens GN=FNBP1 PE=1 SV=2 - [FNBP1_HUMAN]	0.2
Q96EL3	39S ribosomal protein L53, mitochondrial OS=Homo sapiens GN=MRPL53 PE=1 SV=1 - [RM53_HUMAN]	0.2
P62263	40S ribosomal protein S14 OS=Homo sapiens GN=RPS14 PE=1 SV=3 - [RS14_HUMAN]	0.2
Q13428	Treacle protein OS=Homo sapiens GN=TCOF1 PE=1 SV=3 - [TCOF_HUMAN]	0.2
Q8NDH3	Probable aminopeptidase NPEPL1 OS=Homo sapiens GN=NPEPL1 PE=1 SV=3 - [PEPL1_HUMAN]	0.2
Q6P1J9	Parafibromin OS=Homo sapiens GN=CDC73 PE=1 SV=1 - [CDC73_HUMAN]	0.2
Q14160	Protein scribble homolog OS=Homo sapiens GN=SCRIB PE=1 SV=4 - [SCRIB_HUMAN]	0.2
Q16630	Cleavage and polyadenylation specificity factor subunit 6 OS=Homo sapiens GN=CPSF6 PE=1 SV=2 - [CPSF6_HUMAN]	0.2
O43768	Alpha-endosulfine OS=Homo sapiens GN=ENSA PE=1 SV=1 - [ENSA_HUMAN]	0.2
P00167	Cytochrome b5 OS=Homo sapiens GN=CYB5A PE=1 SV=2 - [CYB5_HUMAN]	0.2
Q7Z4V5	Hepatoma-derived growth factor-related protein 2 OS=Homo sapiens GN=HDGFRP2 PE=1 SV=1 - [HDGR2_HUMAN]	0.2
Q12888	Tumor suppressor p53-binding protein 1 OS=Homo sapiens GN=TP53BP1 PE=1 SV=2 - [TP53B_HUMAN]	0.2
Q12899	Tripartite motif-containing protein 26 OS=Homo sapiens GN=TRIM26 PE=2 SV=1 - [TRI26_HUMAN]	0.2
Q9NP66	High mobility group protein 20A OS=Homo sapiens GN=HMG20A PE=1 SV=1 - [HM20A_HUMAN]	0.2
Q02880	DNA topoisomerase 2-beta OS=Homo sapiens GN=TOP2B PE=1 SV=3 - [TOP2B_HUMAN]	0.2
Q9H2U2	Inorganic pyrophosphatase 2, mitochondrial OS=Homo sapiens GN=PPA2 PE=1 SV=2 - [IPYR2_HUMAN]	0.2
P78524	Suppression of tumorigenicity 5 protein OS=Homo sapiens GN=ST5 PE=1 SV=3 - [ST5_HUMAN]	0.2
Q92597	Protein NDRG1 OS=Homo sapiens GN=NDRG1 PE=1 SV=1 - [NDRG1_HUMAN]	0.2
O14776	Transcription elongation regulator 1 OS=Homo sapiens GN=TCERG1 PE=1 SV=2 - [TCRG1_HUMAN]	0.2
Q8WY36	HMG box transcription factor BBX OS=Homo sapiens GN=BBX PE=1 SV=1 - [BBX_HUMAN]	0.2
Q13523	Serine/threonine-protein kinase PRP4 homolog OS=Homo sapiens GN=PRPF4B PE=1 SV=3 - [PRP4B_HUMAN]	0.2
P78406	mRNA export factor OS=Homo sapiens GN=RAE1 PE=1 SV=1 - [RAE1L_HUMAN]	0.2
O43707	Alpha-actinin-4 OS=Homo sapiens GN=ACTN4 PE=1 SV=2 - [ACTN4_HUMAN]	0.2
P29218	Inositol monophosphatase 1 OS=Homo sapiens GN=IMPA1 PE=1 SV=1 - [IMPA1_HUMAN]	0.2
O43809	Cleavage and polyadenylation specificity factor subunit 5 OS=Homo sapiens GN=NUDT21 PE=1 SV=1 - [CPSF5_HUMAN]	0.2
Q93009	Ubiquitin carboxyl-terminal hydrolase 7 OS=Homo sapiens GN=USP7 PE=1 SV=2 - [UBP7_HUMAN]	0.2
Q09161	Nuclear cap-binding protein subunit 1 OS=Homo sapiens GN=NCBP1 PE=1 SV=1 - [NCBP1_HUMAN]	0.2
P12814	Alpha-actinin-1 OS=Homo sapiens GN=ACTN1 PE=1 SV=2 - [ACTN1_HUMAN]	0.2
Q9UMS4	Pre-mRNA-processing factor 19 OS=Homo sapiens GN=PRPF19 PE=1 SV=1 - [PRP19_HUMAN]	0.2
P09972	Fructose-bisphosphate aldolase C OS=Homo sapiens GN=ALDOC PE=1 SV=2 - [ALDOC_HUMAN]	0.2
Q8IX01	SURP and G-patch domain-containing protein 2 OS=Homo sapiens GN=SUGP2 PE=1 SV=2 - [SUGP2_HUMAN]	0.2

Q96P48	Arf-GAP with Rho-GAP domain, ANK repeat and PH domain-containing protein 1 OS=Homo sapiens GN=ARAP1 PE=1 SV=3 - [ARAP1_HUMAN]	0.2
Q96T37	Putative RNA-binding protein 15 OS=Homo sapiens GN=RBM15 PE=1 SV=2 - [RBM15_HUMAN]	0.2
P51452	Dual specificity protein phosphatase 3 OS=Homo sapiens GN=DUSP3 PE=1 SV=1 - [DUS3_HUMAN]	0.2
Q06830	Peroxiredoxin-1 OS=Homo sapiens GN=PRDX1 PE=1 SV=1 - [PRDX1_HUMAN]	0.2
P11177	Pyruvate dehydrogenase E1 component subunit beta, mitochondrial OS=Homo sapiens GN=PDHB PE=1 SV=3 - [ODPB_HUMAN]	0.2
P24539	ATP synthase F(0) complex subunit B1, mitochondrial OS=Homo sapiens GN=ATP5F1 PE=1 SV=2 - [AT5F1_HUMAN]	0.2
Q8TB22	Spermatogenesis-associated protein 20 OS=Homo sapiens GN=SPATA20 PE=2 SV=3 - [SPT20_HUMAN]	0.2
P09622	Dihydrolipoyl dehydrogenase, mitochondrial OS=Homo sapiens GN=DLD PE=1 SV=2 - [DLDH_HUMAN]	0.2
Q9UKK3	Poly [ADP-ribose] polymerase 4 OS=Homo sapiens GN=PARP4 PE=1 SV=3 - [PARP4_HUMAN]	0.2
Q14CX7	N-alpha-acetyltransferase 25, NatB auxiliary subunit OS=Homo sapiens GN=NAA25 PE=1 SV=1 - [NAA25_HUMAN]	0.2
Q9Y4X5	E3 ubiquitin-protein ligase ARIH1 OS=Homo sapiens GN=ARIH1 PE=1 SV=2 - [ARI1_HUMAN]	0.2
P14927	Cytochrome b-c1 complex subunit 7 OS=Homo sapiens GN=UQCRB PE=1 SV=2 - [QCR7_HUMAN]	0.2
Q9NQ50	39S ribosomal protein L40, mitochondrial OS=Homo sapiens GN=MRPL40 PE=1 SV=1 - [RM40_HUMAN]	0.2
Q96TA1	Niban-like protein 1 OS=Homo sapiens GN=FAM129B PE=1 SV=3 - [NIBL1_HUMAN]	0.2
O00193	Small acidic protein OS=Homo sapiens GN=SMAP PE=1 SV=1 - [SMAP_HUMAN]	0.2
Q8ND24	RING finger protein 214 OS=Homo sapiens GN=RNF214 PE=1 SV=2 - [RN214_HUMAN]	0.2
Q96T51	RUN and FYVE domain-containing protein 1 OS=Homo sapiens GN=RUFY1 PE=1 SV=2 - [RUFY1_HUMAN]	0.2
Q9NWB6	Arginine and glutamate-rich protein 1 OS=Homo sapiens GN=ARGLU1 PE=1 SV=1 - [ARGL1_HUMAN]	0.2
Q9H0C8	Integrin-linked kinase-associated serine/threonine phosphatase 2C OS=Homo sapiens GN=ILKAP PE=1 SV=1 - [ILKAP_HUMAN]	0.2
P51114	Fragile X mental retardation syndrome-related protein 1 OS=Homo sapiens GN=FXR1 PE=1 SV=3 - [FXR1_HUMAN]	0.2
O43143	Putative pre-mRNA-splicing factor ATP-dependent RNA helicase DHX15 OS=Homo sapiens GN=DHX15 PE=1 SV=2 - [DHX15_HUMAN]	0.2
P51572	B-cell receptor-associated protein 31 OS=Homo sapiens GN=BCAP31 PE=1 SV=3 - [BAP31_HUMAN]	0.2
Q9UPU7	TBC1 domain family member 2B OS=Homo sapiens GN=TBC1D2B PE=1 SV=2 - [TBD2B_HUMAN]	-0.2
Q13438	Protein OS-9 OS=Homo sapiens GN=OS9 PE=1 SV=1 - [OS9_HUMAN]	-0.2
O94973	AP-2 complex subunit alpha-2 OS=Homo sapiens GN=AP2A2 PE=1 SV=2 - [AP2A2_HUMAN]	-0.2
Q5R3I4	Tetratricopeptide repeat protein 38 OS=Homo sapiens GN=TTC38 PE=1 SV=1 - [TTC38_HUMAN]	-0.2
Q02809	Procollagen-lysine,2-oxoglutarate 5-dioxygenase 1 OS=Homo sapiens GN=PLOD1 PE=1 SV=2 - [PLOD1_HUMAN]	-0.2
Q86U42	Polyadenylate-binding protein 2 OS=Homo sapiens GN=PABPN1 PE=1 SV=3 - [PABP2_HUMAN]	-0.2
Q9H6U8	Alpha-1,2-mannosyltransferase ALG9 OS=Homo sapiens GN=ALG9 PE=1 SV=2 - [ALG9_HUMAN]	-0.2
Q15382	GTP-binding protein Rheb OS=Homo sapiens GN=RHEB PE=1 SV=1 - [RHEB_HUMAN]	-0.2
O43181	NADH dehydrogenase [ubiquinone] iron-sulfur protein 4, mitochondrial OS=Homo sapiens GN=NDUFS4 PE=1 SV=1 - [NDUS4_HUMAN]	-0.2
Q14690	Protein RRP5 homolog OS=Homo sapiens GN=PDCD11 PE=1 SV=3 - [RRP5_HUMAN]	-0.2
Q9NVD7	Alpha-parvin OS=Homo sapiens GN=PARVA PE=1 SV=1 - [PARVA_HUMAN]	-0.2
Q99873	Protein arginine N-methyltransferase 1 OS=Homo sapiens GN=PRMT1 PE=1 SV=2 - [ANM1_HUMAN]	-0.2
P26640	Valine--tRNA ligase OS=Homo sapiens GN=VARS PE=1 SV=4 - [SYVC_HUMAN]	-0.2
Q9H0P0	Cytosolic 5'-nucleotidase 3A OS=Homo sapiens GN=NT5C3A PE=1 SV=3 - [5NT3A_HUMAN]	-0.2
P36776	Lon protease homolog, mitochondrial OS=Homo sapiens GN=LONP1 PE=1 SV=2 - [LONM_HUMAN]	-0.2
Q8IWJ2	GRIP and coiled-coil domain-containing protein 2 OS=Homo sapiens GN=GCC2 PE=1 SV=4 - [GCC2_HUMAN]	-0.2
P49840	Glycogen synthase kinase-3 alpha OS=Homo sapiens GN=GSK3A PE=1 SV=2 - [GSK3A_HUMAN]	-0.2
Q53H12	Acylglycerol kinase, mitochondrial OS=Homo sapiens GN=AGK PE=1 SV=2 - [AGK_HUMAN]	-0.2
Q6P3X3	Tetratricopeptide repeat protein 27 OS=Homo sapiens GN=TTC27 PE=1 SV=1 - [TTC27_HUMAN]	-0.2
P58546	Myotrophin OS=Homo sapiens GN=MTPN PE=1 SV=2 - [MTPN_HUMAN]	-0.2
P82663	28S ribosomal protein S25, mitochondrial OS=Homo sapiens GN=MRPS25 PE=1 SV=1 - [RT25_HUMAN]	-0.2
P54578	Ubiquitin carboxyl-terminal hydrolase 14 OS=Homo sapiens GN=USP14 PE=1 SV=3 - [UBP14_HUMAN]	-0.2
P11021	78 kDa glucose-regulated protein OS=Homo sapiens GN=HSPA5 PE=1 SV=2 - [GRP78_HUMAN]	-0.2
Q8IXM3	39S ribosomal protein L41, mitochondrial OS=Homo sapiens GN=MRPL41 PE=1 SV=1 - [RM41_HUMAN]	-0.2
Q15545	Transcription initiation factor TFIID subunit 7 OS=Homo sapiens GN=TAF7 PE=1 SV=1 - [TAF7_HUMAN]	-0.2
P49327	Fatty acid synthase OS=Homo sapiens GN=FASN PE=1 SV=3 - [FAS_HUMAN]	-0.2
P46779	60S ribosomal protein L28 OS=Homo sapiens GN=RPL28 PE=1 SV=3 - [RL28_HUMAN]	-0.2
Q9Y3C8	Ubiquitin-fold modifier-conjugating enzyme 1 OS=Homo sapiens GN=UFC1 PE=1 SV=3 - [UFC1_HUMAN]	-0.2
P23588	Eukaryotic translation initiation factor 4B OS=Homo sapiens GN=EIF4B PE=1 SV=2 - [IF4B_HUMAN]	-0.2
Q9HD20	Manganese-transporting ATPase 13A1 OS=Homo sapiens GN=ATP13A1 PE=1 SV=2 - [AT131_HUMAN]	-0.2

O00232	26S proteasome non-ATPase regulatory subunit 12 OS=Homo sapiens GN=PSMD12 PE=1 SV=3 - [PSD12_HUMAN]	-0.2
P36507	Dual specificity mitogen-activated protein kinase kinase 2 OS=Homo sapiens GN=MAP2K2 PE=1 SV=1 - [MP2K2_HUMAN]	-0.2
Q96HY6	DDRGK domain-containing protein 1 OS=Homo sapiens GN=DDRGK1 PE=1 SV=2 - [DDRGK_HUMAN]	-0.2
Q9UBS4	DnaJ homolog subfamily B member 11 OS=Homo sapiens GN=DNAJB11 PE=1 SV=1 - [DJB11_HUMAN]	-0.2
Q9NVS9	Pyridoxine-5'-phosphate oxidase OS=Homo sapiens GN=PNPO PE=1 SV=1 - [PNPO_HUMAN]	-0.2
Q6PI26	Protein SHQ1 homolog OS=Homo sapiens GN=SHQ1 PE=1 SV=2 - [SHQ1_HUMAN]	-0.2
O75882	Attractin OS=Homo sapiens GN=ATRN PE=1 SV=2 - [ATRN_HUMAN]	-0.2
Q96EK7	Constitutive coactivator of peroxisome proliferator-activated receptor gamma OS=Homo sapiens GN=FAM120B PE=1 SV=1 - [F120B_HUMAN]	-0.2
Q8TD19	Serine/threonine-protein kinase Nek9 OS=Homo sapiens GN=NEK9 PE=1 SV=2 - [NEK9_HUMAN]	-0.2
P25398	40S ribosomal protein S12 OS=Homo sapiens GN=RPS12 PE=1 SV=3 - [RS12_HUMAN]	-0.2
Q9UKY7	Protein CDV3 homolog OS=Homo sapiens GN=CDV3 PE=1 SV=1 - [CDV3_HUMAN]	-0.2
Q9NR12	PDZ and LIM domain protein 7 OS=Homo sapiens GN=PDLIM7 PE=1 SV=1 - [PDLI7_HUMAN]	-0.2
P48729	Casein kinase I isoform alpha OS=Homo sapiens GN=CSNK1A1 PE=1 SV=2 - [KC1A_HUMAN]	-0.2
P20645	Cation-dependent mannose-6-phosphate receptor OS=Homo sapiens GN=M6PR PE=1 SV=1 - [MPRD_HUMAN]	-0.2
O60783	28S ribosomal protein S14, mitochondrial OS=Homo sapiens GN=MRPS14 PE=1 SV=1 - [RT14_HUMAN]	-0.2
Q9NX62	Inositol monophosphatase 3 OS=Homo sapiens GN=IMPAD1 PE=1 SV=1 - [IMPA3_HUMAN]	-0.2
Q13636	Ras-related protein Rab-31 OS=Homo sapiens GN=RAB31 PE=1 SV=1 - [RAB31_HUMAN]	-0.2
Q96CV9	Optineurin OS=Homo sapiens GN=OPTN PE=1 SV=2 - [OPTN_HUMAN]	-0.2
Q15436	Protein transport protein Sec23A OS=Homo sapiens GN=SEC23A PE=1 SV=2 - [SC23A_HUMAN]	-0.2
Q9Y6E2	Basic leucine zipper and W2 domain-containing protein 2 OS=Homo sapiens GN=BZW2 PE=1 SV=1 - [BZW2_HUMAN]	-0.2
P23396	40S ribosomal protein S3 OS=Homo sapiens GN=RPS3 PE=1 SV=2 - [RS3_HUMAN]	-0.2
P49257	Protein ERGIC-53 OS=Homo sapiens GN=LMAN1 PE=1 SV=2 - [LMAN1_HUMAN]	-0.2
Q9Y371	Endophilin-B1 OS=Homo sapiens GN=SH3GLB1 PE=1 SV=1 - [SHLB1_HUMAN]	-0.2
Q8TDN6	Ribosome biogenesis protein BRX1 homolog OS=Homo sapiens GN=BRIX1 PE=1 SV=2 - [BRX1_HUMAN]	-0.2
P28749	Retinoblastoma-like protein 1 OS=Homo sapiens GN=RBL1 PE=1 SV=3 - [RBL1_HUMAN]	-0.2
A2RRP1	Neuroblastoma-amplified sequence OS=Homo sapiens GN=NBAS PE=1 SV=2 - [NBAS_HUMAN]	-0.2
P49591	Serine--tRNA ligase, cytoplasmic OS=Homo sapiens GN=SARS PE=1 SV=3 - [SYSC_HUMAN]	-0.2
P35222	Catenin beta-1 OS=Homo sapiens GN=CTNNB1 PE=1 SV=1 - [CTNB1_HUMAN]	-0.2
P22830	Ferrochelatase, mitochondrial OS=Homo sapiens GN=FECH PE=1 SV=2 - [HEMH_HUMAN]	-0.2
P37235	Hippocalcin-like protein 1 OS=Homo sapiens GN=HPCAL1 PE=1 SV=3 - [HPCL1_HUMAN]	-0.2
P27361	Mitogen-activated protein kinase 3 OS=Homo sapiens GN=MAPK3 PE=1 SV=4 - [MK03_HUMAN]	-0.2
Q96ME1	F-box/LRR-repeat protein 18 OS=Homo sapiens GN=FBXL18 PE=1 SV=2 - [FXL18_HUMAN]	-0.2
Q9UH65	Switch-associated protein 70 OS=Homo sapiens GN=SWAP70 PE=1 SV=1 - [SWP70_HUMAN]	-0.2
Q8IZT6	Abnormal spindle-like microcephaly-associated protein OS=Homo sapiens GN=ASPM PE=1 SV=2 - [ASPM_HUMAN]	-0.2
P32189	Glycerol kinase OS=Homo sapiens GN=GK PE=1 SV=3 - [GLPK_HUMAN]	-0.2
O96020	G1/S-specific cyclin-E2 OS=Homo sapiens GN=CCNE2 PE=1 SV=1 - [CCNE2_HUMAN]	-0.2
Q9UJ70	N-acetyl-D-glucosamine kinase OS=Homo sapiens GN=NAGK PE=1 SV=4 - [NAGK_HUMAN]	-0.2
Q9UNL2	Translocon-associated protein subunit gamma OS=Homo sapiens GN=SSR3 PE=1 SV=1 - [SSRG_HUMAN]	-0.2
Q5TCZ1	SH3 and PX domain-containing protein 2A OS=Homo sapiens GN=SH3PXD2A PE=1 SV=1 - [SPD2A_HUMAN]	-0.2
O94979	Protein transport protein Sec31A OS=Homo sapiens GN=SEC31A PE=1 SV=3 - [SC31A_HUMAN]	-0.2
Q9NZQ8	Transient receptor potential cation channel subfamily M member 5 OS=Homo sapiens GN=TRPM5 PE=2 SV=1 - [TRPM5_HUMAN]	-0.2
Q9H583	HEAT repeat-containing protein 1 OS=Homo sapiens GN=HEATR1 PE=1 SV=3 - [HEAT1_HUMAN]	-0.2
Q96GK7	Fumarylacetoacetate hydrolase domain-containing protein 2A OS=Homo sapiens GN=FAHD2A PE=1 SV=1 - [FAH2A_HUMAN]	-0.2
Q16643	Drebrin OS=Homo sapiens GN=DBN1 PE=1 SV=4 - [DREB_HUMAN]	-0.2
Q07866	Kinesin light chain 1 OS=Homo sapiens GN=KLC1 PE=1 SV=2 - [KLC1_HUMAN]	-0.2
Q9Y2T2	AP-3 complex subunit mu-1 OS=Homo sapiens GN=AP3M1 PE=1 SV=1 - [AP3M1_HUMAN]	-0.2
Q9H2M9	Rab3 GTPase-activating protein non-catalytic subunit OS=Homo sapiens GN=RAB3GAP2 PE=1 SV=1 - [RBGPR_HUMAN]	-0.2
Q15046	Lysine--tRNA ligase OS=Homo sapiens GN=KARS PE=1 SV=3 - [SYK_HUMAN]	-0.2
P46821	Microtubule-associated protein 1B OS=Homo sapiens GN=MAP1B PE=1 SV=2 - [MAP1B_HUMAN]	-0.2
Q6YP21	Kynurenine--oxoglutarate transaminase 3 OS=Homo sapiens GN=CCBL2 PE=1 SV=1 - [KAT3_HUMAN]	-0.2
P58005	Sestrin-3 OS=Homo sapiens GN=SESN3 PE=2 SV=2 - [SESN3_HUMAN]	-0.2
Q86SQ0	Pleckstrin homology-like domain family B member 2 OS=Homo sapiens GN=PHLDB2 PE=1 SV=2 - [PHLB2_HUMAN]	-0.2
Q7Z3J2	UPF0505 protein C16orf62 OS=Homo sapiens GN=C16orf62 PE=1 SV=2 - [CP062_HUMAN]	-0.2

Q16595	Frataxin, mitochondrial OS=Homo sapiens GN=FXN PE=1 SV=2 - [FRDA_HUMAN]	-0.2
O75179	Ankyrin repeat domain-containing protein 17 OS=Homo sapiens GN=ANKRD17 PE=1 SV=3 - [ANR17_HUMAN]	-0.2
O15144	Actin-related protein 2/3 complex subunit 2 OS=Homo sapiens GN=ARPC2 PE=1 SV=1 - [ARPC2_HUMAN]	-0.2
Q9Y3D9	28S ribosomal protein S23, mitochondrial OS=Homo sapiens GN=MRPS23 PE=1 SV=2 - [RT23_HUMAN]	-0.2
O43166	Signal-induced proliferation-associated 1-like protein 1 OS=Homo sapiens GN=SIPA1L1 PE=1 SV=4 - [SI1L1_HUMAN]	-0.2
Q14974	Importin subunit beta-1 OS=Homo sapiens GN=KPNB1 PE=1 SV=2 - [IMB1_HUMAN]	-0.2
Q96DG6	Carboxymethylenebutenolidase homolog OS=Homo sapiens GN=CMBL PE=1 SV=1 - [CMBL_HUMAN]	-0.2
P27635	60S ribosomal protein L10 OS=Homo sapiens GN=RPL10 PE=1 SV=4 - [RL10_HUMAN]	-0.2
Q13557	Calcium/calmodulin-dependent protein kinase type II subunit delta OS=Homo sapiens GN=CAMK2D PE=1 SV=3 - [KCC2D_HUMAN]	-0.2
Q6GMV3	Putative peptidyl-tRNA hydrolase PTRHD1 OS=Homo sapiens GN=PTRHD1 PE=1 SV=1 - [PTRD1_HUMAN]	-0.2
Q96J02	E3 ubiquitin-protein ligase Itchy homolog OS=Homo sapiens GN=ITCH PE=1 SV=2 - [ITCH_HUMAN]	-0.2
Q9BZE4	Nucleolar GTP-binding protein 1 OS=Homo sapiens GN=GTPBP4 PE=1 SV=3 - [NOG1_HUMAN]	-0.2
Q8IYS2	Uncharacterized protein KIAA2013 OS=Homo sapiens GN=KIAA2013 PE=2 SV=1 - [K2013_HUMAN]	-0.2
P43307	Translocon-associated protein subunit alpha OS=Homo sapiens GN=SSR1 PE=1 SV=3 - [SSRA_HUMAN]	-0.2
Q15075	Early endosome antigen 1 OS=Homo sapiens GN=EEA1 PE=1 SV=2 - [EEA1_HUMAN]	-0.2
Q9Y4P3	Transducin beta-like protein 2 OS=Homo sapiens GN=TBL2 PE=1 SV=1 - [TBL2_HUMAN]	-0.2
P40855	Peroxisomal biogenesis factor 19 OS=Homo sapiens GN=PEX19 PE=1 SV=1 - [PEX19_HUMAN]	-0.2
Q9NUG6	p53 and DNA damage-regulated protein 1 OS=Homo sapiens GN=PDRG1 PE=1 SV=2 - [PDRG1_HUMAN]	-0.2
Q9GZP4	PITH domain-containing protein 1 OS=Homo sapiens GN=PITHD1 PE=1 SV=1 - [PITH1_HUMAN]	-0.2
Q12768	WASH complex subunit strumpellin OS=Homo sapiens GN=KIAA0196 PE=1 SV=1 - [STRUM_HUMAN]	-0.2
P49721	Proteasome subunit beta type-2 OS=Homo sapiens GN=PSMB2 PE=1 SV=1 - [PSB2_HUMAN]	-0.2
O00487	26S proteasome non-ATPase regulatory subunit 14 OS=Homo sapiens GN=PSMD14 PE=1 SV=1 - [PSDE_HUMAN]	-0.2
Q14697	Neutral alpha-glucosidase AB OS=Homo sapiens GN=GANAB PE=1 SV=3 - [GANAB_HUMAN]	-0.2
O75436	Vacuolar protein sorting-associated protein 26A OS=Homo sapiens GN=VPS26A PE=1 SV=2 - [VP26A_HUMAN]	-0.2
Q9Y281	Cofilin-2 OS=Homo sapiens GN=CFL2 PE=1 SV=1 - [COF2_HUMAN]	-0.2
Q96AG4	Leucine-rich repeat-containing protein 59 OS=Homo sapiens GN=LRRC59 PE=1 SV=1 - [LRC59_HUMAN]	-0.2
Q12929	Epidermal growth factor receptor kinase substrate 8 OS=Homo sapiens GN=EPS8 PE=1 SV=1 - [EPS8_HUMAN]	-0.2
Q969V3	Nicalin OS=Homo sapiens GN=NCLN PE=1 SV=2 - [NCLN_HUMAN]	-0.2
Q9Y6E0	Serine/threonine-protein kinase 24 OS=Homo sapiens GN=STK24 PE=1 SV=1 - [STK24_HUMAN]	-0.2
Q96B36	Proline-rich AKT1 substrate 1 OS=Homo sapiens GN=AKT1S1 PE=1 SV=1 - [AKTS1_HUMAN]	-0.2
P47985	Cytochrome b-c1 complex subunit Rieske, mitochondrial OS=Homo sapiens GN=UQCRFS1 PE=1 SV=2 - [UCRI_HUMAN]	-0.2
P08648	Integrin alpha-5 OS=Homo sapiens GN=ITGA5 PE=1 SV=2 - [ITA5_HUMAN]	-0.2
Q92616	Translational activator GCN1 OS=Homo sapiens GN=GCN1L1 PE=1 SV=6 - [GCN1L_HUMAN]	-0.2
H7BZ55	Putative ciliary rootlet coiled-coil protein-like 3 protein OS=Homo sapiens PE=5 SV=2 - [CROL3_HUMAN]	-0.2
Q04637	Eukaryotic translation initiation factor 4 gamma 1 OS=Homo sapiens GN=EIF4G1 PE=1 SV=4 - [IF4G1_HUMAN]	-0.2
P35080	Profilin-2 OS=Homo sapiens GN=PFN2 PE=1 SV=3 - [PROF2_HUMAN]	-0.2
O00161	Synaptosomal-associated protein 23 OS=Homo sapiens GN=SNAP23 PE=1 SV=1 - [SNP23_HUMAN]	-0.2
Q13976	cGMP-dependent protein kinase 1 OS=Homo sapiens GN=PRKG1 PE=1 SV=3 - [KGP1_HUMAN]	-0.2
P49406	39S ribosomal protein L19, mitochondrial OS=Homo sapiens GN=MRPL19 PE=1 SV=2 - [RM19_HUMAN]	-0.2
Q13126	S-methyl-5'-thioadenosine phosphorylase OS=Homo sapiens GN=MTAP PE=1 SV=2 - [MTAP_HUMAN]	-0.2
Q02543	60S ribosomal protein L18a OS=Homo sapiens GN=RPL18A PE=1 SV=2 - [RL18A_HUMAN]	-0.2
Q8TBC4	NEDD8-activating enzyme E1 catalytic subunit OS=Homo sapiens GN=UBA3 PE=1 SV=2 - [UBA3_HUMAN]	-0.2
P42330	Aldo-keto reductase family 1 member C3 OS=Homo sapiens GN=AKR1C3 PE=1 SV=4 - [AK1C3_HUMAN]	-0.2
P34897	Serine hydroxymethyltransferase, mitochondrial OS=Homo sapiens GN=SHMT2 PE=1 SV=3 - [GLYM_HUMAN]	-0.2
Q8NBU5	ATPase family AAA domain-containing protein 1 OS=Homo sapiens GN=ATAD1 PE=1 SV=1 - [ATAD1_HUMAN]	-0.2
P23284	Peptidyl-prolyl cis-trans isomerase B OS=Homo sapiens GN=PPIB PE=1 SV=2 - [PPIB_HUMAN]	-0.2
P05067	Amyloid beta A4 protein OS=Homo sapiens GN=APP PE=1 SV=3 - [A4_HUMAN]	-0.2
Q9P1Z2	Calcium-binding and coiled-coil domain-containing protein 1 OS=Homo sapiens GN=CALCOCO1 PE=1 SV=2 - [CACO1_HUMAN]	-0.2
Q01085	Nucleolysin TIAR OS=Homo sapiens GN=TIAL1 PE=1 SV=1 - [TIAR_HUMAN]	-0.2
Q5T3I0	G patch domain-containing protein 4 OS=Homo sapiens GN=GPATCH4 PE=1 SV=2 - [GPTC4_HUMAN]	-0.2
Q9UIF9	Bromodomain adjacent to zinc finger domain protein 2A OS=Homo sapiens GN=BAZ2A PE=1 SV=4 - [BAZ2A_HUMAN]	-0.2
P21359	Neurofibromin OS=Homo sapiens GN=NF1 PE=1 SV=2 - [NF1_HUMAN]	-0.2
Q15050	Ribosome biogenesis regulatory protein homolog OS=Homo sapiens GN=RRS1 PE=1 SV=2 - [RRS1_HUMAN]	-0.2

P35475	Alpha-L-iduronidase OS=Homo sapiens GN=IDUA PE=1 SV=2 - [IDUA_HUMAN]	-0.2
P61019	Ras-related protein Rab-2A OS=Homo sapiens GN=RAB2A PE=1 SV=1 - [RAB2A_HUMAN]	-0.2
P00403	Cytochrome c oxidase subunit 2 OS=Homo sapiens GN=MT-CO2 PE=1 SV=1 - [COX2_HUMAN]	-0.2
P68104	Elongation factor 1-alpha 1 OS=Homo sapiens GN=EEF1A1 PE=1 SV=1 - [EF1A1_HUMAN]	-0.2
Q9BZQ6	ER degradation-enhancing alpha-mannosidase-like protein 3 OS=Homo sapiens GN=EDEM3 PE=1 SV=2 - [EDEM3_HUMAN]	-0.2
P15374	Ubiquitin carboxyl-terminal hydrolase isozyme L3 OS=Homo sapiens GN=UCHL3 PE=1 SV=1 - [UCHL3_HUMAN]	-0.2
Q9UHV9	Prefoldin subunit 2 OS=Homo sapiens GN=PFDN2 PE=1 SV=1 - [PFD2_HUMAN]	-0.2
Q13444	Disintegrin and metalloproteinase domain-containing protein 15 OS=Homo sapiens GN=ADAM15 PE=1 SV=4 - [ADA15_HUMAN]	-0.2
Q08AF3	Schlafen family member 5 OS=Homo sapiens GN=SLFN5 PE=1 SV=1 - [SLFN5_HUMAN]	-0.2
P60842	Eukaryotic initiation factor 4A-I OS=Homo sapiens GN=EIF4A1 PE=1 SV=1 - [IF4A1_HUMAN]	-0.2
Q7Z6B7	SLIT-ROBO Rho GTPase-activating protein 1 OS=Homo sapiens GN=SRGAP1 PE=1 SV=1 - [SRGP1_HUMAN]	-0.2
O75494	Serine/arginine-rich splicing factor 10 OS=Homo sapiens GN=SRSF10 PE=1 SV=1 - [SRS10_HUMAN]	-0.2
Q13643	Four and a half LIM domains protein 3 OS=Homo sapiens GN=FHL3 PE=1 SV=4 - [FHL3_HUMAN]	-0.2
Q8TAE8	Growth arrest and DNA damage-inducible proteins-interacting protein 1 OS=Homo sapiens GN=GADD45GIP1 PE=1 SV=1 - [G45IP_HUMAN]	-0.2
Q9BZH6	WD repeat-containing protein 11 OS=Homo sapiens GN=WDR11 PE=1 SV=1 - [WDR11_HUMAN]	-0.2
Q6DKJ4	Nucleoredoxin OS=Homo sapiens GN=NXN PE=1 SV=2 - [NXN_HUMAN]	-0.2
Q9Y5Y2	Cytosolic Fe-S cluster assembly factor NUBP2 OS=Homo sapiens GN=NUBP2 PE=1 SV=1 - [NUBP2_HUMAN]	-0.2
P41236	Protein phosphatase inhibitor 2 OS=Homo sapiens GN=PPP1R2 PE=1 SV=2 - [IPP2_HUMAN]	-0.2
P31431	Syndecan-4 OS=Homo sapiens GN=SDC4 PE=1 SV=2 - [SDC4_HUMAN]	-0.2
P54802	Alpha-N-acetylglucosaminidase OS=Homo sapiens GN=NAGLU PE=1 SV=2 - [ANAG_HUMAN]	-0.2
O15321	Transmembrane 9 superfamily member 1 OS=Homo sapiens GN=TM9SF1 PE=2 SV=2 - [TM9S1_HUMAN]	-0.2
O15126	Secretory carrier-associated membrane protein 1 OS=Homo sapiens GN=SCAMP1 PE=1 SV=2 - [SCAM1_HUMAN]	-0.2
P05198	Eukaryotic translation initiation factor 2 subunit 1 OS=Homo sapiens GN=EIF2S1 PE=1 SV=3 - [IF2A_HUMAN]	-0.2
P54252	Ataxin-3 OS=Homo sapiens GN=ATXN3 PE=1 SV=4 - [ATX3_HUMAN]	-0.2
Q5VWJ9	Sorting nexin-30 OS=Homo sapiens GN=SNX30 PE=1 SV=1 - [SNX30_HUMAN]	-0.2
Q32MZ4	Leucine-rich repeat flightless-interacting protein 1 OS=Homo sapiens GN=LRRFIP1 PE=1 SV=2 - [LRRF1_HUMAN]	-0.2
O95714	E3 ubiquitin-protein ligase HERC2 OS=Homo sapiens GN=HERC2 PE=1 SV=2 - [HERC2_HUMAN]	-0.2
P07195	L-lactate dehydrogenase B chain OS=Homo sapiens GN=LDHB PE=1 SV=2 - [LDHB_HUMAN]	-0.2
Q86X55	Histone-arginine methyltransferase CARM1 OS=Homo sapiens GN=CARM1 PE=1 SV=3 - [CARM1_HUMAN]	-0.2
P04843	Dolichyl-diphosphooligosaccharide--protein glycosyltransferase subunit 1 OS=Homo sapiens GN=RPN1 PE=1 SV=1 - [RPN1_HUMAN]	-0.2
Q8IV08	Phospholipase D3 OS=Homo sapiens GN=PLD3 PE=1 SV=1 - [PLD3_HUMAN]	-0.2
Q9NP61	ADP-ribosylation factor GTPase-activating protein 3 OS=Homo sapiens GN=ARFGAP3 PE=1 SV=1 - [ARFG3_HUMAN]	-0.2
O43242	26S proteasome non-ATPase regulatory subunit 3 OS=Homo sapiens GN=PSMD3 PE=1 SV=2 - [PSMD3_HUMAN]	-0.2
P15498	Proto-oncogene vav OS=Homo sapiens GN=VAV1 PE=1 SV=4 - [VAV_HUMAN]	-0.2
P06865	Beta-hexosaminidase subunit alpha OS=Homo sapiens GN=HEXA PE=1 SV=2 - [HEXA_HUMAN]	-0.2
O60331	Phosphatidylinositol 4-phosphate 5-kinase type-1 gamma OS=Homo sapiens GN=PIP5K1C PE=1 SV=2 - [PI51C_HUMAN]	-0.2
Q13404	Ubiquitin-conjugating enzyme E2 variant 1 OS=Homo sapiens GN=UBE2V1 PE=1 SV=2 - [UB2V1_HUMAN]	-0.2
Q9UNM6	26S proteasome non-ATPase regulatory subunit 13 OS=Homo sapiens GN=PSMD13 PE=1 SV=2 - [PSD13_HUMAN]	-0.2
O75787	Renin receptor OS=Homo sapiens GN=ATP6AP2 PE=1 SV=2 - [RENR_HUMAN]	-0.2
Q16864	V-type proton ATPase subunit F OS=Homo sapiens GN=ATP6V1F PE=1 SV=2 - [VATF_HUMAN]	-0.2
P25789	Proteasome subunit alpha type-4 OS=Homo sapiens GN=PSMA4 PE=1 SV=1 - [PSA4_HUMAN]	-0.2
Q9Y5K8	V-type proton ATPase subunit D OS=Homo sapiens GN=ATP6V1D PE=1 SV=1 - [VATD_HUMAN]	-0.2
Q7L576	Cytoplasmic FMR1-interacting protein 1 OS=Homo sapiens GN=CYFIP1 PE=1 SV=1 - [CYFP1_HUMAN]	-0.2
Q9UQ80	Proliferation-associated protein 2G4 OS=Homo sapiens GN=PA2G4 PE=1 SV=3 - [PA2G4_HUMAN]	-0.2
Q13155	Aminoacyl tRNA synthase complex-interacting multifunctional protein 2 OS=Homo sapiens GN=AIMP2 PE=1 SV=2 - [AIMP2_HUMAN]	-0.2
Q5VT25	Serine/threonine-protein kinase MRCK alpha OS=Homo sapiens GN=CDC42BPA PE=1 SV=1 - [MRCKA_HUMAN]	-0.2
P29966	Myristoylated alanine-rich C-kinase substrate OS=Homo sapiens GN=MARCKS PE=1 SV=4 - [MARCS_HUMAN]	-0.2
P25445	Tumor necrosis factor receptor superfamily member 6 OS=Homo sapiens GN=FAS PE=1 SV=1 - [TNR6_HUMAN]	-0.2
Q9HBR0	Putative sodium-coupled neutral amino acid transporter 10 OS=Homo sapiens GN=SLC38A10 PE=1 SV=2 - [S38AA_HUMAN]	-0.2
Q9NX05	Constitutive coactivator of PPAR-gamma-like protein 2 OS=Homo sapiens GN=FAM120C PE=2 SV=3 - [F120C_HUMAN]	-0.2
Q9Y696	Chloride intracellular channel protein 4 OS=Homo sapiens GN=CLIC4 PE=1 SV=4 - [CLIC4_HUMAN]	-0.2
Q9Y3U8	60S ribosomal protein L36 OS=Homo sapiens GN=RPL36 PE=1 SV=3 - [RL36_HUMAN]	-0.2

P27708	CAD protein OS=Homo sapiens GN=CAD PE=1 SV=3 - [PYR1_HUMAN]	-0.2
Q9UJ41	Rab5 GDP/GTP exchange factor OS=Homo sapiens GN=RABGEF1 PE=1 SV=2 - [RABX5_HUMAN]	-0.2
A1L0T0	Acetolactate synthase-like protein OS=Homo sapiens GN=ILVBL PE=1 SV=2 - [ILVBL_HUMAN]	-0.2
Q9UP95	Solute carrier family 12 member 4 OS=Homo sapiens GN=SLC12A4 PE=1 SV=2 - [S12A4_HUMAN]	-0.2
O43493	Trans-Golgi network integral membrane protein 2 OS=Homo sapiens GN=TGOLN2 PE=1 SV=2 - [TGON2_HUMAN]	-0.2
Q86VP6	Cullin-associated NEDD8-dissociated protein 1 OS=Homo sapiens GN=CAND1 PE=1 SV=2 - [CAND1_HUMAN]	-0.2
Q13740	CD166 antigen OS=Homo sapiens GN=ALCAM PE=1 SV=2 - [CD166_HUMAN]	-0.2
P61011	Signal recognition particle 54 kDa protein OS=Homo sapiens GN=SRP54 PE=1 SV=1 - [SRP54_HUMAN]	-0.2
Q9BS26	Endoplasmic reticulum resident protein 44 OS=Homo sapiens GN=ERP44 PE=1 SV=1 - [ERP44_HUMAN]	-0.2
Q96IU4	Alpha/beta hydrolase domain-containing protein 14B OS=Homo sapiens GN=ABHD14B PE=1 SV=1 - [ABHEB_HUMAN]	-0.2
Q9GZZ9	Ubiquitin-like modifier-activating enzyme 5 OS=Homo sapiens GN=UBA5 PE=1 SV=1 - [UBA5_HUMAN]	-0.2
Q9H3H3	UPF0696 protein C11orf68 OS=Homo sapiens GN=C11orf68 PE=1 SV=2 - [CK068_HUMAN]	-0.2
Q5ZPR3	CD276 antigen OS=Homo sapiens GN=CD276 PE=1 SV=1 - [CD276_HUMAN]	-0.2
O15037	Protein KHNYN OS=Homo sapiens GN=KHNYN PE=2 SV=3 - [KHNYN_HUMAN]	-0.2
Q96A49	Synapse-associated protein 1 OS=Homo sapiens GN=SYAP1 PE=1 SV=1 - [SYAP1_HUMAN]	-0.2
P25490	Transcriptional repressor protein YY1 OS=Homo sapiens GN=YY1 PE=1 SV=2 - [TYY1_HUMAN]	-0.2
Q9NYM9	BET1-like protein OS=Homo sapiens GN=BET1L PE=1 SV=1 - [BET1L_HUMAN]	-0.2
P62873	Guanine nucleotide-binding protein G(I)/G(S)/G(T) subunit beta-1 OS=Homo sapiens GN=GNB1 PE=1 SV=3 - [GBB1_HUMAN]	-0.2
Q68D91	Metallo-beta-lactamase domain-containing protein 2 OS=Homo sapiens GN=MBLAC2 PE=1 SV=3 - [MBLC2_HUMAN]	-0.2
P35610	Sterol O-acyltransferase 1 OS=Homo sapiens GN=SOAT1 PE=1 SV=3 - [SOAT1_HUMAN]	-0.2
P14735	Insulin-degrading enzyme OS=Homo sapiens GN=IDE PE=1 SV=4 - [IDE_HUMAN]	-0.2
P54136	Arginine--tRNA ligase, cytoplasmic OS=Homo sapiens GN=RARS PE=1 SV=2 - [SYRC_HUMAN]	-0.2
Q9UJY1	Heat shock protein beta-8 OS=Homo sapiens GN=HSPB8 PE=1 SV=1 - [HSPB8_HUMAN]	-0.2
Q96F24	Nuclear receptor-binding factor 2 OS=Homo sapiens GN=NRBF2 PE=1 SV=1 - [NRBF2_HUMAN]	-0.2
Q9NXR7	BRCA1-A complex subunit BRE OS=Homo sapiens GN=BRE PE=1 SV=2 - [BRE_HUMAN]	-0.2
P62854	40S ribosomal protein S26 OS=Homo sapiens GN=RPS26 PE=1 SV=3 - [RS26_HUMAN]	-0.2
Q8NBJ4	Golgi membrane protein 1 OS=Homo sapiens GN=GOLM1 PE=1 SV=1 - [GOLM1_HUMAN]	-0.2
P80217	Interferon-induced 35 kDa protein OS=Homo sapiens GN=IFI35 PE=1 SV=5 - [IN35_HUMAN]	-0.2
O95453	Poly(A)-specific ribonuclease PARN OS=Homo sapiens GN=PARN PE=1 SV=1 - [PARN_HUMAN]	-0.2
Q9Y5N5	HemK methyltransferase family member 2 OS=Homo sapiens GN=N6AMT1 PE=1 SV=3 - [HEMK2_HUMAN]	-0.2
Q9BTW9	Tubulin-specific chaperone D OS=Homo sapiens GN=TBCD PE=1 SV=2 - [TBCD_HUMAN]	-0.2
P58335	Anthrax toxin receptor 2 OS=Homo sapiens GN=ANTXR2 PE=1 SV=5 - [ANTR2_HUMAN]	-0.2
P08240	Signal recognition particle receptor subunit alpha OS=Homo sapiens GN=SRPR PE=1 SV=2 - [SRPR_HUMAN]	-0.2
P52907	F-actin-capping protein subunit alpha-1 OS=Homo sapiens GN=CAPZA1 PE=1 SV=3 - [CAZA1_HUMAN]	-0.2
Q9P2K8	Eukaryotic translation initiation factor 2-alpha kinase 4 OS=Homo sapiens GN=EIF2AK4 PE=1 SV=3 - [E2AK4_HUMAN]	-0.2
O00115	Deoxyribonuclease-2-alpha OS=Homo sapiens GN=DNASE2 PE=1 SV=2 - [DNS2A_HUMAN]	-0.2
P42858	Huntingtin OS=Homo sapiens GN=HTT PE=1 SV=2 - [HD_HUMAN]	-0.2
Q9Y2S2	Lambda-crystallin homolog OS=Homo sapiens GN=CRYL1 PE=1 SV=3 - [CRYL1_HUMAN]	-0.2
O75569	Interferon-inducible double-stranded RNA-dependent protein kinase activator A OS=Homo sapiens GN=PRKRA PE=1 SV=1 - [PRKRA_HUMAN]	-0.2
O75143	Autophagy-related protein 13 OS=Homo sapiens GN=ATG13 PE=1 SV=1 - [ATG13_HUMAN]	-0.2
Q04206	Transcription factor p65 OS=Homo sapiens GN=RELA PE=1 SV=2 - [TF65_HUMAN]	-0.2
Q8WTT2	Nucleolar complex protein 3 homolog OS=Homo sapiens GN=NOC3L PE=1 SV=1 - [NOC3L_HUMAN]	-0.2
Q9ULU4	Protein kinase C-binding protein 1 OS=Homo sapiens GN=ZMYND8 PE=1 SV=2 - [PKCB1_HUMAN]	-0.2
Q16881	Thioredoxin reductase 1, cytoplasmic OS=Homo sapiens GN=TXNRD1 PE=1 SV=3 - [TRXR1_HUMAN]	-0.2
Q9H3N1	Thioredoxin-related transmembrane protein 1 OS=Homo sapiens GN=TMX1 PE=1 SV=1 - [TMX1_HUMAN]	-0.2
Q9ULV4	Coronin-1C OS=Homo sapiens GN=CORO1C PE=1 SV=1 - [COR1C_HUMAN]	-0.2
Q16342	Programmed cell death protein 2 OS=Homo sapiens GN=PDCD2 PE=1 SV=2 - [PDCD2_HUMAN]	-0.2
P48444	Coatomer subunit delta OS=Homo sapiens GN=ARCN1 PE=1 SV=1 - [COPD_HUMAN]	-0.2
Q9H936	Mitochondrial glutamate carrier 1 OS=Homo sapiens GN=SLC25A22 PE=1 SV=1 - [GHC1_HUMAN]	-0.2
O43795	Unconventional myosin-Ib OS=Homo sapiens GN=MYO1B PE=1 SV=3 - [MYO1B_HUMAN]	-0.2
Q5EBL4	RILP-like protein 1 OS=Homo sapiens GN=RILPL1 PE=1 SV=1 - [RIPL1_HUMAN]	-0.2
Q92734	Protein TFG OS=Homo sapiens GN=TFG PE=1 SV=2 - [TFG_HUMAN]	-0.2
P55268	Laminin subunit beta-2 OS=Homo sapiens GN=LAMB2 PE=1 SV=2 - [LAMB2_HUMAN]	-0.2

Q8IU85	Calcium/calmodulin-dependent protein kinase type 1D OS=Homo sapiens GN=CAMK1D PE=1 SV=1 - [KCC1D_HUMAN]	-0.2
P12955	Xaa-Pro dipeptidase OS=Homo sapiens GN=PEPD PE=1 SV=3 - [PEPD_HUMAN]	-0.2
P50579	Methionine aminopeptidase 2 OS=Homo sapiens GN=METAP2 PE=1 SV=1 - [MAP2_HUMAN]	-0.2
P51798	H(+)/Cl(-) exchange transporter 7 OS=Homo sapiens GN=CLCN7 PE=1 SV=2 - [CLCN7_HUMAN]	-0.2
Q8IXI2	Mitochondrial Rho GTPase 1 OS=Homo sapiens GN=RHOT1 PE=1 SV=2 - [MIRO1_HUMAN]	-0.2
Q70UQ0	Inhibitor of nuclear factor kappa-B kinase-interacting protein OS=Homo sapiens GN=IKBIP PE=1 SV=1 - [IKIP_HUMAN]	-0.2
Q8WWX9	Selenoprotein M OS=Homo sapiens GN=SELM PE=1 SV=3 - [SELM_HUMAN]	-0.2
P30519	Heme oxygenase 2 OS=Homo sapiens GN=HMOX2 PE=1 SV=2 - [HMOX2_HUMAN]	-0.2
P61599	N-alpha-acetyltransferase 20 OS=Homo sapiens GN=NAA20 PE=1 SV=1 - [NAA20_HUMAN]	-0.2
P46776	60S ribosomal protein L27a OS=Homo sapiens GN=RPL27A PE=1 SV=2 - [RL27A_HUMAN]	-0.2
Q14005	Pro-interleukin-16 OS=Homo sapiens GN=IL16 PE=1 SV=4 - [IL16_HUMAN]	-0.2
Q6NXE6	Armadillo repeat-containing protein 6 OS=Homo sapiens GN=ARMC6 PE=1 SV=2 - [ARMC6_HUMAN]	-0.2
P83731	60S ribosomal protein L24 OS=Homo sapiens GN=RPL24 PE=1 SV=1 - [RL24_HUMAN]	-0.2
Q5GLZ8	Probable E3 ubiquitin-protein ligase HERC4 OS=Homo sapiens GN=HERC4 PE=1 SV=1 - [HERC4_HUMAN]	-0.2
Q9Y5L0	Transportin-3 OS=Homo sapiens GN=TNPO3 PE=1 SV=3 - [TNPO3_HUMAN]	-0.2
P12931	Proto-oncogene tyrosine-protein kinase Src OS=Homo sapiens GN=SRC PE=1 SV=3 - [SRC_HUMAN]	-0.2
Q13200	26S proteasome non-ATPase regulatory subunit 2 OS=Homo sapiens GN=PSMD2 PE=1 SV=3 - [PSMD2_HUMAN]	-0.2
P42766	60S ribosomal protein L35 OS=Homo sapiens GN=RPL35 PE=1 SV=2 - [RL35_HUMAN]	-0.2
P17050	Alpha-N-acetylgalactosaminidase OS=Homo sapiens GN=NAGA PE=1 SV=2 - [NAGAB_HUMAN]	-0.2
Q9UGP8	Translocation protein SEC63 homolog OS=Homo sapiens GN=SEC63 PE=1 SV=2 - [SEC63_HUMAN]	-0.2
Q8TEX9	Importin-4 OS=Homo sapiens GN=IPO4 PE=1 SV=2 - [IPO4_HUMAN]	-0.2
Q13488	V-type proton ATPase 116 kDa subunit a isoform 3 OS=Homo sapiens GN=TCIRG1 PE=1 SV=3 - [VPP3_HUMAN]	-0.2
Q8NEU8	DCC-interacting protein 13-beta OS=Homo sapiens GN=APPL2 PE=1 SV=3 - [DP13B_HUMAN]	-0.2
Q14393	Growth arrest-specific protein 6 OS=Homo sapiens GN=GAS6 PE=1 SV=2 - [GAS6_HUMAN]	-0.2
O60701	UDP-glucose 6-dehydrogenase OS=Homo sapiens GN=UGDH PE=1 SV=1 - [UGDH_HUMAN]	-0.2
Q9UBG0	C-type mannose receptor 2 OS=Homo sapiens GN=MRC2 PE=1 SV=2 - [MRC2_HUMAN]	-0.2
Q8NBJ5	Procollagen galactosyltransferase 1 OS=Homo sapiens GN=COLGALT1 PE=1 SV=1 - [GT251_HUMAN]	-0.2
Q96KA5	Cleft lip and palate transmembrane protein 1-like protein OS=Homo sapiens GN=CLPTM1L PE=1 SV=1 - [CLP1L_HUMAN]	-0.2
O60503	Adenylate cyclase type 9 OS=Homo sapiens GN=ADCY9 PE=1 SV=4 - [ADCY9_HUMAN]	-0.2
P12109	Collagen alpha-1(VI) chain OS=Homo sapiens GN=COL6A1 PE=1 SV=3 - [CO6A1_HUMAN]	-0.2
Q15369	Transcription elongation factor B polypeptide 1 OS=Homo sapiens GN=TCEB1 PE=1 SV=1 - [ELOC_HUMAN]	-0.2
P14868	Aspartate--tRNA ligase, cytoplasmic OS=Homo sapiens GN=DARS PE=1 SV=2 - [SYDC_HUMAN]	-0.2
P42566	Epidermal growth factor receptor substrate 15 OS=Homo sapiens GN=EPS15 PE=1 SV=2 - [EPS15_HUMAN]	-0.2
P28161	Glutathione S-transferase Mu 2 OS=Homo sapiens GN=GSTM2 PE=1 SV=2 - [GSTM2_HUMAN]	-0.2
Q9BQ24	Zinc finger FYVE domain-containing protein 21 OS=Homo sapiens GN=ZFYVE21 PE=1 SV=1 - [ZFY21_HUMAN]	-0.2
Q96FQ6	Protein S100-A16 OS=Homo sapiens GN=S100A16 PE=1 SV=1 - [S10AG_HUMAN]	-0.2
Q9H1E3	Nuclear ubiquitous casein and cyclin-dependent kinase substrate 1 OS=Homo sapiens GN=NUCKS1 PE=1 SV=1 - [NUCKS_HUMAN]	-0.2
O00499	Myc box-dependent-interacting protein 1 OS=Homo sapiens GN=BIN1 PE=1 SV=1 - [BIN1_HUMAN]	-0.2
Q5VU43	Myomegalin OS=Homo sapiens GN=PDE4DIP PE=1 SV=1 - [MYOME_HUMAN]	-0.2
Q13948	Protein CASP OS=Homo sapiens GN=CUX1 PE=1 SV=2 - [CASP_HUMAN]	-0.2
Q9P2E5	Chondroitin sulfate glucuronyltransferase OS=Homo sapiens GN=CHPF2 PE=2 SV=2 - [CHPF2_HUMAN]	-0.2
O43157	Plexin-B1 OS=Homo sapiens GN=PLXNB1 PE=1 SV=3 - [PLXB1_HUMAN]	-0.2
Q71RC2	La-related protein 4 OS=Homo sapiens GN=LARP4 PE=1 SV=3 - [LARP4_HUMAN]	-0.2
O75821	Eukaryotic translation initiation factor 3 subunit G OS=Homo sapiens GN=EIF3G PE=1 SV=2 - [EIF3G_HUMAN]	-0.2
P13639	Elongation factor 2 OS=Homo sapiens GN=EEF2 PE=1 SV=4 - [EF2_HUMAN]	-0.2
Q96EL2	28S ribosomal protein S24, mitochondrial OS=Homo sapiens GN=MRPS24 PE=1 SV=1 - [RT24_HUMAN]	-0.2
Q8IWZ3	Ankyrin repeat and KH domain-containing protein 1 OS=Homo sapiens GN=ANKHD1 PE=1 SV=1 - [ANKH1_HUMAN]	-0.2
Q16658	Fascin OS=Homo sapiens GN=FSCN1 PE=1 SV=3 - [FSCN1_HUMAN]	-0.2
O95273	Cyclin-D1-binding protein 1 OS=Homo sapiens GN=CCNDBP1 PE=1 SV=2 - [CCDB1_HUMAN]	-0.2
Q9NX58	Cell growth-regulating nucleolar protein OS=Homo sapiens GN=LYAR PE=1 SV=2 - [LYAR_HUMAN]	-0.2
P06756	Integrin alpha-V OS=Homo sapiens GN=ITGAV PE=1 SV=2 - [ITAV_HUMAN]	-0.2
Q96RS6	NudC domain-containing protein 1 OS=Homo sapiens GN=NUDCD1 PE=1 SV=2 - [NUDC1_HUMAN]	-0.2

P17568	NADH dehydrogenase [ubiquinone] 1 beta subcomplex subunit 7 OS=Homo sapiens GN=NDUFB7 PE=1 SV=4 - [NDUB7_HUMAN]	-0.2
O95486	Protein transport protein Sec24A OS=Homo sapiens GN=SEC24A PE=1 SV=2 - [SC24A_HUMAN]	-0.2
P15144	Aminopeptidase N OS=Homo sapiens GN=ANPEP PE=1 SV=4 - [AMPN_HUMAN]	-0.2
O00560	Syntenin-1 OS=Homo sapiens GN=SDCBP PE=1 SV=1 - [SDCB1_HUMAN]	-0.2
O95782	AP-2 complex subunit alpha-1 OS=Homo sapiens GN=AP2A1 PE=1 SV=3 - [AP2A1_HUMAN]	-0.2
P28331	NADH-ubiquinone oxidoreductase 75 kDa subunit, mitochondrial OS=Homo sapiens GN=NDUFS1 PE=1 SV=3 - [NDUS1_HUMAN]	-0.2
Q13765	Nascent polypeptide-associated complex subunit alpha OS=Homo sapiens GN=NACA PE=1 SV=1 - [NACA_HUMAN]	-0.2
Q96AJ9	Vesicle transport through interaction with t-SNAREs homolog 1A OS=Homo sapiens GN=VTI1A PE=1 SV=2 - [VTI1A_HUMAN]	-0.2
P61916	Epididymal secretory protein E1 OS=Homo sapiens GN=NPC2 PE=1 SV=1 - [NPC2_HUMAN]	-0.2
Q99848	Probable rRNA-processing protein EBP2 OS=Homo sapiens GN=EBNA1BP2 PE=1 SV=2 - [EBP2_HUMAN]	-0.2
Q6RFH5	WD repeat-containing protein 74 OS=Homo sapiens GN=WDR74 PE=1 SV=1 - [WDR74_HUMAN]	-0.2
O43847	Nardilysin OS=Homo sapiens GN=NRD1 PE=1 SV=2 - [NRDC_HUMAN]	-0.2
Q9NQC3	Reticulon-4 OS=Homo sapiens GN=RTN4 PE=1 SV=2 - [RTN4_HUMAN]	-0.2
Q86Y38	Xylosyltransferase 1 OS=Homo sapiens GN=XYLT1 PE=1 SV=1 - [XYLT1_HUMAN]	-0.2
Q99536	Synaptic vesicle membrane protein VAT-1 homolog OS=Homo sapiens GN=VAT1 PE=1 SV=2 - [VAT1_HUMAN]	-0.2
P51668	Ubiquitin-conjugating enzyme E2 D1 OS=Homo sapiens GN=UBE2D1 PE=1 SV=1 - [UB2D1_HUMAN]	-0.2
O14656	Torsin-1A OS=Homo sapiens GN=TOR1A PE=1 SV=1 - [TOR1A_HUMAN]	-0.2
Q07065	Cytoskeleton-associated protein 4 OS=Homo sapiens GN=CKAP4 PE=1 SV=2 - [CKAP4_HUMAN]	-0.2
Q04446	1,4-alpha-glucan-branching enzyme OS=Homo sapiens GN=GBE1 PE=1 SV=3 - [GLGB_HUMAN]	-0.2
P78362	SRSF protein kinase 2 OS=Homo sapiens GN=SRPK2 PE=1 SV=3 - [SRPK2_HUMAN]	-0.2
Q68CQ7	Glycosyltransferase 8 domain-containing protein 1 OS=Homo sapiens GN=GLT8D1 PE=1 SV=2 - [GL8D1_HUMAN]	-0.2
Q15139	Serine/threonine-protein kinase D1 OS=Homo sapiens GN=PRKD1 PE=1 SV=2 - [KPCD1_HUMAN]	-0.2
Q9NR30	Nucleolar RNA helicase 2 OS=Homo sapiens GN=DDX21 PE=1 SV=5 - [DDX21_HUMAN]	-0.2
O00541	Pescadillo homolog OS=Homo sapiens GN=PES1 PE=1 SV=1 - [PESC_HUMAN]	-0.2
Q13619	Cullin-4A OS=Homo sapiens GN=CUL4A PE=1 SV=3 - [CUL4A_HUMAN]	-0.2
Q8IWX8	Calcium homeostasis endoplasmic reticulum protein OS=Homo sapiens GN=CHERP PE=1 SV=3 - [CHERP_HUMAN]	-0.2
P30039	Phenazine biosynthesis-like domain-containing protein OS=Homo sapiens GN=PBLD PE=1 SV=2 - [PBLD_HUMAN]	-0.2
Q00536	Cyclin-dependent kinase 16 OS=Homo sapiens GN=CDK16 PE=1 SV=1 - [CDK16_HUMAN]	-0.2
P30050	60S ribosomal protein L12 OS=Homo sapiens GN=RPL12 PE=1 SV=1 - [RL12_HUMAN]	-0.2
Q9UNK0	Syntaxin-8 OS=Homo sapiens GN=STX8 PE=1 SV=2 - [STX8_HUMAN]	-0.2
O94885	SAM and SH3 domain-containing protein 1 OS=Homo sapiens GN=SASH1 PE=1 SV=3 - [SASH1_HUMAN]	-0.2
O00244	Copper transport protein ATOX1 OS=Homo sapiens GN=ATOX1 PE=1 SV=1 - [ATOX1_HUMAN]	-0.2
Q9Y6M9	NADH dehydrogenase [ubiquinone] 1 beta subcomplex subunit 9 OS=Homo sapiens GN=NDUFB9 PE=1 SV=3 - [NDUB9_HUMAN]	-0.2
P49841	Glycogen synthase kinase-3 beta OS=Homo sapiens GN=GSK3B PE=1 SV=2 - [GSK3B_HUMAN]	-0.2
Q9C0E2	Exportin-4 OS=Homo sapiens GN=XPO4 PE=1 SV=2 - [XPO4_HUMAN]	-0.2
Q6UWH4	Protein FAM198B OS=Homo sapiens GN=FAM198B PE=2 SV=1 - [F198B_HUMAN]	-0.2
P55060	Exportin-2 OS=Homo sapiens GN=CSE1L PE=1 SV=3 - [XPO2_HUMAN]	-0.2
O95816	BAG family molecular chaperone regulator 2 OS=Homo sapiens GN=BAG2 PE=1 SV=1 - [BAG2_HUMAN]	-0.2
P62847	40S ribosomal protein S24 OS=Homo sapiens GN=RPS24 PE=1 SV=1 - [RS24_HUMAN]	-0.2
Q9BZQ8	Protein Niban OS=Homo sapiens GN=FAM129A PE=1 SV=1 - [NIBAN_HUMAN]	-0.2
O95249	Golgi SNAP receptor complex member 1 OS=Homo sapiens GN=GOSR1 PE=1 SV=1 - [GOSR1_HUMAN]	-0.2
Q9H993	UPF0364 protein C6orf211 OS=Homo sapiens GN=C6orf211 PE=1 SV=1 - [CF211_HUMAN]	-0.2
P05386	60S acidic ribosomal protein P1 OS=Homo sapiens GN=RPLP1 PE=1 SV=1 - [RLA1_HUMAN]	-0.2
Q9Y3A6	Transmembrane emp24 domain-containing protein 5 OS=Homo sapiens GN=TMED5 PE=1 SV=1 - [TMED5_HUMAN]	-0.2
P46736	Lys-63-specific deubiquitinase BRCC36 OS=Homo sapiens GN=BRCC3 PE=1 SV=2 - [BRCC3_HUMAN]	-0.2
Q9ULP9	TBC1 domain family member 24 OS=Homo sapiens GN=TBC1D24 PE=1 SV=2 - [TBC24_HUMAN]	-0.2
Q13510	Acid ceramidase OS=Homo sapiens GN=ASAH1 PE=1 SV=5 - [ASAH1_HUMAN]	-0.2
Q96EB6	NAD-dependent protein deacetylase sirtuin-1 OS=Homo sapiens GN=SIRT1 PE=1 SV=2 - [SIR1_HUMAN]	-0.2
Q5T4S7	E3 ubiquitin-protein ligase UBR4 OS=Homo sapiens GN=UBR4 PE=1 SV=1 - [UBR4_HUMAN]	-0.2
O00148	ATP-dependent RNA helicase DDX39A OS=Homo sapiens GN=DDX39A PE=1 SV=2 - [DX39A_HUMAN]	-0.2
Q13042	Cell division cycle protein 16 homolog OS=Homo sapiens GN=CDC16 PE=1 SV=2 - [CDC16_HUMAN]	-0.2
Q9NWU2	Glucose-induced degradation protein 8 homolog OS=Homo sapiens GN=GID8 PE=1 SV=1 - [GID8_HUMAN]	-0.2

Q53H96	Pyrroline-5-carboxylate reductase 3 OS=Homo sapiens GN=PYCRL PE=1 SV=3 - [P5CR3_HUMAN]	-0.2
Q9ULC4	Malignant T-cell-amplified sequence 1 OS=Homo sapiens GN=MCTS1 PE=1 SV=1 - [MCTS1_HUMAN]	-0.3
O95573	Long-chain-fatty-acid--CoA ligase 3 OS=Homo sapiens GN=ACSL3 PE=1 SV=3 - [ACSL3_HUMAN]	-0.3
Q9Y5V3	Melanoma-associated antigen D1 OS=Homo sapiens GN=MAGED1 PE=1 SV=3 - [MAGD1_HUMAN]	-0.3
P05387	60S acidic ribosomal protein P2 OS=Homo sapiens GN=RPLP2 PE=1 SV=1 - [RLA2_HUMAN]	-0.3
Q9UBS8	E3 ubiquitin-protein ligase RNF14 OS=Homo sapiens GN=RNF14 PE=1 SV=1 - [RNF14_HUMAN]	-0.3
P29992	Guanine nucleotide-binding protein subunit alpha-11 OS=Homo sapiens GN=GNA11 PE=1 SV=2 - [GNA11_HUMAN]	-0.3
O14980	Exportin-1 OS=Homo sapiens GN=XPO1 PE=1 SV=1 - [XPO1_HUMAN]	-0.3
Q6NW29	RWD domain-containing protein 4 OS=Homo sapiens GN=RWDD4 PE=1 SV=3 - [RWDD4_HUMAN]	-0.3
P48723	Heat shock 70 kDa protein 13 OS=Homo sapiens GN=HSPA13 PE=1 SV=1 - [HSP13_HUMAN]	-0.3
Q07954	Prolow-density lipoprotein receptor-related protein 1 OS=Homo sapiens GN=LRP1 PE=1 SV=2 - [LRP1_HUMAN]	-0.3
Q8NBM8	Prenylcysteine oxidase-like OS=Homo sapiens GN=PCYOX1L PE=1 SV=2 - [PCYXL_HUMAN]	-0.3
Q13287	N-myc-interactor OS=Homo sapiens GN=NMI PE=1 SV=2 - [NMI_HUMAN]	-0.3
Q9UPN7	Serine/threonine-protein phosphatase 6 regulatory subunit 1 OS=Homo sapiens GN=PPP6R1 PE=1 SV=5 - [PP6R1_HUMAN]	-0.3
Q5T013	Putative hydroxypyruvate isomerase OS=Homo sapiens GN=HYI PE=1 SV=2 - [HYI_HUMAN]	-0.3
O00400	Acetyl-coenzyme A transporter 1 OS=Homo sapiens GN=SLC33A1 PE=1 SV=1 - [ACATN_HUMAN]	-0.3
Q9H6X2	Anthrax toxin receptor 1 OS=Homo sapiens GN=ANTXR1 PE=1 SV=2 - [ANTR1_HUMAN]	-0.3
P49356	Protein farnesyltransferase subunit beta OS=Homo sapiens GN=FNTB PE=1 SV=1 - [FNTB_HUMAN]	-0.3
Q7L5N1	COP9 signalosome complex subunit 6 OS=Homo sapiens GN=COPS6 PE=1 SV=1 - [CSN6_HUMAN]	-0.3
P15880	40S ribosomal protein S2 OS=Homo sapiens GN=RPS2 PE=1 SV=2 - [RS2_HUMAN]	-0.3
P24387	Corticotropin-releasing factor-binding protein OS=Homo sapiens GN=CRHBP PE=1 SV=2 - [CRHBP_HUMAN]	-0.3
P12268	Inosine-5'-monophosphate dehydrogenase 2 OS=Homo sapiens GN=IMPDH2 PE=1 SV=2 - [IMDH2_HUMAN]	-0.3
O95373	Importin-7 OS=Homo sapiens GN=IPO7 PE=1 SV=1 - [IPO7_HUMAN]	-0.3
Q6FI81	Anamorsin OS=Homo sapiens GN=CIAPIN1 PE=1 SV=2 - [CPIN1_HUMAN]	-0.3
P51970	NADH dehydrogenase [ubiquinone] 1 alpha subcomplex subunit 8 OS=Homo sapiens GN=NDUFA8 PE=1 SV=3 - [NDUA8_HUMAN]	-0.3
O60762	Dolichol-phosphate mannosyltransferase subunit 1 OS=Homo sapiens GN=DPM1 PE=1 SV=1 - [DPM1_HUMAN]	-0.3
Q9UGJ1	Gamma-tubulin complex component 4 OS=Homo sapiens GN=TUBGCP4 PE=1 SV=1 - [GCP4_HUMAN]	-0.3
P20020	Plasma membrane calcium-transporting ATPase 1 OS=Homo sapiens GN=ATP2B1 PE=1 SV=3 - [AT2B1_HUMAN]	-0.3
Q14315	Filamin-C OS=Homo sapiens GN=FLNC PE=1 SV=3 - [FLNC_HUMAN]	-0.3
Q8WWI1	LIM domain only protein 7 OS=Homo sapiens GN=LMO7 PE=1 SV=3 - [LMO7_HUMAN]	-0.3
P61221	ATP-binding cassette sub-family E member 1 OS=Homo sapiens GN=ABCE1 PE=1 SV=1 - [ABCE1_HUMAN]	-0.3
P06748	Nucleophosmin OS=Homo sapiens GN=NPM1 PE=1 SV=2 - [NPM_HUMAN]	-0.3
P04040	Catalase OS=Homo sapiens GN=CAT PE=1 SV=3 - [CATA_HUMAN]	-0.3
Q9ULC3	Ras-related protein Rab-23 OS=Homo sapiens GN=RAB23 PE=1 SV=1 - [RAB23_HUMAN]	-0.3
Q68E01	Integrator complex subunit 3 OS=Homo sapiens GN=INTS3 PE=1 SV=1 - [INT3_HUMAN]	-0.3
Q10469	Alpha-1,6-mannosyl-glycoprotein 2-beta-N-acetylglucosaminyltransferase OS=Homo sapiens GN=MGAT2 PE=1 SV=1 - [MGAT2_HUMAN]	-0.3
Q2M1P5	Kinesin-like protein KIF7 OS=Homo sapiens GN=KIF7 PE=1 SV=2 - [KIF7_HUMAN]	-0.3
O43264	Centromere/kinetochore protein zw10 homolog OS=Homo sapiens GN=ZW10 PE=1 SV=3 - [ZW10_HUMAN]	-0.3
O75251	NADH dehydrogenase [ubiquinone] iron-sulfur protein 7, mitochondrial OS=Homo sapiens GN=NDUFS7 PE=1 SV=3 - [NDUS7_HUMAN]	-0.3
Q9H3F6	BTB/POZ domain-containing adapter for CUL3-mediated RhoA degradation protein 3 OS=Homo sapiens GN=KCTD10 PE=1 SV=1 - [BACD3_HUMAN]	-0.3
Q9UBK9	Protein UXT OS=Homo sapiens GN=UXT PE=1 SV=1 - [UXT_HUMAN]	-0.3
Q7Z2Z1	Treslin OS=Homo sapiens GN=TICRR PE=1 SV=2 - [TICRR_HUMAN]	-0.3
P17812	CTP synthase 1 OS=Homo sapiens GN=CTPS1 PE=1 SV=2 - [PYRG1_HUMAN]	-0.3
Q14679	Tubulin polyglutamylase TTLL4 OS=Homo sapiens GN=TTLL4 PE=1 SV=2 - [TTLL4_HUMAN]	-0.3
P32321	Deoxycytidylate deaminase OS=Homo sapiens GN=DCTD PE=1 SV=2 - [DCTD_HUMAN]	-0.3
P09493	Tropomyosin alpha-1 chain OS=Homo sapiens GN=TPM1 PE=1 SV=2 - [TPM1_HUMAN]	-0.3
O95218	Zinc finger Ran-binding domain-containing protein 2 OS=Homo sapiens GN=ZRANB2 PE=1 SV=2 - [ZRAB2_HUMAN]	-0.3
P40189	Interleukin-6 receptor subunit beta OS=Homo sapiens GN=IL6ST PE=1 SV=2 - [IL6RB_HUMAN]	-0.3
P53396	ATP-citrate synthase OS=Homo sapiens GN=ACLY PE=1 SV=3 - [ACLY_HUMAN]	-0.3
P17405	Sphingomyelin phosphodiesterase OS=Homo sapiens GN=SMPD1 PE=1 SV=4 - [ASM_HUMAN]	-0.3
P00813	Adenosine deaminase OS=Homo sapiens GN=ADA PE=1 SV=3 - [ADA_HUMAN]	-0.3
O14908	PDZ domain-containing protein GIPC1 OS=Homo sapiens GN=GIPC1 PE=1 SV=2 - [GIPC1_HUMAN]	-0.3

Q03426	Mevalonate kinase OS=Homo sapiens GN=MVK PE=1 SV=1 - [KIME_HUMAN]	-0.3
Q96AC1	Fermitin family homolog 2 OS=Homo sapiens GN=FERMT2 PE=1 SV=1 - [FERM2_HUMAN]	-0.3
P53701	Cytochrome c-type heme lyase OS=Homo sapiens GN=HCCS PE=1 SV=1 - [CCHL_HUMAN]	-0.3
O94822	E3 ubiquitin-protein ligase listerin OS=Homo sapiens GN=LTN1 PE=1 SV=6 - [LTN1_HUMAN]	-0.3
Q969G6	Riboflavin kinase OS=Homo sapiens GN=RFK PE=1 SV=2 - [RIFK_HUMAN]	-0.3
O60841	Eukaryotic translation initiation factor 5B OS=Homo sapiens GN=EIF5B PE=1 SV=4 - [IF2P_HUMAN]	-0.3
P10620	Microsomal glutathione S-transferase 1 OS=Homo sapiens GN=MGST1 PE=1 SV=1 - [MGST1_HUMAN]	-0.3
Q8TED1	Probable glutathione peroxidase 8 OS=Homo sapiens GN=GPX8 PE=1 SV=2 - [GPX8_HUMAN]	-0.3
P20337	Ras-related protein Rab-3B OS=Homo sapiens GN=RAB3B PE=1 SV=2 - [RAB3B_HUMAN]	-0.3
P59998	Actin-related protein 2/3 complex subunit 4 OS=Homo sapiens GN=ARPC4 PE=1 SV=3 - [ARPC4_HUMAN]	-0.3
Q6ZVM7	TOM1-like protein 2 OS=Homo sapiens GN=TOM1L2 PE=1 SV=1 - [TM1L2_HUMAN]	-0.3
P17252	Protein kinase C alpha type OS=Homo sapiens GN=PRKCA PE=1 SV=4 - [KPCA_HUMAN]	-0.3
P12074	Cytochrome c oxidase subunit 6A1, mitochondrial OS=Homo sapiens GN=COX6A1 PE=1 SV=4 - [CX6A1_HUMAN]	-0.3
Q5VIR6	Vacuolar protein sorting-associated protein 53 homolog OS=Homo sapiens GN=VPS53 PE=1 SV=1 - [VPS53_HUMAN]	-0.3
P15291	Beta-1,4-galactosyltransferase 1 OS=Homo sapiens GN=B4GALT1 PE=1 SV=5 - [B4GT1_HUMAN]	-0.3
P20042	Eukaryotic translation initiation factor 2 subunit 2 OS=Homo sapiens GN=EIF2S2 PE=1 SV=2 - [IF2B_HUMAN]	-0.3
Q9UPY8	Microtubule-associated protein RP/EB family member 3 OS=Homo sapiens GN=MAPRE3 PE=1 SV=1 - [MARE3_HUMAN]	-0.3
Q96GP6	Scavenger receptor class F member 2 OS=Homo sapiens GN=SCARF2 PE=1 SV=4 - [SREC2_HUMAN]	-0.3
O75044	SLIT-ROBO Rho GTPase-activating protein 2 OS=Homo sapiens GN=SRGAP2 PE=1 SV=2 - [SRGP2_HUMAN]	-0.3
Q6DD88	Atlastin-3 OS=Homo sapiens GN=ATL3 PE=1 SV=1 - [ATLA3_HUMAN]	-0.3
Q86XA9	HEAT repeat-containing protein 5A OS=Homo sapiens GN=HEATR5A PE=1 SV=2 - [HTR5A_HUMAN]	-0.3
P28072	Proteasome subunit beta type-6 OS=Homo sapiens GN=PSMB6 PE=1 SV=4 - [PSB6_HUMAN]	-0.3
Q9GZM8	Nuclear distribution protein nudE-like 1 OS=Homo sapiens GN=NDEL1 PE=1 SV=1 - [NDEL1_HUMAN]	-0.3
Q9H792	Pseudopodium-enriched atypical kinase 1 OS=Homo sapiens GN=PEAK1 PE=1 SV=4 - [PEAK1_HUMAN]	-0.3
P84157	Matrix-remodeling-associated protein 7 OS=Homo sapiens GN=MXRA7 PE=1 SV=1 - [MXRA7_HUMAN]	-0.3
Q13642	Four and a half LIM domains protein 1 OS=Homo sapiens GN=FHL1 PE=1 SV=4 - [FHL1_HUMAN]	-0.3
Q9NUY8	TBC1 domain family member 23 OS=Homo sapiens GN=TBC1D23 PE=1 SV=3 - [TBC23_HUMAN]	-0.3
O60547	GDP-mannose 4,6 dehydratase OS=Homo sapiens GN=GMDS PE=1 SV=1 - [GMDS_HUMAN]	-0.3
Q9NY27	Serine/threonine-protein phosphatase 4 regulatory subunit 2 OS=Homo sapiens GN=PPP4R2 PE=1 SV=3 - [PP4R2_HUMAN]	-0.3
Q9Y2Y0	ADP-ribosylation factor-like protein 2-binding protein OS=Homo sapiens GN=ARL2BP PE=1 SV=1 - [AR2BP_HUMAN]	-0.3
Q8WXH0	Nesprin-2 OS=Homo sapiens GN=SYNE2 PE=1 SV=3 - [SYNE2_HUMAN]	-0.3
Q9NRY5	Protein FAM114A2 OS=Homo sapiens GN=FAM114A2 PE=1 SV=4 - [F1142_HUMAN]	-0.3
Q9GZZ1	N-alpha-acetyltransferase 50 OS=Homo sapiens GN=NAA50 PE=1 SV=1 - [NAA50_HUMAN]	-0.3
P51571	Translocon-associated protein subunit delta OS=Homo sapiens GN=SSR4 PE=1 SV=1 - [SSRD_HUMAN]	-0.3
Q8NBN3	Transmembrane protein 87A OS=Homo sapiens GN=TMEM87A PE=1 SV=3 - [TM87A_HUMAN]	-0.3
Q9NVZ3	Adaptin ear-binding coat-associated protein 2 OS=Homo sapiens GN=NECAP2 PE=1 SV=1 - [NECP2_HUMAN]	-0.3
Q01850	Cerebellar degeneration-related protein 2 OS=Homo sapiens GN=CDR2 PE=1 SV=2 - [CDR2_HUMAN]	-0.3
Q96K49	Transmembrane protein 87B OS=Homo sapiens GN=TMEM87B PE=1 SV=1 - [TM87B_HUMAN]	-0.3
P35573	Glycogen debranching enzyme OS=Homo sapiens GN=AGL PE=1 SV=3 - [GDE_HUMAN]	-0.3
P15848	Arylsulfatase B OS=Homo sapiens GN=ARSB PE=1 SV=1 - [ARSB_HUMAN]	-0.3
O00469	Procollagen-lysine,2-oxoglutarate 5-dioxygenase 2 OS=Homo sapiens GN=PLOD2 PE=1 SV=2 - [PLOD2_HUMAN]	-0.3
Q96GA7	Serine dehydratase-like OS=Homo sapiens GN=SDSL PE=1 SV=1 - [SDSL_HUMAN]	-0.3
Q8N8A6	ATP-dependent RNA helicase DDX51 OS=Homo sapiens GN=DDX51 PE=1 SV=3 - [DDX51_HUMAN]	-0.3
Q8N556	Actin filament-associated protein 1 OS=Homo sapiens GN=AFAP1 PE=1 SV=2 - [AFAP1_HUMAN]	-0.3
O14579	Coatomer subunit epsilon OS=Homo sapiens GN=COPE PE=1 SV=3 - [COPE_HUMAN]	-0.3
Q9BQG0	Myb-binding protein 1A OS=Homo sapiens GN=MYBBP1A PE=1 SV=2 - [MBB1A_HUMAN]	-0.3
Q9NYH9	U3 small nucleolar RNA-associated protein 6 homolog OS=Homo sapiens GN=UTP6 PE=1 SV=2 - [UTP6_HUMAN]	-0.3
Q16832	Discoidin domain-containing receptor 2 OS=Homo sapiens GN=DDR2 PE=1 SV=2 - [DDR2_HUMAN]	-0.3
Q9P299	Coatomer subunit zeta-2 OS=Homo sapiens GN=COPZ2 PE=2 SV=1 - [COPZ2_HUMAN]	-0.3
O60828	Polyglutamine-binding protein 1 OS=Homo sapiens GN=PQBP1 PE=1 SV=1 - [PQBP1_HUMAN]	-0.3
Q9UBM7	7-dehydrocholesterol reductase OS=Homo sapiens GN=DHCR7 PE=1 SV=1 - [DHCR7_HUMAN]	-0.3
Q9H173	Nucleotide exchange factor SIL1 OS=Homo sapiens GN=SIL1 PE=1 SV=1 - [SIL1_HUMAN]	-0.3
P56192	Methionine--tRNA ligase, cytoplasmic OS=Homo sapiens GN=MARS PE=1 SV=2 - [SYMC_HUMAN]	-0.3

Q8TF40	Folliculin-interacting protein 1 OS=Homo sapiens GN=FNIP1 PE=1 SV=3 - [FNIP1_HUMAN]	-0.3
Q9NZW5	MAGUK p55 subfamily member 6 OS=Homo sapiens GN=MPP6 PE=1 SV=2 - [MPP6_HUMAN]	-0.3
Q9P2J5	Leucine--tRNA ligase, cytoplasmic OS=Homo sapiens GN=LARS PE=1 SV=2 - [SYLC_HUMAN]	-0.3
P09960	Leukotriene A-4 hydrolase OS=Homo sapiens GN=LTA4H PE=1 SV=2 - [LKHA4_HUMAN]	-0.3
Q86Y82	Syntaxin-12 OS=Homo sapiens GN=STX12 PE=1 SV=1 - [STX12_HUMAN]	-0.3
Q86TX2	Acyl-coenzyme A thioesterase 1 OS=Homo sapiens GN=ACOT1 PE=1 SV=1 - [ACOT1_HUMAN]	-0.3
Q9Y385	Ubiquitin-conjugating enzyme E2 J1 OS=Homo sapiens GN=UBE2J1 PE=1 SV=2 - [UB2J1_HUMAN]	-0.3
P41743	Protein kinase C iota type OS=Homo sapiens GN=PRKCI PE=1 SV=2 - [KPCI_HUMAN]	-0.3
P49821	NADH dehydrogenase [ubiquinone] flavoprotein 1, mitochondrial OS=Homo sapiens GN=NDUFV1 PE=1 SV=4 - [NDUV1_HUMAN]	-0.3
Q02952	A-kinase anchor protein 12 OS=Homo sapiens GN=AKAP12 PE=1 SV=4 - [AKA12_HUMAN]	-0.3
Q9NZI7	Upstream-binding protein 1 OS=Homo sapiens GN=UBP1 PE=2 SV=1 - [UBIP1_HUMAN]	-0.3
P35237	Serpin B6 OS=Homo sapiens GN=SERPINB6 PE=1 SV=3 - [SPB6_HUMAN]	-0.3
P26639	Threonine--tRNA ligase, cytoplasmic OS=Homo sapiens GN=TARS PE=1 SV=3 - [SYTC_HUMAN]	-0.3
P11362	Fibroblast growth factor receptor 1 OS=Homo sapiens GN=FGFR1 PE=1 SV=3 - [FGFR1_HUMAN]	-0.3
O43665	Regulator of G-protein signaling 10 OS=Homo sapiens GN=RGS10 PE=1 SV=2 - [RGS10_HUMAN]	-0.3
O00291	Huntingtin-interacting protein 1 OS=Homo sapiens GN=HIP1 PE=1 SV=5 - [HIP1_HUMAN]	-0.3
Q9BRK5	45 kDa calcium-binding protein OS=Homo sapiens GN=SDF4 PE=1 SV=1 - [CAB45_HUMAN]	-0.3
Q8TES7	Fas-binding factor 1 OS=Homo sapiens GN=FBF1 PE=1 SV=2 - [FBF1_HUMAN]	-0.3
P55010	Eukaryotic translation initiation factor 5 OS=Homo sapiens GN=EIF5 PE=1 SV=2 - [IF5_HUMAN]	-0.3
Q13492	Phosphatidylinositol-binding clathrin assembly protein OS=Homo sapiens GN=PICALM PE=1 SV=2 - [PICAL_HUMAN]	-0.3
Q00341	Vigilin OS=Homo sapiens GN=HDLBP PE=1 SV=2 - [VIGLN_HUMAN]	-0.3
Q9H6S1	5-azacytidine-induced protein 2 OS=Homo sapiens GN=AZI2 PE=1 SV=1 - [AZI2_HUMAN]	-0.3
Q8TD43	Transient receptor potential cation channel subfamily M member 4 OS=Homo sapiens GN=TRPM4 PE=1 SV=1 - [TRPM4_HUMAN]	-0.3
Q96T76	MMS19 nucleotide excision repair protein homolog OS=Homo sapiens GN=MMS19 PE=1 SV=2 - [MMS19_HUMAN]	-0.3
O14907	Tax1-binding protein 3 OS=Homo sapiens GN=TAX1BP3 PE=1 SV=2 - [TX1B3_HUMAN]	-0.3
P63241	Eukaryotic translation initiation factor 5A-1 OS=Homo sapiens GN=EIF5A PE=1 SV=2 - [IF5A1_HUMAN]	-0.3
P53618	Coatomer subunit beta OS=Homo sapiens GN=COPB1 PE=1 SV=3 - [COPB_HUMAN]	-0.3
P22694	cAMP-dependent protein kinase catalytic subunit beta OS=Homo sapiens GN=PRKACB PE=1 SV=2 - [KAPCB_HUMAN]	-0.3
Q15311	RalA-binding protein 1 OS=Homo sapiens GN=RALBP1 PE=1 SV=3 - [RBP1_HUMAN]	-0.3
O15320	cTAGE family member 5 OS=Homo sapiens GN=CTAGE5 PE=1 SV=4 - [CTGE5_HUMAN]	-0.3
Q9UH62	Armadillo repeat-containing X-linked protein 3 OS=Homo sapiens GN=ARMCX3 PE=1 SV=1 - [ARMX3_HUMAN]	-0.3
Q9NUQ6	SPATS2-like protein OS=Homo sapiens GN=SPATS2L PE=1 SV=2 - [SPS2L_HUMAN]	-0.3
Q13867	Bleomycin hydrolase OS=Homo sapiens GN=BLMH PE=1 SV=1 - [BLMH_HUMAN]	-0.3
P11279	Lysosome-associated membrane glycoprotein 1 OS=Homo sapiens GN=LAMP1 PE=1 SV=3 - [LAMP1_HUMAN]	-0.3
Q16363	Laminin subunit alpha-4 OS=Homo sapiens GN=LAMA4 PE=1 SV=4 - [LAMA4_HUMAN]	-0.3
P62861	40S ribosomal protein S30 OS=Homo sapiens GN=FAU PE=1 SV=1 - [RS30_HUMAN]	-0.3
P27986	Phosphatidylinositol 3-kinase regulatory subunit alpha OS=Homo sapiens GN=PIK3R1 PE=1 SV=2 - [P85A_HUMAN]	-0.3
Q15386	Ubiquitin-protein ligase E3C OS=Homo sapiens GN=UBE3C PE=1 SV=3 - [UBE3C_HUMAN]	-0.3
Q96JB1	Dynein heavy chain 8, axonemal OS=Homo sapiens GN=DNAH8 PE=1 SV=2 - [DYH8_HUMAN]	-0.3
Q8N999	Uncharacterized protein C12orf29 OS=Homo sapiens GN=C12orf29 PE=1 SV=2 - [CL029_HUMAN]	-0.3
P39023	60S ribosomal protein L3 OS=Homo sapiens GN=RPL3 PE=1 SV=2 - [RL3_HUMAN]	-0.3
Q9Y3B8	Oligoribonuclease, mitochondrial OS=Homo sapiens GN=REXO2 PE=1 SV=3 - [ORN_HUMAN]	-0.3
Q9H4I3	TraB domain-containing protein OS=Homo sapiens GN=TRABD PE=1 SV=1 - [TRABD_HUMAN]	-0.3
P40616	ADP-ribosylation factor-like protein 1 OS=Homo sapiens GN=ARL1 PE=1 SV=1 - [ARL1_HUMAN]	-0.3
O43414	ERI1 exoribonuclease 3 OS=Homo sapiens GN=ERI3 PE=1 SV=2 - [ERI3_HUMAN]	-0.3
Q15181	Inorganic pyrophosphatase OS=Homo sapiens GN=PPA1 PE=1 SV=2 - [IPYR_HUMAN]	-0.3
Q99720	Sigma non-opioid intracellular receptor 1 OS=Homo sapiens GN=SIGMAR1 PE=1 SV=1 - [SGMR1_HUMAN]	-0.3
Q9Y673	Dolichyl-phosphate beta-glucosyltransferase OS=Homo sapiens GN=ALG5 PE=1 SV=1 - [ALG5_HUMAN]	-0.3
Q92990	Glomulin OS=Homo sapiens GN=GLMN PE=1 SV=2 - [GLMN_HUMAN]	-0.3
Q13308	Inactive tyrosine-protein kinase 7 OS=Homo sapiens GN=PTK7 PE=1 SV=2 - [PTK7_HUMAN]	-0.3
Q9H074	Polyadenylate-binding protein-interacting protein 1 OS=Homo sapiens GN=PAIP1 PE=1 SV=1 - [PAIP1_HUMAN]	-0.3
Q8IUF8	Bifunctional lysine-specific demethylase and histidyl-hydroxylase MINA OS=Homo sapiens GN=MINA PE=1 SV=1 - [MINA_HUMAN]	-0.3

Q14643	Inositol 1,4,5-trisphosphate receptor type 1 OS=Homo sapiens GN=ITPR1 PE=1 SV=3 - [ITPR1_HUMAN]	-0.3
Q9NX47	E3 ubiquitin-protein ligase MARCH5 OS=Homo sapiens GN=MARCH5 PE=1 SV=1 - [MARH5_HUMAN]	-0.3
Q9Y3E5	Peptidyl-tRNA hydrolase 2, mitochondrial OS=Homo sapiens GN=PTRH2 PE=1 SV=1 - [PTH2_HUMAN]	-0.3
Q14847	LIM and SH3 domain protein 1 OS=Homo sapiens GN=LASP1 PE=1 SV=2 - [LASP1_HUMAN]	-0.3
Q15005	Signal peptidase complex subunit 2 OS=Homo sapiens GN=SPCS2 PE=1 SV=3 - [SPCS2_HUMAN]	-0.3
P00966	Argininosuccinate synthase OS=Homo sapiens GN=ASS1 PE=1 SV=2 - [ASSY_HUMAN]	-0.3
Q9H2J4	Phosducin-like protein 3 OS=Homo sapiens GN=PDCL3 PE=1 SV=1 - [PDCL3_HUMAN]	-0.3
P62495	Eukaryotic peptide chain release factor subunit 1 OS=Homo sapiens GN=ETF1 PE=1 SV=3 - [ERF1_HUMAN]	-0.3
Q13618	Cullin-3 OS=Homo sapiens GN=CUL3 PE=1 SV=2 - [CUL3_HUMAN]	-0.3
P0C0L4	Complement C4-A OS=Homo sapiens GN=C4A PE=1 SV=2 - [CO4A_HUMAN]	-0.3
P46734	Dual specificity mitogen-activated protein kinase kinase 3 OS=Homo sapiens GN=MAP2K3 PE=1 SV=2 - [MP2K3_HUMAN]	-0.3
Q9Y2J4	Angiomotin-like protein 2 OS=Homo sapiens GN=AMOTL2 PE=1 SV=3 - [AMOL2_HUMAN]	-0.3
Q9UNF1	Melanoma-associated antigen D2 OS=Homo sapiens GN=MAGED2 PE=1 SV=2 - [MAGD2_HUMAN]	-0.3
Q3YEC7	Rab-like protein 6 OS=Homo sapiens GN=RABL6 PE=1 SV=2 - [RABL6_HUMAN]	-0.3
Q9P2D0	Inhibitor of Bruton tyrosine kinase OS=Homo sapiens GN=IBTK PE=1 SV=3 - [IBTK_HUMAN]	-0.3
Q7L5N7	Lysophosphatidylcholine acyltransferase 2 OS=Homo sapiens GN=LPCAT2 PE=1 SV=1 - [PCAT2_HUMAN]	-0.3
Q92696	Geranylgeranyl transferase type-2 subunit alpha OS=Homo sapiens GN=RABGGTA PE=1 SV=2 - [PGTA_HUMAN]	-0.3
P54886	Delta-1-pyrroline-5-carboxylate synthase OS=Homo sapiens GN=ALDH18A1 PE=1 SV=2 - [P5CS_HUMAN]	-0.3
Q99424	Peroxisomal acyl-coenzyme A oxidase 2 OS=Homo sapiens GN=ACOX2 PE=1 SV=1 - [ACOX2_HUMAN]	-0.3
O43169	Cytochrome b5 type B OS=Homo sapiens GN=CYB5B PE=1 SV=2 - [CYB5B_HUMAN]	-0.3
P60981	Destrin OS=Homo sapiens GN=DSTN PE=1 SV=3 - [DEST_HUMAN]	-0.3
Q9H3K2	Growth hormone-inducible transmembrane protein OS=Homo sapiens GN=GHITM PE=1 SV=2 - [GHITM_HUMAN]	-0.3
P10619	Lysosomal protective protein OS=Homo sapiens GN=CTSA PE=1 SV=2 - [PPGB_HUMAN]	-0.3
Q9NRN7	L-aminoadipate-semialdehyde dehydrogenase-phosphopantetheinyl transferase OS=Homo sapiens GN=AASDHPPT PE=1 SV=2 - [ADPPT_HUMAN]	-0.3
Q15738	Sterol-4-alpha-carboxylate 3-dehydrogenase, decarboxylating OS=Homo sapiens GN=NSDHL PE=1 SV=2 - [NSDHL_HUMAN]	-0.3
Q9GZM5	Protein YIPF3 OS=Homo sapiens GN=YIPF3 PE=1 SV=1 - [YIPF3_HUMAN]	-0.3
P53621	Coatomer subunit alpha OS=Homo sapiens GN=COPA PE=1 SV=2 - [COPA_HUMAN]	-0.3
Q8IZ52	Chondroitin sulfate synthase 2 OS=Homo sapiens GN=CHPF PE=1 SV=2 - [CHSS2_HUMAN]	-0.3
Q9HCU5	Prolactin regulatory element-binding protein OS=Homo sapiens GN=PREB PE=1 SV=2 - [PREB_HUMAN]	-0.3
O43678	NADH dehydrogenase [ubiquinone] 1 alpha subcomplex subunit 2 OS=Homo sapiens GN=NDUFA2 PE=1 SV=3 - [NDUA2_HUMAN]	-0.3
P13797	Plastin-3 OS=Homo sapiens GN=PLS3 PE=1 SV=4 - [PLST_HUMAN]	-0.3
P20338	Ras-related protein Rab-4A OS=Homo sapiens GN=RAB4A PE=1 SV=3 - [RAB4A_HUMAN]	-0.3
Q15293	Reticulocalbin-1 OS=Homo sapiens GN=RCN1 PE=1 SV=1 - [RCN1_HUMAN]	-0.3
Q92905	COP9 signalosome complex subunit 5 OS=Homo sapiens GN=COPS5 PE=1 SV=4 - [CSN5_HUMAN]	-0.3
Q5VZK9	Leucine-rich repeat-containing protein 16A OS=Homo sapiens GN=LRRC16A PE=1 SV=1 - [LR16A_HUMAN]	-0.3
Q6PKG0	La-related protein 1 OS=Homo sapiens GN=LARP1 PE=1 SV=2 - [LARP1_HUMAN]	-0.3
O75911	Short-chain dehydrogenase/reductase 3 OS=Homo sapiens GN=DHRS3 PE=1 SV=2 - [DHRS3_HUMAN]	-0.3
Q8IUR0	Trafficking protein particle complex subunit 5 OS=Homo sapiens GN=TRAPPC5 PE=1 SV=1 - [TPPC5_HUMAN]	-0.3
O95081	Arf-GAP domain and FG repeat-containing protein 2 OS=Homo sapiens GN=AGFG2 PE=1 SV=2 - [AGFG2_HUMAN]	-0.3
Q7LGA3	Heparan sulfate 2-O-sulfotransferase 1 OS=Homo sapiens GN=HS2ST1 PE=1 SV=1 - [HS2ST_HUMAN]	-0.3
P61421	V-type proton ATPase subunit d 1 OS=Homo sapiens GN=ATP6V0D1 PE=1 SV=1 - [VA0D1_HUMAN]	-0.3
Q4G0N4	NAD kinase 2, mitochondrial OS=Homo sapiens GN=NADK2 PE=1 SV=2 - [NAKD2_HUMAN]	-0.3
O95302	Peptidyl-prolyl cis-trans isomerase FKBP9 OS=Homo sapiens GN=FKBP9 PE=1 SV=2 - [FKBP9_HUMAN]	-0.3
P13489	Ribonuclease inhibitor OS=Homo sapiens GN=RNH1 PE=1 SV=2 - [RINI_HUMAN]	-0.3
Q9P035	Very-long-chain (3R)-3-hydroxyacyl-CoA dehydratase 3 OS=Homo sapiens GN=PTPLAD1 PE=1 SV=2 - [HACD3_HUMAN]	-0.3
P14406	Cytochrome c oxidase subunit 7A2, mitochondrial OS=Homo sapiens GN=COX7A2 PE=1 SV=1 - [CX7A2_HUMAN]	-0.3
Q4G148	Glucoside xylosyltransferase 1 OS=Homo sapiens GN=GXYLT1 PE=1 SV=2 - [GXLT1_HUMAN]	-0.3
O43324	Eukaryotic translation elongation factor 1 epsilon-1 OS=Homo sapiens GN=EEF1E1 PE=1 SV=1 - [MCA3_HUMAN]	-0.3
Q92604	Acyl-CoA:lysophosphatidylglycerol acyltransferase 1 OS=Homo sapiens GN=LPGAT1 PE=2 SV=1 - [LGAT1_HUMAN]	-0.3
Q5SWX8	Protein odr-4 homolog OS=Homo sapiens GN=ODR4 PE=2 SV=1 - [ODR4_HUMAN]	-0.3
P19634	Sodium/hydrogen exchanger 1 OS=Homo sapiens GN=SLC9A1 PE=1 SV=2 - [SL9A1_HUMAN]	-0.3
P11047	Laminin subunit gamma-1 OS=Homo sapiens GN=LAMC1 PE=1 SV=3 - [LAMC1_HUMAN]	-0.3

O43920	NADH dehydrogenase [ubiquinone] iron-sulfur protein 5 OS=Homo sapiens GN=NDUFS5 PE=1 SV=3 - [NDUS5_HUMAN]	-0.3
Q9NRW7	Vacuolar protein sorting-associated protein 45 OS=Homo sapiens GN=VPS45 PE=1 SV=1 - [VPS45_HUMAN]	-0.3
P01033	Metalloproteinase inhibitor 1 OS=Homo sapiens GN=TIMP1 PE=1 SV=1 - [TIMP1_HUMAN]	-0.3
Q9H8Y8	Golgi reassembly-stacking protein 2 OS=Homo sapiens GN=GORASP2 PE=1 SV=3 - [GORS2_HUMAN]	-0.3
Q99436	Proteasome subunit beta type-7 OS=Homo sapiens GN=PSMB7 PE=1 SV=1 - [PSB7_HUMAN]	-0.3
Q9Y5Y5	Peroxisomal membrane protein PEX16 OS=Homo sapiens GN=PEX16 PE=1 SV=2 - [PEX16_HUMAN]	-0.3
O95167	NADH dehydrogenase [ubiquinone] 1 alpha subcomplex subunit 3 OS=Homo sapiens GN=NDUFA3 PE=1 SV=1 - [NDUA3_HUMAN]	-0.3
Q8TDD1	ATP-dependent RNA helicase DDX54 OS=Homo sapiens GN=DDX54 PE=1 SV=2 - [DDX54_HUMAN]	-0.3
O75223	Gamma-glutamylcyclotransferase OS=Homo sapiens GN=GGCT PE=1 SV=1 - [GGCT_HUMAN]	-0.3
Q07890	Son of sevenless homolog 2 OS=Homo sapiens GN=SOS2 PE=1 SV=2 - [SOS2_HUMAN]	-0.3
O94854	Uncharacterized protein KIAA0754 OS=Homo sapiens GN=KIAA0754 PE=1 SV=4 - [K0754_HUMAN]	-0.3
Q9BYG8	Gasdermin-C OS=Homo sapiens GN=GSDMC PE=2 SV=3 - [GSDMC_HUMAN]	-0.3
Q6ZSS7	Major facilitator superfamily domain-containing protein 6 OS=Homo sapiens GN=MFSD6 PE=1 SV=2 - [MFSD6_HUMAN]	-0.3
Q5T6V5	UPF0553 protein C9orf64 OS=Homo sapiens GN=C9orf64 PE=1 SV=1 - [CI064_HUMAN]	-0.3
Q6SZW1	Sterile alpha and TIR motif-containing protein 1 OS=Homo sapiens GN=SARM1 PE=1 SV=1 - [SARM1_HUMAN]	-0.3
Q16527	Cysteine and glycine-rich protein 2 OS=Homo sapiens GN=CSRP2 PE=1 SV=3 - [CSRP2_HUMAN]	-0.3
Q14112	Nidogen-2 OS=Homo sapiens GN=NID2 PE=1 SV=3 - [NID2_HUMAN]	-0.3
Q9NQW7	Xaa-Pro aminopeptidase 1 OS=Homo sapiens GN=XPNPEP1 PE=1 SV=3 - [XPP1_HUMAN]	-0.3
P55735	Protein SEC13 homolog OS=Homo sapiens GN=SEC13 PE=1 SV=3 - [SEC13_HUMAN]	-0.3
P61009	Signal peptidase complex subunit 3 OS=Homo sapiens GN=SPCS3 PE=1 SV=1 - [SPCS3_HUMAN]	-0.3
Q70E73	Ras-associated and pleckstrin homology domains-containing protein 1 OS=Homo sapiens GN=RAPH1 PE=1 SV=3 - [RAPH1_HUMAN]	-0.3
Q9HCU0	Endosialin OS=Homo sapiens GN=CD248 PE=1 SV=1 - [CD248_HUMAN]	-0.3
O43747	AP-1 complex subunit gamma-1 OS=Homo sapiens GN=AP1G1 PE=1 SV=5 - [AP1G1_HUMAN]	-0.3
P39656	Dolichyl-diphosphooligosaccharide--protein glycosyltransferase 48 kDa subunit OS=Homo sapiens GN=DDOST PE=1 SV=4 - [OST48_HUMAN]	-0.3
P49589	Cysteine--tRNA ligase, cytoplasmic OS=Homo sapiens GN=CARS PE=1 SV=3 - [SYCC_HUMAN]	-0.3
O94829	Importin-13 OS=Homo sapiens GN=IPO13 PE=1 SV=3 - [IPO13_HUMAN]	-0.3
Q9UHG3	Prenylcysteine oxidase 1 OS=Homo sapiens GN=PCYOX1 PE=1 SV=3 - [PCYOX_HUMAN]	-0.4
Q9UJM3	ERBB receptor feedback inhibitor 1 OS=Homo sapiens GN=ERRFI1 PE=1 SV=1 - [ERRFI_HUMAN]	-0.4
Q92747	Actin-related protein 2/3 complex subunit 1A OS=Homo sapiens GN=ARPC1A PE=1 SV=2 - [ARC1A_HUMAN]	-0.4
Q6UB35	Monofunctional C1-tetrahydrofolate synthase, mitochondrial OS=Homo sapiens GN=MTHFD1L PE=1 SV=1 - [C1TM_HUMAN]	-0.4
Q9Y5M8	Signal recognition particle receptor subunit beta OS=Homo sapiens GN=SRPRB PE=1 SV=3 - [SRPRB_HUMAN]	-0.4
O60613	15 kDa selenoprotein OS=Homo sapiens GN=SEP15 PE=1 SV=3 - [SEP15_HUMAN]	-0.4
Q9UI09	NADH dehydrogenase [ubiquinone] 1 alpha subcomplex subunit 12 OS=Homo sapiens GN=NDUFA12 PE=1 SV=1 - [NDUAC_HUMAN]	-0.4
Q9H4G0	Band 4.1-like protein 1 OS=Homo sapiens GN=EPB41L1 PE=1 SV=2 - [E41L1_HUMAN]	-0.4
Q9UJV9	Probable ATP-dependent RNA helicase DDX41 OS=Homo sapiens GN=DDX41 PE=1 SV=2 - [DDX41_HUMAN]	-0.4
Q9HD15	Steroid receptor RNA activator 1 OS=Homo sapiens GN=SRA1 PE=1 SV=1 - [SRA1_HUMAN]	-0.4
Q12979	Active breakpoint cluster region-related protein OS=Homo sapiens GN=ABR PE=2 SV=2 - [ABR_HUMAN]	-0.4
Q99707	Methionine synthase OS=Homo sapiens GN=MTR PE=1 SV=2 - [METH_HUMAN]	-0.4
Q9Y570	Protein phosphatase methylesterase 1 OS=Homo sapiens GN=PPME1 PE=1 SV=3 - [PPME1_HUMAN]	-0.4
Q14919	Dr1-associated corepressor OS=Homo sapiens GN=DRAP1 PE=1 SV=3 - [NC2A_HUMAN]	-0.4
P04264	Keratin, type II cytoskeletal 1 OS=Homo sapiens GN=KRT1 PE=1 SV=6 - [K2C1_HUMAN]	-0.4
Q8TDX7	Serine/threonine-protein kinase Nek7 OS=Homo sapiens GN=NEK7 PE=1 SV=1 - [NEK7_HUMAN]	-0.4
Q6IAN0	Dehydrogenase/reductase SDR family member 7B OS=Homo sapiens GN=DHRS7B PE=1 SV=2 - [DRS7B_HUMAN]	-0.4
Q9Y4K4	Mitogen-activated protein kinase kinase kinase kinase 5 OS=Homo sapiens GN=MAP4K5 PE=1 SV=1 - [M4K5_HUMAN]	-0.4
Q9UKD2	mRNA turnover protein 4 homolog OS=Homo sapiens GN=MRTO4 PE=1 SV=2 - [MRT4_HUMAN]	-0.4
Q9HC35	Echinoderm microtubule-associated protein-like 4 OS=Homo sapiens GN=EML4 PE=1 SV=3 - [EMAL4_HUMAN]	-0.4
Q9Y680	Peptidyl-prolyl cis-trans isomerase FKBP7 OS=Homo sapiens GN=FKBP7 PE=1 SV=1 - [FKBP7_HUMAN]	-0.4
P61086	Ubiquitin-conjugating enzyme E2 K OS=Homo sapiens GN=UBE2K PE=1 SV=3 - [UBE2K_HUMAN]	-0.4
Q9BTT0	Acidic leucine-rich nuclear phosphoprotein 32 family member E OS=Homo sapiens GN=ANP32E PE=1 SV=1 - [AN32E_HUMAN]	-0.4
P62277	40S ribosomal protein S13 OS=Homo sapiens GN=RPS13 PE=1 SV=2 - [RS13_HUMAN]	-0.4
O43402	ER membrane protein complex subunit 8 OS=Homo sapiens GN=EMC8 PE=1 SV=1 - [EMC8_HUMAN]	-0.4
P50583	Bis(5'-nucleosyl)-tetraphosphatase [asymmetrical] OS=Homo sapiens GN=NUDT2 PE=1 SV=3 - [AP4A_HUMAN]	-0.4

Q9Y4K1	Absent in melanoma 1 protein OS=Homo sapiens GN=AIM1 PE=1 SV=3 - [AIM1_HUMAN]	-0.4
P62273	40S ribosomal protein S29 OS=Homo sapiens GN=RPS29 PE=1 SV=2 - [RS29_HUMAN]	-0.4
Q4G0F5	Vacuolar protein sorting-associated protein 26B OS=Homo sapiens GN=VPS26B PE=1 SV=2 - [VP26B_HUMAN]	-0.4
Q9H4A6	Golgi phosphoprotein 3 OS=Homo sapiens GN=GOLPH3 PE=1 SV=1 - [GOLP3_HUMAN]	-0.4
Q8NDA2	Hemicentin-2 OS=Homo sapiens GN=HMCN2 PE=2 SV=2 - [HMCN2_HUMAN]	-0.4
Q13724	Mannosyl-oligosaccharide glucosidase OS=Homo sapiens GN=MOGS PE=1 SV=5 - [MOGS_HUMAN]	-0.4
Q9NYC9	Dynein heavy chain 9, axonemal OS=Homo sapiens GN=DNAH9 PE=1 SV=3 - [DYH9_HUMAN]	-0.4
Q92783	Signal transducing adapter molecule 1 OS=Homo sapiens GN=STAM PE=1 SV=3 - [STAM1_HUMAN]	-0.4
Q5SW96	Low density lipoprotein receptor adapter protein 1 OS=Homo sapiens GN=LDLRAP1 PE=1 SV=3 - [ARH_HUMAN]	-0.4
Q08431	Lactadherin OS=Homo sapiens GN=MFGE8 PE=1 SV=2 - [MFGM_HUMAN]	-0.4
P62910	60S ribosomal protein L32 OS=Homo sapiens GN=RPL32 PE=1 SV=2 - [RL32_HUMAN]	-0.4
P84085	ADP-ribosylation factor 5 OS=Homo sapiens GN=ARF5 PE=1 SV=2 - [ARF5_HUMAN]	-0.4
Q9P032	NADH dehydrogenase [ubiquinone] 1 alpha subcomplex assembly factor 4 OS=Homo sapiens GN=NDUFAF4 PE=1 SV=1 - [NDUF4_HUMAN]	-0.4
Q14165	Malectin OS=Homo sapiens GN=MLEC PE=1 SV=1 - [MLEC_HUMAN]	-0.4
P52758	Ribonuclease UK114 OS=Homo sapiens GN=HRSP12 PE=1 SV=1 - [UK114_HUMAN]	-0.4
P28074	Proteasome subunit beta type-5 OS=Homo sapiens GN=PSMB5 PE=1 SV=3 - [PSB5_HUMAN]	-0.4
Q96C36	Pyrroline-5-carboxylate reductase 2 OS=Homo sapiens GN=PYCR2 PE=1 SV=1 - [P5CR2_HUMAN]	-0.4
Q13637	Ras-related protein Rab-32 OS=Homo sapiens GN=RAB32 PE=1 SV=3 - [RAB32_HUMAN]	-0.4
Q9NQS1	Cell death regulator Aven OS=Homo sapiens GN=AVEN PE=1 SV=1 - [AVEN_HUMAN]	-0.4
P60520	Gamma-aminobutyric acid receptor-associated protein-like 2 OS=Homo sapiens GN=GABARAPL2 PE=1 SV=1 - [GBRL2_HUMAN]	-0.4
Q8N9N2	Activating signal cointegrator 1 complex subunit 1 OS=Homo sapiens GN=ASCC1 PE=1 SV=1 - [ASCC1_HUMAN]	-0.4
P54727	UV excision repair protein RAD23 homolog B OS=Homo sapiens GN=RAD23B PE=1 SV=1 - [RD23B_HUMAN]	-0.4
Q9NR45	Sialic acid synthase OS=Homo sapiens GN=NANS PE=1 SV=2 - [SIAS_HUMAN]	-0.4
P07814	Bifunctional glutamate/proline--tRNA ligase OS=Homo sapiens GN=EPRS PE=1 SV=5 - [SYEP_HUMAN]	-0.4
Q15113	Procollagen C-endopeptidase enhancer 1 OS=Homo sapiens GN=PCOLCE PE=1 SV=2 - [PCOC1_HUMAN]	-0.4
A6ZKI3	Protein FAM127A OS=Homo sapiens GN=FAM127A PE=1 SV=1 - [F127A_HUMAN]	-0.4
P61254	60S ribosomal protein L26 OS=Homo sapiens GN=RPL26 PE=1 SV=1 - [RL26_HUMAN]	-0.4
Q5T7M9	Protein FAM69A OS=Homo sapiens GN=FAM69A PE=2 SV=1 - [FA69A_HUMAN]	-0.4
O96011	Peroxisomal membrane protein 11B OS=Homo sapiens GN=PEX11B PE=1 SV=1 - [PX11B_HUMAN]	-0.4
P41091	Eukaryotic translation initiation factor 2 subunit 3 OS=Homo sapiens GN=EIF2S3 PE=1 SV=3 - [IF2G_HUMAN]	-0.4
Q9Y6A4	Cilia- and flagella-associated protein 20 OS=Homo sapiens GN=CFAP20 PE=1 SV=1 - [CFA20_HUMAN]	-0.4
Q7Z7M9	Polypeptide N-acetylgalactosaminyltransferase 5 OS=Homo sapiens GN=GALNT5 PE=1 SV=1 - [GALT5_HUMAN]	-0.4
O15344	E3 ubiquitin-protein ligase Midline-1 OS=Homo sapiens GN=MID1 PE=1 SV=1 - [TRI18_HUMAN]	-0.4
Q8IWB7	WD repeat and FYVE domain-containing protein 1 OS=Homo sapiens GN=WDFY1 PE=1 SV=1 - [WDFY1_HUMAN]	-0.4
P63244	Guanine nucleotide-binding protein subunit beta-2-like 1 OS=Homo sapiens GN=GNB2L1 PE=1 SV=3 - [GBLP_HUMAN]	-0.4
Q16401	26S proteasome non-ATPase regulatory subunit 5 OS=Homo sapiens GN=PSMD5 PE=1 SV=3 - [PSMD5_HUMAN]	-0.4
Q8NB49	Phospholipid-transporting ATPase IG OS=Homo sapiens GN=ATP11C PE=1 SV=3 - [AT11C_HUMAN]	-0.4
Q06210	Glutamine--fructose-6-phosphate aminotransferase [isomerizing] 1 OS=Homo sapiens GN=GFPT1 PE=1 SV=3 - [GFPT1_HUMAN]	-0.4
Q92896	Golgi apparatus protein 1 OS=Homo sapiens GN=GLG1 PE=1 SV=2 - [GSLG1_HUMAN]	-0.4
Q9Y6M7	Sodium bicarbonate cotransporter 3 OS=Homo sapiens GN=SLC4A7 PE=1 SV=2 - [S4A7_HUMAN]	-0.4
P10398	Serine/threonine-protein kinase A-Raf OS=Homo sapiens GN=ARAF PE=1 SV=2 - [ARAF_HUMAN]	-0.4
P52294	Importin subunit alpha-5 OS=Homo sapiens GN=KPNA1 PE=1 SV=3 - [IMA5_HUMAN]	-0.4
O00743	Serine/threonine-protein phosphatase 6 catalytic subunit OS=Homo sapiens GN=PPP6C PE=1 SV=1 - [PPP6_HUMAN]	-0.4
Q9NVA2	Septin-11 OS=Homo sapiens GN=SEPT11 PE=1 SV=3 - [SEP11_HUMAN]	-0.4
P02511	Alpha-crystallin B chain OS=Homo sapiens GN=CRYAB PE=1 SV=2 - [CRYAB_HUMAN]	-0.4
Q9H425	Uncharacterized protein C1orf198 OS=Homo sapiens GN=C1orf198 PE=1 SV=1 - [CA198_HUMAN]	-0.4
Q16706	Alpha-mannosidase 2 OS=Homo sapiens GN=MAN2A1 PE=1 SV=2 - [MA2A1_HUMAN]	-0.4
P00519	Tyrosine-protein kinase ABL1 OS=Homo sapiens GN=ABL1 PE=1 SV=4 - [ABL1_HUMAN]	-0.4
P40261	Nicotinamide N-methyltransferase OS=Homo sapiens GN=NNMT PE=1 SV=1 - [NNMT_HUMAN]	-0.4
Q9NZJ7	Mitochondrial carrier homolog 1 OS=Homo sapiens GN=MTCH1 PE=1 SV=1 - [MTCH1_HUMAN]	-0.4
Q15366	Poly(rC)-binding protein 2 OS=Homo sapiens GN=PCBP2 PE=1 SV=1 - [PCBP2_HUMAN]	-0.4
P52594	Arf-GAP domain and FG repeat-containing protein 1 OS=Homo sapiens GN=AGFG1 PE=1 SV=2 - [AGFG1_HUMAN]	-0.4

P22692	Insulin-like growth factor-binding protein 4 OS=Homo sapiens GN=IGFBP4 PE=1 SV=2 - [IBP4_HUMAN]	-0.4
Q9GZT6	Coiled-coil domain-containing protein 90B, mitochondrial OS=Homo sapiens GN=CCDC90B PE=1 SV=2 - [CC90B_HUMAN]	-0.4
Q92974	Rho guanine nucleotide exchange factor 2 OS=Homo sapiens GN=ARHGEF2 PE=1 SV=4 - [ARHG2_HUMAN]	-0.4
Q9BVP2	Guanine nucleotide-binding protein-like 3 OS=Homo sapiens GN=GNL3 PE=1 SV=2 - [GNL3_HUMAN]	-0.4
P67812	Signal peptidase complex catalytic subunit SEC11A OS=Homo sapiens GN=SEC11A PE=1 SV=1 - [SC11A_HUMAN]	-0.4
Q9Y6R1	Electrogenic sodium bicarbonate cotransporter 1 OS=Homo sapiens GN=SLC4A4 PE=1 SV=1 - [S4A4_HUMAN]	-0.4
Q8IZ07	Ankyrin repeat domain-containing protein 13A OS=Homo sapiens GN=ANKRD13A PE=1 SV=3 - [AN13A_HUMAN]	-0.4
Q9H3U5	Major facilitator superfamily domain-containing protein 1 OS=Homo sapiens GN=MFSD1 PE=2 SV=2 - [MFSD1_HUMAN]	-0.4
Q96KC8	DnaJ homolog subfamily C member 1 OS=Homo sapiens GN=DNAJC1 PE=1 SV=1 - [DNJC1_HUMAN]	-0.4
O94955	Rho-related BTB domain-containing protein 3 OS=Homo sapiens GN=RHOBTB3 PE=1 SV=2 - [RHBT3_HUMAN]	-0.4
Q92629	Delta-sarcoglycan OS=Homo sapiens GN=SGCD PE=1 SV=2 - [SGCD_HUMAN]	-0.4
Q96SL4	Glutathione peroxidase 7 OS=Homo sapiens GN=GPX7 PE=1 SV=1 - [GPX7_HUMAN]	-0.4
P22392	Nucleoside diphosphate kinase B OS=Homo sapiens GN=NME2 PE=1 SV=1 - [NDKB_HUMAN]	-0.4
Q8NE86	Calcium uniporter protein, mitochondrial OS=Homo sapiens GN=MCU PE=1 SV=1 - [MCU_HUMAN]	-0.4
Q8NCH0	Carbohydrate sulfotransferase 14 OS=Homo sapiens GN=CHST14 PE=1 SV=2 - [CHSTE_HUMAN]	-0.4
O75362	Zinc finger protein 217 OS=Homo sapiens GN=ZNF217 PE=1 SV=1 - [ZN217_HUMAN]	-0.4
Q8NHP6	Motile sperm domain-containing protein 2 OS=Homo sapiens GN=MOSPD2 PE=1 SV=1 - [MSPD2_HUMAN]	-0.4
Q9NPA8	Transcription and mRNA export factor ENY2 OS=Homo sapiens GN=ENY2 PE=1 SV=1 - [ENY2_HUMAN]	-0.4
P56199	Integrin alpha-1 OS=Homo sapiens GN=ITGA1 PE=1 SV=2 - [ITA1_HUMAN]	-0.4
Q99715	Collagen alpha-1(XII) chain OS=Homo sapiens GN=COL12A1 PE=1 SV=2 - [COCA1_HUMAN]	-0.4
O15372	Eukaryotic translation initiation factor 3 subunit H OS=Homo sapiens GN=EIF3H PE=1 SV=1 - [EIF3H_HUMAN]	-0.4
Q6P996	Pyridoxal-dependent decarboxylase domain-containing protein 1 OS=Homo sapiens GN=PDXDC1 PE=1 SV=2 - [PDXD1_HUMAN]	-0.4
P11717	Cation-independent mannose-6-phosphate receptor OS=Homo sapiens GN=IGF2R PE=1 SV=3 - [MPRI_HUMAN]	-0.4
Q9HB90	Ras-related GTP-binding protein C OS=Homo sapiens GN=RRAGC PE=1 SV=1 - [RRAGC_HUMAN]	-0.4
Q9Y2G5	GDP-fucose protein O-fucosyltransferase 2 OS=Homo sapiens GN=POFUT2 PE=1 SV=3 - [OFUT2_HUMAN]	-0.4
Q9BTY2	Plasma alpha-L-fucosidase OS=Homo sapiens GN=FUCA2 PE=1 SV=2 - [FUCO2_HUMAN]	-0.4
Q9H6S3	Epidermal growth factor receptor kinase substrate 8-like protein 2 OS=Homo sapiens GN=EPS8L2 PE=1 SV=2 - [ES8L2_HUMAN]	-0.4
Q9HD45	Transmembrane 9 superfamily member 3 OS=Homo sapiens GN=TM9SF3 PE=1 SV=2 - [TM9S3_HUMAN]	-0.4
Q9BRK3	Matrix-remodeling-associated protein 8 OS=Homo sapiens GN=MXRA8 PE=2 SV=1 - [MXRA8_HUMAN]	-0.4
Q9NS86	LanC-like protein 2 OS=Homo sapiens GN=LANCL2 PE=1 SV=1 - [LANC2_HUMAN]	-0.4
P36871	Phosphoglucomutase-1 OS=Homo sapiens GN=PGM1 PE=1 SV=3 - [PGM1_HUMAN]	-0.4
Q6UX72	UDP-GlcNAc:betaGal beta-1,3-N-acetylglucosaminyltransferase 9 OS=Homo sapiens GN=B3GNT9 PE=2 SV=1 - [B3GN9_HUMAN]	-0.4
Q5T4B2	Probable inactive glycosyltransferase 25 family member 3 OS=Homo sapiens GN=CERCAM PE=2 SV=1 - [GT253_HUMAN]	-0.4
P62879	Guanine nucleotide-binding protein G(I)/G(S)/G(T) subunit beta-2 OS=Homo sapiens GN=GNB2 PE=1 SV=3 - [GBB2_HUMAN]	-0.4
P42574	Caspase-3 OS=Homo sapiens GN=CASP3 PE=1 SV=2 - [CASP3_HUMAN]	-0.4
O43776	Asparagine--tRNA ligase, cytoplasmic OS=Homo sapiens GN=NARS PE=1 SV=1 - [SYNC_HUMAN]	-0.4
P50281	Matrix metalloproteinase-14 OS=Homo sapiens GN=MMP14 PE=1 SV=3 - [MMP14_HUMAN]	-0.4
P24043	Laminin subunit alpha-2 OS=Homo sapiens GN=LAMA2 PE=1 SV=4 - [LAMA2_HUMAN]	-0.4
Q9UMX0	Ubiquilin-1 OS=Homo sapiens GN=UBQLN1 PE=1 SV=2 - [UBQL1_HUMAN]	-0.4
O75718	Cartilage-associated protein OS=Homo sapiens GN=CRTAP PE=1 SV=1 - [CRTAP_HUMAN]	-0.4
Q5W0V3	Protein FAM160B1 OS=Homo sapiens GN=FAM160B1 PE=2 SV=1 - [F16B1_HUMAN]	-0.4
A4UGR9	Xin actin-binding repeat-containing protein 2 OS=Homo sapiens GN=XIRP2 PE=1 SV=2 - [XIRP2_HUMAN]	-0.4
Q96EQ0	Small glutamine-rich tetratricopeptide repeat-containing protein beta OS=Homo sapiens GN=SGTB PE=1 SV=1 - [SGTB_HUMAN]	-0.4
P47974	Zinc finger protein 36, C3H1 type-like 2 OS=Homo sapiens GN=ZFP36L2 PE=1 SV=3 - [TISD_HUMAN]	-0.4
Q8IVF6	Ankyrin repeat domain-containing protein 18A OS=Homo sapiens GN=ANKRD18A PE=2 SV=3 - [AN18A_HUMAN]	-0.4
Q8IYU8	Calcium uptake protein 2, mitochondrial OS=Homo sapiens GN=MICU2 PE=1 SV=2 - [MICU2_HUMAN]	-0.4
P45984	Mitogen-activated protein kinase 9 OS=Homo sapiens GN=MAPK9 PE=1 SV=2 - [MK09_HUMAN]	-0.4
Q8TEU7	Rap guanine nucleotide exchange factor 6 OS=Homo sapiens GN=RAPGEF6 PE=1 SV=2 - [RPGF6_HUMAN]	-0.4
Q9UBI6	Guanine nucleotide-binding protein G(I)/G(S)/G(O) subunit gamma-12 OS=Homo sapiens GN=GNG12 PE=1 SV=3 - [GBG12_HUMAN]	-0.4
Q8N5I4	Dehydrogenase/reductase SDR family member on chromosome X OS=Homo sapiens GN=DHRSX PE=2 SV=2 - [DHRSX_HUMAN]	-0.4
Q53TN4	Cytochrome b reductase 1 OS=Homo sapiens GN=CYBRD1 PE=1 SV=1 - [CYBR1_HUMAN]	-0.4
Q9UIV1	CCR4-NOT transcription complex subunit 7 OS=Homo sapiens GN=CNOT7 PE=1 SV=3 - [CNOT7_HUMAN]	-0.4

P49588	Alanine--tRNA ligase, cytoplasmic OS=Homo sapiens GN=AARS PE=1 SV=2 - [SYAC_HUMAN]	-0.4
Q9P0U1	Mitochondrial import receptor subunit TOM7 homolog OS=Homo sapiens GN=TOMM7 PE=1 SV=1 - [TOM7_HUMAN]	-0.4
Q969X5	Endoplasmic reticulum-Golgi intermediate compartment protein 1 OS=Homo sapiens GN=ERGIC1 PE=1 SV=1 - [ERGI1_HUMAN]	-0.4
O95864	Fatty acid desaturase 2 OS=Homo sapiens GN=FADS2 PE=1 SV=1 - [FADS2_HUMAN]	-0.4
Q9HD26	Golgi-associated PDZ and coiled-coil motif-containing protein OS=Homo sapiens GN=GOPC PE=1 SV=1 - [GOPC_HUMAN]	-0.4
P47813	Eukaryotic translation initiation factor 1A, X-chromosomal OS=Homo sapiens GN=EIF1AX PE=1 SV=2 - [IF1AX_HUMAN]	-0.4
Q58WW2	DDB1- and CUL4-associated factor 6 OS=Homo sapiens GN=DCAF6 PE=1 SV=1 - [DCAF6_HUMAN]	-0.4
Q9Y487	V-type proton ATPase 116 kDa subunit a isoform 2 OS=Homo sapiens GN=ATP6V0A2 PE=1 SV=2 - [VPP2_HUMAN]	-0.4
Q14558	Phosphoribosyl pyrophosphate synthase-associated protein 1 OS=Homo sapiens GN=PRPSAP1 PE=1 SV=2 - [KPRA_HUMAN]	-0.4
P49902	Cytosolic purine 5'-nucleotidase OS=Homo sapiens GN=NT5C2 PE=1 SV=1 - [5NTC_HUMAN]	-0.4
O15258	Protein RER1 OS=Homo sapiens GN=RER1 PE=1 SV=1 - [RER1_HUMAN]	-0.4
P62070	Ras-related protein R-Ras2 OS=Homo sapiens GN=RRAS2 PE=1 SV=1 - [RRAS2_HUMAN]	-0.4
Q5HYK7	SH3 domain-containing protein 19 OS=Homo sapiens GN=SH3D19 PE=1 SV=2 - [SH319_HUMAN]	-0.4
Q99541	Perilipin-2 OS=Homo sapiens GN=PLIN2 PE=1 SV=2 - [PLIN2_HUMAN]	-0.4
Q8TCJ2	Dolichyl-diphosphooligosaccharide--protein glycosyltransferase subunit STT3B OS=Homo sapiens GN=STT3B PE=1 SV=1 - [STT3B_HUMAN]	-0.4
Q92520	Protein FAM3C OS=Homo sapiens GN=FAM3C PE=1 SV=1 - [FAM3C_HUMAN]	-0.4
Q15052	Rho guanine nucleotide exchange factor 6 OS=Homo sapiens GN=ARHGEF6 PE=1 SV=2 - [ARHG6_HUMAN]	-0.5
P41252	Isoleucine--tRNA ligase, cytoplasmic OS=Homo sapiens GN=IARS PE=1 SV=2 - [SYIC_HUMAN]	-0.5
P02462	Collagen alpha-1(IV) chain OS=Homo sapiens GN=COL4A1 PE=1 SV=3 - [CO4A1_HUMAN]	-0.5
Q92544	Transmembrane 9 superfamily member 4 OS=Homo sapiens GN=TM9SF4 PE=1 SV=2 - [TM9S4_HUMAN]	-0.5
Q9NUM4	Transmembrane protein 106B OS=Homo sapiens GN=TMEM106B PE=1 SV=2 - [T106B_HUMAN]	-0.5
P51649	Succinate-semialdehyde dehydrogenase, mitochondrial OS=Homo sapiens GN=ALDH5A1 PE=1 SV=2 - [SSDH_HUMAN]	-0.5
Q9NXH9	tRNA (guanine(26)-N(2))-dimethyltransferase OS=Homo sapiens GN=TRMT1 PE=1 SV=1 - [TRM1_HUMAN]	-0.5
P42696	RNA-binding protein 34 OS=Homo sapiens GN=RBM34 PE=1 SV=2 - [RBM34_HUMAN]	-0.5
O94919	Endonuclease domain-containing 1 protein OS=Homo sapiens GN=ENDOD1 PE=1 SV=2 - [ENDD1_HUMAN]	-0.5
Q15121	Astrocytic phosphoprotein PEA-15 OS=Homo sapiens GN=PEA15 PE=1 SV=2 - [PEA15_HUMAN]	-0.5
O75335	Liprin-alpha-4 OS=Homo sapiens GN=PPFIA4 PE=2 SV=3 - [LIPA4_HUMAN]	-0.5
Q53GQ0	Estradiol 17-beta-dehydrogenase 12 OS=Homo sapiens GN=HSD17B12 PE=1 SV=2 - [DHB12_HUMAN]	-0.5
P49914	5-formyltetrahydrofolate cyclo-ligase OS=Homo sapiens GN=MTHFS PE=1 SV=2 - [MTHFS_HUMAN]	-0.5
Q9Y3S2	Zinc finger protein 330 OS=Homo sapiens GN=ZNF330 PE=1 SV=1 - [ZN330_HUMAN]	-0.5
Q6P4E1	Protein CASC4 OS=Homo sapiens GN=CASC4 PE=1 SV=1 - [CASC4_HUMAN]	-0.5
Q9UHY1	Nuclear receptor-binding protein OS=Homo sapiens GN=NRBP1 PE=1 SV=1 - [NRBP_HUMAN]	-0.5
Q9UBZ9	DNA repair protein REV1 OS=Homo sapiens GN=REV1 PE=1 SV=1 - [REV1_HUMAN]	-0.5
P35442	Thrombospondin-2 OS=Homo sapiens GN=THBS2 PE=1 SV=2 - [TSP2_HUMAN]	-0.5
O95394	Phosphoacetylglucosamine mutase OS=Homo sapiens GN=PGM3 PE=1 SV=1 - [AGM1_HUMAN]	-0.5
Q9H6R3	Acyl-CoA synthetase short-chain family member 3, mitochondrial OS=Homo sapiens GN=ACSS3 PE=1 SV=1 - [ACSS3_HUMAN]	-0.5
O95671	N-acetylserotonin O-methyltransferase-like protein OS=Homo sapiens GN=ASMTL PE=1 SV=3 - [ASML_HUMAN]	-0.5
Q9BRT3	Migration and invasion enhancer 1 OS=Homo sapiens GN=MIEN1 PE=1 SV=1 - [MIEN1_HUMAN]	-0.5
Q8TEQ6	Gem-associated protein 5 OS=Homo sapiens GN=GEMIN5 PE=1 SV=3 - [GEMI5_HUMAN]	-0.5
P19623	Spermidine synthase OS=Homo sapiens GN=SRM PE=1 SV=1 - [SPEE_HUMAN]	-0.5
Q969M3	Protein YIPF5 OS=Homo sapiens GN=YIPF5 PE=1 SV=1 - [YIPF5_HUMAN]	-0.5
Q9BPX7	UPF0415 protein C7orf25 OS=Homo sapiens GN=C7orf25 PE=1 SV=1 - [CG025_HUMAN]	-0.5
O15173	Membrane-associated progesterone receptor component 2 OS=Homo sapiens GN=PGRMC2 PE=1 SV=1 - [PGRC2_HUMAN]	-0.5
Q9UPQ0	LIM and calponin homology domains-containing protein 1 OS=Homo sapiens GN=LIMCH1 PE=1 SV=4 - [LIMC1_HUMAN]	-0.5
Q9NYU1	UDP-glucose:glycoprotein glucosyltransferase 2 OS=Homo sapiens GN=UGGT2 PE=1 SV=4 - [UGGG2_HUMAN]	-0.5
Q96RF0	Sorting nexin-18 OS=Homo sapiens GN=SNX18 PE=1 SV=2 - [SNX18_HUMAN]	-0.5
P51884	Lumican OS=Homo sapiens GN=LUM PE=1 SV=2 - [LUM_HUMAN]	-0.5
Q9NP79	Vacuolar protein sorting-associated protein VTA1 homolog OS=Homo sapiens GN=VTA1 PE=1 SV=1 - [VTA1_HUMAN]	-0.5
Q00534	Cyclin-dependent kinase 6 OS=Homo sapiens GN=CDK6 PE=1 SV=1 - [CDK6_HUMAN]	-0.5
Q04721	Neurogenic locus notch homolog protein 2 OS=Homo sapiens GN=NOTCH2 PE=1 SV=3 - [NOTC2_HUMAN]	-0.5
Q8NHP8	Putative phospholipase B-like 2 OS=Homo sapiens GN=PLBD2 PE=1 SV=2 - [PLBL2_HUMAN]	-0.5
Q96D15	Reticulocalbin-3 OS=Homo sapiens GN=RCN3 PE=1 SV=1 - [RCN3_HUMAN]	-0.5
P43005	Excitatory amino acid transporter 3 OS=Homo sapiens GN=SLC1A1 PE=1 SV=2 - [EAA3_HUMAN]	-0.5

O00410	Importin-5 OS=Homo sapiens GN=IPO5 PE=1 SV=4 - [IPO5_HUMAN]	-0.5
Q96EM0	Trans-L-3-hydroxyproline dehydratase OS=Homo sapiens GN=L3HYPDH PE=1 SV=2 - [T3HPD_HUMAN]	-0.5
Q13563	Polycystin-2 OS=Homo sapiens GN=PKD2 PE=1 SV=3 - [PKD2_HUMAN]	-0.5
P01023	Alpha-2-macroglobulin OS=Homo sapiens GN=A2M PE=1 SV=3 - [A2MG_HUMAN]	-0.5
P46977	Dolichyl-diphosphooligosaccharide--protein glycosyltransferase subunit STT3A OS=Homo sapiens GN=STT3A PE=1 SV=2 - [STT3A_HUMAN]	-0.5
Q8NBS9	Thioredoxin domain-containing protein 5 OS=Homo sapiens GN=TXNDC5 PE=1 SV=2 - [TXND5_HUMAN]	-0.5
Q96JJ7	Protein disulfide-isomerase TMX3 OS=Homo sapiens GN=TMX3 PE=1 SV=2 - [TMX3_HUMAN]	-0.5
Q15758	Neutral amino acid transporter B(0) OS=Homo sapiens GN=SLC1A5 PE=1 SV=2 - [AAAT_HUMAN]	-0.5
P18077	60S ribosomal protein L35a OS=Homo sapiens GN=RPL35A PE=1 SV=2 - [RL35A_HUMAN]	-0.5
O94855	Protein transport protein Sec24D OS=Homo sapiens GN=SEC24D PE=1 SV=2 - [SC24D_HUMAN]	-0.5
O43752	Syntaxin-6 OS=Homo sapiens GN=STX6 PE=1 SV=1 - [STX6_HUMAN]	-0.5
Q00587	Cdc42 effector protein 1 OS=Homo sapiens GN=CDC42EP1 PE=1 SV=1 - [BORG5_HUMAN]	-0.5
Q8N8S7	Protein enabled homolog OS=Homo sapiens GN=ENAH PE=1 SV=2 - [ENAH_HUMAN]	-0.5
P62633	Cellular nucleic acid-binding protein OS=Homo sapiens GN=CNBP PE=1 SV=1 - [CNBP_HUMAN]	-0.5
Q8N0Y7	Probable phosphoglycerate mutase 4 OS=Homo sapiens GN=PGAM4 PE=2 SV=1 - [PGAM4_HUMAN]	-0.5
P08758	Annexin A5 OS=Homo sapiens GN=ANXA5 PE=1 SV=2 - [ANXA5_HUMAN]	-0.5
P04844	Dolichyl-diphosphooligosaccharide--protein glycosyltransferase subunit 2 OS=Homo sapiens GN=RPN2 PE=1 SV=3 - [RPN2_HUMAN]	-0.5
Q9NW08	DNA-directed RNA polymerase III subunit RPC2 OS=Homo sapiens GN=POLR3B PE=1 SV=2 - [RPC2_HUMAN]	-0.5
Q8WW59	SPRY domain-containing protein 4 OS=Homo sapiens GN=SPRYD4 PE=1 SV=2 - [SPRY4_HUMAN]	-0.5
Q99805	Transmembrane 9 superfamily member 2 OS=Homo sapiens GN=TM9SF2 PE=1 SV=1 - [TM9S2_HUMAN]	-0.5
Q9UI14	Prenylated Rab acceptor protein 1 OS=Homo sapiens GN=RABAC1 PE=1 SV=1 - [PRAF1_HUMAN]	-0.5
P61619	Protein transport protein Sec61 subunit alpha isoform 1 OS=Homo sapiens GN=SEC61A1 PE=1 SV=2 - [S61A1_HUMAN]	-0.5
Q8NCG7	Sn1-specific diacylglycerol lipase beta OS=Homo sapiens GN=DAGLB PE=1 SV=2 - [DGLB_HUMAN]	-0.5
Q5W111	SPRY domain-containing protein 7 OS=Homo sapiens GN=SPRYD7 PE=1 SV=2 - [SPRY7_HUMAN]	-0.5
Q9NZL4	Hsp70-binding protein 1 OS=Homo sapiens GN=HSPBP1 PE=1 SV=1 - [HPBP1_HUMAN]	-0.5
O14732	Inositol monophosphatase 2 OS=Homo sapiens GN=IMPA2 PE=1 SV=1 - [IMPA2_HUMAN]	-0.5
Q9BX95	Sphingosine-1-phosphate phosphatase 1 OS=Homo sapiens GN=SGPP1 PE=1 SV=2 - [SGPP1_HUMAN]	-0.5
Q13530	Serine incorporator 3 OS=Homo sapiens GN=SERINC3 PE=2 SV=2 - [SERC3_HUMAN]	-0.5
P08174	Complement decay-accelerating factor OS=Homo sapiens GN=CD55 PE=1 SV=4 - [DAF_HUMAN]	-0.5
O95772	MLN64 N-terminal domain homolog OS=Homo sapiens GN=STARD3NL PE=1 SV=1 - [MENTO_HUMAN]	-0.5
Q53EP0	Fibronectin type III domain-containing protein 3B OS=Homo sapiens GN=FNDC3B PE=1 SV=2 - [FND3B_HUMAN]	-0.5
Q32P28	Prolyl 3-hydroxylase 1 OS=Homo sapiens GN=LEPRE1 PE=1 SV=2 - [P3H1_HUMAN]	-0.5
P06737	Glycogen phosphorylase, liver form OS=Homo sapiens GN=PYGL PE=1 SV=4 - [PYGL_HUMAN]	-0.5
P86790	Vacuolar fusion protein CCZ1 homolog B OS=Homo sapiens GN=CCZ1B PE=1 SV=1 - [CCZ1B_HUMAN]	-0.5
Q8IUH4	Palmitoyltransferase ZDHHC13 OS=Homo sapiens GN=ZDHHC13 PE=1 SV=3 - [ZDH13_HUMAN]	-0.5
P16989	Y-box-binding protein 3 OS=Homo sapiens GN=YBX3 PE=1 SV=4 - [YBOX3_HUMAN]	-0.5
Q96AY3	Peptidyl-prolyl cis-trans isomerase FKBP10 OS=Homo sapiens GN=FKBP10 PE=1 SV=1 - [FKB10_HUMAN]	-0.5
Q8TDQ7	Glucosamine-6-phosphate isomerase 2 OS=Homo sapiens GN=GNPDA2 PE=1 SV=1 - [GNPI2_HUMAN]	-0.5
O00391	Sulfhydryl oxidase 1 OS=Homo sapiens GN=QSOX1 PE=1 SV=3 - [QSOX1_HUMAN]	-0.5
P13995	Bifunctional methylenetetrahydrofolate dehydrogenase/cyclohydrolase, mitochondrial OS=Homo sapiens GN=MTHFD2 PE=1 SV=2 - [MTDC_HUMAN]	-0.5
Q9Y4D2	Sn1-specific diacylglycerol lipase alpha OS=Homo sapiens GN=DAGLA PE=1 SV=3 - [DGLA_HUMAN]	-0.5
Q9NWM8	Peptidyl-prolyl cis-trans isomerase FKBP14 OS=Homo sapiens GN=FKBP14 PE=1 SV=1 - [FKB14_HUMAN]	-0.5
O60784	Target of Myb protein 1 OS=Homo sapiens GN=TOM1 PE=1 SV=2 - [TOM1_HUMAN]	-0.5
Q8N3K9	Cardiomyopathy-associated protein 5 OS=Homo sapiens GN=CMYA5 PE=1 SV=3 - [CMYA5_HUMAN]	-0.5
Q8IY95	Transmembrane protein 192 OS=Homo sapiens GN=TMEM192 PE=1 SV=1 - [TM192_HUMAN]	-0.5
P54577	Tyrosine--tRNA ligase, cytoplasmic OS=Homo sapiens GN=YARS PE=1 SV=4 - [SYYC_HUMAN]	-0.6
O43852	Calumenin OS=Homo sapiens GN=CALU PE=1 SV=2 - [CALU_HUMAN]	-0.6
O95340	Bifunctional 3'-phosphoadenosine 5'-phosphosulfate synthase 2 OS=Homo sapiens GN=PAPSS2 PE=1 SV=2 - [PAPS2_HUMAN]	-0.6
Q9NPJ3	Acyl-coenzyme A thioesterase 13 OS=Homo sapiens GN=ACOT13 PE=1 SV=1 - [ACO13_HUMAN]	-0.6
Q5VTQ0	Tetratricopeptide repeat protein 39B OS=Homo sapiens GN=TTC39B PE=2 SV=4 - [TT39B_HUMAN]	-0.6
Q9Y210	Short transient receptor potential channel 6 OS=Homo sapiens GN=TRPC6 PE=1 SV=1 - [TRPC6_HUMAN]	-0.6
Q9NR48	Histone-lysine N-methyltransferase ASH1L OS=Homo sapiens GN=ASH1L PE=1 SV=2 - [ASH1L_HUMAN]	-0.6

P13674	Prolyl 4-hydroxylase subunit alpha-1 OS=Homo sapiens GN=P4HA1 PE=1 SV=2 - [P4HA1_HUMAN]	-0.6
O60427	Fatty acid desaturase 1 OS=Homo sapiens GN=FADS1 PE=1 SV=3 - [FADS1_HUMAN]	-0.6
P61927	60S ribosomal protein L37 OS=Homo sapiens GN=RPL37 PE=1 SV=2 - [RL37_HUMAN]	-0.6
Q3C1V8	Brain-specific homeobox protein homolog OS=Homo sapiens GN=BSX PE=2 SV=2 - [BSH_HUMAN]	-0.6
A8MT70	Zinc finger B-box domain-containing protein 1 OS=Homo sapiens GN=ZBBX PE=2 SV=3 - [ZBBX_HUMAN]	-0.6
P61587	Rho-related GTP-binding protein RhoE OS=Homo sapiens GN=RND3 PE=1 SV=1 - [RND3_HUMAN]	-0.6
Q9NSY2	StAR-related lipid transfer protein 5 OS=Homo sapiens GN=STARD5 PE=1 SV=2 - [STAR5_HUMAN]	-0.6
Q13445	Transmembrane emp24 domain-containing protein 1 OS=Homo sapiens GN=TMED1 PE=1 SV=1 - [TMED1_HUMAN]	-0.6
P23381	Tryptophan--tRNA ligase, cytoplasmic OS=Homo sapiens GN=WARS PE=1 SV=2 - [SYWC_HUMAN]	-0.6
P09936	Ubiquitin carboxyl-terminal hydrolase isozyme L1 OS=Homo sapiens GN=UCHL1 PE=1 SV=2 - [UCHL1_HUMAN]	-0.6
Q0IIM8	TBC1 domain family member 8B OS=Homo sapiens GN=TBC1D8B PE=1 SV=2 - [TBC8B_HUMAN]	-0.6
P09619	Platelet-derived growth factor receptor beta OS=Homo sapiens GN=PDGFRB PE=1 SV=1 - [PGFRB_HUMAN]	-0.6
A8MXV4	Nucleoside diphosphate-linked moiety X motif 19, mitochondrial OS=Homo sapiens GN=NUDT19 PE=1 SV=1 - [NUD19_HUMAN]	-0.6
P17302	Gap junction alpha-1 protein OS=Homo sapiens GN=GJA1 PE=1 SV=2 - [CXA1_HUMAN]	-0.6
Q16222	UDP-N-acetylhexosamine pyrophosphorylase OS=Homo sapiens GN=UAP1 PE=1 SV=3 - [UAP1_HUMAN]	-0.6
P35052	Glypican-1 OS=Homo sapiens GN=GPC1 PE=1 SV=2 - [GPC1_HUMAN]	-0.6
P61225	Ras-related protein Rap-2b OS=Homo sapiens GN=RAP2B PE=1 SV=1 - [RAP2B_HUMAN]	-0.6
O94925	Glutaminase kidney isoform, mitochondrial OS=Homo sapiens GN=GLS PE=1 SV=1 - [GLSK_HUMAN]	-0.6
Q9H939	Proline-serine-threonine phosphatase-interacting protein 2 OS=Homo sapiens GN=PSTPIP2 PE=1 SV=4 - [PPIP2_HUMAN]	-0.6
Q9ULT0	Tetratricopeptide repeat protein 7A OS=Homo sapiens GN=TTC7A PE=1 SV=3 - [TTC7A_HUMAN]	-0.6
O00462	Beta-mannosidase OS=Homo sapiens GN=MANBA PE=1 SV=3 - [MANBA_HUMAN]	-0.6
P45877	Peptidyl-prolyl cis-trans isomerase C OS=Homo sapiens GN=PPIC PE=1 SV=1 - [PPIC_HUMAN]	-0.6
O00505	Importin subunit alpha-4 OS=Homo sapiens GN=KPNA3 PE=1 SV=2 - [IMA4_HUMAN]	-0.6
Q6AWC2	Protein WWC2 OS=Homo sapiens GN=WWC2 PE=1 SV=2 - [WWC2_HUMAN]	-0.6
P28845	Corticosteroid 11-beta-dehydrogenase isozyme 1 OS=Homo sapiens GN=HSD11B1 PE=1 SV=3 - [DHI1_HUMAN]	-0.6
O15118	Niemann-Pick C1 protein OS=Homo sapiens GN=NPC1 PE=1 SV=2 - [NPC1_HUMAN]	-0.6
Q9H7M9	Platelet receptor Gi24 OS=Homo sapiens GN=C10orf54 PE=1 SV=3 - [GI24_HUMAN]	-0.6
P50225	Sulfotransferase 1A1 OS=Homo sapiens GN=SULT1A1 PE=1 SV=3 - [ST1A1_HUMAN]	-0.6
P02794	Ferritin heavy chain OS=Homo sapiens GN=FTH1 PE=1 SV=2 - [FRIH_HUMAN]	-0.6
Q9Y5U9	Immediate early response 3-interacting protein 1 OS=Homo sapiens GN=IER3IP1 PE=1 SV=1 - [IR3IP_HUMAN]	-0.6
Q8IZV5	Retinol dehydrogenase 10 OS=Homo sapiens GN=RDH10 PE=1 SV=1 - [RDH10_HUMAN]	-0.6
Q9C0H2	Protein tweety homolog 3 OS=Homo sapiens GN=TTYH3 PE=1 SV=3 - [TTYH3_HUMAN]	-0.6
P61803	Dolichyl-diphosphooligosaccharide--protein glycosyltransferase subunit DAD1 OS=Homo sapiens GN=DAD1 PE=1 SV=3 - [DAD1_HUMAN]	-0.6
P13612	Integrin alpha-4 OS=Homo sapiens GN=ITGA4 PE=1 SV=3 - [ITA4_HUMAN]	-0.6
Q8IY22	C-Maf-inducing protein OS=Homo sapiens GN=CMIP PE=1 SV=3 - [CMIP_HUMAN]	-0.6
Q9UDY8	Mucosa-associated lymphoid tissue lymphoma translocation protein 1 OS=Homo sapiens GN=MALT1 PE=1 SV=1 - [MALT1_HUMAN]	-0.6
O15260	Surfeit locus protein 4 OS=Homo sapiens GN=SURF4 PE=1 SV=3 - [SURF4_HUMAN]	-0.6
Q9H0A8	COMM domain-containing protein 4 OS=Homo sapiens GN=COMMD4 PE=1 SV=1 - [COMD4_HUMAN]	-0.6
O60291	E3 ubiquitin-protein ligase MGRN1 OS=Homo sapiens GN=MGRN1 PE=1 SV=2 - [MGRN1_HUMAN]	-0.6
Q9GZY8	Mitochondrial fission factor OS=Homo sapiens GN=MFF PE=1 SV=1 - [MFF_HUMAN]	-0.6
Q9ULG6	Cell cycle progression protein 1 OS=Homo sapiens GN=CCPG1 PE=2 SV=3 - [CCPG1_HUMAN]	-0.6
P61457	Pterin-4-alpha-carbinolamine dehydratase OS=Homo sapiens GN=PCBD1 PE=1 SV=2 - [PHS_HUMAN]	-0.6
Q16799	Reticulon-1 OS=Homo sapiens GN=RTN1 PE=1 SV=1 - [RTN1_HUMAN]	-0.6
Q13505	Metaxin-1 OS=Homo sapiens GN=MTX1 PE=1 SV=2 - [MTX1_HUMAN]	-0.6
Q96EK6	Glucosamine 6-phosphate N-acetyltransferase OS=Homo sapiens GN=GNPNAT1 PE=1 SV=1 - [GNA1_HUMAN]	-0.6
O60513	Beta-1,4-galactosyltransferase 4 OS=Homo sapiens GN=B4GALT4 PE=1 SV=1 - [B4GT4_HUMAN]	-0.6
O95980	Reversion-inducing cysteine-rich protein with Kazal motifs OS=Homo sapiens GN=RECK PE=1 SV=1 - [RECK_HUMAN]	-0.6
Q96JY6	PDZ and LIM domain protein 2 OS=Homo sapiens GN=PDLIM2 PE=1 SV=1 - [PDLI2_HUMAN]	-0.6
P15924	Desmoplakin OS=Homo sapiens GN=DSP PE=1 SV=3 - [DESP_HUMAN]	-0.6
P07996	Thrombospondin-1 OS=Homo sapiens GN=THBS1 PE=1 SV=2 - [TSP1_HUMAN]	-0.7
P15531	Nucleoside diphosphate kinase A OS=Homo sapiens GN=NME1 PE=1 SV=1 - [NDKA_HUMAN]	-0.7
Q06033	Inter-alpha-trypsin inhibitor heavy chain H3 OS=Homo sapiens GN=ITIH3 PE=1 SV=2 - [ITIH3_HUMAN]	-0.7

Q9UBX5	Fibulin-5 OS=Homo sapiens GN=FBLN5 PE=1 SV=1 - [FBLN5_HUMAN]	-0.7
Q96BA8	Cyclic AMP-responsive element-binding protein 3-like protein 1 OS=Homo sapiens GN=CREB3L1 PE=1 SV=1 - [CR3L1_HUMAN]	-0.7
P53985	Monocarboxylate transporter 1 OS=Homo sapiens GN=SLC16A1 PE=1 SV=3 - [MOT1_HUMAN]	-0.7
O94966	Ubiquitin carboxyl-terminal hydrolase 19 OS=Homo sapiens GN=USP19 PE=1 SV=2 - [UBP19_HUMAN]	-0.7
P08253	72 kDa type IV collagenase OS=Homo sapiens GN=MMP2 PE=1 SV=2 - [MMP2_HUMAN]	-0.7
O75339	Cartilage intermediate layer protein 1 OS=Homo sapiens GN=CILP PE=1 SV=4 - [CILP1_HUMAN]	-0.7
Q16585	Beta-sarcoglycan OS=Homo sapiens GN=SGCB PE=1 SV=1 - [SGCB_HUMAN]	-0.7
Q96QT4	Transient receptor potential cation channel subfamily M member 7 OS=Homo sapiens GN=TRPM7 PE=1 SV=1 - [TRPM7_HUMAN]	-0.7
P17813	Endoglin OS=Homo sapiens GN=ENG PE=1 SV=2 - [EGLN_HUMAN]	-0.7
P98155	Very low-density lipoprotein receptor OS=Homo sapiens GN=VLDLR PE=1 SV=1 - [VLDLR_HUMAN]	-0.7
P04114	Apolipoprotein B-100 OS=Homo sapiens GN=APOB PE=1 SV=2 - [APOB_HUMAN]	-0.7
P30536	Translocator protein OS=Homo sapiens GN=TSPO PE=1 SV=3 - [TSPOA_HUMAN]	-0.7
P08962	CD63 antigen OS=Homo sapiens GN=CD63 PE=1 SV=2 - [CD63_HUMAN]	-0.7
O00300	Tumor necrosis factor receptor superfamily member 11B OS=Homo sapiens GN=TNFRSF11B PE=1 SV=3 - [TR11B_HUMAN]	-0.7
P98082	Disabled homolog 2 OS=Homo sapiens GN=DAB2 PE=1 SV=3 - [DAB2_HUMAN]	-0.7
Q07092	Collagen alpha-1(XVI) chain OS=Homo sapiens GN=COL16A1 PE=1 SV=2 - [COGA1_HUMAN]	-0.7
P05783	Keratin, type I cytoskeletal 18 OS=Homo sapiens GN=KRT18 PE=1 SV=2 - [K1C18_HUMAN]	-0.7
P20908	Collagen alpha-1(V) chain OS=Homo sapiens GN=COL5A1 PE=1 SV=3 - [CO5A1_HUMAN]	-0.7
O75298	Reticulon-2 OS=Homo sapiens GN=RTN2 PE=1 SV=1 - [RTN2_HUMAN]	-0.7
Q8TD30	Alanine aminotransferase 2 OS=Homo sapiens GN=GPT2 PE=1 SV=1 - [ALAT2_HUMAN]	-0.7
O60443	Non-syndromic hearing impairment protein 5 OS=Homo sapiens GN=DFNA5 PE=1 SV=2 - [DFNA5_HUMAN]	-0.7
P05997	Collagen alpha-2(V) chain OS=Homo sapiens GN=COL5A2 PE=1 SV=3 - [CO5A2_HUMAN]	-0.7
P29279	Connective tissue growth factor OS=Homo sapiens GN=CTGF PE=1 SV=2 - [CTGF_HUMAN]	-0.7
O00622	Protein CYR61 OS=Homo sapiens GN=CYR61 PE=1 SV=1 - [CYR61_HUMAN]	-0.7
P50454	Serpin H1 OS=Homo sapiens GN=SERPINH1 PE=1 SV=2 - [SERPH_HUMAN]	-0.7
Q5S007	Leucine-rich repeat serine/threonine-protein kinase 2 OS=Homo sapiens GN=LRRK2 PE=1 SV=2 - [LRRK2_HUMAN]	-0.8
Q4ZHG4	Fibronectin type III domain-containing protein 1 OS=Homo sapiens GN=FNDC1 PE=1 SV=4 - [FNDC1_HUMAN]	-0.8
P20839	Inosine-5'-monophosphate dehydrogenase 1 OS=Homo sapiens GN=IMPDH1 PE=1 SV=2 - [IMDH1_HUMAN]	-0.8
P02786	Transferrin receptor protein 1 OS=Homo sapiens GN=TFRC PE=1 SV=2 - [TFR1_HUMAN]	-0.8
Q14714	Sarcospan OS=Homo sapiens GN=SSPN PE=2 SV=3 - [SSPN_HUMAN]	-0.8
P60891	Ribose-phosphate pyrophosphokinase 1 OS=Homo sapiens GN=PRPS1 PE=1 SV=2 - [PRPS1_HUMAN]	-0.8
Q10472	Polypeptide N-acetylgalactosaminyltransferase 1 OS=Homo sapiens GN=GALNT1 PE=1 SV=1 - [GALT1_HUMAN]	-0.8
P08195	4F2 cell-surface antigen heavy chain OS=Homo sapiens GN=SLC3A2 PE=1 SV=3 - [4F2_HUMAN]	-0.8
Q9BV68	E3 ubiquitin-protein ligase RNF126 OS=Homo sapiens GN=RNF126 PE=1 SV=1 - [RN126_HUMAN]	-0.8
Q4LDE5	Sushi, von Willebrand factor type A, EGF and pentraxin domain-containing protein 1 OS=Homo sapiens GN=SVEP1 PE=1 SV=3 - [SVEP1_HUMAN]	-0.8
O14495	Lipid phosphate phosphohydrolase 3 OS=Homo sapiens GN=PPAP2B PE=1 SV=1 - [LPP3_HUMAN]	-0.8
P05186	Alkaline phosphatase, tissue-nonspecific isozyme OS=Homo sapiens GN=ALPL PE=1 SV=4 - [PPBT_HUMAN]	-0.8
P39059	Collagen alpha-1(XV) chain OS=Homo sapiens GN=COL15A1 PE=1 SV=2 - [COFA1_HUMAN]	-0.8
P35580	Myosin-10 OS=Homo sapiens GN=MYH10 PE=1 SV=3 - [MYH10_HUMAN]	-0.8
Q9NYL4	Peptidyl-prolyl cis-trans isomerase FKBP11 OS=Homo sapiens GN=FKBP11 PE=1 SV=1 - [FKB11_HUMAN]	-0.8
P13473	Lysosome-associated membrane glycoprotein 2 OS=Homo sapiens GN=LAMP2 PE=1 SV=2 - [LAMP2_HUMAN]	-0.8
P32322	Pyrroline-5-carboxylate reductase 1, mitochondrial OS=Homo sapiens GN=PYCR1 PE=1 SV=2 - [P5CR1_HUMAN]	-0.8
Q9HAP2	MLX-interacting protein OS=Homo sapiens GN=MLXIP PE=1 SV=2 - [MLXIP_HUMAN]	-0.8
Q12841	Follistatin-related protein 1 OS=Homo sapiens GN=FSTL1 PE=1 SV=1 - [FSTL1_HUMAN]	-0.8
O15460	Prolyl 4-hydroxylase subunit alpha-2 OS=Homo sapiens GN=P4HA2 PE=1 SV=1 - [P4HA2_HUMAN]	-0.8
Q9H788	SH2 domain-containing protein 4A OS=Homo sapiens GN=SH2D4A PE=1 SV=1 - [SH24A_HUMAN]	-0.8
P36956	Sterol regulatory element-binding protein 1 OS=Homo sapiens GN=SREBF1 PE=1 SV=2 - [SRBP1_HUMAN]	-0.9
P41250	Glycine--tRNA ligase OS=Homo sapiens GN=GARS PE=1 SV=3 - [SYG_HUMAN]	-0.9
O94832	Unconventional myosin-Id OS=Homo sapiens GN=MYO1D PE=1 SV=2 - [MYO1D_HUMAN]	-0.9
O94808	Glutamine--fructose-6-phosphate aminotransferase [isomerizing] 2 OS=Homo sapiens GN=GFPT2 PE=1 SV=3 - [GFPT2_HUMAN]	-0.9
Q99538	Legumain OS=Homo sapiens GN=LGMN PE=1 SV=1 - [LGMN_HUMAN]	-0.9
Q96PY5	Formin-like protein 2 OS=Homo sapiens GN=FMNL2 PE=1 SV=3 - [FMNL2_HUMAN]	-0.9

P54687	Branched-chain-amino-acid aminotransferase, cytosolic OS=Homo sapiens GN=BCAT1 PE=1 SV=3 - [BCAT1_HUMAN]	-0.9
P35749	Myosin-11 OS=Homo sapiens GN=MYH11 PE=1 SV=3 - [MYH11_HUMAN]	-0.9
Q3SY69	Mitochondrial 10-formyltetrahydrofolate dehydrogenase OS=Homo sapiens GN=ALDH1L2 PE=1 SV=2 - [AL1L2_HUMAN]	-0.9
P02751	Fibronectin OS=Homo sapiens GN=FN1 PE=1 SV=4 - [FINC_HUMAN]	-0.9
Q96EN8	Molybdenum cofactor sulfurase OS=Homo sapiens GN=MOCOS PE=1 SV=2 - [MOCOS_HUMAN]	-0.9
Q8N5C1	Protein FAM26E OS=Homo sapiens GN=FAM26E PE=2 SV=1 - [FA26E_HUMAN]	-0.9
Q8WUJ3	Cell migration-inducing and hyaluronan-binding protein OS=Homo sapiens GN=CEMIP PE=1 SV=2 - [CEMIP_HUMAN]	-0.9
Q10588	ADP-ribosyl cyclase/cyclic ADP-ribose hydrolase 2 OS=Homo sapiens GN=BST1 PE=1 SV=2 - [BST1_HUMAN]	-0.9
Q06278	Aldehyde oxidase OS=Homo sapiens GN=AOX1 PE=1 SV=2 - [AOXA_HUMAN]	-0.9
Q9NPR9	Protein GPR108 OS=Homo sapiens GN=GPR108 PE=2 SV=3 - [GP108_HUMAN]	-0.9
P22105	Tenascin-X OS=Homo sapiens GN=TNXB PE=1 SV=3 - [TENX_HUMAN]	-0.9
Q9NX08	COMM domain-containing protein 8 OS=Homo sapiens GN=COMMD8 PE=1 SV=1 - [COMD8_HUMAN]	-0.9
P08123	Collagen alpha-2(I) chain OS=Homo sapiens GN=COL1A2 PE=1 SV=7 - [CO1A2_HUMAN]	-0.9
Q9UKX5	Integrin alpha-11 OS=Homo sapiens GN=ITGA11 PE=1 SV=2 - [ITA11_HUMAN]	-0.9
Q76M96	Coiled-coil domain-containing protein 80 OS=Homo sapiens GN=CCDC80 PE=1 SV=1 - [CCD80_HUMAN]	-0.9
Q15392	Delta(24)-sterol reductase OS=Homo sapiens GN=DHCR24 PE=1 SV=2 - [DHC24_HUMAN]	-0.9
P02792	Ferritin light chain OS=Homo sapiens GN=FTL PE=1 SV=2 - [FRIL_HUMAN]	-1.0
P02461	Collagen alpha-1(III) chain OS=Homo sapiens GN=COL3A1 PE=1 SV=4 - [CO3A1_HUMAN]	-1.0
P07093	Glia-derived nexin OS=Homo sapiens GN=SERPINE2 PE=1 SV=1 - [GDN_HUMAN]	-1.0
O95833	Chloride intracellular channel protein 3 OS=Homo sapiens GN=CLIC3 PE=1 SV=2 - [CLIC3_HUMAN]	-1.0
O15083	ERC protein 2 OS=Homo sapiens GN=ERC2 PE=1 SV=3 - [ERC2_HUMAN]	-1.0
Q05707	Collagen alpha-1(XIV) chain OS=Homo sapiens GN=COL14A1 PE=1 SV=3 - [COEA1_HUMAN]	-1.0
O76070	Gamma-synuclein OS=Homo sapiens GN=SNCG PE=1 SV=2 - [SYUG_HUMAN]	-1.0
O43175	D-3-phosphoglycerate dehydrogenase OS=Homo sapiens GN=PHGDH PE=1 SV=4 - [SERA_HUMAN]	-1.0
Q9NRA2	Sialin OS=Homo sapiens GN=SLC17A5 PE=1 SV=2 - [S17A5_HUMAN]	-1.0
P07585	Decorin OS=Homo sapiens GN=DCN PE=1 SV=1 - [PGS2_HUMAN]	-1.0
P35555	Fibrillin-1 OS=Homo sapiens GN=FBN1 PE=1 SV=3 - [FBN1_HUMAN]	-1.0
Q9UNW1	Multiple inositol polyphosphate phosphatase 1 OS=Homo sapiens GN=MINPP1 PE=1 SV=1 - [MINP1_HUMAN]	-1.1
O75781	Paralemmin-1 OS=Homo sapiens GN=PALM PE=1 SV=2 - [PALM_HUMAN]	-1.1
P09486	SPARC OS=Homo sapiens GN=SPARC PE=1 SV=1 - [SPRC_HUMAN]	-1.1
Q6NXT6	Transmembrane anterior posterior transformation protein 1 homolog OS=Homo sapiens GN=TAPT1 PE=1 SV=1 - [TAPT1_HUMAN]	-1.1
P35556	Fibrillin-2 OS=Homo sapiens GN=FBN2 PE=1 SV=3 - [FBN2_HUMAN]	-1.1
P02452	Collagen alpha-1(I) chain OS=Homo sapiens GN=COL1A1 PE=1 SV=5 - [CO1A1_HUMAN]	-1.1
P05121	Plasminogen activator inhibitor 1 OS=Homo sapiens GN=SERPINE1 PE=1 SV=1 - [PAI1_HUMAN]	-1.1
P00325	Alcohol dehydrogenase 1B OS=Homo sapiens GN=ADH1B PE=1 SV=2 - [ADH1B_HUMAN]	-1.1
P35520	Cystathionine beta-synthase OS=Homo sapiens GN=CBS PE=1 SV=2 - [CBS_HUMAN]	-1.1
Q96CG8	Collagen triple helix repeat-containing protein 1 OS=Homo sapiens GN=CTHRC1 PE=1 SV=1 - [CTHR1_HUMAN]	-1.1
P36959	GMP reductase 1 OS=Homo sapiens GN=GMPR PE=1 SV=1 - [GMPR1_HUMAN]	-1.1
P19320	Vascular cell adhesion protein 1 OS=Homo sapiens GN=VCAM1 PE=1 SV=1 - [VCAM1_HUMAN]	-1.1
P18085	ADP-ribosylation factor 4 OS=Homo sapiens GN=ARF4 PE=1 SV=3 - [ARF4_HUMAN]	-1.1
Q16890	Tumor protein D53 OS=Homo sapiens GN=TPD52L1 PE=1 SV=1 - [TPD53_HUMAN]	-1.2
Q16822	Phosphoenolpyruvate carboxykinase [GTP], mitochondrial OS=Homo sapiens GN=PCK2 PE=1 SV=3 - [PCKGM_HUMAN]	-1.2
Q8WV93	Lactation elevated protein 1 OS=Homo sapiens GN=LACE1 PE=2 SV=2 - [LACE1_HUMAN]	-1.2
O43592	Exportin-T OS=Homo sapiens GN=XPOT PE=1 SV=2 - [XPOT_HUMAN]	-1.2
Q9Y617	Phosphoserine aminotransferase OS=Homo sapiens GN=PSAT1 PE=1 SV=2 - [SERC_HUMAN]	-1.3
Q5T0U0	Coiled-coil domain-containing protein 122 OS=Homo sapiens GN=CCDC122 PE=1 SV=1 - [CC122_HUMAN]	-1.3
P08729	Keratin, type II cytoskeletal 7 OS=Homo sapiens GN=KRT7 PE=1 SV=5 - [K2C7_HUMAN]	-1.4
Q01650	Large neutral amino acids transporter small subunit 1 OS=Homo sapiens GN=SLC7A5 PE=1 SV=2 - [LAT1_HUMAN]	-1.4
P26022	Pentraxin-related protein PTX3 OS=Homo sapiens GN=PTX3 PE=1 SV=3 - [PTX3_HUMAN]	-1.4
Q96Q07	BTB/POZ domain-containing protein 9 OS=Homo sapiens GN=BTBD9 PE=2 SV=2 - [BTBD9_HUMAN]	-1.4
P08243	Asparagine synthetase [glutamine-hydrolyzing] OS=Homo sapiens GN=ASNS PE=1 SV=4 - [ASNS_HUMAN]	-1.5
Q9NZU5	LIM and cysteine-rich domains protein 1 OS=Homo sapiens GN=LMCD1 PE=1 SV=1 - [LMCD1_HUMAN]	-1.5
O14880	Microsomal glutathione S-transferase 3 OS=Homo sapiens GN=MGST3 PE=1 SV=1 - [MGST3_HUMAN]	-1.5

Q16853	Membrane primary amine oxidase OS=Homo sapiens GN=AOC3 PE=1 SV=3 - [AOC3_HUMAN]	-1.5
P47895	Aldehyde dehydrogenase family 1 member A3 OS=Homo sapiens GN=ALDH1A3 PE=1 SV=2 - [AL1A3_HUMAN]	-1.6
P53801	Pituitary tumor-transforming gene 1 protein-interacting protein OS=Homo sapiens GN=PTTG1IP PE=1 SV=1 - [PTTG_HUMAN]	-1.6
Q9Y2J2	Band 4.1-like protein 3 OS=Homo sapiens GN=EPB41L3 PE=1 SV=2 - [E41L3_HUMAN]	-1.6
Q16678	Cytochrome P450 1B1 OS=Homo sapiens GN=CYP1B1 PE=1 SV=2 - [CP1B1_HUMAN]	-1.7
P17936	Insulin-like growth factor-binding protein 3 OS=Homo sapiens GN=IGFBP3 PE=1 SV=2 - [IBP3_HUMAN]	-1.7
