# Supplementary material for: Quantitative proteomic profiling of primary cancer-associated fibroblasts in oesophageal adenocarcinoma
Source: Br J Cancer. 2018 Mar 29;118(9):1200–7. doi: 10.1038/s41416-018-0042-9 (PMC5943522; doi:10.1038/s41416-018-0042-9)
Supplement: Supplementary file 1 — Supplementary Table 1. Analysed proteins (peptide FDR < 0.05) [file 41416_2018_42_MOESM1_ESM.pdf]

Supplementary Table 1. Analysed proteins (peptide FDR p<0.05)

Accession	Description	ΣCoverage	Σ# Proteins	Σ# Unique Peptides	Σ# Peptides	Σ# PSMs	CAF1/NOF1	CAF2/NOF2	CAF3/NOF3	CAF4/NOF4	Score A(3,6)	Coverage A(3,6)	# Peptides A(3,6)	# PSM A(3,6)	# AAs	MW [kDa]	calc. pI
Q9NRX3	NADH dehydrogenase [ubiquinone] 1 alpha subcomplex subunit 4-like 2 OS=Homo sapiens GN=NDUFA4L2 PE=2 SV=1 - [NUA4L_HUMAN]	9.20	1	1	1	1	0.092	0.553	0.956	0.107	2.54	9.20	1	1	87	10.0	9.92
P25940	Collagen alpha-3(V) chain OS=Homo sapiens GN=COL5A3 PE=1 SV=3 - [CO5A3_HUMAN]	1.55	1	1	1	1	0.833	0.206	0.265	0.121	0.00	1.55	1	1	1745	172.0	6.87
Q9H7Z6	Histone acetyltransferase KAT8 OS=Homo sapiens GN=KAT8 PE=1 SV=2 - [KAT8_HUMAN]	1.53	1	1	1	1	0.264	0.628	0.723	0.121	0.00	1.53	1	1	458	52.4	8.27
Q8WYJ6	Septin-1 OS=Homo sapiens GN=SEPT1 PE=1 SV=2 - [SEPT1_HUMAN]	8.45	1	2	2	2	1.574	0.082	0.101	0.122	3.27	8.45	2	2	367	41.9	5.80
Q8NA66	Cyclic nucleotide-binding domain-containing protein 1 OS=Homo sapiens GN=CNBD1 PE=2 SV=1 - [CNBD1_HUMAN	2.75	1	1	1	2	0.256	0.468	0.816	0.131	0.00	2.75	1	2	436	50.2	9.07
P53801	Pituitary tumor-transforming gene 1 protein-interacting protein OS=Homo sapiens GN=PTTG1IP PE=1 SV=1 - [PTTG_HUMAN]	10.56	1	2	2	4	1.324	0.162	0.379	0.136	4.18	10.56	3	4	180	20.3	8.79
Q15772	Striated muscle preferentially expressed protein kinase OS=Homo sapiens GN=SPEG PE=1 SV=4 - [SPEG_HUMAN]	0.55	1	1	2	4	0.257	0.901	0.618	0.143	7.79	0.55	2	4	3267	354.1	8.51
P26927	Hepatocyte growth factor-like protein OS=Homo sapiens GN=MST1 PE=1 SV=2 - [HGFL_HUMAN]	3.38	2	1	1	2	0.198	0.468	0.598	0.164	0.00	3.38	1	2	711	80.3	7.68

Q13360	Zinc finger protein 177 OS=Homo sapiens GN=ZNF177 PE=2 SV=4 - [ZN177_HUMAN]	1.46	1	1	1	1	3.089	1.671	1.425	0.164	2.39	1.46	1	1	481	54.7	8.18
P17936	Insulin-like growth factor-binding protein 3 OS=Homo sapiens GN=IGFBP3 PE=1 SV=2 - [IBP3_HUMAN]	20.62	1	5	5	22	0.301	0.198	0.802	0.167	41.32	20.62	10	22	291	31.7	8.69
P26022	Pentraxin-related protein PTX3 OS=Homo sapiens GN=PTX3 PE=1 SV=3 - [PTX3_HUMAN]	23.62	1	7	7	19	0.148	0.563	1.086	0.168	36.29	23.62	12	19	381	41.9	5.01
Q16647	Prostacyclin synthase OS=Homo sapiens GN=PTGIS PE=1 SV=1 - [PTGIS_HUMAN]	30.80	1	12	12	97	0.351	1.698	1.745	0.170	223.29	30.80	22	97	500	57.1	7.31
Q9NZU5	LIM and cysteine-rich domains protein 1 OS=Homo sapiens GN=LMCD1 PE=1 SV=1 - [LMCD1_HUMAN	49.04	1	18	18	70	0.259	0.325	0.987	0.179	182.03	49.04	28	70	365	40.8	7.93
Q14116	Interleukin-18 OS=Homo sapiens GN=IL18 PE=1 SV=1 - [IL18_HUMAN]	11.92	1	3	3	6	6.183	0.093	0.473	0.185	12.68	11.92	5	6	193	22.3	4.67
P32297	Neuronal acetylcholine receptor subunit alpha-3 OS=Homo sapiens GN=CHRNA3 PE=1 SV=4 - [ACHA3_HUMAN]	4.16	1	1	1	3		0.275	0.756	0.195	0.00	4.16	1	3	505	57.4	6.46
Q9Y4C4	Malignant fibrous histiocytoma-amplified sequence 1 OS=Homo sapiens GN=MFHAS1 PE=1 SV=2 - [MFHA1_HUMAN]	1.90	1	2	2	9	0.199	1.380	0.658	0.198	16.24	1.90	3	9	1052	116.9	7.88
O95760	Interleukin-33 OS=Homo sapiens GN=IL33 PE=1 SV=1 - [IL33_HUMAN]	2.96	1	1	1	1	1.812	0.792	0.475	0.205	2.36	2.96	1	1	270	30.7	8.66

P08243	Asparagine synthetase [glutamine-hydrolyzing] OS=Homo sapiens GN=ASNS PE=1 SV=4 - [ASNS_HUMAN]	27.09	1	14	14	57	0.284	0.485	0.574	0.207	114.24	27.09	22	57	561	64.3	6.86
P08729	Keratin, type II cytoskeletal 7 OS=Homo sapiens GN=KRT7 PE=1 SV=5 - [K2C7_HUMAN]	46.48	3	19	26	218	0.281	0.287	1.116	0.212	463.08	46.48	47	218	469	51.4	5.48
P22674	Cyclin-O OS=Homo sapiens GN=CCNO PE=1 SV=2 - [CCNO_HUMAN]	4.57	1	1	1	1		0.841	1.654	0.214	2.49	4.57	1	1	350	38.1	8.06
Q2WGJ9	Fer-1-like protein 6 OS=Homo sapiens GN=FER1L6 PE=2 SV=2 - [FR1L6_HUMAN]	0.59	1	1	1	2	0.673	0.733	0.920	0.224	1.77	0.59	1	2	1857	209.2	6.38
P47895	Aldehyde dehydrogenase family 1 member A3 OS=Homo sapiens GN=ALDH1A3 PE=1 SV=2 - [AL1A3_HUMAN]	34.57	1	15	18	55	0.402	0.329	0.450	0.232	148.96	34.57	28	55	512	56.1	7.25
Q8IVG5	Sterile alpha motif domain-containing protein 9-like OS=Homo sapiens GN=SAMD9L PE=1 SV=2 - [SAM9L_HUMAN]	2.78	3	3	5	5	1.245	0.665	0.521	0.232	9.59	2.78	5	5	1584	184.4	8.02
P35908	Keratin, type II cytoskeletal 2 epidermal OS=Homo sapiens GN=KRT2 PE=1 SV=2 - [K22E_HUMAN]	23.47	2	8	14	39	0.432	3.835	0.819	0.232	107.32	23.47	21	39	639	65.4	8.00
Q9BXM0	Periaxin OS=Homo sapiens GN=PRX PE=1 SV=2 - [PRAX_HUMAN]	1.92	1	2	3	3	3.267	0.114	0.139	0.233	5.72	1.92	3	3	1461	154.8	7.50
P05120	Plasminogen activator inhibitor 2 OS=Homo sapiens GN=SERPINB2 PE=1 SV=2 - [PAI2_HUMAN]	1.93	1	1	1	15	0.053	0.706	1.550	0.236	28.50	1.93	2	15	415	46.6	5.63

Q495C1	Probable E3 SUMO-protein ligase RNF212 OS=Homo sapiens GN=RNF212 PE=2 SV=1 - [RN212_HUMAN]	4.38	1	1	2	3	0.375	0.595	0.844	0.253	7.71	4.38	2	3	297	33.3	8.68
P07197	Neurofilament medium polypeptide OS=Homo sapiens GN=NEFM PE=1 SV=3 - [NFM_HUMAN]	2.18	2	1	3	130	0.298	0.705	0.973	0.254	321.02	2.18	6	130	916	102.4	4.91
Q9Y2J2	Band 4.1-like protein 3 OS=Homo sapiens GN=EPB41L3 PE=1 SV=2 - [E41L3_HUMAN]	18.49	2	12	15	46	0.147	0.566	0.516	0.256	126.27	18.49	24	46	1087	120.6	5.19
Q8N4C9	Uncharacterized protein C17orf78 OS=Homo sapiens GN=C17orf78 PE=2 SV=2 - [CQ078_HUMAN]	10.91	1	2	2	2	0.265	0.700	0.881	0.257	4.62	10.91	2	2	275	30.5	9.55
Q10588	ADP-ribosyl cyclase/cyclic ADP-ribose hydrolase 2 OS=Homo sapiens GN=BST1 PE=1 SV=2 - [BST1_HUMAN]	8.18	1	3	3	7	0.807	0.582	0.654	0.260	26.25	8.18	4	7	318	35.7	7.80
P02533	Keratin, type I cytoskeletal 14 OS=Homo sapiens GN=KRT14 PE=1 SV=4 - [K1C14_HUMAN]	20.13	7	2	12	40	1.763	2.020	0.506	0.261	77.58	20.13	18	40	472	51.5	5.16
P05783	Keratin, type I cytoskeletal 18 OS=Homo sapiens GN=KRT18 PE=1 SV=2 - [K1C18_HUMAN]	60.00	5	21	24	101	0.402	0.906	1.184	0.261	228.99	60.00	37	101	430	48.0	5.45
O76070	Gamma-synuclein OS=Homo sapiens GN=SNCG PE=1 SV=2 - [SYUG_HUMAN]	55.91	1	5	5	16	0.436	0.535	0.873	0.263	27.75	55.91	8	16	127	13.3	4.86
Q3ZCW2	Galectin-related protein OS=Homo sapiens GN=LGALSL PE=1 SV=2 - [LEGL_HUMAN]	5.23	1	1	1	2	0.389	0.816	0.550	0.265	7.24	5.23	2	2	172	19.0	5.35

O95229	ZW10 interactor OS=Homo sapiens GN=ZWINT PE=1 SV=2 - [ZWINT_HUMA	7.58	1	2	2	2	0.370	0.602	0.806	0.269	5.36	7.58	2	2	277	31.3	5.15
P43007	Neutral amino acid transporter A OS=Homo sapiens GN=SLC1A4 PE=1 SV=1 - [SATT_HUMAN]	4.14	1	1	2	3	0.553	0.318	0.323	0.272	8.51	4.14	3	3	532	55.7	6.25
Q13416	Origin recognition complex subunit 2 OS=Homo sapiens GN=ORC2 PE=1 SV=2 - [ORC2_HUMAN]	1.21	1	1	1	5	0.331	0.276	0.321	0.275	7.62	1.21	2	5	577	65.9	6.51
Q99542	Matrix metalloproteinase-19 OS=Homo sapiens GN=MMP19 PE=1 SV=1 - [MMP19_HUMAN]	4.53	1	1	1	2	0.884	0.672	1.696	0.275	6.41	4.53	1	2	508	57.3	7.59
Q7Z7L9	Zinc finger and SCAN domain-containing protein 2 OS=Homo sapiens GN=ZSCAN2 PE=2 SV=2 - [ZSCA2_HUMAN]	6.51	94	1	2	4	0.394	1.966	3.412	0.280	6.69	6.51	2	4	614	69.5	7.34
P36959	GMP reductase 1 OS=Homo sapiens GN=GMPR PE=1 SV=1 - [GMPR1_HUMAN]	15.65	1	2	6	14	0.398	0.566	0.683	0.281	28.18	15.65	10	14	345	37.4	7.06
Q8N228	Sex comb on midleg-like protein 4 OS=Homo sapiens GN=SCML4 PE=2 SV=2 - [SCML4_HUMAN]	4.83	1	1	1	2	3.049	0.176	0.250	0.284	5.32	4.83	1	2	414	45.0	9.74
Q15063	Periostin OS=Homo sapiens GN=POSTN PE=1 SV=2 - [POSTN_HUMAN]	11.84	1	8	8	18	0.392	1.256	1.276	0.285	24.56	11.84	13	18	836	93.3	7.53
P59923	Zinc finger protein 445 OS=Homo sapiens GN=ZNF445 PE=2 SV=1 - [ZN445_HUMAN]	2.33	11	1	2	3	0.747	0.707	0.846	0.285	2.55	2.33	3	3	1031	118.9	9.39

Q9Y617	Phosphoserine aminotransferase OS=Homo sapiens GN=PSAT1 PE=1 SV=2 - [SERC_HUMAN]	47.57	1	18	18	87	0.283	0.555	0.659	0.287	200.78	47.57	31	87	370	40.4	7.66
Q6ZVL6	UPF0606 protein KIAA1549L OS=Homo sapiens GN=KIAA1549L PE=2 SV=2 - [K154L_HUMAN]	0.54	1	1	1	1	1.816	0.091	0.159	0.289	0.00	0.54	1	1	1849	198.9	8.31
Q8IWU6	Extracellular sulfatase Sulf-1 OS=Homo sapiens GN=SULF1 PE=1 SV=1 - [SULF1_HUMAN]	9.07	1	9	9	22	0.235	1.161	3.680	0.292	38.36	9.07	14	22	871	101.0	9.09
P02647	Apolipoprotein A-I OS=Homo sapiens GN=APOA1 PE=1 SV=1 - [APOA1_HUMAN]	5.62	1	1	2	3	0.288	0.087	0.193	0.292	7.62	5.62	2	3	267	30.8	5.76
Q16822	Phosphoenolpyruvate carboxykinase [GTP], mitochondrial OS=Homo sapiens GN=PCK2 PE=1 SV=3 - [PCKGM_HUMAN]	39.53	1	19	20	101	0.367	0.566	0.627	0.303	264.70	39.53	34	101	640	70.7	7.62
P04264	Keratin, type II cytoskeletal 1 OS=Homo sapiens GN=KRT1 PE=1 SV=6 - [K2C1_HUMAN]	42.86	1	22	28	127	0.956	2.561	0.514	0.305	315.28	42.86	46	127	644	66.0	8.12
Q9UJV8	Purine-rich element-binding protein gamma OS=Homo sapiens GN=PURG PE=2 SV=1 - [PURG_HUMAN]	7.49	2	1	3	12	0.339	0.325	0.345	0.305	16.45	7.49	4	12	347	39.5	9.55
Q6ZMR3	L-lactate dehydrogenase A-like 6A OS=Homo sapiens GN=LDHAL6A PE=2 SV=1 - [LDH6A_HUMAN]	7.83	1	1	4	82	0.279	0.218	0.209	0.307	162.19	7.83	6	82	332	36.5	6.99
Q08629	Testican-1 OS=Homo sapiens GN=SPOCK1 PE=1 SV=1 - [TICN1_HUMAN]	2.96	1	1	1	2	0.127	0.491	0.651	0.307	0.00	2.96	2	2	439	49.1	6.10

P07093	Glia-derived nexin OS=Homo sapiens GN=SERPINE2 PE=1 SV=1 - [GDN_HUMAN]	22.61	1	10	10	36	0.215	1.038	0.863	0.309	83.67	22.61	14	36	398	44.0	9.29
Q16890	Tumor protein D53 OS=Homo sapiens GN=TPD52L1 PE=1 SV=1 - [TPD53_HUMAN]	37.25	1	7	7	22	0.193	0.280	1.137	0.310	63.26	37.25	10	22	204	22.4	5.62
Q96GN5	Cell division cycle-associated 7-like protein OS=Homo sapiens GN=CDCA7L PE=1 SV=2 - [CDA7L_HUMAN]	2.64	1	1	1	4	1.599	0.278	0.781	0.310	7.80	2.64	1	4	454	52.2	5.74
Q8NFX7	Syntaxin-binding protein 6 OS=Homo sapiens GN=STXBP6 PE=1 SV=2 - [STXB6_HUMAN]	4.29	1	1	1	3	0.417	1.244	6.089	0.310	7.05	4.29	2	3	210	23.5	9.04
Q9UPV0	Centrosomal protein of 164 kDa OS=Homo sapiens GN=CEP164 PE=1 SV=3 - [CE164_HUMAN]	1.85	1	2	3	3	0.775	0.248	0.390	0.311	8.06	1.85	3	3	1460	164.2	5.36
Q16799	Reticulon-1 OS=Homo sapiens GN=RTN1 PE=1 SV=1 - [RTN1_HUMAN]	15.72	1	8	8	43	0.519	0.461	2.187	0.312	117.51	15.72	13	43	776	83.6	4.69
Q5TBA9	Protein furry homolog OS=Homo sapiens GN=FRY PE=1 SV=1 - [FRY_HUMAN]	3.09	2	5	7	17	0.715	0.928	1.058	0.313	19.47	3.09	9	17	3013	338.7	5.99
Q8N6Y2	Leucine-rich repeat-containing protein 17 OS=Homo sapiens GN=LRRC17 PE=2 SV=1 - [LRC17_HUMAN]	7.26	1	3	3	10	0.159	24.723	4.984	0.314	27.70	7.26	5	10	441	51.8	8.24
P22105	Tenascin-X OS=Homo sapiens GN=TNXB PE=1 SV=3 - [TENX_HUMAN]	8.37	2	22	22	42	0.601	0.648	0.637	0.315	94.56	8.37	32	42	4289	464.0	5.34
A1L162	Glutamate-rich protein 2 OS=Homo sapiens GN=ERICH2 PE=2 SV=1 - [ERIC2_HUMAN]	7.05	1	1	1	1	0.738	0.601	0.665	0.318	2.44	7.05	1	1	156	17.7	3.98

O75648	Mitochondrial tRNA-specific 2-thiouridylase 1 OS=Homo sapiens GN=TRMU PE=1 SV=2 - [MTU1_HUMAN]	3.09	1	1	1	1	0.456	0.597	0.411	0.320	0.00	3.09	1	1	421	47.7	8.03
O75969	A-kinase anchor protein 3 OS=Homo sapiens GN=AKAP3 PE=1 SV=2 - [AKAP3_HUMAN]	1.17	1	1	1	1	0.496	0.698	0.903	0.320	2.23	1.17	1	1	853	94.7	6.18
Q8N1L9	Basic leucine zipper transcriptional factor ATF-like 2 OS=Homo sapiens GN=BATF2 PE=1 SV=1 - [BATF2_HUMAN]	9.12	1	1	1	1	0.350	0.433	0.520	0.321	0.00	9.12	1	1	274	29.4	7.55
Q9H9H5	MAP6 domain-containing protein 1 OS=Homo sapiens GN=MAP6D1 PE=1 SV=1 - [MA6D1_HUMAN]	6.03	1	1	1	1	0.892	0.205	0.184	0.322	0.00	6.03	1	1	199	21.0	10.07
Q8NHZ7	Methyl-CpG-binding domain protein 3-like 2 OS=Homo sapiens GN=MBD3L2 PE=2 SV=3 - [MB3L2_HUMAN]	8.17	1	1	1	2	0.306	1.272	0.977	0.324	2.04	8.17	1	2	208	23.0	11.53
Q4AC99	Probable inactive 1-aminocyclopropane-1-carboxylate synthase-like protein 2 OS=Homo sapiens GN=ACCSL PE=2 SV=3 - [1A1L2_HUMAN]	1.41	1	1	1	2	1.107	0.061	0.126	0.326	4.18	1.41	1	2	568	65.2	6.49
Q14CS0	UBX domain-containing protein 2B OS=Homo sapiens GN=UBXN2B PE=1 SV=1 - [UBX2B_HUMAN]	2.42	1	1	1	1	0.778	1.025	0.993	0.328	2.19	2.42	1	1	331	37.1	5.64
Q8N9W5	Dynein assembly factor 3, axonemal OS=Homo sapiens GN=DNAAF3 PE=1 SV=3 - [DAAF3_HUMAN]	2.03	1	1	1	1	1.555	1.131	1.203	0.330	1.98	2.03	1	1	541	59.4	5.90

Q96MR9	Zinc finger protein 560 OS=Homo sapiens GN=ZNF560 PE=2 SV=2 - [ZN560_HUMAN]	2.41	94	1	2	3		0.081	0.208	0.331	2.39	2.41	2	3	790	91.1	8.69
Q9NQX4	Unconventional myosin-Vc OS=Homo sapiens GN=MYO5C PE=1 SV=2 - [MYO5C_HUMAN]	1.95	1	3	4	13	0.422	0.655	1.073	0.333	7.11	1.95	4	13	1742	202.7	7.71
P08174	Complement decay-accelerating factor OS=Homo sapiens GN=CD55 PE=1 SV=4 - [DAF_HUMAN]	16.27	1	7	7	19	0.632	1.293	0.885	0.334	51.35	16.27	10	19	381	41.4	7.59
Q7Z614	Sorting nexin-20 OS=Homo sapiens GN=SNX20 PE=1 SV=1 - [SNX20_HUMAN]	3.16	1	1	1	2	0.665	0.799	1.716	0.335	3.04	3.16	1	2	316	36.2	8.65
Q9UFN0	Protein NipSnap homolog 3A OS=Homo sapiens GN=NIPSNAP3A PE=1 SV=2 - [NPS3A_HUMAN]	10.93	2	2	2	3	0.570	0.769	1.594	0.339	9.13	10.93	3	3	247	28.4	9.16
Q5T0U0	Coiled-coil domain-containing protein 122 OS=Homo sapiens GN=CCDC122 PE=1 SV=1 - [CC122_HUMAN]	6.23	1	2	2	6	0.641	0.353	0.329	0.342	9.10	6.23	3	6	273	32.2	7.01
P35520	Cystathionine beta-synthase OS=Homo sapiens GN=CBS PE=1 SV=2 - [CBS_HUMAN]	21.78	1	13	13	44	0.613	0.411	0.536	0.342	83.26	21.78	22	44	551	60.5	6.65
Q53QZ3	Rho GTPase-activating protein 15 OS=Homo sapiens GN=ARHGAP15 PE=1 SV=2 - [RHG15_HUMAN]	2.11	1	1	1	1	1.914	0.933	1.525	0.346	2.79	2.11	1	1	475	54.5	9.39
O43175	D-3-phosphoglycerate dehydrogenase OS=Homo sapiens GN=PHGDH PE=1 SV=4 - [SERA_HUMAN]	39.96	1	20	20	170	0.409	0.705	0.607	0.348	454.63	39.96	37	170	533	56.6	6.71

P13535	Myosin-8 OS=Homo sapiens GN=MYH8 PE=1 SV=3 - [MYH8_HUMAN]	4.70	1	2	10	85		1.481	1.607	0.349	156.65	4.70	12	85	1937	222.6	5.74
Q9BZZ2	Sialoadhesin OS=Homo sapiens GN=SIGLEC1 PE=1 SV=2 - [SN_HUMAN]	1.23	1	1	2	3	0.700	1.111	0.613	0.349	6.76	1.23	3	3	1709	182.5	6.62
P29373	Cellular retinoic acid-binding protein 2 OS=Homo sapiens GN=CRABP2 PE=1 SV=2 - [RABP2_HUMAN]	53.62	1	6	6	22	0.226	1.713	1.300	0.350	41.51	53.62	11	22	138	15.7	5.40
Q92485	Acid sphingomyelinase-like phosphodiesterase 3b OS=Homo sapiens GN=SMPDL3B PE=2 SV=2 - [ASM3B_HUMAN]	1.32	1	1	1	1	0.655	0.931	0.734	0.351	2.33	1.32	1	1	455	50.8	5.64
P01583	Interleukin-1 alpha OS=Homo sapiens GN=IL1A PE=1 SV=1 - [IL1A_HUMAN]	4.06	1	1	1	1	0.465	0.509	0.570	0.351	0.00	4.06	1	1	271	30.6	5.20
Q01650	Large neutral amino acids transporter small subunit 1 OS=Homo sapiens GN=SLC7A5 PE=1 SV=2 - [LAT1_HUMAN]	6.31	1	2	2	14	0.271	0.287	0.460	0.352	53.07	6.31	4	14	507	55.0	7.72
Q96A84	EMI domain-containing protein 1 OS=Homo sapiens GN=EMID1 PE=2 SV=1 - [EMID1_HUMAN]	6.80	1	1	1	2	0.356	0.612	0.351	0.353	4.82	6.80	1	2	441	45.3	9.10
Q16678	Cytochrome P450 1B1 OS=Homo sapiens GN=CYP1B1 PE=1 SV=2 - [CP1B1_HUMAN]	6.26	1	3	3	16	0.474	0.162	0.345	0.354	43.05	6.26	6	16	543	60.8	8.98
Q9NX94	WW domain binding protein 1-like OS=Homo sapiens GN=WBP1L PE=2 SV=2 - [WBP1L_HUMAN]	7.31	1	2	2	2	0.719	1.362	2.292	0.356	5.83	7.31	2	2	342	37.8	5.19

Q99728	BRCA1-associated RING domain protein 1 OS=Homo sapiens GN=BARD1 PE=1 SV=2 - [BARD1_HUMAN	1.80	1	1	1	1	0.738	1.021	1.155	0.358	2.31	1.80	1	1	777	86.6	8.72
Q9UI08	Ena/VASP-like protein OS=Homo sapiens GN=EVL PE=1 SV=2 - [EVL_HUMAN]	5.05	1	2	2	4	0.666	0.571	0.799	0.358	4.61	5.05	3	4	416	44.6	8.84
O95833	Chloride intracellular channel protein 3 OS=Homo sapiens GN=CLIC3 PE=1 SV=2 - [CLIC3_HUMAN]	42.80	1	9	10	23	0.452	0.464	0.730	0.364	45.57	42.80	14	23	236	26.6	6.43
Q8WWQ2	Inactive heparanase-2 OS=Homo sapiens GN=HPSE2 PE=1 SV=3 - [HPSE2_HUMAN]	2.36	1	1	1	2	0.607	1.087	1.878	0.365	0.00	2.36	1	2	592	66.6	9.94
Q99674	Cell growth regulator with EF hand domain protein 1 OS=Homo sapiens GN=CGREF1 PE=2 SV=2 - [CGRE1_HUMAN]	5.32	2	1	2	4	0.330	0.776	0.885	0.367	9.32	5.32	2	4	301	31.9	4.42
Q6ZRI0	Otogelin OS=Homo sapiens GN=OTOG PE=1 SV=3 - [OTOG_HUMAN]	0.41	1	2	2	2	0.416	0.573	0.638	0.368	4.65	0.41	2	2	2925	314.6	5.91
P02461	Collagen alpha-1(III) chain OS=Homo sapiens GN=COL3A1 PE=1 SV=4 - [CO3A1_HUMA N]	47.61	1	50	52	411	0.325	0.736	0.737	0.368	1045.31	47.61	86	411	1466	138.5	6.61
P22557	5-aminolevulinate synthase, erythroid-specific, mitochondrial OS=Homo sapiens GN=ALAS2 PE=1 SV=2 - [HEM0_HUMAN]	2.90	1	1	2	2	1.314	0.201	0.250	0.368	2.31	2.90	2	2	587	64.6	8.12
O75154	Rab11 family-interacting protein 3 OS=Homo sapiens GN=RAB11FIP3 PE=1 SV=1 - [RFIP3_HUMAN]	4.63	1	1	2	3	1.904	1.422	2.253	0.369	3.82	4.63	2	3	756	82.4	4.51

O15083	ERC protein 2 OS=Homo sapiens GN=ERC2 PE=1 SV=3 - [ERC2_HUMAN]	11.18	1	2	14	66	0.418	0.651	0.615	0.369	114.48	11.18	21	66	957	110.5	6.99
Q9Y446	Plakophilin-3 OS=Homo sapiens GN=PKP3 PE=1 SV=1 - [PKP3_HUMAN]	2.01	1	1	1	2	0.457	0.379		0.370	0.00	2.01	1	2	797	87.0	9.32
Q96MR6	Cilia- and flagella-associated protein 57 OS=Homo sapiens GN=CFAP57 PE=1 SV=3 - [CFA57_HUMAN	0.56	1	1	1	2	0.598	1.139	1.582	0.371	4.93	0.56	1	2	1250	144.9	5.80
Q3C1V8	Brain-specific homeobox protein homolog OS=Homo sapiens GN=BSX PE=2 SV=2 - [BSH_HUMAN]	15.45	1	2	2	3	0.767	0.709	1.005	0.371	6.36	15.45	2	3	233	25.9	7.50
P54687	Branched-chain-amino-acid aminotransferase, cytosolic OS=Homo sapiens GN=BCAT1 PE=1 SV=3 - [BCAT1_HUMAN]	25.91	1	9	9	25	0.362	0.749	0.945	0.373	63.79	25.91	12	25	386	42.9	5.30
P07585	Decorin OS=Homo sapiens GN=DCN PE=1 SV=1 - [PGS2_HUMAN]	33.43	1	12	13	97	0.428	0.582	0.626	0.375	228.03	33.43	24	97	359	39.7	8.54
Q8IYE0	Coiled-coil domain-containing protein 146 OS=Homo sapiens GN=CCDC146 PE=2 SV=2 - [CC146_HUMAN]	3.56	1	2	4	6	0.468	0.747	0.886	0.375	9.70	3.56	4	6	955	112.7	8.48
Q16853	Membrane primary amine oxidase OS=Homo sapiens GN=AOC3 PE=1 SV=3 - [AOC3_HUMAN]	5.90	2	5	5	17	0.145	0.310	0.870	0.376	41.82	5.90	10	17	763	84.6	6.52
P13645	Keratin, type I cytoskeletal 10 OS=Homo sapiens GN=KRT10 PE=1 SV=6 - [K1C10_HUMAN]	38.70	10	19	25	98	0.869	2.779	0.809	0.378	222.37	38.70	34	98	584	58.8	5.21

Q9UKS7	Zinc finger protein Helios OS=Homo sapiens GN=IKZF2 PE=1 SV=2 - [IKZF2_HUMAN]	1.90	1	1	1	1	0.434	0.729	0.733	0.379	0.00	1.90	1	1	526	59.5	6.77
Q76M96	Coiled-coil domain-containing protein 80 OS=Homo sapiens GN=CCDC80 PE=1 SV=1 - [CCD80_HUMAN]	27.79	1	17	17	54	0.309	0.860	0.705	0.381	128.16	27.79	29	54	950	108.1	9.72
A6NCE7	Microtubule-associated proteins 1A/1B light chain 3 beta 2 OS=Homo sapiens GN=MAP1LC3B 2 PE=2 SV=1 - [MP3B2_HUMA	24.00	2	1	4	9	0.408	0.659	1.346	0.383	10.97	24.00	7	9	125	14.6	8.68
Q15562	Transcriptional enhancer factor TEF-4 OS=Homo sapiens GN=TEAD2 PE=1 SV=2 - [TEAD2_HUMAN]	8.50	4	3	3	8	0.443	0.739	0.688	0.384	6.29	8.50	3	8	447	49.2	6.47
P51911	Calponin-1 OS=Homo sapiens GN=CNN1 PE=1 SV=2 - [CNN1_HUMAN]	63.97	1	19	23	225	0.577	1.244	2.011	0.385	504.51	63.97	35	225	297	33.1	9.07
Q9BUZ4	TNF receptor-associated factor 4 OS=Homo sapiens GN=TRAF4 PE=1 SV=1 - [TRAF4_HUMAN	1.49	1	1	1	11	0.569	0.819	2.010	0.386	22.92	1.49	2	11	470	53.5	8.15
Q8NHP8	Putative phospholipase B-like 2 OS=Homo sapiens GN=PLBD2 PE=1 SV=2 - [PLBL2_HUMAN]	20.20	1	10	10	37	0.507	1.188	1.077	0.388	65.39	20.20	17	37	589	65.4	6.80
Q14123	Calcium/calmodulin-dependent 3',5'-cyclic nucleotide phosphodiesterase 1C OS=Homo sapiens GN=PDE1C PE=1 SV=1 - [PDE1C_HUMAN]	4.51	1	2	2	2	1.191	0.476	0.765	0.389	2.74	4.51	2	2	709	80.7	8.82

Q16611	Bcl-2 homologous antagonist/killer OS=Homo sapiens GN=BAK1 PE=1 SV=1 - [BAK_HUMAN]	21.33	1	2	2	7	1.225	0.809	0.835	0.390	16.05	21.33	3	7	211	23.4	6.01
P35555	Fibrillin-1 OS=Homo sapiens GN=FBN1 PE=1 SV=3 - [FBN1_HUMAN]	5.89	1	15	17	52	0.393	0.586	0.560	0.392	110.11	5.89	25	52	2871	312.0	4.93
Q9P2N4	A disintegrin and metalloproteinase with thrombospondin motifs 9 OS=Homo sapiens GN=ADAMTS9 PE=1 SV=4 - [ATS9_HUMAN]	0.88	1	1	1	1	1.918	0.314	0.646	0.392	2.46	0.88	1	1	1935	216.4	7.77
P51805	Plexin-A3 OS=Homo sapiens GN=PLXNA3 PE=1 SV=2 - [PLXA3_HUMAN]	3.42	1	2	7	20	0.725	0.683	1.181	0.393	38.13	3.42	12	20	1871	207.6	7.31
Q8NGX8	Olfactory receptor 6Y1 OS=Homo sapiens GN=OR6Y1 PE=2 SV=1 - [OR6Y1_HUMAN]	2.77	1	1	1	1		0.431		0.394	3.29	2.77	1	1	325	36.6	8.06
P21439	Multidrug resistance protein 3 OS=Homo sapiens GN=ABCB4 PE=1 SV=2 - [MDR3_HUMAN]	0.62	1	1	1	2	2.140	0.762	0.903	0.395	4.73	0.62	1	2	1286	141.4	8.48
Q86VW0	SEC14 domain and spectrin repeat-containing protein 1 OS=Homo sapiens GN=SESTD1 PE=1 SV=2 - [SESD1_HUMAN]	2.73	1	2	2	3	1.335	0.107	0.208	0.396	4.61	2.73	2	3	696	79.3	5.10
P43403	Tyrosine-protein kinase ZAP-70 OS=Homo sapiens GN=ZAP70 PE=1 SV=1 - [ZAP70_HUMAN]	6.95	1	2	2	2	0.529	1.374	2.838	0.398	5.51	6.95	2	2	619	69.8	7.69
Q9BT73	Proteasome assembly chaperone 3 OS=Homo sapiens GN=PSMG3 PE=1 SV=1 - [PSMG3_HUMAN]	12.30	1	1	1	1	2.507	2.020		0.398	5.03	12.30	1	1	122	13.1	7.88

Q13449	Limbic system-associated membrane protein OS=Homo sapiens GN=LSAMP PE=1 SV=2 - [LSAMP_HUMAN]	13.31	1	4	4	11	0.544	1.513	0.811	0.398	29.33	13.31	6	11	338	37.4	6.98
Q6X4U4	Sclerostin domain-containing protein 1 OS=Homo sapiens GN=SOSTDC1 PE=1 SV=2 - [SOSD1_HUMAN]	6.80	1	1	1	1	0.706	1.366	0.983	0.400	2.17	6.80	1	1	206	23.3	9.74
Q9UPN9	E3 ubiquitin-protein ligase TRIM33 OS=Homo sapiens GN=TRIM33 PE=1 SV=3 - [TRI33_HUMAN]	0.89	1	1	1	1	1.468			0.404	0.00	0.89	1	1	1127	122.5	6.67
P05997	Collagen alpha-2(V) chain OS=Homo sapiens GN=COL5A2 PE=1 SV=3 - [CO5A2_HUMAN]	21.28	1	20	22	83	0.759	0.724	0.667	0.405	191.54	21.28	37	83	1499	144.8	6.46
P21291	Cysteine and glycine-rich protein 1 OS=Homo sapiens GN=CSRP1 PE=1 SV=3 - [CSRP1_HUMAN]	54.40	1	11	11	140	1.508	1.222	1.789	0.407	424.48	54.40	20	140	193	20.6	8.57
Q96CG8	Collagen triple helix repeat-containing protein 1 OS=Homo sapiens GN=CTHRC1 PE=1 SV=1 - [CTHR1_HUMAN]	32.10	1	6	6	27	0.521	0.688	0.354	0.407	79.14	32.10	10	27	243	26.2	7.99
Q08431	Lactadherin OS=Homo sapiens GN=MFGE8 PE=1 SV=2 - [MFGM_HUMAN]	18.60	1	7	7	18	0.563	0.861	1.793	0.409	40.39	18.60	10	18	387	43.1	8.15
Q9UNW1	Multiple inositol polyphosphate phosphatase 1 OS=Homo sapiens GN=MINPP1 PE=1 SV=1 - [MINP1_HUMAN]	10.06	1	5	5	11	0.637	0.412	0.510	0.410	32.55	10.06	7	11	487	55.0	7.81
O75051	Plexin-A2 OS=Homo sapiens GN=PLXNA2 PE=1 SV=4 - [PLXA2_HUMAN]	3.59	1	1	7	21	0.352	0.966	0.644	0.410	34.42	3.59	11	21	1894	211.0	6.48

P02452	Collagen alpha-1(I) chain OS=Homo sapiens GN=COL1A1 PE=1 SV=5 - [CO1A1_HUMAN]	59.22	2	59	62	389	0.325	0.566	0.611	0.411	1027.67	59.22	103	389	1464	138.9	5.80
Q9H788	SH2 domain-containing protein 4A OS=Homo sapiens GN=SH2D4A PE=1 SV=1 - [SH24A_HUMAN]	27.09	1	13	13	39	0.585	0.552	0.783	0.411	84.89	27.09	17	39	454	52.7	8.06
Q8N6D5	Ankyrin repeat domain-containing protein 29 OS=Homo sapiens GN=ANKRD29 PE=2 SV=2 - [ANR29_HUMAN]	2.99	1	1	1	2	1.054	0.127	0.285	0.411	2.34	2.99	1	2	301	32.4	9.41
P02792	Ferritin light chain OS=Homo sapiens GN=FTL PE=1 SV=2 - [FRIL_HUMAN]	38.29	1	6	7	32	0.487	0.684	0.555	0.412	84.02	38.29	11	32	175	20.0	5.78
Q8IVF2	Protein AHNAK2 OS=Homo sapiens GN=AHNAK2 PE=1 SV=2 - [AHNK2_HUMAN]	46.66	1	117	124	424	0.921	1.908	1.567	0.412	1016.23	46.66	200	424	5795	616.2	5.36
O60602	Toll-like receptor 5 OS=Homo sapiens GN=TLR5 PE=1 SV=4 - [TLR5_HUMAN]	0.93	1	1	1	2	3.942	1.075	1.043	0.412	2.15	0.93	1	2	858	97.8	6.68
Q9BV73	Centrosome-associated protein CEP250 OS=Homo sapiens GN=CEP250 PE=1 SV=2 - [CP250_HUMAN	1.47	1	1	5	16	1.139	0.985	0.650	0.413	27.51	1.47	6	16	2442	281.0	5.02
O15460	Prolyl 4-hydroxylase subunit alpha-2 OS=Homo sapiens GN=P4HA2 PE=1 SV=1 - [P4HA2_HUMAN]	43.18	1	23	23	131	0.477	0.685	0.709	0.414	348.29	43.18	38	131	535	60.9	5.71
P43308	Translocon-associated protein subunit beta OS=Homo sapiens GN=SSR2 PE=1 SV=1 - [SSRB_HUMAN]	3.28	1	1	1	1	0.601	0.649	0.240	0.414	1.92	3.28	1	1	183	20.1	8.35

O75781	Paralemmin-1 OS=Homo sapiens GN=PALM PE=1 SV=2 - [PALM_HUMAN]	35.66	1	12	13	62	0.350	0.579	0.536	0.415	151.75	35.66	20	62	387	42.1	5.00
P35527	Keratin, type I cytoskeletal 9 OS=Homo sapiens GN=KRT9 PE=1 SV=3 - [K1C9_HUMAN]	23.60	2	13	15	49	0.950	2.470	0.395	0.415	97.92	23.60	21	49	623	62.0	5.24
P41250	Glycine--tRNA ligase OS=Homo sapiens GN=GARS PE=1 SV=3 - [SYG_HUMAN]	46.14	1	33	34	127	0.533	0.612	0.730	0.416	275.02	46.14	55	127	739	83.1	7.03
Q9UGJ0	5'-AMP-activated protein kinase subunit gamma-2 OS=Homo sapiens GN=PRKAG2 PE=1 SV=1 - [AAKG2_HUMAN]	4.39	1	1	3	8	0.693	0.609	0.991	0.417	14.21	4.39	6	8	569	63.0	9.35
P19320	Vascular cell adhesion protein 1 OS=Homo sapiens GN=VCAM1 PE=1 SV=1 - [VCAM1_HUMAN]	35.99	1	26	27	121	1.279	0.321	0.239	0.418	288.99	35.99	47	121	739	81.2	5.22
O43592	Exportin-T OS=Homo sapiens GN=XPOT PE=1 SV=2 - [XPOT_HUMAN]	10.60	1	9	9	27	0.366	0.496	0.462	0.418	55.89	10.60	16	27	962	109.9	5.39
P78330	Phosphoserine phosphatase OS=Homo sapiens GN=PSPH PE=1 SV=2 - [SERB_HUMAN]	20.00	1	4	4	12	0.708	1.179	1.366	0.419	26.37	20.00	8	12	225	25.0	5.69
Q96RS0	Trimethylguanosine synthase OS=Homo sapiens GN=TGS1 PE=1 SV=3 - [TGS1_HUMAN]	0.70	1	1	1	4	0.414	2.079	3.087	0.419	8.19	0.70	1	4	853	96.6	4.94
Q5K651	Sterile alpha motif domain-containing protein 9 OS=Homo sapiens GN=SAMD9 PE=1 SV=1 - [SAMD9_HUMAN]	2.08	1	3	4	6	1.923	1.407	0.873	0.420	18.45	2.08	4	6	1589	184.2	7.83

P02751	Fibronectin OS=Homo sapiens GN=FN1 PE=1 SV=4 - [FINC_HUMAN]	34.07	1	60	60	428	0.267	0.799	1.020	0.420	1115.25	34.07	104	428	2386	262.5	5.71
P49918	Cyclin-dependent kinase inhibitor 1C OS=Homo sapiens GN=CDKN1C PE=1 SV=1 - [CDN1C_HUMAN]	5.06	1	1	1	1	2.232	0.109	0.458	0.421	2.57	5.06	1	1	316	32.2	5.52
O14880	Microsomal glutathione S-transferase 3 OS=Homo sapiens GN=MGST3 PE=1 SV=1 - [MGST3_HUMAN]	25.00	1	2	2	8	0.496	0.260	0.288	0.422	27.28	25.00	3	8	152	16.5	9.38
Q6IMI6	Sulfotransferase 1C3 OS=Homo sapiens GN=SULT1C3 PE=1 SV=1 - [ST1C3_HUMAN	5.59	1	1	1	1	1.120	0.688	1.043	0.422	0.00	5.59	1	1	304	35.9	6.92
Q6ZNJ1	Neurobeachin-like protein 2 OS=Homo sapiens GN=NBEAL2 PE=1 SV=2 - [NBEL2_HUMAN]	0.73	1	1	2	3	0.824	1.509	1.691	0.423	2.39	0.73	2	3	2754	302.3	6.38
P35237	Serpin B6 OS=Homo sapiens GN=SERPINB6 PE=1 SV=3 - [SPB6_HUMAN]	51.33	1	18	19	116	0.857	1.124	1.019	0.423	224.84	51.33	32	116	376	42.6	5.27
Q96A00	Protein phosphatase 1 regulatory subunit 14A OS=Homo sapiens GN=PPP1R14A PE=1 SV=1 - [PP14A_HUMAN]	11.56	1	1	2	8	0.471	5.514	1.902	0.423	14.75	11.56	3	8	147	16.7	9.38
A0PK00	Transmembrane protein 120B OS=Homo sapiens GN=TMEM120B PE=2 SV=1 - [T120B_HUMAN]	6.78	1	1	2	11	0.670	1.195	0.857	0.424	48.87	6.78	3	11	339	40.2	8.90
Q99470	Stromal cell-derived factor 2 OS=Homo sapiens GN=SDF2 PE=1 SV=2 - [SDF2_HUMAN]	24.64	1	3	3	12	1.896	0.880	0.778	0.424	24.27	24.64	5	12	211	23.0	7.33
P36871	Phosphoglucomutase-1 OS=Homo sapiens GN=PGM1 PE=1 SV=3 - [PGM1_HUMAN]	57.30	1	32	32	280	0.747	0.837	1.081	0.426	740.98	57.30	55	280	562	61.4	6.76

O95837	Guanine nucleotide-binding protein subunit alpha-14 OS=Homo sapiens GN=GNA14 PE=2 SV=1 - [GNA14_HUMAN]	22.82	1	2	7	22	0.529	1.005	1.962	0.426	55.92	22.82	12	22	355	41.5	6.07
Q8NE00	Transmembrane protein 104 OS=Homo sapiens GN=TMEM104 PE=1 SV=2 - [TM104_HUMAN]	2.82	1	1	1	1	0.384	0.385	0.560	0.426	2.13	2.82	1	1	496	55.6	7.20
A6PW82	Putative uncharacterized protein CXorf30 OS=Homo sapiens GN=CXorf30 PE=2 SV=2 - [CX030_HUMAN]	2.21	1	1	1	2	0.786	0.125	0.160	0.427	3.24	2.21	1	2	633	72.0	6.09
Q92769	Histone deacetylase 2 OS=Homo sapiens GN=HDAC2 PE=1 SV=2 - [HDAC2_HUMAN]	15.37	1	3	6	24	1.539	1.127	1.556	0.429	50.52	15.37	10	24	488	55.3	5.91
P09936	Ubiquitin carboxyl-terminal hydrolase isozyme L1 OS=Homo sapiens GN=UCHL1 PE=1 SV=2 - [UCHL1_HUMAN]	69.96	2	16	17	370	0.666	0.704	0.977	0.430	928.84	69.96	30	370	223	24.8	5.48
Q5XUX0	F-box only protein 31 OS=Homo sapiens GN=FBXO31 PE=1 SV=2 - [FBX31_HUMAN]	1.86	1	1	1	32	0.596	0.580	0.924	0.430	63.50	1.86	1	32	539	60.6	7.02
Q6ZUB1	Spermatogenesis-associated protein 31E1 OS=Homo sapiens GN=SPATA31E1 PE=2 SV=2 - [S31E1_HUMAN]	0.76	1	1	1	1	0.903	0.494	1.047	0.431	2.94	0.76	1	1	1445	157.0	9.10
Q07075	Glutamyl aminopeptidase OS=Homo sapiens GN=ENPEP PE=1 SV=3 - [AMPE_HUMAN]	1.15	1	1	1	2	0.167	0.693	0.560	0.431	2.23	1.15	2	2	957	109.2	5.47
Q68CZ1	Protein fantom OS=Homo sapiens GN=RPGRIP1L PE=1 SV=2 - [FTM_HUMAN]	2.66	1	2	4	9	1.058	1.584	0.996	0.432	18.96	2.66	4	9	1315	151.1	5.35

Q68DU8	BTB/POZ domain-containing protein KCTD16 OS=Homo sapiens GN=KCTD16 PE=2 SV=1 - [KCD16_HUMAN]	2.34	1	1	1	1	0.823	0.294	0.695	0.432	0.00	2.34	1	1	428	49.1	8.09
Q8N474	Secreted frizzled-related protein 1 OS=Homo sapiens GN=SFRP1 PE=1 SV=1 - [SFRP1_HUMAN	2.55	1	1	1	2	0.234	1.817	0.551	0.433	5.69	2.55	2	2	314	35.4	8.85
Q9NUB1	Acetyl-coenzyme A synthetase 2-like, mitochondrial OS=Homo sapiens GN=ACSS1 PE=1 SV=2 - [ACS2L_HUMAN]	1.16	1	1	1	1	0.698	0.608	0.384	0.434	0.00	1.16	1	1	689	74.8	7.11
Q96Q07	BTB/POZ domain-containing protein 9 OS=Homo sapiens GN=BTBD9 PE=2 SV=2 - [BTBD9_HUMAN]	7.03	1	3	3	3	0.457	0.368	0.254	0.435	1.81	7.03	3	3	612	69.1	5.77
Q7Z736	Pleckstrin homology domain-containing family H member 3 OS=Homo sapiens GN=PLEKHH3 PE=1 SV=2 - [PKHH3_HUMAN]	1.13	1	1	1	2	1.245	0.293	0.341	0.435	0.00	1.13	1	2	793	85.3	7.83
Q6NZY7	Cdc42 effector protein 5 OS=Homo sapiens GN=CDC42EP5 PE=1 SV=1 - [BORG3_HUMAN]	5.41	1	1	1	2	0.407	0.544	1.871	0.435	4.05	5.41	1	2	148	15.2	7.52
P42771	Cyclin-dependent kinase inhibitor 2A, isoforms 1/2/3 OS=Homo sapiens GN=CDKN2A PE=1 SV=2 - [CD2A1_HUMAN]	15.38	2	2	2	4	0.657	0.803	5.510	0.438	15.83	15.38	4	4	156	16.5	5.81
Q7L5D6	Golgi to ER traffic protein 4 homolog OS=Homo sapiens GN=GET4 PE=1 SV=1 - [GET4_HUMAN]	4.59	1	1	1	3	0.911	0.538	0.552	0.439	11.24	4.59	2	3	327	36.5	5.41

Q13683	Integrin alpha-7 OS=Homo sapiens GN=ITGA7 PE=1 SV=3 - [ITA7_HUMAN]	13.55	1	16	16	43	0.192	3.336	2.086	0.439	85.72	13.55	27	43	1181	128.9	5.73
Q8IZV5	Retinol dehydrogenase 10 OS=Homo sapiens GN=RDH10 PE=1 SV=1 - [RDH10_HUMAN]	23.75	1	7	7	22	0.734	0.598	0.806	0.439	59.16	23.75	13	22	341	38.1	7.40
P48547	Potassium voltage-gated channel subfamily C member 1 OS=Homo sapiens GN=KCNC1 PE=2 SV=1 - [KCNC1_HUMAN]	2.74	1	1	1	13	0.543	0.505	0.491	0.441	5.97	2.74	1	13	511	57.9	6.58
P08727	Keratin, type I cytoskeletal 19 OS=Homo sapiens GN=KRT19 PE=1 SV=4 - [K1C19_HUMAN]	48.50	6	10	18	58	0.879	2.442	1.075	0.442	121.60	48.50	28	58	400	44.1	5.14
P10070	Zinc finger protein GLI2 OS=Homo sapiens GN=GLI2 PE=1 SV=4 - [GLI2_HUMAN]	4.22	4	3	4	5	1.022	1.198	1.372	0.442	5.15	4.22	5	5	1586	167.7	7.33
O60882	Matrix metalloproteinase-20 OS=Homo sapiens GN=MMP20 PE=1 SV=3 - [MMP20_HUMAN]	2.07	1	1	1	2	1.060	0.970	0.606	0.442	4.87	2.07	1	2	483	54.4	8.87
Q9HC77	Centromere protein J OS=Homo sapiens GN=CENPJ PE=1 SV=2 - [CENPJ_HUMAN]	1.35	1	1	2	2	1.014	1.104	1.097	0.442	0.00	1.35	2	2	1338	152.9	6.61
Q8N608	Inactive dipeptidyl peptidase 10 OS=Homo sapiens GN=DPP10 PE=1 SV=2 - [DPP10_HUMAN]	2.01	1	2	2	2	1.053	1.186	0.351	0.442	3.94	2.01	2	2	796	90.8	6.57
P00325	Alcohol dehydrogenase 1B OS=Homo sapiens GN=ADH1B PE=1 SV=2 - [ADH1B_HUMAN]	27.73	3	13	13	58	0.405	0.737	0.329	0.443	130.76	27.73	23	58	375	39.8	8.29

O00423	Echinoderm microtubule-associated protein-like 1 OS=Homo sapiens GN=EML1 PE=1 SV=3 - [EMAL1_HUMAN]	34.97	1	28	28	105	1.330	0.968	1.711	0.444	235.11	34.97	45	105	815	89.8	7.06
Q9NT68	Teneurin-2 OS=Homo sapiens GN=TENM2 PE=1 SV=3 - [TEN2_HUMAN]	0.40	1	1	1	1	0.673	0.653	0.737	0.444	2.52	0.40	1	1	2774	307.6	6.68
Q9BQ52	Zinc phosphodiesterase ELAC protein 2 OS=Homo sapiens GN=ELAC2 PE=1 SV=2 - [RNZ2_HUMAN]	1.33	1	1	1	1	2.398	1.465	1.179	0.445	3.03	1.33	1	1	826	92.2	7.90
P17302	Gap junction alpha-1 protein OS=Homo sapiens GN=GJA1 PE=1 SV=2 - [CXA1_HUMAN]	5.76	1	2	2	5	0.612	0.957	0.748	0.446	16.21	5.76	3	5	382	43.0	8.76
Q3SY69	Mitochondrial 10-formyltetrahydrofolate dehydrogenase OS=Homo sapiens GN=ALDH1L2 PE=1 SV=2 - [AL1L2_HUMAN]	36.51	1	28	33	131	0.469	0.630	0.702	0.446	317.39	36.51	52	131	923	101.7	6.52
P30530	Tyrosine-protein kinase receptor UFO OS=Homo sapiens GN=AXL PE=1 SV=3 - [UFO_HUMAN]	1.90	1	1	1	3	1.002	0.607	1.152	0.447	9.25	1.90	2	3	894	98.3	5.43
Q3B820	Protein FAM161A OS=Homo sapiens GN=FAM161A PE=1 SV=2 - [F161A_HUMAN]	2.27	1	2	2	2	0.408	0.573	0.803	0.449	4.03	2.27	2	2	660	76.7	8.03
Q96PY5	Formin-like protein 2 OS=Homo sapiens GN=FMNL2 PE=1 SV=3 - [FMNL2_HUMAN]	3.96	1	2	4	8	0.859	0.402	0.578	0.449	15.48	3.96	6	8	1086	123.2	7.40
P35580	Myosin-10 OS=Homo sapiens GN=MYH10 PE=1 SV=3 - [MYH10_HUMAN]	64.32	2	120	160	1926	0.431	0.640	0.843	0.449	5387.89	64.32	258	1926	1976	228.9	5.54

Q01995	Transgelin OS=Homo sapiens GN=TAGLN PE=1 SV=4 - [TAGL_HUMAN]	77.61	1	19	19	851	0.575	**1.507**	2.652	0.451	2095.24	77.61	36	851	201	22.6	8.84
Q96BW5	Phosphotriesterase-related protein OS=Homo sapiens GN=PTER PE=1 SV=1 - [PTER_HUMAN]	4.87	1	2	2	4	0.547	0.968	0.610	0.452	10.18	4.87	3	4	349	39.0	6.52
Q3B726	DNA-directed RNA polymerase I subunit RPA43 OS=Homo sapiens GN=TWISTNB PE=1 SV=1 - [RPA43_HUMAN]	2.07	1	1	1	3	0.577	0.527	0.643	0.454	7.30	2.07	2	3	338	37.4	6.98
P15531	Nucleoside diphosphate kinase A OS=Homo sapiens GN=NME1 PE=1 SV=1 - [NDKA_HUMAN]	57.89	1	4	9	246	0.726	0.633	0.717	0.455	478.19	57.89	17	246	152	17.1	6.19
Q9UKW4	Guanine nucleotide exchange factor VAV3 OS=Homo sapiens GN=VAV3 PE=1 SV=1 - [VAV3_HUMAN]	1.77	2	1	2	4	0.414		1.311	0.455	6.63	1.77	2	4	847	97.7	7.05
O75911	Short-chain dehydrogenase/reductase 3 OS=Homo sapiens GN=DHRS3 PE=1 SV=2 - [DHRS3_HUMAN]	23.18	1	6	6	26	0.480	1.590	1.181	0.455	51.06	23.18	11	26	302	33.5	8.84
P02795	Metallothionein-2 OS=Homo sapiens GN=MT2A PE=1 SV=1 - [MT2_HUMAN]	13.11	5	1	1	3	0.416	0.160	0.410	0.459	7.96	13.11	2	3	61	6.0	7.83
P32322	Pyrroline-5-carboxylate reductase 1, mitochondrial OS=Homo sapiens GN=PYCR1 PE=1 SV=2 - [P5CR1_HUMAN]	40.44	1	7	8	22	0.435	0.788	0.640	0.460	79.31	40.44	13	22	319	33.3	7.61

P29350	Tyrosine-protein phosphatase non-receptor type 6 OS=Homo sapiens GN=PTPN6 PE=1 SV=1 - [PTN6_HUMAN]	5.88	1	2	3	5	1.050	0.205	0.291	0.461	0.00	5.88	4	5	595	67.5	7.78
A2RUC4	tRNA wybutosine-synthesizing protein 5 OS=Homo sapiens GN=TYW5 PE=1 SV=1 - [TYW5_HUMAN]	12.70	1	2	2	4	1.203	0.847	1.158	0.462	2.40	12.70	2	4	315	36.5	7.55
Q99490	Arf-GAP with GTPase, ANK repeat and PH domain-containing protein 2 OS=Homo sapiens GN=AGAP2 PE=1 SV=2 - [AGAP2_HUMAN]	8.05	1	3	4	9	0.833	0.983	0.738	0.463	9.77	8.05	4	9	1192	124.6	9.89
Q9UKT9	Zinc finger protein Aiolos OS=Homo sapiens GN=IKZF3 PE=1 SV=2 - [IKZF3_HUMAN]	3.54	2	1	2	2	0.404	1.608	3.089	0.464	3.51	3.54	2	2	509	58.0	6.55
Q9BXU7	Ubiquitin carboxyl-terminal hydrolase 26 OS=Homo sapiens GN=USP26 PE=1 SV=1 - [UBP26_HUMAN]	0.99	1	1	1	1	1.581	0.150	0.272	0.465	2.36	0.99	1	1	913	104.0	8.70
Q8TEQ8	GPI ethanolamine phosphate transferase 3 OS=Homo sapiens GN=PIGO PE=1 SV=3 - [PIGO_HUMAN]	2.11	1	2	2	6	0.878	1.227	0.923	0.465	12.95	2.11	3	6	1089	118.6	8.12
P08123	Collagen alpha-2(I) chain OS=Homo sapiens GN=COL1A2 PE=1 SV=7 - [CO1A2_HUMAN]	66.62	1	61	61	431	0.406	0.686	0.595	0.465	1085.92	66.62	99	431	1366	129.2	8.95
P24390	ER lumen protein-retaining receptor 1 OS=Homo sapiens GN=KDELR1 PE=1 SV=1 - [ERD21_HUMAN]	3.77	2	1	1	2	0.352	0.317	0.385	0.465	2.81	3.77	2	2	212	24.5	8.62

Q6ZQY3	Acidic amino acid decarboxylase GADL1 OS=Homo sapiens GN=GADL1 PE=1 SV=4 - [GADL1_HUMAN]	1.54	1	1	1	2	1.048	0.647	0.627	0.466	4.43	1.54	1	2	521	59.2	6.37
B0FP48	Uroplakin-3b-like protein OS=Homo sapiens GN=UPK3BL PE=2 SV=1 - [UPK3L_HUMAN]	5.32	1	1	1	1	1.174	2.006	0.909	0.470	0.00	5.32	1	1	263	28.4	8.21
Q969R5	Lethal(3)malignant brain tumor-like protein 2 OS=Homo sapiens GN=L3MBTL2 PE=1 SV=1 - [LMBL2_HUMAN	1.70	1	1	1	1	1.202	0.668	1.230	0.472	3.38	1.70	1	1	705	79.1	6.84
H3BS89	Transmembrane protein 178B OS=Homo sapiens GN=TMEM178B PE=2 SV=1 - [T178B_HUMAN]	4.42	1	1	1	1	0.724	0.699	0.544	0.472	3.29	4.42	1	1	294	33.4	9.25
O00391	Sulfhydryl oxidase 1 OS=Homo sapiens GN=QSOX1 PE=1 SV=3 - [QSOX1_HUMAN]	25.70	1	18	18	44	0.562	1.186	0.733	0.472	107.79	25.70	29	44	747	82.5	8.92
P20908	Collagen alpha-1(V) chain OS=Homo sapiens GN=COL5A1 PE=1 SV=3 - [CO5A1_HUMAN]	16.16	2	19	20	56	0.556	0.785	0.705	0.472	130.40	16.16	26	56	1838	183.4	5.06
Q9C019	Tripartite motif-containing protein 15 OS=Homo sapiens GN=TRIM15 PE=2 SV=1 - [TRI15_HUMAN]	2.15	1	1	1	2	0.830	0.912	1.400	0.473	2.58	2.15	1	2	465	52.1	5.57
Q8NFJ5	Retinoic acid-induced protein 3 OS=Homo sapiens GN=GPRC5A PE=1 SV=2 - [RAI3_HUMAN]	7.56	1	2	2	5	3.569	0.526	0.860	0.473	11.52	7.56	3	5	357	40.2	8.15
Q9UGU0	Transcription factor 20 OS=Homo sapiens GN=TCF20 PE=1 SV=3 - [TCF20_HUMAN]	1.22	1	2	2	3	1.349	0.674	0.606	0.474	3.26	1.22	3	3	1960	211.6	9.04

Q9NYQ8	Protocadherin Fat 2 OS=Homo sapiens GN=FAT2 PE=1 SV=2 - [FAT2_HUMAN]	1.33	1	3	3	6	3.178	1.418	1.659	0.474	8.76	1.33	3	6	4349	479.0	5.16
O75023	Leukocyte immunoglobulin-like receptor subfamily B member 5 OS=Homo sapiens GN=LILRB5 PE=1 SV=1 - [LIRB5_HUMAN]	3.39	1	2	2	5	0.989	0.441	0.610	0.475	4.13	3.39	2	5	590	64.1	7.30
P15498	Proto-oncogene vav OS=Homo sapiens GN=VAV1 PE=1 SV=4 - [VAV_HUMAN]	3.55	1	3	4	5	0.767	1.215	0.983	0.475	11.27	3.55	4	5	845	98.3	6.62
Q8N5C1	Protein FAM26E OS=Homo sapiens GN=FAM26E PE=2 SV=1 - [FA26E_HUMAN]	10.68	1	3	3	5	0.829	0.317	0.701	0.477	16.53	10.68	3	5	309	35.1	8.13
P29966	Myristoylated alanine-rich C-kinase substrate OS=Homo sapiens GN=MARCKS PE=1 SV=4 - [MARCS_HUMA	36.75	1	9	9	106	0.889	1.058	1.071	0.478	342.33	36.75	15	106	332	31.5	4.45
Q9UHN1	DNA polymerase subunit gamma-2, mitochondrial OS=Homo sapiens GN=POLG2 PE=1 SV=1 - [DPOG2_HUMA	2.47	1	1	1	1	1.189	0.135	0.228	0.478	0.00	2.47	1	1	485	54.9	8.35
P09493	Tropomyosin alpha-1 chain OS=Homo sapiens GN=TPM1 PE=1 SV=2 - [TPM1_HUMAN]	83.45	1	17	46	1389	0.667	0.984	1.528	0.479	3283.35	83.45	76	1389	284	32.7	4.74
P12757	Ski-like protein OS=Homo sapiens GN=SKIL PE=1 SV=2 - [SKIL_HUMAN]	3.51	1	1	3	6	0.664	0.792	1.672	0.480	12.48	3.51	3	6	684	76.9	7.11
O94832	Unconventional myosin-Id OS=Homo sapiens GN=MYO1D PE=1 SV=2 - [MYO1D_HUMAN]	17.10	1	14	18	45	0.233	0.945	0.906	0.481	85.53	17.10	26	45	1006	116.1	9.39

P09486	SPARC OS=Homo sapiens GN=SPARC PE=1 SV=1 - [SPRC_HUMAN]	33.66	1	12	12	68	0.364	0.397	0.658	0.481	171.44	33.66	21	68	303	34.6	4.84
P02100	Hemoglobin subunit epsilon OS=Homo sapiens GN=HBE1 PE=1 SV=2 - [HBE_HUMAN]	6.12	3	1	1	4	1.475	0.435	1.055	0.481	14.59	6.12	2	4	147	16.2	8.63
Q9BXW9	Fanconi anemia group D2 protein OS=Homo sapiens GN=FANCD2 PE=1 SV=2 - [FACD2_HUMAN]	0.55	1	1	1	1	0.512	0.494	1.037	0.483	2.24	0.55	1	1	1451	164.0	5.88
Q8N556	Actin filament-associated protein 1 OS=Homo sapiens GN=AFAP1 PE=1 SV=2 - [AFAP1_HUMAN]	17.95	1	12	12	27	0.709	0.947	1.270	0.484	45.72	17.95	20	27	730	80.7	8.68
Q92625	Ankyrin repeat and SAM domain-containing protein 1A OS=Homo sapiens GN=ANKS1A PE=1 SV=4 - [ANS1A_HUMAN]	5.47	1	4	5	14	1.148	1.584	1.678	0.484	40.68	5.47	6	14	1134	123.0	6.38
Q5T5P2	Sickle tail protein homolog OS=Homo sapiens GN=KIAA1217 PE=1 SV=2 - [SKT_HUMAN]	2.16	2	1	4	11	0.283		0.503	0.484	4.39	2.16	4	11	1943	214.0	7.06
P01563	Interferon alpha-2 OS=Homo sapiens GN=IFNA2 PE=1 SV=1 - [IFNA2_HUMAN	4.79	1	1	1	1	1.039	0.867	0.755	0.484	2.16	4.79	1	1	188	21.5	6.76
P08582	Melanotransferrin OS=Homo sapiens GN=MFI2 PE=1 SV=2 - [TRFM_HUMAN]	7.45	1	5	5	11	0.356	1.267	1.174	0.484	20.36	7.45	8	11	738	80.2	5.94
Q02108	Guanylate cyclase soluble subunit alpha-3 OS=Homo sapiens GN=GUCY1A3 PE=1 SV=2 - [GCYA3_HUMAN]	3.77	1	3	3	6	0.563	0.721	1.121	0.484	14.76	3.77	4	6	690	77.4	7.11

O60291	E3 ubiquitin-protein ligase MGRN1 OS=Homo sapiens GN=MGRN1 PE=1 SV=2 - [MGRN1_HUMAN]	8.51	2	4	4	11	0.896	0.648	0.603	0.485	23.57	8.51	6	11	552	60.7	5.15
Q9NWD9	Protein BEX4 OS=Homo sapiens GN=BEX4 PE=2 SV=1 - [BEX4_HUMAN]	15.00	1	1	1	1	1.413	0.109	0.308	0.486	0.00	15.00	1	1	120	14.1	6.39
P02042	Hemoglobin subunit delta OS=Homo sapiens GN=HBD PE=1 SV=2 - [HBD_HUMAN]	17.01	2	2	2	6	1.039	0.377	0.982	0.486	7.48	17.01	3	6	147	16.0	8.05
O75815	Breast cancer anti-estrogen resistance protein 3 OS=Homo sapiens GN=BCAR3 PE=1 SV=1 - [BCAR3_HUMAN]	4.12	1	2	2	2	1.806	1.237	1.495	0.487	2.59	4.12	2	2	825	92.5	7.96
Q5VV67	Peroxisome proliferator-activated receptor gamma coactivator-related protein 1 OS=Homo sapiens GN=PPRC1 PE=1 SV=1 - [PPRC1_HUMAN	1.02	1	1	2	2	0.853	0.470	0.604	0.487	3.69	1.02	2	2	1664	177.4	6.51
Q04671	P protein OS=Homo sapiens GN=OCA2 PE=1 SV=2 - [P_HUMAN]	6.21	1	2	2	4	1.062	0.913	1.149	0.487	0.00	6.21	2	4	838	92.8	7.27
Q6UWR7	Ectonucleotide pyrophosphatase/phosphodiesterase family member 6 OS=Homo sapiens GN=ENPP6 PE=1 SV=2 - [ENPP6_HUMAN]	3.41	1	1	1	1	1.210	0.894	0.838	0.488	0.00	3.41	1	1	440	50.2	7.99
Q9BTT6	Leucine-rich repeat-containing protein 1 OS=Homo sapiens GN=LRRC1 PE=1 SV=1 - [LRRC1_HUMAN]	13.55	1	3	6	15	0.885	1.131	1.252	0.488	32.85	13.55	11	15	524	59.2	5.02

P27144	Adenylate kinase 4, mitochondrial OS=Homo sapiens GN=AK4 PE=1 SV=1 - [KAD4_HUMAN]	54.26	1	12	12	45	0.991	0.884	0.619	0.489	84.04	54.26	20	45	223	25.3	8.40
P23352	Anosmin-1 OS=Homo sapiens GN=KAL1 PE=1 SV=3 - [KALM_HUMAN]	2.06	1	1	1	2	0.380	0.489	0.432	0.489	4.74	2.06	1	2	680	76.1	9.16
Q70E73	Ras-associated and pleckstrin homology domains-containing protein 1 OS=Homo sapiens GN=RAPH1 PE=1 SV=3 - [RAPH1_HUMAN]	17.84	1	18	18	36	0.765	0.806	1.147	0.489	76.28	17.84	24	36	1250	135.2	8.85
Q86UV6	Tripartite motif-containing protein 74 OS=Homo sapiens GN=TRIM74 PE=2 SV=1 - [TRI74_HUMAN]	4.40	2	1	1	1	1.055	0.350	0.743	0.490	2.80	4.40	1	1	250	28.5	6.27
Q9BZQ8	Protein Niban OS=Homo sapiens GN=FAM129A PE=1 SV=1 - [NIBAN_HUMAN]	24.68	1	22	22	83	0.451	2.280	1.099	0.490	211.65	24.68	37	83	928	103.1	4.78
O43156	TELO2-interacting protein 1 homolog OS=Homo sapiens GN=TTI1 PE=1 SV=3 - [TTI1_HUMAN]	2.30	1	2	2	3	1.018	0.997	0.526	0.491	3.10	2.30	2	3	1089	122.0	5.97
Q9NR56	Muscleblind-like protein 1 OS=Homo sapiens GN=MBNL1 PE=1 SV=2 - [MBNL1_HUMAN]	14.18	2	2	6	33	0.490	0.708	1.069	0.492	55.69	14.18	11	33	388	41.8	8.90
Q9UKE5	TRAF2 and NCK-interacting protein kinase OS=Homo sapiens GN=TNIK PE=1 SV=1 - [TNIK_HUMAN]	8.75	2	4	11	19	0.638	1.060	1.123	0.495	40.46	8.75	15	19	1360	154.8	7.17
Q9UKX5	Integrin alpha-11 OS=Homo sapiens GN=ITGA11 PE=1 SV=2 - [ITA11_HUMAN]	11.87	1	13	13	32	0.614	0.428	0.557	0.496	46.17	11.87	20	32	1188	133.4	6.70

O95319	CUGBP Elav-like family member 2 OS=Homo sapiens GN=CELF2 PE=1 SV=1 - [CELF2_HUMAN]	3.94	1	1	2	4	0.619	3.149	1.563	0.497	8.55	3.94	2	4	508	54.3	8.76
O75909	Cyclin-K OS=Homo sapiens GN=CCNK PE=1 SV=2 - [CCNK_HUMAN]	2.76	1	1	1	2	0.489	1.293	0.935	0.498	6.14	2.76	1	2	580	64.2	8.41
O94808	Glutamine--fructose-6-phosphate aminotransferase [isomerizing] 2 OS=Homo sapiens GN=GFPT2 PE=1 SV=3 - [GFPT2_HUMAN]	19.79	1	7	12	31	0.217	0.912	1.013	0.499	75.95	19.79	19	31	682	76.9	7.37
O60637	Tetraspanin-3 OS=Homo sapiens GN=TSPAN3 PE=2 SV=1 - [TSN3_HUMAN]	2.77	1	1	1	5	0.612	0.488	0.652	0.500	11.49	2.77	2	5	253	28.0	5.81
Q86YC3	Negative regulator of reactive oxygen species OS=Homo sapiens GN=NRROS PE=1 SV=1 - [NRROS_HUMAN]	1.59	1	1	1	3	1.766	0.671	2.424	0.500	6.82	1.59	1	3	692	76.3	6.16
Q8TD30	Alanine aminotransferase 2 OS=Homo sapiens GN=GPT2 PE=1 SV=1 - [ALAT2_HUMAN]	5.54	1	3	3	7	0.510	0.718	0.772	0.500	10.64	5.54	4	7	523	57.9	7.71
P36956	Sterol regulatory element-binding protein 1 OS=Homo sapiens GN=SREBF1 PE=1 SV=2 - [SRBP1_HUMAN]	1.66	1	2	2	2	0.348	0.485	1.089	0.502	0.00	1.66	2	2	1147	121.6	8.13
Q9H7C4	Syncoilin OS=Homo sapiens GN=SYNC PE=1 SV=3 - [SYNCI_HUMAN]	32.78	1	13	13	56	0.567	1.600	1.350	0.502	141.04	32.78	20	56	482	55.3	4.61
O94925	Glutaminase kidney isoform, mitochondrial OS=Homo sapiens GN=GLS PE=1 SV=1 - [GLSK_HUMAN]	42.75	1	22	23	101	0.418	0.872	1.123	0.502	315.41	42.75	34	101	669	73.4	7.77

Q9UBY9	Heat shock protein beta-7 OS=Homo sapiens GN=HSPB7 PE=1 SV=1 - [HSPB7_HUMAN]	55.88	1	7	7	56	0.313	1.969	1.873	0.503	129.94	55.88	11	56	170	18.6	6.52
Q9HBA9	Putative N-acetylated-alpha-linked acidic dipeptidase OS=Homo sapiens GN=FOLH1B PE=2 SV=1 - [FOH1B_HUMA	5.66	3	3	3	3	1.955	0.206	0.630	0.503	3.81	5.66	3	3	442	50.0	7.02
Q16270	Insulin-like growth factor-binding protein 7 OS=Homo sapiens GN=IGFBP7 PE=1 SV=1 - [IBP7_HUMAN]	34.75	1	10	11	29	0.760	0.743	1.554	0.503	69.83	34.75	15	29	282	29.1	7.90
Q8WXI7	Mucin-16 OS=Homo sapiens GN=MUC16 PE=1 SV=2 - [MUC16_HUMAN]	0.76	1	7	7	11	1.269	0.604	1.015	0.503	12.77	0.76	7	11	22152	####	6.00
Q9NRP0	Oligosaccharyltransferase complex subunit OSTC OS=Homo sapiens GN=OSTC PE=1 SV=1 - [OSTC_HUMAN]	8.05	1	1	1	1	0.683	0.510	0.436	0.503	4.11	8.05	1	1	149	16.8	9.13
Q8NEA6	Zinc finger protein GLIS3 OS=Homo sapiens GN=GLIS3 PE=2 SV=5 - [GLIS3_HUMAN]	4.13	4	1	2	2	1.664	0.973	0.612	0.504	0.00	4.13	2	2	775	83.6	8.07
P40121	Macrophage-capping protein OS=Homo sapiens GN=CAPG PE=1 SV=2 - [CAPG_HUMAN]	42.24	1	13	13	49	0.629	2.886	1.204	0.505	121.70	42.24	21	49	348	38.5	6.19
Q8WV93	Lactation elevated protein 1 OS=Homo sapiens GN=LACE1 PE=2 SV=2 - [LACE1_HUMAN	4.78	1	2	2	2	0.614	0.166	0.428	0.505	4.58	4.78	2	2	481	54.8	7.27
A0MZ66	Shootin-1 OS=Homo sapiens GN=KIAA1598 PE=1 SV=4 - [SHOT1_HUMAN]	3.01	1	2	2	5	1.317	1.116	0.625	0.506	12.79	3.01	3	5	631	71.6	5.33

Q9UMS6	Synaptopodin-2 OS=Homo sapiens GN=SYNPO2 PE=1 SV=2 - [SYNP2_HUMAN]	34.86	2	37	37	271	0.621	1.493	2.147	0.507	531.35	34.86	63	271	1093	117.4	8.57
Q8TE73	Dynein heavy chain 5, axonemal OS=Homo sapiens GN=DNAH5 PE=1 SV=3 - [DYH5_HUMAN]	1.69	1	6	7	10	0.940	1.030	1.382	0.507	17.07	1.69	7	10	4624	528.7	6.10
Q96NY9	Crossover junction endonuclease MUS81 OS=Homo sapiens GN=MUS81 PE=1 SV=3 - [MUS81_HUMAN]	9.44	1	2	2	2	0.955	0.923	1.077	0.508	0.00	9.44	2	2	551	61.1	9.73
Q96MT0	Putative uncharacterized protein FLJ31958 OS=Homo sapiens PE=2 SV=1 - [YJ006_HUMAN]	6.75	1	1	1	2	0.677	0.551	0.717	0.511	4.76	6.75	1	2	163	17.0	5.45
P45877	Peptidyl-prolyl cis-trans isomerase C OS=Homo sapiens GN=PPIC PE=1 SV=1 - [PPIC_HUMAN]	34.91	1	5	6	62	0.680	0.722	0.769	0.511	138.45	34.91	8	62	212	22.7	8.40
P54760	Ephrin type-B receptor 4 OS=Homo sapiens GN=EPHB4 PE=1 SV=2 - [EPHB4_HUMAN]	4.05	8	2	4	6	0.967	1.184	1.295	0.511	13.16	4.05	5	6	987	108.2	6.90
Q9HAF1	Chromatin modification-related protein MEAF6 OS=Homo sapiens GN=MEAF6 PE=1 SV=1 - [EAF6_HUMAN]	12.04	1	2	2	4	1.460	1.129	1.082	0.513	6.82	12.04	2	4	191	21.6	9.32
Q02952	A-kinase anchor protein 12 OS=Homo sapiens GN=AKAP12 PE=1 SV=4 - [AKA12_HUMAN]	40.46	1	55	55	358	0.673	1.234	1.085	0.514	1070.16	40.46	91	358	1782	191.4	4.41
Q86Z20	Coiled-coil domain-containing protein 125 OS=Homo sapiens GN=CCDC125 PE=1 SV=2 - [CC125_HUMAN]	3.91	1	1	2	2	0.965	0.557	1.166	0.514	1.74	3.91	2	2	511	58.6	7.24

O14558	Heat shock protein beta-6 OS=Homo sapiens GN=HSPB6 PE=1 SV=2 - [HSPB6_HUMAN]	61.25	1	6	6	66	0.328	2.588	1.620	0.515	162.77	61.25	11	66	160	17.1	6.40
Q6AWC2	Protein WWC2 OS=Homo sapiens GN=WWC2 PE=1 SV=2 - [WWC2_HUMAN]	6.04	1	6	6	10	1.104	0.402	0.792	0.515	18.28	6.04	8	10	1192	133.8	5.53
P62837	Ubiquitin-conjugating enzyme E2 D2 OS=Homo sapiens GN=UBE2D2 PE=1 SV=1 - [UB2D2_HUMAN]	4.76	1	1	1	10	0.840	0.622	0.460	0.515	18.48	4.76	2	10	147	16.7	7.83
P18085	ADP-ribosylation factor 4 OS=Homo sapiens GN=ARF4 PE=1 SV=3 - [ARF4_HUMAN]	44.44	2	4	8	107	0.453	0.369	0.498	0.517	207.78	44.44	15	107	180	20.5	7.14
Q9BUD6	Spondin-2 OS=Homo sapiens GN=SPON2 PE=1 SV=3 - [SPON2_HUMAN]	2.42	1	1	1	5	0.597	0.299	0.836	0.517	12.16	2.42	2	5	331	35.8	5.52
P51513	RNA-binding protein Nova-1 OS=Homo sapiens GN=NOVA1 PE=1 SV=1 - [NOVA1_HUMAN]	5.49	1	1	2	3	0.443	2.226	1.543	0.518	7.25	5.49	3	3	510	52.0	8.88
Q8TCC7	Solute carrier family 22 member 8 OS=Homo sapiens GN=SLC22A8 PE=1 SV=1 - [S22A8_HUMAN]	1.29	1	1	1	1	0.290	0.462	0.719	0.519	2.63	1.29	1	1	542	59.8	8.85
Q86TX2	Acyl-coenzyme A thioesterase 1 OS=Homo sapiens GN=ACOT1 PE=1 SV=1 - [ACOT1_HUMAN]	33.73	2	13	13	77	0.670	1.078	1.101	0.519	209.28	33.73	22	77	421	46.2	7.34
Q9H3S4	Thiamin pyrophosphokinase 1 OS=Homo sapiens GN=TPK1 PE=1 SV=1 - [TPK1_HUMAN]	3.70	1	1	1	4	0.547	0.805	0.836	0.521	6.96	3.70	2	4	243	27.2	5.20

Q06136	3-ketodihydrosphingosine reductase OS=Homo sapiens GN=KDSR PE=1 SV=1 - [KDSR_HUMAN]	7.83	1	2	2	4	0.405	1.225	0.666	0.521	9.19	7.83	2	4	332	36.2	7.12
O43556	Epsilon-sarcoglycan OS=Homo sapiens GN=SGCE PE=1 SV=6 - [SGCE_HUMAN]	8.24	1	2	2	7	1.255	0.544	0.724	0.522	15.31	8.24	4	7	437	49.8	6.55
Q9H3G5	Probable serine carboxypeptidase CPVL OS=Homo sapiens GN=CPVL PE=1 SV=2 - [CPVL_HUMAN]	2.73	1	1	1	1	0.802	0.703	0.803	0.522	1.86	2.73	1	1	476	54.1	5.62
Q92466	DNA damage-binding protein 2 OS=Homo sapiens GN=DDB2 PE=1 SV=1 - [DDB2_HUMAN]	4.92	1	2	2	9	2.283	1.616	1.343	0.524	21.28	4.92	4	9	427	47.8	9.47
P48509	CD151 antigen OS=Homo sapiens GN=CD151 PE=1 SV=3 - [CD151_HUMAN]	12.65	1	4	4	10	0.681	0.779	0.904	0.524	20.32	12.65	6	10	253	28.3	7.47
O14734	Acyl-coenzyme A thioesterase 8 OS=Homo sapiens GN=ACOT8 PE=1 SV=1 - [ACOT8_HUMAN]	13.79	1	4	4	6	0.639	0.946	1.068	0.524	8.66	13.79	6	6	319	35.9	7.56
Q15113	Procollagen C-endopeptidase enhancer 1 OS=Homo sapiens GN=PCOLCE PE=1 SV=2 - [PCOC1_HUMAN]	23.39	1	8	8	25	0.568	0.928	1.143	0.525	73.12	23.39	13	25	449	47.9	7.43
Q8TDS4	Hydroxycarboxylic acid receptor 2 OS=Homo sapiens GN=HCAR2 PE=1 SV=1 - [HCAR2_HUMAN]	2.20	2	1	1	1	0.791	0.292	0.553	0.525	1.88	2.20	1	1	363	41.8	9.11
Q8IYB0	Putative uncharacterized protein MGC39545 OS=Homo sapiens PE=5 SV=2 - [YK038_HUMAN]	10.20	1	1	1	1	0.654	1.987	4.752	0.526	2.94	10.20	1	1	196	21.2	9.76

Q16821	Protein phosphatase 1 regulatory subunit 3A OS=Homo sapiens GN=PPP1R3A PE=1 SV=3 - [PPR3A_HUMAN]	2.41	1	2	2	2	0.336	0.355	0.561	0.526	2.42	2.41	2	2	1122	125.7	5.00
P13473	Lysosome-associated membrane glycoprotein 2 OS=Homo sapiens GN=LAMP2 PE=1 SV=2 - [LAMP2_HUMAN]	9.27	1	4	4	20	0.689	0.561	0.500	0.526	42.35	9.27	7	20	410	44.9	5.63
P50454	Serpin H1 OS=Homo sapiens GN=SERPINH1 PE=1 SV=2 - [SERPH_HUMAN]	60.29	1	27	27	537	0.480	0.701	0.738	0.527	1349.02	60.29	45	537	418	46.4	8.69
Q99757	Thioredoxin, mitochondrial OS=Homo sapiens GN=TXN2 PE=1 SV=2 - [THIOM_HUMAN]	9.04	1	1	1	1	0.324	1.033	0.341	0.528	3.09	9.04	1	1	166	18.4	8.29
P12109	Collagen alpha-1(VI) chain OS=Homo sapiens GN=COL6A1 PE=1 SV=3 - [CO6A1_HUMAN]	44.16	1	36	36	370	0.625	1.020	1.478	0.529	1022.46	44.16	58	370	1028	108.5	5.43
Q9ULW8	Protein-arginine deiminase type-3 OS=Homo sapiens GN=PADI3 PE=1 SV=2 - [PADI3_HUMAN	2.26	1	1	1	1	1.093	0.623	1.070	0.529	3.02	2.26	1	1	664	74.7	5.54
Q9H4M7	Pleckstrin homology domain-containing family A member 4 OS=Homo sapiens GN=PLEKHA4 PE=1 SV=2 - [PKHA4_HUMAN]	7.32	1	3	3	4	1.448	1.296	2.045	0.530	7.75	7.32	3	4	779	85.3	10.56
Q9UJX5	Anaphase-promoting complex subunit 4 OS=Homo sapiens GN=ANAPC4 PE=1 SV=2 - [APC4_HUMAN]	0.87	1	1	1	1	1.111	1.049	0.719	0.530	1.92	0.87	1	1	808	92.1	5.53

P38571	Lysosomal acid lipase/cholesteryl ester hydrolase OS=Homo sapiens GN=LIPA PE=1 SV=2 - [LICH_HUMAN]	6.77	1	2	2	4	0.725	1.153	0.921	0.530	8.13	6.77	3	4	399	45.4	6.92
Q96Q04	Serine/threonine-protein kinase LMTK3 OS=Homo sapiens GN=LMTK3 PE=2 SV=2 - [LMTK3_HUMAN]	2.26	1	1	1	1	1.182	1.009	0.781	0.531	3.23	2.26	1	1	1460	153.6	4.86
Q5U5X0	Complex III assembly factor LYRM7 OS=Homo sapiens GN=LYRM7 PE=1 SV=1 - [LYRM7_HUMAN]	20.19	2	1	2	8	0.880	0.667	0.587	0.531	4.86	20.19	2	8	104	11.9	9.66
Q8TC84	Fibronectin type 3 and ankyrin repeat domains protein 1 OS=Homo sapiens GN=FANK1 PE=2 SV=3 - [FANK1_HUMAN	3.48	1	1	1	1	0.549	0.539	0.681	0.532	2.43	3.48	1	1	345	38.3	8.73
P23142	Fibulin-1 OS=Homo sapiens GN=FBLN1 PE=1 SV=4 - [FBLN1_HUMAN]	4.41	1	3	3	5	0.421	2.960	1.233	0.533	13.26	4.41	5	5	703	77.2	5.22
Q7Z388	Probable C-mannosyltransferase DPY19L4 OS=Homo sapiens GN=DPY19L4 PE=1 SV=1 - [D19L4_HUMAN]	0.97	1	1	1	1	0.918	0.820	0.974	0.533	2.13	0.97	1	1	723	83.7	8.16
P45381	Aspartoacylase OS=Homo sapiens GN=ASPA PE=1 SV=1 - [ACY2_HUMAN]	4.15	1	1	1	2	0.653	0.298	0.370	0.535	2.92	4.15	2	2	313	35.7	6.52
Q96RT6	cTAGE family member 2 OS=Homo sapiens GN=CTAGE1 PE=1 SV=2 - [CTGE2_HUMAN]	7.25	2	1	6	21	0.565	0.933	1.303	0.536	36.62	7.25	7	21	745	85.2	6.16
P13647	Keratin, type II cytoskeletal 5 OS=Homo sapiens GN=KRT5 PE=1 SV=3 - [K2C5_HUMAN]	11.69	2	2	8	97	1.123	1.824	1.256	0.537	192.51	11.69	12	97	590	62.3	7.74

Q6PIJ6	F-box only protein 38 OS=Homo sapiens GN=FBXO38 PE=1 SV=3 - [FBX38_HUMAN]	0.84	1	1	1	1	1.138	1.007	1.287	0.538	0.00	0.84	1	1	1188	133.9	6.33
O14683	Tumor protein p53-inducible protein 11 OS=Homo sapiens GN=TP53I11 PE=1 SV=2 - [P5I11_HUMAN]	22.22	1	4	4	37	0.624	0.669	0.945	0.538	127.09	22.22	7	37	189	21.0	9.55
Q5JRM2	Uncharacterized protein CXorf66 OS=Homo sapiens GN=CXorf66 PE=2 SV=1 - [CX066_HUMAN]	4.99	1	1	1	4	0.749	1.066	0.971	0.539	0.00	4.99	1	4	361	39.9	9.54
Q9UPS8	Ankyrin repeat domain-containing protein 26 OS=Homo sapiens GN=ANKRD26 PE=1 SV=3 - [ANR26_HUMAN]	1.99	1	3	4	7	1.085	1.072	0.912	0.539	16.82	1.99	5	7	1709	196.2	5.72
Q8TAD4	Zinc transporter 5 OS=Homo sapiens GN=SLC30A5 PE=1 SV=1 - [ZNT5_HUMAN]	0.78	1	1	1	1	0.521	0.604	0.702	0.541	2.47	0.78	1	1	765	84.0	7.31
Q8IXL7	Methionine-R-sulfoxide reductase B3 OS=Homo sapiens GN=MSRB3 PE=1 SV=2 - [MSRB3_HUMAN]	18.75	1	4	4	13	0.636	0.850	1.057	0.544	24.23	18.75	8	13	192	20.7	7.33
A6NNM8	Tubulin polyglutamylase TTLL13 OS=Homo sapiens GN=TTLL13 PE=2 SV=2 - [TTL13_HUMAN]	0.86	1	1	1	12	0.675	0.946	2.221	0.544	22.92	0.86	2	12	815	93.6	8.91
Q5H9U9	Probable ATP-dependent RNA helicase DDX60-like OS=Homo sapiens GN=DDX60L PE=2 SV=2 - [DDX6L_HUMAN	0.76	1	1	1	2	0.688	0.598	0.828	0.544	2.16	0.76	1	2	1706	197.5	8.29
Q96EK6	Glucosamine 6-phosphate N-acetyltransferase OS=Homo sapiens GN=GNPNAT1 PE=1 SV=1 - [GNA1_HUMAN]	45.11	1	6	6	10	0.466	0.854	0.800	0.544	32.26	45.11	9	10	184	20.7	7.99

O00462	Beta-mannosidase OS=Homo sapiens GN=MANBA PE=1 SV=3 - [MANBA_HUMAN]	9.33	1	7	8	12	0.651	0.641	0.755	0.546	26.14	9.33	10	12	879	100.8	5.52
P49588	Alanine--tRNA ligase, cytoplasmic OS=Homo sapiens GN=AARS PE=1 SV=2 - [SYAC_HUMAN]	54.03	2	46	48	248	0.697	0.914	0.856	0.546	572.67	54.03	75	248	968	106.7	5.53
Q8WUJ3	Cell migration-inducing and hyaluronan-binding protein OS=Homo sapiens GN=CEMIP PE=1 SV=2 - [CEMIP_HUMAN]	39.31	1	47	47	185	0.470	0.501	0.619	0.547	438.18	39.31	80	185	1361	152.9	7.85
Q8NI22	Multiple coagulation factor deficiency protein 2 OS=Homo sapiens GN=MCFD2 PE=1 SV=1 - [MCFD2_HUMA	28.08	1	2	2	2	0.593	0.439	0.696	0.547	8.97	28.08	2	2	146	16.4	4.63
Q8N436	Inactive carboxypeptidase-like protein X2 OS=Homo sapiens GN=CPXM2 PE=2 SV=3 - [CPXM2_HUMAN]	1.85	1	1	1	1	1.443	1.505	1.911	0.548	0.00	1.85	1	1	756	85.8	6.87
P02511	Alpha-crystallin B chain OS=Homo sapiens GN=CRYAB PE=1 SV=2 - [CRYAB_HUMAN]	58.29	1	11	11	67	0.584	0.925	1.105	0.548	163.69	58.29	17	67	175	20.1	7.33
Q71RG4	Transmembrane and ubiquitin-like domain-containing protein 2 OS=Homo sapiens GN=TMUB2 PE=2 SV=2 - [TMUB2_HUMAN]	4.36	1	1	1	3	0.430	0.560	0.461	0.548	9.30	4.36	2	3	321	33.8	5.03
P46821	Microtubule-associated protein 1B OS=Homo sapiens GN=MAP1B PE=1 SV=2 - [MAP1B_HUMAN]	48.01	1	100	102	673	0.619	1.423	1.274	0.548	1841.61	48.01	165	673	2468	270.5	4.81

Q6PRD1	Probable G-protein coupled receptor 179 OS=Homo sapiens GN=GPR179 PE=1 SV=2 - [GP179_HUMAN	1.65	1	2	2	3	0.783	1.080	1.702	0.549	4.56	1.65	2	3	2367	257.2	5.71
P13797	Plastin-3 OS=Homo sapiens GN=PLS3 PE=1 SV=4 - [PLST_HUMAN]	58.41	1	28	37	318	0.675	0.863	1.332	0.549	722.71	58.41	67	318	630	70.8	5.60
H0Y354	Protein FAM72C OS=Homo sapiens GN=FAM72C PE=3 SV=2 - [FA72C_HUMAN]	10.07	2	1	1	1	1.198	0.825	1.296	0.549	0.00	10.07	1	1	149	16.7	6.68
P23381	Tryptophan--tRNA ligase, cytoplasmic OS=Homo sapiens GN=WARS PE=1 SV=2 - [SYWC_HUMAN]	46.28	1	26	26	213	1.047	0.589	0.585	0.550	579.60	46.28	42	213	471	53.1	6.23
Q9Y4D1	Disheveled-associated activator of morphogenesis 1 OS=Homo sapiens GN=DAAM1 PE=1 SV=2 - [DAAM1_HUMA N]	1.86	1	2	2	2	0.624	1.472	1.119	0.550	2.44	1.86	2	2	1078	123.4	7.23
O15054	Lysine-specific demethylase 6B OS=Homo sapiens GN=KDM6B PE=1 SV=4 - [KDM6B_HUMA N]	7.12	1	3	4	6	0.703	2.141	1.338	0.551	4.39	7.12	4	6	1643	176.5	8.54
P13995	Bifunctional methylenetetrahydrofolate dehydrogenase/cyclohydrolase, mitochondrial OS=Homo sapiens GN=MTHFD2 PE=1 SV=2 - [MTDC_HUMAN]	17.14	1	6	7	21	0.547	0.793	0.886	0.551	34.48	17.14	13	21	350	37.9	8.73
P52294	Importin subunit alpha-5 OS=Homo sapiens GN=KPNA1 PE=1 SV=3 - [IMA5_HUMAN]	16.73	1	4	9	20	0.640	0.986	0.990	0.552	45.03	16.73	12	20	538	60.2	5.01
P29475	Nitric oxide synthase, brain OS=Homo sapiens GN=NOS1 PE=1 SV=2 - [NOS1_HUMAN]	1.67	1	2	2	2	0.203	0.766	1.478	0.552	2.01	1.67	2	2	1434	160.9	7.42

A6NNF4	Zinc finger protein 726 OS=Homo sapiens GN=ZNF726 PE=2 SV=4 - [ZN726_HUMAN]	13.55	94	2	3	6	1.356	0.152	0.309	0.552	2.14	13.55	3	6	738	85.5	9.09
Q15043	Zinc transporter ZIP14 OS=Homo sapiens GN=SLC39A14 PE=1 SV=3 - [S39AE_HUMAN	6.71	1	2	2	4	0.381	0.557	0.703	0.553	16.53	6.71	3	4	492	54.2	5.33
P13612	Integrin alpha-4 OS=Homo sapiens GN=ITGA4 PE=1 SV=3 - [ITA4_HUMAN]	6.20	1	7	7	17	0.525	0.479	1.096	0.553	23.88	6.20	12	17	1032	114.8	6.48
P0CW24	Paraneoplastic antigen-like protein 6A OS=Homo sapiens GN=PNMA6A PE=2 SV=1 - [PNM6A_HUMAN]	1.50	2	1	1	1	0.694	0.765	0.832	0.553	0.00	1.50	1	1	399	44.0	5.38
Q5XX13	F-box/WD repeat-containing protein 10 OS=Homo sapiens GN=FBXW10 PE=2 SV=2 - [FBW10_HUMAN]	1.14	1	1	1	1	1.450	0.738	1.420	0.554	2.43	1.14	1	1	1052	119.8	9.39
Q8N4P3	Guanosine-3',5'-bis(diphosphate) 3'-pyrophosphohydrolase MESH1 OS=Homo sapiens GN=HDDC3 PE=1 SV=3 - [MESH1_HUMAN]	13.97	1	1	2	3	2.050	1.512	2.246	0.554	7.86	13.97	2	3	179	20.3	6.74
P28676	Grancalcin OS=Homo sapiens GN=GCA PE=1 SV=2 - [GRAN_HUMAN]	8.29	1	2	2	4	1.176	3.165	1.810	0.555	10.45	8.29	3	4	217	24.0	5.21
P98155	Very low-density lipoprotein receptor OS=Homo sapiens GN=VLDLR PE=1 SV=1 - [VLDLR_HUMAN]	17.30	1	11	11	30	0.702	0.560	0.633	0.555	72.51	17.30	18	30	873	96.0	4.79
O00425	Insulin-like growth factor 2 mRNA-binding protein 3 OS=Homo sapiens GN=IGF2BP3 PE=1 SV=2 - [IF2B3_HUMAN]	34.02	1	14	17	60	0.588	1.307	1.309	0.557	157.04	34.02	26	60	579	63.7	8.87

Q06278	Aldehyde oxidase OS=Homo sapiens GN=AOX1 PE=1 SV=2 - [AOXA_HUMAN]	5.61	1	5	6	9	0.287	0.807	0.589	0.557	14.95	5.61	8	9	1338	147.8	7.17
P35556	Fibrillin-2 OS=Homo sapiens GN=FBN2 PE=1 SV=3 - [FBN2_HUMAN]	3.85	1	8	10	19	0.252	0.619	0.584	0.559	36.54	3.85	13	19	2912	314.6	4.86
P13674	Prolyl 4-hydroxylase subunit alpha-1 OS=Homo sapiens GN=P4HA1 PE=1 SV=2 - [P4HA1_HUMAN]	61.24	1	37	37	412	0.688	0.669	0.752	0.559	1209.28	61.24	59	412	534	61.0	6.01
Q9ULG6	Cell cycle progression protein 1 OS=Homo sapiens GN=CCPG1 PE=2 SV=3 - [CCPG1_HUMAN]	4.36	4	3	5	6	0.602	0.714	0.708	0.560	11.71	4.36	6	6	757	87.3	5.95
Q9BV19	Uncharacterized protein C1orf50 OS=Homo sapiens GN=C1orf50 PE=1 SV=2 - [CA050_HUMAN]	6.53	1	1	1	2	0.668	1.593	1.000	0.560	2.33	6.53	2	2	199	21.9	5.88
P09619	Platelet-derived growth factor receptor beta OS=Homo sapiens GN=PDGFRB PE=1 SV=1 - [PGFRB_HUMAN	21.25	5	18	19	74	0.658	0.630	0.723	0.560	215.74	21.25	35	74	1106	123.9	4.98
P05121	Plasminogen activator inhibitor 1 OS=Homo sapiens GN=SERPINE1 PE=1 SV=1 - [PAI1_HUMAN]	17.66	1	7	7	22	0.192	0.456	1.017	0.560	48.43	17.66	12	22	402	45.0	7.20
P22413	Ectonucleotide pyrophosphatase/phosphodiesterase family member 1 OS=Homo sapiens GN=ENPP1 PE=1 SV=2 - [ENPP1_HUMAN]	0.76	1	1	1	1	0.913	2.501	1.477	0.561	2.22	0.76	1	1	925	104.9	7.14
Q6DKJ4	Nucleoredoxin OS=Homo sapiens GN=NXN PE=1 SV=2 - [NXN_HUMAN]	29.20	1	11	11	29	0.778	1.031	1.246	0.561	86.55	29.20	16	29	435	48.4	4.97

Q07092	Collagen alpha-1(XVI) chain OS=Homo sapiens GN=COL16A1 PE=1 SV=2 - [COGA1_HUMAN]	4.11	1	5	5	19	0.344	0.894	0.743	0.562	36.69	4.11	9	19	1604	157.7	7.84
P50135	Histamine N-methyltransferase OS=Homo sapiens GN=HNMT PE=1 SV=1 - [HNMT_HUMAN]	10.62	1	3	3	6	1.537	1.538	0.625	0.562	14.13	10.62	5	6	292	33.3	5.34
Q9NSU2	Three-prime repair exonuclease 1 OS=Homo sapiens GN=TREX1 PE=1 SV=1 - [TREX1_HUMAN]	4.34	1	1	1	1	0.964	0.916	1.125	0.562	0.00	4.34	1	1	369	38.9	8.41
P07355	Annexin A2 OS=Homo sapiens GN=ANXA2 PE=1 SV=2 - [ANXA2_HUMAN]	80.24	2	38	38	1417	0.988	1.012	1.129	0.562	3745.74	80.24	65	1417	339	38.6	7.75
Q01484	Ankyrin-2 OS=Homo sapiens GN=ANK2 PE=1 SV=4 - [ANK2_HUMAN]	3.31	1	8	10	24	0.411	1.047	1.194	0.562	68.72	3.31	18	24	3957	433.4	5.14
A6NL05	Protein FAM74A7 OS=Homo sapiens GN=FAM74A7 PE=3 SV=3 - [F74A7_HUMAN]	6.29	2	1	1	2	0.888	1.344	1.630	0.562	2.31	6.29	1	2	159	18.3	9.54
Q7Z398	Zinc finger protein 550 OS=Homo sapiens GN=ZNF550 PE=2 SV=2 - [ZN550_HUMAN]	3.32	1	1	1	1	1.180	1.211	0.827	0.563	0.00	3.32	1	1	422	48.4	8.63
Q9NXZ2	Probable ATP-dependent RNA helicase DDX43 OS=Homo sapiens GN=DDX43 PE=2 SV=2 - [DDX43_HUMAN	2.93	1	1	2	2	0.564	0.324		0.564	4.41	2.93	2	2	648	72.8	8.75
Q9H425	Uncharacterized protein C1orf198 OS=Homo sapiens GN=C1orf198 PE=1 SV=1 - [CA198_HUMAN]	59.02	1	14	14	50	1.124	0.449	1.145	0.564	137.21	59.02	21	50	327	36.3	5.72

P61457	Pterin-4-alpha-carbinolamine dehydratase OS=Homo sapiens GN=PCBD1 PE=1 SV=2 - [PHS_HUMAN]	50.96	1	5	6	36	0.724	0.636	0.656	0.565	78.76	50.96	12	36	104	12.0	6.80
O14732	Inositol monophosphatase 2 OS=Homo sapiens GN=IMPA2 PE=1 SV=1 - [IMPA2_HUMAN]	24.31	1	6	6	18	0.568	0.907	0.896	0.565	48.22	24.31	9	18	288	31.3	6.61
P35442	Thrombospondin-2 OS=Homo sapiens GN=THBS2 PE=1 SV=2 - [TSP2_HUMAN]	6.31	1	5	6	21	0.692	0.995	0.675	0.565	51.97	6.31	9	21	1172	129.9	4.83
Q8TDX7	Serine/threonine-protein kinase Nek7 OS=Homo sapiens GN=NEK7 PE=1 SV=1 - [NEK7_HUMAN]	40.40	1	8	11	50	0.832	0.923	0.930	0.566	113.69	40.40	18	50	302	34.5	8.25
P09488	Glutathione S-transferase Mu 1 OS=Homo sapiens GN=GSTM1 PE=1 SV=3 - [GSTM1_HUMAN]	58.72	1	1	16	130	0.480	0.087	0.380	0.566	344.04	58.72	25	130	218	25.7	6.70
O95864	Fatty acid desaturase 2 OS=Homo sapiens GN=FADS2 PE=1 SV=1 - [FADS2_HUMAN]	8.78	1	3	4	20	0.674	0.715	1.066	0.566	45.56	8.78	6	20	444	52.2	8.82
P52952	Homeobox protein Nkx-2.5 OS=Homo sapiens GN=NKX2-5 PE=1 SV=1 - [NKX25_HUMAN]	3.09	1	1	1	1	0.678	0.238	0.585	0.566	1.76	3.09	1	1	324	34.9	9.38
Q99715	Collagen alpha-1(XII) chain OS=Homo sapiens GN=COL12A1 PE=1 SV=2 - [COCA1_HUMAN]	41.69	1	108	108	571	1.176	0.533	0.868	0.567	1394.20	41.69	185	571	3063	332.9	5.53
O14717	tRNA (cytosine(38)-C(5))-methyltransferase OS=Homo sapiens GN=TRDMT1 PE=1 SV=1 - [TRDMT_HUMAN]	3.07	1	1	1	1	0.634			0.567	3.67	3.07	1	1	391	44.6	6.09

Q16222	UDP-N-acetylhexosamine pyrophosphorylase OS=Homo sapiens GN=UAP1 PE=1 SV=3 - [UAP1_HUMAN]	22.03	1	11	11	41	0.462	0.710	0.927	0.567	91.05	22.03	18	41	522	58.7	6.33
Q9UNN8	Endothelial protein C receptor OS=Homo sapiens GN=PROCR PE=1 SV=1 - [EPCR_HUMAN]	5.46	1	1	1	1	1.286	1.214	0.295	0.567	6.20	5.46	1	1	238	26.7	7.18
Q16832	Discoidin domain-containing receptor 2 OS=Homo sapiens GN=DDR2 PE=1 SV=2 - [DDR2_HUMAN]	14.27	1	9	9	44	0.594	1.304	0.940	0.567	96.23	14.27	17	44	855	96.7	5.36
Q12841	Follistatin-related protein 1 OS=Homo sapiens GN=FSTL1 PE=1 SV=1 - [FSTL1_HUMAN]	44.48	1	16	16	56	0.637	0.457	0.580	0.567	130.91	44.48	27	56	308	35.0	5.52
Q2M3G0	ATP-binding cassette sub-family B member 5 OS=Homo sapiens GN=ABCB5 PE=1 SV=4 - [ABCB5_HUMAN]	0.95	1	1	1	1	0.668	0.519	0.444	0.568	0.00	0.95	1	1	1257	138.6	7.53
P53985	Monocarboxylate transporter 1 OS=Homo sapiens GN=SLC16A1 PE=1 SV=3 - [MOT1_HUMAN]	6.40	1	2	2	8	0.455	0.840	0.668	0.568	26.09	6.40	3	8	500	53.9	8.66
Q16585	Beta-sarcoglycan OS=Homo sapiens GN=SGCB PE=1 SV=1 - [SGCB_HUMAN]	29.56	1	6	6	27	1.124	0.353	0.629	0.569	68.37	29.56	11	27	318	34.8	8.62
P78381	UDP-galactose translocator OS=Homo sapiens GN=SLC35A2 PE=1 SV=1 - [S35A2_HUMAN]	3.54	1	1	1	1	1.647	0.191	1.355	0.569	0.00	3.54	1	1	396	41.3	9.96
P15924	Desmoplakin OS=Homo sapiens GN=DSP PE=1 SV=3 - [DESP_HUMAN]	3.17	1	7	11	20	0.557	0.718	0.763	0.570	34.33	3.17	13	20	2871	331.6	6.81

Q8IXT1	DNA damage-induced apoptosis suppressor protein OS=Homo sapiens GN=DDIAS PE=2 SV=2 - [DDIAS_HUMAN]	0.70	1	1	1	2	0.955	1.362	0.775	0.570	3.99	0.70	1	2	998	111.5	7.18
Q9BQI7	PH and SEC7 domain-containing protein 2 OS=Homo sapiens GN=PSD2 PE=2 SV=3 - [PSD2_HUMAN]	0.78	1	1	1	1	0.958	1.179	1.278	0.571	0.00	0.78	1	1	771	84.6	5.19
Q9NZP8	Complement C1r subcomponent-like protein OS=Homo sapiens GN=C1RL PE=1 SV=2 - [C1RL_HUMAN]	1.64	1	1	1	2	1.529	3.191	1.335	0.571	5.21	1.64	2	2	487	53.5	7.20
P29017	T-cell surface glycoprotein CD1c OS=Homo sapiens GN=CD1C PE=1 SV=2 - [CD1C_HUMAN]	5.11	1	2	2	3	1.058	1.002	1.140	0.572	0.00	5.11	2	3	333	37.6	6.14
Q8N7R7	Cyclin-Y-like protein 1 OS=Homo sapiens GN=CCNYL1 PE=1 SV=2 - [CCYL1_HUMAN]	6.96	1	2	3	6	0.839	1.157	0.763	0.572	15.42	6.96	4	6	359	40.7	6.07
Q9H6S3	Epidermal growth factor receptor kinase substrate 8-like protein 2 OS=Homo sapiens GN=EPS8L2 PE=1 SV=2 - [ES8L2_HUMAN]	32.17	1	18	18	71	0.797	0.891	0.794	0.572	181.72	32.17	28	71	715	80.6	6.84
Q96EN8	Molybdenum cofactor sulfurase OS=Homo sapiens GN=MOCOS PE=1 SV=2 - [MOCOS_HUMAN]	2.48	1	2	2	9	0.413	0.683	0.538	0.573	17.47	2.48	4	9	888	98.1	6.70
Q9NWM8	Peptidyl-prolyl cis-trans isomerase FKBP14 OS=Homo sapiens GN=FKBP14 PE=1 SV=1 - [FKB14_HUMAN]	37.44	1	6	6	27	0.640	0.686	0.832	0.573	67.75	37.44	9	27	211	24.2	6.07

Q99569	Plakophilin-4 OS=Homo sapiens GN=PKP4 PE=1 SV=2 - [PKP4_HUMAN]	3.36	1	3	4	6	0.451	1.725	1.013	0.573	12.24	3.36	5	6	1192	131.8	8.94
O60774	Putative dimethylaniline monooxygenase [N-oxide-forming] 6 OS=Homo sapiens GN=FMO6P PE=5 SV=1 - [FMO6_HUMAN]	3.34	1	1	1	1	1.359	0.188	0.344	0.573	0.00	3.34	1	1	539	61.3	6.39
Q7Z7A1	Centriolin OS=Homo sapiens GN=CNTRL PE=1 SV=2 - [CNTRL_HUMAN]	2.28	1	2	7	9	1.302	1.142	0.986	0.574	10.38	2.28	7	9	2325	268.7	5.55
Q14714	Sarcospan OS=Homo sapiens GN=SSPN PE=2 SV=3 - [SSPN_HUMAN]	11.11	1	2	2	3	0.431	0.510	0.931	0.574	6.04	11.11	2	3	243	26.6	7.80
Q9NZP5	Olfactory receptor 5AC2 OS=Homo sapiens GN=OR5AC2 PE=2 SV=2 - [O5AC2_HUMAN]	2.27	1	1	1	1	1.502	0.811	1.307	0.575	1.90	2.27	1	1	309	35.3	8.94
Q14847	LIM and SH3 domain protein 1 OS=Homo sapiens GN=LASP1 PE=1 SV=2 - [LASP1_HUMAN]	76.63	1	21	21	379	0.834	0.756	1.157	0.576	955.83	76.63	33	379	261	29.7	7.05
Q9NUU6	Inactive ubiquitin thioesterase FAM105A OS=Homo sapiens GN=FAM105A PE=2 SV=1 - [F105A_HUMAN]	6.74	1	3	3	3	5.808	1.355	1.205	0.576	2.18	6.74	3	3	356	42.2	9.29
Q71UM5	40S ribosomal protein S27-like OS=Homo sapiens GN=RPS27L PE=1 SV=3 - [RS27L_HUMAN]	39.29	1	1	4	38	0.764	1.050	0.981	0.576	94.98	39.29	8	38	84	9.5	9.45
Q14739	Lamin-B receptor OS=Homo sapiens GN=LBR PE=1 SV=2 - [LBR_HUMAN]	4.88	1	3	3	5	1.173	0.170	0.503	0.576	10.36	4.88	4	5	615	70.7	9.36

Q9BV68	E3 ubiquitin-protein ligase RNF126 OS=Homo sapiens GN=RNF126 PE=1 SV=1 - [RN126_HUMAN]	4.91	1	2	2	5	0.708	0.528	0.571	0.577	4.42	4.91	3	5	326	35.6	5.72
Q99536	Synaptic vesicle membrane protein VAT-1 homolog OS=Homo sapiens GN=VAT1 PE=1 SV=2 - [VAT1_HUMAN]	49.62	1	14	14	135	0.904	0.990	0.970	0.577	402.74	49.62	20	135	393	41.9	6.29
Q8WWV3	Reticulon-4-interacting protein 1, mitochondrial OS=Homo sapiens GN=RTN4IP1 PE=1 SV=2 - [RT4I1_HUMAN]	3.54	1	1	1	4	0.740	0.736	0.675	0.577	16.54	3.54	2	4	396	43.6	9.11
Q6N043	Zinc finger protein 280D OS=Homo sapiens GN=ZNF280D PE=1 SV=3 - [Z280D_HUMAN]	1.33	1	1	1	1	0.346	1.130	0.774	0.578	2.46	1.33	1	1	979	109.2	8.19
Q8NA54	IQ and ubiquitin-like domain-containing protein OS=Homo sapiens GN=IQUB PE=1 SV=2 - [IQUB_HUMAN]	0.76	1	1	1	1	0.787	1.423	0.550	0.579	2.04	0.76	1	1	791	92.5	6.67
Q9NRQ2	Phospholipid scramblase 4 OS=Homo sapiens GN=PLSCR4 PE=1 SV=2 - [PLS4_HUMAN]	3.95	1	1	1	2	0.705	0.532	0.788	0.579	2.70	3.95	2	2	329	37.0	5.82
Q9H6S1	5-azacytidine-induced protein 2 OS=Homo sapiens GN=AZI2 PE=1 SV=1 - [AZI2_HUMAN]	7.65	1	3	3	6	0.853	0.835	0.857	0.580	16.96	7.65	4	6	392	44.9	6.60
P24593	Insulin-like growth factor-binding protein 5 OS=Homo sapiens GN=IGFBP5 PE=1 SV=1 - [IBP5_HUMAN]	41.54	1	12	12	115	1.019	1.114	1.454	0.580	287.03	41.54	21	115	272	30.6	8.21
P06241	Tyrosine-protein kinase Fyn OS=Homo sapiens GN=FYN PE=1 SV=3 - [FYN_HUMAN]	13.59	7	2	8	47	0.641	0.976	0.921	0.581	90.35	13.59	15	47	537	60.7	6.67

Q0IIM8	TBC1 domain family member 8B OS=Homo sapiens GN=TBC1D8B PE=1 SV=2 - [TBC8B_HUMAN]	2.05	1	2	3	12	0.417	0.880	0.924	0.581	29.37	2.05	5	12	1120	128.6	5.95
O95613	Pericentrin OS=Homo sapiens GN=PCNT PE=1 SV=4 - [PCNT_HUMAN]	1.89	1	4	5	8	1.581	0.695	1.133	0.582	12.80	1.89	5	8	3336	377.8	5.55
Q9NZI8	Insulin-like growth factor 2 mRNA-binding protein 1 OS=Homo sapiens GN=IGF2BP1 PE=1 SV=2 - [IF2B1_HUMAN]	10.40	1	3	7	28	0.510	2.342	6.230	0.582	65.10	10.40	10	28	577	63.4	9.20
Q7Z444	GTPase ERas OS=Homo sapiens GN=ERAS PE=2 SV=1 - [RASE_HUMAN]	4.72	1	1	1	2	0.743	0.944	0.839	0.582	1.64	4.72	1	2	233	25.3	6.58
P98160	Basement membrane-specific heparan sulfate proteoglycan core protein OS=Homo sapiens GN=HSPG2 PE=1 SV=4 - [PGBM_HUMAN]	5.51	1	18	18	43	0.486	0.875	1.288	0.583	96.85	5.51	30	43	4391	468.5	6.51
P22415	Upstream stimulatory factor 1 OS=Homo sapiens GN=USF1 PE=1 SV=1 - [USF1_HUMAN]	1.94	2	1	1	5	0.883	1.037	1.197	0.583	1.85	1.94	1	5	310	33.5	5.54
A6NKC4	Putative high affinity immunoglobulin gamma Fc receptor IC OS=Homo sapiens GN=FCGR1C PE=5 SV=2 - [FCGRC_HUMAN]	3.21	3	1	1	2	0.436	0.715	0.908	0.583	3.86	3.21	1	2	280	32.1	8.68
O15245	Solute carrier family 22 member 1 OS=Homo sapiens GN=SLC22A1 PE=1 SV=2 - [S22A1_HUMAN]	1.99	1	1	1	2	0.388	0.542	0.948	0.583	3.85	1.99	1	2	554	61.1	6.83

Q8TEA8	D-tyrosyl-tRNA(Tyr) deacylase 1 OS=Homo sapiens GN=DTD1 PE=1 SV=2 - [DTD1_HUMAN]	20.10	1	5	5	6	0.710	0.798	0.702	0.584	12.65	20.10	6	6	209	23.4	8.24
Q14416	Metabotropic glutamate receptor 2 OS=Homo sapiens GN=GRM2 PE=1 SV=2 - [GRM2_HUMAN]	0.80	1	1	1	1	0.558	0.732	0.624	0.584	2.37	0.80	1	1	872	95.5	8.12
Q9C0D0	Phosphatase and actin regulator 1 OS=Homo sapiens GN=PHACTR1 PE=2 SV=3 - [PHAR1_HUMAN]	3.79	1	1	2	10	2.441	0.441	0.691	0.584	27.35	3.79	2	10	580	66.3	6.96
Q9UJ78	Zinc finger MYM-type protein 5 OS=Homo sapiens GN=ZMYM5 PE=1 SV=4 - [ZMYM5_HUMAN]	1.64	1	1	1	1	1.445	1.091	0.929	0.584	0.00	1.64	1	1	669	74.8	8.16
Q9NR45	Sialic acid synthase OS=Homo sapiens GN=NANS PE=1 SV=2 - [SIAS_HUMAN]	47.35	1	14	14	87	0.603	0.976	1.002	0.585	259.29	47.35	24	87	359	40.3	6.74
Q9Y4K1	Absent in melanoma 1 protein OS=Homo sapiens GN=AIM1 PE=1 SV=3 - [AIM1_HUMAN]	2.50	1	4	4	22	1.179	0.698	0.809	0.585	42.30	2.50	7	22	1723	188.6	5.86
Q96BM9	ADP-ribosylation factor-like protein 8A OS=Homo sapiens GN=ARL8A PE=1 SV=1 - [ARL8A_HUMAN]	28.49	1	2	6	17	0.855	0.936	0.804	0.586	31.82	28.49	9	17	186	21.4	7.77
Q15646	2'-5'-oligoadenylate synthase-like protein OS=Homo sapiens GN=OASL PE=1 SV=2 - [OASL_HUMAN]	2.33	1	1	1	1				0.587	2.47	2.33	1	1	514	59.2	7.87

Q86XA9	HEAT repeat-containing protein 5A OS=Homo sapiens GN=HEATR5A PE=1 SV=2 - [HTR5A_HUMAN]	2.89	1	5	5	15	0.887	0.832	1.030	0.587	37.26	2.89	7	15	2040	221.9	6.58
Q9NRA2	Sialin OS=Homo sapiens GN=SLC17A5 PE=1 SV=2 - [S17A5_HUMAN]	3.84	1	2	2	3	0.654	0.335	0.464	0.588	4.90	3.84	3	3	495	54.6	8.27
P02786	Transferrin receptor protein 1 OS=Homo sapiens GN=TFRC PE=1 SV=2 - [TFR1_HUMAN]	39.47	2	30	30	145	0.751	0.668	0.407	0.588	329.57	39.47	51	145	760	84.8	6.61
Q8IZ07	Ankyrin repeat domain-containing protein 13A OS=Homo sapiens GN=ANKRD13A PE=1 SV=3 - [AN13A_HUMAN]	38.14	1	17	17	57	0.612	0.874	1.096	0.589	139.96	38.14	26	57	590	67.6	5.02
Q04721	Neurogenic locus notch homolog protein 2 OS=Homo sapiens GN=NOTCH2 PE=1 SV=3 - [NOTC2_HUMAN]	5.87	1	9	11	28	0.463	0.869	0.946	0.589	65.28	5.87	19	28	2471	265.2	5.14
P54577	Tyrosine--tRNA ligase, cytoplasmic OS=Homo sapiens GN=YARS PE=1 SV=4 - [SYYC_HUMAN]	57.20	1	36	37	136	0.587	0.772	0.763	0.589	300.07	57.20	58	136	528	59.1	7.05
Q96G01	Protein bicaudal D homolog 1 OS=Homo sapiens GN=BICD1 PE=1 SV=3 - [BICD1_HUMAN	2.36	1	1	2	4	1.575	0.778	0.877	0.589	7.80	2.36	3	4	975	110.7	5.81
P0C874	Putative spermatogenesis-associated protein 31D3 OS=Homo sapiens GN=SPATA31D3 PE=5 SV=1 - [S31D3_HUMAN]	4.47	2	2	2	2	2.008	1.641	1.390	0.590	4.63	4.47	2	2	917	102.4	8.07

Q9NYC9	Dynein heavy chain 9, axonemal OS=Homo sapiens GN=DNAH9 PE=1 SV=3 - [DYH9_HUMAN]	1.09	1	2	7	34	0.533	0.797	0.978	0.591	73.38	1.09	8	34	4486	511.6	5.91
O60238	BCL2/adenovirus E1B 19 kDa protein-interacting protein 3-like OS=Homo sapiens GN=BNIP3L PE=1 SV=1 - [BNI3L_HUMAN]	10.50	1	1	1	3	1.658	0.993	1.565	0.591	13.46	10.50	1	3	219	23.9	5.85
P51797	Chloride transport protein 6 OS=Homo sapiens GN=CLCN6 PE=1 SV=2 - [CLCN6_HUMAN]	1.27	1	1	1	1	1.296	1.069	0.663	0.592	3.42	1.27	1	1	869	97.2	6.81
O14513	Nck-associated protein 5 OS=Homo sapiens GN=NCKAP5 PE=1 SV=2 - [NCKP5_HUMAN]	0.58	1	1	1	1	2.294	0.935	0.991	0.592	2.64	0.58	1	1	1909	208.4	8.07
Q9HC35	Echinoderm microtubule-associated protein-like 4 OS=Homo sapiens GN=EML4 PE=1 SV=3 - [EMAL4_HUMAN]	24.87	1	21	22	79	0.631	0.877	1.064	0.593	171.42	24.87	36	79	981	108.8	6.40
P60981	Destrin OS=Homo sapiens GN=DSTN PE=1 SV=3 - [DEST_HUMAN]	66.06	1	15	17	139	0.928	0.725	0.983	0.593	308.88	66.06	26	139	165	18.5	7.85
Q9NVC3	Putative sodium-coupled neutral amino acid transporter 7 OS=Homo sapiens GN=SLC38A7 PE=2 SV=1 - [S38A7_HUMAN	2.60	1	1	1	7	0.537	0.445	0.170	0.593	21.89	2.60	1	7	462	49.9	5.08
P63172	Dynein light chain Tctex-type 1 OS=Homo sapiens GN=DYNLT1 PE=1 SV=1 - [DYLT1_HUMAN	14.16	1	1	1	7	0.672	1.140	1.250	0.593	33.22	14.16	2	7	113	12.4	5.08
Q9Y2T6	G-protein coupled receptor 55 OS=Homo sapiens GN=GPR55 PE=1 SV=2 - [GPR55_HUMAN]	3.76	1	1	1	1	1.279	0.126	0.315	0.593	1.62	3.76	1	1	319	36.6	8.43

P11308	Transcriptional regulator ERG OS=Homo sapiens GN=ERG PE=1 SV=2 - [ERG_HUMAN]	3.50	1	1	1	1	0.770	1.036	1.331	0.593	0.00	3.50	1	1	486	54.6	7.47
Q5T013	Putative hydroxypyruvate isomerase OS=Homo sapiens GN=HYI PE=1 SV=2 - [HYI_HUMAN]	19.13	1	5	5	13	1.079	0.786	0.896	0.594	27.99	19.13	8	13	277	30.4	5.50
P43003	Excitatory amino acid transporter 1 OS=Homo sapiens GN=SLC1A3 PE=1 SV=1 - [EAA1_HUMAN]	5.72	1	1	2	2	0.363	0.524	0.718	0.594	2.57	5.72	2	2	542	59.5	8.41
Q86YW9	Mediator of RNA polymerase II transcription subunit 12-like protein OS=Homo sapiens GN=MED12L PE=1 SV=2 - [MD12L_HUMAN]	1.03	1	2	2	4	0.723	0.591	0.869	0.594	0.00	1.03	2	4	2145	240.0	7.77
O75335	Liprin-alpha-4 OS=Homo sapiens GN=PPFIA4 PE=2 SV=3 - [LIPA4_HUMAN]	7.00	3	2	8	25	0.877	0.650	0.800	0.594	67.42	7.00	11	25	1185	134.3	6.74
P24592	Insulin-like growth factor-binding protein 6 OS=Homo sapiens GN=IGFBP6 PE=1 SV=1 - [IBP6_HUMAN]	4.17	1	1	1	2	0.775	0.987	1.145	0.595	4.17	4.17	1	2	240	25.3	7.81
Q9UDY8	Mucosa-associated lymphoid tissue lymphoma translocation protein 1 OS=Homo sapiens GN=MALT1 PE=1 SV=1 - [MALT1_HUMAN]	7.65	1	5	6	23	0.894	0.422	0.685	0.595	47.52	7.65	9	23	824	92.2	5.73
P11216	Glycogen phosphorylase, brain form OS=Homo sapiens GN=PYGB PE=1 SV=5 - [PYGB_HUMAN]	45.08	1	30	39	233	0.857	1.329	1.069	0.595	555.80	45.08	63	233	843	96.6	6.86

Q86UW6	NEDD4-binding protein 2 OS=Homo sapiens GN=N4BP2 PE=1 SV=2 - [N4BP2_HUMAN]	1.69	2	1	3	4	0.442	1.020	0.834	0.595	4.59	1.69	3	4	1770	198.7	5.21
O60437	Periplakin OS=Homo sapiens GN=PPL PE=1 SV=4 - [PEPL_HUMAN]	8.71	1	14	16	41	0.404	2.566	0.684	0.597	94.31	8.71	22	41	1756	204.6	5.60
Q00534	Cyclin-dependent kinase 6 OS=Homo sapiens GN=CDK6 PE=1 SV=1 - [CDK6_HUMAN]	17.18	6	4	5	21	0.524	0.787	0.950	0.597	49.97	17.18	9	21	326	36.9	6.46
O14544	Suppressor of cytokine signaling 6 OS=Homo sapiens GN=SOCS6 PE=1 SV=2 - [SOCS6_HUMAN]	1.87	1	1	2	2	0.774	1.177	1.345	0.599	4.09	1.87	2	2	535	59.5	7.25
Q9Y279	V-set and immunoglobulin domain-containing protein 4 OS=Homo sapiens GN=VSIG4 PE=1 SV=1 - [VSIG4_HUMAN]	2.51	1	1	1	2	0.478	0.722	0.912	0.599	1.93	2.51	1	2	399	44.0	6.35
Q6UXY1	Brain-specific angiogenesis inhibitor 1-associated protein 2-like protein 2 OS=Homo sapiens GN=BAIAP2L2 PE=1 SV=1 - [BI2L2_HUMAN]	7.75	1	2	2	4	0.910	0.615	1.186	0.599	3.03	7.75	2	4	529	58.9	9.51
Q96EQ0	Small glutamine-rich tetratricopeptide repeat-containing protein beta OS=Homo sapiens GN=SGTB PE=1 SV=1 - [SGTB_HUMAN]	9.54	1	3	3	7	0.381	0.958	1.038	0.599	20.22	9.54	4	7	304	33.4	4.92
Q16527	Cysteine and glycine-rich protein 2 OS=Homo sapiens GN=CSRP2 PE=1 SV=3 - [CSRP2_HUMAN]	63.21	1	12	12	125	0.650	0.569	1.789	0.599	324.50	63.21	22	125	193	20.9	8.62

Q99952	Tyrosine-protein phosphatase non-receptor type 18 OS=Homo sapiens GN=PTPN18 PE=1 SV=2 - [PTN18_HUMAN]	6.96	1	2	2	5	0.678	1.008	1.029	0.600	5.16	6.96	2	5	460	50.5	8.25
Q01813	ATP-dependent 6-phosphofructokinase, platelet type OS=Homo sapiens GN=PFKP PE=1 SV=2 - [PFKAP_HUMAN]	43.62	1	31	35	221	1.017	1.101	1.056	0.600	494.98	43.62	58	221	784	85.5	7.55
A8TX70	Collagen alpha-5(VI) chain OS=Homo sapiens GN=COL6A5 PE=1 SV=1 - [CO6A5_HUMAN]	1.34	1	3	4	4	0.958	1.054	0.901	0.601	1.66	1.34	4	4	2615	289.7	6.95
Q5JSL3	Dedicator of cytokinesis protein 11 OS=Homo sapiens GN=DOCK11 PE=1 SV=2 - [DOC11_HUMAN]	3.23	1	5	5	14	0.633	1.185	1.087	0.601	28.77	3.23	7	14	2073	237.5	7.74
O00507	Probable ubiquitin carboxyl-terminal hydrolase FAF-Y OS=Homo sapiens GN=USP9Y PE=2 SV=2 - [USP9Y_HUMAN]	6.18	3	2	17	36	1.409	1.003	0.700	0.602	81.35	6.18	27	36	2555	290.9	5.86
O43852	Calumenin OS=Homo sapiens GN=CALU PE=1 SV=2 - [CALU_HUMAN]	75.24	1	27	27	620	0.631	0.656	0.759	0.603	1784.81	75.24	43	620	315	37.1	4.64
Q14667	UPF0378 protein KIAA0100 OS=Homo sapiens GN=KIAA0100 PE=1 SV=3 - [K0100_HUMAN]	2.60	1	2	4	4	0.736	1.266	1.951	0.604	4.99	2.60	4	4	2235	253.5	7.14
P12277	Creatine kinase B-type OS=Homo sapiens GN=CKB PE=1 SV=1 - [KCRB_HUMAN]	43.04	3	13	14	48	0.974	3.016	1.067	0.604	127.26	43.04	20	48	381	42.6	5.59
Q05315	Galectin-10 OS=Homo sapiens GN=CLC PE=1 SV=3 - [LEG10_HUMAN]	4.23	1	1	1	1	0.763	1.467	1.010	0.604	0.00	4.23	1	1	142	16.4	7.37

P00338	L-lactate dehydrogenase A chain OS=Homo sapiens GN=LDHA PE=1 SV=2 - [LDHA_HUMAN]	70.48	2	26	30	616	0.981	0.841	0.689	0.604	1335.66	70.48	49	616	332	36.7	8.27
Q13637	Ras-related protein Rab-32 OS=Homo sapiens GN=RAB32 PE=1 SV=3 - [RAB32_HUMAN]	36.89	1	7	7	24	0.946	0.626	0.996	0.605	54.22	36.89	12	24	225	25.0	6.54
Q6P2C0	WD repeat-containing protein 93 OS=Homo sapiens GN=WDR93 PE=2 SV=1 - [WDR93_HUMAN]	1.60	1	1	1	1	0.620	0.677	0.524	0.605	2.48	1.60	1	1	686	77.3	6.39
Q9NZJ9	Diphosphoinositol polyphosphate phosphohydrolase 2 OS=Homo sapiens GN=NUDT4 PE=1 SV=2 - [NUDT4_HUMAN]	32.22	1	2	6	23	0.707	1.754	1.293	0.605	57.29	32.22	9	23	180	20.3	6.35
P09544	Protein Wnt-2 OS=Homo sapiens GN=WNT2 PE=1 SV=1 - [WNT2_HUMAN]	3.89	1	1	1	1		0.960	1.247	0.605	3.74	3.89	1	1	360	40.4	8.70
Q8WUY3	Protein prune homolog 2 OS=Homo sapiens GN=PRUNE2 PE=1 SV=3 - [PRUN2_HUMAN]	4.27	1	10	10	22	0.494	2.139	1.367	0.605	58.79	4.27	14	22	3088	340.4	4.45
Q9ULT0	Tetratricopeptide repeat protein 7A OS=Homo sapiens GN=TTC7A PE=1 SV=3 - [TTC7A_HUMAN	5.36	1	4	4	4	0.695	0.732	0.623	0.605	8.73	5.36	4	4	858	96.1	6.46
Q9UIF9	Bromodomain adjacent to zinc finger domain protein 2A OS=Homo sapiens GN=BAZ2A PE=1 SV=4 - [BAZ2A_HUMAN]	0.79	1	2	2	2	0.717	1.104	1.231	0.605	2.13	0.79	2	2	1905	211.1	6.64
Q12979	Active breakpoint cluster region-related protein OS=Homo sapiens GN=ABR PE=2 SV=2 - [ABR_HUMAN]	8.85	1	5	7	15	0.881	0.781	0.898	0.605	24.21	8.85	9	15	859	97.5	6.55

Q8N5M1	ATP synthase mitochondrial F1 complex assembly factor 2 OS=Homo sapiens GN=ATPAF2 PE=1 SV=1 - [ATPF2_HUMAN]	4.15	1	1	1	4	0.906	0.613	0.677	0.606	10.65	4.15	2	4	289	32.8	7.09
Q86Y37	CDK2-associated and cullin domain-containing protein 1 OS=Homo sapiens GN=CACUL1 PE=1 SV=1 - [CACL1_HUMAN]	8.13	1	2	2	2	0.928	0.514	1.018	0.606	5.96	8.13	2	2	369	41.0	5.19
Q68D10	Protein SPT2 homolog OS=Homo sapiens GN=SPTY2D1 PE=1 SV=3 - [SPT2_HUMAN]	7.01	1	3	4	4	0.793	0.904	0.846	0.606	9.44	7.01	4	4	685	75.6	9.79
Q96CJ1	ELL-associated factor 2 OS=Homo sapiens GN=EAF2 PE=1 SV=1 - [EAF2_HUMAN]	3.08	1	1	1	1	0.893	1.348	1.499	0.606	2.41	3.08	1	1	260	28.8	5.17
Q10472	Polypeptide N-acetylgalactosaminyltransferase 1 OS=Homo sapiens GN=GALNT1 PE=1 SV=1 - [GALT1_HUMAN]	11.09	1	4	5	14	0.446	0.849	0.515	0.607	40.16	11.09	8	14	559	64.2	7.72
Q9Y3B3	Transmembrane emp24 domain-containing protein 7 OS=Homo sapiens GN=TMED7 PE=1 SV=2 - [TMED7_HUMA	26.79	1	5	5	22	0.913	0.985	0.903	0.607	69.08	26.79	8	22	224	25.2	6.89
Q75V66	Anoctamin-5 OS=Homo sapiens GN=ANO5 PE=1 SV=1 - [ANO5_HUMAN]	1.97	1	1	1	3	0.722	0.929	1.002	0.607	0.00	1.97	1	3	913	107.1	6.80
Q6IQ22	Ras-related protein Rab-12 OS=Homo sapiens GN=RAB12 PE=1 SV=3 - [RAB12_HUMAN]	33.61	2	6	7	32	0.573	0.850	0.865	0.607	72.88	33.61	10	32	244	27.2	8.41
Q9GZZ8	Extracellular glycoprotein lacritin OS=Homo sapiens GN=LACRT PE=1 SV=1 - [LACRT_HUMAN]	8.70	1	1	1	1	1.493	1.043	1.669	0.607	2.32	8.70	1	1	138	14.2	5.50

Q6DD88	Atlastin-3 OS=Homo sapiens GN=ATL3 PE=1 SV=1 - [ATLA3_HUMAN]	40.67	1	19	19	88	0.844	1.144	0.803	0.608	222.27	40.67	29	88	541	60.5	5.66
Q96MX3	Zinc finger protein 48 OS=Homo sapiens GN=ZNF48 PE=1 SV=2 - [ZNF48_HUMAN]	1.62	1	1	1	1	1.668	0.978	0.719	0.608	1.99	1.62	1	1	618	67.8	9.36
P29536	Leiomodin-1 OS=Homo sapiens GN=LMOD1 PE=1 SV=3 - [LMOD1_HUMAN]	30.50	1	22	22	73	0.603	2.100	1.979	0.609	148.47	30.50	31	73	600	67.0	9.33
O95490	Latrophilin-2 OS=Homo sapiens GN=LPHN2 PE=1 SV=2 - [LPHN2_HUMAN]	0.89	1	1	1	1	1.008	1.231	0.945	0.609	2.82	0.89	1	1	1459	163.2	6.43
Q9Y3S2	Zinc finger protein 330 OS=Homo sapiens GN=ZNF330 PE=1 SV=1 - [ZN330_HUMAN]	11.25	1	4	4	5	0.645	0.757	0.918	0.609	11.80	11.25	4	5	320	36.2	6.16
Q14192	Four and a half LIM domains protein 2 OS=Homo sapiens GN=FHL2 PE=1 SV=3 - [FHL2_HUMAN]	54.84	1	15	15	77	1.772	0.802	0.950	0.609	167.99	54.84	22	77	279	32.2	7.55
Q9UBI6	Guanine nucleotide-binding protein G(I)/G(S)/G(O) subunit gamma-12 OS=Homo sapiens GN=GNG12 PE=1 SV=3 - [GBG12_HUMAN]	70.83	1	6	6	40	0.685	0.804	0.816	0.609	83.19	70.83	11	40	72	8.0	8.97
P14209	CD99 antigen OS=Homo sapiens GN=CD99 PE=1 SV=1 - [CD99_HUMAN]	13.51	1	2	2	8	2.471	1.071	0.854	0.610	22.57	13.51	4	8	185	18.8	4.75
O60939	Sodium channel subunit beta-2 OS=Homo sapiens GN=SCN2B PE=1 SV=1 - [SCN2B_HUMAN	3.26	1	1	1	6	0.741	1.163	1.155	0.610	10.67	3.26	2	6	215	24.3	6.39
Q5TBB1	Ribonuclease H2 subunit B OS=Homo sapiens GN=RNASEH2B PE=1 SV=1 - [RNH2B_HUMAN]	3.85	1	1	1	4	0.956	1.174	1.419	0.610	9.58	3.85	2	4	312	35.1	9.13

Q5R372	Rab GTPase-activating protein 1-like OS=Homo sapiens GN=RABGAP1L PE=1 SV=1 - [RBG1L_HUMAN]	9.08	1	6	7	20	0.812	1.188	1.267	0.610	46.32	9.08	9	20	815	92.5	5.31
P31483	Nucleolysin TIA-1 isoform p40 OS=Homo sapiens GN=TIA1 PE=1 SV=3 - [TIA1_HUMAN]	9.59	1	1	4	8	1.028	0.582	1.037	0.611	22.55	9.59	5	8	386	42.9	7.74
Q96D70	R3H domain-containing protein 4 OS=Homo sapiens GN=R3HDM4 PE=1 SV=3 - [R3HD4_HUMAN]	2.61	1	1	1	1	0.450	0.278	0.663	0.611	2.09	2.61	1	1	268	30.3	8.56
Q9H8T0	AKT-interacting protein OS=Homo sapiens GN=AKTIP PE=1 SV=1 - [AKTIP_HUMAN]	2.74	1	1	1	2	1.076	1.470	1.362	0.611	3.49	2.74	1	2	292	33.1	9.31
Q8TC99	Fibronectin type III domain-containing protein 8 OS=Homo sapiens GN=FNDC8 PE=2 SV=2 - [FNDC8_HUMAN	3.09	1	1	1	1	0.432	1.322	2.689	0.612	2.32	3.09	1	1	324	35.9	5.08
Q8NG48	Protein Lines homolog OS=Homo sapiens GN=LINS PE=2 SV=2 - [LINES_HUMAN]	2.91	1	1	1	1	0.861	0.983	1.225	0.612	0.00	2.91	1	1	757	85.8	6.52
O60443	Non-syndromic hearing impairment protein 5 OS=Homo sapiens GN=DFNA5 PE=1 SV=2 - [DFNA5_HUMAN]	27.02	1	12	12	39	0.510	0.541	0.803	0.612	84.99	27.02	22	39	496	54.5	5.17
Q9UBS3	DnaJ homolog subfamily B member 9 OS=Homo sapiens GN=DNAJB9 PE=1 SV=1 - [DNJB9_HUMAN]	6.28	1	1	1	1	0.612	0.789	0.807	0.613	4.69	6.28	1	1	223	25.5	8.27
Q9H5V8	CUB domain-containing protein 1 OS=Homo sapiens GN=CDCP1 PE=1 SV=3 - [CDCP1_HUMAN]	9.93	1	3	3	3	0.518	1.217	0.662	0.613	0.00	9.93	3	3	836	92.9	7.96

Q8IXM6	Nurim OS=Homo sapiens GN=NRM PE=1 SV=1 - [NRM_HUMAN]	6.11	1	1	1	1	1.064	1.017	1.219	0.613	0.00	6.11	1	1	262	29.4	8.63
P49591	Serine--tRNA ligase, cytoplasmic OS=Homo sapiens GN=SARS PE=1 SV=3 - [SYSC_HUMAN]	27.82	1	15	15	35	0.868	0.908	1.233	0.614	92.57	27.82	21	35	514	58.7	6.43
P30536	Translocator protein OS=Homo sapiens GN=TSPO PE=1 SV=3 - [TSPOA_HUMAN]	9.47	1	2	2	11	0.631	0.488	0.652	0.614	24.05	9.47	3	11	169	18.8	9.36
P08195	4F2 cell-surface antigen heavy chain OS=Homo sapiens GN=SLC3A2 PE=1 SV=3 - [4F2_HUMAN]	28.73	1	17	17	84	0.767	0.404	0.597	0.614	225.03	28.73	30	84	630	68.0	5.01
P49641	Alpha-mannosidase 2x OS=Homo sapiens GN=MAN2A2 PE=2 SV=3 - [MA2A2_HUMAN]	2.17	1	3	3	6	1.045	1.118	1.127	0.614	9.27	2.17	5	6	1150	130.5	6.84
Q9UGI8	Testin OS=Homo sapiens GN=TES PE=1 SV=1 - [TES_HUMAN]	44.66	1	22	22	113	1.084	1.295	1.190	0.614	262.52	44.66	36	113	421	48.0	7.68
Q7L8C5	Synaptotagmin-13 OS=Homo sapiens GN=SYT13 PE=1 SV=1 - [SYT13_HUMAN]	4.46	1	1	1	1	1.899	1.513	0.999	0.614	0.00	4.46	1	1	426	46.9	7.66
P02462	Collagen alpha-1(IV) chain OS=Homo sapiens GN=COL4A1 PE=1 SV=3 - [CO4A1_HUMAN]	5.33	1	4	6	19	0.463	0.817	1.322	0.615	46.60	5.33	12	19	1669	160.5	8.28
Q8NBM8	Prenylcysteine oxidase-like OS=Homo sapiens GN=PCYOX1L PE=1 SV=2 - [PCYXL_HUMAN]	4.25	1	2	2	3	0.719	1.208	0.917	0.615	5.87	4.25	3	3	494	54.6	7.31
Q5VZK9	Leucine-rich repeat-containing protein 16A OS=Homo sapiens GN=LRRC16A PE=1 SV=1 - [LR16A_HUMAN]	16.12	1	19	21	43	0.806	0.799	0.983	0.616	100.36	16.12	31	43	1371	151.5	7.85

O14972	Down syndrome critical region protein 3 OS=Homo sapiens GN=DSCR3 PE=2 SV=1 - [DSCR3_HUMAN]	9.76	1	2	2	8	0.876	1.252	0.887	0.617	25.26	9.76	3	8	297	33.0	7.68
O95340	Bifunctional 3'-phosphoadenosine 5'-phosphosulfate synthase 2 OS=Homo sapiens GN=PAPSS2 PE=1 SV=2 - [PAPS2_HUMAN]	32.25	1	16	18	105	0.958	0.567	0.702	0.617	258.82	32.25	28	105	614	69.5	8.03
Q4LDE5	Sushi, von Willebrand factor type A, EGF and pentraxin domain-containing protein 1 OS=Homo sapiens GN=SVEP1 PE=1 SV=3 - [SVEP1_HUMAN]	10.14	1	32	32	98	0.628	0.447	0.688	0.617	253.38	10.14	52	98	3571	389.9	5.50
Q7L5N7	Lysophosphatidylcholine acyltransferase 2 OS=Homo sapiens GN=LPCAT2 PE=1 SV=1 - [PCAT2_HUMAN]	5.15	1	3	3	7	0.845	1.001	0.808	0.617	13.21	5.15	5	7	544	60.2	6.55
Q12767	Uncharacterized protein KIAA0195 OS=Homo sapiens GN=KIAA0195 PE=1 SV=1 - [K0195_HUMAN]	3.76	1	2	2	5	1.358	0.588	1.083	0.618	11.78	3.76	3	5	1356	151.1	6.44
Q68DD2	Cytosolic phospholipase A2 zeta OS=Homo sapiens GN=PLA2G4F PE=2 SV=3 - [PA24F_HUMAN]	1.30	1	1	1	1	0.411	0.479	0.877	0.618	0.00	1.30	1	1	849	95.0	5.44
P42224	Signal transducer and activator of transcription 1-alpha/beta OS=Homo sapiens GN=STAT1 PE=1 SV=2 - [STAT1_HUMAN]	57.87	1	50	50	800	1.361	0.465	1.063	0.618	2221.76	57.87	88	800	750	87.3	6.05

Q10571	Probable tumor suppressor protein MN1 OS=Homo sapiens GN=MN1 PE=1 SV=3 - [MN1_HUMAN]	0.53	1	1	1	1	0.523	0.996	0.880	0.618	0.00	0.53	1	1	1320	135.9	6.71
P13761	HLA class II histocompatibility antigen, DRB1-7 beta chain OS=Homo sapiens GN=HLA-DRB1 PE=1 SV=1 - [2B17_HUMAN]	6.77	7	1	2	2	1.157	2.197	1.196	0.618	2.01	6.77	2	2	266	29.8	7.44
Q5HYA8	Meckelin OS=Homo sapiens GN=TMEM67 PE=1 SV=2 - [MKS3_HUMAN]	1.21	1	1	1	1	0.904	0.786	0.603	0.619	0.00	1.21	1	1	995	111.7	6.77
Q9UKU0	Long-chain-fatty-acid--CoA ligase 6 OS=Homo sapiens GN=ACSL6 PE=2 SV=4 - [ACSL6_HUMAN]	5.60	1	2	4	10	0.725	0.724	1.170	0.619	14.17	5.60	6	10	697	77.7	7.43
Q5T4B2	Probable inactive glycosyltransferase 25 family member 3 OS=Homo sapiens GN=CERCAM PE=2 SV=1 - [GT253_HUMAN]	12.44	1	7	7	37	0.763	0.689	0.973	0.619	87.43	12.44	14	37	595	67.5	6.06
P61225	Ras-related protein Rap-2b OS=Homo sapiens GN=RAP2B PE=1 SV=1 - [RAP2B_HUMAN]	31.15	1	5	7	19	0.633	0.866	0.544	0.620	46.38	31.15	11	19	183	20.5	4.81
Q8TDB4	Protein MGARP OS=Homo sapiens GN=MGARP PE=1 SV=1 - [HUMMR_HUMAN]	21.67	1	4	4	20	0.862	0.458	0.917	0.620	46.59	21.67	6	20	240	25.4	4.44
Q96J65	Multidrug resistance-associated protein 9 OS=Homo sapiens GN=ABCC12 PE=1 SV=2 - [MRP9_HUMAN]	2.72	1	2	3	13	0.655	0.624	1.299	0.621	27.91	2.72	4	13	1359	152.2	8.35
O43157	Plexin-B1 OS=Homo sapiens GN=PLXNB1 PE=1 SV=3 - [PLXB1_HUMAN]	5.34	2	10	10	25	0.976	0.867	0.971	0.622	43.88	5.34	14	25	2135	232.2	5.49

Q15118	[Pyruvate dehydrogenase (acetyl-transferring)] kinase isozyme 1, mitochondrial OS=Homo sapiens GN=PDK1 PE=1 SV=1 - [PDK1_HUMAN]	5.05	1	2	2	3	0.828	1.418	0.971	0.622	6.99	5.05	3	3	436	49.2	8.81
Q9Y3B8	Oligoribonuclease, mitochondrial OS=Homo sapiens GN=REXO2 PE=1 SV=3 - [ORN_HUMAN]	54.85	1	14	14	102	0.740	0.999	0.970	0.622	274.67	54.85	24	102	237	26.8	6.87
O14893	Gem-associated protein 2 OS=Homo sapiens GN=GEMIN2 PE=1 SV=1 - [GEMI2_HUMAN]	11.43	1	2	2	4	1.108	1.170	1.375	0.623	11.72	11.43	3	4	280	31.6	5.58
P0C1S8	Wee1-like protein kinase 2 OS=Homo sapiens GN=WEE2 PE=2 SV=2 - [WEE2_HUMAN]	3.70	1	2	2	2	0.670	0.444	0.580	0.623	0.00	3.70	2	2	567	62.9	6.51
Q9Y5U9	Immediate early response 3-interacting protein 1 OS=Homo sapiens GN=IER3IP1 PE=1 SV=1 - [IR3IP_HUMAN]	34.15	1	2	2	6	0.855	0.592	0.536	0.624	25.39	34.15	2	6	82	9.0	8.22
Q9Y570	Protein phosphatase methylesterase 1 OS=Homo sapiens GN=PPME1 PE=1 SV=3 - [PPME1_HUMAN]	44.82	1	13	14	56	1.274	0.550	0.858	0.625	131.14	44.82	23	56	386	42.3	5.97
Q92629	Delta-sarcoglycan OS=Homo sapiens GN=SGCD PE=1 SV=2 - [SGCD_HUMAN]	40.14	1	10	10	30	1.178	0.543	0.809	0.625	62.64	40.14	15	30	289	32.1	9.11
Q92484	Acid sphingomyelinase-like phosphodiesterase 3a OS=Homo sapiens GN=SMPDL3A PE=1 SV=2 - [ASM3A_HUMAN]	10.38	1	5	5	11	1.538	0.866	0.372	0.626	30.76	10.38	7	11	453	51.2	6.33

Q8N0W3	L-fucose kinase OS=Homo sapiens GN=FUK PE=2 SV=2 - [FUK_HUMAN]	0.74	1	1	1	1	0.470	1.178	0.784	0.626	1.98	0.74	1	1	1084	117.5	6.25
O60513	Beta-1,4-galactosyltransferase 4 OS=Homo sapiens GN=B4GALT4 PE=1 SV=1 - [B4GT4_HUMAN]	6.40	1	2	2	5	0.565	0.539	0.899	0.626	17.37	6.40	2	5	344	40.0	9.07
O75506	Heat shock factor-binding protein 1 OS=Homo sapiens GN=HSBP1 PE=1 SV=1 - [HSBP1_HUMAN]	55.26	1	2	3	9	1.065	1.511	0.972	0.626	22.69	55.26	3	9	76	8.5	4.36
Q15375	Ephrin type-A receptor 7 OS=Homo sapiens GN=EPHA7 PE=1 SV=3 - [EPHA7_HUMAN]	3.81	8	2	4	5	0.806	0.845	1.162	0.627	8.80	3.81	5	5	998	112.0	5.80
Q96MG8	Protein-L-isoaspartate O-methyltransferase domain-containing protein 1 OS=Homo sapiens GN=PCMTD1 PE=2 SV=2 - [PCMD1_HUMAN]	2.52	1	1	1	2	0.835	1.186	0.384	0.627	2.42	2.52	2	2	357	40.6	5.66
Q14573	Inositol 1,4,5-trisphosphate receptor type 3 OS=Homo sapiens GN=ITPR3 PE=1 SV=2 - [ITPR3_HUMAN]	2.77	1	3	9	20	0.807	1.884	1.217	0.627	47.63	2.77	12	20	2671	303.9	6.48
Q9NX08	COMM domain-containing protein 8 OS=Homo sapiens GN=COMMD8 PE=1 SV=1 - [COMD8_HUMAN]	26.23	1	4	5	16	0.494	0.439	0.569	0.627	36.58	26.23	9	16	183	21.1	5.43
Q6UWY0	Arylsulfatase K OS=Homo sapiens GN=ARSK PE=1 SV=1 - [ARSK_HUMAN]	6.34	1	3	3	8	1.228	1.370	1.241	0.628	21.95	6.34	6	8	536	61.4	8.92
P53675	Clathrin heavy chain 2 OS=Homo sapiens GN=CLTCL1 PE=1 SV=2 - [CLH2_HUMAN]	10.12	1	1	19	209	1.289	0.171	0.286	0.628	509.60	10.12	32	209	1640	186.9	5.85

Q6UWL6	Kin of IRRE-like protein 2 OS=Homo sapiens GN=KIRREL2 PE=1 SV=2 - [KIRR2_HUMAN]	2.26	1	1	1	1	1.047	0.434	0.895	0.628	2.35	2.26	1	1	708	75.0	6.93
Q6UX72	UDP-GlcNAc:betaGal beta-1,3-N-acetylglucosaminyltransferase 9 OS=Homo sapiens GN=B3GNT9 PE=2 SV=1 - [B3GN9_HUMAN]	13.93	1	3	3	5	0.947	0.842	0.609	0.628	9.99	13.93	4	5	402	43.7	9.17
P52790	Hexokinase-3 OS=Homo sapiens GN=HK3 PE=1 SV=2 - [HXK3_HUMAN]	3.47	1	1	3	11	0.592	0.871	0.769	0.629	26.83	3.47	4	11	923	99.0	5.40
Q9HD26	Golgi-associated PDZ and coiled-coil motif-containing protein OS=Homo sapiens GN=GOPC PE=1 SV=1 - [GOPC_HUMAN]	36.80	1	15	15	29	0.756	0.692	0.724	0.629	79.54	36.80	21	29	462	50.5	5.92
O95405	Zinc finger FYVE domain-containing protein 9 OS=Homo sapiens GN=ZFYVE9 PE=1 SV=2 - [ZFYV9_HUMAN]	0.63	1	1	1	2	0.713	0.717	1.033	0.629	4.61	0.63	2	2	1425	156.3	5.01
Q8WXC3	Pyrin domain-containing protein 1 OS=Homo sapiens GN=PYDC1 PE=1 SV=1 - [PYDC1_HUMAN]	15.73	1	1	1	1	1.327	1.130	1.310	0.630	2.02	15.73	1	1	89	10.1	6.79
Q86VQ1	Glucocorticoid-induced transcript 1 protein OS=Homo sapiens GN=GLCCI1 PE=1 SV=1 - [GLCI1_HUMAN]	6.40	1	1	1	2	1.186	1.603	1.802	0.630	3.44	6.40	1	2	547	58.0	9.44
Q9UL62	Short transient receptor potential channel 5 OS=Homo sapiens GN=TRPC5 PE=1 SV=1 - [TRPC5_HUMAN	1.95	1	1	1	1	1.651	1.040	0.974	0.631	0.00	1.95	1	1	973	111.3	7.30

Q9NRY6	Phospholipid scramblase 3 OS=Homo sapiens GN=PLSCR3 PE=1 SV=2 - [PLS3_HUMAN]	13.22	1	3	3	7	1.077	1.140	1.083	0.631	9.99	13.22	5	7	295	31.6	6.65
Q86WA9	Sodium-independent sulfate anion transporter OS=Homo sapiens GN=SLC26A11 PE=2 SV=2 - [S2611_HUMAN]	2.31	1	1	2	4	0.926	3.140	1.170	0.631	9.96	2.31	2	4	606	65.3	7.31
Q9H334	Forkhead box protein P1 OS=Homo sapiens GN=FOXP1 PE=1 SV=1 - [FOXP1_HUMAN]	1.33	1	1	1	2	0.954	1.495	1.362	0.631	0.00	1.33	1	2	677	75.3	6.67
P16035	Metalloproteinase inhibitor 2 OS=Homo sapiens GN=TIMP2 PE=1 SV=2 - [TIMP2_HUMAN]	6.82	1	1	1	6	0.599	0.908	0.826	0.631	10.22	6.82	2	6	220	24.4	7.49
Q14314	Fibroleukin OS=Homo sapiens GN=FGL2 PE=1 SV=1 - [FGL2_HUMAN]	12.07	1	5	6	10	1.025	6.621	1.757	0.631	18.01	12.07	8	10	439	50.2	7.39
P60891	Ribose-phosphate pyrophosphokinase 1 OS=Homo sapiens GN=PRPS1 PE=1 SV=2 - [PRPS1_HUMAN]	32.39	2	7	13	60	0.575	0.468	0.765	0.631	137.13	32.39	22	60	318	34.8	6.98
P40123	Adenylyl cyclase-associated protein 2 OS=Homo sapiens GN=CAP2 PE=1 SV=1 - [CAP2_HUMAN]	33.75	1	13	15	73	0.894	0.991	2.477	0.632	198.24	33.75	24	73	477	52.8	6.37
P48426	Phosphatidylinositol 5-phosphate 4-kinase type-2 alpha OS=Homo sapiens GN=PIP4K2A PE=1 SV=2 - [PI42A_HUMAN]	26.35	1	5	11	26	0.629	0.999	1.217	0.632	50.75	26.35	16	26	406	46.2	6.99

Q86UG4	Solute carrier organic anion transporter family member 6A1 OS=Homo sapiens GN=SLCO6A1 PE=2 SV=2 - [SO6A1_HUMAN]	1.25	1	1	1	1	0.873		3.062	0.632	0.00	1.25	1	1	719	79.2	8.59
P50281	Matrix metalloproteinase-14 OS=Homo sapiens GN=MMP14 PE=1 SV=3 - [MMP14_HUMAN]	12.89	3	8	8	30	0.719	0.883	0.769	0.633	57.45	12.89	14	30	582	65.9	7.77
O60688	Protein yippee-like 1 OS=Homo sapiens GN=YPEL1 PE=2 SV=1 - [YPEL1_HUMAN]	15.13	2	1	1	1	1.587	0.986	0.530	0.633	0.00	15.13	1	1	119	13.6	8.03
P07951	Tropomyosin beta chain OS=Homo sapiens GN=TPM2 PE=1 SV=1 - [TPM2_HUMAN]	83.80	1	23	51	1472	0.933	1.017	1.812	0.634	3382.28	83.80	80	1472	284	32.8	4.70
Q5R3I4	Tetratricopeptide repeat protein 38 OS=Homo sapiens GN=TTC38 PE=1 SV=1 - [TTC38_HUMAN	6.40	1	3	3	10	1.496	1.064	0.742	0.634	19.78	6.40	5	10	469	52.8	5.99
P30542	Adenosine receptor A1 OS=Homo sapiens GN=ADORA1 PE=1 SV=1 - [AA1R_HUMAN]	9.51	1	1	1	5	1.206	2.344	1.601	0.635	4.57	9.51	2	5	326	36.5	8.62
P56556	NADH dehydrogenase [ubiquinone] 1 alpha subcomplex subunit 6 OS=Homo sapiens GN=NDUFA6 PE=1 SV=3 - [NDUA6_HUMAN]	17.53	1	2	2	6	0.681	0.908	1.122	0.635	2.50	17.53	2	6	154	17.9	10.14
O14917	Protocadherin-17 OS=Homo sapiens GN=PCDH17 PE=2 SV=2 - [PCD17_HUMAN]	1.21	1	1	1	3	1.164	0.939	1.842	0.635	0.00	1.21	1	3	1159	126.2	5.19

A8K855	EF-hand calcium-binding domain-containing protein 7 OS=Homo sapiens GN=EFCAB7 PE=2 SV=1 - [EFCB7_HUMAN]	2.70	1	1	1	1	1.042	1.297	0.977	0.635	0.00	2.70	1	1	629	71.9	6.38
Q96CX2	BTB/POZ domain-containing protein KCTD12 OS=Homo sapiens GN=KCTD12 PE=1 SV=1 - [KCD12_HUMAN]	31.38	1	8	8	65	1.202	1.662	1.323	0.636	173.74	31.38	14	65	325	35.7	5.64
Q13563	Polycystin-2 OS=Homo sapiens GN=PKD2 PE=1 SV=3 - [PKD2_HUMAN]	12.71	1	11	11	27	0.520	0.817	0.906	0.636	64.62	12.71	19	27	968	109.6	5.69
P28329	Choline O-acetyltransferase OS=Homo sapiens GN=CHAT PE=1 SV=4 - [CLAT_HUMAN]	2.67	1	1	2	2	1.027	0.347	0.780	0.636	4.82	2.67	2	2	748	82.5	8.59
Q8IY82	Coiled-coil domain-containing protein lobo homolog OS=Homo sapiens GN=CCDC135 PE=2 SV=3 - [CC135_HUMAN]	1.95	1	2	3	3	0.831	1.251	1.295	0.636	7.28	1.95	3	3	874	103.4	5.67
Q8WUF5	RelA-associated inhibitor OS=Homo sapiens GN=PPP1R13L PE=1 SV=4 - [IASPP_HUMAN]	7.85	1	5	5	13	0.474	1.460	1.618	0.637	24.08	7.85	8	13	828	89.0	6.81
Q8NB12	Histone-lysine N-methyltransferase SMYD1 OS=Homo sapiens GN=SMYD1 PE=1 SV=1 - [SMYD1_HUMAN]	2.86	1	1	1	1	2.737	0.834	1.824	0.637	2.98	2.86	1	1	490	56.6	7.09
Q99424	Peroxisomal acyl-coenzyme A oxidase 2 OS=Homo sapiens GN=ACOX2 PE=1 SV=1 - [ACOX2_HUMAN]	8.96	1	5	5	17	0.546	0.916	1.226	0.637	30.50	8.96	7	17	681	76.8	7.56

Q9H5Y7	SLIT and NTRK-like protein 6 OS=Homo sapiens GN=SLITRK6 PE=2 SV=3 - [SLIK6_HUMAN]	0.71	1	1	1	1	1.201	0.826	0.746	0.638	2.35	0.71	1	1	841	95.0	6.52
Q96GE6	Calmodulin-like protein 4 OS=Homo sapiens GN=CALML4 PE=2 SV=3 - [CALL4_HUMAN]	6.12	1	1	1	3	0.822	0.907	0.637	0.638	4.41	6.12	1	3	196	21.9	7.53
O43657	Tetraspanin-6 OS=Homo sapiens GN=TSPAN6 PE=1 SV=1 - [TSN6_HUMAN]	3.27	1	1	1	2	0.986	0.616	0.938	0.638	4.05	3.27	2	2	245	27.5	8.10
Q9C0D2	Centrosomal protein of 295 kDa OS=Homo sapiens GN=CEP295 PE=2 SV=4 - [CE295_HUMAN]	1.08	2	2	4	7	1.185	1.144	1.173	0.638	14.16	1.08	4	7	2601	295.0	6.00
Q9NWX6	Probable tRNA(His) guanylyltransferase OS=Homo sapiens GN=THG1L PE=1 SV=2 - [THG1_HUMAN]	3.69	1	1	1	2	0.696	0.711	0.516	0.638	0.00	3.69	2	2	298	34.8	8.00
P04114	Apolipoprotein B-100 OS=Homo sapiens GN=APOB PE=1 SV=2 - [APOB_HUMAN]	1.82	2	7	9	28	0.438	0.568	0.913	0.639	44.22	1.82	13	28	4563	515.3	7.05
Q8N0Y7	Probable phosphoglycerate mutase 4 OS=Homo sapiens GN=PGAM4 PE=2 SV=1 - [PGAM4_HUMAN]	41.34	1	2	9	225	1.061	0.492	0.724	0.639	611.44	41.34	15	225	254	28.8	6.65
Q99985	Semaphorin-3C OS=Homo sapiens GN=SEMA3C PE=1 SV=2 - [SEM3C_HUMAN]	1.33	1	1	1	2	0.366	1.153	1.694	0.639	3.00	1.33	2	2	751	85.2	8.69
P08493	Matrix Gla protein OS=Homo sapiens GN=MGP PE=1 SV=2 - [MGP_HUMAN]	10.68	1	1	1	3	0.398	1.140	1.625	0.639	6.83	10.68	2	3	103	12.3	9.67

P28324	ETS domain-containing protein Elk-4 OS=Homo sapiens GN=ELK4 PE=1 SV=3 - [ELK4_HUMAN]	2.09	1	1	1	1	0.556	0.671	1.235	0.639	2.18	2.09	1	1	431	46.9	7.91
Q969T9	WW domain-binding protein 2 OS=Homo sapiens GN=WBP2 PE=1 SV=1 - [WBP2_HUMAN]	10.34	1	4	4	10	0.994	1.088	1.061	0.640	22.43	10.34	7	10	261	28.1	5.91
Q05707	Collagen alpha-1(XIV) chain OS=Homo sapiens GN=COL14A1 PE=1 SV=3 - [COEA1_HUMAN]	24.00	1	34	34	108	0.683	0.679	0.198	0.640	241.68	24.00	55	108	1796	193.4	5.30
P06737	Glycogen phosphorylase, liver form OS=Homo sapiens GN=PYGL PE=1 SV=4 - [PYGL_HUMAN]	39.43	1	27	34	136	0.739	0.678	0.725	0.640	329.26	39.43	56	136	847	97.1	7.17
Q13023	A-kinase anchor protein 6 OS=Homo sapiens GN=AKAP6 PE=1 SV=3 - [AKAP6_HUMAN]	1.55	1	2	2	5	0.973	0.212	0.398	0.641	1.66	1.55	2	5	2319	256.6	5.01
O75056	Syndecan-3 OS=Homo sapiens GN=SDC3 PE=1 SV=2 - [SDC3_HUMAN]	3.85	1	1	1	1	0.923	2.307	2.645	0.642	3.26	3.85	1	1	442	45.5	4.69
Q7Z4S6	Kinesin-like protein KIF21A OS=Homo sapiens GN=KIF21A PE=1 SV=2 - [KI21A_HUMAN]	1.37	2	1	2	6	0.702	0.583	0.964	0.642	17.09	1.37	2	6	1674	187.1	6.42
Q7Z406	Myosin-14 OS=Homo sapiens GN=MYH14 PE=1 SV=2 - [MYH14_HUMAN]	10.58	1	4	24	520	0.792	0.450	0.633	0.642	1258.43	10.58	39	520	1995	227.7	5.60
Q92968	Peroxisomal membrane protein PEX13 OS=Homo sapiens GN=PEX13 PE=1 SV=2 - [PEX13_HUMAN]	8.44	1	2	2	5	0.978	1.676	1.127	0.643	28.09	8.44	2	5	403	44.1	8.05

Q9C093	Sperm flagellar protein 2 OS=Homo sapiens GN=SPEF2 PE=1 SV=2 - [SPEF2_HUMAN]	2.74	2	4	6	8	1.023	0.525	0.774	0.643	17.50	2.74	6	8	1822	209.7	5.54
P54727	UV excision repair protein RAD23 homolog B OS=Homo sapiens GN=RAD23B PE=1 SV=1 - [RD23B_HUMAN]	30.07	1	11	14	218	0.642	0.974	0.868	0.643	517.82	30.07	23	218	409	43.1	4.84
Q86WT1	Tetratricopeptide repeat protein 30A OS=Homo sapiens GN=TTC30A PE=2 SV=3 - [TT30A_HUMAN]	3.01	1	1	1	1	1.060	0.790	1.530	0.643	4.31	3.01	1	1	665	76.1	5.20
Q9H7X7	Intraflagellar transport protein 22 homolog OS=Homo sapiens GN=IFT22 PE=2 SV=1 - [IFT22_HUMAN]	12.97	1	2	2	5	1.302	0.801	0.780	0.644	10.50	12.97	3	5	185	20.8	5.15
Q15833	Syntaxin-binding protein 2 OS=Homo sapiens GN=STXBP2 PE=1 SV=2 - [STXB2_HUMAN]	4.38	1	2	3	7	0.434	1.226	0.520	0.644	11.17	4.38	5	7	593	66.4	6.55
Q96AQ1	Coiled-coil domain-containing protein 74A OS=Homo sapiens GN=CCDC74A PE=2 SV=1 - [CC74A_HUMAN]	10.32	2	1	3	3	1.061	0.638	0.812	0.644	4.75	10.32	3	3	378	41.6	10.52
Q8NEZ3	WD repeat-containing protein 19 OS=Homo sapiens GN=WDR19 PE=1 SV=2 - [WDR19_HUMAN]	0.60	1	1	1	1	0.999	0.900	0.594	0.644	0.00	0.60	1	1	1342	151.5	6.35
P49589	Cysteine--tRNA ligase, cytoplasmic OS=Homo sapiens GN=CARS PE=1 SV=3 - [SYCC_HUMAN]	46.79	1	33	33	110	0.822	0.797	0.842	0.645	279.79	46.79	50	110	748	85.4	6.76
P62736	Actin, aortic smooth muscle OS=Homo sapiens GN=ACTA2 PE=1 SV=1 - [ACTA_HUMAN]	77.45	2	3	35	4891	0.721	1.176	2.659	0.645	#####	77.45	60	4891	377	42.0	5.39

A8MZF0	Proline-rich protein 33 OS=Homo sapiens GN=PRR33 PE=4 SV=2 - [PRR33_HUMAN]	4.83	1	1	1	4	0.724	0.531	0.770	0.646	3.21	4.83	2	4	331	35.2	8.35
Q9UPZ3	Hermansky-Pudlak syndrome 5 protein OS=Homo sapiens GN=HPS5 PE=1 SV=2 - [HPS5_HUMAN]	2.57	2	2	3	5	1.089	0.892	0.804	0.646	4.64	2.57	4	5	1129	127.4	5.54
P11047	Laminin subunit gamma-1 OS=Homo sapiens GN=LAMC1 PE=1 SV=3 - [LAMC1_HUMAN]	21.88	1	34	34	149	0.756	0.733	1.066	0.647	370.09	21.88	57	149	1609	177.5	5.12
Q9UNF0	Protein kinase C and casein kinase substrate in neurons protein 2 OS=Homo sapiens GN=PACSIN2 PE=1 SV=2 - [PACN2_HUMAN	42.59	1	18	19	63	0.861	1.063	1.232	0.648	151.45	42.59	29	63	486	55.7	5.20
Q9HCE0	Ectopic P granules protein 5 homolog OS=Homo sapiens GN=EPG5 PE=2 SV=2 - [EPG5_HUMAN]	1.09	1	2	2	3		0.983	1.173	0.648	8.03	1.09	3	3	2579	292.3	6.43
Q07837	Neutral and basic amino acid transport protein rBAT OS=Homo sapiens GN=SLC3A1 PE=1 SV=2 - [SLC31_HUMAN]	1.61	1	1	1	1	1.893	0.101	0.273	0.648	0.00	1.61	1	1	685	78.8	5.96
Q9UPQ0	LIM and calponin homology domains-containing protein 1 OS=Homo sapiens GN=LIMCH1 PE=1 SV=4 - [LIMC1_HUMAN]	35.36	1	34	34	116	1.648	0.551	0.479	0.648	272.48	35.36	51	116	1083	121.8	6.47
Q76LX8	A disintegrin and metalloproteinase with thrombospondin motifs 13 OS=Homo sapiens GN=ADAMTS13 PE=1 SV=1 - [ATS13_HUMAN]	0.63	1	1	1	1	0.742	0.776	1.075	0.648	2.10	0.63	1	1	1427	153.5	7.17

P35749	Myosin-11 OS=Homo sapiens GN=MYH11 PE=1 SV=3 - [MYH11_HUMAN]	28.65	1	24	62	942	0.320	0.713	0.482	0.648	2533.67	28.65	96	942	1972	227.2	5.50
Q9Y4H2	Insulin receptor substrate 2 OS=Homo sapiens GN=IRS2 PE=1 SV=2 - [IRS2_HUMAN]	5.08	1	3	4	8	0.959	1.031	0.928	0.648	16.50	5.08	5	8	1338	137.2	8.65
O14639	Actin-binding LIM protein 1 OS=Homo sapiens GN=ABLIM1 PE=1 SV=3 - [ABLM1_HUMAN]	6.94	1	3	4	5	0.738	2.067	1.197	0.648	7.08	6.94	5	5	778	87.6	8.59
Q8NHP7	Exonuclease 3'-5' domain-containing protein 1 OS=Homo sapiens GN=EXD1 PE=2 SV=4 - [EXD1_HUMAN]	4.09	1	2	2	3	1.243	1.031	1.134	0.649	2.57	4.09	2	3	514	58.3	5.69
P51884	Lumican OS=Homo sapiens GN=LUM PE=1 SV=2 - [LUM_HUMAN]	13.02	1	4	4	8	0.381	1.012	1.024	0.649	16.21	13.02	7	8	338	38.4	6.61
Q9Y4D2	Sn1-specific diacylglycerol lipase alpha OS=Homo sapiens GN=DAGLA PE=1 SV=3 - [DGLA_HUMAN]	1.82	1	2	2	2	0.671	0.665	0.769	0.649	0.00	1.82	2	2	1042	114.9	6.35
Q8IVH4	Methylmalonic aciduria type A protein, mitochondrial OS=Homo sapiens GN=MMAA PE=1 SV=1 - [MMAA_HUMAN]	5.74	1	2	2	3	0.888	6.371	1.732	0.649	8.33	5.74	2	3	418	46.5	9.29
Q9NP58	ATP-binding cassette sub-family B member 6, mitochondrial OS=Homo sapiens GN=ABCB6 PE=1 SV=1 - [ABCB6_HUMAN]	6.77	1	4	4	18	1.096	1.130	1.016	0.650	19.41	6.77	5	18	842	93.8	8.48
Q08AN1	Zinc finger protein 616 OS=Homo sapiens GN=ZNF616 PE=2 SV=2 - [ZN616_HUMAN]	8.58	120	1	3	9	0.348	1.083	0.656	0.650	12.73	8.58	4	9	781	90.2	9.57

Q86VP1	Tax1-binding protein 1 OS=Homo sapiens GN=TAX1BP1 PE=1 SV=2 - [TAXB1_HUMAN]	1.27	1	1	1	2	0.701	1.044	0.779	0.650	5.49	1.27	2	2	789	90.8	5.43
Q58WW2	DDB1- and CUL4-associated factor 6 OS=Homo sapiens GN=DCAF6 PE=1 SV=1 - [DCAF6_HUMAN	6.86	1	6	6	7	0.563	0.908	0.908	0.650	14.58	6.86	6	7	860	96.2	5.27
Q9GZP1	Neurensin-2 OS=Homo sapiens GN=NRSN2 PE=2 SV=1 - [NRSN2_HUMAN]	13.24	1	2	2	3	0.569	1.927	1.593	0.650	5.83	13.24	2	3	204	22.0	4.83
P17858	ATP-dependent 6-phosphofructokinase, liver type OS=Homo sapiens GN=PFKL PE=1 SV=6 - [PFKAL_HUMAN]	32.31	1	22	25	124	1.004	0.868	0.833	0.650	311.56	32.31	45	124	780	85.0	7.50
Q969T3	Sorting nexin-21 OS=Homo sapiens GN=SNX21 PE=2 SV=1 - [SNX21_HUMAN]	6.17	1	2	2	6	0.822	1.501	1.025	0.651	8.93	6.17	3	6	373	41.3	5.24
Q96AC1	Fermitin family homolog 2 OS=Homo sapiens GN=FERMT2 PE=1 SV=1 - [FERM2_HUMAN]	42.65	2	27	28	134	0.813	0.911	1.049	0.651	322.99	42.65	43	134	680	77.8	6.70
P53804	E3 ubiquitin-protein ligase TTC3 OS=Homo sapiens GN=TTC3 PE=1 SV=2 - [TTC3_HUMAN]	3.65	1	5	6	8	1.165	1.029	0.913	0.652	7.53	3.65	6	8	2025	229.7	7.52
Q6ZW49	PAX-interacting protein 1 OS=Homo sapiens GN=PAXIP1 PE=1 SV=2 - [PAXI1_HUMAN]	4.12	1	2	3	3	0.939	0.908	1.303	0.652	2.72	4.12	3	3	1069	121.3	6.70
Q5VWN6	Protein FAM208B OS=Homo sapiens GN=FAM208B PE=1 SV=1 - [F208B_HUMAN]	1.93	2	3	5	8	1.181	0.591	0.728	0.653	10.42	1.93	5	8	2430	268.7	5.90

P21128	Poly(U)-specific endoribonuclease OS=Homo sapiens GN=ENDOU PE=1 SV=2 - [ENDOU_HUMAN]	4.88	1	1	1	1	0.655	0.985	1.155	0.653	0.00	4.88	1	1	410	46.8	5.43
Q7L0X2	Glutamate-rich protein 6 OS=Homo sapiens GN=ERICH6 PE=2 SV=1 - [ERIP6_HUMAN]	1.51	1	1	1	1	1.792	0.648	0.776	0.653	0.00	1.51	1	1	663	75.2	4.93
Q6P4E1	Protein CASC4 OS=Homo sapiens GN=CASC4 PE=1 SV=1 - [CASC4_HUMAN]	16.17	1	7	7	16	0.805	0.634	0.866	0.653	33.42	16.17	10	16	433	48.8	5.82
P01023	Alpha-2-macroglobulin OS=Homo sapiens GN=A2M PE=1 SV=3 - [A2MG_HUMAN]	7.87	2	11	12	57	0.489	1.155	0.712	0.653	98.16	7.87	18	57	1474	163.2	6.46
Q9HD23	Magnesium transporter MRS2 homolog, mitochondrial OS=Homo sapiens GN=MRS2 PE=1 SV=1 - [MRS2_HUMAN]	2.26	1	1	1	1	1.547	2.031	2.252	0.654	2.49	2.26	1	1	443	50.3	6.87
Q99500	Sphingosine 1-phosphate receptor 3 OS=Homo sapiens GN=S1PR3 PE=1 SV=2 - [S1PR3_HUMAN]	3.17	1	1	1	5	0.704	1.239	1.136	0.654	9.16	3.17	2	5	378	42.2	9.70
Q96S97	Myeloid-associated differentiation marker OS=Homo sapiens GN=MYADM PE=1 SV=2 - [MYADM_HUMAN]	3.42	1	1	1	5	0.701	0.411	0.531	0.654	17.83	3.42	2	5	322	35.3	8.15
Q9HD15	Steroid receptor RNA activator 1 OS=Homo sapiens GN=SRA1 PE=1 SV=1 - [SRA1_HUMAN]	27.12	1	4	4	16	0.678	0.871	0.913	0.655	49.95	27.12	6	16	236	25.7	7.03
Q9HB75	p53-induced death domain-containing protein 1 OS=Homo sapiens GN=PIDD1 PE=1 SV=2 - [PIDD1_HUMAN]	5.49	1	2	2	2	1.933	0.515	1.070	0.655	5.00	5.49	2	2	910	99.6	6.43

Q92974	Rho guanine nucleotide exchange factor 2 OS=Homo sapiens GN=ARHGEF2 PE=1 SV=4 - [ARHG2_HUMAN	31.24	1	30	30	79	0.730	0.824	0.813	0.655	202.34	31.24	45	79	986	111.5	7.27
Q9NUQ6	SPATS2-like protein OS=Homo sapiens GN=SPATS2L PE=1 SV=2 - [SPS2L_HUMAN]	32.62	1	15	15	60	0.488	1.238	1.005	0.656	145.62	32.62	26	60	558	61.7	9.64
P16260	Graves disease carrier protein OS=Homo sapiens GN=SLC25A16 PE=1 SV=3 - [GDC_HUMAN]	3.31	1	1	1	1	0.473	0.632	0.623	0.656	3.83	3.31	1	1	332	36.2	9.85
E5RJ46	Uncharacterize d protein C8orf87 OS=Homo sapiens GN=C8orf87 PE=4 SV=1 - [CH087_HUMAN]	19.80	1	1	1	4	1.206	1.304	1.004	0.656	0.00	19.80	1	4	101	11.4	11.37
P42696	RNA-binding protein 34 OS=Homo sapiens GN=RBM34 PE=1 SV=2 - [RBM34_HUMA N]	8.14	1	3	3	11	0.668	0.998	0.651	0.656	31.78	8.14	4	11	430	48.5	10.11
Q06210	Glutamine--fructose-6-phosphate aminotransfera se [isomerizing] 1 OS=Homo sapiens GN=GFPT1 PE=1 SV=3 - [GFPT1_HUMAN]	55.51	1	27	32	197	0.875	0.623	0.899	0.657	543.99	55.51	54	197	699	78.8	7.11
Q9NR12	PDZ and LIM domain protein 7 OS=Homo sapiens GN=PDLIM7 PE=1 SV=1 - [PDLI7_HUMAN]	65.43	1	22	23	160	0.852	0.926	1.202	0.657	293.13	65.43	36	160	457	49.8	8.41
Q8NA70	Protein FAM47B OS=Homo sapiens GN=FAM47B PE=2 SV=2 - [FA47B_HUMAN]	1.09	1	1	1	1	1.011	0.846	0.958	0.657	2.48	1.09	1	1	645	73.9	8.73
O94829	Importin-13 OS=Homo sapiens GN=IPO13 PE=1 SV=3 - [IPO13_HUMAN]	7.37	1	5	5	15	0.855	0.750	0.875	0.657	36.82	7.37	9	15	963	108.1	5.30

Q9BZF9	Uveal autoantigen with coiled-coil domains and ankyrin repeats OS=Homo sapiens GN=UACA PE=1 SV=2 - [UACA_HUMAN]	38.28	1	48	53	139	0.569	1.154	1.218	0.657	298.95	38.28	74	139	1416	162.4	7.03
P43005	Excitatory amino acid transporter 3 OS=Homo sapiens GN=SLC1A1 PE=1 SV=2 - [EAA3_HUMAN]	15.08	1	6	6	15	0.851	0.445	0.919	0.657	33.17	15.08	8	15	524	57.1	5.71
Q6ZRS2	Helicase SRCAP OS=Homo sapiens GN=SRCAP PE=1 SV=3 - [SRCAP_HUMAN]	1.21	3	3	5	8	0.606	0.678	1.114	0.658	16.62	1.21	7	8	3230	343.3	5.96
Q6P2I7	Endogenous Bornavirus-like nucleoprotein 2 OS=Homo sapiens GN=EBLN2 PE=2 SV=1 - [EBLN2_HUMAN]	4.78	1	1	1	1	0.895		1.698	0.658	0.00	4.78	1	1	272	30.4	9.04
P02794	Ferritin heavy chain OS=Homo sapiens GN=FTH1 PE=1 SV=2 - [FRIH_HUMAN]	24.04	1	5	5	18	0.504	0.704	0.962	0.658	46.05	24.04	8	18	183	21.2	5.55
P27540	Aryl hydrocarbon receptor nuclear translocator OS=Homo sapiens GN=ARNT PE=1 SV=1 - [ARNT_HUMAN]	3.55	1	2	2	3	0.914	0.876	0.789	0.658	3.93	3.55	3	3	789	86.6	6.54
O75629	Protein CREG1 OS=Homo sapiens GN=CREG1 PE=1 SV=1 - [CREG1_HUMAN]	5.45	1	1	1	4	0.508	0.815	0.701	0.658	4.82	5.45	2	4	220	24.1	7.59
Q9BRT3	Migration and invasion enhancer 1 OS=Homo sapiens GN=MIEN1 PE=1 SV=1 - [MIEN1_HUMAN]	33.04	1	6	6	30	0.688	0.742	0.771	0.658	73.18	33.04	9	30	115	12.4	4.37

Q9P1Z2	Calcium-binding and coiled-coil domain-containing protein 1 OS=Homo sapiens GN=CALCOCO1 PE=1 SV=2 - [CACO1_HUMAN]	12.74	2	7	9	24	0.950	0.948	1.034	0.658	50.73	12.74	11	24	691	77.3	4.82
P28300	Protein-lysine 6-oxidase OS=Homo sapiens GN=LOX PE=1 SV=2 - [LYOX_HUMAN]	35.49	1	9	9	21	0.274	0.425	1.078	0.658	47.10	35.49	13	21	417	46.9	8.09
P60903	Protein S100-A10 OS=Homo sapiens GN=S100A10 PE=1 SV=2 - [S10AA_HUMAN]	46.39	1	8	8	55	1.568	1.988	1.389	0.658	99.28	46.39	12	55	97	11.2	7.37
P26232	Catenin alpha-2 OS=Homo sapiens GN=CTNNA2 PE=1 SV=5 - [CTNA2_HUMAN]	13.43	1	4	13	66	0.477	1.253	1.406	0.659	187.51	13.43	21	66	953	105.2	5.71
P47974	Zinc finger protein 36, C3H1 type-like 2 OS=Homo sapiens GN=ZFP36L2 PE=1 SV=3 - [TISD_HUMAN]	5.06	1	2	2	18	0.774	0.722	0.669	0.659	40.76	5.06	3	18	494	51.0	8.16
P42262	Glutamate receptor 2 OS=Homo sapiens GN=GRIA2 PE=1 SV=3 - [GRIA2_HUMAN]	2.38	3	1	2	8	1.091	0.452	0.964	0.659	16.26	2.38	2	8	883	98.8	7.58
P35573	Glycogen debranching enzyme OS=Homo sapiens GN=AGL PE=1 SV=3 - [GDE_HUMAN]	19.84	1	24	24	63	0.670	0.959	0.965	0.659	163.24	19.84	37	63	1532	174.7	6.76
P58401	Neurexin-2-beta OS=Homo sapiens GN=NRXN2 PE=2 SV=1 - [NRX2B_HUMAN	5.26	2	2	3	8	1.185	0.479	0.925	0.659	1.80	5.26	3	8	666	70.9	6.38
Q15527	Surfeit locus protein 2 OS=Homo sapiens GN=SURF2 PE=1 SV=3 - [SURF2_HUMAN]	5.08	1	1	1	4	0.594	0.698	1.471	0.659	11.67	5.08	1	4	256	29.6	9.22

P51151	Ras-related protein Rab-9A OS=Homo sapiens GN=RAB9A PE=1 SV=1 - [RAB9A_HUMAN]	17.41	1	3	3	5	0.771	1.597	0.893	0.660	9.94	17.41	5	5	201	22.8	5.47
A8MTJ3	Guanine nucleotide-binding protein G(t) subunit alpha-3 OS=Homo sapiens GN=GNAT3 PE=2 SV=2 - [GNAT3_HUMAN]	7.34	2	1	4	36	1.047	0.641	0.465	0.660	98.73	7.34	7	36	354	40.3	6.01
Q01459	Di-N-acetylchitobiase OS=Homo sapiens GN=CTBS PE=1 SV=1 - [DIAC_HUMAN]	6.23	1	2	2	6	0.782	1.617	1.319	0.660	9.80	6.23	4	6	385	43.7	6.64
Q53EL6	Programmed cell death protein 4 OS=Homo sapiens GN=PDCD4 PE=1 SV=2 - [PDCD4_HUMAN	9.38	1	4	4	12	1.275	1.925	1.145	0.662	23.96	9.38	7	12	469	51.7	5.21
P46527	Cyclin-dependent kinase inhibitor 1B OS=Homo sapiens GN=CDKN1B PE=1 SV=1 - [CDN1B_HUMAN]	10.61	1	2	2	10	0.829	1.453	0.647	0.662	30.74	10.61	3	10	198	22.1	7.02
P06454	Prothymosin alpha OS=Homo sapiens GN=PTMA PE=1 SV=2 - [PTMA_HUMAN]	9.91	1	1	1	6	0.704		0.752	0.662	12.33	9.91	1	6	111	12.2	3.78
Q86XT4	E3 ubiquitin-protein ligase TRIM50 OS=Homo sapiens GN=TRIM50 PE=1 SV=1 - [TRI50_HUMAN]	5.34	3	1	2	5	1.018	1.118	1.017	0.662	11.08	5.34	2	5	487	54.7	7.49
Q9P0S3	ORM1-like protein 1 OS=Homo sapiens GN=ORMDL1 PE=2 SV=1 - [ORML1_HUMAN]	11.76	1	1	1	2	0.898	0.702	0.858	0.662	0.00	11.76	1	2	153	17.4	9.64
Q96LJ7	Dehydrogenase/reductase SDR family member 1 OS=Homo sapiens GN=DHRS1 PE=1 SV=1 - [DHRS1_HUMAN]	12.14	1	3	3	9	0.974	1.379	1.739	0.662	28.93	12.14	4	9	313	33.9	7.83

Q9H1E3	Nuclear ubiquitous casein and cyclin-dependent kinase substrate 1 OS=Homo sapiens GN=NUCKS1 PE=1 SV=1 -	13.58	1	4	4	23	0.868	1.020	0.893	0.663	45.60	13.58	7	23	243	27.3	5.08
Q6UWJ1	Transmembrane and coiled-coil domain-containing protein 3 OS=Homo sapiens GN=TMCO3 PE=2 SV=1 - [TMCO3_HUMAN]	2.81	1	2	2	4	1.730	0.556	1.561	0.663	10.15	2.81	2	4	677	75.5	6.90
O00291	Huntingtin-interacting protein 1 OS=Homo sapiens GN=HIP1 PE=1 SV=5 - [HIP1_HUMAN]	24.01	1	24	26	72	0.629	1.074	0.941	0.663	160.33	24.01	40	72	1037	116.1	5.30
Q6ZRF8	RING finger protein 207 OS=Homo sapiens GN=RNF207 PE=2 SV=2 - [RN207_HUMAN]	3.79	1	2	2	2	1.585	1.146	1.673	0.664	4.52	3.79	2	2	634	70.8	6.68
Q9BZW7	Testis-specific gene 10 protein OS=Homo sapiens GN=TSGA10 PE=1 SV=1 - [TSG10_HUMAN	4.44	1	2	2	2	0.760	0.501	1.049	0.665	2.49	4.44	2	2	698	81.4	5.97
Q9UPM8	AP-4 complex subunit epsilon-1 OS=Homo sapiens GN=AP4E1 PE=1 SV=2 - [AP4E1_HUMAN]	3.87	1	3	3	6	0.917	0.639	0.611	0.665	9.34	3.87	4	6	1137	127.2	5.99
Q4L235	Acyl-CoA synthetase family member 4 OS=Homo sapiens GN=AASDH PE=1 SV=3 - [ACSF4_HUMAN]	1.82	1	1	2	2	0.407	0.516	0.393	0.665	2.31	1.82	2	2	1098	122.5	7.24
P21810	Biglycan OS=Homo sapiens GN=BGN PE=1 SV=2 - [PGS1_HUMAN]	16.85	1	5	6	27	0.302	0.611	1.203	0.665	66.30	16.85	8	27	368	41.6	7.52
P05362	Intercellular adhesion molecule 1 OS=Homo sapiens GN=ICAM1 PE=1 SV=2 - [ICAM1_HUMAN]	20.30	1	9	10	49	1.049	1.521	1.617	0.665	129.63	20.30	18	49	532	57.8	7.99

Q68DX3	FERM and PDZ domain-containing protein 2 OS=Homo sapiens GN=FRMPD2 PE=1 SV=3 - [FRPD2_HUMAN]	4.43	1	3	3	4	0.875	1.494	1.088	0.665	2.09	4.43	3	4	1309	144.2	6.74
Q9P2G4	Microtubule-associated protein 10 OS=Homo sapiens GN=MAP10 PE=1 SV=2 - [MAP10_HUMAN]	0.66	1	1	1	1	0.348	0.762	0.840	0.666	1.65	0.66	1	1	905	100.3	7.17
P57764	Gasdermin-D OS=Homo sapiens GN=GSDMD PE=1 SV=1 - [GSDMD_HUMAN]	15.29	1	7	7	27	1.230	1.107	0.817	0.666	75.01	15.29	11	27	484	52.8	5.08
Q9UI32	Glutaminase liver isoform, mitochondrial OS=Homo sapiens GN=GLS2 PE=1 SV=2 - [GLSL_HUMAN]	3.99	1	1	2	2	0.749	0.995	0.578	0.666	5.57	3.99	2	2	602	66.3	7.30
Q6MZM0	Hephaestin-like protein 1 OS=Homo sapiens GN=HEPHL1 PE=2 SV=2 - [HPHL1_HUMAN]	0.69	1	1	1	1	1.046	0.795	0.932	0.666	2.89	0.69	1	1	1159	131.5	6.74
Q9UPS6	Histone-lysine N-methyltransferase SETD1B OS=Homo sapiens GN=SETD1B PE=1 SV=3 - [SET1B_HUMAN]	0.31	1	1	1	4	1.351	0.336	0.895	0.666	10.26	0.31	1	4	1966	212.7	4.96
O75503	Ceroid-lipofuscinosis neuronal protein 5 OS=Homo sapiens GN=CLN5 PE=1 SV=2 - [CLN5_HUMAN]	17.04	1	6	6	16	0.754	1.293	0.888	0.666	43.39	17.04	9	16	358	41.5	7.40
O00743	Serine/threonine-protein phosphatase 6 catalytic subunit OS=Homo sapiens GN=PPP6C PE=1 SV=1 - [PPP6_HUMAN]	11.80	1	4	4	9	0.962	0.792	0.664	0.666	20.66	11.80	5	9	305	35.1	5.69

Q8NB49	Phospholipid-transporting ATPase IG OS=Homo sapiens GN=ATP11C PE=1 SV=3 - [AT11C_HUMAN]	5.21	1	5	5	13	0.943	0.719	0.793	0.666	31.86	5.21	8	13	1132	129.4	6.67
O94955	Rho-related BTB domain-containing protein 3 OS=Homo sapiens GN=RHOBTB3 PE=1 SV=2 - [RHBT3_HUMAN]	4.75	1	3	3	5	0.620	0.852	0.963	0.666	11.55	4.75	3	5	611	69.4	7.62
P13489	Ribonuclease inhibitor OS=Homo sapiens GN=RNH1 PE=1 SV=2 - [RINI_HUMAN]	38.83	2	16	18	405	0.776	0.733	1.023	0.667	1006.91	38.83	28	405	461	49.9	4.82
P54802	Alpha-N-acetylglucosaminidase OS=Homo sapiens GN=NAGLU PE=1 SV=2 - [ANAG_HUMAN]	23.96	1	13	13	46	0.846	0.964	1.106	0.667	109.99	23.96	23	46	743	82.2	6.65
Q9BTE1	Dynactin subunit 5 OS=Homo sapiens GN=DCTN5 PE=1 SV=1 - [DCTN5_HUMAN]	9.89	1	2	2	2	0.705	0.740	1.046	0.668	2.21	9.89	2	2	182	20.1	8.02
Q13557	Calcium/calmodulin-dependent protein kinase type II subunit delta OS=Homo sapiens GN=CAMK2D PE=1 SV=3 - [KCC2D_HUMAN	37.88	2	12	18	85	0.669	1.111	1.224	0.668	217.17	37.88	26	85	499	56.3	7.25
Q86XE0	Sorting nexin-32 OS=Homo sapiens GN=SNX32 PE=2 SV=1 - [SNX32_HUMAN]	4.47	1	1	2	3	1.101	0.817	1.134	0.668	7.84	4.47	2	3	403	46.4	7.09
Q99732	Lipopolysaccharide-induced tumor necrosis factor-alpha factor OS=Homo sapiens GN=LITAF PE=1 SV=2 - [LITAF_HUMAN]	4.35	1	1	1	3	0.802	0.481	0.691	0.668	6.92	4.35	1	3	161	17.1	6.44
P40145	Adenylate cyclase type 8 OS=Homo sapiens GN=ADCY8 PE=1 SV=1 - [ADCY8_HUMAN]	1.60	2	2	2	2	0.914	0.786	0.914	0.669	4.06	1.60	2	2	1251	140.0	6.99

Q92696	Geranylgeranyl transferase type-2 subunit alpha OS=Homo sapiens GN=RABGGTA PE=1 SV=2 - [PGTA_HUMAN]	22.40	1	12	12	36	0.867	0.713	0.911	0.669	76.64	22.40	19	36	567	65.0	5.67
O15173	Membrane-associated progesterone receptor component 2 OS=Homo sapiens GN=PGRMC2 PE=1 SV=1 - [PGRC2_HUMAN]	31.84	1	9	11	66	1.039	0.698	0.517	0.669	156.39	31.84	19	66	223	23.8	4.88
Q3B7J2	Glucose-fructose oxidoreductase domain-containing protein 2 OS=Homo sapiens GN=GFOD2 PE=2 SV=1 - [GFOD2_HUMAN]	1.56	1	1	1	1	1.000	0.792	0.906	0.669	1.96	1.56	1	1	385	42.2	6.44
Q16798	NADP-dependent malic enzyme, mitochondrial OS=Homo sapiens GN=ME3 PE=2 SV=2 - [MAON_HUMAN]	4.30	1	2	3	16	0.632	1.310	0.599	0.669	28.76	4.30	4	16	604	67.0	7.97
P17483	Homeobox protein Hox-B4 OS=Homo sapiens GN=HOXB4 PE=1 SV=2 - [HXB4_HUMAN]	6.37	1	1	1	1	1.644	0.969	1.091	0.669	0.00	6.37	1	1	251	27.6	9.77
Q7Z4I7	LIM and senescent cell antigen-like-containing domain protein 2 OS=Homo sapiens GN=LIMS2 PE=1 SV=1 - [LIMS2_HUMAN]	13.49	1	2	5	18	0.930	1.041	1.078	0.669	43.26	13.49	8	18	341	38.9	8.05
O94854	Uncharacterized protein KIAA0754 OS=Homo sapiens GN=KIAA0754 PE=1 SV=4 - [K0754_HUMAN]	1.70	1	2	2	3	0.965	0.638	0.980	0.669	4.33	1.70	2	3	1291	135.1	4.21
P52429	Diacylglycerol kinase epsilon OS=Homo sapiens GN=DGKE PE=1 SV=1 - [DGKE_HUMAN]	2.82	1	1	1	3	0.709	0.439	0.840	0.670	7.06	2.82	1	3	567	63.9	7.74

Q8NG78	Olfactory receptor 8G5 OS=Homo sapiens GN=OR8G5 PE=2 SV=2 - [OR8G5_HUMAN]	5.49	1	2	2	2	1.046	1.187	1.031	0.670	4.91	5.49	2	2	346	38.8	8.32
P35221	Catenin alpha-1 OS=Homo sapiens GN=CTNNA1 PE=1 SV=1 - [CTNA1_HUMAN]	48.45	1	29	39	250	1.053	0.992	1.092	0.670	709.79	48.45	66	250	906	100.0	6.29
P98082	Disabled homolog 2 OS=Homo sapiens GN=DAB2 PE=1 SV=3 - [DAB2_HUMAN]	37.66	1	25	25	150	0.757	0.402	0.661	0.670	361.86	37.66	43	150	770	82.4	5.53
O75339	Cartilage intermediate layer protein 1 OS=Homo sapiens GN=CILP PE=1 SV=4 - [CILP1_HUMAN]	1.52	2	2	2	6	0.846	0.547	0.493	0.671	11.27	1.52	2	6	1184	132.5	8.41
Q8TBX8	Phosphatidylinositol 5-phosphate 4-kinase type-2 gamma OS=Homo sapiens GN=PIP4K2C PE=1 SV=3 - [PI42C_HUMAN]	20.67	1	8	9	20	0.921	1.260	1.111	0.672	46.80	20.67	13	20	421	47.3	6.84
Q9Y385	Ubiquitin-conjugating enzyme E2 J1 OS=Homo sapiens GN=UBE2J1 PE=1 SV=2 - [UB2J1_HUMAN]	11.64	1	3	3	10	0.775	0.727	1.085	0.672	29.12	11.64	6	10	318	35.2	6.74
Q9H920	RING finger protein 121 OS=Homo sapiens GN=RNF121 PE=1 SV=1 - [RN121_HUMAN]	5.20	1	1	1	1	0.660	0.490	0.506	0.672	5.86	5.20	1	1	327	37.9	7.06
A8MUI8	Putative UPF0607 protein ENSP00000383783 OS=Homo sapiens PE=3 SV=2 - [YA034_HUMAN]	3.81	1	1	1	1	1.279	1.292	0.929	0.672	0.00	3.81	1	1	341	37.6	9.88
Q14118	Dystroglycan OS=Homo sapiens GN=DAG1 PE=1 SV=2 - [DAG1_HUMAN]	7.04	1	4	4	8	0.941	1.034	1.114	0.672	22.27	7.04	7	8	895	97.4	8.56

Q92546	RAB6A-GEF complex partner protein 2 OS=Homo sapiens GN=RGP1 PE=1 SV=1 - [RGP1_HUMAN]	2.56	1	1	1	3	0.991	0.929	0.971	0.672	4.21	2.56	2	3	391	42.4	5.96
Q15392	Delta(24)-sterol reductase OS=Homo sapiens GN=DHCR24 PE=1 SV=2 - [DHC24_HUMAN	18.22	1	8	8	31	0.658	0.242	0.666	0.672	76.46	18.22	15	31	516	60.1	8.16
Q14195	Dihydropyrimidinase-related protein 3 OS=Homo sapiens GN=DPYSL3 PE=1 SV=1 - [DPYL3_HUMAN]	57.37	1	20	24	232	0.909	1.761	1.509	0.673	665.53	57.37	41	232	570	61.9	6.49
P41743	Protein kinase C iota type OS=Homo sapiens GN=PRKCI PE=1 SV=2 - [KPCI_HUMAN]	5.20	1	3	3	5	0.795	0.865	0.985	0.673	9.84	5.20	5	5	596	68.2	5.85
O43752	Syntaxin-6 OS=Homo sapiens GN=STX6 PE=1 SV=1 - [STX6_HUMAN]	12.94	1	3	3	8	0.561	0.801	0.786	0.673	16.68	12.94	6	8	255	29.2	4.93
P31270	Homeobox protein Hox-A11 OS=Homo sapiens GN=HOXA11 PE=2 SV=2 - [HXA11_HUMAN]	2.88	1	1	1	1	1.084	0.345	0.714	0.673	2.49	2.88	1	1	313	34.5	8.70
Q8IWK6	Probable G-protein coupled receptor 125 OS=Homo sapiens GN=GPR125 PE=1 SV=2 - [GP125_HUMAN	2.57	1	2	2	6	0.705	0.998	2.015	0.674	2.98	2.57	2	6	1321	146.1	8.48
Q9H3U5	Major facilitator superfamily domain-containing protein 1 OS=Homo sapiens GN=MFSD1 PE=2 SV=2 - [MFSD1_HUMA	4.30	1	2	2	14	0.843	0.798	0.833	0.674	19.82	4.30	4	14	465	51.2	6.74
Q14332	Frizzled-2 OS=Homo sapiens GN=FZD2 PE=2 SV=1 - [FZD2_HUMAN]	3.54	1	2	2	2	0.796	0.753	0.711	0.675	1.88	3.54	2	2	565	63.5	8.10

O43739	Cytohesin-3 OS=Homo sapiens GN=CYTH3 PE=1 SV=2 - [CYH3_HUMAN]	6.75	3	2	3	4	0.799	0.810	1.188	0.675	8.84	6.75	4	4	400	46.3	5.47
Q13642	Four and a half LIM domains protein 1 OS=Homo sapiens GN=FHL1 PE=1 SV=4 - [FHL1_HUMAN]	35.91	1	12	12	112	1.219	0.548	1.050	0.675	282.43	35.91	21	112	323	36.2	8.97
Q8IUG5	Unconventional myosin-XVIIIb OS=Homo sapiens GN=MYO18B PE=1 SV=1 - [MY18B_HUMAN]	1.40	1	3	4	5	1.074	0.985	0.955	0.675	10.77	1.40	4	5	2567	285.0	6.90
Q9H4I2	Zinc fingers and homeoboxes protein 3 OS=Homo sapiens GN=ZHX3 PE=1 SV=3 - [ZHX3_HUMAN]	2.41	1	2	3	3	0.825	0.736	1.130	0.675	4.27	2.41	3	3	956	104.6	6.07
Q92520	Protein FAM3C OS=Homo sapiens GN=FAM3C PE=1 SV=1 - [FAM3C_HUMAN]	35.68	1	8	8	42	0.616	0.762	0.945	0.676	96.08	35.68	13	42	227	24.7	8.29
O14730	Serine/threonine-protein kinase RIO3 OS=Homo sapiens GN=RIOK3 PE=1 SV=2 - [RIOK3_HUMAN]	5.59	1	3	3	8	1.153	1.050	1.058	0.676	4.27	5.59	4	8	519	59.1	5.76
P07947	Tyrosine-protein kinase Yes OS=Homo sapiens GN=YES1 PE=1 SV=3 - [YES_HUMAN]	15.65	7	1	9	54	0.850	1.216	0.813	0.676	116.33	15.65	17	54	543	60.8	6.74
Q56VL3	OCIA domain-containing protein 2 OS=Homo sapiens GN=OCIAD2 PE=1 SV=1 - [OCAD2_HUMAN]	20.13	1	3	3	9	0.454	2.162	1.004	0.676	20.81	20.13	4	9	154	16.9	9.03
Q9NVZ3	Adaptin ear-binding coat-associated protein 2 OS=Homo sapiens GN=NECAP2 PE=1 SV=1 - [NECP2_HUMAN]	24.71	1	5	6	15	0.920	0.826	0.888	0.677	36.33	24.71	8	15	263	28.3	8.38

Q5T4F7	Secreted frizzled-related protein 5 OS=Homo sapiens GN=SFRP5 PE=2 SV=3 - [SFRP5_HUMAN	1.89	1	1	1	1	0.524	0.846	0.709	0.678	1.86	1.89	1	1	317	35.5	8.56
O75190	DnaJ homolog subfamily B member 6 OS=Homo sapiens GN=DNAJB6 PE=1 SV=2 - [DNJB6_HUMAN]	22.09	3	4	6	20	1.446	1.919	1.125	0.678	61.39	22.09	8	20	326	36.1	9.16
Q9H0A8	COMM domain-containing protein 4 OS=Homo sapiens GN=COMMD4 PE=1 SV=1 - [COMD4_HUMA N]	9.05	1	2	2	8	0.761	0.570	0.633	0.679	17.69	9.05	3	8	199	21.8	7.31
Q14315	Filamin-C OS=Homo sapiens GN=FLNC PE=1 SV=3 - [FLNC_HUMAN]	67.27	1	145	165	2369	0.573	0.870	1.450	0.680	6058.40	67.27	274	2369	2725	290.8	5.97
Q8WXE9	Stonin-2 OS=Homo sapiens GN=STON2 PE=1 SV=1 - [STON2_HUMA N]	0.99	1	1	1	7	1.356	1.010	1.443	0.680	16.35	0.99	2	7	905	101.1	5.39
Q8IXZ2	Zinc finger CCCH domain-containing protein 3 OS=Homo sapiens GN=ZC3H3 PE=1 SV=3 - [ZC3H3_HUMAN]	4.11	1	2	2	2	0.827	1.017	1.003	0.680	0.00	4.11	2	2	948	101.9	10.95
P20839	Inosine-5'-monophosphat e dehydrogenase 1 OS=Homo sapiens GN=IMPDH1 PE=1 SV=2 - [IMDH1_HUMAN]	15.56	1	5	9	48	0.541	0.563	0.602	0.680	98.96	15.56	16	48	514	55.4	6.90
Q8IWB9	Testis-expressed sequence 2 protein OS=Homo sapiens GN=TEX2 PE=1 SV=2 - [TEX2_HUMAN]	4.79	1	5	5	13	0.647	1.142	1.112	0.681	26.08	4.79	8	13	1127	125.2	6.01
P02790	Hemopexin OS=Homo sapiens GN=HPX PE=1 SV=2 - [HEMO_HUMAN]	1.30	1	1	1	2	0.565	0.829	0.697	0.681	4.26	1.30	1	2	462	51.6	7.02

P23469	Receptor-type tyrosine-protein phosphatase epsilon OS=Homo sapiens GN=PTPRE PE=1 SV=1 - [PTPRE_HUMAN	0.86	1	1	1	5	0.983	0.957	1.153	0.681	10.96	0.86	1	5	700	80.6	7.02
Q8NBS9	Thioredoxin domain-containing protein 5 OS=Homo sapiens GN=TXNDC5 PE=1 SV=2 - [TXND5_HUMAN]	45.60	1	21	23	250	0.653	0.785	0.694	0.681	562.48	45.60	35	250	432	47.6	5.97
Q13094	Lymphocyte cytosolic protein 2 OS=Homo sapiens GN=LCP2 PE=1 SV=1 - [LCP2_HUMAN]	1.88	1	1	1	2	1.082	1.026	1.729	0.681	0.00	1.88	1	2	533	60.2	6.27
Q13322	Growth factor receptor-bound protein 10 OS=Homo sapiens GN=GRB10 PE=1 SV=2 - [GRB10_HUMAN]	1.52	1	1	1	2	0.727	0.856	1.315	0.681	4.00	1.52	2	2	594	67.2	7.87
Q9HCJ6	Synaptic vesicle membrane protein VAT-1 homolog-like OS=Homo sapiens GN=VAT1L PE=1 SV=2 - [VAT1L_HUMAN]	1.67	1	1	1	1	2.018	0.924	2.434	0.681	2.95	1.67	1	1	419	45.9	5.06
Q9NZQ8	Transient receptor potential cation channel subfamily M member 5 OS=Homo sapiens GN=TRPM5 PE=2 SV=1 - [TRPM5_HUMA N]	2.06	1	3	3	3	0.862	0.913	1.174	0.681	6.83	2.06	3	3	1165	131.4	6.77
P78563	Double-stranded RNA-specific editase 1 OS=Homo sapiens GN=ADARB1 PE=1 SV=1 - [RED1_HUMAN]	4.72	1	3	3	8	0.719	2.450	2.645	0.681	16.07	4.72	4	8	741	80.7	9.01
P23634	Plasma membrane calcium-transporting ATPase 4 OS=Homo sapiens GN=ATP2B4 PE=1 SV=2 - [AT2B4_HUMAN]	36.02	1	25	43	191	0.856	1.431	1.172	0.682	458.06	36.02	74	191	1241	137.8	6.60

Q7Z6J2	General receptor for phosphoinositides 1-associated scaffold protein OS=Homo sapiens GN=GRASP PE=1 SV=1 - [GRASP_HUMAN	1.52	1	1	1	1	0.838	0.707	0.874	0.682	1.83	1.52	1	1	395	42.6	8.84
Q96QT4	Transient receptor potential cation channel subfamily M member 7 OS=Homo sapiens GN=TRPM7 PE=1 SV=1 - [TRPM7_HUMAN]	0.86	1	2	2	3	0.587	0.611	0.649	0.682	5.34	0.86	2	3	1865	212.6	7.88
P0DKL9	ARL14 effector protein-like OS=Homo sapiens GN=ARL14EPL PE=4 SV=1 - [A14EL_HUMAN]	4.61	1	1	1	3	1.211	0.510	0.999	0.682	4.79	4.61	1	3	152	17.7	8.34
O75352	Mannose-P-dolichol utilization defect 1 protein OS=Homo sapiens GN=MPDU1 PE=1 SV=2 - [MPU1_HUMAN]	4.05	1	1	1	2	1.063	0.308	0.291	0.682	2.55	4.05	2	2	247	26.6	8.94
A8MT70	Zinc finger B-box domain-containing protein 1 OS=Homo sapiens GN=ZBBX PE=2 SV=3 - [ZBBX_HUMAN]	3.38	1	2	2	2	0.515	0.711	0.810	0.682	0.00	3.38	2	2	800	91.4	5.59
P78362	SRSF protein kinase 2 OS=Homo sapiens GN=SRPK2 PE=1 SV=3 - [SRPK2_HUMAN]	7.70	1	6	7	18	0.688	0.908	1.085	0.683	40.23	7.70	11	18	688	77.5	4.97
O14782	Kinesin-like protein KIF3C OS=Homo sapiens GN=KIF3C PE=1 SV=3 - [KIF3C_HUMAN]	7.44	2	2	4	6	0.950	1.286	0.971	0.683	14.00	7.44	5	6	793	89.4	8.22
O75884	Putative hydrolase RBBP9 OS=Homo sapiens GN=RBBP9 PE=1 SV=2 - [RBBP9_HUMAN]	25.27	1	3	3	8	1.615	0.783	0.763	0.683	20.27	25.27	5	8	186	21.0	6.20

P58546	Myotrophin OS=Homo sapiens GN=MTPN PE=1 SV=2 - [MTPN_HUMAN]	46.61	1	7	7	54	0.963	0.932	0.992	0.684	128.69	46.61	10	54	118	12.9	5.52
Q9ULV0	Unconventional myosin-Vb OS=Homo sapiens GN=MYO5B PE=1 SV=3 - [MYO5B_HUMAN]	2.11	2	1	4	5	0.948	2.711	0.948	0.684	9.58	2.11	5	5	1848	213.5	7.20
P18084	Integrin beta-5 OS=Homo sapiens GN=ITGB5 PE=1 SV=1 - [ITB5_HUMAN]	18.90	1	13	13	36	1.468	1.010	0.778	0.684	71.37	18.90	19	36	799	88.0	6.06
Q86YS7	C2 domain-containing protein 5 OS=Homo sapiens GN=C2CD5 PE=1 SV=1 - [C2CD5_HUMAN]	1.90	1	2	2	2		2.369		0.684	5.51	1.90	2	2	1000	110.4	5.69
P04259	Keratin, type II cytoskeletal 6B OS=Homo sapiens GN=KRT6B PE=1 SV=5 - [K2C6B_HUMAN]	14.72	4	1	10	104	1.289	0.596	0.407	0.685	213.62	14.72	14	104	564	60.0	8.00
Q9H7M9	Platelet receptor Gi24 OS=Homo sapiens GN=C10orf54 PE=1 SV=3 - [GI24_HUMAN]	9.00	1	3	3	5	0.818	0.491	0.679	0.685	6.92	9.00	5	5	311	33.9	6.95
Q9C099	Leucine-rich repeat and coiled-coil domain-containing protein 1 OS=Homo sapiens GN=LRRCC1 PE=1 SV=2 - [LRCC1_HUMAN	7.46	1	5	6	6	1.524	0.861	1.126	0.685	3.79	7.46	6	6	1032	119.5	5.88
Q9Y281	Cofilin-2 OS=Homo sapiens GN=CFL2 PE=1 SV=1 - [COF2_HUMAN]	78.31	1	9	14	246	0.909	0.966	0.913	0.685	548.92	78.31	23	246	166	18.7	7.88
Q7Z449	Cytochrome P450 2U1 OS=Homo sapiens GN=CYP2U1 PE=1 SV=1 - [CP2U1_HUMAN]	4.96	1	2	3	5	0.651	0.845	1.229	0.685	17.21	4.96	3	5	544	61.9	8.40

P13196	5-aminolevulinate synthase, nonspecific, mitochondrial OS=Homo sapiens GN=ALAS1 PE=1 SV=2 - [HEM1_HUMAN]	2.03	1	1	1	1	1.472	0.596	1.493	0.686	3.38	2.03	1	1	640	70.5	8.46
P62070	Ras-related protein R-Ras2 OS=Homo sapiens GN=RRAS2 PE=1 SV=1 - [RRAS2_HUMAN]	58.82	1	9	11	63	0.851	0.701	0.743	0.686	165.09	58.82	17	63	204	23.4	6.01
Q02153	Guanylate cyclase soluble subunit beta-1 OS=Homo sapiens GN=GUCY1B3 PE=1 SV=1 - [GCYB1_HUMAN]	9.21	1	5	5	8	0.685	0.903	1.004	0.686	16.39	9.21	7	8	619	70.5	5.35
Q9UBI1	COMM domain-containing protein 3 OS=Homo sapiens GN=COMMD3 PE=1 SV=1 - [COMD3_HUMAN]	9.74	1	2	2	2	1.015	1.150	1.219	0.686	7.38	9.74	2	2	195	22.1	5.99
Q9NVA2	Septin-11 OS=Homo sapiens GN=SEPT11 PE=1 SV=3 - [SEP11_HUMAN]	49.65	1	9	22	103	0.749	0.894	0.807	0.686	245.15	49.65	34	103	429	49.4	6.81
A8MXY4	Zinc finger protein 99 OS=Homo sapiens GN=ZNF99 PE=2 SV=3 - [ZNF99_HUMAN]	5.44	1	2	3	5	0.901	0.385	0.660	0.686	8.27	5.44	4	5	864	100.7	9.45
Q7Z2Z1	Treslin OS=Homo sapiens GN=TICRR PE=1 SV=2 - [TICRR_HUMAN]	1.88	1	3	3	3	0.729	0.919	0.959	0.686	1.62	1.88	3	3	1910	210.7	8.78
Q8TAL6	Fin bud initiation factor homolog OS=Homo sapiens GN=FIBIN PE=1 SV=1 - [FIBIN_HUMAN]	11.85	1	2	2	2	1.541	0.738	1.068	0.686	3.08	11.85	2	2	211	24.3	5.49
P42574	Caspase-3 OS=Homo sapiens GN=CASP3 PE=1 SV=2 - [CASP3_HUMAN]	34.30	1	8	8	28	0.718	0.804	0.728	0.686	78.98	34.30	13	28	277	31.6	6.54

Q6AW86	Zinc finger protein 324B OS=Homo sapiens GN=ZNF324B PE=2 SV=1 - [Z324B_HUMAN]	1.29	2	1	1	2	0.810	1.043	0.655	0.686	4.08	1.29	1	2	544	60.6	9.69
O95394	Phosphoacetylglucosamine mutase OS=Homo sapiens GN=PGM3 PE=1 SV=1 - [AGM1_HUMAN]	48.71	1	20	20	89	0.632	0.731	0.842	0.687	229.41	48.71	31	89	542	59.8	6.25
Q15121	Astrocytic phosphoprotein PEA-15 OS=Homo sapiens GN=PEA15 PE=1 SV=2 - [PEA15_HUMAN]	70.00	1	12	12	71	0.585	0.718	0.911	0.687	163.70	70.00	18	71	130	15.0	5.02
Q9H8W4	Pleckstrin homology domain-containing family F member 2 OS=Homo sapiens GN=PLEKHF2 PE=1 SV=1 - [PKHF2_HUMAN]	4.02	2	1	1	3	1.752	1.846	1.343	0.687	2.02	4.02	2	3	249	27.8	8.21
Q13287	N-myc-interactor OS=Homo sapiens GN=NMI PE=1 SV=2 - [NMI_HUMAN]	18.57	1	5	5	16	0.820	1.112	0.757	0.688	31.06	18.57	9	16	307	35.0	5.34
O75473	Leucine-rich repeat-containing G-protein coupled receptor 5 OS=Homo sapiens GN=LGR5 PE=1 SV=1 - [LGR5_HUMAN]	3.64	3	2	4	10	0.940	1.424	2.062	0.688	17.57	3.64	4	10	907	99.9	6.60
P41252	Isoleucine--tRNA ligase, cytoplasmic OS=Homo sapiens GN=IARS PE=1 SV=2 - [SYIC_HUMAN]	31.70	1	43	43	163	0.680	0.774	0.815	0.688	388.33	31.70	68	163	1262	144.4	6.15
Q3SXZ3	Zinc finger protein 718 OS=Homo sapiens GN=ZNF718 PE=2 SV=1 - [ZN718_HUMAN]	6.28	94	1	2	4	1.154	2.098	2.451	0.688	0.00	6.28	2	4	478	55.4	9.45

Q9UKI2	Cdc42 effector protein 3 OS=Homo sapiens GN=CDC42EP3 PE=1 SV=1 - [BORG2_HUMAN]	18.90	1	4	4	11	0.756	0.930	1.304	0.688	44.28	18.90	5	11	254	27.7	5.78
P36897	TGF-beta receptor type-1 OS=Homo sapiens GN=TGFBR1 PE=1 SV=1 - [TGFR1_HUMAN]	4.97	1	3	3	4	1.628	0.828	0.499	0.688	5.94	4.97	4	4	503	55.9	7.55
Q8IZU1	Protein FAM9A OS=Homo sapiens GN=FAM9A PE=1 SV=1 - [FAM9A_HUMAN]	3.92	1	1	1	1	0.670	0.693	0.835	0.688	2.32	3.92	1	1	332	37.3	4.83
Q8TEU7	Rap guanine nucleotide exchange factor 6 OS=Homo sapiens GN=RAPGEF6 PE=1 SV=2 - [RPGF6_HUMAN	1.37	1	2	3	10	0.777	0.772	0.684	0.689	17.08	1.37	4	10	1601	179.3	6.40
Q96JC1	Vam6/Vps39-like protein OS=Homo sapiens GN=VPS39 PE=1 SV=2 - [VPS39_HUMAN]	4.18	1	4	4	5	1.170	1.090	0.876	0.689	9.60	4.18	5	5	886	101.7	6.99
Q8WW59	SPRY domain-containing protein 4 OS=Homo sapiens GN=SPRYD4 PE=1 SV=2 - [SPRY4_HUMAN]	16.91	1	2	2	5	0.675	0.659	0.777	0.689	15.49	16.91	2	5	207	23.1	6.93
Q5VT25	Serine/threonine-protein kinase MRCK alpha OS=Homo sapiens GN=CDC42BPA PE=1 SV=1 - [MRCKA_HUMA	15.01	1	19	25	51	0.990	0.787	1.015	0.689	133.93	15.01	35	51	1732	197.2	6.58
O95980	Reversion-inducing cysteine-rich protein with Kazal motifs OS=Homo sapiens GN=RECK PE=1 SV=1 - [RECK_HUMAN]	12.46	1	13	13	32	0.685	0.437	0.707	0.689	73.50	12.46	18	32	971	106.4	6.74
Q53EP0	Fibronectin type III domain-containing protein 3B OS=Homo sapiens GN=FNDC3B PE=1 SV=2 - [FND3B_HUMAN	22.01	1	21	21	62	0.811	0.539	0.758	0.690	158.36	22.01	32	62	1204	132.8	5.95

Q9UJ72	Annexin A10 OS=Homo sapiens GN=ANXA10 PE=1 SV=3 - [ANX10_HUMAN]	5.56	1	2	2	3	0.796	1.680	1.300	0.690	8.07	5.56	2	3	324	37.3	5.33
P09104	Gamma-enolase OS=Homo sapiens GN=ENO2 PE=1 SV=3 - [ENOG_HUMAN]	51.15	1	12	17	196	1.796	2.467	1.413	0.690	507.54	51.15	25	196	434	47.2	5.03
P06756	Integrin alpha-V OS=Homo sapiens GN=ITGAV PE=1 SV=2 - [ITAV_HUMAN]	33.40	1	32	32	208	1.210	0.921	0.683	0.690	507.27	33.40	58	208	1048	116.0	5.68
Q96J92	Serine/threonine-protein kinase WNK4 OS=Homo sapiens GN=WNK4 PE=1 SV=1 - [WNK4_HUMAN]	4.59	1	2	6	24	0.192	1.264	1.572	0.690	45.97	4.59	10	24	1243	134.7	5.48
P62273	40S ribosomal protein S29 OS=Homo sapiens GN=RPS29 PE=1 SV=2 - [RS29_HUMAN]	23.21	1	2	2	2	0.947	0.621	0.895	0.690	2.03	23.21	2	2	56	6.7	10.13
Q16825	Tyrosine-protein phosphatase non-receptor type 21 OS=Homo sapiens GN=PTPN21 PE=1 SV=2 - [PTN21_HUMAN]	0.68	1	1	1	2	0.613	2.696	1.283	0.691	1.75	0.68	2	2	1174	133.2	7.90
Q13572	Inositol-tetrakisphosphate 1-kinase OS=Homo sapiens GN=ITPK1 PE=1 SV=2 - [ITPK1_HUMAN]	3.14	1	1	1	2	0.879	1.001	1.492	0.691	4.75	3.14	2	2	414	45.6	6.16
O43310	CBP80/20-dependent translation initiation factor OS=Homo sapiens GN=CTIF PE=1 SV=1 - [CTIF_HUMAN]	15.22	1	7	7	13	0.670	1.316	0.944	0.691	33.54	15.22	8	13	598	67.5	6.54
Q9NW08	DNA-directed RNA polymerase III subunit RPC2 OS=Homo sapiens GN=POLR3B PE=1 SV=2 - [RPC2_HUMAN]	3.09	1	3	3	4	0.469	0.725	1.018	0.691	11.96	3.09	4	4	1133	127.7	8.50

O43776	Asparagine--tRNA ligase, cytoplasmic OS=Homo sapiens GN=NARS PE=1 SV=1 - [SYNC_HUMAN]	41.61	1	25	25	170	0.677	0.762	0.842	0.691	464.23	41.61	43	170	548	62.9	6.25
O95365	Zinc finger and BTB domain-containing protein 7A OS=Homo sapiens GN=ZBTB7A PE=1 SV=1 - [ZBT7A_HUMAN]	8.90	1	2	2	2	0.848	0.697	1.376	0.691	3.00	8.90	2	2	584	61.4	5.19
P61224	Ras-related protein Rap-1b OS=Homo sapiens GN=RAP1B PE=1 SV=1 - [RAP1B_HUMAN]	76.09	2	4	12	184	1.104	1.170	1.107	0.691	487.96	76.09	19	184	184	20.8	5.78
P21554	Cannabinoid receptor 1 OS=Homo sapiens GN=CNR1 PE=1 SV=1 - [CNR1_HUMAN]	2.54	1	1	1	1	0.473	0.788	0.799	0.691	0.00	2.54	1	1	472	52.8	8.16
P62861	40S ribosomal protein S30 OS=Homo sapiens GN=FAU PE=1 SV=1 - [RS30_HUMAN]	18.64	1	2	2	10	0.848	1.034	0.723	0.691	21.98	18.64	4	10	59	6.6	12.15
Q16445	Gamma-aminobutyric acid receptor subunit alpha-6 OS=Homo sapiens GN=GABRA6 PE=1 SV=2 - [GBRA6_HUMAN]	3.75	1	1	1	1	1.051	1.137	0.924	0.692	0.00	3.75	1	1	453	51.0	8.22
Q9Y4E6	WD repeat-containing protein 7 OS=Homo sapiens GN=WDR7 PE=1 SV=2 - [WDR7_HUMAN]	2.55	1	2	2	2	1.669	1.988	1.223	0.692	2.27	2.55	2	2	1490	163.7	6.92
Q15048	Leucine-rich repeat-containing protein 14 OS=Homo sapiens GN=LRRC14 PE=2 SV=1 - [LRC14_HUMAN]	1.83	1	1	1	2	1.034	0.510	0.440	0.692	4.85	1.83	1	2	493	54.5	6.90
O14908	PDZ domain-containing protein GIPC1 OS=Homo sapiens GN=GIPC1 PE=1 SV=2 - [GIPC1_HUMAN]	45.05	2	12	14	50	0.699	0.876	0.924	0.692	131.68	45.05	21	50	333	36.0	6.28

P20848	Putative alpha-1-antitrypsin-related protein OS=Homo sapiens GN=SERPINA2 PE=1 SV=1 - [A1ATR_HUMAN]	5.24	1	2	2	2	0.758	0.551	1.358	0.692	1.99	5.24	2	2	420	47.9	7.90
Q8N687	Beta-defensin 125 OS=Homo sapiens GN=DEFB125 PE=2 SV=2 - [DB125_HUMAN]	6.41	1	1	1	1	1.737	1.226	1.201	0.693	2.02	6.41	1	1	156	17.5	5.49
P58215	Lysyl oxidase homolog 3 OS=Homo sapiens GN=LOXL3 PE=2 SV=1 - [LOXL3_HUMAN]	1.73	1	1	1	4	0.546	0.598	0.534	0.693	13.74	1.73	2	4	753	83.1	6.86
Q9NQE9	Histidine triad nucleotide-binding protein 3 OS=Homo sapiens GN=HINT3 PE=1 SV=1 - [HINT3_HUMAN]	13.74	1	2	2	6	1.079	1.067	1.026	0.693	7.33	13.74	3	6	182	20.3	6.60
P51580	Thiopurine S-methyltransferase OS=Homo sapiens GN=TPMT PE=1 SV=1 - [TPMT_HUMAN]	26.12	1	9	9	28	0.810	1.330	1.227	0.693	49.33	26.12	14	28	245	28.2	6.23
Q86WA8	Lon protease homolog 2, peroxisomal OS=Homo sapiens GN=LONP2 PE=1 SV=1 - [LONP2_HUMAN]	4.34	1	3	3	4	0.266	0.901	1.088	0.693	5.36	4.34	4	4	852	94.6	7.30
Q9Y2E5	Epididymis-specific alpha-mannosidase OS=Homo sapiens GN=MAN2B2 PE=1 SV=4 - [MA2B2_HUMAN]	8.72	1	6	6	17	0.894	1.056	1.494	0.693	50.66	8.72	8	17	1009	113.9	7.24
Q96C00	Zinc finger and BTB domain-containing protein 9 OS=Homo sapiens GN=ZBTB9 PE=1 SV=1 - [ZBTB9_HUMAN]	2.96	1	1	1	1	0.834	1.728	1.741	0.693	3.28	2.96	1	1	473	50.6	6.76
Q2M1P5	Kinesin-like protein KIF7 OS=Homo sapiens GN=KIF7 PE=1 SV=2 - [KIF7_HUMAN]	3.50	1	3	6	13	1.105	0.722	0.830	0.694	27.82	3.50	6	13	1343	150.5	6.79

P61086	Ubiquitin-conjugating enzyme E2 K OS=Homo sapiens GN=UBE2K PE=1 SV=3 - [UBE2K_HUMAN]	52.50	1	9	10	55	0.805	0.830	0.792	0.694	141.11	52.50	15	55	200	22.4	5.44
P10398	Serine/threonine-protein kinase A-Raf OS=Homo sapiens GN=ARAF PE=1 SV=2 - [ARAF_HUMAN]	10.40	1	4	5	12	0.875	0.623	0.846	0.694	28.22	10.40	7	12	606	67.5	9.01
Q8NEZ4	Histone-lysine N-methyltransferase 2C OS=Homo sapiens GN=KMT2C PE=1 SV=3 - [KMT2C_HUMAN]	0.33	1	2	2	6	0.644	0.906	0.921	0.694	12.80	0.33	2	6	4911	541.0	6.49
Q8N9B5	Junction-mediating and -regulatory protein OS=Homo sapiens GN=JMY PE=1 SV=2 - [JMY_HUMAN]	1.42	1	1	2	3	0.988	1.026	1.027	0.694	2.41	1.42	3	3	988	111.4	6.18
H7BZ55	Putative ciliary rootlet coiled-coil protein-like 3 protein OS=Homo sapiens PE=5 SV=2 - [CROL3_HUMAN]	6.13	3	6	11	17	1.235	0.708	0.737	0.695	21.91	6.13	12	17	2252	248.2	5.83
Q9Y5J5	Pleckstrin homology-like domain family A member 3 OS=Homo sapiens GN=PHLDA3 PE=1 SV=1 - [PHLA3_HUMAN]	6.30	1	1	1	1	1.350	1.161	1.088	0.695	2.38	6.30	1	1	127	13.9	9.67
Q8NE86	Calcium uniporter protein, mitochondrial OS=Homo sapiens GN=MCU PE=1 SV=1 - [MCU_HUMAN]	24.79	1	8	8	20	0.710	0.727	0.957	0.695	41.60	24.79	14	20	351	39.8	8.65
Q9Y296	Trafficking protein particle complex subunit 4 OS=Homo sapiens GN=TRAPPC4 PE=1 SV=1 - [TPPC4_HUMAN]	10.05	1	2	2	2	0.808	1.301	2.058	0.695	6.67	10.05	2	2	219	24.3	6.21

Q9Y5Z7	Host cell factor 2 OS=Homo sapiens GN=HCFC2 PE=1 SV=1 - [HCFC2_HUMAN]	2.53	1	1	1	1	0.797	0.236	0.252	0.696	0.00	2.53	1	1	792	86.7	8.48
Q12884	Prolyl endopeptidase FAP OS=Homo sapiens GN=FAP PE=1 SV=5 - [SEPR_HUMAN]	22.76	1	18	19	107	0.507	2.050	1.395	0.696	216.80	22.76	33	107	760	87.7	6.65
Q8NFZ5	TNFAIP3-interacting protein 2 OS=Homo sapiens GN=TNIP2 PE=1 SV=1 - [TNIP2_HUMAN]	6.76	1	1	2	5	0.451	1.042	0.978	0.696	17.57	6.76	3	5	429	48.7	6.44
Q8WUA8	Tsukushin OS=Homo sapiens GN=TSKU PE=2 SV=3 - [TSK_HUMAN]	4.25	1	1	1	1	0.800	0.869	0.606	0.696	0.00	4.25	1	1	353	37.8	6.87
Q8NAC3	Interleukin-17 receptor C OS=Homo sapiens GN=IL17RC PE=1 SV=2 - [I17RC_HUMAN]	1.52	1	1	1	1	1.325	0.189	0.345	0.697	2.27	1.52	1	1	791	86.2	6.10
Q8N9N2	Activating signal cointegrator 1 complex subunit 1 OS=Homo sapiens GN=ASCC1 PE=1 SV=1 - [ASCC1_HUMAN	13.50	1	5	5	12	0.923	0.701	0.617	0.697	27.74	13.50	7	12	400	45.5	5.54
Q9BRQ0	Pygopus homolog 2 OS=Homo sapiens GN=PYGO2 PE=1 SV=2 - [PYGO2_HUMA N]	1.97	1	1	1	1	1.080	1.194	0.878	0.697	1.84	1.97	1	1	406	41.2	7.28
Q15012	Lysosomal-associated transmembran e protein 4A OS=Homo sapiens GN=LAPTM4A PE=1 SV=1 - [LAP4A_HUMAN]	5.58	1	1	1	3	1.167	1.030	1.132	0.697	4.87	5.58	2	3	233	26.8	6.49
Q9HBL0	Tensin-1 OS=Homo sapiens GN=TNS1 PE=1 SV=2 - [TENS1_HUMAN]	36.02	1	44	47	217	0.467	1.480	1.419	0.697	560.37	36.02	78	217	1735	185.6	7.75

Q5T0N5	Formin-binding protein 1-like OS=Homo sapiens GN=FNBP1L PE=1 SV=3 - [FBP1L_HUMAN]	10.25	1	5	6	14	0.661	1.178	1.146	0.697	32.01	10.25	10	14	605	70.0	6.64
P10301	Ras-related protein R-Ras OS=Homo sapiens GN=RRAS PE=1 SV=1 - [RRAS_HUMAN]	56.88	1	8	10	64	1.051	1.284	1.215	0.697	169.93	56.88	16	64	218	23.5	6.93
Q9HDC5	Junctophilin-1 OS=Homo sapiens GN=JPH1 PE=1 SV=2 - [JPH1_HUMAN]	1.51	1	1	1	1	0.678	0.718	0.890	0.698	2.46	1.51	1	1	661	71.6	9.32
Q9BRS8	La-related protein 6 OS=Homo sapiens GN=LARP6 PE=1 SV=1 - [LARP6_HUMAN]	11.61	1	5	5	14	0.635	1.059	1.250	0.698	17.68	11.61	9	14	491	54.7	8.25
Q6ZP82	Coiled-coil domain-containing protein 141 OS=Homo sapiens GN=CCDC141 PE=1 SV=2 - [CC141_HUMAN]	4.21	1	3	4	4	1.347	0.664	1.278	0.698	5.60	4.21	4	4	1450	166.2	5.58
Q9H3Z4	DnaJ homolog subfamily C member 5 OS=Homo sapiens GN=DNAJC5 PE=1 SV=1 - [DNJC5_HUMAN]	15.15	1	3	3	14	0.889	0.565	0.584	0.698	30.24	15.15	5	14	198	22.1	5.07
Q6EEV4	DNA-directed RNA polymerase II subunit GRINL1A, isoforms 4/5 OS=Homo sapiens GN=POLR2M PE=1 SV=1 - [GL1AD_HUMAN]	25.00	1	2	2	5	0.568	1.295	1.350	0.698	19.70	25.00	3	5	148	15.1	8.37
O14494	Lipid phosphate phosphohydrolase 1 OS=Homo sapiens GN=PPAP2A PE=1 SV=1 - [LPP1_HUMAN]	11.97	1	3	3	8	2.495	0.502	0.698	0.698	22.48	11.97	5	8	284	32.1	7.97
Q53TN4	Cytochrome b reductase 1 OS=Homo sapiens GN=CYBRD1 PE=1 SV=1 - [CYBR1_HUMAN]	18.53	1	4	4	30	0.769	0.544	0.953	0.698	65.23	18.53	8	30	286	31.6	8.76

Q9P299	Coatomer subunit zeta-2 OS=Homo sapiens GN=COPZ2 PE=2 SV=1 - [COPZ2_HUMAN]	27.14	1	6	6	27	0.784	0.813	0.979	0.699	62.20	27.14	10	27	210	23.5	5.17
Q9NSY0	Nuclear receptor-binding protein 2 OS=Homo sapiens GN=NRBP2 PE=1 SV=2 - [NRBP2_HUMAN	4.19	1	3	3	4	0.970	0.931	1.014	0.699	14.39	4.19	3	4	501	57.8	6.49
P52630	Signal transducer and activator of transcription 2 OS=Homo sapiens GN=STAT2 PE=1 SV=1 - [STAT2_HUMAN]	14.34	1	14	14	35	1.271	0.880	0.865	0.699	73.38	14.34	18	35	851	97.9	5.49
Q9UKY4	Protein O-mannosyl-transferase 2 OS=Homo sapiens GN=POMT2 PE=1 SV=2 - [POMT2_HUMAN]	0.93	1	1	1	3	0.721	0.602	0.369	0.700	8.49	0.93	2	3	750	84.2	9.16
P80217	Interferon-induced 35 kDa protein OS=Homo sapiens GN=IFI35 PE=1 SV=5 - [IN35_HUMAN]	11.89	1	4	4	12	0.876	1.084	0.919	0.700	29.01	11.89	7	12	286	31.5	6.09
Q15369	Transcription elongation factor B polypeptide 1 OS=Homo sapiens GN=TCEB1 PE=1 SV=1 - [ELOC_HUMAN]	58.04	1	5	5	39	1.048	0.821	1.043	0.700	100.42	58.04	9	39	112	12.5	4.78
Q9H2F9	Coiled-coil domain-containing protein 68 OS=Homo sapiens GN=CCDC68 PE=2 SV=1 - [CCD68_HUMAN]	3.28	1	1	1	3	1.472	0.927	1.216	0.700	1.69	3.28	1	3	335	38.8	8.65
P61964	WD repeat-containing protein 5 OS=Homo sapiens GN=WDR5 PE=1 SV=1 - [WDR5_HUMAN]	8.68	1	2	2	2	0.695	1.243	0.943	0.700	4.95	8.68	2	2	334	36.6	8.27

P09884	DNA polymerase alpha catalytic subunit OS=Homo sapiens GN=POLA1 PE=1 SV=2 - [DPOLA_HUMAN]	0.55	1	1	1	1	0.505	0.201	0.194	0.700	1.80	0.55	1	1	1462	165.8	5.85
O43399	Tumor protein D54 OS=Homo sapiens GN=TPD52L2 PE=1 SV=2 - [TPD54_HUMAN]	77.67	1	19	19	204	0.826	1.158	1.096	0.701	522.75	77.67	34	204	206	22.2	5.36
P08962	CD63 antigen OS=Homo sapiens GN=CD63 PE=1 SV=2 - [CD63_HUMAN]	19.33	1	7	7	72	0.570	0.601	0.581	0.701	143.61	19.33	9	72	238	25.6	7.81
Q9Y4R8	Telomere length regulation protein TEL2 homolog OS=Homo sapiens GN=TELO2 PE=1 SV=2 - [TELO2_HUMAN	1.31	1	1	1	2	1.056	1.022	1.090	0.701	4.04	1.31	2	2	837	91.7	5.76
P29992	Guanine nucleotide-binding protein subunit alpha-11 OS=Homo sapiens GN=GNA11 PE=1 SV=2 - [GNA11_HUMAN]	48.47	1	8	16	57	0.950	0.732	0.962	0.701	141.89	48.47	26	57	359	42.1	5.69
O14495	Lipid phosphate phosphohydrolase 3 OS=Homo sapiens GN=PPAP2B PE=1 SV=1 - [LPP3_HUMAN]	18.65	1	7	7	25	1.082	0.498	0.283	0.701	48.04	18.65	12	25	311	35.1	9.14
Q8NDM7	Cilia- and flagella-associated protein 43 OS=Homo sapiens GN=CFAP43 PE=2 SV=3 - [CFA43_HUMAN	1.98	1	1	3	12	0.438	1.069	0.984	0.701	12.77	1.98	3	12	1665	191.9	5.99
O60502	Protein O-GlcNAcase OS=Homo sapiens GN=MGEA5 PE=1 SV=2 - [OGA_HUMAN]	9.17	1	8	9	22	0.699	1.061	1.046	0.702	45.70	9.17	14	22	916	102.8	4.91
Q15417	Calponin-3 OS=Homo sapiens GN=CNN3 PE=1 SV=1 - [CNN3_HUMAN]	63.53	1	17	22	331	1.065	0.682	1.457	0.702	837.86	63.53	31	331	329	36.4	6.05

Q8TCJ2	Dolichyl-diphosphooligosaccharide--protein glycosyltransferase subunit STT3B OS=Homo sapiens GN=STT3B PE=1 SV=1 - [STT3B_HUMAN]	5.21	1	5	5	10	0.766	0.667	0.732	0.702	25.22	5.21	8	10	826	93.6	8.91
Q86XR7	TIR domain-containing adapter molecule 2 OS=Homo sapiens GN=TICAM2 PE=1 SV=1 - [TCAM2_HUMA	6.38	1	1	1	4	1.279	0.850	0.838	0.702	6.94	6.38	2	4	235	26.9	5.07
P08294	Extracellular superoxide dismutase [Cu-Zn] OS=Homo sapiens GN=SOD3 PE=1 SV=2 - [SODE_HUMAN]	10.00	1	2	2	8	0.588	3.835	2.176	0.703	25.00	10.00	3	8	240	25.8	6.61
Q9NRX6	Protein kish-B OS=Homo sapiens GN=TMEM167B PE=2 SV=1 - [KISHB_HUMAN]	9.46	1	1	1	1	0.705	1.017	0.850	0.703	1.97	9.46	1	1	74	8.3	9.61
Q96JH8	Ras-associating and dilute domain-containing protein OS=Homo sapiens GN=RADIL PE=1 SV=5 - [RADIL_HUMAN]	2.60	2	1	2	3	2.628			0.704	4.23	2.60	3	3	1075	117.4	7.09
P00519	Tyrosine-protein kinase ABL1 OS=Homo sapiens GN=ABL1 PE=1 SV=4 - [ABL1_HUMAN]	4.69	1	4	4	5	0.645	0.990	1.026	0.704	12.17	4.69	4	5	1130	122.8	8.66
B5MCY1	Tudor domain-containing protein 15 OS=Homo sapiens GN=TDRD15 PE=2 SV=1 - [TDR15_HUMAN]	0.52	1	1	1	1	1.356	1.144	1.150	0.704	2.78	0.52	1	1	1934	221.6	7.03
Q8TAL5	Uncharacterized protein C9orf43 OS=Homo sapiens GN=C9orf43 PE=2 SV=1 - [CI043_HUMAN]	1.74	1	1	1	1	1.137	0.399	0.668	0.704	1.98	1.74	1	1	461	52.2	8.87

P05067	Amyloid beta A4 protein OS=Homo sapiens GN=APP PE=1 SV=3 - [A4_HUMAN]	18.18	1	11	12	31	0.941	0.771	1.039	0.704	77.21	18.18	19	31	770	86.9	4.82
Q13576	Ras GTPase-activating-like protein IQGAP2 OS=Homo sapiens GN=IQGAP2 PE=1 SV=4 - [IQGA2_HUMAN]	2.60	2	2	4	16	0.744	1.085	0.995	0.704	25.01	2.60	6	16	1575	180.5	5.64
Q96JY6	PDZ and LIM domain protein 2 OS=Homo sapiens GN=PDLIM2 PE=1 SV=1 - [PDLI2_HUMAN]	43.47	1	12	12	54	0.687	0.532	0.738	0.705	146.26	43.47	21	54	352	37.4	8.72
P30837	Aldehyde dehydrogenase X, mitochondrial OS=Homo sapiens GN=ALDH1B1 PE=1 SV=3 - [AL1B1_HUMAN]	36.56	1	17	19	90	0.552	2.120	3.674	0.705	236.30	36.56	32	90	517	57.2	6.80
Q9C0H2	Protein tweety homolog 3 OS=Homo sapiens GN=TTYH3 PE=1 SV=3 - [TTYH3_HUMAN]	8.03	1	4	4	7	0.764	0.500	0.613	0.705	13.78	8.03	6	7	523	57.5	5.39
P68133	Actin, alpha skeletal muscle OS=Homo sapiens GN=ACTA1 PE=1 SV=1 - [ACTS_HUMAN]	77.45	1	3	36	4663	0.879	1.034	1.217	0.705	#####	77.45	59	4663	377	42.0	5.39
Q86V21	Acetoacetyl-CoA synthetase OS=Homo sapiens GN=AACS PE=1 SV=1 - [AACS_HUMAN]	7.89	1	6	7	15	0.776	0.753	1.018	0.705	26.97	7.89	8	15	672	75.1	6.24
P15848	Arylsulfatase B OS=Homo sapiens GN=ARSB PE=1 SV=1 - [ARSB_HUMAN]	18.39	2	9	9	25	0.540	1.364	0.893	0.706	65.64	18.39	13	25	533	59.6	8.21
Q12934	Filensin OS=Homo sapiens GN=BFSP1 PE=1 SV=3 - [BFSP1_HUMAN]	1.20	1	1	1	4	0.841	1.221	1.890	0.706	10.98	1.20	1	4	665	74.5	5.14

P50583	Bis(5'-nucleosyl)-tetraphosphatase [asymmetrical] OS=Homo sapiens GN=NUDT2 PE=1 SV=3 - [AP4A_HUMAN]	14.97	1	2	2	10	0.908	0.714	0.724	0.706	30.31	14.97	3	10	147	16.8	5.35
Q9UEW8	STE20/SPS1-related proline-alanine-rich protein kinase OS=Homo sapiens GN=STK39 PE=1 SV=3 - [STK39_HUMAN]	23.85	1	8	12	31	0.735	1.062	1.755	0.706	82.23	23.85	17	31	545	59.4	6.29
O94855	Protein transport protein Sec24D OS=Homo sapiens GN=SEC24D PE=1 SV=2 - [SC24D_HUMAN]	20.64	1	19	20	76	0.687	0.689	0.743	0.706	175.87	20.64	30	76	1032	112.9	7.25
Q06418	Tyrosine-protein kinase receptor TYRO3 OS=Homo sapiens GN=TYRO3 PE=1 SV=1 - [TYRO3_HUMAN]	0.90	1	1	1	1	0.866	0.655	1.152	0.707	2.21	0.90	1	1	890	96.8	5.67
Q8N831	Testis-specific Y-encoded-like protein 6 OS=Homo sapiens GN=TSPYL6 PE=2 SV=1 - [TSYL6_HUMAN]	8.05	1	1	2	2		1.149		0.707	2.34	8.05	2	2	410	45.8	5.68
Q96BJ8	Engulfment and cell motility protein 3 OS=Homo sapiens GN=ELMO3 PE=2 SV=3 - [ELMO3_HUMAN]	1.94	1	1	1	1	0.768	0.934	1.159	0.707	2.17	1.94	1	1	720	81.4	6.30
P28289	Tropomodulin-1 OS=Homo sapiens GN=TMOD1 PE=1 SV=1 - [TMOD1_HUMAN]	10.86	1	3	4	9	1.151	2.008	1.042	0.707	16.62	10.86	6	9	359	40.5	5.10
O75368	SH3 domain-binding glutamic acid-rich-like protein OS=Homo sapiens GN=SH3BGRL PE=1 SV=1 - [SH3L1_HUMAN]	91.23	1	11	11	135	1.069	1.307	1.190	0.707	266.99	91.23	19	135	114	12.8	5.25

Q9Y2H1	Serine/threonine-protein kinase 38-like OS=Homo sapiens GN=STK38L PE=1 SV=3 - [ST38L_HUMAN]	1.72	1	1	1	2	0.577	0.926	0.818	0.707	7.47	1.72	1	2	464	54.0	6.81
O00560	Syntenin-1 OS=Homo sapiens GN=SDCBP PE=1 SV=1 - [SDCB1_HUMAN]	12.08	1	3	3	5	0.790	0.844	1.103	0.707	8.19	12.08	4	5	298	32.4	7.53
Q8TCG5	Carnitine O-palmitoyltransferase 1, brain isoform OS=Homo sapiens GN=CPT1C PE=1 SV=1 - [CPT1C_HUMAN]	2.99	1	2	2	2	1.271	0.911	1.106	0.708	1.83	2.99	2	2	803	90.9	8.12
Q7Z304	MAM domain-containing protein 2 OS=Homo sapiens GN=MAMDC2 PE=2 SV=3 - [MAMC2_HUMAN]	1.31	1	1	1	1	0.652	0.770	0.672	0.708	0.00	1.31	1	1	686	77.5	5.16
Q15942	Zyxin OS=Homo sapiens GN=ZYX PE=1 SV=1 - [ZYX_HUMAN]	43.88	1	19	19	405	0.862	1.156	1.699	0.708	1180.55	43.88	32	405	572	61.2	6.67
Q05586	Glutamate receptor ionotropic, NMDA 1 OS=Homo sapiens GN=GRIN1 PE=1 SV=1 - [NMDZ1_HUMAN]	0.85	1	1	1	3	0.987	0.863	1.084	0.708	8.34	0.85	1	3	938	105.3	8.88
Q9UJV3	Probable E3 ubiquitin-protein ligase MID2 OS=Homo sapiens GN=MID2 PE=1 SV=3 - [TRIM1_HUMAN]	4.22	1	2	3	3	0.869	0.734	0.515	0.708	7.24	4.22	3	3	735	83.2	7.39
Q9H8Y8	Golgi reassembly-stacking protein 2 OS=Homo sapiens GN=GORASP2 PE=1 SV=3 - [GORS2_HUMA	24.12	1	8	9	44	0.883	0.823	0.810	0.708	118.07	24.12	13	44	452	47.1	4.82

Q09666	Neuroblast differentiation-associated protein AHNAK OS=Homo sapiens GN=AHNAK PE=1 SV=2 - [AHNK_HUMAN]	81.24	2	393	397	4715	1.423	1.205	1.134	0.708	#####	81.24	680	4715	5890	628.7	6.15
O14529	Homeobox protein cut-like 2 OS=Homo sapiens GN=CUX2 PE=1 SV=4 - [CUX2_HUMAN]	2.36	1	2	2	2	0.803	0.977	0.705	0.709	4.34	2.36	2	2	1486	161.6	5.48
Q07864	DNA polymerase epsilon catalytic subunit A OS=Homo sapiens GN=POLE PE=1 SV=5 - [DPOE1_HUMAN]	1.49	1	2	3	5	0.680	0.862	0.970	0.709	5.56	1.49	4	5	2286	261.4	6.39
P08648	Integrin alpha-5 OS=Homo sapiens GN=ITGA5 PE=1 SV=2 - [ITA5_HUMAN]	17.06	1	13	14	64	0.767	0.923	1.144	0.709	170.38	17.06	21	64	1049	114.5	5.77
P50148	Guanine nucleotide-binding protein G(q) subunit alpha OS=Homo sapiens GN=GNAQ PE=1 SV=4 - [GNAQ_HUMAN]	33.15	1	7	13	44	0.966	1.068	1.089	0.709	98.69	33.15	19	44	359	42.1	5.68
Q9Y2M5	Kelch-like protein 20 OS=Homo sapiens GN=KLHL20 PE=1 SV=4 - [KLH20_HUMAN]	2.63	1	1	1	1	0.812	2.041	2.324	0.710	0.00	2.63	1	1	609	67.9	6.87
Q9H3H3	UPF0696 protein C11orf68 OS=Homo sapiens GN=C11orf68 PE=1 SV=2 - [CK068_HUMAN]	13.15	1	3	3	12	0.882	0.853	0.912	0.710	30.91	13.15	6	12	251	27.3	5.45
Q9ULJ8	Neurabin-1 OS=Homo sapiens GN=PPP1R9A PE=1 SV=2 - [NEB1_HUMAN]	2.19	1	2	4	10	1.643	0.811	0.906	0.710	18.58	2.19	5	10	1098	123.3	5.10
Q9HAP2	MLX-interacting protein OS=Homo sapiens GN=MLXIP PE=1 SV=2 - [MLXIP_HUMAN]	7.94	1	6	6	10	0.486	0.535	0.614	0.710	30.67	7.94	8	10	919	101.1	8.35

P22392	Nucleoside diphosphate kinase B OS=Homo sapiens GN=NME2 PE=1 SV=1 - [NDKB_HUMAN]	84.21	2	9	14	373	0.770	0.793	0.720	0.710	751.93	84.21	25	373	152	17.3	8.41
Q9BQD3	KxDL motif-containing protein 1 OS=Homo sapiens GN=KXD1 PE=1 SV=2 - [KXDL1_HUMAN]	4.55	1	2	2	2	0.747	0.800	1.230	0.710	5.25	4.55	2	2	176	19.7	4.89
Q8WXG6	MAP kinase-activating death domain protein OS=Homo sapiens GN=MADD PE=1 SV=2 - [MADD_HUMAN]	1.52	1	2	2	3	0.698	0.510	0.785	0.710	8.30	1.52	2	3	1647	183.2	6.04
O00329	Phosphatidylinositol 4,5-bisphosphate 3-kinase catalytic subunit delta isoform OS=Homo sapiens GN=PIK3CD PE=1 SV=2 - [PK3CD_HUMAN]	0.67	1	1	1	1	0.721	0.957	0.848	0.711	2.14	0.67	1	1	1044	119.4	7.18
Q6PIY5	Uncharacterized protein C1orf228 OS=Homo sapiens GN=C1orf228 PE=2 SV=2 - [CA228_HUMAN]	4.32	1	1	2	2	1.065	0.360	0.500	0.711	3.05	4.32	2	2	440	48.8	5.74
Q8IYR2	SET and MYND domain-containing protein 4 OS=Homo sapiens GN=SMYD4 PE=2 SV=3 - [SMYD4_HUMAN]	1.62	1	1	1	3	0.778	1.418	1.662	0.711	3.09	1.62	2	3	804	89.2	6.83
P12110	Collagen alpha-2(VI) chain OS=Homo sapiens GN=COL6A2 PE=1 SV=4 - [CO6A2_HUMAN]	41.51	1	37	37	201	0.628	1.202	1.362	0.711	482.57	41.51	62	201	1019	108.5	6.21
Q9Y6R6	Zinc finger protein 780B OS=Homo sapiens GN=ZNF780B PE=2 SV=1 - [Z780B_HUMAN]	2.64	1	2	2	2	1.107	1.075	1.220	0.711	5.78	2.64	2	2	833	96.7	8.92

P50579	Methionine aminopeptidase 2 OS=Homo sapiens GN=METAP2 PE=1 SV=1 - [MAP2_HUMAN]	31.38	1	12	12	68	0.729	1.258	0.844	0.712	219.66	31.38	19	68	478	52.9	5.82
Q6EMK4	Vasorin OS=Homo sapiens GN=VASN PE=1 SV=1 - [VASN_HUMAN]	9.96	1	7	7	29	0.826	0.975	0.798	0.712	88.84	9.96	11	29	673	71.7	7.39
Q8WVJ2	NudC domain-containing protein 2 OS=Homo sapiens GN=NUDCD2 PE=1 SV=1 - [NUDC2_HUMAN]	37.58	1	5	5	19	1.037	1.519	0.968	0.712	48.97	37.58	9	19	157	17.7	5.07
P21589	5'-nucleotidase OS=Homo sapiens GN=NT5E PE=1 SV=1 - [5NTD_HUMAN]	52.44	1	26	27	148	0.926	2.102	1.120	0.712	332.69	52.44	43	148	574	63.3	7.03
Q9H223	EH domain-containing protein 4 OS=Homo sapiens GN=EHD4 PE=1 SV=1 - [EHD4_HUMAN]	36.78	2	16	26	92	0.831	1.371	1.057	0.712	193.44	36.78	41	92	541	61.1	6.76
P50452	Serpin B8 OS=Homo sapiens GN=SERPINB8 PE=1 SV=2 - [SPB8_HUMAN]	10.70	1	3	4	9	1.492	1.551	1.094	0.712	20.06	10.70	6	9	374	42.7	5.57
A1IGU5	Rho guanine nucleotide exchange factor 37 OS=Homo sapiens GN=ARHGEF37 PE=2 SV=2 - [ARH37_HUMAN	0.89	1	1	1	1	0.726	0.967	0.917	0.712	2.31	0.89	1	1	675	76.2	6.93
Q9NRX5	Serine incorporator 1 OS=Homo sapiens GN=SERINC1 PE=1 SV=1 - [SERC1_HUMAN]	3.75	1	1	1	2	0.768	0.563	0.868	0.712	5.02	3.75	2	2	453	50.5	5.85
Q8NCA9	Zinc finger protein 784 OS=Homo sapiens GN=ZNF784 PE=2 SV=1 - [ZN784_HUMAN]	6.50	1	1	1	1	0.615	0.886	0.679	0.712	0.00	6.50	1	1	323	34.2	7.80
Q8WTZ3	Zinc finger protein ENSP00000375192 OS=Homo sapiens PE=2 SV=1 - [YS049_HUMAN]	6.72	1	1	1	1	1.155	1.110	0.981	0.712	0.00	6.72	1	1	238	27.1	8.54

Q14679	Tubulin polyglutamylase TTLL4 OS=Homo sapiens GN=TTLL4 PE=1 SV=2 - [TTLL4_HUMAN]	1.75	1	2	2	2	0.668	1.088	0.919	0.713	4.97	1.75	2	2	1199	133.3	8.85
Q5SVZ6	Zinc finger MYM-type protein 1 OS=Homo sapiens GN=ZMYM1 PE=2 SV=1 - [ZMYM1_HUMAN]	1.40	1	1	1	1	1.329	0.609	1.010	0.713	2.84	1.40	1	1	1142	128.6	7.44
P39059	Collagen alpha-1(XV) chain OS=Homo sapiens GN=COL15A1 PE=1 SV=2 - [COFA1_HUMAN]	5.91	1	7	7	21	0.370	0.828	0.573	0.713	41.57	5.91	12	21	1388	141.6	5.00
Q96LR5	Ubiquitin-conjugating enzyme E2 E2 OS=Homo sapiens GN=UBE2E2 PE=1 SV=1 - [UB2E2_HUMAN]	11.44	2	1	2	6	1.034	1.707	1.484	0.713	17.60	11.44	2	6	201	22.2	7.71
Q14525	Keratin, type I cuticular Ha3-II OS=Homo sapiens GN=KRT33B PE=2 SV=3 - [KT33B_HUMAN]	4.21	7	1	2	6	0.507	0.431	0.315	0.714	9.47	4.21	3	6	404	46.2	4.84
Q8NHU3	Phosphatidylcholine:ceramide cholinephosphotransferase 2 OS=Homo sapiens GN=SGMS2 PE=1 SV=1 - [SMS2_HUMAN]	2.19	1	1	1	2	0.590	0.456	0.729	0.714	4.63	2.19	1	2	365	42.3	8.81
Q13885	Tubulin beta-2A chain OS=Homo sapiens GN=TUBB2A PE=1 SV=1 - [TBB2A_HUMAN]	63.37	1	5	23	1090	1.009	1.030	1.475	0.715	2299.56	63.37	43	1090	445	49.9	4.89
Q8TD57	Dynein heavy chain 3, axonemal OS=Homo sapiens GN=DNAH3 PE=2 SV=1 - [DYH3_HUMAN]	0.53	1	1	2	3		0.759	2.174	0.715	2.59	0.53	2	3	4116	470.5	6.43
P62633	Cellular nucleic acid-binding protein OS=Homo sapiens GN=CNBP PE=1 SV=1 - [CNBP_HUMAN]	24.86	1	4	4	13	0.781	0.548	0.758	0.715	32.72	24.86	6	13	177	19.4	7.71

O95453	Poly(A)-specific ribonuclease PARN OS=Homo sapiens GN=PARN PE=1 SV=1 - [PARN_HUMAN]	9.70	1	5	5	11	1.135	0.899	0.933	0.715	30.85	9.70	7	11	639	73.4	6.20
Q9H009	Nascent polypeptide-associated complex subunit alpha-2 OS=Homo sapiens GN=NACA2 PE=1 SV=1 - [NACA2_HUMAN]	6.98	1	1	1	22	1.152	1.200	0.782	0.715	53.01	6.98	2	22	215	23.2	4.73
Q9Y303	Putative N-acetylglucosamine-6-phosphate deacetylase OS=Homo sapiens GN=AMDHD2 PE=1 SV=2 - [NAGA_HUMAN]	7.58	1	2	2	4	1.113	1.138	0.835	0.715	7.59	7.58	3	4	409	43.7	6.70
O95295	SNARE-associated protein Snapin OS=Homo sapiens GN=SNAPIN PE=1 SV=1 - [SNAPN_HUMAN]	30.15	1	3	3	10	1.043	0.825	1.048	0.715	27.14	30.15	4	10	136	14.9	9.31
Q7Z3Z3	Piwi-like protein 3 OS=Homo sapiens GN=PIWIL3 PE=2 SV=2 - [PIWL3_HUMAN	1.47	1	1	1	2	0.712	1.389	1.854	0.716	6.73	1.47	1	2	882	101.0	9.51
P21246	Pleiotrophin OS=Homo sapiens GN=PTN PE=1 SV=1 - [PTN_HUMAN]	12.50	1	2	2	5	1.153	1.372	0.496	0.716	2.38	12.50	4	5	168	18.9	9.60
P68402	Platelet-activating factor acetylhydrolase IB subunit beta OS=Homo sapiens GN=PAFAH1B2 PE=1 SV=1 -	41.48	1	6	7	21	1.020	0.836	0.820	0.716	29.94	41.48	9	21	229	25.6	5.92
P78347	General transcription factor II-I OS=Homo sapiens GN=GTF2I PE=1 SV=2 - [GTF2I_HUMAN]	30.06	3	26	26	72	0.907	1.281	1.086	0.716	160.16	30.06	37	72	998	112.3	6.39

P51957	Serine/threonine-protein kinase Nek4 OS=Homo sapiens GN=NEK4 PE=1 SV=2 - [NEK4_HUMAN]	1.43	1	1	1	1	0.783	0.687	0.910	0.716	2.31	1.43	1	1	841	94.5	7.90
Q6UB35	Monofunctional C1-tetrahydrofolate synthase, mitochondrial OS=Homo sapiens GN=MTHFD1L PE=1 SV=1 - [C1TM_HUMAN]	19.94	1	18	19	63	0.953	0.751	0.711	0.716	126.90	19.94	33	63	978	105.7	8.06
Q06033	Inter-alpha-trypsin inhibitor heavy chain H3 OS=Homo sapiens GN=ITIH3 PE=1 SV=2 - [ITIH3_HUMAN]	7.64	1	8	8	16	0.522	0.668	0.731	0.717	24.70	7.64	12	16	890	99.8	5.74
Q8N370	Large neutral amino acids transporter small subunit 4 OS=Homo sapiens GN=SLC43A2 PE=1 SV=1 - [LAT4_HUMAN]	1.41	1	1	1	1	1.079	1.135	1.127	0.717	2.58	1.41	1	1	569	62.7	7.94
P58335	Anthrax toxin receptor 2 OS=Homo sapiens GN=ANTXR2 PE=1 SV=5 - [ANTR2_HUMAN]	10.63	1	3	6	12	0.774	1.283	0.789	0.717	15.97	10.63	10	12	489	53.6	7.46
Q9H4I9	Essential MCU regulator, mitochondrial OS=Homo sapiens GN=SMDT1 PE=1 SV=1 - [EMRE_HUMAN]	9.35	1	1	1	1	1.246	0.713	0.601	0.718	3.47	9.35	1	1	107	11.4	7.27
P35611	Alpha-adducin OS=Homo sapiens GN=ADD1 PE=1 SV=2 - [ADDA_HUMAN]	27.54	1	19	20	123	0.931	1.162	0.951	0.718	281.50	27.54	33	123	737	80.9	5.83
O75146	Huntingtin-interacting protein 1-related protein OS=Homo sapiens GN=HIP1R PE=1 SV=2 - [HIP1R_HUMAN]	6.65	1	4	6	9	0.834	1.473	0.976	0.718	20.89	6.65	7	9	1068	119.3	6.67

Q8NHU2	Cilia- and flagella-associated protein 61 OS=Homo sapiens GN=CFAP61 PE=2 SV=3 - [CFA61_HUMAN	1.62	1	2	2	2	0.831	1.293	0.755	0.718	2.29	1.62	2	2	1237	141.3	6.16
O95684	FGFR1 oncogene partner OS=Homo sapiens GN=FGFR1OP PE=1 SV=1 - [FR1OP_HUMAN]	4.01	1	1	1	1	0.817	1.403	0.949	0.718	0.00	4.01	1	1	399	43.0	4.81
P20338	Ras-related protein Rab-4A OS=Homo sapiens GN=RAB4A PE=1 SV=3 - [RAB4A_HUMAN]	30.28	2	5	7	46	0.792	0.968	0.918	0.718	117.77	30.28	11	46	218	24.4	6.07
O15344	E3 ubiquitin-protein ligase Midline-1 OS=Homo sapiens GN=MID1 PE=1 SV=1 - [TRI18_HUMAN]	10.19	1	5	6	12	0.706	0.869	0.863	0.718	24.30	10.19	9	12	667	75.2	6.80
Q92783	Signal transducing adapter molecule 1 OS=Homo sapiens GN=STAM PE=1 SV=3 - [STAM1_HUMA	26.11	1	11	13	26	0.878	0.735	0.831	0.719	67.26	26.11	20	26	540	59.1	4.82
Q15847	Adipogenesis regulatory factor OS=Homo sapiens GN=ADIRF PE=1 SV=1 - [ADIRF_HUMAN]	36.84	1	2	3	11	0.644	1.299	1.095	0.719	27.97	36.84	4	11	76	7.8	5.31
O95865	N(G),N(G)-dimethylarginine dimethylaminohydrolase 2 OS=Homo sapiens GN=DDAH2 PE=1 SV=1 - [DDAH2_HUMAN]	34.04	1	7	8	62	1.383	1.244	1.062	0.719	170.79	34.04	16	62	285	29.6	6.01
P35610	Sterol O-acyltransferase 1 OS=Homo sapiens GN=SOAT1 PE=1 SV=3 - [SOAT1_HUMAN]	4.36	1	2	2	4	0.926	0.873	0.995	0.719	9.53	4.36	3	4	550	64.7	8.94
Q05682	Caldesmon OS=Homo sapiens GN=CALD1 PE=1 SV=3 - [CALD1_HUMAN]	58.13	2	66	68	732	1.273	0.951	1.467	0.719	1863.26	58.13	105	732	793	93.2	5.66

O43150	Arf-GAP with SH3 domain, ANK repeat and PH domain-containing protein 2 OS=Homo sapiens GN=ASAP2 PE=1 SV=3 - [ASAP2_HUMAN]	4.17	1	3	5	42	0.908	0.976	0.997	0.719	80.55	4.17	8	42	1006	111.6	6.68
Q9UJ70	N-acetyl-D-glucosamine kinase OS=Homo sapiens GN=NAGK PE=1 SV=4 - [NAGK_HUMAN]	43.31	1	14	14	105	0.911	0.913	1.100	0.719	323.01	43.31	25	105	344	37.4	6.24
Q9NSY2	StAR-related lipid transfer protein 5 OS=Homo sapiens GN=STARD5 PE=1 SV=2 - [STAR5_HUMAN]	11.74	1	3	3	10	0.389	0.775	0.968	0.720	22.48	11.74	6	10	213	23.8	6.67
Q16512	Serine/threonine-protein kinase N1 OS=Homo sapiens GN=PKN1 PE=1 SV=2 - [PKN1_HUMAN]	14.54	2	9	12	22	0.860	1.038	1.213	0.720	57.16	14.54	15	22	942	103.9	6.37
Q8TDQ7	Glucosamine-6-phosphate isomerase 2 OS=Homo sapiens GN=GNPDA2 PE=1 SV=1 - [GNPI2_HUMAN]	26.09	1	4	6	15	0.717	0.687	0.680	0.720	28.69	26.09	8	15	276	31.1	6.95
Q7Z7M0	Multiple epidermal growth factor-like domains protein 8 OS=Homo sapiens GN=MEGF8 PE=1 SV=2 - [MEGF8_HUMAN]	0.46	1	1	1	3	0.803	1.148	0.950	0.720	9.65	0.46	1	3	2845	302.9	6.87
Q6IN84	rRNA methyltransferase 1, mitochondrial OS=Homo sapiens GN=MRM1 PE=1 SV=1 - [MRM1_HUMAN]	5.10	1	1	2	4	0.572	1.809	1.257	0.720	6.84	5.10	3	4	353	38.6	7.94
P52594	Arf-GAP domain and FG repeat-containing protein 1 OS=Homo sapiens GN=AGFG1 PE=1 SV=2 - [AGFG1_HUMAN]	11.39	1	5	5	21	0.805	0.679	0.866	0.720	62.37	11.39	7	21	562	58.2	8.63

Q496M5	Inactive serine/threonine-protein kinase PLK5 OS=Homo sapiens GN=PLK5 PE=1 SV=4 - [PLK5_HUMAN]	8.33	1	1	1	2	0.676	1.081	1.338	0.721	0.00	8.33	1	2	336	36.3	6.47
Q8NDA8	Maestro heat-like repeat-containing protein family member 1 OS=Homo sapiens GN=MROH1 PE=2 SV=3 - [MROH1_HUMAN]	0.85	1	2	2	2	0.918	0.917	0.988	0.722	2.44	0.85	2	2	1641	181.1	6.89
Q8TC20	Cancer-associated gene 1 protein OS=Homo sapiens GN=CAGE1 PE=1 SV=2 - [CAGE1_HUMAN]	1.80	1	1	1	1	1.053	0.778	1.266	0.722	0.00	1.80	1	1	777	90.2	5.29
O60447	Ecotropic viral integration site 5 protein homolog OS=Homo sapiens GN=EVI5 PE=1 SV=3 - [EVI5_HUMAN]	6.54	1	6	6	14	0.988	0.854	1.030	0.722	30.46	6.54	8	14	810	92.9	6.10
Q7RTR2	Protein NLRC3 OS=Homo sapiens GN=NLRC3 PE=2 SV=2 - [NLRC3_HUMAN]	0.66	1	1	1	1	1.220	1.126	0.944	0.722	2.39	0.66	1	1	1065	114.6	8.29
P51788	Chloride channel protein 2 OS=Homo sapiens GN=CLCN2 PE=1 SV=2 - [CLCN2_HUMAN	3.90	1	3	3	7	0.551	1.042	1.433	0.722	15.82	3.90	3	7	898	98.5	8.38
Q12893	Transmembrane protein 115 OS=Homo sapiens GN=TMEM115 PE=1 SV=1 - [TM115_HUMAN]	11.11	1	2	3	6	1.099	1.319	1.612	0.722	21.33	11.11	3	6	351	38.2	8.16
Q9UJ42	Probable G-protein coupled receptor 160 OS=Homo sapiens GN=GPR160 PE=2 SV=1 - [GP160_HUMAN	3.25	1	1	1	1	0.896	1.347	1.200	0.722	2.38	3.25	1	1	338	39.8	8.51

O60331	Phosphatidylinositol 4-phosphate 5-kinase type-1 gamma OS=Homo sapiens GN=PIP5K1C PE=1 SV=2 - [PI51C_HUMAN]	6.89	1	3	3	5	0.751	1.004	1.104	0.722	12.28	6.89	5	5	668	73.2	5.29
O43301	Heat shock 70 kDa protein 12A OS=Homo sapiens GN=HSPA12A PE=1 SV=2 - [HS12A_HUMAN]	10.52	1	6	6	15	0.837	1.472	1.520	0.722	30.77	10.52	11	15	675	74.9	6.77
Q9ULC8	Probable palmitoyltransferase ZDHHC8 OS=Homo sapiens GN=ZDHHC8 PE=1 SV=3 - [ZDHC8_HUMAN]	7.97	1	3	3	4	1.227	1.086	0.889	0.723	10.29	7.97	3	4	765	81.4	9.17
Q12983	BCL2/adenovirus E1B 19 kDa protein-interacting protein 3 OS=Homo sapiens GN=BNIP3 PE=1 SV=2 - [BNIP3_HUMAN]	5.67	1	1	1	4	1.203	1.279	1.193	0.723	10.21	5.67	2	4	194	21.5	6.80
Q13433	Zinc transporter ZIP6 OS=Homo sapiens GN=SLC39A6 PE=1 SV=3 - [S39A6_HUMAN	1.19	1	1	1	2	0.670	1.160	0.722	0.723	1.80	1.19	2	2	755	85.0	6.95
P46736	Lys-63-specific deubiquitinase BRCC36 OS=Homo sapiens GN=BRCC3 PE=1 SV=2 - [BRCC3_HUMAN]	15.19	1	4	4	11	0.891	0.917	0.737	0.723	30.34	15.19	6	11	316	36.0	5.92
Q9P0K9	DOMON domain-containing protein FRRS1L OS=Homo sapiens GN=FRRS1L PE=2 SV=2 - [FRS1L_HUMAN]	10.76	1	1	1	1	1.616	1.674	0.942	0.723	2.71	10.76	1	1	344	37.2	7.62
P13987	CD59 glycoprotein OS=Homo sapiens GN=CD59 PE=1 SV=1 - [CD59_HUMAN]	25.00	1	4	4	62	0.801	0.771	0.723	0.723	174.71	25.00	7	62	128	14.2	6.48

Q6P4A8	Phospholipase B-like 1 OS=Homo sapiens GN=PLBD1 PE=1 SV=2 - [PLBL1_HUMAN]	4.16	1	1	1	1	0.329	3.239	1.475	0.723	0.00	4.16	1	1	553	63.2	9.06
P56199	Integrin alpha-1 OS=Homo sapiens GN=ITGA1 PE=1 SV=2 - [ITA1_HUMAN]	22.22	1	28	29	122	0.938	0.452	0.990	0.724	265.34	22.22	49	122	1179	130.8	6.29
Q9H943	Uncharacterized protein C10orf68 OS=Homo sapiens GN=C10orf68 PE=2 SV=2 - [CJ068_HUMAN]	2.87	1	1	2	3	0.504	1.060	1.476	0.724	0.00	2.87	2	3	628	71.5	9.35
O00716	Transcription factor E2F3 OS=Homo sapiens GN=E2F3 PE=1 SV=1 - [E2F3_HUMAN]	4.95	1	2	2	2	0.512	1.320	0.610	0.724	5.28	4.95	2	2	465	49.1	5.44
Q96SL1	Disrupted in renal carcinoma protein 2 OS=Homo sapiens GN=DIRC2 PE=1 SV=1 - [DIRC2_HUMAN	6.28	1	2	2	7	0.915	0.869	0.783	0.724	11.39	6.28	3	7	478	52.1	8.47
Q9BTT0	Acidic leucine-rich nuclear phosphoprotein 32 family member E OS=Homo sapiens GN=ANP32E PE=1 SV=1 - [AN32E_HUMAN]	20.90	1	4	5	7	0.690	0.900	0.821	0.724	19.77	20.90	5	7	268	30.7	3.85
P07814	Bifunctional glutamate/proline--tRNA ligase OS=Homo sapiens GN=EPRS PE=1 SV=5 - [SYEP_HUMAN]	46.56	2	66	67	446	0.705	0.834	0.822	0.724	1083.23	46.56	111	446	1512	170.5	7.33
P20061	Transcobalamin-1 OS=Homo sapiens GN=TCN1 PE=1 SV=2 - [TCO1_HUMAN]	1.62	1	1	1	2	0.744	0.687	0.580	0.725	4.14	1.62	1	2	433	48.2	5.03
P02549	Spectrin alpha chain, erythrocytic 1 OS=Homo sapiens GN=SPTA1 PE=1 SV=5 - [SPTA1_HUMAN]	1.74	1	2	5	12	1.365	0.310	0.224	0.725	22.17	1.74	6	12	2419	279.8	5.05

P11532	Dystrophin OS=Homo sapiens GN=DMD PE=1 SV=3 - [DMD_HUMAN]	5.51	1	14	19	29	0.894	1.835	2.185	0.725	56.03	5.51	24	29	3685	426.5	5.88
Q16643	Drebrin OS=Homo sapiens GN=DBN1 PE=1 SV=4 - [DREB_HUMAN]	40.68	1	23	23	237	0.792	0.914	1.108	0.726	698.35	40.68	37	237	649	71.4	4.45
Q9C0F0	Putative Polycomb group protein ASXL3 OS=Homo sapiens GN=ASXL3 PE=2 SV=3 - [ASXL3_HUMAN]	0.80	1	1	2	3	1.344	0.699	1.162	0.726	6.45	0.80	3	3	2248	241.8	6.14
Q9NUE0	Palmitoyltransf erase ZDHHC18 OS=Homo sapiens GN=ZDHHC18 PE=2 SV=2 - [ZDH18_HUMAN]	3.61	1	1	1	2	1.152	1.025	0.685	0.726	3.83	3.61	2	2	388	42.0	9.04
Q4ZHG4	Fibronectin type III domain-containing protein 1 OS=Homo sapiens GN=FNDC1 PE=1 SV=4 - [FNDC1_HUMAN	2.59	1	2	2	2	0.338	0.733	0.680	0.726	4.34	2.59	2	2	1894	205.4	9.32
Q9NUP9	Protein lin-7 homolog C OS=Homo sapiens GN=LIN7C PE=1 SV=1 - [LIN7C_HUMAN]	70.05	2	6	13	35	1.197	0.904	0.958	0.726	83.58	70.05	18	35	197	21.8	8.43
Q8IY22	C-Maf-inducing protein OS=Homo sapiens GN=CMIP PE=1 SV=3 - [CMIP_HUMAN]	2.85	1	2	2	2	0.549	0.556	0.785	0.726	2.34	2.85	2	2	773	86.3	6.70
Q96GD3	Polycomb protein SCMH1 OS=Homo sapiens GN=SCMH1 PE=1 SV=1 - [SCMH1_HUMA N]	1.36	1	1	1	1	0.541	0.600	0.672	0.726	2.22	1.36	1	1	660	73.3	9.25
Q8NCG7	Sn1-specific diacylglycerol lipase beta OS=Homo sapiens GN=DAGLB PE=1 SV=2 - [DGLB_HUMAN]	2.53	1	2	2	3	0.726	0.645	0.692	0.726	0.00	2.53	3	3	672	73.7	6.55

O75436	Vacuolar protein sorting-associated protein 26A OS=Homo sapiens GN=VPS26A PE=1 SV=2 - [VP26A_HUMAN]	38.84	1	12	13	26	1.080	0.914	0.858	0.726	54.54	38.84	18	26	327	38.1	6.57
P50552	Vasodilator-stimulated phosphoprotein OS=Homo sapiens GN=VASP PE=1 SV=3 - [VASP_HUMAN]	37.89	1	15	15	177	0.854	1.138	1.599	0.726	386.40	37.89	27	177	380	39.8	8.94
O15061	Synemin OS=Homo sapiens GN=SYNM PE=1 SV=2 - [SYNEM_HUMAN]	31.69	1	43	43	133	1.006	3.910	2.767	0.727	355.34	31.69	65	133	1565	172.7	5.16
P15151	Poliovirus receptor OS=Homo sapiens GN=PVR PE=1 SV=2 - [PVR_HUMAN]	17.03	1	6	6	39	1.728	0.560	0.932	0.727	86.52	17.03	12	39	417	45.3	6.52
Q96C36	Pyrroline-5-carboxylate reductase 2 OS=Homo sapiens GN=PYCR2 PE=1 SV=1 - [P5CR2_HUMAN]	20.63	1	7	8	23	0.589	0.840	0.912	0.727	51.81	20.63	11	23	320	33.6	7.77
Q6W2J9	BCL-6 corepressor OS=Homo sapiens GN=BCOR PE=1 SV=1 - [BCOR_HUMAN]	1.54	1	2	2	5	0.946	0.844	0.957	0.727	8.97	1.54	2	5	1755	192.1	6.48
P15586	N-acetylglucosamine-6-sulfatase OS=Homo sapiens GN=GNS PE=1 SV=3 - [GNS_HUMAN]	23.19	1	12	12	49	0.756	1.315	1.109	0.728	79.08	23.19	19	49	552	62.0	8.31
Q9UNH6	Sorting nexin-7 OS=Homo sapiens GN=SNX7 PE=1 SV=1 - [SNX7_HUMAN]	10.08	1	5	5	14	0.971	0.979	0.955	0.728	26.90	10.08	7	14	387	45.3	5.11
O60716	Catenin delta-1 OS=Homo sapiens GN=CTNND1 PE=1 SV=1 - [CTND1_HUMAN]	36.26	1	32	32	191	1.201	1.022	1.250	0.728	375.47	36.26	55	191	968	108.1	6.23
Q6ZU65	Ubinuclein-2 OS=Homo sapiens GN=UBN2 PE=1 SV=2 - [UBN2_HUMAN]	3.04	1	3	3	6	0.591	2.160	3.290	0.728	1.81	3.04	3	6	1347	146.0	9.19

Q9NX47	E3 ubiquitin-protein ligase MARCH5 OS=Homo sapiens GN=MARCH5 PE=1 SV=1 - [MARH5_HUMAN]	10.79	1	2	2	4	0.899	0.721	0.878	0.728	4.36	10.79	3	4	278	31.2	8.70
P12074	Cytochrome c oxidase subunit 6A1, mitochondrial OS=Homo sapiens GN=COX6A1 PE=1 SV=4 - [CX6A1_HUMAN]	43.12	1	2	2	6	0.804	0.936	0.784	0.728	13.29	43.12	3	6	109	12.1	9.32
Q5VT06	Centrosome-associated protein 350 OS=Homo sapiens GN=CEP350 PE=1 SV=1 - [CE350_HUMAN]	0.19	1	1	1	1	0.139	0.596	1.086	0.728	2.14	0.19	1	1	3117	350.7	6.33
Q8N999	Uncharacterized protein C12orf29 OS=Homo sapiens GN=C12orf29 PE=1 SV=2 - [CL029_HUMAN]	6.46	1	2	2	4	0.668	0.849	0.865	0.728	11.22	6.46	3	4	325	37.5	7.06
Q9Y2N7	Hypoxia-inducible factor 3-alpha OS=Homo sapiens GN=HIF3A PE=1 SV=2 - [HIF3A_HUMAN]	4.78	1	2	2	2	1.125	0.997	0.856	0.729	4.96	4.78	2	2	669	72.4	6.02
Q9NZK5	Adenosine deaminase CECR1 OS=Homo sapiens GN=CECR1 PE=1 SV=2 - [CECR1_HUMAN]	7.83	1	2	2	2	0.912	0.922	0.856	0.729	2.59	7.83	2	2	511	58.9	7.91
O95302	Peptidyl-prolyl cis-trans isomerase FKBP9 OS=Homo sapiens GN=FKBP9 PE=1 SV=2 - [FKBP9_HUMAN]	33.33	2	21	22	149	0.925	0.763	0.848	0.729	332.71	33.33	38	149	570	63.0	5.08
Q9ULP9	TBC1 domain family member 24 OS=Homo sapiens GN=TBC1D24 PE=1 SV=2 - [TBC24_HUMAN]	11.45	1	6	6	24	0.985	0.694	0.962	0.729	60.70	11.45	11	24	559	62.9	7.36
Q9Y5X2	Sorting nexin-8 OS=Homo sapiens GN=SNX8 PE=1 SV=1 - [SNX8_HUMAN]	5.81	1	2	2	7	0.440	4.164	0.987	0.729	3.28	5.81	2	7	465	52.5	7.39

Q8N2C7	Protein unc-80 homolog OS=Homo sapiens GN=UNC80 PE=2 SV=2 - [UNC80_HUMAN]	0.28	1	1	1	1	0.536	0.235	0.403	0.730	2.07	0.28	1	1	3258	363.2	6.86
P07864	L-lactate dehydrogenase C chain OS=Homo sapiens GN=LDHC PE=1 SV=4 - [LDHC_HUMAN]	5.72	1	1	2	65	1.209	1.911	1.490	0.730	136.30	5.72	3	65	332	36.3	7.46
Q00536	Cyclin-dependent kinase 16 OS=Homo sapiens GN=CDK16 PE=1 SV=1 - [CDK16_HUMAN]	13.10	6	4	7	18	0.948	1.100	0.885	0.730	38.52	13.10	9	18	496	55.7	7.62
P31629	Transcription factor HIVEP2 OS=Homo sapiens GN=HIVEP2 PE=1 SV=2 - [ZEP2_HUMAN]	0.65	1	1	1	1	1.276	0.876	1.333	0.730	2.36	0.65	1	1	2446	268.9	6.96
Q96QE3	ATPase family AAA domain-containing protein 5 OS=Homo sapiens GN=ATAD5 PE=1 SV=4 - [ATAD5_HUMAN]	0.98	1	2	2	2	0.609	1.165	0.660	0.731	0.00	0.98	2	2	1844	207.4	9.19
O00408	cGMP-dependent 3',5'-cyclic phosphodiesterase OS=Homo sapiens GN=PDE2A PE=1 SV=1 - [PDE2A_HUMAN]	0.64	1	1	1	1	0.631	0.746	0.697	0.731	1.96	0.64	1	1	941	105.6	5.41
P58005	Sestrin-3 OS=Homo sapiens GN=SESN3 PE=2 SV=2 - [SESN3_HUMAN]	6.30	1	3	3	7	0.924	0.690	1.110	0.731	16.06	6.30	5	7	492	57.3	6.25
Q96A73	Putative monooxygenase p33MONOX OS=Homo sapiens GN=KIAA1191 PE=1 SV=1 - [P33MX_HUMAN]	16.07	1	5	5	12	1.044	1.455	1.154	0.731	23.45	16.07	8	12	305	33.2	9.39
Q9Y639	Neuroplastin OS=Homo sapiens GN=NPTN PE=1 SV=2 - [NPTN_HUMAN]	18.59	1	7	7	51	1.043	1.193	1.194	0.731	115.80	18.59	13	51	398	44.4	7.99

Q96GE4	Centrosomal protein of 95 kDa OS=Homo sapiens GN=CEP95 PE=1 SV=1 - [CEP95_HUMAN]	1.22	1	1	1	1	0.698	0.792	1.032	0.731	0.00	1.22	1	1	821	95.2	8.59
P12429	Annexin A3 OS=Homo sapiens GN=ANXA3 PE=1 SV=3 - [ANXA3_HUMAN]	41.80	1	13	15	41	0.585	6.231	1.324	0.731	110.46	41.80	20	41	323	36.4	5.92
Q16690	Dual specificity protein phosphatase 5 OS=Homo sapiens GN=DUSP5 PE=1 SV=2 - [DUS5_HUMAN]	5.21	1	1	1	1	1.236	1.734	0.937	0.732	0.00	5.21	1	1	384	42.0	8.35
P20042	Eukaryotic translation initiation factor 2 subunit 2 OS=Homo sapiens GN=EIF2S2 PE=1 SV=2 - [IF2B_HUMAN]	42.04	1	15	15	110	0.859	0.853	0.854	0.732	287.02	42.04	25	110	333	38.4	5.80
Q9HCR9	Dual 3',5'-cyclic-AMP and -GMP phosphodiesterase 11A OS=Homo sapiens GN=PDE11A PE=1 SV=2 - [PDE11_HUMAN]	2.79	1	2	2	2	0.730	0.915	0.858	0.732	2.77	2.79	2	2	933	104.7	6.60
Q9P0J6	39S ribosomal protein L36, mitochondrial OS=Homo sapiens GN=MRPL36 PE=1 SV=1 - [RM36_HUMAN]	5.83	1	1	1	1	0.742	0.552	0.637	0.732	2.28	5.83	1	1	103	11.8	11.27
Q96RL7	Vacuolar protein sorting-associated protein 13A OS=Homo sapiens GN=VPS13A PE=1 SV=2 - [VP13A_HUMAN]	1.48	1	3	4	6	0.854	1.350	1.091	0.732	8.89	1.48	5	6	3174	360.0	6.33
P20930	Filaggrin OS=Homo sapiens GN=FLG PE=1 SV=3 - [FILA_HUMAN]	0.34	1	2	2	3	1.171	1.027	0.577	0.732	7.57	0.34	2	3	4061	434.9	9.25
Q13308	Inactive tyrosine-protein kinase 7 OS=Homo sapiens GN=PTK7 PE=1 SV=2 - [PTK7_HUMAN]	27.85	1	24	24	79	1.032	0.753	0.651	0.732	212.44	27.85	35	79	1070	118.3	7.09

Q14558	Phosphoribosyl pyrophosphate synthase-associated protein 1 OS=Homo sapiens GN=PRPSAP1 PE=1 SV=2 - [KPRA_HUMAN]	38.48	1	8	11	35	0.576	0.885	0.742	0.733	114.65	38.48	18	35	356	39.4	7.20
P21333	Filamin-A OS=Homo sapiens GN=FLNA PE=1 SV=4 - [FLNA_HUMAN]	69.81	1	159	178	5362	0.946	1.021	1.351	0.733	#####	69.81	305	5362	2647	280.6	6.06
Q8TAX9	Gasdermin-B OS=Homo sapiens GN=GSDMB PE=1 SV=2 - [GSDMB_HUMAN]	4.62	1	2	2	2	1.118	0.886	1.524	0.733	0.00	4.62	2	2	411	46.8	5.26
Q09MP3	RAD51-associated protein 2 OS=Homo sapiens GN=RAD51AP2 PE=1 SV=1 - [R51A2_HUMAN]	0.86	1	1	1	1	0.573	0.861	1.022	0.733	1.82	0.86	1	1	1159	133.8	7.34
P59510	A disintegrin and metalloproteinase with thrombospondin motifs 20 OS=Homo sapiens GN=ADAMTS20 PE=2 SV=2 - [ATS20_HUMAN]	0.63	1	1	1	2	0.959	0.219	0.738	0.733	0.00	0.63	1	2	1910	214.6	7.21
P62879	Guanine nucleotide-binding protein G(I)/G(S)/G(T) subunit beta-2 OS=Homo sapiens GN=GNB2 PE=1 SV=3 - [GBB2_HUMAN]	29.41	1	4	8	44	0.881	0.705	0.682	0.733	116.98	29.41	13	44	340	37.3	6.00
Q8IVH8	Mitogen-activated protein kinase kinase kinase kinase 3 OS=Homo sapiens GN=MAP4K3 PE=1 SV=1 - [M4K3_HUMAN]	5.48	1	4	4	11	0.892	0.913	0.848	0.734	30.74	5.48	5	11	894	101.3	7.56
Q9NW68	BSD domain-containing protein 1 OS=Homo sapiens GN=BSDC1 PE=1 SV=1 - [BSDC1_HUMAN]	11.40	1	4	4	16	0.849	0.984	0.993	0.734	41.42	11.40	7	16	430	47.1	4.49

Q9P0U1	Mitochondrial import receptor subunit TOM7 homolog OS=Homo sapiens GN=TOMM7 PE=1 SV=1 - [TOM7_HUMAN	54.55	1	3	3	8	0.814	0.782	0.588	0.734	15.04	54.55	4	8	55	6.2	10.29
Q8WWB3	DPY30 domain-containing protein 1 OS=Homo sapiens GN=DYDC1 PE=1 SV=1 - [DYDC1_HUMAN]	3.95	1	1	1	1		0.992	0.836	0.734	2.30	3.95	1	1	177	20.9	4.67
Q5TGL8	PX domain-containing protein 1 OS=Homo sapiens GN=PXDC1 PE=2 SV=3 - [PXDC1_HUMAN]	4.33	1	1	1	3	0.561	0.716	0.664	0.734	5.08	4.33	2	3	231	26.5	5.03
P35222	Catenin beta-1 OS=Homo sapiens GN=CTNNB1 PE=1 SV=1 - [CTNB1_HUMAN]	27.66	1	15	18	94	0.943	1.056	0.995	0.734	234.45	27.66	29	94	781	85.4	5.86
O75563	Src kinase-associated phosphoprotei n 2 OS=Homo sapiens GN=SKAP2 PE=1 SV=1 - [SKAP2_HUMAN]	1.67	1	1	1	1	1.144	0.716	0.569	0.734	2.22	1.67	1	1	359	41.2	4.69
P56555	Down syndrome critical region protein 4 OS=Homo sapiens GN=DSCR4 PE=2 SV=1 - [DSCR4_HUMAN]	8.47	1	1	1	3	0.666	1.283	1.048	0.734	0.00	8.47	1	3	118	12.9	7.91
Q9Y6R0	Numb-like protein OS=Homo sapiens GN=NUMBL PE=1 SV=1 - [NUMBL_HUMAN]	18.88	1	8	11	39	0.927	1.131	1.178	0.734	81.55	18.88	21	39	609	64.9	8.85
O60678	Protein arginine N-methyltransfer ase 3 OS=Homo sapiens GN=PRMT3 PE=1 SV=3 - [ANM3_HUMAN]	2.45	1	1	1	1	0.692	0.564	0.533	0.735	5.39	2.45	1	1	531	59.8	5.35
O00505	Importin subunit alpha-4 OS=Homo sapiens GN=KPNA3 PE=1 SV=2 - [IMA4_HUMAN]	11.13	1	2	7	31	0.687	0.604	0.579	0.735	50.50	11.13	10	31	521	57.8	4.94

O43490	Prominin-1 OS=Homo sapiens GN=PROM1 PE=1 SV=1 - [PROM1_HUMAN]	1.73	1	1	1	2	1.094	1.070	1.241	0.735	4.17	1.73	1	2	865	97.1	7.27
P0CJ78	Zinc finger protein 865 OS=Homo sapiens GN=ZNF865 PE=3 SV=1 - [ZN865_HUMAN]	0.94	1	1	1	1	1.139	1.044	1.659	0.735	0.00	0.94	1	1	1059	111.0	9.19
Q6UVK1	Chondroitin sulfate proteoglycan 4 OS=Homo sapiens GN=CSPG4 PE=1 SV=2 - [CSPG4_HUMAN]	2.84	1	6	7	30	1.243	2.769	1.871	0.735	40.30	2.84	11	30	2322	250.4	5.47
Q86VV8	Rotatin OS=Homo sapiens GN=RTTN PE=1 SV=3 - [RTTN_HUMAN]	0.72	1	1	2	2	1.352	0.522	0.772	0.735	2.28	0.72	2	2	2226	248.5	6.70
Q92896	Golgi apparatus protein 1 OS=Homo sapiens GN=GLG1 PE=1 SV=2 - [GSLG1_HUMAN	25.28	1	31	31	147	0.659	0.877	0.810	0.735	352.89	25.28	50	147	1179	134.5	6.90
A6NIX2	Wilms tumor protein 1-interacting protein OS=Homo sapiens GN=WTIP PE=1 SV=3 - [WTIP_HUMAN]	5.81	1	2	2	2	1.158	1.796	1.823	0.735	2.29	5.81	2	2	430	45.1	8.12
Q8TC21	Zinc finger protein 596 OS=Homo sapiens GN=ZNF596 PE=2 SV=2 - [ZN596_HUMAN]	2.38	1	1	1	2	1.168	1.166	0.536	0.735	0.00	2.38	1	2	504	58.5	8.92
Q9UN81	LINE-1 retrotransposable element ORF1 protein OS=Homo sapiens GN=L1RE1 PE=1 SV=1 - [LORF1_HUMAN]	2.66	1	1	1	1	1.156	1.881	0.654	0.736	1.72	2.66	1	1	338	40.0	9.51
A6ZKI3	Protein FAM127A OS=Homo sapiens GN=FAM127A PE=1 SV=1 - [F127A_HUMAN]	15.04	3	2	2	2	0.741	0.846	0.757	0.736	4.85	15.04	2	2	113	13.2	5.07

Q13404	Ubiquitin-conjugating enzyme E2 variant 1 OS=Homo sapiens GN=UBE2V1 PE=1 SV=2 - [UB2V1_HUMAN]	46.26	1	3	7	136	0.873	0.861	1.049	0.736	388.87	46.26	12	136	147	16.5	7.93
O95081	Arf-GAP domain and FG repeat-containing protein 2 OS=Homo sapiens GN=AGFG2 PE=1 SV=2 - [AGFG2_HUMAN]	6.86	1	3	3	3	0.635	0.772	1.096	0.736	5.45	6.86	3	3	481	48.9	9.11
Q15208	Serine/threonine-protein kinase 38 OS=Homo sapiens GN=STK38 PE=1 SV=1 - [STK38_HUMAN	13.12	1	5	5	19	1.065	0.903	1.238	0.736	68.84	13.12	10	19	465	54.2	7.15
Q659A1	Little elongation complex subunit 2 OS=Homo sapiens GN=ICE2 PE=1 SV=2 - [ICE2_HUMAN]	4.28	1	3	3	5	1.104	0.904	0.778	0.737	2.31	4.28	4	5	982	109.9	7.09
Q16851	UTP--glucose-1-phosphate uridylyltransferase OS=Homo sapiens GN=UGP2 PE=1 SV=5 - [UGPA_HUMAN]	49.21	1	27	27	117	0.841	1.141	0.996	0.737	255.23	49.21	43	117	508	56.9	8.15
Q562R1	Beta-actin-like protein 2 OS=Homo sapiens GN=ACTBL2 PE=1 SV=2 - [ACTBL_HUMAN]	45.74	1	8	20	2039	1.043	0.814	1.169	0.737	5603.95	45.74	32	2039	376	42.0	5.59
P80404	4-aminobutyrate aminotransferase, mitochondrial OS=Homo sapiens GN=ABAT PE=1 SV=3 - [GABT_HUMAN]	2.00	1	1	1	3	0.599	0.580	0.701	0.737	8.66	2.00	2	3	500	56.4	7.96
P17174	Aspartate aminotransferase, cytoplasmic OS=Homo sapiens GN=GOT1 PE=1 SV=3 - [AATC_HUMAN]	33.41	1	14	14	51	0.810	1.197	0.999	0.738	111.99	33.41	22	51	413	46.2	7.01

Q9Y6I8	Peroxisomal membrane protein 4 OS=Homo sapiens GN=PXMP4 PE=1 SV=3 - [PXMP4_HUMAN]	3.77	1	1	1	1	1.055	0.646	0.727	0.738	2.01	3.77	1	1	212	24.2	10.07
Q14194	Dihydropyrimidinase-related protein 1 OS=Homo sapiens GN=CRMP1 PE=1 SV=1 - [DPYL1_HUMAN]	13.29	1	3	6	73	0.780	2.187	5.743	0.738	164.27	13.29	10	73	572	62.1	7.03
Q9Y576	Ankyrin repeat and SOCS box protein 1 OS=Homo sapiens GN=ASB1 PE=1 SV=1 - [ASB1_HUMAN]	2.69	1	1	1	3	0.680	0.691	0.873	0.738	4.41	2.69	2	3	335	37.0	8.21
Q96PR1	Potassium voltage-gated channel subfamily C member 2 OS=Homo sapiens GN=KCNC2 PE=1 SV=1 - [KCNC2_HUMAN]	1.41	1	1	1	1	0.718	0.589	0.577	0.738	1.82	1.41	1	1	638	70.2	7.96
Q0VDI3	Transmembrane protein C5orf28 OS=Homo sapiens GN=C5orf28 PE=2 SV=1 - [CE028_HUMAN]	6.51	1	1	1	1	0.451	0.709	0.938	0.738	0.00	6.51	1	1	215	24.2	8.59
P30622	CAP-Gly domain-containing linker protein 1 OS=Homo sapiens GN=CLIP1 PE=1 SV=2 - [CLIP1_HUMAN]	43.67	1	58	62	238	0.943	0.838	0.859	0.738	572.57	43.67	96	238	1438	162.1	5.36
P11169	Solute carrier family 2, facilitated glucose transporter member 3 OS=Homo sapiens GN=SLC2A3 PE=1 SV=1 - [GTR3_HUMAN]	1.41	2	1	1	2	0.839	0.868	0.746	0.739	2.18	1.41	1	2	496	53.9	7.20
Q9Y696	Chloride intracellular channel protein 4 OS=Homo sapiens GN=CLIC4 PE=1 SV=4 - [CLIC4_HUMAN]	75.49	3	19	20	171	0.747	0.965	1.017	0.739	429.90	75.49	32	171	253	28.8	5.59

P27986	Phosphatidylinositol 3-kinase regulatory subunit alpha OS=Homo sapiens GN=PIK3R1 PE=1 SV=2 - [P85A_HUMAN]	13.26	2	8	10	24	0.663	0.947	1.043	0.739	50.62	13.26	16	24	724	83.5	6.16
Q9BZA8	Protocadherin-11 Y-linked OS=Homo sapiens GN=PCDH11Y PE=1 SV=1 - [PC11Y_HUMAN]	0.67	2	1	1	1	0.635	0.718	0.331	0.739	1.83	0.67	1	1	1340	146.7	5.21
Q07960	Rho GTPase-activating protein 1 OS=Homo sapiens GN=ARHGAP1 PE=1 SV=1 - [RHG01_HUMAN]	50.57	1	20	20	173	0.726	1.032	1.234	0.739	482.85	50.57	34	173	439	50.4	6.29
Q8IZS8	Voltage-dependent calcium channel subunit alpha-2/delta-3 OS=Homo sapiens GN=CACNA2D3 PE=1 SV=1 - [CA2D3_HUMAN]	2.11	1	2	2	3	1.000	1.101	1.043	0.739	6.79	2.11	2	3	1091	122.9	5.78
P78363	Retinal-specific ATP-binding cassette transporter OS=Homo sapiens GN=ABCA4 PE=1 SV=3 - [ABCA4_HUMAN]	0.31	1	1	1	2	0.807	0.289	0.439	0.739	4.85	0.31	1	2	2273	255.8	6.29
Q8TCQ1	E3 ubiquitin-protein ligase MARCH1 OS=Homo sapiens GN=MARCH1 PE=1 SV=1 - [MARH1_HUMAN]	2.08	2	1	1	1	0.724	0.458	0.896	0.739	0.00	2.08	1	1	289	32.3	8.37
O75381	Peroxisomal membrane protein PEX14 OS=Homo sapiens GN=PEX14 PE=1 SV=1 - [PEX14_HUMAN]	12.20	1	3	4	8	1.027	1.395	1.550	0.740	30.31	12.20	5	8	377	41.2	4.94
Q9Y3Q7	Disintegrin and metalloproteinase domain-containing protein 18 OS=Homo sapiens GN=ADAM18 PE=2 SV=1 - [ADA18_HUMAN]	2.44	1	1	1	1	0.970	0.831	1.798	0.740	0.00	2.44	1	1	739	82.8	7.28

P10721	Mast/stem cell growth factor receptor Kit OS=Homo sapiens GN=KIT PE=1 SV=1 - [KIT_HUMAN]	1.64	5	1	2	4	0.868	0.898	0.987	0.740	9.37	1.64	3	4	976	109.8	6.98
Q9BTV4	Transmembrane protein 43 OS=Homo sapiens GN=TMEM43 PE=1 SV=1 - [TMM43_HUMAN]	46.50	3	19	19	108	0.858	0.840	0.926	0.740	216.86	46.50	32	108	400	44.8	8.13
Q96D15	Reticulocalbin-3 OS=Homo sapiens GN=RCN3 PE=1 SV=1 - [RCN3_HUMAN]	48.78	1	14	14	282	0.691	0.630	0.831	0.741	760.86	48.78	26	282	328	37.5	4.89
Q99933	BAG family molecular chaperone regulator 1 OS=Homo sapiens GN=BAG1 PE=1 SV=4 - [BAG1_HUMAN]	2.32	1	1	1	2	1.262	1.792	0.770	0.741	4.27	2.32	1	2	345	38.8	7.81
Q96S38	Ribosomal protein S6 kinase delta-1 OS=Homo sapiens GN=RPS6KC1 PE=1 SV=2 - [KS6C1_HUMAN]	1.69	1	2	2	2	0.605	0.748	0.882	0.741	2.02	1.69	2	2	1066	118.6	4.87
Q9NZU1	Leucine-rich repeat transmembrane protein FLRT1 OS=Homo sapiens GN=FLRT1 PE=1 SV=3 - [FLRT1_HUMAN	1.86	1	1	1	1	0.728	0.947	0.909	0.741	2.64	1.86	1	1	646	71.3	6.38
Q9NRY5	Protein FAM114A2 OS=Homo sapiens GN=FAM114A2 PE=1 SV=4 - [F1142_HUMAN]	23.96	1	10	10	27	0.778	0.833	0.795	0.741	68.70	23.96	13	27	505	55.4	4.88
Q8ND07	Basal body-orientation factor 1 OS=Homo sapiens GN=CCDC176 PE=2 SV=3 - [BBOF1_HUMAN]	1.32	1	1	1	1	0.997	0.907	1.190	0.741	0.00	1.32	1	1	529	61.9	9.04
Q9H6S0	Probable ATP-dependent RNA helicase YTHDC2 OS=Homo sapiens GN=YTHDC2 PE=1 SV=2 - [YTDC2_HUMAN]	1.61	1	2	3	20	1.093	0.890	1.204	0.742	47.81	1.61	5	20	1430	160.1	8.40

P45984	Mitogen-activated protein kinase 9 OS=Homo sapiens GN=MAPK9 PE=1 SV=2 - [MK09_HUMAN]	25.24	3	5	9	19	0.938	0.756	0.593	0.742	41.60	25.24	15	19	424	48.1	5.63
P98172	Ephrin-B1 OS=Homo sapiens GN=EFNB1 PE=1 SV=1 - [EFNB1_HUMAN]	5.20	1	1	1	2	1.539	1.601	1.125	0.743	4.65	5.20	2	2	346	38.0	8.94
Q96KQ7	Histone-lysine N-methyltransferase EHMT2 OS=Homo sapiens GN=EHMT2 PE=1 SV=3 - [EHMT2_HUMAN]	3.72	1	2	2	3				0.743	5.64	3.72	2	3	1210	132.3	5.45
Q9UGJ1	Gamma-tubulin complex component 4 OS=Homo sapiens GN=TUBGCP4 PE=1 SV=1 - [GCP4_HUMAN]	3.75	1	2	2	9	0.762	0.952	1.021	0.743	34.64	3.75	3	9	667	76.0	6.65
Q5SW96	Low density lipoprotein receptor adapter protein 1 OS=Homo sapiens GN=LDLRAP1 PE=1 SV=3 - [ARH_HUMAN]	9.74	1	3	3	8	1.128	0.474	0.854	0.743	19.23	9.74	6	8	308	33.9	6.70
Q9P0R6	GSK3-beta interaction protein OS=Homo sapiens GN=GSKIP PE=1 SV=2 - [GSKIP_HUMAN]	7.19	1	1	1	2	0.699	1.011	1.025	0.743	4.83	7.19	1	2	139	15.6	4.48
Q9Y6N9	Harmonin OS=Homo sapiens GN=USH1C PE=1 SV=3 - [USH1C_HUMAN]	1.27	1	1	1	1	0.852	0.711	0.945	0.744	2.33	1.27	1	1	552	62.2	5.49
Q15125	3-beta-hydroxysteroid-Delta(8),Delta(7)-isomerase OS=Homo sapiens GN=EBP PE=1 SV=3 - [EBP_HUMAN]	5.22	1	1	1	5	0.598	0.986	0.983	0.744	7.23	5.22	2	5	230	26.3	7.90
O15162	Phospholipid scramblase 1 OS=Homo sapiens GN=PLSCR1 PE=1 SV=1 - [PLS1_HUMAN]	2.20	1	1	1	4	1.175	1.054	0.713	0.744	6.13	2.20	2	4	318	35.0	4.94

Q9H2Y7	Zinc finger protein 106 OS=Homo sapiens GN=ZNF106 PE=1 SV=1 - [ZN106_HUMAN]	1.49	1	2	3	15	0.608	1.015	1.100	0.744	25.67	1.49	3	15	1883	208.8	7.15
Q9NS86	LanC-like protein 2 OS=Homo sapiens GN=LANCL2 PE=1 SV=1 - [LANC2_HUMAN]	9.33	1	3	3	7	0.900	0.665	0.703	0.744	13.89	9.33	6	7	450	50.8	7.43
Q96HC4	PDZ and LIM domain protein 5 OS=Homo sapiens GN=PDLIM5 PE=1 SV=5 - [PDLI5_HUMAN]	41.78	1	22	22	193	1.111	1.504	1.306	0.744	438.38	41.78	40	193	596	63.9	8.21
Q5VTQ0	Tetratricopeptide repeat protein 39B OS=Homo sapiens GN=TTC39B PE=2 SV=4 - [TT39B_HUMAN	2.05	1	2	2	2	0.789	0.640	0.556	0.744	3.70	2.05	2	2	682	76.9	7.39
Q9BSY9	Desumoylating isopeptidase 2 OS=Homo sapiens GN=DESI2 PE=1 SV=1 - [DESI2_HUMAN]	4.64	1	1	1	4	0.581	0.626	0.894	0.744	11.62	4.64	2	4	194	21.4	4.92
P52306	Rap1 GTPase-GDP dissociation stimulator 1 OS=Homo sapiens GN=RAP1GDS1 PE=1 SV=3 - [GDS1_HUMAN]	41.19	2	19	21	80	0.785	1.011	1.237	0.745	213.46	41.19	34	80	607	66.3	5.31
Q8IY95	Transmembrane protein 192 OS=Homo sapiens GN=TMEM192 PE=1 SV=1 - [TM192_HUMAN]	12.18	1	3	3	10	0.611	0.650	0.801	0.745	19.86	12.18	5	10	271	30.9	7.99
Q8TEW8	Partitioning defective 3 homolog B OS=Homo sapiens GN=PARD3B PE=1 SV=2 - [PAR3L_HUMAN]	13.03	1	14	15	33	0.741	0.927	1.418	0.745	66.22	13.03	25	33	1205	132.4	8.37
P33261	Cytochrome P450 2C19 OS=Homo sapiens GN=CYP2C19 PE=1 SV=3 - [CP2CJ_HUMAN]	2.45	4	1	2	3	0.509	0.819	1.319	0.745	1.68	2.45	2	3	490	55.9	7.39

O43921	Ephrin-A2 OS=Homo sapiens GN=EFNA2 PE=1 SV=1 - [EFNA2_HUMAN]	6.57	1	1	1	1	1.150	0.720	0.927	0.745	0.00	6.57	1	1	213	23.9	7.40
Q96K49	Transmembrane protein 87B OS=Homo sapiens GN=TMEM87B PE=1 SV=1 - [TM87B_HUMAN]	8.65	1	4	4	11	1.213	0.668	1.010	0.745	24.31	8.65	7	11	555	63.5	7.44
P61960	Ubiquitin-fold modifier 1 OS=Homo sapiens GN=UFM1 PE=1 SV=1 - [UFM1_HUMAN]	25.88	1	2	2	8	0.486	0.732	0.784	0.745	23.70	25.88	4	8	85	9.1	9.31
P20339	Ras-related protein Rab-5A OS=Homo sapiens GN=RAB5A PE=1 SV=2 - [RAB5A_HUMAN]	38.60	1	4	6	22	0.813	0.851	0.837	0.745	61.64	38.60	10	22	215	23.6	8.15
O00400	Acetyl-coenzyme A transporter 1 OS=Homo sapiens GN=SLC33A1 PE=1 SV=1 - [ACATN_HUMAN]	12.93	1	5	5	15	0.783	0.853	0.962	0.746	31.99	12.93	8	15	549	60.9	7.33
Q9Y3P9	Rab GTPase-activating protein 1 OS=Homo sapiens GN=RABGAP1 PE=1 SV=3 - [RBGP1_HUMAN]	16.37	1	12	15	49	0.824	1.221	1.451	0.746	106.71	16.37	25	49	1069	121.7	5.25
Q96JX3	Protein SERAC1 OS=Homo sapiens GN=SERAC1 PE=1 SV=1 - [SRAC1_HUMAN]	1.53	1	1	1	2	1.526	0.815	0.736	0.746	0.00	1.53	1	2	654	74.1	7.68
Q9Y547	Intraflagellar transport protein 25 homolog OS=Homo sapiens GN=HSPB11 PE=1 SV=1 - [IFT25_HUMAN]	10.42	1	1	1	2	1.471	1.066	0.378	0.746	8.98	10.42	1	2	144	16.3	5.03
Q9H1R2	Dual specificity protein phosphatase 15 OS=Homo sapiens GN=DUSP15 PE=1 SV=4 - [DUS15_HUMAN]	4.07	1	1	1	1	1.745	1.392	1.059	0.746	0.00	4.07	1	1	295	31.9	8.38

O95197	Reticulon-3 OS=Homo sapiens GN=RTN3 PE=1 SV=2 - [RTN3_HUMAN]	1.84	1	2	3	11	0.758	0.766	0.716	0.746	14.68	1.84	5	11	1032	112.5	4.96
Q9BZE2	tRNA pseudouridine(38/39) synthase OS=Homo sapiens GN=PUS3 PE=1 SV=3 - [PUS3_HUMAN]	1.66	1	1	1	2	0.102	0.927	1.242	0.747	4.57	1.66	1	2	481	55.6	7.49
Q9BTX7	Alpha-tocopherol transfer protein-like OS=Homo sapiens GN=TTPAL PE=2 SV=2 - [TTPAL_HUMAN	11.99	1	3	4	5	0.620	1.323	2.346	0.747	11.95	11.99	4	5	342	38.5	6.44
Q9GZM8	Nuclear distribution protein nudE-like 1 OS=Homo sapiens GN=NDEL1 PE=1 SV=1 - [NDEL1_HUMAN]	7.54	1	2	3	6	0.789	0.760	1.028	0.747	9.07	7.54	5	6	345	38.4	5.24
Q9Y3L5	Ras-related protein Rap-2c OS=Homo sapiens GN=RAP2C PE=1 SV=1 - [RAP2C_HUMAN]	32.79	2	2	5	14	1.317	2.226	1.044	0.747	33.73	32.79	8	14	183	20.7	4.94
P15374	Ubiquitin carboxyl-terminal hydrolase isozyme L3 OS=Homo sapiens GN=UCHL3 PE=1 SV=1 - [UCHL3_HUMAN]	42.17	1	9	9	41	0.812	1.003	1.042	0.747	95.55	42.17	17	41	230	26.2	4.92
Q9P242	Neuronal tyrosine-phosphorylated phosphoinositide-3-kinase adapter 2 OS=Homo sapiens GN=NYAP2 PE=1 SV=3 - [NYAP2_HUMAN]	5.05	1	2	2	2	0.961	0.975	1.211	0.747	5.75	5.05	2	2	653	70.5	8.48
O75603	Chorion-specific transcription factor GCMb OS=Homo sapiens GN=GCM2 PE=1 SV=1 - [GCM2_HUMAN]	6.32	1	2	2	2	0.897	0.703	1.112	0.747	5.19	6.32	2	2	506	56.6	7.39

Q92558	Wiskott-Aldrich syndrome protein family member 1 OS=Homo sapiens GN=WASF1 PE=1 SV=1 - [WASF1_HUMAN]	6.98	1	4	5	5	0.804	1.211	1.031	0.747	12.34	6.98	5	5	559	61.6	6.46
Q14019	Coactosin-like protein OS=Homo sapiens GN=COTL1 PE=1 SV=3 - [COTL1_HUMAN]	73.94	1	14	14	128	0.975	0.757	0.967	0.748	283.70	73.94	22	128	142	15.9	5.67
P04075	Fructose-bisphosphate aldolase A OS=Homo sapiens GN=ALDOA PE=1 SV=2 - [ALDOA_HUMAN]	79.95	2	28	37	1871	1.036	0.909	1.012	0.748	4998.81	79.95	64	1871	364	39.4	8.09
Q99619	SPRY domain-containing SOCS box protein 2 OS=Homo sapiens GN=SPSB2 PE=1 SV=1 - [SPSB2_HUMAN]	3.04	1	1	1	1	0.539	0.509	0.726	0.748	2.70	3.04	1	1	263	28.6	8.18
P00751	Complement factor B OS=Homo sapiens GN=CFB PE=1 SV=2 - [CFAB_HUMAN]	2.49	1	2	2	6	0.562	1.621	1.641	0.748	8.93	2.49	4	6	764	85.5	7.06
P0CG34	Thymosin beta-15A OS=Homo sapiens GN=TMSB15A PE=1 SV=1 - [TB15A_HUMAN]	28.89	1	2	2	130	1.868	2.115	1.466	0.749	307.92	28.89	3	130	45	5.2	5.36
Q9NUM4	Transmembrane protein 106B OS=Homo sapiens GN=TMEM106B PE=1 SV=2 - [T106B_HUMAN]	11.68	1	3	3	5	0.924	0.773	0.565	0.749	7.66	11.68	4	5	274	31.1	6.99
Q9H2J4	Phosducin-like protein 3 OS=Homo sapiens GN=PDCL3 PE=1 SV=1 - [PDCL3_HUMAN]	26.36	1	5	5	9	0.761	0.697	1.014	0.749	16.66	26.36	7	9	239	27.6	4.84
P63098	Calcineurin subunit B type 1 OS=Homo sapiens GN=PPP3R1 PE=1 SV=2 - [CANB1_HUMAN]	72.94	1	9	9	35	0.954	1.119	1.108	0.749	68.06	72.94	14	35	170	19.3	4.81

Q9UQ52	Contactin-6 OS=Homo sapiens GN=CNTN6 PE=1 SV=1 - [CNTN6_HUMAN]	2.14	1	2	2	4	1.003	0.962	1.223	0.749	8.86	2.14	2	4	1028	113.9	6.00
Q05639	Elongation factor 1-alpha 2 OS=Homo sapiens GN=EEF1A2 PE=1 SV=1 - [EF1A2_HUMAN]	37.37	1	2	17	355	1.510	0.901	1.288	0.749	857.90	37.37	27	355	463	50.4	9.03
Q9P289	Serine/threonine-protein kinase 26 OS=Homo sapiens GN=STK26 PE=1 SV=2 - [STK26_HUMAN	23.32	1	3	10	33	0.628	1.382	1.766	0.749	76.21	23.32	18	33	416	46.5	5.29
P50542	Peroxisomal targeting signal 1 receptor OS=Homo sapiens GN=PEX5 PE=1 SV=3 - [PEX5_HUMAN]	12.68	1	6	6	23	1.060	1.028	0.898	0.749	45.84	12.68	8	23	639	70.8	4.54
Q9NX45	Spermatogenesis- and oogenesis-specific basic helix-loop-helix-containing protein 2 OS=Homo sapiens GN=SOHLH2 PE=2 SV=2 - [SOLH2_HUMAN]	4.71	1	1	1	1	1.052	0.884	1.122	0.749	0.00	4.71	1	1	425	46.9	7.17
Q6ZSY5	Protein phosphatase 1 regulatory subunit 3F OS=Homo sapiens GN=PPP1R3F PE=1 SV=3 - [PPR3F_HUMAN]	1.38	1	1	1	1	0.996	1.202	1.042	0.750	1.99	1.38	1	1	799	82.7	4.60
Q92882	Osteoclast-stimulating factor 1 OS=Homo sapiens GN=OSTF1 PE=1 SV=2 - [OSTF1_HUMAN]	21.03	1	3	3	10	1.009	0.796	1.097	0.750	19.79	21.03	5	10	214	23.8	5.68
P51606	N-acylglucosamine 2-epimerase OS=Homo sapiens GN=RENBP PE=1 SV=2 - [RENBP_HUMAN]	1.41	1	1	1	2	0.657	1.186	1.456	0.750	2.19	1.41	1	2	427	48.8	6.37

Q03113	Guanine nucleotide-binding protein subunit alpha-12 OS=Homo sapiens GN=GNA12 PE=1 SV=4 - [GNA12_HUMAN]	13.39	1	3	5	22	1.085	1.331	0.922	0.750	66.06	13.39	10	22	381	44.3	9.83
P31431	Syndecan-4 OS=Homo sapiens GN=SDC4 PE=1 SV=2 - [SDC4_HUMAN]	20.20	1	3	3	10	0.855	1.007	1.020	0.750	28.92	20.20	6	10	198	21.6	4.50
Q8WVD5	RING finger protein 141 OS=Homo sapiens GN=RNF141 PE=1 SV=1 - [RN141_HUMAN]	4.35	1	1	1	1	0.606	0.900	1.284	0.750	1.84	4.35	1	1	230	25.5	5.20
O15063	Uncharacterized protein KIAA0355 OS=Homo sapiens GN=KIAA0355 PE=1 SV=2 - [K0355_HUMAN]	1.03	1	1	1	1	0.651	0.931	1.228	0.750	2.40	1.03	1	1	1070	115.9	7.15
P19634	Sodium/hydrogen exchanger 1 OS=Homo sapiens GN=SLC9A1 PE=1 SV=2 - [SL9A1_HUMAN]	13.99	1	7	7	28	0.726	0.721	0.963	0.750	65.63	13.99	9	28	815	90.7	7.21
Q9UN79	Transcription factor SOX-13 OS=Homo sapiens GN=SOX13 PE=1 SV=3 - [SOX13_HUMAN]	3.22	1	1	2	2	0.798	1.120	1.021	0.751	4.77	3.22	2	2	622	69.2	6.71
O43791	Speckle-type POZ protein OS=Homo sapiens GN=SPOP PE=1 SV=1 - [SPOP_HUMAN]	4.01	2	1	1	2	1.044	0.911	1.102	0.751	4.59	4.01	1	2	374	42.1	5.80
Q9BXB5	Oxysterol-binding protein-related protein 10 OS=Homo sapiens GN=OSBPL10 PE=1 SV=2 - [OSB10_HUMAN]	6.81	1	3	4	7	1.370	0.890	1.273	0.751	13.57	6.81	5	7	764	83.9	8.31
O00418	Eukaryotic elongation factor 2 kinase OS=Homo sapiens GN=EEF2K PE=1 SV=2 - [EF2K_HUMAN]	4.28	1	3	3	7	0.543	0.986	1.099	0.751	16.40	4.28	4	7	725	82.1	5.33

P05787	Keratin, type II cytoskeletal 8 OS=Homo sapiens GN=KRT8 PE=1 SV=7 - [K2C8_HUMAN]	38.92	2	16	23	169	0.874	7.391	1.230	0.751	333.33	38.92	36	169	483	53.7	5.59
P61020	Ras-related protein Rab-5B OS=Homo sapiens GN=RAB5B PE=1 SV=1 - [RAB5B_HUMAN]	31.16	1	3	5	18	0.783	1.134	1.093	0.751	43.43	31.16	9	18	215	23.7	8.13
P26006	Integrin alpha-3 OS=Homo sapiens GN=ITGA3 PE=1 SV=5 - [ITA3_HUMAN]	8.28	1	7	7	24	1.627	0.871	1.210	0.752	77.80	8.28	13	24	1051	116.5	6.77
P20810	Calpastatin OS=Homo sapiens GN=CAST PE=1 SV=4 - [ICAL_HUMAN]	45.62	1	31	31	186	0.868	1.277	1.134	0.752	433.94	45.62	49	186	708	76.5	5.07
O15050	TPR and ankyrin repeat-containing protein 1 OS=Homo sapiens GN=TRANK1 PE=2 SV=4 - [TRNK1_HUMAN]	0.72	1	2	2	2	0.779	0.760	0.689	0.752	2.17	0.72	2	2	2925	336.0	6.76
O15440	Multidrug resistance-associated protein 5 OS=Homo sapiens GN=ABCC5 PE=1 SV=2 - [MRP5_HUMAN]	0.84	1	1	1	2	0.534	0.517	0.659	0.752	0.00	0.84	1	2	1437	160.6	8.66
Q5JPB2	Zinc finger protein 831 OS=Homo sapiens GN=ZNF831 PE=2 SV=4 - [ZN831_HUMAN]	1.01	1	2	2	3	0.771	1.524	1.371	0.752	8.76	1.01	2	3	1677	177.8	8.34
Q9NPA8	Transcription and mRNA export factor ENY2 OS=Homo sapiens GN=ENY2 PE=1 SV=1 - [ENY2_HUMAN]	32.67	1	3	3	9	0.766	0.732	0.772	0.752	19.73	32.67	4	9	101	11.5	9.33
Q8IYB1	Protein MB21D2 OS=Homo sapiens GN=MB21D2 PE=1 SV=3 - [M21D2_HUMAN]	1.63	1	1	1	1	0.675	0.594	0.844	0.752	2.46	1.63	1	1	491	55.8	7.03

Q9ULI3	Protein HEG homolog 1 OS=Homo sapiens GN=HEG1 PE=1 SV=3 - [HEG1_HUMAN]	8.18	1	10	10	20	1.256	0.735	1.273	0.752	61.67	8.18	13	20	1381	147.4	6.18
P41091	Eukaryotic translation initiation factor 2 subunit 3 OS=Homo sapiens GN=EIF2S3 PE=1 SV=3 - [IF2G_HUMAN]	38.56	2	15	15	70	0.803	0.743	0.803	0.752	184.90	38.56	25	70	472	51.1	8.40
P41236	Protein phosphatase inhibitor 2 OS=Homo sapiens GN=PPP1R2 PE=1 SV=2 - [IPP2_HUMAN]	33.66	2	8	8	36	0.727	1.050	0.983	0.752	79.91	33.66	12	36	205	23.0	4.74
Q6AI08	HEAT repeat-containing protein 6 OS=Homo sapiens GN=HEATR6 PE=1 SV=1 - [HEAT6_HUMAN]	2.29	1	2	2	4	0.661	0.915	1.090	0.752	10.48	2.29	4	4	1181	128.7	7.03
Q9HCM9	E3 ubiquitin-protein ligase TRIM39 OS=Homo sapiens GN=TRIM39 PE=1 SV=2 - [TRI39_HUMAN]	2.51	1	1	1	1	1.123	1.307	1.202	0.753	0.00	2.51	1	1	518	59.7	7.68
Q96AB6	Protein N-terminal asparagine amidohydrolase OS=Homo sapiens GN=NTAN1 PE=1 SV=3 - [NTAN1_HUMAN]	8.71	1	2	2	3	1.363	0.828	1.353	0.753	7.54	8.71	3	3	310	34.7	6.27
P28074	Proteasome subunit beta type-5 OS=Homo sapiens GN=PSMB5 PE=1 SV=3 - [PSB5_HUMAN]	42.59	1	10	10	48	0.677	0.767	0.873	0.753	133.39	42.59	17	48	263	28.5	6.92
Q13029	PR domain zinc finger protein 2 OS=Homo sapiens GN=PRDM2 PE=1 SV=3 - [PRDM2_HUMAN]	1.69	1	2	3	12	0.847	0.897	1.037	0.753	23.80	1.69	3	12	1718	188.8	7.31
Q9NXR7	BRCA1-A complex subunit BRE OS=Homo sapiens GN=BRE PE=1 SV=2 - [BRE_HUMAN]	5.48	1	2	2	6	1.009	0.597	1.200	0.753	13.19	5.48	4	6	383	43.5	5.81

O94921	Cyclin-dependent kinase 14 OS=Homo sapiens GN=CDK14 PE=1 SV=3 - [CDK14_HUMAN]	5.54	6	1	2	12	1.059	0.379	0.579	0.753	23.06	5.54	3	12	469	53.0	8.92
A2RU48	Single-pass membrane and coiled-coil domain-containing protein 3 OS=Homo sapiens GN=SMCO3 PE=2 SV=1 - [SMCO3_HUMAN]	2.67	1	1	1	1	0.672	0.986	0.660	0.754	2.54	2.67	1	1	225	24.9	8.22
P35318	ADM OS=Homo sapiens GN=ADM PE=1 SV=1 - [ADML_HUMAN]	4.32	1	1	1	1	0.807	0.504	0.802	0.755	2.44	4.32	1	1	185	20.4	10.84
Q99436	Proteasome subunit beta type-7 OS=Homo sapiens GN=PSMB7 PE=1 SV=1 - [PSB7_HUMAN]	14.44	1	4	4	42	0.791	0.765	0.810	0.755	112.76	14.44	8	42	277	29.9	7.68
Q13530	Serine incorporator 3 OS=Homo sapiens GN=SERINC3 PE=2 SV=2 - [SERC3_HUMAN]	3.81	1	2	2	3	0.910	0.517	0.647	0.756	2.11	3.81	3	3	473	52.5	7.46
O60711	Leupaxin OS=Homo sapiens GN=LPXN PE=1 SV=1 - [LPXN_HUMAN]	2.85	1	1	1	3	0.814	1.301	1.567	0.756	8.94	2.85	2	3	386	43.3	6.01
P62166	Neuronal calcium sensor 1 OS=Homo sapiens GN=NCS1 PE=1 SV=2 - [NCS1_HUMAN]	41.58	1	8	8	25	0.752	1.367	1.096	0.756	44.62	41.58	12	25	190	21.9	4.83
O00115	Deoxyribonuclease-2-alpha OS=Homo sapiens GN=DNASE2 PE=1 SV=2 - [DNS2A_HUMAN]	13.06	1	5	5	23	0.649	1.146	0.932	0.757	46.85	13.06	8	23	360	39.6	8.05
Q8IV08	Phospholipase D3 OS=Homo sapiens GN=PLD3 PE=1 SV=1 - [PLD3_HUMAN]	18.98	2	9	9	57	0.676	1.181	0.927	0.757	109.43	18.98	16	57	490	54.7	6.47

Q04917	14-3-3 protein eta OS=Homo sapiens GN=YWHAH PE=1 SV=4 - [1433F_HUMAN]	67.07	2	14	19	200	1.101	1.177	0.909	0.757	511.35	67.07	30	200	246	28.2	4.84
Q9H1A4	Anaphase-promoting complex subunit 1 OS=Homo sapiens GN=ANAPC1 PE=1 SV=1 - [APC1_HUMAN]	1.85	1	3	3	4	0.889	1.097	0.915	0.757	8.36	1.85	4	4	1944	216.4	6.30
Q9H792	Pseudopodium-enriched atypical kinase 1 OS=Homo sapiens GN=PEAK1 PE=1 SV=4 - [PEAK1_HUMAN]	20.68	1	31	31	84	0.797	0.680	1.104	0.757	230.38	20.68	46	84	1746	193.0	6.89
P08758	Annexin A5 OS=Homo sapiens GN=ANXA5 PE=1 SV=2 - [ANXA5_HUMAN]	85.94	1	31	32	975	0.698	0.675	0.671	0.757	2578.75	85.94	50	975	320	35.9	5.05
A2VDJ0	Transmembrane protein 131-like OS=Homo sapiens GN=KIAA0922 PE=1 SV=2 - [T131L_HUMAN]	1.80	1	2	2	2	1.174	0.986	0.963	0.757	4.56	1.80	2	2	1609	179.2	6.86
Q63HQ0	AP-1 complex-associated regulatory protein OS=Homo sapiens GN=AP1AR PE=1 SV=1 - [AP1AR_HUMAN]	5.63	8	1	2	6	0.948	0.606	0.784	0.757	16.82	5.63	2	6	302	34.3	4.87
Q6ZR37	Pleckstrin homology domain-containing family G member 7 OS=Homo sapiens GN=PLEKHG7 PE=2 SV=1 - [PKHG7_HUMAN]	3.96	1	1	1	1	1.121	0.613	0.712	0.757	0.00	3.96	1	1	379	44.3	8.27
Q14005	Pro-interleukin-16 OS=Homo sapiens GN=IL16 PE=1 SV=4 - [IL16_HUMAN]	5.56	1	4	4	10	0.931	0.953	0.802	0.758	21.93	5.56	5	10	1332	141.7	8.06
Q9UBZ9	DNA repair protein REV1 OS=Homo sapiens GN=REV1 PE=1 SV=1 - [REV1_HUMAN]	1.20	1	2	2	2	0.745	0.729	0.652	0.758	2.10	1.20	2	2	1251	138.2	8.51

Q8WVK2	U4/U6.U5 small nuclear ribonucleoprotein 27 kDa protein OS=Homo sapiens GN=SNRNP27 PE=1 SV=1 - [SNR27_HUMAN]	7.10	1	1	1	2	0.914	1.064	1.074	0.758	4.64	7.10	1	2	155	18.8	11.62
P52756	RNA-binding protein 5 OS=Homo sapiens GN=RBM5 PE=1 SV=2 - [RBM5_HUMAN]	8.96	1	3	6	10	1.679	1.184	1.373	0.759	15.96	8.96	8	10	815	92.1	6.28
O95866	Protein G6b OS=Homo sapiens GN=G6B PE=1 SV=1 - [G6B_HUMAN]	7.47	1	1	1	1	0.778	1.431	1.363	0.759	2.81	7.47	1	1	241	26.1	9.61
Q9UEE5	Serine/threonine-protein kinase 17A OS=Homo sapiens GN=STK17A PE=1 SV=2 - [ST17A_HUMAN]	1.93	1	1	1	4	0.851	0.825	0.866	0.759	8.76	1.93	2	4	414	46.5	5.15
O15254	Peroxisomal acyl-coenzyme A oxidase 3 OS=Homo sapiens GN=ACOX3 PE=1 SV=2 - [ACOX3_HUMAN]	16.86	1	9	10	18	0.822	1.189	1.405	0.759	41.31	16.86	13	18	700	77.6	7.25
Q8WVB6	Chromosome transmission fidelity protein 18 homolog OS=Homo sapiens GN=CHTF18 PE=1 SV=1 - [CTF18_HUMAN]	6.05	1	3	4	11	1.002	0.617	1.213	0.759	11.74	6.05	5	11	975	107.3	7.21
O43414	ERI1 exoribonuclease 3 OS=Homo sapiens GN=ERI3 PE=1 SV=2 - [ERI3_HUMAN]	9.79	1	3	3	18	1.005	0.670	0.745	0.759	36.94	9.79	6	18	337	37.2	8.07
P48058	Glutamate receptor 4 OS=Homo sapiens GN=GRIA4 PE=2 SV=2 - [GRIA4_HUMAN]	0.67	1	1	1	1	1.734	1.783	3.858	0.759	2.30	0.67	1	1	902	100.8	8.10
Q00341	Vigilin OS=Homo sapiens GN=HDLBP PE=1 SV=2 - [VIGLN_HUMAN]	52.76	2	67	70	535	0.730	0.884	0.899	0.759	1387.18	52.76	112	535	1268	141.4	6.87

P16989	Y-box-binding protein 3 OS=Homo sapiens GN=YBX3 PE=1 SV=4 - [YBOX3_HUMAN]	29.57	2	5	11	45	0.573	0.892	0.656	0.759	127.90	29.57	16	45	372	40.1	9.77
P46939	Utrophin OS=Homo sapiens GN=UTRN PE=1 SV=2 - [UTRO_HUMAN]	34.72	4	110	114	443	1.220	0.932	0.984	0.759	1121.16	34.72	183	443	3433	394.2	5.33
Q13753	Laminin subunit gamma-2 OS=Homo sapiens GN=LAMC2 PE=1 SV=2 - [LAMC2_HUMAN]	1.68	1	1	1	2	1.344	0.330	1.508	0.760	0.00	1.68	1	2	1193	130.9	6.19
Q9ULC3	Ras-related protein Rab-23 OS=Homo sapiens GN=RAB23 PE=1 SV=1 - [RAB23_HUMAN]	44.73	1	11	11	29	0.879	0.883	0.866	0.760	72.85	44.73	17	29	237	26.6	6.60
O00189	AP-4 complex subunit mu-1 OS=Homo sapiens GN=AP4M1 PE=1 SV=2 - [AP4M1_HUMAN]	3.75	1	2	2	4	0.958	1.028	0.915	0.760	6.41	3.75	4	4	453	49.9	7.20
Q86WN1	FCH and double SH3 domains protein 1 OS=Homo sapiens GN=FCHSD1 PE=2 SV=1 - [FCSD1_HUMAN	10.58	1	6	6	16	0.715	1.087	1.550	0.760	43.82	10.58	11	16	690	76.9	5.34
Q9BSW7	Synaptotagmin-17 OS=Homo sapiens GN=SYT17 PE=1 SV=1 - [SYT17_HUMAN]	1.48	1	1	1	1	0.719	0.884	0.598	0.760	2.53	1.48	1	1	474	53.8	7.50
O95772	MLN64 N-terminal domain homolog OS=Homo sapiens GN=STARD3NL PE=1 SV=1 - [MENTO_HUMAN]	8.97	1	2	2	14	0.470	0.830	0.734	0.760	37.16	8.97	3	14	234	26.6	4.81
Q6ZVM7	TOM1-like protein 2 OS=Homo sapiens GN=TOM1L2 PE=1 SV=1 - [TM1L2_HUMAN]	11.83	1	5	5	15	0.690	0.873	0.843	0.760	31.15	11.83	8	15	507	55.5	4.79

A6NNS2	Dehydrogenase /reductase SDR family member 7C OS=Homo sapiens GN=DHRS7C PE=2 SV=3 - [DRS7C_HUMAN]	6.73	1	2	2	4	1.194	1.332	1.090	0.760	10.28	6.73	2	4	312	34.9	8.47
P11717	Cation-independent mannose-6-phosphate receptor OS=Homo sapiens GN=IGF2R PE=1 SV=3 - [MPRI_HUMAN]	18.55	1	41	42	106	0.784	0.624	0.791	0.760	262.69	18.55	63	106	2491	274.2	5.94
O43324	Eukaryotic translation elongation factor 1 epsilon-1 OS=Homo sapiens GN=EEF1E1 PE=1 SV=1 - [MCA3_HUMAN]	22.41	1	5	5	12	0.745	0.750	0.918	0.761	20.81	22.41	7	12	174	19.8	8.54
P08247	Synaptophysin OS=Homo sapiens GN=SYP PE=1 SV=3 - [SYPH_HUMAN]	3.19	1	1	1	2	1.697	1.431	1.475	0.761	4.83	3.19	1	2	313	33.8	4.81
Q9BX95	Sphingosine-1-phosphate phosphatase 1 OS=Homo sapiens GN=SGPP1 PE=1 SV=2 - [SGPP1_HUMAN]	5.67	1	2	2	2	0.379	0.880	0.921	0.761	3.44	5.67	2	2	441	49.1	8.82
P15090	Fatty acid-binding protein, adipocyte OS=Homo sapiens GN=FABP4 PE=1 SV=3 - [FABP4_HUMAN	15.91	1	1	2	4	0.768	1.016	8.560	0.761	6.36	15.91	4	4	132	14.7	7.14
P61619	Protein transport protein Sec61 subunit alpha isoform 1 OS=Homo sapiens GN=SEC61A1 PE=1 SV=2 - [S61A1_HUMAN]	12.18	2	7	7	48	0.648	0.683	0.730	0.761	102.14	12.18	13	48	476	52.2	8.06
Q13492	Phosphatidylinositol-binding clathrin assembly protein OS=Homo sapiens GN=PICALM PE=1 SV=2 - [PICAL_HUMAN]	31.44	1	17	18	96	0.886	0.749	0.822	0.762	214.48	31.44	28	96	652	70.7	7.90

Q5ST30	Valine--tRNA ligase, mitochondrial OS=Homo sapiens GN=VARS2 PE=1 SV=2 - [SYVM_HUMAN]	5.27	1	4	4	4	0.582	1.752	0.860	0.762	10.84	5.27	4	4	1063	118.4	7.02
O95671	N-acetylserotonin O-methyltransferase-like protein OS=Homo sapiens GN=ASMTL PE=1 SV=3 - [ASML_HUMAN]	23.83	1	12	12	37	0.652	0.605	0.827	0.762	101.86	23.83	19	37	621	68.8	6.07
A9UHW6	MIF4G domain-containing protein OS=Homo sapiens GN=MIF4GD PE=1 SV=1 - [MI4GD_HUMAN]	12.61	1	2	3	5	0.873	1.022	1.240	0.762	10.40	12.61	4	5	222	25.4	5.33
Q9NV70	Exocyst complex component 1 OS=Homo sapiens GN=EXOC1 PE=1 SV=4 - [EXOC1_HUMAN]	31.10	1	22	22	56	1.019	0.838	0.963	0.762	155.13	31.10	33	56	894	101.9	6.61
Q9BQA1	Methylosome protein 50 OS=Homo sapiens GN=WDR77 PE=1 SV=1 - [MEP50_HUMAN]	17.25	1	5	5	7	0.729	1.147	1.255	0.762	13.54	17.25	7	7	342	36.7	5.17
Q6FIF0	AN1-type zinc finger protein 6 OS=Homo sapiens GN=ZFAND6 PE=1 SV=2 - [ZFAN6_HUMAN]	4.33	1	1	1	2	0.496	1.018	0.891	0.762	4.40	4.33	2	2	208	22.5	7.20
Q9UBM7	7-dehydrocholesterol reductase OS=Homo sapiens GN=DHCR7 PE=1 SV=1 - [DHCR7_HUMAN]	6.11	1	3	3	11	0.990	0.574	1.041	0.762	23.86	6.11	6	11	475	54.5	8.70
P11230	Acetylcholine receptor subunit beta OS=Homo sapiens GN=CHRNB1 PE=1 SV=3 - [ACHB_HUMAN]	1.20	1	1	1	2	0.682	0.665	0.378	0.763	4.82	1.20	1	2	501	56.7	5.81
Q7L576	Cytoplasmic FMR1-interacting protein 1 OS=Homo sapiens GN=CYFIP1 PE=1 SV=1 - [CYFP1_HUMAN]	24.42	1	14	25	65	0.982	0.868	0.922	0.763	158.29	24.42	39	65	1253	145.1	6.90

P24844	Myosin regulatory light polypeptide 9 OS=Homo sapiens GN=MYL9 PE=1 SV=4 - [MYL9_HUMAN]	83.72	1	6	14	405	0.766	1.051	1.237	0.763	1016.80	83.72	23	405	172	19.8	4.92
O95685	Protein phosphatase 1 regulatory subunit 3D OS=Homo sapiens GN=PPP1R3D PE=1 SV=1 - [PPR3D_HUMAN]	4.68	1	1	1	2	0.801	0.959	0.966	0.763	3.53	4.68	2	2	299	32.5	8.07
P18089	Alpha-2B adrenergic receptor OS=Homo sapiens GN=ADRA2B PE=1 SV=3 - [ADA2B_HUMAN]	3.13	1	1	1	21	1.047	1.031	0.909	0.763	55.56	3.13	1	21	447	49.5	8.73
P51157	Ras-related protein Rab-28 OS=Homo sapiens GN=RAB28 PE=1 SV=2 - [RAB28_HUMAN]	9.95	1	2	2	4	0.959	1.761	1.365	0.764	0.00	9.95	3	4	221	24.8	5.97
P49023	Paxillin OS=Homo sapiens GN=PXN PE=1 SV=3 - [PAXI_HUMAN]	34.52	1	13	13	54	0.783	1.071	1.021	0.764	149.20	34.52	22	54	591	64.5	6.19
O60784	Target of Myb protein 1 OS=Homo sapiens GN=TOM1 PE=1 SV=2 - [TOM1_HUMAN]	26.42	1	11	11	53	0.965	0.568	0.556	0.764	125.96	26.42	20	53	492	53.8	4.70
O00222	Metabotropic glutamate receptor 8 OS=Homo sapiens GN=GRM8 PE=2 SV=2 - [GRM8_HUMAN]	1.10	1	1	1	1	0.534	0.252	0.374	0.764	2.20	1.10	1	1	908	101.7	8.15
Q99961	Endophilin-A2 OS=Homo sapiens GN=SH3GL1 PE=1 SV=1 - [SH3G1_HUMAN]	36.14	2	15	15	56	0.819	0.913	1.035	0.764	127.66	36.14	24	56	368	41.5	5.43
A5PLN9	Trafficking protein particle complex subunit 13 OS=Homo sapiens GN=TRAPPC13 PE=1 SV=2 - [TPC13_HUMAN]	10.55	1	4	4	9	0.853	0.938	1.029	0.765	13.36	10.55	7	9	417	46.5	5.60

Q5HYK7	SH3 domain-containing protein 19 OS=Homo sapiens GN=SH3D19 PE=1 SV=2 - [SH319_HUMAN]	17.59	1	12	12	42	0.553	1.130	0.657	0.765	100.87	17.59	20	42	790	86.5	8.32
Q96AA8	Janus kinase and microtubule-interacting protein 2 OS=Homo sapiens GN=JAKMIP2 PE=2 SV=1 - [JKIP2_HUMAN]	1.11	1	1	1	1	0.697	0.410	0.616	0.765	2.11	1.11	1	1	810	94.9	6.16
P30039	Phenazine biosynthesis-like domain-containing protein OS=Homo sapiens GN=PBLD PE=1 SV=2 - [PBLD_HUMAN]	19.44	1	5	5	8	0.909	0.930	0.970	0.765	22.38	19.44	7	8	288	31.8	6.52
Q9NVD7	Alpha-parvin OS=Homo sapiens GN=PARVA PE=1 SV=1 - [PARVA_HUMAN]	34.41	1	12	15	49	0.777	0.972	1.035	0.765	137.08	34.41	20	49	372	42.2	5.95
Q9UHY1	Nuclear receptor-binding protein OS=Homo sapiens GN=NRBP1 PE=1 SV=1 - [NRBP_HUMAN]	15.70	1	7	7	13	0.672	0.662	0.822	0.765	33.97	15.70	10	13	535	59.8	5.08
Q8IWA5	Choline transporter-like protein 2 OS=Homo sapiens GN=SLC44A2 PE=1 SV=3 - [CTL2_HUMAN]	13.60	1	8	8	23	1.564	0.619	0.953	0.765	43.36	13.60	12	23	706	80.1	8.57
P10746	Uroporphyrinogen-III synthase OS=Homo sapiens GN=UROS PE=1 SV=1 - [HEM4_HUMAN]	16.98	1	3	3	8	0.895	1.037	1.194	0.765	22.20	16.98	5	8	265	28.6	5.48
Q16401	26S proteasome non-ATPase regulatory subunit 5 OS=Homo sapiens GN=PSMD5 PE=1 SV=3 - [PSMD5_HUMA	40.48	1	14	14	56	0.929	0.701	0.707	0.766	169.04	40.48	22	56	504	56.2	5.48
P15144	Aminopeptidase N OS=Homo sapiens GN=ANPEP PE=1 SV=4 - [AMPN_HUMAN]	27.92	1	30	30	136	0.476	1.349	1.069	0.766	310.03	27.92	49	136	967	109.5	5.48

Q9HCE6	Rho guanine nucleotide exchange factor 10-like protein OS=Homo sapiens GN=ARHGEF10L PE=1 SV=3 - [ARGAL_HUMAN]	1.56	1	2	2	3	0.914	1.240	1.204	0.766	10.73	1.56	2	3	1279	140.3	5.88
O96020	G1/S-specific cyclin-E2 OS=Homo sapiens GN=CCNE2 PE=1 SV=1 - [CCNE2_HUMAN]	8.17	1	2	2	4	0.823	1.039	0.915	0.766	5.30	8.17	2	4	404	46.7	7.83
Q9NQC3	Reticulon-4 OS=Homo sapiens GN=RTN4 PE=1 SV=2 - [RTN4_HUMAN]	9.65	1	9	9	141	1.012	0.778	0.877	0.766	431.13	9.65	14	141	1192	129.9	4.50
P54289	Voltage-dependent calcium channel subunit alpha-2/delta-1 OS=Homo sapiens GN=CACNA2D1 PE=1 SV=3 - [CA2D1_HUMAN]	21.85	1	23	23	68	1.525	0.705	0.852	0.766	155.67	21.85	37	68	1103	124.5	5.27
Q13619	Cullin-4A OS=Homo sapiens GN=CUL4A PE=1 SV=3 - [CUL4A_HUMAN]	20.55	1	8	19	45	0.831	0.772	0.809	0.766	86.87	20.55	29	45	759	87.6	8.13
Q9HCM4	Band 4.1-like protein 5 OS=Homo sapiens GN=EPB41L5 PE=1 SV=3 - [E41L5_HUMAN]	1.64	1	1	1	4	0.635	1.440	1.606	0.767	5.90	1.64	2	4	733	81.8	6.58
P49841	Glycogen synthase kinase-3 beta OS=Homo sapiens GN=GSK3B PE=1 SV=2 - [GSK3B_HUMAN]	24.76	1	4	8	33	0.884	0.739	0.986	0.767	65.26	24.76	13	33	420	46.7	8.78
P54274	Telomeric repeat-binding factor 1 OS=Homo sapiens GN=TERF1 PE=1 SV=3 - [TERF1_HUMAN	8.43	2	2	3	3	1.015	0.945	1.067	0.767	6.81	8.43	3	3	439	50.2	6.37
P43630	Killer cell immunoglobulin-like receptor 3DL2 OS=Homo sapiens GN=KIR3DL2 PE=2 SV=1 - [KI3L2_HUMAN]	3.74	1	1	1	1	0.600	1.145	1.616	0.767	2.70	3.74	1	1	455	50.2	8.46

Q96EM0	Trans-L-3-hydroxyproline dehydratase OS=Homo sapiens GN=L3HYPDH PE=1 SV=2 - [T3HPD_HUMAN]	29.66	1	7	7	16	0.977	0.469	0.756	0.768	46.36	29.66	9	16	354	38.1	6.68
P15954	Cytochrome c oxidase subunit 7C, mitochondrial OS=Homo sapiens GN=COX7C PE=1 SV=1 - [COX7C_HUMAN]	23.81	2	1	2	4	0.637	0.569	0.551	0.768	8.81	23.81	3	4	63	7.2	10.27
Q96MW7	Tigger transposable element-derived protein 1 OS=Homo sapiens GN=TIGD1 PE=1 SV=1 - [TIGD1_HUMAN]	3.38	1	2	2	2	0.741	0.949	1.139	0.768	5.78	3.38	2	2	591	67.3	8.46
O43252	Bifunctional 3'-phosphoadenosine 5'-phosphosulfate synthase 1 OS=Homo sapiens GN=PAPSS1 PE=1 SV=2 - [PAPS1_HUMAN]	36.06	1	16	18	75	0.817	0.973	1.116	0.768	163.61	36.06	27	75	624	70.8	6.86
Q9UNA0	A disintegrin and metalloproteinase with thrombospondin motifs 5 OS=Homo sapiens GN=ADAMTS5 PE=1 SV=2 - [ATS5_HUMAN]	1.29	1	1	1	1	0.479	0.943	1.079	0.768	0.00	1.29	1	1	930	101.7	8.85
P61586	Transforming protein RhoA OS=Homo sapiens GN=RHOA PE=1 SV=1 - [RHOA_HUMAN]	60.10	2	10	11	82	1.214	1.091	1.047	0.768	196.43	60.10	19	82	193	21.8	6.10
P04792	Heat shock protein beta-1 OS=Homo sapiens GN=HSPB1 PE=1 SV=2 - [HSPB1_HUMAN]	65.85	1	15	15	496	0.745	0.995	1.307	0.768	1267.86	65.85	26	496	205	22.8	6.40
P00558	Phosphoglycerate kinase 1 OS=Homo sapiens GN=PGK1 PE=1 SV=3 - [PGK1_HUMAN]	87.05	1	40	40	788	1.193	1.123	0.944	0.768	1894.68	87.05	71	788	417	44.6	8.10

O75044	SLIT-ROBO Rho GTPase-activating protein 2 OS=Homo sapiens GN=SRGAP2 PE=1 SV=2 - [SRGP2_HUMAN]	11.48	2	5	11	23	0.944	0.787	0.778	0.769	58.49	11.48	15	23	1071	120.8	6.70
Q587J7	Putative ATP-dependent RNA helicase TDRD12 OS=Homo sapiens GN=TDRD12 PE=2 SV=2 - [TDR12_HUMAN]	2.29	1	2	2	6	0.423	0.992	0.982	0.769	17.20	2.29	2	6	1177	132.5	6.74
Q13541	Eukaryotic translation initiation factor 4E-binding protein 1 OS=Homo sapiens GN=EIF4EBP1 PE=1 SV=3 - [4EBP1_HUMAN]	22.03	1	1	1	4	0.848	1.975	1.247	0.769	17.92	22.03	2	4	118	12.6	5.48
P33176	Kinesin-1 heavy chain OS=Homo sapiens GN=KIF5B PE=1 SV=1 - [KINH_HUMAN]	55.66	2	49	57	304	0.969	0.924	1.037	0.769	829.55	55.66	90	304	963	109.6	6.51
Q86VW2	Rho guanine nucleotide exchange factor 25 OS=Homo sapiens GN=ARHGEF25 PE=1 SV=2 - [ARHGP_HUMAN	1.03	1	1	1	1	0.857	1.071	1.061	0.769	1.72	1.03	1	1	580	63.8	6.61
P42226	Signal transducer and activator of transcription 6 OS=Homo sapiens GN=STAT6 PE=1 SV=1 - [STAT6_HUMAN]	21.25	1	17	17	49	1.006	1.282	1.076	0.769	119.51	21.25	27	49	847	94.1	6.23
Q9Y371	Endophilin-B1 OS=Homo sapiens GN=SH3GLB1 PE=1 SV=1 - [SHLB1_HUMAN]	25.75	2	11	12	56	0.799	0.979	1.040	0.769	113.80	25.75	20	56	365	40.8	6.04
Q8NDH2	Coiled-coil domain-containing protein 168 OS=Homo sapiens GN=CCDC168 PE=2 SV=2 - [CC168_HUMAN]	2.24	1	4	5	5	0.624	1.263	1.143	0.769	9.65	2.24	5	5	2452	277.8	9.31

Q9NP79	Vacuolar protein sorting-associated protein VTA1 homolog OS=Homo sapiens GN=VTA1 PE=1 SV=1 - [VTA1_HUMAN]	26.71	1	8	8	20	0.796	0.571	0.773	0.769	50.86	26.71	11	20	307	33.9	6.29
Q9NYL4	Peptidyl-prolyl cis-trans isomerase FKBP11 OS=Homo sapiens GN=FKBP11 PE=1 SV=1 - [FKB11_HUMAN]	23.38	1	5	5	14	0.540	0.579	0.424	0.770	28.71	23.38	8	14	201	22.2	9.39
Q14012	Calcium/calmodulin-dependent protein kinase type 1 OS=Homo sapiens GN=CAMK1 PE=1 SV=1 - [KCC1A_HUMAN]	25.14	1	6	8	21	0.970	1.044	1.191	0.770	47.21	25.14	15	21	370	41.3	5.29
Q5W0V3	Protein FAM160B1 OS=Homo sapiens GN=FAM160B1 PE=2 SV=1 - [F16B1_HUMAN]	6.67	1	5	5	11	0.698	0.717	0.662	0.770	21.80	6.67	8	11	765	86.5	5.29
Q8NDA2	Hemicentin-2 OS=Homo sapiens GN=HMCN2 PE=2 SV=2 - [HMCN2_HUMAN]	0.53	1	2	2	5	0.685	0.852	0.807	0.770	2.17	0.53	2	5	5065	542.3	5.83
Q6ZRG5	Putative uncharacterized protein FLJ43944 OS=Homo sapiens PE=5 SV=1 - [YQ015_HUMAN]	5.88	1	1	1	1	0.972	1.657	1.414	0.770	2.16	5.88	1	1	221	24.1	4.41
Q8WUH1	Protein Churchill OS=Homo sapiens GN=CHURC1 PE=1 SV=2 - [CHUR_HUMAN]	9.35	1	1	1	5	0.723	1.069	0.912	0.770	10.70	9.35	2	5	139	16.1	5.54
A6NDU8	UPF0600 protein C5orf51 OS=Homo sapiens GN=C5orf51 PE=1 SV=1 - [CE051_HUMAN]	2.38	1	1	1	1	0.934	0.754	0.944	0.771	2.11	2.38	1	1	294	33.6	5.26
Q9Y5Y5	Peroxisomal membrane protein PEX16 OS=Homo sapiens GN=PEX16 PE=1 SV=2 - [PEX16_HUMAN]	10.12	1	3	3	10	0.855	0.537	0.913	0.771	20.90	10.12	5	10	336	38.6	9.85

P51649	Succinate-semialdehyde dehydrogenase, mitochondrial OS=Homo sapiens GN=ALDH5A1 PE=1 SV=2 - [SSDH_HUMAN]	7.29	1	2	2	5	0.592	0.638	0.947	0.771	8.45	7.29	3	5	535	57.2	8.28
P17661	Desmin OS=Homo sapiens GN=DES PE=1 SV=3 - [DESM_HUMAN]	68.30	3	25	32	957	0.650	8.452	2.594	0.771	1924.69	68.30	57	957	470	53.5	5.27
Q3V6T2	Girdin OS=Homo sapiens GN=CCDC88A PE=1 SV=2 - [GRDN_HUMAN]	12.61	4	19	24	54	0.821	1.519	1.027	0.771	109.78	12.61	33	54	1871	215.9	6.21
A8MXV4	Nucleoside diphosphate-linked moiety X motif 19, mitochondrial OS=Homo sapiens GN=NUDT19 PE=1 SV=1 - [NUD19_HUMAN]	5.60	1	2	2	7	0.778	0.587	0.576	0.771	22.25	5.60	3	7	375	42.2	7.64
Q9UMX0	Ubiquilin-1 OS=Homo sapiens GN=UBQLN1 PE=1 SV=2 - [UBQL1_HUMAN]	17.66	1	3	9	45	0.494	0.973	0.741	0.771	135.35	17.66	15	45	589	62.5	5.11
Q9H8M7	Protein FAM188A OS=Homo sapiens GN=FAM188A PE=1 SV=1 - [F188A_HUMAN]	8.31	1	3	3	7	0.878	0.959	1.167	0.771	16.35	8.31	5	7	445	49.7	4.77
Q8TES7	Fas-binding factor 1 OS=Homo sapiens GN=FBF1 PE=1 SV=2 - [FBF1_HUMAN]	3.27	8	2	3	6	0.952	0.717	0.825	0.771	7.90	3.27	3	6	1133	125.4	7.08
Q5MNZ6	WD repeat domain phosphoinositide-interacting protein 3 OS=Homo sapiens GN=WDR45B PE=2 SV=2 - [WIPI3_HUMAN]	4.94	1	1	2	2	0.967	0.872	1.048	0.771	2.38	4.94	2	2	344	38.1	7.59
O60701	UDP-glucose 6-dehydrogenase OS=Homo sapiens GN=UGDH PE=1 SV=1 - [UGDH_HUMAN]	63.16	1	29	29	251	0.855	0.757	1.018	0.771	589.40	63.16	48	251	494	55.0	7.12

Q4LE39	AT-rich interactive domain-containing protein 4B OS=Homo sapiens GN=ARID4B PE=1 SV=2 - [ARI4B_HUMAN]	0.61	1	1	1	4	0.773	0.643	0.684	0.772	6.57	0.61	2	4	1312	147.7	5.12
Q8WTS6	Histone-lysine N-methyltransferase SETD7 OS=Homo sapiens GN=SETD7 PE=1 SV=1 - [SETD7_HUMAN]	23.50	1	7	7	24	0.776	1.264	0.964	0.772	71.03	23.50	12	24	366	40.7	4.63
Q6NT32	Carboxylesterase 5A OS=Homo sapiens GN=CES5A PE=2 SV=1 - [EST5A_HUMAN]	9.57	1	1	2	2	1.100	0.613	0.770	0.772	2.30	9.57	2	2	575	63.9	6.44
Q9BW71	HIRA-interacting protein 3 OS=Homo sapiens GN=HIRIP3 PE=1 SV=3 - [HIRP3_HUMAN]	3.42	1	2	2	3	0.956	0.907	1.013	0.772	4.88	3.42	2	3	556	61.9	8.54
O60504	Vinexin OS=Homo sapiens GN=SORBS3 PE=1 SV=2 - [VINEX_HUMAN]	19.08	1	14	14	71	1.094	1.166	1.159	0.772	160.95	19.08	26	71	671	75.3	9.45
Q71F56	Mediator of RNA polymerase II transcription subunit 13-like OS=Homo sapiens GN=MED13L PE=1 SV=1 - [MD13L_HUMAN]	0.32	1	1	1	1	0.894	1.120	0.773	0.772	1.69	0.32	1	1	2210	242.4	6.04
Q5JVS0	Intracellular hyaluronan-binding protein 4 OS=Homo sapiens GN=HABP4 PE=1 SV=1 - [HABP4_HUMAN]	19.13	1	6	6	17	1.136	1.519	1.191	0.772	42.71	19.13	9	17	413	45.8	7.46
Q9NRZ9	Lymphoid-specific helicase OS=Homo sapiens GN=HELLS PE=1 SV=1 - [HELLS_HUMAN]	5.01	1	2	2	24	1.622	1.218	1.226	0.773	35.82	5.01	2	24	838	97.0	7.93

Q8IV35	WD repeat-containing protein 49 OS=Homo sapiens GN=WDR49 PE=2 SV=1 - [WDR49_HUMAN]	0.86	1	1	1	2	0.935		1.302	0.773	6.90	0.86	1	2	697	79.2	8.47
Q8TB69	Zinc finger protein 519 OS=Homo sapiens GN=ZNF519 PE=2 SV=2 - [ZN519_HUMAN]	4.63	96	1	3	6	1.133	0.756	0.789	0.773	6.32	4.63	4	6	540	63.0	9.36
Q9BW60	Elongation of very long chain fatty acids protein 1 OS=Homo sapiens GN=ELOVL1 PE=1 SV=1 - [ELOV1_HUMAN]	4.30	1	1	1	27	0.855	0.814	1.205	0.773	41.96	4.30	2	27	279	32.6	9.60
P36507	Dual specificity mitogen-activated protein kinase kinase 2 OS=Homo sapiens GN=MAP2K2 PE=1 SV=1 - [MP2K2_HUMA	23.75	1	6	11	47	1.001	0.851	0.974	0.773	102.65	23.75	19	47	400	44.4	6.55
Q9BQ24	Zinc finger FYVE domain-containing protein 21 OS=Homo sapiens GN=ZFYVE21 PE=1 SV=1 - [ZFY21_HUMAN]	9.83	1	2	3	7	0.897	0.757	1.006	0.773	12.88	9.83	4	7	234	26.5	8.41
Q9BRK5	45 kDa calcium-binding protein OS=Homo sapiens GN=SDF4 PE=1 SV=1 - [CAB45_HUMAN]	38.12	1	10	11	59	0.803	0.873	0.838	0.773	179.48	38.12	16	59	362	41.8	4.86
Q9UNE0	Tumor necrosis factor receptor superfamily member EDAR OS=Homo sapiens GN=EDAR PE=1 SV=1 - [EDAR_HUMAN]	1.34	1	1	1	3	0.587	1.907	1.040	0.773	6.55	1.34	1	3	448	48.6	5.17
O00305	Voltage-dependent L-type calcium channel subunit beta-4 OS=Homo sapiens GN=CACNB4 PE=1 SV=2 - [CACB4_HUMAN]	9.81	3	2	3	6	1.426	1.033	0.771	0.773	9.42	9.81	4	6	520	58.1	9.32

Q6NUN9	Zinc finger protein 746 OS=Homo sapiens GN=ZNF746 PE=1 SV=1 - [ZN746_HUMAN]	8.54	3	3	3	3	0.860	0.923	1.088	0.773	6.67	8.54	3	3	644	69.1	6.86
P53582	Methionine aminopeptidas e 1 OS=Homo sapiens GN=METAP1 PE=1 SV=2 - [MAP11_HUMA N]	25.13	1	9	9	28	0.935	1.201	1.280	0.773	61.92	25.13	15	28	386	43.2	7.17
Q5HYI8	Rab-like protein 3 OS=Homo sapiens GN=RABL3 PE=1 SV=1 - [RABL3_HUMAN	9.75	1	2	2	6	0.870	0.629	0.650	0.774	17.70	9.75	2	6	236	26.4	7.11
O00233	26S proteasome non-ATPase regulatory subunit 9 OS=Homo sapiens GN=PSMD9 PE=1 SV=3 - [PSMD9_HUMA	35.87	1	8	9	44	1.002	1.087	1.122	0.774	103.09	35.87	15	44	223	24.7	6.95
Q9UHV9	Prefoldin subunit 2 OS=Homo sapiens GN=PFDN2 PE=1 SV=1 - [PFD2_HUMAN]	40.91	1	6	6	20	0.802	1.051	0.910	0.774	48.47	40.91	9	20	154	16.6	6.58
Q86UX2	Inter-alpha-trypsin inhibitor heavy chain H5 OS=Homo sapiens GN=ITIH5 PE=2 SV=2 - [ITIH5_HUMAN]	0.64	1	1	1	1	0.854	1.013	1.057	0.774	2.24	0.64	1	1	942	104.5	8.28
P24387	Corticotropin-releasing factor-binding protein OS=Homo sapiens GN=CRHBP PE=1 SV=2 - [CRHBP_HUMAN	6.21	1	2	2	2	0.745	0.977	0.864	0.774	4.90	6.21	2	2	322	36.1	6.52
Q5T0Z8	Uncharacterize d protein C6orf132 OS=Homo sapiens GN=C6orf132 PE=1 SV=4 - [CF132_HUMAN]	0.93	1	1	1	1	0.868	0.854	0.941	0.774	0.00	0.93	1	1	1188	124.0	9.45
Q92692	Nectin-2 OS=Homo sapiens GN=PVRL2 PE=1 SV=1 - [PVRL2_HUMAN]	9.29	1	4	4	13	1.502	0.927	1.064	0.774	22.88	9.29	8	13	538	57.7	4.82

Q99614	Tetratricopeptide repeat protein 1 OS=Homo sapiens GN=TTC1 PE=1 SV=1 - [TTC1_HUMAN]	39.38	1	11	11	28	0.878	1.074	1.039	0.774	75.72	39.38	15	28	292	33.5	4.84
Q9NYU1	UDP-glucose:glycoprotein glucosyltransferase 2 OS=Homo sapiens GN=UGGT2 PE=1 SV=4 - [UGGG2_HUMAN]	14.78	1	20	21	69	0.835	0.548	0.682	0.774	163.09	14.78	36	69	1516	174.6	6.89
Q86VP3	Phosphofurin acidic cluster sorting protein 2 OS=Homo sapiens GN=PACS2 PE=1 SV=3 - [PACS2_HUMAN]	3.15	1	3	3	6	0.852	0.610	0.716	0.775	7.05	3.15	4	6	889	97.6	6.60
Q96J02	E3 ubiquitin-protein ligase Itchy homolog OS=Homo sapiens GN=ITCH PE=1 SV=2 - [ITCH_HUMAN]	11.18	2	7	7	12	0.673	1.036	1.151	0.775	33.46	11.18	10	12	903	102.7	6.30
Q3MHD2	Protein LSM12 homolog OS=Homo sapiens GN=LSM12 PE=1 SV=2 - [LSM12_HUMAN]	13.85	1	2	2	3	0.991	1.192	0.672	0.775	8.27	13.85	3	3	195	21.7	7.74
P53611	Geranylgeranyl transferase type-2 subunit beta OS=Homo sapiens GN=RABGGTB PE=1 SV=2 - [PGTB2_HUMAN	5.14	1	2	2	3	0.958	0.828	1.011	0.775	5.32	5.14	3	3	331	36.9	5.03
O60841	Eukaryotic translation initiation factor 5B OS=Homo sapiens GN=EIF5B PE=1 SV=4 - [IF2P_HUMAN]	30.49	1	39	40	119	0.674	0.926	0.934	0.775	278.36	30.49	58	119	1220	138.7	5.49
Q53QV2	Protein LBH OS=Homo sapiens GN=LBH PE=1 SV=1 - [LBH_HUMAN]	38.10	1	3	3	9	0.664	0.706	2.328	0.775	34.26	38.10	4	9	105	12.2	4.41
O95749	Geranylgeranyl pyrophosphate synthase OS=Homo sapiens GN=GGPS1 PE=1 SV=1 - [GGPPS_HUMAN]	18.00	1	5	5	17	0.943	1.126	1.100	0.775	35.58	18.00	8	17	300	34.8	6.14

O43707	Alpha-actinin-4 OS=Homo sapiens GN=ACTN4 PE=1 SV=2 - [ACTN4_HUMAN]	77.28	1	52	76	2837	0.929	1.381	1.528	0.776	7260.34	77.28	131	2837	911	104.8	5.44
Q8IXJ6	NAD-dependent protein deacetylase sirtuin-2 OS=Homo sapiens GN=SIRT2 PE=1 SV=2 - [SIR2_HUMAN]	4.88	1	2	2	4	0.907	1.087	1.094	0.776	16.01	4.88	2	4	389	43.2	5.36
Q96A49	Synapse-associated protein 1 OS=Homo sapiens GN=SYAP1 PE=1 SV=1 - [SYAP1_HUMAN]	28.98	1	13	13	36	0.902	0.824	0.875	0.776	63.30	28.98	19	36	352	39.9	4.53
Q9Y5L0	Transportin-3 OS=Homo sapiens GN=TNPO3 PE=1 SV=3 - [TNPO3_HUMAN]	6.39	1	5	5	9	0.915	0.932	0.805	0.776	18.90	6.39	6	9	923	104.1	5.57
Q9ULV4	Coronin-1C OS=Homo sapiens GN=CORO1C PE=1 SV=1 - [COR1C_HUMAN]	39.87	2	28	30	170	0.771	0.891	0.965	0.776	304.88	39.87	50	170	474	53.2	7.08
P09668	Pro-cathepsin H OS=Homo sapiens GN=CTSH PE=1 SV=4 - [CATH_HUMAN]	6.57	1	2	2	2	0.884	0.781	0.873	0.777	2.98	6.57	2	2	335	37.4	8.07
P63151	Serine/threonine-protein phosphatase 2A 55 kDa regulatory subunit B alpha isoform OS=Homo sapiens GN=PPP2R2A PE=1 SV=1 - [2ABA_HUMAN]	30.65	4	11	11	36	1.085	1.057	1.035	0.777	91.22	30.65	16	36	447	51.7	6.20
Q9GZT6	Coiled-coil domain-containing protein 90B, mitochondrial OS=Homo sapiens GN=CCDC90B PE=1 SV=2 - [CC90B_HUMAN]	25.59	1	8	8	20	0.810	0.727	0.770	0.777	45.12	25.59	12	20	254	29.5	7.55
P23560	Brain-derived neurotrophic factor OS=Homo sapiens GN=BDNF PE=1 SV=1 - [BDNF_HUMAN]	4.45	1	1	1	2	0.796	0.632	0.958	0.777	2.84	4.45	2	2	247	27.8	8.79

B2RUZ4	Small integral membrane protein 1 OS=Homo sapiens GN=SMIM1 PE=1 SV=1 - [SMIM1_HUMAN]	20.51	1	1	1	1	1.650	1.632	2.622	0.778	4.88	20.51	1	1	78	8.7	9.03
Q96Q35	Amyotrophic lateral sclerosis 2 chromosomal region candidate gene 12 protein OS=Homo sapiens GN=ALS2CR12 PE=1 SV=2 -	7.87	1	3	3	3	1.043	1.129	0.743	0.778	5.13	7.87	3	3	445	52.4	6.71
Q7LGA3	Heparan sulfate 2-O-sulfotransferase 1 OS=Homo sapiens GN=HS2ST1 PE=1 SV=1 - [HS2ST_HUMAN]	26.69	1	8	8	15	0.843	0.883	0.736	0.778	34.87	26.69	10	15	356	41.9	8.69
Q5EBL4	RILP-like protein 1 OS=Homo sapiens GN=RILPL1 PE=1 SV=1 - [RIPL1_HUMAN]	30.02	2	10	11	37	0.781	0.879	1.064	0.778	90.76	30.02	17	37	403	47.1	5.21
Q9BY32	Inosine triphosphate pyrophosphatase OS=Homo sapiens GN=ITPA PE=1 SV=2 - [ITPA_HUMAN]	19.59	1	3	3	6	0.743	0.983	0.972	0.778	15.23	19.59	4	6	194	21.4	5.66
Q86VP6	Cullin-associated NEDD8-dissociated protein 1 OS=Homo sapiens GN=CAND1 PE=1 SV=2 - [CAND1_HUMAN	30.33	1	32	37	168	0.880	0.933	0.869	0.778	430.88	30.33	61	168	1230	136.3	5.78
Q9UIV1	CCR4-NOT transcription complex subunit 7 OS=Homo sapiens GN=CNOT7 PE=1 SV=3 - [CNOT7_HUMAN]	8.77	1	2	2	7	0.778	0.889	0.558	0.778	25.94	8.77	3	7	285	32.7	4.84
P39900	Macrophage metalloelastase OS=Homo sapiens GN=MMP12 PE=1 SV=1 - [MMP12_HUMAN]	2.13	1	1	1	3	0.799	0.779	0.763	0.778	6.55	2.13	1	3	470	54.0	8.66

Q9BZF2	Oxysterol-binding protein-related protein 7 OS=Homo sapiens GN=OSBPL7 PE=2 SV=1 - [OSBL7_HUMAN]	1.78	1	1	1	3	1.192	1.145	1.594	0.778	5.45	1.78	1	3	842	95.4	8.05
Q9Y4X4	Krueppel-like factor 12 OS=Homo sapiens GN=KLF12 PE=1 SV=2 - [KLF12_HUMAN]	2.24	1	1	1	1	0.988	0.232	0.570	0.778	1.61	2.24	1	1	402	44.2	9.70
Q02750	Dual specificity mitogen-activated protein kinase kinase 1 OS=Homo sapiens GN=MAP2K1 PE=1 SV=2 - [MP2K1_HUMA	30.53	1	7	12	107	1.012	0.884	0.989	0.779	242.62	30.53	21	107	393	43.4	6.62
Q13480	GRB2-associated-binding protein 1 OS=Homo sapiens GN=GAB1 PE=1 SV=2 - [GAB1_HUMAN]	9.94	1	6	6	12	1.135	0.973	0.985	0.779	19.60	9.94	10	12	694	76.6	5.96
Q9UII5	Zinc finger protein 107 OS=Homo sapiens GN=ZNF107 PE=2 SV=1 - [ZN107_HUMAN]	15.71	94	1	2	6	1.009	0.974	1.071	0.779	0.00	15.71	2	6	783	90.6	9.31
P07602	Prosaposin OS=Homo sapiens GN=PSAP PE=1 SV=2 - [SAP_HUMAN]	33.78	1	18	18	105	0.824	1.174	1.035	0.779	168.40	33.78	32	105	524	58.1	5.17
Q96AY3	Peptidyl-prolyl cis-trans isomerase FKBP10 OS=Homo sapiens GN=FKBP10 PE=1 SV=1 - [FKB10_HUMAN]	61.86	2	32	33	508	0.558	0.675	0.764	0.779	1370.08	61.86	54	508	582	64.2	5.62
P11279	Lysosome-associated membrane glycoprotein 1 OS=Homo sapiens GN=LAMP1 PE=1 SV=3 - [LAMP1_HUMAN]	4.80	1	2	2	61	0.813	0.831	0.808	0.779	124.72	4.80	4	61	417	44.9	8.75
P04275	von Willebrand factor OS=Homo sapiens GN=VWF PE=1 SV=4 - [VWF_HUMAN]	2.03	1	2	3	4	1.224	1.113	1.419	0.779	7.17	2.03	3	4	2813	309.1	5.48

Q96JD6	1,5-anhydro-D-fructose reductase OS=Homo sapiens GN=AKR1E2 PE=1 SV=2 - [AKCL2_HUMAN]	1.88	1	1	1	2	1.099	0.665	0.676	0.779	3.63	1.88	1	2	320	36.6	7.49
O60237	Protein phosphatase 1 regulatory subunit 12B OS=Homo sapiens GN=PPP1R12B PE=1 SV=2 - [MYPT2_HUMAN]	9.16	1	9	10	52	0.845	2.175	1.668	0.779	131.07	9.16	19	52	982	110.3	5.67
Q5HYI7	Metaxin-3 OS=Homo sapiens GN=MTX3 PE=1 SV=2 - [MTX3_HUMAN]	8.65	1	3	3	8	1.070	0.782	0.678	0.779	15.87	8.65	5	8	312	35.1	7.80
Q96T76	MMS19 nucleotide excision repair protein homolog OS=Homo sapiens GN=MMS19 PE=1 SV=2 - [MMS19_HUMAN]	9.22	2	8	9	18	0.786	0.736	1.029	0.779	48.38	9.22	13	18	1030	113.2	6.35
Q8NEW0	Zinc transporter 7 OS=Homo sapiens GN=SLC30A7 PE=2 SV=1 - [ZNT7_HUMAN]	3.99	1	1	1	8	0.735	0.690	0.617	0.780	12.90	3.99	1	8	376	41.6	6.95
Q96GZ6	Solute carrier family 41 member 3 OS=Homo sapiens GN=SLC41A3 PE=2 SV=2 - [S41A3_HUMAN]	1.97	3	1	1	4	0.632	0.776	0.849	0.780	6.45	1.97	2	4	507	54.7	7.83
Q8N0X7	Spartin OS=Homo sapiens GN=SPG20 PE=1 SV=1 - [SPG20_HUMAN]	16.22	1	9	11	29	0.906	0.865	1.005	0.780	50.58	16.22	17	29	666	72.8	5.91
Q5VWQ0	Round spermatid basic protein 1 OS=Homo sapiens GN=RSBN1 PE=1 SV=2 - [RSBN1_HUMAN]	6.48	1	3	4	5	0.940			0.780	7.98	6.48	5	5	802	90.0	8.60
O15260	Surfeit locus protein 4 OS=Homo sapiens GN=SURF4 PE=1 SV=3 - [SURF4_HUMAN]	6.32	1	2	2	16	0.663	0.670	0.551	0.780	39.93	6.32	4	16	269	30.4	7.78

Q13643	Four and a half LIM domains protein 3 OS=Homo sapiens GN=FHL3 PE=1 SV=4 - [FHL3_HUMAN]	16.43	1	6	6	26	0.773	0.826	1.275	0.780	58.19	16.43	10	26	280	31.2	6.20
Q6ZTR7	Protein FAM92B OS=Homo sapiens GN=FAM92B PE=2 SV=1 - [FA92B_HUMAN]	6.91	1	2	2	4	0.675	1.077	1.119	0.780	9.63	6.91	2	4	304	34.8	8.66
O00161	Synaptosomal-associated protein 23 OS=Homo sapiens GN=SNAP23 PE=1 SV=1 - [SNP23_HUMAN]	40.28	1	9	9	41	0.951	0.836	0.821	0.780	96.34	40.28	15	41	211	23.3	5.01
Q9NNX1	Tuftelin OS=Homo sapiens GN=TUFT1 PE=2 SV=1 - [TUFT1_HUMAN]	5.38	2	2	2	2	1.313	0.748	0.526	0.780	5.07	5.38	2	2	390	44.2	6.00
P60484	Phosphatidylinositol 3,4,5-trisphosphate 3-phosphatase and dual-specificity protein phosphatase PTEN OS=Homo sapiens GN=PTEN PE=1 SV=1 - [PTEN_HUMAN]	6.45	1	3	3	5	1.163	0.889	0.791	0.781	12.52	6.45	4	5	403	47.1	6.37
P51946	Cyclin-H OS=Homo sapiens GN=CCNH PE=1 SV=1 - [CCNH_HUMAN]	8.05	1	2	2	3	0.902	1.006	0.920	0.781	5.69	8.05	2	3	323	37.6	7.15
P53004	Biliverdin reductase A OS=Homo sapiens GN=BLVRA PE=1 SV=2 - [BIEA_HUMAN]	39.86	1	14	15	44	0.990	1.199	0.847	0.781	89.98	39.86	24	44	296	33.4	6.44
Q9NYV4	Cyclin-dependent kinase 12 OS=Homo sapiens GN=CDK12 PE=1 SV=2 - [CDK12_HUMAN]	3.15	6	2	4	14	0.861	0.942	0.900	0.781	28.36	3.15	5	14	1490	164.1	9.44
Q16739	Ceramide glucosyltransferase OS=Homo sapiens GN=UGCG PE=1 SV=1 - [CEGT_HUMAN]	3.81	1	1	2	2	1.438	1.084	0.963	0.781	0.00	3.81	2	2	394	44.8	7.78

O95297	Myelin protein zero-like protein 1 OS=Homo sapiens GN=MPZL1 PE=1 SV=1 - [MPZL1_HUMAN	21.56	1	5	5	16	1.120	0.700	0.901	0.781	35.91	21.56	8	16	269	29.1	8.72
Q16363	Laminin subunit alpha-4 OS=Homo sapiens GN=LAMA4 PE=1 SV=4 - [LAMA4_HUMAN]	13.17	1	19	19	48	0.528	0.872	1.243	0.781	115.23	13.17	28	48	1823	202.4	6.28
P35613	Basigin OS=Homo sapiens GN=BSG PE=1 SV=2 - [BASI_HUMAN]	28.57	1	9	9	92	0.978	0.833	0.880	0.781	231.87	28.57	15	92	385	42.2	5.66
P52788	Spermine synthase OS=Homo sapiens GN=SMS PE=1 SV=2 - [SPSY_HUMAN]	45.08	1	16	16	49	0.971	1.259	0.897	0.782	94.32	45.08	23	49	366	41.2	5.02
Q13464	Rho-associated protein kinase 1 OS=Homo sapiens GN=ROCK1 PE=1 SV=1 - [ROCK1_HUMA N]	28.73	1	34	39	110	0.857	1.498	1.406	0.782	229.17	28.73	62	110	1354	158.1	5.90
P86790	Vacuolar fusion protein CCZ1 homolog B OS=Homo sapiens GN=CCZ1B PE=1 SV=1 - [CCZ1B_HUMAN]	15.77	1	7	7	28	0.674	0.769	0.510	0.782	29.72	15.77	9	28	482	55.8	6.48
O75369	Filamin-B OS=Homo sapiens GN=FLNB PE=1 SV=2 - [FLNB_HUMAN]	59.38	1	123	137	1353	1.442	0.688	1.067	0.782	3235.99	59.38	230	1353	2602	278.0	5.73
Q6XZF7	Dynamin-binding protein OS=Homo sapiens GN=DNMBP PE=1 SV=1 - [DNMBP_HUMA N]	9.89	1	15	15	40	0.832	1.305	1.044	0.782	82.15	9.89	23	40	1577	177.2	5.39
Q969J3	Loss of heterozygosity 12 chromosomal region 1 protein OS=Homo sapiens GN=LOH12CR1 PE=1 SV=1 - [L12R1_HUMAN	39.29	1	7	7	14	0.905	1.046	0.886	0.782	30.59	39.29	10	14	196	22.2	6.35

Q8NFZ0	F-box only protein 18 OS=Homo sapiens GN=FBXO18 PE=1 SV=2 - [FBX18_HUMAN]	0.86	1	1	1	1	1.415	0.894	0.785	0.782	0.00	0.86	1	1	1043	117.6	8.25
Q8WUM4	Programmed cell death 6-interacting protein OS=Homo sapiens GN=PDCD6IP PE=1 SV=1 - [PDC6I_HUMAN]	55.30	1	47	47	324	0.930	1.060	1.028	0.782	686.41	55.30	76	324	868	96.0	6.52
Q9C0C2	182 kDa tankyrase-1-binding protein OS=Homo sapiens GN=TNKS1BP1 PE=1 SV=4 - [TB182_HUMAN]	48.24	1	65	65	266	0.974	1.079	0.850	0.783	800.48	48.24	98	266	1729	181.7	4.86
P29279	Connective tissue growth factor OS=Homo sapiens GN=CTGF PE=1 SV=2 - [CTGF_HUMAN]	33.81	1	13	13	53	0.694	0.392	0.660	0.783	108.13	33.81	20	53	349	38.1	8.00
P01116	GTPase KRas OS=Homo sapiens GN=KRAS PE=1 SV=1 - [RASK_HUMAN]	46.56	1	3	5	22	0.931	1.177	1.078	0.783	48.08	46.56	8	22	189	21.6	6.77
Q92544	Transmembrane 9 superfamily member 4 OS=Homo sapiens GN=TM9SF4 PE=1 SV=2 - [TM9S4_HUMAN]	8.26	1	5	5	17	0.782	0.682	0.702	0.783	31.60	8.26	9	17	642	74.5	6.54
P54578	Ubiquitin carboxyl-terminal hydrolase 14 OS=Homo sapiens GN=USP14 PE=1 SV=3 - [UBP14_HUMAN]	49.19	1	23	23	85	0.860	0.958	0.957	0.783	208.72	49.19	30	85	494	56.0	5.30
A1A4S6	Rho GTPase-activating protein 10 OS=Homo sapiens GN=ARHGAP10 PE=1 SV=1 - [RHG10_HUMAN]	10.43	1	7	9	17	1.191	0.807	1.390	0.783	37.15	10.43	13	17	786	89.3	7.18
P55060	Exportin-2 OS=Homo sapiens GN=CSE1L PE=1 SV=3 - [XPO2_HUMAN]	27.09	1	26	26	98	0.804	0.960	0.844	0.783	249.83	27.09	43	98	971	110.3	5.77

O00410	Importin-5 OS=Homo sapiens GN=IPO5 PE=1 SV=4 - [IPO5_HUMAN]	26.89	1	25	25	111	0.504	0.782	0.781	0.783	299.20	26.89	40	111	1097	123.5	4.94
Q9H8W5	Tripartite motif-containing protein 45 OS=Homo sapiens GN=TRIM45 PE=1 SV=2 - [TRI45_HUMAN]	5.86	1	3	3	3	1.626	0.388	0.498	0.783	2.64	5.86	3	3	580	64.3	7.91
Q96M89	Coiled-coil domain-containing protein 138 OS=Homo sapiens GN=CCDC138 PE=1 SV=1 - [CC138_HUMAN]	1.80	1	1	2	3	1.159	1.348	1.234	0.783	6.90	1.80	2	3	665	76.2	8.53
P04049	RAF proto-oncogene serine/threonine-protein kinase OS=Homo sapiens GN=RAF1 PE=1 SV=1 - [RAF1_HUMAN]	6.64	1	3	4	5	1.035	1.524	1.671	0.783	12.89	6.64	5	5	648	73.0	9.20
Q9UQ13	Leucine-rich repeat protein SHOC-2 OS=Homo sapiens GN=SHOC2 PE=1 SV=2 - [SHOC2_HUMAN]	4.47	1	2	2	2	1.096	1.202	1.088	0.783	8.40	4.47	2	2	582	64.8	8.46
Q9UHE8	Metalloreductase STEAP1 OS=Homo sapiens GN=STEAP1 PE=1 SV=1 - [STEA1_HUMAN]	9.14	2	4	4	13	0.379	1.406	0.649	0.784	32.43	9.14	6	13	339	39.8	9.23
Q6WRX3	Protein zyg-11 homolog A OS=Homo sapiens GN=ZYG11A PE=2 SV=3 - [ZY11A_HUMAN]	1.71	1	1	2	2	1.379	1.134	1.191	0.784	4.24	1.71	2	2	759	85.8	7.97
P49754	Vacuolar protein sorting-associated protein 41 homolog OS=Homo sapiens GN=VPS41 PE=1 SV=3 - [VPS41_HUMAN]	7.26	1	6	6	10	0.768	1.197	0.930	0.784	28.80	7.26	7	10	854	98.5	5.85
Q9NQS1	Cell death regulator Aven OS=Homo sapiens GN=AVEN PE=1 SV=1 - [AVEN_HUMAN]	9.94	2	3	3	12	0.652	0.776	0.749	0.784	34.57	9.94	3	12	362	38.5	4.98

Q96H22	Centromere protein N OS=Homo sapiens GN=CENPN PE=1 SV=2 - [CENPN_HUMAN]	3.24	1	1	1	2	1.400	0.980	0.509	0.784	6.29	3.24	1	2	339	39.5	9.13
Q07866	Kinesin light chain 1 OS=Homo sapiens GN=KLC1 PE=1 SV=2 - [KLC1_HUMAN]	55.15	1	26	36	199	0.948	0.833	1.016	0.784	535.95	55.15	57	199	573	65.3	6.20
Q7Z7G1	Cytokine-dependent hematopoietic cell linker OS=Homo sapiens GN=CLNK PE=1 SV=2 - [CLNK_HUMAN]	2.34	1	1	1	1	0.864	0.931	1.128	0.784	0.00	2.34	1	1	428	49.5	9.01
Q53SF7	Cordon-bleu protein-like 1 OS=Homo sapiens GN=COBLL1 PE=1 SV=2 - [COBL1_HUMAN]	8.06	1	8	8	15	0.863	0.967	1.529	0.784	31.53	8.06	11	15	1204	131.7	6.67
P23528	Cofilin-1 OS=Homo sapiens GN=CFL1 PE=1 SV=3 - [COF1_HUMAN]	87.35	1	19	24	504	1.091	1.070	1.116	0.784	1182.38	87.35	38	504	166	18.5	8.09
Q5VIR6	Vacuolar protein sorting-associated protein 53 homolog OS=Homo sapiens GN=VPS53 PE=1 SV=1 - [VPS53_HUMAN]	6.58	1	5	5	11	0.840	0.909	0.923	0.784	22.70	6.58	7	11	699	79.6	6.02
Q01469	Fatty acid-binding protein, epidermal OS=Homo sapiens GN=FABP5 PE=1 SV=3 - [FABP5_HUMAN	42.96	2	7	7	40	0.527	1.399	1.403	0.785	100.98	42.96	11	40	135	15.2	7.01
P60660	Myosin light polypeptide 6 OS=Homo sapiens GN=MYL6 PE=1 SV=2 - [MYL6_HUMAN]	84.77	2	7	12	819	0.952	0.944	1.148	0.785	1984.98	84.77	23	819	151	16.9	4.65
Q9UBG0	C-type mannose receptor 2 OS=Homo sapiens GN=MRC2 PE=1 SV=2 - [MRC2_HUMAN]	13.12	1	13	13	54	0.753	0.841	1.026	0.785	179.59	13.12	23	54	1479	166.6	5.83

Q9UNF1	Melanoma-associated antigen D2 OS=Homo sapiens GN=MAGED2 PE=1 SV=2 - [MAGD2_HUMAN]	27.56	1	14	15	89	0.665	0.814	0.850	0.785	210.11	27.56	23	89	606	64.9	9.32
Q6IAN0	Dehydrogenase/reductase SDR family member 7B OS=Homo sapiens GN=DHRS7B PE=1 SV=2 - [DRS7B_HUMAN]	15.38	1	4	4	11	0.804	0.764	0.744	0.785	28.40	15.38	6	11	325	35.1	9.55
P49356	Protein farnesyltransferase subunit beta OS=Homo sapiens GN=FNTB PE=1 SV=1 - [FNTB_HUMAN]	5.72	1	4	4	11	0.851	0.909	0.794	0.785	20.12	5.72	7	11	437	48.7	5.82
P55010	Eukaryotic translation initiation factor 5 OS=Homo sapiens GN=EIF5 PE=1 SV=2 - [IF5_HUMAN]	34.34	1	17	17	107	0.735	0.900	0.874	0.785	210.57	34.34	26	107	431	49.2	5.58
Q8N3D4	EH domain-binding protein 1-like protein 1 OS=Homo sapiens GN=EHBP1L1 PE=1 SV=2 - [EH1L1_HUMAN]	17.33	1	20	21	65	0.908	1.167	1.497	0.786	189.18	17.33	33	65	1523	161.8	4.83
Q9H7D0	Dedicator of cytokinesis protein 5 OS=Homo sapiens GN=DOCK5 PE=1 SV=3 - [DOCK5_HUMAN]	5.08	1	7	10	16	1.325	0.918	1.038	0.786	26.52	5.08	13	16	1870	215.2	7.96
Q9H3K2	Growth hormone-inducible transmembrane protein OS=Homo sapiens GN=GHITM PE=1 SV=2 - [GHITM_HUMAN]	6.38	1	2	2	19	0.889	0.929	0.612	0.786	59.66	6.38	3	19	345	37.2	9.94
P62140	Serine/threonine-protein phosphatase PP1-beta catalytic subunit OS=Homo sapiens GN=PPP1CB PE=1 SV=3 - [PP1B_HUMAN]	48.62	1	6	14	89	0.930	1.096	1.352	0.786	225.10	48.62	25	89	327	37.2	6.19

Q6ZVF9	G protein-regulated inducer of neurite outgrowth 3 OS=Homo sapiens GN=GPRIN3 PE=2 SV=2 - [GRIN3_HUMAN]	2.06	3	2	2	2	0.923	0.564	0.822	0.786	3.91	2.06	2	2	776	82.4	7.56
Q5GLZ8	Probable E3 ubiquitin-protein ligase HERC4 OS=Homo sapiens GN=HERC4 PE=1 SV=1 - [HERC4_HUMAN]	15.99	1	14	14	37	0.815	0.757	1.045	0.786	91.46	15.99	24	37	1057	118.5	6.19
P62873	Guanine nucleotide-binding protein G(I)/G(S)/G(T) subunit beta-1 OS=Homo sapiens GN=GNB1 PE=1 SV=3 - [GBB1_HUMAN]	36.47	2	7	10	53	0.968	0.690	0.868	0.786	129.12	36.47	16	53	340	37.4	6.00
O00471	Exocyst complex component 5 OS=Homo sapiens GN=EXOC5 PE=1 SV=1 - [EXOC5_HUMAN]	18.50	1	15	15	38	1.072	0.859	0.912	0.786	88.93	18.50	26	38	708	81.8	6.71
Q8TED9	Actin filament-associated protein 1-like 1 OS=Homo sapiens GN=AFAP1L1 PE=1 SV=2 - [AF1L1_HUMAN]	1.95	1	1	1	1	0.586	0.566	0.681	0.787	2.18	1.95	1	1	768	86.4	6.80
Q12929	Epidermal growth factor receptor kinase substrate 8 OS=Homo sapiens GN=EPS8 PE=1 SV=1 - [EPS8_HUMAN]	24.45	1	15	15	34	0.717	1.094	1.018	0.787	81.29	24.45	24	34	822	91.8	7.50
Q8NI08	Nuclear receptor coactivator 7 OS=Homo sapiens GN=NCOA7 PE=1 SV=2 - [NCOA7_HUMA	0.64	1	1	1	2	0.857	0.883	1.213	0.787	1.65	0.64	1	2	942	106.1	5.59
Q13017	Rho GTPase-activating protein 5 OS=Homo sapiens GN=ARHGAP5 PE=1 SV=2 - [RHG05_HUMAN]	7.52	2	7	8	17	0.740	1.291	1.073	0.787	38.65	7.52	12	17	1502	172.4	6.62

Q9Y4K3	TNF receptor-associated factor 6 OS=Homo sapiens GN=TRAF6 PE=1 SV=1 - [TRAF6_HUMAN	2.30	1	1	1	2	1.033	0.619	0.886	0.787	2.66	2.30	2	2	522	59.5	6.44
Q92804	TATA-binding protein-associated factor 2N OS=Homo sapiens GN=TAF15 PE=1 SV=1 - [RBP56_HUMAN	8.28	1	2	5	35	0.702	0.960	0.695	0.787	74.54	8.28	8	35	592	61.8	8.02
Q99439	Calponin-2 OS=Homo sapiens GN=CNN2 PE=1 SV=4 - [CNN2_HUMAN]	67.96	1	16	18	239	0.953	0.896	1.313	0.787	572.55	67.96	30	239	309	33.7	7.33
Q96JB1	Dynein heavy chain 8, axonemal OS=Homo sapiens GN=DNAH8 PE=1 SV=2 - [DYH8_HUMAN]	1.58	1	4	5	5	0.735	0.946	0.738	0.788	10.52	1.58	5	5	4490	514.3	6.32
Q5BJH7	Protein YIF1B OS=Homo sapiens GN=YIF1B PE=1 SV=1 - [YIF1B_HUMAN]	28.34	1	5	5	11	1.001	0.941	0.778	0.788	31.07	28.34	5	11	314	34.4	9.16
Q92581	Sodium/hydrogen exchanger 6 OS=Homo sapiens GN=SLC9A6 PE=1 SV=2 - [SL9A6_HUMAN]	1.79	1	1	1	2	0.853	0.402	0.489	0.788	7.69	1.79	2	2	669	74.1	6.48
Q8WUX9	Charged multivesicular body protein 7 OS=Homo sapiens GN=CHMP7 PE=1 SV=1 - [CHMP7_HUMAN]	5.74	1	3	3	9	0.784	1.065	0.983	0.788	16.68	5.74	3	9	453	50.9	5.35
O75083	WD repeat-containing protein 1 OS=Homo sapiens GN=WDR1 PE=1 SV=4 - [WDR1_HUMAN]	70.30	1	35	35	368	0.962	1.042	1.244	0.788	1020.07	70.30	59	368	606	66.2	6.65
P50225	Sulfotransferase 1A1 OS=Homo sapiens GN=SULT1A1 PE=1 SV=3 - [ST1A1_HUMAN]	24.75	3	7	7	37	0.713	0.607	0.660	0.788	92.53	24.75	13	37	295	34.1	6.62

Q9HD45	Transmembrane 9 superfamily member 3 OS=Homo sapiens GN=TM9SF3 PE=1 SV=2 - [TM9S3_HUMAN]	11.54	1	6	6	15	0.917	0.689	0.587	0.788	26.94	11.54	9	15	589	67.8	7.21
Q92562	Polyphosphoinositide phosphatase OS=Homo sapiens GN=FIG4 PE=1 SV=1 - [FIG4_HUMAN]	1.87	1	2	2	5	0.911	1.177	1.050	0.788	10.44	1.87	2	5	907	103.6	6.92
P35475	Alpha-L-iduronidase OS=Homo sapiens GN=IDUA PE=1 SV=2 - [IDUA_HUMAN]	17.76	1	10	10	32	0.888	0.840	0.960	0.788	64.86	17.76	19	32	653	72.6	9.14
Q15678	Tyrosine-protein phosphatase non-receptor type 14 OS=Homo sapiens GN=PTPN14 PE=1 SV=2 - [PTN14_HUMAN]	3.71	1	3	3	3	0.648	1.328	1.158	0.788	6.93	3.71	3	3	1187	135.2	8.31
Q9H6Y7	E3 ubiquitin-protein ligase RNF167 OS=Homo sapiens GN=RNF167 PE=1 SV=1 - [RN167_HUMAN]	2.57	1	1	1	2	0.951	0.980	0.760	0.788	3.66	2.57	2	2	350	38.3	5.63
B1ANS9	WD repeat-containing protein 64 OS=Homo sapiens GN=WDR64 PE=2 SV=1 - [WDR64_HUMAN]	1.85	1	1	2	2	1.114	0.726	0.940	0.788	5.32	1.85	2	2	1081	123.6	8.19
Q86UD0	Suppressor APC domain-containing protein 2 OS=Homo sapiens GN=SAPCD2 PE=2 SV=2 - [SAPC2_HUMAN]	1.78	1	1	1	3	0.787	0.872	1.201	0.789	4.30	1.78	2	3	394	42.6	8.82
P33897	ATP-binding cassette sub-family D member 1 OS=Homo sapiens GN=ABCD1 PE=1 SV=2 - [ABCD1_HUMAN]	10.07	2	6	7	24	1.123	1.376	1.428	0.789	59.17	10.07	11	24	745	82.9	8.95

O60684	Importin subunit alpha-7 OS=Homo sapiens GN=KPNA6 PE=1 SV=1 - [IMA7_HUMAN]	22.01	1	6	10	48	0.925	1.015	1.103	0.789	123.76	22.01	16	48	536	60.0	4.98
P12883	Myosin-7 OS=Homo sapiens GN=MYH7 PE=1 SV=5 - [MYH7_HUMAN]	5.84	1	1	13	95	0.789	0.916	0.941	0.789	192.94	5.84	16	95	1935	223.0	5.80
Q8WUG5	Solute carrier family 22 member 17 OS=Homo sapiens GN=SLC22A17 PE=2 SV=1 - [S22AH_HUMAN]	3.72	1	1	1	1	0.644	0.569	0.476	0.789	0.00	3.72	1	1	538	57.6	7.39
P62495	Eukaryotic peptide chain release factor subunit 1 OS=Homo sapiens GN=ETF1 PE=1 SV=3 - [ERF1_HUMAN]	26.77	1	11	11	43	0.884	0.742	0.826	0.790	113.26	26.77	17	43	437	49.0	5.71
Q8IWT3	Cullin-9 OS=Homo sapiens GN=CUL9 PE=1 SV=2 - [CUL9_HUMAN]	0.36	1	1	1	2	1.490	1.125	1.188	0.790	5.28	0.36	1	2	2517	281.0	5.45
Q9BZM5	NKG2D ligand 2 OS=Homo sapiens GN=ULBP2 PE=1 SV=1 - [N2DL2_HUMAN]	7.32	1	1	1	2	0.744	1.874	1.383	0.790	5.15	7.32	1	2	246	27.3	7.30
O43613	Orexin receptor type 1 OS=Homo sapiens GN=HCRTR1 PE=2 SV=2 - [OX1R_HUMAN]	2.82	1	1	1	1	0.705	1.121	1.108	0.790	0.00	2.82	1	1	425	47.5	9.10
Q9NQS3	Nectin-3 OS=Homo sapiens GN=PVRL3 PE=1 SV=1 - [PVRL3_HUMAN]	1.64	1	1	1	2	1.306	0.965		0.791	5.04	1.64	1	2	549	61.0	6.19
Q8NDI1	EH domain-binding protein 1 OS=Homo sapiens GN=EHBP1 PE=1 SV=3 - [EHBP1_HUMAN]	8.53	1	8	10	17	0.999	1.467	1.132	0.791	39.11	8.53	11	17	1231	139.9	5.35
O14733	Dual specificity mitogen-activated protein kinase kinase 7 OS=Homo sapiens GN=MAP2K7 PE=1 SV=2 - [MP2K7_HUMA	5.73	1	2	3	7	0.940	0.622	0.687	0.791	11.10	5.73	3	7	419	47.5	9.16

Q9NPB1	5'(3')-deoxyribonucleotidase, mitochondrial OS=Homo sapiens GN=NT5M PE=1 SV=1 - [NT5M_HUMAN]	5.26	1	1	1	2	0.935	0.768	1.621	0.791	0.00	5.26	1	2	228	25.8	8.00
Q13618	Cullin-3 OS=Homo sapiens GN=CUL3 PE=1 SV=2 - [CUL3_HUMAN]	28.65	1	19	21	47	0.772	0.759	0.846	0.791	108.34	28.65	33	47	768	88.9	8.48
Q14596	Next to BRCA1 gene 1 protein OS=Homo sapiens GN=NBR1 PE=1 SV=3 - [NBR1_HUMAN]	2.07	1	1	1	1	0.897	1.392	1.557	0.792	4.31	2.07	1	1	966	107.3	5.12
A2VEC9	SCO-spondin OS=Homo sapiens GN=SSPO PE=2 SV=1 - [SSPO_HUMAN]	0.45	1	2	2	2	1.144	1.000	0.984	0.792	5.06	0.45	2	2	5147	547.1	6.02
Q9Y2S2	Lambda-crystallin homolog OS=Homo sapiens GN=CRYL1 PE=1 SV=3 - [CRYL1_HUMAN]	15.05	1	5	5	21	0.706	1.091	0.922	0.792	56.27	15.05	8	21	319	35.4	6.18
P06865	Beta-hexosaminidase subunit alpha OS=Homo sapiens GN=HEXA PE=1 SV=2 - [HEXA_HUMAN]	22.12	1	12	14	78	0.748	1.006	0.922	0.792	176.98	22.12	23	78	529	60.7	5.16
Q9UNM6	26S proteasome non-ATPase regulatory subunit 13 OS=Homo sapiens GN=PSMD13 PE=1 SV=2 - [PSD13_HUMAN	46.81	1	17	17	59	0.878	0.884	0.876	0.792	136.77	46.81	27	59	376	42.9	5.81
Q8N2U9	PQ-loop repeat-containing protein 1 OS=Homo sapiens GN=PQLC1 PE=2 SV=1 - [PQLC1_HUMAN	8.86	1	2	2	2	1.154	0.833	0.614	0.792	4.27	8.86	2	2	271	30.5	8.18
Q8N8A2	Serine/threonine-protein phosphatase 6 regulatory ankyrin repeat subunit B OS=Homo sapiens GN=ANKRD44 PE=1 SV=3 - [ANR44_HUMAN]	5.14	1	5	5	11	0.758	0.976	1.105	0.793	30.28	5.14	7	11	993	107.5	6.30

P11362	Fibroblast growth factor receptor 1 OS=Homo sapiens GN=FGFR1 PE=1 SV=3 - [FGFR1_HUMAN]	10.34	7	6	8	28	1.204	0.697	0.640	0.793	60.95	10.34	13	28	822	91.8	6.21
Q8NFU3	Thiosulfate sulfurtransferase/rhodanese-like domain-containing protein 1 OS=Homo sapiens GN=TSTD1 PE=1 SV=3 - [TSTD1_HUMAN]	7.83	1	1	1	3	1.277	2.440	0.904	0.793	3.33	7.83	2	3	115	12.5	6.07
Q6NW29	RWD domain-containing protein 4 OS=Homo sapiens GN=RWDD4 PE=1 SV=3 - [RWDD4_HUMAN]	18.62	2	3	4	15	0.646	0.784	1.046	0.793	31.28	18.62	6	15	188	21.2	5.31
Q9GZT4	Serine racemase OS=Homo sapiens GN=SRR PE=1 SV=1 - [SRR_HUMAN]	23.24	1	6	6	12	1.188	0.897	0.980	0.794	35.99	23.24	9	12	340	36.5	6.54
Q9Y2G5	GDP-fucose protein O-fucosyltransferase 2 OS=Homo sapiens GN=POFUT2 PE=1 SV=3 - [OFUT2_HUMA	21.68	2	10	12	38	0.562	0.775	0.845	0.794	88.70	21.68	17	38	429	49.9	6.60
P86434	Putative uncharacterized protein ADORA2A-AS1 OS=Homo sapiens GN=ADORA2A-AS1 PE=5 SV=1 - [AAS1_HUMAN]	11.32	1	1	1	1	1.638	1.600	1.404	0.794	0.00	11.32	1	1	159	17.2	6.65
Q9UJU6	Drebrin-like protein OS=Homo sapiens GN=DBNL PE=1 SV=1 - [DBNL_HUMAN]	41.16	1	16	16	112	0.885	1.171	1.100	0.795	291.12	41.16	28	112	430	48.2	5.05
O15078	Centrosomal protein of 290 kDa OS=Homo sapiens GN=CEP290 PE=1 SV=2 - [CE290_HUMAN]	2.74	3	4	8	40	0.819	0.819	0.866	0.795	81.16	2.74	11	40	2479	290.2	5.95

Q12830	Nucleosome-remodeling factor subunit BPTF OS=Homo sapiens GN=BPTF PE=1 SV=3 - [BPTF_HUMAN]	1.48	1	3	3	3	0.490	2.169	1.311	0.795	3.16	1.48	3	3	3046	338.1	6.54
Q14653	Interferon regulatory factor 3 OS=Homo sapiens GN=IRF3 PE=1 SV=1 - [IRF3_HUMAN]	9.13	1	4	4	9	1.129	1.184	1.245	0.795	13.25	9.13	6	9	427	47.2	5.34
Q8WTW3	Conserved oligomeric Golgi complex subunit 1 OS=Homo sapiens GN=COG1 PE=1 SV=1 - [COG1_HUMAN]	7.24	1	6	6	10	0.804	0.856	0.904	0.795	24.00	7.24	8	10	980	108.9	7.31
P68036	Ubiquitin-conjugating enzyme E2 L3 OS=Homo sapiens GN=UBE2L3 PE=1 SV=1 - [UB2L3_HUMAN]	58.44	2	9	9	77	1.083	0.964	1.027	0.795	203.26	58.44	15	77	154	17.9	8.51
Q96K12	Fatty acyl-CoA reductase 2 OS=Homo sapiens GN=FAR2 PE=2 SV=1 - [FACR2_HUMAN]	3.11	1	1	1	1	0.885	0.987	2.261	0.795	0.00	3.11	1	1	515	59.4	9.38
P67812	Signal peptidase complex catalytic subunit SEC11A OS=Homo sapiens GN=SEC11A PE=1 SV=1 -	27.93	2	8	8	47	0.763	0.749	0.763	0.795	97.24	27.93	14	47	179	20.6	9.48
Q15843	NEDD8 OS=Homo sapiens GN=NEDD8 PE=1 SV=1 - [NEDD8_HUMAN]	46.91	1	5	6	107	1.498	1.156	1.057	0.795	253.83	46.91	9	107	81	9.1	8.43
O95817	BAG family molecular chaperone regulator 3 OS=Homo sapiens GN=BAG3 PE=1 SV=3 - [BAG3_HUMAN]	45.39	1	19	19	76	0.622	0.947	1.034	0.796	190.83	45.39	33	76	575	61.6	6.95

P49914	5-formyltetrahydrofolate cyclo-ligase OS=Homo sapiens GN=MTHFS PE=1 SV=2 - [MTHFS_HUMAN]	21.18	1	4	4	10	0.720	0.802	0.608	0.796	18.56	21.18	7	10	203	23.2	7.88
P10124	Serglycin OS=Homo sapiens GN=SRGN PE=1 SV=3 - [SRGN_HUMAN]	3.80	1	1	1	1	0.525	1.006	0.753	0.796	1.97	3.80	1	1	158	17.6	4.96
O75569	Interferon-inducible double-stranded RNA-dependent protein kinase activator A OS=Homo sapiens GN=PRKRA PE=1 SV=1 - [PRKRA_HUMAN]	43.45	1	13	13	70	0.962	0.869	0.842	0.796	175.46	43.45	24	70	313	34.4	8.41
Q70IA6	MOB kinase activator 2 OS=Homo sapiens GN=MOB2 PE=1 SV=1 - [MOB2_HUMAN]	8.86	1	2	2	7	1.284	0.937	0.975	0.797	19.36	8.86	4	7	237	26.9	6.79
Q15036	Sorting nexin-17 OS=Homo sapiens GN=SNX17 PE=1 SV=1 - [SNX17_HUMAN]	8.09	2	4	4	9	1.160	0.859	0.944	0.797	20.09	8.09	6	9	470	52.9	7.46
Q8WXH0	Nesprin-2 OS=Homo sapiens GN=SYNE2 PE=1 SV=3 - [SYNE2_HUMAN]	2.63	2	11	19	32	0.708	0.851	0.763	0.797	57.78	2.63	21	32	6885	795.9	5.36
Q5T1J5	Putative coiled-coil-helix-coiled-coil-helix domain-containing protein CHCHD2P9, mitochondrial OS=Homo sapiens GN=CHCHD2P9 PE=5 SV=1 - [CHCH9_HUMAN]	8.61	2	1	1	3	0.633	0.626	0.870	0.797	9.81	8.61	2	3	151	15.5	9.89
Q9BXW6	Oxysterol-binding protein-related protein 1 OS=Homo sapiens GN=OSBPL1A PE=1 SV=2 - [OSBL1_HUMAN]	6.32	1	4	4	7	1.016	1.035	1.617	0.797	7.64	6.32	5	7	950	108.4	6.38

Q01518	Adenylyl cyclase-associated protein 1 OS=Homo sapiens GN=CAP1 PE=1 SV=5 - [CAP1_HUMAN]	49.05	1	22	24	355	1.044	1.148	1.297	0.797	1006.65	49.05	43	355	475	51.9	8.06
O00159	Unconventional myosin-Ic OS=Homo sapiens GN=MYO1C PE=1 SV=4 - [MYO1C_HUMAN]	49.67	1	51	51	312	1.235	1.034	1.093	0.797	839.43	49.67	84	312	1063	121.6	9.41
O14907	Tax1-binding protein 3 OS=Homo sapiens GN=TAX1BP3 PE=1 SV=2 - [TX1B3_HUMAN]	28.23	1	2	2	21	0.575	0.922	1.078	0.797	104.64	28.23	4	21	124	13.7	8.48
Q15124	Phosphoglucomutase-like protein 5 OS=Homo sapiens GN=PGM5 PE=1 SV=2 - [PGM5_HUMAN]	20.63	1	9	11	37	0.637	5.371	2.982	0.797	77.17	20.63	19	37	567	62.2	7.21
Q9H939	Proline-serine-threonine phosphatase-interacting protein 2 OS=Homo sapiens GN=PSTPIP2 PE=1 SV=4 - [PPIP2_HUMAN]	7.19	1	2	2	6	0.665	0.627	0.633	0.798	10.66	7.19	3	6	334	38.8	8.48
P09960	Leukotriene A-4 hydrolase OS=Homo sapiens GN=LTA4H PE=1 SV=2 - [LKHA4_HUMAN]	47.46	1	24	25	109	0.728	0.835	0.925	0.798	277.10	47.46	44	109	611	69.2	6.18
O95379	Tumor necrosis factor alpha-induced protein 8 OS=Homo sapiens GN=TNFAIP8 PE=1 SV=1 - [TFIP8_HUMAN	10.10	1	1	1	2	0.714	1.054	1.514	0.798	6.66	10.10	2	2	198	23.0	7.93
P32298	G protein-coupled receptor kinase 4 OS=Homo sapiens GN=GRK4 PE=1 SV=3 - [GRK4_HUMAN]	2.60	1	1	1	1	1.031	0.909	1.580	0.798	2.69	2.60	1	1	578	66.5	7.71
P30519	Heme oxygenase 2 OS=Homo sapiens GN=HMOX2 PE=1 SV=2 - [HMOX2_HUMAN]	35.13	1	9	9	51	0.776	0.889	0.947	0.798	140.91	35.13	15	51	316	36.0	5.41

Q9NP61	ADP-ribosylation factor GTPase-activating protein 3 OS=Homo sapiens GN=ARFGAP3 PE=1 SV=1 - [ARFG3_HUMAN]	30.81	1	17	17	52	0.841	0.916	0.825	0.798	95.55	30.81	24	52	516	56.9	7.36
Q8NAT2	Tudor domain-containing protein 5 OS=Homo sapiens GN=TDRD5 PE=1 SV=3 - [TDRD5_HUMAN]	1.63	1	2	2	9	0.662	1.087	1.300	0.798	17.85	1.63	3	9	981	109.7	8.02
Q969G5	Protein kinase C delta-binding protein OS=Homo sapiens GN=PRKCDBP PE=1 SV=3 - [PRDBP_HUMAN]	23.75	1	7	7	69	1.304	0.674	1.193	0.798	166.87	23.75	13	69	261	27.7	6.43
Q13129	Zinc finger protein Rlf OS=Homo sapiens GN=RLF PE=1 SV=2 - [RLF_HUMAN]	0.63	1	1	2	3	1.352	1.084	0.846	0.799	6.49	0.63	2	3	1914	217.8	6.77
Q2M2I8	AP2-associated protein kinase 1 OS=Homo sapiens GN=AAK1 PE=1 SV=3 - [AAK1_HUMAN]	19.46	1	14	15	47	0.996	0.895	1.041	0.799	130.77	19.46	24	47	961	103.8	6.60
Q96IZ0	PRKC apoptosis WT1 regulator protein OS=Homo sapiens GN=PAWR PE=1 SV=1 - [PAWR_HUMAN]	29.12	1	6	8	48	0.981	0.763	1.027	0.799	146.39	29.12	14	48	340	36.5	5.41
Q96A26	Protein FAM162A OS=Homo sapiens GN=FAM162A PE=1 SV=2 - [F162A_HUMAN]	25.32	1	3	3	12	1.078	1.441	0.784	0.799	30.65	25.32	4	12	154	17.3	9.77
P34896	Serine hydroxymethyltransferase, cytosolic OS=Homo sapiens GN=SHMT1 PE=1 SV=1 - [GLYC_HUMAN]	6.00	1	1	2	3	1.366	0.780	0.795	0.799	3.74	6.00	2	3	483	53.0	7.71

O60346	PH domain leucine-rich repeat-containing protein phosphatase 1 OS=Homo sapiens GN=PHLPP1 PE=1 SV=3 - [PHLP1_HUMAN]	1.16	1	1	3	4	0.992	1.111	1.247	0.800	0.00	1.16	3	4	1717	184.6	6.28
P63313	Thymosin beta-10 OS=Homo sapiens GN=TMSB10 PE=1 SV=2 - [TYB10_HUMAN]	52.27	1	6	9	160	1.227	1.068	0.783	0.800	377.78	52.27	14	160	44	5.0	5.36
Q92902	Hermansky-Pudlak syndrome 1 protein OS=Homo sapiens GN=HPS1 PE=1 SV=2 - [HPS1_HUMAN]	3.29	1	2	2	3	0.843	0.888	0.874	0.800	5.44	3.29	3	3	700	79.2	5.91
P11766	Alcohol dehydrogenase class-3 OS=Homo sapiens GN=ADH5 PE=1 SV=4 - [ADHX_HUMAN]	35.29	1	15	15	138	1.057	0.845	1.104	0.800	360.63	35.29	24	138	374	39.7	7.49
Q9UHD9	Ubiquilin-2 OS=Homo sapiens GN=UBQLN2 PE=1 SV=2 - [UBQL2_HUMAN]	20.03	1	4	8	70	0.763	1.414	0.904	0.800	162.64	20.03	13	70	624	65.7	5.22
Q9H6H4	Receptor expression-enhancing protein 4 OS=Homo sapiens GN=REEP4 PE=1 SV=1 - [REEP4_HUMAN]	8.95	1	1	1	2	0.811	2.020	2.375	0.800	10.27	8.95	1	2	257	29.4	9.73
Q9Y4E8	Ubiquitin carboxyl-terminal hydrolase 15 OS=Homo sapiens GN=USP15 PE=1 SV=3 - [UBP15_HUMAN]	5.91	1	7	7	24	0.994	0.917	0.981	0.800	50.51	5.91	11	24	981	112.3	5.22
Q5T2S8	Armadillo repeat-containing protein 4 OS=Homo sapiens GN=ARMC4 PE=1 SV=1 - [ARMC4_HUMA	1.15	1	1	1	1	0.596	0.722	0.467	0.800	1.94	1.15	1	1	1044	115.6	7.77

Q6PID6	Tetratricopeptide repeat protein 33 OS=Homo sapiens GN=TTC33 PE=1 SV=2 - [TTC33_HUMAN	4.20	1	1	1	1	0.845	1.001	1.064	0.800	2.52	4.20	1	1	262	29.4	5.44
O15118	Niemann-Pick C1 protein OS=Homo sapiens GN=NPC1 PE=1 SV=2 - [NPC1_HUMAN]	5.71	2	7	7	18	0.939	0.477	0.480	0.800	34.14	5.71	10	18	1278	142.1	5.36
Q8TF01	Arginine/serine-rich protein PNISR OS=Homo sapiens GN=PNISR PE=1 SV=2 - [PNISR_HUMAN]	1.61	1	1	1	2	1.289	1.226	1.817	0.801	4.16	1.61	1	2	805	92.5	10.02
P29218	Inositol monophosphatase 1 OS=Homo sapiens GN=IMPA1 PE=1 SV=1 - [IMPA1_HUMAN]	36.46	1	11	11	37	1.315	1.338	1.013	0.801	82.71	36.46	18	37	277	30.2	5.26
Q9HB90	Ras-related GTP-binding protein C OS=Homo sapiens GN=RRAGC PE=1 SV=1 - [RRAGC_HUMAN	9.02	2	4	4	8	0.724	0.643	0.696	0.801	12.90	9.02	6	8	399	44.2	5.10
Q96JG8	Melanoma-associated antigen D4 OS=Homo sapiens GN=MAGED4 PE=1 SV=3 - [MAGD4_HUMAN]	5.13	1	2	2	4	2.280	1.406	3.010	0.801	15.87	5.13	2	4	741	81.3	6.79
P63241	Eukaryotic translation initiation factor 5A-1 OS=Homo sapiens GN=EIF5A PE=1 SV=2 - [IF5A1_HUMAN]	72.08	2	7	15	389	0.845	0.766	0.804	0.801	1211.25	72.08	25	389	154	16.8	5.24
Q9BUL8	Programmed cell death protein 10 OS=Homo sapiens GN=PDCD10 PE=1 SV=1 - [PDC10_HUMAN	19.81	1	5	5	15	1.083	1.045	1.131	0.801	26.74	19.81	8	15	212	24.7	8.19
Q9BYP7	Serine/threonine-protein kinase WNK3 OS=Homo sapiens GN=WNK3 PE=1 SV=3 - [WNK3_HUMAN]	2.78	2	2	6	21	1.075	1.130	0.877	0.801	38.24	2.78	10	21	1800	198.3	6.07

Q9NZW5	MAGUK p55 subfamily member 6 OS=Homo sapiens GN=MPP6 PE=1 SV=2 - [MPP6_HUMAN]	24.26	1	12	13	47	0.642	1.016	0.801	0.801	102.71	24.26	21	47	540	61.1	6.18
Q5T4S7	E3 ubiquitin-protein ligase UBR4 OS=Homo sapiens GN=UBR4 PE=1 SV=1 - [UBR4_HUMAN]	14.12	1	67	68	213	0.897	0.748	0.877	0.802	467.99	14.12	105	213	5183	573.5	6.04
Q9BXK5	Bcl-2-like protein 13 OS=Homo sapiens GN=BCL2L13 PE=1 SV=1 - [B2L13_HUMAN]	21.86	1	9	9	38	0.987	0.623	0.933	0.802	63.81	21.86	16	38	485	52.7	4.44
P27816	Microtubule-associated protein 4 OS=Homo sapiens GN=MAP4 PE=1 SV=3 - [MAP4_HUMAN]	64.84	1	74	76	861	1.022	1.157	1.186	0.802	1983.99	64.84	128	861	1152	120.9	5.43
O75695	Protein XRP2 OS=Homo sapiens GN=RP2 PE=1 SV=4 - [XRP2_HUMAN]	18.00	1	6	6	9	1.187	0.727	0.734	0.802	16.74	18.00	7	9	350	39.6	5.12
Q969U7	Proteasome assembly chaperone 2 OS=Homo sapiens GN=PSMG2 PE=1 SV=1 - [PSMG2_HUMAN]	3.41	1	1	1	3	0.935	0.738	0.902	0.802	6.45	3.41	2	3	264	29.4	6.98
P27361	Mitogen-activated protein kinase 3 OS=Homo sapiens GN=MAPK3 PE=1 SV=4 - [MK03_HUMAN]	42.74	8	11	16	122	0.958	0.708	1.057	0.802	268.54	42.74	29	122	379	43.1	6.74
Q6PKG0	La-related protein 1 OS=Homo sapiens GN=LARP1 PE=1 SV=2 - [LARP1_HUMAN]	21.90	2	19	19	64	0.760	0.820	0.713	0.802	170.08	21.90	32	64	1096	123.4	8.82
Q9BUF5	Tubulin beta-6 chain OS=Homo sapiens GN=TUBB6 PE=1 SV=1 - [TBB6_HUMAN]	58.74	2	12	22	609	1.172	0.861	1.199	0.802	1548.07	58.74	39	609	446	49.8	4.88

Q9UKU9	Angiopoietin-related protein 2 OS=Homo sapiens GN=ANGPTL2 PE=2 SV=1 - [ANGL2_HUMAN]	7.10	1	3	3	10	0.973	0.837	0.683	0.803	14.99	7.10	3	10	493	57.1	7.53
Q6SZW1	Sterile alpha and TIR motif-containing protein 1 OS=Homo sapiens GN=SARM1 PE=1 SV=1 - [SARM1_HUMAN]	12.15	1	7	7	12	1.185	0.501	0.807	0.803	34.72	12.15	9	12	724	79.3	6.55
O60477	BMP/retinoic acid-inducible neural-specific protein 1 OS=Homo sapiens GN=BRINP1 PE=1 SV=2 - [BRNP1_HUMAN]	1.18	1	1	1	1	0.763	0.752	0.803	0.803	2.14	1.18	1	1	761	88.7	8.91
Q6P996	Pyridoxal-dependent decarboxylase domain-containing protein 1 OS=Homo sapiens GN=PDXDC1 PE=1 SV=2 - [PDXD1_HUMAN]	22.59	2	14	14	52	0.715	0.715	0.776	0.803	148.16	22.59	21	52	788	86.7	5.38
Q8WWI1	LIM domain only protein 7 OS=Homo sapiens GN=LMO7 PE=1 SV=3 - [LMO7_HUMAN]	37.55	1	59	59	296	0.845	0.688	1.004	0.804	682.26	37.55	101	296	1683	192.6	8.09
Q9BTY2	Plasma alpha-L-fucosidase OS=Homo sapiens GN=FUCA2 PE=1 SV=2 - [FUCO2_HUMAN]	15.85	1	8	8	18	0.603	0.920	0.709	0.804	48.64	15.85	13	18	467	54.0	6.25
O95239	Chromosome-associated kinesin KIF4A OS=Homo sapiens GN=KIF4A PE=1 SV=3 - [KIF4A_HUMAN]	6.90	1	6	7	12	1.070	0.927	0.897	0.804	32.30	6.90	7	12	1232	139.8	6.27
Q9BYK8	Helicase with zinc finger domain 2 OS=Homo sapiens GN=HELZ2 PE=1 SV=6 - [HELZ2_HUMAN]	3.21	1	5	7	13	1.574	0.926	0.966	0.804	16.47	3.21	7	13	2649	294.5	7.49

Q460N3	Poly [ADP-ribose] polymerase 15 OS=Homo sapiens GN=PARP15 PE=1 SV=2 - [PAR15_HUMAN]	1.77	1	1	1	1	1.437	1.094	1.406	0.804	1.95	1.77	1	1	678	74.5	8.82
Q969X5	Endoplasmic reticulum-Golgi intermediate compartment protein 1 OS=Homo sapiens GN=ERGIC1 PE=1 SV=1 - [ERGI1_HUMAN]	25.86	1	6	6	26	0.647	0.676	0.824	0.804	54.43	25.86	10	26	290	32.6	7.06
P53396	ATP-citrate synthase OS=Homo sapiens GN=ACLY PE=1 SV=3 - [ACLY_HUMAN]	40.87	1	41	41	193	0.949	0.659	0.994	0.804	506.39	40.87	69	193	1101	120.8	7.33
Q5T7M9	Protein FAM69A OS=Homo sapiens GN=FAM69A PE=2 SV=1 - [FA69A_HUMAN]	6.54	1	3	3	8	0.746	0.671	0.848	0.805	19.26	6.54	3	8	428	49.0	7.17
O60941	Dystrobrevin beta OS=Homo sapiens GN=DTNB PE=1 SV=1 - [DTNB_HUMAN]	5.74	1	1	4	8	0.801	1.428	1.248	0.805	13.28	5.74	5	8	627	71.3	7.91
O95822	Malonyl-CoA decarboxylase, mitochondrial OS=Homo sapiens GN=MLYCD PE=1 SV=3 - [DCMC_HUMAN]	12.37	1	4	5	9	0.836	0.922	1.144	0.805	16.85	12.37	7	9	493	55.0	8.95
P20073	Annexin A7 OS=Homo sapiens GN=ANXA7 PE=1 SV=3 - [ANXA7_HUMAN]	28.07	1	13	14	70	0.905	1.109	1.105	0.805	184.46	28.07	19	70	488	52.7	5.68
Q7Z6Z7	E3 ubiquitin-protein ligase HUWE1 OS=Homo sapiens GN=HUWE1 PE=1 SV=3 - [HUWE1_HUMAN]	11.07	1	46	46	126	0.864	1.044	1.127	0.805	301.89	11.07	69	126	4374	481.6	5.22
Q96T83	Sodium/hydrogen exchanger 7 OS=Homo sapiens GN=SLC9A7 PE=1 SV=1 - [SL9A7_HUMAN]	4.00	1	2	2	4	0.459	0.601	1.000	0.805	11.29	4.00	4	4	725	80.1	6.42

O43299	AP-5 complex subunit zeta-1 OS=Homo sapiens GN=AP5Z1 PE=1 SV=2 - [AP5Z1_HUMAN]	2.97	1	2	2	2	0.950	1.330	1.517	0.805	2.28	2.97	2	2	807	88.5	7.01
P84157	Matrix-remodeling-associated protein 7 OS=Homo sapiens GN=MXRA7 PE=1 SV=1 - [MXRA7_HUMAN]	27.94	1	5	5	49	0.965	0.711	0.763	0.805	96.14	27.94	8	49	204	21.5	4.26
P11171	Protein 4.1 OS=Homo sapiens GN=EPB41 PE=1 SV=4 - [41_HUMAN]	10.30	1	7	7	27	0.561	1.251	1.298	0.805	69.87	10.30	9	27	864	97.0	5.58
P04066	Tissue alpha-L-fucosidase OS=Homo sapiens GN=FUCA1 PE=1 SV=4 - [FUCO_HUMAN]	7.51	1	3	3	6	0.744	1.664	0.819	0.806	12.91	7.51	4	6	466	53.7	6.84
P09228	Cystatin-SA OS=Homo sapiens GN=CST2 PE=1 SV=1 - [CYTT_HUMAN]	10.64	1	1	1	1	1.071	1.049	1.719	0.806	3.61	10.64	1	1	141	16.4	4.93
Q6FI81	Anamorsin OS=Homo sapiens GN=CIAPIN1 PE=1 SV=2 - [CPIN1_HUMAN]	29.17	1	10	10	39	1.065	0.720	0.713	0.806	76.66	29.17	15	39	312	33.6	5.62
P42126	Enoyl-CoA delta isomerase 1, mitochondrial OS=Homo sapiens GN=ECI1 PE=1 SV=1 - [ECI1_HUMAN]	20.86	1	6	6	34	0.978	1.068	1.000	0.806	63.79	20.86	11	34	302	32.8	8.54
P35080	Profilin-2 OS=Homo sapiens GN=PFN2 PE=1 SV=3 - [PROF2_HUMAN]	36.43	1	6	6	45	1.001	0.790	0.978	0.806	113.38	36.43	11	45	140	15.0	6.99
Q9UBD5	Origin recognition complex subunit 3 OS=Homo sapiens GN=ORC3 PE=1 SV=1 - [ORC3_HUMAN]	3.94	1	1	3	3	0.991	1.064	0.863	0.806	2.11	3.94	3	3	711	82.2	7.61
Q9Y3E5	Peptidyl-tRNA hydrolase 2, mitochondrial OS=Homo sapiens GN=PTRH2 PE=1 SV=1 - [PTH2_HUMAN]	17.32	1	3	3	7	0.940	0.707	0.798	0.806	22.31	17.32	5	7	179	19.2	8.73

Q9UHG3	Prenylcysteine oxidase 1 OS=Homo sapiens GN=PCYOX1 PE=1 SV=3 - [PCYOX_HUMAN]	30.50	1	15	15	70	0.992	0.637	0.729	0.806	187.22	30.50	24	70	505	56.6	6.18
Q03393	6-pyruvoyl tetrahydrobiopterin synthase OS=Homo sapiens GN=PTS PE=1 SV=1 - [PTPS_HUMAN]	15.17	1	2	2	4	1.927	0.762	0.700	0.806	11.63	15.17	3	4	145	16.4	6.68
O43747	AP-1 complex subunit gamma-1 OS=Homo sapiens GN=AP1G1 PE=1 SV=5 - [AP1G1_HUMAN]	13.50	3	12	14	52	0.900	0.663	0.849	0.806	123.29	13.50	22	52	822	91.3	6.80
Q96PY6	Serine/threonine-protein kinase Nek1 OS=Homo sapiens GN=NEK1 PE=1 SV=2 - [NEK1_HUMAN]	2.86	1	4	4	6	0.932	1.214	0.985	0.807	8.39	2.86	6	6	1258	142.7	5.94
Q15327	Ankyrin repeat domain-containing protein 1 OS=Homo sapiens GN=ANKRD1 PE=1 SV=2 - [ANKR1_HUMAN]	6.27	1	1	2	2	1.114	0.274	0.359	0.807	1.82	6.27	2	2	319	36.2	7.50
Q96FQ6	Protein S100-A16 OS=Homo sapiens GN=S100A16 PE=1 SV=1 - [S10AG_HUMAN]	38.83	1	5	5	49	0.605	0.852	1.293	0.807	137.75	38.83	9	49	103	11.8	6.79
Q8NEU8	DCC-interacting protein 13-beta OS=Homo sapiens GN=APPL2 PE=1 SV=3 - [DP13B_HUMAN	27.41	1	14	14	25	0.618	0.944	0.920	0.807	70.89	27.41	20	25	664	74.4	4.94
A7E2Y1	Myosin-7B OS=Homo sapiens GN=MYH7B PE=1 SV=3 - [MYH7B_HUMAN]	2.99	4	2	8	70	0.866	1.086	0.886	0.807	123.95	2.99	9	70	1941	221.3	6.01
Q9P0J7	E3 ubiquitin-protein ligase KCMF1 OS=Homo sapiens GN=KCMF1 PE=1 SV=2 - [KCMF1_HUMAN]	10.24	1	3	3	9	1.256	0.867	1.060	0.808	21.12	10.24	4	9	381	41.9	5.66

P63092	Guanine nucleotide-binding protein G(s) subunit alpha isoforms short OS=Homo sapiens GN=GNAS PE=1 SV=1 - [GNAS2_HUMAN]	28.93	2	9	10	70	0.971	1.080	1.042	0.808	185.31	28.93	16	70	394	45.6	5.82
Q3T8J9	GON-4-like protein OS=Homo sapiens GN=GON4L PE=1 SV=1 - [GON4L_HUMAN]	0.94	2	3	3	4	1.061	1.032	0.958	0.808	9.07	0.94	3	4	2241	248.5	5.01
Q9Y5M8	Signal recognition particle receptor subunit beta OS=Homo sapiens GN=SRPRB PE=1 SV=3 - [SRPRB_HUMAN	49.45	1	13	13	39	0.645	0.749	0.840	0.808	96.53	49.45	18	39	271	29.7	9.04
Q04206	Transcription factor p65 OS=Homo sapiens GN=RELA PE=1 SV=2 - [TF65_HUMAN]	22.50	1	12	12	36	0.827	0.824	0.957	0.808	89.61	22.50	20	36	551	60.2	5.68
Q9H9G7	Protein argonaute-3 OS=Homo sapiens GN=AGO3 PE=1 SV=2 - [AGO3_HUMAN]	7.09	2	1	5	11	1.195	1.078	1.621	0.808	22.95	7.09	7	11	860	97.3	9.11
P78310	Coxsackievirus and adenovirus receptor OS=Homo sapiens GN=CXADR PE=1 SV=1 - [CXAR_HUMAN]	3.56	1	1	1	1	1.419	0.529	1.183	0.808	1.75	3.56	1	1	365	40.0	7.56
Q8IWE4	DCN1-like protein 3 OS=Homo sapiens GN=DCUN1D3 PE=1 SV=1 - [DCNL3_HUMAN]	8.55	1	2	2	3	0.805	0.739	1.003	0.808	11.83	8.55	2	3	304	34.3	5.12
Q9BXS0	Collagen alpha-1(XXV) chain OS=Homo sapiens GN=COL25A1 PE=1 SV=2 - [COPA1_HUMAN]	3.36	1	1	1	4	1.467	0.528	1.574	0.808	0.00	3.36	1	4	654	64.7	8.40
Q7Z2T5	TRMT1-like protein OS=Homo sapiens GN=TRMT1L PE=1 SV=2 - [TRM1L_HUMAN]	4.77	1	2	2	2		1.129		0.808	7.78	4.77	2	2	733	81.7	7.88

P56385	ATP synthase subunit e, mitochondrial OS=Homo sapiens GN=ATP5I PE=1 SV=2 - [ATP5I_HUMAN]	31.88	1	2	2	8	1.171	1.125	0.757	0.809	20.05	31.88	4	8	69	7.9	9.35
P08240	Signal recognition particle receptor subunit alpha OS=Homo sapiens GN=SRPR PE=1 SV=2 - [SRPR_HUMAN]	41.54	2	26	26	83	0.766	0.971	0.913	0.809	203.55	41.54	40	83	638	69.8	8.95
O15143	Actin-related protein 2/3 complex subunit 1B OS=Homo sapiens GN=ARPC1B PE=1 SV=3 - [ARC1B_HUMAN]	39.52	1	12	12	150	0.985	0.979	1.130	0.809	367.25	39.52	20	150	372	40.9	8.35
P05154	Plasma serine protease inhibitor OS=Homo sapiens GN=SERPINA5 PE=1 SV=3 - [IPSP_HUMAN]	4.43	1	1	2	5	0.933	0.437	1.284	0.809	5.69	4.43	2	5	406	45.6	9.26
O15484	Calpain-5 OS=Homo sapiens GN=CAPN5 PE=1 SV=2 - [CAN5_HUMAN]	5.63	1	4	4	15	0.899	1.233	1.010	0.809	25.51	5.63	6	15	640	73.1	7.64
Q9UHD8	Septin-9 OS=Homo sapiens GN=SEPT9 PE=1 SV=2 - [SEPT9_HUMAN]	55.29	1	30	31	289	0.886	1.213	1.459	0.809	768.28	55.29	52	289	586	65.4	8.97
P0CG29	Glutathione S-transferase theta-2 OS=Homo sapiens GN=GSTT2 PE=1 SV=1 - [GST2_HUMAN]	29.92	2	6	7	18	0.848	0.798	1.014	0.809	40.40	29.92	11	18	244	27.5	7.02
Q00013	55 kDa erythrocyte membrane protein OS=Homo sapiens GN=MPP1 PE=1 SV=2 - [EM55_HUMAN]	12.23	1	5	5	16	0.780	1.171	0.980	0.809	34.78	12.23	7	16	466	52.3	7.37
Q13867	Bleomycin hydrolase OS=Homo sapiens GN=BLMH PE=1 SV=1 - [BLMH_HUMAN]	20.00	1	8	8	28	0.701	0.983	0.731	0.810	50.01	20.00	14	28	455	52.5	6.27

Q9ULU4	Protein kinase C-binding protein 1 OS=Homo sapiens GN=ZMYND8 PE=1 SV=2 - [PKCB1_HUMAN	2.36	1	2	2	4	1.515	0.826	0.665	0.810	12.18	2.36	2	4	1186	131.6	7.20
P0C7P0	CDGSH iron-sulfur domain-containing protein 3, mitochondrial OS=Homo sapiens GN=CISD3 PE=1 SV=1 - [CISD3_HUMAN]	4.72	1	1	1	1	0.676	0.814	0.668	0.810	1.99	4.72	1	1	127	14.2	10.55
O14980	Exportin-1 OS=Homo sapiens GN=XPO1 PE=1 SV=1 - [XPO1_HUMAN]	19.79	1	18	18	91	0.837	0.955	0.714	0.810	229.14	19.79	31	91	1071	123.3	6.06
Q9Y6A4	Cilia- and flagella-associated protein 20 OS=Homo sapiens GN=CFAP20 PE=1 SV=1 - [CFA20_HUMAN	8.29	1	2	2	2	0.906	0.766	0.616	0.810	2.39	8.29	2	2	193	22.8	9.76
Q9HBH5	Retinol dehydrogenase 14 OS=Homo sapiens GN=RDH14 PE=1 SV=1 - [RDH14_HUMAN]	30.95	1	8	8	30	0.868	1.274	1.088	0.810	82.66	30.95	13	30	336	36.8	8.79
O14613	Cdc42 effector protein 2 OS=Homo sapiens GN=CDC42EP2 PE=1 SV=1 - [BORG1_HUMA N]	32.86	1	4	4	7	1.003	0.718	1.196	0.810	20.68	32.86	6	7	210	22.5	5.12
P50851	Lipopolysaccharide-responsive and beige-like anchor protein OS=Homo sapiens GN=LRBA PE=1 SV=4 - [LRBA_HUMAN]	5.87	1	15	16	35	0.892	0.912	1.045	0.810	72.40	5.87	25	35	2863	318.9	5.60
P15311	Ezrin OS=Homo sapiens GN=EZR PE=1 SV=4 - [EZRI_HUMAN]	43.69	1	18	33	301	1.019	1.999	1.372	0.810	746.54	43.69	51	301	586	69.4	6.27
Q9UGP8	Translocation protein SEC63 homolog OS=Homo sapiens GN=SEC63 PE=1 SV=2 - [SEC63_HUMAN]	30.39	1	20	20	67	0.839	0.900	0.811	0.811	176.69	30.39	28	67	760	87.9	5.31

P61254	60S ribosomal protein L26 OS=Homo sapiens GN=RPL26 PE=1 SV=1 - [RL26_HUMAN]	49.66	2	2	13	105	0.733	0.843	0.662	0.811	191.85	49.66	22	105	145	17.2	10.55
P61163	Alpha-centractin OS=Homo sapiens GN=ACTR1A PE=1 SV=1 - [ACTZ_HUMAN]	39.63	2	6	12	199	1.106	1.044	1.365	0.811	461.09	39.63	21	199	376	42.6	6.64
Q9NZL4	Hsp70-binding protein 1 OS=Homo sapiens GN=HSPBP1 PE=1 SV=1 - [HPBP1_HUMAN]	12.98	1	5	5	13	0.713	0.575	0.785	0.811	31.54	12.98	7	13	362	39.4	5.21
Q13332	Receptor-type tyrosine-protein phosphatase S OS=Homo sapiens GN=PTPRS PE=1 SV=3 - [PTPRS_HUMAN	4.21	2	4	5	6	0.796	0.869	0.652	0.811	16.78	4.21	6	6	1948	216.9	6.46
P40855	Peroxisomal biogenesis factor 19 OS=Homo sapiens GN=PEX19 PE=1 SV=1 - [PEX19_HUMAN]	41.47	1	10	10	67	0.853	0.904	0.944	0.811	190.38	41.47	17	67	299	32.8	4.34
O14933	Ubiquitin/ISG15-conjugating enzyme E2 L6 OS=Homo sapiens GN=UBE2L6 PE=1 SV=4 - [UB2L6_HUMAN]	41.83	1	5	6	31	1.839	1.032	1.102	0.811	76.24	41.83	10	31	153	17.8	7.88
P04216	Thy-1 membrane glycoprotein OS=Homo sapiens GN=THY1 PE=1 SV=2 - [THY1_HUMAN]	9.32	1	1	1	5	0.468	0.559	0.587	0.811	15.53	9.32	2	5	161	17.9	8.73
Q96DR7	Rho guanine nucleotide exchange factor 26 OS=Homo sapiens GN=ARHGEF26 PE=1 SV=4 - [ARHGQ_HUMA	1.26	1	1	1	1	0.730	0.744	0.500	0.811	2.55	1.26	1	1	871	97.3	8.95
Q96DP5	Methionyl-tRNA formyltransferase, mitochondrial OS=Homo sapiens GN=MTFMT PE=1 SV=2 - [FMT_HUMAN]	7.71	1	3	3	4	1.124	1.226	0.926	0.811	6.47	7.71	4	4	389	43.8	9.66

Q9Y3Q8	TSC22 domain family protein 4 OS=Homo sapiens GN=TSC22D4 PE=1 SV=2 - [T22D4_HUMAN]	30.63	1	8	10	35	0.845	0.916	1.385	0.811	78.80	30.63	16	35	395	41.0	7.21
O15400	Syntaxin-7 OS=Homo sapiens GN=STX7 PE=1 SV=4 - [STX7_HUMAN]	30.27	1	7	7	40	0.887	1.204	1.159	0.811	107.91	30.27	13	40	261	29.8	5.55
O15013	Rho guanine nucleotide exchange factor 10 OS=Homo sapiens GN=ARHGEF10 PE=1 SV=4 - [ARHGA_HUMAN	3.58	1	6	6	13	0.917	1.337	1.009	0.811	26.78	3.58	10	13	1369	151.5	5.68
P50895	Basal cell adhesion molecule OS=Homo sapiens GN=BCAM PE=1 SV=2 - [BCAM_HUMAN]	26.59	1	12	12	39	1.581	1.580	1.974	0.811	94.13	26.59	21	39	628	67.4	5.81
Q9NYG2	Palmitoyltransferase ZDHHC3 OS=Homo sapiens GN=ZDHHC3 PE=1 SV=2 - [ZDHC3_HUMAN]	5.35	1	1	1	1	0.750	0.525	0.757	0.811	3.28	5.35	1	1	299	34.1	8.18
Q13418	Integrin-linked protein kinase OS=Homo sapiens GN=ILK PE=1 SV=2 - [ILK_HUMAN]	33.19	1	16	16	126	0.935	1.135	1.344	0.812	306.97	33.19	29	126	452	51.4	8.07
Q5JTJ3	Cytochrome c oxidase assembly factor 6 homolog OS=Homo sapiens GN=COA6 PE=1 SV=1 - [COA6_HUMAN]	21.60	1	2	2	5	0.910	1.004	0.855	0.812	18.52	21.60	3	5	125	14.1	8.25
Q92791	Synaptonemal complex protein SC65 OS=Homo sapiens GN=LEPREL4 PE=1 SV=1 - [SC65_HUMAN]	13.73	1	6	6	37	0.700	0.885	0.778	0.812	84.28	13.73	11	37	437	50.3	4.77
E0CX11	Uncharacterized protein C7orf73 OS=Homo sapiens GN=C7orf73 PE=3 SV=1 - [CG073_HUMAN]	14.89	2	1	2	2	1.413	0.833	0.370	0.812	0.00	14.89	2	2	47	5.3	9.13

P47755	F-actin-capping protein subunit alpha-2 OS=Homo sapiens GN=CAPZA2 PE=1 SV=3 - [CAZA2_HUMAN]	59.44	1	9	12	178	0.894	1.057	1.126	0.812	394.49	59.44	19	178	286	32.9	5.85
Q99835	Smoothened homolog OS=Homo sapiens GN=SMO PE=1 SV=1 - [SMO_HUMAN]	0.89	1	1	1	2	0.842	0.766	0.982	0.812	4.74	0.89	1	2	787	86.3	8.34
Q9UMX1	Suppressor of fused homolog OS=Homo sapiens GN=SUFU PE=1 SV=2 - [SUFU_HUMAN]	3.51	1	1	1	2	1.337	1.054	0.820	0.812	3.29	3.51	2	2	484	53.9	5.33
Q99426	Tubulin-folding cofactor B OS=Homo sapiens GN=TBCB PE=1 SV=2 - [TBCB_HUMAN]	20.08	1	5	5	36	0.883	1.245	1.353	0.813	75.97	20.08	8	36	244	27.3	5.15
Q92673	Sortilin-related receptor OS=Homo sapiens GN=SORL1 PE=1 SV=2 - [SORL_HUMAN]	0.99	1	1	1	1	0.748	1.391	1.295	0.813	0.00	0.99	1	1	2214	248.3	5.55
Q01082	Spectrin beta chain, non-erythrocytic 1 OS=Homo sapiens GN=SPTBN1 PE=1 SV=2 - [SPTB2_HUMAN]	64.55	2	130	149	1152	1.185	1.008	0.861	0.813	2844.78	64.55	256	1152	2364	274.4	5.57
Q13740	CD166 antigen OS=Homo sapiens GN=ALCAM PE=1 SV=2 - [CD166_HUMAN]	28.82	1	18	18	76	0.857	0.899	0.877	0.814	173.90	28.82	28	76	583	65.1	6.25
Q96CW1	AP-2 complex subunit mu OS=Homo sapiens GN=AP2M1 PE=1 SV=2 - [AP2M1_HUMAN]	48.74	1	22	22	104	0.953	0.733	0.816	0.814	273.62	48.74	37	104	435	49.6	9.54
Q8NEC5	Cation channel sperm-associated protein 1 OS=Homo sapiens GN=CATSPER1 PE=1 SV=3 - [CTSR1_HUMAN]	1.54	1	1	1	1	1.002	1.077	1.494	0.814	0.00	1.54	1	1	780	90.0	7.71

Q96CV9	Optineurin OS=Homo sapiens GN=OPTN PE=1 SV=2 - [OPTN_HUMAN]	48.87	1	27	29	130	0.747	0.957	1.107	0.814	306.68	48.87	48	130	577	65.9	5.24
Q9HC62	Sentrin-specific protease 2 OS=Homo sapiens GN=SENP2 PE=1 SV=3 - [SENP2_HUMAN]	1.70	1	1	1	1	0.820	1.528	1.495	0.814	0.00	1.70	1	1	589	67.8	9.48
Q68CQ1	Maestro heat-like repeat-containing protein family member 7 OS=Homo sapiens GN=MROH7 PE=2 SV=4 - [MROH7_HUMA N]	1.13	1	1	1	4	1.142	0.792	0.645	0.814	0.00	1.13	1	4	1323	145.6	6.96
P40424	Pre-B-cell leukemia transcription factor 1 OS=Homo sapiens GN=PBX1 PE=1 SV=1 - [PBX1_HUMAN]	17.44	2	4	5	12	0.875	2.566	0.892	0.814	33.78	17.44	6	12	430	46.6	7.03
Q16881	Thioredoxin reductase 1, cytoplasmic OS=Homo sapiens GN=TXNRD1 PE=1 SV=3 - [TRXR1_HUMAN]	43.30	1	26	26	241	1.006	0.592	1.151	0.814	553.00	43.30	43	241	649	70.9	7.39
P35711	Transcription factor SOX-5 OS=Homo sapiens GN=SOX5 PE=1 SV=3 - [SOX5_HUMAN]	2.49	2	1	2	2	0.929	0.510	0.696	0.814	5.03	2.49	2	2	763	84.0	6.60
O95747	Serine/threonine-protein kinase OSR1 OS=Homo sapiens GN=OXSR1 PE=1 SV=1 - [OXSR1_HUMA N]	37.00	1	13	16	58	1.196	0.899	1.009	0.814	148.25	37.00	26	58	527	58.0	6.43
Q9NS69	Mitochondrial import receptor subunit TOM22 homolog OS=Homo sapiens GN=TOMM22 PE=1 SV=3 - [TOM22_HUMA	8.45	1	1	1	9	0.881	0.608	0.618	0.814	12.36	8.45	2	9	142	15.5	4.34
P61927	60S ribosomal protein L37 OS=Homo sapiens GN=RPL37 PE=1 SV=2 - [RL37_HUMAN]	26.80	1	5	5	13	0.607	0.768	0.634	0.814	21.66	26.80	6	13	97	11.1	11.74

O43169	Cytochrome b5 type B OS=Homo sapiens GN=CYB5B PE=1 SV=2 - [CYB5B_HUMAN]	35.62	1	3	3	9	0.900	0.780	0.759	0.814	28.57	35.62	4	9	146	16.3	4.97
Q13907	Isopentenyl-diphosphate Delta-isomerase 1 OS=Homo sapiens GN=IDI1 PE=1 SV=2 - [IDI1_HUMAN]	24.23	2	4	5	14	1.281	0.784	1.246	0.815	40.13	24.23	6	14	227	26.3	6.34
P11233	Ras-related protein Ral-A OS=Homo sapiens GN=RALA PE=1 SV=1 - [RALA_HUMAN]	50.00	1	4	7	36	1.174	0.846	0.825	0.815	89.74	50.00	12	36	206	23.6	7.11
Q99538	Legumain OS=Homo sapiens GN=LGMN PE=1 SV=1 - [LGMN_HUMAN]	12.93	1	5	5	61	0.464	0.359	0.700	0.815	158.37	12.93	8	61	433	49.4	6.55
Q8N1F8	Serine/threonine-protein kinase 11-interacting protein OS=Homo sapiens GN=STK11IP PE=1 SV=3 - [S11IP_HUMAN	1.46	1	1	2	6	0.687	0.807	0.766	0.815	11.21	1.46	3	6	1099	121.3	5.39
Q13508	Ecto-ADP-ribosyltransferase 3 OS=Homo sapiens GN=ART3 PE=1 SV=2 - [NAR3_HUMAN]	4.37	3	1	2	3	1.807	1.012	1.418	0.815	4.64	4.37	2	3	389	43.9	6.06
O15084	Serine/threonine-protein phosphatase 6 regulatory ankyrin repeat subunit A OS=Homo sapiens GN=ANKRD28 PE=1 SV=5 - [ANR28_HUMAN]	3.23	1	2	2	4	0.904	1.416	0.739	0.815	14.76	3.23	3	4	1053	112.9	6.25
Q96B36	Proline-rich AKT1 substrate 1 OS=Homo sapiens GN=AKT1S1 PE=1 SV=1 - [AKTS1_HUMAN]	12.89	1	3	3	18	0.836	0.907	0.993	0.815	38.90	12.89	6	18	256	27.4	4.75
P52565	Rho GDP-dissociation inhibitor 1 OS=Homo sapiens GN=ARHGDIA PE=1 SV=3 - [GDIR1_HUMAN]	47.55	1	11	11	183	1.239	1.120	1.180	0.815	540.96	47.55	18	183	204	23.2	5.11

Q93100	Phosphorylase b kinase regulatory subunit beta OS=Homo sapiens GN=PHKB PE=1 SV=3 - [KPBB_HUMAN]	7.69	3	8	9	15	0.773	1.184	1.289	0.815	27.65	7.69	13	15	1093	124.8	6.95
Q8TEL6	Short transient receptor potential channel 4-associated protein OS=Homo sapiens GN=TRPC4AP PE=1 SV=2 - [TP4AP_HUMAN	1.25	1	1	1	1	0.771	1.554	2.352	0.815	2.89	1.25	1	1	797	90.8	7.61
P07384	Calpain-1 catalytic subunit OS=Homo sapiens GN=CAPN1 PE=1 SV=1 - [CAN1_HUMAN]	37.25	1	24	24	93	1.049	0.910	1.145	0.815	232.13	37.25	37	93	714	81.8	5.67
P61970	Nuclear transport factor 2 OS=Homo sapiens GN=NUTF2 PE=1 SV=1 - [NTF2_HUMAN]	61.42	1	5	6	41	1.044	1.180	1.016	0.816	83.66	61.42	8	41	127	14.5	5.38
Q9NXH9	tRNA (guanine(26)-N(2))-dimethyltransferase OS=Homo sapiens GN=TRMT1 PE=1 SV=1 - [TRM1_HUMAN]	6.83	2	3	4	10	0.591	0.845	0.704	0.816	26.86	6.83	5	10	659	72.2	7.64
O60271	C-Jun-amino-terminal kinase-interacting protein 4 OS=Homo sapiens GN=SPAG9 PE=1 SV=4 - [JIP4_HUMAN]	30.89	2	32	34	115	0.863	1.038	0.931	0.816	325.10	30.89	55	115	1321	146.1	5.15
Q9Y5J6	Mitochondrial import inner membrane translocase subunit Tim10 B OS=Homo sapiens GN=TIMM10B PE=1 SV=1 - [T10B_HUMAN]	7.77	1	1	1	1	0.768	0.503	0.630	0.816	2.06	7.77	1	1	103	11.6	7.43
Q9NUL3	Double-stranded RNA-binding protein Staufen homolog 2 OS=Homo sapiens GN=STAU2 PE=1 SV=1 - [STAU2_HUMAN]	16.67	1	7	8	26	1.166	1.233	1.192	0.816	52.32	16.67	14	26	570	62.6	9.61

P42785	Lysosomal Pro-X carboxypeptidase OS=Homo sapiens GN=PRCP PE=1 SV=1 - [PCP_HUMAN]	17.54	1	7	7	23	0.881	1.197	1.099	0.817	49.14	17.54	12	23	496	55.8	7.21
Q9Y5R8	Trafficking protein particle complex subunit 1 OS=Homo sapiens GN=TRAPPC1 PE=1 SV=1 - [TPPC1_HUMAN]	14.48	1	2	2	4	1.164	1.210	1.158	0.817	4.83	14.48	3	4	145	16.8	9.16
P07437	Tubulin beta chain OS=Homo sapiens GN=TUBB PE=1 SV=2 - [TBB5_HUMAN]	69.59	1	4	22	1365	1.125	1.145	1.152	0.817	3114.02	69.59	40	1365	444	49.6	4.89
Q96BY6	Dedicator of cytokinesis protein 10 OS=Homo sapiens GN=DOCK10 PE=1 SV=3 - [DOC10_HUMAN]	1.83	1	3	3	3	0.838	1.146	0.837	0.817	2.21	1.83	3	3	2186	249.4	7.14
P07339	Cathepsin D OS=Homo sapiens GN=CTSD PE=1 SV=1 - [CATD_HUMAN]	43.93	1	16	16	102	0.935	1.071	0.990	0.817	230.60	43.93	25	102	412	44.5	6.54
P62987	Ubiquitin-60S ribosomal protein L40 OS=Homo sapiens GN=UBA52 PE=1 SV=2 - [RL40_HUMAN]	64.84	3	2	15	454	1.139	1.517	1.063	0.817	1059.83	64.84	25	454	128	14.7	9.83
Q15628	Tumor necrosis factor receptor type 1-associated DEATH domain protein OS=Homo sapiens GN=TRADD PE=1 SV=2 - [TRADD_HUMAN]	26.28	1	7	7	14	1.072	1.041	1.157	0.817	42.43	26.28	10	14	312	34.2	6.27
Q9Y2H0	Disks large-associated protein 4 OS=Homo sapiens GN=DLGAP4 PE=1 SV=3 - [DLGP4_HUMAN]	7.86	1	8	8	17	0.939	0.852	0.867	0.817	35.45	7.86	12	17	992	107.9	7.08

P15291	Beta-1,4-galactosyltransferase 1 OS=Homo sapiens GN=B4GALT1 PE=1 SV=5 - [B4GT1_HUMAN]	5.03	1	2	2	4	0.989	0.670	0.860	0.817	11.18	5.03	3	4	398	43.9	8.65
O43813	LanC-like protein 1 OS=Homo sapiens GN=LANCL1 PE=1 SV=1 - [LANC1_HUMAN]	7.77	1	3	3	16	0.902	1.094	1.167	0.817	34.11	7.77	6	16	399	45.3	7.75
Q68CQ7	Glycosyltransferase 8 domain-containing protein 1 OS=Homo sapiens GN=GLT8D1 PE=1 SV=2 - [GL8D1_HUMAN]	14.56	1	5	5	8	0.649	1.234	0.924	0.817	23.70	14.56	6	8	371	41.9	9.35
Q5TEJ8	Protein THEMIS2 OS=Homo sapiens GN=THEMIS2 PE=2 SV=1 - [THMS2_HUMAN]	1.87	1	1	1	1	0.602	0.739	0.597	0.817	2.84	1.87	1	1	643	72.0	6.10
Q5HYK9	Zinc finger protein 667 OS=Homo sapiens GN=ZNF667 PE=2 SV=2 - [ZN667_HUMAN]	2.62	2	2	2	2	0.666	0.806	0.871	0.817	0.00	2.62	2	2	610	70.1	9.73
Q5TGP6	Maestro heat-like repeat-containing protein family member 9 OS=Homo sapiens GN=MROH9 PE=2 SV=1 - [MROH9_HUMAN]	2.97	1	1	1	1	1.425	0.651	0.846	0.817	2.30	2.97	1	1	573	65.0	6.34
Q9Y6J0	Calcineurin-binding protein cabin-1 OS=Homo sapiens GN=CABIN1 PE=1 SV=1 - [CABIN_HUMAN]	0.95	1	1	1	4	0.594	1.864	0.970	0.817	12.29	0.95	1	4	2220	246.2	6.02
O00487	26S proteasome non-ATPase regulatory subunit 14 OS=Homo sapiens GN=PSMD14 PE=1 SV=1 - [PSDE_HUMAN]	25.16	1	8	8	24	0.911	0.908	0.826	0.817	52.03	25.16	13	24	310	34.6	6.52

Q9P1Z0	Zinc finger and BTB domain-containing protein 4 OS=Homo sapiens GN=ZBTB4 PE=1 SV=3 - [ZBTB4_HUMAN]	1.68	1	1	1	1	0.538	2.221	1.554	0.817	3.25	1.68	1	1	1013	105.0	8.37
Q15056	Eukaryotic translation initiation factor 4H OS=Homo sapiens GN=EIF4H PE=1 SV=5 - [IF4H_HUMAN]	48.39	1	13	13	119	0.824	1.187	0.981	0.818	292.81	48.39	18	119	248	27.4	7.23
P30044	Peroxiredoxin-5, mitochondrial OS=Homo sapiens GN=PRDX5 PE=1 SV=4 - [PRDX5_HUMAN	37.38	1	9	9	230	0.977	0.836	0.907	0.818	579.70	37.38	17	230	214	22.1	8.70
Q9Y5S1	Transient receptor potential cation channel subfamily V member 2 OS=Homo sapiens GN=TRPV2 PE=1 SV=1 - [TRPV2_HUMAN]	14.53	1	11	11	40	1.901	0.902	1.016	0.818	102.83	14.53	18	40	764	85.9	5.83
Q9UEY8	Gamma-adducin OS=Homo sapiens GN=ADD3 PE=1 SV=1 - [ADDG_HUMAN]	16.57	1	11	11	68	0.952	1.080	1.007	0.818	140.93	16.57	18	68	706	79.1	6.32
P56192	Methionine--tRNA ligase, cytoplasmic OS=Homo sapiens GN=MARS PE=1 SV=2 - [SYMC_HUMAN]	27.22	1	21	21	85	0.813	0.831	0.810	0.818	205.38	27.22	38	85	900	101.1	6.16
P35240	Merlin OS=Homo sapiens GN=NF2 PE=1 SV=1 - [MERL_HUMAN]	12.77	1	7	7	16	0.932	1.133	0.862	0.818	33.41	12.77	11	16	595	69.6	6.47
P12268	Inosine-5'-monophosphate dehydrogenase 2 OS=Homo sapiens GN=IMPDH2 PE=1 SV=2 - [IMDH2_HUMAN]	44.94	1	18	22	160	0.785	0.922	0.761	0.818	396.80	44.94	36	160	514	55.8	6.90
P06733	Alpha-enolase OS=Homo sapiens GN=ENO1 PE=1 SV=2 - [ENOA_HUMAN]	83.18	1	40	45	1638	1.101	1.099	0.890	0.819	4393.01	83.18	69	1638	434	47.1	7.39

O94875	Sorbin and SH3 domain-containing protein 2 OS=Homo sapiens GN=SORBS2 PE=1 SV=3 - [SRBS2_HUMAN]	13.64	1	11	12	33	8.338	0.798	1.294	0.819	69.78	13.64	20	33	1100	124.0	8.31
Q13509	Tubulin beta-3 chain OS=Homo sapiens GN=TUBB3 PE=1 SV=2 - [TBB3_HUMAN]	47.11	2	4	18	912	1.184	1.653	1.403	0.819	2002.06	47.11	34	912	450	50.4	4.93
Q9UKY7	Protein CDV3 homolog OS=Homo sapiens GN=CDV3 PE=1 SV=1 - [CDV3_HUMAN]	45.74	1	12	12	74	0.786	1.091	0.985	0.819	197.86	45.74	17	74	258	27.3	6.40
Q9H269	Vacuolar protein sorting-associated protein 16 homolog OS=Homo sapiens GN=VPS16 PE=1 SV=2 - [VPS16_HUMAN]	16.21	1	11	11	20	1.121	0.939	0.958	0.819	43.89	16.21	16	20	839	94.6	6.77
Q9H993	UPF0364 protein C6orf211 OS=Homo sapiens GN=C6orf211 PE=1 SV=1 - [CF211_HUMAN]	13.38	1	8	8	17	0.854	0.835	0.739	0.819	32.37	13.38	12	17	441	51.1	5.76
Q9P265	Disco-interacting protein 2 homolog B OS=Homo sapiens GN=DIP2B PE=1 SV=3 - [DIP2B_HUMAN]	7.11	1	8	8	30	0.958	0.709	0.827	0.819	53.68	7.11	12	30	1576	171.4	8.09
P41587	Vasoactive intestinal polypeptide receptor 2 OS=Homo sapiens GN=VIPR2 PE=1 SV=2 - [VIPR2_HUMAN]	2.51	1	1	1	1	2.703	0.760	0.829	0.819	4.05	2.51	1	1	438	49.4	7.94
P08172	Muscarinic acetylcholine receptor M2 OS=Homo sapiens GN=CHRM2 PE=1 SV=1 - [ACM2_HUMAN]	3.86	1	2	2	4	1.136	0.442	0.389	0.819	13.44	3.86	3	4	466	51.7	8.85

P56962	Syntaxin-17 OS=Homo sapiens GN=STX17 PE=1 SV=2 - [STX17_HUMAN]	8.28	1	3	3	5	1.315	1.063	0.751	0.819	8.95	8.28	4	5	302	33.4	6.57
P14868	Aspartate--tRNA ligase, cytoplasmic OS=Homo sapiens GN=DARS PE=1 SV=2 - [SYDC_HUMAN]	57.09	1	27	27	147	0.871	0.871	0.839	0.820	357.62	57.09	42	147	501	57.1	6.55
P41212	Transcription factor ETV6 OS=Homo sapiens GN=ETV6 PE=1 SV=1 - [ETV6_HUMAN]	3.76	1	2	2	3			1.331	0.820	3.92	3.76	3	3	452	53.0	7.43
Q14643	Inositol 1,4,5-trisphosphate receptor type 1 OS=Homo sapiens GN=ITPR1 PE=1 SV=3 - [ITPR1_HUMAN]	7.58	1	13	21	62	0.903	0.731	0.805	0.820	132.41	7.58	32	62	2758	313.7	6.04
Q6VY07	Phosphofurin acidic cluster sorting protein 1 OS=Homo sapiens GN=PACS1 PE=1 SV=2 - [PACS1_HUMAN]	13.81	1	10	10	21	0.858	0.943	1.096	0.820	63.44	13.81	13	21	963	104.8	7.74
P61764	Syntaxin-binding protein 1 OS=Homo sapiens GN=STXBP1 PE=1 SV=1 - [STXB1_HUMAN]	31.82	1	16	17	58	1.243	1.003	0.980	0.820	115.83	31.82	27	58	594	67.5	6.96
A6NDB9	Paralemmin-3 OS=Homo sapiens GN=PALM3 PE=1 SV=2 - [PALM3_HUMAN]	4.46	1	1	3	20	1.051	1.375	0.669	0.820	42.59	4.46	4	20	673	71.7	4.53
O15144	Actin-related protein 2/3 complex subunit 2 OS=Homo sapiens GN=ARPC2 PE=1 SV=1 - [ARPC2_HUMAN]	53.33	1	19	19	162	0.945	0.837	0.940	0.820	400.18	53.33	31	162	300	34.3	7.36
Q9P232	Contactin-3 OS=Homo sapiens GN=CNTN3 PE=1 SV=3 - [CNTN3_HUMAN]	0.88	1	1	1	1	0.693	1.084	0.763	0.821	0.00	0.88	1	1	1028	112.8	6.30

Q14766	Latent-transforming growth factor beta-binding protein 1 OS=Homo sapiens GN=LTBP1 PE=1 SV=4 - [LTBP1_HUMAN]	2.96	1	3	3	5	0.993	1.554	1.206	0.821	12.31	2.96	4	5	1721	186.7	5.96
P63167	Dynein light chain 1, cytoplasmic OS=Homo sapiens GN=DYNLL1 PE=1 SV=1 - [DYL1_HUMAN]	44.94	1	5	6	26	1.068	1.021	0.925	0.821	48.32	44.94	9	26	89	10.4	7.40
P09211	Glutathione S-transferase P OS=Homo sapiens GN=GSTP1 PE=1 SV=2 - [GSTP1_HUMAN]	64.29	1	10	10	400	1.001	0.912	0.976	0.821	1028.01	64.29	17	400	210	23.3	5.64
P04899	Guanine nucleotide-binding protein G(i) subunit alpha-2 OS=Homo sapiens GN=GNAI2 PE=1 SV=3 - [GNAI2_HUMAN]	57.18	4	10	19	125	0.921	0.859	0.916	0.821	327.89	57.18	32	125	355	40.4	5.54
Q5VT97	Rho GTPase-activating protein SYDE2 OS=Homo sapiens GN=SYDE2 PE=1 SV=2 - [SYDE2_HUMAN]	2.51	3	2	3	16	0.966	0.538	0.890	0.821	32.07	2.51	4	16	1194	133.1	8.57
Q14789	Golgin subfamily B member 1 OS=Homo sapiens GN=GOLGB1 PE=1 SV=2 - [GOGB1_HUMAN]	25.25	2	75	81	227	0.803	1.040	1.061	0.821	547.11	25.25	120	227	3259	375.8	5.00
A0AVI2	Fer-1-like protein 5 OS=Homo sapiens GN=FER1L5 PE=2 SV=2 - [FR1L5_HUMAN]	2.96	1	3	5	5	0.940	1.025	1.191	0.821	9.63	2.96	5	5	2093	241.8	7.96
Q03135	Caveolin-1 OS=Homo sapiens GN=CAV1 PE=1 SV=4 - [CAV1_HUMAN]	45.51	2	9	9	300	2.484	0.950	1.109	0.821	731.09	45.51	16	300	178	20.5	6.02
Q9NZD8	Maspardin OS=Homo sapiens GN=SPG21 PE=1 SV=1 - [SPG21_HUMAN]	13.31	1	2	3	3	1.143	1.595	1.023	0.821	7.59	13.31	3	3	308	34.9	6.28

Q9H0H0	Integrator complex subunit 2 OS=Homo sapiens GN=INTS2 PE=1 SV=2 - [INT2_HUMAN]	1.25	1	2	2	2	1.099	0.675	0.870	0.822	2.10	1.25	2	2	1204	134.2	6.05
Q4VCS5	Angiomotin OS=Homo sapiens GN=AMOT PE=1 SV=1 - [AMOT_HUMAN]	3.04	1	1	3	3	1.106	0.464	0.319	0.822	2.07	3.04	3	3	1084	118.0	7.64
Q9BSB4	Autophagy-related protein 101 OS=Homo sapiens GN=ATG101 PE=1 SV=1 - [ATGA1_HUMAN]	11.47	1	2	2	2	1.076	1.137	1.171	0.822	4.58	11.47	2	2	218	25.0	6.15
Q9P2J5	Leucine--tRNA ligase, cytoplasmic OS=Homo sapiens GN=LARS PE=1 SV=2 - [SYLC_HUMAN]	34.10	1	36	36	156	0.711	0.852	0.883	0.822	371.63	34.10	55	156	1176	134.4	7.30
Q16658	Fascin OS=Homo sapiens GN=FSCN1 PE=1 SV=3 - [FSCN1_HUMAN]	57.20	1	26	26	315	1.119	0.650	0.866	0.822	758.17	57.20	45	315	493	54.5	7.24
P52848	Bifunctional heparan sulfate N-deacetylase/N-sulfotransferas e 1 OS=Homo sapiens GN=NDST1 PE=1 SV=1 - [NDST1_HUMAN	1.13	1	1	1	1	0.884	0.723	1.097	0.822	0.00	1.13	1	1	882	100.8	7.97
Q9NZD2	Glycolipid transfer protein OS=Homo sapiens GN=GLTP PE=1 SV=3 - [GLTP_HUMAN]	16.27	1	4	4	14	0.800	1.286	1.105	0.822	33.81	16.27	6	14	209	23.8	7.39
Q12766	HMG domain-containing protein 3 OS=Homo sapiens GN=HMGXB3 PE=2 SV=2 - [HMGX3_HUMA N]	1.50	1	2	2	2	2.490	1.605	1.564	0.822	5.01	1.50	2	2	1538	168.2	8.05
Q9C0H6	Kelch-like protein 4 OS=Homo sapiens GN=KLHL4 PE=1 SV=2 - [KLHL4_HUMAN]	2.92	2	2	2	2	0.912	0.967	1.220	0.822	2.23	2.92	2	2	718	80.2	6.86

Q9H981	Actin-related protein 8 OS=Homo sapiens GN=ACTR8 PE=1 SV=2 - [ARP8_HUMAN]	1.92	1	1	1	1	0.710	0.667	0.686	0.822	2.13	1.92	1	1	624	70.4	7.78
Q9Y4K4	Mitogen-activated protein kinase kinase kinase kinase 5 OS=Homo sapiens GN=MAP4K5 PE=1 SV=1 - [M4K5_HUMAN]	10.05	1	8	8	19	0.659	0.862	0.780	0.822	42.23	10.05	14	19	846	95.0	7.64
Q9BV57	1,2-dihydroxy-3-keto-5-methylthiopentene dioxygenase OS=Homo sapiens GN=ADI1 PE=1 SV=1 - [MTND_HUMAN]	50.84	1	6	6	13	1.241	1.234	1.242	0.823	28.07	50.84	11	13	179	21.5	5.68
P49902	Cytosolic purine 5'-nucleotidase OS=Homo sapiens GN=NT5C2 PE=1 SV=1 - [5NTC_HUMAN]	37.25	1	17	17	40	1.242	0.424	0.670	0.823	93.24	37.25	24	40	561	64.9	6.14
Q6NXE6	Armadillo repeat-containing protein 6 OS=Homo sapiens GN=ARMC6 PE=1 SV=2 - [ARMC6_HUMA	8.18	1	5	5	10	0.906	0.772	1.040	0.823	24.78	8.18	8	10	501	54.1	6.24
Q8WUB2	Protein FAM216A OS=Homo sapiens GN=FAM216A PE=2 SV=1 - [F216A_HUMAN]	15.02	1	2	2	3	0.277	1.196	0.833	0.823	2.07	15.02	2	3	273	30.8	9.33
Q02809	Procollagen-lysine,2-oxoglutarate 5-dioxygenase 1 OS=Homo sapiens GN=PLOD1 PE=1 SV=2 - [PLOD1_HUMAN]	47.87	1	30	31	125	0.768	0.909	1.028	0.823	340.57	47.87	49	125	727	83.5	6.95
O00499	Myc box-dependent-interacting protein 1 OS=Homo sapiens GN=BIN1 PE=1 SV=1 - [BIN1_HUMAN]	34.06	1	15	15	63	0.930	0.725	0.871	0.823	163.06	34.06	24	63	593	64.7	5.06
Q96SQ9	Cytochrome P450 2S1 OS=Homo sapiens GN=CYP2S1 PE=2 SV=2 - [CP2S1_HUMAN]	11.31	1	5	5	14	3.227	0.750	1.119	0.823	35.04	11.31	7	14	504	55.8	8.62

P54252	Ataxin-3 OS=Homo sapiens GN=ATXN3 PE=1 SV=4 - [ATX3_HUMAN]	11.26	1	4	4	6	0.751	1.146	0.935	0.823	11.97	11.26	5	6	364	41.8	4.91
Q86SF2	N-acetylgalactosaminyltransferase 7 OS=Homo sapiens GN=GALNT7 PE=1 SV=1 - [GALT7_HUMAN]	12.02	1	6	6	17	0.802	1.260	0.955	0.823	39.35	12.02	9	17	657	75.3	7.11
O43294	Transforming growth factor beta-1-induced transcript 1 protein OS=Homo sapiens GN=TGFB1I1 PE=1 SV=2 - [TGFI1_HUMAN]	22.78	1	7	7	66	0.862	1.164	1.820	0.823	208.76	22.78	9	66	461	49.8	7.03
Q6NZI2	Polymerase I and transcript release factor OS=Homo sapiens GN=PTRF PE=1 SV=1 - [PTRF_HUMAN]	42.56	1	23	23	343	1.838	0.992	1.396	0.823	788.95	42.56	34	343	390	43.4	5.60
Q9Y4D7	Plexin-D1 OS=Homo sapiens GN=PLXND1 PE=1 SV=3 - [PLXD1_HUMAN]	0.52	1	1	1	1	1.202	0.541	0.440	0.823	0.00	0.52	1	1	1925	211.9	7.15
O75330	Hyaluronan mediated motility receptor OS=Homo sapiens GN=HMMR PE=1 SV=2 - [HMMR_HUMAN	7.46	1	4	6	9	0.634	0.851	0.887	0.823	17.79	7.46	6	9	724	84.0	5.83
Q15035	Translocating chain-associated membrane protein 2 OS=Homo sapiens GN=TRAM2 PE=1 SV=1 - [TRAM2_HUMA	2.97	1	1	1	7	1.162	1.138	1.143	0.823	17.12	2.97	2	7	370	43.3	9.22
P60709	Actin, cytoplasmic 1 OS=Homo sapiens GN=ACTB PE=1 SV=1 - [ACTB_HUMAN]	79.73	2	12	38	4774	0.978	0.976	1.180	0.823	#####	79.73	67	4774	375	41.7	5.48
Q9Y5A7	NEDD8 ultimate buster 1 OS=Homo sapiens GN=NUB1 PE=1 SV=2 - [NUB1_HUMAN]	10.08	1	5	8	17	1.006	1.020	0.940	0.823	39.64	10.08	10	17	615	70.5	5.96

P00568	Adenylate kinase isoenzyme 1 OS=Homo sapiens GN=AK1 PE=1 SV=3 - [KAD1_HUMAN]	57.22	1	11	12	84	1.173	1.058	1.216	0.824	206.90	57.22	17	84	194	21.6	8.63
Q9NR20	Dual specificity tyrosine-phosphorylation-regulated kinase 4 OS=Homo sapiens GN=DYRK4 PE=1 SV=2 - [DYRK4_HUMAN]	3.08	1	2	2	3	0.816	0.916	1.158	0.824	5.53	3.08	2	3	520	59.6	8.90
Q9NSB8	Homer protein homolog 2 OS=Homo sapiens GN=HOMER2 PE=1 SV=1 - [HOME2_HUMAN]	7.63	1	2	3	4	0.815	0.943	1.011	0.824	8.07	7.63	3	4	354	40.6	6.38
Q8N668	COMM domain-containing protein 1 OS=Homo sapiens GN=COMMD1 PE=1 SV=1 - [COMD1_HUMAN]	26.32	1	3	4	8	1.070	1.280	1.568	0.824	13.66	26.32	4	8	190	21.2	6.20
O95359	Transforming acidic coiled-coil-containing protein 2 OS=Homo sapiens GN=TACC2 PE=1 SV=3 - [TACC2_HUMAN]	1.90	1	2	5	19	0.260	0.708	1.693	0.824	48.77	1.90	6	19	2948	309.2	4.79
Q6PI77	Protein BHLHb9 OS=Homo sapiens GN=BHLHB9 PE=2 SV=1 - [BHLH9_HUMAN]	2.56	1	1	2	8	0.870	0.927	1.222	0.824	14.71	2.56	2	8	547	60.3	7.58
P59998	Actin-related protein 2/3 complex subunit 4 OS=Homo sapiens GN=ARPC4 PE=1 SV=3 - [ARPC4_HUMAN]	45.83	1	8	8	89	0.825	0.737	0.933	0.825	197.93	45.83	13	89	168	19.7	8.43
P61803	Dolichyl-diphosphooligosaccharide--protein glycosyltransferase subunit DAD1 OS=Homo sapiens GN=DAD1 PE=1 SV=3 - [DAD1_HUMAN]	19.47	1	2	2	16	0.554	0.574	0.662	0.825	43.91	19.47	4	16	113	12.5	7.08

Q04446	1,4-alpha-glucan-branching enzyme OS=Homo sapiens GN=GBE1 PE=1 SV=3 - [GLGB_HUMAN]	40.88	1	28	29	215	0.968	0.692	0.921	0.825	472.02	40.88	49	215	702	80.4	6.32
P36969	Phospholipid hydroperoxide glutathione peroxidase, mitochondrial OS=Homo sapiens GN=GPX4 PE=1 SV=3 - [GPX4_HUMAN]	40.10	1	8	8	36	1.247	0.950	0.988	0.825	87.12	40.10	14	36	197	22.2	8.37
Q8NHM5	Lysine-specific demethylase 2B OS=Homo sapiens GN=KDM2B PE=1 SV=1 - [KDM2B_HUMAN]	0.97	1	1	1	1	1.046	1.110	0.817	0.825	0.00	0.97	1	1	1336	152.5	8.56
Q15311	RalA-binding protein 1 OS=Homo sapiens GN=RALBP1 PE=1 SV=3 - [RBP1_HUMAN]	7.02	1	4	4	6	0.683	0.839	0.857	0.825	13.67	7.02	4	6	655	76.0	5.88
Q9NRW3	DNA dC->dU-editing enzyme APOBEC-3C OS=Homo sapiens GN=APOBEC3C PE=1 SV=2 - [ABC3C_HUMAN]	3.16	1	1	1	1	0.864	0.706	0.755	0.825	0.00	3.16	1	1	190	22.8	7.59
B3KS81	Serine/arginine repetitive matrix protein 5 OS=Homo sapiens GN=SRRM5 PE=1 SV=3 - [SRRM5_HUMAN]	1.68	1	1	1	5	1.393	1.356	1.785	0.825	9.56	1.68	1	5	715	80.3	12.06
Q14BN4	Sarcolemmal membrane-associated protein OS=Homo sapiens GN=SLMAP PE=1 SV=1 - [SLMAP_HUMAN]	17.51	1	15	16	37	0.705	2.106	2.001	0.825	84.21	17.51	23	37	828	95.1	5.47
Q9NQW7	Xaa-Pro aminopeptidase 1 OS=Homo sapiens GN=XPNPEP1 PE=1 SV=3 - [XPP1_HUMAN]	27.93	1	17	18	52	0.895	0.703	0.797	0.825	151.30	27.93	28	52	623	69.9	5.67
Q5SW79	Centrosomal protein of 170 kDa OS=Homo sapiens GN=CEP170 PE=1 SV=1 - [CE170_HUMAN]	22.35	2	31	32	91	0.741	1.107	1.010	0.825	207.26	22.35	51	91	1584	175.2	7.11

Q15477	Helicase SKI2W OS=Homo sapiens GN=SKIV2L PE=1 SV=3 - [SKIV2_HUMAN]	16.61	1	18	19	75	0.918	0.913	0.994	0.825	156.21	16.61	35	75	1246	137.7	6.06
O43237	Cytoplasmic dynein 1 light intermediate chain 2 OS=Homo sapiens GN=DYNC1LI2 PE=1 SV=1 - [DC1L2_HUMAN]	45.12	1	16	17	68	0.962	1.108	1.143	0.826	166.70	45.12	26	68	492	54.1	6.38
Q6ZUI0	Tumor protein p63-regulated gene 1 protein OS=Homo sapiens GN=TPRG1 PE=2 SV=1 - [TPRG1_HUMAN]	3.27	1	1	1	1	0.699	0.971	0.888	0.826	2.20	3.27	1	1	275	31.2	7.09
Q92633	Lysophosphatidic acid receptor 1 OS=Homo sapiens GN=LPAR1 PE=1 SV=3 - [LPAR1_HUMAN]	2.47	1	1	1	3	1.574	0.465	0.424	0.826	2.37	2.47	2	3	364	41.1	8.53
Q8WW24	Tektin-4 OS=Homo sapiens GN=TEKT4 PE=2 SV=1 - [TEKT4_HUMAN]	2.99	2	1	2	4	1.589	1.054	0.957	0.826	9.16	2.99	2	4	435	50.6	6.44
P05556	Integrin beta-1 OS=Homo sapiens GN=ITGB1 PE=1 SV=2 - [ITB1_HUMAN]	28.57	1	26	26	610	0.865	0.986	1.082	0.826	1378.20	28.57	46	610	798	88.4	5.39
Q8IWE2	Protein NOXP20 OS=Homo sapiens GN=FAM114A1 PE=1 SV=2 - [NXP20_HUMAN]	40.67	1	19	19	79	0.859	0.914	1.110	0.826	197.46	40.67	29	79	563	60.7	4.68
O75298	Reticulon-2 OS=Homo sapiens GN=RTN2 PE=1 SV=1 - [RTN2_HUMAN]	5.69	1	2	2	2	1.022	0.341	0.468	0.826	2.05	5.69	2	2	545	59.2	5.31
P78356	Phosphatidylinositol 5-phosphate 4-kinase type-2 beta OS=Homo sapiens GN=PIP4K2B PE=1 SV=1 - [PI42B_HUMAN	21.63	1	5	10	28	0.990	0.846	1.001	0.826	60.03	21.63	14	28	416	47.3	7.33

Q9Y673	Dolichyl-phosphate beta-glucosyltransferase OS=Homo sapiens GN=ALG5 PE=1 SV=1 - [ALG5_HUMAN]	16.98	1	5	5	16	1.145	0.714	0.641	0.826	36.74	16.98	9	16	324	36.9	9.28
Q3SXM5	Inactive hydroxysteroid dehydrogenase-like protein 1 OS=Homo sapiens GN=HSDL1 PE=1 SV=3 - [HSDL1_HUMAN]	11.21	1	4	4	11	1.094	0.812	0.902	0.826	29.24	11.21	7	11	330	37.0	8.72
Q5H9R7	Serine/threonine-protein phosphatase 6 regulatory subunit 3 OS=Homo sapiens GN=PPP6R3 PE=1 SV=2 - [PP6R3_HUMAN]	11.23	1	9	9	20	0.775	1.042	1.127	0.826	48.97	11.23	14	20	873	97.6	4.60
Q9ULU8	Calcium-dependent secretion activator 1 OS=Homo sapiens GN=CADPS PE=1 SV=3 - [CAPS1_HUMAN]	3.18	1	3	3	5	0.973	1.095	0.945	0.826	2.14	3.18	3	5	1353	152.7	5.71
Q9NUG6	p53 and DNA damage-regulated protein 1 OS=Homo sapiens GN=PDRG1 PE=1 SV=2 - [PDRG1_HUMAN]	21.80	1	3	3	4	0.919	0.984	0.793	0.827	6.75	21.80	4	4	133	15.5	6.06
Q9NYP3	Protein downstream neighbor of Son OS=Homo sapiens GN=DONSON PE=1 SV=2 - [DONS_HUMAN]	4.95	1	1	1	2		1.597		0.827	5.16	4.95	1	2	566	62.7	8.66
P62277	40S ribosomal protein S13 OS=Homo sapiens GN=RPS13 PE=1 SV=2 - [RS13_HUMAN]	42.38	1	8	8	145	0.962	0.625	0.692	0.827	295.65	42.38	12	145	151	17.2	10.54
Q8TD19	Serine/threonine-protein kinase Nek9 OS=Homo sapiens GN=NEK9 PE=1 SV=2 - [NEK9_HUMAN]	15.22	1	15	15	80	0.494	1.106	1.133	0.827	213.97	15.22	22	80	979	107.1	5.74

Q7Z3J2	UPF0505 protein C16orf62 OS=Homo sapiens GN=C16orf62 PE=1 SV=2 - [CP062_HUMAN]	9.35	1	8	8	16	0.971	0.751	0.949	0.827	34.48	9.35	10	16	963	109.5	7.21
Q9GZS3	WD repeat-containing protein 61 OS=Homo sapiens GN=WDR61 PE=1 SV=1 - [WDR61_HUMAN]	32.46	1	8	8	26	1.052	0.854	0.952	0.827	72.73	32.46	11	26	305	33.6	5.47
Q9HBI1	Beta-parvin OS=Homo sapiens GN=PARVB PE=1 SV=1 - [PARVB_HUMAN]	11.54	1	2	4	9	0.898	0.682	1.157	0.827	20.61	11.54	6	9	364	41.7	6.73
Q96KC8	DnaJ homolog subfamily C member 1 OS=Homo sapiens GN=DNAJC1 PE=1 SV=1 - [DNJC1_HUMAN]	16.06	1	9	9	24	0.752	0.871	0.617	0.827	46.72	16.06	12	24	554	63.8	8.63
Q9Y2T2	AP-3 complex subunit mu-1 OS=Homo sapiens GN=AP3M1 PE=1 SV=1 - [AP3M1_HUMAN]	26.79	1	7	10	38	0.970	0.985	0.922	0.827	78.36	26.79	15	38	418	46.9	6.93
P62256	Ubiquitin-conjugating enzyme E2 H OS=Homo sapiens GN=UBE2H PE=1 SV=1 - [UBE2H_HUMAN]	34.97	1	7	7	18	1.290	1.089	0.993	0.828	38.71	34.97	9	18	183	20.6	4.67
Q9UIC8	Leucine carboxyl methyltransferase 1 OS=Homo sapiens GN=LCMT1 PE=1 SV=2 - [LCMT1_HUMAN	22.16	1	8	8	15	1.185	0.819	0.965	0.828	30.21	22.16	11	15	334	38.4	6.04
Q96CX3	Zinc finger protein 501 OS=Homo sapiens GN=ZNF501 PE=2 SV=2 - [ZN501_HUMAN]	14.76	94	1	2	3	0.907	0.513	1.476	0.828	2.34	14.76	2	3	271	31.2	9.19
Q86V20	Protein FAM35A OS=Homo sapiens GN=FAM35A PE=2 SV=1 - [FA35A_HUMAN]	2.75	1	1	2	6	1.509	1.014	1.472	0.828	14.66	2.75	2	6	835	93.6	6.86

Q9H1Y0	Autophagy protein 5 OS=Homo sapiens GN=ATG5 PE=1 SV=2 - [ATG5_HUMAN]	13.82	1	3	3	11	0.917	0.800	0.726	0.828	27.13	13.82	5	11	275	32.4	5.77
Q8IVF4	Dynein heavy chain 10, axonemal OS=Homo sapiens GN=DNAH10 PE=1 SV=4 - [DYH10_HUMAN]	1.36	1	4	6	7	1.282	0.922	0.813	0.828	8.67	1.36	6	7	4471	514.5	5.88
Q03426	Mevalonate kinase OS=Homo sapiens GN=MVK PE=1 SV=1 - [KIME_HUMAN]	13.89	1	3	3	11	1.012	0.604	0.939	0.828	21.10	13.89	5	11	396	42.4	6.46
Q9GZX9	Twisted gastrulation protein homolog 1 OS=Homo sapiens GN=TWSG1 PE=2 SV=1 - [TWSG1_HUMAN]	15.25	1	3	3	22	1.152	1.025	0.956	0.828	51.89	15.25	6	22	223	25.0	5.34
Q8IWB6	Inactive serine/threonine-protein kinase TEX14 OS=Homo sapiens GN=TEX14 PE=1 SV=2 - [TEX14_HUMAN]	1.54	1	2	2	2	0.748	0.902	0.838	0.828	0.00	1.54	2	2	1497	167.8	5.15
Q8WWX9	Selenoprotein M OS=Homo sapiens GN=SELM PE=1 SV=3 - [SELM_HUMAN]	35.86	1	5	5	32	0.890	0.928	0.899	0.829	81.42	35.86	8	32	145	16.2	5.54
Q16181	Septin-7 OS=Homo sapiens GN=SEPT7 PE=1 SV=2 - [SEPT7_HUMAN]	64.99	2	33	35	343	0.828	0.946	0.969	0.829	881.54	64.99	59	343	437	50.6	8.63
Q16775	Hydroxyacylglutathione hydrolase, mitochondrial OS=Homo sapiens GN=HAGH PE=1 SV=2 - [GLO2_HUMAN]	27.27	1	6	6	42	1.105	0.996	1.217	0.829	121.72	27.27	10	42	308	33.8	8.12
Q9HBL8	NmrA-like family domain-containing protein 1 OS=Homo sapiens GN=NMRAL1 PE=1 SV=1 - [NMRL1_HUMAN]	22.41	1	7	7	19	1.031	1.178	1.266	0.829	36.54	22.41	10	19	299	33.3	7.52

Q9UGC6	Regulator of G-protein signaling 17 OS=Homo sapiens GN=RGS17 PE=1 SV=2 - [RGS17_HUMAN]	5.71	1	1	1	1	0.536	1.038	0.579	0.829	1.98	5.71	1	1	210	24.3	5.71
Q5T160	Probable arginine--tRNA ligase, mitochondrial OS=Homo sapiens GN=RARS2 PE=1 SV=1 - [SYRM_HUMAN]	20.42	1	12	12	34	1.004	0.859	1.102	0.829	65.38	20.42	20	34	578	65.5	8.21
Q96GX9	Methylthioribulose-1-phosphate dehydratase OS=Homo sapiens GN=APIP PE=1 SV=1 - [MTNB_HUMAN]	16.94	1	4	4	15	0.876	0.935	1.117	0.829	33.05	16.94	6	15	242	27.1	7.12
O00470	Homeobox protein Meis1 OS=Homo sapiens GN=MEIS1 PE=1 SV=1 - [MEIS1_HUMAN]	5.90	4	2	2	3	0.557	2.049	1.058	0.829	8.77	5.90	2	3	390	43.0	6.33
Q9HAV0	Guanine nucleotide-binding protein subunit beta-4 OS=Homo sapiens GN=GNB4 PE=1 SV=3 - [GBB4_HUMAN]	19.41	1	2	5	20	1.011	1.526	1.021	0.829	51.97	19.41	8	20	340	37.5	6.00
Q96RR4	Calcium/calmodulin-dependent protein kinase kinase 2 OS=Homo sapiens GN=CAMKK2 PE=1 SV=2 - [KKCC2_HUMAN]	8.33	1	5	5	10	0.957	0.983	1.100	0.830	16.68	8.33	8	10	588	64.7	6.68
Q14974	Importin subunit beta-1 OS=Homo sapiens GN=KPNB1 PE=1 SV=2 - [IMB1_HUMAN]	35.62	1	26	26	292	0.850	0.990	0.867	0.830	740.81	35.62	46	292	876	97.1	4.78
Q07889	Son of sevenless homolog 1 OS=Homo sapiens GN=SOS1 PE=1 SV=1 - [SOS1_HUMAN]	3.83	1	5	5	14	0.756	1.497	1.134	0.830	17.76	3.83	6	14	1333	152.4	6.84

Q9NZZ3	Charged multivesicular body protein 5 OS=Homo sapiens GN=CHMP5 PE=1 SV=1 - [CHMP5_HUMAN]	36.07	1	6	6	19	1.099	0.913	0.984	0.830	45.24	36.07	8	19	219	24.6	4.83
Q6QNY0	Biogenesis of lysosome-related organelles complex 1 subunit 3 OS=Homo sapiens GN=BLOC1S3 PE=1 SV=1 - [BL1S3_HUMAN]	13.86	1	2	2	5	0.935	0.730	0.996	0.830	6.88	13.86	4	5	202	21.2	5.15
P24043	Laminin subunit alpha-2 OS=Homo sapiens GN=LAMA2 PE=1 SV=4 - [LAMA2_HUMAN]	2.27	1	7	8	16	1.233	0.577	0.500	0.830	31.89	2.27	11	16	3122	343.7	6.40
P10619	Lysosomal protective protein OS=Homo sapiens GN=CTSA PE=1 SV=2 - [PPGB_HUMAN]	22.29	1	10	11	36	0.685	0.908	0.895	0.830	73.08	22.29	16	36	480	54.4	6.61
O15230	Laminin subunit alpha-5 OS=Homo sapiens GN=LAMA5 PE=1 SV=8 - [LAMA5_HUMAN]	2.54	1	6	7	14	1.238	0.996	1.572	0.830	16.56	2.54	9	14	3695	399.5	7.02
Q92843	Bcl-2-like protein 2 OS=Homo sapiens GN=BCL2L2 PE=1 SV=2 - [B2CL2_HUMAN]	5.18	1	1	1	1	0.986	1.091	1.528	0.830	0.00	5.18	1	1	193	20.7	5.34
O95628	CCR4-NOT transcription complex subunit 4 OS=Homo sapiens GN=CNOT4 PE=1 SV=3 - [CNOT4_HUMAN]	3.48	1	1	1	4	0.693	1.277	1.449	0.830	5.59	3.48	1	4	575	63.5	7.03
Q5VWJ9	Sorting nexin-30 OS=Homo sapiens GN=SNX30 PE=1 SV=1 - [SNX30_HUMAN]	6.41	1	3	3	16	0.973	0.893	0.890	0.830	33.00	6.41	6	16	437	49.6	5.35
Q8NI99	Angiopoietin-related protein 6 OS=Homo sapiens GN=ANGPTL6 PE=1 SV=1 - [ANGL6_HUMAN]	3.62	1	1	1	1	1.377	1.401	0.782	0.830	0.00	3.62	1	1	470	51.7	8.53

Q15067	Peroxisomal acyl-coenzyme A oxidase 1 OS=Homo sapiens GN=ACOX1 PE=1 SV=3 - [ACOX1_HUMAN]	13.64	1	6	6	9	0.779	1.228	1.066	0.830	16.79	13.64	7	9	660	74.4	8.16
Q16576	Histone-binding protein RBBP7 OS=Homo sapiens GN=RBBP7 PE=1 SV=1 - [RBBP7_HUMAN]	15.53	1	3	8	22	0.960	1.064	1.062	0.831	65.81	15.53	12	22	425	47.8	5.05
Q6ZSS7	Major facilitator superfamily domain-containing protein 6 OS=Homo sapiens GN=MFSD6 PE=1 SV=2 - [MFSD6_HUMA	6.45	1	2	3	7	1.038	0.732	0.605	0.831	16.95	6.45	4	7	791	88.0	5.53
Q8WXR4	Myosin-IIIb OS=Homo sapiens GN=MYO3B PE=2 SV=4 - [MYO3B_HUMAN]	0.67	1	1	1	1	0.846	1.141	1.308	0.831	2.32	0.67	1	1	1341	151.7	8.15
Q9NRV9	Heme-binding protein 1 OS=Homo sapiens GN=HEBP1 PE=1 SV=1 - [HEBP1_HUMAN]	54.50	1	8	8	60	1.372	0.511	0.860	0.831	133.47	54.50	15	60	189	21.1	5.80
A6NEL2	Ankyrin repeat domain-containing protein SOWAHB OS=Homo sapiens GN=SOWAHB PE=2 SV=1 - [SWAHB_HUMAN]	1.13	1	1	1	1	1.294	0.553	0.954	0.831	2.49	1.13	1	1	793	85.7	9.60
Q9H9P5	Putative E3 ubiquitin-protein ligase UNKL OS=Homo sapiens GN=UNKL PE=1 SV=3 - [UNKL_HUMAN]	4.71	1	2	2	4	0.846	1.343	1.437	0.831	13.20	4.71	2	4	680	73.8	8.06
Q5VUY0	Arylacetamide deacetylase-like 3 OS=Homo sapiens GN=AADACL3 PE=2 SV=4 - [ADCL3_HUMAN]	5.14	1	1	1	1	0.916	0.922	1.257	0.831	0.00	5.14	1	1	350	39.7	7.39
Q9BW19	Kinesin-like protein KIFC1 OS=Homo sapiens GN=KIFC1 PE=1 SV=2 - [KIFC1_HUMAN]	5.20	1	3	3	4	1.007	0.917	1.007	0.831	0.00	5.20	3	4	673	73.7	8.98

P46977	Dolichyl-diphosphooligosaccharide--protein glycosyltransferase subunit STT3A OS=Homo sapiens GN=STT3A PE=1 SV=2 - [STT3A_HUMAN]	14.47	1	11	11	81	0.635	0.707	0.655	0.831	209.31	14.47	21	81	705	80.5	8.07
Q96QS3	Homeobox protein ARX OS=Homo sapiens GN=ARX PE=1 SV=1 - [ARX_HUMAN]	2.67	1	1	1	1	2.424	1.591	2.648	0.831	1.62	2.67	1	1	562	58.1	5.24
Q13948	Protein CASP OS=Homo sapiens GN=CUX1 PE=1 SV=2 - [CASP_HUMAN]	27.14	2	16	16	38	0.746	1.003	0.891	0.831	82.67	27.14	23	38	678	77.4	5.44
Q9UPU7	TBC1 domain family member 2B OS=Homo sapiens GN=TBC1D2B PE=1 SV=2 - [TBD2B_HUMAN]	12.98	1	11	11	17	0.711	0.959	1.021	0.831	32.23	12.98	14	17	963	109.8	6.16
Q5T5C0	Syntaxin-binding protein 5 OS=Homo sapiens GN=STXBP5 PE=1 SV=1 - [STXB5_HUMAN]	6.26	1	5	5	10	0.839	1.281	1.031	0.831	19.65	6.26	7	10	1151	127.5	7.28
Q8IVM0	Coiled-coil domain-containing protein 50 OS=Homo sapiens GN=CCDC50 PE=1 SV=1 - [CCD50_HUMAN]	30.72	1	9	9	42	0.820	1.007	1.105	0.831	103.77	30.72	16	42	306	35.8	6.65
O75882	Attractin OS=Homo sapiens GN=ATRN PE=1 SV=2 - [ATRN_HUMAN]	3.64	1	6	6	12	0.887	0.779	1.047	0.831	30.64	3.64	7	12	1429	158.4	7.31
Q12904	Aminoacyl tRNA synthase complex-interacting multifunctional protein 1 OS=Homo sapiens GN=AIMP1 PE=1 SV=2 - [AIMP1_HUMAN]	56.41	3	13	14	64	1.042	0.999	0.918	0.831	159.68	56.41	21	64	312	34.3	8.43

P31749	RAC-alpha serine/threonine-protein kinase OS=Homo sapiens GN=AKT1 PE=1 SV=2 - [AKT1_HUMAN]	18.75	2	5	8	12	1.067	0.732	0.822	0.832	29.24	18.75	10	12	480	55.7	6.07
Q96EK7	Constitutive coactivator of peroxisome proliferator-activated receptor gamma OS=Homo sapiens GN=FAM120B PE=1 SV=1 - [F120B_HUMAN	10.99	1	7	7	12	0.768	0.937	1.202	0.832	18.70	10.99	8	12	910	103.7	5.69
Q5VZF2	Muscleblind-like protein 2 OS=Homo sapiens GN=MBNL2 PE=1 SV=2 - [MBNL2_HUMAN]	13.94	1	2	6	30	0.769	0.792	0.795	0.832	47.46	13.94	9	30	373	40.5	8.38
Q9H3H5	UDP-N-acetylglucosamine--dolichyl-phosphate N-acetylglucosaminephosphotransferase OS=Homo sapiens GN=DPAGT1 PE=1 SV=2 - [GPT_HUMAN]	2.45	1	2	2	2	1.330	3.185	1.969	0.832	0.00	2.45	2	2	408	46.1	8.00
Q13813	Spectrin alpha chain, non-erythrocytic 1 OS=Homo sapiens GN=SPTAN1 PE=1 SV=3 - [SPTN1_HUMAN]	68.81	1	169	170	1151	1.190	1.058	0.899	0.833	2961.27	68.81	286	1151	2472	284.4	5.35
P47813	Eukaryotic translation initiation factor 1A, X-chromosomal OS=Homo sapiens GN=EIF1AX PE=1 SV=2 - [IF1AX_HUMAN]	29.86	2	6	6	38	0.625	0.837	0.755	0.833	93.80	29.86	11	38	144	16.5	5.24
O00148	ATP-dependent RNA helicase DDX39A OS=Homo sapiens GN=DDX39A PE=1 SV=2 - [DX39A_HUMAN	34.43	1	4	15	103	1.045	0.817	0.662	0.833	191.01	34.43	23	103	427	49.1	5.68
Q13976	cGMP-dependent protein kinase 1 OS=Homo sapiens GN=PRKG1 PE=1 SV=3 - [KGP1_HUMAN]	29.66	4	18	19	64	0.976	0.638	1.156	0.833	153.73	29.66	30	64	671	76.3	6.04

P32321	Deoxycytidylate deaminase OS=Homo sapiens GN=DCTD PE=1 SV=2 - [DCTD_HUMAN]	23.03	1	3	3	20	1.037	0.703	0.775	0.833	50.07	23.03	6	20	178	20.0	7.56
P05155	Plasma protease C1 inhibitor OS=Homo sapiens GN=SERPING1 PE=1 SV=2 - [IC1_HUMAN]	6.20	1	3	3	5	0.747	1.045	0.598	0.833	10.99	6.20	5	5	500	55.1	6.55
O95070	Protein YIF1A OS=Homo sapiens GN=YIF1A PE=1 SV=2 - [YIF1A_HUMAN]	2.39	1	1	1	1	0.218	0.323	0.609	0.833	1.95	2.39	1	1	293	32.0	8.95
Q9BYM8	RanBP-type and C3HC4-type zinc finger-containing protein 1 OS=Homo sapiens GN=RBCK1 PE=1 SV=2 - [HOIL1_HUMAN]	25.69	1	12	12	24	1.055	1.043	1.165	0.833	47.61	25.69	19	24	510	57.5	5.67
P52907	F-actin-capping protein subunit alpha-1 OS=Homo sapiens GN=CAPZA1 PE=1 SV=3 - [CAZA1_HUMAN]	58.74	1	9	12	201	0.831	0.802	0.890	0.833	507.24	58.74	22	201	286	32.9	5.69
Q8NEZ2	Vacuolar protein sorting-associated protein 37A OS=Homo sapiens GN=VPS37A PE=1 SV=1 - [VP37A_HUMAN]	12.34	3	5	6	18	0.946	0.958	1.128	0.833	36.48	12.34	10	18	397	44.3	5.57
Q9H0B6	Kinesin light chain 2 OS=Homo sapiens GN=KLC2 PE=1 SV=1 - [KLC2_HUMAN]	39.55	1	16	23	83	0.838	0.993	0.940	0.834	191.55	39.55	39	83	622	68.9	7.15
O15014	Zinc finger protein 609 OS=Homo sapiens GN=ZNF609 PE=1 SV=2 - [ZN609_HUMAN]	0.85	1	1	1	1	1.665	0.881	0.594	0.834	0.00	0.85	1	1	1411	151.1	8.03
Q96KP1	Exocyst complex component 2 OS=Homo sapiens GN=EXOC2 PE=1 SV=1 - [EXOC2_HUMAN]	17.42	1	13	15	37	1.121	0.840	1.004	0.834	61.64	17.42	20	37	924	104.0	6.90

Q68D51	DENN domain-containing protein 2C OS=Homo sapiens GN=DENND2C PE=1 SV=2 - [DEN2C_HUMAN]	2.26	2	1	2	4	0.634	1.062	1.408	0.834	7.93	2.26	3	4	928	106.8	8.48
Q9NQX7	Integral membrane protein 2C OS=Homo sapiens GN=ITM2C PE=1 SV=1 - [ITM2C_HUMAN]	14.98	1	3	3	17	0.691	0.826	0.963	0.834	31.23	14.98	6	17	267	30.2	8.00
Q14642	Type I inositol 1,4,5-trisphosphate 5-phosphatase OS=Homo sapiens GN=INPP5A PE=1 SV=1 - [I5P1_HUMAN]	13.11	1	5	5	15	1.061	1.391	1.122	0.834	17.32	13.11	9	15	412	47.8	7.03
P54136	Arginine--tRNA ligase, cytoplasmic OS=Homo sapiens GN=RARS PE=1 SV=2 - [SYRC_HUMAN]	43.64	1	31	31	171	0.773	0.942	0.924	0.835	438.67	43.64	49	171	660	75.3	6.68
Q8IWX8	Calcium homeostasis endoplasmic reticulum protein OS=Homo sapiens GN=CHERP PE=1 SV=3 - [CHERP_HUMAN]	7.42	1	7	7	23	1.005	0.860	0.755	0.835	53.59	7.42	12	23	916	103.6	9.04
Q5SRE5	Nucleoporin NUP188 homolog OS=Homo sapiens GN=NUP188 PE=1 SV=1 - [NU188_HUMAN]	6.23	2	8	9	12	1.033	1.108	0.998	0.835	30.59	6.23	10	12	1749	195.9	6.73
Q9BY89	Uncharacterized protein KIAA1671 OS=Homo sapiens GN=KIAA1671 PE=1 SV=2 - [K1671_HUMAN]	0.66	1	1	1	2	1.282	1.059	0.822	0.835	4.54	0.66	1	2	1806	196.6	8.47
Q5BJD5	Transmembrane protein 41B OS=Homo sapiens GN=TMEM41B PE=1 SV=1 - [TM41B_HUMAN]	2.41	1	1	1	1	0.617	0.497	0.791	0.835	1.89	2.41	1	1	291	32.5	9.58

Q6I9Y2	THO complex subunit 7 homolog OS=Homo sapiens GN=THOC7 PE=1 SV=3 - [THOC7_HUMAN]	15.69	1	2	3	4	0.761	1.151	0.904	0.835	8.51	15.69	4	4	204	23.7	5.67
Q9H9B1	Histone-lysine N-methyltransferase EHMT1 OS=Homo sapiens GN=EHMT1 PE=1 SV=4 - [EHMT1_HUMAN]	1.77	1	1	1	1	1.083	1.180	1.226	0.835	0.00	1.77	1	1	1298	141.4	5.76
P46940	Ras GTPase-activating-like protein IQGAP1 OS=Homo sapiens GN=IQGAP1 PE=1 SV=1 - [IQGA1_HUMAN]	45.20	1	75	77	525	1.115	1.180	1.081	0.835	1354.42	45.20	133	525	1657	189.1	6.48
Q9BV86	N-terminal Xaa-Pro-Lys N-methyltransferase 1 OS=Homo sapiens GN=NTMT1 PE=1 SV=3 - [NTM1A_HUMA	34.08	1	7	7	26	0.794	0.952	1.093	0.835	54.40	34.08	11	26	223	25.4	5.52
Q66K74	Microtubule-associated protein 1S OS=Homo sapiens GN=MAP1S PE=1 SV=2 - [MAP1S_HUMAN]	18.70	1	16	16	50	0.900	1.104	1.175	0.835	120.92	18.70	21	50	1059	112.1	7.30
P16615	Sarcoplasmic/endoplasmic reticulum calcium ATPase 2 OS=Homo sapiens GN=ATP2A2 PE=1 SV=1 - [AT2A2_HUMAN]	35.41	2	33	41	328	0.877	0.902	1.038	0.835	915.23	35.41	72	328	1042	114.7	5.34
P36776	Lon protease homolog, mitochondrial OS=Homo sapiens GN=LONP1 PE=1 SV=2 - [LONM_HUMAN]	33.37	2	25	26	124	0.803	1.097	0.861	0.835	298.79	33.37	39	124	959	106.4	6.39
Q9NZ56	Formin-2 OS=Homo sapiens GN=FMN2 PE=1 SV=4 - [FMN2_HUMAN]	4.12	2	3	7	16	0.810	0.702	1.554	0.835	33.92	4.12	8	16	1722	180.0	5.47

Q5CZC0	Fibrous sheath-interacting protein 2 OS=Homo sapiens GN=FSIP2 PE=2 SV=4 - [FSIP2_HUMAN]	0.78	1	4	5	5	0.971	0.552	0.673	0.835	6.18	0.78	5	5	6907	780.1	6.71
Q16595	Frataxin, mitochondrial OS=Homo sapiens GN=FXN PE=1 SV=2 - [FRDA_HUMAN]	15.24	1	4	4	10	0.672	0.988	1.062	0.836	13.06	15.24	6	10	210	23.1	8.69
A6NC57	Ankyrin repeat domain-containing protein 62 OS=Homo sapiens GN=ANKRD62 PE=2 SV=4 - [ANR62_HUMAN]	1.09	1	1	1	1	0.882	0.888	1.023	0.836	2.65	1.09	1	1	917	106.4	6.67
Q92604	Acyl-CoA:lysophosphatidylglycerol acyltransferase 1 OS=Homo sapiens GN=LPGAT1 PE=2 SV=1 - [LGAT1_HUMAN]	4.86	1	2	2	6	1.033	0.616	0.710	0.836	12.97	4.86	3	6	370	43.1	8.92
Q9H6W3	Bifunctional lysine-specific demethylase and histidyl-hydroxylase NO66 OS=Homo sapiens GN=NO66 PE=1 SV=2 - [NO66_HUMAN]	10.92	1	6	6	11	0.959	1.090	1.078	0.836	22.95	10.92	7	11	641	71.0	6.46
Q6IBS0	Twinfilin-2 OS=Homo sapiens GN=TWF2 PE=1 SV=2 - [TWF2_HUMAN]	50.43	1	12	14	80	0.966	1.015	1.037	0.836	262.95	50.43	22	80	349	39.5	6.84
P40261	Nicotinamide N-methyltransferase OS=Homo sapiens GN=NNMT PE=1 SV=1 - [NNMT_HUMAN]	25.38	1	7	7	109	0.906	0.585	0.770	0.836	351.74	25.38	13	109	264	29.6	5.74
Q9NVJ2	ADP-ribosylation factor-like protein 8B OS=Homo sapiens GN=ARL8B PE=1 SV=1 - [ARL8B_HUMAN]	34.41	1	2	6	16	1.172	0.933	0.746	0.836	35.60	34.41	9	16	186	21.5	8.43

P14923	Junction plakoglobin OS=Homo sapiens GN=JUP PE=1 SV=3 - [PLAK_HUMAN]	17.32	1	8	11	32	2.459	1.649	1.563	0.836	78.07	17.32	17	32	745	81.7	6.14
Q9Y450	HBS1-like protein OS=Homo sapiens GN=HBS1L PE=1 SV=1 - [HBS1L_HUMAN]	32.60	1	17	17	51	0.764	1.043	1.042	0.836	118.20	32.60	26	51	684	75.4	6.61
P61758	Prefoldin subunit 3 OS=Homo sapiens GN=VBP1 PE=1 SV=3 - [PFD3_HUMAN]	61.42	1	12	13	76	0.822	0.957	0.786	0.836	199.52	61.42	21	76	197	22.6	7.11
Q12962	Transcription initiation factor TFIID subunit 10 OS=Homo sapiens GN=TAF10 PE=1 SV=1 - [TAF10_HUMAN]	15.14	1	2	2	5	0.637	0.867	0.639	0.836	17.54	15.14	3	5	218	21.7	6.57
Q9NYL9	Tropomodulin-3 OS=Homo sapiens GN=TMOD3 PE=1 SV=1 - [TMOD3_HUMA N]	58.81	1	15	17	87	1.171	0.957	0.895	0.837	211.41	58.81	28	87	352	39.6	5.19
P25098	Beta-adrenergic receptor kinase 1 OS=Homo sapiens GN=ADRBK1 PE=1 SV=2 - [ARBK1_HUMAN	5.66	1	3	4	5	1.055	0.987	1.093	0.837	8.65	5.66	5	5	689	79.5	7.28
P04406	Glyceraldehyde-3-phosphate dehydrogenase OS=Homo sapiens GN=GAPDH PE=1 SV=3 - [G3P_HUMAN]	86.87	1	34	34	2249	1.147	1.037	1.073	0.837	5688.32	86.87	56	2249	335	36.0	8.46
P05198	Eukaryotic translation initiation factor 2 subunit 1 OS=Homo sapiens GN=EIF2S1 PE=1 SV=3 - [IF2A_HUMAN]	65.40	1	24	25	120	0.851	0.854	0.900	0.837	298.99	65.40	40	120	315	36.1	5.08
O14653	Golgi SNAP receptor complex member 2 OS=Homo sapiens GN=GOSR2 PE=1 SV=2 - [GOSR2_HUMA N]	25.94	1	4	5	12	0.870	0.932	0.918	0.837	34.37	25.94	6	12	212	24.8	8.06

P49721	Proteasome subunit beta type-2 OS=Homo sapiens GN=PSMB2 PE=1 SV=1 - [PSB2_HUMAN]	69.15	1	13	14	75	0.872	0.814	0.877	0.837	160.07	69.15	23	75	201	22.8	7.02
Q96KQ4	Apoptosis-stimulating of p53 protein 1 OS=Homo sapiens GN=PPP1R13B PE=1 SV=3 - [ASPP1_HUMAN]	1.10	1	1	1	1	0.693	1.979	2.250	0.837	3.40	1.10	1	1	1090	119.5	6.76
P00492	Hypoxanthine-guanine phosphoribosyltransferase OS=Homo sapiens GN=HPRT1 PE=1 SV=2 - [HPRT_HUMAN]	52.75	1	8	8	36	1.232	0.922	0.919	0.837	83.57	52.75	14	36	218	24.6	6.68
Q8NEY8	Periphilin-1 OS=Homo sapiens GN=PPHLN1 PE=1 SV=2 - [PPHLN_HUMAN]	4.37	1	2	2	2	1.053	1.016	0.858	0.837	1.84	4.37	2	2	458	52.7	9.11
O94973	AP-2 complex subunit alpha-2 OS=Homo sapiens GN=AP2A2 PE=1 SV=2 - [AP2A2_HUMAN]	30.35	1	15	27	117	0.965	0.854	0.888	0.837	280.22	30.35	44	117	939	103.9	6.96
Q9H4G0	Band 4.1-like protein 1 OS=Homo sapiens GN=EPB41L1 PE=1 SV=2 - [E41L1_HUMAN]	13.62	1	7	9	18	0.697	0.623	0.849	0.838	30.48	13.62	11	18	881	98.4	5.62
Q6GMV3	Putative peptidyl-tRNA hydrolase PTRHD1 OS=Homo sapiens GN=PTRHD1 PE=1 SV=1 - [PTRD1_HUMAN	31.43	1	5	5	18	0.945	0.958	0.790	0.838	53.61	31.43	8	18	140	15.8	9.10
Q9Y4P8	WD repeat domain phosphoinositide-interacting protein 2 OS=Homo sapiens GN=WIPI2 PE=1 SV=1 - [WIPI2_HUMAN]	8.81	1	3	4	5	1.097	1.173	1.819	0.838	14.56	8.81	5	5	454	49.4	6.46
P84085	ADP-ribosylation factor 5 OS=Homo sapiens GN=ARF5 PE=1 SV=2 - [ARF5_HUMAN]	44.44	2	3	8	105	0.750	0.925	0.702	0.838	181.17	44.44	15	105	180	20.5	6.79

Q9UHK6	Alpha-methylacyl-CoA racemase OS=Homo sapiens GN=AMACR PE=1 SV=2 - [AMACR_HUMAN]	2.62	1	1	1	4	1.579	1.276	1.195	0.838	6.27	2.62	2	4	382	42.4	6.44
O75663	TIP41-like protein OS=Homo sapiens GN=TIPRL PE=1 SV=2 - [TIPRL_HUMAN]	45.59	1	11	11	36	0.814	0.986	1.025	0.838	100.40	45.59	16	36	272	31.4	5.91
Q9UKM7	Endoplasmic reticulum mannosyl-oligosaccharide 1,2-alpha-mannosidase OS=Homo sapiens GN=MAN1B1 PE=1 SV=2 - [MA1B1_HUMAN]	16.74	1	9	9	41	0.895	0.728	0.615	0.838	69.63	16.74	14	41	699	79.5	7.72
Q9Y3C6	Peptidyl-prolyl cis-trans isomerase-like 1 OS=Homo sapiens GN=PPIL1 PE=1 SV=1 - [PPIL1_HUMAN]	31.93	1	5	5	34	1.180	0.998	0.951	0.838	64.24	31.93	9	34	166	18.2	7.99
Q15404	Ras suppressor protein 1 OS=Homo sapiens GN=RSU1 PE=1 SV=3 - [RSU1_HUMAN]	43.68	1	14	14	74	0.967	1.043	1.210	0.838	163.18	43.68	23	74	277	31.5	8.65
Q06828	Fibromodulin OS=Homo sapiens GN=FMOD PE=1 SV=2 - [FMOD_HUMAN]	2.93	1	1	1	2	0.667	0.795	0.649	0.838	7.38	2.93	2	2	376	43.2	6.04
Q8N4U5	T-complex protein 11-like protein 2 OS=Homo sapiens GN=TCP11L2 PE=2 SV=1 - [T11L2_HUMAN]	4.82	1	1	2	4	0.700	0.986	0.972	0.838	10.09	4.82	2	4	519	58.1	4.96
Q92830	Histone acetyltransferase KAT2A OS=Homo sapiens GN=KAT2A PE=1 SV=3 - [KAT2A_HUMAN]	4.78	1	2	4	5	0.790	1.239	1.328	0.838	2.25	4.78	4	5	837	93.9	9.04
A1L0T0	Acetolactate synthase-like protein OS=Homo sapiens GN=ILVBL PE=1 SV=2 - [ILVBL_HUMAN]	12.97	1	7	7	20	0.996	0.850	0.862	0.838	47.34	12.97	11	20	632	67.8	8.15

Q86X55	Histone-arginine methyltransferase CARM1 OS=Homo sapiens GN=CARM1 PE=1 SV=3 - [CARM1_HUMAN]	12.50	1	6	6	25	0.797	0.803	0.974	0.838	47.50	12.50	9	25	608	65.8	6.73
P07195	L-lactate dehydrogenase B chain OS=Homo sapiens GN=LDHB PE=1 SV=2 - [LDHB_HUMAN]	53.59	2	19	21	401	0.877	0.888	0.879	0.838	946.17	53.59	36	401	334	36.6	6.05
Q9BY11	Protein kinase C and casein kinase substrate in neurons protein 1 OS=Homo sapiens GN=PACSIN1 PE=1 SV=1 - [PACN1_HUMAN	8.11	1	2	5	17	1.903	1.279	1.054	0.839	21.80	8.11	7	17	444	50.9	5.24
Q5T7N2	LINE-1 type transposase domain-containing protein 1 OS=Homo sapiens GN=L1TD1 PE=1 SV=1 - [LITD1_HUMAN]	2.08	1	1	1	1	0.703	0.554	0.770	0.839	0.00	2.08	1	1	865	98.8	4.93
O95835	Serine/threonine-protein kinase LATS1 OS=Homo sapiens GN=LATS1 PE=1 SV=1 - [LATS1_HUMAN]	3.54	1	3	4	4	0.860	0.755	1.245	0.839	8.30	3.54	4	4	1130	126.8	8.70
Q07954	Prolow-density lipoprotein receptor-related protein 1 OS=Homo sapiens GN=LRP1 PE=1 SV=2 - [LRP1_HUMAN]	21.17	1	83	83	332	0.810	0.875	0.829	0.839	874.48	21.17	139	332	4544	504.3	5.39
O75362	Zinc finger protein 217 OS=Homo sapiens GN=ZNF217 PE=1 SV=1 - [ZN217_HUMAN]	2.48	1	2	2	4	0.743	0.656	0.621	0.839	12.80	2.48	2	4	1048	115.2	8.48
P12955	Xaa-Pro dipeptidase OS=Homo sapiens GN=PEPD PE=1 SV=3 - [PEPD_HUMAN]	40.16	1	15	15	45	0.781	0.932	0.901	0.839	134.46	40.16	23	45	493	54.5	6.00

P48730	Casein kinase I isoform delta OS=Homo sapiens GN=CSNK1D PE=1 SV=2 - [KC1D_HUMAN]	15.90	1	2	6	14	0.811	1.024	1.027	0.839	31.79	15.90	8	14	415	47.3	9.74
Q96Q05	Trafficking protein particle complex subunit 9 OS=Homo sapiens GN=TRAPPC9 PE=1 SV=2 - [TPPC9_HUMAN]	5.05	1	5	5	7	1.044	0.890	1.041	0.840	8.37	5.05	7	7	1148	128.4	6.62
Q9BQR3	Serine protease 27 OS=Homo sapiens GN=PRSS27 PE=1 SV=1 - [PRS27_HUMAN	4.14	1	1	1	1	0.621	0.770	0.550	0.840	2.01	4.14	1	1	290	31.9	8.13
Q9BUW7	UPF0184 protein C9orf16 OS=Homo sapiens GN=C9orf16 PE=1 SV=1 - [CI016_HUMAN]	10.84	1	1	1	2	0.544	0.793	1.232	0.840	3.05	10.84	2	2	83	9.0	4.22
Q8IWB1	Inositol 1,4,5-trisphosphate receptor-interacting protein OS=Homo sapiens GN=ITPRIP PE=1 SV=1 - [IPRI_HUMAN]	13.89	1	6	7	30	0.884	0.890	1.009	0.840	54.49	13.89	11	30	547	62.0	5.88
Q9Y6X6	Unconventional myosin-XVI OS=Homo sapiens GN=MYO16 PE=1 SV=3 - [MYO16_HUMAN]	1.02	1	1	1	1	0.869	0.590	0.729	0.840	0.00	1.02	1	1	1858	206.0	6.81
Q15386	Ubiquitin-protein ligase E3C OS=Homo sapiens GN=UBE3C PE=1 SV=3 - [UBE3C_HUMAN]	16.16	1	12	13	33	0.847	0.662	0.963	0.840	85.54	16.16	19	33	1083	123.8	6.71
Q15436	Protein transport protein Sec23A OS=Homo sapiens GN=SEC23A PE=1 SV=2 - [SC23A_HUMAN]	24.71	1	18	19	99	0.840	0.876	0.966	0.840	254.68	24.71	32	99	765	86.1	7.08
Q99707	Methionine synthase OS=Homo sapiens GN=MTR PE=1 SV=2 - [METH_HUMAN]	4.11	1	5	5	13	0.694	0.783	0.857	0.840	25.82	4.11	8	13	1265	140.4	5.58

P31751	RAC-beta serine/threonine-protein kinase OS=Homo sapiens GN=AKT2 PE=1 SV=2 - [AKT2_HUMAN]	24.95	2	8	11	25	0.994	0.913	1.010	0.840	48.87	24.95	15	25	481	55.7	6.37
Q8WUA7	TBC1 domain family member 22A OS=Homo sapiens GN=TBC1D22A PE=1 SV=2 - [TB22A_HUMAN]	3.09	1	1	2	4	0.970	0.819	1.191	0.840	10.76	3.09	2	4	517	59.1	5.97
O95782	AP-2 complex subunit alpha-1 OS=Homo sapiens GN=AP2A1 PE=1 SV=3 - [AP2A1_HUMAN]	44.93	1	25	37	206	0.933	0.747	0.877	0.840	476.81	44.93	61	206	977	107.5	7.03
P62253	Ubiquitin-conjugating enzyme E2 G1 OS=Homo sapiens GN=UBE2G1 PE=1 SV=3 - [UB2G1_HUMAN]	18.24	1	4	4	9	0.474	0.889	0.742	0.840	12.72	18.24	7	9	170	19.5	5.30
P17931	Galectin-3 OS=Homo sapiens GN=LGALS3 PE=1 SV=5 - [LEG3_HUMAN]	32.40	1	10	11	223	0.855	1.199	1.159	0.840	486.40	32.40	20	223	250	26.1	8.56
O95816	BAG family molecular chaperone regulator 2 OS=Homo sapiens GN=BAG2 PE=1 SV=1 - [BAG2_HUMAN]	60.19	1	13	13	102	0.637	1.223	0.776	0.840	225.72	60.19	23	102	211	23.8	6.70
P13807	Glycogen [starch] synthase, muscle OS=Homo sapiens GN=GYS1 PE=1 SV=2 - [GYS1_HUMAN]	21.98	1	13	15	54	0.754	1.002	0.923	0.840	164.70	21.98	21	54	737	83.7	6.18
P36639	7,8-dihydro-8-oxoguanine triphosphatase OS=Homo sapiens GN=NUDT1 PE=1 SV=3 - [8ODP_HUMAN]	21.32	1	5	5	7	1.129	1.215	0.866	0.840	21.93	21.32	6	7	197	22.5	5.27
Q16555	Dihydropyrimidinase-related protein 2 OS=Homo sapiens GN=DPYSL2 PE=1 SV=1 - [DPYL2_HUMAN]	63.11	1	23	28	617	1.157	1.001	0.841	0.841	1665.56	63.11	49	617	572	62.3	6.38

P26639	Threonine--tRNA ligase, cytoplasmic OS=Homo sapiens GN=TARS PE=1 SV=3 - [SYTC_HUMAN]	45.50	1	25	29	146	0.921	0.735	0.764	0.841	352.28	45.50	49	146	723	83.4	6.67
Q9BSU1	UPF0183 protein C16orf70 OS=Homo sapiens GN=C16orf70 PE=1 SV=1 - [CP070_HUMAN]	2.37	1	1	1	2	1.170	0.931	0.920	0.841	5.15	2.37	2	2	422	47.5	7.72
P24468	COUP transcription factor 2 OS=Homo sapiens GN=NR2F2 PE=1 SV=1 - [COT2_HUMAN]	4.11	3	2	2	2	0.844	1.620	1.410	0.841	2.32	4.11	2	2	414	45.5	8.28
P0CW18	Serine protease 56 OS=Homo sapiens GN=PRSS56 PE=1 SV=1 - [PRS56_HUMAN	2.32	1	1	1	1	1.089	1.525	0.925	0.841	0.00	2.32	1	1	603	64.6	8.76
Q8WVK7	Spindle and kinetochore-associated protein 2 OS=Homo sapiens GN=SKA2 PE=1 SV=1 - [SKA2_HUMAN]	13.22	1	1	1	3	0.770	0.889	0.596	0.841	0.00	13.22	1	3	121	14.2	7.33
P07996	Thrombospondin-1 OS=Homo sapiens GN=THBS1 PE=1 SV=2 - [TSP1_HUMAN]	34.96	1	35	37	226	0.372	0.643	0.805	0.841	550.99	34.96	61	226	1170	129.3	4.94
Q3LFD5	Putative ubiquitin carboxyl-terminal hydrolase 41 OS=Homo sapiens GN=USP41 PE=2 SV=2 - [UBP41_HUMAN]	3.07	2	1	1	1	0.808	1.203	0.965	0.841	2.01	3.07	1	1	358	41.4	8.03
O60218	Aldo-keto reductase family 1 member B10 OS=Homo sapiens GN=AKR1B10 PE=1 SV=2 - [AK1BA_HUMAN	21.52	1	7	7	16	0.871	0.438	1.333	0.841	29.59	21.52	9	16	316	36.0	7.84
C9JLW8	Protein FAM195B OS=Homo sapiens GN=FAM195B PE=1 SV=1 - [F195B_HUMAN]	64.95	1	7	7	12	1.038	0.989	0.909	0.841	24.05	64.95	10	12	97	10.9	9.41

Q5JX69	Protein FAM209B OS=Homo sapiens GN=FAM209B PE=2 SV=1 - [F209B_HUMAN]	7.02	1	1	1	1	1.287	0.703	0.834	0.841	2.05	7.02	1	1	171	19.5	8.84
Q9BSU3	N-alpha-acetyltransferase 11 OS=Homo sapiens GN=NAA11 PE=1 SV=3 - [NAA11_HUMAN	13.54	1	1	3	6	1.048	1.640	5.359	0.841	22.77	13.54	3	6	229	26.0	5.17
Q17RQ9	NTPase KAP family P-loop domain-containing protein 1 OS=Homo sapiens GN=NKPD1 PE=2 SV=1 - [NKPD1_HUMAN]	2.79	1	1	1	1			1.259	0.841	2.74	2.79	1	1	610	67.7	8.85
Q5JSH3	WD repeat-containing protein 44 OS=Homo sapiens GN=WDR44 PE=1 SV=1 - [WDR44_HUMAN]	20.70	1	16	16	36	0.995	1.151	1.252	0.841	108.17	20.70	20	36	913	101.3	5.45
P54619	5'-AMP-activated protein kinase subunit gamma-1 OS=Homo sapiens GN=PRKAG1 PE=1 SV=1 - [AAKG1_HUMAN]	11.48	1	2	4	16	1.075	0.922	0.909	0.841	33.67	11.48	7	16	331	37.6	6.92
Q9Y5Q0	Fatty acid desaturase 3 OS=Homo sapiens GN=FADS3 PE=2 SV=1 - [FADS3_HUMAN]	4.72	1	2	2	2	0.645	0.708	1.303	0.841	6.20	4.72	2	2	445	51.1	7.77
Q8N4V1	Membrane magnesium transporter 1 OS=Homo sapiens GN=MMGT1 PE=1 SV=1 - [MMGT1_HUMAN]	18.32	1	1	1	12	0.790	1.041	0.862	0.842	18.31	18.32	2	12	131	14.7	9.16
Q8TDZ2	Protein-methionine sulfoxide oxidase MICAL1 OS=Homo sapiens GN=MICAL1 PE=1 SV=2 - [MICA1_HUMAN]	17.34	1	18	18	63	0.886	1.037	1.310	0.842	123.56	17.34	30	63	1067	117.8	6.40

Q9NWT1	p21-activated protein kinase-interacting protein 1 OS=Homo sapiens GN=PAK1IP1 PE=1 SV=2 - [PK1IP_HUMAN]	2.30	1	1	1	1	0.917	0.806	0.860	0.842	2.28	2.30	1	1	392	43.9	8.91
Q9UL33	Trafficking protein particle complex subunit 2-like protein OS=Homo sapiens GN=TRAPPC2L PE=1 SV=1 - [TPC2L_HUMAN]	12.14	1	2	2	9	1.141	1.258	1.080	0.842	19.85	12.14	2	9	140	16.1	6.77
Q8IUR0	Trafficking protein particle complex subunit 5 OS=Homo sapiens GN=TRAPPC5 PE=1 SV=1 - [TPPC5_HUMAN]	19.68	1	3	4	13	0.713	0.834	0.927	0.842	25.92	19.68	6	13	188	20.8	9.66
Q9NZI7	Upstream-binding protein 1 OS=Homo sapiens GN=UBP1 PE=2 SV=1 - [UBIP1_HUMAN]	8.70	1	2	3	10	0.624	0.965	0.893	0.842	35.67	8.70	4	10	540	60.5	6.35
Q9Y646	Carboxypeptidase Q OS=Homo sapiens GN=CPQ PE=1 SV=1 - [CBPQ_HUMAN]	22.25	2	9	9	28	0.808	0.905	1.075	0.842	70.60	22.25	12	28	472	51.9	6.18
A4UGR9	Xin actin-binding repeat-containing protein 2 OS=Homo sapiens GN=XIRP2 PE=1 SV=2 - [XIRP2_HUMAN]	1.16	1	4	4	4	0.921	0.627	0.625	0.843	7.67	1.16	4	4	3374	382.1	6.38
Q9UIQ6	Leucyl-cystinyl aminopeptidase OS=Homo sapiens GN=LNPEP PE=1 SV=3 - [LCAP_HUMAN]	12.78	1	12	12	19	1.123	0.832	0.924	0.843	36.89	12.78	16	19	1025	117.3	5.73
Q9P246	Stromal interaction molecule 2 OS=Homo sapiens GN=STIM2 PE=1 SV=2 - [STIM2_HUMAN]	8.98	1	5	6	12	1.681	0.901	0.876	0.843	36.36	8.98	6	12	746	83.9	6.76

Q86TN4	tRNA 2'-phosphotransferase 1 OS=Homo sapiens GN=TRPT1 PE=1 SV=2 - [TRPT1_HUMAN]	11.86	1	3	3	8	1.153	1.187	1.173	0.843	15.00	11.86	5	8	253	27.7	9.98
Q8IWB7	WD repeat and FYVE domain-containing protein 1 OS=Homo sapiens GN=WDFY1 PE=1 SV=1 - [WDFY1_HUMA	11.22	1	4	4	8	1.007	0.673	0.576	0.843	18.68	11.22	6	8	410	46.3	7.33
Q13505	Metaxin-1 OS=Homo sapiens GN=MTX1 PE=1 SV=2 - [MTX1_HUMAN]	10.73	1	4	4	12	0.656	0.718	0.455	0.843	33.36	10.73	6	12	466	51.4	9.79
P14324	Farnesyl pyrophosphate synthase OS=Homo sapiens GN=FDPS PE=1 SV=4 - [FPPS_HUMAN]	13.37	1	5	5	26	1.146	0.719	1.238	0.843	58.09	13.37	9	26	419	48.2	6.15
P61421	V-type proton ATPase subunit d 1 OS=Homo sapiens GN=ATP6V0D1 PE=1 SV=1 - [VA0D1_HUMAN]	17.66	1	7	7	28	0.959	0.716	0.655	0.843	70.00	17.66	13	28	351	40.3	5.00
Q6ICL3	Transport and Golgi organization protein 2 homolog OS=Homo sapiens GN=TANGO2 PE=2 SV=1 - [TNG2_HUMAN]	5.43	1	2	2	3	0.706	1.156	1.282	0.843	5.18	5.43	3	3	276	30.9	5.17
A6NIV6	Leucine-rich repeat and IQ domain-containing protein 4 OS=Homo sapiens GN=LRRIQ4 PE=2 SV=2 - [LRIQ4_HUMAN]	3.93	1	1	2	2	0.554	1.740	1.053	0.843	1.92	3.93	2	2	560	63.9	8.19
O60462	Neuropilin-2 OS=Homo sapiens GN=NRP2 PE=1 SV=2 - [NRP2_HUMAN]	4.73	1	4	4	6	0.741	2.817	0.762	0.843	13.92	4.73	5	6	931	104.8	5.17
P55285	Cadherin-6 OS=Homo sapiens GN=CDH6 PE=1 SV=1 - [CADH6_HUMAN]	3.16	1	2	2	4	1.083	0.736	0.913	0.843	10.54	3.16	3	4	790	88.3	4.93

Q8IU85	Calcium/calmodulin-dependent protein kinase type 1D OS=Homo sapiens GN=CAMK1D PE=1 SV=1 - [KCC1D_HUMAN]	31.17	1	8	11	24	0.885	0.809	0.861	0.844	49.83	31.17	16	24	385	42.9	7.21
Q9BQE3	Tubulin alpha-1C chain OS=Homo sapiens GN=TUBA1C PE=1 SV=1 - [TBA1C_HUMAN]	53.90	3	3	26	1101	1.010	1.000	1.126	0.844	3269.27	53.90	46	1101	449	49.9	5.10
Q96HQ0	Zinc finger protein 419 OS=Homo sapiens GN=ZNF419 PE=2 SV=2 - [ZN419_HUMAN]	1.37	1	1	1	6	1.042	0.844	0.665	0.844	13.72	1.37	1	6	510	58.5	8.98
Q8IV36	Protein HID1 OS=Homo sapiens GN=HID1 PE=1 SV=1 - [HID1_HUMAN]	2.03	1	2	2	3	1.074	1.001	0.914	0.844	8.05	2.03	2	3	788	88.7	6.06
Q9HCU5	Prolactin regulatory element-binding protein OS=Homo sapiens GN=PREB PE=1 SV=2 - [PREB_HUMAN]	25.90	1	7	7	25	0.646	0.780	0.956	0.844	43.67	25.90	11	25	417	45.4	7.88
Q86YW0	1-phosphatidylinositol 4,5-bisphosphate phosphodiesterase zeta-1 OS=Homo sapiens GN=PLCZ1 PE=2 SV=1 - [PLCZ1_HUMAN]	0.99	1	1	1	1	1.350	0.987	0.881	0.844	0.00	0.99	1	1	608	70.4	9.04
Q8TB36	Ganglioside-induced differentiation-associated protein 1 OS=Homo sapiens GN=GDAP1 PE=1 SV=3 - [GDAP1_HUMAN]	2.23	1	1	1	3	0.673	1.443	0.984	0.844	5.52	2.23	2	3	358	41.3	8.34
Q9Y490	Talin-1 OS=Homo sapiens GN=TLN1 PE=1 SV=3 - [TLN1_HUMAN]	61.47	3	124	148	1939	0.953	1.086	1.231	0.844	4995.92	61.47	245	1939	2541	269.6	6.07

P08912	Muscarinic acetylcholine receptor M5 OS=Homo sapiens GN=CHRM5 PE=2 SV=2 - [ACM5_HUMAN]	2.44	1	1	1	1	1.569	1.167	0.856	0.845	1.97	2.44	1	1	532	60.0	9.28
Q86YV9	Hermansky-Pudlak syndrome 6 protein OS=Homo sapiens GN=HPS6 PE=1 SV=1 - [HPS6_HUMAN]	4.65	1	4	4	7	0.990	0.814	1.000	0.845	11.60	4.65	5	7	775	82.9	6.28
Q6DKI1	60S ribosomal protein L7-like 1 OS=Homo sapiens GN=RPL7L1 PE=1 SV=1 - [RL7L_HUMAN]	13.41	1	3	3	5	0.759	1.577	1.292	0.845	10.94	13.41	4	5	246	28.6	10.51
Q15286	Ras-related protein Rab-35 OS=Homo sapiens GN=RAB35 PE=1 SV=1 - [RAB35_HUMAN]	58.21	3	8	12	78	1.102	1.140	0.989	0.845	185.70	58.21	19	78	201	23.0	8.29
Q8TBC4	NEDD8-activating enzyme E1 catalytic subunit OS=Homo sapiens GN=UBA3 PE=1 SV=2 - [UBA3_HUMAN]	30.24	1	12	12	54	0.894	0.848	0.817	0.845	157.36	30.24	19	54	463	51.8	5.45
Q5JPI3	Uncharacterized protein C3orf38 OS=Homo sapiens GN=C3orf38 PE=1 SV=1 - [CC038_HUMAN]	10.03	1	2	2	4	1.169	1.169	1.527	0.845	11.78	10.03	3	4	329	37.5	6.47
Q6ZR85	Uncharacterized protein C17orf107 OS=Homo sapiens GN=C17orf107 PE=2 SV=1 - [CQ107_HUMAN]	5.79	1	1	1	1	0.722	0.933	0.855	0.845	0.00	5.79	1	1	190	19.9	7.46
Q92538	Golgi-specific brefeldin A-resistance guanine nucleotide exchange factor 1 OS=Homo sapiens GN=GBF1 PE=1 SV=2 - [GBF1_HUMAN]	13.39	1	21	23	84	0.819	0.911	0.920	0.845	177.98	13.39	33	84	1859	206.3	5.73

Q9HCU0	Endosialin OS=Homo sapiens GN=CD248 PE=1 SV=1 - [CD248_HUMAN]	12.68	1	7	7	10	0.736	0.600	0.911	0.845	22.07	12.68	8	10	757	80.8	5.35
Q8IUH4	Palmitoyltransferase ZDHHC13 OS=Homo sapiens GN=ZDHHC13 PE=1 SV=3 - [ZDH13_HUMAN]	3.54	2	2	2	6	0.714	0.641	0.587	0.845	13.41	3.54	3	6	622	70.8	8.07
P04632	Calpain small subunit 1 OS=Homo sapiens GN=CAPNS1 PE=1 SV=1 - [CPNS1_HUMAN]	44.03	2	13	13	83	1.325	0.786	1.033	0.845	248.46	44.03	18	83	268	28.3	5.20
Q13884	Beta-1-syntrophin OS=Homo sapiens GN=SNTB1 PE=1 SV=3 - [SNTB1_HUMAN]	15.24	2	5	8	15	1.853	0.950	0.881	0.846	29.97	15.24	13	15	538	58.0	8.63
Q6Y2X3	DnaJ homolog subfamily C member 14 OS=Homo sapiens GN=DNAJC14 PE=2 SV=2 - [DJC14_HUMAN]	0.85	1	1	1	1	0.817	0.890	0.905	0.846	1.79	0.85	1	1	702	78.5	8.09
P41218	Myeloid cell nuclear differentiation antigen OS=Homo sapiens GN=MNDA PE=1 SV=1 - [MNDA_HUMAN]	3.19	1	2	2	4	3.763	0.794	0.838	0.846	11.27	3.19	2	4	407	45.8	9.76
Q8TD55	Pleckstrin homology domain-containing family O member 2 OS=Homo sapiens GN=PLEKHO2 PE=2 SV=1 - [PKHO2_HUMAN]	33.88	1	14	14	59	1.195	0.716	0.915	0.846	138.46	33.88	24	59	490	53.3	5.43
O60683	Peroxisome biogenesis factor 10 OS=Homo sapiens GN=PEX10 PE=1 SV=1 - [PEX10_HUMAN]	5.21	1	1	1	1	0.627	1.317	1.264	0.846	0.00	5.21	1	1	326	37.0	9.99
Q9H7T0	Cation channel sperm-associated protein subunit beta OS=Homo sapiens GN=CATSPERB PE=2 SV=2 - [CTSRB_HUMAN	1.25	1	1	1	2	1.282	0.491	1.383	0.846	4.13	1.25	1	2	1116	126.8	7.17

Q6P0Q8	Microtubule-associated serine/threonine-protein kinase 2 OS=Homo sapiens GN=MAST2 PE=1 SV=2 - [MAST2_HUMA	1.45	1	2	2	2	0.771	1.015	1.403	0.846	3.05	1.45	2	2	1798	196.3	8.16
Q9Y2I9	TBC1 domain family member 30 OS=Homo sapiens GN=TBC1D30 PE=1 SV=2 - [TBC30_HUMAN]	1.41	1	2	2	2	0.898	0.985	1.033	0.846	4.33	1.41	2	2	924	102.7	8.29
Q15738	Sterol-4-alpha-carboxylate 3-dehydrogenase, decarboxylating OS=Homo sapiens GN=NSDHL PE=1 SV=2 - [NSDHL_HUMAN	22.52	1	8	8	14	0.856	0.661	0.901	0.846	32.86	22.52	12	14	373	41.9	8.06
P22692	Insulin-like growth factor-binding protein 4 OS=Homo sapiens GN=IGFBP4 PE=1 SV=2 - [IBP4_HUMAN]	25.19	1	4	4	17	0.755	0.832	0.633	0.847	40.06	25.19	7	17	258	27.9	7.15
Q15382	GTP-binding protein Rheb OS=Homo sapiens GN=RHEB PE=1 SV=1 - [RHEB_HUMAN]	22.83	1	5	5	40	0.910	0.935	0.919	0.847	76.96	22.83	8	40	184	20.5	5.92
Q92871	Phosphomannomutase 1 OS=Homo sapiens GN=PMM1 PE=1 SV=2 - [PMM1_HUMAN]	29.77	1	6	7	22	1.058	0.955	1.537	0.847	43.23	29.77	11	22	262	29.7	5.74
P54920	Alpha-soluble NSF attachment protein OS=Homo sapiens GN=NAPA PE=1 SV=3 - [SNAA_HUMAN]	43.73	2	11	11	24	1.070	0.886	1.074	0.847	73.63	43.73	16	24	295	33.2	5.36
Q9H0R8	Gamma-aminobutyric acid receptor-associated protein-like 1 OS=Homo sapiens GN=GABARAPL1 PE=1 SV=1 - [GBRL1_HUMAN]	26.50	1	2	4	12	1.616	0.528	0.628	0.847	22.06	26.50	6	12	117	14.0	8.73

Q86SQ0	Pleckstrin homology-like domain family B member 2 OS=Homo sapiens GN=PHLDB2 PE=1 SV=2 - [PHLB2_HUMAN]	12.21	1	14	14	31	0.916	1.039	0.707	0.847	55.21	12.21	21	31	1253	142.1	7.43
Q9NYV6	RNA polymerase I-specific transcription initiation factor RRN3 OS=Homo sapiens GN=RRN3 PE=1 SV=1 - [RRN3_HUMAN]	3.99	1	2	2	2	0.790	2.110	1.739	0.847	3.84	3.99	2	2	651	74.1	5.63
Q7Z3U7	Protein MON2 homolog OS=Homo sapiens GN=MON2 PE=1 SV=3 - [MON2_HUMAN]	5.42	1	8	8	14	0.829	0.705	0.616	0.847	26.80	5.42	10	14	1717	190.2	6.06
O60343	TBC1 domain family member 4 OS=Homo sapiens GN=TBC1D4 PE=1 SV=2 - [TBCD4_HUMAN]	5.47	1	6	6	15	1.084	1.134	1.089	0.847	30.39	5.47	7	15	1298	146.5	7.01
P53621	Coatomer subunit alpha OS=Homo sapiens GN=COPA PE=1 SV=2 - [COPA_HUMAN]	37.50	1	51	51	414	0.769	0.767	0.772	0.847	930.63	37.50	84	414	1224	138.3	7.66
Q96JP0	Protein fem-1 homolog C OS=Homo sapiens GN=FEM1C PE=1 SV=1 - [FEM1C_HUMAN]	5.02	2	2	2	3	1.318	0.973	0.663	0.847	6.31	5.02	2	3	617	68.6	7.68
Q14247	Src substrate cortactin OS=Homo sapiens GN=CTTN PE=1 SV=2 - [SRC8_HUMAN]	52.18	1	36	36	240	0.998	1.177	1.006	0.847	632.88	52.18	65	240	550	61.5	5.40
Q92905	COP9 signalosome complex subunit 5 OS=Homo sapiens GN=COPS5 PE=1 SV=4 - [CSN5_HUMAN]	18.86	1	5	5	23	0.908	0.753	0.735	0.848	60.57	18.86	9	23	334	37.6	6.54
P27169	Serum paraoxonase/arylesterase 1 OS=Homo sapiens GN=PON1 PE=1 SV=3 - [PON1_HUMAN]	3.38	2	2	2	3	1.287	0.625	0.820	0.848	6.74	3.38	2	3	355	39.7	5.22

Q96EE3	Nudeoporin SEH1 OS=Homo sapiens GN=SEH1L PE=1 SV=3 - [SEH1_HUMAN]	17.22	1	3	3	6	1.151	0.380	0.498	0.848	27.08	17.22	4	6	360	39.6	8.09
Q9BQI9	Nudear receptor-interacting protein 2 OS=Homo sapiens GN=NRIP2 PE=2 SV=3 - [NRIP2_HUMAN	3.91	1	1	1	1	1.116	0.330	0.624	0.848	2.08	3.91	1	1	281	31.3	8.40
P40818	Ubiquitin carboxyl-terminal hydrolase 8 OS=Homo sapiens GN=USP8 PE=1 SV=1 - [UBP8_HUMAN]	7.07	1	8	8	12	0.897	0.910	1.100	0.848	23.78	7.07	11	12	1118	127.4	8.51
Q13045	Protein flightless-1 homolog OS=Homo sapiens GN=FLII PE=1 SV=2 - [FLII_HUMAN]	23.17	1	28	28	78	0.826	0.998	1.022	0.848	203.95	23.17	41	78	1269	144.7	6.05
P04234	T-cell surface glycoprotein CD3 delta chain OS=Homo sapiens GN=CD3D PE=1 SV=1 - [CD3D_HUMAN]	7.02	1	1	1	1	0.738	2.056	1.725	0.848	0.00	7.02	1	1	171	18.9	5.54
Q9H7C9	Mth938 domain-containing protein OS=Homo sapiens GN=AAMDC PE=1 SV=1 - [AAMDC_HUMA	52.46	1	6	6	18	0.915	1.105	0.924	0.848	47.22	52.46	8	18	122	13.3	8.46
P29317	Ephrin type-A receptor 2 OS=Homo sapiens GN=EPHA2 PE=1 SV=2 - [EPHA2_HUMAN]	13.42	7	9	11	18	1.649	0.721	1.088	0.849	35.25	13.42	18	18	976	108.2	6.23
Q9NXR1	Nudear distribution protein nudE homolog 1 OS=Homo sapiens GN=NDE1 PE=1 SV=2 - [NDE1_HUMAN]	13.29	1	4	5	11	1.069	1.406	1.044	0.849	26.59	13.29	7	11	346	38.8	5.27
P10586	Receptor-type tyrosine-protein phosphatase F OS=Homo sapiens GN=PTPRF PE=1 SV=2 - [PTPRF_HUMAN	6.14	2	4	7	11	0.883	0.929	0.933	0.849	20.94	6.14	9	11	1907	212.7	6.30

Q70Z35	Phosphatidylinositol 3,4,5-trisphosphate-dependent Rac exchanger 2 protein OS=Homo sapiens GN=PREX2 PE=2 SV=1 - [PREX2_HUMAN]	0.56	1	1	1	1	0.428	0.655	0.701	0.849	2.57	0.56	1	1	1606	182.5	7.44
Q13200	26S proteasome non-ATPase regulatory subunit 2 OS=Homo sapiens GN=PSMD2 PE=1 SV=3 - [PSMD2_HUMA	42.29	2	32	32	146	0.800	0.915	0.832	0.849	354.35	42.29	51	146	908	100.1	5.20
Q9H936	Mitochondrial glutamate carrier 1 OS=Homo sapiens GN=SLC25A22 PE=1 SV=1 - [GHC1_HUMAN]	17.34	2	6	6	18	0.852	0.887	0.993	0.849	36.83	17.34	10	18	323	34.4	9.29
Q96JJ7	Protein disulfide-isomerase TMX3 OS=Homo sapiens GN=TMX3 PE=1 SV=2 - [TMX3_HUMAN]	28.19	1	10	11	43	0.981	0.478	0.586	0.849	80.84	28.19	18	43	454	51.8	4.91
P35354	Prostaglandin G/H synthase 2 OS=Homo sapiens GN=PTGS2 PE=1 SV=2 - [PGH2_HUMAN]	5.63	1	2	2	7	1.009	1.283	1.379	0.849	29.57	5.63	3	7	604	69.0	7.39
Q01850	Cerebellar degeneration-related protein 2 OS=Homo sapiens GN=CDR2 PE=1 SV=2 - [CDR2_HUMAN]	17.84	1	6	6	14	0.737	0.773	0.844	0.849	35.91	17.84	9	14	454	51.8	5.06
Q8N3Y1	F-box/WD repeat-containing protein 8 OS=Homo sapiens GN=FBXW8 PE=1 SV=2 - [FBXW8_HUMAN]	2.01	1	1	1	2	0.670	0.543	0.981	0.850	3.97	2.01	2	2	598	67.4	5.67
Q5F1R6	DnaJ homolog subfamily C member 21 OS=Homo sapiens GN=DNAJC21 PE=1 SV=2 - [DJC21_HUMAN]	7.91	1	3	3	11	1.047	0.980	1.028	0.850	37.05	7.91	4	11	531	62.0	5.47

Q96IV0	Peptide-N(4)-(N-acetyl-beta-glucosaminyl)asparagine amidase OS=Homo sapiens GN=NGLY1 PE=1 SV=1 - [NGLY1_HUMAN]	5.35	1	4	4	10	0.689	1.211	1.175	0.850	16.84	5.35	6	10	654	74.3	6.89
Q14258	E3 ubiquitin/ISG15 ligase TRIM25 OS=Homo sapiens GN=TRIM25 PE=1 SV=2 - [TRI25_HUMAN]	34.13	1	18	18	76	1.101	0.864	0.907	0.850	164.53	34.13	30	76	630	70.9	8.09
Q9Y3R5	Protein dopey-2 OS=Homo sapiens GN=DOPEY2 PE=1 SV=5 - [DOP2_HUMAN]	1.83	1	3	4	7	0.740	1.097	1.441	0.851	14.42	1.83	5	7	2298	258.1	6.29
Q15019	Septin-2 OS=Homo sapiens GN=SEPT2 PE=1 SV=1 - [SEPT2_HUMAN]	75.35	1	21	22	323	0.923	1.078	1.135	0.851	907.18	75.35	41	323	361	41.5	6.60
Q9NQ92	Coordinator of PRMT5 and differentiation stimulator OS=Homo sapiens GN=COPRS PE=1 SV=3 - [COPRS_HUMAN]	6.52	1	1	1	1	0.584	0.964	1.136	0.851	3.13	6.52	1	1	184	20.1	4.18
Q93050	V-type proton ATPase 116 kDa subunit a isoform 1 OS=Homo sapiens GN=ATP6V0A1 PE=2 SV=3 - [VPP1_HUMAN]	21.15	1	12	13	32	0.922	0.961	1.070	0.851	86.70	21.15	21	32	837	96.4	6.43
Q6ZN11	Zinc finger protein 793 OS=Homo sapiens GN=ZNF793 PE=2 SV=2 - [ZN793_HUMAN]	2.96	1	1	1	2	0.604	0.636	0.736	0.851	0.00	2.96	1	2	406	46.9	9.33
Q6P9B9	Integrator complex subunit 5 OS=Homo sapiens GN=INTS5 PE=1 SV=1 - [INT5_HUMAN]	4.71	1	4	4	6	1.052	1.092	0.676	0.851	10.71	4.71	6	6	1019	107.9	7.05
O94830	Phospholipase DDHD2 OS=Homo sapiens GN=DDHD2 PE=1 SV=2 - [DDHD2_HUMAN]	1.97	1	1	1	6	1.534	1.502	0.833	0.851	14.85	1.97	1	6	711	81.0	5.39

Q99720	Sigma non-opioid intracellular receptor 1 OS=Homo sapiens GN=SIGMAR1 PE=1 SV=1 - [SGMR1_HUMAN]	12.11	1	3	3	12	0.704	0.992	0.717	0.851	24.40	12.11	5	12	223	25.1	5.96
Q9Y2V7	Conserved oligomeric Golgi complex subunit 6 OS=Homo sapiens GN=COG6 PE=1 SV=2 - [COG6_HUMAN]	6.39	1	2	4	12	0.807	1.256	1.576	0.851	18.33	6.39	6	12	657	73.2	5.76
Q9BS40	Latexin OS=Homo sapiens GN=LXN PE=1 SV=2 - [LXN_HUMAN]	18.92	1	5	5	76	0.834	1.904	1.170	0.852	116.58	18.92	7	76	222	25.7	5.78
Q13510	Acid ceramidase OS=Homo sapiens GN=ASAH1 PE=1 SV=5 - [ASAH1_HUMAN	37.97	1	18	18	74	0.630	1.040	0.901	0.852	185.14	37.97	27	74	395	44.6	7.62
P61160	Actin-related protein 2 OS=Homo sapiens GN=ACTR2 PE=1 SV=1 - [ARP2_HUMAN]	49.24	1	16	17	363	0.883	0.977	1.177	0.852	1001.22	49.24	29	363	394	44.7	6.74
Q08378	Golgin subfamily A member 3 OS=Homo sapiens GN=GOLGA3 PE=1 SV=2 - [GOGA3_HUMAN]	35.05	1	50	51	188	0.820	0.942	1.013	0.852	507.54	35.05	80	188	1498	167.3	5.44
Q9BZ67	FERM domain-containing protein 8 OS=Homo sapiens GN=FRMD8 PE=1 SV=1 - [FRMD8_HUMAN]	6.68	2	3	3	18	1.229	0.983	1.269	0.852	46.19	6.68	4	18	464	51.2	6.23
P07741	Adenine phosphoribosyltransferase OS=Homo sapiens GN=APRT PE=1 SV=2 - [APT_HUMAN]	45.56	1	5	5	39	1.037	0.994	0.989	0.852	102.94	45.56	9	39	180	19.6	6.02
Q9UJM3	ERBB receptor feedback inhibitor 1 OS=Homo sapiens GN=ERRFI1 PE=1 SV=1 - [ERRFI_HUMAN]	4.98	1	2	2	5	0.639	0.658	1.019	0.852	6.01	4.98	3	5	462	50.5	8.10

P0C0L4	Complement C4-A OS=Homo sapiens GN=C4A PE=1 SV=2 - [CO4A_HUMAN]	4.19	2	4	5	17	0.680	1.003	0.657	0.852	31.45	4.19	7	17	1744	192.7	7.08
P30085	UMP-CMP kinase OS=Homo sapiens GN=CMPK1 PE=1 SV=3 - [KCY_HUMAN]	48.47	1	9	9	87	0.973	0.886	1.039	0.852	305.03	48.47	14	87	196	22.2	5.57
P30046	D-dopachrome decarboxylase OS=Homo sapiens GN=DDT PE=1 SV=3 - [DOPD_HUMAN]	22.03	2	3	3	8	0.910	1.040	0.917	0.852	18.47	22.03	5	8	118	12.7	7.30
P07148	Fatty acid-binding protein, liver OS=Homo sapiens GN=FABP1 PE=1 SV=1 - [FABPL_HUMAN	6.30	1	1	1	1	1.396	0.877	0.551	0.852	1.66	6.30	1	1	127	14.2	7.18
Q8IUI8	Cytokine receptor-like factor 3 OS=Homo sapiens GN=CRLF3 PE=1 SV=2 - [CRLF3_HUMAN]	5.20	1	2	2	5	1.092	1.028	0.850	0.852	12.19	5.20	4	5	442	49.7	5.14
Q53H82	Beta-lactamase-like protein 2 OS=Homo sapiens GN=LACTB2 PE=1 SV=2 - [LACB2_HUMAN]	38.89	1	10	10	29	1.069	1.184	1.022	0.852	73.82	38.89	15	29	288	32.8	6.80
Q9BU89	Deoxyhypusine hydroxylase OS=Homo sapiens GN=DOHH PE=1 SV=1 - [DOHH_HUMAN]	24.50	1	4	4	7	1.024	1.388	1.042	0.852	23.23	24.50	5	7	302	32.9	4.83
O15511	Actin-related protein 2/3 complex subunit 5 OS=Homo sapiens GN=ARPC5 PE=1 SV=3 - [ARPC5_HUMAN]	55.63	1	8	10	85	1.055	1.134	1.164	0.853	206.26	55.63	17	85	151	16.3	5.67
P19105	Myosin regulatory light chain 12A OS=Homo sapiens GN=MYL12A PE=1 SV=2 - [ML12A_HUMAN]	66.67	2	5	13	494	1.191	0.837	1.306	0.853	1309.61	66.67	22	494	171	19.8	4.81

P78329	Phylloquinone omega-hydroxylase CYP4F2 OS=Homo sapiens GN=CYP4F2 PE=1 SV=1 - [CP4F2_HUMAN]	2.69	2	2	2	3	1.498	0.828	0.808	0.853	6.74	2.69	2	3	520	59.8	7.08
Q9BXK1	Krueppel-like factor 16 OS=Homo sapiens GN=KLF16 PE=1 SV=1 - [KLF16_HUMAN]	21.43	1	3	3	6	0.912	0.812	0.957	0.853	20.81	21.43	4	6	252	25.4	9.88
Q13162	Peroxiredoxin-4 OS=Homo sapiens GN=PRDX4 PE=1 SV=1 - [PRDX4_HUMAN]	55.35	1	10	13	192	0.818	1.001	1.085	0.853	466.60	55.35	21	192	271	30.5	6.29
Q9Y224	UPF0568 protein C14orf166 OS=Homo sapiens GN=C14orf166 PE=1 SV=1 - [CN166_HUMAN]	59.84	1	13	13	70	0.837	0.952	1.061	0.853	196.43	59.84	25	70	244	28.1	6.65
P47756	F-actin-capping protein subunit beta OS=Homo sapiens GN=CAPZB PE=1 SV=4 - [CAPZB_HUMAN	48.74	1	19	19	287	0.972	0.962	0.903	0.853	629.05	48.74	34	287	277	31.3	5.59
P10599	Thioredoxin OS=Homo sapiens GN=TXN PE=1 SV=3 - [THIO_HUMAN]	42.86	1	7	7	202	1.158	0.685	0.947	0.853	571.17	42.86	11	202	105	11.7	4.92
Q9NT62	Ubiquitin-like-conjugating enzyme ATG3 OS=Homo sapiens GN=ATG3 PE=1 SV=1 - [ATG3_HUMAN]	19.11	1	7	7	31	1.046	0.712	0.945	0.853	58.12	19.11	12	31	314	35.8	4.74
Q16342	Programmed cell death protein 2 OS=Homo sapiens GN=PDCD2 PE=1 SV=2 - [PDCD2_HUMAN	6.40	1	2	2	2	0.797	0.740	1.086	0.853	1.90	6.40	2	2	344	38.6	5.38
Q15751	Probable E3 ubiquitin-protein ligase HERC1 OS=Homo sapiens GN=HERC1 PE=1 SV=2 - [HERC1_HUMAN]	0.78	1	3	3	5	0.708	0.956	0.929	0.854	12.92	0.78	5	5	4861	531.9	6.04

Q6PCB5	Round spermatid basic protein 1-like protein OS=Homo sapiens GN=RSBN1L PE=1 SV=2 - [RSBNL_HUMAN]	0.83	1	1	1	1	1.265	1.824	0.994	0.854	2.38	0.83	1	1	846	94.8	8.78
O76095	Protein JTB OS=Homo sapiens GN=JTB PE=1 SV=1 - [JTB_HUMAN]	13.70	1	2	2	4	0.940	1.413	1.013	0.854	11.10	13.70	3	4	146	16.3	8.28
O95249	Golgi SNAP receptor complex member 1 OS=Homo sapiens GN=GOSR1 PE=1 SV=1 - [GOSR1_HUMA N]	22.80	1	5	6	12	0.890	0.801	0.876	0.854	26.45	22.80	7	12	250	28.6	9.42
Q6WCQ1	Myosin phosphatase Rho-interacting protein OS=Homo sapiens GN=MPRIP PE=1 SV=3 - [MPRIP_HUMAN]	48.10	1	39	40	159	1.108	0.978	1.132	0.854	429.98	48.10	65	159	1025	116.5	6.21
P46459	Vesicle-fusing ATPase OS=Homo sapiens GN=NSF PE=1 SV=3 - [NSF_HUMAN]	39.92	1	30	31	121	0.925	0.863	0.999	0.854	260.26	39.92	50	121	744	82.5	6.95
Q9NYB5	Solute carrier organic anion transporter family member 1C1 OS=Homo sapiens GN=SLCO1C1 PE=2 SV=1 - [SO1C1_HUMA N]	2.25	1	1	1	1	2.465	0.451	2.036	0.854	0.00	2.25	1	1	712	78.6	8.50
Q5TCZ1	SH3 and PX domain-containing protein 2A OS=Homo sapiens GN=SH3PXD2A PE=1 SV=1 - [SPD2A_HUMAN]	10.59	1	8	9	18	0.964	0.898	0.899	0.854	30.74	10.59	15	18	1133	125.2	8.90
Q9NU53	Glycoprotein integral membrane protein 1 OS=Homo sapiens GN=GINM1 PE=2 SV=1 - [GINM1_HUMAN]	3.03	1	1	1	1	1.203	0.338	0.809	0.854	3.07	3.03	1	1	330	36.8	4.91

Q9GZT9	Egl nine homolog 1 OS=Homo sapiens GN=EGLN1 PE=1 SV=1 - [EGLN1_HUMAN]	22.07	2	7	7	30	0.998	1.077	1.067	0.854	77.94	22.07	11	30	426	46.0	8.53
P55212	Caspase-6 OS=Homo sapiens GN=CASP6 PE=1 SV=2 - [CASP6_HUMAN]	3.41	1	1	1	4	1.478	1.228	0.848	0.854	12.64	3.41	2	4	293	33.3	6.93
P28072	Proteasome subunit beta type-6 OS=Homo sapiens GN=PSMB6 PE=1 SV=4 - [PSB6_HUMAN]	12.97	1	3	3	6	0.805	0.848	0.843	0.854	14.51	12.97	4	6	239	25.3	4.92
O14656	Torsin-1A OS=Homo sapiens GN=TOR1A PE=1 SV=1 - [TOR1A_HUMAN]	11.75	1	3	3	11	0.928	0.737	0.881	0.854	28.43	11.75	5	11	332	37.8	6.99
O15204	ADAM DEC1 OS=Homo sapiens GN=ADAMDEC1 PE=1 SV=2 - [ADEC1_HUMAN]	5.53	1	2	2	3	0.818	0.833	0.976	0.854	2.69	5.53	2	3	470	52.7	7.34
Q96SB4	SRSF protein kinase 1 OS=Homo sapiens GN=SRPK1 PE=1 SV=2 - [SRPK1_HUMAN]	14.96	1	6	7	22	0.743	1.118	0.891	0.854	61.56	14.96	12	22	655	74.3	6.16
Q9Y4C2	Protein FAM115A OS=Homo sapiens GN=FAM115A PE=1 SV=3 - [F115A_HUMAN]	9.55	1	9	9	31	1.043	0.707	1.149	0.855	76.69	9.55	15	31	921	102.1	6.54
Q9NZH7	Interleukin-36 beta OS=Homo sapiens GN=IL36B PE=2 SV=1 - [IL36B_HUMAN]	6.10	1	1	1	1	1.228	0.423	0.821	0.855	2.41	6.10	1	1	164	18.5	9.52
Q9GZZ9	Ubiquitin-like modifier-activating enzyme 5 OS=Homo sapiens GN=UBA5 PE=1 SV=1 - [UBA5_HUMAN]	6.93	2	2	3	16	0.834	0.952	0.856	0.855	41.44	6.93	5	16	404	44.8	4.84
O15031	Plexin-B2 OS=Homo sapiens GN=PLXNB2 PE=1 SV=3 - [PLXB2_HUMAN]	19.53	1	26	26	86	1.242	0.890	0.775	0.855	198.89	19.53	38	86	1838	205.0	6.24

O95803	Bifunctional heparan sulfate N-deacetylase/N-sulfotransferase 3 OS=Homo sapiens GN=NDST3 PE=2 SV=1 - [NDST3_HUMAN	0.80	1	1	1	6	0.737	0.869	0.930	0.855	13.57	0.80	1	6	873	100.8	8.06
Q6UWE0	E3 ubiquitin-protein ligase LRSAM1 OS=Homo sapiens GN=LRSAM1 PE=1 SV=1 - [LRSM1_HUMAN]	17.84	1	12	13	40	1.081	1.186	1.384	0.855	107.92	17.84	21	40	723	83.5	5.94
Q15653	NF-kappa-B inhibitor beta OS=Homo sapiens GN=NFKBIB PE=1 SV=2 - [IKBB_HUMAN]	3.65	1	1	1	11	0.763	0.640	0.970	0.855	30.20	3.65	2	11	356	37.7	4.84
Q9NXA8	NAD-dependent protein deacylase sirtuin-5, mitochondrial OS=Homo sapiens GN=SIRT5 PE=1 SV=2 - [SIR5_HUMAN]	21.61	1	5	5	12	0.653	1.071	1.318	0.856	33.83	21.61	7	12	310	33.9	8.47
Q6V1X1	Dipeptidyl peptidase 8 OS=Homo sapiens GN=DPP8 PE=1 SV=1 - [DPP8_HUMAN]	9.80	1	5	6	13	0.946	0.907	1.191	0.856	31.50	9.80	7	13	898	103.3	5.80
Q99816	Tumor susceptibility gene 101 protein OS=Homo sapiens GN=TSG101 PE=1 SV=2 - [TS101_HUMAN]	17.18	1	7	7	25	0.991	1.080	1.184	0.856	62.04	17.18	11	25	390	43.9	6.46
Q13724	Mannosyl-oligosaccharide glucosidase OS=Homo sapiens GN=MOGS PE=1 SV=5 - [MOGS_HUMAN]	29.63	1	19	19	68	0.766	0.795	0.744	0.856	185.28	29.63	30	68	837	91.9	8.90
Q9P2D0	Inhibitor of Bruton tyrosine kinase OS=Homo sapiens GN=IBTK PE=1 SV=3 - [IBTK_HUMAN]	1.26	1	2	2	4	0.814	0.931	0.589	0.856	10.54	1.26	3	4	1353	150.4	7.71

O15427	Monocarboxylate transporter 4 OS=Homo sapiens GN=SLC16A3 PE=1 SV=1 - [MOT4_HUMAN]	11.61	1	4	4	33	1.756	0.741	0.731	0.856	85.55	11.61	7	33	465	49.4	7.96
O15372	Eukaryotic translation initiation factor 3 subunit H OS=Homo sapiens GN=EIF3H PE=1 SV=1 - [EIF3H_HUMAN]	27.56	1	10	10	32	0.768	0.680	0.657	0.856	80.44	27.56	15	32	352	39.9	6.54
Q13425	Beta-2-syntrophin OS=Homo sapiens GN=SNTB2 PE=1 SV=1 - [SNTB2_HUMAN]	37.96	3	17	20	70	1.158	1.073	1.141	0.856	137.56	37.96	32	70	540	57.9	8.82
P84095	Rho-related GTP-binding protein RhoG OS=Homo sapiens GN=RHOG PE=1 SV=1 - [RHOG_HUMAN]	51.31	2	8	9	41	1.063	1.083	0.857	0.856	97.09	51.31	16	41	191	21.3	8.12
O43683	Mitotic checkpoint serine/threonine-protein kinase BUB1 OS=Homo sapiens GN=BUB1 PE=1 SV=1 - [BUB1_HUMAN]	3.04	1	2	2	2	1.396	0.410	0.947	0.856	3.23	3.04	2	2	1085	122.3	6.47
Q04941	Proteolipid protein 2 OS=Homo sapiens GN=PLP2 PE=1 SV=1 - [PLP2_HUMAN]	8.55	1	1	1	14	1.316	1.079	1.006	0.857	39.78	8.55	2	14	152	16.7	7.24
Q93063	Exostosin-2 OS=Homo sapiens GN=EXT2 PE=1 SV=1 - [EXT2_HUMAN]	0.97	1	1	1	3	0.794	0.959	1.024	0.857	6.41	0.97	1	3	718	82.2	6.55
Q8N2K0	Monoacylglycerol lipase ABHD12 OS=Homo sapiens GN=ABHD12 PE=1 SV=2 - [ABD12_HUMAN]	22.11	1	8	8	19	0.776	1.286	1.075	0.857	54.95	22.11	11	19	398	45.1	8.65
Q96S19	UPF0585 protein C16orf13 OS=Homo sapiens GN=C16orf13 PE=1 SV=2 - [CP013_HUMAN]	32.84	1	6	6	14	0.818	1.139	1.088	0.857	33.66	32.84	10	14	204	22.6	7.83

Q92616	Translational activator GCN1 OS=Homo sapiens GN=GCN1L1 PE=1 SV=6 - [GCN1L_HUMAN]	29.91	1	78	79	237	0.866	0.890	0.874	0.857	563.29	29.91	126	237	2671	292.6	7.47
Q13555	Calcium/calmodulin-dependent protein kinase type II subunit gamma OS=Homo sapiens GN=CAMK2G PE=1 SV=3 - [KCC2G_HUMAN]	16.85	3	3	10	35	1.708	1.257	1.636	0.857	79.83	16.85	13	35	558	62.6	7.83
Q9BVT8	Transmembrane and ubiquitin-like domain-containing protein 1 OS=Homo sapiens GN=TMUB1 PE=1 SV=1 - [TMUB1_HUMAN]	8.94	1	2	2	6	0.876	0.982	1.061	0.857	14.02	8.94	3	6	246	26.2	5.72
Q13155	Aminoacyl tRNA synthase complex-interacting multifunctional protein 2 OS=Homo sapiens GN=AIMP2 PE=1 SV=2 - [AIMP2_HUMAN]	35.00	1	11	11	40	0.885	0.861	0.894	0.857	97.86	35.00	18	40	320	35.3	8.22
Q6NUM9	All-trans-retinol 13,14-reductase OS=Homo sapiens GN=RETSAT PE=1 SV=2 - [RETST_HUMAN	24.75	1	16	16	44	1.277	0.801	1.071	0.857	100.95	24.75	28	44	610	66.8	8.28
O00443	Phosphatidylinositol 4-phosphate 3-kinase C2 domain-containing subunit alpha OS=Homo sapiens GN=PIK3C2A PE=1 SV=2 - [P3C2A_HUMAN]	5.69	1	9	10	26	0.932	0.918	0.930	0.858	45.66	5.69	16	26	1686	190.6	8.02
Q9HA77	Probable cysteine--tRNA ligase, mitochondrial OS=Homo sapiens GN=CARS2 PE=1 SV=1 - [SYCM_HUMAN]	12.06	1	6	6	17	0.957	1.675	1.166	0.858	36.68	12.06	12	17	564	62.2	8.34
Q13608	Peroxisome assembly factor 2 OS=Homo sapiens GN=PEX6 PE=1 SV=2 - [PEX6_HUMAN]	3.67	1	2	2	3	0.801	1.373	1.090	0.858	7.71	3.67	3	3	980	104.0	6.34

Q12765	Secernin-1 OS=Homo sapiens GN=SCRN1 PE=1 SV=2 - [SCRN1_HUMAN]	51.69	1	16	16	92	0.943	0.823	0.923	0.858	229.26	51.69	26	92	414	46.4	4.75
Q7Z7F7	39S ribosomal protein L55, mitochondrial OS=Homo sapiens GN=MRPL55 PE=1 SV=1 - [RM55_HUMAN]	12.50	1	1	1	2	1.062	1.358	1.657	0.858	4.79	12.50	2	2	128	15.1	11.15
Q01974	Tyrosine-protein kinase transmembrane receptor ROR2 OS=Homo sapiens GN=ROR2 PE=1 SV=2 - [ROR2_HUMAN]	3.92	1	3	3	9	1.883	1.631	0.549	0.858	19.55	3.92	5	9	943	104.7	6.55
P52824	Diacylglycerol kinase theta OS=Homo sapiens GN=DGKQ PE=1 SV=2 - [DGKQ_HUMAN]	0.96	1	1	1	2	0.871	1.024	1.428	0.858	3.93	0.96	1	2	942	101.1	7.49
Q9NXU5	ADP-ribosylation factor-like protein 15 OS=Homo sapiens GN=ARL15 PE=1 SV=1 - [ARL15_HUMAN]	4.41	1	1	1	4	0.536	0.867	0.922	0.858	9.71	4.41	2	4	204	22.9	5.63
Q9HB20	Pleckstrin homology domain-containing family A member 3 OS=Homo sapiens GN=PLEKHA3 PE=1 SV=2 - [PKHA3_HUMAN]	14.33	1	3	3	3	1.324	0.856	0.808	0.858	9.46	14.33	3	3	300	33.8	6.65
Q7L5Y1	Mitochondrial enolase superfamily member 1 OS=Homo sapiens GN=ENOSF1 PE=1 SV=1 - [ENOF1_HUMAN]	4.29	1	2	2	6	1.039	0.945	0.658	0.858	11.19	4.29	3	6	443	49.8	6.48
Q8N573	Oxidation resistance protein 1 OS=Homo sapiens GN=OXR1 PE=1 SV=2 - [OXR1_HUMAN]	12.36	1	9	9	29	1.219	0.809	1.575	0.858	86.31	12.36	13	29	874	97.9	5.47

Q9BZG1	Ras-related protein Rab-34 OS=Homo sapiens GN=RAB34 PE=1 SV=1 - [RAB34_HUMAN]	34.36	1	7	8	19	1.089	0.662	0.665	0.858	54.18	34.36	10	19	259	29.0	7.88
Q8IWA0	WD repeat-containing protein 75 OS=Homo sapiens GN=WDR75 PE=1 SV=1 - [WDR75_HUMAN]	2.29	1	1	1	3	0.826	1.023	0.928	0.858	9.15	2.29	2	3	830	94.4	5.96
Q9NSB4	Keratin, type II cuticular Hb2 OS=Homo sapiens GN=KRT82 PE=1 SV=3 - [KRT82_HUMAN]	5.65	1	3	3	3	1.192	0.824	0.834	0.858	7.49	5.65	3	3	513	56.6	6.74
Q9BXY0	Protein MAK16 homolog OS=Homo sapiens GN=MAK16 PE=1 SV=2 - [MAK16_HUMAN]	6.33	1	1	1	3	0.522	1.928	0.834	0.859	2.86	6.33	2	3	300	35.3	5.38
O75718	Cartilage-associated protein OS=Homo sapiens GN=CRTAP PE=1 SV=1 - [CRTAP_HUMAN]	39.65	1	16	16	82	0.735	0.636	0.737	0.859	210.81	39.65	26	82	401	46.5	5.73
Q8TAD7	Overexpressed in colon carcinoma 1 protein OS=Homo sapiens GN=OCC1 PE=1 SV=2 - [OCC1_HUMAN]	22.22	1	1	1	4	0.876	0.931	1.075	0.859	12.27	22.22	1	4	63	6.4	6.57
Q9HBU1	Homeobox protein BarH-like 1 OS=Homo sapiens GN=BARX1 PE=1 SV=2 - [BARX1_HUMAN]	5.91	1	1	1	1	0.974	1.002	2.712	0.859	0.00	5.91	1	1	254	27.3	9.70
Q9NRG1	Phosphoribosyltransferase domain-containing protein 1 OS=Homo sapiens GN=PRTFDC1 PE=1 SV=1 - [PRDC1_HUMAN]	9.33	1	2	2	7	1.270	0.963	0.570	0.859	15.47	9.33	4	7	225	25.7	6.15

Q08188	Protein-glutamine gamma-glutamyltransferase E OS=Homo sapiens GN=TGM3 PE=1 SV=4 - [TGM3_HUMAN]	1.30	1	1	1	1	0.862	0.441	1.130	0.859	1.90	1.30	1	1	693	76.6	5.86
Q9Y6Z5	Putative uncharacterized protein MLLT4-AS1 OS=Homo sapiens GN=MLLT4-AS1 PE=5 SV=1 - [MLAS1_HUMAN]	10.63	1	1	1	1	0.883	1.270	1.864	0.859	2.94	10.63	1	1	254	26.1	11.81
O15382	Branched-chain-amino-acid aminotransferase, mitochondrial OS=Homo sapiens GN=BCAT2 PE=1 SV=2 - [BCAT2_HUMAN	15.82	1	6	6	13	0.963	1.460	0.939	0.859	27.04	15.82	9	13	392	44.3	8.65
P35609	Alpha-actinin-2 OS=Homo sapiens GN=ACTN2 PE=1 SV=1 - [ACTN2_HUMAN]	20.36	1	1	22	541	1.171	0.998	1.170	0.859	1569.29	20.36	40	541	894	103.8	5.45
P41162	ETS translocation variant 3 OS=Homo sapiens GN=ETV3 PE=1 SV=2 - [ETV3_HUMAN]	3.71	1	1	1	1	1.074	0.956	1.396	0.859	2.68	3.71	1	1	512	57.0	8.57
P35579	Myosin-9 OS=Homo sapiens GN=MYH9 PE=1 SV=4 - [MYH9_HUMAN]	73.98	1	182	223	5912	1.131	0.953	1.262	0.859	#####	73.98	369	5912	1960	226.4	5.60
Q8IXI2	Mitochondrial Rho GTPase 1 OS=Homo sapiens GN=RHOT1 PE=1 SV=2 - [MIRO1_HUMAN]	7.93	1	4	5	8	0.937	0.892	0.751	0.859	10.89	7.93	6	8	618	70.7	6.27
O15037	Protein KHNYN OS=Homo sapiens GN=KHNYN PE=2 SV=3 - [KHNYN_HUMAN]	4.42	1	2	2	2	1.007	0.696	0.931	0.860	1.94	4.42	2	2	678	74.5	7.02
Q9H9Y6	DNA-directed RNA polymerase I subunit RPA2 OS=Homo sapiens GN=POLR1B PE=1 SV=2 - [RPA2_HUMAN]	0.97	1	1	1	2	0.707	0.737	0.805	0.860	3.44	0.97	2	2	1135	128.1	7.83

P48449	Lanosterol synthase OS=Homo sapiens GN=LSS PE=1 SV=1 - [ERG7_HUMAN]	26.23	1	16	16	46	1.248	0.947	1.253	0.860	100.73	26.23	28	46	732	83.3	6.61
Q9BRK3	Matrix-remodeling-associated protein 8 OS=Homo sapiens GN=MXRA8 PE=2 SV=1 - [MXRA8_HUMAN]	19.46	1	8	9	33	0.586	0.666	0.926	0.860	76.54	19.46	16	33	442	49.1	7.23
Q9Y2Y0	ADP-ribosylation factor-like protein 2-binding protein OS=Homo sapiens GN=ARL2BP PE=1 SV=1 - [AR2BP_HUMAN]	17.18	1	3	3	13	0.772	0.915	0.975	0.860	26.07	17.18	6	13	163	18.8	4.35
P34897	Serine hydroxymethyltransferase, mitochondrial OS=Homo sapiens GN=SHMT2 PE=1 SV=3 - [GLYM_HUMAN]	40.08	1	18	19	105	0.775	1.093	0.846	0.860	280.09	40.08	34	105	504	56.0	8.53
P63244	Guanine nucleotide-binding protein subunit beta-2-like 1 OS=Homo sapiens GN=GNB2L1 PE=1 SV=3 - [GBLP_HUMAN]	60.57	1	16	16	108	0.748	0.728	0.712	0.860	308.73	60.57	25	108	317	35.1	7.69
Q9NQG1	Protein MANBAL OS=Homo sapiens GN=MANBAL PE=1 SV=1 - [MANBL_HUMAN]	9.41	1	1	1	1	1.002	0.730	0.660	0.860	1.89	9.41	1	1	85	9.5	9.16
Q969M3	Protein YIPF5 OS=Homo sapiens GN=YIPF5 PE=1 SV=1 - [YIPF5_HUMAN]	8.17	1	2	2	7	0.674	0.653	0.724	0.860	13.51	8.17	3	7	257	28.0	4.36
Q562F6	Shugoshin-like 2 OS=Homo sapiens GN=SGOL2 PE=1 SV=2 - [SGOL2_HUMAN]	0.55	1	1	1	1	1.287	1.266	0.738	0.860	1.96	0.55	1	1	1265	144.6	7.91
P20742	Pregnancy zone protein OS=Homo sapiens GN=PZP PE=1 SV=4 - [PZP_HUMAN]	2.56	1	2	3	15	1.278	0.951	1.491	0.860	32.01	2.56	4	15	1482	163.8	6.38

Q9GZQ3	COMM domain-containing protein 5 OS=Homo sapiens GN=COMMD5 PE=1 SV=1 - [COMD5_HUMAN]	16.96	1	4	4	9	0.983	0.846	0.827	0.861	20.43	16.96	5	9	224	24.7	7.02
Q9NX58	Cell growth-regulating nucleolar protein OS=Homo sapiens GN=LYAR PE=1 SV=2 - [LYAR_HUMAN]	15.83	1	5	5	13	1.054	0.818	0.684	0.861	47.51	15.83	8	13	379	43.6	9.54
Q6ZN16	Mitogen-activated protein kinase kinase kinase 15 OS=Homo sapiens GN=MAP3K15 PE=1 SV=2 - [M3K15_HUMAN]	0.69	1	1	1	1	0.675	0.979	1.166	0.861	2.35	0.69	1	1	1313	147.3	5.63
Q15181	Inorganic pyrophosphatase OS=Homo sapiens GN=PPA1 PE=1 SV=2 - [IPYR_HUMAN]	64.36	1	18	20	108	0.967	0.693	0.666	0.861	251.64	64.36	34	108	289	32.6	5.86
O00622	Protein CYR61 OS=Homo sapiens GN=CYR61 PE=1 SV=1 - [CYR61_HUMAN]	19.42	1	7	7	19	0.451	0.571	0.578	0.861	41.81	19.42	12	19	381	42.0	8.21
Q9NXN4	Ganglioside-induced differentiation-associated protein 2 OS=Homo sapiens GN=GDAP2 PE=1 SV=1 - [GDAP2_HUMAN]	4.02	1	2	2	3	1.263	3.046	1.405	0.861	6.21	4.02	3	3	497	56.2	5.74
Q15785	Mitochondrial import receptor subunit TOM34 OS=Homo sapiens GN=TOMM34 PE=1 SV=2 - [TOM34_HUMA	32.04	1	7	8	32	0.816	1.112	1.073	0.861	78.28	32.04	12	32	309	34.5	8.98
O95067	G2/mitotic-specific cyclin-B2 OS=Homo sapiens GN=CCNB2 PE=1 SV=1 - [CCNB2_HUMAN]	1.51	1	1	1	1	0.684	1.468	1.096	0.861	1.83	1.51	1	1	398	45.3	8.90
O14579	Coatomer subunit epsilon OS=Homo sapiens GN=COPE PE=1 SV=3 - [COPE_HUMAN]	25.32	1	10	10	37	0.896	0.741	0.707	0.862	87.44	25.32	17	37	308	34.5	5.12

Q8TDY2	RB1-inducible coiled-coil protein 1 OS=Homo sapiens GN=RB1CC1 PE=1 SV=3 - [RBCC1_HUMAN]	4.52	1	6	8	21	0.724	1.354	1.204	0.862	49.83	4.52	11	21	1594	183.0	5.41
O43314	Inositol hexakisphosphate and diphosphoinositol-pentakisphosphate kinase 2 OS=Homo sapiens GN=PPIP5K2 PE=1 SV=3 - [VIP2_HUMAN]	10.78	1	6	13	25	0.783	0.951	1.019	0.862	64.29	10.78	18	25	1243	140.3	8.22
Q8N4L2	Type 2 phosphatidylinositol 4,5-bisphosphate 4-phosphatase OS=Homo sapiens GN=TMEM55A PE=1 SV=1 - [TM55A_HUMAN]	3.89	1	1	1	1	0.648	0.369	0.616	0.862	0.00	3.89	1	1	257	28.1	8.68
Q6P9B6	TLD domain-containing protein 1 OS=Homo sapiens GN=TLDC1 PE=1 SV=2 - [TLDC1_HUMAN]	18.20	1	6	6	24	0.992	0.964	1.146	0.862	56.98	18.20	8	24	456	51.0	6.24
Q9BTW9	Tubulin-specific chaperone D OS=Homo sapiens GN=TBCD PE=1 SV=2 - [TBCD_HUMAN]	12.75	1	13	13	44	0.829	0.803	0.833	0.862	110.95	12.75	21	44	1192	132.5	6.19
P12814	Alpha-actinin-1 OS=Homo sapiens GN=ACTN1 PE=1 SV=2 - [ACTN1_HUMAN]	70.07	1	42	71	2565	1.222	1.150	1.305	0.862	6757.28	70.07	122	2565	892	103.0	5.41
Q9H579	Protein MROH8 OS=Homo sapiens GN=MROH8 PE=2 SV=2 - [MROH8_HUMAN]	1.45	1	1	1	2	1.050	0.699	0.648	0.862	4.65	1.45	1	2	483	54.8	6.40
Q96FI4	Endonuclease 8-like 1 OS=Homo sapiens GN=NEIL1 PE=1 SV=3 - [NEIL1_HUMAN]	2.56	1	1	1	1	0.631	1.097	0.526	0.862	2.01	2.56	1	1	390	43.7	9.92
Q14112	Nidogen-2 OS=Homo sapiens GN=NID2 PE=1 SV=3 - [NID2_HUMAN]	8.95	1	9	11	17	0.504	1.375	0.811	0.863	44.31	8.95	16	17	1375	151.2	5.29

Q9UBF2	Coatomer subunit gamma-2 OS=Homo sapiens GN=COPG2 PE=1 SV=1 - [COPG2_HUMAN]	13.43	1	8	10	21	0.912	1.011	0.848	0.863	55.86	13.43	16	21	871	97.6	5.81
Q9H1J5	Protein Wnt-8a OS=Homo sapiens GN=WNT8A PE=2 SV=2 - [WNT8A_HUMAN]	8.26	1	1	2	4	1.078	1.262	0.907	0.863	5.85	8.26	2	4	351	38.8	7.77
Q10567	AP-1 complex subunit beta-1 OS=Homo sapiens GN=AP1B1 PE=1 SV=2 - [AP1B1_HUMAN]	42.26	1	18	39	155	1.133	0.773	0.920	0.863	365.29	42.26	61	155	949	104.6	5.06
Q99747	Gamma-soluble NSF attachment protein OS=Homo sapiens GN=NAPG PE=1 SV=1 - [SNAG_HUMAN]	43.59	1	13	13	50	1.144	0.771	0.922	0.863	117.39	43.59	21	50	312	34.7	5.41
P68366	Tubulin alpha-4A chain OS=Homo sapiens GN=TUBA4A PE=1 SV=1 - [TBA4A_HUMAN]	58.26	3	6	26	878	1.200	0.806	0.904	0.863	2412.49	58.26	45	878	448	49.9	5.06
Q7L5Y9	Macrophage erythroblast attacher OS=Homo sapiens GN=MAEA PE=1 SV=1 - [MAEA_HUMAN]	7.83	1	4	4	14	1.004	0.624	0.737	0.863	48.16	7.83	6	14	396	45.3	8.69
Q8IUD2	ELKS/Rab6-interacting/CAST family member 1 OS=Homo sapiens GN=ERC1 PE=1 SV=1 - [RB6I2_HUMAN]	39.70	1	31	42	167	0.965	1.198	1.020	0.863	340.44	39.70	67	167	1116	128.0	5.97
Q9BZE9	Tether containing UBX domain for GLUT4 OS=Homo sapiens GN=ASPSCR1 PE=1 SV=1 - [ASPC1_HUMAN]	17.18	1	8	8	27	0.973	0.923	1.034	0.863	60.50	17.18	13	27	553	60.1	6.64
P55011	Solute carrier family 12 member 2 OS=Homo sapiens GN=SLC12A2 PE=1 SV=1 - [S12A2_HUMAN]	8.42	1	7	8	25	0.742	1.108	0.842	0.863	56.62	8.42	13	25	1212	131.4	6.40

P78332	RNA-binding protein 6 OS=Homo sapiens GN=RBM6 PE=1 SV=5 - [RBM6_HUMAN]	1.51	1	1	2	5	1.205	1.370	1.308	0.863	10.23	1.51	3	5	1123	128.6	6.32
Q96DA6	Mitochondrial import inner membrane translocase subunit TIM14 OS=Homo sapiens GN=DNAJC19 PE=1 SV=3 - [TIM14_HUMAN]	48.28	1	7	7	11	1.011	0.971	0.918	0.863	25.62	48.28	10	11	116	12.5	10.10
O75822	Eukaryotic translation initiation factor 3 subunit J OS=Homo sapiens GN=EIF3J PE=1 SV=2 - [EIF3J_HUMAN]	31.40	1	12	12	41	0.842	0.984	0.912	0.863	94.28	31.40	17	41	258	29.0	4.83
Q14764	Major vault protein OS=Homo sapiens GN=MVP PE=1 SV=4 - [MVP_HUMAN]	57.33	1	51	51	560	0.938	1.133	1.248	0.864	1381.47	57.33	86	560	893	99.3	5.48
Q8WUW1	Protein BRICK1 OS=Homo sapiens GN=BRK1 PE=1 SV=1 - [BRK1_HUMAN]	54.67	1	4	5	13	0.963	0.849	0.961	0.864	24.17	54.67	8	13	75	8.7	5.45
Q9NZ08	Endoplasmic reticulum aminopeptidas e 1 OS=Homo sapiens GN=ERAP1 PE=1 SV=3 - [ERAP1_HUMAN]	16.79	1	17	17	68	1.282	0.872	0.708	0.864	189.86	16.79	27	68	941	107.2	6.46
O95273	Cyclin-D1-binding protein 1 OS=Homo sapiens GN=CCNDBP1 PE=1 SV=2 - [CCDB1_HUMAN]	8.33	1	3	3	17	0.932	0.709	0.979	0.864	42.51	8.33	4	17	360	40.2	4.82
Q9UNE7	E3 ubiquitin-protein ligase CHIP OS=Homo sapiens GN=STUB1 PE=1 SV=2 - [CHIP_HUMAN]	51.16	1	15	16	50	0.975	1.011	1.286	0.864	124.87	51.16	25	50	303	34.8	5.87
P14679	Tyrosinase OS=Homo sapiens GN=TYR PE=1 SV=3 - [TYRO_HUMAN]	3.21	1	2	2	2	0.703	0.841	0.818	0.864	2.69	3.21	2	2	529	60.4	6.11

Q5VZB9	Doublesex- and mab-3-related transcription factor A1 OS=Homo sapiens GN=DMRTA1 PE=2 SV=1 - [DMRTA_HUMAN]	5.75	1	1	1	1	0.903	0.997	0.898	0.864	2.16	5.75	1	1	504	53.1	8.90
Q8IYB8	ATP-dependent RNA helicase SUPV3L1, mitochondrial OS=Homo sapiens GN=SUPV3L1 PE=1 SV=1 - [SUV3_HUMAN]	7.25	1	5	5	8	0.778	1.204	1.086	0.864	17.62	7.25	7	8	786	87.9	7.99
O00442	RNA 3'-terminal phosphate cyclase OS=Homo sapiens GN=RTCA PE=1 SV=1 - [RTCA_HUMAN]	21.31	1	6	6	20	0.928	0.988	1.222	0.864	52.15	21.31	11	20	366	39.3	7.85
P51668	Ubiquitin-conjugating enzyme E2 D1 OS=Homo sapiens GN=UBE2D1 PE=1 SV=1 - [UB2D1_HUMAN]	12.24	2	2	2	3	0.975	0.753	0.807	0.864	4.66	12.24	3	3	147	16.6	7.42
P23743	Diacylglycerol kinase alpha OS=Homo sapiens GN=DGKA PE=1 SV=3 - [DGKA_HUMAN]	23.95	1	14	14	33	1.054	1.033	1.422	0.864	92.89	23.95	20	33	735	82.6	6.73
Q15842	ATP-sensitive inward rectifier potassium channel 8 OS=Homo sapiens GN=KCNJ8 PE=1 SV=1 - [KCNJ8_HUMAN]	2.12	1	1	1	1	1.041	0.650	0.515	0.864	2.31	2.12	1	1	424	47.9	9.26
P62699	Protein yippee-like 5 OS=Homo sapiens GN=YPEL5 PE=1 SV=1 - [YPEL5_HUMAN]	23.97	1	3	3	7	1.034	0.779	1.013	0.865	17.96	23.97	5	7	121	13.8	7.31
Q5TEU4	NADH dehydrogenase [ubiquinone] 1 alpha subcomplex assembly factor 5 OS=Homo sapiens GN=NDUFAF5 PE=1 SV=1 - [NDUF5_HUMAN]	2.61	1	1	1	3	0.883	1.143	0.812	0.865	7.22	2.61	2	3	345	38.9	6.57

O75445	Usherin OS=Homo sapiens GN=USH2A PE=1 SV=3 - [USH2A_HUMAN]	0.40	1	2	2	4	0.962	0.727	1.166	0.865	8.02	0.40	2	4	5202	575.2	6.83
Q8TF72	Protein Shroom3 OS=Homo sapiens GN=SHROOM3 PE=1 SV=2 - [SHRM3_HUMAN]	8.57	1	13	13	45	0.575	1.155	1.618	0.865	120.05	8.57	20	45	1996	216.7	7.80
Q13303	Voltage-gated potassium channel subunit beta-2 OS=Homo sapiens GN=KCNAB2 PE=1 SV=2 - [KCAB2_HUMAN]	4.36	1	1	2	7	0.752	0.353	0.449	0.865	9.05	4.36	3	7	367	41.0	9.00
P32856	Syntaxin-2 OS=Homo sapiens GN=STX2 PE=1 SV=3 - [STX2_HUMAN]	26.39	3	5	5	13	1.355	0.863	0.783	0.865	47.85	26.39	6	13	288	33.3	6.28
Q5RI15	Cytochrome c oxidase protein 20 homolog OS=Homo sapiens GN=COX20 PE=1 SV=2 - [COX20_HUMAN]	28.81	1	4	4	16	1.257	1.138	1.152	0.865	47.14	28.81	7	16	118	13.3	8.76
Q969G6	Riboflavin kinase OS=Homo sapiens GN=RFK PE=1 SV=2 - [RIFK_HUMAN]	12.90	1	3	3	8	0.714	0.938	0.819	0.865	18.64	12.90	5	8	155	17.6	8.13
Q15366	Poly(rC)-binding protein 2 OS=Homo sapiens GN=PCBP2 PE=1 SV=1 - [PCBP2_HUMAN]	38.90	1	5	11	136	0.641	0.745	0.803	0.865	358.66	38.90	19	136	365	38.6	6.79
Q8IXM7	Outer dense fiber protein 3-like protein 1 OS=Homo sapiens GN=ODF3L1 PE=2 SV=1 - [OD3L1_HUMAN]	4.38	1	1	1	1	1.276	0.937	0.993	0.865	2.09	4.38	1	1	274	31.0	9.54
P24666	Low molecular weight phosphotyrosine protein phosphatase OS=Homo sapiens GN=ACP1 PE=1 SV=3 - [PPAC_HUMAN]	47.47	1	7	7	37	0.934	0.890	1.023	0.865	110.54	47.47	10	37	158	18.0	6.74

O60493	Sorting nexin-3 OS=Homo sapiens GN=SNX3 PE=1 SV=3 - [SNX3_HUMAN]	46.91	1	9	10	85	0.915	0.913	1.045	0.865	291.57	46.91	18	85	162	18.8	8.66
Q9NUJ3	T-complex protein 11-like protein 1 OS=Homo sapiens GN=TCP11L1 PE=1 SV=1 - [T11L1_HUMAN]	9.23	1	4	4	11	0.951	1.387	1.334	0.866	25.40	9.23	7	11	509	57.0	5.59
Q9UBK9	Protein UXT OS=Homo sapiens GN=UXT PE=1 SV=1 - [UXT_HUMAN]	14.01	1	2	2	5	0.869	0.836	0.762	0.866	11.96	14.01	4	5	157	18.2	7.59
P20929	Nebulin OS=Homo sapiens GN=NEB PE=1 SV=5 - [NEBU_HUMAN]	2.11	1	8	11	15	1.219	0.758	0.931	0.866	26.05	2.11	12	15	6669	772.4	9.07
Q16864	V-type proton ATPase subunit F OS=Homo sapiens GN=ATP6V1F PE=1 SV=2 - [VATF_HUMAN]	77.31	1	7	7	21	0.912	0.788	0.823	0.866	53.55	77.31	10	21	119	13.4	5.52
P42566	Epidermal growth factor receptor substrate 15 OS=Homo sapiens GN=EPS15 PE=1 SV=2 - [EPS15_HUMAN]	21.76	1	16	16	55	0.831	0.825	0.797	0.866	118.07	21.76	20	55	896	98.6	4.64
P63010	AP-2 complex subunit beta OS=Homo sapiens GN=AP2B1 PE=1 SV=1 - [AP2B1_HUMAN]	46.00	1	24	46	232	0.974	0.853	0.899	0.866	533.87	46.00	72	232	937	104.5	5.38
Q9NUQ9	Protein FAM49B OS=Homo sapiens GN=FAM49B PE=1 SV=1 - [FA49B_HUMAN]	18.83	2	6	7	40	0.942	1.076	1.087	0.866	100.46	18.83	12	40	324	36.7	6.06
Q9NP95	Fibroblast growth factor 20 OS=Homo sapiens GN=FGF20 PE=1 SV=1 - [FGF20_HUMAN]	5.69	1	1	1	1	1.334	0.975	0.839	0.866	0.00	5.69	1	1	211	23.5	8.76
Q8N5M9	Protein jagunal homolog 1 OS=Homo sapiens GN=JAGN1 PE=1 SV=1 - [JAGN1_HUMAN]	7.10	1	1	1	2	0.909	0.809	0.557	0.866	6.26	7.10	1	2	183	21.1	9.73

P18077	60S ribosomal protein L35a OS=Homo sapiens GN=RPL35A PE=1 SV=2 - [RL35A_HUMAN]	58.18	1	10	10	46	0.678	0.738	0.584	0.866	104.93	58.18	16	46	110	12.5	11.06
Q17RC7	Exocyst complex component 3-like protein 4 OS=Homo sapiens GN=EXOC3L4 PE=2 SV=2 - [EX3L4_HUMAN]	5.40	1	2	4	12	0.697	0.652	1.060	0.866	23.21	5.40	5	12	722	79.8	6.32
P48556	26S proteasome non-ATPase regulatory subunit 8 OS=Homo sapiens GN=PSMD8 PE=1 SV=2 - [PSMD8_HUMA	44.29	1	15	15	52	0.794	0.784	0.870	0.866	102.61	44.29	23	52	350	39.6	9.70
Q9P035	Very-long-chain (3R)-3-hydroxyacyl-CoA dehydratase 3 OS=Homo sapiens GN=PTPLAD1 PE=1 SV=2 - [HACD3_HUMAN]	30.94	1	9	9	25	0.850	0.695	0.679	0.866	74.52	30.94	12	25	362	43.1	8.94
Q86YR5	G-protein-signaling modulator 1 OS=Homo sapiens GN=GPSM1 PE=1 SV=2 - [GPSM1_HUMA N]	18.07	2	12	12	26	0.808	0.937	1.176	0.866	73.76	18.07	17	26	675	74.5	6.54
P09913	Interferon-induced protein with tetratricopepti de repeats 2 OS=Homo sapiens GN=IFIT2 PE=1 SV=1 - [IFIT2_HUMAN]	14.62	1	6	7	18	7.902	1.273	1.647	0.867	45.63	14.62	10	18	472	54.6	6.76
Q8N8R7	ARL14 effector protein OS=Homo sapiens GN=ARL14EP PE=1 SV=1 - [AL14E_HUMAN]	9.62	1	2	2	4	0.758	1.065	1.241	0.867	5.39	9.62	3	4	260	29.3	8.07
Q15631	Translin OS=Homo sapiens GN=TSN PE=1 SV=1 - [TSN_HUMAN]	45.61	1	9	9	32	0.899	1.071	1.045	0.867	65.66	45.61	14	32	228	26.2	6.44

Q16706	Alpha-mannosidase 2 OS=Homo sapiens GN=MAN2A1 PE=1 SV=2 - [MA2A1_HUMAN]	10.58	1	11	11	32	0.823	0.642	0.735	0.867	69.77	10.58	17	32	1144	131.1	7.58
Q9Y487	V-type proton ATPase 116 kDa subunit a isoform 2 OS=Homo sapiens GN=ATP6V0A2 PE=1 SV=2 - [VPP2_HUMAN]	4.67	1	5	5	9	0.820	0.674	0.649	0.867	21.87	4.67	6	9	856	98.0	6.61
Q96K37	Solute carrier family 35 member E1 OS=Homo sapiens GN=SLC35E1 PE=1 SV=2 - [S35E1_HUMAN]	13.41	1	3	3	4	1.429	0.920	0.666	0.867	3.24	13.41	3	4	410	44.7	9.79
Q01968	Inositol polyphosphate 5-phosphatase OCRL-1 OS=Homo sapiens GN=OCRL PE=1 SV=3 - [OCRL_HUMAN]	9.77	1	7	8	25	0.947	0.984	1.298	0.867	62.62	9.77	12	25	901	104.1	6.55
O60299	Leucine zipper putative tumor suppressor 3 OS=Homo sapiens GN=LZTS3 PE=2 SV=1 - [LZTS3_HUMAN]	3.42	3	2	3	4	0.678	1.348	1.203	0.867	10.26	3.42	3	4	673	71.7	7.64
Q9ULZ3	Apoptosis-associated speck-like protein containing a CARD OS=Homo sapiens GN=PYCARD PE=1 SV=2 - [ASC_HUMAN]	37.95	2	7	7	25	1.108	1.643	0.678	0.867	64.04	37.95	12	25	195	21.6	6.34
O14974	Protein phosphatase 1 regulatory subunit 12A OS=Homo sapiens GN=PPP1R12A PE=1 SV=1 - [MYPT1_HUMAN]	36.89	1	41	42	212	0.857	1.230	1.283	0.867	534.09	36.89	66	212	1030	115.2	5.40
P22102	Trifunctional purine biosynthetic protein adenosine-3 OS=Homo sapiens GN=GART PE=1 SV=1 - [PUR2_HUMAN]	22.67	1	22	22	85	0.788	1.008	0.903	0.868	214.60	22.67	38	85	1010	107.7	6.70

P82921	28S ribosomal protein S21, mitochondrial OS=Homo sapiens GN=MRPS21 PE=1 SV=2 - [RT21_HUMAN]	24.14	1	2	2	2	0.646	1.053	0.981	0.868	6.15	24.14	2	2	87	10.7	10.21
O60645	Exocyst complex component 3 OS=Homo sapiens GN=EXOC3 PE=1 SV=2 - [EXOC3_HUMAN]	30.42	1	19	19	47	0.910	0.833	0.931	0.868	96.85	30.42	30	47	756	86.8	6.11
Q8IXW5	Putative RNA polymerase II subunit B1 CTD phosphatase RPAP2 OS=Homo sapiens GN=RPAP2 PE=1 SV=1 - [RPAP2_HUMAN]	8.33	1	4	4	7	1.070	1.065	0.836	0.868	18.38	8.33	4	7	612	69.5	7.78
Q13614	Myotubularin-related protein 2 OS=Homo sapiens GN=MTMR2 PE=1 SV=4 - [MTMR2_HUMAN]	3.73	2	2	4	13	0.592	1.164	1.051	0.868	24.03	3.73	6	13	643	73.3	7.40
O95639	Cleavage and polyadenylation specificity factor subunit 4 OS=Homo sapiens GN=CPSF4 PE=1 SV=1 - [CPSF4_HUMAN]	7.81	1	2	2	5	0.953	0.902	1.403	0.868	8.56	7.81	3	5	269	30.2	8.31
P50995	Annexin A11 OS=Homo sapiens GN=ANXA11 PE=1 SV=1 - [ANX11_HUMAN]	44.75	1	25	26	228	1.016	1.332	1.323	0.868	563.09	44.75	45	228	505	54.4	7.65
O60763	General vesicular transport factor p115 OS=Homo sapiens GN=USO1 PE=1 SV=2 - [USO1_HUMAN]	45.95	1	40	41	226	0.861	0.953	0.927	0.868	605.52	45.95	62	226	962	107.8	4.91
P63208	S-phase kinase-associated protein 1 OS=Homo sapiens GN=SKP1 PE=1 SV=2 - [SKP1_HUMAN]	65.03	1	11	11	70	1.114	0.956	0.878	0.868	180.49	65.03	20	70	163	18.6	4.54

Q14146	Unhealthy ribosome biogenesis protein 2 homolog OS=Homo sapiens GN=URB2 PE=2 SV=2 - [URB2_HUMAN]	2.17	1	2	2	2	0.851	1.270	1.003	0.868	2.97	2.17	2	2	1524	170.4	7.31
Q9P2L0	WD repeat-containing protein 35 OS=Homo sapiens GN=WDR35 PE=1 SV=3 - [WDR35_HUMAN]	2.71	1	2	2	11	1.119	0.755	0.892	0.869	3.44	2.71	2	11	1181	133.5	6.38
Q9BSJ8	Extended synaptotagmin-1 OS=Homo sapiens GN=ESYT1 PE=1 SV=1 - [ESYT1_HUMAN]	37.50	1	34	34	156	1.392	1.455	1.204	0.869	372.64	37.50	49	156	1104	122.8	5.83
Q9NPI6	mRNA-decapping enzyme 1A OS=Homo sapiens GN=DCP1A PE=1 SV=2 - [DCP1A_HUMAN]	7.39	1	3	3	8	1.168	1.046	0.976	0.869	15.97	7.39	6	8	582	63.3	6.25
P20340	Ras-related protein Rab-6A OS=Homo sapiens GN=RAB6A PE=1 SV=3 - [RAB6A_HUMAN]	38.94	3	8	11	74	0.963	1.003	1.021	0.869	173.26	38.94	16	74	208	23.6	5.54
Q9H4I3	TraB domain-containing protein OS=Homo sapiens GN=TRABD PE=1 SV=1 - [TRABD_HUMAN]	14.36	1	4	4	6	0.898	0.767	0.744	0.869	23.46	14.36	5	6	376	42.3	8.00
Q07973	1,25-dihydroxyvitamin D(3) 24-hydroxylase, mitochondrial OS=Homo sapiens GN=CYP24A1 PE=1 SV=2 - [CP24A_HUMAN]	2.72	1	1	1	2	1.366	0.805	0.421	0.869	4.42	2.72	1	2	514	58.8	8.75
Q7Z7E8	Ubiquitin-conjugating enzyme E2 Q1 OS=Homo sapiens GN=UBE2Q1 PE=1 SV=1 - [UB2Q1_HUMAN]	9.24	1	4	4	11	0.845	0.870	0.931	0.869	21.51	9.24	6	11	422	46.1	5.10

Q9UPP5	Uncharacterized protein KIAA1107 OS=Homo sapiens GN=KIAA1107 PE=1 SV=2 - [K1107_HUMAN]	1.77	1	3	3	3	0.783	0.553	1.182	0.869	6.97	1.77	3	3	1409	155.6	6.19
P28482	Mitogen-activated protein kinase 1 OS=Homo sapiens GN=MAPK1 PE=1 SV=3 - [MK01_HUMAN]	50.00	6	10	17	97	0.938	1.005	1.148	0.869	233.13	50.00	30	97	360	41.4	6.98
Q6YI46	Transmembrane protein 64 OS=Homo sapiens GN=TMEM64 PE=1 SV=2 - [TMM64_HUMAN]	9.74	1	1	1	1	1.082	1.185	1.549	0.869	2.21	9.74	1	1	380	39.6	8.41
Q8TB61	Adenosine 3'-phospho 5'-phosphosulfate transporter 1 OS=Homo sapiens GN=SLC35B2 PE=1 SV=1 - [S35B2_HUMAN]	6.94	1	4	4	10	1.084	0.906	0.969	0.869	19.69	6.94	7	10	432	47.5	9.16
Q5T1M5	FK506-binding protein 15 OS=Homo sapiens GN=FKBP15 PE=1 SV=2 - [FKB15_HUMAN]	15.50	1	18	18	64	0.929	1.096	0.848	0.869	172.48	15.50	28	64	1219	133.5	5.20
Q12768	WASH complex subunit strumpellin OS=Homo sapiens GN=KIAA0196 PE=1 SV=1 - [STRUM_HUMAN]	20.71	1	20	20	49	1.097	0.772	0.831	0.869	100.97	20.71	29	49	1159	134.2	6.98
P16083	Ribosyldihydronicotinamide dehydrogenase [quinone] OS=Homo sapiens GN=NQO2 PE=1 SV=5 - [NQO2_HUMAN]	40.69	1	8	8	28	0.866	0.569	0.467	0.869	94.86	40.69	13	28	231	25.9	6.29
Q9BPX7	UPF0415 protein C7orf25 OS=Homo sapiens GN=C7orf25 PE=1 SV=1 - [CG025_HUMAN]	5.23	2	3	3	9	0.727	0.580	0.639	0.869	18.15	5.23	3	9	421	46.4	6.42
Q9UIW2	Plexin-A1 OS=Homo sapiens GN=PLXNA1 PE=1 SV=3 - [PLXA1_HUMAN]	3.96	2	3	8	26	1.060	0.842	1.241	0.870	50.13	3.96	13	26	1896	210.9	6.92

Q7L523	Ras-related GTP-binding protein A OS=Homo sapiens GN=RRAGA PE=1 SV=1 - [RRAGA_HUMAN	12.46	2	3	4	14	0.866	0.907	1.098	0.870	31.95	12.46	7	14	313	36.5	7.72
Q6ZSR9	Uncharacterized protein FLJ45252 OS=Homo sapiens PE=1 SV=2 - [YJ005_HUMAN]	44.23	1	10	10	62	1.092	1.262	1.293	0.870	147.38	44.23	13	62	355	38.0	5.26
Q96A19	Coiled-coil domain-containing protein 102A OS=Homo sapiens GN=CCDC102A PE=1 SV=2 - [C102A_HUMAN]	7.45	1	3	5	11	1.059	1.635	1.264	0.870	22.25	7.45	7	11	550	62.6	5.58
Q9BQE5	Apolipoprotein L2 OS=Homo sapiens GN=APOL2 PE=1 SV=1 - [APOL2_HUMAN]	28.19	1	11	12	74	1.245	0.941	1.165	0.870	172.05	28.19	20	74	337	37.1	6.74
O75170	Serine/threonine-protein phosphatase 6 regulatory subunit 2 OS=Homo sapiens GN=PPP6R2 PE=1 SV=2 - [PP6R2_HUMAN]	12.22	1	9	9	25	0.967	0.929	1.038	0.871	57.81	12.22	16	25	966	104.9	4.87
O94901	SUN domain-containing protein 1 OS=Homo sapiens GN=SUN1 PE=1 SV=3 - [SUN1_HUMAN]	27.83	1	16	17	41	1.309	1.000	0.989	0.871	99.77	27.83	25	41	812	90.0	7.08
Q92817	Envoplakin OS=Homo sapiens GN=EVPL PE=1 SV=3 - [EVPL_HUMAN]	3.84	2	4	9	15	1.046	1.246	1.891	0.871	25.06	3.84	10	15	2033	231.5	6.96
Q9UBS8	E3 ubiquitin-protein ligase RNF14 OS=Homo sapiens GN=RNF14 PE=1 SV=1 - [RNF14_HUMAN]	6.33	1	3	3	10	0.980	0.749	0.836	0.871	24.26	6.33	4	10	474	53.8	4.75
Q96FZ7	Charged multivesicular body protein 6 OS=Homo sapiens GN=CHMP6 PE=1 SV=3 - [CHMP6_HUMA N]	18.91	1	4	4	42	0.983	0.958	1.158	0.871	124.57	18.91	7	42	201	23.5	5.31

Q9NPD8	Ubiquitin-conjugating enzyme E2 T OS=Homo sapiens GN=UBE2T PE=1 SV=1 - [UBE2T_HUMAN]	4.06	1	1	1	1	0.517	0.438	0.538	0.871	2.37	4.06	1	1	197	22.5	7.99
O60669	Monocarboxylate transporter 2 OS=Homo sapiens GN=SLC16A7 PE=1 SV=2 - [MOT2_HUMAN]	3.77	1	2	2	9	1.097	1.207	1.004	0.871	21.59	3.77	3	9	478	52.2	9.31
Q6ZS17	Protein FAM65A OS=Homo sapiens GN=FAM65A PE=1 SV=1 - [FA65A_HUMAN]	9.08	1	10	10	20	0.914	0.903	0.960	0.871	45.20	9.08	16	20	1223	132.2	6.28
P78537	Biogenesis of lysosome-related organelles complex 1 subunit 1 OS=Homo sapiens GN=BLOC1S1 PE=1 SV=2 - [BL1S1_HUMAN]	38.56	1	5	6	13	0.878	1.035	1.054	0.871	30.34	38.56	8	13	153	17.3	9.33
Q9HCD6	Protein TANC2 OS=Homo sapiens GN=TANC2 PE=1 SV=3 - [TANC2_HUMAN]	0.95	1	2	2	2	0.873	1.198	1.039	0.871	0.00	0.95	2	2	1990	219.5	8.07
Q9NY33	Dipeptidyl peptidase 3 OS=Homo sapiens GN=DPP3 PE=1 SV=2 - [DPP3_HUMAN]	39.76	1	26	26	101	1.015	0.869	0.885	0.871	246.32	39.76	40	101	737	82.5	5.10
Q86UB9	Transmembrane protein 135 OS=Homo sapiens GN=TMEM135 PE=2 SV=2 - [TM135_HUMAN]	3.49	1	2	2	5	1.154	0.726	0.918	0.871	5.77	3.49	4	5	458	52.3	9.45
P46734	Dual specificity mitogen-activated protein kinase kinase 3 OS=Homo sapiens GN=MAP2K3 PE=1 SV=2 - [MP2K3_HUMA	39.19	2	13	13	32	0.540	0.786	0.953	0.872	73.13	39.19	21	32	347	39.3	7.43
P25789	Proteasome subunit alpha type-4 OS=Homo sapiens GN=PSMA4 PE=1 SV=1 - [PSA4_HUMAN]	55.17	2	14	14	73	0.915	0.846	0.795	0.872	182.08	55.17	18	73	261	29.5	7.72

Q99598	Translin-associated protein X OS=Homo sapiens GN=TSNAX PE=1 SV=1 - [TSNAX_HUMAN]	30.34	1	7	7	18	0.797	1.172	0.952	0.872	49.91	30.34	10	18	290	33.1	6.55
Q16850	Lanosterol 14-alpha demethylase OS=Homo sapiens GN=CYP51A1 PE=1 SV=3 - [CP51A_HUMAN]	11.13	1	6	6	11	0.678	0.574	0.886	0.872	28.74	11.13	9	11	503	56.8	8.53
Q4G0P3	Hydrocephalus-inducing protein homolog OS=Homo sapiens GN=HYDIN PE=1 SV=3 - [HYDIN_HUMAN]	0.70	1	4	4	4	1.014	0.708	0.531	0.872	5.30	0.70	4	4	5121	575.5	6.06
Q8IUF1	COBW domain-containing protein 2 OS=Homo sapiens GN=CBWD2 PE=1 SV=1 - [CBWD2_HUMAN]	8.35	6	3	3	7	0.977	1.094	1.161	0.873	16.99	8.35	4	7	395	44.0	4.89
P84022	Mothers against decapentaplegic homolog 3 OS=Homo sapiens GN=SMAD3 PE=1 SV=1 - [SMAD3_HUMA	12.47	2	5	5	18	1.060	0.972	1.153	0.873	49.92	12.47	10	18	425	48.0	7.15
O43255	E3 ubiquitin-protein ligase SIAH2 OS=Homo sapiens GN=SIAH2 PE=1 SV=1 - [SIAH2_HUMAN]	2.47	1	1	1	1	0.725	0.835	0.993	0.873	0.00	2.47	1	1	324	34.6	7.12
P21397	Amine oxidase [flavin-containing] A OS=Homo sapiens GN=MAOA PE=1 SV=1 - [AOFA_HUMAN]	42.13	1	16	19	69	0.682	0.981	0.521	0.873	179.82	42.13	32	69	527	59.6	7.85
Q5T6V5	UPF0553 protein C9orf64 OS=Homo sapiens GN=C9orf64 PE=1 SV=1 - [CI064_HUMAN]	5.57	1	2	2	9	0.935	0.690	0.724	0.873	18.73	5.57	4	9	341	39.0	5.88
Q8WUP2	Filamin-binding LIM protein 1 OS=Homo sapiens GN=FBLIM1 PE=1 SV=2 - [FBLI1_HUMAN]	12.60	1	6	6	28	1.053	0.925	1.560	0.873	76.24	12.60	11	28	373	40.6	6.02

Q7L592	NADH dehydrogenase [ubiquinone] complex I, assembly factor 7 OS=Homo sapiens GN=NDUFAF7 PE=1 SV=1 - [NDUF7_HUMAN]	12.47	1	4	4	8	0.940	1.130	0.871	0.873	11.08	12.47	5	8	441	49.2	8.34
P51587	Breast cancer type 2 susceptibility protein OS=Homo sapiens GN=BRCA2 PE=1 SV=2 - [BRCA2_HUMAN]	0.47	1	2	2	2	1.666	1.396	1.376	0.873	5.44	0.47	2	2	3418	384.0	6.73
A4D0S4	Laminin subunit beta-4 OS=Homo sapiens GN=LAMB4 PE=2 SV=1 - [LAMB4_HUMAN]	0.91	1	1	1	1	2.809	1.022	0.611	0.873	2.75	0.91	1	1	1761	193.4	6.35
Q9Y287	Integral membrane protein 2B OS=Homo sapiens GN=ITM2B PE=1 SV=1 - [ITM2B_HUMAN]	3.76	1	1	1	3	0.938	0.387	0.847	0.874	7.81	3.76	1	3	266	30.3	5.14
Q9NUQ7	Ufm1-specific protease 2 OS=Homo sapiens GN=UFSP2 PE=1 SV=3 - [UFSP2_HUMAN]	19.62	1	5	5	15	1.148	1.073	0.866	0.874	29.28	19.62	8	15	469	53.2	7.01
Q9NQH7	Probable Xaa-Pro aminopeptidase 3 OS=Homo sapiens GN=XPNPEP3 PE=1 SV=1 - [XPP3_HUMAN]	4.73	1	2	2	3	1.352	0.934	1.212	0.874	5.92	4.73	3	3	507	57.0	6.83
Q15011	Homocysteine-responsive endoplasmic reticulum-resident ubiquitin-like domain member 1 protein OS=Homo sapiens GN=HERPUD1 PE=1 SV=1 - [HERP1_HUMAN	3.84	1	1	2	6	0.545	0.595	0.710	0.874	6.65	3.84	3	6	391	43.7	5.25
Q9UBX5	Fibulin-5 OS=Homo sapiens GN=FBLN5 PE=1 SV=1 - [FBLN5_HUMAN]	4.69	1	2	2	5	0.675	0.381	0.697	0.874	10.85	4.69	3	5	448	50.1	4.73

Q14689	Disco-interacting protein 2 homolog A OS=Homo sapiens GN=DIP2A PE=1 SV=2 - [DIP2A_HUMAN]	3.44	1	3	5	7	0.740	0.979	0.938	0.874	17.73	3.44	7	7	1571	170.3	8.03
Q15811	Intersectin-1 OS=Homo sapiens GN=ITSN1 PE=1 SV=3 - [ITSN1_HUMAN]	12.38	1	15	19	40	1.082	0.977	1.033	0.874	92.32	12.38	27	40	1721	195.3	7.77
O15145	Actin-related protein 2/3 complex subunit 3 OS=Homo sapiens GN=ARPC3 PE=1 SV=3 - [ARPC3_HUMAN]	24.72	1	5	5	12	0.970	0.917	0.958	0.874	30.71	24.72	8	12	178	20.5	8.59
P23588	Eukaryotic translation initiation factor 4B OS=Homo sapiens GN=EIF4B PE=1 SV=2 - [IF4B_HUMAN]	34.37	1	20	20	78	0.860	0.897	0.807	0.874	138.62	34.37	31	78	611	69.1	5.73
P59095	StAR-related lipid transfer protein 6 OS=Homo sapiens GN=STARD6 PE=1 SV=1 - [STAR6_HUMAN]	3.64	1	1	1	2	1.945	1.872	1.250	0.874	1.75	3.64	1	2	220	25.0	9.32
Q13526	Peptidyl-prolyl cis-trans isomerase NIMA-interacting 1 OS=Homo sapiens GN=PIN1 PE=1 SV=1 - [PIN1_HUMAN]	48.47	1	6	6	27	1.046	1.059	1.205	0.874	52.05	48.47	8	27	163	18.2	8.82
Q3ZAQ7	Vacuolar ATPase assembly integral membrane protein VMA21 OS=Homo sapiens GN=VMA21 PE=1 SV=1 - [VMA21_HUMAN]	11.88	1	1	1	7	0.870	0.760	0.851	0.875	19.22	11.88	2	7	101	11.3	7.24
P60508	Syncytin-2 OS=Homo sapiens GN=ERVFRD-1 PE=1 SV=1 - [SYCY2_HUMAN]	1.86	1	1	1	1	0.597	1.186	1.232	0.875	2.19	1.86	1	1	538	59.5	8.85

Q8WVP5	Tumor necrosis factor alpha-induced protein 8-like protein 1 OS=Homo sapiens GN=TNFAIP8L1 PE=2 SV=2 - [TP8L1_HUMAN	4.30	2	1	1	2	0.445	1.589	0.820	0.875	2.78	4.30	1	2	186	20.8	9.50
Q15005	Signal peptidase complex subunit 2 OS=Homo sapiens GN=SPCS2 PE=1 SV=3 - [SPCS2_HUMAN	48.67	1	15	15	52	0.863	0.757	0.769	0.875	147.08	48.67	18	52	226	25.0	8.47
Q9HBD1	Roquin-2 OS=Homo sapiens GN=RC3H2 PE=1 SV=2 - [RC3H2_HUMAN]	4.95	1	3	3	4	0.729	0.564	1.268	0.875	7.21	4.95	4	4	1191	131.6	6.89
Q5T1B0	Axonemal dynein light chain domain-containing protein 1 OS=Homo sapiens GN=AXDND1 PE=2 SV=1 - [AXDN1_HUMAN]	1.09	1	1	1	1	1.695	0.817	0.956	0.875	1.92	1.09	1	1	1012	118.0	5.68
P62258	14-3-3 protein epsilon OS=Homo sapiens GN=YWHAE PE=1 SV=1 - [1433E_HUMAN]	69.41	1	21	24	320	1.200	1.074	0.986	0.875	1013.79	69.41	38	320	255	29.2	4.74
Q5SY80	Uncharacterized protein C1orf101 OS=Homo sapiens GN=C1orf101 PE=2 SV=1 - [CA101_HUMAN]	1.89	1	1	1	2	1.516	1.360	1.311	0.875	2.28	1.89	1	2	951	109.6	7.27
Q96GQ5	RUS1 family protein C16orf58 OS=Homo sapiens GN=C16orf58 PE=1 SV=2 - [RUS1_HUMAN]	27.14	1	11	11	41	1.008	1.007	0.844	0.875	94.72	27.14	18	41	468	51.0	6.93
O43194	G-protein coupled receptor 39 OS=Homo sapiens GN=GPR39 PE=1 SV=1 - [GPR39_HUMAN]	1.55	1	1	1	3	0.586	0.731	0.927	0.875	7.30	1.55	1	3	453	51.3	9.29
Q05397	Focal adhesion kinase 1 OS=Homo sapiens GN=PTK2 PE=1 SV=2 - [FAK1_HUMAN]	15.78	1	12	14	39	0.943	1.207	0.964	0.875	88.93	15.78	21	39	1052	119.2	6.62

Q7KZF4	Staphylococcal nuclease domain-containing protein 1 OS=Homo sapiens GN=SND1 PE=1 SV=1 - [SND1_HUMAN]	54.95	1	51	52	344	0.796	0.821	0.949	0.875	897.03	54.95	84	344	910	101.9	7.17
P57721	Poly(rC)-binding protein 3 OS=Homo sapiens GN=PCBP3 PE=2 SV=2 - [PCBP3_HUMAN]	17.52	1	1	6	121	0.618	0.526	0.434	0.875	326.04	17.52	12	121	371	39.4	8.07
Q14999	Cullin-7 OS=Homo sapiens GN=CUL7 PE=1 SV=2 - [CUL7_HUMAN]	5.59	1	6	7	15	1.008	0.974	0.914	0.875	39.14	5.59	11	15	1698	191.0	5.87
P29474	Nitric oxide synthase, endothelial OS=Homo sapiens GN=NOS3 PE=1 SV=3 - [NOS3_HUMAN]	1.16	1	2	2	5				0.875	8.13	1.16	3	5	1203	133.2	7.27
Q96JM4	Leucine-rich repeat and IQ domain-containing protein 1 OS=Homo sapiens GN=LRRIQ1 PE=2 SV=3 - [LRIQ1_HUMAN]	1.34	1	2	3	7	1.169	0.845		0.876	17.95	1.34	3	7	1722	199.2	6.19
P06703	Protein S100-A6 OS=Homo sapiens GN=S100A6 PE=1 SV=1 - [S10A6_HUMAN]	80.00	1	12	12	581	1.495	0.994	0.842	0.876	1208.76	80.00	19	581	90	10.2	5.48
Q13227	G protein pathway suppressor 2 OS=Homo sapiens GN=GPS2 PE=1 SV=3 - [GPS2_HUMAN]	2.45	1	1	1	3	1.511	1.815	1.126	0.876	2.07	2.45	1	3	327	36.7	9.52
Q14254	Flotillin-2 OS=Homo sapiens GN=FLOT2 PE=1 SV=2 - [FLOT2_HUMAN]	40.42	1	18	18	61	1.076	1.249	1.135	0.876	162.86	40.42	29	61	428	47.0	5.25
P39656	Dolichyl-diphosphooligosaccharide--protein glycosyltransferase 48 kDa subunit OS=Homo sapiens GN=DDOST PE=1 SV=4 - [OST48_HUMAN]	37.50	1	16	16	42	0.748	0.800	0.661	0.876	101.22	37.50	24	42	456	50.8	6.55

A6NH57	Putative ADP-ribosylation factor-like protein 5C OS=Homo sapiens GN=ARL5C PE=2 SV=4 - [ARL5C_HUMAN]	10.06	1	1	1	1	1.535	1.954	1.524	0.876	0.00	10.06	1	1	179	20.6	5.72
P61081	NEDD8-conjugating enzyme Ubc12 OS=Homo sapiens GN=UBE2M PE=1 SV=1 - [UBC12_HUMAN]	36.07	1	8	8	38	0.934	0.999	0.879	0.876	81.20	36.07	15	38	183	20.9	7.69
P57103	Sodium/calcium exchanger 3 OS=Homo sapiens GN=SLC8A3 PE=2 SV=2 - [NAC3_HUMAN]	2.80	1	2	2	2	0.725	1.019	2.204	0.876	2.86	2.80	2	2	927	102.9	5.12
Q93015	N-acetyltransferase 6 OS=Homo sapiens GN=NAT6 PE=2 SV=2 - [NAT6_HUMAN]	11.89	1	3	3	5	1.865	1.580	1.497	0.876	7.67	11.89	5	5	286	31.4	7.85
Q6P3X3	Tetratricopeptide repeat protein 27 OS=Homo sapiens GN=TTC27 PE=1 SV=1 - [TTC27_HUMAN	8.90	1	6	6	12	0.786	0.930	1.003	0.876	20.74	8.90	9	12	843	96.6	5.59
Q8TDW5	Synaptotagmin-like protein 5 OS=Homo sapiens GN=SYTL5 PE=1 SV=1 - [SYTL5_HUMAN]	2.05	1	1	2	3	0.923	1.113	1.379	0.877	2.00	2.05	2	3	730	81.5	8.78
O43427	Acidic fibroblast growth factor intracellular-binding protein OS=Homo sapiens GN=FIBP PE=1 SV=3 - [FIBP_HUMAN]	5.77	1	2	2	5	1.103	0.951	0.880	0.877	7.14	5.77	3	5	364	41.9	6.48
Q4G0N4	NAD kinase 2, mitochondrial OS=Homo sapiens GN=NADK2 PE=1 SV=2 - [NAKD2_HUMAN]	15.16	1	6	6	15	0.753	0.848	0.721	0.877	29.70	15.16	9	15	442	49.4	8.18
Q13748	Tubulin alpha-3C/D chain OS=Homo sapiens GN=TUBA3C PE=1 SV=3 - [TBA3C_HUMAN]	52.22	5	1	24	1068	0.829	0.743	0.687	0.877	3120.65	52.22	42	1068	450	49.9	5.10

P12081	Histidine--tRNA ligase, cytoplasmic OS=Homo sapiens GN=HARS PE=1 SV=2 - [SYHC_HUMAN]	48.33	2	17	24	101	0.916	1.092	1.154	0.877	253.57	48.33	40	101	509	57.4	5.88
Q8NFW8	N-acylneuraminate cytidylyltransferase OS=Homo sapiens GN=CMAS PE=1 SV=2 - [NEUA_HUMAN]	21.89	1	6	6	17	0.794	1.337	1.100	0.877	54.53	21.89	11	17	434	48.3	7.93
O14744	Protein arginine N-methyltransferase 5 OS=Homo sapiens GN=PRMT5 PE=1 SV=4 - [ANM5_HUMAN]	16.01	1	10	10	31	0.904	0.850	0.826	0.877	74.30	16.01	15	31	637	72.6	6.29
P42229	Signal transducer and activator of transcription 5A OS=Homo sapiens GN=STAT5A PE=1 SV=1 - [STA5A_HUMAN]	14.48	2	2	11	29	1.240	1.275	1.309	0.877	56.15	14.48	18	29	794	90.6	6.39
Q9NYP8	Uncharacterized protein C21orf62 OS=Homo sapiens GN=C21orf62 PE=1 SV=2 - [CU062_HUMAN]	2.74	1	1	1	1	4.709	0.240	0.310	0.877	1.73	2.74	1	1	219	24.9	8.02
B0I1T2	Unconventional myosin-Ig OS=Homo sapiens GN=MYO1G PE=1 SV=2 - [MYO1G_HUMAN]	2.46	1	1	2	6	2.161	1.078	1.358	0.877	12.06	2.46	3	6	1018	116.4	8.73
P32456	Interferon-induced guanylate-binding protein 2 OS=Homo sapiens GN=GBP2 PE=1 SV=3 - [GBP2_HUMAN]	32.66	2	21	26	129	1.243	1.145	1.171	0.877	293.21	32.66	40	129	591	67.2	5.71
Q6ZUT3	FERM domain-containing protein 7 OS=Homo sapiens GN=FRMD7 PE=1 SV=1 - [FRMD7_HUMAN]	0.84	1	1	1	1	0.813	0.927	1.081	0.877	2.23	0.84	1	1	714	81.6	7.90

P62330	ADP-ribosylation factor 6 OS=Homo sapiens GN=ARF6 PE=1 SV=2 - [ARF6_HUMAN]	43.43	3	6	7	20	1.244	0.593	0.614	0.877	39.25	43.43	12	20	175	20.1	8.95
Q86VS8	Protein Hook homolog 3 OS=Homo sapiens GN=HOOK3 PE=1 SV=2 - [HOOK3_HUMAN]	31.48	1	22	23	63	0.795	1.061	0.937	0.877	155.73	31.48	34	63	718	83.1	5.17
Q86X02	Cerebellar degeneration-related protein 2-like OS=Homo sapiens GN=CDR2L PE=2 SV=2 - [CDR2L_HUMAN	6.67	1	2	3	5	0.984	0.679	1.154	0.877	10.22	6.67	5	5	465	53.0	6.01
P30626	Sorcin OS=Homo sapiens GN=SRI PE=1 SV=1 - [SORCN_HUMAN]	45.96	1	8	8	33	0.834	0.977	1.271	0.877	67.80	45.96	13	33	198	21.7	5.59
O75143	Autophagy-related protein 13 OS=Homo sapiens GN=ATG13 PE=1 SV=1 - [ATG13_HUMAN]	6.58	1	2	3	6	0.758	0.920	0.930	0.877	16.02	6.58	5	6	517	56.5	5.12
O43242	26S proteasome non-ATPase regulatory subunit 3 OS=Homo sapiens GN=PSMD3 PE=1 SV=2 - [PSMD3_HUMA	55.81	1	30	30	171	0.851	0.880	0.842	0.877	394.00	55.81	51	171	534	60.9	8.44
Q15021	Condensin complex subunit 1 OS=Homo sapiens GN=NCAPD2 PE=1 SV=3 - [CND1_HUMAN]	2.78	1	3	3	8	0.866	0.993	0.844	0.877	16.78	2.78	4	8	1401	157.1	6.61
Q9P2M7	Cingulin OS=Homo sapiens GN=CGN PE=1 SV=2 - [CING_HUMAN]	1.17	2	1	4	9	1.089	0.804	0.763	0.877	14.70	1.17	4	9	1197	136.3	5.54
P31150	Rab GDP dissociation inhibitor alpha OS=Homo sapiens GN=GDI1 PE=1 SV=2 - [GDIA_HUMAN]	49.89	1	14	21	157	1.122	0.928	1.171	0.878	374.59	49.89	33	157	447	50.6	5.14

O94929	Actin-binding LIM protein 3 OS=Homo sapiens GN=ABLIM3 PE=1 SV=3 - [ABLM3_HUMAN]	12.01	1	7	8	14	1.045	0.928	0.796	0.878	29.12	12.01	10	14	683	77.8	8.54
Q13765	Nascent polypeptide-associated complex subunit alpha OS=Homo sapiens GN=NACA PE=1 SV=1 - [NACA_HUMAN]	33.95	3	6	6	37	0.729	1.050	0.849	0.878	103.38	33.95	9	37	215	23.4	4.56
O75131	Copine-3 OS=Homo sapiens GN=CPNE3 PE=1 SV=1 - [CPNE3_HUMAN]	35.20	4	17	18	68	0.878	1.089	0.980	0.878	166.66	35.20	30	68	537	60.1	5.85
P04083	Annexin A1 OS=Homo sapiens GN=ANXA1 PE=1 SV=2 - [ANXA1_HUMAN]	69.94	1	29	29	836	1.099	0.674	1.196	0.878	2043.76	69.94	53	836	346	38.7	7.02
Q2TB90	Putative hexokinase HKDC1 OS=Homo sapiens GN=HKDC1 PE=1 SV=3 - [HKDC1_HUMAN]	4.03	1	2	4	11	1.444	0.874	1.031	0.878	13.76	4.03	5	11	917	102.5	7.12
P61158	Actin-related protein 3 OS=Homo sapiens GN=ACTR3 PE=1 SV=3 - [ARP3_HUMAN]	48.56	3	19	19	288	0.966	1.068	1.199	0.878	862.27	48.56	36	288	418	47.3	5.88
Q96AV8	Transcription factor E2F7 OS=Homo sapiens GN=E2F7 PE=1 SV=3 - [E2F7_HUMAN]	2.74	2	2	2	2	1.257	0.128	0.426	0.878	2.96	2.74	2	2	911	99.8	8.16
A6NC98	Coiled-coil domain-containing protein 88B OS=Homo sapiens GN=CCDC88B PE=1 SV=1 - [CC88B_HUMAN]	2.51	1	2	5	5	0.786	0.839	1.185	0.878	9.79	2.51	5	5	1476	164.7	5.11
Q9BYV8	Centrosomal protein of 41 kDa OS=Homo sapiens GN=CEP41 PE=1 SV=1 - [CEP41_HUMAN]	9.65	1	3	3	7	0.862	1.096	1.116	0.878	17.97	9.65	3	7	373	41.3	8.32

Q9BV81	ER membrane protein complex subunit 6 OS=Homo sapiens GN=EMC6 PE=1 SV=1 - [EMC6_HUMAN]	20.00	1	2	2	9	1.036	0.842	0.873	0.879	14.03	20.00	4	9	110	12.0	10.07
P55809	Succinyl-CoA:3-ketoacid coenzyme A transferase 1, mitochondrial OS=Homo sapiens GN=OXCT1 PE=1 SV=1 - [SCOT1_HUMAN]	32.12	1	15	15	41	0.876	1.619	0.931	0.879	121.53	32.12	24	41	520	56.1	7.46
Q99622	Protein C10 OS=Homo sapiens GN=C12orf57 PE=1 SV=1 - [C10_HUMAN]	22.22	1	2	2	7	0.698	1.193	0.986	0.879	28.17	22.22	3	7	126	13.2	5.14
Q9Y608	Leucine-rich repeat flightless-interacting protein 2 OS=Homo sapiens GN=LRRFIP2 PE=1 SV=1 - [LRRF2_HUMAN	20.11	1	14	18	68	0.854	1.086	1.076	0.879	174.42	20.11	24	68	721	82.1	6.95
Q9H4A6	Golgi phosphoprotein 3 OS=Homo sapiens GN=GOLPH3 PE=1 SV=1 - [GOLP3_HUMAN]	14.43	1	3	4	5	0.639	0.795	0.811	0.879	11.58	14.43	5	5	298	33.8	6.44
Q8NH69	Olfactory receptor 5W2 OS=Homo sapiens GN=OR5W2 PE=2 SV=1 - [OR5W2_HUMAN]	2.58	2	1	1	1	1.501	1.025	1.330	0.879	0.00	2.58	1	1	310	35.1	7.01
Q04637	Eukaryotic translation initiation factor 4 gamma 1 OS=Homo sapiens GN=EIF4G1 PE=1 SV=4 - [IF4G1_HUMAN]	34.90	1	41	49	286	0.837	0.860	0.901	0.879	604.74	34.90	79	286	1599	175.4	5.33
Q9UL54	Serine/threonine-protein kinase TAO2 OS=Homo sapiens GN=TAOK2 PE=1 SV=2 - [TAOK2_HUMAN]	3.89	1	2	5	16	1.509	1.261	1.122	0.879	37.95	3.89	9	16	1235	138.2	7.27
Q9H4M9	EH domain-containing protein 1 OS=Homo sapiens GN=EHD1 PE=1 SV=2 - [EHD1_HUMAN]	61.80	2	29	39	237	1.026	1.199	1.415	0.879	573.21	61.80	57	237	534	60.6	6.83

P07202	Thyroid peroxidase OS=Homo sapiens GN=TPO PE=1 SV=4 - [PERT_HUMAN]	5.14	1	2	2	13	2.018	0.279	0.693	0.879	28.78	5.14	2	13	933	102.9	6.76
Q02818	Nucleobindin-1 OS=Homo sapiens GN=NUCB1 PE=1 SV=4 - [NUCB1_HUMAN]	65.73	1	26	27	134	0.837	0.932	1.079	0.879	363.82	65.73	43	134	461	53.8	5.25
O15305	Phosphomannomutase 2 OS=Homo sapiens GN=PMM2 PE=1 SV=1 - [PMM2_HUMAN]	46.75	1	12	13	36	1.003	0.941	1.058	0.879	82.28	46.75	19	36	246	28.1	6.77
Q13630	GDP-L-fucose synthase OS=Homo sapiens GN=TSTA3 PE=1 SV=1 - [FCL_HUMAN]	34.27	1	8	8	20	0.869	1.072	1.109	0.879	57.79	34.27	13	20	321	35.9	6.60
Q99584	Protein S100-A13 OS=Homo sapiens GN=S100A13 PE=1 SV=1 - [S10AD_HUMAN]	55.10	1	8	8	114	0.736	1.120	0.990	0.880	248.05	55.10	15	114	98	11.5	6.16
P15622	Zinc finger protein 250 OS=Homo sapiens GN=ZNF250 PE=2 SV=3 - [ZN250_HUMAN]	2.32	1	1	1	1	0.323	1.101	0.934	0.880	2.66	2.32	1	1	560	63.4	8.28
Q14232	Translation initiation factor eIF-2B subunit alpha OS=Homo sapiens GN=EIF2B1 PE=1 SV=1 - [EI2BA_HUMAN]	50.82	1	14	14	45	1.020	0.926	0.957	0.880	94.42	50.82	22	45	305	33.7	7.33
Q96JB2	Conserved oligomeric Golgi complex subunit 3 OS=Homo sapiens GN=COG3 PE=1 SV=3 - [COG3_HUMAN]	11.71	1	9	9	28	0.844	0.890	1.003	0.880	67.04	11.71	14	28	828	94.0	5.57
Q9UJY1	Heat shock protein beta-8 OS=Homo sapiens GN=HSPB8 PE=1 SV=1 - [HSPB8_HUMAN]	32.14	1	6	6	12	0.772	0.929	1.208	0.880	19.67	32.14	9	12	196	21.6	5.12

Q9H1H9	Kinesin-like protein KIF13A OS=Homo sapiens GN=KIF13A PE=1 SV=2 - [KI13A_HUMAN]	9.75	1	12	16	38	0.893	0.940	1.441	0.880	84.18	9.75	22	38	1805	202.2	5.60
O75526	RNA-binding motif protein, X-linked-like-2 OS=Homo sapiens GN=RBMXL2 PE=1 SV=3 - [RMXL2_HUMAN]	7.65	2	1	3	12	1.176	0.791	0.837	0.880	32.56	7.65	5	12	392	42.8	10.32
Q8N0Z6	Tetratricopeptide repeat protein 5 OS=Homo sapiens GN=TTC5 PE=1 SV=2 - [TTC5_HUMAN]	5.91	1	2	2	8	0.832	0.751	1.296	0.880	17.62	5.91	4	8	440	48.9	6.48
Q6NSW5	Protein FAM45B OS=Homo sapiens GN=FAM45B PE=2 SV=1 - [FA45B_HUMAN]	10.08	2	3	3	7	1.162	0.981	0.962	0.880	13.80	10.08	5	7	357	40.5	6.54
Q12959	Disks large homolog 1 OS=Homo sapiens GN=DLG1 PE=1 SV=2 - [DLG1_HUMAN]	30.53	2	21	23	78	1.002	0.963	1.153	0.880	172.69	30.53	37	78	904	100.4	5.76
Q5T8D3	Acyl-CoA-binding domain-containing protein 5 OS=Homo sapiens GN=ACBD5 PE=1 SV=1 - [ACBD5_HUMAN]	15.36	1	6	6	11	1.049	1.103	0.916	0.880	28.61	15.36	8	11	534	60.1	5.33
Q96CT7	Coiled-coil domain-containing protein 124 OS=Homo sapiens GN=CCDC124 PE=1 SV=1 - [CC124_HUMAN]	55.61	1	13	13	62	0.795	1.055	1.037	0.880	169.35	55.61	23	62	223	25.8	9.54
O60925	Prefoldin subunit 1 OS=Homo sapiens GN=PFDN1 PE=1 SV=2 - [PFD1_HUMAN]	50.00	1	8	8	35	0.921	1.149	1.011	0.880	79.15	50.00	12	35	122	14.2	6.81
Q9UKZ1	CCR4-NOT transcription complex subunit 11 OS=Homo sapiens GN=CNOT11 PE=1 SV=1 - [CNO11_HUMAN]	5.49	1	2	2	4	0.852	0.982	0.843	0.880	8.38	5.49	3	4	510	55.2	6.40

Q15042	Rab3 GTPase-activating protein catalytic subunit OS=Homo sapiens GN=RAB3GAP1 PE=1 SV=3 - [RB3GP_HUMAN	25.28	1	23	23	68	0.947	1.052	1.034	0.881	143.92	25.28	38	68	981	110.5	5.55
Q8TD43	Transient receptor potential cation channel subfamily M member 4 OS=Homo sapiens GN=TRPM4 PE=1 SV=1 - [TRPM4_HUMAN]	6.18	1	7	7	18	1.090	0.636	0.671	0.881	36.41	6.18	11	18	1214	134.2	8.15
Q92747	Actin-related protein 2/3 complex subunit 1A OS=Homo sapiens GN=ARPC1A PE=1 SV=2 - [ARC1A_HUMAN]	21.35	1	8	8	23	0.793	0.690	0.777	0.881	44.66	21.35	12	23	370	41.5	8.18
Q16204	Coiled-coil domain-containing protein 6 OS=Homo sapiens GN=CCDC6 PE=1 SV=2 - [CCDC6_HUMAN]	31.65	1	14	15	64	0.783	1.061	1.281	0.881	143.07	31.65	25	64	474	53.3	7.34
P07737	Profilin-1 OS=Homo sapiens GN=PFN1 PE=1 SV=2 - [PROF1_HUMAN]	75.00	1	13	13	852	1.164	1.047	1.165	0.881	2583.77	75.00	26	852	140	15.0	8.27
Q96CT2	Kelch-like protein 29 OS=Homo sapiens GN=KLHL29 PE=2 SV=3 - [KLH29_HUMAN]	2.40	1	2	2	2	1.332	0.920	1.232	0.881	4.40	2.40	2	2	875	94.2	7.17
Q86VX2	COMM domain-containing protein 7 OS=Homo sapiens GN=COMMD7 PE=1 SV=2 - [COMD7_HUMAN]	7.50	1	1	1	1	0.984	1.101	0.820	0.881	4.50	7.50	1	1	200	22.5	5.92
Q9UQ53	Alpha-1,3-mannosyl-glycoprotein 4-beta-N-acetylglucosaminyltransferase B OS=Homo sapiens GN=MGAT4B PE=1 SV=1 - [MGT4B_HUMAN]	5.66	1	3	3	4	1.079	0.877	0.924	0.881	9.70	5.66	4	4	548	63.2	7.87

Q14376	UDP-glucose 4-epimerase OS=Homo sapiens GN=GALE PE=1 SV=2 - [GALE_HUMAN]	19.54	1	7	7	19	0.976	1.000	1.155	0.881	26.39	19.54	11	19	348	38.3	6.73
O14787	Transportin-2 OS=Homo sapiens GN=TNPO2 PE=1 SV=3 - [TNPO2_HUMAN]	9.48	1	3	7	14	0.867	0.677	0.857	0.881	33.39	9.48	10	14	897	101.3	5.01
Q8NI27	THO complex subunit 2 OS=Homo sapiens GN=THOC2 PE=1 SV=2 - [THOC2_HUMAN]	3.01	1	5	5	10	0.950	0.753	0.929	0.881	27.98	3.01	5	10	1593	182.7	8.44
O43615	Mitochondrial import inner membrane translocase subunit TIM44 OS=Homo sapiens GN=TIMM44 PE=1 SV=2 - [TIM44_HUMAN]	25.88	1	13	13	39	0.877	1.087	0.920	0.882	103.84	25.88	23	39	452	51.3	8.32
Q9NUI1	Peroxisomal 2,4-dienoyl-CoA reductase OS=Homo sapiens GN=DECR2 PE=1 SV=1 - [DECR2_HUMAN]	21.92	1	6	6	28	1.083	1.237	1.275	0.882	51.28	21.92	10	28	292	30.8	9.22
Q8NHQ1	Centrosomal protein of 70 kDa OS=Homo sapiens GN=CEP70 PE=1 SV=2 - [CEP70_HUMAN]	5.19	1	2	2	2	1.017	0.864	0.736	0.882	4.27	5.19	2	2	597	69.7	5.71
Q9BVM2	Protein DPCD OS=Homo sapiens GN=DPCD PE=1 SV=2 - [DPCD_HUMAN]	8.87	1	2	2	4	0.932	0.713	0.846	0.882	8.16	8.87	3	4	203	23.2	9.03
Q9Y5X1	Sorting nexin-9 OS=Homo sapiens GN=SNX9 PE=1 SV=1 - [SNX9_HUMAN]	30.59	1	18	19	60	1.135	0.960	0.905	0.882	140.90	30.59	26	60	595	66.5	5.58
P33527	Multidrug resistance-associated protein 1 OS=Homo sapiens GN=ABCC1 PE=1 SV=3 - [MRP1_HUMAN]	27.63	1	35	36	128	1.537	0.610	0.731	0.882	364.44	27.63	60	128	1531	171.5	7.11

Q6P988	Protein notum homolog OS=Homo sapiens GN=NOTUM PE=2 SV=2 - [NOTUM_HUMAN]	2.42	1	1	1	1	0.697	0.742	0.587	0.882	2.36	2.42	1	1	496	55.7	7.55
Q96CP6	GRAM domain-containing protein 1A OS=Homo sapiens GN=GRAMD1A PE=1 SV=2 - [GRM1A_HUMAN]	7.18	1	3	3	7	0.994	0.853	1.003	0.882	17.78	7.18	4	7	724	80.6	6.74
Q14995	Nuclear receptor subfamily 1 group D member 2 OS=Homo sapiens GN=NR1D2 PE=1 SV=3 - [NR1D2_HUMAN	2.07	1	1	1	1	1.127	1.106	1.059	0.882	2.57	2.07	1	1	579	64.6	7.84
Q96HD9	N-acyl-aromatic-L-amino acid amidohydrolase (carboxylate-forming) OS=Homo sapiens GN=ACY3 PE=1 SV=1 - [ACY3_HUMAN]	3.45	1	1	1	2	1.329	0.265	0.972	0.882	1.89	3.45	1	2	319	35.2	5.90
Q99805	Transmembrane 9 superfamily member 2 OS=Homo sapiens GN=TM9SF2 PE=1 SV=1 - [TM9S2_HUMAN]	10.26	1	5	5	20	0.827	0.619	0.527	0.882	66.29	10.26	8	20	663	75.7	7.44
Q6P158	Putative ATP-dependent RNA helicase DHX57 OS=Homo sapiens GN=DHX57 PE=1 SV=2 - [DHX57_HUMAN	5.27	2	5	6	9	0.738	0.890	1.142	0.882	20.89	5.27	9	9	1386	155.5	7.71
O60825	6-phosphofructo-2-kinase/fructose-2,6-bisphosphatase 2 OS=Homo sapiens GN=PFKFB2 PE=1 SV=2 - [F262_HUMAN]	5.74	2	3	3	4	1.274	1.121	1.355	0.882	5.56	5.74	4	4	505	58.4	8.38
O15228	Dihydroxyacetone phosphate acyltransferase OS=Homo sapiens GN=GNPAT PE=1 SV=1 - [GNPAT_HUMAN]	4.56	1	3	4	8	1.268	0.979	1.045	0.883	13.08	4.56	6	8	680	77.1	6.57

Q96EK5	KIF1-binding protein OS=Homo sapiens GN=KIAA1279 PE=1 SV=1 - [KBP_HUMAN]	15.94	1	7	7	16	0.735	0.954	1.010	0.883	41.38	15.94	11	16	621	71.8	5.49
P49354	Protein farnesyltransferase/geranylgeranyltransferase type-1 subunit alpha OS=Homo sapiens GN=FNTA PE=1 SV=1 - [FNTA_HUMAN]	20.32	1	7	7	20	0.899	1.119	1.053	0.883	55.45	20.32	12	20	379	44.4	5.08
Q9GZY8	Mitochondrial fission factor OS=Homo sapiens GN=MFF PE=1 SV=1 - [MFF_HUMAN]	38.01	1	8	8	41	0.656	0.611	0.523	0.883	68.11	38.01	15	41	342	38.4	8.95
P18065	Insulin-like growth factor-binding protein 2 OS=Homo sapiens GN=IGFBP2 PE=1 SV=2 - [IBP2_HUMAN]	8.62	1	3	3	4	1.236	2.533	2.615	0.883	7.86	8.62	4	4	325	34.8	7.50
Q9H2D1	Mitochondrial folate transporter/carrier OS=Homo sapiens GN=SLC25A32 PE=1 SV=2 - [MFTC_HUMAN]	3.81	1	1	1	2	0.847	0.545	0.854	0.883	0.00	3.81	2	2	315	35.4	9.45
P23610	Factor VIII intron 22 protein OS=Homo sapiens GN=F8A1 PE=1 SV=2 - [F8I2_HUMAN]	7.28	1	2	2	9	1.551	1.168	1.205	0.883	15.33	7.28	4	9	371	39.1	6.84
P04209	Ig lambda chain V-II region NIG-84 OS=Homo sapiens PE=1 SV=1 - [LV211_HUMAN]	14.29	1	1	1	1	1.464	1.709	2.053	0.884	0.00	14.29	1	1	112	11.6	7.12
Q53H96	Pyrroline-5-carboxylate reductase 3 OS=Homo sapiens GN=PYCRL PE=1 SV=3 - [P5CR3_HUMAN]	18.61	1	4	4	14	0.785	0.759	0.860	0.884	42.03	18.61	6	14	274	28.6	7.72
Q99946	Proline-rich transmembrane protein 1 OS=Homo sapiens GN=PRRT1 PE=2 SV=2 - [PRRT1_HUMAN]	4.90	1	1	1	1	1.013	1.302	1.261	0.884	2.18	4.90	1	1	306	31.4	7.65

Q8IWZ6	Bardet-Biedl syndrome 7 protein OS=Homo sapiens GN=BBS7 PE=1 SV=2 - [BBS7_HUMAN]	1.40	1	1	1	1	0.859	0.938	1.000	0.884	1.93	1.40	1	1	715	80.3	6.05
Q14439	Probable G-protein coupled receptor 176 OS=Homo sapiens GN=GPR176 PE=2 SV=1 - [GP176_HUMAN	4.08	1	2	2	14	1.208	1.070	1.078	0.884	31.08	4.08	2	14	515	57.0	8.51
Q8TDG2	Actin-related protein T1 OS=Homo sapiens GN=ACTRT1 PE=2 SV=2 - [ACTT1_HUMAN]	2.93	1	1	1	1	1.098	0.909	1.046	0.884	2.75	2.93	1	1	376	41.7	6.79
Q08AF3	Schlafen family member 5 OS=Homo sapiens GN=SLFN5 PE=1 SV=1 - [SLFN5_HUMAN]	14.37	3	13	14	41	1.152	0.798	0.735	0.884	87.82	14.37	20	41	891	101.0	8.22
Q969M7	NEDD8-conjugating enzyme UBE2F OS=Homo sapiens GN=UBE2F PE=1 SV=1 - [UBE2F_HUMAN]	11.35	1	2	2	3	0.789	1.152	1.567	0.884	6.10	11.35	3	3	185	21.1	6.79
O15379	Histone deacetylase 3 OS=Homo sapiens GN=HDAC3 PE=1 SV=2 - [HDAC3_HUMAN]	5.37	1	2	2	3	0.842	1.382	1.400	0.884	7.37	5.37	3	3	428	48.8	5.16
O14618	Copper chaperone for superoxide dismutase OS=Homo sapiens GN=CCS PE=1 SV=1 - [CCS_HUMAN]	16.79	1	6	6	16	1.096	0.962	1.105	0.884	40.65	16.79	10	16	274	29.0	5.58
Q96MY7	Protein FAM161B OS=Homo sapiens GN=FAM161B PE=1 SV=2 - [F161B_HUMAN]	2.63	1	2	3	6	1.113	0.764	0.571	0.884	10.18	2.63	3	6	647	73.6	9.41
Q5TZA2	Rootletin OS=Homo sapiens GN=CROCC PE=1 SV=1 - [CROCC_HUMAN]	6.05	2	9	11	44	0.891	1.018	0.858	0.885	99.02	6.05	16	44	2017	228.4	5.50

Q8TF40	Folliculin-interacting protein 1 OS=Homo sapiens GN=FNIP1 PE=1 SV=3 - [FNIP1_HUMAN]	2.06	1	2	2	2	0.641	1.014	1.092	0.885	3.33	2.06	2	2	1166	130.5	5.52
P01033	Metalloproteinase inhibitor 1 OS=Homo sapiens GN=TIMP1 PE=1 SV=1 - [TIMP1_HUMAN]	10.14	1	2	2	8	0.625	0.761	0.859	0.885	18.45	10.14	3	8	207	23.2	8.10
O43795	Unconventional myosin-Ib OS=Homo sapiens GN=MYO1B PE=1 SV=3 - [MYO1B_HUMAN]	41.73	2	45	47	244	1.507	0.643	0.678	0.885	565.24	41.73	75	244	1136	131.9	9.38
Q14204	Cytoplasmic dynein 1 heavy chain 1 OS=Homo sapiens GN=DYNC1H1 PE=1 SV=5 - [DYHC1_HUMAN]	46.45	1	209	210	1100	1.004	0.931	0.921	0.885	2755.85	46.45	349	1100	4646	532.1	6.40
O43504	Ragulator complex protein LAMTOR5 OS=Homo sapiens GN=LAMTOR5 PE=1 SV=1 - [LTOR5_HUMAN	32.97	1	2	2	10	0.859	1.046	1.027	0.885	32.44	32.97	3	10	91	9.6	4.87
Q96J77	Tumor protein D55 OS=Homo sapiens GN=TPD52L3 PE=1 SV=2 - [TPD55_HUMAN]	4.29	1	1	1	1	1.854	0.708	0.667	0.885	0.00	4.29	1	1	140	15.5	9.95
Q9H9A6	Leucine-rich repeat-containing protein 40 OS=Homo sapiens GN=LRRC40 PE=1 SV=1 - [LRC40_HUMAN]	27.57	1	16	16	41	1.006	1.033	0.986	0.885	97.22	27.57	24	41	602	68.2	6.43
Q93008	Probable ubiquitin carboxyl-terminal hydrolase FAF-X OS=Homo sapiens GN=USP9X PE=1 SV=3 - [USP9X_HUMAN]	16.42	5	22	38	113	0.923	0.923	0.982	0.885	269.70	16.42	61	113	2570	292.1	5.80
Q13085	Acetyl-CoA carboxylase 1 OS=Homo sapiens GN=ACACA PE=1 SV=2 - [ACACA_HUMAN]	7.63	1	13	15	31	0.913	1.131	1.146	0.885	73.73	7.63	21	31	2346	265.4	6.37

P57071	PR domain zinc finger protein 15 OS=Homo sapiens GN=PRDM15 PE=2 SV=4 - [PRD15_HUMAN]	0.66	1	1	1	1	0.787	0.790	1.305	0.885	2.29	0.66	1	1	1507	169.2	8.24
Q9HC10	Otoferlin OS=Homo sapiens GN=OTOF PE=1 SV=3 - [OTOF_HUMAN]	0.40	1	1	1	1	0.940	1.418	1.846	0.885	3.16	0.40	1	1	1997	226.6	5.69
Q96CW6	Probable RNA polymerase II nuclear localization protein SLC7A6OS OS=Homo sapiens GN=SLC7A6OS PE=1 SV=2 - [S7A6O_HUMAN]	3.56	1	1	1	2	1.053	1.288	1.303	0.885	4.50	3.56	2	2	309	35.0	4.60
P23193	Transcription elongation factor A protein 1 OS=Homo sapiens GN=TCEA1 PE=1 SV=2 - [TCEA1_HUMAN	26.25	2	9	9	21	0.754	1.302	0.945	0.886	50.51	26.25	13	21	301	33.9	8.38
Q9UPQ7	E3 ubiquitin-protein ligase PDZRN3 OS=Homo sapiens GN=PDZRN3 PE=1 SV=2 - [PZRN3_HUMAN]	5.16	1	2	4	5	0.906	1.414	2.034	0.886	11.65	5.16	5	5	1066	119.5	6.02
Q96EW2	HSPB1-associated protein 1 OS=Homo sapiens GN=HSPBAP1 PE=1 SV=1 - [HBAP1_HUMAN]	2.25	1	1	1	2	1.243	1.142	1.053	0.886	2.23	2.25	2	2	488	55.1	5.60
Q15691	Microtubule-associated protein RP/EB family member 1 OS=Homo sapiens GN=MAPRE1 PE=1 SV=3 - [MARE1_HUMAN]	55.60	1	13	17	65	1.054	1.342	1.217	0.886	144.39	55.60	25	65	268	30.0	5.14
P60174	Triosephosphate isomerase OS=Homo sapiens GN=TPI1 PE=1 SV=3 - [TPIS_HUMAN]	81.82	1	23	23	896	1.117	1.158	0.995	0.886	2510.92	81.82	41	896	286	30.8	5.92
Q12974	Protein tyrosine phosphatase type IVA 2 OS=Homo sapiens GN=PTP4A2 PE=1 SV=1 - [TP4A2_HUMAN	46.71	1	3	6	20	1.598	0.891	0.963	0.886	36.36	46.71	9	20	167	19.1	8.37

P28845	Corticosteroid 11-beta-dehydrogenase isozyme 1 OS=Homo sapiens GN=HSD11B1 PE=1 SV=3 - [DHI1_HUMAN]	16.78	1	6	6	16	0.589	0.576	0.579	0.886	24.58	16.78	11	16	292	32.4	8.56
A0FGR8	Extended synaptotagmin-2 OS=Homo sapiens GN=ESYT2 PE=1 SV=1 - [ESYT2_HUMAN]	24.54	2	21	21	71	1.616	1.016	0.925	0.886	179.33	24.54	35	71	921	102.3	9.26
P51178	1-phosphatidylinositol 4,5-bisphosphate phosphodiesterase delta-1 OS=Homo sapiens GN=PLCD1 PE=1 SV=2 - [PLCD1_HUMAN]	15.87	1	11	11	32	1.362	1.074	1.293	0.886	60.55	15.87	17	32	756	85.6	6.70
O60613	15 kDa selenoprotein OS=Homo sapiens GN=SEP15 PE=1 SV=3 - [SEP15_HUMAN]	17.90	1	2	2	15	0.964	0.623	0.710	0.887	35.06	17.90	4	15	162	17.8	5.03
O95382	Mitogen-activated protein kinase kinase kinase 6 OS=Homo sapiens GN=MAP3K6 PE=1 SV=3 - [M3K6_HUMAN]	1.09	1	1	2	8	0.867	0.848	0.683	0.887	20.26	1.09	3	8	1288	142.5	7.11
Q8IY63	Angiomotin-like protein 1 OS=Homo sapiens GN=AMOTL1 PE=1 SV=1 - [AMOL1_HUMAN]	7.53	2	5	8	10	1.000	1.409	1.289	0.887	9.35	7.53	10	10	956	106.5	7.11
O94979	Protein transport protein Sec31A OS=Homo sapiens GN=SEC31A PE=1 SV=3 - [SC31A_HUMAN]	24.59	1	30	31	148	0.970	0.849	0.850	0.887	310.39	24.59	48	148	1220	132.9	6.89
Q5W5X9	Tetratricopeptide repeat protein 23 OS=Homo sapiens GN=TTC23 PE=2 SV=1 - [TTC23_HUMAN	3.58	1	1	1	2	1.237	0.817	0.740	0.887	0.00	3.58	1	2	447	50.0	8.38
P53618	Coatomer subunit beta OS=Homo sapiens GN=COPB1 PE=1 SV=3 - [COPB_HUMAN]	38.30	1	34	34	152	0.873	0.728	0.766	0.887	371.29	38.30	51	152	953	107.1	6.05

Q13616	Cullin-1 OS=Homo sapiens GN=CUL1 PE=1 SV=2 - [CUL1_HUMAN]	16.88	1	15	16	23	0.965	0.989	1.052	0.887	35.05	16.88	20	23	776	89.6	8.00
Q96EB1	Elongator complex protein 4 OS=Homo sapiens GN=ELP4 PE=1 SV=2 - [ELP4_HUMAN]	4.48	1	1	1	2	0.858	1.548	1.237	0.887	6.22	4.48	2	2	424	46.6	8.51
P04844	Dolichyl-diphosphooligosaccharide--protein glycosyltransferase subunit 2 OS=Homo sapiens GN=RPN2 PE=1 SV=3 - [RPN2_HUMAN]	30.43	1	17	17	104	0.659	0.654	0.680	0.887	258.50	30.43	28	104	631	69.2	5.69
P06744	Glucose-6-phosphate isomerase OS=Homo sapiens GN=GPI PE=1 SV=4 - [G6PI_HUMAN]	41.94	2	25	26	315	1.457	0.977	0.887	0.887	707.22	41.94	46	315	558	63.1	8.32
P53677	AP-3 complex subunit mu-2 OS=Homo sapiens GN=AP3M2 PE=1 SV=1 - [AP3M2_HUMAN]	11.72	1	3	6	12	0.951	0.776	0.743	0.887	26.98	11.72	8	12	418	46.9	7.56
P06132	Uroporphyrinogen decarboxylase OS=Homo sapiens GN=UROD PE=1 SV=2 - [DCUP_HUMAN]	11.44	1	4	4	17	0.879	1.168	0.966	0.888	30.55	11.44	7	17	367	40.8	6.14
Q7LG56	Ribonucleoside-diphosphate reductase subunit M2 B OS=Homo sapiens GN=RRM2B PE=1 SV=1 - [RIR2B_HUMAN]	12.25	1	3	5	16	1.121	0.847	1.116	0.888	37.10	12.25	6	16	351	40.7	4.97
Q96AJ9	Vesicle transport through interaction with t-SNAREs homolog 1A OS=Homo sapiens GN=VTI1A PE=1 SV=2 - [VTI1A_HUMAN	33.64	2	8	8	34	1.006	0.704	0.891	0.888	90.21	33.64	13	34	217	25.2	6.40

O95477	ATP-binding cassette sub-family A member 1 OS=Homo sapiens GN=ABCA1 PE=1 SV=3 - [ABCA1_HUMAN]	1.68	1	4	5	7	0.823	1.171	0.667	0.888	10.54	1.68	6	7	2261	254.1	6.86
Q49A88	Coiled-coil domain-containing protein 14 OS=Homo sapiens GN=CCDC14 PE=1 SV=3 - [CCD14_HUMAN]	3.57	1	2	3	4	0.971	3.019	7.966	0.888	6.84	3.57	3	4	953	106.2	8.40
Q13488	V-type proton ATPase 116 kDa subunit a isoform 3 OS=Homo sapiens GN=TCIRG1 PE=1 SV=3 - [VPP3_HUMAN]	15.66	2	11	12	33	1.035	0.760	0.722	0.888	69.98	15.66	19	33	830	92.9	7.12
Q01085	Nucleolysin TIAR OS=Homo sapiens GN=TIAL1 PE=1 SV=1 - [TIAR_HUMAN]	15.47	1	3	6	21	0.788	0.889	0.851	0.888	55.39	15.47	9	21	375	41.6	7.74
Q96LI6	Heat shock transcription factor, Y-linked OS=Homo sapiens GN=HSFY1 PE=1 SV=1 - [HSFY1_HUMAN]	2.00	1	1	1	1	1.282	1.073	1.297	0.888	2.92	2.00	1	1	401	45.1	7.18
O75935	Dynactin subunit 3 OS=Homo sapiens GN=DCTN3 PE=1 SV=1 - [DCTN3_HUMAN]	50.00	3	10	11	55	1.274	0.951	0.993	0.889	124.95	50.00	18	55	186	21.1	5.47
Q92530	Proteasome inhibitor PI31 subunit OS=Homo sapiens GN=PSMF1 PE=1 SV=2 - [PSMF1_HUMAN]	43.17	2	7	7	19	0.883	1.193	0.963	0.889	42.18	43.17	10	19	271	29.8	5.74
Q9NYJ1	Cytochrome c oxidase assembly factor 4 homolog, mitochondrial OS=Homo sapiens GN=COA4 PE=1 SV=2 - [COA4_HUMAN]	41.38	1	3	3	14	0.534	0.843	1.000	0.889	24.21	41.38	5	14	87	10.1	6.04

Q96LD4	Tripartite motif-containing protein 47 OS=Homo sapiens GN=TRIM47 PE=1 SV=2 - [TRI47_HUMAN]	5.02	1	3	3	12	1.360	1.877	1.671	0.889	26.56	5.02	5	12	638	69.5	6.44
Q8IYB5	Stromal membrane-associated protein 1 OS=Homo sapiens GN=SMAP1 PE=1 SV=2 - [SMAP1_HUMAN]	7.07	1	4	4	12	0.873	1.109	1.147	0.889	17.06	7.07	6	12	467	50.4	8.75
P53007	Tricarboxylate transport protein, mitochondrial OS=Homo sapiens GN=SLC25A1 PE=1 SV=2 - [TXTP_HUMAN]	16.08	1	5	5	23	0.816	0.824	0.821	0.889	63.49	16.08	8	23	311	34.0	9.89
P14317	Hematopoietic lineage cell-specific protein OS=Homo sapiens GN=HCLS1 PE=1 SV=3 - [HCLS1_HUMAN]	1.65	1	1	1	3	0.542	0.580	0.690	0.889	7.99	1.65	1	3	486	54.0	4.81
O15498	Synaptobrevin homolog YKT6 OS=Homo sapiens GN=YKT6 PE=1 SV=1 - [YKT6_HUMAN]	55.56	1	11	11	59	0.822	0.825	0.960	0.889	165.55	55.56	20	59	198	22.4	6.92
Q9Y485	DmX-like protein 1 OS=Homo sapiens GN=DMXL1 PE=1 SV=3 - [DMXL1_HUMAN]	0.96	2	3	3	6	0.884	0.872	1.005	0.889	13.83	0.96	4	6	3027	337.6	6.34
P19623	Spermidine synthase OS=Homo sapiens GN=SRM PE=1 SV=1 - [SPEE_HUMAN]	17.55	1	6	6	29	0.620	0.759	0.750	0.889	70.87	17.55	10	29	302	33.8	5.49
P23443	Ribosomal protein S6 kinase beta-1 OS=Homo sapiens GN=RPS6KB1 PE=1 SV=2 - [KS6B1_HUMAN]	8.76	1	3	4	12	0.965	0.887	0.987	0.889	21.92	8.76	7	12	525	59.1	6.65

P15735	Phosphorylase b kinase gamma catalytic chain, liver/testis isoform OS=Homo sapiens GN=PHKG2 PE=1 SV=1 - [PHKG2_HUMAN	7.14	1	3	3	11	0.663	1.126	1.294	0.889	25.12	7.14	5	11	406	46.4	6.38
Q96BP2	Coiled-coil-helix-coiled-coil-helix domain-containing protein 1 OS=Homo sapiens GN=CHCHD1 PE=1 SV=1 - [CHCH1_HUMAN]	26.27	1	2	2	9	1.820	0.991	1.180	0.890	24.22	26.27	3	9	118	13.5	10.21
P55268	Laminin subunit beta-2 OS=Homo sapiens GN=LAMB2 PE=1 SV=2 - [LAMB2_HUMAN]	19.30	1	29	29	103	0.687	0.807	1.187	0.890	238.99	19.30	45	103	1798	195.9	6.52
Q32P28	Prolyl 3-hydroxylase 1 OS=Homo sapiens GN=LEPRE1 PE=1 SV=2 - [P3H1_HUMAN]	34.65	1	17	19	110	0.614	0.639	0.689	0.890	332.35	34.65	31	110	736	83.3	5.14
O75955	Flotillin-1 OS=Homo sapiens GN=FLOT1 PE=1 SV=3 - [FLOT1_HUMAN]	49.88	1	17	18	65	1.071	1.370	0.949	0.890	133.07	49.88	31	65	427	47.3	7.49
Q8TAA9	Vang-like protein 1 OS=Homo sapiens GN=VANGL1 PE=1 SV=1 - [VANG1_HUMAN]	12.21	1	5	5	9	1.064	1.075	0.816	0.890	17.68	12.21	7	9	524	59.9	8.81
Q5VU43	Myomegalin OS=Homo sapiens GN=PDE4DIP PE=1 SV=1 - [MYOME_HUMA N]	7.12	3	14	18	34	0.875	0.813	0.957	0.890	74.69	7.12	23	34	2346	264.9	5.44
Q9Y324	rRNA-processing protein FCF1 homolog OS=Homo sapiens GN=FCF1 PE=2 SV=1 - [FCF1_HUMAN]	3.03	1	1	1	1	0.750	0.937	0.670	0.890	2.49	3.03	1	1	198	23.4	9.70
Q86Y38	Xylosyltransferase 1 OS=Homo sapiens GN=XYLT1 PE=1 SV=1 - [XYLT1_HUMAN]	4.38	1	2	3	5	0.950	0.641	0.946	0.890	9.58	4.38	3	5	959	107.5	9.22

Q9BUM1	Glucose-6-phosphatase 3 OS=Homo sapiens GN=G6PC3 PE=1 SV=2 - [G6PC3_HUMAN]	2.31	1	1	1	1	0.808	0.874	0.680	0.890	0.00	2.31	1	1	346	38.7	8.21
Q8WVM8	Sec1 family domain-containing protein 1 OS=Homo sapiens GN=SCFD1 PE=1 SV=4 - [SCFD1_HUMAN]	36.45	1	20	20	53	1.004	0.987	0.965	0.890	155.55	36.45	31	53	642	72.3	6.27
O75874	Isocitrate dehydrogenase [NADP] cytoplasmic OS=Homo sapiens GN=IDH1 PE=1 SV=2 - [IDHC_HUMAN]	51.69	1	24	24	128	1.058	0.927	1.087	0.891	361.01	51.69	35	128	414	46.6	7.01
Q9BT40	Inositol polyphosphate 5-phosphatase K OS=Homo sapiens GN=INPP5K PE=1 SV=3 - [INP5K_HUMAN]	2.23	1	1	1	6	1.270	0.734	0.887	0.891	18.42	2.23	2	6	448	51.1	6.54
Q9NXW9	Alpha-ketoglutarate-dependent dioxygenase alkB homolog 4 OS=Homo sapiens GN=ALKBH4 PE=1 SV=1 - [ALKB4_HUMAN]	2.32	1	1	1	1	1.431	1.103	0.891	0.891	2.74	2.32	1	1	302	33.8	6.67
P05089	Arginase-1 OS=Homo sapiens GN=ARG1 PE=1 SV=2 - [ARGI1_HUMAN]	2.17	1	2	2	7	1.936	1.060	0.833	0.891	17.21	2.17	3	7	322	34.7	7.21
Q9BY44	Eukaryotic translation initiation factor 2A OS=Homo sapiens GN=EIF2A PE=1 SV=3 - [EIF2A_HUMAN]	35.56	1	17	18	68	0.948	0.918	1.003	0.891	164.40	35.56	29	68	585	64.9	8.87
O60927	Protein phosphatase 1 regulatory subunit 11 OS=Homo sapiens GN=PPP1R11 PE=1 SV=1 - [PP1RB_HUMAN]	11.90	1	1	1	9	1.121	1.065	1.087	0.891	36.83	11.90	2	9	126	13.9	7.01

Q8TBG4	Ethanolamine-phosphate phospho-lyase OS=Homo sapiens GN=ETNPPL PE=1 SV=1 - [AT2L1_HUMAN]	1.60	1	1	1	2	0.545	0.683	0.904	0.892	0.00	1.60	1	2	499	55.6	8.10
Q9UP95	Solute carrier family 12 member 4 OS=Homo sapiens GN=SLC12A4 PE=1 SV=2 - [S12A4_HUMAN]	13.73	2	15	15	39	1.117	0.726	0.683	0.892	97.04	13.73	25	39	1085	120.6	6.44
Q9NYM9	BET1-like protein OS=Homo sapiens GN=BET1L PE=1 SV=1 - [BET1L_HUMAN]	36.04	1	4	4	19	0.746	0.915	0.947	0.892	45.81	36.04	7	19	111	12.4	8.16
O60284	Suppression of tumorigenicity 18 protein OS=Homo sapiens GN=ST18 PE=1 SV=1 - [ST18_HUMAN]	2.29	1	2	2	4	1.429	1.942	1.391	0.892	7.91	2.29	3	4	1047	115.1	6.06
O95825	Quinone oxidoreductase-like protein 1 OS=Homo sapiens GN=CRYZL1 PE=1 SV=2 - [QORL1_HUMAN]	24.93	1	8	8	15	0.866	0.862	0.980	0.892	34.09	24.93	12	15	349	38.7	5.78
P13726	Tissue factor OS=Homo sapiens GN=F3 PE=1 SV=1 - [TF_HUMAN]	29.83	1	8	8	18	3.308	0.400	1.060	0.892	48.80	29.83	10	18	295	33.0	7.03
Q08722	Leukocyte surface antigen CD47 OS=Homo sapiens GN=CD47 PE=1 SV=1 - [CD47_HUMAN]	8.67	1	3	3	21	1.458	1.282	0.761	0.892	41.85	8.67	6	21	323	35.2	7.21
Q4FZB7	Histone-lysine N-methyltransferase SUV420H1 OS=Homo sapiens GN=SUV420H1 PE=1 SV=4 - [SV421_HUMAN]	1.02	1	1	1	1	1.401	1.043	0.856	0.892	2.49	1.02	1	1	885	99.1	8.78
P01112	GTPase HRas OS=Homo sapiens GN=HRAS PE=1 SV=1 - [RASH_HUMAN]	52.91	1	4	6	20	0.989	1.061	1.113	0.892	52.16	52.91	8	20	189	21.3	5.31

Q9UM22	Mammalian ependymin-related protein 1 OS=Homo sapiens GN=EPDR1 PE=1 SV=2 - [EPDR1_HUMAN]	15.63	1	3	3	19	0.629	1.399	0.795	0.892	51.40	15.63	5	19	224	25.4	6.60
Q9UJ83	2-hydroxyacyl-CoA lyase 1 OS=Homo sapiens GN=HACL1 PE=1 SV=2 - [HACL1_HUMAN]	2.08	1	1	1	3	1.250	1.528	1.832	0.893	3.39	2.08	2	3	578	63.7	7.36
Q99501	GAS2-like protein 1 OS=Homo sapiens GN=GAS2L1 PE=1 SV=2 - [GA2L1_HUMAN]	1.47	1	1	1	5	0.975	0.923	0.897	0.893	13.50	1.47	2	5	681	72.7	10.05
Q9NWT6	Hypoxia-inducible factor 1-alpha inhibitor OS=Homo sapiens GN=HIF1AN PE=1 SV=2 - [HIF1N_HUMAN]	17.48	1	6	6	22	0.837	1.200	1.264	0.894	56.20	17.48	8	22	349	40.3	5.57
Q9HBR0	Putative sodium-coupled neutral amino acid transporter 10 OS=Homo sapiens GN=SLC38A10 PE=1 SV=2 - [S38AA_HUMAN	19.39	1	16	16	45	0.772	0.970	0.835	0.894	117.71	19.39	26	45	1119	119.7	5.73
Q6ZS30	Neurobeachin-like protein 1 OS=Homo sapiens GN=NBEAL1 PE=2 SV=3 - [NBEL1_HUMAN]	1.82	1	2	3	5	1.009	1.256	1.468	0.894	12.73	1.82	3	5	2694	307.0	6.44
Q9UBV8	Peflin OS=Homo sapiens GN=PEF1 PE=1 SV=1 - [PEF1_HUMAN]	15.49	1	5	5	19	0.917	0.933	1.052	0.894	37.75	15.49	8	19	284	30.4	6.54
Q9ULC4	Malignant T-cell-amplified sequence 1 OS=Homo sapiens GN=MCTS1 PE=1 SV=1 - [MCTS1_HUMAN]	48.62	1	8	8	30	0.888	0.748	0.870	0.894	78.60	48.62	12	30	181	20.5	8.82
Q9BQS7	Hephaestin OS=Homo sapiens GN=HEPH PE=2 SV=3 - [HEPH_HUMAN]	0.69	1	1	1	1	0.231	2.873	1.577	0.894	0.00	0.69	1	1	1158	130.4	5.99

Q9UH62	Armadillo repeat-containing X-linked protein 3 OS=Homo sapiens GN=ARMCX3 PE=1 SV=1 - [ARMX3_HUMA	13.46	1	4	4	8	0.716	0.683	0.875	0.894	17.27	13.46	5	8	379	42.5	8.37
O75323	Protein NipSnap homolog 2 OS=Homo sapiens GN=GBAS PE=1 SV=1 - [NIPS2_HUMAN]	37.41	1	9	12	41	0.852	1.782	1.284	0.894	85.21	37.41	16	41	286	33.7	9.36
Q8NHU6	Tudor domain-containing protein 7 OS=Homo sapiens GN=TDRD7 PE=1 SV=2 - [TDRD7_HUMAN]	3.46	1	3	3	4	1.353	1.066	1.145	0.895	5.69	3.46	3	4	1098	123.5	7.21
Q12923	Tyrosine-protein phosphatase non-receptor type 13 OS=Homo sapiens GN=PTPN13 PE=1 SV=2 - [PTN13_HUMAN]	1.45	1	3	3	9	1.369	0.539	1.053	0.895	15.69	1.45	5	9	2485	276.7	6.42
P15880	40S ribosomal protein S2 OS=Homo sapiens GN=RPS2 PE=1 SV=2 - [RS2_HUMAN]	60.07	1	19	19	189	0.855	0.807	0.805	0.895	429.45	60.07	31	189	293	31.3	10.24
Q96CN9	GRIP and coiled-coil domain-containing protein 1 OS=Homo sapiens GN=GCC1 PE=1 SV=1 - [GCC1_HUMAN]	10.06	1	6	8	17	0.850	1.035	1.205	0.895	36.58	10.06	10	17	775	87.8	5.45
O14657	Torsin-1B OS=Homo sapiens GN=TOR1B PE=1 SV=2 - [TOR1B_HUMA N]	18.75	1	6	6	19	1.038	0.713	1.071	0.895	50.22	18.75	10	19	336	38.0	8.54
Q6NXT6	Transmembran e anterior posterior transformation protein 1 homolog OS=Homo sapiens GN=TAPT1 PE=1 SV=1 - [TAPT1_HUMAN]	3.00	1	2	2	5	1.233	0.196	0.264	0.895	11.57	3.00	3	5	567	64.2	8.28

O14531	Dihydropyrimidinase-related protein 4 OS=Homo sapiens GN=DPYSL4 PE=1 SV=2 - [DPYL4_HUMAN]	21.33	1	7	7	29	0.972	0.808	0.838	0.895	90.25	21.33	12	29	572	61.8	7.09
Q7L5N1	COP9 signalosome complex subunit 6 OS=Homo sapiens GN=COPS6 PE=1 SV=1 - [CSN6_HUMAN]	33.94	1	9	9	21	0.831	0.781	0.835	0.895	47.66	33.94	12	21	327	36.1	5.73
Q96CD0	F-box/LRR-repeat protein 8 OS=Homo sapiens GN=FBXL8 PE=1 SV=1 - [FBXL8_HUMAN]	11.50	1	3	3	7	0.953	0.804	1.004	0.895	14.06	11.50	4	7	374	40.5	7.31
Q8IVF5	T-lymphoma invasion and metastasis-inducing protein 2 OS=Homo sapiens GN=TIAM2 PE=2 SV=4 - [TIAM2_HUMAN]	0.82	1	1	1	1	0.973	0.874	0.823	0.895	2.96	0.82	1	1	1701	190.0	7.21
P50238	Cysteine-rich protein 1 OS=Homo sapiens GN=CRIP1 PE=1 SV=3 - [CRIP1_HUMAN]	18.18	1	1	2	2	5.338	1.381	0.855	0.895	1.68	18.18	2	2	77	8.5	8.75
Q8TEB1	DDB1- and CUL4-associated factor 11 OS=Homo sapiens GN=DCAF11 PE=1 SV=1 - [DCA11_HUMAN	2.38	1	1	1	4	0.906	1.250	0.936	0.895	6.74	2.38	2	4	546	61.6	6.40
Q0VDG4	Secernin-3 OS=Homo sapiens GN=SCRN3 PE=1 SV=1 - [SCRN3_HUMAN]	10.14	1	4	4	15	0.532	1.031	1.081	0.895	25.87	10.14	5	15	424	48.5	5.55
O00273	DNA fragmentation factor subunit alpha OS=Homo sapiens GN=DFFA PE=1 SV=1 - [DFFA_HUMAN]	25.08	1	7	7	20	0.859	1.062	0.980	0.895	54.12	25.08	11	20	331	36.5	4.79
Q7L7X3	Serine/threonine-protein kinase TAO1 OS=Homo sapiens GN=TAOK1 PE=1 SV=1 - [TAOK1_HUMAN]	12.69	1	8	14	46	0.975	1.031	0.927	0.895	109.78	12.69	23	46	1001	116.0	7.65

Q9P2X0	Dolichol-phosphate mannosyltransferase subunit 3 OS=Homo sapiens GN=DPM3 PE=1 SV=2 - [DPM3_HUMAN]	10.87	1	1	1	8	0.658	0.827	0.981	0.895	17.10	10.87	2	8	92	10.1	5.94
O75608	Acyl-protein thioesterase 1 OS=Homo sapiens GN=LYPLA1 PE=1 SV=1 - [LYPA1_HUMAN]	24.78	1	4	4	23	1.076	0.986	0.938	0.895	39.57	24.78	8	23	230	24.7	6.77
Q9P000	COMM domain-containing protein 9 OS=Homo sapiens GN=COMMD9 PE=1 SV=2 - [COMD9_HUMAN]	40.40	1	6	6	12	0.919	0.959	1.077	0.895	31.55	40.40	8	12	198	21.8	5.88
Q86Y82	Syntaxin-12 OS=Homo sapiens GN=STX12 PE=1 SV=1 - [STX12_HUMAN]	27.90	1	7	7	29	0.843	0.786	0.744	0.895	58.70	27.90	11	29	276	31.6	5.59
Q5SRI9	Glycoprotein endo-alpha-1,2-mannosidase OS=Homo sapiens GN=MANEA PE=1 SV=1 - [MANEA_HUMA	1.95	1	1	1	1	0.740	2.124	1.205	0.895	0.00	1.95	1	1	462	53.6	9.11
Q99645	Epiphycan OS=Homo sapiens GN=EPYC PE=2 SV=3 - [EPYC_HUMAN]	4.35	1	1	1	1	0.475	0.283	0.570	0.896	2.71	4.35	1	1	322	36.6	4.81
Q14318	Peptidyl-prolyl cis-trans isomerase FKBP8 OS=Homo sapiens GN=FKBP8 PE=1 SV=2 - [FKBP8_HUMAN]	22.82	1	7	7	39	0.818	1.150	1.047	0.896	116.78	22.82	12	39	412	44.5	4.84
Q9H0R4	Haloacid dehalogenase-like hydrolase domain-containing protein 2 OS=Homo sapiens GN=HDHD2 PE=1 SV=1 - [HDHD2_HUMAN]	10.81	1	3	3	4	1.379	1.084	0.712	0.896	6.07	10.81	4	4	259	28.5	6.24
Q9Y4A8	Nuclear factor erythroid 2-related factor 3 OS=Homo sapiens GN=NFE2L3 PE=1 SV=1 - [NF2L3_HUMAN]	1.87	1	1	1	2	0.798	0.843	1.020	0.896	0.00	1.87	1	2	694	76.1	5.44

Q8N1B4	Vacuolar protein sorting-associated protein 52 homolog OS=Homo sapiens GN=VPS52 PE=1 SV=1 - [VPS52_HUMAN]	13.14	1	8	8	25	0.954	1.054	0.967	0.896	82.82	13.14	11	25	723	82.2	5.99
Q9Y6A9	Signal peptidase complex subunit 1 OS=Homo sapiens GN=SPCS1 PE=1 SV=4 - [SPCS1_HUMAN	31.37	1	2	2	26	1.001	0.946	0.846	0.896	63.47	31.37	3	26	102	11.8	9.31
Q14738	Serine/threonine-protein phosphatase 2A 56 kDa regulatory subunit delta isoform OS=Homo sapiens GN=PPP2R5D PE=1 SV=1 - [2A5D_HUMAN]	24.09	1	11	14	61	1.105	1.061	1.082	0.896	150.38	24.09	22	61	602	69.9	8.13
Q9NVS9	Pyridoxine-5'-phosphate oxidase OS=Homo sapiens GN=PNPO PE=1 SV=1 - [PNPO_HUMAN]	29.50	1	5	5	20	0.828	0.875	1.009	0.896	56.17	29.50	8	20	261	30.0	7.06
Q8N3K9	Cardiomyopathy-associated protein 5 OS=Homo sapiens GN=CMYA5 PE=1 SV=3 - [CMYA5_HUMAN]	0.98	1	3	4	13	0.600	0.875	0.777	0.896	26.58	0.98	4	13	4069	448.9	4.78
O75494	Serine/arginine-rich splicing factor 10 OS=Homo sapiens GN=SRSF10 PE=1 SV=1 - [SRS10_HUMAN]	22.52	2	7	7	32	0.692	1.013	0.919	0.897	76.11	22.52	11	32	262	31.3	11.27
Q8N2G6	Zinc finger CCHC domain-containing protein 24 OS=Homo sapiens GN=ZCCHC24 PE=1 SV=1 - [ZCH24_HUMAN]	21.99	1	5	5	6	1.436	0.983	1.024	0.897	14.96	21.99	5	6	241	26.9	8.70
P55058	Phospholipid transfer protein OS=Homo sapiens GN=PLTP PE=1 SV=1 - [PLTP_HUMAN]	1.83	1	1	1	2	0.646	0.793	0.803	0.897	3.74	1.83	1	2	493	54.7	7.01

Q16513	Serine/threonine-protein kinase N2 OS=Homo sapiens GN=PKN2 PE=1 SV=1 - [PKN2_HUMAN]	11.79	2	7	10	32	0.914	1.342	0.966	0.897	62.92	11.79	16	32	984	112.0	6.30
P09972	Fructose-bisphosphate aldolase C OS=Homo sapiens GN=ALDOC PE=1 SV=2 - [ALDOC_HUMAN]	58.24	2	14	23	435	1.549	1.205	1.048	0.897	1242.04	58.24	34	435	364	39.4	6.87
Q15293	Reticulocalbin-1 OS=Homo sapiens GN=RCN1 PE=1 SV=1 - [RCN1_HUMAN]	58.61	1	21	21	398	0.851	0.724	0.709	0.897	1174.46	58.61	36	398	331	38.9	5.00
Q9NWU2	Glucose-induced degradation protein 8 homolog OS=Homo sapiens GN=GID8 PE=1 SV=1 - [GID8_HUMAN]	17.54	1	3	3	14	0.700	0.870	0.968	0.897	34.34	17.54	5	14	228	26.7	4.97
Q8TCJ0	F-box only protein 25 OS=Homo sapiens GN=FBXO25 PE=1 SV=3 - [FBX25_HUMAN]	3.00	1	1	1	1	0.927	1.433	1.491	0.897	1.97	3.00	1	1	367	43.3	8.09
Q9UJ41	Rab5 GDP/GTP exchange factor OS=Homo sapiens GN=RABGEF1 PE=1 SV=2 - [RABX5_HUMAN	20.06	1	12	12	40	0.835	0.832	0.906	0.897	107.57	20.06	17	40	708	79.3	6.81
Q96GR2	Long-chain-fatty-acid--CoA ligase ACSBG1 OS=Homo sapiens GN=ACSBG1 PE=1 SV=2 - [ACBG1_HUMAN]	3.59	1	2	2	2	0.504	0.166	0.266	0.897	3.34	3.59	2	2	724	81.2	6.02
O60783	28S ribosomal protein S14, mitochondrial OS=Homo sapiens GN=MRPS14 PE=1 SV=1 - [RT14_HUMAN]	28.91	1	3	3	17	0.686	1.173	0.941	0.897	46.73	28.91	6	17	128	15.1	11.41
Q9ULJ6	Zinc finger MIZ domain-containing protein 1 OS=Homo sapiens GN=ZMIZ1 PE=1 SV=3 - [ZMIZ1_HUMAN]	3.00	2	3	3	5	0.987	1.022	1.048	0.897	7.55	3.00	3	5	1067	115.4	7.46

Q8IY18	Structural maintenance of chromosomes protein 5 OS=Homo sapiens GN=SMC5 PE=1 SV=2 - [SMC5_HUMAN]	1.36	1	1	2	2	0.656	0.911	0.991	0.898	4.43	1.36	2	2	1101	128.7	8.38
P30411	B2 bradykinin receptor OS=Homo sapiens GN=BDKRB2 PE=1 SV=2 - [BKRB2_HUMAN]	5.63	1	2	2	5	1.772	0.563	0.607	0.898	9.77	5.63	3	5	391	44.4	8.13
Q9UBV2	Protein sel-1 homolog 1 OS=Homo sapiens GN=SEL1L PE=1 SV=3 - [SE1L1_HUMAN]	10.83	1	7	7	20	1.041	0.797	0.654	0.898	58.21	10.83	11	20	794	88.7	5.39
Q96Q15	Serine/threonine-protein kinase SMG1 OS=Homo sapiens GN=SMG1 PE=1 SV=3 - [SMG1_HUMAN]	2.08	2	5	6	9	0.766	1.090	1.062	0.898	15.38	2.08	8	9	3661	410.2	6.46
P25788	Proteasome subunit alpha type-3 OS=Homo sapiens GN=PSMA3 PE=1 SV=2 - [PSA3_HUMAN]	37.65	1	10	10	40	1.101	0.821	0.847	0.898	79.52	37.65	17	40	255	28.4	5.33
Q86W42	THO complex subunit 6 homolog OS=Homo sapiens GN=THOC6 PE=1 SV=1 - [THOC6_HUMAN]	4.11	1	1	1	3	1.117	1.190	0.678	0.898	9.04	4.11	1	3	341	37.5	7.43
P55735	Protein SEC13 homolog OS=Homo sapiens GN=SEC13 PE=1 SV=3 - [SEC13_HUMAN]	31.99	1	8	8	55	0.708	0.820	0.760	0.898	144.73	31.99	11	55	322	35.5	5.48
O60547	GDP-mannose 4,6 dehydratase OS=Homo sapiens GN=GMDS PE=1 SV=1 - [GMDS_HUMAN]	36.29	1	11	11	32	0.689	0.982	0.792	0.898	88.07	36.29	17	32	372	41.9	7.31
Q9NU19	TBC1 domain family member 22B OS=Homo sapiens GN=TBC1D22B PE=1 SV=3 - [TB22B_HUMAN]	8.91	1	3	4	5	1.290	0.752	0.979	0.898	10.29	8.91	5	5	505	59.0	7.39

Q00535	Cyclin-dependent-like kinase 5 OS=Homo sapiens GN=CDK5 PE=1 SV=3 - [CDK5_HUMAN]	16.78	6	3	4	28	1.371	1.167	1.139	0.898	71.07	16.78	8	28	292	33.3	7.66
Q8TE68	Epidermal growth factor receptor kinase substrate 8-like protein 1 OS=Homo sapiens GN=EPS8L1 PE=1 SV=3 - [ES8L1_HUMAN]	1.66	1	1	1	1	0.976	1.012	1.739	0.898	2.76	1.66	1	1	723	80.2	6.04
A6NHX0	GATS-like protein 2 OS=Homo sapiens GN=GATSL2 PE=2 SV=3 - [GATL2_HUMAN]	13.98	2	2	2	4	0.471	2.286	1.638	0.898	2.34	13.98	2	4	329	36.0	5.16
Q92734	Protein TFG OS=Homo sapiens GN=TFG PE=1 SV=2 - [TFG_HUMAN]	35.00	2	12	14	84	0.834	0.767	0.942	0.899	222.12	35.00	21	84	400	43.4	5.10
Q66GS9	Centrosomal protein of 135 kDa OS=Homo sapiens GN=CEP135 PE=1 SV=2 - [CP135_HUMAN]	3.95	2	2	5	8	0.963	1.702	1.712	0.899	10.45	3.95	5	8	1140	133.4	6.21
P62191	26S protease regulatory subunit 4 OS=Homo sapiens GN=PSMC1 PE=1 SV=1 - [PRS4_HUMAN]	57.27	1	25	27	204	0.899	1.001	1.058	0.899	455.26	57.27	41	204	440	49.2	6.21
Q15819	Ubiquitin-conjugating enzyme E2 variant 2 OS=Homo sapiens GN=UBE2V2 PE=1 SV=4 - [UB2V2_HUMAN]	57.93	1	4	8	133	0.970	0.952	0.955	0.899	394.82	57.93	13	133	145	16.4	8.09
Q9Y5P4	Collagen type IV alpha-3-binding protein OS=Homo sapiens GN=COL4A3BP PE=1 SV=1 - [C43BP_HUMAN]	5.77	1	3	3	6	0.998	1.300	1.072	0.899	14.18	5.77	6	6	624	70.8	5.48
O43402	ER membrane protein complex subunit 8 OS=Homo sapiens GN=EMC8 PE=1 SV=1 - [EMC8_HUMAN]	46.67	1	9	9	22	0.696	0.776	0.735	0.899	51.46	46.67	14	22	210	23.8	6.40

O00203	AP-3 complex subunit beta-1 OS=Homo sapiens GN=AP3B1 PE=1 SV=3 - [AP3B1_HUMAN]	22.85	2	26	28	70	0.896	0.893	0.891	0.899	152.83	22.85	39	70	1094	121.2	6.04
P62910	60S ribosomal protein L32 OS=Homo sapiens GN=RPL32 PE=1 SV=2 - [RL32_HUMAN]	34.81	1	6	6	34	0.732	0.822	0.688	0.899	88.86	34.81	10	34	135	15.8	11.33
P19784	Casein kinase II subunit alpha' OS=Homo sapiens GN=CSNK2A2 PE=1 SV=1 - [CSK22_HUMAN	50.57	1	15	17	44	0.975	0.925	1.013	0.899	107.87	50.57	27	44	350	41.2	8.56
P51148	Ras-related protein Rab-5C OS=Homo sapiens GN=RAB5C PE=1 SV=2 - [RAB5C_HUMAN]	44.91	1	5	7	58	0.998	0.809	0.968	0.899	178.55	44.91	12	58	216	23.5	8.41
Q06124	Tyrosine-protein phosphatase non-receptor type 11 OS=Homo sapiens GN=PTPN11 PE=1 SV=2 - [PTN11_HUMAN]	39.20	1	23	23	90	1.030	0.908	0.964	0.899	212.42	39.20	37	90	597	68.4	7.30
Q8NFP9	Neurobeachin OS=Homo sapiens GN=NBEA PE=1 SV=3 - [NBEA_HUMAN]	1.05	1	2	3	6	0.968	0.945	0.988	0.899	12.96	1.05	4	6	2946	327.6	6.18
Q9Y2A7	Nck-associated protein 1 OS=Homo sapiens GN=NCKAP1 PE=1 SV=1 - [NCKP1_HUMAN]	18.97	2	24	24	101	1.111	1.058	1.037	0.900	195.98	18.97	44	101	1128	128.7	6.62
O60427	Fatty acid desaturase 1 OS=Homo sapiens GN=FADS1 PE=1 SV=3 - [FADS1_HUMAN]	3.83	1	2	2	10	0.617	0.484	0.759	0.900	12.61	3.83	4	10	444	51.9	8.87
P57058	Hormonally up-regulated neu tumor-associated kinase OS=Homo sapiens GN=HUNK PE=1 SV=1 - [HUNK_HUMAN]	2.94	1	1	2	2	1.295	0.650	0.880	0.900	1.99	2.94	2	2	714	79.6	9.13

Q9UII2	ATPase inhibitor, mitochondrial OS=Homo sapiens GN=ATPIF1 PE=1 SV=1 - [ATIF1_HUMAN]	21.70	1	3	4	21	0.880	1.217	1.219	0.900	41.92	21.70	6	21	106	12.2	9.35
P22061	Protein-L-isoaspartate(D-aspartate) O-methyltransferase OS=Homo sapiens GN=PCMT1 PE=1 SV=4 - [PIMT_HUMAN]	53.74	2	15	15	80	1.034	1.153	1.098	0.900	183.69	53.74	24	80	227	24.6	7.21
O15258	Protein RER1 OS=Homo sapiens GN=RER1 PE=1 SV=1 - [RER1_HUMAN]	9.18	1	2	2	7	0.890	0.610	0.617	0.900	9.43	9.18	3	7	196	22.9	9.54
O95159	Zinc finger protein-like 1 OS=Homo sapiens GN=ZFPL1 PE=1 SV=2 - [ZFPL1_HUMAN]	5.81	1	1	1	2	0.878	0.955	1.351	0.900	4.69	5.81	2	2	310	34.1	8.07
Q96G28	Cilia- and flagella-associated protein 36 OS=Homo sapiens GN=CFAP36 PE=1 SV=2 - [CFA36_HUMAN	4.09	1	1	2	2	0.755	1.208	1.175	0.900	4.60	4.09	2	2	342	39.4	4.97
Q9H9Q2	COP9 signalosome complex subunit 7b OS=Homo sapiens GN=COPS7B PE=1 SV=1 - [CSN7B_HUMAN]	23.48	1	5	5	20	0.787	0.995	1.005	0.900	53.51	23.48	8	20	264	29.6	6.15
P55884	Eukaryotic translation initiation factor 3 subunit B OS=Homo sapiens GN=EIF3B PE=1 SV=3 - [EIF3B_HUMAN]	36.24	1	27	27	109	0.947	0.747	0.784	0.900	294.64	36.24	45	109	814	92.4	5.00
Q9UPT5	Exocyst complex component 7 OS=Homo sapiens GN=EXOC7 PE=1 SV=3 - [EXOC7_HUMAN]	39.86	1	26	26	94	1.153	0.984	1.149	0.900	252.70	39.86	41	94	735	83.3	6.79
P25787	Proteasome subunit alpha type-2 OS=Homo sapiens GN=PSMA2 PE=1 SV=2 - [PSA2_HUMAN]	51.28	1	10	10	55	1.049	0.991	0.953	0.900	127.55	51.28	16	55	234	25.9	7.43

Q6GQQ9	OTU domain-containing protein 7B OS=Homo sapiens GN=OTUD7B PE=1 SV=1 - [OTU7B_HUMAN]	3.56	2	2	2	6	0.913	1.244	1.067	0.900	19.70	3.56	3	6	843	92.5	6.71
P21359	Neurofibromin OS=Homo sapiens GN=NF1 PE=1 SV=2 - [NF1_HUMAN]	5.14	1	16	16	38	0.880	0.782	0.878	0.900	84.65	5.14	22	38	2839	319.2	7.39
Q9NSK0	Kinesin light chain 4 OS=Homo sapiens GN=KLC4 PE=1 SV=3 - [KLC4_HUMAN]	39.42	2	17	22	81	0.865	1.177	1.192	0.901	196.30	39.42	35	81	619	68.6	6.18
Q15147	1-phosphatidylinositol 4,5-bisphosphate phosphodiesterase beta-4 OS=Homo sapiens GN=PLCB4 PE=1 SV=3 - [PLCB4_HUMAN]	1.11	1	1	2	3	1.378	0.689	0.741	0.901	6.23	1.11	2	3	1175	134.4	6.90
Q8NF91	Nesprin-1 OS=Homo sapiens GN=SYNE1 PE=1 SV=4 - [SYNE1_HUMAN]	7.55	2	52	60	142	0.849	1.002	1.067	0.901	297.76	7.55	84	142	8797	####	5.53
Q9NX46	Poly(ADP-ribose) glycohydrolase ARH3 OS=Homo sapiens GN=ADPRHL2 PE=1 SV=1 - [ARHL2_HUMAN]	30.30	1	9	9	39	1.344	1.299	1.235	0.901	82.65	30.30	13	39	363	38.9	5.07
Q86YB8	ERO1-like protein beta OS=Homo sapiens GN=ERO1LB PE=1 SV=2 - [ERO1B_HUMAN]	8.14	1	2	4	8	1.006	1.015	1.400	0.901	17.05	8.14	5	8	467	53.5	7.99
Q96C01	Protein FAM136A OS=Homo sapiens GN=FAM136A PE=1 SV=1 - [F136A_HUMAN]	25.36	1	4	4	12	0.847	1.258	0.975	0.901	26.77	25.36	7	12	138	15.6	7.61
Q9BWD1	Acetyl-CoA acetyltransferase, cytosolic OS=Homo sapiens GN=ACAT2 PE=1 SV=2 - [THIC_HUMAN]	32.49	1	11	12	33	2.168	0.484	0.952	0.901	71.41	32.49	19	33	397	41.3	6.92

P16298	Serine/threonine-protein phosphatase 2B catalytic subunit beta isoform OS=Homo sapiens GN=PPP3CB PE=1 SV=2 - [PP2BB_HUMAN	19.08	1	6	10	40	0.790	1.584	1.456	0.901	76.19	19.08	16	40	524	59.0	5.91
P48723	Heat shock 70 kDa protein 13 OS=Homo sapiens GN=HSPA13 PE=1 SV=1 - [HSP13_HUMAN]	16.56	1	6	6	19	1.106	0.693	0.780	0.901	43.52	16.56	11	19	471	51.9	5.76
Q68E01	Integrator complex subunit 3 OS=Homo sapiens GN=INTS3 PE=1 SV=1 - [INT3_HUMAN]	5.56	1	4	4	5	0.934	0.809	0.682	0.901	11.44	5.56	4	5	1043	118.0	5.80
P18206	Vinculin OS=Homo sapiens GN=VCL PE=1 SV=4 - [VINC_HUMAN]	71.60	1	96	96	1808	1.181	1.018	1.430	0.901	4384.73	71.60	165	1808	1134	123.7	5.66
P39023	60S ribosomal protein L3 OS=Homo sapiens GN=RPL3 PE=1 SV=2 - [RL3_HUMAN]	36.97	1	20	21	154	0.797	0.784	0.688	0.901	379.09	36.97	31	154	403	46.1	10.18
P09769	Tyrosine-protein kinase Fgr OS=Homo sapiens GN=FGR PE=1 SV=2 - [FGR_HUMAN]	4.35	6	1	2	12	0.646	0.766	1.148	0.902	21.80	4.35	3	12	529	59.4	5.59
Q9H3U1	Protein unc-45 homolog A OS=Homo sapiens GN=UNC45A PE=1 SV=1 - [UN45A_HUMAN]	38.45	1	30	30	87	1.032	1.153	1.141	0.902	241.64	38.45	44	87	944	103.0	6.07
Q53HC0	Coiled-coil domain-containing protein 92 OS=Homo sapiens GN=CCDC92 PE=1 SV=2 - [CCD92_HUMAN]	8.16	1	2	2	9	1.029	1.427	1.451	0.902	12.77	8.16	4	9	331	36.9	8.90
O60499	Syntaxin-10 OS=Homo sapiens GN=STX10 PE=1 SV=1 - [STX10_HUMAN]	10.84	1	2	2	5	0.995	1.298	1.098	0.902	6.54	10.84	4	5	249	28.1	4.89

Q9H6Q4	Cytosolic Fe-S cluster assembly factor NARFL OS=Homo sapiens GN=NARFL PE=1 SV=1 - [NARFL_HUMAN	1.68	1	1	1	1	1.299	0.867	0.973	0.902	4.06	1.68	1	1	476	53.0	7.23
Q08209	Serine/threonine-protein phosphatase 2B catalytic subunit alpha isoform OS=Homo sapiens GN=PPP3CA PE=1 SV=1 - [PP2BA_HUMAN	27.26	1	8	12	78	1.135	1.162	1.071	0.902	165.30	27.26	21	78	521	58.7	5.86
O60936	Nucleolar protein 3 OS=Homo sapiens GN=NOL3 PE=1 SV=1 - [NOL3_HUMAN]	26.94	1	4	4	7	1.146	0.890	1.382	0.902	23.27	26.94	6	7	219	24.3	11.33
Q6NSZ9	Zinc finger and SCAN domain-containing protein 25 OS=Homo sapiens GN=ZSCAN25 PE=2 SV=3 - [ZSC25_HUMAN]	2.21	1	1	1	2	0.635	0.594	1.119	0.902	6.21	2.21	1	2	544	61.4	7.72
Q8WX94	NACHT, LRR and PYD domains-containing protein 7 OS=Homo sapiens GN=NLRP7 PE=1 SV=1 - [NALP7_HUMAN]	1.73	1	1	1	1	0.924	1.071	0.953	0.902	0.00	1.73	1	1	980	111.7	6.23
Q6Q759	Sperm-associated antigen 17 OS=Homo sapiens GN=SPAG17 PE=1 SV=1 - [SPG17_HUMAN]	0.36	1	1	1	1	0.583	0.499	0.569	0.902	2.11	0.36	1	1	2223	251.6	6.24
P08559	Pyruvate dehydrogenase E1 component subunit alpha, somatic form, mitochondrial OS=Homo sapiens GN=PDHA1 PE=1 SV=3 - [ODPA_HUMAN]	50.00	2	21	21	87	0.972	1.006	0.972	0.902	181.13	50.00	36	87	390	43.3	8.06
Q14203	Dynactin subunit 1 OS=Homo sapiens GN=DCTN1 PE=1 SV=3 - [DCTN1_HUMAN]	38.97	4	43	46	187	1.094	1.043	0.974	0.902	495.74	38.97	74	187	1278	141.6	5.81

Q9NQY0	Bridging integrator 3 OS=Homo sapiens GN=BIN3 PE=1 SV=1 - [BIN3_HUMAN]	28.06	1	7	7	14	0.914	1.407	1.272	0.902	24.14	28.06	9	14	253	29.6	7.47
P40763	Signal transducer and activator of transcription 3 OS=Homo sapiens GN=STAT3 PE=1 SV=2 - [STAT3_HUMAN]	33.77	1	21	23	75	1.377	1.216	1.086	0.902	194.47	33.77	32	75	770	88.0	6.30
Q93062	RNA-binding protein with multiple splicing OS=Homo sapiens GN=RBPMS PE=1 SV=1 - [RBPMS_HUMAN]	41.84	2	7	7	32	1.176	1.729	1.274	0.902	94.98	41.84	9	32	196	21.8	8.07
Q5VUJ6	Leucine-rich repeat and calponin homology domain-containing protein 2 OS=Homo sapiens GN=LRCH2 PE=2 SV=2 - [LRCH2_HUMAN]	2.09	1	1	1	1	1.310	1.848	0.915	0.903	3.93	2.09	1	1	765	84.5	6.55
Q9Y623	Myosin-4 OS=Homo sapiens GN=MYH4 PE=1 SV=2 - [MYH4_HUMAN]	5.52	2	1	12	86	0.509	0.834	0.931	0.903	164.69	5.52	15	86	1939	222.9	5.85
Q8NB37	Parkinson disease 7 domain-containing protein 1 OS=Homo sapiens GN=PDDC1 PE=1 SV=1 - [PDDC1_HUMAN]	22.73	1	4	4	8	0.964	1.522	1.001	0.903	27.58	22.73	5	8	220	23.3	6.61
Q5TID7	Coiled-coil domain-containing protein 181 OS=Homo sapiens GN=CCDC181 PE=2 SV=1 - [CC181_HUMAN]	1.77	1	1	1	1	0.926	0.594	0.898	0.903	0.00	1.77	1	1	509	60.1	5.91
P55036	26S proteasome non-ATPase regulatory subunit 4 OS=Homo sapiens GN=PSMD4 PE=1 SV=1 - [PSMD4_HUMA	28.38	1	8	9	78	0.862	1.087	1.011	0.903	179.56	28.38	16	78	377	40.7	4.79

Q8N3F8	MICAL-like protein 1 OS=Homo sapiens GN=MICALL1 PE=1 SV=2 - [MILK1_HUMAN]	18.31	1	10	10	19	1.060	1.047	1.182	0.903	52.55	18.31	12	19	863	93.4	7.25
P35813	Protein phosphatase 1A OS=Homo sapiens GN=PPM1A PE=1 SV=1 - [PPM1A_HUMAN]	32.20	1	12	14	57	1.014	1.052	1.426	0.903	120.19	32.20	25	57	382	42.4	5.36
Q96G23	Ceramide synthase 2 OS=Homo sapiens GN=CERS2 PE=1 SV=1 - [CERS2_HUMAN]	7.11	1	3	3	9	0.989	0.862	0.626	0.903	16.52	7.11	4	9	380	44.8	8.98
Q8N1S5	Zinc transporter ZIP11 OS=Homo sapiens GN=SLC39A11 PE=2 SV=3 - [S39AB_HUMAN	7.60	1	1	2	3	1.243	0.702	0.796	0.903	11.01	7.60	2	3	342	35.4	5.60
Q9UI09	NADH dehydrogenase [ubiquinone] 1 alpha subcomplex subunit 12 OS=Homo sapiens GN=NDUFA12 PE=1 SV=1 - [NDUAC_HUMAN]	62.76	1	7	7	23	0.559	0.821	0.776	0.903	55.96	62.76	12	23	145	17.1	9.63
Q6PJG2	ELM2 and SANT domain-containing protein 1 OS=Homo sapiens GN=ELMSAN1 PE=1 SV=2 - [EMSA1_HUMAN]	3.44	1	2	2	5	1.326	1.517	1.443	0.903	2.30	3.44	2	5	1045	114.9	9.19
P78559	Microtubule-associated protein 1A OS=Homo sapiens GN=MAP1A PE=1 SV=6 - [MAP1A_HUMAN]	37.71	1	99	100	391	0.894	0.782	1.044	0.903	1039.79	37.71	143	391	2803	305.3	4.92
O95260	Arginyl-tRNA--protein transferase 1 OS=Homo sapiens GN=ATE1 PE=1 SV=2 - [ATE1_HUMAN]	16.02	1	7	7	18	1.225	0.987	0.913	0.903	38.84	16.02	9	18	518	59.1	7.93

O00238	Bone morphogenetic protein receptor type-1B OS=Homo sapiens GN=BMPR1B PE=1 SV=1 - [BMR1B_HUMAN]	3.78	1	1	1	1	0.697	1.024	0.847	0.903	0.00	3.78	1	1	502	56.9	7.66
P54886	Delta-1-pyrroline-5-carboxylate synthase OS=Homo sapiens GN=ALDH18A1 PE=1 SV=2 - [P5CS_HUMAN]	45.53	1	35	35	162	0.716	0.765	0.786	0.904	342.30	45.53	59	162	795	87.2	7.12
P00387	NADH-cytochrome b5 reductase 3 OS=Homo sapiens GN=CYB5R3 PE=1 SV=3 - [NB5R3_HUMAN]	53.82	1	15	15	102	1.144	0.960	1.119	0.904	217.27	53.82	25	102	301	34.2	7.59
Q9NWV4	UPF0587 protein C1orf123 OS=Homo sapiens GN=C1orf123 PE=1 SV=1 - [CA123_HUMAN]	58.75	1	8	8	52	0.969	0.951	0.871	0.904	119.74	58.75	14	52	160	18.0	5.01
Q9BXS4	Transmembrane protein 59 OS=Homo sapiens GN=TMEM59 PE=1 SV=1 - [TMM59_HUMAN]	6.19	1	2	2	2	0.741	0.683	0.889	0.904	1.94	6.19	2	2	323	36.2	5.10
Q9Y5K8	V-type proton ATPase subunit D OS=Homo sapiens GN=ATP6V1D PE=1 SV=1 - [VATD_HUMAN]	58.30	1	12	13	31	0.850	0.846	0.886	0.904	90.27	58.30	18	31	247	28.2	9.36
P05388	60S acidic ribosomal protein P0 OS=Homo sapiens GN=RPLP0 PE=1 SV=1 - [RLA0_HUMAN]	41.96	2	15	15	190	0.906	1.061	0.974	0.904	365.40	41.96	25	190	317	34.3	5.97
Q9HAS0	Protein Njmu-R1 OS=Homo sapiens GN=C17orf75 PE=1 SV=2 - [NJMU_HUMAN]	9.09	1	4	4	10	1.044	1.093	0.852	0.904	20.49	9.09	6	10	396	44.6	5.03
P62158	Calmodulin OS=Homo sapiens GN=CALM1 PE=1 SV=2 - [CALM_HUMAN]	83.22	3	13	13	491	1.302	1.005	1.082	0.904	1322.98	83.22	24	491	149	16.8	4.22

O43633	Charged multivesicular body protein 2a OS=Homo sapiens GN=CHMP2A PE=1 SV=1 - [CHM2A_HUMAN]	33.33	2	10	10	19	1.192	1.047	1.008	0.904	43.71	33.33	16	19	222	25.1	5.97
Q9NTK5	Obg-like ATPase 1 OS=Homo sapiens GN=OLA1 PE=1 SV=2 - [OLA1_HUMAN]	48.23	1	17	17	110	0.936	1.077	0.896	0.904	268.03	48.23	30	110	396	44.7	7.81
Q2TAA5	GDP-Man:Man(3)GlcNAc(2)-PP-Dol alpha-1,2-mannosyltransferase OS=Homo sapiens GN=ALG11 PE=1 SV=2 - [ALG11_HUMAN]	3.25	1	3	3	8	0.990	0.711	0.967	0.904	14.84	3.25	5	8	492	55.6	8.48
Q10469	Alpha-1,6-mannosyl-glycoprotein 2-beta-N-acetylglucosaminyltransferase OS=Homo sapiens GN=MGAT2 PE=1 SV=1 - [MGAT2_HUMAN]	11.63	1	4	4	9	0.931	0.892	0.726	0.904	22.04	11.63	7	9	447	51.5	8.76
P20618	Proteasome subunit beta type-1 OS=Homo sapiens GN=PSMB1 PE=1 SV=2 - [PSB1_HUMAN]	51.04	1	10	10	70	0.980	0.877	0.890	0.904	152.21	51.04	18	70	241	26.5	8.13
Q8IWR1	Tripartite motif-containing protein 59 OS=Homo sapiens GN=TRIM59 PE=2 SV=1 - [TRI59_HUMAN]	9.43	1	3	3	7	1.735	0.997	0.905	0.904	16.72	9.43	4	7	403	47.1	6.81
Q9NP97	Dynein light chain roadblock-type 1 OS=Homo sapiens GN=DYNLRB1 PE=1 SV=3 - [DLRB1_HUMAN]	29.17	2	2	2	23	0.893	0.998	0.999	0.905	42.37	29.17	4	23	96	10.9	7.25
P63165	Small ubiquitin-related modifier 1 OS=Homo sapiens GN=SUMO1 PE=1 SV=1 - [SUMO1_HUMA	40.59	1	5	5	22	1.403	1.190	0.936	0.905	38.89	40.59	8	22	101	11.5	5.52

Q8WWH5	Probable tRNA pseudouridine synthase 1 OS=Homo sapiens GN=TRUB1 PE=1 SV=1 - [TRUB1_HUMAN]	8.02	1	3	3	4	0.908	1.146	0.893	0.905	5.01	8.02	4	4	349	37.2	8.25
Q9NWZ5	Uridine-cytidine kinase-like 1 OS=Homo sapiens GN=UCKL1 PE=1 SV=2 - [UCKL1_HUMAN]	7.30	1	3	3	3	1.148	0.869	0.928	0.905	8.60	7.30	3	3	548	61.1	7.36
P30041	Peroxiredoxin-6 OS=Homo sapiens GN=PRDX6 PE=1 SV=3 - [PRDX6_HUMAN]	63.39	1	22	22	373	1.159	0.677	1.129	0.905	809.29	63.39	36	373	224	25.0	6.38
P60953	Cell division control protein 42 homolog OS=Homo sapiens GN=CDC42 PE=1 SV=2 - [CDC42_HUMAN]	68.59	2	8	9	88	0.947	1.055	0.995	0.905	277.54	68.59	14	88	191	21.2	6.55
Q96EV8	Dysbindin OS=Homo sapiens GN=DTNBP1 PE=1 SV=1 - [DTBP1_HUMAN]	21.94	1	7	7	17	0.755	1.058	0.960	0.905	36.87	21.94	13	17	351	39.5	4.67
Q9H4G4	Golgi-associated plant pathogenesis-related protein 1 OS=Homo sapiens GN=GLIPR2 PE=1 SV=3 - [GAPR1_HUMAN	55.84	1	7	7	29	1.130	1.016	0.792	0.905	86.13	55.84	11	29	154	17.2	9.41
A0AVT1	Ubiquitin-like modifier-activating enzyme 6 OS=Homo sapiens GN=UBA6 PE=1 SV=1 - [UBA6_HUMAN]	26.52	2	24	25	84	1.017	1.187	1.111	0.905	197.36	26.52	41	84	1052	117.9	6.14
Q9NWK9	Box C/D snoRNA protein 1 OS=Homo sapiens GN=ZNHIT6 PE=1 SV=1 - [BCD1_HUMAN]	4.26	1	1	1	1	1.647	0.856	1.170	0.905	0.00	4.26	1	1	470	53.9	5.80
Q96AG4	Leucine-rich repeat-containing protein 59 OS=Homo sapiens GN=LRRC59 PE=1 SV=1 - [LRC59_HUMAN]	37.79	1	15	15	92	0.734	0.975	0.991	0.905	172.65	37.79	23	92	307	34.9	9.57

Q32MZ4	Leucine-rich repeat flightless-interacting protein 1 OS=Homo sapiens GN=LRRFIP1 PE=1 SV=2 - [LRRF1_HUMAN	24.38	1	15	17	99	0.665	0.975	0.887	0.905	302.69	24.38	25	99	808	89.2	4.65
O60858	E3 ubiquitin-protein ligase TRIM13 OS=Homo sapiens GN=TRIM13 PE=1 SV=2 - [TRI13_HUMAN]	1.72	1	1	1	3	0.659	0.753	0.817	0.905	5.41	1.72	2	3	407	47.0	6.01
P49406	39S ribosomal protein L19, mitochondrial OS=Homo sapiens GN=MRPL19 PE=1 SV=2 - [RM19_HUMAN]	20.21	1	6	6	18	0.900	0.884	0.839	0.905	43.13	20.21	10	18	292	33.5	9.50
Q32MK0	Myosin light chain kinase 3 OS=Homo sapiens GN=MYLK3 PE=2 SV=3 - [MYLK3_HUMAN]	6.35	3	2	3	5	1.124	0.753	0.759	0.906	6.59	6.35	4	5	819	88.3	6.11
Q9UJW0	Dynactin subunit 4 OS=Homo sapiens GN=DCTN4 PE=1 SV=1 - [DCTN4_HUMAN]	29.35	1	10	10	39	1.203	1.087	1.041	0.906	95.35	29.35	16	39	460	52.3	7.34
P35716	Transcription factor SOX-11 OS=Homo sapiens GN=SOX11 PE=1 SV=2 - [SOX11_HUMA N]	4.08	1	1	1	1	1.843	1.394	1.379	0.906	2.62	4.08	1	1	441	46.7	5.05
Q8IX04	Ubiquitin-conjugating enzyme E2 variant 3 OS=Homo sapiens GN=UEVLD PE=1 SV=2 - [UEVLD_HUMAN]	14.44	1	5	5	6	0.836	1.048	1.304	0.906	8.74	14.44	5	6	471	52.2	7.09
Q9NXD2	Myotubularin-related protein 10 OS=Homo sapiens GN=MTMR10 PE=1 SV=3 - [MTMRA_HUMA N]	4.76	1	4	4	8	1.012	1.018	1.143	0.906	19.34	4.76	5	8	777	88.2	8.53
O43768	Alpha-endosulfine OS=Homo sapiens GN=ENSA PE=1 SV=1 - [ENSA_HUMAN]	60.33	1	7	8	33	1.224	1.506	1.009	0.906	82.53	60.33	12	33	121	13.4	7.24

Q13574	Diacylglycerol kinase zeta OS=Homo sapiens GN=DGKZ PE=1 SV=3 - [DGKZ_HUMAN]	6.36	1	4	5	8	0.657	1.316	1.383	0.906	15.76	6.36	5	8	1117	124.1	9.04
P61019	Ras-related protein Rab-2A OS=Homo sapiens GN=RAB2A PE=1 SV=1 - [RAB2A_HUMAN]	53.77	1	5	10	59	0.825	0.976	0.801	0.906	127.96	53.77	18	59	212	23.5	6.54
O15212	Prefoldin subunit 6 OS=Homo sapiens GN=PFDN6 PE=1 SV=1 - [PFD6_HUMAN]	34.88	1	8	8	32	0.781	1.141	0.992	0.906	75.88	34.88	14	32	129	14.6	8.88
P07686	Beta-hexosaminidase subunit beta OS=Homo sapiens GN=HEXB PE=1 SV=3 - [HEXB_HUMAN]	37.59	1	20	22	176	0.814	1.250	1.030	0.906	532.43	37.59	36	176	556	63.1	6.76
Q63HN1	Protein FAM205B OS=Homo sapiens GN=FAM205B PE=2 SV=1 - [F205B_HUMAN]	2.88	1	1	1	1	1.278	0.998	1.365	0.906	0.00	2.88	1	1	556	61.8	7.50
P12235	ADP/ATP translocase 1 OS=Homo sapiens GN=SLC25A4 PE=1 SV=4 - [ADT1_HUMAN]	43.29	2	4	15	87	1.200	1.037	0.897	0.906	227.04	43.29	22	87	298	33.0	9.76
P41223	Protein BUD31 homolog OS=Homo sapiens GN=BUD31 PE=1 SV=2 - [BUD31_HUMAN]	13.89	1	1	1	2	1.408	0.977	1.406	0.907	5.09	13.89	1	2	144	17.0	8.82
Q9Y4C1	Lysine-specific demethylase 3A OS=Homo sapiens GN=KDM3A PE=1 SV=4 - [KDM3A_HUMAN]	0.45	1	1	1	2	1.138	0.808	0.777	0.907	3.45	0.45	1	2	1321	147.2	8.07
Q8N8Z6	Discoidin, CUB and LCCL domain-containing protein 1 OS=Homo sapiens GN=DCBLD1 PE=1 SV=2 - [DCBD1_HUMAN]	5.87	1	3	3	3	0.969	1.239	0.837	0.907	7.63	5.87	3	3	715	77.9	8.05

Q8N5I4	Dehydrogenase /reductase SDR family member on chromosome X OS=Homo sapiens GN=DHRSX PE=2 SV=2 - [DHRSX_HUMAN]	11.82	1	3	3	6	0.783	0.575	0.716	0.907	8.83	11.82	5	6	330	36.4	9.00
P48448	Aldehyde dehydrogenase family 3 member B2 OS=Homo sapiens GN=ALDH3B2 PE=2 SV=3 - [AL3B2_HUMAN]	3.12	1	1	1	13	1.194	0.078	0.786	0.907	17.91	3.12	2	13	385	42.6	5.99
O95630	STAM-binding protein OS=Homo sapiens GN=STAMBP PE=1 SV=1 - [STABP_HUMAN]	20.75	1	7	7	15	1.049	0.927	0.929	0.908	38.69	20.75	10	15	424	48.0	6.29
P62834	Ras-related protein Rap-1A OS=Homo sapiens GN=RAP1A PE=1 SV=1 - [RAP1A_HUMAN]	51.09	1	1	9	152	0.945	1.247	1.065	0.908	392.74	51.09	14	152	184	21.0	6.67
Q13442	28 kDa heat- and acid-stable phosphoprotein OS=Homo sapiens GN=PDAP1 PE=1 SV=1 - [HAP28_HUMAN]	29.28	1	9	9	60	1.001	1.393	1.045	0.908	140.36	29.28	15	60	181	20.6	8.87
O00255	Menin OS=Homo sapiens GN=MEN1 PE=1 SV=4 - [MEN1_HUMAN]	2.28	1	1	1	2	0.860	1.479	1.044	0.908	3.35	2.28	2	2	615	68.0	6.55
Q9Y2I6	Ninein-like protein OS=Homo sapiens GN=NINL PE=1 SV=2 - [NINL_HUMAN]	1.01	1	1	2	4	1.000	0.941	0.618	0.908	7.89	1.01	2	4	1382	156.2	5.06
Q8IWV8	E3 ubiquitin-protein ligase UBR2 OS=Homo sapiens GN=UBR2 PE=1 SV=1 - [UBR2_HUMAN]	4.84	1	8	8	25	0.862	1.045	0.780	0.908	56.50	4.84	11	25	1755	200.4	6.24
Q3ZCM7	Tubulin beta-8 chain OS=Homo sapiens GN=TUBB8 PE=1 SV=2 - [TBB8_HUMAN]	28.15	1	1	11	306	0.847	6.587	2.104	0.908	514.20	28.15	18	306	444	49.7	4.89

Q9Y4K0	Lysyl oxidase homolog 2 OS=Homo sapiens GN=LOXL2 PE=1 SV=1 - [LOXL2_HUMAN]	11.63	1	9	9	27	0.627	0.647	1.327	0.908	66.03	11.63	15	27	774	86.7	6.38
Q13164	Mitogen-activated protein kinase 7 OS=Homo sapiens GN=MAPK7 PE=1 SV=2 - [MK07_HUMAN]	8.70	2	5	6	16	0.953	1.057	1.072	0.908	28.29	8.70	10	16	816	88.3	5.88
Q86TU7	Histone-lysine N-methyltransferase setd3 OS=Homo sapiens GN=SETD3 PE=1 SV=1 - [SETD3_HUMAN]	18.01	1	9	9	22	0.751	0.873	1.092	0.908	47.89	18.01	13	22	594	67.2	5.96
P0DJ07	Protein PET100 homolog, mitochondrial OS=Homo sapiens GN=PET100 PE=1 SV=1 - [PT100_HUMAN	13.70	1	1	1	1	1.192	1.156	0.932	0.908	3.71	13.70	1	1	73	9.1	6.77
Q96PU8	Protein quaking OS=Homo sapiens GN=QKI PE=1 SV=1 - [QKI_HUMAN]	25.51	1	8	8	19	1.145	1.099	0.787	0.908	41.36	25.51	13	19	341	37.6	8.56
O43264	Centromere/kinetochore protein zw10 homolog OS=Homo sapiens GN=ZW10 PE=1 SV=3 - [ZW10_HUMAN	11.04	2	7	8	53	0.810	0.838	0.777	0.908	115.51	11.04	11	53	779	88.8	6.27
P55786	Puromycin-sensitive aminopeptidase OS=Homo sapiens GN=NPEPPS PE=1 SV=2 - [PSA_HUMAN]	39.28	2	38	38	186	1.048	1.014	0.996	0.908	394.70	39.28	63	186	919	103.2	5.72
P62841	40S ribosomal protein S15 OS=Homo sapiens GN=RPS15 PE=1 SV=2 - [RS15_HUMAN]	42.07	1	5	5	48	0.916	1.023	1.042	0.908	115.52	42.07	7	48	145	17.0	10.39
Q92930	Ras-related protein Rab-8B OS=Homo sapiens GN=RAB8B PE=1 SV=2 - [RAB8B_HUMAN]	57.49	2	5	13	80	1.240	1.036	0.657	0.908	170.27	57.49	20	80	207	23.6	9.07

Q9NYU2	UDP-glucose:glycoprotein glucosyltransferase 1 OS=Homo sapiens GN=UGGT1 PE=1 SV=3 - [UGGG1_HUMAN]	36.85	1	46	48	225	0.924	1.024	0.974	0.908	562.93	36.85	76	225	1555	177.1	5.63
Q9NXE4	Sphingomyelin phosphodiesterase 4 OS=Homo sapiens GN=SMPD4 PE=1 SV=2 - [NSMA3_HUMAN]	13.06	1	9	9	15	1.044	1.062	0.791	0.909	22.58	13.06	14	15	827	93.3	7.97
Q14934	Nuclear factor of activated T-cells, cytoplasmic 4 OS=Homo sapiens GN=NFATC4 PE=1 SV=2 - [NFAC4_HUMAN	2.55	1	2	2	4	1.073	1.101	1.048	0.909	6.83	2.55	2	4	902	95.4	5.38
P10620	Microsomal glutathione S-transferase 1 OS=Homo sapiens GN=MGST1 PE=1 SV=1 - [MGST1_HUMAN]	12.26	1	4	4	56	1.116	0.649	0.748	0.909	126.85	12.26	7	56	155	17.6	9.39
P63027	Vesicle-associated membrane protein 2 OS=Homo sapiens GN=VAMP2 PE=1 SV=3 - [VAMP2_HUMAN]	43.10	2	2	6	74	1.109	0.936	0.951	0.909	203.99	43.10	11	74	116	12.7	8.13
P62241	40S ribosomal protein S8 OS=Homo sapiens GN=RPS8 PE=1 SV=2 - [RS8_HUMAN]	39.42	1	11	11	122	0.809	0.987	0.971	0.909	281.84	39.42	18	122	208	24.2	10.32
Q16531	DNA damage-binding protein 1 OS=Homo sapiens GN=DDB1 PE=1 SV=1 - [DDB1_HUMAN]	31.32	1	30	30	88	0.891	0.920	0.911	0.909	203.53	31.32	47	88	1140	126.9	5.26
Q9BXT8	RING finger protein 17 OS=Homo sapiens GN=RNF17 PE=1 SV=3 - [RNF17_HUMAN]	1.97	1	2	3	5	0.934	1.025	1.409	0.909	6.93	1.97	3	5	1623	184.5	5.40
Q9UI14	Prenylated Rab acceptor protein 1 OS=Homo sapiens GN=RABAC1 PE=1 SV=1 - [PRAF1_HUMAN	14.59	1	3	3	17	0.535	0.643	0.745	0.909	64.04	14.59	3	17	185	20.6	7.34

Q9UK99	F-box only protein 3 OS=Homo sapiens GN=FBXO3 PE=1 SV=3 - [FBX3_HUMAN]	4.03	1	1	2	5	1.011	1.269	1.318	0.909	10.42	4.03	3	5	471	54.5	5.00
Q8TBE9	N-acylneuraminate-9-phosphatase OS=Homo sapiens GN=NANP PE=1 SV=1 - [NANP_HUMAN]	7.26	1	1	1	1	1.185	0.798	1.022	0.909	2.14	7.26	1	1	248	27.8	6.40
Q86XZ4	Spermatogenesis-associated serine-rich protein 2 OS=Homo sapiens GN=SPATS2 PE=1 SV=1 - [SPAS2_HUMAN]	10.64	1	5	5	17	0.696	1.011	1.215	0.909	32.71	10.64	7	17	545	59.5	8.90
Q96JH7	Deubiquitinating protein VCIP135 OS=Homo sapiens GN=VCPIP1 PE=1 SV=2 - [VCIP1_HUMAN]	18.17	1	15	17	38	0.893	1.174	1.079	0.909	62.10	18.17	23	38	1222	134.2	7.20
Q9H8X2	Inositol-pentakisphosphate 2-kinase OS=Homo sapiens GN=IPPK PE=1 SV=1 - [IPPK_HUMAN]	2.24	1	1	1	1	0.803	0.661	0.811	0.909	2.18	2.24	1	1	491	56.0	8.37
Q14571	Inositol 1,4,5-trisphosphate receptor type 2 OS=Homo sapiens GN=ITPR2 PE=1 SV=2 - [ITPR2_HUMAN]	2.96	1	2	10	24	1.047	1.481	1.430	0.909	42.86	2.96	15	24	2701	307.9	6.43
Q96J94	Piwi-like protein 1 OS=Homo sapiens GN=PIWIL1 PE=1 SV=1 - [PIWL1_HUMAN	0.70	1	1	1	1	0.954	1.723	1.077	0.909	2.08	0.70	1	1	861	98.5	9.44
O60231	Putative pre-mRNA-splicing factor ATP-dependent RNA helicase DHX16 OS=Homo sapiens GN=DHX16 PE=1 SV=2 - [DHX16_HUMAN	9.03	3	8	9	27	0.991	1.170	0.854	0.910	64.60	9.03	13	27	1041	119.2	6.80
Q9BQ48	39S ribosomal protein L34, mitochondrial OS=Homo sapiens GN=MRPL34 PE=1 SV=1 - [RM34_HUMAN]	11.96	1	2	2	6	1.081	0.949	1.132	0.910	10.94	11.96	4	6	92	10.2	12.25

Q8TAE8	Growth arrest and DNA damage-inducible proteins-interacting protein 1 OS=Homo sapiens GN=GADD45GIP1 PE=1 SV=1 - [G45IP_HUMAN]	14.86	1	3	3	4	0.759	0.966	0.951	0.910	11.66	14.86	4	4	222	25.4	10.02
Q4J6C6	Prolyl endopeptidase-like OS=Homo sapiens GN=PREPL PE=1 SV=1 - [PPCEL_HUMAN]	12.24	1	7	7	20	0.704	0.854	1.555	0.910	49.48	12.24	10	20	727	83.9	6.38
Q86SR1	Polypeptide N-acetylgalactosaminyltransferase 10 OS=Homo sapiens GN=GALNT10 PE=1 SV=2 - [GLT10_HUMAN	12.60	1	5	5	9	0.649	0.847	1.097	0.910	21.99	12.60	6	9	603	68.9	8.59
P48637	Glutathione synthetase OS=Homo sapiens GN=GSS PE=1 SV=1 - [GSHB_HUMAN]	37.13	1	17	17	69	1.065	0.813	0.979	0.910	161.74	37.13	28	69	474	52.4	5.92
P52943	Cysteine-rich protein 2 OS=Homo sapiens GN=CRIP2 PE=1 SV=1 - [CRIP2_HUMAN]	44.71	2	7	8	30	2.315	4.826	3.695	0.910	79.48	44.71	11	30	208	22.5	8.72
Q5SWX8	Protein odr-4 homolog OS=Homo sapiens GN=ODR4 PE=2 SV=1 - [ODR4_HUMAN]	18.72	1	7	8	20	0.935	0.727	0.652	0.910	37.46	18.72	13	20	454	51.1	5.92
Q05066	Sex-determining region Y protein OS=Homo sapiens GN=SRY PE=1 SV=1 - [SRY_HUMAN]	3.43	1	1	1	2	1.424	0.522	0.999	0.910	2.31	3.43	2	2	204	23.9	9.48
Q14657	EKC/KEOPS complex subunit LAGE3 OS=Homo sapiens GN=LAGE3 PE=1 SV=2 - [LAGE3_HUMAN]	16.08	1	1	1	5	1.229	0.999	0.838	0.910	23.87	16.08	2	5	143	14.8	8.63

Q9BR77	Coiled-coil domain-containing protein 77 OS=Homo sapiens GN=CCDC77 PE=2 SV=1 - [CCD77_HUMAN]	3.07	1	1	2	5	0.685	0.732	0.742	0.911	12.66	3.07	2	5	488	57.5	8.72
P61916	Epididymal secretory protein E1 OS=Homo sapiens GN=NPC2 PE=1 SV=1 - [NPC2_HUMAN]	33.11	1	8	8	25	0.549	1.139	0.933	0.911	42.80	33.11	13	25	151	16.6	7.65
Q9Y3I0	tRNA-splicing ligase RtcB homolog OS=Homo sapiens GN=RTCB PE=1 SV=1 - [RTCB_HUMAN]	51.49	1	24	24	154	0.868	1.007	1.018	0.911	404.37	51.49	41	154	505	55.2	7.23
P62995	Transformer-2 protein homolog beta OS=Homo sapiens GN=TRA2B PE=1 SV=1 - [TRA2B_HUMAN]	11.46	1	2	4	12	1.038	1.015	0.764	0.911	34.16	11.46	6	12	288	33.6	11.25
Q4G0F5	Vacuolar protein sorting-associated protein 26B OS=Homo sapiens GN=VPS26B PE=1 SV=2 - [VP26B_HUMAN]	27.98	1	7	8	25	0.954	0.660	0.653	0.911	59.53	27.98	12	25	336	39.1	7.36
P48444	Coatomer subunit delta OS=Homo sapiens GN=ARCN1 PE=1 SV=1 - [COPD_HUMAN]	40.70	1	25	25	138	0.955	0.802	0.816	0.911	309.53	40.70	44	138	511	57.2	6.21
Q9H832	Ubiquitin-conjugating enzyme E2 Z OS=Homo sapiens GN=UBE2Z PE=1 SV=2 - [UBE2Z_HUMAN]	24.86	1	8	8	31	0.886	0.925	1.137	0.911	68.19	24.86	14	31	354	38.2	5.62
Q9UH92	Max-like protein X OS=Homo sapiens GN=MLX PE=1 SV=2 - [MLX_HUMAN]	2.01	1	1	1	1	0.704	1.216	1.205	0.911	0.00	2.01	1	1	298	33.3	8.06
Q3KQZ1	Solute carrier family 25 member 35 OS=Homo sapiens GN=SLC25A35 PE=2 SV=1 - [S2535_HUMAN]	3.67	1	1	1	3	0.680	0.320	0.280	0.911	2.77	3.67	1	3	300	32.4	9.11

Q13219	Pappalysin-1 OS=Homo sapiens GN=PAPPA PE=1 SV=3 - [PAPP1_HUMAN]	0.43	1	1	1	1	1.428	0.841	0.789	0.911	2.02	0.43	1	1	1627	180.9	6.18
Q96K17	Transcription factor BTF3 homolog 4 OS=Homo sapiens GN=BTF3L4 PE=1 SV=1 - [BT3L4_HUMAN]	20.25	1	5	5	13	0.791	0.954	0.879	0.911	30.57	20.25	7	13	158	17.3	6.35
P78417	Glutathione S-transferase omega-1 OS=Homo sapiens GN=GSTO1 PE=1 SV=2 - [GSTO1_HUMAN]	44.81	1	17	17	176	0.882	1.015	1.072	0.911	384.38	44.81	28	176	241	27.5	6.60
Q9H008	Phospholysine phosphohistidine inorganic pyrophosphate phosphatase OS=Homo sapiens GN=LHPP PE=1 SV=2 - [LHPP_HUMAN]	31.11	1	5	5	19	1.140	1.312	1.073	0.911	59.66	31.11	8	19	270	29.1	6.15
O95487	Protein transport protein Sec24B OS=Homo sapiens GN=SEC24B PE=1 SV=2 - [SC24B_HUMAN]	11.04	1	11	12	37	1.053	0.884	0.838	0.911	111.91	11.04	20	37	1268	137.3	6.67
O95486	Protein transport protein Sec24A OS=Homo sapiens GN=SEC24A PE=1 SV=2 - [SC24A_HUMAN]	15.55	1	13	14	49	0.891	0.760	0.827	0.912	116.49	15.55	23	49	1093	119.7	7.66
Q9UBP0	Spastin OS=Homo sapiens GN=SPAST PE=1 SV=1 - [SPAST_HUMAN]	6.33	1	4	5	6	1.054	1.054	1.104	0.912	11.42	6.33	6	6	616	67.2	9.64
P63104	14-3-3 protein zeta/delta OS=Homo sapiens GN=YWHAZ PE=1 SV=1 - [1433Z_HUMAN]	74.29	1	19	24	374	1.115	1.139	1.162	0.912	1148.53	74.29	38	374	245	27.7	4.79
Q9UH65	Switch-associated protein 70 OS=Homo sapiens GN=SWAP70 PE=1 SV=1 - [SWP70_HUMAN]	46.67	1	25	25	112	1.061	0.736	0.840	0.912	303.76	46.67	38	112	585	69.0	5.87

P26640	Valine--tRNA ligase OS=Homo sapiens GN=VARS PE=1 SV=4 - [SYVC_HUMAN]	33.31	1	37	37	205	0.874	0.823	0.994	0.912	555.11	33.31	65	205	1264	140.4	7.59
Q9BVK6	Transmembrane emp24 domain-containing protein 9 OS=Homo sapiens GN=TMED9 PE=1 SV=2 - [TMED9_HUMA	33.62	1	11	12	45	0.830	0.894	0.795	0.912	108.49	33.62	17	45	235	27.3	8.02
Q969E4	Transcription elongation factor A protein-like 3 OS=Homo sapiens GN=TCEAL3 PE=1 SV=1 - [TCAL3_HUMAN]	16.50	3	4	4	9	0.773	1.361	1.113	0.912	17.16	16.50	7	9	200	22.5	4.92
Q8IZP0	Abl interactor 1 OS=Homo sapiens GN=ABI1 PE=1 SV=4 - [ABI1_HUMAN]	24.02	1	7	10	42	1.124	1.275	1.464	0.912	90.02	24.02	18	42	508	55.0	7.06
Q9Y5N5	HemK methyltransferase family member 2 OS=Homo sapiens GN=N6AMT1 PE=1 SV=3 - [HEMK2_HUMA	14.49	1	3	3	17	0.794	0.763	0.961	0.912	38.42	14.49	5	17	214	22.9	5.21
Q9UET6	Putative tRNA (cytidine(32)/guanosine(34)-2'-O)-methyltransferase OS=Homo sapiens GN=FTSJ1 PE=1 SV=2 - [TRM7_HUMAN]	6.08	1	2	2	3	0.659	1.086	1.350	0.912	10.52	6.08	2	3	329	36.1	5.69
Q96GG9	DCN1-like protein 1 OS=Homo sapiens GN=DCUN1D1 PE=1 SV=1 - [DCNL1_HUMAN]	27.41	2	7	7	28	1.021	1.028	1.104	0.912	40.35	27.41	10	28	259	30.1	5.34
Q3SYG4	Protein PTHB1 OS=Homo sapiens GN=BBS9 PE=1 SV=1 - [PTHB1_HUMAN]	3.72	1	2	3	7	0.998		0.847	0.912	21.58	3.72	3	7	887	99.2	5.55
Q5T4W7	Artemin OS=Homo sapiens GN=ARTN PE=1 SV=1 - [ARTN_HUMAN]	6.36	1	1	1	1	0.619	0.732	0.863	0.912	1.61	6.36	1	1	220	22.9	11.71

Q14790	Caspase-8 OS=Homo sapiens GN=CASP8 PE=1 SV=1 - [CASP8_HUMAN]	5.64	1	1	3	4	0.598	1.372	1.169	0.912	4.11	5.64	3	4	479	55.4	5.10
Q9UP83	Conserved oligomeric Golgi complex subunit 5 OS=Homo sapiens GN=COG5 PE=1 SV=3 - [COG5_HUMAN]	8.82	1	7	7	15	0.769	1.056	1.068	0.912	24.22	8.82	11	15	839	92.7	6.60
Q99832	T-complex protein 1 subunit eta OS=Homo sapiens GN=CCT7 PE=1 SV=2 - [TCPH_HUMAN]	60.96	1	33	33	185	0.888	0.955	0.963	0.912	492.05	60.96	52	185	543	59.3	7.65
P49916	DNA ligase 3 OS=Homo sapiens GN=LIG3 PE=1 SV=2 - [DNLI3_HUMAN]	12.09	1	11	11	34	0.919	1.340	1.229	0.912	61.42	12.09	19	34	1009	112.8	9.01
P84090	Enhancer of rudimentary homolog OS=Homo sapiens GN=ERH PE=1 SV=1 - [ERH_HUMAN]	22.12	1	4	4	34	0.985	1.016	0.743	0.912	80.11	22.12	7	34	104	12.3	5.92
P32754	4-hydroxyphenylpyruvate dioxygenase OS=Homo sapiens GN=HPD PE=1 SV=2 - [HPPD_HUMAN]	3.82	1	1	1	1	1.918	0.486	0.737	0.912	2.39	3.82	1	1	393	44.9	7.01
A6ND36	Protein FAM83G OS=Homo sapiens GN=FAM83G PE=1 SV=2 - [FA83G_HUMAN]	2.19	1	1	2	6	0.692	0.899	1.510	0.913	10.98	2.19	3	6	823	90.8	6.39
P53365	Arfaptin-2 OS=Homo sapiens GN=ARFIP2 PE=1 SV=1 - [ARFP2_HUMAN]	15.84	1	4	5	13	1.118	0.941	1.136	0.913	36.93	15.84	8	13	341	37.8	6.04
Q96BJ3	Axin interactor, dorsalization-associated protein OS=Homo sapiens GN=AIDA PE=1 SV=1 - [AIDA_HUMAN]	34.97	1	9	9	33	1.267	1.120	1.029	0.913	85.78	34.97	16	33	306	35.0	6.55

Q92597	Protein NDRG1 OS=Homo sapiens GN=NDRG1 PE=1 SV=1 - [NDRG1_HUMAN]	32.74	1	8	8	77	2.858	0.781	0.789	0.913	206.82	32.74	14	77	394	42.8	5.82
Q13454	Tumor suppressor candidate 3 OS=Homo sapiens GN=TUSC3 PE=1 SV=1 - [TUSC3_HUMAN]	10.34	1	3	4	7	0.583	1.110	0.918	0.913	15.00	10.34	7	7	348	39.7	9.91
P51812	Ribosomal protein S6 kinase alpha-3 OS=Homo sapiens GN=RPS6KA3 PE=1 SV=1 - [KS6A3_HUMAN]	27.84	4	18	18	57	1.010	0.924	0.902	0.913	126.02	27.84	31	57	740	83.7	6.89
Q9NX36	DnaJ homolog subfamily C member 28 OS=Homo sapiens GN=DNAJC28 PE=1 SV=2 - [DJC28_HUMAN]	1.80	1	1	1	10	0.827	1.100	0.948	0.913	23.72	1.80	1	10	388	45.8	9.55
Q8WXF7	Atlastin-1 OS=Homo sapiens GN=ATL1 PE=1 SV=1 - [ATLA1_HUMAN]	24.55	1	11	12	33	1.126	1.029	1.194	0.913	57.91	24.55	19	33	558	63.5	6.18
O43572	A-kinase anchor protein 10, mitochondrial OS=Homo sapiens GN=AKAP10 PE=1 SV=2 - [AKA10_HUMAN]	7.85	1	3	3	4	1.148	1.076	0.823	0.913	11.27	7.85	3	4	662	73.8	6.39
O94885	SAM and SH3 domain-containing protein 1 OS=Homo sapiens GN=SASH1 PE=1 SV=3 - [SASH1_HUMAN]	7.86	2	8	8	13	0.669	0.864	1.006	0.913	32.48	7.86	10	13	1247	136.6	6.09
Q9BTD8	RNA-binding protein 42 OS=Homo sapiens GN=RBM42 PE=1 SV=1 - [RBM42_HUMAN]	9.17	1	4	4	8	1.079	0.933	0.852	0.914	12.96	9.17	6	8	480	50.4	9.63
Q15257	Serine/threonine-protein phosphatase 2A activator OS=Homo sapiens GN=PPP2R4 PE=1 SV=3 - [PTPA_HUMAN]	25.14	1	8	8	56	0.955	0.987	0.966	0.914	114.40	25.14	12	56	358	40.6	5.94

Q9Y2L9	Leucine-rich repeat and calponin homology domain-containing protein 1 OS=Homo sapiens GN=LRCH1 PE=1 SV=3 - [LRCH1_HUMAN]	4.53	1	2	3	7	1.271	0.786	0.757	0.914	15.81	4.53	5	7	728	80.8	5.94
Q8IVF6	Ankyrin repeat domain-containing protein 18A OS=Homo sapiens GN=ANKRD18A PE=2 SV=3 - [AN18A_HUMAN]	3.33	2	3	4	7	0.777	0.678	0.626	0.914	7.34	3.33	5	7	992	115.5	7.83
O14978	Zinc finger protein 263 OS=Homo sapiens GN=ZNF263 PE=2 SV=2 - [ZN263_HUMAN]	2.05	1	1	1	1	1.147	1.306	1.059	0.914	3.12	2.05	1	1	683	77.2	6.99
Q5BJF6	Outer dense fiber protein 2 OS=Homo sapiens GN=ODF2 PE=1 SV=1 - [ODFP2_HUMAN]	2.90	1	2	3	3	2.268	1.224	1.496	0.914	4.03	2.90	3	3	829	95.3	7.62
Q14156	Protein EFR3 homolog A OS=Homo sapiens GN=EFR3A PE=1 SV=2 - [EFR3A_HUMAN]	6.58	1	4	4	11	0.988	1.023	0.897	0.914	22.96	6.58	5	11	821	92.9	6.70
Q86VR2	Protein FAM134C OS=Homo sapiens GN=FAM134C PE=1 SV=1 - [F134C_HUMAN]	11.59	1	4	4	15	0.638	1.040	1.068	0.914	42.40	11.59	7	15	466	51.4	4.97
Q9GZP4	PITH domain-containing protein 1 OS=Homo sapiens GN=PITHD1 PE=1 SV=1 - [PITH1_HUMAN]	13.27	1	3	3	8	0.816	0.779	1.031	0.914	17.70	13.27	5	8	211	24.2	5.74
Q8IV38	Ankyrin repeat and MYND domain-containing protein 2 OS=Homo sapiens GN=ANKMY2 PE=1 SV=1 - [ANKY2_HUMAN]	1.59	1	1	1	4	0.893	1.569	0.990	0.914	9.06	1.59	2	4	441	49.3	6.25

P28749	Retinoblastoma-like protein 1 OS=Homo sapiens GN=RBL1 PE=1 SV=3 - [RBL1_HUMAN]	3.00	1	3	3	9	0.737	1.047	0.722	0.914	19.13	3.00	3	9	1068	120.8	7.50
O00231	26S proteasome non-ATPase regulatory subunit 11 OS=Homo sapiens GN=PSMD11 PE=1 SV=3 - [PSD11_HUMAN	66.59	1	25	25	116	0.931	0.918	0.960	0.914	273.24	66.59	42	116	422	47.4	6.48
Q3YEC7	Rab-like protein 6 OS=Homo sapiens GN=RABL6 PE=1 SV=2 - [RABL6_HUMAN	8.23	1	6	6	14	0.746	0.779	0.759	0.914	27.07	8.23	10	14	729	79.5	5.22
O75063	Glycosaminoglycan xylosylkinase OS=Homo sapiens GN=FAM20B PE=1 SV=1 - [XYLK_HUMAN]	8.07	1	2	2	5	0.701	1.029	1.167	0.914	10.49	8.07	4	5	409	46.4	6.87
Q13158	FAS-associated death domain protein OS=Homo sapiens GN=FADD PE=1 SV=1 - [FADD_HUMAN]	28.85	1	6	6	35	0.816	0.836	1.117	0.915	92.95	28.85	12	35	208	23.3	5.69
Q8WZA9	Immunity-related GTPase family Q protein OS=Homo sapiens GN=IRGQ PE=1 SV=1 - [IRGQ_HUMAN]	19.10	1	11	11	25	1.190	0.854	1.171	0.915	55.74	19.10	16	25	623	62.7	4.88
Q06323	Proteasome activator complex subunit 1 OS=Homo sapiens GN=PSME1 PE=1 SV=1 - [PSME1_HUMAN]	53.01	1	16	16	196	1.474	0.939	0.893	0.915	419.45	53.01	24	196	249	28.7	6.02
O00244	Copper transport protein ATOX1 OS=Homo sapiens GN=ATOX1 PE=1 SV=1 - [ATOX1_HUMAN]	38.24	1	3	3	19	0.818	0.830	0.777	0.915	32.73	38.24	6	19	68	7.4	7.24
Q9H246	Uncharacterized protein C1orf21 OS=Homo sapiens GN=C1orf21 PE=1 SV=1 - [CA021_HUMAN]	8.26	1	1	1	4	1.103	1.331	1.214	0.915	13.26	8.26	2	4	121	13.9	5.22

Q92973	Transportin-1 OS=Homo sapiens GN=TNPO1 PE=1 SV=2 - [TNPO1_HUMAN]	18.49	1	10	14	72	0.922	0.773	0.962	0.915	187.41	18.49	22	72	898	102.3	4.98
Q9UBQ0	Vacuolar protein sorting-associated protein 29 OS=Homo sapiens GN=VPS29 PE=1 SV=1 - [VPS29_HUMAN]	24.18	1	5	5	22	1.067	0.981	0.894	0.915	65.01	24.18	7	22	182	20.5	6.79
Q9NQ66	1-phosphatidylinositol 4,5-bisphosphate phosphodiesterase beta-1 OS=Homo sapiens GN=PLCB1 PE=1 SV=1 - [PLCB1_HUMAN]	8.80	2	10	11	33	1.118	1.486	1.303	0.915	49.52	8.80	17	33	1216	138.5	6.23
P37840	Alpha-synuclein OS=Homo sapiens GN=SNCA PE=1 SV=1 - [SYUA_HUMAN]	22.14	1	2	3	10	0.864	0.566	0.900	0.915	20.03	22.14	5	10	140	14.5	4.70
Q9BV40	Vesicle-associated membrane protein 8 OS=Homo sapiens GN=VAMP8 PE=1 SV=1 - [VAMP8_HUMAN]	9.00	1	1	1	3	0.394	1.060	0.316	0.915	7.89	9.00	2	3	100	11.4	7.34
Q9H2D6	TRIO and F-actin-binding protein OS=Homo sapiens GN=TRIOBP PE=1 SV=3 - [TARA_HUMAN]	16.28	1	37	41	193	0.910	1.104	1.311	0.915	443.88	16.28	72	193	2365	261.2	8.48
Q9H8M2	Bromodomain-containing protein 9 OS=Homo sapiens GN=BRD9 PE=1 SV=2 - [BRD9_HUMAN]	9.55	1	4	4	6	1.163	1.358	2.468	0.915	5.81	9.55	4	6	597	67.0	6.19
Q14141	Septin-6 OS=Homo sapiens GN=SEPT6 PE=1 SV=4 - [SEPT6_HUMAN]	30.88	1	2	14	66	1.325	2.204	2.689	0.915	140.12	30.88	21	66	434	49.7	6.67

Q8N2S1	Latent-transforming growth factor beta-binding protein 4 OS=Homo sapiens GN=LTBP4 PE=1 SV=2 - [LTBP4_HUMAN]	1.35	1	2	2	4	1.902	1.856	1.234	0.915	12.64	1.35	2	4	1624	173.3	5.43
Q14457	Bedin-1 OS=Homo sapiens GN=BECN1 PE=1 SV=2 - [BECN1_HUMAN]	8.22	1	4	4	10	0.789	0.898	0.990	0.915	19.12	8.22	5	10	450	51.9	4.89
P00736	Complement C1r subcomponent OS=Homo sapiens GN=C1R PE=1 SV=2 - [C1R_HUMAN]	1.84	1	2	2	3	1.077	1.503	0.969	0.915	5.78	1.84	3	3	705	80.1	6.21
Q13325	Interferon-induced protein with tetratricopeptide repeats 5 OS=Homo sapiens GN=IFIT5 PE=1 SV=1 - [IFIT5_HUMAN]	11.41	2	4	6	15	1.288	0.740	1.021	0.915	41.05	11.41	10	15	482	55.8	7.40
P52179	Myomesin-1 OS=Homo sapiens GN=MYOM1 PE=1 SV=2 - [MYOM1_HUMAN]	2.61	1	3	4	4	0.530	0.538	0.720	0.916	6.93	2.61	4	4	1685	187.5	6.93
Q8NHP6	Motile sperm domain-containing protein 2 OS=Homo sapiens GN=MOSPD2 PE=1 SV=1 - [MSPD2_HUMAN]	13.32	1	9	9	19	1.091	0.427	0.785	0.916	33.04	13.32	14	19	518	59.7	5.96
P23526	Adenosylhomocysteinase OS=Homo sapiens GN=AHCY PE=1 SV=4 - [SAHH_HUMAN]	46.99	1	19	21	166	0.913	0.857	0.923	0.916	421.34	46.99	36	166	432	47.7	6.34
Q6ZWJ8	Kielin/chordin-like protein OS=Homo sapiens GN=KCP PE=2 SV=2 - [KCP_HUMAN]	0.93	1	1	1	1	0.832	1.016	0.914	0.916	0.00	0.93	1	1	1503	159.8	5.74
Q8IXQ6	Poly [ADP-ribose] polymerase 9 OS=Homo sapiens GN=PARP9 PE=1 SV=2 - [PARP9_HUMAN]	8.20	1	7	7	18	1.919	1.128	0.984	0.916	28.07	8.20	10	18	854	96.3	7.91

Q8WYA1	Aryl hydrocarbon receptor nuclear translocator-like protein 2 OS=Homo sapiens GN=ARNTL2 PE=1 SV=2 - [BMAL2_HUMAN	8.33	1	3	3	4	1.075	1.423	1.075	0.916	5.59	8.33	3	4	636	70.8	7.37
P52746	Zinc finger protein 142 OS=Homo sapiens GN=ZNF142 PE=2 SV=4 - [ZN142_HUMAN]	0.36	1	1	1	2	0.523	1.043	0.926	0.916	1.83	0.36	1	2	1687	187.8	7.91
Q96B23	Uncharacterized protein C18orf25 OS=Homo sapiens GN=C18orf25 PE=1 SV=2 - [CR025_HUMAN]	5.46	1	1	1	2	0.866	1.068	0.883	0.916	11.82	5.46	1	2	403	43.3	4.88
Q6ZMI0	Protein phosphatase 1 regulatory subunit 21 OS=Homo sapiens GN=PPP1R21 PE=1 SV=1 - [PPR21_HUMAN]	25.13	2	19	19	66	0.955	1.026	1.252	0.916	158.67	25.13	30	66	780	88.3	6.84
O00142	Thymidine kinase 2, mitochondrial OS=Homo sapiens GN=TK2 PE=1 SV=4 - [KITM_HUMAN]	3.40	1	1	1	2	0.899	0.917	0.995	0.916	2.46	3.40	2	2	265	31.0	8.46
Q8TBY9	WD repeat-containing protein 66 OS=Homo sapiens GN=WDR66 PE=2 SV=2 - [WDR66_HUMA N]	0.61	1	1	1	2	0.684	0.506	0.877	0.916	3.97	0.61	1	2	1149	129.9	5.08
Q01814	Plasma membrane calcium-transporting ATPase 2 OS=Homo sapiens GN=ATP2B2 PE=1 SV=2 - [AT2B2_HUMAN]	13.11	1	1	17	75	2.750	0.583	0.402	0.917	172.62	13.11	27	75	1243	136.8	5.91
Q9NWZ3	Interleukin-1 receptor-associated kinase 4 OS=Homo sapiens GN=IRAK4 PE=1 SV=1 - [IRAK4_HUMAN	14.13	1	6	6	15	0.860	0.685	1.139	0.917	36.71	14.13	9	15	460	51.5	5.41

Q8N4Q1	Mitochondrial intermembrane space import and assembly protein 40 OS=Homo sapiens GN=CHCHD4 PE=1 SV=1 - [MIA40_HUMAN]	11.97	1	2	2	4	1.029	0.902	0.791	0.917	7.25	11.97	3	4	142	16.0	4.31
Q8IXB1	DnaJ homolog subfamily C member 10 OS=Homo sapiens GN=DNAJC10 PE=1 SV=2 - [DJC10_HUMAN]	11.10	1	9	9	42	0.831	1.089	1.098	0.917	70.82	11.10	16	42	793	91.0	7.18
O96011	Peroxisomal membrane protein 11B OS=Homo sapiens GN=PEX11B PE=1 SV=1 - [PX11B_HUMAN]	12.74	1	3	3	5	1.006	0.495	0.763	0.917	17.16	12.74	4	5	259	28.4	9.85
P61204	ADP-ribosylation factor 3 OS=Homo sapiens GN=ARF3 PE=1 SV=2 - [ARF3_HUMAN]	55.25	4	5	11	217	1.113	0.835	0.905	0.917	435.01	55.25	19	217	181	20.6	7.43
Q6PIU2	Neutral cholesterol ester hydrolase 1 OS=Homo sapiens GN=NCEH1 PE=1 SV=3 - [NCEH1_HUMAN]	28.92	1	11	11	42	1.540	1.356	1.100	0.917	82.41	28.92	19	42	408	45.8	7.23
Q96MG7	Melanoma-associated antigen G1 OS=Homo sapiens GN=NDNL2 PE=1 SV=1 - [MAGG1_HUMA N]	6.58	1	2	2	2	0.627	0.790	1.303	0.917	3.25	6.58	2	2	304	34.3	9.28
P48729	Casein kinase I isoform alpha OS=Homo sapiens GN=CSNK1A1 PE=1 SV=2 - [KC1A_HUMAN]	28.78	2	8	8	21	0.842	0.857	0.843	0.917	56.67	28.78	11	21	337	38.9	9.57
Q9BVI4	Nucleolar complex protein 4 homolog OS=Homo sapiens GN=NOC4L PE=1 SV=1 - [NOC4L_HUMAN	7.95	1	3	3	4	0.832	0.502	0.942	0.917	15.07	7.95	3	4	516	58.4	7.49
Q15046	Lysine--tRNA ligase OS=Homo sapiens GN=KARS PE=1 SV=3 - [SYK_HUMAN]	41.37	1	27	27	164	0.907	0.890	0.804	0.917	409.34	41.37	48	164	597	68.0	6.35

Q9H3P7	Golgi resident protein GCP60 OS=Homo sapiens GN=ACBD3 PE=1 SV=4 - [GCP60_HUMAN]	29.73	1	11	11	44	0.885	0.915	0.990	0.917	92.77	29.73	16	44	528	60.6	5.06
P56545	C-terminal-binding protein 2 OS=Homo sapiens GN=CTBP2 PE=1 SV=1 - [CTBP2_HUMAN]	18.65	1	4	7	21	0.952	1.080	0.984	0.918	58.85	18.65	12	21	445	48.9	6.95
Q9UDY4	DnaJ homolog subfamily B member 4 OS=Homo sapiens GN=DNAJB4 PE=1 SV=1 - [DNJB4_HUMAN]	35.91	1	8	9	30	0.442	1.196	1.527	0.918	71.52	35.91	13	30	337	37.8	8.50
Q9HD34	LYR motif-containing protein 4 OS=Homo sapiens GN=LYRM4 PE=1 SV=1 - [LYRM4_HUMAN]	37.36	1	3	3	5	1.038	0.981	1.030	0.918	6.86	37.36	5	5	91	10.8	10.73
Q96P44	Collagen alpha-1(XXI) chain OS=Homo sapiens GN=COL21A1 PE=2 SV=1 - [COLA1_HUMAN]	5.12	1	2	3	4	0.634	0.839	1.244	0.918	6.16	5.12	3	4	957	99.3	8.32
P25686	DnaJ homolog subfamily B member 2 OS=Homo sapiens GN=DNAJB2 PE=1 SV=3 - [DNJB2_HUMAN]	8.95	2	2	3	12	0.929	1.164	0.757	0.918	30.60	8.95	4	12	324	35.6	5.95
Q96GX1	Tectonic-2 OS=Homo sapiens GN=TCTN2 PE=2 SV=1 - [TECT2_HUMAN]	3.59	1	2	2	2	0.961	0.919	0.730	0.918	2.04	3.59	2	2	697	76.8	5.24
Q8WWC4	Uncharacterized protein C2orf47, mitochondrial OS=Homo sapiens GN=C2orf47 PE=1 SV=1 - [CB047_HUMAN]	5.84	1	2	2	4	0.863	0.770	0.950	0.918	7.97	5.84	3	4	291	32.5	9.17
O60861	Growth arrest-specific protein 7 OS=Homo sapiens GN=GAS7 PE=1 SV=3 - [GAS7_HUMAN]	1.47	1	1	1	2	1.750	0.634	1.050	0.918	2.02	1.47	2	2	476	54.3	7.34

Q9NVH1	DnaJ homolog subfamily C member 11 OS=Homo sapiens GN=DNAJC11 PE=1 SV=2 - [DJC11_HUMAN]	12.88	1	6	6	12	0.922	0.944	0.939	0.918	27.81	12.88	9	12	559	63.2	8.40
Q9BZV1	UBX domain-containing protein 6 OS=Homo sapiens GN=UBXN6 PE=1 SV=1 - [UBXN6_HUMAN]	16.55	1	5	5	16	1.108	1.249	1.268	0.918	40.23	16.55	8	16	441	49.7	6.89
Q13627	Dual specificity tyrosine-phosphorylation-regulated kinase 1A OS=Homo sapiens GN=DYRK1A PE=1 SV=2 - [DYR1A_HUMAN]	2.10	1	1	1	4	1.130	1.390	1.023	0.919	11.04	2.10	2	4	763	85.5	8.75
Q13561	Dynactin subunit 2 OS=Homo sapiens GN=DCTN2 PE=1 SV=4 - [DCTN2_HUMAN]	54.86	2	21	22	154	1.094	1.093	1.099	0.919	394.61	54.86	35	154	401	44.2	5.21
Q93034	Cullin-5 OS=Homo sapiens GN=CUL5 PE=1 SV=4 - [CUL5_HUMAN]	23.85	1	19	19	59	1.172	0.800	0.875	0.919	135.08	23.85	28	59	780	90.9	7.94
Q96MW1	Coiled-coil domain-containing protein 43 OS=Homo sapiens GN=CCDC43 PE=1 SV=2 - [CCD43_HUMAN]	16.07	1	3	3	12	0.952	0.819	0.945	0.919	26.57	16.07	5	12	224	25.2	4.92
Q8NBK3	Sulfatase-modifying factor 1 OS=Homo sapiens GN=SUMF1 PE=1 SV=3 - [SUMF1_HUMAN]	7.22	1	2	2	7	1.120	0.984	1.248	0.919	19.73	7.22	4	7	374	40.5	6.65
Q8TB22	Spermatogenesis-associated protein 20 OS=Homo sapiens GN=SPATA20 PE=2 SV=3 - [SPT20_HUMAN]	17.68	1	12	12	30	1.285	1.198	1.204	0.919	69.79	17.68	19	30	786	87.8	7.43
P41214	Eukaryotic translation initiation factor 2D OS=Homo sapiens GN=EIF2D PE=1 SV=3 - [EIF2D_HUMAN]	11.47	1	5	6	20	1.112	1.151	1.179	0.919	28.67	11.47	8	20	584	64.7	7.65

Q9NPY3	Complement component C1q receptor OS=Homo sapiens GN=CD93 PE=1 SV=3 - [C1QR1_HUMAN]	4.75	1	3	3	4	0.991	0.953	1.137	0.919	6.40	4.75	3	4	652	68.5	5.44
O75348	V-type proton ATPase subunit G 1 OS=Homo sapiens GN=ATP6V1G1 PE=1 SV=3 - [VATG1_HUMAN]	32.20	1	4	4	35	1.086	1.074	0.807	0.919	80.73	32.20	7	35	118	13.7	8.79
Q96II8	Leucine-rich repeat and calponin homology domain-containing protein 3 OS=Homo sapiens GN=LRCH3 PE=1 SV=2 - [LRCH3_HUMAN]	10.04	1	5	6	17	0.978	1.191	0.878	0.919	32.32	10.04	11	17	777	86.0	6.71
P54803	Galactocerebrosidase OS=Homo sapiens GN=GALC PE=1 SV=2 - [GALC_HUMAN]	11.53	1	5	5	10	1.472	0.962	0.889	0.919	16.95	11.53	9	10	685	77.0	6.64
Q5VZ18	SH2 domain-containing adapter protein E OS=Homo sapiens GN=SHE PE=1 SV=1 - [SHE_HUMAN]	1.82	1	1	1	1	0.777	0.435	0.502	0.919	2.43	1.82	1	1	495	53.9	9.16
P61011	Signal recognition particle 54 kDa protein OS=Homo sapiens GN=SRP54 PE=1 SV=1 - [SRP54_HUMAN]	39.88	1	20	20	80	0.811	0.892	0.865	0.919	192.98	39.88	30	80	504	55.7	8.75
O15530	3-phosphoinositide-dependent protein kinase 1 OS=Homo sapiens GN=PDPK1 PE=1 SV=1 - [PDPK1_HUMAN]	17.63	2	7	7	9	0.837	0.815	0.981	0.920	19.34	17.63	9	9	556	63.1	7.36
Q9NPJ3	Acyl-coenzyme A thioesterase 13 OS=Homo sapiens GN=ACOT13 PE=1 SV=1 - [ACO13_HUMAN]	26.43	1	3	3	6	0.966	0.563	0.428	0.920	17.28	26.43	4	6	140	15.0	9.14

Q8NGI4	Olfactory receptor 4D11 OS=Homo sapiens GN=OR4D11 PE=2 SV=1 - [OR4DB_HUMAN]	3.22	1	1	1	2	1.379	0.696	0.953	0.920	5.33	3.22	1	2	311	34.9	8.75
Q99653	Calcineurin B homologous protein 1 OS=Homo sapiens GN=CHP1 PE=1 SV=3 - [CHP1_HUMAN]	77.44	1	13	13	32	0.985	1.027	1.071	0.920	82.35	77.44	19	32	195	22.4	5.10
P13533	Myosin-6 OS=Homo sapiens GN=MYH6 PE=1 SV=5 - [MYH6_HUMAN]	6.24	1	2	12	81	0.623	0.810	0.775	0.920	151.23	6.24	15	81	1939	223.6	5.73
Q66PJ3	ADP-ribosylation factor-like protein 6-interacting protein 4 OS=Homo sapiens GN=ARL6IP4 PE=1 SV=2 - [AR6P4_HUMAN]	4.51	1	1	1	1	0.947	1.272	1.695	0.920	4.59	4.51	1	1	421	44.9	10.93
O00469	Procollagen-lysine,2-oxoglutarate 5-dioxygenase 2 OS=Homo sapiens GN=PLOD2 PE=1 SV=2 - [PLOD2_HUMAN]	34.60	1	26	26	150	0.814	0.705	0.866	0.920	346.87	34.60	45	150	737	84.6	6.71
Q8NBN3	Transmembrane protein 87A OS=Homo sapiens GN=TMEM87A PE=1 SV=3 - [TM87A_HUMAN]	12.25	1	8	8	17	0.875	0.793	0.861	0.920	44.89	12.25	10	17	555	63.4	6.74
Q8N6G6	ADAMTS-like protein 1 OS=Homo sapiens GN=ADAMTSL1 PE=1 SV=4 - [ATL1_HUMAN]	0.91	1	1	1	1	2.998	0.815	1.068	0.920	0.00	0.91	1	1	1762	193.3	7.80
P42677	40S ribosomal protein S27 OS=Homo sapiens GN=RPS27 PE=1 SV=3 - [RS27_HUMAN]	40.48	1	2	5	39	1.265	0.865	0.883	0.920	103.49	40.48	10	39	84	9.5	9.45
Q9BYG8	Gasdermin-C OS=Homo sapiens GN=GSDMC PE=2 SV=3 - [GSDMC_HUMAN]	3.94	1	2	2	3	0.838	0.621	0.797	0.920	2.00	3.94	2	3	508	57.7	5.78

Q9HAV7	GrpE protein homolog 1, mitochondrial OS=Homo sapiens GN=GRPEL1 PE=1 SV=2 - [GRPE1_HUMAN]	41.47	1	9	10	52	0.957	0.902	1.002	0.920	115.95	41.47	15	52	217	24.3	8.12
Q00537	Cyclin-dependent kinase 17 OS=Homo sapiens GN=CDK17 PE=1 SV=2 - [CDK17_HUMAN]	6.50	6	2	4	17	1.061	1.143	1.104	0.920	28.91	6.50	5	17	523	59.5	9.03
O96007	Molybdopterin synthase catalytic subunit OS=Homo sapiens GN=MOCS2 PE=1 SV=1 - [MOC2B_HUMA	21.28	1	5	5	11	1.075	1.108	1.156	0.920	14.79	21.28	9	11	188	20.9	5.44
Q9GZM5	Protein YIPF3 OS=Homo sapiens GN=YIPF3 PE=1 SV=1 - [YIPF3_HUMAN]	18.29	1	5	5	14	0.803	0.693	0.811	0.920	36.37	18.29	8	14	350	38.2	5.76
Q16537	Serine/threonine-protein phosphatase 2A 56 kDa regulatory subunit epsilon isoform OS=Homo sapiens GN=PPP2R5E PE=1 SV=1 - [2A5E_HUMAN]	30.19	1	8	11	35	1.129	0.970	0.862	0.920	93.53	30.19	16	35	467	54.7	6.95
P43686	26S protease regulatory subunit 6B OS=Homo sapiens GN=PSMC4 PE=1 SV=2 - [PRS6B_HUMAN]	59.33	1	20	21	131	0.856	1.029	0.982	0.921	342.92	59.33	36	131	418	47.3	5.21
Q15629	Translocating chain-associated membrane protein 1 OS=Homo sapiens GN=TRAM1 PE=1 SV=3 - [TRAM1_HUMA	13.64	1	5	5	7	1.109	0.664	0.613	0.921	17.00	13.64	6	7	374	43.0	9.63
P49903	Selenide, water dikinase 1 OS=Homo sapiens GN=SEPHS1 PE=1 SV=2 - [SPS1_HUMAN]	16.33	2	5	5	7	1.097	1.079	1.087	0.921	24.74	16.33	6	7	392	42.9	5.97

Q96K21	Abscission/NoCut checkpoint regulator OS=Homo sapiens GN=ZFYVE19 PE=1 SV=3 - [ANCHR_HUMAN]	8.92	1	3	5	20	1.104	1.019	0.789	0.921	37.29	8.92	6	20	471	51.5	5.73
Q8IZU2	WD repeat-containing protein 17 OS=Homo sapiens GN=WDR17 PE=2 SV=2 - [WDR17_HUMAN]	1.29	1	2	2	2	1.168	0.749	0.480	0.921	4.21	1.29	2	2	1322	147.6	6.46
Q7Z624	Calmodulin-lysine N-methyltransferase OS=Homo sapiens GN=CAMKMT PE=1 SV=2 - [CMKMT_HUMAN]	5.88	1	1	2	3	1.152	0.796	2.101	0.921	4.42	5.88	3	3	323	36.1	6.83
Q69YQ0	Cytospin-A OS=Homo sapiens GN=SPECC1L PE=1 SV=2 - [CYTSA_HUMAN]	31.78	5	30	32	127	1.000	1.206	1.158	0.921	328.05	31.78	53	127	1117	124.5	5.72
Q6ZS94	Putative uncharacterized protein C1orf229 OS=Homo sapiens GN=C1orf229 PE=2 SV=1 - [CA229_HUMAN]	8.44	1	1	1	1	0.744	1.173		0.921	2.35	8.44	1	1	237	24.3	11.59
Q9UI95	Mitotic spindle assembly checkpoint protein MAD2B OS=Homo sapiens GN=MAD2L2 PE=1 SV=2 - [MD2L2_HUMAN]	3.79	1	1	1	1	0.585	0.683	0.699	0.921	0.00	3.79	1	1	211	24.3	6.52
P20020	Plasma membrane calcium-transporting ATPase 1 OS=Homo sapiens GN=ATP2B1 PE=1 SV=3 - [AT2B1_HUMAN]	29.17	1	13	36	154	0.833	0.737	0.875	0.921	365.61	29.17	60	154	1258	138.7	6.04
P05496	ATP synthase F(0) complex subunit C1, mitochondrial OS=Homo sapiens GN=ATP5G1 PE=2 SV=2 - [AT5G1_HUMAN]	5.15	3	1	1	5	0.803	0.843	1.047	0.921	8.53	5.15	1	5	136	14.3	9.74

Q9GZN1	Actin-related protein 6 OS=Homo sapiens GN=ACTR6 PE=1 SV=1 - [ARP6_HUMAN]	3.79	1	1	1	1	1.102	0.646	1.177	0.922	2.34	3.79	1	1	396	45.8	5.03
Q53H12	Acylglycerol kinase, mitochondrial OS=Homo sapiens GN=AGK PE=1 SV=2 - [AGK_HUMAN]	14.69	1	5	5	9	0.916	0.790	0.791	0.922	21.16	14.69	5	9	422	47.1	8.09
Q96EC8	Protein YIPF6 OS=Homo sapiens GN=YIPF6 PE=1 SV=2 - [YIPF6_HUMAN]	5.08	1	1	1	7	1.081	0.827	0.973	0.922	12.03	5.08	2	7	236	26.2	5.64
Q96C74	Ropporin-1-like protein OS=Homo sapiens GN=ROPN1L PE=1 SV=2 - [ROP1L_HUMAN]	3.91	1	1	1	2	1.117	0.910	0.692	0.922	4.38	3.91	1	2	230	26.1	7.72
P47224	Guanine nucleotide exchange factor MSS4 OS=Homo sapiens GN=RABIF PE=1 SV=2 - [MSS4_HUMAN]	12.20	1	2	2	2	0.923	0.943	1.185	0.922	4.25	12.20	2	2	123	13.8	5.52
P06748	Nucleophosmin OS=Homo sapiens GN=NPM1 PE=1 SV=2 - [NPM_HUMAN]	45.24	1	17	18	379	0.730	0.886	0.776	0.922	932.24	45.24	25	379	294	32.6	4.78
Q13003	Glutamate receptor ionotropic, kainate 3 OS=Homo sapiens GN=GRIK3 PE=2 SV=3 - [GRIK3_HUMAN]	1.63	1	1	1	1	0.818	1.111	1.336	0.922	2.64	1.63	1	1	919	104.0	7.52
P12544	Granzyme A OS=Homo sapiens GN=GZMA PE=1 SV=2 - [GRAA_HUMAN]	12.21	1	2	2	3	1.163	0.761	0.750	0.922	2.73	12.21	2	3	262	29.0	8.91
Q96PZ7	CUB and sushi domain-containing protein 1 OS=Homo sapiens GN=CSMD1 PE=1 SV=2 - [CSMD1_HUMAN]	0.39	1	1	1	1	0.971	0.748	1.245	0.922	2.93	0.39	1	1	3565	388.6	6.07

Q66K14	TBC1 domain family member 9B OS=Homo sapiens GN=TBC1D9B PE=1 SV=3 - [TBC9B_HUMAN]	13.68	1	14	14	39	1.120	1.163	1.175	0.922	112.94	13.68	21	39	1250	140.4	5.25
Q9H2K8	Serine/threonine-protein kinase TAO3 OS=Homo sapiens GN=TAOK3 PE=1 SV=2 - [TAOK3_HUMAN]	15.48	1	10	13	35	0.843	1.018	0.908	0.923	80.19	15.48	17	35	898	105.3	7.30
Q07352	Zinc finger protein 36, C3H1 type-like 1 OS=Homo sapiens GN=ZFP36L1 PE=1 SV=1 - [TISB_HUMAN]	12.72	1	5	5	14	1.132	0.896	1.340	0.923	29.95	12.72	9	14	338	36.3	7.96
Q86W56	Poly(ADP-ribose) glycohydrolase OS=Homo sapiens GN=PARG PE=1 SV=1 - [PARG_HUMAN]	1.54	1	2	2	2	0.842	0.954	1.134	0.923	2.88	1.54	2	2	976	111.0	6.43
Q15836	Vesicle-associated membrane protein 3 OS=Homo sapiens GN=VAMP3 PE=1 SV=3 - [VAMP3_HUMAN]	50.00	2	2	6	125	0.775	0.932	0.930	0.923	352.69	50.00	11	125	100	11.3	8.79
O43708	Maleylacetoacetate isomerase OS=Homo sapiens GN=GSTZ1 PE=1 SV=3 - [MAAI_HUMAN]	25.46	1	6	7	11	1.129	1.060	0.667	0.923	17.78	25.46	9	11	216	24.2	8.54
Q68CZ2	Tensin-3 OS=Homo sapiens GN=TNS3 PE=1 SV=2 - [TENS3_HUMAN]	20.00	1	18	21	77	1.554	0.795	0.980	0.923	196.35	20.00	31	77	1445	155.2	6.81
Q9NX55	Huntingtin-interacting protein K OS=Homo sapiens GN=HYPK PE=1 SV=2 - [HYPK_HUMAN]	42.64	1	4	4	22	0.987	0.891	0.793	0.923	74.78	42.64	7	22	129	14.7	4.93
Q9H6U6	Breast carcinoma-amplified sequence 3 OS=Homo sapiens GN=BCAS3 PE=1 SV=3 - [BCAS3_HUMAN	5.60	1	4	4	5	0.847	1.207	1.013	0.923	7.53	5.60	4	5	928	101.2	6.70

Q9BSD7	Cancer-related nucleoside-triphosphatase OS=Homo sapiens GN=NTPCR PE=1 SV=1 - [NTPCR_HUMAN]	36.32	1	6	7	46	1.052	1.041	0.879	0.924	115.18	36.32	11	46	190	20.7	9.54
P53701	Cytochrome c-type heme lyase OS=Homo sapiens GN=HCCS PE=1 SV=1 - [CCHL_HUMAN]	21.27	1	7	7	19	0.806	0.779	0.883	0.924	38.68	21.27	13	19	268	30.6	6.68
P27708	CAD protein OS=Homo sapiens GN=CAD PE=1 SV=3 - [PYR1_HUMAN]	19.64	1	33	36	109	0.860	0.927	0.750	0.924	286.42	19.64	55	109	2225	242.8	6.46
Q08AG7	Mitotic-spindle organizing protein 1 OS=Homo sapiens GN=MZT1 PE=1 SV=2 - [MZT1_HUMAN]	21.95	1	2	2	2	1.267	0.866	0.877	0.924	5.25	21.95	2	2	82	8.5	4.78
O75976	Carboxypeptidase D OS=Homo sapiens GN=CPD PE=1 SV=2 - [CBPD_HUMAN]	20.22	2	26	26	73	0.714	1.083	1.146	0.924	185.96	20.22	37	73	1380	152.8	6.05
Q9UK45	U6 snRNA-associated Sm-like protein LSm7 OS=Homo sapiens GN=LSM7 PE=1 SV=1 - [LSM7_HUMAN]	40.78	1	3	4	10	0.942	0.976	1.060	0.924	29.75	40.78	5	10	103	11.6	5.27
P61201	COP9 signalosome complex subunit 2 OS=Homo sapiens GN=COPS2 PE=1 SV=1 - [CSN2_HUMAN]	32.73	1	13	13	50	0.948	0.957	0.945	0.924	132.77	32.73	21	50	443	51.6	5.53
Q96F24	Nuclear receptor-binding factor 2 OS=Homo sapiens GN=NRBF2 PE=1 SV=1 - [NRBF2_HUMAN	26.13	1	5	6	14	0.710	0.861	1.218	0.924	36.15	26.13	9	14	287	32.4	5.87
Q9UBC2	Epidermal growth factor receptor substrate 15-like 1 OS=Homo sapiens GN=EPS15L1 PE=1 SV=1 - [EP15R_HUMAN	26.62	1	18	18	63	1.108	1.025	0.854	0.924	158.46	26.62	27	63	864	94.2	5.11

Q9H4A5	Golgi phosphoprotein 3-like OS=Homo sapiens GN=GOLPH3L PE=1 SV=1 - [GLP3L_HUMAN]	31.93	1	8	9	21	1.065	0.998	1.290	0.924	58.75	31.93	12	21	285	32.7	5.83
Q2M329	Coiled-coil domain-containing protein 96 OS=Homo sapiens GN=CCDC96 PE=2 SV=2 - [CCD96_HUMAN]	1.26	1	1	1	2	0.775	0.544	0.611	0.924	4.23	1.26	1	2	555	62.7	4.94
Q6ZRK6	Coiled-coil domain-containing protein 73 OS=Homo sapiens GN=CCDC73 PE=2 SV=2 - [CCD73_HUMAN]	2.87	1	3	3	5	0.527	0.630	0.598	0.924	12.38	2.87	3	5	1079	124.1	5.58
Q99973	Telomerase protein component 1 OS=Homo sapiens GN=TEP1 PE=1 SV=2 - [TEP1_HUMAN]	3.65	1	8	8	13	0.890	1.092	0.832	0.924	27.71	3.65	10	13	2627	290.3	7.97
Q9NP56	cAMP-specific 3',5'-cyclic phosphodiesterase 7B OS=Homo sapiens GN=PDE7B PE=1 SV=1 - [PDE7B_HUMAN]	6.00	1	1	2	3	0.616	0.754	0.760	0.925	6.68	6.00	2	3	450	51.8	7.03
O00116	Alkyldihydroxyacetonephosphate synthase, peroxisomal OS=Homo sapiens GN=AGPS PE=1 SV=1 - [ADAS_HUMAN]	23.71	1	13	13	61	0.965	0.860	1.108	0.925	174.38	23.71	22	61	658	72.9	7.34
P26038	Moesin OS=Homo sapiens GN=MSN PE=1 SV=3 - [MOES_HUMAN]	71.06	1	50	64	941	1.002	1.135	1.216	0.925	2381.77	71.06	104	941	577	67.8	6.40
Q13432	Protein unc-119 homolog A OS=Homo sapiens GN=UNC119 PE=1 SV=1 - [U119A_HUMAN]	8.33	1	2	2	2	1.026	0.742	1.112	0.925	2.25	8.33	2	2	240	26.9	6.42

Q9BY50	Signal peptidase complex catalytic subunit SEC11C OS=Homo sapiens GN=SEC11C PE=1 SV=3 -	7.81	1	2	2	9	0.991	0.853	1.144	0.925	22.50	7.81	3	9	192	21.5	9.20
Q9Y3U8	60S ribosomal protein L36 OS=Homo sapiens GN=RPL36 PE=1 SV=3 - [RL36_HUMAN]	45.71	1	7	8	88	0.700	0.912	0.947	0.925	154.58	45.71	14	88	105	12.2	11.59
P00973	2'-5'-oligoadenylate synthase 1 OS=Homo sapiens GN=OAS1 PE=1 SV=4 - [OAS1_HUMAN]	5.00	1	2	2	3	2.822	0.705	0.986	0.925	6.32	5.00	3	3	400	46.0	8.22
Q9BRX2	Protein pelota homolog OS=Homo sapiens GN=PELO PE=1 SV=2 - [PELO_HUMAN]	31.95	2	10	11	31	0.868	0.918	1.218	0.925	63.26	31.95	17	31	385	43.3	6.34
P49682	C-X-C chemokine receptor type 3 OS=Homo sapiens GN=CXCR3 PE=1 SV=2 - [CXCR3_HUMAN]	3.26	1	1	1	1	0.754	0.468	0.449	0.925	1.99	3.26	1	1	368	40.6	7.74
Q86U42	Polyadenylate-binding protein 2 OS=Homo sapiens GN=PABPN1 PE=1 SV=3 - [PABP2_HUMAN]	16.99	2	8	8	27	0.865	1.088	0.724	0.926	50.42	16.99	13	27	306	32.7	5.06
Q9BUK6	Protein misato homolog 1 OS=Homo sapiens GN=MSTO1 PE=1 SV=1 - [MSTO1_HUMAN]	8.42	1	3	3	9	0.934	1.229	1.135	0.926	18.61	8.42	5	9	570	61.8	6.11
O94967	WD repeat-containing protein 47 OS=Homo sapiens GN=WDR47 PE=1 SV=1 - [WDR47_HUMAN]	5.55	1	5	5	16	1.378	0.769	1.245	0.926	41.43	5.55	8	16	919	101.9	5.94
Q8IZ13	Protein ZBED8 OS=Homo sapiens GN=ZBED8 PE=2 SV=1 - [ZBED8_HUMAN]	3.70	1	1	1	1	1.311	0.819	0.926	0.926	0.00	3.70	1	1	594	68.3	6.42

P36405	ADP-ribosylation factor-like protein 3 OS=Homo sapiens GN=ARL3 PE=1 SV=2 - [ARL3_HUMAN]	58.24	1	9	9	25	0.971	1.137	1.083	0.926	69.49	58.24	13	25	182	20.4	7.24
P78509	Reelin OS=Homo sapiens GN=RELN PE=1 SV=3 - [RELN_HUMAN]	0.35	1	1	1	1	1.351	1.056	0.986	0.926	0.00	0.35	1	1	3460	388.1	5.88
P21266	Glutathione S-transferase Mu 3 OS=Homo sapiens GN=GSTM3 PE=1 SV=3 - [GSTM3_HUMAN]	81.78	1	23	24	356	1.444	0.547	0.713	0.926	950.83	81.78	44	356	225	26.5	5.54
Q92499	ATP-dependent RNA helicase DDX1 OS=Homo sapiens GN=DDX1 PE=1 SV=2 - [DDX1_HUMAN]	47.30	1	33	33	113	0.947	1.040	1.139	0.926	256.91	47.30	50	113	740	82.4	7.23
P62244	40S ribosomal protein S15a OS=Homo sapiens GN=RPS15A PE=1 SV=2 - [RS15A_HUMAN]	59.23	1	8	8	83	0.883	1.029	0.922	0.926	148.68	59.23	15	83	130	14.8	10.13
P30043	Flavin reductase (NADPH) OS=Homo sapiens GN=BLVRB PE=1 SV=3 - [BLVRB_HUMAN	59.71	1	8	8	61	1.232	1.018	1.161	0.926	186.84	59.71	15	61	206	22.1	7.65
Q9NZN4	EH domain-containing protein 2 OS=Homo sapiens GN=EHD2 PE=1 SV=2 - [EHD2_HUMAN]	68.14	1	30	36	397	2.035	1.079	1.337	0.926	1084.61	68.14	59	397	543	61.1	6.46
Q8IYI6	Exocyst complex component 8 OS=Homo sapiens GN=EXOC8 PE=1 SV=2 - [EXOC8_HUMAN]	13.52	1	11	11	21	1.000	1.107	0.915	0.926	36.67	13.52	17	21	725	81.7	5.49
Q16760	Diacylglycerol kinase delta OS=Homo sapiens GN=DGKD PE=1 SV=4 - [DGKD_HUMAN]	2.22	2	2	3	3	1.386	1.616	1.200	0.927	2.38	2.22	3	3	1214	134.4	7.56

P42025	Beta-centractin OS=Homo sapiens GN=ACTR1B PE=1 SV=1 - [ACTY_HUMAN]	26.06	2	3	8	59	1.239	0.970	0.952	0.927	131.64	26.06	14	59	376	42.3	6.40
Q9H7S9	Zinc finger protein 703 OS=Homo sapiens GN=ZNF703 PE=1 SV=1 - [ZN703_HUMAN]	4.24	2	2	2	8	1.511	1.207	1.013	0.927	20.74	4.24	4	8	590	58.2	8.79
P51648	Fatty aldehyde dehydrogenase OS=Homo sapiens GN=ALDH3A2 PE=1 SV=1 - [AL3A2_HUMAN]	15.88	1	7	7	37	1.116	1.028	0.758	0.927	87.18	15.88	11	37	485	54.8	7.88
Q8N4J0	UPF0586 protein C9orf41 OS=Homo sapiens GN=C9orf41 PE=1 SV=1 - [CI041_HUMAN]	1.71	1	1	1	2	0.532	0.420	0.606	0.927	4.57	1.71	1	2	409	47.2	6.32
Q969S9	Ribosome-releasing factor 2, mitochondrial OS=Homo sapiens GN=GFM2 PE=1 SV=1 - [RRF2M_HUMA	7.57	1	6	6	13	0.976	0.869	0.839	0.927	26.27	7.57	10	13	779	86.5	6.51
Q6BDS2	UHRF1-binding protein 1 OS=Homo sapiens GN=UHRF1BP1 PE=1 SV=1 - [URFB1_HUMAN]	5.56	1	5	8	12	1.208	0.704	1.055	0.927	15.10	5.56	10	12	1440	159.4	6.14
P05186	Alkaline phosphatase, tissue-nonspecific isozyme OS=Homo sapiens GN=ALPL PE=1 SV=4 - [PPBT_HUMAN]	33.40	1	14	14	56	0.631	0.609	0.268	0.927	143.28	33.40	23	56	524	57.3	6.67
Q9NZJ7	Mitochondrial carrier homolog 1 OS=Homo sapiens GN=MTCH1 PE=1 SV=1 - [MTCH1_HUMA	15.94	1	4	4	13	1.050	0.552	0.600	0.927	31.06	15.94	6	13	389	41.5	9.32
Q9NRS6	Sorting nexin-15 OS=Homo sapiens GN=SNX15 PE=1 SV=1 - [SNX15_HUMAN]	14.33	1	4	4	14	1.385	1.378	1.765	0.927	36.54	14.33	6	14	342	38.3	5.19

Q14511	Enhancer of filamentation 1 OS=Homo sapiens GN=NEDD9 PE=1 SV=1 - [CASL_HUMAN]	0.72	1	1	1	1	1.033	0.857	0.861	0.927	2.28	0.72	1	1	834	92.8	6.70
P61599	N-alpha-acetyltransferase 20 OS=Homo sapiens GN=NAA20 PE=1 SV=1 - [NAA20_HUMAN	14.61	1	2	2	6	0.774	0.802	0.938	0.927	10.75	14.61	4	6	178	20.4	5.03
P48147	Prolyl endopeptidase OS=Homo sapiens GN=PREP PE=1 SV=2 - [PPCE_HUMAN]	30.00	1	21	21	87	1.188	0.788	0.944	0.928	209.80	30.00	35	87	710	80.6	5.86
P28161	Glutathione S-transferase Mu 2 OS=Homo sapiens GN=GSTM2 PE=1 SV=2 - [GSTM2_HUMAN]	66.97	1	6	16	164	0.844	0.730	0.988	0.928	421.62	66.97	26	164	218	25.7	6.37
P01891	HLA class I histocompatibility antigen, A-68 alpha chain OS=Homo sapiens GN=HLA-A PE=1 SV=4 - [1A68_HUMAN]	51.78	2	5	21	143	0.951	1.071	0.948	0.928	350.48	51.78	31	143	365	40.9	6.70
Q14324	Myosin-binding protein C, fast-type OS=Homo sapiens GN=MYBPC2 PE=1 SV=2 - [MYPC2_HUMAN]	0.70	1	1	1	18	1.384	0.815	0.911	0.928	42.20	0.70	1	18	1141	128.0	7.52
Q9P206	Uncharacterized protein KIAA1522 OS=Homo sapiens GN=KIAA1522 PE=1 SV=2 - [K1522_HUMAN]	5.70	1	5	5	13	1.048	1.957	1.608	0.928	36.07	5.70	8	13	1035	107.0	9.73
Q8N4C8	Misshapen-like kinase 1 OS=Homo sapiens GN=MINK1 PE=1 SV=2 - [MINK1_HUMAN]	11.49	1	8	14	29	1.212	1.032	1.109	0.928	71.25	11.49	23	29	1332	149.7	7.85
Q8TDY4	Arf-GAP with SH3 domain, ANK repeat and PH domain-containing protein 3 OS=Homo sapiens GN=ASAP3 PE=1 SV=1 - [ASAP3_HUMAN]	6.09	1	4	4	7	0.957	1.195	0.910	0.928	22.14	6.09	6	7	903	99.1	6.40

Q13409	Cytoplasmic dynein 1 intermediate chain 2 OS=Homo sapiens GN=DYNC1I2 PE=1 SV=3 - [DC1I2_HUMAN]	25.71	2	15	15	123	1.102	1.092	1.032	0.928	283.41	25.71	27	123	638	71.4	5.20
P13929	Beta-enolase OS=Homo sapiens GN=ENO3 PE=1 SV=5 - [ENOB_HUMAN]	25.35	1	2	10	302	0.991	0.861	0.932	0.928	641.99	25.35	13	302	434	47.0	7.71
Q32P41	tRNA (guanine(37)-N1)-methyltransfer ase OS=Homo sapiens GN=TRMT5 PE=1 SV=2 - [TRM5_HUMAN]	3.93	1	2	2	4	0.855	1.337	0.896	0.928	8.48	3.93	2	4	509	58.2	8.62
Q9BSJ2	Gamma-tubulin complex component 2 OS=Homo sapiens GN=TUBGCP2 PE=1 SV=2 - [GCP2_HUMAN]	10.09	1	8	8	14	0.950	0.867	0.868	0.928	38.44	10.09	12	14	902	102.5	6.84
P42892	Endothelin-converting enzyme 1 OS=Homo sapiens GN=ECE1 PE=1 SV=2 - [ECE1_HUMAN]	22.73	1	20	20	79	1.361	0.782	0.828	0.928	199.70	22.73	31	79	770	87.1	5.88
P40227	T-complex protein 1 subunit zeta OS=Homo sapiens GN=CCT6A PE=1 SV=3 - [TCPZ_HUMAN]	54.43	2	32	32	173	0.951	0.949	0.912	0.928	362.09	54.43	48	173	531	58.0	6.68
Q13131	5'-AMP-activated protein kinase catalytic subunit alpha-1 OS=Homo sapiens GN=PRKAA1 PE=1 SV=4 - [AAPK1_HUMAN	19.50	2	10	10	28	1.032	0.943	1.084	0.928	77.06	19.50	13	28	559	64.0	8.12
P35606	Coatomer subunit beta' OS=Homo sapiens GN=COPB2 PE=1 SV=2 - [COPB2_HUMA N]	39.51	1	34	34	242	0.930	0.916	0.950	0.929	592.02	39.51	57	242	906	102.4	5.27
P14649	Myosin light chain 6B OS=Homo sapiens GN=MYL6B PE=1 SV=1 - [MYL6B_HUMAN]	38.94	1	5	9	262	1.048	1.092	1.148	0.929	606.14	38.94	14	262	208	22.7	5.73

Q92990	Glomulin OS=Homo sapiens GN=GLMN PE=1 SV=2 - [GLMN_HUMAN]	4.38	1	3	3	9	0.695	0.796	0.897	0.929	13.99	4.38	5	9	594	68.2	5.33
Q9Y3X0	Coiled-coil domain-containing protein 9 OS=Homo sapiens GN=CCDC9 PE=1 SV=1 - [CCDC9_HUMAN]	11.68	1	5	5	22	1.342	1.125	1.202	0.929	42.62	11.68	7	22	531	59.7	5.40
Q9NYH9	U3 small nucleolar RNA-associated protein 6 homolog OS=Homo sapiens GN=UTP6 PE=1 SV=2 - [UTP6_HUMAN]	9.05	1	5	5	11	0.684	0.774	0.810	0.929	22.00	9.05	7	11	597	70.1	7.28
O95163	Elongator complex protein 1 OS=Homo sapiens GN=IKBKAP PE=1 SV=3 - [ELP1_HUMAN]	8.78	1	8	8	12	0.788	0.795	0.795	0.929	22.56	8.78	11	12	1332	150.2	5.94
P62195	26S protease regulatory subunit 8 OS=Homo sapiens GN=PSMC5 PE=1 SV=1 - [PRS8_HUMAN]	65.76	1	25	27	110	1.002	0.997	1.017	0.929	257.13	65.76	42	110	406	45.6	7.55
P42704	Leucine-rich PPR motif-containing protein, mitochondrial OS=Homo sapiens GN=LRPPRC PE=1 SV=3 - [LPPRC_HUMAN]	35.22	1	48	48	147	0.774	1.029	0.966	0.929	364.82	35.22	75	147	1394	157.8	6.13
Q9Y4F5	Centrosomal protein of 170 kDa protein B OS=Homo sapiens GN=CEP170B PE=1 SV=4 - [C170B_HUMAN]	9.31	1	10	12	26	1.089	1.271	1.320	0.929	64.38	9.31	17	26	1589	171.6	6.84
Q9BRK0	Receptor expression-enhancing protein 2 OS=Homo sapiens GN=REEP2 PE=1 SV=2 - [REEP2_HUMAN]	13.10	1	2	2	2	2.806	1.194	2.489	0.929	7.57	13.10	2	2	252	28.2	9.54

Q9BXB4	Oxysterol-binding protein-related protein 11 OS=Homo sapiens GN=OSBPL11 PE=1 SV=2 - [OSB11_HUMAN]	6.16	1	3	4	7	0.855	0.840	0.986	0.930	14.12	6.16	6	7	747	83.6	7.06
O00232	26S proteasome non-ATPase regulatory subunit 12 OS=Homo sapiens GN=PSMD12 PE=1 SV=3 - [PSD12_HUMAN	50.00	1	21	22	79	0.910	0.861	0.922	0.930	211.85	50.00	35	79	456	52.9	7.65
Q9UFG5	UPF0449 protein C19orf25 OS=Homo sapiens GN=C19orf25 PE=1 SV=2 - [CS025_HUMAN]	38.98	1	3	3	12	0.798	1.181	1.014	0.930	33.38	38.98	6	12	118	12.9	5.07
Q96AY4	Tetratricopeptide repeat protein 28 OS=Homo sapiens GN=TTC28 PE=1 SV=4 - [TTC28_HUMAN	8.18	1	16	17	42	1.284	0.906	0.862	0.930	89.14	8.18	24	42	2481	270.7	6.89
Q9Y4A5	Transformation/transcription domain-associated protein OS=Homo sapiens GN=TRRAP PE=1 SV=3 - [TRRAP_HUMAN]	1.79	1	4	5	7	1.254	1.372	1.421	0.930	15.31	1.79	6	7	3859	437.3	8.19
P27797	Calreticulin OS=Homo sapiens GN=CALR PE=1 SV=1 - [CALR_HUMAN]	75.78	1	28	28	678	0.732	1.013	0.920	0.930	1734.33	75.78	44	678	417	48.1	4.44
Q9C0B1	Alpha-ketoglutarate-dependent dioxygenase FTO OS=Homo sapiens GN=FTO PE=1 SV=3 - [FTO_HUMAN]	22.38	1	10	10	19	0.860	1.008	0.972	0.930	40.59	22.38	15	19	505	58.2	5.22
Q2NKX8	DNA excision repair protein ERCC-6-like OS=Homo sapiens GN=ERCC6L PE=1 SV=1 - [ERC6L_HUMAN]	2.88	1	4	4	7	1.200	0.893	0.726	0.930	8.30	2.88	5	7	1250	141.0	5.31

Q53GQ0	Estradiol 17-beta-dehydrogenase 12 OS=Homo sapiens GN=HSD17B12 PE=1 SV=2 - [DHB12_HUMAN]	23.08	1	6	6	21	0.834	0.518	0.641	0.930	54.40	23.08	11	21	312	34.3	9.32
O75223	Gamma-glutamylcyclotransferase OS=Homo sapiens GN=GGCT PE=1 SV=1 - [GGCT_HUMAN]	21.81	1	4	4	27	0.716	0.922	0.653	0.930	62.08	21.81	6	27	188	21.0	5.14
Q8N653	Leucine-zipper-like transcriptional regulator 1 OS=Homo sapiens GN=LZTR1 PE=1 SV=2 - [LZTR1_HUMAN]	1.79	1	2	2	2	1.182	0.698	1.103	0.930	5.07	1.79	2	2	840	94.7	6.57
O60706	ATP-binding cassette sub-family C member 9 OS=Homo sapiens GN=ABCC9 PE=1 SV=2 - [ABCC9_HUMAN]	1.87	2	2	4	15	3.565	1.179	1.083	0.930	28.84	1.87	5	15	1549	174.1	7.37
O15320	cTAGE family member 5 OS=Homo sapiens GN=CTAGE5 PE=1 SV=4 - [CTGE5_HUMAN]	10.95	2	5	9	30	0.727	0.960	0.647	0.931	63.20	10.95	11	30	804	90.9	5.22
Q14165	Malectin OS=Homo sapiens GN=MLEC PE=1 SV=1 - [MLEC_HUMAN]	31.51	1	9	9	44	0.690	0.803	0.650	0.931	105.56	31.51	16	44	292	32.2	5.41
Q9H3M7	Thioredoxin-interacting protein OS=Homo sapiens GN=TXNIP PE=1 SV=1 - [TXNIP_HUMAN]	7.42	1	2	2	5	2.785	1.088	0.873	0.931	14.59	7.42	4	5	391	43.6	7.50
Q0ZGT2	Nexilin OS=Homo sapiens GN=NEXN PE=1 SV=1 - [NEXN_HUMAN]	49.48	2	47	48	299	0.942	1.073	1.153	0.931	729.19	49.48	70	299	675	80.6	5.33
P78324	Tyrosine-protein phosphatase non-receptor type substrate 1 OS=Homo sapiens GN=SIRPA PE=1 SV=2 - [SHPS1_HUMAN]	23.21	4	9	9	34	1.569	0.533	0.571	0.931	89.59	23.21	16	34	504	54.9	6.98

Q8N7U6	EF-hand domain-containing family member B OS=Homo sapiens GN=EFHB PE=2 SV=4 - [EFHB_HUMAN]	1.80	1	1	1	2	3.826	1.243	1.404	0.931	0.00	1.80	1	2	833	93.7	7.58
Q5ZPR3	CD276 antigen OS=Homo sapiens GN=CD276 PE=1 SV=1 - [CD276_HUMAN]	17.98	1	4	4	24	0.863	0.792	0.863	0.931	50.54	17.98	7	24	534	57.2	4.91
Q9BU02	Thiamine-triphosphatase OS=Homo sapiens GN=THTPA PE=1 SV=3 - [THTPA_HUMAN]	29.13	1	5	5	9	0.976	1.396	1.769	0.931	23.47	29.13	8	9	230	25.6	4.82
P20648	Potassium-transporting ATPase alpha chain 1 OS=Homo sapiens GN=ATP4A PE=2 SV=5 - [ATP4A_HUMAN]	6.18	1	2	6	60	1.057	0.699	1.109	0.931	187.00	6.18	9	60	1035	114.0	5.81
P50990	T-complex protein 1 subunit theta OS=Homo sapiens GN=CCT8 PE=1 SV=4 - [TCPQ_HUMAN]	66.06	1	36	36	311	0.872	0.956	0.897	0.931	738.14	66.06	62	311	548	59.6	5.60
P46778	60S ribosomal protein L21 OS=Homo sapiens GN=RPL21 PE=1 SV=2 - [RL21_HUMAN]	38.13	1	8	8	34	0.835	0.953	0.817	0.931	52.07	38.13	12	34	160	18.6	10.49
P62937	Peptidyl-prolyl cis-trans isomerase A OS=Homo sapiens GN=PPIA PE=1 SV=2 - [PPIA_HUMAN]	83.64	4	15	16	953	1.055	1.030	1.047	0.932	2355.85	83.64	26	953	165	18.0	7.81
P30520	Adenylosuccinate synthetase isozyme 2 OS=Homo sapiens GN=ADSS PE=1 SV=3 - [PURA2_HUMAN]	42.54	1	18	19	113	1.070	0.730	1.010	0.932	286.62	42.54	28	113	456	50.1	6.55
P43034	Platelet-activating factor acetylhydrolase IB subunit alpha OS=Homo sapiens GN=PAFAH1B1 PE=1 SV=2 -	33.66	1	14	15	70	1.277	0.986	1.124	0.932	172.09	33.66	25	70	410	46.6	7.37

Q9Y5Y2	Cytosolic Fe-S cluster assembly factor NUBP2 OS=Homo sapiens GN=NUBP2 PE=1 SV=1 - [NUBP2_HUMAN	8.86	1	2	2	3	0.921	0.728	0.927	0.932	6.94	8.86	3	3	271	28.8	5.83
Q86SQ9	Dehydrodolichyl diphosphate syntase complex subunit DHDDS OS=Homo sapiens GN=DHDDS PE=1 SV=3 - [DHDDS_HUMAN]	5.41	1	1	2	3	1.062	0.669	1.279	0.932	6.13	5.41	2	3	333	38.6	8.28
Q63HR2	Tensin-like C1 domain-containing phosphatase OS=Homo sapiens GN=TENC1 PE=1 SV=2 - [TENC1_HUMAN]	6.32	1	5	7	26	1.030	1.231	0.971	0.932	54.81	6.32	11	26	1409	152.5	8.35
O95470	Sphingosine-1-phosphate lyase 1 OS=Homo sapiens GN=SGPL1 PE=1 SV=3 - [SGPL1_HUMAN	13.73	1	7	7	28	0.893	1.149	1.012	0.932	79.20	13.73	11	28	568	63.5	9.16
P83436	Conserved oligomeric Golgi complex subunit 7 OS=Homo sapiens GN=COG7 PE=1 SV=1 - [COG7_HUMAN]	10.65	1	7	7	14	0.966	0.988	1.059	0.932	36.26	10.65	10	14	770	86.3	5.47
Q96RS6	NudC domain-containing protein 1 OS=Homo sapiens GN=NUDCD1 PE=1 SV=2 - [NUDC1_HUMAN]	8.92	1	4	6	16	0.988	0.717	0.775	0.932	37.20	8.92	9	16	583	66.7	5.11
Q6XUX3	Dual serine/threonine and tyrosine protein kinase OS=Homo sapiens GN=DSTYK PE=1 SV=2 - [DUSTY_HUMAN]	1.51	1	1	2	5	0.714	0.309	0.653	0.932	5.94	1.51	3	5	929	105.1	6.74
Q96PE3	Type I inositol 3,4-bisphosphate 4-phosphatase OS=Homo sapiens GN=INPP4A PE=1 SV=1 - [INP4A_HUMAN]	4.81	1	4	4	17	1.095	1.293	1.806	0.932	35.44	4.81	5	17	977	109.9	6.96

P41208	Centrin-2 OS=Homo sapiens GN=CETN2 PE=1 SV=1 - [CETN2_HUMAN]	16.28	2	3	3	8	1.059	1.400	1.215	0.933	19.57	16.28	5	8	172	19.7	5.00
Q2TBE0	CWF19-like protein 2 OS=Homo sapiens GN=CWF19L2 PE=1 SV=4 - [C19L2_HUMAN]	2.80	1	2	3	3	0.757	1.020	0.755	0.933	5.56	2.80	3	3	894	103.7	8.65
P37173	TGF-beta receptor type-2 OS=Homo sapiens GN=TGFBR2 PE=1 SV=2 - [TGFR2_HUMAN]	8.64	1	3	3	3	1.104	1.463	0.726	0.933	11.63	8.64	3	3	567	64.5	5.90
Q86X83	COMM domain-containing protein 2 OS=Homo sapiens GN=COMMD2 PE=1 SV=2 - [COMD2_HUMAN]	12.56	1	3	3	12	0.925	0.955	1.009	0.933	15.27	12.56	6	12	199	22.7	6.73
Q9NZC3	Glycerophosphodiester phosphodiesterase 1 OS=Homo sapiens GN=GDE1 PE=1 SV=1 - [GDE1_HUMAN]	9.67	1	3	3	5	1.282	0.788	1.031	0.933	7.87	9.67	4	5	331	37.7	6.71
Q9H299	SH3 domain-binding glutamic acid-rich-like protein 3 OS=Homo sapiens GN=SH3BGRL3 PE=1 SV=1 - [SH3L3_HUMAN]	92.47	2	10	10	239	0.818	0.918	1.365	0.933	426.57	92.47	16	239	93	10.4	4.93
Q7L099	Protein RUFY3 OS=Homo sapiens GN=RUFY3 PE=1 SV=1 - [RUFY3_HUMAN]	15.78	1	7	7	15	1.079	0.916	1.236	0.933	22.82	15.78	10	15	469	52.9	5.49
P08842	Steryl-sulfatase OS=Homo sapiens GN=STS PE=1 SV=2 - [STS_HUMAN]	6.86	1	4	4	6	1.619	1.000	0.598	0.933	16.00	6.86	6	6	583	65.5	7.66
Q9HCM7	Fibrosin-1-like protein OS=Homo sapiens GN=FBRSL1 PE=1 SV=4 - [FBSL_HUMAN]	0.57	1	1	1	2	0.668	0.749	0.775	0.933	4.27	0.57	1	2	1045	110.8	9.67

Q96GK7	Fumarylacetoacetate hydrolase domain-containing protein 2A OS=Homo sapiens GN=FAHD2A PE=1 SV=1 - [FAH2A_HUMAN	38.85	2	8	8	22	0.828	0.992	0.864	0.933	67.67	38.85	12	22	314	34.6	8.24
Q8TEQ6	Gem-associated protein 5 OS=Homo sapiens GN=GEMIN5 PE=1 SV=3 - [GEMI5_HUMAN]	5.84	1	8	8	15	0.806	0.605	0.606	0.933	34.32	5.84	11	15	1508	168.5	6.62
P78357	Contactin-associated protein 1 OS=Homo sapiens GN=CNTNAP1 PE=1 SV=1 - [CNTP1_HUMAN]	11.63	1	15	15	50	1.243	1.862	1.263	0.933	113.63	11.63	25	50	1384	156.2	7.05
Q8TE56	A disintegrin and metalloproteinase with thrombospondin motifs 17 OS=Homo sapiens GN=ADAMTS17 PE=2 SV=2 - [ATS17_HUMAN]	2.56	1	2	2	2	1.321	1.090	1.016	0.933	4.89	2.56	2	2	1095	121.0	8.06
P46063	ATP-dependent DNA helicase Q1 OS=Homo sapiens GN=RECQL PE=1 SV=3 - [RECQ1_HUMA	28.81	1	17	18	64	1.049	1.050	0.949	0.933	149.61	28.81	26	64	649	73.4	7.88
P50395	Rab GDP dissociation inhibitor beta OS=Homo sapiens GN=GDI2 PE=1 SV=2 - [GDIB_HUMAN]	64.94	2	24	31	253	1.007	0.976	0.982	0.933	579.16	64.94	49	253	445	50.6	6.47
O75787	Renin receptor OS=Homo sapiens GN=ATP6AP2 PE=1 SV=2 - [RENR_HUMAN]	16.00	1	5	5	11	0.831	0.771	0.940	0.934	25.56	16.00	7	11	350	39.0	6.10
P18627	Lymphocyte activation gene 3 protein OS=Homo sapiens GN=LAG3 PE=1 SV=5 - [LAG3_HUMAN]	3.24	1	1	1	1	0.938	0.974	1.331	0.934	2.38	3.24	1	1	525	57.4	7.97
P15529	Membrane cofactor protein OS=Homo sapiens GN=CD46 PE=1 SV=3 - [MCP_HUMAN]	6.63	1	4	4	15	1.108	0.755	1.053	0.934	24.50	6.63	6	15	392	43.7	6.74

P49840	Glycogen synthase kinase-3 alpha OS=Homo sapiens GN=GSK3A PE=1 SV=2 - [GSK3A_HUMAN]	23.19	1	4	8	31	0.986	0.980	0.744	0.934	62.09	23.19	13	31	483	50.9	8.75
Q93075	Putative deoxyribonuclease TATDN2 OS=Homo sapiens GN=TATDN2 PE=2 SV=2 - [TATD2_HUMAN]	1.31	1	1	1	2	0.794	0.610	1.447	0.934	4.93	1.31	1	2	761	85.0	7.39
P35052	Glypican-1 OS=Homo sapiens GN=GPC1 PE=1 SV=2 - [GPC1_HUMAN]	6.09	1	4	4	10	0.518	0.455	0.909	0.934	24.39	6.09	6	10	558	61.6	7.30
Q02218	2-oxoglutarate dehydrogenase, mitochondrial OS=Homo sapiens GN=OGDH PE=1 SV=3 - [ODO1_HUMAN]	25.12	2	23	23	88	1.164	1.288	1.303	0.934	215.80	25.12	38	88	1023	115.9	6.86
O43896	Kinesin-like protein KIF1C OS=Homo sapiens GN=KIF1C PE=1 SV=3 - [KIF1C_HUMAN]	20.94	3	12	19	49	1.031	1.175	1.118	0.934	119.62	20.94	27	49	1103	122.9	6.90
Q9Y4L1	Hypoxia up-regulated protein 1 OS=Homo sapiens GN=HYOU1 PE=1 SV=1 - [HYOU1_HUMAN]	43.14	1	43	44	234	0.841	1.012	0.861	0.934	600.66	43.14	73	234	999	111.3	5.22
Q9UNS1	Protein timeless homolog OS=Homo sapiens GN=TIMELESS PE=1 SV=2 - [TIM_HUMAN]	1.16	1	1	1	1	1.180	1.323	1.013	0.934	2.73	1.16	1	1	1208	138.6	5.40
O75347	Tubulin-specific chaperone A OS=Homo sapiens GN=TBCA PE=1 SV=3 - [TBCA_HUMAN]	66.67	1	11	11	103	0.853	1.000	1.052	0.934	248.46	66.67	21	103	108	12.8	5.29
Q9Y5K6	CD2-associated protein OS=Homo sapiens GN=CD2AP PE=1 SV=1 - [CD2AP_HUMAN]	37.25	1	22	23	58	0.950	1.002	1.307	0.934	146.21	37.25	34	58	639	71.4	6.40

P40197	Platelet glycoprotein V OS=Homo sapiens GN=GP5 PE=1 SV=1 - [GPV_HUMAN]	2.50	1	2	2	2	0.937	1.762	0.932	0.934	3.85	2.50	2	2	560	60.9	9.63
Q68EM7	Rho GTPase-activating protein 17 OS=Homo sapiens GN=ARHGAP17 PE=1 SV=1 - [RHG17_HUMAN]	36.55	1	21	21	58	1.037	0.693	1.105	0.935	163.30	36.55	31	58	881	95.4	7.62
Q9H977	WD repeat-containing protein 54 OS=Homo sapiens GN=WDR54 PE=1 SV=1 - [WDR54_HUMAN]	2.99	1	1	1	4	1.155	0.858	1.048	0.935	7.27	2.99	2	4	334	35.9	6.20
Q7Z2Z2	Elongation factor Tu GTP-binding domain-containing protein 1 OS=Homo sapiens GN=EFTUD1 PE=1 SV=2 - [ETUD1_HUMAN]	10.71	1	12	12	34	1.047	1.315	1.280	0.935	59.66	10.71	20	34	1120	125.3	5.91
Q0PNE2	Elongator complex protein 6 OS=Homo sapiens GN=ELP6 PE=1 SV=1 - [ELP6_HUMAN]	5.64	1	1	1	2	0.768	1.014	0.762	0.935	5.53	5.64	2	2	266	29.8	6.84
Q8IZF3	Probable G-protein coupled receptor 115 OS=Homo sapiens GN=GPR115 PE=2 SV=3 - [GP115_HUMAN	2.30	1	1	1	1	2.679	1.968	1.777	0.935	2.89	2.30	1	1	695	77.7	9.01
O60762	Dolichol-phosphate mannosyltransferase subunit 1 OS=Homo sapiens GN=DPM1 PE=1 SV=1 - [DPM1_HUMAN]	21.92	1	5	5	13	0.816	0.890	0.725	0.935	36.57	21.92	6	13	260	29.6	9.57
Q9BZH6	WD repeat-containing protein 11 OS=Homo sapiens GN=WDR11 PE=1 SV=1 - [WDR11_HUMAN]	8.33	1	10	10	32	0.809	0.833	0.875	0.935	56.99	8.33	16	32	1224	136.6	6.92

Q969N4	Trace amine-associated receptor 8 OS=Homo sapiens GN=TAAR8 PE=2 SV=1 - [TAAR8_HUMAN]	3.80	1	1	1	2	0.821	0.869	0.530	0.935	4.70	3.80	1	2	342	38.0	6.81
Q9NX05	Constitutive coactivator of PPAR-gamma-like protein 2 OS=Homo sapiens GN=FAM120C PE=2 SV=3 - [F120C_HUMAN]	6.48	1	4	6	14	1.075	0.737	0.675	0.935	28.01	6.48	9	14	1096	120.5	9.03
Q9NPQ8	Synembryn-A OS=Homo sapiens GN=RIC8A PE=1 SV=3 - [RIC8A_HUMAN]	23.54	1	10	11	61	1.058	0.847	1.083	0.935	154.64	23.54	19	61	531	59.7	5.33
Q15835	Rhodopsin kinase OS=Homo sapiens GN=GRK1 PE=1 SV=1 - [RK_HUMAN]	1.07	1	1	1	1	0.905	0.808	1.201	0.935	2.31	1.07	1	1	563	63.5	5.87
Q96A54	Adiponectin receptor protein 1 OS=Homo sapiens GN=ADIPOR1 PE=1 SV=1 - [ADR1_HUMAN]	4.00	1	1	1	7	0.851	0.777	0.813	0.935	7.98	4.00	2	7	375	42.6	7.02
O94842	TOX high mobility group box family member 4 OS=Homo sapiens GN=TOX4 PE=1 SV=1 - [TOX4_HUMAN]	5.48	2	2	3	4	1.026	1.260	1.187	0.935	11.33	5.48	4	4	621	66.2	5.06
P0C875	Protein FAM228B OS=Homo sapiens GN=FAM228B PE=2 SV=1 - [F228B_HUMAN]	3.43	1	1	1	1		1.296	2.509	0.935	2.46	3.43	1	1	321	37.4	9.29
P40616	ADP-ribosylation factor-like protein 1 OS=Homo sapiens GN=ARL1 PE=1 SV=1 - [ARL1_HUMAN]	16.57	1	2	2	16	0.915	0.597	0.727	0.935	53.00	16.57	3	16	181	20.4	5.72
Q8N6T3	ADP-ribosylation factor GTPase-activating protein 1 OS=Homo sapiens GN=ARFGAP1 PE=1 SV=2 - [ARFG1_HUMAN]	39.41	1	15	15	68	0.827	0.894	0.998	0.935	144.70	39.41	25	68	406	44.6	5.66

Q8TEH3	DENN domain-containing protein 1A OS=Homo sapiens GN=DENND1A PE=1 SV=2 - [DEN1A_HUMAN]	3.17	1	2	2	4	0.573	1.158	0.961	0.935	9.63	3.17	2	4	1009	110.5	6.96
Q9NRG9	Aladin OS=Homo sapiens GN=AAAS PE=1 SV=1 - [AAAS_HUMAN]	3.48	1	2	2	2	1.078	1.767	1.266	0.935	3.96	3.48	2	2	546	59.5	7.50
P48739	Phosphatidylinositol transfer protein beta isoform OS=Homo sapiens GN=PITPNB PE=1 SV=2 - [PIPNB_HUMAN]	51.29	1	13	14	41	0.837	0.948	0.912	0.935	106.68	51.29	20	41	271	31.5	6.87
P47914	60S ribosomal protein L29 OS=Homo sapiens GN=RPL29 PE=1 SV=2 - [RL29_HUMAN]	22.64	1	4	4	41	0.823	1.111	1.344	0.935	80.81	22.64	6	41	159	17.7	11.66
Q99996	A-kinase anchor protein 9 OS=Homo sapiens GN=AKAP9 PE=1 SV=3 - [AKAP9_HUMAN]	9.26	6	31	39	72	0.854	0.886	0.956	0.935	118.19	9.26	50	72	3911	453.4	4.98
P14735	Insulin-degrading enzyme OS=Homo sapiens GN=IDE PE=1 SV=4 - [IDE_HUMAN]	24.53	1	23	23	54	0.890	0.729	0.897	0.936	98.79	24.53	35	54	1019	117.9	6.61
P08133	Annexin A6 OS=Homo sapiens GN=ANXA6 PE=1 SV=3 - [ANXA6_HUMAN]	72.96	1	56	56	1130	1.236	1.051	1.324	0.936	2983.49	72.96	99	1130	673	75.8	5.60
A5D8V6	Vacuolar protein sorting-associated protein 37C OS=Homo sapiens GN=VPS37C PE=1 SV=2 - [VP37C_HUMAN]	10.99	1	3	3	8	0.956	1.186	1.388	0.936	19.15	10.99	6	8	355	38.6	5.21
Q02779	Mitogen-activated protein kinase kinase kinase 10 OS=Homo sapiens GN=MAP3K10 PE=1 SV=3 - [M3K10_HUMAN]	5.03	3	2	3	9	1.230	0.653	0.549	0.936	14.76	5.03	4	9	954	103.6	7.01

P42356	Phosphatidylinositol 4-kinase alpha OS=Homo sapiens GN=PI4KA PE=1 SV=3 - [PI4KA_HUMAN]	4.31	5	9	9	11	1.205	1.004	0.884	0.936	18.60	4.31	11	11	2044	231.2	6.87
Q9NX62	Inositol monophosphatase 3 OS=Homo sapiens GN=IMPAD1 PE=1 SV=1 - [IMPA3_HUMAN]	10.03	1	4	4	15	0.764	0.865	1.002	0.936	34.49	10.03	7	15	359	38.7	6.86
P61026	Ras-related protein Rab-10 OS=Homo sapiens GN=RAB10 PE=1 SV=1 - [RAB10_HUMAN]	55.00	2	7	11	77	1.393	0.854	0.873	0.936	166.56	55.00	17	77	200	22.5	8.38
P36543	V-type proton ATPase subunit E 1 OS=Homo sapiens GN=ATP6V1E1 PE=1 SV=1 - [VATE1_HUMAN]	40.71	1	5	10	49	1.114	1.010	0.786	0.936	97.25	40.71	17	49	226	26.1	8.00
Q9UJY4	ADP-ribosylation factor-binding protein GGA2 OS=Homo sapiens GN=GGA2 PE=1 SV=3 - [GGA2_HUMAN]	16.48	1	7	7	8	1.046	1.282	0.987	0.936	15.49	16.48	8	8	613	67.1	6.55
Q9NQ89	Uncharacterized protein C12orf4 OS=Homo sapiens GN=C12orf4 PE=2 SV=1 - [CL004_HUMAN]	2.72	1	1	2	2	0.789	0.815	0.972	0.936	5.91	2.72	2	2	552	63.8	6.37
Q86Y56	Dynein assembly factor 5, axonemal OS=Homo sapiens GN=HEATR2 PE=1 SV=4 - [DAAF5_HUMAN]	5.61	1	5	5	12	0.788	1.252	0.821	0.936	31.50	5.61	7	12	855	93.5	6.42
Q7Z7G8	Vacuolar protein sorting-associated protein 13B OS=Homo sapiens GN=VPS13B PE=1 SV=2 - [VP13B_HUMAN]	0.85	1	2	3	4	0.570	1.965	1.943	0.937	4.09	0.85	3	4	4022	448.4	6.46
O75844	CAAX prenyl protease 1 homolog OS=Homo sapiens GN=ZMPSTE24 PE=1 SV=2 - [FACE1_HUMAN]	15.79	1	6	6	13	0.828	0.915	0.879	0.937	42.06	15.79	7	13	475	54.8	7.49

P55145	Mesencephalic astrocyte-derived neurotrophic factor OS=Homo sapiens GN=MANF PE=1 SV=3 - [MANF_HUMAN]	51.65	1	15	15	58	0.870	1.029	0.850	0.937	116.95	51.65	21	58	182	20.7	8.69
Q9H5N1	Rab GTPase-binding effector protein 2 OS=Homo sapiens GN=RABEP2 PE=1 SV=2 - [RABE2_HUMAN	23.37	3	11	12	57	1.069	1.016	1.020	0.937	145.02	23.37	23	57	569	63.5	4.78
O95218	Zinc finger Ran-binding domain-containing protein 2 OS=Homo sapiens GN=ZRANB2 PE=1 SV=2 - [ZRAB2_HUMAN]	16.36	1	4	5	17	0.810	0.830	0.667	0.937	36.81	16.36	7	17	330	37.4	10.01
Q15643	Thyroid receptor-interacting protein 11 OS=Homo sapiens GN=TRIP11 PE=1 SV=3 - [TRIPB_HUMAN]	11.07	2	21	23	45	0.824	0.710	0.812	0.937	97.93	11.07	32	45	1979	227.4	5.26
P61923	Coatomer subunit zeta-1 OS=Homo sapiens GN=COPZ1 PE=1 SV=1 - [COPZ1_HUMA N]	45.76	1	6	6	58	0.971	0.889	0.718	0.937	165.02	45.76	9	58	177	20.2	4.81
P22307	Non-specific lipid-transfer protein OS=Homo sapiens GN=SCP2 PE=1 SV=2 - [NLTP_HUMAN]	38.94	1	24	24	166	1.158	0.895	0.998	0.937	373.26	38.94	38	166	547	59.0	6.89
Q96MW5	Conserved oligomeric Golgi complex subunit 8 OS=Homo sapiens GN=COG8 PE=1 SV=2 - [COG8_HUMAN]	17.81	1	7	7	12	0.768	1.058	1.222	0.937	28.93	17.81	9	12	612	68.4	5.20
P55263	Adenosine kinase OS=Homo sapiens GN=ADK PE=1 SV=2 - [ADK_HUMAN]	24.86	1	11	11	24	0.684	0.994	0.931	0.937	60.36	24.86	15	24	362	40.5	6.70

Q9Y2Z0	Suppressor of G2 allele of SKP1 homolog OS=Homo sapiens GN=SUGT1 PE=1 SV=3 - [SUGT1_HUMAN]	53.15	1	17	17	76	0.781	0.959	1.035	0.937	191.84	53.15	27	76	365	41.0	5.16
P31946	14-3-3 protein beta/alpha OS=Homo sapiens GN=YWHAB PE=1 SV=3 - [1433B_HUMAN]	76.42	2	12	21	390	1.048	0.939	0.945	0.937	1115.16	76.42	29	390	246	28.1	4.83
Q9NRN5	Olfactomedin-like protein 3 OS=Homo sapiens GN=OLFML3 PE=2 SV=1 - [OLFL3_HUMAN]	4.43	1	2	2	3	0.694	1.416	0.590	0.937	5.90	4.43	2	3	406	46.0	6.57
P05386	60S acidic ribosomal protein P1 OS=Homo sapiens GN=RPLP1 PE=1 SV=1 - [RLA1_HUMAN]	34.21	1	2	3	36	0.828	0.780	0.737	0.937	144.70	34.21	4	36	114	11.5	4.32
Q9Y6G5	COMM domain-containing protein 10 OS=Homo sapiens GN=COMMD10 PE=1 SV=1 - [COMDA_HUMAN]	18.81	1	3	3	5	0.969	1.012	0.848	0.937	12.36	18.81	4	5	202	23.0	6.54
Q7RTW8	Otoancorin OS=Homo sapiens GN=OTOA PE=1 SV=1 - [OTOAN_HUMAN]	1.30	1	1	1	1	2.013	1.063	0.998	0.937	2.41	1.30	1	1	1153	128.5	5.82
Q14676	Mediator of DNA damage checkpoint protein 1 OS=Homo sapiens GN=MDC1 PE=1 SV=3 - [MDC1_HUMAN]	1.24	1	2	2	3	0.888	1.817	1.008	0.937	7.80	1.24	3	3	2089	226.5	5.47
Q96GY0	Zinc finger C2HC domain-containing protein 1A OS=Homo sapiens GN=ZC2HC1A PE=1 SV=2 - [ZC21A_HUMAN]	27.38	1	10	10	25	1.115	1.693	1.647	0.937	51.47	27.38	17	25	325	35.1	9.82
Q7Z4H3	HD domain-containing protein 2 OS=Homo sapiens GN=HDDC2 PE=1 SV=1 - [HDDC2_HUMAN]	17.65	1	3	3	9	1.026	1.185	1.376	0.937	21.26	17.65	4	9	204	23.4	5.49

O94788	Retinal dehydrogenase 2 OS=Homo sapiens GN=ALDH1A2 PE=1 SV=3 - [AL1A2_HUMAN]	6.95	1	3	5	11	0.670	2.771	3.635	0.937	25.39	6.95	7	11	518	56.7	6.05
P28827	Receptor-type tyrosine-protein phosphatase mu OS=Homo sapiens GN=PTPRM PE=1 SV=2 - [PTPRM_HUMA	4.68	1	5	6	17	2.308	0.623	1.015	0.938	29.66	4.68	10	17	1452	163.6	6.65
Q15363	Transmembrane emp24 domain-containing protein 2 OS=Homo sapiens GN=TMED2 PE=1 SV=1 - [TMED2_HUMA	29.85	1	6	6	37	0.800	0.930	0.791	0.938	86.85	29.85	9	37	201	22.7	5.17
Q9BVG9	Phosphatidylserine synthase 2 OS=Homo sapiens GN=PTDSS2 PE=1 SV=1 - [PTSS2_HUMAN]	7.60	1	2	2	3	1.409	0.949	0.840	0.938	10.69	7.60	2	3	487	56.2	6.25
O43432	Eukaryotic translation initiation factor 4 gamma 3 OS=Homo sapiens GN=EIF4G3 PE=1 SV=2 - [IF4G3_HUMAN]	26.18	1	31	40	161	1.035	0.795	0.842	0.938	342.51	26.18	62	161	1585	176.5	5.38
P55289	Cadherin-12 OS=Homo sapiens GN=CDH12 PE=2 SV=2 - [CAD12_HUMAN]	1.26	1	1	1	1	0.882	0.990	1.195	0.938	3.27	1.26	1	1	794	88.3	4.81
Q7RTS9	Dymeclin OS=Homo sapiens GN=DYM PE=1 SV=1 - [DYM_HUMAN]	1.49	1	1	1	6	0.800	0.746	0.791	0.938	10.35	1.49	2	6	669	75.9	5.86
Q00587	Cdc42 effector protein 1 OS=Homo sapiens GN=CDC42EP1 PE=1 SV=1 - [BORG5_HUMA N]	23.02	1	7	7	25	0.981	0.416	0.642	0.938	64.98	23.02	12	25	391	40.3	7.15
Q6ZMP0	Thrombospondin type-1 domain-containing protein 4 OS=Homo sapiens GN=THSD4 PE=2 SV=2 - [THSD4_HUMAN]	1.47	1	1	1	2	0.727	0.567	1.119	0.938	8.26	1.47	1	2	1018	112.4	7.65

P56524	Histone deacetylase 4 OS=Homo sapiens GN=HDAC4 PE=1 SV=3 - [HDAC4_HUMAN]	6.37	1	5	5	16	0.959	1.588	0.969	0.938	46.64	6.37	8	16	1084	119.0	6.96
Q7Z739	YTH domain-containing family protein 3 OS=Homo sapiens GN=YTHDF3 PE=1 SV=1 - [YTHD3_HUMAN]	15.73	1	7	8	35	1.030	0.896	0.927	0.938	65.50	15.73	13	35	585	63.8	9.04
Q8TF05	Serine/threonine-protein phosphatase 4 regulatory subunit 1 OS=Homo sapiens GN=PPP4R1 PE=1 SV=1 - [PP4R1_HUMAN]	8.21	1	7	7	22	1.025	1.005	1.222	0.938	52.81	8.21	11	22	950	106.9	4.77
Q99471	Prefoldin subunit 5 OS=Homo sapiens GN=PFDN5 PE=1 SV=2 - [PFD5_HUMAN]	74.68	1	9	9	24	0.882	1.048	0.998	0.938	67.12	74.68	13	24	154	17.3	6.33
Q8TDH9	Biogenesis of lysosome-related organelles complex 1 subunit 5 OS=Homo sapiens GN=BLOC1S5 PE=1 SV=1 - [BL1S5_HUMAN]	26.20	1	4	5	12	0.867	0.955	1.021	0.938	22.34	26.20	7	12	187	21.6	7.59
Q99504	Eyes absent homolog 3 OS=Homo sapiens GN=EYA3 PE=1 SV=3 - [EYA3_HUMAN]	2.44	1	1	1	1	1.226	1.181	1.028	0.938	3.97	2.44	1	1	573	62.6	5.21
O14964	Hepatocyte growth factor-regulated tyrosine kinase substrate OS=Homo sapiens GN=HGS PE=1 SV=1 - [HGS_HUMAN]	21.36	1	19	19	74	1.033	0.965	0.914	0.938	157.15	21.36	32	74	777	86.1	6.16
Q5HYC2	Uncharacterized protein KIAA2026 OS=Homo sapiens GN=KIAA2026 PE=2 SV=2 - [K2026_HUMAN]	2.19	1	2	3	9	1.062	0.595	1.201	0.938	20.93	2.19	3	9	2103	227.9	9.04

Q96FJ0	AMSH-like protease OS=Homo sapiens GN=STAMBPL1 PE=1 SV=2 - [STALP_HUMAN]	21.56	1	9	9	22	0.817	0.951	0.569	0.939	62.68	21.56	13	22	436	49.8	7.23
Q9HB71	Calcyclin-binding protein OS=Homo sapiens GN=CACYBP PE=1 SV=2 - [CYBP_HUMAN]	47.81	1	13	14	49	1.193	1.151	1.088	0.939	129.78	47.81	19	49	228	26.2	8.25
O94919	Endonuclease domain-containing 1 protein OS=Homo sapiens GN=ENDOD1 PE=1 SV=2 - [ENDD1_HUMAN]	11.20	1	4	4	46	0.422	0.744	1.032	0.939	107.01	11.20	7	46	500	55.0	5.71
P25445	Tumor necrosis factor receptor superfamily member 6 OS=Homo sapiens GN=FAS PE=1 SV=1 - [TNR6_HUMAN]	25.67	1	10	10	35	0.905	0.900	0.609	0.939	83.12	25.67	17	35	335	37.7	7.94
Q9BQS8	FYVE and coiled-coil domain-containing protein 1 OS=Homo sapiens GN=FYCO1 PE=1 SV=3 - [FYCO1_HUMAN]	31.53	2	39	40	140	1.023	1.134	1.035	0.939	392.85	31.53	66	140	1478	166.9	4.92
Q14697	Neutral alpha-glucosidase AB OS=Homo sapiens GN=GANAB PE=1 SV=3 - [GANAB_HUMAN]	36.65	1	33	33	356	0.881	0.923	0.798	0.939	1058.94	36.65	56	356	944	106.8	6.14
O43815	Striatin OS=Homo sapiens GN=STRN PE=1 SV=4 - [STRN_HUMAN]	22.69	1	11	13	46	0.891	0.974	0.864	0.939	109.69	22.69	21	46	780	86.1	5.27
P22059	Oxysterol-binding protein 1 OS=Homo sapiens GN=OSBP PE=1 SV=1 - [OSBP1_HUMAN]	21.69	1	15	16	52	1.003	0.934	1.062	0.939	125.43	21.69	24	52	807	89.4	7.30
P22681	E3 ubiquitin-protein ligase CBL OS=Homo sapiens GN=CBL PE=1 SV=2 - [CBL_HUMAN]	12.69	1	8	8	18	0.899	1.229	1.184	0.939	37.65	12.69	12	18	906	99.6	6.54

Q13613	Myotubularin-related protein 1 OS=Homo sapiens GN=MTMR1 PE=1 SV=4 - [MTMR1_HUMAN]	4.51	1	3	3	17	1.004	0.941	1.091	0.939	29.24	4.51	6	17	665	74.6	7.14
Q9NS87	Kinesin-like protein KIF15 OS=Homo sapiens GN=KIF15 PE=1 SV=1 - [KIF15_HUMAN]	2.59	1	2	4	15	0.795	1.013	1.193	0.939	31.95	2.59	4	15	1388	160.1	6.00
Q96SL4	Glutathione peroxidase 7 OS=Homo sapiens GN=GPX7 PE=1 SV=1 - [GPX7_HUMAN]	27.27	1	4	4	13	0.699	0.757	0.591	0.939	31.72	27.27	8	13	187	21.0	8.27
P48163	NADP-dependent malic enzyme OS=Homo sapiens GN=ME1 PE=1 SV=1 - [MAOX_HUMAN]	34.27	1	18	18	51	0.850	0.489	1.071	0.939	116.81	34.27	27	51	572	64.1	6.13
P62333	26S protease regulatory subunit 10B OS=Homo sapiens GN=PSMC6 PE=1 SV=1 - [PRS10_HUMAN]	70.44	1	26	26	119	0.902	1.013	1.098	0.939	304.63	70.44	43	119	389	44.1	7.49
P24310	Cytochrome c oxidase subunit 7A1, mitochondrial OS=Homo sapiens GN=COX7A1 PE=1 SV=2 - [CX7A1_HUMAN]	16.46	1	1	1	1	0.463	0.422	0.851	0.939	3.42	16.46	1	1	79	9.1	10.11
Q9Y5P6	Mannose-1-phosphate guanyltransferase beta OS=Homo sapiens GN=GMPPB PE=1 SV=2 - [GMPPB_HUMAN]	20.00	1	6	6	28	1.122	1.173	0.968	0.939	62.81	20.00	12	28	360	39.8	6.61
Q9H074	Polyadenylate-binding protein-interacting protein 1 OS=Homo sapiens GN=PAIP1 PE=1 SV=1 - [PAIP1_HUMAN]	13.99	1	9	9	32	0.874	0.639	0.762	0.940	72.19	13.99	15	32	479	53.5	4.81
Q86SK9	Stearoyl-CoA desaturase 5 OS=Homo sapiens GN=SCD5 PE=1 SV=2 - [SCD5_HUMAN]	9.70	1	2	2	10	1.416	1.370	0.904	0.940	18.63	9.70	3	10	330	37.6	9.61

Q9H082	Ras-related protein Rab-33B OS=Homo sapiens GN=RAB33B PE=1 SV=1 - [RB33B_HUMAN]	13.97	2	1	3	25	0.764	0.925	1.104	0.940	54.92	13.97	5	25	229	25.7	7.18
O00459	Phosphatidylinositol 3-kinase regulatory subunit beta OS=Homo sapiens GN=PIK3R2 PE=1 SV=2 - [P85B_HUMAN]	16.48	1	8	11	29	0.930	0.892	1.083	0.940	69.48	16.48	15	29	728	81.5	6.43
Q9H2M9	Rab3 GTPase-activating protein non-catalytic subunit OS=Homo sapiens GN=RAB3GAP2 PE=1 SV=1 - [RBGPR_HUMAN	18.59	1	19	20	63	0.840	0.868	0.942	0.940	144.91	18.59	32	63	1393	155.9	5.62
Q9ULV5	Heat shock factor protein 4 OS=Homo sapiens GN=HSF4 PE=1 SV=2 - [HSF4_HUMAN]	3.66	1	1	1	2	1.297	0.569	0.851	0.940	0.00	3.66	1	2	492	53.0	5.40
O14841	5-oxoprolinase OS=Homo sapiens GN=OPLAH PE=1 SV=3 - [OPLA_HUMAN]	4.74	1	5	5	12	0.975	1.123	0.959	0.940	31.25	4.74	8	12	1288	137.4	6.58
Q5JRX3	Presequence protease, mitochondrial OS=Homo sapiens GN=PITRM1 PE=1 SV=3 - [PREP_HUMAN]	44.55	1	38	38	140	0.737	1.381	1.216	0.940	348.42	44.55	58	140	1037	117.3	6.92
P42694	Probable helicase with zinc finger domain OS=Homo sapiens GN=HELZ PE=1 SV=2 - [HELZ_HUMAN]	1.85	1	3	3	7	0.932	0.912	1.035	0.940	14.09	1.85	4	7	1942	218.8	7.42
Q00169	Phosphatidylinositol transfer protein alpha isoform OS=Homo sapiens GN=PITPNA PE=1 SV=2 - [PIPNA_HUMAN]	39.26	1	10	11	43	1.176	0.868	0.990	0.940	95.69	39.26	16	43	270	31.8	6.55
Q9BUN8	Derlin-1 OS=Homo sapiens GN=DERL1 PE=1 SV=1 - [DERL1_HUMAN]	5.58	1	1	1	6	0.823	0.827	0.716	0.940	13.19	5.58	2	6	251	28.8	9.51

O75683	Surfeit locus protein 6 OS=Homo sapiens GN=SURF6 PE=1 SV=3 - [SURF6_HUMAN]	21.61	1	8	8	24	0.927	0.978	0.804	0.940	55.77	21.61	11	24	361	41.4	10.64
O43847	Nardilysin OS=Homo sapiens GN=NRD1 PE=1 SV=2 - [NRDC_HUMAN]	13.74	1	13	14	47	0.768	0.776	0.839	0.941	126.00	13.74	20	47	1150	131.5	5.00
O43854	EGF-like repeat and discoidin I-like domain-containing protein 3 OS=Homo sapiens GN=EDIL3 PE=1 SV=1 - [EDIL3_HUMAN]	4.17	1	2	2	2	1.165	1.234	1.780	0.941	4.36	4.17	2	2	480	53.7	7.28
Q9NZ09	Ubiquitin-associated protein 1 OS=Homo sapiens GN=UBAP1 PE=1 SV=1 - [UBAP1_HUMAN]	5.38	1	3	3	3	1.276	0.932	1.124	0.941	6.34	5.38	3	3	502	55.0	5.11
Q15102	Platelet-activating factor acetylhydrolase IB subunit gamma OS=Homo sapiens GN=PAFAH1B3 PE=1 SV=1 - [PA1B3_HUMAN	16.88	1	3	3	25	1.212	1.865	1.243	0.941	41.39	16.88	6	25	231	25.7	6.84
Q9Y263	Phospholipase A-2-activating protein OS=Homo sapiens GN=PLAA PE=1 SV=2 - [PLAP_HUMAN]	25.53	1	17	17	81	0.941	0.971	1.116	0.941	205.64	25.53	29	81	795	87.1	6.37
Q13641	Trophoblast glycoprotein OS=Homo sapiens GN=TPBG PE=1 SV=1 - [TPBG_HUMAN]	12.62	2	5	6	20	0.823	1.303	0.984	0.941	43.55	12.62	9	20	420	46.0	6.83
Q12965	Unconventional myosin-Ie OS=Homo sapiens GN=MYO1E PE=1 SV=2 - [MYO1E_HUMAN]	9.84	1	8	9	30	0.959	1.016	1.401	0.941	57.39	9.84	15	30	1108	127.0	8.92

Q9UMS0	NFU1 iron-sulfur cluster scaffold homolog, mitochondrial OS=Homo sapiens GN=NFU1 PE=1 SV=2 - [NFU1_HUMAN]	10.24	1	2	2	6	1.135	1.268	1.199	0.941	17.18	10.24	3	6	254	28.4	5.07
P08397	Porphobilinogen deaminase OS=Homo sapiens GN=HMBS PE=1 SV=2 - [HEM3_HUMAN]	24.38	1	9	9	28	0.794	1.133	1.078	0.941	69.41	24.38	15	28	361	39.3	7.18
O75688	Protein phosphatase 1B OS=Homo sapiens GN=PPM1B PE=1 SV=1 - [PPM1B_HUMAN]	25.47	1	10	13	58	0.953	0.845	1.276	0.941	129.80	25.47	18	58	479	52.6	5.05
Q96DT5	Dynein heavy chain 11, axonemal OS=Homo sapiens GN=DNAH11 PE=1 SV=3 - [DYH11_HUMAN]	1.50	2	5	6	7	1.093	0.843	0.901	0.941	1.76	1.50	6	7	4523	520.7	6.44
P50502	Hsc70-interacting protein OS=Homo sapiens GN=ST13 PE=1 SV=2 - [F10A1_HUMAN]	33.60	3	12	12	117	0.922	1.148	1.148	0.941	260.76	33.60	20	117	369	41.3	5.27
P23470	Receptor-type tyrosine-protein phosphatase gamma OS=Homo sapiens GN=PTPRG PE=1 SV=4 - [PTPRG_HUMAN	1.45	1	2	2	6	1.490	0.998	0.587	0.941	14.23	1.45	4	6	1445	161.9	6.42
Q5THJ4	Vacuolar protein sorting-associated protein 13D OS=Homo sapiens GN=VPS13D PE=1 SV=2 - [VP13D_HUMAN]	2.21	1	6	7	11	0.977	1.009	1.315	0.942	13.54	2.21	8	11	4388	491.6	6.58
Q8TED1	Probable glutathione peroxidase 8 OS=Homo sapiens GN=GPX8 PE=1 SV=2 - [GPX8_HUMAN]	29.19	1	5	6	21	0.880	0.768	0.746	0.942	54.26	29.19	9	21	209	23.9	9.35

Q9Y6W5	Wiskott-Aldrich syndrome protein family member 2 OS=Homo sapiens GN=WASF2 PE=1 SV=3 - [WASF2_HUMAN]	22.69	1	11	11	50	1.294	1.202	1.316	0.942	116.12	22.69	19	50	498	54.3	5.53
Q9UJF2	Ras GTPase-activating protein nGAP OS=Homo sapiens GN=RASAL2 PE=1 SV=2 - [NGAP_HUMAN]	3.86	2	2	5	11	0.856	0.502	1.558	0.942	16.14	3.86	8	11	1139	128.5	8.24
Q9Y305	Acyl-coenzyme A thioesterase 9, mitochondrial OS=Homo sapiens GN=ACOT9 PE=1 SV=2 - [ACOT9_HUMA	31.44	1	12	12	44	0.932	1.103	1.156	0.942	114.97	31.44	18	44	439	49.9	8.60
Q9BZQ6	ER degradation-enhancing alpha-mannosidase-like protein 3 OS=Homo sapiens GN=EDEM3 PE=1 SV=2 -	5.26	1	4	4	22	0.943	0.819	0.850	0.942	33.91	5.26	8	22	932	104.6	4.93
O94966	Ubiquitin carboxyl-terminal hydrolase 19 OS=Homo sapiens GN=USP19 PE=1 SV=2 - [UBP19_HUMAN]	2.73	1	2	2	6	0.707	0.455	0.440	0.942	13.05	2.73	3	6	1318	145.6	6.28
P35998	26S protease regulatory subunit 7 OS=Homo sapiens GN=PSMC2 PE=1 SV=3 - [PRS7_HUMAN]	65.13	1	30	30	167	0.853	0.943	0.939	0.942	429.62	65.13	48	167	433	48.6	5.95
Q07065	Cytoskeleton-associated protein 4 OS=Homo sapiens GN=CKAP4 PE=1 SV=2 - [CKAP4_HUMAN]	67.11	3	47	50	1328	0.781	0.784	0.925	0.942	3457.59	67.11	87	1328	602	66.0	5.92
P61313	60S ribosomal protein L15 OS=Homo sapiens GN=RPL15 PE=1 SV=2 - [RL15_HUMAN]	38.73	1	8	8	142	0.883	0.924	0.892	0.942	359.13	38.73	16	142	204	24.1	11.62

Q9H267	Vacuolar protein sorting-associated protein 33B OS=Homo sapiens GN=VPS33B PE=1 SV=2 - [VP33B_HUMAN]	19.12	1	10	10	32	1.095	0.829	0.959	0.942	85.64	19.12	16	32	617	70.5	6.71
Q9NY91	Low affinity sodium-glucose cotransporter OS=Homo sapiens GN=SLC5A4 PE=2 SV=1 - [SC5A4_HUMAN]	1.52	1	1	1	1	0.902	1.101	1.453	0.943	0.00	1.52	1	1	659	72.4	6.15
Q9Y2J4	Angiomotin-like protein 2 OS=Homo sapiens GN=AMOTL2 PE=1 SV=3 - [AMOL2_HUMAN]	5.78	1	4	5	11	0.525	0.905	0.890	0.943	17.52	5.78	6	11	779	85.7	7.30
Q8WVT3	Trafficking protein particle complex subunit 12 OS=Homo sapiens GN=TRAPPC12 PE=1 SV=3 - [TPC12_HUMAN]	15.51	1	8	8	21	1.157	1.186	0.997	0.943	32.88	15.51	13	21	735	79.3	4.91
P60900	Proteasome subunit alpha type-6 OS=Homo sapiens GN=PSMA6 PE=1 SV=1 - [PSA6_HUMAN]	53.66	1	13	13	72	0.984	0.830	0.880	0.943	202.87	53.66	21	72	246	27.4	6.76
P05387	60S acidic ribosomal protein P2 OS=Homo sapiens GN=RPLP2 PE=1 SV=1 - [RLA2_HUMAN]	75.65	1	8	9	265	0.782	0.874	0.794	0.943	707.95	75.65	15	265	115	11.7	4.54
Q8TCU4	Alstrom syndrome protein 1 OS=Homo sapiens GN=ALMS1 PE=1 SV=3 - [ALMS1_HUMAN]	0.77	1	3	3	3	1.023	0.221	1.009	0.943	8.81	0.77	3	3	4167	460.7	6.28
Q16854	Deoxyguanosine kinase, mitochondrial OS=Homo sapiens GN=DGUOK PE=1 SV=2 - [DGUOK_HUMAN]	2.53	1	1	1	1	0.891	0.966	0.732	0.943	2.82	2.53	1	1	277	32.0	8.66
O14737	Programmed cell death protein 5 OS=Homo sapiens GN=PDCD5 PE=1 SV=3 - [PDCD5_HUMAN	48.00	1	6	6	34	0.989	1.162	1.015	0.943	75.19	48.00	10	34	125	14.3	6.04

Q9C091	GREB1-like protein OS=Homo sapiens GN=GREB1L PE=2 SV=2 - [GRB1L_HUMAN]	1.98	1	2	3	4	0.988	0.779	0.934	0.943	0.00	1.98	3	4	1923	214.2	6.62
Q99460	26S proteasome non-ATPase regulatory subunit 1 OS=Homo sapiens GN=PSMD1 PE=1 SV=2 - [PSMD1_HUMA	35.36	1	30	30	153	0.890	0.904	0.932	0.943	380.50	35.36	48	153	953	105.8	5.39
Q9UHG2	ProSAAS OS=Homo sapiens GN=PCSK1N PE=1 SV=1 - [PCSK1_HUMAN]	3.85	1	1	1	1	0.746	1.149	1.007	0.943	2.91	3.85	1	1	260	27.4	6.62
P49006	MARCKS-related protein OS=Homo sapiens GN=MARCKSL1 PE=1 SV=2 - [MRP_HUMAN]	24.10	1	5	5	41	1.121	1.052	0.953	0.943	109.60	24.10	9	41	195	19.5	4.67
Q9UL46	Proteasome activator complex subunit 2 OS=Homo sapiens GN=PSME2 PE=1 SV=4 - [PSME2_HUMA N]	48.95	1	10	10	46	1.229	0.906	0.854	0.943	105.12	48.95	15	46	239	27.4	5.73
Q5T447	E3 ubiquitin-protein ligase HECTD3 OS=Homo sapiens GN=HECTD3 PE=1 SV=1 - [HECD3_HUMAN]	7.90	1	6	6	12	1.106	1.094	1.080	0.943	26.25	7.90	9	12	861	97.1	5.64
P11021	78 kDa glucose-regulated protein OS=Homo sapiens GN=HSPA5 PE=1 SV=2 - [GRP78_HUMAN]	68.96	2	57	60	2133	0.691	1.072	0.931	0.943	5306.99	68.96	108	2133	654	72.3	5.16
Q9GZT8	Putative GTP cyclohydrolase 1 type 2 NIF3L1 OS=Homo sapiens GN=NIF3L1 PE=1 SV=2 - [GTPC1_HUMAN]	13.26	1	4	4	14	1.064	0.949	0.861	0.943	34.00	13.26	7	14	377	41.9	6.65
Q9Y4P3	Transducin beta-like protein 2 OS=Homo sapiens GN=TBL2 PE=1 SV=1 - [TBL2_HUMAN]	37.81	1	18	18	69	0.909	0.862	0.835	0.944	175.29	37.81	28	69	447	49.8	9.44

P30501	HLA class I histocompatibility antigen, Cw-2 alpha chain OS=Homo sapiens GN=HLA-C PE=1 SV=1 - [1C02_HUMAN]	42.90	2	1	13	103	1.703		1.078	0.944	258.18	42.90	21	103	366	41.1	6.11
Q96NE9	FERM domain-containing protein 6 OS=Homo sapiens GN=FRMD6 PE=1 SV=1 - [FRMD6_HUMAN]	4.50	1	2	2	10	0.765	0.986	1.081	0.944	30.73	4.50	4	10	622	72.0	7.46
Q32M45	Anoctamin-4 OS=Homo sapiens GN=ANO4 PE=2 SV=1 - [ANO4_HUMAN]	2.30	1	1	2	2	1.060	1.797	2.392	0.944	4.49	2.30	2	2	955	111.4	8.18
Q9Y6V0	Protein piccolo OS=Homo sapiens GN=PCLO PE=1 SV=4 - [PCLO_HUMAN]	0.53	1	1	3	3	0.601	0.658	0.653	0.944	5.56	0.53	3	3	5065	552.9	6.51
Q14114	Low-density lipoprotein receptor-related protein 8 OS=Homo sapiens GN=LRP8 PE=1 SV=4 - [LRP8_HUMAN]	2.70	1	1	3	6	1.216	0.915	1.438	0.944	9.83	2.70	4	6	963	105.6	5.05
Q6PI26	Protein SHQ1 homolog OS=Homo sapiens GN=SHQ1 PE=1 SV=2 - [SHQ1_HUMAN]	3.29	1	2	2	4	0.976	0.871	0.704	0.944	8.57	3.29	2	4	577	65.1	4.79
Q6ZNL6	FYVE, RhoGEF and PH domain-containing protein 5 OS=Homo sapiens GN=FGD5 PE=1 SV=3 - [FGD5_HUMAN]	0.68	1	1	1	1	0.621	0.838	1.482	0.944	2.88	0.68	1	1	1462	159.8	5.01
Q8NDH3	Probable aminopeptidase NPEPL1 OS=Homo sapiens GN=NPEPL1 PE=1 SV=3 - [PEPL1_HUMAN]	17.78	1	7	7	43	1.325	1.114	1.189	0.944	93.43	17.78	10	43	523	55.8	6.87
Q8N119	Matrix metalloproteinase-21 OS=Homo sapiens GN=MMP21 PE=2 SV=2 - [MMP21_HUMAN]	2.81	1	1	1	2	2.108	0.837	1.161	0.944	4.76	2.81	1	2	569	65.0	9.11

Q9Y3A6	Transmembrane emp24 domain-containing protein 5 OS=Homo sapiens GN=TMED5 PE=1 SV=1 - [TMED5_HUMA	17.47	1	5	5	20	0.861	0.751	0.842	0.944	44.26	17.47	9	20	229	26.0	4.84
Q8IWE5	Pleckstrin homology domain-containing family M member 2 OS=Homo sapiens GN=PLEKHM2 PE=1 SV=2 - [PKHM2_HUMAN]	6.58	1	5	5	14	0.853	0.886	0.995	0.945	36.82	6.58	8	14	1019	112.7	4.93
O15020	Spectrin beta chain, non-erythrocytic 2 OS=Homo sapiens GN=SPTBN2 PE=1 SV=3 - [SPTN2_HUMAN]	7.70	1	5	20	73	0.654	4.352	3.662	0.945	147.63	7.70	30	73	2390	271.2	6.11
Q12981	Vesicle transport protein SEC20 OS=Homo sapiens GN=BNIP1 PE=1 SV=3 - [SEC20_HUMAN	25.88	1	6	6	9	1.020	1.003	0.928	0.945	16.41	25.88	7	9	228	26.1	8.95
Q9Y6Y8	SEC23-interacting protein OS=Homo sapiens GN=SEC23IP PE=1 SV=1 - [S23IP_HUMAN]	29.40	1	26	27	139	0.833	1.013	0.913	0.945	340.09	29.40	47	139	1000	111.0	5.54
Q9ULA0	Aspartyl aminopeptidase OS=Homo sapiens GN=DNPEP PE=1 SV=1 - [DNPEP_HUMAN]	34.74	1	13	13	46	1.027	0.935	0.789	0.945	115.51	34.74	21	46	475	52.4	7.42
P15056	Serine/threonine-protein kinase B-raf OS=Homo sapiens GN=BRAF PE=1 SV=4 - [BRAF_HUMAN]	2.61	1	1	2	5	1.232	0.665	0.765	0.945	12.56	2.61	4	5	766	84.4	7.53
Q92609	TBC1 domain family member 5 OS=Homo sapiens GN=TBC1D5 PE=1 SV=1 - [TBCD5_HUMAN]	13.96	1	8	8	24	0.810	1.065	1.009	0.945	70.18	13.96	10	24	795	88.9	6.54
Q8IZ81	ELMO domain-containing protein 2 OS=Homo sapiens GN=ELMOD2 PE=1 SV=1 - [ELMD2_HUMAN]	17.06	1	5	5	13	1.025	0.799	0.833	0.945	28.23	17.06	7	13	293	34.9	8.05

P28288	ATP-binding cassette sub-family D member 3 OS=Homo sapiens GN=ABCD3 PE=1 SV=1 - [ABCD3_HUMAN]	28.22	4	20	22	88	1.088	0.723	0.954	0.945	213.19	28.22	34	88	659	75.4	9.36
Q9BR76	Coronin-1B OS=Homo sapiens GN=CORO1B PE=1 SV=1 - [COR1B_HUMAN]	37.01	2	15	15	83	1.005	0.941	1.054	0.945	197.31	37.01	26	83	489	54.2	5.88
Q9NQP4	Prefoldin subunit 4 OS=Homo sapiens GN=PFDN4 PE=1 SV=1 - [PFD4_HUMAN]	47.01	1	7	7	85	0.807	1.114	1.049	0.945	244.28	47.01	10	85	134	15.3	4.53
Q5S007	Leucine-rich repeat serine/threonine-protein kinase 2 OS=Homo sapiens GN=LRRK2 PE=1 SV=2 - [LRRK2_HUMAN	2.22	1	6	7	12	0.862	0.341	0.500	0.946	17.63	2.22	8	12	2527	285.9	6.80
P21281	V-type proton ATPase subunit B, brain isoform OS=Homo sapiens GN=ATP6V1B2 PE=1 SV=3 - [VATB2_HUMAN	42.86	2	17	17	60	0.979	0.998	0.871	0.946	153.78	42.86	30	60	511	56.5	5.81
Q9UN86	Ras GTPase-activating protein-binding protein 2 OS=Homo sapiens GN=G3BP2 PE=1 SV=2 - [G3BP2_HUMAN]	24.27	1	11	13	58	0.952	0.953	0.985	0.946	118.60	24.27	20	58	482	54.1	5.55
Q8WXH2	Junctophilin-3 OS=Homo sapiens GN=JPH3 PE=1 SV=2 - [JPH3_HUMAN]	0.80	1	1	1	2	0.880	0.717	0.718	0.946	4.33	0.80	1	2	748	81.4	9.39
P20337	Ras-related protein Rab-3B OS=Homo sapiens GN=RAB3B PE=1 SV=2 - [RAB3B_HUMAN]	48.86	5	5	10	126	0.838	0.744	0.820	0.946	302.70	48.86	18	126	219	24.7	5.02
Q8IWY9	Codanin-1 OS=Homo sapiens GN=CDAN1 PE=1 SV=4 - [CDAN1_HUMAN]	1.79	1	2	2	9	0.849	0.801	1.028	0.946	0.00	1.79	2	9	1227	134.0	6.77

Q9HCP6	Protein-cysteine N-palmitoyltransferase HHAT-like protein OS=Homo sapiens GN=HHATL PE=2 SV=1 - [HHATL_HUMAN]	7.14	1	2	2	5	1.197	0.169	0.240	0.946	6.58	7.14	2	5	504	56.7	7.12
Q9H694	Protein bicaudal C homolog 1 OS=Homo sapiens GN=BICC1 PE=1 SV=2 - [BICC1_HUMAN	6.67	1	5	5	12	0.649	0.859	0.765	0.946	34.23	6.67	8	12	974	104.8	8.54
P63000	Ras-related C3 botulinum toxin substrate 1 OS=Homo sapiens GN=RAC1 PE=1 SV=1 - [RAC1_HUMAN]	44.79	3	6	11	95	1.477	1.015	1.028	0.946	244.71	44.79	20	95	192	21.4	8.50
Q9Y5S9	RNA-binding protein 8A OS=Homo sapiens GN=RBM8A PE=1 SV=1 - [RBM8A_HUMAN]	16.09	1	4	4	14	0.893	0.784	0.636	0.946	35.57	16.09	7	14	174	19.9	5.72
Q5VW36	Focadhesin OS=Homo sapiens GN=FOCAD PE=1 SV=1 - [FOCAD_HUMAN]	7.94	1	13	14	25	0.920	0.915	1.046	0.947	58.12	7.94	19	25	1801	199.9	6.62
O75030	Microphthalmia-associated transcription factor OS=Homo sapiens GN=MITF PE=1 SV=2 - [MITF_HUMAN]	11.03	3	3	5	8	0.689	1.634	1.081	0.947	12.76	11.03	7	8	526	58.8	6.33
Q8N543	Prolyl 3-hydroxylase OGFOD1 OS=Homo sapiens GN=OGFOD1 PE=1 SV=1 - [OGFD1_HUMAN]	3.32	1	1	1	1	1.064	0.960	1.326	0.947	4.46	3.32	1	1	542	63.2	5.11
Q7L3B6	Hsp90 co-chaperone Cdc37-like 1 OS=Homo sapiens GN=CDC37L1 PE=1 SV=1 - [CD37L_HUMAN]	14.24	1	4	4	5	0.719	0.979	1.149	0.947	9.13	14.24	5	5	337	38.8	5.34
O75864	Protein phosphatase 1 regulatory subunit 37 OS=Homo sapiens GN=PPP1R37 PE=1 SV=4 - [PPR37_HUMAN]	2.17	1	2	2	2	1.008	1.092	1.274	0.947	2.41	2.17	2	2	691	74.7	5.06

Q5W0Z9	Probable palmitoyltransferase ZDHHC20 OS=Homo sapiens GN=ZDHHC20 PE=1 SV=1 - [ZDH20_HUMAN]	3.01	1	1	1	3	1.140	0.664	0.598	0.947	8.87	3.01	2	3	365	42.2	7.71
Q8IZY2	ATP-binding cassette sub-family A member 7 OS=Homo sapiens GN=ABCA7 PE=1 SV=3 - [ABCA7_HUMAN]	2.00	1	2	2	2	0.649	0.963	1.123	0.947	2.74	2.00	2	2	2146	234.2	7.24
Q9Y2K2	Serine/threonine-protein kinase SIK3 OS=Homo sapiens GN=SIK3 PE=1 SV=3 - [SIK3_HUMAN]	0.55	1	1	1	2	0.374	0.983	0.927	0.947	4.11	0.55	1	2	1263	139.9	6.70
Q9H4L7	SWI/SNF-related matrix-associated actin-dependent regulator of chromatin subfamily A containing DEAD/H box 1 OS=Homo sapiens GN=SMARCAD1 PE=1 SV=2 - [SMRCD_HUMAN]	0.78	1	1	1	1	1.696	0.881	1.434	0.947	2.09	0.78	1	1	1026	117.3	5.55
Q9NY59	Sphingomyelin phosphodiesterase 3 OS=Homo sapiens GN=SMPD3 PE=1 SV=1 - [NSMA2_HUMAN]	3.66	1	2	2	2	0.865	0.716	0.850	0.947	2.45	3.66	2	2	655	71.0	5.78
Q8NBJ4	Golgi membrane protein 1 OS=Homo sapiens GN=GOLM1 PE=1 SV=1 - [GOLM1_HUMAN]	23.44	2	9	10	27	0.618	1.195	0.837	0.948	55.00	23.44	15	27	401	45.3	4.97
Q9H3F6	BTB/POZ domain-containing adapter for CUL3-mediated RhoA degradation protein 3 OS=Homo sapiens GN=KCTD10 PE=1 SV=1 - [BACD3_HUMAN]	14.70	3	4	4	9	0.978	0.638	0.729	0.948	18.27	14.70	6	9	313	35.4	6.34

Q8TAT6	Nuclear protein localization protein 4 homolog OS=Homo sapiens GN=NPLOC4 PE=1 SV=3 - [NPL4_HUMAN]	28.78	1	16	17	47	1.241	0.899	1.461	0.948	129.77	28.78	27	47	608	68.1	6.38
Q8N4A0	Polypeptide N-acetylgalactosaminyltransferase 4 OS=Homo sapiens GN=GALNT4 PE=1 SV=2 - [GALT4_HUMAN]	10.21	1	4	6	15	0.945	0.849	1.281	0.948	34.96	10.21	8	15	578	66.6	7.61
P01130	Low-density lipoprotein receptor OS=Homo sapiens GN=LDLR PE=1 SV=1 - [LDLR_HUMAN]	10.35	1	7	8	21	0.766	0.643	1.941	0.948	40.38	10.35	13	21	860	95.3	5.05
Q9UBU8	Mortality factor 4-like protein 1 OS=Homo sapiens GN=MORF4L1 PE=1 SV=2 - [MO4L1_HUMAN]	4.97	1	2	2	3	0.849	0.996	0.990	0.948	8.52	4.97	2	3	362	41.4	9.28
Q9H492	Microtubule-associated proteins 1A/1B light chain 3A OS=Homo sapiens GN=MAP1LC3A PE=1 SV=2 - [MLP3A_HUMAN]	24.79	1	1	4	10	0.573	0.896	1.334	0.948	6.32	24.79	7	10	121	14.3	8.68
Q86TB9	Protein PAT1 homolog 1 OS=Homo sapiens GN=PATL1 PE=1 SV=2 - [PATL1_HUMAN]	6.36	1	4	4	8	1.647	1.299	1.187	0.948	13.37	6.36	6	8	770	86.8	6.67
A6NDG6	Phosphoglycolate phosphatase OS=Homo sapiens GN=PGP PE=1 SV=1 - [PGP_HUMAN]	27.10	1	8	8	18	1.178	1.186	1.057	0.948	39.90	27.10	14	18	321	34.0	6.14
Q9NR31	GTP-binding protein SAR1a OS=Homo sapiens GN=SAR1A PE=1 SV=1 - [SAR1A_HUMAN]	35.86	1	4	7	37	1.227	0.698	1.076	0.948	102.70	35.86	12	37	198	22.4	6.68
Q8NC54	Keratinocyte-associated transmembrane protein 2 OS=Homo sapiens GN=KCT2 PE=2 SV=2 - [KCT2_HUMAN]	3.77	1	1	1	3	1.046	0.628	0.485	0.948	7.44	3.77	2	3	265	29.2	5.08

Q15654	Thyroid receptor-interacting protein 6 OS=Homo sapiens GN=TRIP6 PE=1 SV=3 - [TRIP6_HUMAN]	23.95	1	7	7	38	1.136	1.047	1.102	0.948	147.17	23.95	13	38	476	50.3	7.37
Q9H098	Protein FAM107B OS=Homo sapiens GN=FAM107B PE=1 SV=1 - [F107B_HUMAN]	24.43	1	3	3	9	1.672	0.504	1.217	0.948	16.92	24.43	5	9	131	15.5	8.29
Q9H1E5	Thioredoxin-related transmembrane protein 4 OS=Homo sapiens GN=TMX4 PE=1 SV=1 - [TMX4_HUMAN]	3.44	1	1	1	4	1.300	1.248	1.217	0.948	8.41	3.44	2	4	349	38.9	4.37
P22314	Ubiquitin-like modifier-activating enzyme 1 OS=Homo sapiens GN=UBA1 PE=1 SV=3 - [UBA1_HUMAN]	48.58	1	42	43	539	1.017	1.046	1.096	0.949	1329.76	48.58	74	539	1058	117.8	5.76
Q96QR8	Transcriptional activator protein Pur-beta OS=Homo sapiens GN=PURB PE=1 SV=3 - [PURB_HUMAN]	25.00	2	6	9	27	1.140	0.886	0.842	0.949	74.69	25.00	13	27	312	33.2	5.43
Q7Z3C6	Autophagy-related protein 9A OS=Homo sapiens GN=ATG9A PE=1 SV=3 - [ATG9A_HUMAN]	5.72	1	5	5	7	1.110	0.842	0.870	0.949	13.74	5.72	6	7	839	94.4	6.67
Q9H910	Hematological and neurological expressed 1-like protein OS=Homo sapiens GN=HN1L PE=1 SV=1 - [HN1L_HUMAN]	52.63	1	7	7	33	0.967	1.276	1.171	0.949	91.88	52.63	13	33	190	20.1	9.26
Q8WTT2	Nucleolar complex protein 3 homolog OS=Homo sapiens GN=NOC3L PE=1 SV=1 - [NOC3L_HUMAN	11.88	1	10	10	24	0.714	0.953	0.798	0.949	62.60	11.88	16	24	800	92.5	9.17

P27037	Activin receptor type-2A OS=Homo sapiens GN=ACVR2A PE=1 SV=1 - [AVR2A_HUMAN]	3.12	1	1	1	1	0.691	1.338	1.884	0.949	0.00	3.12	1	1	513	57.8	5.92
O95373	Importin-7 OS=Homo sapiens GN=IPO7 PE=1 SV=1 - [IPO7_HUMAN]	13.49	1	12	12	57	0.809	0.742	0.884	0.949	115.54	13.49	22	57	1038	119.4	4.82
Q9NWS0	PIH1 domain-containing protein 1 OS=Homo sapiens GN=PIH1D1 PE=1 SV=1 - [PIHD1_HUMAN]	21.03	1	4	4	15	0.835	1.183	1.109	0.949	40.39	21.03	7	15	290	32.3	5.14
P50991	T-complex protein 1 subunit delta OS=Homo sapiens GN=CCT4 PE=1 SV=4 - [TCPD_HUMAN]	61.78	1	30	31	224	0.911	0.879	1.011	0.949	544.06	61.78	52	224	539	57.9	7.83
Q16134	Electron transfer flavoprotein-ubiquinone oxidoreductase, mitochondrial OS=Homo sapiens GN=ETFDH PE=1 SV=2 - [ETFD_HUMAN]	23.18	1	13	13	52	0.737	0.985	0.989	0.949	134.40	23.18	22	52	617	68.5	7.55
Q9Y239	Nucleotide-binding oligomerization domain-containing protein 1 OS=Homo sapiens GN=NOD1 PE=1 SV=1 - [NOD1_HUMAN]	0.73	1	1	1	1	1.667	1.707	1.182	0.949	0.00	0.73	1	1	953	107.6	7.11
P10606	Cytochrome c oxidase subunit 5B, mitochondrial OS=Homo sapiens GN=COX5B PE=1 SV=2 - [COX5B_HUMAN]	51.16	1	9	9	56	0.930	1.105	0.920	0.949	129.11	51.16	16	56	129	13.7	8.81
O60739	Eukaryotic translation initiation factor 1b OS=Homo sapiens GN=EIF1B PE=1 SV=2 - [EIF1B_HUMAN]	51.33	2	6	6	35	0.843	0.911	1.175	0.949	130.40	51.33	7	35	113	12.8	7.37

Q9BXT5	Testis-expressed sequence 15 protein OS=Homo sapiens GN=TEX15 PE=2 SV=2 - [TEX15_HUMAN	3.41	2	3	6	14	1.306	0.491	1.784	0.949	21.80	3.41	7	14	2789	315.1	6.19
Q13617	Cullin-2 OS=Homo sapiens GN=CUL2 PE=1 SV=2 - [CUL2_HUMAN]	18.52	1	14	15	34	1.058	0.943	1.034	0.949	71.35	18.52	20	34	745	86.9	6.92
P32929	Cystathionine gamma-lyase OS=Homo sapiens GN=CTH PE=1 SV=3 - [CGL_HUMAN]	2.22	1	1	1	1	0.831	1.783	2.132	0.950	2.26	2.22	1	1	405	44.5	6.70
P50402	Emerin OS=Homo sapiens GN=EMD PE=1 SV=1 - [EMD_HUMAN]	42.52	1	10	10	61	0.933	1.279	1.283	0.950	146.75	42.52	18	61	254	29.0	5.50
Q9NVR0	Kelch-like protein 11 OS=Homo sapiens GN=KLHL11 PE=1 SV=1 - [KLH11_HUMAN]	1.13	1	1	1	1	0.736	1.037	0.900	0.950	1.88	1.13	1	1	708	80.1	6.16
Q8NFK1	Gap junction gamma-3 protein OS=Homo sapiens GN=GJC3 PE=2 SV=1 - [CXG3_HUMAN]	6.09	1	1	2	2	0.488	0.563	1.050	0.950	3.82	6.09	2	2	279	31.3	9.17
Q4G148	Glucoside xylosyltransfera se 1 OS=Homo sapiens GN=GXYLT1 PE=1 SV=2 - [GXLT1_HUMAN]	7.95	1	3	3	10	0.708	0.877	0.662	0.950	16.78	7.95	6	10	440	50.5	8.65
Q9NRM1	Enamelin OS=Homo sapiens GN=ENAM PE=2 SV=3 - [ENAM_HUMAN]	0.96	1	1	1	2	0.531	3.093	5.997	0.950	5.98	0.96	1	2	1142	128.7	6.83
Q8NGP4	Olfactory receptor 5M3 OS=Homo sapiens GN=OR5M3 PE=2 SV=2 - [OR5M3_HUMA N]	2.93	1	1	1	1	1.222	1.384	0.950	0.950	2.26	2.93	1	1	307	35.1	9.03
Q8WUB8	PHD finger protein 10 OS=Homo sapiens GN=PHF10 PE=1 SV=3 - [PHF10_HUMAN]	4.62	1	2	2	5	1.156	1.043	0.478	0.950	16.76	4.62	3	5	498	56.0	6.62

Q8TCD5	5'(3')-deoxyribonucleotidase, cytosolic type OS=Homo sapiens GN=NT5C PE=1 SV=2 - [NT5C_HUMAN]	50.75	1	8	8	20	1.477	0.912	1.064	0.950	51.82	50.75	13	20	201	23.4	6.64
P50416	Carnitine O-palmitoyltransferase 1, liver isoform OS=Homo sapiens GN=CPT1A PE=1 SV=2 - [CPT1A_HUMAN]	24.45	1	17	17	88	1.021	1.017	0.923	0.950	231.66	24.45	24	88	773	88.3	8.65
Q9H6R3	Acyl-CoA synthetase short-chain family member 3, mitochondrial OS=Homo sapiens GN=ACSS3 PE=1 SV=1 - [ACSS3_HUMAN	12.97	1	8	8	16	0.657	0.828	0.564	0.950	40.71	12.97	13	16	686	74.7	8.63
P40925	Malate dehydrogenase, cytoplasmic OS=Homo sapiens GN=MDH1 PE=1 SV=4 - [MDHC_HUMAN]	56.29	1	22	23	262	1.100	1.175	0.971	0.950	558.19	56.29	37	262	334	36.4	7.36
Q8IYS2	Uncharacterized protein KIAA2013 OS=Homo sapiens GN=KIAA2013 PE=2 SV=1 - [K2013_HUMAN]	14.04	1	6	6	12	0.830	0.949	0.913	0.951	37.07	14.04	10	12	634	69.1	8.19
Q5MJ10	Sperm protein associated with the nucleus on the X chromosome N2 OS=Homo sapiens GN=SPANXN2 PE=2 SV=1 - [SPXN2_HUMAN]	4.44	1	1	1	2	0.414	0.656	0.810	0.951	1.63	4.44	1	2	180	19.9	3.98
Q96RU3	Formin-binding protein 1 OS=Homo sapiens GN=FNBP1 PE=1 SV=2 - [FNBP1_HUMAN]	20.58	1	14	14	43	1.188	1.171	1.179	0.951	95.92	20.58	20	43	617	71.3	5.72
Q6ZRV2	Protein FAM83H OS=Homo sapiens GN=FAM83H PE=1 SV=3 - [FA83H_HUMAN]	3.39	1	2	3	3	1.145	0.927	1.439	0.951	5.09	3.39	3	3	1179	127.0	6.98

Q8TBH0	Arrestin domain-containing protein 2 OS=Homo sapiens GN=ARRDC2 PE=2 SV=2 - [ARRD2_HUMAN	11.55	1	2	2	2	3.101	1.861	1.502	0.951	5.25	11.55	2	2	407	44.4	9.20
Q68D91	Metallo-beta-lactamase domain-containing protein 2 OS=Homo sapiens GN=MBLAC2 PE=1 SV=3 - [MBLC2_HUMAN]	27.24	1	6	6	17	0.856	0.751	0.918	0.951	43.25	27.24	9	17	279	31.4	6.92
A4D2H0	cTAGE family member 15 OS=Homo sapiens GN=CTAGE15 PE=2 SV=1 - [CTGEF_HUMAN]	2.45	5	1	2	9	0.676	0.525	0.848	0.952	15.12	2.45	2	9	777	87.8	5.24
Q8N122	Regulatory-associated protein of mTOR OS=Homo sapiens GN=RPTOR PE=1 SV=1 - [RPTOR_HUMA N]	7.79	1	9	9	19	0.886	1.027	1.017	0.952	39.52	7.79	13	19	1335	148.9	6.89
P09496	Clathrin light chain A OS=Homo sapiens GN=CLTA PE=1 SV=1 - [CLCA_HUMAN]	27.42	1	11	12	133	0.983	0.971	0.948	0.952	276.20	27.42	18	133	248	27.1	4.51
Q53GT1	Kelch-like protein 22 OS=Homo sapiens GN=KLHL22 PE=1 SV=2 - [KLH22_HUMAN]	2.37	1	1	1	2	0.688	0.936	0.990	0.952	5.68	2.37	1	2	634	71.6	5.49
Q9BU61	NADH dehydrogenase [ubiquinone] 1 alpha subcomplex assembly factor 3 OS=Homo sapiens GN=NDUFAF3 PE=1 SV=1 - [NDUF3_HUMAN]	18.48	1	3	3	20	1.052	0.798	1.054	0.952	49.82	18.48	5	20	184	20.3	8.22
P63220	40S ribosomal protein S21 OS=Homo sapiens GN=RPS21 PE=1 SV=1 - [RS21_HUMAN]	50.60	1	6	6	77	0.799	0.969	0.900	0.952	164.68	50.60	9	77	83	9.1	8.50

Q9NQC7	Ubiquitin carboxyl-terminal hydrolase CYLD OS=Homo sapiens GN=CYLD PE=1 SV=1 - [CYLD_HUMAN]	1.15	1	1	1	2	0.831	1.021	1.661	0.952	2.54	1.15	2	2	956	107.2	5.59
O75179	Ankyrin repeat domain-containing protein 17 OS=Homo sapiens GN=ANKRD17 PE=1 SV=3 - [ANR17_HUMAN]	5.95	1	8	12	23	0.607	0.977	1.121	0.952	48.92	5.95	17	23	2603	274.1	6.52
Q9HD42	Charged multivesicular body protein 1a OS=Homo sapiens GN=CHMP1A PE=1 SV=1 - [CHM1A_HUMAN]	23.47	1	6	6	22	0.884	0.927	1.144	0.952	45.90	23.47	11	22	196	21.7	8.06
O60507	Protein-tyrosine sulfotransferase 1 OS=Homo sapiens GN=TPST1 PE=2 SV=1 - [TPST1_HUMAN	7.03	1	2	2	6	1.424	0.920	0.644	0.953	5.87	7.03	4	6	370	42.2	9.09
P19525	Interferon-induced, double-stranded RNA-activated protein kinase OS=Homo sapiens GN=EIF2AK2 PE=1 SV=2 - [E2AK2_HUMAN	20.69	1	11	11	32	1.942	1.019	0.989	0.953	75.30	20.69	18	32	551	62.1	8.40
Q96BA8	Cyclic AMP-responsive element-binding protein 3-like protein 1 OS=Homo sapiens GN=CREB3L1 PE=1 SV=1 - [CR3L1_HUMAN]	3.08	1	2	2	5	0.496	0.533	0.615	0.953	5.76	3.08	4	5	519	57.0	5.17
Q02763	Angiopoietin-1 receptor OS=Homo sapiens GN=TEK PE=1 SV=2 - [TIE2_HUMAN]	2.22	1	3	3	3	3.219	0.200	0.528	0.953	3.79	2.22	3	3	1124	125.7	6.89
Q53FT3	Protein Hikeshi OS=Homo sapiens GN=C11orf73 PE=1 SV=2 - [HIKES_HUMAN]	8.12	1	1	1	4	1.339	1.337	1.170	0.953	8.17	8.12	2	4	197	21.6	5.45

O15021	Microtubule-associated serine/threonine-protein kinase 4 OS=Homo sapiens GN=MAST4 PE=1 SV=3 - [MAST4_HUMA	1.75	1	3	3	4	0.950	2.311	1.551	0.953	2.65	1.75	4	4	2626	284.2	8.62
P40222	Alpha-taxilin OS=Homo sapiens GN=TXLNA PE=1 SV=3 - [TXLNA_HUMAN]	50.73	2	22	23	87	0.918	1.091	0.999	0.953	225.28	50.73	38	87	546	61.9	6.52
P84101	Small EDRK-rich factor 2 OS=Homo sapiens GN=SERF2 PE=1 SV=1 - [SERF2_HUMAN]	13.56	1	1	1	15	0.723	1.098	0.904	0.953	41.01	13.56	2	15	59	6.9	10.45
Q6RFH5	WD repeat-containing protein 74 OS=Homo sapiens GN=WDR74 PE=1 SV=1 - [WDR74_HUMA N]	7.01	1	2	2	2	1.006	0.767	0.699	0.953	6.19	7.01	2	2	385	42.4	8.32
P68104	Elongation factor 1-alpha 1 OS=Homo sapiens GN=EEF1A1 PE=1 SV=1 - [EF1A1_HUMAN]	71.65	2	18	33	807	0.838	0.840	0.908	0.953	2053.72	71.65	51	807	462	50.1	9.01
P16152	Carbonyl reductase [NADPH] 1 OS=Homo sapiens GN=CBR1 PE=1 SV=3 - [CBR1_HUMAN]	62.45	1	11	14	206	1.139	0.974	0.990	0.953	666.49	62.45	24	206	277	30.4	8.32
Q9P260	LisH domain and HEAT repeat-containing protein KIAA1468 OS=Homo sapiens GN=KIAA1468 PE=1 SV=2 - [K1468_HUMAN]	9.46	1	12	12	22	1.042	1.000	0.992	0.953	48.66	9.46	17	22	1216	134.5	5.45
Q7Z2Y8	Interferon-induced very large GTPase 1 OS=Homo sapiens GN=GVINP1 PE=2 SV=2 - [GVIN1_HUMAN]	2.27	1	4	4	4	0.619	1.126	1.124	0.953	2.27	2.27	4	4	2422	278.9	6.55
Q8WXD0	Relaxin receptor 2 OS=Homo sapiens GN=RXFP2 PE=1 SV=1 - [RXFP2_HUMAN	1.99	1	1	1	1	0.857	2.370	3.885	0.953	0.00	1.99	1	1	754	86.4	8.76

P09497	Clathrin light chain B OS=Homo sapiens GN=CLTB PE=1 SV=1 - [CLCB_HUMAN]	25.76	1	10	10	103	1.266	0.913	1.126	0.953	221.26	25.76	19	103	229	25.2	4.64
Q6ZMG9	Ceramide synthase 6 OS=Homo sapiens GN=CERS6 PE=1 SV=1 - [CERS6_HUMAN]	4.95	2	2	2	3	1.070	1.182	0.966	0.953	5.39	4.95	3	3	384	44.9	7.65
Q709C8	Vacuolar protein sorting-associated protein 13C OS=Homo sapiens GN=VPS13C PE=1 SV=1 - [VP13C_HUMAN]	6.58	2	22	22	40	1.201	0.889	0.965	0.953	80.51	6.58	29	40	3753	422.1	6.83
O00506	Serine/threonine-protein kinase 25 OS=Homo sapiens GN=STK25 PE=1 SV=1 - [STK25_HUMAN	22.77	1	6	9	30	1.278	0.971	1.035	0.954	88.55	22.77	13	30	426	48.1	6.74
Q9NR19	Acetyl-coenzyme A synthetase, cytoplasmic OS=Homo sapiens GN=ACSS2 PE=1 SV=1 - [ACSA_HUMAN]	16.26	1	10	10	28	1.350	0.808	1.058	0.954	58.90	16.26	15	28	701	78.5	6.46
O95167	NADH dehydrogenase [ubiquinone] 1 alpha subcomplex subunit 3 OS=Homo sapiens GN=NDUFA3 PE=1 SV=1 - [NDUA3_HUMAN]	57.14	1	4	4	10	0.639	0.902	0.680	0.954	23.66	57.14	6	10	84	9.3	8.46
Q96JA1	Leucine-rich repeats and immunoglobulin-like domains protein 1 OS=Homo sapiens GN=LRIG1 PE=1 SV=2 - [LRIG1_HUMAN]	1.92	3	2	2	4	0.473	1.477	1.445	0.954	7.31	1.92	3	4	1093	119.0	7.09
Q9UBB4	Ataxin-10 OS=Homo sapiens GN=ATXN10 PE=1 SV=1 - [ATX10_HUMAN]	32.21	1	16	16	61	0.793	0.903	1.195	0.954	134.02	32.21	26	61	475	53.5	5.25

Q14534	Squalene monooxygenase OS=Homo sapiens GN=SQLE PE=1 SV=3 - [ERG1_HUMAN]	4.01	1	2	2	3	0.989	0.560	1.126	0.954	8.32	4.01	2	3	574	63.9	8.63
Q96RT1	Protein LAP2 OS=Homo sapiens GN=ERBB2IP PE=1 SV=2 - [LAP2_HUMAN]	12.68	2	15	16	41	0.880	1.064	1.081	0.954	79.90	12.68	25	41	1412	158.2	5.50
O95861	3'(2'),5'-bisphosphate nucleotidase 1 OS=Homo sapiens GN=BPNT1 PE=1 SV=1 - [BPNT1_HUMAN]	33.12	1	10	10	27	1.061	0.958	0.965	0.954	68.81	33.12	17	27	308	33.4	5.69
A2RRP1	Neuroblastoma-amplified sequence OS=Homo sapiens GN=NBAS PE=1 SV=2 - [NBAS_HUMAN]	7.76	1	17	17	40	0.836	0.796	0.950	0.954	93.33	7.76	25	40	2371	268.4	5.96
Q9Y316	Protein MEMO1 OS=Homo sapiens GN=MEMO1 PE=1 SV=1 - [MEMO1_HUMA	19.19	1	4	4	17	0.902	1.007	0.843	0.954	45.02	19.19	7	17	297	33.7	7.14
Q96T68	Histone-lysine N-methyltransferase SETDB2 OS=Homo sapiens GN=SETDB2 PE=1 SV=2 - [SETB2_HUMAN]	0.97	1	1	1	1	1.013	1.299	0.505	0.954	1.95	0.97	1	1	719	81.8	7.50
O15357	Phosphatidylinositol 3,4,5-trisphosphate 5-phosphatase 2 OS=Homo sapiens GN=INPPL1 PE=1 SV=2 - [SHIP2_HUMAN]	16.69	1	17	17	37	1.071	0.898	1.053	0.954	69.66	16.69	25	37	1258	138.5	6.54
P11234	Ras-related protein Ral-B OS=Homo sapiens GN=RALB PE=1 SV=1 - [RALB_HUMAN]	48.54	1	4	7	22	1.705	1.547	1.113	0.954	51.27	48.54	10	22	206	23.4	6.62
P30153	Serine/threonine-protein phosphatase 2A 65 kDa regulatory subunit A alpha isoform OS=Homo sapiens GN=PPP2R1A PE=1 SV=4 - [2AAA_HUMAN]	48.05	1	20	25	198	1.045	1.072	1.099	0.954	439.22	48.05	42	198	589	65.3	5.11

Q9Y2G8	DnaJ homolog subfamily C member 16 OS=Homo sapiens GN=DNAJC16 PE=2 SV=3 - [DJC16_HUMAN]	4.48	2	4	4	9	1.144	0.992	1.081	0.954	17.46	4.48	6	9	782	90.5	7.12
P45954	Short/branched chain specific acyl-CoA dehydrogenase, mitochondrial OS=Homo sapiens GN=ACADSB PE=1 SV=1 - [ACDSB_HUMAN]	25.69	1	8	8	28	1.063	1.427	0.995	0.954	54.58	25.69	14	28	432	47.5	6.99
P28066	Proteasome subunit alpha type-5 OS=Homo sapiens GN=PSMA5 PE=1 SV=3 - [PSA5_HUMAN]	54.77	1	10	10	139	1.072	1.001	1.014	0.954	381.18	54.77	16	139	241	26.4	4.79
Q9ULV3	Cip1-interacting zinc finger protein OS=Homo sapiens GN=CIZ1 PE=1 SV=2 - [CIZ1_HUMAN]	5.01	1	3	3	4	0.876	0.865	0.905	0.954	6.38	5.01	4	4	898	100.0	6.11
Q05823	2-5A-dependent ribonuclease OS=Homo sapiens GN=RNASEL PE=1 SV=2 - [RN5A_HUMAN]	7.96	5	6	7	12	1.146	0.826	1.005	0.955	30.65	7.96	9	12	741	83.5	6.65
P07237	Protein disulfide-isomerase OS=Homo sapiens GN=P4HB PE=1 SV=3 - [PDIA1_HUMAN	73.23	1	47	48	1521	0.870	0.945	1.056	0.955	3999.59	73.23	82	1521	508	57.1	4.87
Q15813	Tubulin-specific chaperone E OS=Homo sapiens GN=TBCE PE=1 SV=1 - [TBCE_HUMAN]	23.53	1	10	13	39	0.891	1.254	1.196	0.955	71.02	23.53	20	39	527	59.3	6.76
Q96S59	Ran-binding protein 9 OS=Homo sapiens GN=RANBP9 PE=1 SV=1 - [RANB9_HUMAN]	9.05	1	5	6	19	0.726	0.784	1.201	0.955	41.54	9.05	11	19	729	77.8	6.79
Q6P1N9	Putative deoxyribonuclease TATDN1 OS=Homo sapiens GN=TATDN1 PE=1 SV=2 - [TATD1_HUMAN]	26.94	1	8	9	26	0.803	0.993	1.108	0.955	58.60	26.94	16	26	297	33.6	6.96

Q96K76	Ubiquitin carboxyl-terminal hydrolase 47 OS=Homo sapiens GN=USP47 PE=1 SV=3 - [UBP47_HUMAN]	12.80	1	15	16	44	1.015	0.996	0.884	0.955	116.40	12.80	24	44	1375	157.2	5.08
P08069	Insulin-like growth factor 1 receptor OS=Homo sapiens GN=IGF1R PE=1 SV=1 - [IGF1R_HUMAN]	3.29	1	4	4	6	0.992	0.861	0.948	0.955	13.78	3.29	5	6	1367	154.7	5.80
Q9Y4P1	Cysteine protease ATG4B OS=Homo sapiens GN=ATG4B PE=1 SV=2 - [ATG4B_HUMAN]	12.21	1	3	3	5	0.513	0.886	0.837	0.955	15.17	12.21	4	5	393	44.3	5.07
O75915	PRA1 family protein 3 OS=Homo sapiens GN=ARL6IP5 PE=1 SV=1 - [PRAF3_HUMAN]	28.72	1	7	7	65	0.963	1.330	1.177	0.955	215.63	28.72	11	65	188	21.6	9.77
Q9UJX2	Cell division cycle protein 23 homolog OS=Homo sapiens GN=CDC23 PE=1 SV=3 - [CDC23_HUMAN]	1.51	1	1	1	3	0.404	1.275	2.514	0.956	0.00	1.51	2	3	597	68.8	7.02
P62701	40S ribosomal protein S4, X isoform OS=Homo sapiens GN=RPS4X PE=1 SV=2 - [RS4X_HUMAN]	56.27	1	13	20	98	0.888	0.961	0.879	0.956	235.51	56.27	32	98	263	29.6	10.15
Q00610	Clathrin heavy chain 1 OS=Homo sapiens GN=CLTC PE=1 SV=5 - [CLH1_HUMAN]	50.75	1	58	77	1230	1.066	0.911	1.038	0.956	3081.85	50.75	132	1230	1675	191.5	5.69
Q8IVD9	NudC domain-containing protein 3 OS=Homo sapiens GN=NUDCD3 PE=1 SV=3 - [NUDC3_HUMAN]	14.13	1	6	7	15	0.882	1.195	1.073	0.956	28.27	14.13	9	15	361	40.8	5.25
Q6UX98	Probable palmitoyltransferase ZDHHC24 OS=Homo sapiens GN=ZDHHC24 PE=2 SV=1 - [ZDH24_HUMAN]	2.46	1	1	1	1	0.845	0.615	0.547	0.956	1.83	2.46	1	1	284	30.2	8.59

Q8NBJ5	Procollagen galactosyltransferase 1 OS=Homo sapiens GN=COLGALT1 PE=1 SV=1 - [GT251_HUMAN]	33.76	1	21	22	95	0.706	0.848	1.019	0.956	220.11	33.76	38	95	622	71.6	7.31
Q9Y5K5	Ubiquitin carboxyl-terminal hydrolase isozyme L5 OS=Homo sapiens GN=UCHL5 PE=1 SV=3 - [UCHL5_HUMAN]	30.70	2	8	9	31	0.797	0.997	0.836	0.956	70.67	30.70	14	31	329	37.6	5.33
Q53GG5	PDZ and LIM domain protein 3 OS=Homo sapiens GN=PDLIM3 PE=1 SV=1 - [PDLI3_HUMAN]	18.13	1	5	5	23	1.128	3.527	5.195	0.956	68.58	18.13	8	23	364	39.2	6.89
P67775	Serine/threonine-protein phosphatase 2A catalytic subunit alpha isoform OS=Homo sapiens GN=PPP2CA PE=1 SV=1 - [PP2AA_HUMAN	45.31	2	12	12	77	1.010	1.094	1.041	0.956	173.57	45.31	19	77	309	35.6	5.54
Q9H444	Charged multivesicular body protein 4b OS=Homo sapiens GN=CHMP4B PE=1 SV=1 - [CHM4B_HUMAN]	40.18	3	10	10	52	0.884	1.247	1.117	0.956	97.80	40.18	16	52	224	24.9	4.82
Q9Y2H9	Microtubule-associated serine/threonine-protein kinase 1 OS=Homo sapiens GN=MAST1 PE=1 SV=2 - [MAST1_HUMA	4.20	2	3	4	6	1.679	1.648	1.290	0.956	3.39	4.20	4	6	1570	170.6	8.44
P53367	Arfaptin-1 OS=Homo sapiens GN=ARFIP1 PE=1 SV=2 - [ARFP1_HUMAN]	49.60	1	14	16	43	1.030	0.955	1.097	0.956	113.18	49.60	24	43	373	41.7	6.70
Q9Y6C9	Mitochondrial carrier homolog 2 OS=Homo sapiens GN=MTCH2 PE=1 SV=1 - [MTCH2_HUMA	12.87	1	3	3	8	1.236	1.025	0.823	0.956	12.79	12.87	5	8	303	33.3	7.97
P00441	Superoxide dismutase [Cu-Zn] OS=Homo sapiens GN=SOD1 PE=1 SV=2 - [SODC_HUMAN]	86.36	1	12	12	104	1.132	1.049	1.043	0.956	297.49	86.36	16	104	154	15.9	6.13

Q8WWM7	Ataxin-2-like protein OS=Homo sapiens GN=ATXN2L PE=1 SV=2 - [ATX2L_HUMAN]	25.49	1	23	25	115	0.857	0.962	0.995	0.956	244.14	25.49	41	115	1075	113.3	8.59
P62249	40S ribosomal protein S16 OS=Homo sapiens GN=RPS16 PE=1 SV=2 - [RS16_HUMAN]	64.38	1	16	16	109	0.906	0.882	0.810	0.956	203.91	64.38	23	109	146	16.4	10.21
Q9P2R3	Rabankyrin-5 OS=Homo sapiens GN=ANKFY1 PE=1 SV=2 - [ANFY1_HUMAN]	17.37	2	16	17	47	1.106	0.847	0.858	0.956	117.99	17.37	26	47	1169	128.3	6.10
Q96BW1	Uracil phosphoribosyltransferase homolog OS=Homo sapiens GN=UPRT PE=2 SV=1 - [UPP_HUMAN]	3.56	1	1	1	4	1.052	0.712	0.655	0.956	5.96	3.56	2	4	309	33.8	6.04
Q8NBT2	Kinetochore protein Spc24 OS=Homo sapiens GN=SPC24 PE=1 SV=2 - [SPC24_HUMAN]	20.81	3	3	4	20	1.164	1.910	0.525	0.956	45.78	20.81	7	20	197	22.5	4.70
O00429	Dynamin-1-like protein OS=Homo sapiens GN=DNM1L PE=1 SV=2 - [DNM1L_HUMAN]	40.35	1	29	29	98	1.100	1.102	0.948	0.956	270.60	40.35	48	98	736	81.8	6.81
Q6NUP7	Serine/threonine-protein phosphatase 4 regulatory subunit 4 OS=Homo sapiens GN=PPP4R4 PE=1 SV=1 - [PP4R4_HUMAN]	2.18	1	2	2	5	1.971	0.361	0.494	0.956	3.79	2.18	2	5	873	99.4	7.80
Q6UVY6	DBH-like monooxygenase protein 1 OS=Homo sapiens GN=MOXD1 PE=2 SV=1 - [MOXD1_HUMAN]	12.07	1	6	6	10	0.811	2.542	0.905	0.956	18.93	12.07	7	10	613	69.6	6.43
P13639	Elongation factor 2 OS=Homo sapiens GN=EEF2 PE=1 SV=4 - [EF2_HUMAN]	55.59	1	53	56	932	0.764	0.796	0.873	0.957	2515.65	55.59	92	932	858	95.3	6.83

Q04323	UBX domain-containing protein 1 OS=Homo sapiens GN=UBXN1 PE=1 SV=2 - [UBXN1_HUMAN]	51.18	1	9	9	20	0.850	1.035	0.990	0.957	64.71	51.18	12	20	297	33.3	5.25
P67870	Casein kinase II subunit beta OS=Homo sapiens GN=CSNK2B PE=1 SV=1 - [CSK2B_HUMAN]	26.05	1	6	6	19	0.763	0.908	0.917	0.957	51.62	26.05	10	19	215	24.9	5.55
O76054	SEC14-like protein 2 OS=Homo sapiens GN=SEC14L2 PE=1 SV=1 - [S14L2_HUMAN]	11.91	1	5	5	15	0.711	1.307	1.438	0.957	34.84	11.91	8	15	403	46.1	7.84
Q07108	Early activation antigen CD69 OS=Homo sapiens GN=CD69 PE=1 SV=1 - [CD69_HUMAN]	6.53	1	1	1	1	1.216	0.533	0.807	0.957	2.51	6.53	1	1	199	22.5	6.61
P56377	AP-1 complex subunit sigma-2 OS=Homo sapiens GN=AP1S2 PE=1 SV=1 - [AP1S2_HUMAN]	15.92	1	2	2	8	0.817	1.279	1.704	0.957	19.24	15.92	4	8	157	18.6	5.47
Q63HK3	Zinc finger protein with KRAB and SCAN domains 2 OS=Homo sapiens GN=ZKSCAN2 PE=1 SV=2 - [ZKSC2_HUMAN]	2.69	1	1	2	3	1.362	1.454	1.780	0.957	8.47	2.69	2	3	967	110.9	8.35
P52888	Thimet oligopeptidase OS=Homo sapiens GN=THOP1 PE=1 SV=2 - [THOP1_HUMAN]	23.80	1	15	15	41	1.017	0.937	1.001	0.957	95.03	23.80	22	41	689	78.8	6.05
P98196	Probable phospholipid-transporting ATPase IH OS=Homo sapiens GN=ATP11A PE=1 SV=3 - [AT11A_HUMAN]	2.20	1	3	3	3	1.167	0.647	0.627	0.957	5.72	2.20	3	3	1134	129.7	6.60
Q9Y3D9	28S ribosomal protein S23, mitochondrial OS=Homo sapiens GN=MRPS23 PE=1 SV=2 - [RT23_HUMAN]	31.05	1	6	6	10	0.654	0.923	1.020	0.957	27.18	31.05	9	10	190	21.8	8.90

P27987	Inositol-trisphosphate 3-kinase B OS=Homo sapiens GN=ITPKB PE=1 SV=5 - [IP3KB_HUMAN]	7.29	1	5	5	11	0.692	1.628	1.027	0.958	22.37	7.29	6	11	946	102.3	8.43
P26572	Alpha-1,3-mannosyl-glycoprotein 2-beta-N-acetylglucosaminyltransferase OS=Homo sapiens GN=MGAT1 PE=2 SV=2 - [MGAT1_HUMAN]	24.94	1	10	10	19	1.256	0.788	0.790	0.958	39.12	24.94	15	19	445	50.8	9.16
Q2KHT3	Protein CLEC16A OS=Homo sapiens GN=CLEC16A PE=2 SV=2 - [CL16A_HUMAN]	0.76	1	1	1	1	0.878	0.862	0.726	0.958	1.79	0.76	1	1	1053	117.6	5.86
P22830	Ferrochelatase, mitochondrial OS=Homo sapiens GN=FECH PE=1 SV=2 - [HEMH_HUMAN]	23.64	1	10	10	37	0.671	0.865	1.017	0.958	89.62	23.64	16	37	423	47.8	8.73
Q92508	Piezo-type mechanosensitive ion channel component 1 OS=Homo sapiens GN=PIEZO1 PE=1 SV=4 - [PIEZ1_HUMAN]	4.56	1	9	10	41	1.288	0.635	0.754	0.958	96.03	4.56	16	41	2521	286.6	7.47
Q8IUX8	Epidermal growth factor-like protein 6 OS=Homo sapiens GN=EGFL6 PE=2 SV=1 - [EGFL6_HUMAN]	1.27	1	1	1	5	0.718	1.100	0.828	0.958	14.65	1.27	1	5	553	61.3	8.06
P34949	Mannose-6-phosphate isomerase OS=Homo sapiens GN=MPI PE=1 SV=2 - [MPI_HUMAN]	23.17	1	8	8	18	0.824	1.033	0.760	0.958	47.33	23.17	10	18	423	46.6	5.95
P08572	Collagen alpha-2(IV) chain OS=Homo sapiens GN=COL4A2 PE=1 SV=4 - [CO4A2_HUMAN]	5.14	1	6	6	16	0.827	1.014	1.700	0.958	43.37	5.14	9	16	1712	167.4	8.66
P49815	Tuberin OS=Homo sapiens GN=TSC2 PE=1 SV=2 - [TSC2_HUMAN]	5.26	1	8	8	22	0.884	1.062	1.078	0.958	49.96	5.26	13	22	1807	200.5	7.31

P29144	Tripeptidyl-peptidase 2 OS=Homo sapiens GN=TPP2 PE=1 SV=4 - [TPP2_HUMAN]	33.55	1	36	36	134	0.945	0.950	0.880	0.958	317.68	33.55	55	134	1249	138.3	6.32
Q9UNK0	Syntaxin-8 OS=Homo sapiens GN=STX8 PE=1 SV=2 - [STX8_HUMAN]	29.66	1	6	6	28	0.809	0.811	0.867	0.958	69.56	29.66	10	28	236	26.9	4.98
Q5T9S5	Coiled-coil domain-containing protein 18 OS=Homo sapiens GN=CCDC18 PE=2 SV=1 - [CCD18_HUMAN]	2.27	1	2	4	12	1.257	1.043	0.872	0.958	26.73	2.27	4	12	1454	168.9	5.66
O14617	AP-3 complex subunit delta-1 OS=Homo sapiens GN=AP3D1 PE=1 SV=1 - [AP3D1_HUMAN]	14.40	1	15	15	47	0.981	0.970	1.143	0.959	97.96	14.40	22	47	1153	130.1	8.48
Q13177	Serine/threonine-protein kinase PAK 2 OS=Homo sapiens GN=PAK2 PE=1 SV=3 - [PAK2_HUMAN]	42.56	1	16	20	101	1.031	1.010	0.912	0.959	257.43	42.56	32	101	524	58.0	5.96
Q8TBA6	Golgin subfamily A member 5 OS=Homo sapiens GN=GOLGA5 PE=1 SV=3 - [GOGA5_HUMAN]	33.11	1	21	21	70	0.798	0.975	0.946	0.959	167.53	33.11	32	70	731	83.0	5.83
Q8WZA0	Protein LZIC OS=Homo sapiens GN=LZIC PE=1 SV=1 - [LZIC_HUMAN]	32.63	1	7	7	24	1.032	1.172	1.043	0.959	47.50	32.63	12	24	190	21.5	4.94
Q99943	1-acyl-sn-glycerol-3-phosphate acyltransferase alpha OS=Homo sapiens GN=AGPAT1 PE=1 SV=2 - [PLCA_HUMAN]	10.60	1	2	2	4	3.550	1.786	2.661	0.959	8.30	10.60	3	4	283	31.7	9.38
Q9BQ51	Programmed cell death 1 ligand 2 OS=Homo sapiens GN=PDCD1LG2 PE=1 SV=2 - [PD1L2_HUMAN]	9.16	1	2	2	3	2.730	0.450	0.698	0.959	9.51	9.16	2	3	273	30.9	8.03

Q9BZF1	Oxysterol-binding protein-related protein 8 OS=Homo sapiens GN=OSBPL8 PE=1 SV=3 - [OSBL8_HUMAN]	17.77	1	12	13	40	0.772	1.056	0.889	0.959	99.53	17.77	20	40	889	101.1	6.96
Q7Z7L7	Protein zer-1 homolog OS=Homo sapiens GN=ZER1 PE=1 SV=1 - [ZER1_HUMAN]	1.04	1	1	1	2	1.141	0.916	1.158	0.959	0.00	1.04	1	2	766	88.1	5.62
Q9UQ80	Proliferation-associated protein 2G4 OS=Homo sapiens GN=PA2G4 PE=1 SV=3 - [PA2G4_HUMAN]	41.37	2	16	17	141	0.814	0.936	0.741	0.959	340.19	41.37	27	141	394	43.8	6.55
O95433	Activator of 90 kDa heat shock protein ATPase homolog 1 OS=Homo sapiens GN=AHSA1 PE=1 SV=1 - [AHSA1_HUMAN]	52.66	1	16	16	113	0.867	0.800	0.955	0.959	364.95	52.66	27	113	338	38.3	5.53
P28715	DNA repair protein complementing XP-G cells OS=Homo sapiens GN=ERCC5 PE=1 SV=3 - [ERCC5_HUMAN]	0.84	1	1	1	1	1.277	1.130	1.280	0.959	2.56	0.84	1	1	1186	133.0	5.22
Q96SU4	Oxysterol-binding protein-related protein 9 OS=Homo sapiens GN=OSBPL9 PE=1 SV=2 - [OSBL9_HUMAN]	13.59	1	6	7	10	0.784	0.955	0.819	0.959	25.92	13.59	9	10	736	83.1	6.18
Q9Y6B6	GTP-binding protein SAR1b OS=Homo sapiens GN=SAR1B PE=1 SV=1 - [SAR1B_HUMAN]	32.83	1	3	5	25	1.264	0.793	0.987	0.959	69.20	32.83	9	25	198	22.4	6.11
Q8TE02	Elongator complex protein 5 OS=Homo sapiens GN=ELP5 PE=1 SV=2 - [ELP5_HUMAN]	4.43	1	1	1	4	0.746	0.944	1.117	0.959	12.76	4.43	2	4	316	34.8	4.97
Q14590	Zinc finger protein 235 OS=Homo sapiens GN=ZNF235 PE=2 SV=3 - [ZN235_HUMAN]	11.11	120	2	4	7	0.748	1.206	0.901	0.959	2.55	11.11	5	7	738	83.9	8.56

P51809	Vesicle-associated membrane protein 7 OS=Homo sapiens GN=VAMP7 PE=1 SV=3 - [VAMP7_HUMAN]	16.82	1	3	4	7	0.918	0.820	0.944	0.959	12.00	16.82	6	7	220	24.9	8.60
Q6T4R5	Nance-Horan syndrome protein OS=Homo sapiens GN=NHS PE=1 SV=2 - [NHS_HUMAN]	2.42	1	3	3	5	1.077	1.036	1.104	0.959	11.44	2.42	4	5	1651	179.0	6.86
O43516	WAS/WASL-interacting protein family member 1 OS=Homo sapiens GN=WIPF1 PE=1 SV=3 - [WIPF1_HUMAN]	15.51	1	5	5	16	1.089	1.662	1.433	0.960	25.04	15.51	8	16	503	51.2	11.47
Q9H5V9	UPF0428 protein CXorf56 OS=Homo sapiens GN=CXorf56 PE=1 SV=1 - [CX056_HUMAN]	3.15	1	1	1	1	1.701	0.920	1.076	0.960	2.56	3.15	1	1	222	25.6	8.73
Q4V328	GRIP1-associated protein 1 OS=Homo sapiens GN=GRIPAP1 PE=1 SV=1 - [GRAP1_HUMAN]	32.94	2	24	24	74	1.127	1.239	1.231	0.960	167.76	32.94	36	74	841	95.9	5.11
Q9UHB6	LIM domain and actin-binding protein 1 OS=Homo sapiens GN=LIMA1 PE=1 SV=1 - [LIMA1_HUMAN]	44.01	1	33	33	184	0.994	0.872	0.650	0.960	507.22	44.01	48	184	759	85.2	6.84
Q9UBP9	PTB domain-containing engulfment adapter protein 1 OS=Homo sapiens GN=GULP1 PE=1 SV=1 - [GULP1_HUMAN	25.33	1	9	9	26	0.786	1.222	0.978	0.960	49.64	25.33	15	26	304	34.5	7.90
Q9Y2K6	Ubiquitin carboxyl-terminal hydrolase 20 OS=Homo sapiens GN=USP20 PE=1 SV=2 - [UBP20_HUMAN]	2.08	1	1	1	2	0.767	1.240	1.291	0.960	4.14	2.08	1	2	914	101.9	6.11

Q9HBM0	Vezatin OS=Homo sapiens GN=VEZT PE=1 SV=3 - [VEZA_HUMAN]	8.60	1	6	7	13	1.067	1.394	1.198	0.960	29.68	8.60	9	13	779	88.6	5.20
Q14CM0	FERM and PDZ domain-containing protein 4 OS=Homo sapiens GN=FRMPD4 PE=1 SV=1 - [FRPD4_HUMAN]	1.74	1	1	2	2	0.372	0.699	1.226	0.960	2.24	1.74	2	2	1322	144.3	5.25
Q9HCJ1	Progressive ankylosis protein homolog OS=Homo sapiens GN=ANKH PE=1 SV=2 - [ANKH_HUMAN]	4.07	1	1	2	5	2.087	0.300	0.384	0.960	4.62	4.07	3	5	492	54.2	7.88
P55196	Afadin OS=Homo sapiens GN=MLLT4 PE=1 SV=3 - [AFAD_HUMAN]	5.76	1	10	10	17	1.039	1.778	1.374	0.960	31.33	5.76	15	17	1824	206.7	6.47
P18621	60S ribosomal protein L17 OS=Homo sapiens GN=RPL17 PE=1 SV=3 - [RL17_HUMAN]	44.02	1	10	11	188	0.826	0.951	0.823	0.961	448.26	44.02	20	188	184	21.4	10.17
P28070	Proteasome subunit beta type-4 OS=Homo sapiens GN=PSMB4 PE=1 SV=4 - [PSB4_HUMAN]	38.26	1	7	7	38	0.977	0.983	1.030	0.961	114.32	38.26	13	38	264	29.2	5.97
Q9Y5V3	Melanoma-associated antigen D1 OS=Homo sapiens GN=MAGED1 PE=1 SV=3 - [MAGD1_HUMAN]	18.77	2	9	10	37	0.725	0.805	0.861	0.961	82.17	18.77	15	37	778	86.1	5.83
Q96HN2	Putative adenosylhomocysteinase 3 OS=Homo sapiens GN=AHCYL2 PE=1 SV=1 - [SAHH3_HUMAN]	22.91	1	2	17	51	1.102	1.106	1.092	0.961	86.92	22.91	28	51	611	66.7	7.36
Q96QK1	Vacuolar protein sorting-associated protein 35 OS=Homo sapiens GN=VPS35 PE=1 SV=2 - [VPS35_HUMAN]	30.28	1	22	23	101	1.030	0.995	0.978	0.961	248.52	30.28	37	101	796	91.6	5.49

Q9C0B0	RING finger protein unkempt homolog OS=Homo sapiens GN=UNK PE=1 SV=2 - [UNK_HUMAN]	2.59	1	2	2	3	1.482	1.080	1.056	0.961	7.30	2.59	3	3	810	88.0	6.86
P09382	Galectin-1 OS=Homo sapiens GN=LGALS1 PE=1 SV=2 - [LEG1_HUMAN]	82.22	1	14	14	703	1.185	1.012	1.105	0.961	1983.67	82.22	24	703	135	14.7	5.50
Q8IWR0	Zinc finger CCCH domain-containing protein 7A OS=Homo sapiens GN=ZC3H7A PE=1 SV=1 - [Z3H7A_HUMAN]	7.31	1	6	7	14	0.980	0.921	1.008	0.961	21.96	7.31	10	14	971	110.5	7.30
O75094	Slit homolog 3 protein OS=Homo sapiens GN=SLIT3 PE=2 SV=3 - [SLIT3_HUMAN]	1.77	1	2	2	5	0.446	1.054	1.002	0.961	10.19	1.77	3	5	1523	167.6	7.65
Q7L1Q6	Basic leucine zipper and W2 domain-containing protein 1 OS=Homo sapiens GN=BZW1 PE=1 SV=1 - [BZW1_HUMAN]	34.61	1	16	17	93	0.913	1.014	0.901	0.961	176.17	34.61	27	93	419	48.0	5.92
Q9BX68	Histidine triad nucleotide-binding protein 2, mitochondrial OS=Homo sapiens GN=HINT2 PE=1 SV=1 - [HINT2_HUMAN	51.53	1	6	6	39	1.264	1.339	1.117	0.961	87.37	51.53	10	39	163	17.2	9.16
Q9BTD3	Transmembrane protein 121 OS=Homo sapiens GN=TMEM121 PE=2 SV=1 - [TM121_HUMAN]	2.82	1	1	1	3	1.057	1.041	0.811	0.961	1.77	2.82	1	3	319	35.8	9.26
Q9Y217	Myotubularin-related protein 6 OS=Homo sapiens GN=MTMR6 PE=1 SV=3 - [MTMR6_HUMAN]	15.30	2	8	9	22	0.846	0.925	1.065	0.961	51.11	15.30	15	22	621	71.9	7.66

Q9P275	Ubiquitin carboxyl-terminal hydrolase 36 OS=Homo sapiens GN=USP36 PE=1 SV=3 - [UBP36_HUMAN]	2.23	1	2	2	2	0.950	0.244	0.613	0.961	2.71	2.23	2	2	1121	122.6	9.67
Q7Z5H3	Rho GTPase-activating protein 22 OS=Homo sapiens GN=ARHGAP22 PE=1 SV=1 - [RHG22_HUMAN]	2.15	1	1	1	6	0.731	1.329	1.337	0.961	12.34	2.15	2	6	698	76.7	8.15
Q7Z4G4	tRNA (guanine(10)-N2)-methyltransferase homolog OS=Homo sapiens GN=TRMT11 PE=1 SV=1 - [TRM11_HUMAN]	1.30	1	1	1	1	0.974	1.114	0.825	0.961	1.65	1.30	1	1	463	53.4	7.78
Q9P2B4	CTTNBP2 N-terminal-like protein OS=Homo sapiens GN=CTTNBP2NL PE=1 SV=2 - [CT2NL_HUMAN]	44.91	1	26	27	69	1.070	1.101	1.038	0.962	156.89	44.91	40	69	639	70.1	8.06
P14406	Cytochrome c oxidase subunit 7A2, mitochondrial OS=Homo sapiens GN=COX7A2 PE=1 SV=1 - [CX7A2_HUMAN]	27.71	1	2	2	20	1.080	0.598	0.607	0.962	51.60	27.71	4	20	83	9.4	9.76
Q13615	Myotubularin-related protein 3 OS=Homo sapiens GN=MTMR3 PE=1 SV=3 - [MTMR3_HUMAN]	2.17	1	2	2	4	0.904	0.787	0.823	0.962	11.53	2.17	2	4	1198	133.5	5.80
Q14677	Clathrin interactor 1 OS=Homo sapiens GN=CLINT1 PE=1 SV=1 - [EPN4_HUMAN]	27.36	1	15	15	63	1.078	1.082	0.950	0.962	176.75	27.36	24	63	625	68.2	6.42
Q13126	S-methyl-5'-thioadenosine phosphorylase OS=Homo sapiens GN=MTAP PE=1 SV=2 - [MTAP_HUMAN]	35.69	1	8	8	25	0.873	0.701	0.768	0.962	61.70	35.69	11	25	283	31.2	7.18

P18124	60S ribosomal protein L7 OS=Homo sapiens GN=RPL7 PE=1 SV=1 - [RL7_HUMAN]	56.05	1	20	20	240	0.872	1.068	0.917	0.962	471.67	56.05	33	240	248	29.2	10.65
Q92576	PHD finger protein 3 OS=Homo sapiens GN=PHF3 PE=1 SV=3 - [PHF3_HUMAN]	1.47	1	3	3	4	0.875	0.749	0.412	0.962	11.97	1.47	3	4	2039	229.3	6.96
P31949	Protein S100-A11 OS=Homo sapiens GN=S100A11 PE=1 SV=2 - [S10AB_HUMAN]	45.71	1	6	6	244	1.114	0.865	1.038	0.962	753.90	45.71	11	244	105	11.7	7.12
P05114	Non-histone chromosomal protein HMG-14 OS=Homo sapiens GN=HMGN1 PE=1 SV=3 - [HMGN1_HUMA N]	43.00	1	6	6	29	1.295	1.254	1.201	0.962	69.65	43.00	9	29	100	10.7	9.60
Q9BRJ6	Uncharacterize d protein C7orf50 OS=Homo sapiens GN=C7orf50 PE=1 SV=1 - [CG050_HUMAN]	17.01	1	2	2	7	0.790	1.287	0.936	0.963	22.04	17.01	4	7	194	22.1	9.64
Q9NP85	Podocin OS=Homo sapiens GN=NPHS2 PE=1 SV=1 - [PODO_HUMAN]	2.09	1	1	1	3	0.879	1.061	0.955	0.963	6.40	2.09	1	3	383	42.2	8.75
Q9H6T3	RNA polymerase II-associated protein 3 OS=Homo sapiens GN=RPAP3 PE=1 SV=2 - [RPAP3_HUMAN]	18.80	1	11	11	28	0.787	1.176	1.093	0.963	59.81	18.80	16	28	665	75.7	6.84
Q9H1P3	Oxysterol-binding protein-related protein 2 OS=Homo sapiens GN=OSBPL2 PE=1 SV=1 - [OSBL2_HUMAN]	10.63	1	4	4	15	1.139	1.078	0.923	0.963	32.00	10.63	6	15	480	55.2	6.35
Q9Y5B0	RNA polymerase II subunit A C-terminal domain phosphatase OS=Homo sapiens GN=CTDP1 PE=1 SV=3 - [CTDP1_HUMAN	12.70	1	8	8	16	1.088	1.041	1.012	0.963	35.68	12.70	12	16	961	104.3	5.27

Q9H4A3	Serine/threonine-protein kinase WNK1 OS=Homo sapiens GN=WNK1 PE=1 SV=2 - [WNK1_HUMAN]	10.29	1	20	25	71	1.123	1.092	0.976	0.963	156.57	10.29	42	71	2382	250.6	6.34
Q9HD33	39S ribosomal protein L47, mitochondrial OS=Homo sapiens GN=MRPL47 PE=1 SV=2 - [RM47_HUMAN]	20.80	1	7	7	16	0.831	1.051	0.886	0.963	27.71	20.80	11	16	250	29.4	10.37
P40429	60S ribosomal protein L13a OS=Homo sapiens GN=RPL13A PE=1 SV=2 - [RL13A_HUMAN]	36.45	2	11	11	94	0.969	0.993	0.908	0.963	166.73	36.45	17	94	203	23.6	10.93
O95336	6-phosphogluconolactonase OS=Homo sapiens GN=PGLS PE=1 SV=2 - [6PGL_HUMAN]	52.71	1	11	11	55	0.978	0.977	0.917	0.963	115.40	52.71	18	55	258	27.5	6.05
Q8WWI5	Choline transporter-like protein 1 OS=Homo sapiens GN=SLC44A1 PE=1 SV=1 - [CTL1_HUMAN]	1.83	1	1	1	1	1.393	1.346	0.668	0.963	0.00	1.83	1	1	657	73.3	8.60
O43292	Glycosylphosphatidylinositol anchor attachment 1 protein OS=Homo sapiens GN=GPAA1 PE=1 SV=3 - [GPAA1_HUMAN]	2.09	1	1	1	6	0.858	0.755	0.704	0.963	15.96	2.09	2	6	621	67.6	8.06
Q96DL1	NXPE family member 2 OS=Homo sapiens GN=NXPE2 PE=2 SV=2 - [NXPE2_HUMAN]	1.25	1	1	1	1	1.770	0.406	1.737	0.963	0.00	1.25	1	1	559	64.9	8.60
Q96BZ9	TBC1 domain family member 20 OS=Homo sapiens GN=TBC1D20 PE=1 SV=1 - [TBC20_HUMAN]	12.90	1	6	6	10	0.920	0.775	1.052	0.963	19.01	12.90	10	10	403	45.8	6.86
P60842	Eukaryotic initiation factor 4A-I OS=Homo sapiens GN=EIF4A1 PE=1 SV=1 - [IF4A1_HUMAN]	56.40	1	11	24	361	0.890	0.829	0.824	0.963	912.69	56.40	42	361	406	46.1	5.48

O15111	Inhibitor of nudear factor kappa-B kinase subunit alpha OS=Homo sapiens GN=CHUK PE=1 SV=2 - [IKKA_HUMAN]	6.31	1	4	4	6	0.850	0.733	1.042	0.963	11.20	6.31	6	6	745	84.6	6.73
P54819	Adenylate kinase 2, mitochondrial OS=Homo sapiens GN=AK2 PE=1 SV=2 - [KAD2_HUMAN]	41.42	1	10	10	62	0.829	1.075	0.808	0.964	194.38	41.42	18	62	239	26.5	7.81
Q7Z4N8	Prolyl 4-hydroxylase subunit alpha-3 OS=Homo sapiens GN=P4HA3 PE=1 SV=1 - [P4HA3_HUMAN]	13.24	1	6	6	9	0.964	1.300	0.495	0.964	25.70	13.24	8	9	544	61.1	6.49
Q96ER3	Protein SAAL1 OS=Homo sapiens GN=SAAL1 PE=1 SV=2 - [SAAL1_HUMAN]	4.64	1	2	2	2			2.862	0.964	5.73	4.64	2	2	474	53.5	4.50
Q16827	Receptor-type tyrosine-protein phosphatase O OS=Homo sapiens GN=PTPRO PE=1 SV=2 - [PTPRO_HUMA	0.49	1	1	1	2	0.704	0.809	0.672	0.964	3.73	0.49	1	2	1216	138.3	6.01
Q8IY31	Intraflagellar transport protein 20 homolog OS=Homo sapiens GN=IFT20 PE=1 SV=1 - [IFT20_HUMAN]	10.61	1	1	1	2	0.675	0.708	1.183	0.964	4.72	10.61	2	2	132	15.3	5.12
Q6ZMW3	Echinoderm microtubule-associated protein-like 6 OS=Homo sapiens GN=EML6 PE=2 SV=2 - [EMAL6_HUMAN]	1.94	1	3	4	5	0.766	1.119	1.063	0.964	7.76	1.94	4	5	1958	217.8	7.44
Q9Y6M9	NADH dehydrogenase [ubiquinone] 1 beta subcomplex subunit 9 OS=Homo sapiens GN=NDUFB9 PE=1 SV=3 - [NDUB9_HUMAN]	40.22	1	7	7	26	0.668	0.966	0.767	0.964	70.85	40.22	12	26	179	21.8	8.38

Q5VZ89	DENN domain-containing protein 4C OS=Homo sapiens GN=DENND4C PE=1 SV=2 - [DEN4C_HUMAN]	3.59	1	5	5	9	1.039	1.170	1.084	0.964	15.88	3.59	7	9	1673	186.7	6.86
Q96C11	FGGY carbohydrate kinase domain-containing protein OS=Homo sapiens GN=FGGY PE=1 SV=2 - [FGGY_HUMAN]	4.54	1	2	2	3	0.825	0.853	0.857	0.964	5.14	4.54	3	3	551	60.0	6.35
Q8NEY1	Neuron navigator 1 OS=Homo sapiens GN=NAV1 PE=1 SV=2 - [NAV1_HUMAN]	10.44	1	16	16	34	1.206	0.855	1.199	0.964	86.68	10.44	25	34	1877	202.3	8.07
Q8IXM3	39S ribosomal protein L41, mitochondrial OS=Homo sapiens GN=MRPL41 PE=1 SV=1 - [RM41_HUMAN]	15.33	1	2	2	8	0.681	1.052	0.943	0.964	13.54	15.33	4	8	137	15.4	9.57
P49427	Ubiquitin-conjugating enzyme E2 R1 OS=Homo sapiens GN=CDC34 PE=1 SV=2 - [UB2R1_HUMAN]	11.02	1	2	3	11	0.822	1.291	1.195	0.964	18.37	11.02	5	11	236	26.7	4.54
Q96A65	Exocyst complex component 4 OS=Homo sapiens GN=EXOC4 PE=1 SV=1 - [EXOC4_HUMAN]	23.10	3	20	21	56	1.098	0.964	0.956	0.964	147.18	23.10	29	56	974	110.4	6.49
Q13042	Cell division cycle protein 16 homolog OS=Homo sapiens GN=CDC16 PE=1 SV=2 - [CDC16_HUMAN]	6.61	1	4	4	9	0.829	0.762	0.812	0.964	23.86	6.61	5	9	620	71.6	5.85
Q8WZ42	Titin OS=Homo sapiens GN=TTN PE=1 SV=4 - [TITIN_HUMAN]	1.11	3	24	30	36	1.032	0.864	0.781	0.964	60.30	1.11	30	36	34350	####	6.35
P27635	60S ribosomal protein L10 OS=Homo sapiens GN=RPL10 PE=1 SV=4 - [RL10_HUMAN]	46.73	2	13	13	121	0.932	0.837	0.791	0.964	380.24	46.73	19	121	214	24.6	10.08

P50914	60S ribosomal protein L14 OS=Homo sapiens GN=RPL14 PE=1 SV=4 - [RL14_HUMAN]	50.70	2	14	14	143	0.835	1.051	1.014	0.964	288.19	50.70	18	143	215	23.4	10.93
Q6UXX5	Inter-alpha-trypsin inhibitor heavy chain H6 OS=Homo sapiens GN=ITIH6 PE=2 SV=1 - [ITIH6_HUMAN]	0.46	1	1	1	1	0.636	0.412	0.574	0.965	0.00	0.46	1	1	1313	143.1	9.01
O60469	Down syndrome cell adhesion molecule OS=Homo sapiens GN=DSCAM PE=1 SV=2 - [DSCAM_HUMAN]	3.23	1	3	5	6	0.966	0.701	0.792	0.965	10.75	3.23	5	6	2012	222.1	7.68
Q9NRW7	Vacuolar protein sorting-associated protein 45 OS=Homo sapiens GN=VPS45 PE=1 SV=1 - [VPS45_HUMAN]	16.14	1	9	9	23	0.999	0.663	0.645	0.965	61.95	16.14	15	23	570	65.0	8.24
P46782	40S ribosomal protein S5 OS=Homo sapiens GN=RPS5 PE=1 SV=4 - [RS5_HUMAN]	36.76	1	10	10	96	0.857	1.000	0.951	0.965	251.15	36.76	19	96	204	22.9	9.72
Q96KH6	Uncharacterized protein C18orf12 OS=Homo sapiens GN=C18orf12 PE=2 SV=1 - [CR012_HUMAN]	9.55	1	1	1	1	2.424	1.169	1.855	0.965	0.00	9.55	1	1	178	19.7	6.61
Q9P266	Junctional protein associated with coronary artery disease OS=Homo sapiens GN=KIAA1462 PE=1 SV=3 - [JCAD_HUMAN]	10.23	1	10	10	24	1.158	0.628	1.593	0.965	50.95	10.23	17	24	1359	148.3	7.09
O60884	DnaJ homolog subfamily A member 2 OS=Homo sapiens GN=DNAJA2 PE=1 SV=1 - [DNJA2_HUMAN]	37.38	1	11	11	52	0.906	1.025	0.960	0.965	137.32	37.38	18	52	412	45.7	6.48

Q8WV92	MIT domain-containing protein 1 OS=Homo sapiens GN=MITD1 PE=1 SV=1 - [MITD1_HUMAN]	4.02	1	1	1	4	0.833	1.369	0.983	0.965	6.08	4.02	2	4	249	29.3	8.00
Q15008	26S proteasome non-ATPase regulatory subunit 6 OS=Homo sapiens GN=PSMD6 PE=1 SV=1 - [PSMD6_HUMA	40.36	1	20	20	81	0.947	1.046	0.958	0.965	203.24	40.36	33	81	389	45.5	5.62
Q00688	Peptidyl-prolyl cis-trans isomerase FKBP3 OS=Homo sapiens GN=FKBP3 PE=1 SV=1 - [FKBP3_HUMAN]	71.43	1	19	19	78	1.306	1.560	1.098	0.965	216.39	71.43	28	78	224	25.2	9.28
Q9NQ88	Fructose-2,6-bisphosphatase TIGAR OS=Homo sapiens GN=TIGAR PE=1 SV=1 - [TIGAR_HUMAN]	40.00	1	7	8	20	1.090	1.179	0.848	0.965	55.73	40.00	12	20	270	30.0	7.69
Q8IWA4	Mitofusin-1 OS=Homo sapiens GN=MFN1 PE=1 SV=2 - [MFN1_HUMAN]	10.80	1	5	5	7	1.222	0.809	0.746	0.965	15.36	10.80	6	7	741	84.0	6.25
P08865	40S ribosomal protein SA OS=Homo sapiens GN=RPSA PE=1 SV=4 - [RSSA_HUMAN]	51.19	2	14	14	133	0.819	0.963	0.938	0.965	380.70	51.19	22	133	295	32.8	4.87
Q9NZV5	Selenoprotein N OS=Homo sapiens GN=SEPN1 PE=1 SV=5 - [SELN_HUMAN]	7.63	1	5	5	9	0.993	0.714	0.904	0.965	11.96	7.63	6	9	590	65.8	5.48
O75461	Transcription factor E2F6 OS=Homo sapiens GN=E2F6 PE=1 SV=1 - [E2F6_HUMAN]	3.91	1	1	1	1	0.657	0.771	1.240	0.965	0.00	3.91	1	1	281	31.8	5.50
O95999	B-cell lymphoma/leukemia 10 OS=Homo sapiens GN=BCL10 PE=1 SV=1 - [BCL10_HUMAN]	14.59	1	3	4	11	1.097	0.962	0.981	0.965	19.22	14.59	6	11	233	26.2	5.74

Q8N0V3	Putative ribosome-binding factor A, mitochondrial OS=Homo sapiens GN=RBFA PE=1 SV=3 - [RBFA_HUMAN]	5.83	1	1	1	2	0.658	0.498	0.405	0.965	7.68	5.83	2	2	343	38.3	7.85
O75821	Eukaryotic translation initiation factor 3 subunit G OS=Homo sapiens GN=EIF3G PE=1 SV=2 - [EIF3G_HUMAN]	42.50	1	15	15	53	0.889	0.784	0.701	0.965	129.94	42.50	21	53	320	35.6	6.13
Q8TEX9	Importin-4 OS=Homo sapiens GN=IPO4 PE=1 SV=2 - [IPO4_HUMAN]	7.31	1	7	7	25	0.806	0.801	0.946	0.965	42.54	7.31	10	25	1081	118.6	4.96
Q04695	Keratin, type I cytoskeletal 17 OS=Homo sapiens GN=KRT17 PE=1 SV=2 - [K1C17_HUMAN]	16.67	6	1	10	26	0.771	0.095	1.527	0.965	47.23	16.67	13	26	432	48.1	5.02
P60520	Gamma-aminobutyric acid receptor-associated protein-like 2 OS=Homo sapiens GN=GABARAPL2 PE=1 SV=1 - [GBRL2_HUMAN]	39.32	1	4	5	9	0.833	0.637	0.694	0.966	17.41	39.32	6	9	117	13.7	8.10
P10768	S-formylglutathione hydrolase OS=Homo sapiens GN=ESD PE=1 SV=2 - [ESTD_HUMAN]	37.94	1	9	9	58	1.103	0.704	0.843	0.966	152.59	37.94	16	58	282	31.4	7.02
Q9P219	Protein Daple OS=Homo sapiens GN=CCDC88C PE=1 SV=3 - [DAPLE_HUMAN]	1.58	3	1	5	19	0.712	0.762	0.708	0.966	41.78	1.58	6	19	2028	228.1	6.23
Q14692	Ribosome biogenesis protein BMS1 homolog OS=Homo sapiens GN=BMS1 PE=1 SV=1 - [BMS1_HUMAN]	2.18	1	3	4	6	0.912	1.248	0.844	0.966	10.06	2.18	6	6	1282	145.7	6.44

O14818	Proteasome subunit alpha type-7 OS=Homo sapiens GN=PSMA7 PE=1 SV=1 - [PSA7_HUMAN]	47.58	1	8	12	58	1.042	1.096	1.049	0.966	147.74	47.58	19	58	248	27.9	8.46
Q9H7Z7	Prostaglandin E synthase 2 OS=Homo sapiens GN=PTGES2 PE=1 SV=1 - [PGES2_HUMAN]	29.18	1	10	10	31	1.120	1.163	1.096	0.966	75.90	29.18	14	31	377	41.9	9.16
O15457	MutS protein homolog 4 OS=Homo sapiens GN=MSH4 PE=1 SV=2 - [MSH4_HUMAN]	1.50	1	1	2	3	0.915	0.866	0.957	0.966	3.60	1.50	2	3	936	104.7	7.37
P20594	Atrial natriuretic peptide receptor 2 OS=Homo sapiens GN=NPR2 PE=1 SV=1 -	3.53	1	3	3	4	1.289	0.551	0.812	0.966	7.60	3.53	4	4	1047	116.9	6.87
Q5SQI0	Alpha-tubulin N-acetyltransferase 1 OS=Homo sapiens GN=ATAT1 PE=1 SV=1 - [ATAT_HUMAN]	6.18	1	2	2	3	1.273	1.114	1.290	0.966	2.78	6.18	3	3	421	46.8	9.95
Q86SX6	Glutaredoxin-related protein 5, mitochondrial OS=Homo sapiens GN=GLRX5 PE=1 SV=2 - [GLRX5_HUMAN	21.02	1	4	4	10	0.806	1.162	0.686	0.966	19.44	21.02	6	10	157	16.6	6.79
P36578	60S ribosomal protein L4 OS=Homo sapiens GN=RPL4 PE=1 SV=5 - [RL4_HUMAN]	46.84	1	29	30	244	0.816	1.046	0.977	0.966	487.35	46.84	47	244	427	47.7	11.06
P23284	Peptidyl-prolyl cis-trans isomerase B OS=Homo sapiens GN=PPIB PE=1 SV=2 - [PPIB_HUMAN]	68.98	1	21	22	671	0.801	0.933	0.858	0.966	1512.01	68.98	37	671	216	23.7	9.41
Q9BV44	THUMP domain-containing protein 3 OS=Homo sapiens GN=THUMPD3 PE=1 SV=1 - [THUM3_HUMA N]	9.66	1	5	5	8	0.832	0.954	1.139	0.966	16.45	9.66	6	8	507	57.0	6.37

Q96H79	Zinc finger CCCH-type antiviral protein 1-like OS=Homo sapiens GN=ZC3HAV1L PE=1 SV=2 - [ZCCHL_HUMAN]	18.33	1	4	4	5	1.765	0.785	0.704	0.966	12.37	18.33	5	5	300	32.9	8.13
P30050	60S ribosomal protein L12 OS=Homo sapiens GN=RPL12 PE=1 SV=1 - [RL12_HUMAN]	67.27	1	10	10	98	0.802	0.816	0.830	0.966	275.04	67.27	16	98	165	17.8	9.42
Q14155	Rho guanine nucleotide exchange factor 7 OS=Homo sapiens GN=ARHGEF7 PE=1 SV=2 - [ARHG7_HUMAN	17.31	1	12	13	44	1.252	1.191	1.062	0.966	108.61	17.31	19	44	803	90.0	7.09
P98194	Calcium-transporting ATPase type 2C member 1 OS=Homo sapiens GN=ATP2C1 PE=1 SV=3 - [AT2C1_HUMAN]	11.86	1	8	8	23	1.276	0.759	0.871	0.967	52.28	11.86	14	23	919	100.5	6.74
Q7Z410	Transmembrane protease serine 9 OS=Homo sapiens GN=TMPRSS9 PE=1 SV=2 - [TMPS9_HUMA	1.04	1	1	1	1	1.286	0.632	0.454	0.967	2.18	1.04	1	1	1059	113.9	8.07
Q09019	Dystrophia myotonica WD repeat-containing protein OS=Homo sapiens GN=DMWD PE=1 SV=3 - [DMWD_HUMAN]	8.31	1	3	3	9	1.571	0.675	1.147	0.967	20.64	8.31	4	9	674	70.4	7.24
Q9H0X4	Protein ITFG3 OS=Homo sapiens GN=ITFG3 PE=1 SV=1 - [ITFG3_HUMAN]	5.98	1	4	4	19	1.252	0.731	0.799	0.967	34.77	5.98	7	19	552	59.6	6.28
O95376	E3 ubiquitin-protein ligase ARIH2 OS=Homo sapiens GN=ARIH2 PE=1 SV=1 - [ARI2_HUMAN]	7.91	1	3	3	6	1.115	1.159	1.115	0.967	19.85	7.91	5	6	493	57.8	5.63

Q9H2U2	Inorganic pyrophosphatase 2, mitochondrial OS=Homo sapiens GN=PPA2 PE=1 SV=2 - [IPYR2_HUMAN]	52.40	1	16	18	68	1.111	1.432	1.073	0.967	165.79	52.40	29	68	334	37.9	7.39
Q9GZV5	WW domain-containing transcription regulator protein 1 OS=Homo sapiens GN=WWTR1 PE=1 SV=1 - [WWTR1_HUMAN]	3.75	1	2	2	4	0.977	0.944	1.230	0.967	8.50	3.75	3	4	400	44.1	5.82
Q5JU85	IQ motif and SEC7 domain-containing protein 2 OS=Homo sapiens GN=IQSEC2 PE=1 SV=1 - [IQEC2_HUMAN]	2.71	1	2	4	4	1.253	0.635	0.881	0.967	5.00	2.71	4	4	1478	161.6	8.56
P12931	Proto-oncogene tyrosine-protein kinase Src OS=Homo sapiens GN=SRC PE=1 SV=3 - [SRC_HUMAN]	20.15	3	4	10	37	0.778	0.911	0.801	0.967	85.54	20.15	16	37	536	59.8	7.42
P18827	Syndecan-1 OS=Homo sapiens GN=SDC1 PE=1 SV=3 - [SDC1_HUMAN]	3.87	1	1	1	2	1.629	0.683	0.523	0.967	2.77	3.87	2	2	310	32.4	4.63
Q8WUH2	Transforming growth factor-beta receptor-associated protein 1 OS=Homo sapiens GN=TGFBRAP1 PE=1 SV=1 - [TGFA1_HUMAN]	0.81	1	1	1	1	1.529	0.893	0.937	0.967	2.18	0.81	1	1	860	97.1	6.55
Q6P1Q0	LETM1 domain-containing protein 1 OS=Homo sapiens GN=LETMD1 PE=1 SV=1 - [LTMD1_HUMAN]	3.06	1	1	1	2	0.881	1.266	0.869	0.967	2.31	3.06	2	2	360	41.8	10.32
Q9H9A5	CCR4-NOT transcription complex subunit 10 OS=Homo sapiens GN=CNOT10 PE=1 SV=1 - [CNO10_HUMAN]	4.30	2	3	3	3	1.216	0.904	0.863	0.967	4.81	4.30	3	3	744	82.3	7.78

Q96FH0	Protein MEF2BNB OS=Homo sapiens GN=MEF2BNB PE=1 SV=1 - [MF2NB_HUMAN]	15.97	1	2	2	5	0.988	1.003	1.175	0.967	13.58	15.97	3	5	119	13.4	5.80
Q08426	Peroxisomal bifunctional enzyme OS=Homo sapiens GN=EHHADH PE=1 SV=3 - [ECHP_HUMAN]	5.95	1	4	4	11	0.995	1.827	1.286	0.967	17.87	5.95	6	11	723	79.4	9.14
Q96JP5	E3 ubiquitin-protein ligase ZFP91 OS=Homo sapiens GN=ZFP91 PE=1 SV=1 - [ZFP91_HUMAN]	5.96	1	2	2	5	0.910	0.923	0.671	0.968	5.24	5.96	4	5	570	63.4	7.36
Q8TC41	Probable E3 ubiquitin-protein ligase RNF217 OS=Homo sapiens GN=RNF217 PE=2 SV=4 - [RN217_HUMAN]	4.98	1	2	2	3	1.309	1.304	1.133	0.968	4.09	4.98	2	3	542	59.3	7.52
Q9H6X4	Transmembrane protein 134 OS=Homo sapiens GN=TMEM134 PE=2 SV=1 - [TM134_HUMAN]	4.10	1	1	1	2	0.965	0.757	0.901	0.968	6.73	4.10	2	2	195	21.6	6.54
O95248	Myotubularin-related protein 5 OS=Homo sapiens GN=SBF1 PE=1 SV=3 - [MTMR5_HUMAN]	9.96	1	17	17	55	1.171	0.996	0.926	0.968	111.03	9.96	29	55	1867	208.2	6.90
Q9NUG4	Cerebral cavernous malformations 2 protein-like OS=Homo sapiens GN=CCM2L PE=2 SV=3 - [CCM2L_HUMAN]	2.98	1	1	1	1	0.556	0.904	1.175	0.968	2.51	2.98	1	1	571	62.1	5.95
P14543	Nidogen-1 OS=Homo sapiens GN=NID1 PE=1 SV=3 - [NID1_HUMAN]	4.33	1	4	5	7	0.895	1.060	0.841	0.968	14.33	4.33	7	7	1247	136.3	5.29
Q96TA1	Niban-like protein 1 OS=Homo sapiens GN=FAM129B PE=1 SV=3 - [NIBL1_HUMAN]	44.10	1	29	30	209	1.280	0.971	1.215	0.968	497.29	44.10	47	209	746	84.1	6.19

Q9Y3E7	Charged multivesicular body protein 3 OS=Homo sapiens GN=CHMP3 PE=1 SV=3 - [CHMP3_HUMAN]	6.31	1	2	2	9	0.884	1.027	1.004	0.968	15.93	6.31	3	9	222	25.1	5.12
Q6WKZ4	Rab11 family-interacting protein 1 OS=Homo sapiens GN=RAB11FIP1 PE=1 SV=3 - [RFIP1_HUMAN]	13.25	2	10	10	28	1.039	1.158	0.833	0.968	54.99	13.25	16	28	1283	137.1	5.43
Q96Q11	CCA tRNA nucleotidyltransferase 1, mitochondrial OS=Homo sapiens GN=TRNT1 PE=1 SV=2 - [TRNT1_HUMAN]	33.87	1	14	14	32	0.929	1.128	0.916	0.968	60.44	33.87	21	32	434	50.1	8.10
Q8NCW5	NAD(P)H-hydrate epimerase OS=Homo sapiens GN=APOA1BP PE=1 SV=2 - [NNRE_HUMAN]	22.92	1	5	5	26	1.162	1.035	1.153	0.968	43.41	22.92	7	26	288	31.7	7.66
Q96ME1	F-box/LRR-repeat protein 18 OS=Homo sapiens GN=FBXL18 PE=1 SV=2 - [FXL18_HUMAN]	5.22	1	4	4	12	0.788	0.745	1.078	0.968	24.29	5.22	6	12	805	88.3	8.32
Q12792	Twinfilin-1 OS=Homo sapiens GN=TWF1 PE=1 SV=3 - [TWF1_HUMAN]	32.86	1	10	11	58	1.075	1.114	1.014	0.968	136.39	32.86	19	58	350	40.3	6.96
Q9BXF6	Rab11 family-interacting protein 5 OS=Homo sapiens GN=RAB11FIP5 PE=1 SV=1 - [RFIP5_HUMAN]	23.28	1	10	10	39	0.928	0.982	1.290	0.968	102.99	23.28	16	39	653	70.4	9.23
P49773	Histidine triad nucleotide-binding protein 1 OS=Homo sapiens GN=HINT1 PE=1 SV=2 - [HINT1_HUMAN]	68.25	1	6	6	77	1.179	0.794	1.158	0.968	189.01	68.25	11	77	126	13.8	6.95
Q712K3	Ubiquitin-conjugating enzyme E2 R2 OS=Homo sapiens GN=UBE2R2 PE=1 SV=1 - [UB2R2_HUMAN]	29.41	1	6	7	28	0.961	1.047	0.935	0.969	63.32	29.41	10	28	238	27.1	4.42

P25208	Nuclear transcription factor Y subunit beta OS=Homo sapiens GN=NFYB PE=1 SV=2 - [NFYB_HUMAN]	6.28	1	1	1	7	0.673	1.358	0.700	0.969	18.88	6.28	2	7	207	22.8	4.59
Q9BTZ2	Dehydrogenase/reductase SDR family member 4 OS=Homo sapiens GN=DHRS4 PE=1 SV=3 - [DHRS4_HUMAN]	16.19	3	6	6	17	1.060	0.947	1.082	0.969	44.64	16.19	10	17	278	29.5	8.56
Q8IVL6	Prolyl 3-hydroxylase 3 OS=Homo sapiens GN=LEPREL2 PE=1 SV=1 - [P3H3_HUMAN]	16.98	1	10	10	33	0.738	1.109	1.093	0.969	92.46	16.98	16	33	736	81.8	6.32
Q8N6M0	OTU domain-containing protein 6B OS=Homo sapiens GN=OTUD6B PE=1 SV=1 - [OTU6B_HUMAN]	8.87	2	1	3	5	0.882	1.418	1.308	0.969	11.36	8.87	4	5	293	33.8	6.05
Q4G176	Acyl-CoA synthetase family member 3, mitochondrial OS=Homo sapiens GN=ACSF3 PE=1 SV=3 - [ACSF3_HUMAN	8.33	1	5	5	15	0.855	0.890	0.889	0.969	40.40	8.33	9	15	576	64.1	8.37
P19086	Guanine nucleotide-binding protein G(z) subunit alpha OS=Homo sapiens GN=GNAZ PE=2 SV=3 - [GNAZ_HUMAN]	4.23	1	1	2	6	1.014	0.479	0.565	0.969	9.47	4.23	3	6	355	40.9	7.61
P34932	Heat shock 70 kDa protein 4 OS=Homo sapiens GN=HSPA4 PE=1 SV=4 - [HSP74_HUMAN]	59.17	1	42	46	280	0.913	0.986	1.000	0.969	752.33	59.17	74	280	840	94.3	5.19
Q02543	60S ribosomal protein L18a OS=Homo sapiens GN=RPL18A PE=1 SV=2 - [RL18A_HUMAN]	31.82	1	6	6	51	0.811	0.892	0.856	0.969	147.10	31.82	12	51	176	20.7	10.71
Q12955	Ankyrin-3 OS=Homo sapiens GN=ANK3 PE=1 SV=3 - [ANK3_HUMAN]	0.94	1	3	4	6	1.202	1.068	1.190	0.969	12.44	0.94	5	6	4377	480.1	6.49

O14966	Ras-related protein Rab-7L1 OS=Homo sapiens GN=RAB29 PE=1 SV=1 - [RAB7L_HUMAN]	12.32	1	2	2	2	1.101	0.846	0.902	0.969	5.90	12.32	2	2	203	23.1	7.18
Q9NSY1	BMP-2-inducible protein kinase OS=Homo sapiens GN=BMP2K PE=1 SV=2 - [BMP2K_HUMAN]	6.98	1	7	8	26	0.849	1.295	1.142	0.969	49.19	6.98	15	26	1161	129.1	6.51
O15126	Secretory carrier-associated membrane protein 1 OS=Homo sapiens GN=SCAMP1 PE=1 SV=2 - [SCAM1_HUMA	31.07	1	7	7	21	0.875	0.981	0.776	0.969	51.43	31.07	10	21	338	37.9	7.42
Q8NHH9	Atlastin-2 OS=Homo sapiens GN=ATL2 PE=1 SV=2 - [ATLA2_HUMAN]	12.52	1	4	6	15	1.107	1.270	0.780	0.969	27.42	12.52	10	15	583	66.2	5.48
P61221	ATP-binding cassette sub-family E member 1 OS=Homo sapiens GN=ABCE1 PE=1 SV=1 - [ABCE1_HUMAN]	36.73	1	20	20	99	0.681	0.930	0.817	0.969	222.53	36.73	32	99	599	67.3	8.34
Q6YHK3	CD109 antigen OS=Homo sapiens GN=CD109 PE=1 SV=2 - [CD109_HUMAN]	3.67	1	5	5	8	0.993	1.218	1.168	0.969	16.43	3.67	5	8	1445	161.6	5.85
O43174	Cytochrome P450 26A1 OS=Homo sapiens GN=CYP26A1 PE=1 SV=2 - [CP26A_HUMAN]	2.82	1	1	1	2	0.812	1.054	0.774	0.969	0.00	2.82	1	2	497	56.2	8.69
Q5VYM1	Uncharacterized protein C9orf131 OS=Homo sapiens GN=C9orf131 PE=2 SV=3 - [CI131_HUMAN]	1.58	1	1	1	1	0.954	0.854	0.975	0.969	1.87	1.58	1	1	1079	117.6	7.36
Q9C040	Tripartite motif-containing protein 2 OS=Homo sapiens GN=TRIM2 PE=1 SV=1 - [TRIM2_HUMAN]	3.09	1	3	3	15	0.468	1.553	0.996	0.969	30.19	3.09	6	15	744	81.5	6.96

Q9H2S1	Small conductance calcium-activated potassium channel protein 2 OS=Homo sapiens GN=KCNN2 PE=1 SV=2 -	4.84	1	2	2	3	1.718	0.623	1.020	0.969	5.84	4.84	2	3	579	63.7	9.52
Q9UMZ2	Synergin gamma OS=Homo sapiens GN=SYNRG PE=1 SV=2 - [SYNRG_HUMAN]	3.73	1	4	5	14	1.076	0.986	1.274	0.969	25.25	3.73	6	14	1314	140.6	5.03
Q9NXS2	Glutaminyl-peptide cyclotransferase-like protein OS=Homo sapiens GN=QPCTL PE=1 SV=2 - [QPCTL_HUMAN]	10.21	1	5	5	13	0.778	0.718	0.767	0.969	20.69	10.21	9	13	382	42.9	9.82
Q96IJ6	Mannose-1-phosphate guanyltransferase alpha OS=Homo sapiens GN=GMPPA PE=1 SV=1 - [GMPPA_HUMAN]	22.86	1	8	8	25	1.157	1.137	0.998	0.970	66.97	22.86	13	25	420	46.3	7.21
Q6PK18	2-oxoglutarate and iron-dependent oxygenase domain-containing protein 3 OS=Homo sapiens GN=OGFOD3 PE=1 SV=2 - [OGFD3_HUMAN]	4.39	1	1	1	8	0.935	0.938	0.931	0.970	19.15	4.39	2	8	319	35.6	8.18
P38935	DNA-binding protein SMUBP-2 OS=Homo sapiens GN=IGHMBP2 PE=1 SV=3 - [SMBP2_HUMAN]	3.63	1	2	4	4	0.837	1.130	1.040	0.970	7.16	3.63	4	4	993	109.1	8.97
P80303	Nucleobindin-2 OS=Homo sapiens GN=NUCB2 PE=1 SV=2 - [NUCB2_HUMAN]	51.67	1	18	19	113	0.933	0.976	0.852	0.970	248.12	51.67	28	113	420	50.2	5.12
P01008	Antithrombin-III OS=Homo sapiens GN=SERPINC1 PE=1 SV=1 - [ANT3_HUMAN]	2.80	1	1	1	1	1.066	0.975	0.851	0.970	0.00	2.80	1	1	464	52.6	6.71

Q9UPQ3	Arf-GAP with GTPase, ANK repeat and PH domain-containing protein 1 OS=Homo sapiens GN=AGAP1 PE=1 SV=4 - [AGAP1_HUMAN]	3.85	2	1	3	3		0.219	0.709	0.970	2.65	3.85	3	3	857	94.4	7.94
P09543	2',3'-cyclic-nucleotide 3'-phosphodiesterase OS=Homo sapiens GN=CNP PE=1 SV=2 - [CN37_HUMAN]	48.46	2	23	24	114	1.481	1.229	1.029	0.970	303.27	48.46	39	114	421	47.5	9.07
Q6UW02	Cytochrome P450 20A1 OS=Homo sapiens GN=CYP20A1 PE=2 SV=1 - [CP20A_HUMAN]	4.76	1	2	2	4	1.252	0.700	0.594	0.970	7.62	4.76	4	4	462	52.4	6.21
Q13564	NEDD8-activating enzyme E1 regulatory subunit OS=Homo sapiens GN=NAE1 PE=1 SV=1 - [ULA1_HUMAN]	15.17	1	9	9	31	0.951	1.029	0.924	0.970	67.57	15.17	13	31	534	60.2	5.40
P17980	26S protease regulatory subunit 6A OS=Homo sapiens GN=PSMC3 PE=1 SV=3 - [PRS6A_HUMAN]	57.63	1	22	22	168	0.952	1.068	1.032	0.970	433.70	57.63	35	168	439	49.2	5.24
P62820	Ras-related protein Rab-1A OS=Homo sapiens GN=RAB1A PE=1 SV=3 - [RAB1A_HUMAN]	80.98	2	7	17	128	1.088	0.879	0.938	0.970	307.38	80.98	28	128	205	22.7	6.21
P04843	Dolichyl-diphosphooligosaccharide--protein glycosyltransferase subunit 1 OS=Homo sapiens GN=RPN1 PE=1 SV=1 - [RPN1_HUMAN]	45.80	1	29	29	412	0.814	0.871	0.808	0.971	993.78	45.80	50	412	607	68.5	6.38
Q969V6	MKL/myocardin-like protein 1 OS=Homo sapiens GN=MKL1 PE=1 SV=1 - [MKL1_HUMAN]	4.73	2	2	4	15	1.165	1.377	1.585	0.971	39.15	4.73	6	15	931	98.9	5.86

Q8NBJ9	SID1 transmembrane family member 2 OS=Homo sapiens GN=SIDT2 PE=1 SV=2 - [SIDT2_HUMAN	6.61	1	4	4	7	0.816	0.851	0.975	0.971	19.43	6.61	5	7	832	94.4	6.96
Q9UBN7	Histone deacetylase 6 OS=Homo sapiens GN=HDAC6 PE=1 SV=2 - [HDAC6_HUMAN]	1.40	1	2	2	5	1.247	0.718	0.667	0.971	13.11	1.40	3	5	1215	131.3	5.30
Q9HCE5	N6-adenosine-methyltransferase subunit METTL14 OS=Homo sapiens GN=METTL14 PE=1 SV=2 - [MET14_HUMAN]	5.92	1	3	3	7	1.209	0.969	0.743	0.971	14.41	5.92	5	7	456	52.1	6.21
P62942	Peptidyl-prolyl cis-trans isomerase FKBP1A OS=Homo sapiens GN=FKBP1A PE=1 SV=2 - [FKB1A_HUMAN]	59.26	1	4	4	117	1.099	1.292	1.430	0.971	340.89	59.26	6	117	108	11.9	8.16
Q9UI12	V-type proton ATPase subunit H OS=Homo sapiens GN=ATP6V1H PE=1 SV=1 - [VATH_HUMAN]	26.29	1	10	10	35	0.983	0.996	1.014	0.971	97.27	26.29	17	35	483	55.8	6.48
Q9UKZ4	Teneurin-1 OS=Homo sapiens GN=TENM1 PE=1 SV=2 - [TEN1_HUMAN]	0.88	1	1	2	2	1.384	1.381	1.762	0.971	1.74	0.88	2	2	2725	304.8	6.42
O00300	Tumor necrosis factor receptor superfamily member 11B OS=Homo sapiens GN=TNFRSF11B PE=1 SV=3 - [TR11B_HUMAN]	17.71	2	8	8	24	0.694	0.466	0.440	0.971	50.55	17.71	11	24	401	46.0	8.29
Q9H497	Torsin-3A OS=Homo sapiens GN=TOR3A PE=1 SV=1 - [TOR3A_HUMAN]	4.53	1	2	2	4	0.757	0.820	0.929	0.971	2.54	4.53	2	4	397	46.2	6.01
Q9P2E9	Ribosome-binding protein 1 OS=Homo sapiens GN=RRBP1 PE=1 SV=4 - [RRBP1_HUMAN]	67.94	3	82	84	1043	0.873	0.892	0.861	0.971	2436.23	67.94	139	1043	1410	152.4	8.60

P23396	40S ribosomal protein S3 OS=Homo sapiens GN=RPS3 PE=1 SV=2 - [RS3_HUMAN]	76.13	1	22	22	135	0.878	0.893	0.875	0.971	299.04	76.13	36	135	243	26.7	9.66
Q9BYT8	Neurolysin, mitochondrial OS=Homo sapiens GN=NLN PE=1 SV=1 - [NEUL_HUMAN]	24.72	1	16	16	54	1.015	1.103	0.988	0.972	145.61	24.72	22	54	704	80.6	6.64
Q0VDF9	Heat shock 70 kDa protein 14 OS=Homo sapiens GN=HSPA14 PE=1 SV=1 - [HSP7E_HUMAN]	13.56	1	6	6	12	0.782	0.757	0.813	0.972	34.25	13.56	8	12	509	54.8	5.59
Q8IZT6	Abnormal spindle-like microcephaly-associated protein OS=Homo sapiens GN=ASPM PE=1 SV=2 - [ASPM_HUMAN]	1.64	1	5	6	6	1.257	0.909	0.811	0.972	7.92	1.64	6	6	3477	409.5	10.45
Q5W111	SPRY domain-containing protein 7 OS=Homo sapiens GN=SPRYD7 PE=1 SV=2 - [SPRY7_HUMAN]	19.39	1	3	3	9	0.670	0.611	0.652	0.972	18.56	19.39	5	9	196	21.7	6.70
P61962	DDB1- and CUL4-associated factor 7 OS=Homo sapiens GN=DCAF7 PE=1 SV=1 - [DCAF7_HUMAN	20.18	1	6	6	13	1.191	1.245	1.381	0.972	25.25	20.18	10	13	342	38.9	5.52
Q9GZP9	Derlin-2 OS=Homo sapiens GN=DERL2 PE=1 SV=1 - [DERL2_HUMAN]	4.60	1	1	1	1	0.726	0.596	0.550	0.972	3.63	4.60	1	1	239	27.5	7.28
P09417	Dihydropteridine reductase OS=Homo sapiens GN=QDPR PE=1 SV=2 - [DHPR_HUMAN]	20.90	1	4	4	16	1.042	0.921	0.927	0.972	39.63	20.90	6	16	244	25.8	7.37
Q76FK4	Nucleolar protein 8 OS=Homo sapiens GN=NOL8 PE=1 SV=1 - [NOL8_HUMAN]	3.94	2	2	3	4	0.842	1.042	1.233	0.972	3.54	3.94	3	4	1167	131.5	7.09

Q96LZ7	Regulator of microtubule dynamics protein 2 OS=Homo sapiens GN=RMDN2 PE=1 SV=2 - [RMD2_HUMAN]	7.56	1	3	3	8	1.274	0.626	0.916	0.972	12.31	7.56	5	8	410	47.4	6.54
Q8IUZ5	5-phosphohydroxy-L-lysine phospho-lyase OS=Homo sapiens GN=PHYKPL PE=1 SV=1 - [AT2L2_HUMAN]	19.33	1	7	7	20	1.123	0.982	1.052	0.972	52.58	19.33	12	20	450	49.7	6.76
P08621	U1 small nuclear ribonucleoprotein 70 kDa OS=Homo sapiens GN=SNRNP70 PE=1 SV=2 - [RU17_HUMAN]	31.12	1	15	15	110	1.011	1.307	1.045	0.972	256.63	31.12	25	110	437	51.5	9.94
P0CW22	40S ribosomal protein S17-like OS=Homo sapiens GN=RPS17L PE=1 SV=1 - [RS17L_HUMAN]	60.00	1	11	11	42	0.994	1.222	0.978	0.972	108.89	60.00	16	42	135	15.5	9.85
P78396	Cyclin-A1 OS=Homo sapiens GN=CCNA1 PE=1 SV=1 - [CCNA1_HUMAN]	2.58	1	1	1	1	0.674	0.817	0.796	0.972	3.62	2.58	1	1	465	52.3	5.10
Q8WUF8	Protein FAM172A OS=Homo sapiens GN=FAM172A PE=2 SV=1 - [F172A_HUMAN]	8.65	1	3	3	5	0.650	1.007	1.017	0.972	7.63	8.65	4	5	416	47.9	6.09
Q9NYK5	39S ribosomal protein L39, mitochondrial OS=Homo sapiens GN=MRPL39 PE=1 SV=3 - [RM39_HUMAN]	17.46	1	6	6	9	1.032	0.985	0.783	0.972	19.60	17.46	8	9	338	38.7	7.65
Q9P218	Collagen alpha-1(XX) chain OS=Homo sapiens GN=COL20A1 PE=1 SV=4 - [COKA1_HUMAN]	1.40	1	1	1	1	0.926	0.845	1.010	0.972	0.00	1.40	1	1	1284	135.7	8.06
O60506	Heterogeneous nuclear ribonucleoprotein Q OS=Homo sapiens GN=SYNCRIP PE=1 SV=2 - [HNRPQ_HUMA	42.38	1	21	29	238	0.913	1.122	0.838	0.972	648.47	42.38	50	238	623	69.6	8.59

Q9Y6E2	Basic leucine zipper and W2 domain-containing protein 2 OS=Homo sapiens GN=BZW2 PE=1 SV=1 - [BZW2_HUMAN]	21.96	1	10	11	30	0.669	1.012	0.826	0.973	63.03	21.96	14	30	419	48.1	6.68
Q9P2K5	Myelin expression factor 2 OS=Homo sapiens GN=MYEF2 PE=1 SV=3 - [MYEF2_HUMAN]	8.17	1	5	5	6	1.387	1.425	2.166	0.973	14.16	8.17	6	6	600	64.1	8.75
O60934	Nibrin OS=Homo sapiens GN=NBN PE=1 SV=1 - [NBN_HUMAN]	14.19	1	8	9	20	0.841	0.980	0.863	0.973	39.30	14.19	10	20	754	84.9	6.90
O15541	RING finger protein 113A OS=Homo sapiens GN=RNF113A PE=1 SV=1 - [R113A_HUMAN]	16.03	1	5	6	8	0.848	1.004	0.769	0.973	9.63	16.03	6	8	343	38.8	5.69
O75122	CLIP-associating protein 2 OS=Homo sapiens GN=CLASP2 PE=1 SV=2 - [CLAP2_HUMAN]	7.57	1	9	9	18	0.788	1.014	1.150	0.973	44.94	7.57	12	18	1294	141.0	8.47
Q8IY47	Kelch repeat and BTB domain-containing protein 2 OS=Homo sapiens GN=KBTBD2 PE=1 SV=2 - [KBTB2_HUMAN	0.96	1	1	1	1	1.357	0.964	0.908	0.973	2.21	0.96	1	1	623	71.3	5.62
Q6ZT12	E3 ubiquitin-protein ligase UBR3 OS=Homo sapiens GN=UBR3 PE=2 SV=2 - [UBR3_HUMAN]	1.06	1	2	2	8	0.709	1.065	0.949	0.973	17.82	1.06	3	8	1888	212.3	6.10
Q12913	Receptor-type tyrosine-protein phosphatase eta OS=Homo sapiens GN=PTPRJ PE=1 SV=3 - [PTPRJ_HUMAN	0.90	1	1	1	4	1.578	0.480	0.985	0.973	9.70	0.90	2	4	1337	145.9	5.58
Q99447	Ethanolamine-phosphate cytidylyltransferase OS=Homo sapiens GN=PCYT2 PE=1 SV=1 - [PCY2_HUMAN]	28.28	1	10	10	49	1.118	0.935	1.322	0.973	109.21	28.28	17	49	389	43.8	6.92

Q9BPX6	Calcium uptake protein 1, mitochondrial OS=Homo sapiens GN=MICU1 PE=1 SV=1 - [MICU1_HUMAN]	15.34	1	8	8	21	0.764	0.715	0.915	0.973	48.29	15.34	13	21	476	54.3	8.41
P43307	Translocon-associated protein subunit alpha OS=Homo sapiens GN=SSR1 PE=1 SV=3 - [SSRA_HUMAN]	11.89	1	3	3	123	0.809	0.935	0.818	0.973	278.50	11.89	6	123	286	32.2	4.49
O95841	Angiopoietin-related protein 1 OS=Homo sapiens GN=ANGPTL1 PE=2 SV=1 - [ANGL1_HUMAN]	1.43	1	1	1	1	1.359	1.012	1.227	0.973	2.11	1.43	1	1	491	56.7	8.21
Q9H2H8	Peptidyl-prolyl cis-trans isomerase-like 3 OS=Homo sapiens GN=PPIL3 PE=1 SV=1 - [PPIL3_HUMAN]	47.83	1	8	8	23	1.172	0.866	0.768	0.973	45.56	47.83	13	23	161	18.1	6.79
Q9BYJ9	YTH domain-containing family protein 1 OS=Homo sapiens GN=YTHDF1 PE=1 SV=1 - [YTHD1_HUMAN]	4.11	1	1	2	13	0.952	0.765	0.798	0.974	22.41	4.11	4	13	559	60.8	8.79
Q9Y6K5	2'-5'-oligoadenylate synthase 3 OS=Homo sapiens GN=OAS3 PE=1 SV=3 - [OAS3_HUMAN]	4.78	1	5	5	13	7.193	1.554	1.029	0.974	31.50	4.78	8	13	1087	121.1	8.40
P02689	Myelin P2 protein OS=Homo sapiens GN=PMP2 PE=1 SV=3 - [MYP2_HUMAN]	28.79	2	3	4	7	1.215	1.155	1.186	0.974	8.47	28.79	5	7	132	14.9	9.83
Q15155	Nodal modulator 1 OS=Homo sapiens GN=NOMO1 PE=1 SV=5 - [NOMO1_HUMAN]	20.13	1	3	21	98	0.874	0.958	0.993	0.974	272.36	20.13	33	98	1222	134.2	5.81
Q8N5K1	CDGSH iron-sulfur domain-containing protein 2 OS=Homo sapiens GN=CISD2 PE=1 SV=1 - [CISD2_HUMAN]	40.00	1	5	5	19	0.888	0.875	0.977	0.974	34.90	40.00	8	19	135	15.3	9.61

Q9Y4I1	Unconventional myosin-Va OS=Homo sapiens GN=MYO5A PE=1 SV=2 - [MYO5A_HUMAN]	10.03	1	17	21	42	1.133	1.089	1.140	0.974	92.28	10.03	28	42	1855	215.3	8.48
P51659	Peroxisomal multifunctional enzyme type 2 OS=Homo sapiens GN=HSD17B4 PE=1 SV=3 - [DHB4_HUMAN]	41.44	2	28	28	162	1.070	1.015	1.247	0.974	390.69	41.44	45	162	736	79.6	8.84
Q8NB90	Spermatogenesis-associated protein 5 OS=Homo sapiens GN=SPATA5 PE=1 SV=3 - [SPAT5_HUMAN]	16.69	1	10	12	34	1.059	0.987	0.825	0.974	60.72	16.69	18	34	893	97.8	5.66
P07948	Tyrosine-protein kinase Lyn OS=Homo sapiens GN=LYN PE=1 SV=3 - [LYN_HUMAN]	14.06	7	4	7	25	0.806	1.341	1.096	0.974	50.33	14.06	11	25	512	58.5	7.11
O94822	E3 ubiquitin-protein ligase listerin OS=Homo sapiens GN=LTN1 PE=1 SV=6 - [LTN1_HUMAN]	2.77	1	6	6	9	0.789	0.833	0.839	0.974	16.19	2.77	7	9	1766	200.4	6.25
Q147X3	N-alpha-acetyltransferase 30 OS=Homo sapiens GN=NAA30 PE=1 SV=1 - [NAA30_HUMAN	17.40	1	4	4	6	0.982	0.901	1.129	0.975	22.26	17.40	5	6	362	39.3	5.52
Q9NQR7	Coiled-coil domain-containing protein 177 OS=Homo sapiens GN=CCDC177 PE=2 SV=3 - [CC177_HUMAN]	4.10	1	1	2	3	1.961	1.362	1.139	0.975	2.75	4.10	2	3	707	79.7	10.74
Q9Y678	Coatomer subunit gamma-1 OS=Homo sapiens GN=COPG1 PE=1 SV=1 - [COPG1_HUMAN]	51.72	1	36	38	180	0.969	0.872	0.892	0.975	465.50	51.72	59	180	874	97.7	5.47
Q9Y6I9	Testis-expressed sequence 264 protein OS=Homo sapiens GN=TEX264 PE=1 SV=1 - [TX264_HUMAN	11.18	1	3	3	9	1.026	0.803	0.985	0.975	11.20	11.18	6	9	313	34.2	4.86

P08034	Gap junction beta-1 protein OS=Homo sapiens GN=GJB1 PE=1 SV=1 - [CXB1_HUMAN]	6.01	1	1	1	1	1.459	1.988	0.361	0.975	2.87	6.01	1	1	283	32.0	8.94
P11055	Myosin-3 OS=Homo sapiens GN=MYH3 PE=1 SV=3 - [MYH3_HUMAN]	4.02	1	1	8	75	0.772	0.627	0.770	0.975	132.79	4.02	11	75	1940	223.8	5.81
O95714	E3 ubiquitin-protein ligase HERC2 OS=Homo sapiens GN=HERC2 PE=1 SV=2 - [HERC2_HUMAN]	2.19	1	8	8	19	0.757	0.836	0.820	0.975	29.64	2.19	9	19	4834	526.9	6.28
Q5QJ74	Tubulin-specific chaperone cofactor E-like protein OS=Homo sapiens GN=TBCEL PE=2 SV=2 - [TBCEL_HUMAN]	22.64	1	6	7	13	0.991	1.392	1.186	0.976	36.84	22.64	8	13	424	48.2	5.38
Q9H2G2	STE20-like serine/threonine-protein kinase OS=Homo sapiens GN=SLK PE=1 SV=1 - [SLK_HUMAN]	26.07	3	32	34	95	1.018	1.227	1.090	0.976	196.54	26.07	48	95	1235	142.6	5.15
Q9H3S7	Tyrosine-protein phosphatase non-receptor type 23 OS=Homo sapiens GN=PTPN23 PE=1 SV=1 - [PTN23_HUMAN]	18.28	1	25	25	61	1.199	0.918	1.024	0.976	130.06	18.28	40	61	1636	178.9	6.92
Q9UNL2	Translocon-associated protein subunit gamma OS=Homo sapiens GN=SSR3 PE=1 SV=1 - [SSRG_HUMAN]	18.38	1	4	4	66	0.849	0.898	0.839	0.976	253.25	18.38	5	66	185	21.1	9.61
Q68DH5	LMBR1 domain-containing protein 2 OS=Homo sapiens GN=LMBRD2 PE=1 SV=1 - [LMBD2_HUMAN]	1.58	1	1	1	2	0.942			0.976	5.31	1.58	1	2	695	81.1	7.50

P15170	Eukaryotic peptide chain release factor GTP-binding subunit ERF3A OS=Homo sapiens GN=GSPT1 PE=1 SV=1 - [ERF3A_HUMAN]	40.48	1	8	26	94	1.165	0.955	0.984	0.976	216.00	40.48	40	94	499	55.7	5.62
P61018	Ras-related protein Rab-4B OS=Homo sapiens GN=RAB4B PE=1 SV=1 - [RAB4B_HUMAN]	23.47	2	3	5	29	0.910	0.860	0.926	0.976	64.43	23.47	9	29	213	23.6	6.06
O00178	GTP-binding protein 1 OS=Homo sapiens GN=GTPBP1 PE=1 SV=3 - [GTPB1_HUMAN]	23.17	1	11	11	27	1.045	1.063	0.949	0.976	81.78	23.17	19	27	669	72.4	8.34
Q7L8J4	SH3 domain-binding protein 5-like OS=Homo sapiens GN=SH3BP5L PE=1 SV=1 - [3BP5L_HUMAN]	5.85	1	2	2	5	0.873	1.048	1.216	0.976	19.88	5.85	4	5	393	43.5	5.77
Q8WUD1	Ras-related protein Rab-2B OS=Homo sapiens GN=RAB2B PE=1 SV=1 - [RAB2B_HUMAN]	38.43	1	1	6	26	1.188	1.103	1.421	0.976	75.94	38.43	12	26	216	24.2	7.83
O75165	DnaJ homolog subfamily C member 13 OS=Homo sapiens GN=DNAJC13 PE=1 SV=5 - [DJC13_HUMAN]	23.85	1	46	46	118	1.024	0.960	0.906	0.976	301.73	23.85	64	118	2243	254.3	6.74
Q2Y0W8	Electroneutral sodium bicarbonate exchanger 1 OS=Homo sapiens GN=SLC4A8 PE=1 SV=1 - [S4A8_HUMAN]	1.56	2	1	2	2	1.138	1.112	1.208	0.976	4.72	1.56	2	2	1093	122.9	6.68
Q9UID3	Vacuolar protein sorting-associated protein 51 homolog OS=Homo sapiens GN=VPS51 PE=1 SV=2 - [VPS51_HUMAN]	9.08	1	6	6	11	0.729	1.202	1.227	0.976	23.41	9.08	9	11	782	86.0	6.47

P40926	Malate dehydrogenase, mitochondrial OS=Homo sapiens GN=MDH2 PE=1 SV=3 - [MDHM_HUMAN]	52.66	2	15	15	347	1.077	1.223	1.025	0.977	786.27	52.66	27	347	338	35.5	8.68
P62829	60S ribosomal protein L23 OS=Homo sapiens GN=RPL23 PE=1 SV=1 - [RL23_HUMAN]	50.00	1	6	6	67	0.887	0.907	0.810	0.977	147.18	50.00	11	67	140	14.9	10.51
Q9Y3C8	Ubiquitin-fold modifier-conjugating enzyme 1 OS=Homo sapiens GN=UFC1 PE=1 SV=3 - [UFC1_HUMAN]	14.97	1	4	4	10	0.870	1.149	0.715	0.977	21.59	14.97	4	10	167	19.4	7.40
P78318	Immunoglobulin-binding protein 1 OS=Homo sapiens GN=IGBP1 PE=1 SV=1 - [IGBP1_HUMAN]	19.76	1	7	7	15	1.244	1.069	1.161	0.977	38.61	19.76	11	15	339	39.2	5.38
P00505	Aspartate aminotransferase, mitochondrial OS=Homo sapiens GN=GOT2 PE=1 SV=3 - [AATM_HUMAN]	46.28	1	23	23	111	1.095	1.422	1.168	0.977	252.87	46.28	34	111	430	47.5	9.01
Q9HAB8	Phosphopantothenate--cysteine ligase OS=Homo sapiens GN=PPCS PE=1 SV=2 - [PPCS_HUMAN]	32.48	1	11	11	38	0.923	0.941	0.801	0.977	92.02	32.48	19	38	311	34.0	6.71
P30566	Adenylosuccinate lyase OS=Homo sapiens GN=ADSL PE=1 SV=2 - [PUR8_HUMAN]	17.36	1	8	8	17	0.805	1.317	1.023	0.977	44.81	17.36	11	17	484	54.9	7.11
O15240	Neurosecretory protein VGF OS=Homo sapiens GN=VGF PE=1 SV=2 - [VGF_HUMAN]	2.28	1	1	1	1	1.523	0.939	1.366	0.977	0.00	2.28	1	1	615	67.2	4.78
P18669	Phosphoglycerate mutase 1 OS=Homo sapiens GN=PGAM1 PE=1 SV=2 - [PGAM1_HUMAN]	76.77	1	11	21	758	1.226	0.836	1.000	0.977	2255.41	76.77	37	758	254	28.8	7.18

P51451	Tyrosine-protein kinase Blk OS=Homo sapiens GN=BLK PE=1 SV=3 - [BLK_HUMAN]	7.52	6	2	3	15	0.793	1.128	1.026	0.977	24.44	7.52	4	15	505	57.7	7.87
Q9Y4E1	WASH complex subunit FAM21C OS=Homo sapiens GN=FAM21C PE=1 SV=3 - [FA21C_HUMAN]	22.15	2	3	22	76	1.409	1.211	0.898	0.977	185.99	22.15	36	76	1318	144.6	4.79
Q9BQB6	Vitamin K epoxide reductase complex subunit 1 OS=Homo sapiens GN=VKORC1 PE=1 SV=1 - [VKOR1_HUMAN]	4.91	1	2	2	11	0.929	1.004	0.753	0.977	16.05	4.91	2	11	163	18.2	9.36
Q6ZMN8	Cyclin-I2 OS=Homo sapiens GN=CCNI2 PE=2 SV=1 - [CCNI2_HUMAN]	8.13	1	2	2	3	1.067	0.676	0.927	0.977	0.00	8.13	2	3	369	40.6	7.90
Q96F86	Enhancer of mRNA-decapping protein 3 OS=Homo sapiens GN=EDC3 PE=1 SV=1 - [EDC3_HUMAN]	14.17	1	6	7	19	1.051	0.901	1.127	0.978	45.03	14.17	11	19	508	56.0	7.11
Q9BTV6	Diphthamide biosynthesis protein 7 OS=Homo sapiens GN=DPH7 PE=1 SV=2 - [DPH7_HUMAN]	5.75	1	2	2	3	0.979	1.146	1.046	0.978	4.21	5.75	2	3	452	50.5	6.60
Q9BUT9	Protein FAM195A OS=Homo sapiens GN=FAM195A PE=1 SV=2 - [F195A_HUMAN]	3.75	1	1	1	2	0.987	1.201	0.682	0.978	2.16	3.75	1	2	160	17.8	9.41
O00299	Chloride intracellular channel protein 1 OS=Homo sapiens GN=CLIC1 PE=1 SV=4 - [CLIC1_HUMAN]	72.61	2	13	14	125	1.086	0.953	0.996	0.978	305.23	72.61	21	125	241	26.9	5.17
P49757	Protein numb homolog OS=Homo sapiens GN=NUMB PE=1 SV=2 - [NUMB_HUMAN]	23.96	1	13	16	51	1.204	1.020	1.262	0.978	104.32	23.96	27	51	651	70.8	8.51

Q6NUK4	Receptor expression-enhancing protein 3 OS=Homo sapiens GN=REEP3 PE=1 SV=1 - [REEP3_HUMAN]	8.63	1	2	2	4	1.278	1.157	2.639	0.978	9.38	8.63	3	4	255	29.2	9.57
Q99873	Protein arginine N-methyltransferase 1 OS=Homo sapiens GN=PRMT1 PE=1 SV=2 - [ANM1_HUMAN]	45.15	2	15	15	63	1.064	0.827	0.751	0.978	150.36	45.15	26	63	361	41.5	5.43
Q7Z460	CLIP-associating protein 1 OS=Homo sapiens GN=CLASP1 PE=1 SV=1 - [CLAP1_HUMAN]	11.96	1	19	19	58	0.946	0.915	0.992	0.978	127.45	11.96	26	58	1538	169.3	9.03
Q9BZE1	39S ribosomal protein L37, mitochondrial OS=Homo sapiens GN=MRPL37 PE=1 SV=2 - [RM37_HUMAN]	29.31	1	11	12	22	1.113	0.941	0.892	0.978	54.16	29.31	16	22	423	48.1	8.59
P23497	Nuclear autoantigen Sp-100 OS=Homo sapiens GN=SP100 PE=1 SV=3 - [SP100_HUMAN	13.88	2	11	14	49	1.411	1.274	1.404	0.978	101.88	13.88	22	49	879	100.4	8.22
Q15173	Serine/threonine-protein phosphatase 2A 56 kDa regulatory subunit beta isoform OS=Homo sapiens GN=PPP2R5B PE=1 SV=1 - [2A5B_HUMAN]	12.27	1	4	5	14	2.136	1.022	1.218	0.978	37.23	12.27	9	14	497	57.4	6.71
P20645	Cation-dependent mannose-6-phosphate receptor OS=Homo sapiens GN=M6PR PE=1 SV=1 - [MPRD_HUMAN]	21.66	1	4	5	28	0.834	0.823	0.943	0.978	74.72	21.66	7	28	277	31.0	5.83
P61981	14-3-3 protein gamma OS=Homo sapiens GN=YWHAG PE=1 SV=2 - [1433G_HUMAN]	73.28	2	11	17	310	1.276	0.987	1.082	0.979	837.60	73.28	27	310	247	28.3	4.89

Q8N1G4	Leucine-rich repeat-containing protein 47 OS=Homo sapiens GN=LRRC47 PE=1 SV=1 - [LRC47_HUMAN]	24.87	1	14	14	69	0.918	0.987	1.123	0.979	166.51	24.87	26	69	583	63.4	8.28
Q9UNI6	Dual specificity protein phosphatase 12 OS=Homo sapiens GN=DUSP12 PE=1 SV=1 - [DUS12_HUMAN]	6.18	1	2	2	3	0.914	1.076	1.206	0.979	2.12	6.18	2	3	340	37.7	6.84
P30048	Thioredoxin-dependent peroxide reductase, mitochondrial OS=Homo sapiens GN=PRDX3 PE=1 SV=3 - [PRDX3_HUMAN]	32.03	1	11	11	124	1.038	1.155	0.858	0.979	288.35	32.03	20	124	256	27.7	7.78
P16591	Tyrosine-protein kinase Fer OS=Homo sapiens GN=FER PE=1 SV=2 - [FER_HUMAN]	5.84	1	4	5	6	1.102	0.917	1.220	0.979	13.40	5.84	6	6	822	94.6	7.14
Q04760	Lactoylglutathione lyase OS=Homo sapiens GN=GLO1 PE=1 SV=4 - [LGUL_HUMAN]	37.50	1	7	7	28	1.148	0.946	0.910	0.979	80.04	37.50	11	28	184	20.8	5.31
Q8NCA5	Protein FAM98A OS=Homo sapiens GN=FAM98A PE=1 SV=1 - [FA98A_HUMAN]	26.40	1	10	13	50	0.867	1.002	1.059	0.979	146.69	26.40	22	50	519	55.4	9.03
Q6UUV9	CREB-regulated transcription coactivator 1 OS=Homo sapiens GN=CRTC1 PE=1 SV=2 - [CRTC1_HUMAN	1.58	1	1	1	3	0.864	1.459	1.315	0.979	8.01	1.58	2	3	634	67.3	6.05
P62906	60S ribosomal protein L10a OS=Homo sapiens GN=RPL10A PE=1 SV=2 - [RL10A_HUMAN]	45.62	1	13	13	203	0.768	1.019	0.894	0.979	426.86	45.62	21	203	217	24.8	9.94
P56589	Peroxisomal biogenesis factor 3 OS=Homo sapiens GN=PEX3 PE=1 SV=1 - [PEX3_HUMAN]	2.68	1	1	1	4	1.228	0.753	0.933	0.979	8.62	2.68	2	4	373	42.1	8.15

Q86VN1	Vacuolar protein-sorting-associated protein 36 OS=Homo sapiens GN=VPS36 PE=1 SV=1 - [VPS36_HUMAN]	12.18	1	4	5	11	1.057	0.835	0.913	0.979	25.64	12.18	8	11	386	43.8	7.20
Q8WXA9	Splicing regulatory glutamine/lysine-rich protein 1 OS=Homo sapiens GN=SREK1 PE=1 SV=1 - [SREK1_HUMAN]	8.27	1	3	3	8	1.059	1.102	1.094	0.979	20.85	8.27	4	8	508	59.3	10.39
P19474	E3 ubiquitin-protein ligase TRIM21 OS=Homo sapiens GN=TRIM21 PE=1 SV=1 - [RO52_HUMAN]	22.95	1	11	11	42	1.669	1.079	1.348	0.980	98.11	22.95	19	42	475	54.1	6.38
P31948	Stress-induced-phosphoprotein 1 OS=Homo sapiens GN=STIP1 PE=1 SV=1 - [STIP1_HUMAN]	68.88	1	52	52	294	0.923	1.100	1.019	0.980	663.48	68.88	76	294	543	62.6	6.80
Q96NW7	Leucine-rich repeat-containing protein 7 OS=Homo sapiens GN=LRRC7 PE=1 SV=1 - [LRRC7_HUMAN]	2.02	1	1	3	8	0.823	0.812	0.838	0.980	9.88	2.02	4	8	1537	172.5	6.81
O60268	Uncharacterized protein KIAA0513 OS=Homo sapiens GN=KIAA0513 PE=2 SV=1 - [K0513_HUMAN]	5.60	1	2	2	3	1.098	0.977	1.532	0.980	9.75	5.60	2	3	411	46.6	5.06
O95210	Starch-binding domain-containing protein 1 OS=Homo sapiens GN=STBD1 PE=1 SV=1 - [STBD1_HUMAN]	20.11	1	6	6	21	1.054	0.767	0.847	0.980	60.93	20.11	10	21	358	39.0	5.73
P49368	T-complex protein 1 subunit gamma OS=Homo sapiens GN=CCT3 PE=1 SV=4 - [TCPG_HUMAN]	62.39	1	36	37	266	1.017	1.066	0.995	0.980	564.85	62.39	61	266	545	60.5	6.49

Q9H871	Protein RMD5 homolog A OS=Homo sapiens GN=RMND5A PE=1 SV=1 - [RMD5A_HUMAN]	6.39	2	2	2	2	1.542	1.026	0.836	0.980	3.00	6.39	2	2	391	44.0	6.06
Q6B0B8	Tigger transposable element-derived protein 3 OS=Homo sapiens GN=TIGD3 PE=1 SV=1 - [TIGD3_HUMAN]	1.70	1	1	1	1	0.923	0.757	0.743	0.980	2.30	1.70	1	1	471	52.0	7.40
O95208	Epsin-2 OS=Homo sapiens GN=EPN2 PE=1 SV=3 - [EPN2_HUMAN]	13.10	1	7	7	16	1.134	0.851	0.780	0.980	35.69	13.10	9	16	641	68.4	7.52
Q9HCK1	DBF4-type zinc finger-containing protein 2 OS=Homo sapiens GN=ZDBF2 PE=1 SV=3 - [ZDBF2_HUMAN]	1.15	1	2	3	5	1.044	0.975	1.609	0.980	4.98	1.15	3	5	2354	265.5	6.16
P27824	Calnexin OS=Homo sapiens GN=CANX PE=1 SV=2 - [CALX_HUMAN]	35.64	1	23	25	304	0.993	0.819	0.865	0.980	864.98	35.64	38	304	592	67.5	4.60
Q8IUA7	ATP-binding cassette sub-family A member 9 OS=Homo sapiens GN=ABCA9 PE=1 SV=1 - [ABCA9_HUMAN]	2.77	1	2	2	2	1.829	1.200	1.254	0.980	2.46	2.77	2	2	1624	184.2	6.93
O43865	Putative adenosylhomocysteinase 2 OS=Homo sapiens GN=AHCYL1 PE=1 SV=2 - [SAHH2_HUMAN]	32.08	1	7	21	84	1.074	0.868	0.861	0.980	189.40	32.08	37	84	530	58.9	6.89
Q8IWJ2	GRIP and coiled-coil domain-containing protein 2 OS=Homo sapiens GN=GCC2 PE=1 SV=4 - [GCC2_HUMAN]	15.20	4	22	28	114	0.713	0.926	0.958	0.980	260.18	15.20	43	114	1684	195.8	5.14
P51149	Ras-related protein Rab-7a OS=Homo sapiens GN=RAB7A PE=1 SV=1 - [RAB7A_HUMAN]	76.81	1	16	16	126	1.021	0.815	0.921	0.980	245.68	76.81	26	126	207	23.5	6.70

Q9H3Y8	Pancreatic progenitor cell differentiation and proliferation factor OS=Homo sapiens GN=PPDPF PE=2 SV=1 - [PPDPF_HUMAN]	14.04	1	1	1	4	0.968	3.197	1.527	0.980	5.56	14.04	2	4	114	11.8	7.42
Q9H7E9	UPF0488 protein C8orf33 OS=Homo sapiens GN=C8orf33 PE=1 SV=1 - [CH033_HUMAN]	18.34	1	4	4	9	1.189	1.026	1.281	0.980	15.63	18.34	6	9	229	25.0	9.95
P48048	ATP-sensitive inward rectifier potassium channel 1 OS=Homo sapiens GN=KCNJ1 PE=1 SV=1 - [KCNJ1_HUMAN]	1.53	1	1	1	1	1.405	0.803	0.598	0.981	1.96	1.53	1	1	391	44.8	8.82
Q9NVE7	Pantothenate kinase 4 OS=Homo sapiens GN=PANK4 PE=1 SV=1 - [PANK4_HUMAN]	21.22	1	14	14	38	1.128	0.904	1.066	0.981	73.87	21.22	22	38	773	85.9	6.28
P49821	NADH dehydrogenase [ubiquinone] flavoprotein 1, mitochondrial OS=Homo sapiens GN=NDUFV1 PE=1 SV=4 - [NDUV1_HUMAN]	27.80	1	12	12	43	0.660	0.985	0.701	0.981	102.10	27.80	21	43	464	50.8	8.21
P19256	Lymphocyte function-associated antigen 3 OS=Homo sapiens GN=CD58 PE=1 SV=1 - [LFA3_HUMAN]	4.00	1	1	1	3	0.796	0.966	1.019	0.981	5.57	4.00	2	3	250	28.1	6.76
Q6ZVK8	8-oxo-dGDP phosphatase NUDT18 OS=Homo sapiens GN=NUDT18 PE=1 SV=3 - [NUD18_HUMAN]	9.91	1	3	3	5	0.610	0.927	0.834	0.981	13.65	9.91	4	5	323	35.5	6.23
Q9NRX4	14 kDa phosphohistidine phosphatase OS=Homo sapiens GN=PHPT1 PE=1 SV=1 - [PHP14_HUMAN]	52.80	1	6	6	30	1.219	1.276	1.113	0.981	100.51	52.80	8	30	125	13.8	6.07

Q14624	Inter-alpha-trypsin inhibitor heavy chain H4 OS=Homo sapiens GN=ITIH4 PE=1 SV=4 - [ITIH4_HUMAN]	1.94	1	2	2	5	0.494	0.975	0.656	0.982	8.18	1.94	4	5	930	103.3	6.98
P21964	Catechol O-methyltransferase OS=Homo sapiens GN=COMT PE=1 SV=2 - [COMT_HUMAN]	45.39	1	12	12	98	1.298	0.814	0.776	0.982	315.43	45.39	18	98	271	30.0	5.47
Q15139	Serine/threonine-protein kinase D1 OS=Homo sapiens GN=PRKD1 PE=1 SV=2 - [KPCD1_HUMAN	8.88	2	5	7	15	0.900	0.728	0.966	0.982	22.52	8.88	10	15	912	101.6	6.62
P11182	Lipoamide acyltransferase component of branched-chain alpha-keto acid dehydrogenase complex, mitochondrial OS=Homo sapiens GN=DBT PE=1 SV=3 - [ODB2_HUMAN]	13.90	1	7	7	21	1.054	1.156	1.020	0.982	31.66	13.90	10	21	482	53.5	8.51
Q7Z2E3	Aprataxin OS=Homo sapiens GN=APTX PE=1 SV=2 - [APTX_HUMAN]	2.25	1	1	1	2	1.892	1.441	1.081	0.982	0.00	2.25	1	2	356	40.7	9.17
Q15645	Pachytene checkpoint protein 2 homolog OS=Homo sapiens GN=TRIP13 PE=1 SV=2 - [PCH2_HUMAN]	2.08	1	1	1	2	1.559	1.566	0.907	0.982	1.76	2.08	2	2	432	48.5	6.09
Q9NVT9	Armadillo repeat-containing protein 1 OS=Homo sapiens GN=ARMC1 PE=1 SV=1 - [ARMC1_HUMA	5.67	1	3	3	7	0.951	1.330	1.307	0.982	16.85	5.67	5	7	282	31.3	5.74
P42345	Serine/threonine-protein kinase mTOR OS=Homo sapiens GN=MTOR PE=1 SV=1 - [MTOR_HUMAN]	7.89	1	18	19	36	1.046	0.929	0.862	0.983	76.03	7.89	27	36	2549	288.7	7.17

P61247	40S ribosomal protein S3a OS=Homo sapiens GN=RPS3A PE=1 SV=2 - [RS3A_HUMAN]	53.03	1	22	22	263	0.865	0.948	0.906	0.983	625.45	53.03	35	263	264	29.9	9.73
Q15935	Zinc finger protein 77 OS=Homo sapiens GN=ZNF77 PE=2 SV=2 - [ZNF77_HUMAN]	6.79	28	1	2	4	1.771	1.143	1.282	0.983	2.82	6.79	2	4	545	61.9	8.69
Q9P0M9	39S ribosomal protein L27, mitochondrial OS=Homo sapiens GN=MRPL27 PE=1 SV=1 - [RM27_HUMAN]	31.08	1	4	4	17	1.043	0.745	0.875	0.983	37.18	31.08	7	17	148	16.1	10.42
Q03001	Dystonin OS=Homo sapiens GN=DST PE=1 SV=4 - [DYST_HUMAN]	13.22	1	86	91	222	0.876	0.949	0.963	0.983	502.91	13.22	133	222	7570	860.1	5.25
Q9Y376	Calcium-binding protein 39 OS=Homo sapiens GN=CAB39 PE=1 SV=1 - [CAB39_HUMAN]	32.55	1	10	11	42	1.049	0.791	0.800	0.983	96.97	32.55	17	42	341	39.8	6.89
Q96PE2	Rho guanine nucleotide exchange factor 17 OS=Homo sapiens GN=ARHGEF17 PE=1 SV=1 - [ARHGH_HUMAN	8.82	1	14	16	26	1.099	1.293	1.295	0.983	59.34	8.82	20	26	2063	221.5	6.29
Q15018	BRISC complex subunit Abro1 OS=Homo sapiens GN=FAM175B PE=1 SV=2 - [F175B_HUMAN]	23.37	1	8	8	17	1.055	0.927	0.885	0.983	38.07	23.37	10	17	415	46.9	6.21
P04181	Ornithine aminotransferase, mitochondrial OS=Homo sapiens GN=OAT PE=1 SV=1 - [OAT_HUMAN]	47.61	1	17	19	64	0.904	0.990	1.054	0.983	164.87	47.61	28	64	439	48.5	7.03
Q7Z401	C-myc promoter-binding protein OS=Homo sapiens GN=DENND4A PE=1 SV=2 - [MYCPP_HUMAN]	3.06	1	5	5	7	1.210	0.890	0.861	0.983	11.55	3.06	6	7	1863	209.1	7.31

Q16543	Hsp90 co-chaperone Cdc37 OS=Homo sapiens GN=CDC37 PE=1 SV=1 - [CDC37_HUMAN]	44.71	1	18	18	98	0.851	1.109	1.285	0.984	221.24	44.71	30	98	378	44.4	5.25
Q5VZE5	N-alpha-acetyltransferase 35, NatC auxiliary subunit OS=Homo sapiens GN=NAA35 PE=1 SV=1 - [NAA35_HUMAN]	16.97	1	11	11	25	0.746	0.963	1.055	0.984	61.64	16.97	18	25	725	83.6	7.05
Q9NVM4	Protein arginine N-methyltransferase 7 OS=Homo sapiens GN=PRMT7 PE=1 SV=1 - [ANM7_HUMAN]	2.75	1	1	2	2	0.961	0.997	0.732	0.984	4.95	2.75	2	2	692	78.4	5.57
Q9C0I1	Myotubularin-related protein 12 OS=Homo sapiens GN=MTMR12 PE=1 SV=2 - [MTMRC_HUMAN]	8.43	1	5	5	7	1.087	1.188	1.201	0.984	10.82	8.43	5	7	747	86.1	6.62
Q8NGR3	Olfactory receptor 1K1 OS=Homo sapiens GN=OR1K1 PE=1 SV=1 - [OR1K1_HUMAN]	2.22	1	1	1	1	1.151	0.882	0.914	0.984	1.93	2.22	1	1	316	34.2	7.93
Q9NUY8	TBC1 domain family member 23 OS=Homo sapiens GN=TBC1D23 PE=1 SV=3 - [TBC23_HUMAN]	5.72	1	4	4	12	0.816	0.720	0.858	0.984	23.66	5.72	6	12	699	78.3	5.41
P83731	60S ribosomal protein L24 OS=Homo sapiens GN=RPL24 PE=1 SV=1 - [RL24_HUMAN]	49.68	1	11	11	85	0.801	0.803	0.860	0.984	204.87	49.68	16	85	157	17.8	11.25
P53667	LIM domain kinase 1 OS=Homo sapiens GN=LIMK1 PE=1 SV=3 - [LIMK1_HUMAN]	4.79	1	3	3	7	0.863	0.889	0.960	0.984	11.41	4.79	5	7	647	72.5	6.98
O95970	Leucine-rich glioma-inactivated protein 1 OS=Homo sapiens GN=LGI1 PE=1 SV=1 - [LGI1_HUMAN]	1.62	1	1	1	1	1.373	0.909	1.137	0.984	1.85	1.62	1	1	557	63.8	8.24

O60313	Dynamin-like 120 kDa protein, mitochondrial OS=Homo sapiens GN=OPA1 PE=1 SV=3 - [OPA1_HUMAN]	37.71	1	29	29	113	1.002	1.141	0.949	0.984	288.72	37.71	46	113	960	111.6	7.87
Q96F07	Cytoplasmic FMR1-interacting protein 2 OS=Homo sapiens GN=CYFIP2 PE=1 SV=2 - [CYFP2_HUMAN]	10.09	1	1	13	43	0.792	1.109	0.826	0.984	99.11	10.09	20	43	1278	148.3	7.31
Q9UBQ7	Glyoxylate reductase/hydroxypyruvate reductase OS=Homo sapiens GN=GRHPR PE=1 SV=1 - [GRHPR_HUMAN]	32.32	1	8	8	47	1.216	0.834	0.944	0.984	117.80	32.32	14	47	328	35.6	7.39
Q8IZ52	Chondroitin sulfate synthase 2 OS=Homo sapiens GN=CHPF PE=1 SV=2 - [CHSS2_HUMAN	4.90	1	3	3	10	0.651	0.621	1.029	0.985	28.19	4.90	6	10	775	85.4	6.93
O75431	Metaxin-2 OS=Homo sapiens GN=MTX2 PE=1 SV=1 - [MTX2_HUMAN]	26.24	1	5	5	17	1.027	0.675	0.739	0.985	50.75	26.24	8	17	263	29.7	6.29
Q9NRY4	Rho GTPase-activating protein 35 OS=Homo sapiens GN=ARHGAP35 PE=1 SV=3 - [RHG35_HUMAN]	8.61	1	11	12	25	0.993	0.817	0.995	0.985	47.08	8.61	20	25	1499	170.4	6.64
Q9H1Z4	WD repeat-containing protein 13 OS=Homo sapiens GN=WDR13 PE=1 SV=2 - [WDR13_HUMAN]	17.53	1	7	7	23	0.956	1.073	1.163	0.985	58.12	17.53	10	23	485	53.7	9.14
Q13107	Ubiquitin carboxyl-terminal hydrolase 4 OS=Homo sapiens GN=USP4 PE=1 SV=3 - [UBP4_HUMAN]	11.84	1	7	8	24	1.076	1.104	1.059	0.985	71.18	11.84	12	24	963	108.5	5.71

Q9NRP2	COX assembly mitochondrial protein 2 homolog OS=Homo sapiens GN=CMC2 PE=1 SV=1 - [COXM2_HUMAN]	12.66	1	1	1	2	1.112	1.235	0.989	0.985	4.90	12.66	1	2	79	9.5	7.87
Q9NV96	Cell cycle control protein 50A OS=Homo sapiens GN=TMEM30A PE=1 SV=1 - [CC50A_HUMAN]	16.90	1	7	7	20	1.198	0.798	0.922	0.985	44.99	16.90	11	20	361	40.7	8.59
O43678	NADH dehydrogenase [ubiquinone] 1 alpha subcomplex subunit 2 OS=Homo sapiens GN=NDUFA2 PE=1 SV=3 - [NDUA2_HUMAN]	49.49	1	5	5	19	0.517	0.916	0.789	0.985	36.66	49.49	9	19	99	10.9	9.57
O43164	E3 ubiquitin-protein ligase Praja-2 OS=Homo sapiens GN=PJA2 PE=1 SV=4 - [PJA2_HUMAN]	5.79	1	3	3	10	0.847	1.010	1.343	0.985	20.63	5.79	6	10	708	78.2	4.39
P32119	Peroxiredoxin-2 OS=Homo sapiens GN=PRDX2 PE=1 SV=5 - [PRDX2_HUMAN]	45.96	1	12	13	218	0.992	1.156	1.026	0.985	412.25	45.96	23	218	198	21.9	5.97
O60312	Probable phospholipid-transporting ATPase VA OS=Homo sapiens GN=ATP10A PE=2 SV=2 - [AT10A_HUMAN]	1.47	1	1	2	3	0.997	1.395	0.913	0.986	2.56	1.47	2	3	1499	167.6	8.37
P49755	Transmembrane emp24 domain-containing protein 10 OS=Homo sapiens GN=TMED10 PE=1 SV=2 - [TMEDA_HUMA	49.32	1	12	12	149	0.967	1.123	1.018	0.986	363.74	49.32	20	149	219	25.0	7.44
P48643	T-complex protein 1 subunit epsilon OS=Homo sapiens GN=CCT5 PE=1 SV=1 - [TCPE_HUMAN]	55.27	1	32	33	181	0.896	1.064	1.002	0.986	420.38	55.27	55	181	541	59.6	5.66

Q15545	Transcription initiation factor TFIID subunit 7 OS=Homo sapiens GN=TAF7 PE=1 SV=1 - [TAF7_HUMAN]	5.44	1	2	2	3	0.754	0.755	1.139	0.986	5.37	5.44	3	3	349	40.2	5.20
Q8N2K1	Ubiquitin-conjugating enzyme E2 J2 OS=Homo sapiens GN=UBE2J2 PE=1 SV=3 - [UB2J2_HUMAN]	5.79	1	1	1	4	0.899	0.570	0.701	0.986	14.13	5.79	2	4	259	28.9	8.40
Q6ZQQ6	WD repeat-containing protein 87 OS=Homo sapiens GN=WDR87 PE=1 SV=3 - [WDR87_HUMAN]	1.67	1	4	4	4	1.062	1.005	1.289	0.986	6.94	1.67	4	4	2873	333.0	7.28
Q9P0K7	Ankycorbin OS=Homo sapiens GN=RAI14 PE=1 SV=2 - [RAI14_HUMAN]	63.47	1	66	70	356	1.082	0.836	1.134	0.986	870.60	63.47	112	356	980	110.0	6.21
P35251	Replication factor C subunit 1 OS=Homo sapiens GN=RFC1 PE=1 SV=4 - [RFC1_HUMAN]	1.92	1	3	3	8	0.975	1.294	1.074	0.986	17.42	1.92	4	8	1148	128.2	9.36
Q5J8M3	ER membrane protein complex subunit 4 OS=Homo sapiens GN=EMC4 PE=1 SV=2 - [EMC4_HUMAN]	22.40	1	3	3	10	0.965	0.909	0.873	0.986	19.72	22.40	6	10	183	20.1	8.62
Q9BV29	Uncharacterized protein C15orf57 OS=Homo sapiens GN=C15orf57 PE=1 SV=2 - [CO057_HUMAN]	4.86	1	1	1	2	0.923	0.399	0.610	0.986	2.79	4.86	2	2	185	20.6	4.53
P50479	PDZ and LIM domain protein 4 OS=Homo sapiens GN=PDLIM4 PE=1 SV=2 - [PDLI4_HUMAN]	61.82	1	15	15	158	0.883	1.029	1.377	0.986	317.77	61.82	28	158	330	35.4	7.91
Q96RQ3	Methylcrotonoyl-CoA carboxylase subunit alpha, mitochondrial OS=Homo sapiens GN=MCCC1 PE=1 SV=3 - [MCCA_HUMAN]	15.72	1	8	8	17	0.898	1.368	1.086	0.986	52.32	15.72	11	17	725	80.4	7.78

Q13242	Serine/arginine-rich splicing factor 9 OS=Homo sapiens GN=SRSF9 PE=1 SV=1 - [SRSF9_HUMAN]	34.84	1	7	7	45	0.888	1.075	0.807	0.986	101.22	34.84	13	45	221	25.5	8.65
P31939	Bifunctional purine biosynthesis protein PURH OS=Homo sapiens GN=ATIC PE=1 SV=3 - [PUR9_HUMAN]	74.32	1	32	33	186	1.020	0.923	0.903	0.986	517.99	74.32	54	186	592	64.6	6.71
P17987	T-complex protein 1 subunit alpha OS=Homo sapiens GN=TCP1 PE=1 SV=1 - [TCPA_HUMAN]	53.96	1	27	28	248	0.904	0.878	0.941	0.986	601.78	53.96	47	248	556	60.3	6.11
O94985	Calsyntenin-1 OS=Homo sapiens GN=CLSTN1 PE=1 SV=1 - [CSTN1_HUMAN]	1.02	1	1	1	2	0.850	0.880	0.970	0.987	6.33	1.02	1	2	981	109.7	4.91
Q9H0E2	Toll-interacting protein OS=Homo sapiens GN=TOLLIP PE=1 SV=1 - [TOLIP_HUMAN]	22.99	1	6	6	20	1.168	1.033	0.984	0.987	38.49	22.99	10	20	274	30.3	5.97
A0PJK1	Sodium/glucose cotransporter 5 OS=Homo sapiens GN=SLC5A10 PE=1 SV=2 - [SC5AA_HUMAN]	2.35	1	1	1	2	0.377	0.413	0.562	0.987	0.00	2.35	1	2	596	64.3	7.61
O00186	Syntaxin-binding protein 3 OS=Homo sapiens GN=STXBP3 PE=1 SV=2 - [STXB3_HUMAN]	33.78	1	16	16	65	1.285	1.044	1.051	0.987	197.22	33.78	24	65	592	67.7	7.80
Q9H1X3	DnaJ homolog subfamily C member 25 OS=Homo sapiens GN=DNAJC25 PE=2 SV=1 - [DJC25_HUMAN]	8.61	1	3	3	4	0.924	0.946	0.978	0.987	9.96	8.61	3	4	360	42.4	9.13
Q15434	RNA-binding motif, single-stranded-interacting protein 2 OS=Homo sapiens GN=RBMS2 PE=1 SV=1 - [RBMS2_HUMAN]	18.92	1	5	7	26	1.622	0.995	1.133	0.987	64.34	18.92	12	26	407	43.9	9.07

P29353	SHC-transforming protein 1 OS=Homo sapiens GN=SHC1 PE=1 SV=4 - [SHC1_HUMAN]	24.01	2	11	11	26	0.906	0.895	0.993	0.987	63.62	24.01	13	26	583	62.8	6.44
Q03154	Aminoacylase-1 OS=Homo sapiens GN=ACY1 PE=1 SV=1 - [ACY1_HUMAN]	27.70	1	10	10	24	0.940	0.844	0.750	0.987	73.25	27.70	16	24	408	45.9	6.18
Q14671	Pumilio homolog 1 OS=Homo sapiens GN=PUM1 PE=1 SV=3 - [PUM1_HUMAN]	15.85	2	12	16	41	0.963	0.934	1.033	0.987	134.34	15.85	22	41	1186	126.4	6.84
P14625	Endoplasmin OS=Homo sapiens GN=HSP90B1 PE=1 SV=1 - [ENPL_HUMAN]	63.51	2	63	65	1360	0.814	1.030	0.939	0.987	3518.18	63.51	109	1360	803	92.4	4.84
Q96S52	GPI transamidase component PIG-S OS=Homo sapiens GN=PIGS PE=1 SV=3 - [PIGS_HUMAN]	17.48	1	8	8	26	0.894	1.003	0.913	0.987	60.30	17.48	13	26	555	61.6	6.49
Q8TCY9	Up-regulator of cell proliferation OS=Homo sapiens GN=URGCP PE=1 SV=2 - [URGCP_HUMAN	0.97	1	1	1	1	1.126	0.819	0.995	0.987	0.00	0.97	1	1	931	104.9	6.42
Q13049	E3 ubiquitin-protein ligase TRIM32 OS=Homo sapiens GN=TRIM32 PE=1 SV=2 - [TRI32_HUMAN]	13.17	1	7	7	21	1.047	0.780	0.963	0.987	55.21	13.17	9	21	653	71.9	6.98
P49257	Protein ERGIC-53 OS=Homo sapiens GN=LMAN1 PE=1 SV=2 - [LMAN1_HUMAN]	44.12	1	21	22	158	0.897	0.879	0.791	0.988	398.24	44.12	38	158	510	57.5	6.77
Q96H20	Vacuolar-sorting protein SNF8 OS=Homo sapiens GN=SNF8 PE=1 SV=1 - [SNF8_HUMAN]	8.91	1	2	2	4	1.092	1.108	0.848	0.988	15.39	8.91	3	4	258	28.8	6.65
P24534	Elongation factor 1-beta OS=Homo sapiens GN=EEF1B2 PE=1 SV=3 - [EF1B_HUMAN]	58.22	1	11	15	273	0.895	0.901	0.893	0.988	886.90	58.22	23	273	225	24.7	4.67

P53680	AP-2 complex subunit sigma OS=Homo sapiens GN=AP2S1 PE=1 SV=2 - [AP2S1_HUMAN]	27.46	1	3	3	13	1.536	0.828	0.697	0.988	36.03	27.46	4	13	142	17.0	6.18
Q14919	Dr1-associated corepressor OS=Homo sapiens GN=DRAP1 PE=1 SV=3 - [NC2A_HUMAN]	20.98	1	4	4	10	0.733	0.728	0.795	0.988	21.53	20.98	8	10	205	22.3	5.17
Q9BZL1	Ubiquitin-like protein 5 OS=Homo sapiens GN=UBL5 PE=1 SV=1 - [UBL5_HUMAN]	27.40	1	2	2	13	0.871	0.892	1.258	0.988	29.36	27.40	4	13	73	8.5	8.44
O43822	Protein C21orf2 OS=Homo sapiens GN=C21orf2 PE=1 SV=1 - [CU002_HUMAN]	2.73	1	1	1	3	0.985	1.052	1.268	0.988	3.98	2.73	2	3	256	28.3	7.34
O75157	TSC22 domain family protein 2 OS=Homo sapiens GN=TSC22D2 PE=1 SV=3 - [T22D2_HUMAN]	6.15	1	3	6	21	0.911	0.847	1.097	0.988	46.63	6.15	9	21	780	79.2	5.02
Q9NR46	Endophilin-B2 OS=Homo sapiens GN=SH3GLB2 PE=1 SV=1 - [SHLB2_HUMAN]	29.11	1	11	12	37	1.137	1.344	1.471	0.988	86.14	29.11	19	37	395	43.9	5.99
O15235	28S ribosomal protein S12, mitochondrial OS=Homo sapiens GN=MRPS12 PE=1 SV=1 - [RT12_HUMAN]	8.70	1	2	2	3	1.120	1.517	0.944	0.988	2.03	8.70	2	3	138	15.2	10.29
Q9BPW8	Protein NipSnap homolog 1 OS=Homo sapiens GN=NIPSNAP1 PE=1 SV=1 - [NIPS1_HUMAN]	39.08	1	9	12	39	1.109	1.459	1.216	0.989	64.52	39.08	17	39	284	33.3	9.31
P27348	14-3-3 protein theta OS=Homo sapiens GN=YWHAQ PE=1 SV=1 - [1433T_HUMAN	77.96	2	17	23	247	1.256	1.312	1.092	0.989	777.24	77.96	36	247	245	27.7	4.78
Q5T011	Protein SZT2 OS=Homo sapiens GN=SZT2 PE=2 SV=3 - [SZT2_HUMAN]	1.25	1	3	4	6	1.090	1.231	1.237	0.989	15.71	1.25	4	6	3432	377.8	6.27

Q9NZM1	Myoferlin OS=Homo sapiens GN=MYOF PE=1 SV=1 - [MYOF_HUMAN]	56.43	1	111	113	997	0.852	0.933	0.994	0.989	2618.52	56.43	189	997	2061	234.6	6.18
Q14831	Metabotropic glutamate receptor 7 OS=Homo sapiens GN=GRM7 PE=1 SV=1 - [GRM7_HUMAN]	7.21	1	3	3	5	0.892	0.904	0.967	0.989	1.74	7.21	3	5	915	102.2	7.88
P78368	Casein kinase I isoform gamma-2 OS=Homo sapiens GN=CSNK1G2 PE=1 SV=1 - [KC1G2_HUMAN]	4.34	3	2	2	6	1.159	0.747	0.791	0.989	9.52	4.34	4	6	415	47.4	9.06
Q9NQ03	Transcriptional repressor scratch 2 OS=Homo sapiens GN=SCRT2 PE=2 SV=3 - [SCRT2_HUMAN]	10.42	1	1	1	1	1.196	1.989	2.370	0.989	3.53	10.42	1	1	307	32.6	9.22
Q96PU4	E3 ubiquitin-protein ligase UHRF2 OS=Homo sapiens GN=UHRF2 PE=1 SV=1 - [UHRF2_HUMAN]	4.99	1	3	3	8	0.958	1.155	1.054	0.989	17.63	4.99	5	8	802	89.9	8.21
Q14011	Cold-inducible RNA-binding protein OS=Homo sapiens GN=CIRBP PE=1 SV=1 - [CIRBP_HUMAN]	39.53	1	7	7	20	1.089	1.068	1.058	0.989	54.05	39.53	11	20	172	18.6	9.51
Q9NR48	Histone-lysine N-methyltransferase ASH1L OS=Homo sapiens GN=ASH1L PE=1 SV=2 - [ASH1L_HUMAN]	1.01	1	3	4	4	0.560	0.568	0.569	0.989	7.31	1.01	4	4	2969	332.6	9.39
Q9UNN5	FAS-associated factor 1 OS=Homo sapiens GN=FAF1 PE=1 SV=2 - [FAF1_HUMAN]	13.08	1	9	9	22	0.965	1.157	1.052	0.990	55.35	13.08	11	22	650	73.9	4.88
Q9Y3B4	Splicing factor 3B subunit 6 OS=Homo sapiens GN=SF3B6 PE=1 SV=1 - [SF3B6_HUMAN]	28.00	1	3	3	7	1.031	1.593	0.963	0.990	21.99	28.00	5	7	125	14.6	9.38

Q9H4A4	Aminopeptidase B OS=Homo sapiens GN=RNPEP PE=1 SV=2 - [AMPB_HUMAN]	43.08	1	23	23	166	1.061	0.891	0.926	0.990	394.91	43.08	42	166	650	72.5	5.74
P62826	GTP-binding nuclear protein Ran OS=Homo sapiens GN=RAN PE=1 SV=3 - [RAN_HUMAN]	25.00	1	9	9	59	1.000	1.219	0.821	0.990	156.68	25.00	14	59	216	24.4	7.49
Q9C0B7	Transport and Golgi organization protein 6 homolog OS=Homo sapiens GN=TANGO6 PE=1 SV=2 - [TNG6_HUMAN]	4.39	1	3	3	3	0.912	0.516	0.837	0.990	2.20	4.39	3	3	1094	120.7	6.11
P17568	NADH dehydrogenase [ubiquinone] 1 beta subcomplex subunit 7 OS=Homo sapiens GN=NDUFB7 PE=1 SV=4 - [NDUB7_HUMAN]	62.77	1	7	7	26	0.692	1.022	0.723	0.990	57.52	62.77	10	26	137	16.4	8.92
P06730	Eukaryotic translation initiation factor 4E OS=Homo sapiens GN=EIF4E PE=1 SV=2 - [IF4E_HUMAN]	23.50	1	5	6	20	1.106	0.987	1.000	0.990	46.59	23.50	8	20	217	25.1	6.15
Q96AH8	Ras-related protein Rab-7b OS=Homo sapiens GN=RAB7B PE=2 SV=1 - [RAB7B_HUMAN]	13.57	1	2	2	5	1.241	0.583	0.789	0.990	11.48	13.57	3	5	199	22.5	6.77
Q9H6U8	Alpha-1,2-mannosyltransferase ALG9 OS=Homo sapiens GN=ALG9 PE=1 SV=2 - [ALG9_HUMAN]	9.17	1	4	4	13	0.906	1.057	0.728	0.990	37.22	9.17	7	13	611	69.8	8.68
Q96JY0	Protein maelstrom homolog OS=Homo sapiens GN=MAEL PE=1 SV=1 - [MAEL_HUMAN]	3.23	1	1	1	4	0.950	0.937	1.007	0.990	5.97	3.23	1	4	434	49.2	8.69

P20591	Interferon-induced GTP-binding protein Mx1 OS=Homo sapiens GN=MX1 PE=1 SV=4 - [MX1_HUMAN]	42.90	1	22	25	103	11.859	0.941	2.417	0.990	243.66	42.90	41	103	662	75.5	5.83
Q96AA3	Protein RFT1 homolog OS=Homo sapiens GN=RFT1 PE=1 SV=1 - [RFT1_HUMAN]	3.14	1	2	2	4	0.801	0.696	0.681	0.990	2.15	3.14	4	4	541	60.3	8.85
Q9BXJ9	N-alpha-acetyltransferase 15, NatA auxiliary subunit OS=Homo sapiens GN=NAA15 PE=1 SV=1 - [NAA15_HUMAN]	30.60	1	25	30	102	0.885	0.842	0.902	0.990	238.41	30.60	49	102	866	101.2	7.42
Q8N4P6	Leucine-rich repeat-containing protein 71 OS=Homo sapiens GN=LRRC71 PE=2 SV=1 - [LRC71_HUMAN]	6.26	1	2	3	3	1.087	0.820	1.389	0.991	4.87	6.26	3	3	559	61.8	9.22
P46952	3-hydroxyanthranilate 3,4-dioxygenase OS=Homo sapiens GN=HAAO PE=1 SV=2 - [3HAO_HUMAN]	15.03	1	4	5	10	1.498	0.839	0.719	0.991	23.30	15.03	8	10	286	32.5	5.88
Q9Y4Z0	U6 snRNA-associated Sm-like protein LSm4 OS=Homo sapiens GN=LSM4 PE=1 SV=1 - [LSM4_HUMAN]	25.18	1	5	5	13	0.894	1.097	0.877	0.991	32.60	25.18	7	13	139	15.3	9.99
Q6ICG6	Uncharacterized protein KIAA0930 OS=Homo sapiens GN=KIAA0930 PE=1 SV=1 - [K0930_HUMAN]	2.97	1	1	1	4	0.999	1.166	1.380	0.991	11.21	2.97	2	4	404	45.8	7.94
Q96C19	EF-hand domain-containing protein D2 OS=Homo sapiens GN=EFHD2 PE=1 SV=1 - [EFHD2_HUMAN]	54.17	1	16	16	62	0.962	1.426	1.233	0.991	153.06	54.17	28	62	240	26.7	5.20

Q6PCD5	E3 ubiquitin-protein ligase RFWD3 OS=Homo sapiens GN=RFWD3 PE=1 SV=3 - [RFWD3_HUMAN]	0.90	1	1	1	1	1.139	0.818	1.078	0.991	3.27	0.90	1	1	774	85.0	6.46
Q4KMQ2	Anoctamin-6 OS=Homo sapiens GN=ANO6 PE=1 SV=2 - [ANO6_HUMAN]	10.88	1	9	9	31	0.982	0.921	1.100	0.991	69.90	10.88	15	31	910	106.1	7.77
Q9UGV2	Protein NDRG3 OS=Homo sapiens GN=NDRG3 PE=1 SV=2 - [NDRG3_HUMAN]	15.20	1	4	4	15	1.065	1.076	1.255	0.991	44.15	15.20	7	15	375	41.4	5.31
P60983	Glia maturation factor beta OS=Homo sapiens GN=GMFB PE=1 SV=2 - [GMFB_HUMAN]	42.96	1	5	6	32	1.016	0.975	0.997	0.991	64.10	42.96	12	32	142	16.7	5.29
Q9H892	Tetratricopeptide repeat protein 12 OS=Homo sapiens GN=TTC12 PE=2 SV=2 - [TTC12_HUMAN	0.99	1	1	1	3	0.866	0.954	0.980	0.991	4.01	0.99	2	3	705	78.7	5.66
Q96ER9	Coiled-coil domain-containing protein 51 OS=Homo sapiens GN=CCDC51 PE=1 SV=2 - [CCD51_HUMAN]	18.98	1	7	7	19	0.859	0.911	1.271	0.991	50.74	18.98	10	19	411	45.8	8.19
O43716	Glutamyl-tRNA(Gln) amidotransferase subunit C, mitochondrial OS=Homo sapiens GN=GATC PE=2 SV=1 - [GATC_HUMAN]	8.09	1	1	1	2	1.647	0.885	0.602	0.992	5.54	8.09	2	2	136	15.1	5.05
O00193	Small acidic protein OS=Homo sapiens GN=SMAP PE=1 SV=1 - [SMAP_HUMAN]	19.67	1	5	5	24	1.043	1.453	0.957	0.992	83.32	19.67	7	24	183	20.3	4.72
A8MYZ6	Forkhead box protein O6 OS=Homo sapiens GN=FOXO6 PE=1 SV=1 - [FOXO6_HUMAN]	2.44	4	1	1	1	0.895	1.640	1.505	0.992	2.06	2.44	1	1	492	50.6	5.45

Q8N9M1	Uncharacterized protein C19orf47 OS=Homo sapiens GN=C19orf47 PE=1 SV=1 - [CS047_HUMAN]	3.32	1	1	1	2	0.859	0.831	1.260	0.992	2.17	3.32	2	2	422	44.7	10.11
Q99595	Mitochondrial import inner membrane translocase subunit Tim17-A OS=Homo sapiens GN=TIMM17A PE=1 SV=1 - [TI17A_HUMAN]	4.09	1	1	1	2	1.081	0.772	0.756	0.992	2.04	4.09	2	2	171	18.0	7.87
Q8ND56	Protein LSM14 homolog A OS=Homo sapiens GN=LSM14A PE=1 SV=3 - [LS14A_HUMAN]	13.82	1	5	7	21	1.266	0.917	0.772	0.992	33.16	13.82	8	21	463	50.5	9.52
Q7L311	Armadillo repeat-containing X-linked protein 2 OS=Homo sapiens GN=ARMCX2 PE=2 SV=1 - [ARMX2_HUMA	5.38	1	4	4	16	1.218	0.919	1.171	0.992	38.18	5.38	8	16	632	65.6	8.53
P15104	Glutamine synthetase OS=Homo sapiens GN=GLUL PE=1 SV=4 - [GLNA_HUMAN]	13.94	1	5	5	16	1.614	1.081	2.387	0.992	28.46	13.94	9	16	373	42.0	6.89
O96033	Molybdopterin synthase sulfur carrier subunit OS=Homo sapiens GN=MOCS2 PE=1 SV=1 - [MOC2A_HUMAN]	21.59	1	2	2	7	1.156	0.788	1.005	0.992	13.56	21.59	3	7	88	9.7	4.72
Q8TE77	Protein phosphatase Slingshot homolog 3 OS=Homo sapiens GN=SSH3 PE=1 SV=2 - [SSH3_HUMAN]	21.85	1	10	11	35	1.320	1.037	1.234	0.992	83.59	21.85	17	35	659	73.0	5.30
Q6P1X6	UPF0598 protein C8orf82 OS=Homo sapiens GN=C8orf82 PE=1 SV=2 - [CH082_HUMAN]	19.91	1	3	4	12	1.351	1.079	0.878	0.992	23.05	19.91	6	12	216	23.9	9.14

O43447	Peptidyl-prolyl cis-trans isomerase H OS=Homo sapiens GN=PPIH PE=1 SV=1 - [PPIH_HUMAN]	37.29	1	6	7	72	1.034	1.170	0.852	0.992	158.41	37.29	10	72	177	19.2	8.07
P46937	Transcriptional coactivator YAP1 OS=Homo sapiens GN=YAP1 PE=1 SV=2 - [YAP1_HUMAN]	29.17	1	11	11	62	0.925	1.298	1.262	0.992	162.51	29.17	18	62	504	54.4	5.17
P98174	FYVE, RhoGEF and PH domain-containing protein 1 OS=Homo sapiens GN=FGD1 PE=1 SV=2 - [FGD1_HUMAN]	0.94	1	1	1	1	1.260	0.983	0.565	0.992	1.80	0.94	1	1	961	106.5	6.58
Q68DQ2	Very large A-kinase anchor protein OS=Homo sapiens GN=CRYBG3 PE=1 SV=3 - [CRBG3_HUMAN]	3.80	2	9	10	19	1.048	0.845	0.917	0.992	30.25	3.80	11	19	2970	330.4	5.20
O00258	Tail-anchored protein insertion receptor WRB OS=Homo sapiens GN=WRB PE=1 SV=2 - [WRB_HUMAN]	4.02	1	1	1	2	1.097	1.129	1.251	0.992	3.90	4.02	2	2	174	19.8	9.67
Q9H2B2	Synaptotagmin-4 OS=Homo sapiens GN=SYT4 PE=1 SV=1 - [SYT4_HUMAN]	2.12	1	1	1	1	0.875	1.094	1.087	0.992	2.88	2.12	1	1	425	47.9	8.53
P61966	AP-1 complex subunit sigma-1A OS=Homo sapiens GN=AP1S1 PE=1 SV=1 - [AP1S1_HUMAN]	15.82	1	2	2	74	1.268	0.695	0.788	0.993	258.36	15.82	4	74	158	18.7	5.73
Q13438	Protein OS-9 OS=Homo sapiens GN=OS9 PE=1 SV=1 - [OS9_HUMAN]	10.94	1	7	7	24	0.983	0.814	0.789	0.993	51.60	10.94	10	24	667	75.5	4.87
Q3ZCQ8	Mitochondrial import inner membrane translocase subunit TIM50 OS=Homo sapiens GN=TIMM50 PE=1 SV=2 - [TIM50_HUMAN]	24.65	1	8	8	22	1.104	0.974	1.007	0.993	44.49	24.65	12	22	353	39.6	8.37

Q9P0K1	Disintegrin and metalloproteinase domain-containing protein 22 OS=Homo sapiens GN=ADAM22 PE=1 SV=1 - [ADA22_HUMAN]	1.21	1	1	1	1	0.746	0.987	0.949	0.993	2.05	1.21	1	1	906	100.4	7.20
Q13427	Peptidyl-prolyl cis-trans isomerase G OS=Homo sapiens GN=PPIG PE=1 SV=2 - [PPIG_HUMAN]	11.27	1	7	7	16	0.982	0.953	0.689	0.993	52.93	11.27	10	16	754	88.6	10.29
Q6PIW4	Fidgetin-like protein 1 OS=Homo sapiens GN=FIGNL1 PE=1 SV=2 - [FIGL1_HUMAN]	2.08	1	2	2	4	1.027	0.968	1.260	0.993	11.17	2.08	2	4	674	74.0	7.85
Q9GZZ1	N-alpha-acetyltransferase 50 OS=Homo sapiens GN=NAA50 PE=1 SV=1 - [NAA50_HUMAN	38.46	1	6	6	26	0.941	0.759	0.744	0.993	52.34	38.46	10	26	169	19.4	8.81
P51571	Translocon-associated protein subunit delta OS=Homo sapiens GN=SSR4 PE=1 SV=1 - [SSRD_HUMAN]	34.10	1	5	5	70	0.805	0.763	0.765	0.993	165.79	34.10	9	70	173	19.0	6.15
O60220	Mitochondrial import inner membrane translocase subunit Tim8 A OS=Homo sapiens GN=TIMM8A PE=1 SV=1 - [TIM8A_HUMAN]	19.59	1	2	2	4	1.068	1.092	1.068	0.993	7.40	19.59	2	4	97	11.0	5.16
Q96E09	Protein FAM122A OS=Homo sapiens GN=FAM122A PE=1 SV=1 - [F122A_HUMAN]	3.48	1	1	1	1	0.977	1.439	1.046	0.993	2.73	3.48	1	1	287	30.5	6.79
Q8N5N7	39S ribosomal protein L50, mitochondrial OS=Homo sapiens GN=MRPL50 PE=1 SV=2 - [RM50_HUMAN]	31.65	1	5	5	12	0.852	1.199	1.167	0.993	28.52	31.65	8	12	158	18.3	7.88

O95810	Serum deprivation-response protein OS=Homo sapiens GN=SDPR PE=1 SV=3 - [SDPR_HUMAN]	31.76	1	11	11	114	3.415	2.279	1.743	0.993	294.65	31.76	20	114	425	47.1	5.21
Q5JVF3	PCI domain-containing protein 2 OS=Homo sapiens GN=PCID2 PE=1 SV=2 - [PCID2_HUMAN]	5.76	1	2	2	9	0.918	1.193	1.143	0.994	14.65	5.76	4	9	399	46.0	8.53
Q567U6	Coiled-coil domain-containing protein 93 OS=Homo sapiens GN=CCDC93 PE=1 SV=2 - [CCD93_HUMAN]	20.44	1	14	15	34	1.066	1.021	1.078	0.994	77.07	20.44	21	34	631	73.2	8.15
O00165	HCLS1-associated protein X-1 OS=Homo sapiens GN=HAX1 PE=1 SV=2 - [HAX1_HUMAN]	23.30	1	4	4	9	0.936	1.081	0.948	0.994	18.03	23.30	6	9	279	31.6	4.92
O75116	Rho-associated protein kinase 2 OS=Homo sapiens GN=ROCK2 PE=1 SV=4 - [ROCK2_HUMAN]	35.73	1	45	51	169	1.005	1.049	1.176	0.994	397.00	35.73	77	169	1388	160.8	6.02
Q6N021	Methylcytosine dioxygenase TET2 OS=Homo sapiens GN=TET2 PE=1 SV=3 - [TET2_HUMAN]	0.65	1	1	1	1	0.964	0.731	0.962	0.994	0.00	0.65	1	1	2002	223.7	7.99
Q15742	NGFI-A-binding protein 2 OS=Homo sapiens GN=NAB2 PE=1 SV=1 - [NAB2_HUMAN]	2.48	1	1	1	3	0.792	0.980	1.122	0.994	6.12	2.48	2	3	525	56.6	6.96
O15264	Mitogen-activated protein kinase 13 OS=Homo sapiens GN=MAPK13 PE=1 SV=1 - [MK13_HUMAN]	10.14	3	3	4	8	0.489	0.612	0.991	0.994	15.72	10.14	6	8	365	42.1	8.38
Q9BTX1	Nucleoporin NDC1 OS=Homo sapiens GN=NDC1 PE=1 SV=2 - [NDC1_HUMAN]	5.49	1	3	3	9	1.335	1.247	0.913	0.994	13.47	5.49	6	9	674	76.3	9.09

Q9NTJ5	Phosphatidylinositide phosphatase SAC1 OS=Homo sapiens GN=SACM1L PE=1 SV=2 - [SAC1_HUMAN]	16.35	1	10	10	48	1.012	0.871	0.751	0.994	107.11	16.35	18	48	587	66.9	7.12
O15226	NF-kappa-B-repressing factor OS=Homo sapiens GN=NKRF PE=1 SV=2 - [NKRF_HUMAN]	6.23	1	5	5	20	1.061	1.087	0.997	0.995	36.36	6.23	8	20	690	77.6	8.79
O60568	Procollagen-lysine,2-oxoglutarate 5-dioxygenase 3 OS=Homo sapiens GN=PLOD3 PE=1 SV=1 - [PLOD3_HUMAN]	33.06	1	18	20	132	0.752	0.870	1.048	0.995	333.86	33.06	37	132	738	84.7	6.05
P49848	Transcription initiation factor TFIID subunit 6 OS=Homo sapiens GN=TAF6 PE=1 SV=1 - [TAF6_HUMAN]	9.16	1	4	5	8	0.883	1.431	1.356	0.995	15.39	9.16	6	8	677	72.6	8.60
Q9Y3D6	Mitochondrial fission 1 protein OS=Homo sapiens GN=FIS1 PE=1 SV=2 - [FIS1_HUMAN]	25.66	1	4	4	25	0.976	1.215	1.062	0.995	31.38	25.66	6	25	152	16.9	8.79
P46108	Adapter molecule crk OS=Homo sapiens GN=CRK PE=1 SV=2 - [CRK_HUMAN]	49.67	1	13	13	57	1.129	1.034	1.175	0.995	148.59	49.67	24	57	304	33.8	5.55
P49593	Protein phosphatase 1F OS=Homo sapiens GN=PPM1F PE=1 SV=3 - [PPM1F_HUMAN]	27.53	1	9	9	24	1.162	1.071	1.151	0.995	59.03	27.53	15	24	454	49.8	5.10
P09326	CD48 antigen OS=Homo sapiens GN=CD48 PE=1 SV=2 - [CD48_HUMAN]	2.88	1	1	1	1	2.975	0.635	1.107	0.995	0.00	2.88	1	1	243	27.7	8.07
Q7L5L3	Glycerophosphodiester phosphodiesterase domain-containing protein 3 OS=Homo sapiens GN=GDPD3 PE=2 SV=3 - [GDPD3_HUMAN]	2.20	1	1	1	33	0.771	0.804	0.754	0.995	88.94	2.20	2	33	318	36.6	7.97

O95219	Sorting nexin-4 OS=Homo sapiens GN=SNX4 PE=1 SV=1 - [SNX4_HUMAN]	23.33	1	8	8	17	1.187	0.939	1.204	0.995	33.06	23.33	11	17	450	51.9	5.99
P11908	Ribose-phosphate pyrophosphokinase 2 OS=Homo sapiens GN=PRPS2 PE=1 SV=2 - [PRPS2_HUMAN]	21.70	1	4	10	43	1.025	0.839	0.773	0.995	93.73	21.70	16	43	318	34.7	6.61
Q8NFF5	FAD synthase OS=Homo sapiens GN=FLAD1 PE=1 SV=1 - [FAD1_HUMAN]	8.35	1	4	4	7	1.140	0.847	0.935	0.995	17.30	8.35	6	7	587	65.2	6.93
O00584	Ribonuclease T2 OS=Homo sapiens GN=RNASET2 PE=1 SV=2 - [RNT2_HUMAN]	29.69	1	6	6	13	0.894	1.342	1.011	0.995	33.89	29.69	9	13	256	29.5	7.08
Q9UPN7	Serine/threonine-protein phosphatase 6 regulatory subunit 1 OS=Homo sapiens GN=PPP6R1 PE=1 SV=5 - [PP6R1_HUMAN]	9.76	1	4	4	11	0.647	0.871	0.948	0.995	26.28	9.76	5	11	881	96.7	4.55
Q9Y496	Kinesin-like protein KIF3A OS=Homo sapiens GN=KIF3A PE=1 SV=4 - [KIF3A_HUMAN]	8.44	3	5	6	23	1.215	1.066	1.264	0.996	53.16	8.44	9	23	699	80.0	6.54
Q8IYX0	Zinc finger protein 679 OS=Homo sapiens GN=ZNF679 PE=2 SV=2 - [ZN679_HUMAN]	2.92	1	1	1	1	0.651	1.050	0.672	0.996	0.00	2.92	1	1	411	47.1	8.91
Q9H2U1	ATP-dependent RNA helicase DHX36 OS=Homo sapiens GN=DHX36 PE=1 SV=2 - [DHX36_HUMAN	12.90	1	9	11	20	0.915	0.820	0.857	0.996	49.01	12.90	16	20	1008	114.7	7.68
O15069	NAC-alpha domain-containing protein 1 OS=Homo sapiens GN=NACAD PE=1 SV=3 - [NACAD_HUMAN]	2.37	1	3	3	8	1.404	1.758	1.699	0.996	13.11	2.37	5	8	1562	161.0	4.23

Q9P032	NADH dehydrogenase [ubiquinone] 1 alpha subcomplex assembly factor 4 OS=Homo sapiens GN=NDUFAF4 PE=1 SV=1 - [NDUF4_HUMAN]	35.43	1	7	7	21	0.639	0.688	0.840	0.996	51.41	35.43	9	21	175	20.3	8.82
P14550	Alcohol dehydrogenase [NADP(+)] OS=Homo sapiens GN=AKR1A1 PE=1 SV=3 - [AK1A1_HUMAN]	40.31	1	12	13	68	1.165	1.087	1.081	0.996	158.25	40.31	21	68	325	36.5	6.79
O95292	Vesicle-associated membrane protein-associated protein B/C OS=Homo sapiens GN=VAPB PE=1 SV=3 - [VAPB_HUMAN]	49.38	1	12	13	53	1.233	1.059	0.927	0.996	99.71	49.38	20	53	243	27.2	7.30
P48200	Iron-responsive element-binding protein 2 OS=Homo sapiens GN=IREB2 PE=1 SV=3 - [IREB2_HUMAN]	4.26	1	4	4	6	1.823	1.208	1.541	0.996	9.95	4.26	6	6	963	105.0	7.05
Q2NL82	Pre-rRNA-processing protein TSR1 homolog OS=Homo sapiens GN=TSR1 PE=1 SV=1 - [TSR1_HUMAN]	8.58	1	5	5	12	1.078	1.083	1.583	0.996	33.18	8.58	8	12	804	91.8	7.42
P30101	Protein disulfide-isomerase A3 OS=Homo sapiens GN=PDIA3 PE=1 SV=4 - [PDIA3_HUMAN	72.28	2	44	44	822	0.849	0.990	0.990	0.996	2218.08	72.28	74	822	505	56.7	6.35
Q14693	Phosphatidate phosphatase LPIN1 OS=Homo sapiens GN=LPIN1 PE=1 SV=2 - [LPIN1_HUMAN]	5.28	1	2	4	9	1.005	1.030	1.027	0.997	27.21	5.28	5	9	890	98.6	6.58
Q9H9E3	Conserved oligomeric Golgi complex subunit 4 OS=Homo sapiens GN=COG4 PE=1 SV=3 - [COG4_HUMAN]	10.70	1	7	9	25	0.795	0.927	1.234	0.997	47.32	10.70	14	25	785	89.0	5.19

P27105	Erythrocyte band 7 integral membrane protein OS=Homo sapiens GN=STOM PE=1 SV=3 - [STOM_HUMAN	54.86	2	16	16	152	2.200	0.622	0.738	0.997	352.26	54.86	28	152	288	31.7	7.88
Q14161	ARF GTPase-activating protein GIT2 OS=Homo sapiens GN=GIT2 PE=1 SV=2 - [GIT2_HUMAN]	21.61	1	14	16	51	1.126	1.024	1.091	0.997	96.93	21.61	23	51	759	84.5	7.23
P25398	40S ribosomal protein S12 OS=Homo sapiens GN=RPS12 PE=1 SV=3 - [RS12_HUMAN]	45.45	1	5	5	145	0.814	0.836	0.898	0.997	396.16	45.45	9	145	132	14.5	7.21
Q9UKS6	Protein kinase C and casein kinase substrate in neurons protein 3 OS=Homo sapiens GN=PACSIN3 PE=1 SV=2 - [PACN3_HUMAN	20.99	1	9	9	31	1.413	2.311	1.696	0.997	65.01	20.99	16	31	424	48.5	6.18
P04156	Major prion protein OS=Homo sapiens GN=PRNP PE=1 SV=1 - [PRIO_HUMAN]	8.70	1	2	2	5	1.307	0.358	0.734	0.997	17.62	8.70	2	5	253	27.6	9.00
O60888	Protein CutA OS=Homo sapiens GN=CUTA PE=1 SV=2 - [CUTA_HUMAN]	15.64	1	2	2	42	1.249	0.886	0.855	0.997	115.38	15.64	4	42	179	19.1	5.50
O94804	Serine/threonine-protein kinase 10 OS=Homo sapiens GN=STK10 PE=1 SV=1 - [STK10_HUMAN	19.83	1	17	18	50	1.112	0.924	1.242	0.997	112.33	19.83	24	50	968	112.1	6.95
Q9UIL4	Kinesin-like protein KIF25 OS=Homo sapiens GN=KIF25 PE=2 SV=2 - [KIF25_HUMAN]	4.17	1	1	1	1	1.665	0.956	1.174	0.997	0.00	4.17	1	1	384	40.7	8.31
O60749	Sorting nexin-2 OS=Homo sapiens GN=SNX2 PE=1 SV=2 - [SNX2_HUMAN]	49.90	1	22	25	76	1.115	1.018	1.038	0.997	177.90	49.90	40	76	519	58.4	5.12

Q6DN90	IQ motif and SEC7 domain-containing protein 1 OS=Homo sapiens GN=IQSEC1 PE=1 SV=1 - [IQEC1_HUMAN]	6.75	1	5	7	13	1.012	1.066	1.106	0.997	24.49	6.75	9	13	963	108.2	6.93
Q6P1N0	Coiled-coil and C2 domain-containing protein 1A OS=Homo sapiens GN=CC2D1A PE=1 SV=1 - [C2D1A_HUMAN]	25.45	1	22	22	58	0.927	1.146	1.258	0.997	115.12	25.45	34	58	951	104.0	8.09
Q9NQR4	Omega-amidase NIT2 OS=Homo sapiens GN=NIT2 PE=1 SV=1 - [NIT2_HUMAN]	39.86	1	11	11	57	1.088	0.949	1.115	0.997	132.71	39.86	18	57	276	30.6	7.21
P54840	Glycogen [starch] synthase, liver OS=Homo sapiens GN=GYS2 PE=1 SV=2 - [GYS2_HUMAN]	5.97	1	2	4	8	0.985	1.143	1.105	0.997	21.79	5.97	5	8	703	80.9	6.83
Q9BVP2	Guanine nucleotide-binding protein-like 3 OS=Homo sapiens GN=GNL3 PE=1 SV=2 - [GNL3_HUMAN]	23.50	1	10	10	32	0.687	0.727	0.650	0.997	77.05	23.50	14	32	549	62.0	9.16
Q13586	Stromal interaction molecule 1 OS=Homo sapiens GN=STIM1 PE=1 SV=3 - [STIM1_HUMAN]	28.03	1	18	18	57	1.080	1.004	0.929	0.997	146.04	28.03	26	57	685	77.4	6.67
Q96AG3	Solute carrier family 25 member 46 OS=Homo sapiens GN=SLC25A46 PE=1 SV=1 - [S2546_HUMAN]	9.57	1	4	4	7	0.951	0.999	1.244	0.997	9.72	9.57	7	7	418	46.1	7.43
Q9UL25	Ras-related protein Rab-21 OS=Homo sapiens GN=RAB21 PE=1 SV=3 - [RAB21_HUMAN]	34.22	2	7	7	50	1.106	0.950	0.900	0.997	125.49	34.22	12	50	225	24.3	7.94

Q96EL2	28S ribosomal protein S24, mitochondrial OS=Homo sapiens GN=MRPS24 PE=1 SV=1 - [RT24_HUMAN]	21.56	1	3	3	7	0.739	1.029	0.721	0.998	10.78	21.56	5	7	167	19.0	9.38
P15927	Replication protein A 32 kDa subunit OS=Homo sapiens GN=RPA2 PE=1 SV=1 - [RFA2_HUMAN]	15.56	1	4	4	17	1.182	1.206	1.079	0.998	42.34	15.56	7	17	270	29.2	6.15
Q7Z5G4	Golgin subfamily A member 7 OS=Homo sapiens GN=GOLGA7 PE=1 SV=2 - [GOGA7_HUMAN]	32.12	1	4	4	15	1.325	0.988	0.893	0.998	36.54	32.12	7	15	137	15.8	7.05
Q9BTY7	Protein HGH1 homolog OS=Homo sapiens GN=HGH1 PE=1 SV=1 - [HGH1_HUMAN]	14.10	1	6	6	19	0.763	1.318	1.321	0.998	32.67	14.10	9	19	390	42.1	4.81
Q8WXI9	Transcriptional repressor p66-beta OS=Homo sapiens GN=GATAD2B PE=1 SV=1 - [P66B_HUMAN]	10.12	1	4	5	30	1.193	1.386	1.059	0.998	59.51	10.12	9	30	593	65.2	9.70
Q9Y6F6	Protein MRVI1 OS=Homo sapiens GN=MRVI1 PE=1 SV=2 - [MRVI1_HUMAN]	30.62	1	24	25	98	0.887	1.482	1.959	0.998	231.58	30.62	40	98	885	96.1	5.58
P04062	Glucosylceramidase OS=Homo sapiens GN=GBA PE=1 SV=3 - [GLCM_HUMAN]	19.22	1	8	9	40	0.958	1.172	1.003	0.998	90.81	19.22	15	40	536	59.7	7.61
Q8IV48	3'-5' exoribonuclease 1 OS=Homo sapiens GN=ERI1 PE=1 SV=3 - [ERI1_HUMAN]	12.32	1	4	4	9	0.654	1.116	0.917	0.998	20.92	12.32	7	9	349	40.0	6.70
Q96FW1	Ubiquitin thioesterase OTUB1 OS=Homo sapiens GN=OTUB1 PE=1 SV=2 - [OTUB1_HUMAN]	36.90	1	11	11	39	1.127	1.105	0.973	0.998	105.07	36.90	16	39	271	31.3	4.94

Q14108	Lysosome membrane protein 2 OS=Homo sapiens GN=SCARB2 PE=1 SV=2 - [SCRB2_HUMAN]	27.20	1	11	11	101	0.914	0.705	0.556	0.998	240.32	27.20	19	101	478	54.3	5.14
P38606	V-type proton ATPase catalytic subunit A OS=Homo sapiens GN=ATP6V1A PE=1 SV=2 - [VATA_HUMAN]	39.22	1	24	24	99	1.024	0.898	1.046	0.998	245.50	39.22	36	99	617	68.3	5.52
Q15126	Phosphomevalonate kinase OS=Homo sapiens GN=PMVK PE=1 SV=3 - [PMVK_HUMAN]	51.04	1	9	9	21	1.192	1.393	1.510	0.999	30.45	51.04	14	21	192	22.0	5.73
Q12802	A-kinase anchor protein 13 OS=Homo sapiens GN=AKAP13 PE=1 SV=2 - [AKP13_HUMAN]	3.48	1	6	9	17	0.782	1.213	0.946	0.999	31.08	3.48	11	17	2813	307.4	5.24
Q5XKK7	Protein FAM219B OS=Homo sapiens GN=FAM219B PE=1 SV=1 - [F219B_HUMAN]	8.59	1	1	1	8	1.049	0.748	1.147	0.999	20.45	8.59	2	8	198	21.1	8.25
Q9UJU3	Zinc finger protein 112 OS=Homo sapiens GN=ZNF112 PE=2 SV=2 - [ZN112_HUMAN]	9.97	11	2	4	6	0.636	1.849	0.991	0.999	2.55	9.97	6	6	913	105.8	8.27
Q6P9G9	Zinc finger protein 449 OS=Homo sapiens GN=ZNF449 PE=2 SV=3 - [ZN449_HUMAN]	4.63	94	1	2	3	0.887	0.873	1.358	0.999	2.24	4.63	2	3	518	59.9	7.31
Q9UKG1	DCC-interacting protein 13-alpha OS=Homo sapiens GN=APPL1 PE=1 SV=1 - [DP13A_HUMAN]	23.98	1	17	18	57	0.836	1.089	1.029	0.999	134.22	23.98	28	57	709	79.6	5.41
P46781	40S ribosomal protein S9 OS=Homo sapiens GN=RPS9 PE=1 SV=3 - [RS9_HUMAN]	39.18	2	12	13	148	0.780	0.887	0.720	0.999	332.94	39.18	23	148	194	22.6	10.65

Q9Y5U2	Protein TSSC4 OS=Homo sapiens GN=TSSC4 PE=1 SV=3 - [TSSC4_HUMAN]	10.03	1	3	3	8	0.940	0.956	0.841	0.999	22.46	10.03	4	8	329	34.3	5.19
Q9NWM3	CUE domain-containing protein 1 OS=Homo sapiens GN=CUEDC1 PE=1 SV=1 - [CUED1_HUMAN]	5.18	1	2	2	6	0.791	1.021	0.958	0.999	15.03	5.18	3	6	386	42.2	5.68
O94826	Mitochondrial import receptor subunit TOM70 OS=Homo sapiens GN=TOMM70A PE=1 SV=1 - [TOM70_HUMA	48.36	1	27	27	135	0.891	0.962	0.827	0.999	321.20	48.36	39	135	608	67.4	7.12
O00303	Eukaryotic translation initiation factor 3 subunit F OS=Homo sapiens GN=EIF3F PE=1 SV=1 - [EIF3F_HUMAN]	23.81	1	8	8	32	0.963	1.000	1.005	0.999	76.58	23.81	13	32	357	37.5	5.45
Q86SZ2	Trafficking protein particle complex subunit 6B OS=Homo sapiens GN=TRAPPC6B PE=1 SV=1 - [TPC6B_HUMAN]	39.24	1	8	8	18	1.154	1.023	0.909	0.999	43.29	39.24	11	18	158	18.0	8.68
O43772	Mitochondrial carnitine/acylcarnitine carrier protein OS=Homo sapiens GN=SLC25A20 PE=1 SV=1 - [MCAT_HUMAN]	26.91	1	9	9	34	1.143	0.763	0.789	0.999	64.54	26.91	17	34	301	32.9	9.41
Q9BWT3	Poly(A) polymerase gamma OS=Homo sapiens GN=PAPOLG PE=1 SV=2 - [PAPOG_HUMAN]	2.99	2	2	3	4	1.197	1.129	1.466	0.999	8.04	2.99	3	4	736	82.8	9.14
Q7KYR7	Butyrophilin subfamily 2 member A1 OS=Homo sapiens GN=BTN2A1 PE=1 SV=3 - [BT2A1_HUMAN]	10.25	1	5	6	10	1.279	0.756	0.976	0.999	23.98	10.25	8	10	527	59.6	6.48

Q5JPH6	Probable glutamate--tRNA ligase, mitochondrial OS=Homo sapiens GN=EARS2 PE=1 SV=2 - [SYEM_HUMAN]	10.71	1	7	7	16	0.942	1.054	0.820	0.999	26.12	10.71	11	16	523	58.7	8.76
P33240	Cleavage stimulation factor subunit 2 OS=Homo sapiens GN=CSTF2 PE=1 SV=1 - [CSTF2_HUMAN]	22.88	1	4	9	25	1.078	1.040	1.267	1.000	46.80	22.88	13	25	577	60.9	6.83
Q92833	Protein Jumonji OS=Homo sapiens GN=JARID2 PE=1 SV=2 - [JARD2_HUMAN]	0.80	1	1	1	1	2.068	0.820	1.711	1.000	1.77	0.80	1	1	1246	138.6	9.38
Q92542	Nicastrin OS=Homo sapiens GN=NCSTN PE=1 SV=2 - [NICA_HUMAN]	12.27	1	8	8	20	1.360	0.854	0.857	1.000	44.79	12.27	12	20	709	78.4	5.99
P31040	Succinate dehydrogenase [ubiquinone] flavoprotein subunit, mitochondrial OS=Homo sapiens GN=SDHA PE=1 SV=2 - [SDHA_HUMAN]	43.37	1	22	22	80	0.757	1.116	0.987	1.000	220.83	43.37	33	80	664	72.6	7.39
P20749	B-cell lymphoma 3 protein OS=Homo sapiens GN=BCL3 PE=1 SV=2 - [BCL3_HUMAN]	5.51	1	2	2	2	1.028	1.068	0.937	1.000	4.31	5.51	2	2	454	47.6	8.41
P14618	Pyruvate kinase PKM OS=Homo sapiens GN=PKM PE=1 SV=4 - [KPYM_HUMAN]	75.52	3	45	47	1875	1.298	1.175	1.060	1.000	5351.87	75.52	78	1875	531	57.9	7.84
Q96AT9	Ribulose-phosphate 3-epimerase OS=Homo sapiens GN=RPE PE=1 SV=1 - [RPE_HUMAN]	25.00	2	4	4	12	0.913	1.038	0.974	1.000	33.11	25.00	6	12	228	24.9	5.58
Q6UWP7	Lysocardiolipin acyltransferase 1 OS=Homo sapiens GN=LCLAT1 PE=1 SV=1 - [LCLT1_HUMAN]	17.87	1	7	7	26	0.968	0.877	0.837	1.000	65.16	17.87	12	26	414	48.9	8.62

Q6N063	2-oxoglutarate and iron-dependent oxygenase domain-containing protein 2 OS=Homo sapiens GN=OGFOD2 PE=2 SV=2 - [OGFD2_HUMAN]	6.57	1	2	2	3	1.017	1.058	0.851	1.000	11.04	6.57	2	3	350	39.0	5.68
Q15750	TGF-beta-activated kinase 1 and MAP3K7-binding protein 1 OS=Homo sapiens GN=TAB1 PE=1 SV=1 - [TAB1_HUMAN]	20.63	1	7	7	24	0.914	1.016	1.084	1.000	64.58	20.63	11	24	504	54.6	5.52
P17812	CTP synthase 1 OS=Homo sapiens GN=CTPS1 PE=1 SV=2 - [PYRG1_HUMAN]	27.41	1	16	18	71	0.758	0.766	0.777	1.000	177.20	27.41	29	71	591	66.6	6.46
Q96QC0	Serine/threonine-protein phosphatase 1 regulatory subunit 10 OS=Homo sapiens GN=PPP1R10 PE=1 SV=1 - [PP1RA_HUMAN]	1.81	1	2	2	3	1.155	1.899	1.273	1.000	6.68	1.81	2	3	940	99.0	9.17
P40189	Interleukin-6 receptor subunit beta OS=Homo sapiens GN=IL6ST PE=1 SV=2 - [IL6RB_HUMAN]	13.29	1	10	10	27	0.775	0.710	0.881	1.000	73.05	13.29	17	27	918	103.5	5.95
P17900	Ganglioside GM2 activator OS=Homo sapiens GN=GM2A PE=1 SV=4 - [SAP3_HUMAN]	22.80	2	5	5	9	0.844	1.048	1.360	1.000	20.44	22.80	7	9	193	20.8	5.31
P46779	60S ribosomal protein L28 OS=Homo sapiens GN=RPL28 PE=1 SV=3 - [RL28_HUMAN]	51.09	1	10	10	152	0.771	0.973	0.857	1.000	299.82	51.09	19	152	137	15.7	12.02
Q9BT78	COP9 signalosome complex subunit 4 OS=Homo sapiens GN=COPS4 PE=1 SV=1 - [CSN4_HUMAN]	63.79	1	20	21	68	1.011	1.112	1.071	1.000	172.41	63.79	35	68	406	46.2	5.83

Q9H5K3	Protein O-mannose kinase OS=Homo sapiens GN=POMK PE=1 SV=1 - [SG196_HUMAN	8.29	1	2	2	6	1.389	0.925	1.011	1.000	20.72	8.29	3	6	350	40.0	6.10
P45880	Voltage-dependent anion-selective channel protein 2 OS=Homo sapiens GN=VDAC2 PE=1 SV=2 - [VDAC2_HUMAN	46.60	1	12	13	140	0.965	0.909	0.759	1.001	324.39	46.60	19	140	294	31.5	7.56
Q13443	Disintegrin and metalloproteinase domain-containing protein 9 OS=Homo sapiens GN=ADAM9 PE=1 SV=1 - [ADAM9_HUMAN]	10.74	1	7	7	18	0.910	0.934	1.030	1.001	34.62	10.74	11	18	819	90.5	7.52
C4AMC7	Putative WAS protein family homolog 3 OS=Homo sapiens GN=WASH3P PE=2 SV=2 - [WASH3_HUMAN]	24.62	5	9	9	30	0.872	0.903	1.036	1.001	63.84	24.62	12	30	463	50.0	5.49
Q8TCF1	AN1-type zinc finger protein 1 OS=Homo sapiens GN=ZFAND1 PE=1 SV=1 - [ZFAN1_HUMAN]	8.96	1	2	2	5	1.014	0.967	1.654	1.001	14.49	8.96	3	5	268	30.8	7.39
P48553	Trafficking protein particle complex subunit 10 OS=Homo sapiens GN=TRAPPC10 PE=1 SV=2 - [TPC10_HUMAN]	0.87	1	1	1	1	0.978	0.860	1.156	1.001	0.00	0.87	1	1	1259	142.1	6.04
O00401	Neural Wiskott-Aldrich syndrome protein OS=Homo sapiens GN=WASL PE=1 SV=2 - [WASL_HUMAN]	11.09	1	4	4	18	1.091	1.161	1.157	1.001	39.02	11.09	7	18	505	54.8	7.93
Q969K3	E3 ubiquitin-protein ligase RNF34 OS=Homo sapiens GN=RNF34 PE=1 SV=1 - [RNF34_HUMAN]	4.03	1	1	2	2	0.814	0.850	1.561	1.001	1.72	4.03	2	2	372	41.6	4.96

O75356	Ectonucleoside triphosphate diphosphohydrolase 5 OS=Homo sapiens GN=ENTPD5 PE=1 SV=1 - [ENTP5_HUMAN]	5.61	1	2	2	3	1.048	0.939	1.015	1.002	6.14	5.61	2	3	428	47.5	6.33
P61513	60S ribosomal protein L37a OS=Homo sapiens GN=RPL37A PE=1 SV=2 - [RL37A_HUMAN]	36.96	1	5	5	18	1.071	0.792	0.895	1.002	40.76	36.96	7	18	92	10.3	10.43
O00461	Golgi integral membrane protein 4 OS=Homo sapiens GN=GOLIM4 PE=1 SV=1 - [GOLI4_HUMAN]	20.11	1	13	13	62	0.768	0.930	0.938	1.002	168.03	20.11	25	62	696	81.8	4.77
Q6IA69	Glutamine-dependent NAD(+) synthetase OS=Homo sapiens GN=NADSYN1 PE=1 SV=3 - [NADE_HUMAN]	7.22	1	4	4	16	0.994	0.797	1.126	1.002	28.23	7.22	7	16	706	79.2	6.44
Q96I76	G patch domain-containing protein 3 OS=Homo sapiens GN=GPATCH3 PE=2 SV=1 - [GPTC3_HUMAN	3.62	1	1	2	2	1.960	1.050	2.009	1.002	4.75	3.62	2	2	525	59.3	5.01
Q9H173	Nucleotide exchange factor SIL1 OS=Homo sapiens GN=SIL1 PE=1 SV=1 - [SIL1_HUMAN]	18.44	1	8	8	23	0.584	0.792	0.722	1.002	59.46	18.44	13	23	461	52.1	5.36
Q9NZ43	Vesicle transport protein USE1 OS=Homo sapiens GN=USE1 PE=1 SV=2 - [USE1_HUMAN]	14.67	1	6	6	13	0.831	1.031	0.958	1.002	30.52	14.67	8	13	259	29.4	9.07
Q2T9F4	Integrator complex subunit 4-like protein 2 OS=Homo sapiens GN=INTS4L2 PE=2 SV=1 - [IN4L2_HUMAN]	2.73	3	1	1	2	0.934	0.942	0.807	1.002	6.87	2.73	1	2	439	48.9	6.21

Q7Z6J0	E3 ubiquitin-protein ligase SH3RF1 OS=Homo sapiens GN=SH3RF1 PE=1 SV=2 - [SH3R1_HUMAN]	8.00	1	2	5	6	2.008	2.064	2.993	1.002	10.52	8.00	6	6	888	93.1	8.54
A8K7I4	Calcium-activated chloride channel regulator 1 OS=Homo sapiens GN=CLCA1 PE=1 SV=3 - [CLCA1_HUMAN	1.75	1	1	1	1	1.071	1.085	1.102	1.002	0.00	1.75	1	1	914	100.2	6.37
P25786	Proteasome subunit alpha type-1 OS=Homo sapiens GN=PSMA1 PE=1 SV=1 - [PSA1_HUMAN]	57.79	1	13	13	121	1.006	1.022	0.956	1.002	332.07	57.79	23	121	263	29.5	6.61
O60831	PRA1 family protein 2 OS=Homo sapiens GN=PRAF2 PE=1 SV=1 - [PRAF2_HUMAN]	26.97	1	5	5	13	0.857	0.811	0.964	1.002	26.84	26.97	9	13	178	19.2	9.19
A5YKK6	CCR4-NOT transcription complex subunit 1 OS=Homo sapiens GN=CNOT1 PE=1 SV=2 - [CNOT1_HUMAN]	9.64	1	20	20	59	0.933	0.975	0.775	1.002	132.13	9.64	33	59	2376	266.8	7.11
Q96PU5	E3 ubiquitin-protein ligase NEDD4-like OS=Homo sapiens GN=NEDD4L PE=1 SV=2 - [NED4L_HUMAN]	4.51	1	3	3	23	1.157	2.131	1.235	1.002	44.76	4.51	5	23	975	111.9	5.82
O75521	Enoyl-CoA delta isomerase 2, mitochondrial OS=Homo sapiens GN=ECI2 PE=1 SV=4 - [ECI2_HUMAN]	40.61	1	12	12	34	0.721	1.052	1.117	1.002	95.88	40.61	18	34	394	43.6	9.00
O75439	Mitochondrial-processing peptidase subunit beta OS=Homo sapiens GN=PMPCB PE=1 SV=2 - [MPPB_HUMAN]	38.65	1	15	16	57	0.899	1.228	1.054	1.002	131.27	38.65	26	57	489	54.3	6.83

Q9GZU7	Carboxy-terminal domain RNA polymerase II polypeptide A small phosphatase 1 OS=Homo sapiens GN=CTDSP1 PE=1 SV=1 - [CTDS1_HUMAN]	20.69	2	4	4	7	1.064	0.886	2.054	1.002	25.37	20.69	4	7	261	29.2	6.00
P02545	Prelamin-A/C OS=Homo sapiens GN=LMNA PE=1 SV=1 - [LMNA_HUMAN]	72.89	1	66	69	1963	1.081	1.488	1.400	1.003	4666.32	72.89	125	1963	664	74.1	7.02
P30740	Leukocyte elastase inhibitor OS=Homo sapiens GN=SERPINB1 PE=1 SV=1 - [ILEU_HUMAN]	44.59	1	16	16	62	2.027	0.761	0.885	1.003	151.60	44.59	28	62	379	42.7	6.28
P67936	Tropomyosin alpha-4 chain OS=Homo sapiens GN=TPM4 PE=1 SV=3 - [TPM4_HUMAN]	83.87	2	21	42	1796	1.135	0.946	1.243	1.003	4443.01	83.87	70	1796	248	28.5	4.69
Q6ZXV5	Transmembrane and TPR repeat-containing protein 3 OS=Homo sapiens GN=TMTC3 PE=1 SV=2 - [TMTC3_HUMA	21.31	1	19	20	64	0.909	0.839	0.903	1.003	169.38	21.31	32	64	915	103.9	8.87
Q9H0N5	Pterin-4-alpha-carbinolamine dehydratase 2 OS=Homo sapiens GN=PCBD2 PE=1 SV=4 - [PHS2_HUMAN]	24.62	1	2	3	16	1.215	0.676	0.868	1.003	33.29	24.62	5	16	130	14.4	9.11
O76074	cGMP-specific 3',5'-cyclic phosphodiesterase OS=Homo sapiens GN=PDE5A PE=1 SV=2 - [PDE5A_HUMAN]	38.51	1	35	35	199	0.947	1.534	1.588	1.003	470.10	38.51	61	199	875	99.9	6.09
Q9HCS7	Pre-mRNA-splicing factor SYF1 OS=Homo sapiens GN=XAB2 PE=1 SV=2 - [SYF1_HUMAN]	5.03	1	5	5	10	1.173	0.722	0.702	1.003	20.95	5.03	6	10	855	99.9	6.23
P16333	Cytoplasmic protein NCK1 OS=Homo sapiens GN=NCK1 PE=1 SV=1 - [NCK1_HUMAN]	32.63	2	12	12	32	1.441	0.801	1.014	1.003	84.17	32.63	18	32	377	42.8	6.47

Q8N1F7	Nuclear pore complex protein Nup93 OS=Homo sapiens GN=NUP93 PE=1 SV=2 - [NUP93_HUMAN]	37.48	1	28	28	96	0.974	0.906	0.831	1.003	230.61	37.48	40	96	819	93.4	5.72
Q99445	Glycosyl-phosphatidylinositol-anchored molecule-like protein OS=Homo sapiens GN=GML PE=2 SV=1 - [GML_HUMAN]	6.33	1	1	1	1	1.025	0.533	0.932	1.003	2.29	6.33	1	1	158	17.7	6.48
Q6PGP7	Tetratricopeptide repeat protein 37 OS=Homo sapiens GN=TTC37 PE=1 SV=1 - [TTC37_HUMAN	20.72	1	28	29	68	0.828	0.908	1.057	1.003	167.77	20.72	46	68	1564	175.4	7.53
Q9UMY4	Sorting nexin-12 OS=Homo sapiens GN=SNX12 PE=1 SV=3 - [SNX12_HUMAN]	48.84	1	10	11	41	1.120	0.827	1.220	1.004	113.34	48.84	17	41	172	19.7	7.87
Q9Y3D2	Methionine-R-sulfoxide reductase B2, mitochondrial OS=Homo sapiens GN=MSRB2 PE=2 SV=2 - [MSRB2_HUMAN]	57.14	1	7	7	33	0.940	1.360	1.225	1.004	83.96	57.14	11	33	182	19.5	8.63
Q9UGM6	Tryptophan--tRNA ligase, mitochondrial OS=Homo sapiens GN=WARS2 PE=1 SV=1 - [SYWM_HUMAN]	24.44	1	6	6	13	0.946	1.648	1.091	1.004	37.19	24.44	11	13	360	40.1	9.28
Q86TG7	Retrotransposon-derived protein PEG10 OS=Homo sapiens GN=PEG10 PE=1 SV=2 - [PEG10_HUMAN]	3.25	1	1	1	1		1.738	3.751	1.004	2.43	3.25	1	1	708	80.1	6.39
P49768	Presenilin-1 OS=Homo sapiens GN=PSEN1 PE=1 SV=1 - [PSN1_HUMAN]	13.06	2	3	3	8	1.328	0.483	0.626	1.004	24.67	13.06	5	8	467	52.6	5.31
Q9UQN3	Charged multivesicular body protein 2b OS=Homo sapiens GN=CHMP2B PE=1 SV=1 - [CHM2B_HUMAN]	19.72	1	5	5	21	0.942	0.883	1.170	1.004	47.03	19.72	8	21	213	23.9	8.76

Q9Y2Q3	Glutathione S-transferase kappa 1 OS=Homo sapiens GN=GSTK1 PE=1 SV=3 - [GSTK1_HUMAN]	44.25	1	9	9	52	1.024	1.334	1.339	1.004	133.06	44.25	15	52	226	25.5	8.41
P67809	Nuclease-sensitive element-binding protein 1 OS=Homo sapiens GN=YBX1 PE=1 SV=3 - [YBOX1_HUMA	33.95	2	3	9	44	0.808	1.719	1.497	1.004	132.62	33.95	13	44	324	35.9	9.88
Q9UBS4	DnaJ homolog subfamily B member 11 OS=Homo sapiens GN=DNAJB11 PE=1 SV=1 - [DJB11_HUMAN]	38.27	1	15	15	79	0.837	0.870	0.837	1.004	204.76	38.27	26	79	358	40.5	6.18
O00571	ATP-dependent RNA helicase DDX3X OS=Homo sapiens GN=DDX3X PE=1 SV=3 - [DDX3X_HUMAN	42.75	1	7	29	164	1.067	1.108	0.892	1.004	407.84	42.75	46	164	662	73.2	7.18
Q86UV5	Ubiquitin carboxyl-terminal hydrolase 48 OS=Homo sapiens GN=USP48 PE=1 SV=1 - [UBP48_HUMAN]	1.06	1	1	1	3	1.032	1.239	1.079	1.004	9.55	1.06	2	3	1035	119.0	6.05
Q5VYK3	Proteasome-associated protein ECM29 homolog OS=Homo sapiens GN=ECM29 PE=1 SV=2 - [ECM29_HUMAN]	16.69	1	24	24	53	0.872	0.965	1.035	1.004	117.78	16.69	32	53	1845	204.2	7.12
Q5M9Q1	NKAP-like protein OS=Homo sapiens GN=NKAPL PE=2 SV=3 - [NKAPL_HUMAN]	2.49	2	1	1	2	1.021	1.199	1.262	1.004	2.25	2.49	2	2	402	46.3	9.72
Q3KRB8	Rho GTPase-activating protein 11B OS=Homo sapiens GN=ARHGAP11B PE=1 SV=1 - [RHGBB_HUMAN]	4.49	2	1	2	8	0.612	0.790	0.541	1.005	17.06	4.49	2	8	267	30.2	9.14

Q9Y3D7	Mitochondrial import inner membrane translocase subunit TIM16 OS=Homo sapiens GN=PAM16 PE=1 SV=2 - [TIM16_HUMAN]	33.60	1	4	5	15	0.964	1.060	0.919	1.005	35.20	33.60	7	15	125	13.8	9.70
Q9NVG8	TBC1 domain family member 13 OS=Homo sapiens GN=TBC1D13 PE=1 SV=3 - [TBC13_HUMAN]	32.00	1	10	10	38	0.982	0.834	1.013	1.005	104.53	32.00	16	38	400	46.5	5.24
P0DJD0	RANBP2-like and GRIP domain-containing protein 1 OS=Homo sapiens GN=RGPD1 PE=2 SV=1 - [RGPD1_HUMAN]	16.08	6	1	29	74	1.077	0.992	1.165	1.005	161.88	16.08	38	74	1748	196.5	6.14
O95571	Persulfide dioxygenase ETHE1, mitochondrial OS=Homo sapiens GN=ETHE1 PE=1 SV=2 - [ETHE1_HUMAN]	38.98	1	7	7	29	1.452	1.424	1.204	1.005	78.42	38.98	11	29	254	27.9	6.83
Q8NB66	Protein unc-13 homolog C OS=Homo sapiens GN=UNC13C PE=2 SV=3 - [UN13C_HUMAN]	1.36	1	2	3	7	0.784	0.888	1.288	1.005	10.20	1.36	3	7	2214	250.8	5.92
P20936	Ras GTPase-activating protein 1 OS=Homo sapiens GN=RASA1 PE=1 SV=1 - [RASA1_HUMAN]	12.03	1	10	10	26	0.925	1.005	1.079	1.005	71.51	12.03	16	26	1047	116.3	6.54
Q13761	Runt-related transcription factor 3 OS=Homo sapiens GN=RUNX3 PE=1 SV=2 - [RUNX3_HUMAN]	3.86	1	1	1	1	0.914	1.627	1.657	1.005	3.29	3.86	1	1	415	44.3	9.47
P61812	Transforming growth factor beta-2 OS=Homo sapiens GN=TGFB2 PE=1 SV=1 - [TGFB2_HUMAN]	5.31	2	2	3	5	2.231	0.588	0.742	1.005	4.19	5.31	4	5	414	47.7	8.53

Q9Y6I3	Epsin-1 OS=Homo sapiens GN=EPN1 PE=1 SV=2 - [EPN1_HUMAN]	23.09	1	11	11	26	0.993	1.302	1.164	1.005	67.97	23.09	16	26	576	60.3	4.83
P04439	HLA class I histocompatibility antigen, A-3 alpha chain OS=Homo sapiens GN=HLA-A PE=1 SV=2 - [1A03_HUMAN]	52.60	6	4	20	173	2.512	0.809	1.259	1.005	417.39	52.60	33	173	365	40.8	6.00
P22234	Multifunctional protein ADE2 OS=Homo sapiens GN=PAICS PE=1 SV=3 - [PUR6_HUMAN]	29.65	1	16	16	56	0.784	0.981	0.690	1.005	125.10	29.65	23	56	425	47.0	7.23
Q8NBQ5	Estradiol 17-beta-dehydrogenase 11 OS=Homo sapiens GN=HSD17B11 PE=1 SV=3 - [DHB11_HUMAN]	18.00	1	4	4	9	0.995	1.014	0.811	1.005	23.10	18.00	7	9	300	32.9	9.07
P17813	Endoglin OS=Homo sapiens GN=ENG PE=1 SV=2 - [EGLN_HUMAN]	22.34	1	11	11	22	0.728	0.383	0.514	1.005	37.52	22.34	16	22	658	70.5	6.61
Q9H8S5	Cyclin N-terminal domain-containing protein 2 OS=Homo sapiens GN=CNTD2 PE=2 SV=2 - [CNTD2_HUMAN	4.89	1	1	1	2	0.730	0.990	1.097	1.005	6.70	4.89	1	2	307	33.6	8.60
Q96RF0	Sorting nexin-18 OS=Homo sapiens GN=SNX18 PE=1 SV=2 - [SNX18_HUMAN]	27.87	1	15	16	97	0.437	0.743	0.868	1.005	258.36	27.87	25	97	628	68.9	5.68
Q8N766	ER membrane protein complex subunit 1 OS=Homo sapiens GN=EMC1 PE=1 SV=1 - [EMC1_HUMAN]	20.75	1	17	17	59	1.021	0.765	0.907	1.005	157.08	20.75	28	59	993	111.7	7.66
O95202	LETM1 and EF-hand domain-containing protein 1, mitochondrial OS=Homo sapiens GN=LETM1 PE=1 SV=1 - [LETM1_HUMAN]	35.18	1	24	24	167	0.904	0.981	0.862	1.005	467.36	35.18	38	167	739	83.3	6.70

Q658Y4	Protein FAM91A1 OS=Homo sapiens GN=FAM91A1 PE=1 SV=3 - [F91A1_HUMAN]	6.09	4	4	5	21	1.055	0.975	0.849	1.005	44.74	6.09	8	21	838	93.8	6.39
O94888	UBX domain-containing protein 7 OS=Homo sapiens GN=UBXN7 PE=1 SV=2 - [UBXN7_HUMAN]	9.20	1	5	5	13	0.963	0.997	0.723	1.006	28.91	9.20	8	13	489	54.8	5.16
Q99829	Copine-1 OS=Homo sapiens GN=CPNE1 PE=1 SV=1 - [CPNE1_HUMAN]	29.24	1	15	15	58	0.992	1.083	1.232	1.006	125.27	29.24	23	58	537	59.0	5.83
P38646	Stress-70 protein, mitochondrial OS=Homo sapiens GN=HSPA9 PE=1 SV=2 - [GRP75_HUMAN]	61.86	1	46	47	639	0.812	1.131	0.957	1.006	1536.79	61.86	79	639	679	73.6	6.16
Q9NRF8	CTP synthase 2 OS=Homo sapiens GN=CTPS2 PE=1 SV=1 - [PYRG2_HUMAN]	14.68	1	5	7	19	1.060	1.005	1.065	1.006	41.24	14.68	10	19	586	65.6	6.90
Q9UGP4	LIM domain-containing protein 1 OS=Homo sapiens GN=LIMD1 PE=1 SV=1 - [LIMD1_HUMAN]	17.16	1	9	9	35	0.936	1.328	1.695	1.006	75.15	17.16	15	35	676	72.1	6.65
P22694	cAMP-dependent protein kinase catalytic subunit beta OS=Homo sapiens GN=PRKACB PE=1 SV=2 - [KAPCB_HUMAN	29.63	2	4	14	33	0.692	0.758	0.803	1.006	73.98	29.63	19	33	351	40.6	8.78
Q6PJP8	DNA cross-link repair 1A protein OS=Homo sapiens GN=DCLRE1A PE=1 SV=3 - [DCR1A_HUMAN]	3.75	1	2	3	6	0.599	0.839	1.007	1.006	12.93	3.75	4	6	1040	116.3	7.97
P04220	Ig mu heavy chain disease protein OS=Homo sapiens PE=1 SV=1 - [MUCB_HUMAN]	3.84	2	1	1	1	1.126	1.294	1.411	1.006	0.00	3.84	1	1	391	43.0	5.24

Q14202	Zinc finger MYM-type protein 3 OS=Homo sapiens GN=ZMYM3 PE=1 SV=2 - [ZMYM3_HUMAN]	1.17	1	1	1	5	1.004	1.793	1.590	1.006	17.56	1.17	2	5	1370	152.3	6.35
Q49B96	Cytochrome c oxidase assembly protein COX19 OS=Homo sapiens GN=COX19 PE=1 SV=1 - [COX19_HUMAN]	21.11	1	2	2	3	1.101	1.438	1.413	1.006	4.52	21.11	3	3	90	10.4	8.72
Q9Y3A2	Probable U3 small nucleolar RNA-associated protein 11 OS=Homo sapiens GN=UTP11L PE=1 SV=2 - [UTP11_HUMAN]	4.74	1	1	1	2	1.143	1.512	1.014	1.006	5.57	4.74	1	2	253	30.4	10.15
Q9Y666	Solute carrier family 12 member 7 OS=Homo sapiens GN=SLC12A7 PE=1 SV=3 - [S12A7_HUMAN]	7.29	1	7	7	17	1.104	0.754	0.902	1.007	48.50	7.29	9	17	1083	119.0	6.71
P13284	Gamma-interferon-inducible lysosomal thiol reductase OS=Homo sapiens GN=IFI30 PE=1 SV=3 - [GILT_HUMAN]	4.40	1	1	1	2	0.924	1.950	0.788	1.007	4.26	4.40	1	2	250	27.9	4.88
Q13151	Heterogeneous nuclear ribonucleoprotein A0 OS=Homo sapiens GN=HNRNPA0 PE=1 SV=1 - [ROA0_HUMAN]	41.64	1	8	10	36	0.944	0.919	0.885	1.007	97.60	41.64	15	36	305	30.8	9.29
P25685	DnaJ homolog subfamily B member 1 OS=Homo sapiens GN=DNAJB1 PE=1 SV=4 - [DNJB1_HUMAN]	29.12	1	11	11	24	1.016	1.026	0.872	1.007	49.49	29.12	16	24	340	38.0	8.63
P48059	LIM and senescent cell antigen-like-containing domain protein 1 OS=Homo sapiens GN=LIMS1 PE=1 SV=4 - [LIMS1_HUMAN]	22.15	1	5	8	31	1.195	1.300	0.914	1.007	78.64	22.15	14	31	325	37.2	8.05

A8MPX8	Protein phosphatase 2C-like domain-containing protein 1 OS=Homo sapiens GN=PP2D1 PE=2 SV=2 - [PP2D1_HUMAN]	2.86	1	1	1	2	1.522	0.273	0.494	1.007	0.00	2.86	1	2	630	71.6	8.72
Q9NZB2	Constitutive coactivator of PPAR-gamma-like protein 1 OS=Homo sapiens GN=FAM120A PE=1 SV=2 - [F120A_HUMAN]	25.76	2	19	21	111	1.076	1.009	0.956	1.007	307.99	25.76	34	111	1118	121.8	8.88
O94992	Protein HEXIM1 OS=Homo sapiens GN=HEXIM1 PE=1 SV=1 - [HEXI1_HUMAN]	14.48	1	5	5	14	1.227	1.284	0.920	1.007	43.49	14.48	8	14	359	40.6	4.89
Q15814	Tubulin-specific chaperone C OS=Homo sapiens GN=TBCC PE=1 SV=2 - [TBCC_HUMAN]	25.72	1	7	8	31	1.013	1.000	1.074	1.007	82.10	25.72	13	31	346	39.2	5.71
Q8N9V6	Ankyrin repeat domain-containing protein 53 OS=Homo sapiens GN=ANKRD53 PE=2 SV=3 - [ANR53_HUMAN]	3.96	1	1	1	1	0.826	0.595	0.834	1.007	2.48	3.96	1	1	530	59.5	9.52
P49327	Fatty acid synthase OS=Homo sapiens GN=FASN PE=1 SV=3 - [FAS_HUMAN]	35.88	1	68	70	288	0.883	0.681	1.046	1.007	607.58	35.88	117	288	2511	273.3	6.44
Q14746	Conserved oligomeric Golgi complex subunit 2 OS=Homo sapiens GN=COG2 PE=1 SV=1 - [COG2_HUMAN]	4.34	1	4	4	7	1.018	0.819	1.048	1.007	12.27	4.34	5	7	738	83.2	6.62
Q96P16	Regulation of nuclear pre-mRNA domain-containing protein 1A OS=Homo sapiens GN=RPRD1A PE=1 SV=1 - [RPR1A_HUMAN]	11.86	1	2	4	5	0.741	1.063	1.089	1.008	9.22	11.86	4	5	312	35.7	7.55

P40938	Replication factor C subunit 3 OS=Homo sapiens GN=RFC3 PE=1 SV=2 - [RFC3_HUMAN]	3.37	1	1	1	4	1.181	1.221	1.476	1.008	13.27	3.37	2	4	356	40.5	8.34
Q8TBB5	Kelch domain-containing protein 4 OS=Homo sapiens GN=KLHDC4 PE=1 SV=1 - [KLDC4_HUMAN]	4.62	1	2	2	3	0.782	1.088	1.535	1.008	5.57	4.62	3	3	520	57.9	5.72
Q9UHP3	Ubiquitin carboxyl-terminal hydrolase 25 OS=Homo sapiens GN=USP25 PE=1 SV=4 - [UBP25_HUMAN]	4.83	2	4	6	14	0.850	1.086	1.016	1.008	21.11	4.83	8	14	1055	122.1	5.34
P16234	Platelet-derived growth factor receptor alpha OS=Homo sapiens GN=PDGFRA PE=1 SV=1 - [PGFRA_HUMAN	17.81	6	16	18	49	2.569	0.990	0.661	1.008	117.67	17.81	28	49	1089	122.6	5.17
Q7Z3E5	LisH domain-containing protein ARMC9 OS=Homo sapiens GN=ARMC9 PE=1 SV=2 - [ARMC9_HUMAN]	9.42	1	7	8	23	1.593	0.700	1.495	1.008	57.48	9.42	13	23	817	91.7	6.20
Q6NUQ1	RAD50-interacting protein 1 OS=Homo sapiens GN=RINT1 PE=1 SV=1 - [RINT1_HUMAN]	4.17	1	3	4	5	0.746	0.794	0.701	1.008	7.96	4.17	4	5	792	90.6	5.45
Q9BR61	Acyl-CoA-binding domain-containing protein 6 OS=Homo sapiens GN=ACBD6 PE=1 SV=1 - [ACBD6_HUMAN]	13.12	1	4	4	21	1.166	1.032	0.955	1.008	23.72	13.12	6	21	282	31.1	5.11
Q6UXV4	MICOS complex subunit MIC27 OS=Homo sapiens GN=APOOL PE=1 SV=1 - [MIC27_HUMAN]	29.85	1	5	5	15	0.942	0.849	1.329	1.008	50.95	29.85	6	15	268	29.1	9.52

Q13347	Eukaryotic translation initiation factor 3 subunit I OS=Homo sapiens GN=EIF3I PE=1 SV=1 - [EIF3I_HUMAN]	45.23	1	13	14	69	0.995	0.969	1.037	1.008	169.16	45.23	22	69	325	36.5	5.64
Q9NSD9	Phenylalanine--tRNA ligase beta subunit OS=Homo sapiens GN=FARSB PE=1 SV=3 - [SYFB_HUMAN]	39.56	2	24	24	77	0.888	0.994	0.977	1.008	206.20	39.56	36	77	589	66.1	6.84
Q5VTT5	Myomesin-3 OS=Homo sapiens GN=MYOM3 PE=2 SV=1 - [MYOM3_HUMAN]	0.84	1	1	1	1	0.626	0.973	0.538	1.008	2.71	0.84	1	1	1437	162.1	6.23
Q96FV2	Secernin-2 OS=Homo sapiens GN=SCRN2 PE=1 SV=3 - [SCRN2_HUMAN]	14.35	1	4	4	17	1.322	0.815	1.003	1.008	48.85	14.35	7	17	425	46.6	5.67
Q9P2X3	Protein IMPACT OS=Homo sapiens GN=IMPACT PE=1 SV=2 - [IMPCT_HUMAN]	19.06	1	5	5	18	1.134	1.019	0.954	1.008	48.98	19.06	8	18	320	36.5	4.97
Q0GE19	Sodium/bile acid cotransporter 7 OS=Homo sapiens GN=SLC10A7 PE=2 SV=1 - [NTCP7_HUMAN	1.68	1	1	1	1	0.397		0.103	1.009	1.98	1.68	1	1	358	39.5	9.50
Q08AM6	Protein VAC14 homolog OS=Homo sapiens GN=VAC14 PE=1 SV=1 - [VAC14_HUMAN]	9.59	1	7	7	15	1.040	0.994	1.184	1.009	39.16	9.59	11	15	782	87.9	6.13
Q5T0F9	Coiled-coil and C2 domain-containing protein 1B OS=Homo sapiens GN=CC2D1B PE=1 SV=1 - [C2D1B_HUMAN]	8.51	1	7	8	21	1.031	1.124	1.238	1.009	41.27	8.51	12	21	858	94.2	5.26
Q14008	Cytoskeleton-associated protein 5 OS=Homo sapiens GN=CKAP5 PE=1 SV=3 - [CKAP5_HUMAN]	27.36	1	54	55	167	0.864	0.991	0.910	1.009	344.78	27.36	88	167	2032	225.4	7.80

Q8NE01	Metal transporter CNNM3 OS=Homo sapiens GN=CNNM3 PE=1 SV=1 - [CNNM3_HUMA	6.36	1	3	4	8	1.208	1.044	1.075	1.009	12.48	6.36	6	8	707	76.1	6.09
Q00577	Transcriptional activator protein Pur-alpha OS=Homo sapiens GN=PURA PE=1 SV=2 - [PURA_HUMAN]	23.91	3	5	8	25	1.448	0.955	1.013	1.009	52.79	23.91	10	25	322	34.9	6.44
P16383	GC-rich sequence DNA-binding factor 2 OS=Homo sapiens GN=GCFC2 PE=1 SV=2 - [GCFC2_HUMAN]	5.12	1	4	4	20	1.225	0.816	0.954	1.009	58.69	5.12	6	20	781	89.3	5.99
Q4VC31	Coiled-coil domain-containing protein 58 OS=Homo sapiens GN=CCDC58 PE=1 SV=1 - [CCD58_HUMAN]	23.61	1	3	3	9	0.918	1.021	0.891	1.009	14.11	23.61	4	9	144	16.6	7.81
Q8IYD1	Eukaryotic peptide chain release factor GTP-binding subunit ERF3B OS=Homo sapiens GN=GSPT2 PE=1 SV=2 - [ERF3B_HUMAN]	28.18	1	4	22	79	1.046	1.085	1.129	1.009	177.64	28.18	34	79	628	68.8	5.43
Q8NFQ8	Torsin-1A-interacting protein 2 OS=Homo sapiens GN=TOR1AIP2 PE=1 SV=1 - [TOIP2_HUMAN]	35.53	1	12	13	34	1.213	1.115	1.018	1.009	92.61	35.53	19	34	470	51.2	4.96
Q16563	Synaptophysin-like protein 1 OS=Homo sapiens GN=SYPL1 PE=1 SV=1 - [SYPL1_HUMAN]	2.32	1	1	1	2	1.279	1.051	0.924	1.009	2.09	2.32	1	2	259	28.5	8.43
Q6PCE3	Glucose 1,6-bisphosphate synthase OS=Homo sapiens GN=PGM2L1 PE=1 SV=3 - [PGM2L_HUMAN]	13.34	1	10	10	30	1.190	1.220	0.859	1.009	57.13	13.34	15	30	622	70.4	7.15
P52758	Ribonuclease UK114 OS=Homo sapiens GN=HRSP12 PE=1 SV=1 - [UK114_HUMAN]	38.69	1	5	5	11	0.582	1.037	0.578	1.009	24.03	38.69	6	11	137	14.5	8.68

Q9UNS2	COP9 signalosome complex subunit 3 OS=Homo sapiens GN=COPS3 PE=1 SV=3 - [CSN3_HUMAN]	30.02	1	11	11	35	0.888	1.030	0.840	1.009	92.48	30.02	18	35	423	47.8	6.65
Q8WVY7	Ubiquitin-like domain-containing CTD phosphatase 1 OS=Homo sapiens GN=UBLCP1 PE=1 SV=2 - [UBCP1_HUMAN]	28.93	1	12	12	52	0.939	0.916	1.052	1.009	105.82	28.93	18	52	318	36.8	6.46
O75074	Low-density lipoprotein receptor-related protein 3 OS=Homo sapiens GN=LRP3 PE=2 SV=2 - [LRP3_HUMAN]	2.34	1	1	1	3	1.143	1.049	1.183	1.009	0.00	2.34	1	3	770	82.8	6.21
Q15283	Ras GTPase-activating protein 2 OS=Homo sapiens GN=RASA2 PE=1 SV=3 - [RASA2_HUMAN]	5.88	1	4	6	13	0.977	0.868	0.848	1.009	26.29	5.88	9	13	850	96.6	7.20
Q9P2R7	Succinyl-CoA ligase [ADP-forming] subunit beta, mitochondrial OS=Homo sapiens GN=SUCLA2 PE=1 SV=3 - [SUCB1_HUMAN]	23.33	1	11	11	43	1.036	1.108	1.022	1.009	87.55	23.33	18	43	463	50.3	7.42
P04626	Receptor tyrosine-protein kinase erbB-2 OS=Homo sapiens GN=ERBB2 PE=1 SV=1 - [ERBB2_HUMAN	4.94	4	3	6	17	1.355	0.811	0.884	1.009	29.00	4.94	10	17	1255	137.8	5.91
Q9C0B5	Palmitoyltransferase ZDHHC5 OS=Homo sapiens GN=ZDHHC5 PE=1 SV=2 - [ZDHC5_HUMAN]	18.88	1	11	11	26	1.428	0.889	0.985	1.010	63.86	18.88	15	26	715	77.5	9.01
Q9BSR8	Protein YIPF4 OS=Homo sapiens GN=YIPF4 PE=1 SV=1 - [YIPF4_HUMAN]	7.38	1	2	2	9	0.923	0.917	0.947	1.010	16.61	7.38	4	9	244	27.1	4.65

P78371	T-complex protein 1 subunit beta OS=Homo sapiens GN=CCT2 PE=1 SV=4 - [TCPB_HUMAN]	68.97	1	33	33	345	0.944	1.038	1.081	1.010	873.23	68.97	58	345	535	57.5	6.46
Q9Y227	Ectonucleoside triphosphate diphosphohydrolase 4 OS=Homo sapiens GN=ENTPD4 PE=1 SV=1 - [ENTP4_HUMAN]	1.95	1	1	1	1	1.156	0.724	1.058	1.010	2.72	1.95	1	1	616	70.2	8.29
Q6PI78	Transmembrane protein 65 OS=Homo sapiens GN=TMEM65 PE=1 SV=2 - [TMM65_HUMAN]	13.75	1	3	3	10	1.202	1.065	0.681	1.010	15.77	13.75	5	10	240	25.5	8.60
Q9Y2V2	Calcium-regulated heat stable protein 1 OS=Homo sapiens GN=CARHSP1 PE=1 SV=2 - [CHSP1_HUMAN]	51.70	1	5	5	20	1.345	0.931	0.817	1.010	49.93	51.70	6	20	147	15.9	8.21
Q96GP6	Scavenger receptor class F member 2 OS=Homo sapiens GN=SCARF2 PE=1 SV=4 - [SREC2_HUMAN]	10.46	1	8	8	20	0.966	0.662	0.737	1.010	40.81	10.46	12	20	870	92.4	8.44
Q86UT6	NLR family member X1 OS=Homo sapiens GN=NLRX1 PE=1 SV=1 - [NLRX1_HUMAN]	6.36	1	5	5	14	0.843	0.885	1.013	1.010	39.25	6.36	8	14	975	107.5	7.37
Q9UNH7	Sorting nexin-6 OS=Homo sapiens GN=SNX6 PE=1 SV=1 - [SNX6_HUMAN]	36.95	2	19	21	111	1.114	0.898	0.959	1.010	263.17	36.95	35	111	406	46.6	6.16
Q86X27	Ras-specific guanine nucleotide-releasing factor RalGPS2 OS=Homo sapiens GN=RALGPS2 PE=1 SV=1 - [RGPS2_HUMAN]	11.32	2	6	6	15	0.819	0.862	1.208	1.010	34.16	11.32	9	15	583	65.1	8.73
Q9NXC5	WD repeat-containing protein mio OS=Homo sapiens GN=MIOS PE=1 SV=2 - [MIO_HUMAN]	6.86	1	5	5	13	0.915	0.936	0.874	1.010	32.24	6.86	8	13	875	98.5	6.73

Q96DN5	TBC1 domain family member 31 OS=Homo sapiens GN=TBC1D31 PE=2 SV=2 - [TBC31_HUMAN]	0.84	1	1	1	1		1.324	0.881	1.010	2.38	0.84	1	1	1066	124.1	8.62
Q9BXC9	Bardet-Biedl syndrome 2 protein OS=Homo sapiens GN=BBS2 PE=1 SV=1 - [BBS2_HUMAN]	1.94	1	1	1	6	0.908	1.055	0.828	1.010	18.15	1.94	2	6	721	79.8	6.15
O75427	Leucine-rich repeat and calponin homology domain-containing protein 4 OS=Homo sapiens GN=LRCH4 PE=1 SV=2 - [LRCH4_HUMAN]	14.20	1	7	7	18	0.970	1.127	1.323	1.010	30.19	14.20	11	18	683	73.4	8.22
Q9P0L0	Vesicle-associated membrane protein-associated protein A OS=Homo sapiens GN=VAPA PE=1 SV=3 - [VAPA_HUMAN]	46.99	1	14	15	120	1.133	1.069	0.930	1.010	257.86	46.99	23	120	249	27.9	8.62
P49454	Centromere protein F OS=Homo sapiens GN=CENPF PE=1 SV=2 - [CENPF_HUMAN]	4.08	2	7	15	28	1.480	0.958	1.609	1.010	59.46	4.08	16	28	3210	367.5	5.07
Q92997	Segment polarity protein dishevelled homolog DVL-3 OS=Homo sapiens GN=DVL3 PE=1 SV=2 - [DVL3_HUMAN]	10.20	3	7	7	15	0.968	1.203	1.371	1.010	26.34	10.20	10	15	716	78.0	6.65
Q63HN8	E3 ubiquitin-protein ligase RNF213 OS=Homo sapiens GN=RNF213 PE=1 SV=3 - [RN213_HUMAN]	10.29	1	44	46	105	1.526	1.086	1.035	1.011	236.92	10.29	68	105	5207	591.0	6.48
Q9HCM3	UPF0606 protein KIAA1549 OS=Homo sapiens GN=KIAA1549 PE=1 SV=4 - [K1549_HUMAN]	0.36	1	1	1	3	0.818	0.958	0.892	1.011	2.09	0.36	2	3	1950	210.6	6.11

Q96DD0	Leucine-rich repeat-containing protein 39 OS=Homo sapiens GN=LRRC39 PE=2 SV=1 - [LRC39_HUMAN]	7.76	1	1	2	3	0.679	0.741	1.068	1.011	0.00	7.76	2	3	335	38.8	6.34
O14562	Ubiquitin domain-containing protein UBFD1 OS=Homo sapiens GN=UBFD1 PE=1 SV=2 - [UBFD1_HUMAN]	25.89	1	9	9	26	0.813	1.143	0.926	1.011	59.47	25.89	12	26	309	33.4	5.77
P07902	Galactose-1-phosphate uridylyltransferase OS=Homo sapiens GN=GALT PE=1 SV=3 - [GALT_HUMAN]	16.62	1	5	5	18	1.208	1.026	1.052	1.011	46.57	16.62	8	18	379	43.3	6.99
Q6P5R6	60S ribosomal protein L22-like 1 OS=Homo sapiens GN=RPL22L1 PE=1 SV=2 - [RL22L_HUMAN]	45.08	1	4	4	12	0.711	1.467	1.392	1.011	22.38	45.08	5	12	122	14.6	9.38
O00139	Kinesin-like protein KIF2A OS=Homo sapiens GN=KIF2A PE=1 SV=3 - [KIF2A_HUMAN]	14.45	2	11	11	26	0.819	1.035	0.978	1.011	48.36	14.45	16	26	706	79.9	6.68
Q9H6X2	Anthrax toxin receptor 1 OS=Homo sapiens GN=ANTXR1 PE=1 SV=2 - [ANTR1_HUMAN]	17.20	1	8	11	43	1.656	0.410	0.703	1.011	87.86	17.20	18	43	564	62.7	7.61
P62851	40S ribosomal protein S25 OS=Homo sapiens GN=RPS25 PE=1 SV=1 - [RS25_HUMAN]	42.40	1	8	8	155	0.863	1.059	0.918	1.011	276.11	42.40	14	155	125	13.7	10.11
Q9NUQ8	ATP-binding cassette sub-family F member 3 OS=Homo sapiens GN=ABCF3 PE=1 SV=2 - [ABCF3_HUMAN]	19.61	1	13	13	33	0.963	0.783	1.052	1.011	69.99	19.61	20	33	709	79.7	6.34
Q9BX67	Junctional adhesion molecule C OS=Homo sapiens GN=JAM3 PE=1 SV=1 - [JAM3_HUMAN]	9.35	1	3	3	8	1.686	0.959	1.075	1.011	17.91	9.35	5	8	310	35.0	7.59

P09525	Annexin A4 OS=Homo sapiens GN=ANXA4 PE=1 SV=4 - [ANXA4_HUMAN]	68.34	1	23	24	249	0.956	1.133	1.068	1.011	600.15	68.34	41	249	319	35.9	6.13
Q13363	C-terminal-binding protein 1 OS=Homo sapiens GN=CTBP1 PE=1 SV=2 - [CTBP1_HUMAN]	22.95	1	6	9	36	1.014	1.194	1.008	1.012	96.59	22.95	17	36	440	47.5	6.77
Q9UQ16	Dynamin-3 OS=Homo sapiens GN=DNM3 PE=1 SV=4 - [DYN3_HUMAN]	11.97	1	1	13	37	1.280	1.184	1.528	1.012	57.81	11.97	18	37	869	97.7	8.35
Q96LQ0	Protein phosphatase 1 regulatory subunit 36 OS=Homo sapiens GN=PPP1R36 PE=1 SV=1 - [PPR36_HUMAN]	1.66	1	1	1	1	0.230	1.163	3.075	1.012	2.54	1.66	1	1	422	49.4	9.36
O75581	Low-density lipoprotein receptor-related protein 6 OS=Homo sapiens GN=LRP6 PE=1 SV=2 - [LRP6_HUMAN]	1.12	1	2	2	5	0.798	0.836	0.970	1.012	11.29	1.12	2	5	1613	180.3	5.31
O14681	Etoposide-induced protein 2.4 homolog OS=Homo sapiens GN=EI24 PE=1 SV=4 - [EI24_HUMAN]	6.47	1	2	3	13	0.746	1.039	0.874	1.012	27.19	6.47	4	13	340	38.9	9.72
P62750	60S ribosomal protein L23a OS=Homo sapiens GN=RPL23A PE=1 SV=1 - [RL23A_HUMAN]	52.56	1	14	14	145	1.059	1.292	1.024	1.012	376.75	52.56	24	145	156	17.7	10.45
Q96J42	Thioredoxin domain-containing protein 15 OS=Homo sapiens GN=TXNDC15 PE=1 SV=1 - [TXD15_HUMAN]	11.39	1	2	3	7	0.592	1.036	0.806	1.012	10.61	11.39	3	7	360	39.9	4.83
Q9NVX2	Notchless protein homolog 1 OS=Homo sapiens GN=NLE1 PE=1 SV=4 - [NLE1_HUMAN]	2.27	1	1	1	4	1.024	0.780	0.670	1.012	14.62	2.27	1	4	485	53.3	7.34

O00458	Interferon-related developmental regulator 1 OS=Homo sapiens GN=IFRD1 PE=1 SV=4 - [IFRD1_HUMAN]	1.77	1	1	1	1	0.702	1.064	0.825	1.012	2.51	1.77	1	1	451	50.2	7.18
Q92626	Peroxidasin homolog OS=Homo sapiens GN=PXDN PE=1 SV=2 - [PXDN_HUMAN]	23.87	1	28	29	102	1.182	0.591	0.679	1.012	280.45	23.87	47	102	1479	165.2	7.17
Q6IQ26	DENN domain-containing protein 5A OS=Homo sapiens GN=DENND5A PE=1 SV=2 - [DEN5A_HUMAN]	4.43	1	3	5	11	1.072	0.904	1.094	1.012	17.53	4.43	6	11	1287	147.0	6.65
A6NIH7	Protein unc-119 homolog B OS=Homo sapiens GN=UNC119B PE=1 SV=1 - [U119B_HUMAN]	38.65	1	6	6	15	1.114	1.201	1.148	1.012	33.85	38.65	9	15	251	28.1	5.68
Q7L1W4	Volume-regulated anion channel subunit LRRC8D OS=Homo sapiens GN=LRRC8D PE=1 SV=1 - [LRC8D_HUMAN]	4.08	1	2	3	4	1.099	1.257	0.990	1.012	2.00	4.08	4	4	858	98.1	7.72
P08473	Neprilysin OS=Homo sapiens GN=MME PE=1 SV=2 - [NEP_HUMAN]	12.80	1	9	9	15	3.612	0.665	0.919	1.012	32.67	12.80	12	15	750	85.5	5.73
O43617	Trafficking protein particle complex subunit 3 OS=Homo sapiens GN=TRAPPC3 PE=1 SV=1 - [TPPC3_HUMAN]	31.11	1	5	5	25	1.274	1.200	1.190	1.012	44.39	31.11	9	25	180	20.3	4.96
Q9HAU5	Regulator of nonsense transcripts 2 OS=Homo sapiens GN=UPF2 PE=1 SV=1 - [RENT2_HUMAN]	8.88	1	9	9	27	0.939	0.899	0.842	1.013	57.42	8.88	11	27	1272	147.7	5.69
Q96JJ3	Engulfment and cell motility protein 2 OS=Homo sapiens GN=ELMO2 PE=1 SV=2 - [ELMO2_HUMAN]	19.31	1	13	13	33	1.078	0.767	0.852	1.013	70.71	19.31	19	33	720	82.6	5.90

Q6ZTQ4	Cadherin-related family member 3 OS=Homo sapiens GN=CDHR3 PE=2 SV=1 - [CDHR3_HUMAN	2.49	1	2	2	4	1.119	0.925	1.216	1.013	1.94	2.49	2	4	885	97.9	5.41
Q9C0C9	E2/E3 hybrid ubiquitin-protein ligase UBE2O OS=Homo sapiens GN=UBE2O PE=1 SV=3 - [UBE2O_HUMAN]	10.99	1	11	12	23	0.926	1.035	0.974	1.013	64.71	10.99	14	23	1292	141.2	5.12
P56537	Eukaryotic translation initiation factor 6 OS=Homo sapiens GN=EIF6 PE=1 SV=1 - [IF6_HUMAN]	26.94	1	4	4	26	1.136	0.970	1.208	1.013	59.77	26.94	7	26	245	26.6	4.68
P18433	Receptor-type tyrosine-protein phosphatase alpha OS=Homo sapiens GN=PTPRA PE=1 SV=2 - [PTPRA_HUMAN	3.24	1	3	3	6	1.688	1.032	1.078	1.013	6.32	3.24	4	6	802	90.5	6.70
P46934	E3 ubiquitin-protein ligase NEDD4 OS=Homo sapiens GN=NEDD4 PE=1 SV=4 - [NEDD4_HUMAN]	5.31	1	5	5	6	0.796	0.916	0.629	1.013	18.89	5.31	6	6	1319	149.0	6.58
Q969R8	Integrin-alpha FG-GAP repeat-containing protein 2 OS=Homo sapiens GN=ITFG2 PE=1 SV=1 - [ITFG2_HUMAN]	6.71	1	2	2	3	1.867	1.302	1.519	1.013	3.36	6.71	2	3	447	49.3	5.22
P42766	60S ribosomal protein L35 OS=Homo sapiens GN=RPL35 PE=1 SV=2 - [RL35_HUMAN]	43.09	1	8	10	69	0.846	0.697	0.840	1.014	85.06	43.09	15	69	123	14.5	11.05
Q9BRZ2	E3 ubiquitin-protein ligase TRIM56 OS=Homo sapiens GN=TRIM56 PE=1 SV=3 - [TRI56_HUMAN]	21.19	2	13	13	41	0.882	0.990	1.045	1.014	73.56	21.19	21	41	755	81.4	7.74
O75954	Tetraspanin-9 OS=Homo sapiens GN=TSPAN9 PE=1 SV=1 - [TSN9_HUMAN]	6.28	1	1	1	17	1.076	0.809	0.912	1.014	17.75	6.28	2	17	239	26.8	7.68

O00399	Dynactin subunit 6 OS=Homo sapiens GN=DCTN6 PE=1 SV=1 - [DCTN6_HUMAN]	31.58	1	5	5	34	1.141	1.030	0.888	1.014	76.74	31.58	10	34	190	20.7	6.32
P47712	Cytosolic phospholipase A2 OS=Homo sapiens GN=PLA2G4A PE=1 SV=2 - [PA24A_HUMAN]	8.68	1	7	7	16	0.974	0.906	0.931	1.014	34.13	8.68	9	16	749	85.2	5.38
Q9UK41	Vacuolar protein sorting-associated protein 28 homolog OS=Homo sapiens GN=VPS28 PE=1 SV=1 - [VPS28_HUMAN]	44.34	1	9	9	29	0.879	1.061	1.160	1.014	52.64	44.34	12	29	221	25.4	5.54
P45974	Ubiquitin carboxyl-terminal hydrolase 5 OS=Homo sapiens GN=USP5 PE=1 SV=2 - [UBP5_HUMAN]	35.08	1	26	26	117	1.172	1.102	1.263	1.014	278.66	35.08	43	117	858	95.7	5.03
Q9Y2S7	Polymerase delta-interacting protein 2 OS=Homo sapiens GN=POLDIP2 PE=1 SV=1 - [PDIP2_HUMAN	22.28	1	7	7	21	0.771	1.222	1.053	1.014	48.06	22.28	13	21	368	42.0	8.63
P17405	Sphingomyelin phosphodiesterase OS=Homo sapiens GN=SMPD1 PE=1 SV=4 - [ASM_HUMAN]	6.04	1	3	3	4	0.529	1.163	0.960	1.014	7.30	6.04	4	4	629	69.7	7.28
Q92572	AP-3 complex subunit sigma-1 OS=Homo sapiens GN=AP3S1 PE=1 SV=1 - [AP3S1_HUMAN]	37.82	1	7	7	33	1.014	0.910	0.991	1.014	66.69	37.82	14	33	193	21.7	5.39
Q9BQA9	Uncharacterized protein C17orf62 OS=Homo sapiens GN=C17orf62 PE=1 SV=1 - [CQ062_HUMAN]	16.04	1	3	3	11	1.058	0.766	0.767	1.015	29.31	16.04	5	11	187	20.8	6.80
Q02878	60S ribosomal protein L6 OS=Homo sapiens GN=RPL6 PE=1 SV=3 - [RL6_HUMAN]	44.44	1	17	17	188	0.903	1.023	0.993	1.015	373.81	44.44	33	188	288	32.7	10.58

Q96L58	Beta-1,3-galactosyltransferase 6 OS=Homo sapiens GN=B3GALT6 PE=1 SV=2 - [B3GT6_HUMAN]	6.99	1	2	2	8	0.673	0.973	1.368	1.015	18.99	6.99	3	8	329	37.1	9.66
P51636	Caveolin-2 OS=Homo sapiens GN=CAV2 PE=1 SV=2 - [CAV2_HUMAN]	3.70	1	1	1	5	2.944	0.623	0.961	1.015	14.53	3.70	2	5	162	18.3	5.27
Q9H0G5	Nuclear speckle splicing regulatory protein 1 OS=Homo sapiens GN=NSRP1 PE=1 SV=1 - [NSRP1_HUMAN]	1.08	1	1	1	3	0.696	1.144		1.015	9.14	1.08	1	3	558	66.4	8.84
P31644	Gamma-aminobutyric acid receptor subunit alpha-5 OS=Homo sapiens GN=GABRA5 PE=1 SV=1 - [GBRA5_HUMAN]	3.46	2	2	2	2	0.872	0.978	0.936	1.015	3.80	3.46	2	2	462	52.1	9.16
Q16774	Guanylate kinase OS=Homo sapiens GN=GUK1 PE=1 SV=2 - [KGUA_HUMAN]	9.64	1	3	3	19	1.014	0.956	0.883	1.015	43.47	9.64	5	19	197	21.7	6.55
Q6AZZ1	E3 ubiquitin-protein ligase TRIM68 OS=Homo sapiens GN=TRIM68 PE=1 SV=1 - [TRI68_HUMAN]	7.01	1	2	2	4	0.738	0.479	0.777	1.015	6.75	7.01	2	4	485	56.2	6.48
P78344	Eukaryotic translation initiation factor 4 gamma 2 OS=Homo sapiens GN=EIF4G2 PE=1 SV=1 - [IF4G2_HUMAN]	29.11	1	22	23	77	0.857	0.944	0.928	1.015	174.60	29.11	35	77	907	102.3	7.14
O75312	Zinc finger protein ZPR1 OS=Homo sapiens GN=ZPR1 PE=1 SV=1 - [ZPR1_HUMAN]	16.99	1	7	7	35	0.778	0.946	1.092	1.015	91.41	16.99	14	35	459	50.9	4.73

O43184	Disintegrin and metalloproteinase domain-containing protein 12 OS=Homo sapiens GN=ADAM12 PE=1 SV=3 - [ADA12_HUMAN]	0.88	1	1	1	1	0.871	2.046	1.921	1.015	2.19	0.88	1	1	909	99.5	8.40
Q9H944	Mediator of RNA polymerase II transcription subunit 20 OS=Homo sapiens GN=MED20 PE=1 SV=1 - [MED20_HUMAN]	10.38	1	2	2	9	1.195	0.853	1.016	1.015	27.09	10.38	4	9	212	23.2	6.87
Q9UPY5	Cystine/glutamate transporter OS=Homo sapiens GN=SLC7A11 PE=1 SV=1 - [XCT_HUMAN]	4.79	1	2	2	7	1.084	0.086	0.370	1.015	15.50	4.79	3	7	501	55.4	9.19
Q9C0J8	pre-mRNA 3' end processing protein WDR33 OS=Homo sapiens GN=WDR33 PE=1 SV=2 - [WDR33_HUMAN]	3.74	1	4	4	8	1.189	1.750	1.476	1.016	13.43	3.74	6	8	1336	145.8	9.17
Q9UBQ5	Eukaryotic translation initiation factor 3 subunit K OS=Homo sapiens GN=EIF3K PE=1 SV=1 - [EIF3K_HUMAN]	33.49	1	6	6	19	1.039	0.985	0.962	1.016	51.43	33.49	9	19	218	25.0	4.93
P00966	Argininosuccinate synthase OS=Homo sapiens GN=ASS1 PE=1 SV=2 - [ASSY_HUMAN]	43.93	1	18	18	116	0.638	0.772	0.865	1.016	215.82	43.93	27	116	412	46.5	8.02
Q9H0N0	Ras-related protein Rab-6C OS=Homo sapiens GN=RAB6C PE=1 SV=2 - [RAB6C_HUMAN]	7.48	1	1	3	5	1.483	1.677	2.256	1.016	9.86	7.48	3	5	254	28.3	7.71
Q99570	Phosphoinositide 3-kinase regulatory subunit 4 OS=Homo sapiens GN=PIK3R4 PE=1 SV=3 - [PI3R4_HUMAN]	5.60	1	4	5	10	0.867	0.907	0.952	1.016	23.90	5.60	7	10	1358	153.0	7.17

Q5HY92	Fidgetin OS=Homo sapiens GN=FIGN PE=1 SV=2 - [FIGN_HUMAN]	1.19	1	1	1	1	1.137	0.956	0.953	1.016	0.00	1.19	1	1	759	82.1	6.71
P53999	Activated RNA polymerase II transcriptional coactivator p15 OS=Homo sapiens GN=SUB1 PE=1 SV=3 - [TCP4_HUMAN]	51.18	1	11	11	52	0.789	0.999	0.702	1.016	107.93	51.18	17	52	127	14.4	9.60
Q8TER5	Rho guanine nucleotide exchange factor 40 OS=Homo sapiens GN=ARHGEF40 PE=1 SV=3 - [ARH40_HUMAN	8.23	1	8	9	17	0.977	1.066	1.031	1.016	45.05	8.23	11	17	1519	164.6	6.15
O15085	Rho guanine nucleotide exchange factor 11 OS=Homo sapiens GN=ARHGEF11 PE=1 SV=1 - [ARHGB_HUMAN	8.54	1	9	10	19	0.990	0.830	1.035	1.016	41.89	8.54	13	19	1522	167.6	5.50
P27695	DNA-(apurinic or apyrimidinic site) lyase OS=Homo sapiens GN=APEX1 PE=1 SV=2 - [APEX1_HUMAN]	42.14	1	16	16	104	1.095	1.117	0.924	1.016	310.44	42.14	25	104	318	35.5	8.12
P22897	Macrophage mannose receptor 1 OS=Homo sapiens GN=MRC1 PE=1 SV=1 - [MRC1_HUMAN]	1.51	1	1	2	2	0.415	1.125	1.544	1.016	2.63	1.51	2	2	1456	165.9	6.54
O15347	High mobility group protein B3 OS=Homo sapiens GN=HMGB3 PE=1 SV=4 - [HMGB3_HUMA N]	24.00	1	5	5	13	1.063	1.656	0.760	1.016	35.35	24.00	6	13	200	23.0	8.37
P0C7M7	Acyl-coenzyme A synthetase ACSM4, mitochondrial OS=Homo sapiens GN=ACSM4 PE=2 SV=1 - [ACSM4_HUMA N]	1.38	1	1	1	1	0.888	0.892	0.652	1.016	0.00	1.38	1	1	580	65.7	8.59
Q12882	Dihydropyrimidine dehydrogenase [NADP(+)] OS=Homo sapiens GN=DPYD PE=1 SV=2 - [DPYD_HUMAN]	4.68	1	5	5	10	0.317	1.697	0.921	1.016	25.79	4.68	7	10	1025	111.3	7.05

Q9H553	Alpha-1,3/1,6-mannosyltransferase ALG2 OS=Homo sapiens GN=ALG2 PE=1 SV=1 - [ALG2_HUMAN]	21.88	1	8	8	21	1.086	1.000	1.001	1.016	51.11	21.88	13	21	416	47.1	7.05
Q96P70	Importin-9 OS=Homo sapiens GN=IPO9 PE=1 SV=3 - [IPO9_HUMAN]	12.68	1	12	12	33	0.960	1.147	0.901	1.016	72.48	12.68	18	33	1041	115.9	4.81
Q9H467	CUE domain-containing protein 2 OS=Homo sapiens GN=CUEDC2 PE=1 SV=1 - [CUED2_HUMAN]	3.14	1	1	1	2	0.894	0.743	1.075	1.016	4.36	3.14	2	2	287	32.0	4.81
Q8TCT9	Minor histocompatibility antigen H13 OS=Homo sapiens GN=HM13 PE=1 SV=1 - [HM13_HUMAN]	15.38	1	6	6	45	0.857	0.640	0.706	1.016	109.43	15.38	10	45	377	41.5	6.43
P62854	40S ribosomal protein S26 OS=Homo sapiens GN=RPS26 PE=1 SV=3 - [RS26_HUMAN]	26.96	2	3	3	20	0.859	0.785	0.764	1.017	40.89	26.96	4	20	115	13.0	11.00
Q96N67	Dedicator of cytokinesis protein 7 OS=Homo sapiens GN=DOCK7 PE=1 SV=4 - [DOCK7_HUMAN]	15.56	3	25	28	91	1.072	1.084	0.947	1.017	199.68	15.56	46	91	2140	242.4	6.80
Q15758	Neutral amino acid transporter B(0) OS=Homo sapiens GN=SLC1A5 PE=1 SV=2 - [AAAT_HUMAN]	19.22	1	9	9	103	1.143	0.405	0.504	1.017	211.44	19.22	13	103	541	56.6	5.48
Q8NBI6	Xyloside xylosyltransferase 1 OS=Homo sapiens GN=XXYLT1 PE=1 SV=1 - [XXLT1_HUMAN]	7.38	1	2	2	3	0.964	0.967	0.736	1.017	9.23	7.38	3	3	393	43.8	8.13
P51114	Fragile X mental retardation syndrome-related protein 1 OS=Homo sapiens GN=FXR1 PE=1 SV=3 - [FXR1_HUMAN]	37.36	1	17	20	91	0.909	1.334	1.244	1.017	289.35	37.36	32	91	621	69.7	6.15

Q5VWZ2	Lysophospholipase-like protein 1 OS=Homo sapiens GN=LYPLAL1 PE=1 SV=3 - [LYPL1_HUMAN]	15.61	1	4	4	7	0.819	1.115	0.867	1.017	15.07	15.61	7	7	237	26.3	7.84
Q9P0J0	NADH dehydrogenase [ubiquinone] 1 alpha subcomplex subunit 13 OS=Homo sapiens GN=NDUFA13 PE=1 SV=3 - [NDUAD_HUMAN]	40.97	1	6	6	17	0.800	1.081	0.924	1.017	46.96	40.97	9	17	144	16.7	8.43
Q9UL01	Dermatan-sulfate epimerase OS=Homo sapiens GN=DSE PE=1 SV=1 - [DSE_HUMAN]	2.82	1	3	3	9	0.669	0.956	1.045	1.017	20.73	2.82	4	9	958	109.7	8.09
P17050	Alpha-N-acetylgalactosaminidase OS=Homo sapiens GN=NAGA PE=1 SV=2 - [NAGAB_HUMAN]	9.98	1	4	4	10	1.018	0.929	0.649	1.017	22.84	9.98	7	10	411	46.5	5.19
Q86XX4	Extracellular matrix protein FRAS1 OS=Homo sapiens GN=FRAS1 PE=1 SV=2 - [FRAS1_HUMAN]	0.37	1	1	2	3	1.474	0.780	0.687	1.017	7.34	0.37	2	3	4008	442.9	5.57
Q9NW15	Anoctamin-10 OS=Homo sapiens GN=ANO10 PE=1 SV=2 - [ANO10_HUMAN]	13.18	2	10	10	33	1.039	1.022	0.888	1.017	74.61	13.18	14	33	660	76.3	7.40
Q9UDY2	Tight junction protein ZO-2 OS=Homo sapiens GN=TJP2 PE=1 SV=2 - [ZO2_HUMAN]	16.97	1	16	18	64	0.797	1.797	2.280	1.017	145.25	16.97	29	64	1190	133.9	7.40
Q14964	Ras-related protein Rab-39A OS=Homo sapiens GN=RAB39A PE=2 SV=2 - [RB39A_HUMAN]	11.52	2	1	2	22	1.032	1.128	0.853	1.017	46.55	11.52	3	22	217	25.0	7.65
Q9NYY8	FAST kinase domain-containing protein 2 OS=Homo sapiens GN=FASTKD2 PE=1 SV=1 - [FAKD2_HUMAN]	1.27	1	1	1	2	0.768	0.559	0.831	1.017	3.60	1.27	2	2	710	81.4	8.05

P24821	Tenascin OS=Homo sapiens GN=TNC PE=1 SV=3 - [TENA_HUMAN]	13.77	1	20	20	45	0.322	1.106	1.391	1.017	111.82	13.77	28	45	2201	240.7	4.89
Q13951	Core-binding factor subunit beta OS=Homo sapiens GN=CBFB PE=1 SV=2 - [PEBB_HUMAN]	9.34	1	2	2	9	0.756	1.051	0.656	1.017	24.77	9.34	3	9	182	21.5	6.60
Q8WXX0	Dynein heavy chain 7, axonemal OS=Homo sapiens GN=DNAH7 PE=1 SV=2 - [DYH7_HUMAN]	0.45	1	2	2	4	0.844	0.632	0.503	1.018	8.33	0.45	2	4	4024	460.9	6.00
Q86U28	Iron-sulfur cluster assembly 2 homolog, mitochondrial OS=Homo sapiens GN=ISCA2 PE=1 SV=2 - [ISCA2_HUMAN	7.79	1	1	1	2	0.687	0.949	0.595	1.018	3.05	7.79	2	2	154	16.5	5.25
Q9HAZ2	PR domain zinc finger protein 16 OS=Homo sapiens GN=PRDM16 PE=1 SV=3 - [PRD16_HUMAN]	0.71	1	1	1	4	1.244	1.000	1.412	1.018	8.78	0.71	1	4	1276	140.2	6.21
Q9BRA2	Thioredoxin domain-containing protein 17 OS=Homo sapiens GN=TXNDC17 PE=1 SV=1 - [TXD17_HUMAN]	18.70	1	2	2	41	0.904	1.034	1.215	1.018	157.21	18.70	4	41	123	13.9	5.52
Q8NE71	ATP-binding cassette sub-family F member 1 OS=Homo sapiens GN=ABCF1 PE=1 SV=2 - [ABCF1_HUMAN]	32.19	3	23	26	91	0.924	0.873	0.981	1.018	231.21	32.19	43	91	845	95.9	6.80
P17655	Calpain-2 catalytic subunit OS=Homo sapiens GN=CAPN2 PE=1 SV=6 - [CAN2_HUMAN]	56.29	1	33	33	263	1.184	0.993	1.085	1.018	828.87	56.29	50	263	700	79.9	4.98
Q06481	Amyloid-like protein 2 OS=Homo sapiens GN=APLP2 PE=1 SV=2 - [APLP2_HUMAN]	16.12	1	9	11	28	0.785	0.961	1.244	1.018	70.39	16.12	18	28	763	86.9	4.79

P46019	Phosphorylase b kinase regulatory subunit alpha, liver isoform OS=Homo sapiens GN=PHKA2 PE=1 SV=1 - [KPB2_HUMAN]	2.59	1	2	3	3	1.337	0.825	1.054	1.018	7.65	2.59	3	3	1235	138.3	6.44
Q9BW61	DET1- and DDB1-associated protein 1 OS=Homo sapiens GN=DDA1 PE=1 SV=1 - [DDA1_HUMAN]	28.43	1	4	4	12	1.017	1.145	1.139	1.018	22.91	28.43	7	12	102	11.8	8.68
Q8TEW0	Partitioning defective 3 homolog OS=Homo sapiens GN=PARD3 PE=1 SV=2 - [PARD3_HUMAN]	4.72	1	6	7	13	1.144	1.238	1.301	1.018	29.35	4.72	9	13	1356	151.3	7.68
Q9BXS5	AP-1 complex subunit mu-1 OS=Homo sapiens GN=AP1M1 PE=1 SV=3 - [AP1M1_HUMAN]	44.44	2	15	15	58	1.083	0.799	0.975	1.019	125.83	44.44	21	58	423	48.6	7.30
P46091	G-protein coupled receptor 1 OS=Homo sapiens GN=GPR1 PE=1 SV=2 - [GPR1_HUMAN]	2.82	1	1	1	1	0.673	0.927	1.101	1.019	0.00	2.82	1	1	355	41.4	7.40
O43526	Potassium voltage-gated channel subfamily KQT member 2 OS=Homo sapiens GN=KCNQ2 PE=1 SV=2 - [KCNQ2_HUMAN]	2.64	1	2	2	3	0.912	0.861	0.946	1.019	2.28	2.64	2	3	872	95.8	9.23
Q96SY0	von Willebrand factor A domain-containing protein 9 OS=Homo sapiens GN=VWA9 PE=1 SV=2 - [VWA9_HUMAN	4.83	1	1	1	5	1.318	0.841	0.695	1.019	6.06	4.83	2	5	518	57.4	5.12
Q9UM00	Transmembrane and coiled-coil domain-containing protein 1 OS=Homo sapiens GN=TMCO1 PE=1 SV=1 - [TMCO1_HUMAN]	16.49	1	3	4	6	1.235	0.874	1.037	1.019	10.66	16.49	5	6	188	21.2	9.74

O00483	Cytochrome c oxidase subunit NDUFA4 OS=Homo sapiens GN=NDUFA4 PE=1 SV=1 - [NDUA4_HUMAN]	43.21	1	4	4	26	0.832	0.890	0.732	1.019	67.10	43.21	6	26	81	9.4	9.38
Q96ST2	Protein IWS1 homolog OS=Homo sapiens GN=IWS1 PE=1 SV=2 - [IWS1_HUMAN]	9.04	1	6	7	19	0.981	0.832	0.912	1.019	26.69	9.04	10	19	819	91.9	4.69
P56937	3-keto-steroid reductase OS=Homo sapiens GN=HSD17B7 PE=1 SV=1 - [DHB7_HUMAN]	4.40	1	1	2	2	1.027	0.903	1.034	1.019	1.66	4.40	2	2	341	38.2	8.10
Q9BV20	Methylthioribose-1-phosphate isomerase OS=Homo sapiens GN=MRI1 PE=1 SV=1 - [MTNA_HUMAN]	29.81	1	7	7	17	1.291	0.831	1.244	1.019	37.00	29.81	11	17	369	39.1	6.30
Q96HY6	DDRGK domain-containing protein 1 OS=Homo sapiens GN=DDRGK1 PE=1 SV=2 - [DDRGK_HUMAN]	29.30	1	7	7	41	0.887	0.799	0.807	1.019	119.77	29.30	12	41	314	35.6	5.12
Q9HA65	TBC1 domain family member 17 OS=Homo sapiens GN=TBC1D17 PE=1 SV=2 - [TBC17_HUMAN]	10.65	1	7	7	25	1.052	1.031	0.917	1.019	51.92	10.65	14	25	648	72.7	5.19
Q8WVX3	Uncharacterized protein C4orf3 OS=Homo sapiens GN=C4orf3 PE=1 SV=2 - [CD003_HUMAN	24.24	1	1	1	4	1.212	0.749	0.897	1.019	3.15	24.24	1	4	66	7.6	5.29
Q14696	LDLR chaperone MESD OS=Homo sapiens GN=MESDC2 PE=1 SV=2 - [MESD_HUMAN]	33.76	1	7	7	18	0.981	0.908	0.779	1.020	55.60	33.76	10	18	234	26.1	7.78
Q9Y3F4	Serine-threonine kinase receptor-associated protein OS=Homo sapiens GN=STRAP PE=1 SV=1 - [STRAP_HUMAN]	68.57	1	20	20	81	0.971	0.957	1.113	1.020	239.31	68.57	31	81	350	38.4	5.12

Q9C0D6	FH2 domain-containing protein 1 OS=Homo sapiens GN=FHDC1 PE=1 SV=2 - [FHDC1_HUMAN]	1.05	1	1	2	6	0.804	0.849	1.024	1.020	11.00	1.05	2	6	1143	124.7	9.03
P13164	Interferon-induced transmembrane protein 1 OS=Homo sapiens GN=IFITM1 PE=1 SV=3 - [IFM1_HUMAN]	13.60	3	2	2	12	2.232	0.550	0.509	1.020	48.90	13.60	3	12	125	14.0	7.93
P11413	Glucose-6-phosphate 1-dehydrogenase OS=Homo sapiens GN=G6PD PE=1 SV=4 - [G6PD_HUMAN]	55.53	1	32	32	475	1.434	0.644	0.823	1.020	1255.64	55.53	58	475	515	59.2	6.84
Q8NG68	Tubulin--tyrosine ligase OS=Homo sapiens GN=TTL PE=1 SV=2 - [TTL_HUMAN]	3.98	1	1	1	2	1.201	0.834	0.765	1.020	4.70	3.98	2	2	377	43.2	6.74
Q9UNZ2	NSFL1 cofactor p47 OS=Homo sapiens GN=NSFL1C PE=1 SV=2 - [NSF1C_HUMAN]	48.11	1	16	16	66	1.017	1.123	1.185	1.020	171.69	48.11	26	66	370	40.5	5.10
O95456	Proteasome assembly chaperone 1 OS=Homo sapiens GN=PSMG1 PE=1 SV=1 - [PSMG1_HUMAN]	4.86	1	1	1	5	0.742	0.801	0.775	1.020	12.53	4.86	2	5	288	32.8	7.17
Q5TH69	Brefeldin A-inhibited guanine nucleotide-exchange protein 3 OS=Homo sapiens GN=ARFGEF3 PE=1 SV=3 - [BIG3_HUMAN]	1.98	1	3	3	4	1.320	0.672	0.708	1.020	3.78	1.98	3	4	2177	240.5	5.82
O43665	Regulator of G-protein signaling 10 OS=Homo sapiens GN=RGS10 PE=1 SV=2 - [RGS10_HUMAN]	21.97	1	3	3	5	0.929	0.747	0.560	1.020	10.89	21.97	3	5	173	20.2	5.49
Q9NPR9	Protein GPR108 OS=Homo sapiens GN=GPR108 PE=2 SV=3 - [GP108_HUMAN]	4.60	1	2	2	5	0.522	0.280	0.438	1.020	12.69	4.60	3	5	543	60.6	8.69

Q6NVY1	3-hydroxyisobutyryl-CoA hydrolase, mitochondrial OS=Homo sapiens GN=HIBCH PE=1 SV=2 - [HIBCH_HUMAN]	33.42	1	11	11	49	1.009	1.446	0.749	1.020	103.00	33.42	17	49	386	43.5	8.19
O00748	Cocaine esterase OS=Homo sapiens GN=CES2 PE=1 SV=1 - [EST2_HUMAN]	4.65	1	2	2	10	1.045	1.064	1.367	1.020	25.02	4.65	3	10	559	61.8	6.10
Q8N475	Follistatin-related protein 5 OS=Homo sapiens GN=FSTL5 PE=2 SV=2 - [FSTL5_HUMAN]	2.48	1	1	1	1	0.963	1.141	1.125	1.020	0.00	2.48	1	1	847	95.7	5.95
Q969N2	GPI transamidase component PIG-T OS=Homo sapiens GN=PIGT PE=1 SV=1 - [PIGT_HUMAN]	17.13	1	9	9	25	1.005	0.784	0.899	1.020	73.32	17.13	15	25	578	65.7	8.38
Q86WG5	Myotubularin-related protein 13 OS=Homo sapiens GN=SBF2 PE=1 SV=1 - [MTMRD_HUMAN]	2.27	1	3	3	13	0.849	1.469	1.140	1.020	27.23	2.27	6	13	1849	208.3	7.06
Q495W5	Alpha-(1,3)-fucosyltransferase 11 OS=Homo sapiens GN=FUT11 PE=1 SV=1 - [FUT11_HUMAN]	9.35	1	3	3	4	1.330	1.187	0.682	1.020	11.28	9.35	4	4	492	55.8	5.94
O95166	Gamma-aminobutyric acid receptor-associated protein OS=Homo sapiens GN=GABARAP PE=1 SV=1 - [GBRAP_HUMAN]	24.79	1	2	4	7	0.933	1.246	1.054	1.020	11.82	24.79	5	7	117	13.9	8.79
P17535	Transcription factor jun-D OS=Homo sapiens GN=JUND PE=1 SV=3 - [JUND_HUMAN]	6.34	1	1	2	5	0.724	1.284	0.597	1.021	13.89	6.34	2	5	347	35.2	7.37
P53602	Diphosphomevalonate decarboxylase OS=Homo sapiens GN=MVD PE=1 SV=1 - [MVD1_HUMAN]	21.50	1	7	7	30	1.028	0.667	1.214	1.021	80.55	21.50	13	30	400	43.4	7.23

Q8IUF8	Bifunctional lysine-specific demethylase and histidyl-hydroxylase MINA OS=Homo sapiens GN=MINA PE=1 SV=1 - [MINA_HUMAN]	6.02	1	2	3	4	1.062	0.659	0.581	1.021	10.00	6.02	4	4	465	52.8	6.70
Q9BRK4	Leucine zipper putative tumor suppressor 2 OS=Homo sapiens GN=LZTS2 PE=1 SV=2 - [LZTS2_HUMAN]	2.99	1	2	2	2	1.069	0.986	0.917	1.021	0.00	2.99	2	2	669	72.7	6.51
Q86T82	Ubiquitin carboxyl-terminal hydrolase 37 OS=Homo sapiens GN=USP37 PE=1 SV=2 - [UBP37_HUMAN]	0.92	1	1	1	1	1.136	0.334	0.440	1.021	2.85	0.92	1	1	979	110.1	6.20
Q155Q3	Dixin OS=Homo sapiens GN=DIXDC1 PE=1 SV=2 - [DIXC1_HUMAN]	1.90	3	1	2	6	1.036	0.723	0.830	1.021	12.90	1.90	2	6	683	77.4	6.24
Q9HCH5	Synaptotagmin-like protein 2 OS=Homo sapiens GN=SYTL2 PE=1 SV=3 - [SYTL2_HUMAN]	3.53	1	3	3	5	0.595	1.521	1.680	1.021	10.66	3.53	4	5	934	104.9	8.00
O95476	CTD nuclear envelope phosphatase 1 OS=Homo sapiens GN=CTDNEP1 PE=1 SV=2 - [CNEP1_HUMAN]	11.48	1	3	3	5	0.976	0.938	0.736	1.022	8.67	11.48	4	5	244	28.4	9.76
Q9Y6D5	Brefeldin A-inhibited guanine nucleotide-exchange protein 2 OS=Homo sapiens GN=ARFGEF2 PE=1 SV=3 - [BIG2_HUMAN]	10.03	1	13	17	32	1.042	0.861	0.824	1.022	66.41	10.03	23	32	1785	201.9	6.33
Q7Z392	Trafficking protein particle complex subunit 11 OS=Homo sapiens GN=TRAPPC11 PE=1 SV=2 - [TPC11_HUMAN]	6.53	1	6	6	14	1.071	0.805	0.926	1.022	38.19	6.53	8	14	1133	128.8	7.14

Q8WU76	Sec1 family domain-containing protein 2 OS=Homo sapiens GN=SCFD2 PE=1 SV=2 - [SCFD2_HUMAN]	7.16	1	4	4	17	0.832	0.929	1.083	1.022	46.34	7.16	7	17	684	75.1	6.68
Q9UK76	Hematological and neurological expressed 1 protein OS=Homo sapiens GN=HN1 PE=1 SV=3 - [HN1_HUMAN]	37.66	1	5	5	46	0.820	1.129	0.913	1.022	114.13	37.66	9	46	154	16.0	5.60
P50336	Protoporphyrinogen oxidase OS=Homo sapiens GN=PPOX PE=1 SV=1 - [PPOX_HUMAN]	11.11	1	4	4	11	1.403	1.576	1.212	1.022	29.43	11.11	7	11	477	50.7	8.16
O95751	Protein LDOC1 OS=Homo sapiens GN=LDOC1 PE=1 SV=1 - [LDOC1_HUMAN]	12.33	1	1	1	1	1.076	1.105	1.048	1.022	0.00	12.33	1	1	146	17.0	4.25
Q9Y210	Short transient receptor potential channel 6 OS=Homo sapiens GN=TRPC6 PE=1 SV=1 - [TRPC6_HUMAN	2.36	1	2	2	4	0.395	0.811	0.628	1.022	13.57	2.36	2	4	931	106.3	6.67
Q13043	Serine/threonine-protein kinase 4 OS=Homo sapiens GN=STK4 PE=1 SV=2 - [STK4_HUMAN]	20.74	1	7	8	15	0.981	1.088	1.011	1.022	35.59	20.74	10	15	487	55.6	5.07
Q9NP81	Serine--tRNA ligase, mitochondrial OS=Homo sapiens GN=SARS2 PE=1 SV=1 - [SYSM_HUMAN]	15.25	1	8	8	28	0.929	1.345	1.083	1.023	63.80	15.25	15	28	518	58.2	8.13
Q96P48	Arf-GAP with Rho-GAP domain, ANK repeat and PH domain-containing protein 1 OS=Homo sapiens GN=ARAP1 PE=1 SV=3 - [ARAP1_HUMAN]	3.79	3	6	7	13	1.085	1.310	1.074	1.023	24.69	3.79	10	13	1450	162.1	6.23

Q969V5	Mitochondrial ubiquitin ligase activator of NFKB 1 OS=Homo sapiens GN=MUL1 PE=1 SV=1 - [MUL1_HUMAN]	9.66	1	4	4	7	1.145	0.865	0.964	1.023	19.15	9.66	6	7	352	39.8	8.13
Q8N129	Protein canopy homolog 4 OS=Homo sapiens GN=CNPY4 PE=2 SV=1 - [CNPY4_HUMAN]	22.98	1	5	5	19	1.101	0.938	1.150	1.023	53.10	22.98	10	19	248	28.3	4.64
Q8N335	Glycerol-3-phosphate dehydrogenase 1-like protein OS=Homo sapiens GN=GPD1L PE=1 SV=1 - [GPD1L_HUMAN]	27.92	2	11	11	34	1.000	1.207	1.013	1.023	71.10	27.92	19	34	351	38.4	7.02
A6NHQ2	rRNA/tRNA 2'-O-methyltransferase fibrillarin-like protein 1 OS=Homo sapiens GN=FBLL1 PE=3 SV=1 - [FBLL1_HUMAN]	13.51	1	2	3	7	1.317	1.079	0.870	1.023	17.13	13.51	4	7	333	34.7	10.33
Q96T58	Msx2-interacting protein OS=Homo sapiens GN=SPEN PE=1 SV=1 - [MINT_HUMAN]	1.47	1	2	4	10	0.742	0.847	0.982	1.023	7.37	1.47	4	10	3664	402.0	7.64
Q96PD2	Discoidin, CUB and LCCL domain-containing protein 2 OS=Homo sapiens GN=DCBLD2 PE=1 SV=1 - [DCBD2_HUMAN]	9.03	1	4	5	8	0.602	0.955	1.019	1.023	16.48	9.03	6	8	775	85.0	7.17
Q8N1F1	Putative uncharacterized protein LRRC75A-AS1, mitochondrial OS=Homo sapiens GN=LRRC75A-AS1 PE=5 SV=1 - [C1AS1_HUMAN]	12.31	1	1	1	1	1.015	2.286	2.399	1.023	2.33	12.31	1	1	130	13.8	9.26
Q92575	UBX domain-containing protein 4 OS=Homo sapiens GN=UBXN4 PE=1 SV=2 - [UBXN4_HUMAN]	17.52	1	9	9	40	0.826	1.085	0.809	1.023	87.46	17.52	14	40	508	56.7	6.38

O95819	Mitogen-activated protein kinase kinase kinase kinase 4 OS=Homo sapiens GN=MAP4K4 PE=1 SV=2 - [M4K4_HUMAN]	16.71	2	10	17	66	0.907	1.218	1.106	1.023	144.46	16.71	29	66	1239	142.0	7.46
Q7Z5L7	Podocan OS=Homo sapiens GN=PODN PE=1 SV=2 - [PODN_HUMAN]	9.46	1	5	6	14	1.688	0.854	0.723	1.023	28.18	9.46	9	14	613	68.9	6.99
Q5TAQ9	DDB1- and CUL4-associated factor 8 OS=Homo sapiens GN=DCAF8 PE=1 SV=1 - [DCAF8_HUMAN	12.06	1	6	6	18	0.631	1.183	0.955	1.023	54.90	12.06	10	18	597	66.8	5.39
Q86WI1	Fibrocystin-L OS=Homo sapiens GN=PKHD1L1 PE=2 SV=2 - [PKHL1_HUMAN]	0.73	1	1	1	1	0.949	0.869	1.489	1.023	2.62	0.73	1	1	4243	465.4	6.11
Q9BZL4	Protein phosphatase 1 regulatory subunit 12C OS=Homo sapiens GN=PPP1R12C PE=1 SV=1 - [PP12C_HUMAN]	37.98	1	25	25	89	1.160	1.229	1.386	1.023	194.71	37.98	43	89	782	84.8	5.57
Q96T88	E3 ubiquitin-protein ligase UHRF1 OS=Homo sapiens GN=UHRF1 PE=1 SV=1 - [UHRF1_HUMAN]	1.77	1	1	1	1	1.437	0.570	0.983	1.023	2.03	1.77	1	1	793	89.8	7.56
Q9UM47	Neurogenic locus notch homolog protein 3 OS=Homo sapiens GN=NOTCH3 PE=1 SV=2 - [NOTC3_HUMA N]	1.46	1	1	4	10	0.953	0.954	1.012	1.024	31.40	1.46	6	10	2321	243.5	5.39
Q9Y5S2	Serine/threonine-protein kinase MRCK beta OS=Homo sapiens GN=CDC42BPB PE=1 SV=2 - [MRCKB_HUMA	22.97	1	33	40	100	1.076	1.121	1.151	1.024	242.26	22.97	62	100	1711	194.2	6.37
P0DI81	Trafficking protein particle complex subunit 2 OS=Homo sapiens GN=TRAPPC2 PE=1 SV=1 - [TPC2A_HUMAN]	15.00	1	2	2	7	1.412	1.072	1.022	1.024	12.29	15.00	3	7	140	16.4	6.52

Q9NZJ4	Sacsin OS=Homo sapiens GN=SACS PE=1 SV=2 - [SACS_HUMAN]	2.77	1	11	11	23	0.876	1.413	1.368	1.024	51.19	2.77	16	23	4579	520.8	7.05
P31513	Dimethylaniline monooxygenase [N-oxide-forming] 3 OS=Homo sapiens GN=FMO3 PE=1 SV=5 - [FMO3_HUMAN]	3.01	1	2	2	4	0.932	1.028	14.660	1.024	6.22	3.01	4	4	532	60.0	7.78
Q9H4B4	Serine/threonine-protein kinase PLK3 OS=Homo sapiens GN=PLK3 PE=1 SV=2 - [PLK3_HUMAN]	1.70	1	1	1	2	0.753	0.756	0.849	1.024	0.00	1.70	1	2	646	71.6	9.16
P19838	Nudear factor NF-kappa-B p105 subunit OS=Homo sapiens GN=NFKB1 PE=1 SV=2 - [NFKB1_HUMAN]	18.90	1	17	17	49	0.891	0.988	1.104	1.024	135.20	18.90	27	49	968	105.3	5.40
Q8TDB6	E3 ubiquitin-protein ligase DTX3L OS=Homo sapiens GN=DTX3L PE=1 SV=1 - [DTX3L_HUMAN]	17.30	1	12	12	36	1.826	1.344	1.229	1.025	72.92	17.30	19	36	740	83.5	8.06
Q9BQ39	ATP-dependent RNA helicase DDX50 OS=Homo sapiens GN=DDX50 PE=1 SV=1 - [DDX50_HUMAN	8.41	1	3	6	15	0.888	1.274	1.039	1.025	40.07	8.41	9	15	737	82.5	9.17
P35612	Beta-adducin OS=Homo sapiens GN=ADD2 PE=1 SV=3 - [ADDB_HUMAN]	3.72	1	2	3	6	0.405	11.879	3.441	1.025	16.17	3.72	6	6	726	80.8	5.92
Q86TI2	Dipeptidyl peptidase 9 OS=Homo sapiens GN=DPP9 PE=1 SV=3 - [DPP9_HUMAN]	12.63	1	9	10	27	0.936	0.893	0.851	1.025	70.43	12.63	15	27	863	98.2	6.46
O75962	Triple functional domain protein OS=Homo sapiens GN=TRIO PE=1 SV=2 - [TRIO_HUMAN]	13.72	2	35	36	105	1.089	0.756	1.065	1.025	272.70	13.72	52	105	3097	346.7	6.37

Q15370	Transcription elongation factor B polypeptide 2 OS=Homo sapiens GN=TCEB2 PE=1 SV=1 - [ELOB_HUMAN]	24.58	1	4	4	19	1.005	1.008	1.135	1.025	41.35	24.58	5	19	118	13.1	4.88
Q15052	Rho guanine nucleotide exchange factor 6 OS=Homo sapiens GN=ARHGEF6 PE=1 SV=2 - [ARHG6_HUMAN	12.11	1	7	8	18	0.726	0.603	0.775	1.025	44.96	12.11	10	18	776	87.4	6.05
Q9NY35	Claudin domain-containing protein 1 OS=Homo sapiens GN=CLDND1 PE=1 SV=1 - [CLDN1_HUMAN]	3.56	1	1	1	6	1.332	0.733	0.850	1.025	12.63	3.56	2	6	253	28.6	5.82
Q96CS3	FAS-associated factor 2 OS=Homo sapiens GN=FAF2 PE=1 SV=2 - [FAF2_HUMAN]	37.08	1	12	12	65	0.926	0.947	0.888	1.025	168.90	37.08	21	65	445	52.6	5.62
Q7Z7H5	Transmembrane emp24 domain-containing protein 4 OS=Homo sapiens GN=TMED4 PE=1 SV=1 - [TMED4_HUMA	31.28	1	6	8	22	1.077	1.119	0.894	1.025	59.58	31.28	11	22	227	25.9	8.28
Q7Z4V5	Hepatoma-derived growth factor-related protein 2 OS=Homo sapiens GN=HDGFRP2 PE=1 SV=1 - [HDGR2_HUMAN]	13.26	1	7	8	25	1.145	1.367	0.956	1.026	60.85	13.26	12	25	671	74.3	7.49
O60256	Phosphoribosyl pyrophosphate synthase-associated protein 2 OS=Homo sapiens GN=PRPSAP2 PE=1 SV=1 - [KPRB_HUMAN]	29.00	1	5	9	23	1.073	0.772	0.874	1.026	64.15	29.00	14	23	369	40.9	7.44
P60866	40S ribosomal protein S20 OS=Homo sapiens GN=RPS20 PE=1 SV=1 - [RS20_HUMAN]	41.18	1	6	7	78	0.922	0.971	1.018	1.026	163.50	41.18	12	78	119	13.4	9.94

Q96HY7	Probable 2-oxoglutarate dehydrogenase E1 component DHKTD1, mitochondrial OS=Homo sapiens GN=DHTKD1 PE=1 SV=2 - [DHTK1_HUMAN]	1.74	1	2	2	3	1.357	1.366	0.680	1.026	5.93	1.74	3	3	919	103.0	6.93
Q8NBU5	ATPase family AAA domain-containing protein 1 OS=Homo sapiens GN=ATAD1 PE=1 SV=1 - [ATAD1_HUMAN]	21.88	1	7	7	20	1.014	0.800	0.767	1.026	51.90	21.88	9	20	361	40.7	6.90
P50570	Dynamin-2 OS=Homo sapiens GN=DNM2 PE=1 SV=2 - [DYN2_HUMAN]	25.29	1	10	26	83	1.291	1.097	0.922	1.026	186.98	25.29	38	83	870	98.0	7.44
Q86TV6	Tetratricopeptide repeat protein 7B OS=Homo sapiens GN=TTC7B PE=1 SV=3 - [TTC7B_HUMAN	8.07	1	4	4	6	1.347	0.943	0.743	1.026	8.91	8.07	6	6	843	94.1	6.89
O94763	Unconventional prefoldin RPB5 interactor 1 OS=Homo sapiens GN=URI1 PE=1 SV=3 - [RMP_HUMAN]	8.41	2	4	6	8	0.794	1.262	0.952	1.026	15.15	8.41	7	8	535	59.8	5.05
Q8TEA7	TBC domain-containing protein kinase-like protein OS=Homo sapiens GN=TBCK PE=1 SV=4 - [TBCK_HUMAN]	4.82	1	4	4	7	0.862	1.141	1.198	1.026	12.30	4.82	6	7	893	100.6	6.58
Q9H2M3	S-methylmethionine--homocysteine S-methyltransferase BHMT2 OS=Homo sapiens GN=BHMT2 PE=1 SV=1 - [BHMT2_HUMAN]	2.75	2	1	1	2	0.511	0.444	0.805	1.026	4.99	2.75	1	2	363	40.3	5.87
Q9UBK8	Methionine synthase reductase OS=Homo sapiens GN=MTRR PE=1 SV=3 - [MTRR_HUMAN]	2.48	1	1	1	4	0.712	0.882	2.203	1.026	11.26	2.48	2	4	725	80.4	6.49

O75691	Small subunit processome component 20 homolog OS=Homo sapiens GN=UTP20 PE=1 SV=3 - [UTP20_HUMAN]	1.72	1	4	4	5	0.958	1.013	0.796	1.026	15.37	1.72	5	5	2785	318.2	7.39
O00154	Cytosolic acyl coenzyme A thioester hydrolase OS=Homo sapiens GN=ACOT7 PE=1 SV=3 - [BACH_HUMAN]	29.47	1	9	9	64	1.267	1.059	2.245	1.027	142.18	29.47	16	64	380	41.8	8.54
Q5THR3	EF-hand calcium-binding domain-containing protein 6 OS=Homo sapiens GN=EFCAB6 PE=1 SV=1 - [EFCB6_HUMAN]	0.87	1	1	1	1	0.896	0.870	1.061	1.027	2.23	0.87	1	1	1501	172.8	8.40
P46783	40S ribosomal protein S10 OS=Homo sapiens GN=RPS10 PE=1 SV=1 - [RS10_HUMAN]	51.52	2	13	14	105	1.052	1.109	1.003	1.027	235.88	51.52	23	105	165	18.9	10.15
Q15878	Voltage-dependent R-type calcium channel subunit alpha-1E OS=Homo sapiens GN=CACNA1E PE=1 SV=3 - [CAC1E_HUMAN]	0.56	1	1	1	1	0.877	1.026	1.569	1.027	1.85	0.56	1	1	2313	261.6	8.24
P21283	V-type proton ATPase subunit C 1 OS=Homo sapiens GN=ATP6V1C1 PE=1 SV=4 - [VATC1_HUMAN]	31.68	1	14	14	61	1.035	0.983	1.034	1.027	113.74	31.68	22	61	382	43.9	7.46
P26641	Elongation factor 1-gamma OS=Homo sapiens GN=EEF1G PE=1 SV=3 - [EF1G_HUMAN]	32.04	1	18	18	235	1.024	1.034	1.098	1.027	459.89	32.04	27	235	437	50.1	6.67
Q9P2D3	HEAT repeat-containing protein 5B OS=Homo sapiens GN=HEATR5B PE=1 SV=2 - [HTR5B_HUMAN]	1.55	1	2	3	17	1.404	0.964	0.876	1.027	35.37	1.55	4	17	2071	224.2	7.17

Q96AX1	Vacuolar protein sorting-associated protein 33A OS=Homo sapiens GN=VPS33A PE=1 SV=1 - [VP33A_HUMAN]	13.09	1	7	7	16	0.990	1.164	1.226	1.027	36.05	13.09	11	16	596	67.6	6.96
Q96Q45	Transmembrane protein 237 OS=Homo sapiens GN=TMEM237 PE=1 SV=2 - [TM237_HUMAN]	14.22	1	5	5	13	1.253	0.579	0.832	1.027	26.06	14.22	7	13	408	45.5	6.47
P10176	Cytochrome c oxidase subunit 8A, mitochondrial OS=Homo sapiens GN=COX8A PE=1 SV=2 - [COX8A_HUMAN]	13.04	1	1	1	11	0.898	0.879	0.960	1.027	20.33	13.04	2	11	69	7.6	10.24
P78367	Homeobox protein Nkx-3.2 OS=Homo sapiens GN=NKX3-2 PE=2 SV=2 - [NKX32_HUMAN]	11.71	1	2	2	3	1.819	0.926	1.199	1.027	7.64	11.71	2	3	333	34.8	7.93
Q13098	COP9 signalosome complex subunit 1 OS=Homo sapiens GN=GPS1 PE=1 SV=4 - [CSN1_HUMAN]	23.83	1	14	14	53	0.837	0.933	0.977	1.028	139.68	23.83	19	53	491	55.5	6.74
P27448	MAP/microtubule affinity-regulating kinase 3 OS=Homo sapiens GN=MARK3 PE=1 SV=4 - [MARK3_HUMAN]	11.42	2	6	8	16	1.299	0.994	1.039	1.028	40.14	11.42	11	16	753	84.4	9.57
P53990	IST1 homolog OS=Homo sapiens GN=IST1 PE=1 SV=1 - [IST1_HUMAN]	25.82	1	11	11	39	1.202	0.986	0.950	1.028	100.17	25.82	17	39	364	39.7	5.35
Q07890	Son of sevenless homolog 2 OS=Homo sapiens GN=SOS2 PE=1 SV=2 - [SOS2_HUMAN]	2.55	1	2	3	5	0.890	0.720	0.760	1.028	9.44	2.55	4	5	1332	152.9	6.84

P49419	Alpha-aminoadipic semialdehyde dehydrogenase OS=Homo sapiens GN=ALDH7A1 PE=1 SV=5 - [AL7A1_HUMAN]	39.52	1	19	19	114	1.112	1.104	0.891	1.028	307.86	39.52	33	114	539	58.5	7.99
P51116	Fragile X mental retardation syndrome-related protein 2 OS=Homo sapiens GN=FXR2 PE=1 SV=2 - [FXR2_HUMAN]	17.09	1	5	8	24	1.031	1.043	1.099	1.028	84.11	17.09	11	24	673	74.2	6.23
O95573	Long-chain-fatty-acid--CoA ligase 3 OS=Homo sapiens GN=ACSL3 PE=1 SV=3 - [ACSL3_HUMAN]	32.08	1	15	19	72	0.972	0.702	0.716	1.028	168.22	32.08	30	72	720	80.4	8.38
Q9H6B4	CXADR-like membrane protein OS=Homo sapiens GN=CLMP PE=1 SV=1 - [CLMP_HUMAN]	15.55	1	4	4	13	1.330	0.896	0.957	1.028	31.44	15.55	6	13	373	41.3	7.99
P62888	60S ribosomal protein L30 OS=Homo sapiens GN=RPL30 PE=1 SV=2 - [RL30_HUMAN]	55.65	1	7	8	48	0.921	1.068	1.004	1.028	107.68	55.65	13	48	115	12.8	9.63
O94986	Centrosomal protein of 152 kDa OS=Homo sapiens GN=CEP152 PE=1 SV=4 - [CE152_HUMAN]	3.80	1	5	5	7	0.656	0.878	0.916	1.028	14.26	3.80	5	7	1710	195.5	5.60
Q86YT6	E3 ubiquitin-protein ligase MIB1 OS=Homo sapiens GN=MIB1 PE=1 SV=1 - [MIB1_HUMAN]	5.17	1	5	5	7	0.694	1.007	1.048	1.028	18.03	5.17	5	7	1006	110.1	6.92
Q96CC6	Inactive rhomboid protein 1 OS=Homo sapiens GN=RHBDF1 PE=1 SV=2 - [RHDF1_HUMAN]	7.72	1	7	7	13	1.535	0.989	0.994	1.028	25.78	7.72	10	13	855	97.3	8.44
P51687	Sulfite oxidase, mitochondrial OS=Homo sapiens GN=SUOX PE=1 SV=2 - [SUOX_HUMAN]	5.50	1	3	3	5	1.289	0.977	1.200	1.028	12.63	5.50	4	5	545	60.2	6.11

Q9P0W5	Schwannomin-interacting protein 1 OS=Homo sapiens GN=SCHIP1 PE=1 SV=1 - [SCHI1_HUMAN]	2.26	1	1	1	1	0.858	1.033	1.056	1.028	0.00	2.26	1	1	487	53.4	5.12
Q9BZQ4	Nicotinamide mononucleotide adenylyltransferase 2 OS=Homo sapiens GN=NMNAT2 PE=1 SV=1 - [NMNA2_HUMA	4.56	1	1	1	1	0.929	1.036	1.290	1.028	3.25	4.56	1	1	307	34.4	7.06
Q14678	KN motif and ankyrin repeat domain-containing protein 1 OS=Homo sapiens GN=KANK1 PE=1 SV=3 - [KANK1_HUMAN]	3.55	1	2	6	15	0.706	1.041	1.390	1.029	38.72	3.55	8	15	1352	147.2	5.30
Q8IY26	Presqualene diphosphate phosphatase OS=Homo sapiens GN=PPAPDC2 PE=1 SV=3 - [PPAC2_HUMAN]	3.39	1	1	1	4	1.392	1.037	0.939	1.029	6.44	3.39	2	4	295	32.2	9.99
O43149	Zinc finger ZZ-type and EF-hand domain-containing protein 1 OS=Homo sapiens GN=ZZEF1 PE=1 SV=6 - [ZZEF1_HUMAN]	2.67	1	5	7	11	1.275	0.912	1.181	1.029	15.91	2.67	8	11	2961	330.9	5.95
P26368	Splicing factor U2AF 65 kDa subunit OS=Homo sapiens GN=U2AF2 PE=1 SV=4 - [U2AF2_HUMAN]	27.16	1	9	9	24	0.991	1.026	0.909	1.029	57.68	27.16	15	24	475	53.5	9.09
O95793	Double-stranded RNA-binding protein Staufen homolog 1 OS=Homo sapiens GN=STAU1 PE=1 SV=2 - [STAU1_HUMAN]	30.16	1	13	14	49	1.056	1.093	1.140	1.029	106.04	30.16	22	49	577	63.1	9.44
P82912	28S ribosomal protein S11, mitochondrial OS=Homo sapiens GN=MRPS11 PE=1 SV=2 - [RT11_HUMAN]	5.67	1	1	1	6	1.058	1.516	0.889	1.029	12.61	5.67	2	6	194	20.6	10.81

Q9H190	Syntenin-2 OS=Homo sapiens GN=SDCBP2 PE=2 SV=2 - [SDCB2_HUMAN]	2.05	1	1	1	2	0.789	1.027	0.725	1.029	4.64	2.05	1	2	292	31.6	9.07
P46100	Transcriptional regulator ATRX OS=Homo sapiens GN=ATRX PE=1 SV=5 - [ATRX_HUMAN]	2.09	1	2	3	4	1.399	1.871	1.133	1.029	8.15	2.09	4	4	2492	282.4	6.58
Q96N64	PWWP domain-containing protein 2A OS=Homo sapiens GN=PWWP2A PE=1 SV=2 - [PWP2A_HUMAN]	1.85	1	1	1	2	0.721	0.993	1.267	1.029	2.05	1.85	1	2	755	81.9	9.00
B2RXF5	Zinc finger and BTB domain-containing protein 42 OS=Homo sapiens GN=ZBTB42 PE=1 SV=2 - [ZBT42_HUMAN]	1.90	1	1	1	5	1.058	1.083	1.039	1.029	6.74	1.90	2	5	422	46.5	8.47
P61106	Ras-related protein Rab-14 OS=Homo sapiens GN=RAB14 PE=1 SV=4 - [RAB14_HUMAN]	74.88	2	12	13	101	1.054	0.762	0.848	1.029	273.71	74.88	21	101	215	23.9	6.21
P00403	Cytochrome c oxidase subunit 2 OS=Homo sapiens GN=MT-CO2 PE=1 SV=1 - [COX2_HUMAN]	11.89	1	3	3	43	0.827	0.982	0.706	1.030	90.31	11.89	5	43	227	25.5	4.82
P37275	Zinc finger E-box-binding homeobox 1 OS=Homo sapiens GN=ZEB1 PE=1 SV=2 - [ZEB1_HUMAN]	4.89	1	4	4	9	1.307	1.572	1.384	1.030	14.88	4.89	6	9	1124	124.0	4.94
P82094	TATA element modulatory factor OS=Homo sapiens GN=TMF1 PE=1 SV=2 - [TMF1_HUMAN]	22.05	1	18	20	40	0.973	0.943	1.043	1.030	79.91	22.05	28	40	1093	122.8	4.92
P62857	40S ribosomal protein S28 OS=Homo sapiens GN=RPS28 PE=1 SV=1 - [RS28_HUMAN]	52.17	1	4	4	158	0.788	1.016	0.978	1.030	405.22	52.17	6	158	69	7.8	10.70

Q8N8S7	Protein enabled homolog OS=Homo sapiens GN=ENAH PE=1 SV=2 - [ENAH_HUMAN]	36.21	1	18	18	109	0.735	0.546	0.627	1.030	225.85	36.21	28	109	591	66.5	6.93
Q96GS4	Uncharacterized protein C17orf59 OS=Homo sapiens GN=C17orf59 PE=1 SV=2 - [CQ059_HUMAN]	16.53	1	5	5	8	1.043	0.996	1.220	1.030	13.24	16.53	7	8	357	37.2	5.76
P41240	Tyrosine-protein kinase CSK OS=Homo sapiens GN=CSK PE=1 SV=1 - [CSK_HUMAN]	24.89	1	11	11	35	1.012	0.979	1.084	1.030	64.75	24.89	18	35	450	50.7	7.06
P05141	ADP/ATP translocase 2 OS=Homo sapiens GN=SLC25A5 PE=1 SV=7 - [ADT2_HUMAN]	48.32	2	6	18	107	1.289	1.188	0.821	1.030	242.16	48.32	30	107	298	32.8	9.69
P82909	28S ribosomal protein S36, mitochondrial OS=Homo sapiens GN=MRPS36 PE=1 SV=2 - [RT36_HUMAN]	57.28	1	5	5	20	1.621	1.871	1.730	1.030	59.08	57.28	7	20	103	11.5	9.99
O15040	Tectonin beta-propeller repeat-containing protein 2 OS=Homo sapiens GN=TECPR2 PE=1 SV=4 - [TCPR2_HUMAN	2.41	1	3	3	6	0.721	1.229	1.207	1.030	14.54	2.41	3	6	1411	153.8	5.55
Q9BRU9	rRNA-processing protein UTP23 homolog OS=Homo sapiens GN=UTP23 PE=1 SV=2 - [UTP23_HUMAN]	11.24	1	3	3	5	0.959	1.058	0.989	1.031	11.98	11.24	3	5	249	28.4	10.07
P55957	BH3-interacting domain death agonist OS=Homo sapiens GN=BID PE=1 SV=1 - [BID_HUMAN]	31.79	1	4	4	15	1.005	0.556	1.191	1.031	45.70	31.79	6	15	195	22.0	5.44
P54098	DNA polymerase subunit gamma-1 OS=Homo sapiens GN=POLG PE=1 SV=1 - [DPOG1_HUMAN]	2.10	1	2	2	5	0.914	1.281	1.133	1.031	9.74	2.10	3	5	1239	139.5	6.90

Q9Y5H9	Protocadherin alpha-2 OS=Homo sapiens GN=PCDHA2 PE=1 SV=1 - [PCDA2_HUMAN]	1.16	1	1	1	1	1.006	0.804	1.189	1.031	2.14	1.16	1	1	948	102.0	5.20
Q96JG6	Coiled-coil domain-containing protein 132 OS=Homo sapiens GN=CCDC132 PE=1 SV=3 - [CC132_HUMAN]	8.20	1	7	7	13	1.061	1.281	1.022	1.031	30.57	8.20	10	13	964	111.1	6.20
Q8WV41	Sorting nexin-33 OS=Homo sapiens GN=SNX33 PE=1 SV=1 - [SNX33_HUMAN]	11.85	1	5	6	13	1.005	1.014	0.851	1.031	36.83	11.85	9	13	574	65.2	6.79
Q3KQU3	MAP7 domain-containing protein 1 OS=Homo sapiens GN=MAP7D1 PE=1 SV=1 - [MA7D1_HUMAN]	26.28	2	18	20	82	0.887	1.236	1.413	1.031	195.84	26.28	28	82	841	92.8	10.11
Q14432	cGMP-inhibited 3',5'-cyclic phosphodiesterase A OS=Homo sapiens GN=PDE3A PE=1 SV=3 - [PDE3A_HUMAN]	3.07	1	3	3	6	0.388	0.940	1.948	1.031	10.05	3.07	3	6	1141	124.9	6.00
P57076	UPF0769 protein C21orf59 OS=Homo sapiens GN=C21orf59 PE=1 SV=1 - [CU059_HUMAN]	2.41	1	1	1	2	0.719	0.826	0.749	1.031	4.72	2.41	1	2	290	33.2	7.44
Q9P2B2	Prostaglandin F2 receptor negative regulator OS=Homo sapiens GN=PTGFRN PE=1 SV=2 - [FPRP_HUMAN]	18.66	1	16	16	49	0.905	0.558	0.544	1.031	93.70	18.66	26	49	879	98.5	6.61
Q9P2K8	Eukaryotic translation initiation factor 2-alpha kinase 4 OS=Homo sapiens GN=EIF2AK4 PE=1 SV=3 - [E2AK4_HUMAN]	11.16	1	13	14	36	0.835	0.812	0.804	1.031	72.02	11.16	19	36	1649	186.8	6.28
P32969	60S ribosomal protein L9 OS=Homo sapiens GN=RPL9 PE=1 SV=1 - [RL9_HUMAN]	43.75	1	10	10	56	0.865	0.942	0.858	1.031	110.11	43.75	15	56	192	21.8	9.95

Q9NWV8	BRISC and BRCA1-A complex member 1 OS=Homo sapiens GN=BABAM1 PE=1 SV=1 - [BABA1_HUMAN]	25.53	1	5	6	21	0.951	1.008	1.230	1.032	59.21	25.53	7	21	329	36.5	4.64
Q9UL18	Protein argonaute-1 OS=Homo sapiens GN=AGO1 PE=1 SV=3 - [AGO1_HUMAN]	11.32	2	4	9	19	1.389	1.258	0.896	1.032	32.04	11.32	11	19	857	97.2	9.16
Q8IZL8	Proline-, glutamic acid- and leucine-rich protein 1 OS=Homo sapiens GN=PELP1 PE=1 SV=2 - [PELP1_HUMAN]	7.43	1	7	7	23	0.919	0.950	0.869	1.032	49.48	7.43	14	23	1130	119.6	4.34
Q13542	Eukaryotic translation initiation factor 4E-binding protein 2 OS=Homo sapiens GN=EIF4EBP2 PE=1 SV=1 - [4EBP2_HUMAN]	10.83	1	1	1	2	0.613	0.946	0.618	1.032	4.68	10.83	2	2	120	12.9	6.67
Q00765	Receptor expression-enhancing protein 5 OS=Homo sapiens GN=REEP5 PE=1 SV=3 - [REEP5_HUMAN]	21.69	1	7	7	52	1.420	0.844	0.772	1.032	118.81	21.69	11	52	189	21.5	8.10
Q9Y6Q2	Stonin-1 OS=Homo sapiens GN=STON1 PE=1 SV=2 - [STON1_HUMAN]	2.18	1	2	2	2	0.946	1.316	1.240	1.032	4.22	2.18	2	2	735	83.1	6.20
Q9NUJ1	Mycophenolic acid acyl-glucuronide esterase, mitochondrial OS=Homo sapiens GN=ABHD10 PE=1 SV=1 - [ABHDA_HUMAN]	21.90	1	6	6	13	0.933	1.286	0.847	1.032	34.48	21.90	10	13	306	33.9	8.57
Q13459	Unconventional myosin-IXb OS=Homo sapiens GN=MYO9B PE=1 SV=3 - [MYO9B_HUMAN]	14.09	1	26	28	52	0.703	0.948	1.205	1.032	115.94	14.09	38	52	2157	243.2	8.75

Q15149	Plectin OS=Homo sapiens GN=PLEC PE=1 SV=3 - [PLEC_HUMAN]	60.35	3	290	305	2695	1.359	1.214	1.095	1.032	6623.17	60.35	530	2695	4684	531.5	5.96
Q8NC06	Acyl-CoA-binding domain-containing protein 4 OS=Homo sapiens GN=ACBD4 PE=1 SV=2 - [ACBD4_HUMAN]	4.85	1	1	1	1		1.813	1.475	1.032	2.73	4.85	1	1	268	30.3	7.12
Q9Y2X0	Mediator of RNA polymerase II transcription subunit 16 OS=Homo sapiens GN=MED16 PE=1 SV=2 - [MED16_HUMAN]	0.91	1	1	1	1	1.005	1.052	0.869	1.032	0.00	0.91	1	1	877	96.7	7.37
Q8TD16	Protein bicaudal D homolog 2 OS=Homo sapiens GN=BICD2 PE=1 SV=1 - [BICD2_HUMAN	18.33	1	13	14	37	0.745	0.872	1.069	1.033	124.83	18.33	18	37	824	93.5	5.44
Q9H0P0	Cytosolic 5'-nucleotidase 3A OS=Homo sapiens GN=NT5C3A PE=1 SV=3 - [5NT3A_HUMAN]	16.96	2	5	6	14	1.003	0.776	0.692	1.033	33.96	16.96	8	14	336	37.9	7.12
Q86UE4	Protein LYRIC OS=Homo sapiens GN=MTDH PE=1 SV=2 - [LYRIC_HUMAN]	49.83	1	27	27	150	1.017	0.870	0.962	1.033	368.79	49.83	43	150	582	63.8	9.32
Q15070	Mitochondrial inner membrane protein OXA1L OS=Homo sapiens GN=OXA1L PE=1 SV=3 - [OXA1L_HUMAN	14.02	1	6	6	17	0.970	1.111	0.994	1.033	28.24	14.02	11	17	435	48.5	9.45
Q9UKA9	Polypyrimidine tract-binding protein 2 OS=Homo sapiens GN=PTBP2 PE=1 SV=1 - [PTBP2_HUMAN]	1.88	1	1	1	1	1.441	1.275	0.929	1.033	1.83	1.88	1	1	531	57.5	8.66
Q96B70	Leukocyte receptor cluster member 9 OS=Homo sapiens GN=LENG9 PE=2 SV=2 - [LENG9_HUMAN]	4.39	1	2	2	3	0.919	1.160	1.273	1.033	7.89	4.39	3	3	501	53.1	7.84

O60503	Adenylate cyclase type 9 OS=Homo sapiens GN=ADCY9 PE=1 SV=4 - [ADCY9_HUMAN]	7.91	1	11	11	21	1.214	0.585	0.775	1.033	57.09	7.91	15	21	1353	150.6	7.34
Q9H9S5	Fukutin-related protein OS=Homo sapiens GN=FKRP PE=1 SV=1 - [FKRP_HUMAN]	3.84	2	2	2	2	0.860	0.843	1.084	1.033	3.82	3.84	2	2	495	54.5	7.40
Q96EP0	E3 ubiquitin-protein ligase RNF31 OS=Homo sapiens GN=RNF31 PE=1 SV=1 - [RNF31_HUMAN]	6.06	1	5	5	17	1.144	0.961	0.972	1.033	34.30	6.06	10	17	1072	119.6	6.57
A6NLC5	UPF0524 protein C3orf70 OS=Homo sapiens GN=C3orf70 PE=2 SV=1 - [CC070_HUMAN]	6.00	1	1	1	1	1.457	1.110	1.695	1.033	2.07	6.00	1	1	250	27.8	4.96
Q5T0D9	Tumor protein p63-regulated gene 1-like protein OS=Homo sapiens GN=TPRG1L PE=1 SV=1 - [TPRGL_HUMAN]	20.96	1	3	3	10	0.790	0.921	0.934	1.033	21.87	20.96	4	10	272	30.2	7.34
O94916	Nuclear factor of activated T-cells 5 OS=Homo sapiens GN=NFAT5 PE=1 SV=1 - [NFAT5_HUMAN]	2.09	1	3	3	4	1.515	1.003	1.232	1.033	10.67	2.09	4	4	1531	165.7	5.24
Q15517	Corneodesmosin OS=Homo sapiens GN=CDSN PE=1 SV=3 - [CDSN_HUMAN]	7.75	1	2	2	2	0.497	1.945	0.968	1.033	2.10	7.75	2	2	529	51.5	8.35
Q10589	Bone marrow stromal antigen 2 OS=Homo sapiens GN=BST2 PE=1 SV=1 - [BST2_HUMAN]	5.56	1	1	1	4	3.117	0.616	1.788	1.033	8.09	5.56	2	4	180	19.8	5.60
Q96DG6	Carboxymethylenebutenolidase homolog OS=Homo sapiens GN=CMBL PE=1 SV=1 - [CMBL_HUMAN]	25.31	1	7	7	14	0.725	0.950	0.898	1.033	34.52	25.31	12	14	245	28.0	7.18

Q16394	Exostosin-1 OS=Homo sapiens GN=EXT1 PE=1 SV=2 - [EXT1_HUMAN]	1.61	1	1	1	1	1.185	1.140	1.630	1.033	1.90	1.61	1	1	746	86.2	9.04
O60341	Lysine-specific histone demethylase 1A OS=Homo sapiens GN=KDM1A PE=1 SV=2 - [KDM1A_HUMAN]	2.23	1	2	2	7	1.671	1.050	0.676	1.034	14.71	2.23	4	7	852	92.8	6.52
Q9H0J9	Poly [ADP-ribose] polymerase 12 OS=Homo sapiens GN=PARP12 PE=1 SV=1 - [PAR12_HUMAN]	4.99	1	3	3	4	1.491	0.939	1.312	1.034	5.84	4.99	3	4	701	79.0	8.51
Q9UJ68	Mitochondrial peptide methionine sulfoxide reductase OS=Homo sapiens GN=MSRA PE=1 SV=1 - [MSRA_HUMAN]	33.62	1	6	6	26	1.212	1.150	0.998	1.034	66.80	33.62	10	26	235	26.1	8.09
Q8WYA6	Beta-catenin-like protein 1 OS=Homo sapiens GN=CTNNBL1 PE=1 SV=1 - [CTBL1_HUMAN]	18.29	1	9	9	14	1.014	1.184	1.027	1.034	40.74	18.29	11	14	563	65.1	5.05
Q86T65	Disheveled-associated activator of morphogenesis 2 OS=Homo sapiens GN=DAAM2 PE=2 SV=3 - [DAAM2_HUMAN]	0.84	1	1	1	1		1.454	1.453	1.034	3.34	0.84	1	1	1068	123.4	6.80
Q8N8A6	ATP-dependent RNA helicase DDX51 OS=Homo sapiens GN=DDX51 PE=1 SV=3 - [DDX51_HUMAN	2.55	1	2	2	6	0.749	0.730	0.823	1.034	7.51	2.55	3	6	666	72.4	8.16
P09871	Complement C1s subcomponent OS=Homo sapiens GN=C1S PE=1 SV=1 - [C1S_HUMAN]	5.09	1	3	3	6	1.095	1.048	1.127	1.034	13.23	5.09	4	6	688	76.6	4.96
Q9UMY1	Nucleolar protein 7 OS=Homo sapiens GN=NOL7 PE=1 SV=2 - [NOL7_HUMAN]	10.51	1	3	3	8	0.738	0.958	0.959	1.034	24.94	10.51	5	8	257	29.4	9.67

Q96DE0	U8 snoRNA-decapping enzyme OS=Homo sapiens GN=NUDT16 PE=1 SV=2 - [NUD16_HUMAN]	17.95	2	4	4	12	0.952	0.824	1.144	1.034	23.62	17.95	8	12	195	21.3	6.89
Q99848	Probable rRNA-processing protein EBP2 OS=Homo sapiens GN=EBNA1BP2 PE=1 SV=2 - [EBP2_HUMAN]	38.89	1	13	13	36	0.754	0.882	0.761	1.034	80.67	38.89	22	36	306	34.8	10.10
O75379	Vesicle-associated membrane protein 4 OS=Homo sapiens GN=VAMP4 PE=1 SV=2 - [VAMP4_HUMAN]	36.88	1	4	4	5	0.779	0.524	1.041	1.034	18.01	36.88	4	5	141	16.4	7.34
O14795	Protein unc-13 homolog B OS=Homo sapiens GN=UNC13B PE=1 SV=2 - [UN13B_HUMAN]	3.02	1	3	4	9	1.918	1.208	1.319	1.034	25.70	3.02	6	9	1591	180.6	5.99
Q92890	Ubiquitin fusion degradation protein 1 homolog OS=Homo sapiens GN=UFD1L PE=1 SV=3 - [UFD1_HUMAN]	39.41	1	10	10	57	1.151	0.908	1.262	1.034	167.43	39.41	19	57	307	34.5	6.70
Q96IW7	Vesicle-trafficking protein SEC22a OS=Homo sapiens GN=SEC22A PE=1 SV=1 - [SC22A_HUMAN	6.19	1	2	2	3	1.107	0.938	1.044	1.034	5.59	6.19	2	3	307	34.9	8.24
Q53S33	BolA-like protein 3 OS=Homo sapiens GN=BOLA3 PE=1 SV=1 - [BOLA3_HUMAN]	34.58	1	4	5	5	0.784	1.000	0.974	1.035	12.75	34.58	5	5	107	12.1	9.64
O76094	Signal recognition particle subunit SRP72 OS=Homo sapiens GN=SRP72 PE=1 SV=3 - [SRP72_HUMAN]	40.98	1	25	28	73	0.888	0.952	0.973	1.035	169.22	40.98	38	73	671	74.6	9.26

Q14C86	GTPase-activating protein and VPS9 domain-containing protein 1 OS=Homo sapiens GN=GAPVD1 PE=1 SV=2 - [GAPD1_HUMAN]	12.72	1	18	18	45	0.981	0.816	0.999	1.035	101.31	12.72	28	45	1478	164.9	5.22
Q14197	Peptidyl-tRNA hydrolase ICT1, mitochondrial OS=Homo sapiens GN=ICT1 PE=1 SV=1 - [ICT1_HUMAN]	37.38	1	7	7	25	1.044	1.055	0.905	1.035	54.20	37.38	12	25	206	23.6	10.07
Q8WWL7	G2/mitotic-specific cyclin-B3 OS=Homo sapiens GN=CCNB3 PE=1 SV=2 - [CCNB3_HUMAN]	1.86	1	2	2	2	0.980	1.224	1.958	1.035	0.00	1.86	2	2	1395	157.8	6.68
Q9Y5T5	Ubiquitin carboxyl-terminal hydrolase 16 OS=Homo sapiens GN=USP16 PE=1 SV=1 - [UBP16_HUMAN]	6.20	1	3	4	7	1.047	1.191	1.354	1.035	12.85	6.20	5	7	823	93.5	6.93
O43488	Aflatoxin B1 aldehyde reductase member 2 OS=Homo sapiens GN=AKR7A2 PE=1 SV=3 - [ARK72_HUMAN]	18.66	3	8	8	22	1.172	1.344	1.296	1.035	53.07	18.66	13	22	359	39.6	7.17
Q9GZN8	UPF0687 protein C20orf27 OS=Homo sapiens GN=C20orf27 PE=1 SV=3 - [CT027_HUMAN]	4.02	1	1	1	2	1.084	1.234	1.386	1.035	0.00	4.02	1	2	174	19.3	6.84
Q6IAA8	Ragulator complex protein LAMTOR1 OS=Homo sapiens GN=LAMTOR1 PE=1 SV=2 - [LTOR1_HUMAN	40.37	1	5	5	17	1.064	1.055	0.920	1.035	64.15	40.37	9	17	161	17.7	5.15
A8MWD9	Putative small nuclear ribonucleoprotein G-like protein 15 OS=Homo sapiens GN=SNRPGP15 PE=5 SV=2 - [RUXGL_HUMAN	27.63	2	4	4	21	1.059	1.124	0.752	1.035	50.88	27.63	6	21	76	8.5	8.84

O00170	AH receptor-interacting protein OS=Homo sapiens GN=AIP PE=1 SV=2 - [AIP_HUMAN]	42.12	1	12	12	36	1.057	0.995	0.877	1.035	81.33	42.12	17	36	330	37.6	6.29
Q8NFB2	Transmembrane protein 185A OS=Homo sapiens GN=TMEM185A PE=1 SV=2 - [T185A_HUMAN]	8.57	2	2	2	3	1.277	0.832	1.025	1.035	5.08	8.57	2	3	350	40.6	7.24
Q8WY64	E3 ubiquitin-protein ligase MYLIP OS=Homo sapiens GN=MYLIP PE=1 SV=2 - [MYLIP_HUMAN]	4.27	1	2	2	2	0.898	1.316	0.865	1.035	1.98	4.27	2	2	445	49.9	7.24
Q96DZ1	Endoplasmic reticulum lectin 1 OS=Homo sapiens GN=ERLEC1 PE=1 SV=1 - [ERLEC_HUMAN]	21.33	1	9	9	42	1.247	0.742	0.727	1.035	107.67	21.33	16	42	483	54.8	6.28
Q12846	Syntaxin-4 OS=Homo sapiens GN=STX4 PE=1 SV=2 - [STX4_HUMAN]	42.09	1	10	10	51	1.452	1.154	1.067	1.035	121.37	42.09	17	51	297	34.2	6.28
Q9BX70	BTB/POZ domain-containing protein 2 OS=Homo sapiens GN=BTBD2 PE=1 SV=1 - [BTBD2_HUMAN]	4.19	1	2	2	2	1.067	0.774	0.720	1.036	3.27	4.19	2	2	525	55.9	5.90
P23458	Tyrosine-protein kinase JAK1 OS=Homo sapiens GN=JAK1 PE=1 SV=2 - [JAK1_HUMAN]	13.00	3	12	14	27	1.136	0.981	0.964	1.036	51.41	13.00	18	27	1154	133.2	7.55
P0CG23	Zinc finger protein 853 OS=Homo sapiens GN=ZNF853 PE=2 SV=1 - [ZN853_HUMAN]	1.97	1	1	1	1	0.724	1.210	1.298	1.036	2.56	1.97	1	1	659	74.8	5.25
Q9UJX6	Anaphase-promoting complex subunit 2 OS=Homo sapiens GN=ANAPC2 PE=1 SV=1 - [ANC2_HUMAN]	10.10	1	5	5	7	1.012	1.130	0.993	1.036	19.11	10.10	6	7	822	93.8	5.22

Q6ZMZ0	E3 ubiquitin-protein ligase RNF19B OS=Homo sapiens GN=RNF19B PE=1 SV=2 - [RN19B_HUMAN]	1.64	1	1	1	1	1.109	1.614	1.677	1.036	2.40	1.64	1	1	732	77.9	6.19
Q9H1K1	Iron-sulfur cluster assembly enzyme ISCU, mitochondrial OS=Homo sapiens GN=ISCU PE=1 SV=2 - [ISCU_HUMAN]	30.54	1	6	6	11	0.882	1.205	1.190	1.036	31.68	30.54	8	11	167	18.0	9.48
Q8N511	Transmembrane protein 199 OS=Homo sapiens GN=TMEM199 PE=1 SV=1 - [TM199_HUMAN]	7.21	1	2	2	9	0.931	0.797	1.006	1.036	17.21	7.21	3	9	208	23.1	9.01
Q63HM1	Kynurenine formamidase OS=Homo sapiens GN=AFMID PE=2 SV=2 - [KFA_HUMAN]	2.64	1	1	1	6	1.027	1.028	1.362	1.036	12.24	2.64	2	6	303	34.0	5.91
Q5XXA6	Anoctamin-1 OS=Homo sapiens GN=ANO1 PE=1 SV=1 - [ANO1_HUMAN]	10.45	2	8	10	24	0.590	0.608	1.998	1.036	65.10	10.45	14	24	986	114.0	8.54
P11310	Medium-chain specific acyl-CoA dehydrogenase, mitochondrial OS=Homo sapiens GN=ACADM PE=1 SV=1 - [ACADM_HUMAN]	26.37	1	11	11	29	0.867	1.406	0.999	1.036	67.21	26.37	19	29	421	46.6	8.37
O60610	Protein diaphanous homolog 1 OS=Homo sapiens GN=DIAPH1 PE=1 SV=2 - [DIAP1_HUMAN]	12.97	1	16	17	42	0.968	1.425	1.028	1.036	102.40	12.97	23	42	1272	141.3	5.41
Q9BYC5	Alpha-(1,6)-fucosyltransferase OS=Homo sapiens GN=FUT8 PE=1 SV=2 - [FUT8_HUMAN]	2.96	1	2	2	5	1.018	1.202	0.991	1.036	6.80	2.96	3	5	575	66.5	7.66
Q8N584	Tetratricopeptide repeat protein 39C OS=Homo sapiens GN=TTC39C PE=2 SV=2 - [TT39C_HUMAN	3.43	1	2	2	5	1.468	0.894	0.927	1.036	7.54	3.43	3	5	583	65.8	6.99

P54709	Sodium/potassium-transporting ATPase subunit beta-3 OS=Homo sapiens GN=ATP1B3 PE=1 SV=1 - [AT1B3_HUMAN]	35.84	1	11	11	33	1.520	0.653	0.591	1.036	79.81	35.84	17	33	279	31.5	8.35
P13693	Translationally-controlled tumor protein OS=Homo sapiens GN=TPT1 PE=1 SV=1 - [TCTP_HUMAN]	44.19	3	9	9	206	0.922	0.790	0.952	1.036	575.52	44.19	15	206	172	19.6	4.93
Q92551	Inositol hexakisphosphate kinase 1 OS=Homo sapiens GN=IP6K1 PE=1 SV=3 - [IP6K1_HUMAN]	3.17	1	1	1	1	1.199	0.921	1.321	1.037	3.68	3.17	1	1	441	50.2	7.24
H0Y7S4	Putative PRAME family member 26 OS=Homo sapiens GN=PRAMEF26 PE=3 SV=2 - [PRA26_HUMAN	1.83	8	1	1	1	1.260	1.374	1.476	1.037	1.88	1.83	1	1	382	44.3	8.28
Q6ZNW5	GDP-D-glucose phosphorylase 1 OS=Homo sapiens GN=GDPGP1 PE=1 SV=2 - [GDPP1_HUMAN]	7.53	1	2	2	4	0.880	1.130	1.044	1.037	5.73	7.53	4	4	385	42.3	6.47
P09661	U2 small nuclear ribonucleoprotein A' OS=Homo sapiens GN=SNRPA1 PE=1 SV=2 - [RU2A_HUMAN]	31.76	1	8	8	36	1.013	1.322	1.021	1.037	67.56	31.76	15	36	255	28.4	8.62
Q8IUC8	Polypeptide N-acetylgalactosaminyltransferase 13 OS=Homo sapiens GN=GALNT13 PE=2 SV=2 - [GLT13_HUMAN	2.88	1	1	2	3	1.823	1.192	0.664	1.037	7.00	2.88	2	3	556	64.0	6.83
P07108	Acyl-CoA-binding protein OS=Homo sapiens GN=DBI PE=1 SV=2 - [ACBP_HUMAN]	75.86	1	8	9	128	1.062	1.062	1.012	1.037	283.29	75.86	14	128	87	10.0	6.57
Q6VN20	Ran-binding protein 10 OS=Homo sapiens GN=RANBP10 PE=1 SV=1 - [RBP10_HUMAN]	10.48	1	3	3	7	1.062	1.225	1.189	1.037	17.53	10.48	4	7	620	67.2	6.77

Q96EG3	Zinc finger protein 837 OS=Homo sapiens GN=ZNF837 PE=2 SV=2 - [ZN837_HUMAN]	2.07	1	1	1	1	1.199	1.086	2.476	1.037	2.51	2.07	1	1	531	58.0	9.25
P61006	Ras-related protein Rab-8A OS=Homo sapiens GN=RAB8A PE=1 SV=1 - [RAB8A_HUMAN]	47.83	2	5	12	85	1.234	0.991	0.855	1.037	191.20	47.83	20	85	207	23.7	9.07
A1L188	Uncharacterized protein C17orf89 OS=Homo sapiens GN=C17orf89 PE=2 SV=1 - [CQ089_HUMAN]	14.86	1	1	1	12	0.751	0.734	0.930	1.037	34.82	14.86	1	12	74	7.8	9.32
O15431	High affinity copper uptake protein 1 OS=Homo sapiens GN=SLC31A1 PE=1 SV=1 - [COPT1_HUMAN]	10.00	1	1	1	4	1.507	0.686	0.825	1.037	10.60	10.00	2	4	190	21.1	7.42
Q8TB96	T-cell immunomodulatory protein OS=Homo sapiens GN=ITFG1 PE=1 SV=1 - [TIP_HUMAN]	3.92	1	3	3	5	0.946	0.795	1.142	1.037	8.11	3.92	3	5	612	68.1	5.39
P10644	cAMP-dependent protein kinase type I-alpha regulatory subunit OS=Homo sapiens GN=PRKAR1A PE=1 SV=1 - [KAP0_HUMAN]	47.24	1	14	17	65	0.931	1.147	1.221	1.038	185.37	47.24	25	65	381	43.0	5.35
Q460N5	Poly [ADP-ribose] polymerase 14 OS=Homo sapiens GN=PARP14 PE=1 SV=3 - [PAR14_HUMAN]	4.78	1	9	9	14	2.290	1.148	1.029	1.038	23.80	4.78	13	14	1801	202.7	7.18
A1A5D9	Bicaudal D-related protein 2 OS=Homo sapiens GN=CCDC64B PE=2 SV=2 - [BICR2_HUMAN]	8.07	1	2	3	5	1.793	0.694	1.499	1.038	7.96	8.07	3	5	508	56.8	5.02

P18031	Tyrosine-protein phosphatase non-receptor type 1 OS=Homo sapiens GN=PTPN1 PE=1 SV=1 - [PTN1_HUMAN]	34.02	1	14	14	51	1.184	0.611	1.128	1.038	133.34	34.02	20	51	435	49.9	6.27
Q86UU1	Pleckstrin homology-like domain family B member 1 OS=Homo sapiens GN=PHLDB1 PE=1 SV=1 - [PHLB1_HUMAN]	23.38	1	28	29	97	0.932	1.197	1.097	1.038	217.37	23.38	49	97	1377	151.1	8.63
P19447	TFIIH basal transcription factor complex helicase XPB subunit OS=Homo sapiens GN=ERCC3 PE=1 SV=1 - [ERCC3_HUMAN]	4.73	1	3	3	4	0.844	1.226	0.810	1.038	11.30	4.73	3	4	782	89.2	7.23
O14641	Segment polarity protein dishevelled homolog DVL-2 OS=Homo sapiens GN=DVL2 PE=1 SV=1 - [DVL2_HUMAN]	10.87	1	6	6	18	1.070	1.273	1.271	1.038	39.27	10.87	10	18	736	78.9	6.02
O75386	Tubby-related protein 3 OS=Homo sapiens GN=TULP3 PE=1 SV=2 - [TULP3_HUMAN]	5.88	1	3	3	9	1.129	0.916	1.123	1.038	24.98	5.88	5	9	442	49.6	7.74
O95989	Diphosphoinositol polyphosphate phosphohydrolase 1 OS=Homo sapiens GN=NUDT3 PE=1 SV=1 - [NUDT3_HUMAN]	30.81	1	4	5	10	1.077	1.077	0.968	1.038	29.31	30.81	6	10	172	19.5	6.34
O60942	mRNA-capping enzyme OS=Homo sapiens GN=RNGTT PE=1 SV=1 - [MCE1_HUMAN]	1.01	1	1	1	1	1.414	0.927	1.211	1.038	2.23	1.01	1	1	597	68.5	8.13
Q14257	Reticulocalbin-2 OS=Homo sapiens GN=RCN2 PE=1 SV=1 - [RCN2_HUMAN]	54.57	1	14	15	46	1.121	0.904	0.900	1.038	152.13	54.57	20	46	317	36.9	4.40

Q96BY7	Autophagy-related protein 2 homolog B OS=Homo sapiens GN=ATG2B PE=1 SV=5 - [ATG2B_HUMAN]	5.29	1	8	8	10	1.223	0.795	1.209	1.038	15.80	5.29	10	10	2078	232.6	5.76
A6NI56	Coiled-coil domain-containing protein 154 OS=Homo sapiens GN=CCDC154 PE=2 SV=4 - [CC154_HUMAN]	1.19	1	1	1	1	0.716	1.068	0.843	1.039	0.00	1.19	1	1	674	76.0	8.38
Q8NBX0	Saccharopine dehydrogenase-like oxidoreductase OS=Homo sapiens GN=SCCPDH PE=1 SV=1 - [SCPDL_HUMAN]	17.48	1	6	6	25	1.045	1.269	0.987	1.039	59.58	17.48	10	25	429	47.1	9.14
Q13362	Serine/threonine-protein phosphatase 2A 56 kDa regulatory subunit gamma isoform OS=Homo sapiens GN=PPP2R5C PE=1 SV=3 - [2A5G_HUMAN]	14.89	1	3	7	25	1.276	0.904	0.902	1.039	44.93	14.89	12	25	524	61.0	6.87
Q9HB40	Retinoid-inducible serine carboxypeptidase OS=Homo sapiens GN=SCPEP1 PE=1 SV=1 - [RISC_HUMAN]	19.91	1	9	9	30	0.972	0.888	1.066	1.039	72.77	19.91	12	30	452	50.8	5.81
Q86XL3	Ankyrin repeat and LEM domain-containing protein 2 OS=Homo sapiens GN=ANKLE2 PE=1 SV=4 - [ANKL2_HUMAN]	21.22	1	15	15	42	0.847	0.886	1.011	1.039	65.03	21.22	24	42	938	104.0	7.09
Q96AX9	E3 ubiquitin-protein ligase MIB2 OS=Homo sapiens GN=MIB2 PE=1 SV=3 - [MIB2_HUMAN]	4.24	1	3	4	6	1.155	1.159	1.429	1.039	9.08	4.24	5	6	1013	109.9	8.44

P36873	Serine/threonine-protein phosphatase PP1-gamma catalytic subunit OS=Homo sapiens GN=PPP1CC PE=1 SV=1 - [PP1G_HUMAN]	45.82	1	3	13	84	0.923	0.964	1.041	1.039	229.33	45.82	22	84	323	37.0	6.54
Q14004	Cyclin-dependent kinase 13 OS=Homo sapiens GN=CDK13 PE=1 SV=2 - [CDK13_HUMAN]	3.77	6	4	6	16	0.654	0.900	0.864	1.039	29.84	3.77	7	16	1512	164.8	9.69
A6QL64	Ankyrin repeat domain-containing protein 36A OS=Homo sapiens GN=ANKRD36 PE=2 SV=3 - [AN36A_HUMAN]	0.36	1	1	1	1	1.133	1.113	1.104	1.039	2.42	0.36	1	1	1941	217.3	8.66
Q15050	Ribosome biogenesis regulatory protein homolog OS=Homo sapiens GN=RRS1 PE=1 SV=2 - [RRS1_HUMAN]	27.40	1	8	8	26	0.649	1.129	0.791	1.039	55.69	27.40	13	26	365	41.2	10.70
Q96A57	Transmembrane protein 230 OS=Homo sapiens GN=TMEM230 PE=1 SV=1 - [TM230_HUMAN]	9.17	1	1	1	6	0.648	0.906	0.929	1.040	11.96	9.17	2	6	120	13.2	9.31
P84098	60S ribosomal protein L19 OS=Homo sapiens GN=RPL19 PE=1 SV=1 - [RL19_HUMAN]	39.29	1	10	11	139	0.701	1.023	0.907	1.040	284.92	39.29	16	139	196	23.5	11.47
Q6NUQ4	Transmembrane protein 214 OS=Homo sapiens GN=TMEM214 PE=1 SV=2 - [TM214_HUMAN]	22.79	1	15	15	58	0.935	1.140	0.938	1.040	113.00	22.79	24	58	689	77.1	9.14
Q3T906	N-acetylglucosamine-1-phosphotransferase subunits alpha/beta OS=Homo sapiens GN=GNPTAB PE=1 SV=1 - [GNPTA_HUMAN]	0.64	1	1	1	2	0.794	0.961	1.328	1.040	1.92	0.64	2	2	1256	143.5	7.17

Q14160	Protein scribble homolog OS=Homo sapiens GN=SCRIB PE=1 SV=4 - [SCRIB_HUMAN]	12.09	1	13	18	52	1.117	1.252	1.108	1.040	126.51	12.09	29	52	1630	174.8	5.07
O15397	Importin-8 OS=Homo sapiens GN=IPO8 PE=1 SV=2 - [IPO8_HUMAN]	6.46	1	6	6	11	0.819	1.030	1.260	1.040	33.92	6.46	8	11	1037	119.9	5.16
Q5SNT6	WASH complex subunit FAM21B OS=Homo sapiens GN=FAM21B PE=2 SV=2 - [FA21B_HUMAN]	28.89	2	6	25	74	1.117	1.192	1.024	1.040	187.62	28.89	40	74	1253	136.9	4.82
Q9NZM4	Glioma tumor suppressor candidate region gene 1 protein OS=Homo sapiens GN=GLTSCR1 PE=1 SV=2 - [GSCR1_HUMAN	3.40	1	3	3	3	0.919	0.851	1.246	1.040	5.10	3.40	3	3	1560	158.4	6.65
P09012	U1 small nudear ribonudeoprot ein A OS=Homo sapiens GN=SNRPA PE=1 SV=3 -	27.30	1	6	8	71	0.859	1.042	1.002	1.040	158.53	27.30	16	71	282	31.3	9.83
Q8IY81	pre-rRNA processing protein FTSJ3 OS=Homo sapiens GN=FTSJ3 PE=1 SV=2 - [SPB1_HUMAN]	17.36	1	15	15	42	0.829	1.067	0.904	1.040	108.30	17.36	23	42	847	96.5	8.40
P53384	Cytosolic Fe-S duster assembly factor NUBP1 OS=Homo sapiens GN=NUBP1 PE=1 SV=2 - [NUBP1_HUMAN	18.13	1	4	4	5	1.145	1.341	1.052	1.040	11.48	18.13	5	5	320	34.5	5.33
P09914	Interferon-induced protein with tetratricopepti de repeats 1 OS=Homo sapiens GN=IFIT1 PE=1 SV=2 - [IFIT1_HUMAN]	18.41	2	7	9	28	6.339	1.268	2.217	1.040	69.49	18.41	15	28	478	55.3	7.20
Q12874	Splicing factor 3A subunit 3 OS=Homo sapiens GN=SF3A3 PE=1 SV=1 - [SF3A3_HUMAN]	32.34	1	12	12	50	1.043	1.189	0.891	1.040	137.19	32.34	21	50	501	58.8	5.38

Q9UBW8	COP9 signalosome complex subunit 7a OS=Homo sapiens GN=COPS7A PE=1 SV=1 - [CSN7A_HUMAN]	45.09	1	11	11	26	0.916	1.153	1.080	1.040	45.42	45.09	17	26	275	30.3	8.22
Q6UVJ0	Spindle assembly abnormal protein 6 homolog OS=Homo sapiens GN=SASS6 PE=1 SV=1 - [SAS6_HUMAN]	5.18	1	1	4	8	1.059	0.908	0.851	1.041	11.73	5.18	4	8	657	74.4	7.55
Q99541	Perilipin-2 OS=Homo sapiens GN=PLIN2 PE=1 SV=2 - [PLIN2_HUMAN]	45.77	1	17	18	70	0.275	0.933	0.919	1.041	187.74	45.77	29	70	437	48.0	6.80
P41229	Lysine-specific demethylase 5C OS=Homo sapiens GN=KDM5C PE=1 SV=2 - [KDM5C_HUMAN]	2.82	1	3	3	7	1.427	1.163	1.266	1.041	2.64	2.82	3	7	1560	175.6	5.58
Q8IVF7	Formin-like protein 3 OS=Homo sapiens GN=FMNL3 PE=1 SV=3 - [FMNL3_HUMAN]	4.96	2	4	6	12	1.120	0.995	1.239	1.041	24.91	4.96	8	12	1028	117.1	6.65
Q5XKP0	Protein QIL1 OS=Homo sapiens GN=QIL1 PE=1 SV=1 - [QIL1_HUMAN]	5.93	1	1	1	4	1.081	1.008	0.722	1.041	8.63	5.93	2	4	118	13.1	9.42
P39019	40S ribosomal protein S19 OS=Homo sapiens GN=RPS19 PE=1 SV=2 - [RS19_HUMAN]	54.48	1	13	13	250	0.906	0.948	1.020	1.041	532.73	54.48	25	250	145	16.1	10.32
O00468	Agrin OS=Homo sapiens GN=AGRN PE=1 SV=5 - [AGRIN_HUMAN]	3.19	1	7	7	14	1.084	0.784	1.450	1.041	20.59	3.19	10	14	2067	217.1	6.39
P57053	Histone H2B type F-S OS=Homo sapiens GN=H2BFS PE=1 SV=2 - [H2BFS_HUMAN]	66.67	1	1	12	420	1.164	1.230	0.599	1.041	924.28	66.67	22	420	126	13.9	10.37

Q99590	Protein SCAF11 OS=Homo sapiens GN=SCAF11 PE=1 SV=2 - [SCAFB_HUMAN]	2.26	1	4	4	13	0.978	1.038	0.795	1.041	22.01	2.26	6	13	1463	164.6	8.41
Q86UP2	Kinectin OS=Homo sapiens GN=KTN1 PE=1 SV=1 - [KTN1_HUMAN]	66.47	2	98	100	426	1.031	1.029	0.948	1.041	1062.22	66.47	158	426	1357	156.2	5.64
Q8NCH0	Carbohydrate sulfotransferase 14 OS=Homo sapiens GN=CHST14 PE=1 SV=2 - [CHSTE_HUMAN	7.18	1	3	3	15	0.943	0.552	0.638	1.041	33.01	7.18	5	15	376	43.0	9.48
Q9BYD3	39S ribosomal protein L4, mitochondrial OS=Homo sapiens GN=MRPL4 PE=1 SV=1 - [RM04_HUMAN]	12.54	1	3	4	15	0.775	1.078	0.881	1.042	26.72	12.54	6	15	311	34.9	9.72
Q7KZI7	Serine/threonine-protein kinase MARK2 OS=Homo sapiens GN=MARK2 PE=1 SV=2 - [MARK2_HUMAN]	3.93	1	2	4	4	1.540	0.962	1.050	1.042	6.70	3.93	4	4	788	87.9	9.72
Q96EY4	Translation machinery-associated protein 16 OS=Homo sapiens GN=TMA16 PE=1 SV=2 - [TMA16_HUMAN]	4.43	1	1	1	2	1.167	0.964	0.836	1.042	2.95	4.43	2	2	203	23.8	9.26
Q9H3N1	Thioredoxin-related transmembrane protein 1 OS=Homo sapiens GN=TMX1 PE=1 SV=1 - [TMX1_HUMAN]	26.43	1	10	10	60	1.022	0.766	0.658	1.042	139.02	26.43	17	60	280	31.8	4.98
Q9Y2G3	Probable phospholipid-transporting ATPase IF OS=Homo sapiens GN=ATP11B PE=1 SV=2 - [AT11B_HUMAN]	3.65	1	4	4	6	1.293	0.857	0.860	1.042	15.22	3.65	6	6	1177	134.1	6.96
A4D1E9	GTP-binding protein 10 OS=Homo sapiens GN=GTPBP10 PE=1 SV=1 - [GTPBA_HUMAN]	2.84	1	1	1	2	0.494	1.243	1.221	1.042	0.00	2.84	2	2	387	42.9	9.03

Q60I27	ALS2 C-terminal-like protein OS=Homo sapiens GN=ALS2CL PE=1 SV=1 - [AL2CL_HUMAN]	0.73	1	1	1	2	4.998	0.991	1.668	1.042	5.09	0.73	1	2	953	107.7	6.15
Q8N9R8	Protein SCAI OS=Homo sapiens GN=SCAI PE=1 SV=2 - [SCAI_HUMAN]	2.48	1	1	1	2	1.030	0.961	1.095	1.042	6.33	2.48	2	2	606	70.4	8.60
Q9Y2C4	Nuclease EXOG, mitochondrial OS=Homo sapiens GN=EXOG PE=1 SV=2 - [EXOG_HUMAN]	6.79	1	2	3	7	1.062	1.095	1.137	1.042	16.69	6.79	4	7	368	41.1	8.27
P82914	28S ribosomal protein S15, mitochondrial OS=Homo sapiens GN=MRPS15 PE=1 SV=1 - [RT15_HUMAN]	14.40	2	5	5	15	0.917	0.913	0.719	1.042	28.87	14.40	7	15	257	29.8	10.48
Q9Y2B0	Protein canopy homolog 2 OS=Homo sapiens GN=CNPY2 PE=1 SV=1 - [CNPY2_HUMAN]	61.54	1	11	11	80	0.802	0.989	0.932	1.042	183.02	61.54	20	80	182	20.6	4.92
Q96TC7	Regulator of microtubule dynamics protein 3 OS=Homo sapiens GN=RMDN3 PE=1 SV=2 - [RMD3_HUMAN]	30.00	1	10	10	32	1.349	0.733	0.752	1.042	92.94	30.00	14	32	470	52.1	5.10
Q9NP80	Calcium-independent phospholipase A2-gamma OS=Homo sapiens GN=PNPLA8 PE=1 SV=1 - [PLPL8_HUMAN]	7.42	2	4	7	12	0.931	0.901	1.106	1.042	19.59	7.42	9	12	782	88.4	9.23
P30086	Phosphatidylethanolamine-binding protein 1 OS=Homo sapiens GN=PEBP1 PE=1 SV=3 - [PEBP1_HUMAN]	81.28	1	12	12	212	1.176	1.002	1.100	1.042	419.47	81.28	20	212	187	21.0	7.53

P48454	Serine/threonine-protein phosphatase 2B catalytic subunit gamma isoform OS=Homo sapiens GN=PPP3CC PE=1 SV=3 - [PP2BC_HUMAN	8.20	1	2	4	22	1.881	1.188	1.389	1.042	40.11	8.20	8	22	512	58.1	6.98
Q9UL26	Ras-related protein Rab-22A OS=Homo sapiens GN=RAB22A PE=1 SV=2 - [RB22A_HUMAN]	16.49	1	2	3	14	1.227	1.044	0.886	1.043	35.96	16.49	5	14	194	21.8	8.15
O94964	Protein SOGA1 OS=Homo sapiens GN=SOGA1 PE=1 SV=2 - [SOGA1_HUMA N]	3.30	1	3	4	7	1.119	1.280	1.044	1.043	18.67	3.30	5	7	1423	159.7	6.46
A6NE01	Protein FAM186A OS=Homo sapiens GN=FAM186A PE=2 SV=3 - [F186A_HUMAN]	0.34	1	1	1	2	0.966	1.072	0.744	1.043	4.85	0.34	1	2	2351	262.6	8.60
Q96D46	60S ribosomal export protein NMD3 OS=Homo sapiens GN=NMD3 PE=1 SV=1 - [NMD3_HUMAN]	12.72	1	5	5	7	0.682	1.006	1.793	1.043	24.47	12.72	5	7	503	57.6	7.14
Q5T5N4	Uncharacterized protein C6orf118 OS=Homo sapiens GN=C6orf118 PE=2 SV=1 - [CF118_HUMAN]	1.71	1	1	1	3	1.017	0.959	0.848	1.043	2.46	1.71	1	3	469	53.7	8.37
Q8IVC4	Zinc finger protein 584 OS=Homo sapiens GN=ZNF584 PE=2 SV=1 - [ZN584_HUMAN]	2.38	1	1	1	1	1.165	0.127	1.202	1.043	0.00	2.38	1	1	421	48.2	8.76
Q05086	Ubiquitin-protein ligase E3A OS=Homo sapiens GN=UBE3A PE=1 SV=4 - [UBE3A_HUMAN]	14.06	1	10	10	22	0.927	1.189	0.865	1.043	64.10	14.06	14	22	875	100.6	5.22
Q15059	Bromodomain-containing protein 3 OS=Homo sapiens GN=BRD3 PE=1 SV=1 - [BRD3_HUMAN]	8.26	1	5	6	9	1.414	2.038	1.236	1.043	14.78	8.26	8	9	726	79.5	9.36

Q2TAA2	Isoamyl acetate-hydrolyzing esterase 1 homolog OS=Homo sapiens GN=IAH1 PE=1 SV=1 - [IAH1_HUMAN]	11.29	1	3	3	21	1.097	1.139	0.942	1.043	44.50	11.29	6	21	248	27.6	5.30
Q99714	3-hydroxyacyl-CoA dehydrogenase type-2 OS=Homo sapiens GN=HSD17B10 PE=1 SV=3 - [HCD2_HUMAN]	60.92	1	11	11	55	1.096	0.998	0.698	1.043	193.24	60.92	17	55	261	26.9	7.78
P21854	B-cell differentiation antigen CD72 OS=Homo sapiens GN=CD72 PE=1 SV=1 - [CD72_HUMAN]	3.90	1	1	1	2	1.545	0.803	1.110	1.043	5.93	3.90	1	2	359	40.2	8.28
P55039	Developmentally-regulated GTP-binding protein 2 OS=Homo sapiens GN=DRG2 PE=1 SV=1 - [DRG2_HUMAN]	35.99	1	11	11	24	1.090	1.147	1.039	1.043	55.25	35.99	17	24	364	40.7	8.88
Q8NBR6	Protein FAM63B OS=Homo sapiens GN=FAM63B PE=1 SV=2 - [FA63B_HUMAN]	2.74	1	2	2	3	1.149	1.110	1.597	1.043	5.75	2.74	3	3	621	67.1	4.51
Q96SZ5	2-aminoethanethiol dioxygenase OS=Homo sapiens GN=ADO PE=1 SV=2 - [AEDO_HUMAN]	22.59	1	6	6	13	1.148	0.997	1.181	1.044	18.16	22.59	9	13	270	29.7	6.04
Q9C035	Tripartite motif-containing protein 5 OS=Homo sapiens GN=TRIM5 PE=1 SV=1 - [TRIM5_HUMAN]	21.30	1	8	8	16	1.239	0.977	1.116	1.044	38.89	21.30	11	16	493	56.3	6.05
Q01105	Protein SET OS=Homo sapiens GN=SET PE=1 SV=3 - [SET_HUMAN]	45.17	2	11	11	191	1.043	1.111	0.991	1.044	546.81	45.17	15	191	290	33.5	4.32

P50151	Guanine nucleotide-binding protein G(I)/G(S)/G(O) subunit gamma-10 OS=Homo sapiens GN=GNG10 PE=1 SV=1 - [GBG10_HUMAN]	30.88	1	1	2	3	1.127	0.366	0.371	1.044	10.11	30.88	2	3	68	7.2	7.85
Q9UPM6	LIM/homeobox protein Lhx6 OS=Homo sapiens GN=LHX6 PE=2 SV=2 - [LHX6_HUMAN]	2.48	1	1	1	1	0.943	1.207	1.704	1.044	3.16	2.48	1	1	363	40.0	8.40
Q5VW32	BRO1 domain-containing protein BROX OS=Homo sapiens GN=BROX PE=1 SV=1 - [BROX_HUMAN]	26.76	1	9	9	19	1.287	1.295	1.056	1.044	31.59	26.76	11	19	411	46.4	7.65
Q10713	Mitochondrial-processing peptidase subunit alpha OS=Homo sapiens GN=PMPCA PE=1 SV=2 - [MPPA_HUMAN]	29.33	1	12	12	39	0.989	1.406	1.216	1.044	94.28	29.33	22	39	525	58.2	6.92
Q9ULI0	ATPase family AAA domain-containing protein 2B OS=Homo sapiens GN=ATAD2B PE=1 SV=3 - [ATD2B_HUMAN]	1.51	1	2	2	2	1.142	0.958	1.019	1.044	4.59	1.51	2	2	1458	164.8	6.80
O60828	Polyglutamine-binding protein 1 OS=Homo sapiens GN=PQBP1 PE=1 SV=1 - [PQBP1_HUMAN]	17.36	1	4	4	8	0.797	0.893	0.606	1.044	19.50	17.36	6	8	265	30.5	6.33
Q5T5Y3	Calmodulin-regulated spectrin-associated protein 1 OS=Homo sapiens GN=CAMSAP1 PE=1 SV=2 - [CAMP1_HUMAN]	4.93	1	5	7	15	1.054	1.124	1.084	1.045	35.17	4.93	12	15	1602	177.9	6.73
Q9H0V9	VIP36-like protein OS=Homo sapiens GN=LMAN2L PE=1 SV=1 - [LMA2L_HUMAN]	24.71	1	7	7	28	1.010	1.066	0.951	1.045	79.13	24.71	10	28	348	39.7	8.38

Q96T23	Remodeling and spacing factor 1 OS=Homo sapiens GN=RSF1 PE=1 SV=2 - [RSF1_HUMAN]	5.76	1	7	7	18	0.929	1.189	1.131	1.045	44.10	5.76	11	18	1441	163.7	5.01
Q9Y508	E3 ubiquitin-protein ligase RNF114 OS=Homo sapiens GN=RNF114 PE=1 SV=1 - [RN114_HUMAN]	23.68	1	5	5	13	1.211	0.850	1.239	1.045	28.97	23.68	8	13	228	25.7	7.25
Q13838	Spliceosome RNA helicase DDX39B OS=Homo sapiens GN=DDX39B PE=1 SV=1 - [DX39B_HUMAN]	41.36	1	8	19	131	1.135	1.105	1.305	1.045	326.72	41.36	26	131	428	49.0	5.67
Q9BV79	Trans-2-enoyl-CoA reductase, mitochondrial OS=Homo sapiens GN=MECR PE=1 SV=2 - [MECR_HUMAN]	14.21	1	5	5	21	0.980	1.420	1.116	1.045	37.61	14.21	9	21	373	40.4	8.76
Q9Y285	Phenylalanine--tRNA ligase alpha subunit OS=Homo sapiens GN=FARSA PE=1 SV=3 - [SYFA_HUMAN]	31.69	1	15	15	81	0.801	0.929	1.010	1.045	225.73	31.69	24	81	508	57.5	7.80
Q8N5Z0	Kynurenine/alpha-aminoadipate aminotransferase, mitochondrial OS=Homo sapiens GN=AADAT PE=1 SV=2 -	1.88	1	1	1	1	0.452	0.767	0.439	1.045	2.85	1.88	1	1	425	47.3	6.96
Q15084	Protein disulfide-isomerase A6 OS=Homo sapiens GN=PDIA6 PE=1 SV=1 - [PDIA6_HUMAN	50.00	1	21	22	283	0.764	0.965	0.850	1.045	731.99	50.00	38	283	440	48.1	5.08
P55210	Caspase-7 OS=Homo sapiens GN=CASP7 PE=1 SV=1 - [CASP7_HUMAN]	24.75	1	7	7	27	1.562	0.730	0.751	1.045	63.73	24.75	12	27	303	34.3	6.07
Q8NFZ8	Cell adhesion molecule 4 OS=Homo sapiens GN=CADM4 PE=1 SV=1 - [CADM4_HUMAN]	2.32	1	1	1	1	1.495	1.134	1.451	1.045	2.35	2.32	1	1	388	42.8	6.30

Q16891	MICOS complex subunit MIC60 OS=Homo sapiens GN=IMMT PE=1 SV=1 - [MIC60_HUMAN]	48.94	1	40	43	202	0.929	1.011	0.826	1.045	497.05	48.94	71	202	758	83.6	6.48
Q92947	Glutaryl-CoA dehydrogenase, mitochondrial OS=Homo sapiens GN=GCDH PE=1 SV=1 - [GCDH_HUMAN]	21.69	2	8	9	24	0.960	1.206	1.145	1.045	62.91	21.69	13	24	438	48.1	8.06
Q9UKK9	ADP-sugar pyrophosphatase OS=Homo sapiens GN=NUDT5 PE=1 SV=1 - [NUDT5_HUMAN]	55.71	1	10	10	50	0.934	0.880	0.843	1.046	103.74	55.71	16	50	219	24.3	4.94
P46060	Ran GTPase-activating protein 1 OS=Homo sapiens GN=RANGAP1 PE=1 SV=1 - [RAGP1_HUMAN]	38.84	1	25	25	125	1.069	0.934	1.008	1.046	295.48	38.84	43	125	587	63.5	4.68
P05161	Ubiquitin-like protein ISG15 OS=Homo sapiens GN=ISG15 PE=1 SV=5 - [ISG15_HUMAN]	35.15	1	5	5	15	5.412	0.669	1.285	1.046	37.18	35.15	8	15	165	17.9	7.44
Q15393	Splicing factor 3B subunit 3 OS=Homo sapiens GN=SF3B3 PE=1 SV=4 - [SF3B3_HUMAN]	23.58	1	28	29	107	0.962	1.119	0.857	1.046	244.91	23.58	49	107	1217	135.5	5.26
Q07020	60S ribosomal protein L18 OS=Homo sapiens GN=RPL18 PE=1 SV=2 - [RL18_HUMAN]	41.49	1	8	8	107	0.744	0.849	0.935	1.046	221.45	41.49	12	107	188	21.6	11.72
Q99442	Translocation protein SEC62 OS=Homo sapiens GN=SEC62 PE=1 SV=1 - [SEC62_HUMAN]	17.79	1	9	9	26	0.899	1.243	0.857	1.046	50.83	17.79	14	26	399	45.8	7.12
Q9UPN3	Microtubule-actin cross-linking factor 1, isoforms 1/2/3/5 OS=Homo sapiens GN=MACF1 PE=1 SV=4 - [MACF1_HUMAN]	37.30	2	255	262	981	1.134	0.871	1.125	1.046	2388.65	37.30	406	981	7388	837.8	5.39

Q8WW01	tRNA-splicing endonuclease subunit Sen15 OS=Homo sapiens GN=TSEN15 PE=1 SV=1 - [SEN15_HUMAN]	23.98	1	2	2	2	0.783	1.582	1.200	1.046	9.78	23.98	2	2	171	18.6	4.58
P68400	Casein kinase II subunit alpha OS=Homo sapiens GN=CSNK2A1 PE=1 SV=1 - [CSK21_HUMAN	52.69	2	13	15	37	0.993	0.981	1.098	1.046	103.70	52.69	22	37	391	45.1	7.74
Q08J23	tRNA (cytosine(34)-C(5))-methyltransferase OS=Homo sapiens GN=NSUN2 PE=1 SV=2 - [NSUN2_HUMAN]	22.69	1	17	17	59	0.925	0.927	0.996	1.046	149.78	22.69	26	59	767	86.4	6.77
P08670	Vimentin OS=Homo sapiens GN=VIM PE=1 SV=4 - [VIME_HUMAN]	89.06	3	52	62	5709	1.151	0.833	1.138	1.046	#####	89.06	117	5709	466	53.6	5.12
O95757	Heat shock 70 kDa protein 4L OS=Homo sapiens GN=HSPA4L PE=1 SV=3 - [HS74L_HUMAN]	21.10	1	14	19	51	1.241	1.437	1.497	1.046	127.79	21.10	27	51	839	94.5	5.88
P09341	Growth-regulated alpha protein OS=Homo sapiens GN=CXCL1 PE=1 SV=1 - [GROA_HUMAN]	10.28	1	1	1	6	3.383	0.539	0.357	1.046	10.23	10.28	2	6	107	11.3	10.43
Q96LX8	Zinc finger protein 597 OS=Homo sapiens GN=ZNF597 PE=2 SV=1 - [ZN597_HUMAN]	4.48	94	1	2	3	2.298	1.477	1.512	1.046	2.07	4.48	2	3	424	48.0	6.76
Q96GA7	Serine dehydratase-like OS=Homo sapiens GN=SDSL PE=1 SV=1 - [SDSL_HUMAN]	24.62	1	5	6	9	0.860	0.650	0.795	1.047	24.19	24.62	8	9	329	34.7	6.89
O60551	Glycylpeptide N-tetradecanoyltransferase 2 OS=Homo sapiens GN=NMT2 PE=1 SV=1 - [NMT2_HUMAN]	27.11	1	9	10	22	0.815	1.126	1.058	1.047	71.55	27.11	13	22	498	56.9	7.58

A2RU30	Protein TESPA1 OS=Homo sapiens GN=TESPA1 PE=1 SV=2 - [TESP1_HUMAN]	3.07	1	1	1	1	1.590	1.367	1.939	1.047	0.00	3.07	1	1	521	59.2	5.71
P51858	Hepatoma-derived growth factor OS=Homo sapiens GN=HDGF PE=1 SV=1 - [HDGF_HUMAN]	43.75	1	11	12	69	1.185	1.161	1.015	1.047	151.54	43.75	19	69	240	26.8	4.73
P49247	Ribose-5-phosphate isomerase OS=Homo sapiens GN=RPIA PE=1 SV=3 - [RPIA_HUMAN]	9.97	1	3	3	6	1.213	1.298	1.073	1.047	15.45	9.97	3	6	311	33.2	8.54
P49716	CCAAT/enhancer-binding protein delta OS=Homo sapiens GN=CEBPD PE=1 SV=2 - [CEBPD_HUMAN]	8.18	1	2	2	4	1.154	2.909	1.297	1.047	11.33	8.18	2	4	269	28.4	8.28
Q9H9C1	Spermatogenesis-defective protein 39 homolog OS=Homo sapiens GN=VIPAS39 PE=1 SV=1 - [SPE39_HUMAN]	21.91	1	10	10	36	1.048	1.098	1.292	1.047	96.44	21.91	16	36	493	57.0	7.40
O00559	Receptor-binding cancer antigen expressed on SiSo cells OS=Homo sapiens GN=EBAG9 PE=1 SV=1 - [RCAS1_HUMAN]	26.76	1	3	3	3	0.419	0.780	0.870	1.047	10.46	26.76	3	3	213	24.4	6.29
O60234	Glia maturation factor gamma OS=Homo sapiens GN=GMFG PE=1 SV=1 - [GMFG_HUMAN]	14.08	1	1	3	16	1.095	1.062	1.019	1.047	27.23	14.08	5	16	142	16.8	5.26
Q6UUV7	CREB-regulated transcription coactivator 3 OS=Homo sapiens GN=CRTC3 PE=1 SV=2 - [CRTC3_HUMAN	1.62	1	1	1	2	0.871	1.290	1.011	1.047	7.63	1.62	2	2	619	66.9	6.84
Q9H0W8	Protein SMG9 OS=Homo sapiens GN=SMG9 PE=1 SV=1 - [SMG9_HUMAN]	12.88	1	5	5	21	1.030	1.051	1.335	1.047	39.98	12.88	7	21	520	57.6	7.01

P82663	28S ribosomal protein S25, mitochondrial OS=Homo sapiens GN=MRPS25 PE=1 SV=1 - [RT25_HUMAN]	30.06	1	5	5	9	0.864	0.961	0.847	1.047	25.38	30.06	7	9	173	20.1	8.82
Q8NBV8	Synaptotagmin-8 OS=Homo sapiens GN=SYT8 PE=2 SV=4 - [SYT8_HUMAN]	2.00	1	1	1	2	0.674	0.421	0.403	1.047	5.52	2.00	1	2	401	44.1	9.54
B5MCN3	Putative SEC14-like protein 6 OS=Homo sapiens GN=SEC14L6 PE=5 SV=1 - [S14L6_HUMAN]	2.27	1	1	1	1	1.179	1.138	0.974	1.047	2.51	2.27	1	1	397	45.3	7.06
P57105	Synaptojanin-2-binding protein OS=Homo sapiens GN=SYNJ2BP PE=1 SV=2 - [SYJ2B_HUMAN]	42.76	1	5	5	19	1.033	0.992	0.908	1.048	46.29	42.76	8	19	145	15.9	6.30
O14545	TRAF-type zinc finger domain-containing protein 1 OS=Homo sapiens GN=TRAFD1 PE=1 SV=1 - [TRAD1_HUMAN]	6.01	1	3	3	11	0.814	1.390	1.005	1.048	32.76	6.01	5	11	582	64.8	5.29
Q9BQQ3	Golgi reassembly-stacking protein 1 OS=Homo sapiens GN=GORASP1 PE=1 SV=3 - [GORS1_HUMA	3.41	1	1	2	10	0.831	0.834	0.786	1.048	14.68	3.41	4	10	440	46.5	4.50
Q8TC07	TBC1 domain family member 15 OS=Homo sapiens GN=TBC1D15 PE=1 SV=2 - [TBC15_HUMAN]	13.31	1	8	8	27	0.937	1.027	1.102	1.048	63.16	13.31	13	27	691	79.4	5.67
O76031	ATP-dependent Clp protease ATP-binding subunit clpX-like, mitochondrial OS=Homo sapiens GN=CLPX PE=1 SV=2 - [CLPX_HUMAN]	22.75	1	12	12	36	0.864	0.985	0.976	1.048	95.92	22.75	18	36	633	69.2	7.58
Q9BX51	Gamma-glutamyltransferase light chain 1 OS=Homo sapiens GN=GGTLC1 PE=2 SV=2 - [GGTL1_HUMAN]	5.33	1	1	1	1	1.199	0.490	0.784	1.048	3.05	5.33	1	1	225	24.3	5.16

Q9P0I2	ER membrane protein complex subunit 3 OS=Homo sapiens GN=EMC3 PE=1 SV=3 - [EMC3_HUMAN]	31.03	1	6	6	14	1.276	0.910	0.808	1.048	43.34	31.03	9	14	261	29.9	6.81
Q6IN85	Serine/threonine-protein phosphatase 4 regulatory subunit 3A OS=Homo sapiens GN=SMEK1 PE=1 SV=1 - [P4R3A_HUMAN]	7.32	2	6	6	15	0.948	1.218	0.925	1.048	34.58	7.32	9	15	833	95.3	4.94
O60318	Germinal-center associated nuclear protein OS=Homo sapiens GN=MCM3AP PE=1 SV=2 - [GANP_HUMAN]	5.15	1	6	7	11	1.081	0.983	1.240	1.048	23.94	5.15	9	11	1980	218.3	6.39
O15042	U2 snRNP-associated SURP motif-containing protein OS=Homo sapiens GN=U2SURP PE=1 SV=2 - [SR140_HUMAN]	11.56	1	11	11	34	0.945	1.144	0.842	1.048	73.84	11.56	15	34	1029	118.2	8.47
Q15119	[Pyruvate dehydrogenase (acetyl-transferring)] kinase isozyme 2, mitochondrial OS=Homo sapiens GN=PDK2 PE=1 SV=2 - [PDK2_HUMAN]	4.42	1	2	2	3	0.955	1.519	1.136	1.048	4.99	4.42	3	3	407	46.1	6.61
Q96QZ7	Membrane-associated guanylate kinase, WW and PDZ domain-containing protein 1 OS=Homo sapiens GN=MAGI1 PE=1 SV=3 - [MAGI1_HUMAN	4.09	1	4	4	6	1.059	0.937	1.166	1.049	13.12	4.09	6	6	1491	164.5	7.58
P26196	Probable ATP-dependent RNA helicase DDX6 OS=Homo sapiens GN=DDX6 PE=1 SV=2 - [DDX6_HUMAN]	45.34	3	18	19	83	0.881	1.156	1.102	1.049	178.04	45.34	30	83	483	54.4	8.66

Q8NBF2	NHL repeat-containing protein 2 OS=Homo sapiens GN=NHLRC2 PE=1 SV=1 - [NHLC2_HUMAN]	8.68	1	5	5	24	1.276	1.228	1.078	1.049	64.65	8.68	8	24	726	79.4	5.55
Q6ZQX7	Uncharacterized protein C17orf97 OS=Homo sapiens GN=C17orf97 PE=2 SV=2 - [CQ097_HUMAN]	22.08	1	1	1	4	1.024	0.985	0.891	1.049	11.07	22.08	2	4	453	49.6	7.97
P61956	Small ubiquitin-related modifier 2 OS=Homo sapiens GN=SUMO2 PE=1 SV=3 - [SUMO2_HUMA	27.37	1	2	3	65	1.403	1.230	0.903	1.049	117.57	27.37	6	65	95	10.9	5.50
Q9NW61	Pleckstrin homology domain-containing family J member 1 OS=Homo sapiens GN=PLEKHJ1 PE=2 SV=1 - [PKHJ1_HUMAN	16.78	1	2	2	2	0.820	1.365	1.674	1.049	2.37	16.78	2	2	149	17.5	7.84
P01034	Cystatin-C OS=Homo sapiens GN=CST3 PE=1 SV=1 - [CYTC_HUMAN]	19.18	1	3	3	6	1.253	0.865	1.516	1.049	24.53	19.18	3	6	146	15.8	8.75
P43897	Elongation factor Ts, mitochondrial OS=Homo sapiens GN=TSFM PE=1 SV=2 - [EFTS_HUMAN]	29.54	1	7	7	19	0.888	1.085	0.956	1.049	44.61	29.54	10	19	325	35.4	8.38
Q00325	Phosphate carrier protein, mitochondrial OS=Homo sapiens GN=SLC25A3 PE=1 SV=2 - [MPCP_HUMAN]	25.69	1	14	14	153	1.076	1.002	0.881	1.049	304.71	25.69	24	153	362	40.1	9.38
P12694	2-oxoisovalerate dehydrogenase subunit alpha, mitochondrial OS=Homo sapiens GN=BCKDHA PE=1 SV=2 - [ODBA_HUMAN]	13.26	1	6	6	13	1.036	1.627	1.098	1.049	35.60	13.26	9	13	445	50.4	8.27

A3KMH1	von Willebrand factor A domain-containing protein 8 OS=Homo sapiens GN=VWA8 PE=1 SV=2 - [VWA8_HUMAN	13.44	1	22	22	42	0.974	1.258	1.025	1.049	95.55	13.44	31	42	1905	214.7	7.40
Q92934	Bcl2-associated agonist of cell death OS=Homo sapiens GN=BAD PE=1 SV=3 - [BAD_HUMAN]	9.52	1	1	1	6	1.142	1.517	1.146	1.049	15.33	9.52	2	6	168	18.4	7.15
Q9HC36	rRNA methyltransferase 3, mitochondrial OS=Homo sapiens GN=RNMTL1 PE=1 SV=2 - [MRM3_HUMAN]	4.52	1	1	2	3	0.854	1.352	1.184	1.049	5.60	4.52	3	3	420	47.0	8.73
Q9UNX3	60S ribosomal protein L26-like 1 OS=Homo sapiens GN=RPL26L1 PE=1 SV=1 - [RL26L_HUMAN]	48.28	2	1	12	93	0.823	0.967	0.672	1.049	172.12	48.28	20	93	145	17.2	10.55
Q8NAV1	Pre-mRNA-splicing factor 38A OS=Homo sapiens GN=PRPF38A PE=1 SV=1 - [PR38A_HUMAN]	7.05	1	2	2	9	0.572	1.186	1.041	1.049	30.65	7.05	3	9	312	37.5	9.96
P51452	Dual specificity protein phosphatase 3 OS=Homo sapiens GN=DUSP3 PE=1 SV=1 - [DUS3_HUMAN]	41.62	1	6	6	20	1.299	1.005	1.187	1.050	64.01	41.62	10	20	185	20.5	7.80
O15294	UDP-N-acetylglucosamine--peptide N-acetylglucosaminyltransferase 110 kDa subunit OS=Homo sapiens GN=OGT PE=1 SV=3 - [OGT1_HUMAN]	17.50	1	15	15	46	0.767	1.097	1.041	1.050	130.01	17.50	22	46	1046	116.8	6.70
Q9Y6E0	Serine/threonine-protein kinase 24 OS=Homo sapiens GN=STK24 PE=1 SV=1 - [STK24_HUMAN	30.02	1	6	12	25	1.049	0.790	0.746	1.050	63.30	30.02	17	25	443	49.3	5.69

Q96JB5	CDK5 regulatory subunit-associated protein 3 OS=Homo sapiens GN=CDK5RAP3 PE=1 SV=2 - [CK5P3_HUMAN]	33.60	3	17	18	67	0.904	0.856	0.767	1.050	173.88	33.60	33	67	506	56.9	4.75
P43487	Ran-specific GTPase-activating protein OS=Homo sapiens GN=RANBP1 PE=1 SV=1 - [RANG_HUMAN]	33.33	2	6	6	58	1.005	1.031	0.943	1.050	121.45	33.33	10	58	201	23.3	5.29
Q07021	Complement component 1 Q subcomponent-binding protein, mitochondrial OS=Homo sapiens GN=C1QBP PE=1 SV=1 -	51.42	1	8	8	87	0.932	1.024	0.617	1.050	307.14	51.42	12	87	282	31.3	4.84
Q99627	COP9 signalosome complex subunit 8 OS=Homo sapiens GN=COPS8 PE=1 SV=1 - [CSN8_HUMAN]	33.49	1	5	5	29	0.875	1.089	1.041	1.050	85.99	33.49	8	29	209	23.2	5.38
Q96ND0	Protein FAM210A OS=Homo sapiens GN=FAM210A PE=1 SV=2 - [F210A_HUMAN]	2.21	1	1	1	1	1.050	1.112	0.934	1.050	2.16	2.21	1	1	272	30.8	9.76
Q5QP82	DDB1- and CUL4-associated factor 10 OS=Homo sapiens GN=DCAF10 PE=1 SV=1 - [DCA10_HUMAN	8.41	1	2	2	3	0.899	0.647	0.700	1.051	7.91	8.41	2	3	559	60.5	7.50
Q9P253	Vacuolar protein sorting-associated protein 18 homolog OS=Homo sapiens GN=VPS18 PE=1 SV=2 - [VPS18_HUMAN]	13.26	1	13	13	34	0.927	1.161	1.163	1.051	72.13	13.26	20	34	973	110.1	6.07
Q9UHQ4	B-cell receptor-associated protein 29 OS=Homo sapiens GN=BCAP29 PE=1 SV=2 - [BAP29_HUMAN]	32.78	1	9	9	48	1.104	1.249	0.856	1.051	118.10	32.78	14	48	241	28.3	9.54

O76003	Glutaredoxin-3 OS=Homo sapiens GN=GLRX3 PE=1 SV=2 - [GLRX3_HUMAN]	39.10	1	12	12	44	1.094	0.907	1.053	1.051	130.92	39.10	19	44	335	37.4	5.39
A6NF36	Coiled-coil domain-containing protein 182 OS=Homo sapiens GN=CCDC182 PE=2 SV=1 - [CC182_HUMAN]	16.99	1	1	2	20			1.223	1.051	41.67	16.99	2	20	153	17.5	6.58
P27658	Collagen alpha-1(VIII) chain OS=Homo sapiens GN=COL8A1 PE=1 SV=2 - [CO8A1_HUMAN]	4.17	1	2	3	4	1.546	0.661	0.627	1.051	5.07	4.17	4	4	744	73.3	9.61
P16930	Fumarylacetoacetase OS=Homo sapiens GN=FAH PE=1 SV=2 - [FAAA_HUMAN]	23.87	1	9	9	35	0.995	0.951	0.957	1.051	75.88	23.87	16	35	419	46.3	6.95
Q9H857	5'-nucleotidase domain-containing protein 2 OS=Homo sapiens GN=NT5DC2 PE=1 SV=1 - [NT5D2_HUMAN]	9.04	1	5	5	15	0.493	1.225	0.989	1.051	34.36	9.04	10	15	520	60.7	6.77
Q9HA64	Ketosamine-3-kinase OS=Homo sapiens GN=FN3KRP PE=1 SV=2 - [KT3K_HUMAN]	18.12	1	6	6	18	1.280	1.267	1.040	1.051	42.95	18.12	11	18	309	34.4	7.33
Q9Y2E4	Disco-interacting protein 2 homolog C OS=Homo sapiens GN=DIP2C PE=2 SV=2 - [DIP2C_HUMAN]	3.47	1	3	5	11	1.177	1.107	1.303	1.051	24.19	3.47	5	11	1556	170.7	7.39
Q9UBB5	Methyl-CpG-binding domain protein 2 OS=Homo sapiens GN=MBD2 PE=1 SV=1 - [MBD2_HUMAN]	15.57	1	4	5	9	1.221	0.737	0.750	1.051	16.90	15.57	8	9	411	43.2	10.04
Q96C90	Protein phosphatase 1 regulatory subunit 14B OS=Homo sapiens GN=PPP1R14B PE=1 SV=3 - [PP14B_HUMAN]	30.61	1	3	3	8	0.774	1.102	1.528	1.052	22.79	30.61	5	8	147	15.9	4.86

Q13033	Striatin-3 OS=Homo sapiens GN=STRN3 PE=1 SV=3 - [STRN3_HUMAN]	20.08	1	9	13	37	0.999	1.073	1.207	1.052	71.89	20.08	20	37	797	87.2	5.36
P00813	Adenosine deaminase OS=Homo sapiens GN=ADA PE=1 SV=3 - [ADA_HUMAN]	14.60	1	5	6	12	0.720	0.627	0.972	1.052	24.16	14.60	8	12	363	40.7	5.95
P46776	60S ribosomal protein L27a OS=Homo sapiens GN=RPL27A PE=1 SV=2 - [RL27A_HUMAN]	26.35	1	6	6	49	0.823	0.804	0.820	1.052	82.94	26.35	10	49	148	16.6	11.00
Q15642	Cdc42-interacting protein 4 OS=Homo sapiens GN=TRIP10 PE=1 SV=3 - [CIP4_HUMAN]	42.93	1	24	25	91	1.076	1.080	1.226	1.052	212.24	42.93	41	91	601	68.3	5.73
Q8NBJ7	Sulfatase-modifying factor 2 OS=Homo sapiens GN=SUMF2 PE=1 SV=2 - [SUMF2_HUMAN]	39.53	1	12	12	55	1.220	0.955	0.913	1.052	138.85	39.53	20	55	301	33.8	8.00
Q9BPX3	Condensin complex subunit 3 OS=Homo sapiens GN=NCAPG PE=1 SV=1 - [CND3_HUMAN]	3.55	1	3	3	5	0.821	1.139	0.758	1.052	11.35	3.55	3	5	1015	114.3	5.59
P62993	Growth factor receptor-bound protein 2 OS=Homo sapiens GN=GRB2 PE=1 SV=1 - [GRB2_HUMAN]	42.86	1	10	10	43	1.138	1.098	1.246	1.052	114.20	42.86	18	43	217	25.2	6.32
Q16878	Cysteine dioxygenase type 1 OS=Homo sapiens GN=CDO1 PE=1 SV=2 - [CDO1_HUMAN]	7.00	1	1	2	14	1.073	0.171	1.197	1.052	18.48	7.00	3	14	200	23.0	6.60
P51816	AF4/FMR2 family member 2 OS=Homo sapiens GN=AFF2 PE=1 SV=4 - [AFF2_HUMAN]	1.45	1	1	2	2	0.682	0.816	0.713	1.052	2.58	1.45	2	2	1311	144.7	8.05

Q9BPX5	Actin-related protein 2/3 complex subunit 5-like protein OS=Homo sapiens GN=ARPC5L PE=1 SV=1 - [ARP5L_HUMAN]	34.64	1	5	6	39	0.975	1.101	1.073	1.052	72.66	34.64	9	39	153	16.9	6.60
Q9NQC8	Intraflagellar transport protein 46 homolog OS=Homo sapiens GN=IFT46 PE=1 SV=1 - [IFT46_HUMAN]	3.62	1	1	1	2	1.139	1.788	1.464	1.052	3.80	3.62	2	2	304	34.3	4.54
Q99807	Ubiquinone biosynthesis protein COQ7 homolog OS=Homo sapiens GN=COQ7 PE=1 SV=3 - [COQ7_HUMAN]	4.15	1	1	1	1	1.337	1.364	1.370	1.053	1.68	4.15	1	1	217	24.3	8.59
C9J6K1	Putative uncharacterized protein C19orf81 OS=Homo sapiens GN=C19orf81 PE=4 SV=1 - [CS081_HUMAN]	9.09	1	1	1	2	0.923	1.365	0.472	1.053	6.38	9.09	1	2	198	22.4	7.40
P46109	Crk-like protein OS=Homo sapiens GN=CRKL PE=1 SV=1 - [CRKL_HUMAN]	41.91	1	9	9	24	1.020	1.546	1.072	1.053	54.03	41.91	14	24	303	33.8	6.74
Q53FA7	Quinone oxidoreductase PIG3 OS=Homo sapiens GN=TP53I3 PE=1 SV=2 - [QORX_HUMAN]	19.88	1	6	6	26	1.547	1.196	1.376	1.053	47.91	19.88	10	26	332	35.5	7.17
Q5T2W1	Na(+)/H(+) exchange regulatory cofactor NHE-RF3 OS=Homo sapiens GN=PDZK1 PE=1 SV=2 - [NHRF3_HUMAN]	2.70	1	1	1	1	1.160	0.775	1.652	1.053	0.00	2.70	1	1	519	57.1	5.55
Q15075	Early endosome antigen 1 OS=Homo sapiens GN=EEA1 PE=1 SV=2 - [EEA1_HUMAN]	55.92	5	85	88	382	0.860	0.844	0.779	1.053	884.29	55.92	139	382	1411	162.4	5.68

Q9NZT2	Opioid growth factor receptor OS=Homo sapiens GN=OGFR PE=1 SV=3 - [OGFR_HUMAN]	15.95	1	9	10	26	1.378	1.047	1.143	1.053	70.59	15.95	12	26	677	73.3	4.84
Q86V48	Leucine zipper protein 1 OS=Homo sapiens GN=LUZP1 PE=1 SV=2 - [LUZP1_HUMAN]	33.18	1	31	33	114	0.909	0.826	1.144	1.053	261.76	33.18	54	114	1076	120.2	8.50
Q9C0E2	Exportin-4 OS=Homo sapiens GN=XPO4 PE=1 SV=2 - [XPO4_HUMAN]	4.43	1	4	4	6	1.002	0.785	0.804	1.053	9.31	4.43	6	6	1151	130.1	5.05
O95169	NADH dehydrogenase [ubiquinone] 1 beta subcomplex subunit 8, mitochondrial OS=Homo sapiens GN=NDUFB8 PE=1 SV=1 - [NDUB8_HUMAN]	19.89	1	4	4	7	0.687	1.024	0.934	1.053	13.75	19.89	5	7	186	21.8	6.80
Q9UPU9	Protein Smaug homolog 1 OS=Homo sapiens GN=SAMD4A PE=1 SV=3 - [SMAG1_HUMA N]	8.22	1	5	5	10	1.070	0.824	1.054	1.053	27.77	8.22	8	10	718	79.4	8.32
Q9BUB5	MAP kinase-interacting serine/threonin e-protein kinase 1 OS=Homo sapiens GN=MKNK1 PE=1 SV=1 - [MKNK1_HUMA	4.95	1	2	2	3				1.053	6.03	4.95	3	3	465	51.3	6.68
Q92567	Protein FAM168A OS=Homo sapiens GN=FAM168A PE=1 SV=2 - [F168A_HUMAN]	7.38	1	2	2	7	0.910	0.960	1.009	1.053	11.73	7.38	4	7	244	26.2	8.98
Q9NPH2	Inositol-3-phosphate synthase 1 OS=Homo sapiens GN=ISYNA1 PE=1 SV=1 - [INO1_HUMAN]	8.78	1	4	4	8	1.635	1.942	2.919	1.053	21.04	8.78	5	8	558	61.0	5.76
P11586	C-1-tetrahydrofolat e synthase, cytoplasmic OS=Homo sapiens GN=MTHFD1 PE=1 SV=3 - [C1TC_HUMAN]	42.57	1	39	40	192	1.096	1.176	0.901	1.054	479.55	42.57	64	192	935	101.5	7.30

Q9Y3A5	Ribosome maturation protein SBDS OS=Homo sapiens GN=SBDS PE=1 SV=4 - [SBDS_HUMAN]	44.00	1	13	13	42	0.976	0.784	1.052	1.054	99.44	44.00	20	42	250	28.7	8.75
Q27J81	Inverted formin-2 OS=Homo sapiens GN=INF2 PE=1 SV=2 - [INF2_HUMAN]	29.22	1	27	27	98	1.264	1.012	1.479	1.054	238.60	29.22	41	98	1249	135.5	5.38
Q8IY17	Neuropathy target esterase OS=Homo sapiens GN=PNPLA6 PE=1 SV=2 - [PLPL6_HUMAN]	12.74	1	15	15	41	1.114	1.051	1.149	1.054	117.19	12.74	24	41	1366	149.9	7.81
P07858	Cathepsin B OS=Homo sapiens GN=CTSB PE=1 SV=3 - [CATB_HUMAN]	36.28	1	15	15	194	0.736	1.354	1.218	1.054	459.69	36.28	24	194	339	37.8	6.30
Q15528	Mediator of RNA polymerase II transcription subunit 22 OS=Homo sapiens GN=MED22 PE=1 SV=2 - [MED22_HUMAN]	10.00	1	2	2	8	0.756	1.053	0.860	1.054	19.92	10.00	3	8	200	22.2	4.68
Q8NFJ9	Bardet-Biedl syndrome 1 protein OS=Homo sapiens GN=BBS1 PE=1 SV=1 - [BBS1_HUMAN]	3.71	1	2	2	5	1.248	1.257	1.845	1.054	10.72	3.71	3	5	593	65.0	7.93
Q8NC60	Nitric oxide-associated protein 1 OS=Homo sapiens GN=NOA1 PE=1 SV=2 - [NOA1_HUMAN]	4.15	1	2	2	2	1.073	1.207	1.208	1.054	5.44	4.15	2	2	698	78.4	8.66
Q9UPY8	Microtubule-associated protein RP/EB family member 3 OS=Homo sapiens GN=MAPRE3 PE=1 SV=1 - [MARE3_HUMAN]	29.54	1	7	10	24	0.706	0.719	0.966	1.054	54.18	29.54	14	24	281	32.0	5.54
Q99797	Mitochondrial intermediate peptidase OS=Homo sapiens GN=MIPEP PE=1 SV=2 - [MIPEP_HUMAN]	18.51	1	10	11	25	0.822	1.191	1.018	1.054	47.05	18.51	16	25	713	80.6	7.05

P23919	Thymidylate kinase OS=Homo sapiens GN=DTYMK PE=1 SV=4 - [KTHY_HUMAN]	57.08	1	14	14	76	1.244	1.259	0.871	1.054	186.37	57.08	22	76	212	23.8	8.27
O43182	Rho GTPase-activating protein 6 OS=Homo sapiens GN=ARHGAP6 PE=1 SV=3 - [RHG06_HUMAN]	2.87	1	2	2	2	0.942	1.199	1.721	1.054	0.00	2.87	2	2	974	105.9	7.36
A6NF01	Putative nuclear envelope pore membrane protein POM 121B OS=Homo sapiens GN=POM121B PE=5 SV=2 - [P121B_HUMAN	1.68	3	1	1	1	0.833	1.153	0.905	1.054	3.62	1.68	1	1	834	83.0	9.95
Q8TCS8	Polyribonucleotide nucleotidyltransferase 1, mitochondrial OS=Homo sapiens GN=PNPT1 PE=1 SV=2 - [PNPT1_HUMAN]	17.11	1	14	14	31	1.012	1.045	0.870	1.055	67.06	17.11	20	31	783	85.9	7.77
Q9Y5B8	Nucleoside diphosphate kinase 7 OS=Homo sapiens GN=NME7 PE=1 SV=1 - [NDK7_HUMAN]	13.83	1	4	4	6	0.709	1.305	1.086	1.055	16.16	13.83	5	6	376	42.5	6.47
Q96E39	RNA binding motif protein, X-linked-like-1 OS=Homo sapiens GN=RBMXL1 PE=1 SV=1 - [RMXL1_HUMAN]	25.13	2	4	11	51	0.878	1.414	0.904	1.055	132.92	25.13	17	51	390	42.1	9.89
Q5JTV8	Torsin-1A-interacting protein 1 OS=Homo sapiens GN=TOR1AIP1 PE=1 SV=2 - [TOIP1_HUMAN]	44.08	1	18	19	72	0.944	1.142	0.956	1.055	176.83	44.08	33	72	583	66.2	8.18
P57678	Gem-associated protein 4 OS=Homo sapiens GN=GEMIN4 PE=1 SV=2 - [GEMI4_HUMAN]	5.86	1	5	7	21	0.947	0.903	1.125	1.055	39.60	5.86	12	21	1058	120.0	6.04

Q8N9U0	Tandem C2 domains nudear protein OS=Homo sapiens GN=TC2N PE=1 SV=2 - [TAC2N_HUMAN]	2.24	1	1	1	1	0.276	4.633	8.059	1.055	2.05	2.24	1	1	490	55.2	9.19
Q9H0Z9	RNA-binding protein 38 OS=Homo sapiens GN=RBM38 PE=1 SV=2 - [RBM38_HUMAN]	6.69	2	2	2	4	0.776	1.775	1.967	1.055	9.04	6.69	3	4	239	25.5	8.65
Q9HC07	Transmembrane protein 165 OS=Homo sapiens GN=TMEM165 PE=1 SV=1 - [TM165_HUMAN]	19.44	1	3	3	27	1.315	0.969	1.025	1.055	71.20	19.44	4	27	324	34.9	7.02
O15121	Sphingolipid delta(4)-desaturase DES1 OS=Homo sapiens GN=DEGS1 PE=1 SV=1 - [DEGS1_HUMAN]	17.96	1	3	4	14	1.683	1.004	1.194	1.055	41.73	17.96	6	14	323	37.8	7.46
Q9UN70	Protocadherin gamma-C3 OS=Homo sapiens GN=PCDHGC3 PE=1 SV=1 - [PCDGK_HUMAN]	4.50	9	3	4	6	1.107	1.703	1.235	1.055	13.09	4.50	5	6	934	101.0	5.21
Q9BUE0	Mediator of RNA polymerase II transcription subunit 18 OS=Homo sapiens GN=MED18 PE=1 SV=1 - [MED18_HUMAN]	11.54	1	2	2	4	0.988	0.965	0.946	1.055	4.82	11.54	3	4	208	23.6	6.54
Q96CU9	FAD-dependent oxidoreductase domain-containing protein 1 OS=Homo sapiens GN=FOXRED1 PE=1 SV=2 - [FXRD1_HUMAN]	4.32	1	2	2	7	1.266	1.213	0.946	1.055	14.42	4.32	4	7	486	53.8	7.78
Q15633	RISC-loading complex subunit TARBP2 OS=Homo sapiens GN=TARBP2 PE=1 SV=3 - [TRBP2_HUMAN]	4.92	1	1	2	4	0.936	0.820	1.113	1.055	6.69	4.92	3	4	366	39.0	6.54

Q9Y2L5	Trafficking protein partide complex subunit 8 OS=Homo sapiens GN=TRAPPC8 PE=1 SV=2 - [TPPC8_HUMAN]	5.64	1	8	8	16	1.256	1.236	1.027	1.056	32.92	5.64	13	16	1435	160.9	6.87
Q9HB55	Cytochrome P450 3A43 OS=Homo sapiens GN=CYP3A43 PE=1 SV=1 - [CP343_HUMAN]	1.39	1	1	1	1	1.056	1.140	1.037	1.056	2.10	1.39	1	1	503	57.6	8.10
Q8NFA2	NADPH oxidase organizer 1 OS=Homo sapiens GN=NOXO1 PE=1 SV=1 - [NOXO1_HUMAN]	1.86	1	1	1	1	0.747	1.401	1.491	1.056	2.50	1.86	1	1	376	41.2	9.83
Q8NB46	Serine/threonine-protein phosphatase 6 regulatory ankyrin repeat subunit C OS=Homo sapiens GN=ANKRD52 PE=1 SV=3 - [ANR52_HUMAN]	1.77	1	2	2	8	0.707	0.873	1.196	1.056	19.42	1.77	3	8	1076	115.0	6.48
Q08380	Galectin-3-binding protein OS=Homo sapiens GN=LGALS3BP PE=1 SV=1 - [LG3BP_HUMAN]	15.04	1	10	10	39	1.411	0.961	0.975	1.056	96.92	15.04	16	39	585	65.3	5.27
O15027	Protein transport protein Sec16A OS=Homo sapiens GN=SEC16A PE=1 SV=3 - [SC16A_HUMAN]	15.56	1	24	24	74	0.947	1.047	1.007	1.056	163.86	15.56	38	74	2179	233.4	5.63
P05023	Sodium/potassium-transporting ATPase subunit alpha-1 OS=Homo sapiens GN=ATP1A1 PE=1 SV=1 - [AT1A1_HUMAN]	45.06	4	39	48	384	1.570	0.839	0.722	1.056	993.96	45.06	81	384	1023	112.8	5.49
P51608	Methyl-CpG-binding protein 2 OS=Homo sapiens GN=MECP2 PE=1 SV=1 - [MECP2_HUMAN]	33.54	1	14	14	48	1.108	1.423	1.402	1.056	92.05	33.54	23	48	486	52.4	9.95

P62424	60S ribosomal protein L7a OS=Homo sapiens GN=RPL7A PE=1 SV=2 - [RL7A_HUMAN]	54.14	1	23	24	156	0.864	1.066	0.982	1.056	299.22	54.14	37	156	266	30.0	10.61
Q96GF1	E3 ubiquitin-protein ligase RNF185 OS=Homo sapiens GN=RNF185 PE=1 SV=1 - [RN185_HUMAN]	4.69	1	1	1	3	1.586	0.930	1.331	1.056	4.66	4.69	2	3	192	20.4	6.52
Q9P2C4	Transmembrane protein 181 OS=Homo sapiens GN=TMEM181 PE=1 SV=2 - [TM181_HUMAN]	2.61	1	1	1	4	1.350	1.065	1.030	1.056	18.29	2.61	2	4	612	69.3	8.92
Q9H061	Transmembrane protein 126A OS=Homo sapiens GN=TMEM126A PE=1 SV=1 - [T126A_HUMAN]	26.67	1	6	6	21	1.213	1.024	0.781	1.056	37.84	26.67	9	21	195	21.5	9.26
Q92888	Rho guanine nucleotide exchange factor 1 OS=Homo sapiens GN=ARHGEF1 PE=1 SV=2 - [ARHG1_HUMAN	33.00	1	24	25	76	1.122	0.960	0.943	1.057	204.95	33.00	37	76	912	102.4	5.66
Q96KG9	N-terminal kinase-like protein OS=Homo sapiens GN=SCYL1 PE=1 SV=1 - [NTKL_HUMAN]	25.87	1	15	15	39	0.987	1.015	1.040	1.057	91.54	25.87	22	39	808	89.6	6.30
O43251	RNA binding protein fox-1 homolog 2 OS=Homo sapiens GN=RBFOX2 PE=1 SV=3 - [RFOX2_HUMAN]	11.28	3	4	4	15	1.251	1.604	1.168	1.057	32.15	11.28	7	15	390	41.3	7.27
Q96BN8	Ubiquitin thioesterase otulin OS=Homo sapiens GN=OTULIN PE=1 SV=3 - [OTUL_HUMAN]	19.03	1	6	6	18	0.996	0.779	0.718	1.057	37.63	19.03	9	18	352	40.2	5.47
Q6ZUX3	Protein FAM179A OS=Homo sapiens GN=FAM179A PE=2 SV=2 - [F179A_HUMAN]	2.36	1	2	2	3	2.134	0.710	0.627	1.057	7.43	2.36	2	3	1019	111.1	9.42

O14490	Disks large-associated protein 1 OS=Homo sapiens GN=DLGAP1 PE=1 SV=1 - [DLGP1_HUMAN]	1.64	1	1	2	2	0.676	1.413	2.959	1.057	4.65	1.64	2	2	977	108.8	7.08
Q8NHZ8	Anaphase-promoting complex subunit CDC26 OS=Homo sapiens GN=CDC26 PE=1 SV=1 - [CDC26_HUMAN]	14.12	1	1	1	2	1.145	1.286	0.923	1.057	0.00	14.12	1	2	85	9.8	6.81
Q8TAF3	WD repeat-containing protein 48 OS=Homo sapiens GN=WDR48 PE=1 SV=1 - [WDR48_HUMAN]	13.15	1	7	8	21	1.441	1.079	1.049	1.057	51.03	13.15	11	21	677	76.2	7.03
O15446	DNA-directed RNA polymerase I subunit RPA34 OS=Homo sapiens GN=CD3EAP PE=1 SV=1 - [RPA34_HUMAN]	6.86	1	2	2	5	0.814	1.073	1.281	1.057	18.07	6.86	3	5	510	55.0	8.51
Q96RK4	Bardet-Biedl syndrome 4 protein OS=Homo sapiens GN=BBS4 PE=1 SV=2 - [BBS4_HUMAN]	3.08	2	2	2	4	1.180	1.045	1.262	1.057	7.49	3.08	3	4	519	58.2	7.31
Q9Y2Z4	Tyrosine--tRNA ligase, mitochondrial OS=Homo sapiens GN=YARS2 PE=1 SV=2 - [SYYM_HUMAN]	12.58	1	5	5	11	0.968	1.205	1.049	1.057	34.39	12.58	9	11	477	53.2	8.98
Q9NTG7	NAD-dependent protein deacetylase sirtuin-3, mitochondrial OS=Homo sapiens GN=SIRT3 PE=1 SV=2 - [SIR3_HUMAN]	11.53	1	3	3	5	1.729	2.208	1.321	1.057	5.13	11.53	4	5	399	43.5	8.70
P51665	26S proteasome non-ATPase regulatory subunit 7 OS=Homo sapiens GN=PSMD7 PE=1 SV=2 - [PSMD7_HUMA	41.67	1	11	11	51	1.036	1.243	1.186	1.057	140.72	41.67	20	51	324	37.0	6.77

O60725	Protein-S-isoprenylcysteine O-methyltransferase OS=Homo sapiens GN=ICMT PE=1 SV=1 - [ICMT_HUMAN]	3.17	1	1	1	1	1.285	0.730	0.673	1.058	0.00	3.17	1	1	284	31.9	7.96
Q96M27	Protein PRRC1 OS=Homo sapiens GN=PRRC1 PE=1 SV=1 - [PRRC1_HUMAN]	30.79	1	10	10	37	0.889	0.943	0.866	1.058	93.01	30.79	17	37	445	46.7	5.83
Q9C037	E3 ubiquitin-protein ligase TRIM4 OS=Homo sapiens GN=TRIM4 PE=1 SV=2 - [TRIM4_HUMAN]	20.00	1	11	11	27	0.853	0.957	0.919	1.058	55.95	20.00	17	27	500	57.4	8.10
O14559	Rho GTPase-activating protein 33 OS=Homo sapiens GN=ARHGAP33 PE=1 SV=2 - [RHG33_HUMAN]	1.63	1	1	1	1	1.010	1.049	0.599	1.058	3.49	1.63	1	1	1287	137.1	8.75
Q14157	Ubiquitin-associated protein 2-like OS=Homo sapiens GN=UBAP2L PE=1 SV=2 - [UBP2L_HUMAN]	21.71	1	19	19	135	1.008	1.097	1.003	1.058	373.67	21.71	27	135	1087	114.5	7.11
P25325	3-mercaptopyruvate sulfurtransferase OS=Homo sapiens GN=MPST PE=1 SV=3 - [THTM_HUMAN]	28.62	1	6	7	17	1.344	1.185	1.064	1.058	40.94	28.62	10	17	297	33.2	6.60
Q6IQ23	Pleckstrin homology domain-containing family A member 7 OS=Homo sapiens GN=PLEKHA7 PE=1 SV=2 - [PKHA7_HUMAN]	1.61	1	1	1	1	1.103	1.067	1.811	1.058	0.00	1.61	1	1	1121	127.1	9.35
P49674	Casein kinase I isoform epsilon OS=Homo sapiens GN=CSNK1E PE=1 SV=1 - [KC1E_HUMAN]	19.47	1	3	7	18	1.100	1.143	1.066	1.058	42.84	19.47	10	18	416	47.3	9.66
P10909	Clusterin OS=Homo sapiens GN=CLU PE=1 SV=1 - [CLUS_HUMAN]	18.04	1	7	7	23	0.521	1.527	2.540	1.058	63.81	18.04	10	23	449	52.5	6.27

Q8TBF2	Prostamide/prostaglandin F synthase OS=Homo sapiens GN=FAM213B PE=2 SV=1 - [PGFS_HUMAN]	9.60	1	2	2	5	0.966	1.265	1.322	1.059	18.26	9.60	3	5	198	21.2	6.67
Q9BSF4	Uncharacterized protein C19orf52 OS=Homo sapiens GN=C19orf52 PE=1 SV=2 - [CS052_HUMAN]	11.54	1	2	2	8	0.870	0.827	0.870	1.059	26.37	11.54	4	8	260	29.2	8.09
Q86XK2	F-box only protein 11 OS=Homo sapiens GN=FBXO11 PE=1 SV=3 - [FBX11_HUMAN]	1.08	1	1	1	3	1.237	0.899	1.219	1.059	2.37	1.08	1	3	927	103.5	6.99
Q8ND04	Protein SMG8 OS=Homo sapiens GN=SMG8 PE=1 SV=1 - [SMG8_HUMAN]	2.12	1	2	3	3	0.937	0.902	1.191	1.059	4.58	2.12	3	3	991	109.6	7.68
Q15006	ER membrane protein complex subunit 2 OS=Homo sapiens GN=EMC2 PE=1 SV=1 - [EMC2_HUMAN]	26.60	1	8	8	20	1.090	0.914	1.051	1.059	39.67	26.60	13	20	297	34.8	6.57
Q5JRA6	Melanoma inhibitory activity protein 3 OS=Homo sapiens GN=MIA3 PE=1 SV=1 - [MIA3_HUMAN]	19.72	1	35	36	132	0.841	0.994	0.932	1.059	304.86	19.72	54	132	1907	213.6	4.84
Q9P2E3	NFX1-type zinc finger-containing protein 1 OS=Homo sapiens GN=ZNFX1 PE=1 SV=2 - [ZNFX1_HUMAN]	4.54	1	7	7	29	0.834	0.940	1.243	1.059	53.19	4.54	9	29	1918	220.1	7.30
A2RU67	Uncharacterized protein KIAA1467 OS=Homo sapiens GN=KIAA1467 PE=1 SV=1 - [K1467_HUMAN]	3.22	1	2	2	3	1.102	0.461	0.376	1.059	10.62	3.22	2	3	622	67.0	5.02
Q8TF74	WAS/WASL-interacting protein family member 2 OS=Homo sapiens GN=WIPF2 PE=1 SV=1 - [WIPF2_HUMAN]	28.86	1	9	9	31	1.162	0.888	1.068	1.059	73.05	28.86	14	31	440	46.3	10.93

O43493	Trans-Golgi network integral membrane protein 2 OS=Homo sapiens GN=TGOLN2 PE=1 SV=2 - [TGON2_HUMA	29.17	1	12	12	50	0.864	0.739	0.924	1.059	109.72	29.17	17	50	480	51.1	5.73
O75396	Vesicle-trafficking protein SEC22b OS=Homo sapiens GN=SEC22B PE=1 SV=4 - [SC22B_HUMAN	44.19	1	9	9	185	0.891	0.848	0.925	1.059	423.15	44.19	16	185	215	24.6	6.92
P45985	Dual specificity mitogen-activated protein kinase kinase 4 OS=Homo sapiens GN=MAP2K4 PE=1 SV=1 - [MP2K4_HUMA	17.79	1	6	6	22	1.028	1.045	0.914	1.059	43.07	17.79	12	22	399	44.3	8.07
P08047	Transcription factor Sp1 OS=Homo sapiens GN=SP1 PE=1 SV=3 - [SP1_HUMAN]	3.57	1	2	2	3	1.591	0.769	1.014	1.060	1.92	3.57	2	3	785	80.6	7.34
Q9UIA9	Exportin-7 OS=Homo sapiens GN=XPO7 PE=1 SV=3 - [XPO7_HUMAN]	16.65	1	16	17	34	1.039	1.071	1.065	1.060	76.66	16.65	26	34	1087	123.8	6.32
Q9UJW9	SERTA domain-containing protein 3 OS=Homo sapiens GN=SERTAD3 PE=1 SV=2 - [SRTD3_HUMAN]	3.06	1	1	1	1	0.849	0.838	1.025	1.060	2.53	3.06	1	1	196	21.8	4.67
Q8IWW6	Rho GTPase-activating protein 12 OS=Homo sapiens GN=ARHGAP12 PE=1 SV=1 - [RHG12_HUMAN]	9.34	1	8	8	15	0.894	1.019	1.069	1.060	43.28	9.34	11	15	846	96.2	7.61
Q9ULB4	Cadherin-9 OS=Homo sapiens GN=CDH9 PE=2 SV=2 - [CADH9_HUMAN]	1.90	1	1	1	1	1.159	0.756	0.922	1.060	3.25	1.90	1	1	789	88.6	4.91
Q92844	TRAF family member-associated NF-kappa-B activator OS=Homo sapiens GN=TANK PE=1 SV=2 - [TANK_HUMAN]	8.24	1	4	4	6	1.327	1.019	1.038	1.060	13.90	8.24	6	6	425	47.8	5.68

Q01432	AMP deaminase 3 OS=Homo sapiens GN=AMPD3 PE=1 SV=1 - [AMPD3_HUMA	14.73	2	12	13	34	1.103	0.835	1.634	1.060	69.68	14.73	18	34	767	88.8	6.98
Q9BZP3	Putative uncharacterized protein encoded by LINC00470 OS=Homo sapiens GN=LINC00470 PE=5 SV=1 - [CR002_HUMAN	16.28	1	1	1	1	0.557			1.060	0.00	16.28	1	1	86	9.6	5.01
P17706	Tyrosine-protein phosphatase non-receptor type 2 OS=Homo sapiens GN=PTPN2 PE=1 SV=2 - [PTN2_HUMAN]	1.69	1	1	1	4	0.730	0.902	0.776	1.060	6.20	1.69	2	4	415	48.4	8.29
Q14344	Guanine nucleotide-binding protein subunit alpha-13 OS=Homo sapiens GN=GNA13 PE=1 SV=2 - [GNA13_HUMAN]	35.28	1	11	13	56	1.375	1.040	0.788	1.060	153.45	35.28	24	56	377	44.0	8.00
Q9NSE4	Isoleucine--tRNA ligase, mitochondrial OS=Homo sapiens GN=IARS2 PE=1 SV=2 - [SYIM_HUMAN]	29.94	1	28	28	101	1.002	1.142	1.002	1.060	269.23	29.94	43	101	1012	113.7	7.20
Q5FWF4	DNA annealing helicase and endonuclease ZRANB3 OS=Homo sapiens GN=ZRANB3 PE=1 SV=2 - [ZRAB3_HUMAN]	0.56	1	1	1	1	0.877	1.166	1.558	1.060	1.75	0.56	1	1	1079	123.2	8.44
P61088	Ubiquitin-conjugating enzyme E2 N OS=Homo sapiens GN=UBE2N PE=1 SV=1 - [UBE2N_HUMAN]	65.79	2	10	10	90	1.114	0.992	1.024	1.060	217.96	65.79	16	90	152	17.1	6.57
Q8IZH2	5'-3' exoribonuclease 1 OS=Homo sapiens GN=XRN1 PE=1 SV=1 - [XRN1_HUMAN]	9.03	1	14	14	41	1.031	1.123	0.944	1.060	103.44	9.03	23	41	1706	194.0	7.21

Q96CN4	EVI5-like protein OS=Homo sapiens GN=EVI5L PE=1 SV=1 - [EVI5L_HUMAN]	3.53	1	3	3	13	0.921	1.139	1.327	1.061	18.49	3.53	6	13	794	91.3	5.34
Q00796	Sorbitol dehydrogenase OS=Homo sapiens GN=SORD PE=1 SV=4 - [DHSO_HUMAN]	25.77	1	9	9	33	1.062	0.868	0.837	1.061	72.58	25.77	15	33	357	38.3	7.97
Q9Y3I1	F-box only protein 7 OS=Homo sapiens GN=FBXO7 PE=1 SV=1 - [FBX7_HUMAN]	5.36	1	2	2	2	0.219	0.196	0.751	1.061	2.82	5.36	2	2	522	58.5	6.55
Q6PHR2	Serine/threonine-protein kinase ULK3 OS=Homo sapiens GN=ULK3 PE=1 SV=2 - [ULK3_HUMAN]	4.45	1	2	2	5	0.897	0.156	0.638	1.061	3.08	4.45	3	5	472	53.4	7.36
Q15334	Lethal(2) giant larvae protein homolog 1 OS=Homo sapiens GN=LLGL1 PE=1 SV=3 - [L2GL1_HUMAN]	11.56	1	10	10	21	1.299	1.080	1.323	1.061	51.19	11.56	16	21	1064	115.3	6.29
Q13501	Sequestosome-1 OS=Homo sapiens GN=SQSTM1 PE=1 SV=1 - [SQSTM_HUMAN]	23.41	1	8	8	28	0.758	0.766	0.962	1.061	77.91	23.41	12	28	440	47.7	5.22
O15270	Serine palmitoyltransferase 2 OS=Homo sapiens GN=SPTLC2 PE=1 SV=1 - [SPTC2_HUMAN]	10.32	1	5	5	12	0.940	0.803	1.104	1.061	24.34	10.32	8	12	562	62.9	7.78
P07942	Laminin subunit beta-1 OS=Homo sapiens GN=LAMB1 PE=1 SV=2 - [LAMB1_HUMAN]	27.10	1	47	48	126	1.024	1.004	0.841	1.061	314.74	27.10	66	126	1786	197.9	4.94
P30042	ES1 protein homolog, mitochondrial OS=Homo sapiens GN=C21orf33 PE=1 SV=3 - [ES1_HUMAN]	37.69	1	9	9	42	0.991	1.392	1.029	1.061	124.10	37.69	15	42	268	28.2	8.27

Q86VH4	Leucine-rich repeat transmembrane neuronal protein 4 OS=Homo sapiens GN=LRRTM4 PE=2 SV=2 - [LRRT4_HUMAN]	1.02	1	1	1	1	0.761	0.857	1.053	1.061	0.00	1.02	1	1	590	67.2	8.95
Q9H7D7	WD repeat-containing protein 26 OS=Homo sapiens GN=WDR26 PE=1 SV=3 - [WDR26_HUMAN]	5.90	1	3	3	7	1.085	0.716	1.126	1.061	19.07	5.90	6	7	661	72.1	6.16
Q96B45	UPF0693 protein C10orf32 OS=Homo sapiens GN=C10orf32 PE=1 SV=1 - [CJ032_HUMAN]	28.57	1	2	2	9	1.273	1.159	1.130	1.062	25.97	28.57	4	9	105	11.6	6.79
Q9BYI3	Hyccin OS=Homo sapiens GN=FAM126A PE=1 SV=2 - [HYCCI_HUMAN]	10.17	1	5	5	19	0.972	0.978	1.041	1.062	45.81	10.17	8	19	521	57.6	8.16
P04080	Cystatin-B OS=Homo sapiens GN=CSTB PE=1 SV=2 - [CYTB_HUMAN]	61.22	1	5	5	53	1.459	0.979	0.872	1.062	119.31	61.22	9	53	98	11.1	7.56
Q96S82	Ubiquitin-like protein 7 OS=Homo sapiens GN=UBL7 PE=1 SV=2 - [UBL7_HUMAN]	8.68	1	2	3	15	0.703	1.117	1.363	1.062	32.55	8.68	5	15	380	40.5	5.07
P11177	Pyruvate dehydrogenase E1 component subunit beta, mitochondrial OS=Homo sapiens GN=PDHB PE=1 SV=3 - [ODPB_HUMAN]	34.54	1	10	10	79	0.985	1.304	1.173	1.062	190.51	34.54	18	79	359	39.2	6.65
Q9Y6M1	Insulin-like growth factor 2 mRNA-binding protein 2 OS=Homo sapiens GN=IGF2BP2 PE=1 SV=2 - [IF2B2_HUMAN]	33.56	1	15	17	68	0.998	0.811	0.845	1.062	176.37	33.56	27	68	599	66.1	8.46
Q96N66	Lysophospholipid acyltransferase 7 OS=Homo sapiens GN=MBOAT7 PE=1 SV=2 - [MBOA7_HUMAN]	8.26	1	5	5	22	0.964	0.980	0.898	1.062	52.75	8.26	8	22	472	52.7	8.97

O75420	PERQ amino acid-rich with GYF domain-containing protein 1 OS=Homo sapiens GN=GIGYF1 PE=1 SV=2 - [PERQ1_HUMAN]	3.29	1	3	3	15	1.022	0.993	0.995	1.062	46.81	3.29	5	15	1035	114.5	5.39
Q9Y343	Sorting nexin-24 OS=Homo sapiens GN=SNX24 PE=1 SV=1 - [SNX24_HUMAN]	4.14	1	1	1	3	1.341	1.040	0.997	1.062	6.11	4.14	2	3	169	19.8	7.24
Q9H1K0	Rabenosyn-5 OS=Homo sapiens GN=ZFYVE20 PE=1 SV=2 - [RBNS5_HUMAN]	7.27	1	6	6	10	1.181	0.988	1.219	1.062	22.16	7.27	8	10	784	88.8	5.50
P02656	Apolipoprotein C-III OS=Homo sapiens GN=APOC3 PE=1 SV=1 - [APOC3_HUMAN]	16.16	1	1	1	4	0.952	0.536	0.846	1.062	9.51	16.16	2	4	99	10.8	5.41
O75886	Signal transducing adapter molecule 2 OS=Homo sapiens GN=STAM2 PE=1 SV=1 - [STAM2_HUMA	12.57	1	5	7	18	0.980	1.066	1.134	1.063	39.16	12.57	12	18	525	58.1	5.07
O43396	Thioredoxin-like protein 1 OS=Homo sapiens GN=TXNL1 PE=1 SV=3 - [TXNL1_HUMAN]	49.83	1	10	10	60	1.262	1.066	1.169	1.063	166.38	49.83	16	60	289	32.2	4.96
Q8IUR7	Armadillo repeat-containing protein 8 OS=Homo sapiens GN=ARMC8 PE=1 SV=2 - [ARMC8_HUMA	13.22	1	7	7	17	1.028	1.123	1.097	1.063	47.30	13.22	11	17	673	75.5	6.73
P40939	Trifunctional enzyme subunit alpha, mitochondrial OS=Homo sapiens GN=HADHA PE=1 SV=2 - [ECHA_HUMAN]	53.21	1	44	45	309	0.965	1.033	1.023	1.063	768.80	53.21	71	309	763	82.9	9.04
Q9NZ45	CDGSH iron-sulfur domain-containing protein 1 OS=Homo sapiens GN=CISD1 PE=1 SV=1 - [CISD1_HUMAN]	43.52	1	4	4	18	0.885	1.065	0.748	1.063	56.88	43.52	6	18	108	12.2	9.09

Q96DM3	Uncharacterized protein C18orf8 OS=Homo sapiens GN=C18orf8 PE=2 SV=2 - [MIC1_HUMAN]	5.18	1	3	3	7	1.150	1.106	1.049	1.063	17.75	5.18	5	7	657	74.9	7.83
Q5SSJ5	Heterochromatin protein 1-binding protein 3 OS=Homo sapiens GN=HP1BP3 PE=1 SV=1 - [HP1B3_HUMAN]	34.90	1	23	23	148	1.350	1.099	0.816	1.063	309.76	34.90	39	148	553	61.2	9.67
Q13395	Probable methyltransferase TARBP1 OS=Homo sapiens GN=TARBP1 PE=1 SV=1 - [TARB1_HUMAN]	1.36	1	2	3	6	0.956	1.003	1.300	1.064	4.53	1.36	3	6	1621	181.6	7.05
Q99497	Protein DJ-1 OS=Homo sapiens GN=PARK7 PE=1 SV=2 - [PARK7_HUMAN]	81.48	1	20	20	272	1.078	1.046	0.997	1.064	690.38	81.48	34	272	189	19.9	6.79
Q6NXT1	Ankyrin repeat domain-containing protein 54 OS=Homo sapiens GN=ANKRD54 PE=1 SV=2 - [ANR54_HUMAN]	4.33	1	2	2	3	1.016	1.068	1.033	1.064	4.49	4.33	3	3	300	32.5	6.28
P52895	Aldo-keto reductase family 1 member C2 OS=Homo sapiens GN=AKR1C2 PE=1 SV=3 - [AK1C2_HUMAN	54.49	1	2	14	171	1.784	0.799	0.641	1.064	404.98	54.49	20	171	323	36.7	7.49
O43491	Band 4.1-like protein 2 OS=Homo sapiens GN=EPB41L2 PE=1 SV=1 - [E41L2_HUMAN]	56.42	1	51	55	301	2.250	0.734	0.810	1.064	695.73	56.42	89	301	1005	112.5	5.44
O15269	Serine palmitoyltransferase 1 OS=Homo sapiens GN=SPTLC1 PE=1 SV=1 - [SPTC1_HUMAN]	31.50	1	12	12	36	0.975	0.971	0.796	1.064	111.30	31.50	19	36	473	52.7	6.01
P50548	ETS domain-containing transcription factor ERF OS=Homo sapiens GN=ERF PE=1 SV=2 - [ERF_HUMAN]	6.39	1	3	3	4	1.124	1.087	1.020	1.064	7.08	6.39	4	4	548	58.7	7.30

Q99700	Ataxin-2 OS=Homo sapiens GN=ATXN2 PE=1 SV=2 - [ATX2_HUMAN]	15.99	1	16	19	65	1.026	1.083	1.047	1.064	173.41	15.99	30	65	1313	140.2	9.57
Q6NXR0	Interferon-inducible GTPase 5 OS=Homo sapiens GN=IRGC PE=2 SV=1 - [IIGP5_HUMAN]	9.50	2	3	4	5	0.719	1.066	1.536	1.064	6.58	9.50	4	5	463	50.3	5.35
A6NHZ5	Leucine-rich repeat-containing protein 14B OS=Homo sapiens GN=LRRC14B PE=3 SV=3 - [LR14B_HUMAN]	1.56	1	1	1	1	1.208	1.100	1.123	1.065	0.00	1.56	1	1	514	56.7	7.17
Q9H6K1	Uncharacterized protein C6orf106 OS=Homo sapiens GN=C6orf106 PE=1 SV=2 - [CF106_HUMAN]	4.70	1	1	1	2	1.069	1.927	5.531	1.065	0.00	4.70	1	2	298	32.9	4.58
Q9Y6C2	EMILIN-1 OS=Homo sapiens GN=EMILIN1 PE=1 SV=2 - [EMIL1_HUMAN]	14.67	1	12	13	35	0.593	1.447	1.168	1.065	85.72	14.67	21	35	1016	106.6	5.15
Q92820	Gamma-glutamyl hydrolase OS=Homo sapiens GN=GGH PE=1 SV=2 - [GGH_HUMAN]	27.67	1	8	8	36	1.194	1.570	0.927	1.065	96.28	27.67	11	36	318	35.9	7.11
Q2M389	WASH complex subunit 7 OS=Homo sapiens GN=KIAA1033 PE=1 SV=2 - [WASH7_HUMAN]	13.73	1	14	14	40	1.178	0.985	1.049	1.065	88.96	13.73	24	40	1173	136.3	7.44
P53992	Protein transport protein Sec24C OS=Homo sapiens GN=SEC24C PE=1 SV=3 - [SC24C_HUMAN]	20.75	1	20	22	80	1.148	0.842	1.120	1.065	200.93	20.75	31	80	1094	118.2	7.06
Q96IU4	Alpha/beta hydrolase domain-containing protein 14B OS=Homo sapiens GN=ABHD14B PE=1 SV=1 - [ABHEB_HUMAN]	34.76	1	5	5	39	0.987	0.687	0.750	1.066	94.65	34.76	10	39	210	22.3	6.40

Q6YP21	Kynurenine--oxoglutarate transaminase 3 OS=Homo sapiens GN=CCBL2 PE=1 SV=1 - [KAT3_HUMAN]	28.85	2	12	12	28	0.880	0.829	0.814	1.066	58.44	28.85	17	28	454	51.4	8.19
Q9Y265	RuvB-like 1 OS=Homo sapiens GN=RUVBL1 PE=1 SV=1 - [RUVB1_HUMAN]	45.83	1	18	18	57	1.060	1.208	1.083	1.066	140.84	45.83	29	57	456	50.2	6.42
Q96I24	Far upstream element-binding protein 3 OS=Homo sapiens GN=FUBP3 PE=1 SV=2 - [FUBP3_HUMAN]	44.93	1	19	20	92	0.900	1.063	0.958	1.066	211.89	44.93	33	92	572	61.6	8.38
Q9Y3B7	39S ribosomal protein L11, mitochondrial OS=Homo sapiens GN=MRPL11 PE=1 SV=1 - [RM11_HUMAN]	50.00	1	9	9	37	0.962	1.225	1.028	1.066	97.77	50.00	13	37	192	20.7	9.91
Q9UIJ7	GTP:AMP phosphotransferase AK3, mitochondrial OS=Homo sapiens GN=AK3 PE=1 SV=4 - [KAD3_HUMAN]	67.40	1	17	17	94	1.347	1.056	1.113	1.066	203.86	67.40	29	94	227	25.5	9.16
Q9C0C4	Semaphorin-4C OS=Homo sapiens GN=SEMA4C PE=1 SV=2 - [SEM4C_HUMAN]	3.00	1	2	2	5	1.544	0.958	0.805	1.066	2.42	3.00	2	5	833	92.6	7.28
Q9UKK3	Poly [ADP-ribose] polymerase 4 OS=Homo sapiens GN=PARP4 PE=1 SV=3 - [PARP4_HUMAN]	19.90	1	32	32	94	1.115	1.053	1.206	1.066	222.16	19.90	49	94	1724	192.5	5.66
Q9NZ52	ADP-ribosylation factor-binding protein GGA3 OS=Homo sapiens GN=GGA3 PE=1 SV=1 - [GGA3_HUMAN]	4.29	1	3	3	5	0.813	1.026	1.018	1.066	8.30	4.29	5	5	723	78.3	5.58
Q15276	Rab GTPase-binding effector protein 1 OS=Homo sapiens GN=RABEP1 PE=1 SV=2 - [RABE1_HUMAN	29.81	2	22	24	64	0.860	1.015	1.045	1.066	174.95	29.81	32	64	862	99.2	5.01

P35914	Hydroxymethylglutaryl-CoA lyase, mitochondrial OS=Homo sapiens GN=HMGCL PE=1 SV=2 - [HMGCL_HUMAN]	36.31	2	11	12	40	0.911	1.257	1.069	1.066	99.27	36.31	18	40	325	34.3	8.54
P04150	Glucocorticoid receptor OS=Homo sapiens GN=NR3C1 PE=1 SV=1 - [GCR_HUMAN]	10.30	1	6	6	9	0.964	1.585	1.007	1.066	25.51	10.30	6	9	777	85.6	6.38
Q15435	Protein phosphatase 1 regulatory subunit 7 OS=Homo sapiens GN=PPP1R7 PE=1 SV=1 - [PP1R7_HUMAN]	52.78	1	20	20	76	1.087	0.905	1.035	1.066	205.93	52.78	31	76	360	41.5	4.91
Q5VSL9	Striatin-interacting protein 1 OS=Homo sapiens GN=STRIP1 PE=1 SV=1 - [STRP1_HUMAN]	11.11	1	6	7	13	1.112	0.789	0.970	1.067	24.30	11.11	9	13	837	95.5	6.29
Q01970	1-phosphatidylinositol 4,5-bisphosphate phosphodiesterase beta-3 OS=Homo sapiens GN=PLCB3 PE=1 SV=2 - [PLCB3_HUMAN]	17.91	3	19	23	104	0.966	1.052	1.154	1.067	235.54	17.91	35	104	1234	138.7	5.90
Q9Y2R9	28S ribosomal protein S7, mitochondrial OS=Homo sapiens GN=MRPS7 PE=1 SV=2 - [RT07_HUMAN]	34.30	1	6	7	34	0.801	1.067	1.216	1.067	108.11	34.30	11	34	242	28.1	9.99
Q53EU6	Glycerol-3-phosphate acyltransferase 3 OS=Homo sapiens GN=AGPAT9 PE=1 SV=2 - [GPAT3_HUMAN]	7.14	1	2	3	4	0.652	0.431	0.385	1.067	4.62	7.14	3	4	434	48.7	8.87
Q99798	Aconitate hydratase, mitochondrial OS=Homo sapiens GN=ACO2 PE=1 SV=2 - [ACON_HUMAN]	41.92	1	28	28	109	0.984	1.341	1.013	1.067	252.69	41.92	43	109	780	85.4	7.61

P62847	40S ribosomal protein S24 OS=Homo sapiens GN=RPS24 PE=1 SV=1 - [RS24_HUMAN]	20.30	1	3	3	31	0.643	0.797	0.859	1.067	85.44	20.30	5	31	133	15.4	10.78
Q8WUR7	UPF0235 protein C15orf40 OS=Homo sapiens GN=C15orf40 PE=1 SV=2 - [CO040_HUMAN]	11.11	1	1	1	3	0.961	1.140	1.892	1.067	2.32	11.11	2	3	153	16.3	9.83
P48506	Glutamate--cysteine ligase catalytic subunit OS=Homo sapiens GN=GCLC PE=1 SV=2 - [GSH1_HUMAN]	25.12	1	12	13	43	1.908	1.041	1.132	1.067	107.67	25.12	23	43	637	72.7	6.09
Q14956	Transmembrane glycoprotein NMB OS=Homo sapiens GN=GPNMB PE=1 SV=2 - [GPNMB_HUMAN]	1.57	1	1	1	4	0.546	0.652	0.485	1.067	3.90	1.57	2	4	572	63.9	6.64
Q9UN37	Vacuolar protein sorting-associated protein 4A OS=Homo sapiens GN=VPS4A PE=1 SV=1 - [VPS4A_HUMAN]	25.17	1	6	14	48	1.070	0.960	1.192	1.067	101.86	25.17	22	48	437	48.9	7.80
P13378	Homeobox protein Hox-D8 OS=Homo sapiens GN=HOXD8 PE=2 SV=2 - [HXD8_HUMAN]	5.17	1	1	1	2	0.982	1.038	0.797	1.067	4.84	5.17	1	2	290	31.9	8.50
Q96EP5	DAZ-associated protein 1 OS=Homo sapiens GN=DAZAP1 PE=1 SV=1 - [DAZP1_HUMAN]	25.80	1	7	7	23	1.324	1.439	1.016	1.067	79.43	25.80	10	23	407	43.4	8.56
O96019	Actin-like protein 6A OS=Homo sapiens GN=ACTL6A PE=1 SV=1 - [ACL6A_HUMAN]	34.03	2	12	12	40	1.216	1.213	0.833	1.067	111.09	34.03	19	40	429	47.4	5.60
O75832	26S proteasome non-ATPase regulatory subunit 10 OS=Homo sapiens GN=PSMD10 PE=1 SV=1 - [PSD10_HUMAN	48.23	1	9	9	40	1.069	0.929	1.003	1.067	77.21	48.23	14	40	226	24.4	6.10

Q15024	Exosome complex component RRP42 OS=Homo sapiens GN=EXOSC7 PE=1 SV=3 - [EXOS7_HUMAN]	23.02	1	5	5	8	1.137	1.215	1.090	1.068	17.82	23.02	7	8	291	31.8	5.19
Q08257	Quinone oxidoreductase OS=Homo sapiens GN=CRYZ PE=1 SV=1 - [QOR_HUMAN]	33.13	1	10	10	37	1.097	1.143	1.150	1.068	104.04	33.13	19	37	329	35.2	8.44
Q96DB5	Regulator of microtubule dynamics protein 1 OS=Homo sapiens GN=RMDN1 PE=1 SV=1 - [RMD1_HUMAN]	25.48	1	7	8	27	1.211	1.263	0.893	1.068	69.80	25.48	12	27	314	35.8	8.50
Q12905	Interleukin enhancer-binding factor 2 OS=Homo sapiens GN=ILF2 PE=1 SV=2 - [ILF2_HUMAN]	39.49	1	14	14	60	1.301	1.141	0.730	1.068	150.71	39.49	21	60	390	43.0	5.26
Q15650	Activating signal cointegrator 1 OS=Homo sapiens GN=TRIP4 PE=1 SV=4 - [TRIP4_HUMAN	12.56	1	6	7	15	1.378	1.107	1.177	1.068	37.32	12.56	10	15	581	66.1	7.85
P20674	Cytochrome c oxidase subunit 5A, mitochondrial OS=Homo sapiens GN=COX5A PE=1 SV=2 - [COX5A_HUMAN]	58.00	1	8	9	119	0.897	1.187	0.810	1.068	273.66	58.00	15	119	150	16.8	6.79
Q96EB6	NAD-dependent protein deacetylase sirtuin-1 OS=Homo sapiens GN=SIRT1 PE=1 SV=2 - [SIR1_HUMAN]	6.83	1	4	4	6	0.620	0.716	1.009	1.068	14.47	6.83	5	6	747	81.6	4.67
Q9NRL3	Striatin-4 OS=Homo sapiens GN=STRN4 PE=1 SV=2 - [STRN4_HUMAN]	11.02	1	5	8	20	0.904	0.824	1.224	1.069	44.75	11.02	12	20	753	80.5	5.40

Q86UK0	ATP-binding cassette sub-family A member 12 OS=Homo sapiens GN=ABCA12 PE=1 SV=3 - [ABCAC_HUMAN]	1.97	2	2	7	15	1.089	0.958	1.093	1.069	28.25	1.97	8	15	2595	293.0	7.75
Q969Q0	60S ribosomal protein L36a-like OS=Homo sapiens GN=RPL36AL PE=1 SV=3 - [RL36L_HUMAN]	40.57	1	1	7	29	1.301	1.394	0.981	1.069	63.59	40.57	13	29	106	12.5	10.65
Q9UHJ6	Sedoheptulokinase OS=Homo sapiens GN=SHPK PE=1 SV=3 - [SHPK_HUMAN]	12.55	1	5	5	11	0.838	1.034	1.033	1.069	27.70	12.55	7	11	478	51.5	6.83
Q02880	DNA topoisomerase 2-beta OS=Homo sapiens GN=TOP2B PE=1 SV=3 - [TOP2B_HUMA	16.48	1	18	29	83	1.123	1.296	1.040	1.069	149.48	16.48	44	83	1626	183.2	8.00
Q08623	Pseudouridine-5'-monophosphatase OS=Homo sapiens GN=HDHD1 PE=1 SV=3 - [HDHD1_HUMAN]	27.63	1	5	5	13	1.165	1.208	1.003	1.069	35.13	27.63	6	13	228	25.2	5.31
Q8WUH6	Transmembrane protein 263 OS=Homo sapiens GN=TMEM263 PE=1 SV=1 - [TM263_HUMAN]	12.93	1	1	1	6	0.799	0.964	1.250	1.069	16.33	12.93	2	6	116	11.7	9.32
Q15582	Transforming growth factor-beta-induced protein ig-h3 OS=Homo sapiens GN=TGFBI PE=1 SV=1 - [BGH3_HUMAN]	33.82	1	20	20	43	0.738	2.974	1.400	1.069	85.92	33.82	28	43	683	74.6	7.71
Q92925	SWI/SNF-related matrix-associated actin-dependent regulator of chromatin subfamily D member 2 OS=Homo sapiens GN=SMARCD2 PE=1 SV=3 - [SMRD2_HUMA	10.17	2	3	5	15	1.053	1.299	1.391	1.069	44.04	10.17	9	15	531	58.9	9.64

Q86YM7	Homer protein homolog 1 OS=Homo sapiens GN=HOMER1 PE=1 SV=2 - [HOME1_HUMAN]	7.63	1	2	3	5	0.737	0.663	0.802	1.069	11.94	7.63	4	5	354	40.3	5.44
Q13009	T-lymphoma invasion and metastasis-inducing protein 1 OS=Homo sapiens GN=TIAM1 PE=1 SV=2 - [TIAM1_HUMAN]	0.69	1	1	1	3	1.171		1.279	1.070	9.48	0.69	1	3	1591	177.4	6.61
Q96LW7	Bcl10-interacting CARD protein OS=Homo sapiens GN=C9orf89 PE=1 SV=1 - [BINCA_HUMAN]	5.70	1	1	1	8	0.895	0.787	0.917	1.070	35.71	5.70	2	8	228	25.6	9.20
P60228	Eukaryotic translation initiation factor 3 subunit E OS=Homo sapiens GN=EIF3E PE=1 SV=1 - [EIF3E_HUMAN]	40.67	1	18	18	78	0.913	0.939	0.860	1.070	167.72	40.67	28	78	445	52.2	6.04
Q8NC51	Plasminogen activator inhibitor 1 RNA-binding protein OS=Homo sapiens GN=SERBP1 PE=1 SV=2 - [PAIRB_HUMAN]	42.65	1	24	24	156	1.136	1.474	0.969	1.070	379.82	42.65	36	156	408	44.9	8.65
Q8IZ21	Phosphatase and actin regulator 4 OS=Homo sapiens GN=PHACTR4 PE=1 SV=1 - [PHAR4_HUMAN]	1.85	1	1	1	2	1.307	0.994	1.092	1.070	5.71	1.85	2	2	702	78.2	6.62
Q16656	Nuclear respiratory factor 1 OS=Homo sapiens GN=NRF1 PE=1 SV=1 - [NRF1_HUMAN]	1.79	1	1	1	1	0.987	1.109	0.859	1.070	2.05	1.79	1	1	503	53.5	5.05
Q04837	Single-stranded DNA-binding protein, mitochondrial OS=Homo sapiens GN=SSBP1 PE=1 SV=1 - [SSBP_HUMAN]	40.54	1	5	5	24	1.007	1.663	1.056	1.070	65.39	40.54	9	24	148	17.2	9.60

P27338	Amine oxidase [flavin-containing] B OS=Homo sapiens GN=MAOB PE=1 SV=3 - [AOFB_HUMAN]	14.23	1	4	7	28	0.554	8.607	1.973	1.070	71.48	14.23	13	28	520	58.7	7.50
Q13233	Mitogen-activated protein kinase kinase kinase 1 OS=Homo sapiens GN=MAP3K1 PE=1 SV=4 - [M3K1_HUMAN]	0.79	1	1	1	1	0.810	0.805	0.970	1.070	2.26	0.79	1	1	1512	164.4	7.74
Q02388	Collagen alpha-1(VII) chain OS=Homo sapiens GN=COL7A1 PE=1 SV=2 - [CO7A1_HUMAN]	13.49	1	27	28	58	1.590	0.985	0.888	1.071	152.69	13.49	41	58	2944	295.0	6.27
P62899	60S ribosomal protein L31 OS=Homo sapiens GN=RPL31 PE=1 SV=1 - [RL31_HUMAN]	34.40	1	6	6	56	0.921	1.047	0.990	1.071	146.34	34.40	9	56	125	14.5	10.54
P31937	3-hydroxyisobutyrate dehydrogenase, mitochondrial OS=Homo sapiens GN=HIBADH PE=1 SV=2 - [3HIDH_HUMAN]	23.51	1	6	6	22	1.224	1.542	1.200	1.071	68.76	23.51	10	22	336	35.3	8.13
P62266	40S ribosomal protein S23 OS=Homo sapiens GN=RPS23 PE=1 SV=3 - [RS23_HUMAN]	56.64	2	12	12	70	0.954	1.099	0.949	1.071	170.88	56.64	18	70	143	15.8	10.49
Q8N4Q0	Zinc-binding alcohol dehydrogenase domain-containing protein 2 OS=Homo sapiens GN=ZADH2 PE=1 SV=1 - [ZADH2_HUMAN]	4.24	1	1	1	2	0.831	0.880	1.175	1.071	5.72	4.24	1	2	377	40.1	8.18
Q9NPI1	Bromodomain-containing protein 7 OS=Homo sapiens GN=BRD7 PE=1 SV=1 - [BRD7_HUMAN]	2.61	1	1	1	4	1.238	1.674		1.071	11.66	2.61	2	4	651	74.1	6.39

Q04828	Aldo-keto reductase family 1 member C1 OS=Homo sapiens GN=AKR1C1 PE=1 SV=1 - [AK1C1_HUMAN	73.68	3	3	20	253	1.035	0.501	0.709	1.071	576.49	73.68	31	253	323	36.8	7.88
Q96G03	Phosphoglucomutase-2 OS=Homo sapiens GN=PGM2 PE=1 SV=4 - [PGM2_HUMAN]	30.88	1	17	18	60	1.000	1.297	0.954	1.071	125.16	30.88	29	60	612	68.2	6.73
Q9HB21	Pleckstrin homology domain-containing family A member 1 OS=Homo sapiens GN=PLEKHA1 PE=1 SV=2 - [PKHA1_HUMAN]	3.96	1	2	2	3	1.075	0.507	0.561	1.071	6.67	3.96	3	3	404	45.5	8.56
Q9NWU5	39S ribosomal protein L22, mitochondrial OS=Homo sapiens GN=MRPL22 PE=1 SV=1 - [RM22_HUMAN]	19.42	1	4	4	11	0.999	0.867	0.808	1.071	25.19	19.42	6	11	206	23.6	9.94
Q9H0X9	Oxysterol-binding protein-related protein 5 OS=Homo sapiens GN=OSBPL5 PE=1 SV=1 - [OSBL5_HUMAN]	10.13	1	6	7	23	0.863	0.890	1.129	1.071	63.08	10.13	9	23	879	98.6	7.47
P30281	G1/S-specific cyclin-D3 OS=Homo sapiens GN=CCND3 PE=1 SV=2 - [CCND3_HUMAN]	3.08	1	1	1	1	1.206	1.182	1.175	1.072	0.00	3.08	1	1	292	32.5	7.06
Q96AM1	Mas-related G-protein coupled receptor member F OS=Homo sapiens GN=MRGPRF PE=2 SV=1 - [MRGRF_HUMA	15.74	1	3	3	7	1.644	0.906	0.736	1.072	14.07	15.74	4	7	343	38.1	8.56
P30419	Glycylpeptide N-tetradecanoyltransferase 1 OS=Homo sapiens GN=NMT1 PE=1 SV=2 - [NMT1_HUMAN]	25.81	2	10	12	69	1.093	0.982	0.978	1.072	196.20	25.81	22	69	496	56.8	7.80
P99999	Cytochrome c OS=Homo sapiens GN=CYCS PE=1 SV=2 - [CYC_HUMAN]	64.76	1	9	9	74	1.255	1.034	1.049	1.072	202.77	64.76	17	74	105	11.7	9.57

Q92574	Hamartin OS=Homo sapiens GN=TSC1 PE=1 SV=2 - [TSC1_HUMAN]	4.38	1	3	3	6	1.183	0.867	1.131	1.072	19.72	4.38	6	6	1164	129.7	6.47
Q9NRR5	Ubiquilin-4 OS=Homo sapiens GN=UBQLN4 PE=1 SV=2 - [UBQL4_HUMAN]	16.47	1	4	8	31	0.825	1.059	0.945	1.072	79.09	16.47	14	31	601	63.8	5.22
P61353	60S ribosomal protein L27 OS=Homo sapiens GN=RPL27 PE=1 SV=2 - [RL27_HUMAN]	58.82	1	11	12	76	0.937	1.091	1.038	1.072	156.10	58.82	18	76	136	15.8	10.56
P05455	Lupus La protein OS=Homo sapiens GN=SSB PE=1 SV=2 - [LA_HUMAN]	58.09	1	33	33	128	1.024	0.986	0.858	1.072	286.67	58.09	50	128	408	46.8	7.12
Q9Y312	Protein AAR2 homolog OS=Homo sapiens GN=AAR2 PE=1 SV=2 - [AAR2_HUMAN]	5.99	1	2	2	2	0.998	1.749	1.134	1.072	4.66	5.99	2	2	384	43.4	5.96
Q5SRE7	Phytanoyl-CoA dioxygenase domain-containing protein 1 OS=Homo sapiens GN=PHYHD1 PE=1 SV=2 - [PHYD1_HUMAN]	29.21	1	7	7	17	1.159	1.293	0.743	1.072	45.19	29.21	9	17	291	32.4	6.32
Q05655	Protein kinase C delta type OS=Homo sapiens GN=PRKCD PE=1 SV=2 - [KPCD_HUMAN]	8.28	1	5	6	8	1.372	0.791	1.060	1.073	15.35	8.28	8	8	676	77.5	7.75
Q9Y546	Leucine-rich repeat-containing protein 42 OS=Homo sapiens GN=LRRC42 PE=1 SV=1 - [LRC42_HUMAN]	2.10	1	1	1	1	1.131	0.956	0.956	1.073	2.21	2.10	1	1	428	48.5	7.46
Q9BTA9	WW domain-containing adapter protein with coiled-coil OS=Homo sapiens GN=WAC PE=1 SV=3 - [WAC_HUMAN]	1.85	1	1	1	3	0.974	1.105	0.894	1.073	7.27	1.85	2	3	647	70.7	9.45

P82932	28S ribosomal protein S6, mitochondrial OS=Homo sapiens GN=MRPS6 PE=1 SV=3 - [RT06_HUMAN]	34.40	1	6	6	24	0.833	0.937	0.733	1.073	53.45	34.40	9	24	125	14.2	9.26
Q96T51	RUN and FYVE domain-containing protein 1 OS=Homo sapiens GN=RUFY1 PE=1 SV=2 - [RUFY1_HUMAN]	25.99	1	19	20	58	0.976	1.306	1.162	1.073	111.90	25.99	31	58	708	79.8	5.74
Q8IYB7	DIS3-like exonuclease 2 OS=Homo sapiens GN=DIS3L2 PE=1 SV=4 - [DI3L2_HUMAN]	8.02	1	6	6	16	1.108	1.060	1.270	1.073	42.02	8.02	11	16	885	99.2	6.10
Q6XE24	RNA-binding motif, single-stranded-interacting protein 3 OS=Homo sapiens GN=RBMS3 PE=1 SV=1 - [RBMS3_HUMAN]	24.71	1	6	9	22	0.742	0.974	0.686	1.073	49.74	24.71	12	22	437	47.8	8.03
Q96GC5	39S ribosomal protein L48, mitochondrial OS=Homo sapiens GN=MRPL48 PE=1 SV=2 - [RM48_HUMAN]	16.98	1	3	3	14	0.964	1.393	0.871	1.073	42.72	16.98	6	14	212	23.9	8.98
P18615	Negative elongation factor E OS=Homo sapiens GN=NELFE PE=1 SV=3 - [NELFE_HUMAN]	18.95	1	5	6	19	0.933	1.145	1.144	1.073	42.78	18.95	9	19	380	43.2	9.33
Q86UK7	Zinc finger protein 598 OS=Homo sapiens GN=ZNF598 PE=1 SV=1 - [ZN598_HUMAN]	10.95	1	9	9	20	1.032	0.859	0.914	1.073	59.87	10.95	14	20	904	98.6	8.40
P34059	N-acetylgalactosamine-6-sulfatase OS=Homo sapiens GN=GALNS PE=1 SV=1 - [GALNS_HUMAN	14.37	1	8	8	36	1.233	1.310	0.998	1.074	90.01	14.37	10	36	522	58.0	6.74
P14854	Cytochrome c oxidase subunit 6B1 OS=Homo sapiens GN=COX6B1 PE=1 SV=2 - [CX6B1_HUMAN]	58.14	1	4	4	33	0.921	1.047	0.848	1.074	92.76	58.14	7	33	86	10.2	7.05

Q7Z5Q1	Cytoplasmic polyadenylation element-binding protein 2 OS=Homo sapiens GN=CPEB2 PE=2 SV=3 - [CPEB2_HUMAN]	4.41	1	2	2	6	0.926	1.430	1.419	1.074	14.41	4.41	4	6	589	64.9	7.21
P62280	40S ribosomal protein S11 OS=Homo sapiens GN=RPS11 PE=1 SV=3 - [RS11_HUMAN]	54.43	1	16	16	74	0.800	1.031	0.938	1.074	162.66	54.43	26	74	158	18.4	10.30
O14727	Apoptotic protease-activating factor 1 OS=Homo sapiens GN=APAF1 PE=1 SV=2 - [APAF_HUMAN]	3.53	1	4	4	4	0.976	1.098	0.639	1.074	11.44	3.53	4	4	1248	141.7	6.40
Q9NX20	39S ribosomal protein L16, mitochondrial OS=Homo sapiens GN=MRPL16 PE=1 SV=1 - [RM16_HUMAN]	18.33	1	4	4	10	1.093	1.376	0.824	1.074	20.04	18.33	7	10	251	28.4	10.13
Q6NYC8	Phostensin OS=Homo sapiens GN=PPP1R18 PE=1 SV=1 - [PPR18_HUMAN]	26.59	1	11	11	39	1.221	1.230	1.143	1.074	103.82	26.59	16	39	613	67.9	5.40
Q96NC0	Zinc finger matrin-type protein 2 OS=Homo sapiens GN=ZMAT2 PE=1 SV=1 - [ZMAT2_HUMAN]	6.53	1	2	2	3	1.050	1.423	0.965	1.074	1.71	6.53	2	3	199	23.6	9.01
P62316	Small nuclear ribonucleoprotein Sm D2 OS=Homo sapiens GN=SNRPD2 PE=1 SV=1 - [SMD2_HUMAN]	61.86	1	7	7	112	1.020	1.058	0.858	1.074	294.22	61.86	10	112	118	13.5	9.91
Q9GZR2	RNA exonuclease 4 OS=Homo sapiens GN=REXO4 PE=1 SV=2 - [REXO4_HUMAN]	5.45	1	2	2	2	1.085	1.213	0.909	1.074	2.09	5.45	2	2	422	46.6	9.77
P49915	GMP synthase [glutamine-hydrolyzing] OS=Homo sapiens GN=GMPS PE=1 SV=1 - [GUAA_HUMAN]	26.41	1	17	17	48	1.028	1.030	1.019	1.074	120.88	26.41	25	48	693	76.7	6.87

Q9Y5Z4	Heme-binding protein 2 OS=Homo sapiens GN=HEBP2 PE=1 SV=1 - [HEBP2_HUMAN]	30.24	1	6	6	59	1.185	0.981	0.937	1.074	169.70	30.24	11	59	205	22.9	4.63
O14561	Acyl carrier protein, mitochondrial OS=Homo sapiens GN=NDUFAB1 PE=1 SV=3 - [ACPM_HUMAN]	15.38	1	3	3	22	1.212	1.188	0.734	1.074	36.21	15.38	5	22	156	17.4	4.93
Q9NRZ7	1-acyl-sn-glycerol-3-phosphate acyltransferase gamma OS=Homo sapiens GN=AGPAT3 PE=1 SV=1 - [PLCC_HUMAN]	19.68	1	7	7	25	1.199	0.771	0.820	1.074	64.39	19.68	10	25	376	43.4	8.72
O75110	Probable phospholipid-transporting ATPase IIA OS=Homo sapiens GN=ATP9A PE=1 SV=3 - [ATP9A_HUMAN]	3.53	1	3	3	3	1.168	1.120	1.157	1.074	5.80	3.53	3	3	1047	118.5	7.77
Q7Z5V6	Protein phosphatase 1 regulatory subunit 32 OS=Homo sapiens GN=PPP1R32 PE=1 SV=1 - [PPR32_HUMAN]	7.29	1	1	1	1	0.642	0.720	1.131	1.074	2.67	7.29	1	1	425	47.3	8.54
P06753	Tropomyosin alpha-3 chain OS=Homo sapiens GN=TPM3 PE=1 SV=2 - [TPM3_HUMAN]	69.82	1	10	37	1043	1.456	0.958	1.265	1.074	2421.10	69.82	58	1043	285	32.9	4.72
Q53FP2	Transmembrane protein 35 OS=Homo sapiens GN=TMEM35 PE=2 SV=2 - [TMM35_HUMAN]	14.37	1	3	3	11	0.655	3.184	1.582	1.075	25.20	14.37	4	11	167	18.4	10.08
Q3MIN7	Ral guanine nucleotide dissociation stimulator-like 3 OS=Homo sapiens GN=RGL3 PE=1 SV=2 - [RGL3_HUMAN]	2.68	1	1	1	6	1.146	1.120	1.193	1.075	9.60	2.68	1	6	710	78.0	7.68
O15056	Synaptojanin-2 OS=Homo sapiens GN=SYNJ2 PE=1 SV=3 - [SYNJ2_HUMAN]	3.14	1	4	4	9	1.069	1.650	1.128	1.075	18.77	3.14	5	9	1496	165.4	7.34

O00217	NADH dehydrogenase [ubiquinone] iron-sulfur protein 8, mitochondrial OS=Homo sapiens GN=NDUFS8 PE=1 SV=1 - [NDUS8_HUMAN]	10.95	1	2	2	13	0.611	1.088	0.735	1.075	36.09	10.95	4	13	210	23.7	6.34
O95661	GTP-binding protein Di-Ras3 OS=Homo sapiens GN=DIRAS3 PE=1 SV=1 - [DIRA3_HUMAN]	3.06	1	1	1	2	1.050	0.763	0.706	1.075	1.62	3.06	1	2	229	25.8	9.39
Q96E52	Metalloendopeptidase OMA1, mitochondrial OS=Homo sapiens GN=OMA1 PE=1 SV=1 - [OMA1_HUMAN]	2.86	1	2	2	2	0.807	1.036	1.357	1.075	3.95	2.86	2	2	524	60.1	9.25
O43715	TP53-regulated inhibitor of apoptosis 1 OS=Homo sapiens GN=TRIAP1 PE=1 SV=1 - [TRIA1_HUMAN	44.74	1	3	3	8	0.706	1.248	0.996	1.075	28.89	44.74	5	8	76	8.8	5.48
P07738	Bisphosphoglycerate mutase OS=Homo sapiens GN=BPGM PE=1 SV=2 - [PMGE_HUMAN]	28.96	1	5	5	13	1.049	0.995	1.128	1.075	22.69	28.96	10	13	259	30.0	6.54
P28331	NADH-ubiquinone oxidoreductase 75 kDa subunit, mitochondrial OS=Homo sapiens GN=NDUFS1 PE=1 SV=3 - [NDUS1_HUMAN	37.41	1	21	21	46	0.612	0.966	0.835	1.075	134.84	37.41	31	46	727	79.4	6.23
Q6KCM7	Calcium-binding mitochondrial carrier protein SCaMC-2 OS=Homo sapiens GN=SLC25A25 PE=1 SV=1 - [SCMC2_HUMAN]	6.82	2	2	2	4	1.038	1.709	2.105	1.075	9.08	6.82	2	4	469	52.6	8.35
P55899	IgG receptor FcRn large subunit p51 OS=Homo sapiens GN=FCGRT PE=1 SV=1 - [FCGRN_HUMAN]	16.16	1	5	5	7	1.135	1.052	0.924	1.075	11.08	16.16	7	7	365	39.7	6.54

Q15185	Prostaglandin E synthase 3 OS=Homo sapiens GN=PTGES3 PE=1 SV=1 - [TEBP_HUMAN]	39.38	1	6	6	24	1.066	1.083	0.882	1.076	78.87	39.38	10	24	160	18.7	4.54
O95155	Ubiquitin conjugation factor E4 B OS=Homo sapiens GN=UBE4B PE=1 SV=1 - [UBE4B_HUMAN]	12.67	1	14	15	51	0.740	0.912	1.122	1.076	117.38	12.67	22	51	1302	146.1	6.55
P17252	Protein kinase C alpha type OS=Homo sapiens GN=PRKCA PE=1 SV=4 - [KPCA_HUMAN]	19.49	1	14	17	49	1.087	0.693	0.602	1.076	106.13	19.49	26	49	672	76.7	7.05
Q9BVQ7	Spermatogenesis-associated protein 5-like protein 1 OS=Homo sapiens GN=SPATA5L1 PE=1 SV=2 - [SPA5L_HUMAN]	7.04	1	3	3	7	0.810	0.931	1.001	1.076	28.65	7.04	4	7	753	80.7	8.09
Q14765	Signal transducer and activator of transcription 4 OS=Homo sapiens GN=STAT4 PE=1 SV=1 - [STAT4_HUMAN]	0.80	1	1	1	1	1.971	1.328	0.949	1.076	0.00	0.80	1	1	748	85.9	6.65
O43583	Density-regulated protein OS=Homo sapiens GN=DENR PE=1 SV=2 - [DENR_HUMAN]	26.77	1	5	5	19	0.980	1.147	1.390	1.076	41.73	26.77	7	19	198	22.1	5.30
P25490	Transcriptional repressor protein YY1 OS=Homo sapiens GN=YY1 PE=1 SV=2 - [TYY1_HUMAN]	7.49	2	3	3	4	0.838	0.713	0.872	1.076	5.98	7.49	4	4	414	44.7	6.25
Q06830	Peroxiredoxin-1 OS=Homo sapiens GN=PRDX1 PE=1 SV=1 - [PRDX1_HUMAN]	68.84	1	17	21	492	1.637	0.720	1.179	1.076	1056.96	68.84	35	492	199	22.1	8.13
Q92785	Zinc finger protein ubi-d4 OS=Homo sapiens GN=DPF2 PE=1 SV=2 - [REQU_HUMAN]	12.02	1	4	4	14	1.161	1.537	0.636	1.076	36.34	12.02	5	14	391	44.1	6.33

P26373	60S ribosomal protein L13 OS=Homo sapiens GN=RPL13 PE=1 SV=4 - [RL13_HUMAN]	40.28	1	10	10	73	0.767	1.079	0.945	1.076	151.97	40.28	16	73	211	24.2	11.65
O96005	Cleft lip and palate transmembrane protein 1 OS=Homo sapiens GN=CLPTM1 PE=1 SV=1 - [CLPT1_HUMAN]	11.51	1	7	7	30	1.393	0.780	0.842	1.076	70.57	11.51	12	30	669	76.0	6.30
O95870	Abhydrolase domain-containing protein 16A OS=Homo sapiens GN=ABHD16A PE=1 SV=3 - [ABHGA_HUMAN]	14.16	1	5	5	8	0.948	1.070	0.951	1.076	26.44	14.16	7	8	558	63.2	8.13
P36957	Dihydrolipoyllysine-residue succinyltransferase component of 2-oxoglutarate dehydrogenase complex, mitochondrial OS=Homo sapiens GN=DLST PE=1 SV=4 - [ODO2_HUMAN]	26.05	1	10	10	74	1.113	1.348	1.193	1.076	187.93	26.05	18	74	453	48.7	8.95
Q13822	Ectonucleotide pyrophosphatase/phosphodiesterase family member 2 OS=Homo sapiens GN=ENPP2 PE=1 SV=3 - [ENPP2_HUMAN]	0.81	1	1	1	1	0.720	1.104	0.668	1.077	1.78	0.81	1	1	863	98.9	7.37
Q5SQS8	Uncharacterized protein C10orf120 OS=Homo sapiens GN=C10orf120 PE=2 SV=1 - [CJ120_HUMAN]	3.58	1	1	1	1	0.133	0.895	1.883	1.077	2.56	3.58	1	1	335	39.2	9.44
P49917	DNA ligase 4 OS=Homo sapiens GN=LIG4 PE=1 SV=2 - [DNLI4_HUMAN]	1.43	1	1	1	7	0.999	0.864	1.088	1.077	16.44	1.43	2	7	911	103.9	7.96
Q8NFC6	Biorientation of chromosomes in cell division protein 1-like 1 OS=Homo sapiens GN=BOD1L1 PE=1 SV=2 - [BD1L1_HUMAN	3.47	1	8	11	24	1.133	1.337	0.931	1.077	40.95	3.47	15	24	3051	330.3	5.08

O14647	Chromodomain-helicase-DNA-binding protein 2 OS=Homo sapiens GN=CHD2 PE=1 SV=2 - [CHD2_HUMAN]	3.56	1	2	4	5	1.173	1.240	0.958	1.077	10.52	3.56	5	5	1828	211.2	8.10
Q5VZ66	Janus kinase and microtubule-interacting protein 3 OS=Homo sapiens GN=JAKMIP3 PE=2 SV=2 - [JKIP3_HUMAN]	4.62	1	2	5	23	1.282	0.867	0.813	1.077	46.42	4.62	6	23	844	98.5	5.66
P19174	1-phosphatidylinositol 4,5-bisphosphate phosphodiesterase gamma-1 OS=Homo sapiens GN=PLCG1 PE=1 SV=1 - [PLCG1_HUMAN]	14.73	1	17	18	36	0.955	1.017	1.145	1.077	82.95	14.73	25	36	1290	148.4	6.05
P49116	Nuclear receptor subfamily 2 group C member 2 OS=Homo sapiens GN=NR2C2 PE=1 SV=1 - [NR2C2_HUMAN	5.54	1	3	3	4	1.178	0.816	1.218	1.077	9.63	5.54	4	4	596	65.4	6.28
P11172	Uridine 5'-monophosphate synthase OS=Homo sapiens GN=UMPS PE=1 SV=1 - [UMPS_HUMAN]	30.63	1	16	16	40	1.038	0.958	0.959	1.077	79.44	30.63	26	40	480	52.2	7.24
O43924	Retinal rod rhodopsin-sensitive cGMP 3',5'-cyclic phosphodiesterase subunit delta OS=Homo sapiens GN=PDE6D PE=1 SV=1 - [PDE6D_HUMAN	20.67	1	2	2	3	1.043	1.038	1.233	1.077	11.83	20.67	3	3	150	17.4	5.67
Q9Y3C0	WASH complex subunit CCDC53 OS=Homo sapiens GN=CCDC53 PE=1 SV=1 - [CCD53_HUMAN]	3.61	1	1	1	1	0.793	1.349	1.227	1.077	0.00	3.61	1	1	194	21.2	4.46

P36941	Tumor necrosis factor receptor superfamily member 3 OS=Homo sapiens GN=LTBR PE=1 SV=1 - [TNR3_HUMAN]	6.21	1	2	2	5	0.938	0.778	1.047	1.078	13.01	6.21	3	5	435	46.7	5.83
P49441	Inositol polyphosphate 1-phosphatase OS=Homo sapiens GN=INPP1 PE=1 SV=1 - [INPP_HUMAN]	21.80	1	8	8	12	1.226	1.097	1.005	1.078	27.16	21.80	12	12	399	44.0	5.26
P08651	Nuclear factor 1 C-type OS=Homo sapiens GN=NFIC PE=1 SV=2 - [NFIC_HUMAN]	12.20	1	4	5	13	0.897	2.302	1.167	1.078	30.04	12.20	8	13	508	55.6	8.38
O00764	Pyridoxal kinase OS=Homo sapiens GN=PDXK PE=1 SV=1 - [PDXK_HUMAN]	31.09	1	8	8	37	0.881	1.006	0.841	1.078	74.90	31.09	14	37	312	35.1	6.13
P09038	Fibroblast growth factor 2 OS=Homo sapiens GN=FGF2 PE=1 SV=3 - [FGF2_HUMAN]	7.29	1	2	2	8	1.055	0.669	0.728	1.078	14.53	7.29	3	8	288	30.8	11.18
Q8N183	Mimitin, mitochondrial OS=Homo sapiens GN=NDUFAF2 PE=1 SV=1 - [MIMIT_HUMAN]	41.42	1	7	7	12	0.951	0.964	0.790	1.078	26.73	41.42	8	12	169	19.8	8.97
Q9Y295	Developmentally-regulated GTP-binding protein 1 OS=Homo sapiens GN=DRG1 PE=1 SV=1 - [DRG1_HUMAN]	35.42	1	11	11	44	0.949	0.926	1.009	1.078	122.78	35.42	19	44	367	40.5	8.90
O75164	Lysine-specific demethylase 4A OS=Homo sapiens GN=KDM4A PE=1 SV=2 - [KDM4A_HUMAN]	1.03	1	1	1	4	1.075	1.283	0.525	1.078	10.69	1.03	1	4	1064	120.6	5.85
O15234	Protein CASC3 OS=Homo sapiens GN=CASC3 PE=1 SV=2 - [CASC3_HUMAN]	3.56	1	2	2	4	1.036	1.069	0.821	1.078	7.93	3.56	4	4	703	76.2	6.48

A6ND91	Putative L-aspartate dehydrogenase OS=Homo sapiens GN=ASPDH PE=2 SV=2 - [ASPD_HUMAN]	2.83	1	1	1	1	0.836	0.343	0.683	1.078	1.77	2.83	1	1	283	29.9	7.28
P29692	Elongation factor 1-delta OS=Homo sapiens GN=EEF1D PE=1 SV=5 - [EF1D_HUMAN]	63.70	1	14	18	267	0.884	1.039	1.073	1.078	631.62	63.70	29	267	281	31.1	5.01
Q2TAZ0	Autophagy-related protein 2 homolog A OS=Homo sapiens GN=ATG2A PE=1 SV=3 - [ATG2A_HUMAN]	1.19	1	2	2	3	1.325	1.608	1.478	1.078	1.93	1.19	2	3	1938	212.7	5.88
P47897	Glutamine--tRNA ligase OS=Homo sapiens GN=QARS PE=1 SV=1 - [SYQ_HUMAN]	42.58	1	29	29	129	1.004	0.828	0.917	1.078	321.50	42.58	49	129	775	87.7	7.15
Q8N684	Cleavage and polyadenylation specificity factor subunit 7 OS=Homo sapiens GN=CPSF7 PE=1 SV=1 - [CPSF7_HUMAN]	19.96	1	7	7	16	1.200	1.136	1.115	1.078	46.62	19.96	11	16	471	52.0	8.00
Q9UMX5	Neudesin OS=Homo sapiens GN=NENF PE=1 SV=1 - [NENF_HUMAN]	66.86	1	10	10	55	0.930	0.993	0.990	1.078	129.19	66.86	17	55	172	18.8	5.69
Q7Z4H8	KDEL motif-containing protein 2 OS=Homo sapiens GN=KDELC2 PE=1 SV=2 - [KDEL2_HUMAN]	36.69	1	15	15	42	1.322	0.800	0.714	1.079	117.66	36.69	22	42	507	58.5	8.24
Q9NP73	Putative bifunctional UDP-N-acetylglucosamine transferase and deubiquitinase ALG13 OS=Homo sapiens GN=ALG13 PE=1 SV=2 - [ALG13_HUMAN]	4.13	1	3	3	3	1.242	1.751	2.327	1.079	2.90	4.13	3	3	1137	126.0	6.74

Q86V88	Magnesium-dependent phosphatase 1 OS=Homo sapiens GN=MDP1 PE=1 SV=1 - [MGDP1_HUMAN]	14.20	1	2	2	2	1.178	1.765	0.957	1.079	9.28	14.20	2	2	176	20.1	6.39
Q96BI1	Solute carrier family 22 member 18 OS=Homo sapiens GN=SLC22A18 PE=1 SV=3 - [S22AI_HUMAN]	5.90	1	3	3	9	1.268	0.716	0.870	1.079	23.14	5.90	5	9	424	44.8	9.57
Q9BQP7	Mitochondrial genome maintenance exonuclease 1 OS=Homo sapiens GN=MGME1 PE=1 SV=1 - [MGME1_HUMAN]	3.49	1	1	1	2	0.667	1.666	1.633	1.079	7.12	3.49	1	2	344	39.4	7.68
P51854	Transketolase-like protein 1 OS=Homo sapiens GN=TKTL1 PE=1 SV=2 - [TKTL1_HUMAN]	3.52	1	1	3	6	0.860	0.682	0.645	1.079	7.16	3.52	4	6	596	65.3	5.86
Q9Y5F3	Protocadherin beta-1 OS=Homo sapiens GN=PCDHB1 PE=2 SV=2 - [PCDB1_HUMAN]	0.98	1	1	1	1	0.810	0.990	0.663	1.079	2.58	0.98	1	1	818	90.4	5.39
Q13873	Bone morphogenetic protein receptor type-2 OS=Homo sapiens GN=BMPR2 PE=1 SV=2 - [BMPR2_HUMAN]	1.83	1	2	2	4	1.060	1.177	1.173	1.079	4.44	1.83	3	4	1038	115.1	6.20
O43570	Carbonic anhydrase 12 OS=Homo sapiens GN=CA12 PE=1 SV=1 - [CAH12_HUMAN]	2.54	1	1	1	1	1.219	2.139	0.943	1.079	0.00	2.54	1	1	354	39.4	7.23
Q9H8Y5	Ankyrin repeat and zinc finger domain-containing protein 1 OS=Homo sapiens GN=ANKZF1 PE=1 SV=1 - [ANKZ1_HUMAN]	8.40	1	6	6	8	0.949	1.205	1.080	1.080	12.26	8.40	7	8	726	80.9	8.41
P35268	60S ribosomal protein L22 OS=Homo sapiens GN=RPL22 PE=1 SV=2 - [RL22_HUMAN]	53.13	1	6	6	50	1.062	1.026	1.078	1.080	130.07	53.13	9	50	128	14.8	9.19

Q00987	E3 ubiquitin-protein ligase Mdm2 OS=Homo sapiens GN=MDM2 PE=1 SV=1 - [MDM2_HUMAN]	5.30	1	2	2	10	0.631	0.733	1.159	1.080	0.00	5.30	2	10	491	55.2	4.69
Q9UJV9	Probable ATP-dependent RNA helicase DDX41 OS=Homo sapiens GN=DDX41 PE=1 SV=2 - [DDX41_HUMAN	4.02	1	2	3	5	0.610	0.793	0.822	1.080	12.50	4.02	3	5	622	69.8	6.84
Q15907	Ras-related protein Rab-11B OS=Homo sapiens GN=RAB11B PE=1 SV=4 - [RB11B_HUMAN]	56.88	2	2	12	57	0.929	0.819	0.919	1.080	122.88	56.88	19	57	218	24.5	5.94
P35659	Protein DEK OS=Homo sapiens GN=DEK PE=1 SV=1 - [DEK_HUMAN]	20.53	1	11	11	59	1.094	1.137	0.829	1.080	146.97	20.53	19	59	375	42.6	8.56
Q7L014	Probable ATP-dependent RNA helicase DDX46 OS=Homo sapiens GN=DDX46 PE=1 SV=2 - [DDX46_HUMAN	28.32	1	27	28	76	1.069	1.239	1.036	1.080	163.58	28.32	44	76	1031	117.3	9.29
O75394	39S ribosomal protein L33, mitochondrial OS=Homo sapiens GN=MRPL33 PE=1 SV=1 - [RM33_HUMAN]	47.69	1	2	3	5	1.246	1.353	1.104	1.080	11.74	47.69	5	5	65	7.6	10.80
Q96S55	ATPase WRNIP1 OS=Homo sapiens GN=WRNIP1 PE=1 SV=2 - [WRIP1_HUMAN]	4.51	1	3	3	8	1.226	1.093	0.936	1.080	12.35	4.51	5	8	665	72.1	6.10
Q9H019	Mitochondrial fission regulator 1-like OS=Homo sapiens GN=MTFR1L PE=1 SV=2 - [MFR1L_HUMAN]	21.58	1	4	4	6	1.791	1.334	1.310	1.080	16.38	21.58	5	6	292	31.9	6.10
Q8NI36	WD repeat-containing protein 36 OS=Homo sapiens GN=WDR36 PE=1 SV=1 - [WDR36_HUMAN]	6.10	1	6	6	22	1.012	0.938	1.137	1.081	46.14	6.10	9	22	951	105.3	7.53

Q9BU23	Lipase maturation factor 2 OS=Homo sapiens GN=LMF2 PE=1 SV=2 - [LMF2_HUMAN]	8.06	1	5	5	28	1.022	0.965	1.020	1.081	46.58	8.06	9	28	707	79.6	10.10
Q96A33	Coiled-coil domain-containing protein 47 OS=Homo sapiens GN=CCDC47 PE=1 SV=1 - [CCD47_HUMAN]	31.68	1	13	13	56	0.945	0.870	0.946	1.081	154.06	31.68	21	56	483	55.8	4.87
Q99519	Sialidase-1 OS=Homo sapiens GN=NEU1 PE=1 SV=1 - [NEUR1_HUMAN]	14.22	1	3	3	5	1.693	0.656	0.789	1.081	18.03	14.22	4	5	415	45.4	5.88
Q9ULT8	E3 ubiquitin-protein ligase HECTD1 OS=Homo sapiens GN=HECTD1 PE=1 SV=3 - [HECD1_HUMAN]	8.93	1	21	23	74	0.896	0.957	0.908	1.081	194.31	8.93	35	74	2610	289.2	5.35
Q9NPB8	Glycerophosphocholine phosphodiesterase GPCPD1 OS=Homo sapiens GN=GPCPD1 PE=1 SV=2 - [GPCP1_HUMAN]	10.27	1	7	7	18	2.538	0.847	0.753	1.081	40.06	10.27	12	18	672	76.0	5.53
Q9Y277	Voltage-dependent anion-selective channel protein 3 OS=Homo sapiens GN=VDAC3 PE=1 SV=1 - [VDAC3_HUMAN	49.47	1	10	12	74	1.537	1.136	0.674	1.081	170.98	49.47	17	74	283	30.6	8.66
Q9NRN7	L-aminoadipate-semialdehyde dehydrogenase-phosphopantetheinyl transferase OS=Homo sapiens GN=AASDHPPT PE=1 SV=2 - [ADPPT_HUMAN]	19.42	1	5	5	7	0.801	0.774	0.718	1.081	16.87	19.42	7	7	309	35.8	6.80
Q9HC38	Glyoxalase domain-containing protein 4 OS=Homo sapiens GN=GLOD4 PE=1 SV=1 - [GLOD4_HUMAN]	37.38	1	13	13	48	1.292	1.132	1.108	1.081	111.62	37.38	20	48	313	34.8	5.60

Q9P2T1	GMP reductase 2 OS=Homo sapiens GN=GMPR2 PE=1 SV=1 - [GMPR2_HUMAN]	10.06	1	2	5	15	1.146	0.862	1.060	1.081	26.58	10.06	8	15	348	37.9	7.23
Q7L9L4	MOB kinase activator 1B OS=Homo sapiens GN=MOB1B PE=1 SV=3 - [MOB1B_HUMAN]	17.13	2	4	4	10	1.238	1.092	1.036	1.081	19.89	17.13	6	10	216	25.1	6.73
O75531	Barrier-to-autointegration factor OS=Homo sapiens GN=BANF1 PE=1 SV=1 - [BAF_HUMAN]	50.56	1	6	6	18	1.121	1.606	1.124	1.082	37.21	50.56	9	18	89	10.1	6.09
P13073	Cytochrome c oxidase subunit 4 isoform 1, mitochondrial OS=Homo sapiens GN=COX4I1 PE=1 SV=1 - [COX41_HUMAN]	35.50	1	8	8	51	1.013	1.059	1.042	1.082	131.04	35.50	14	51	169	19.6	9.51
Q9H490	Phosphatidylinositol glycan anchor biosynthesis class U protein OS=Homo sapiens GN=PIGU PE=1 SV=3 - [PIGU_HUMAN]	6.21	1	2	2	3	0.849	0.659	0.719	1.082	8.98	6.21	3	3	435	50.0	7.72
P85037	Forkhead box protein K1 OS=Homo sapiens GN=FOXK1 PE=1 SV=1 - [FOXK1_HUMAN]	17.19	1	10	11	25	0.860	1.186	1.149	1.082	53.96	17.19	16	25	733	75.4	9.32
O95562	Vesicle transport protein SFT2B OS=Homo sapiens GN=SFT2D2 PE=1 SV=1 - [SFT2B_HUMAN	6.25	1	1	1	6	0.813	1.102	1.033	1.082	11.01	6.25	2	6	160	17.8	9.13
Q587I9	Vesicle transport protein SFT2C OS=Homo sapiens GN=SFT2D3 PE=2 SV=1 - [SFT2C_HUMAN	23.26	1	3	3	15	0.901	1.552	1.428	1.082	35.69	23.26	5	15	215	21.8	9.89
Q9NP77	RNA polymerase II subunit A C-terminal domain phosphatase SSU72 OS=Homo sapiens GN=SSU72 PE=1 SV=1 - [SSU72_HUMAN	22.16	1	4	4	7	1.172	1.137	1.125	1.082	19.41	22.16	6	7	194	22.6	5.33

O75947	ATP synthase subunit d, mitochondrial OS=Homo sapiens GN=ATP5H PE=1 SV=3 - [ATP5H_HUMAN]	77.02	1	14	14	68	1.107	1.120	0.854	1.082	145.65	77.02	22	68	161	18.5	5.30
P09110	3-ketoacyl-CoA thiolase, peroxisomal OS=Homo sapiens GN=ACAA1 PE=1 SV=2 - [THIK_HUMAN]	26.89	1	9	9	34	1.165	1.085	1.442	1.082	101.13	26.89	14	34	424	44.3	8.44
P49069	Calcium signal-modulating cyclophilin ligand OS=Homo sapiens GN=CAMLG PE=1 SV=1 - [CAMLG_HUMAN]	22.64	1	5	5	16	0.981	0.941	1.253	1.082	41.64	22.64	7	16	296	32.9	8.05
Q969V3	Nicalin OS=Homo sapiens GN=NCLN PE=1 SV=2 - [NCLN_HUMAN]	35.35	1	17	17	73	0.934	0.775	0.824	1.082	185.07	35.35	30	73	563	62.9	6.89
Q8TBF5	Phosphatidylinositol-glycan biosynthesis class X protein OS=Homo sapiens GN=PIGX PE=2 SV=3 - [PIGX_HUMAN]	3.49	1	1	1	3	1.099	0.955	0.739	1.082	5.44	3.49	2	3	258	28.8	6.33
Q9BRJ2	39S ribosomal protein L45, mitochondrial OS=Homo sapiens GN=MRPL45 PE=1 SV=2 - [RM45_HUMAN]	12.75	3	4	5	17	0.981	1.240	0.918	1.082	21.77	12.75	7	17	306	35.3	9.03
Q969Q6	Serine/threonine-protein phosphatase 2A regulatory subunit B'' subunit gamma OS=Homo sapiens GN=PPP2R3C PE=1 SV=1 - [P2R3C_HUMAN]	3.09	2	2	2	4	0.936	0.761	0.768	1.082	6.84	3.09	2	4	453	53.3	5.19
Q15003	Condensin complex subunit 2 OS=Homo sapiens GN=NCAPH PE=1 SV=3 - [CND2_HUMAN]	3.64	1	2	2	2	1.216	1.087	0.700	1.083	7.41	3.64	2	2	741	82.5	5.06

P11137	Microtubule-associated protein 2 OS=Homo sapiens GN=MAP2 PE=1 SV=4 - [MTAP2_HUMAN]	6.68	1	11	11	25	0.981	1.058	0.871	1.083	41.91	6.68	18	25	1827	199.4	4.91
Q9H1A3	Methyltransferase-like protein 9 OS=Homo sapiens GN=METTL9 PE=1 SV=1 - [METL9_HUMAN]	4.09	1	1	1	2	1.119	0.732	0.924	1.083	3.87	4.09	2	2	318	36.5	7.36
Q15904	V-type proton ATPase subunit S1 OS=Homo sapiens GN=ATP6AP1 PE=1 SV=2 - [VAS1_HUMAN]	5.96	1	2	2	8	0.930	0.868	1.031	1.083	17.43	5.96	4	8	470	52.0	6.14
P42765	3-ketoacyl-CoA thiolase, mitochondrial OS=Homo sapiens GN=ACAA2 PE=1 SV=2 - [THIM_HUMAN]	53.65	2	22	22	228	1.034	1.050	1.069	1.083	637.82	53.65	35	228	397	41.9	8.09
O60337	E3 ubiquitin-protein ligase MARCH6 OS=Homo sapiens GN=MARCH6 PE=1 SV=2 - [MARH6_HUMAN]	0.99	1	1	1	3	1.007	0.590	1.064	1.083	4.78	0.99	2	3	910	102.5	6.28
Q9NTX7	E3 ubiquitin-protein ligase RNF146 OS=Homo sapiens GN=RNF146 PE=1 SV=1 - [RN146_HUMAN]	5.29	1	2	2	5	0.831	1.010	0.947	1.083	13.80	5.29	2	5	359	38.9	5.33
Q9Y5X3	Sorting nexin-5 OS=Homo sapiens GN=SNX5 PE=1 SV=1 - [SNX5_HUMAN]	40.10	1	16	18	56	1.160	0.988	0.880	1.083	131.29	40.10	28	56	404	46.8	6.76
Q7Z6J9	tRNA-splicing endonuclease subunit Sen54 OS=Homo sapiens GN=TSEN54 PE=1 SV=3 - [SEN54_HUMAN]	1.90	1	1	1	4	0.729	1.199	1.110	1.083	6.13	1.90	2	4	526	58.8	7.91
Q9NS00	Glycoprotein-N-acetylgalactosamine 3-beta-galactosyltransferase 1 OS=Homo sapiens GN=C1GALT1 PE=1 SV=1 - [C1GLT_HUMAN]	3.31	1	1	1	1	1.746	1.577	1.002	1.083	2.15	3.31	1	1	363	42.2	6.64

Q9Y230	RuvB-like 2 OS=Homo sapiens GN=RUVBL2 PE=1 SV=3 - [RUVB2_HUMAN]	55.08	1	25	25	89	0.886	1.086	0.935	1.083	221.28	55.08	39	89	463	51.1	5.64
Q92995	Ubiquitin carboxyl-terminal hydrolase 13 OS=Homo sapiens GN=USP13 PE=1 SV=2 - [UBP13_HUMAN]	4.75	1	3	3	4	0.528	1.566	1.098	1.083	5.86	4.75	4	4	863	97.3	5.53
Q8TAG9	Exocyst complex component 6 OS=Homo sapiens GN=EXOC6 PE=1 SV=3 - [EXOC6_HUMAN]	5.10	1	3	4	12	0.859	0.959	1.124	1.084	17.04	5.10	6	12	804	93.7	6.20
Q3KQV9	UDP-N-acetylhexosamine pyrophosphorylase-like protein 1 OS=Homo sapiens GN=UAP1L1 PE=1 SV=2 - [UAP1L_HUMAN]	9.07	1	4	4	13	1.223	1.189	1.360	1.084	18.84	9.07	8	13	507	57.0	6.32
Q7Z422	SUZ domain-containing protein 1 OS=Homo sapiens GN=SZRD1 PE=1 SV=1 - [SZRD1_HUMAN]	51.32	1	4	4	12	0.906	1.253	1.373	1.084	33.62	51.32	5	12	152	17.0	8.95
Q9NQG6	Mitochondrial dynamics protein MID51 OS=Homo sapiens GN=MIEF1 PE=1 SV=1 - [MID51_HUMAN]	4.32	1	2	2	2	1.174	0.778	0.896	1.084	0.00	4.32	2	2	463	51.3	7.64
O43681	ATPase ASNA1 OS=Homo sapiens GN=ASNA1 PE=1 SV=2 - [ASNA_HUMAN]	22.99	1	6	6	57	0.993	1.130	1.015	1.084	176.91	22.99	10	57	348	38.8	4.91
Q13163	Dual specificity mitogen-activated protein kinase kinase 5 OS=Homo sapiens GN=MAP2K5 PE=1 SV=2 - [MP2K5_HUMA	2.68	1	1	1	2	1.203	0.886	1.223	1.084	0.00	2.68	1	2	448	50.1	6.39

Q9H4L4	Sentrin-specific protease 3 OS=Homo sapiens GN=SENP3 PE=1 SV=2 - [SENP3_HUMAN]	5.23	1	3	3	4	1.025	0.963	0.918	1.084	3.89	5.23	4	4	574	65.0	8.56
Q8IXQ4	GPALPP motifs-containing protein 1 OS=Homo sapiens GN=GPALPP1 PE=1 SV=1 - [GPAM1_HUMAN]	2.94	1	1	1	1	0.828	0.768	0.820	1.084	2.37	2.94	1	1	340	38.1	5.41
P30491	HLA class I histocompatibility antigen, B-53 alpha chain OS=Homo sapiens GN=HLA-B PE=1 SV=1 - [1B53_HUMAN]	38.12	6	1	13	153	0.581	1.122	0.968	1.084	353.38	38.12	19	153	362	40.5	6.46
P28838	Cytosol aminopeptidase OS=Homo sapiens GN=LAP3 PE=1 SV=3 - [AMPL_HUMAN]	59.73	1	30	30	171	1.773	1.035	1.023	1.084	459.81	59.73	49	171	519	56.1	7.93
Q96HE7	ERO1-like protein alpha OS=Homo sapiens GN=ERO1L PE=1 SV=2 - [ERO1A_HUMAN]	42.31	1	15	17	108	1.076	0.853	0.756	1.085	278.19	42.31	27	108	468	54.4	5.68
Q9H270	Vacuolar protein sorting-associated protein 11 homolog OS=Homo sapiens GN=VPS11 PE=1 SV=1 - [VPS11_HUMAN]	10.73	1	9	10	27	0.907	0.846	0.925	1.085	51.81	10.73	15	27	941	107.8	7.05
P50213	Isocitrate dehydrogenase [NAD] subunit alpha, mitochondrial OS=Homo sapiens GN=IDH3A PE=1 SV=1 - [IDH3A_HUMAN]	33.88	1	14	14	51	1.186	1.054	0.953	1.085	115.99	33.88	20	51	366	39.6	6.92
Q15554	Telomeric repeat-binding factor 2 OS=Homo sapiens GN=TERF2 PE=1 SV=3 - [TERF2_HUMAN	1.29	1	1	1	2	0.665	1.525	0.927	1.085	3.44	1.29	2	2	542	59.6	9.35
Q6PML9	Zinc transporter 9 OS=Homo sapiens GN=SLC30A9 PE=1 SV=1 - [ZNT9_HUMAN]	14.26	1	7	7	15	1.130	1.137	1.157	1.085	33.39	14.26	9	15	568	63.5	8.32

Q9Y6D6	Brefeldin A-inhibited guanine nucleotide-exchange protein 1 OS=Homo sapiens GN=ARFGEF1 PE=1 SV=2 - [BIG1_HUMAN]	8.33	1	10	14	43	1.071	0.764	0.841	1.085	88.95	8.33	22	43	1849	208.6	5.85
P32455	Interferon-induced guanylate-binding protein 1 OS=Homo sapiens GN=GBP1 PE=1 SV=2 - [GBP1_HUMAN]	43.75	2	27	33	184	1.490	0.866	1.017	1.085	435.72	43.75	52	184	592	67.9	6.32
Q9UNP9	Peptidyl-prolyl cis-trans isomerase E OS=Homo sapiens GN=PPIE PE=1 SV=1 - [PPIE_HUMAN]	19.60	1	3	4	5	0.841	1.129	1.236	1.085	13.93	19.60	5	5	301	33.4	5.60
Q96JK2	DDB1- and CUL4-associated factor 5 OS=Homo sapiens GN=DCAF5 PE=1 SV=2 - [DCAF5_HUMAN	1.49	1	1	1	4	0.934	0.920	1.121	1.085	14.39	1.49	2	4	942	103.9	5.76
O95236	Apolipoprotein L3 OS=Homo sapiens GN=APOL3 PE=2 SV=3 - [APOL3_HUMAN]	2.74	1	1	1	4	1.006	0.648	1.034	1.085	10.90	2.74	2	4	402	44.3	8.16
Q9Y520	Protein PRRC2C OS=Homo sapiens GN=PRRC2C PE=1 SV=4 - [PRC2C_HUMAN	18.13	1	43	49	135	0.957	1.046	1.024	1.085	303.55	18.13	74	135	2896	316.7	9.13
P49458	Signal recognition particle 9 kDa protein OS=Homo sapiens GN=SRP9 PE=1 SV=2 - [SRP09_HUMAN]	32.56	1	3	3	14	0.845	1.200	0.854	1.086	31.19	32.56	6	14	86	10.1	7.97
Q15722	Leukotriene B4 receptor 1 OS=Homo sapiens GN=LTB4R PE=1 SV=2 - [LT4R1_HUMAN]	2.56	1	1	1	1	1.041	1.039	0.906	1.086	2.34	2.56	1	1	352	37.5	11.11
Q01167	Forkhead box protein K2 OS=Homo sapiens GN=FOXK2 PE=1 SV=3 - [FOXK2_HUMAN]	3.79	1	2	3	7	1.089	1.307	1.039	1.086	10.83	3.79	5	7	660	69.0	9.54

Q96IG2	F-box/LRR-repeat protein 20 OS=Homo sapiens GN=FBXL20 PE=1 SV=2 - [FXL20_HUMAN]	3.21	1	1	2	2	1.240	1.163	1.641	1.086	4.58	3.21	2	2	436	48.4	7.49
Q6UWU2	Beta-galactosidase-1-like protein OS=Homo sapiens GN=GLB1L PE=2 SV=1 - [GLB1L_HUMAN]	3.67	1	1	3	5	0.732	0.418	0.414	1.086	12.08	3.67	3	5	654	74.1	8.92
Q9BRQ8	Apoptosis-inducing factor 2 OS=Homo sapiens GN=AIFM2 PE=1 SV=1 - [AIFM2_HUMAN]	4.83	1	2	2	5	2.018	0.837	0.688	1.086	14.27	4.83	3	5	373	40.5	9.11
Q8IUD6	E3 ubiquitin-protein ligase RNF135 OS=Homo sapiens GN=RNF135 PE=1 SV=2 - [RN135_HUMAN]	6.94	1	1	2	2	0.981	0.797	1.169	1.086	5.17	6.94	2	2	432	47.9	6.81
P14314	Glucosidase 2 subunit beta OS=Homo sapiens GN=PRKCSH PE=1 SV=2 - [GLU2B_HUMAN]	32.39	1	23	23	317	0.968	1.061	1.047	1.086	771.00	32.39	38	317	528	59.4	4.41
Q86VY4	Testis-specific Y-encoded-like protein 5 OS=Homo sapiens GN=TSPYL5 PE=1 SV=2 - [TSYL5_HUMAN]	8.15	1	3	3	7	0.731	1.258	1.618	1.086	13.89	8.15	4	7	417	45.1	9.54
Q6STE5	SWI/SNF-related matrix-associated actin-dependent regulator of chromatin subfamily D member 3 OS=Homo sapiens GN=SMARCD3 PE=1 SV=1 - [SMRD3_HUMA	8.70	2	1	3	8	0.867	1.042	0.939	1.087	22.09	8.70	5	8	483	55.0	9.35
Q969S3	Zinc finger protein 622 OS=Homo sapiens GN=ZNF622 PE=1 SV=1 - [ZN622_HUMAN]	18.03	1	7	7	14	0.844	0.868	0.915	1.087	28.68	18.03	10	14	477	54.2	6.15

Q6P2Q9	Pre-mRNA-processing-splicing factor 8 OS=Homo sapiens GN=PRPF8 PE=1 SV=2 - [PRP8_HUMAN]	19.19	2	41	41	113	1.021	1.034	0.892	1.087	287.29	19.19	63	113	2335	273.4	8.84
P22090	40S ribosomal protein S4, Y isoform 1 OS=Homo sapiens GN=RPS4Y1 PE=1 SV=2 - [RS4Y1_HUMAN]	23.57	1	2	10	44	0.869	0.768	1.532	1.087	114.40	23.57	16	44	263	29.4	10.24
Q6ICB4	Sesquipedalian-2 OS=Homo sapiens GN=FAM109B PE=1 SV=1 - [SESQ2_HUMAN]	6.56	1	2	2	3	0.963	0.938	0.873	1.087	4.48	6.56	3	3	259	28.3	7.11
Q86YS6	Ras-related protein Rab-43 OS=Homo sapiens GN=RAB43 PE=1 SV=1 - [RAB43_HUMAN]	26.42	3	3	7	37	1.337	0.846	1.196	1.087	83.01	26.42	12	37	212	23.3	5.64
P12259	Coagulation factor V OS=Homo sapiens GN=F5 PE=1 SV=4 - [FA5_HUMAN]	0.45	1	1	1	1	0.703	0.895	1.250	1.087	0.00	0.45	1	1	2224	251.5	6.05
Q02978	Mitochondrial 2-oxoglutarate/malate carrier protein OS=Homo sapiens GN=SLC25A11 PE=1 SV=3 - [M2OM_HUMAN]	25.16	1	8	8	20	1.389	0.771	0.767	1.087	49.12	25.16	13	20	314	34.0	9.91
Q9BVS4	Serine/threonine-protein kinase RIO2 OS=Homo sapiens GN=RIOK2 PE=1 SV=2 - [RIOK2_HUMAN]	9.96	1	4	4	5	0.855	1.109	1.043	1.087	13.07	9.96	4	5	552	63.2	5.94
Q96MF7	E3 SUMO-protein ligase NSE2 OS=Homo sapiens GN=NSMCE2 PE=1 SV=2 - [NSE2_HUMAN]	12.15	1	3	3	3	1.026	0.564	0.932	1.087	6.50	12.15	3	3	247	27.9	7.74
Q14997	Proteasome activator complex subunit 4 OS=Homo sapiens GN=PSME4 PE=1 SV=2 - [PSME4_HUMAN]	6.29	2	10	10	16	1.053	0.694	1.077	1.088	35.84	6.29	13	16	1843	211.2	6.90

Q9HCI5	Melanoma-associated antigen E1 OS=Homo sapiens GN=MAGEE1 PE=1 SV=2 - [MAGE1_HUMAN]	1.46	1	1	1	3	1.619	0.859	1.582	1.088	5.08	1.46	1	3	957	103.2	5.33
Q8IVB5	LIX1-like protein OS=Homo sapiens GN=LIX1L PE=2 SV=1 - [LIX1L_HUMAN]	13.35	1	3	3	18	1.388	1.068	1.301	1.088	27.13	13.35	6	18	337	36.5	8.56
Q9Y2S0	DNA-directed RNA polymerases I and III subunit RPAC2 OS=Homo sapiens GN=POLR1D PE=1 SV=1 - [RPAC2_HUMAN]	18.80	1	2	2	7	0.938	0.809	0.720	1.088	19.00	18.80	4	7	133	15.2	5.80
Q96FX7	tRNA (adenine(58)-N(1))-methyltransferase catalytic subunit TRMT61A OS=Homo sapiens GN=TRMT61A PE=1 SV=1 - [TRM61_HUMAN]	4.84	1	1	1	1	0.918	1.201	0.714	1.088	4.13	4.84	1	1	289	31.4	7.36
Q5VVP1	Spermatogenesis-associated protein 31A6 OS=Homo sapiens GN=SPATA31A6 PE=3 SV=1 - [S31A6_HUMAN]	1.27	7	1	1	1	4.024	2.815	1.544	1.088	0.00	1.27	1	1	1343	147.7	8.70
Q99735	Microsomal glutathione S-transferase 2 OS=Homo sapiens GN=MGST2 PE=1 SV=1 - [MGST2_HUMAN]	9.52	1	1	1	5	0.827	0.700	1.135	1.088	10.20	9.52	2	5	147	16.6	9.55
Q6N069	N-alpha-acetyltransferase 16, NatA auxiliary subunit OS=Homo sapiens GN=NAA16 PE=1 SV=2 - [NAA16_HUMAN]	11.69	1	5	12	25	1.102	0.879	1.168	1.088	57.01	11.69	15	25	864	101.4	7.87
Q9HAU4	E3 ubiquitin-protein ligase SMURF2 OS=Homo sapiens GN=SMURF2 PE=1 SV=1 - [SMUF2_HUMAN]	16.31	2	10	10	25	1.085	0.706	1.173	1.088	68.85	16.31	15	25	748	86.1	7.96

Q9Y237	Peptidyl-prolyl cis-trans isomerase NIMA-interacting 4 OS=Homo sapiens GN=PIN4 PE=1 SV=1 - [PIN4_HUMAN]	31.30	1	5	5	13	0.914	1.573	1.033	1.088	35.76	31.30	6	13	131	13.8	9.77
Q96KP4	Cytosolic non-specific dipeptidase OS=Homo sapiens GN=CNDP2 PE=1 SV=2 - [CNDP2_HUMAN]	46.95	1	19	19	100	1.198	0.868	0.779	1.088	231.50	46.95	29	100	475	52.8	5.97
Q8N6S5	ADP-ribosylation factor-like protein 6-interacting protein 6 OS=Homo sapiens GN=ARL6IP6 PE=1 SV=1 - [AR6P6_HUMAN]	17.70	1	3	3	9	1.228	1.756	1.112	1.088	15.30	17.70	6	9	226	24.7	6.33
Q9HDB5	Neurexin-3-beta OS=Homo sapiens GN=NRXN3 PE=1 SV=4 - [NRX3B_HUMAN	4.71	2	1	1	1	0.739	0.630	1.688	1.088	2.05	4.71	1	1	637	69.3	7.21
Q8IVE3	Pleckstrin homology domain-containing family H member 2 OS=Homo sapiens GN=PLEKHH2 PE=2 SV=2 - [PKHH2_HUMAN]	2.01	1	3	3	5	1.558	0.563	1.147	1.089	8.64	2.01	4	5	1493	168.1	7.56
Q99733	Nucleosome assembly protein 1-like 4 OS=Homo sapiens GN=NAP1L4 PE=1 SV=1 - [NP1L4_HUMAN]	33.33	1	10	11	118	1.102	1.251	1.235	1.089	328.96	33.33	20	118	375	42.8	4.69
Q9H2K0	Translation initiation factor IF-3, mitochondrial OS=Homo sapiens GN=MTIF3 PE=1 SV=4 - [IF3M_HUMAN]	5.76	1	2	2	4	1.137	1.380	1.173	1.089	9.67	5.76	3	4	278	31.7	9.69
Q92615	La-related protein 4B OS=Homo sapiens GN=LARP4B PE=1 SV=3 - [LAR4B_HUMAN]	17.89	1	11	11	27	1.137	0.845	1.012	1.089	64.31	17.89	18	27	738	80.5	6.92

Q92805	Golgin subfamily A member 1 OS=Homo sapiens GN=GOLGA1 PE=1 SV=3 - [GOGA1_HUMAN]	15.25	2	10	11	38	1.012	0.925	1.230	1.089	77.79	15.25	17	38	767	88.1	5.27
Q9Y680	Peptidyl-prolyl cis-trans isomerase FKBP7 OS=Homo sapiens GN=FKBP7 PE=1 SV=1 - [FKBP7_HUMAN]	54.44	1	17	17	72	0.735	0.707	0.644	1.089	191.75	54.44	27	72	259	30.0	6.54
O95786	Probable ATP-dependent RNA helicase DDX58 OS=Homo sapiens GN=DDX58 PE=1 SV=2 - [DDX58_HUMAN	9.19	2	8	8	24	3.576	1.058	1.306	1.089	54.14	9.19	12	24	925	106.5	6.40
Q9UQ03	Coronin-2B OS=Homo sapiens GN=CORO2B PE=2 SV=4 - [COR2B_HUMAN]	12.50	1	6	6	11	0.768	1.729	1.796	1.089	29.80	12.50	7	11	480	54.9	8.27
Q9P2E5	Chondroitin sulfate glucuronyltransferase OS=Homo sapiens GN=CHPF2 PE=2 SV=2 - [CHPF2_HUMAN]	10.75	1	7	7	19	0.720	0.747	0.846	1.089	48.58	10.75	11	19	772	85.9	7.83
O60832	H/ACA ribonucleoprotein complex subunit 4 OS=Homo sapiens GN=DKC1 PE=1 SV=3 - [DKC1_HUMAN]	19.26	1	11	11	35	0.970	1.149	0.808	1.089	87.17	19.26	19	35	514	57.6	9.42
P47985	Cytochrome b-c1 complex subunit Rieske, mitochondrial OS=Homo sapiens GN=UQCRFS1 PE=1 SV=2 - [UCRI_HUMAN]	21.90	2	6	6	12	0.692	1.059	0.736	1.089	34.97	21.90	9	12	274	29.6	8.32
Q13496	Myotubularin OS=Homo sapiens GN=MTM1 PE=1 SV=2 - [MTM1_HUMAN]	5.97	1	3	3	5	1.237	1.233	1.330	1.089	9.72	5.97	5	5	603	69.9	8.18
P56851	Epididymal secretory protein E3-beta OS=Homo sapiens GN=EDDM3B PE=2 SV=2 - [EP3B_HUMAN]	4.08	1	1	1	1	1.069	0.697	0.800	1.089	2.04	4.08	1	1	147	17.6	6.99

Q6ZUT6	Uncharacterized protein C15orf52 OS=Homo sapiens GN=C15orf52 PE=1 SV=1 - [CO052_HUMAN]	11.42	1	6	6	12	1.359	2.570	2.798	1.089	20.48	11.42	8	12	534	57.3	9.13
P53041	Serine/threonine-protein phosphatase 5 OS=Homo sapiens GN=PPP5C PE=1 SV=1 - [PPP5_HUMAN]	29.26	2	16	16	48	1.016	0.843	0.944	1.089	110.43	29.26	24	48	499	56.8	6.28
Q9Y3D3	28S ribosomal protein S16, mitochondrial OS=Homo sapiens GN=MRPS16 PE=1 SV=1 - [RT16_HUMAN]	13.14	1	1	1	7	0.824	1.101	1.013	1.089	15.00	13.14	2	7	137	15.3	9.50
Q8WVF1	Protein OSCP1 OS=Homo sapiens GN=OSCP1 PE=1 SV=4 - [OSCP1_HUMAN]	4.88	1	2	2	3	0.997	0.895	1.068	1.089	6.23	4.88	3	3	389	44.6	5.83
A5PLL7	Transmembrane protein 189 OS=Homo sapiens GN=TMEM189 PE=1 SV=3 - [TM189_HUMAN]	4.07	1	1	1	2	1.256	0.685	1.031	1.089	2.95	4.07	2	2	270	31.1	6.83
Q99081	Transcription factor 12 OS=Homo sapiens GN=TCF12 PE=1 SV=1 - [HTF4_HUMAN]	4.11	2	2	2	6	0.826	1.183	1.090	1.089	20.98	4.11	4	6	682	72.9	7.02
P41227	N-alpha-acetyltransferase 10 OS=Homo sapiens GN=NAA10 PE=1 SV=1 - [NAA10_HUMAN	45.11	1	7	9	25	0.853	1.103	1.219	1.090	76.92	45.11	13	25	235	26.4	5.64
P46777	60S ribosomal protein L5 OS=Homo sapiens GN=RPL5 PE=1 SV=3 - [RL5_HUMAN]	39.06	1	19	20	137	0.994	1.191	1.009	1.090	340.81	39.06	33	137	297	34.3	9.72
Q13247	Serine/arginine-rich splicing factor 6 OS=Homo sapiens GN=SRSF6 PE=1 SV=2 - [SRSF6_HUMAN]	24.42	1	7	11	59	0.885	1.119	0.959	1.090	112.45	24.42	18	59	344	39.6	11.43

Q93052	Lipoma-preferred partner OS=Homo sapiens GN=LPP PE=1 SV=1 - [LPP_HUMAN]	38.07	1	20	20	164	1.438	1.429	1.609	1.090	427.51	38.07	35	164	612	65.7	7.37
Q14185	Dedicator of cytokinesis protein 1 OS=Homo sapiens GN=DOCK1 PE=1 SV=2 - [DOCK1_HUMAN]	15.39	1	25	28	59	1.181	1.025	0.923	1.090	128.51	15.39	39	59	1865	215.2	7.56
Q96Q42	Alsin OS=Homo sapiens GN=ALS2 PE=1 SV=2 - [ALS2_HUMAN]	1.63	1	2	2	3	0.794	0.803	0.905	1.090	7.60	1.63	2	3	1657	183.5	6.27
Q96JK4	HHIP-like protein 1 OS=Homo sapiens GN=HHIPL1 PE=2 SV=2 - [HIPL1_HUMAN]	1.41	1	1	1	2	1.503	0.227	0.445	1.090	4.92	1.41	1	2	782	86.7	7.58
Q9Y4G8	Rap guanine nucleotide exchange factor 2 OS=Homo sapiens GN=RAPGEF2 PE=1 SV=1 - [RPGF2_HUMAN	3.54	1	3	4	18	1.396	1.324	1.097	1.090	41.67	3.54	6	18	1499	167.3	6.67
Q02224	Centromere-associated protein E OS=Homo sapiens GN=CENPE PE=1 SV=2 - [CENPE_HUMAN]	6.52	3	10	19	34	1.194	1.376	0.915	1.090	46.16	6.52	20	34	2701	316.2	5.64
O75390	Citrate synthase, mitochondrial OS=Homo sapiens GN=CS PE=1 SV=2 - [CISY_HUMAN]	28.54	1	13	13	77	1.168	1.489	1.129	1.091	195.50	28.54	20	77	466	51.7	8.32
Q9ULH7	MKL/myocardin-like protein 2 OS=Homo sapiens GN=MKL2 PE=1 SV=3 - [MKL2_HUMAN]	8.82	3	4	8	43	1.428	1.875	1.264	1.091	91.46	8.82	12	43	1088	118.1	6.28
Q07666	KH domain-containing, RNA-binding, signal transduction-associated protein 1 OS=Homo sapiens GN=KHDRBS1 PE=1 SV=1 - [KHDR1_HUMAN]	25.06	2	10	13	60	1.014	1.449	0.984	1.091	144.54	25.06	17	60	443	48.2	8.66

Q9NWY4	UPF0609 protein C4orf27 OS=Homo sapiens GN=C4orf27 PE=1 SV=2 - [CD027_HUMAN]	5.78	1	1	2	6	1.070	1.147	1.129	1.091	15.23	5.78	4	6	346	39.4	6.80
O15355	Protein phosphatase 1G OS=Homo sapiens GN=PPM1G PE=1 SV=1 - [PPM1G_HUMAN]	34.25	1	14	14	52	0.831	1.013	0.911	1.091	126.83	34.25	22	52	546	59.2	4.36
O75534	Cold shock domain-containing protein E1 OS=Homo sapiens GN=CSDE1 PE=1 SV=2 - [CSDE1_HUMAN]	31.20	1	28	28	106	0.844	0.931	1.023	1.091	260.12	31.20	45	106	798	88.8	6.25
Q9UBB9	Tuftelin-interacting protein 11 OS=Homo sapiens GN=TFIP11 PE=1 SV=1 - [TFP11_HUMAN]	5.62	1	3	4	7	0.837	1.113	1.464	1.091	12.47	5.62	4	7	837	96.8	5.67
Q9BVC5	Ashwin OS=Homo sapiens GN=C2orf49 PE=1 SV=1 - [ASHWN_HUMAN]	6.47	1	1	1	4	1.076	1.156	1.038	1.091	10.43	6.47	2	4	232	25.8	9.74
P62913	60S ribosomal protein L11 OS=Homo sapiens GN=RPL11 PE=1 SV=2 - [RL11_HUMAN]	42.13	1	6	6	52	1.126	1.115	1.016	1.091	118.11	42.13	12	52	178	20.2	9.60
Q8IVT5	Kinase suppressor of Ras 1 OS=Homo sapiens GN=KSR1 PE=1 SV=3 - [KSR1_HUMAN]	5.20	1	5	5	12	1.654	0.942	0.701	1.092	47.09	5.20	5	12	923	102.1	8.66
Q9NX70	Mediator of RNA polymerase II transcription subunit 29 OS=Homo sapiens GN=MED29 PE=1 SV=1 - [MED29_HUMAN]	9.50	1	2	2	2	0.967	0.878	1.094	1.092	4.56	9.50	2	2	200	21.1	6.29
O15504	Nucleoporin-like protein 2 OS=Homo sapiens GN=NUPL2 PE=1 SV=1 - [NUPL2_HUMAN]	7.57	1	2	2	4	1.039	1.218	1.110	1.092	6.90	7.57	4	4	423	44.8	9.19

Q8IWA6	Coiled-coil domain-containing protein 60 OS=Homo sapiens GN=CCDC60 PE=2 SV=2 - [CCD60_HUMAN]	1.27	1	1	1	1	1.084	0.847	1.089	1.092	2.02	1.27	1	1	550	63.1	9.69
Q8NEL9	Phospholipase DDHD1 OS=Homo sapiens GN=DDHD1 PE=1 SV=2 - [DDHD1_HUMAN]	4.44	1	3	5	6	0.609	0.825	1.108	1.092	11.25	4.44	5	6	900	100.4	5.67
Q8TDD1	ATP-dependent RNA helicase DDX54 OS=Homo sapiens GN=DDX54 PE=1 SV=2 - [DDX54_HUMAN	12.03	2	7	9	24	0.664	0.851	0.610	1.092	36.02	12.03	12	24	881	98.5	10.02
P51692	Signal transducer and activator of transcription 5B OS=Homo sapiens GN=STAT5B PE=1 SV=2 - [STA5B_HUMAN]	17.53	2	3	12	32	0.924	1.061	1.108	1.092	65.83	17.53	19	32	787	89.8	6.05
Q9UI15	Transgelin-3 OS=Homo sapiens GN=TAGLN3 PE=1 SV=2 - [TAGL3_HUMAN]	18.09	1	2	3	79	1.330	1.787	1.077	1.092	193.07	18.09	5	79	199	22.5	7.33
Q12899	Tripartite motif-containing protein 26 OS=Homo sapiens GN=TRIM26 PE=2 SV=1 - [TRI26_HUMAN]	14.10	1	6	6	14	1.095	1.024	1.420	1.092	36.52	14.10	9	14	539	62.1	5.03
P62318	Small nuclear ribonucleoprotein Sm D3 OS=Homo sapiens GN=SNRPD3 PE=1 SV=1 - [SMD3_HUMAN]	31.75	1	3	3	15	1.025	1.134	0.915	1.092	49.57	31.75	6	15	126	13.9	10.32
O15523	ATP-dependent RNA helicase DDX3Y OS=Homo sapiens GN=DDX3Y PE=1 SV=2 - [DDX3Y_HUMAN	36.97	1	2	24	149	1.484	0.740	1.172	1.093	356.65	36.97	39	149	660	73.1	7.55
O75475	PC4 and SFRS1-interacting protein OS=Homo sapiens GN=PSIP1 PE=1 SV=1 - [PSIP1_HUMAN	25.47	1	14	15	44	1.286	2.069	0.986	1.093	94.85	25.47	25	44	530	60.1	9.13

Q8WU90	Zinc finger CCCH domain-containing protein 15 OS=Homo sapiens GN=ZC3H15 PE=1 SV=1 - [ZC3HF_HUMAN]	28.17	1	11	11	40	0.733	1.122	0.950	1.093	106.04	28.17	15	40	426	48.6	5.31
Q05932	Folylpolyglutamate synthase, mitochondrial OS=Homo sapiens GN=FPGS PE=1 SV=3 - [FOLC_HUMAN]	1.70	1	1	1	1	0.962	0.745	0.864	1.093	2.37	1.70	1	1	587	64.6	7.94
P62314	Small nuclear ribonucleoprotein Sm D1 OS=Homo sapiens GN=SNRPD1 PE=1 SV=1 - [SMD1_HUMAN]	27.73	1	3	3	14	0.842	0.891	0.925	1.093	30.64	27.73	4	14	119	13.3	11.56
O75489	NADH dehydrogenase [ubiquinone] iron-sulfur protein 3, mitochondrial OS=Homo sapiens GN=NDUFS3 PE=1 SV=1 - [NDUS3_HUMAN]	33.71	1	9	9	27	0.683	1.037	0.894	1.093	60.21	33.71	13	27	264	30.2	7.50
P55795	Heterogeneous nuclear ribonucleoprotein H2 OS=Homo sapiens GN=HNRNPH2 PE=1 SV=1 - [HNRH2_HUMAN]	43.65	1	6	14	138	1.161	1.284	1.283	1.093	451.88	43.65	22	138	449	49.2	6.30
A0AV96	RNA-binding protein 47 OS=Homo sapiens GN=RBM47 PE=1 SV=2 - [RBM47_HUMAN]	1.35	1	1	1	2	0.889	0.971	0.904	1.093	4.63	1.35	1	2	593	64.1	7.68
Q9UL16	Protein CFAP45, mitochondrial OS=Homo sapiens GN=CFAP45 PE=2 SV=2 - [CFA45_HUMAN]	5.81	2	3	4	5	1.090		1.447	1.093	10.95	5.81	4	5	551	65.7	8.90
Q9BWM7	Sideroflexin-3 OS=Homo sapiens GN=SFXN3 PE=1 SV=2 - [SFXN3_HUMAN]	33.54	1	8	9	42	1.320	1.101	1.098	1.094	97.52	33.54	15	42	325	36.0	9.09

Q9Y2D5	A-kinase anchor protein 2 OS=Homo sapiens GN=AKAP2 PE=1 SV=3 - [AKAP2_HUMAN]	51.46	1	38	38	254	1.395	0.774	1.048	1.094	639.96	51.46	60	254	859	94.6	5.11
O96018	Amyloid beta A4 precursor protein-binding family A member 3 OS=Homo sapiens GN=APBA3 PE=1 SV=1 - [APBA3_HUMAN]	5.74	1	1	1	1	2.674	1.228	1.651	1.094	2.23	5.74	1	1	575	61.4	4.94
Q9H6Y2	WD repeat-containing protein 55 OS=Homo sapiens GN=WDR55 PE=1 SV=2 - [WDR55_HUMAN]	5.74	1	2	2	3	0.644	1.101	1.282	1.094	6.66	5.74	3	3	383	42.0	4.92
Q92545	Transmembrane protein 131 OS=Homo sapiens GN=TMEM131 PE=1 SV=3 - [TM131_HUMAN]	1.54	1	2	2	2	1.714	1.703	0.969	1.094	4.74	1.54	2	2	1883	205.0	8.59
Q6ZW31	Rho GTPase-activating protein SYDE1 OS=Homo sapiens GN=SYDE1 PE=1 SV=1 - [SYDE1_HUMAN]	4.08	1	3	3	4	0.942	1.059	1.058	1.094	6.64	4.08	4	4	735	79.7	8.43
O75477	Erlin-1 OS=Homo sapiens GN=ERLIN1 PE=1 SV=1 - [ERLN1_HUMAN]	37.86	1	9	16	84	1.032	0.969	0.768	1.094	188.07	37.86	28	84	346	38.9	7.87
Q9UKD2	mRNA turnover protein 4 homolog OS=Homo sapiens GN=MRTO4 PE=1 SV=2 - [MRT4_HUMAN]	29.29	1	6	6	16	0.722	0.670	0.656	1.094	10.46	29.29	11	16	239	27.5	8.29
Q01740	Dimethylaniline monooxygenase [N-oxide-forming] 1 OS=Homo sapiens GN=FMO1 PE=2 SV=3 - [FMO1_HUMAN]	1.13	1	1	1	1	0.588	1.123	1.083	1.094	2.69	1.13	1	1	532	60.3	7.20
P55854	Small ubiquitin-related modifier 3 OS=Homo sapiens GN=SUMO3 PE=1 SV=2 - [SUMO3_HUMA	24.27	1	2	3	29	1.487	1.576	1.090	1.094	57.95	24.27	5	29	103	11.6	5.49

Q9Y3E1	Hepatoma-derived growth factor-related protein 3 OS=Homo sapiens GN=HDGFRP3 PE=1 SV=1 - [HDGR3_HUMAN]	27.59	1	4	5	13	1.040	1.153	1.017	1.094	29.02	27.59	8	13	203	22.6	7.99
Q13601	KRR1 small subunit processome component homolog OS=Homo sapiens GN=KRR1 PE=1 SV=4 - [KRR1_HUMAN]	13.91	1	5	5	11	0.973	0.890	0.758	1.094	18.12	13.91	8	11	381	43.6	9.77
Q9UHB9	Signal recognition particle subunit SRP68 OS=Homo sapiens GN=SRP68 PE=1 SV=2 - [SRP68_HUMAN]	40.67	1	25	25	104	0.830	0.912	0.957	1.094	256.86	40.67	40	104	627	70.7	8.56
Q16254	Transcription factor E2F4 OS=Homo sapiens GN=E2F4 PE=1 SV=2 - [E2F4_HUMAN]	2.18	1	1	1	2	0.742	0.649	1.030	1.094	4.16	2.18	1	2	413	43.9	4.75
Q8ND82	Zinc finger protein 280C OS=Homo sapiens GN=ZNF280C PE=1 SV=1 - [Z280C_HUMAN]	1.90	1	1	1	1	0.934	1.033	0.895	1.094	0.00	1.90	1	1	737	83.0	9.11
Q9Y399	28S ribosomal protein S2, mitochondrial OS=Homo sapiens GN=MRPS2 PE=1 SV=1 - [RT02_HUMAN]	14.19	1	5	5	14	0.707	0.768	1.001	1.094	37.77	14.19	6	14	296	33.2	9.26
Q8IYB3	Serine/arginine repetitive matrix protein 1 OS=Homo sapiens GN=SRRM1 PE=1 SV=2 - [SRRM1_HUMAN]	14.71	1	8	10	55	1.137	1.339	0.912	1.094	89.05	14.71	14	55	904	102.3	11.84
Q13936	Voltage-dependent L-type calcium channel subunit alpha-1C OS=Homo sapiens GN=CACNA1C PE=1 SV=4 - [CAC1C_HUMAN]	1.67	2	3	3	4	0.832	1.119	3.857	1.095	9.93	1.67	3	4	2221	248.8	6.76

Q92688	Acidic leucine-rich nuclear phosphoprotein 32 family member B OS=Homo sapiens GN=ANP32B PE=1 SV=1 - [AN32B_HUMAN]	29.48	2	4	10	49	0.932	1.073	0.769	1.095	91.25	29.48	16	49	251	28.8	4.06
Q86W92	Liprin-beta-1 OS=Homo sapiens GN=PPFIBP1 PE=1 SV=2 - [LIPB1_HUMAN]	22.75	3	14	18	54	0.881	1.113	1.080	1.095	145.92	22.75	28	54	1011	114.0	5.55
Q9UJA5	tRNA (adenine(58)-N(1))-methyltransferase non-catalytic subunit TRM6 OS=Homo sapiens GN=TRMT6 PE=1 SV=1 - [TRM6_HUMAN]	7.65	1	4	4	5	0.493	1.041	1.009	1.095	6.29	7.65	5	5	497	55.8	7.55
O60293	Zinc finger C3H1 domain-containing protein OS=Homo sapiens GN=ZFC3H1 PE=1 SV=3 - [ZC3H1_HUMAN]	2.56	1	3	3	4	1.257	1.278	1.234	1.095	6.33	2.56	3	4	1989	226.2	8.13
O95741	Copine-6 OS=Homo sapiens GN=CPNE6 PE=1 SV=3 - [CPNE6_HUMAN]	3.23	4	1	2	10	0.725	1.170	1.343	1.095	23.30	3.23	2	10	557	62.0	5.52
P30876	DNA-directed RNA polymerase II subunit RPB2 OS=Homo sapiens GN=POLR2B PE=1 SV=1 - [RPB2_HUMAN]	17.38	1	15	15	41	0.874	1.149	0.959	1.095	99.61	17.38	22	41	1174	133.8	6.87
Q9HBH1	Peptide deformylase, mitochondrial OS=Homo sapiens GN=PDF PE=1 SV=1 - [DEFM_HUMAN]	5.76	1	2	2	3	1.175	1.627	1.218	1.095	6.32	5.76	3	3	243	27.0	9.16
P42858	Huntingtin OS=Homo sapiens GN=HTT PE=1 SV=2 - [HD_HUMAN]	3.79	1	12	13	31	0.966	0.674	0.723	1.095	56.52	3.79	20	31	3142	347.4	6.20

Q9P0J1	[Pyruvate dehydrogenase [acetyl-transferring]]-phosphatase 1, mitochondrial OS=Homo sapiens GN=PDP1 PE=1 SV=3 - [PDP1_HUMAN]	20.48	1	9	9	13	1.527	1.182	1.008	1.095	24.59	20.48	11	13	537	61.0	6.67
P13498	Cytochrome b-245 light chain OS=Homo sapiens GN=CYBA PE=1 SV=3 - [CY24A_HUMAN]	7.69	1	1	1	4	0.837	0.603	0.810	1.095	4.66	7.69	2	4	195	21.0	9.54
O94851	Protein-methionine sulfoxide oxidase MICAL2 OS=Homo sapiens GN=MICAL2 PE=1 SV=1 - [MICA2_HUMAN]	7.30	1	7	7	38	0.878	1.219	1.328	1.096	77.72	7.30	13	38	1124	126.6	8.65
O15066	Kinesin-like protein KIF3B OS=Homo sapiens GN=KIF3B PE=1 SV=1 - [KIF3B_HUMAN]	9.91	2	4	6	27	1.064	0.857	1.254	1.096	71.60	9.91	8	27	747	85.1	7.69
Q96PE1	G-protein coupled receptor 124 OS=Homo sapiens GN=GPR124 PE=1 SV=2 - [GP124_HUMAN]	9.42	1	8	8	11	0.883	1.556	1.683	1.096	22.38	9.42	9	11	1338	142.6	8.44
P23434	Glycine cleavage system H protein, mitochondrial OS=Homo sapiens GN=GCSH PE=1 SV=2 - [GCSH_HUMAN]	17.34	1	2	2	2	1.131	1.166	0.640	1.096	10.36	17.34	2	2	173	18.9	4.88
O43809	Cleavage and polyadenylation specificity factor subunit 5 OS=Homo sapiens GN=NUDT21 PE=1 SV=1 - [CPSF5_HUMAN]	28.19	1	7	7	27	1.148	1.208	0.944	1.096	66.34	28.19	11	27	227	26.2	8.82
Q07157	Tight junction protein ZO-1 OS=Homo sapiens GN=TJP1 PE=1 SV=3 - [ZO1_HUMAN]	27.06	1	39	39	146	1.216	1.207	1.166	1.096	333.83	27.06	64	146	1748	195.3	6.70

Q6NZY4	Zinc finger CCHC domain-containing protein 8 OS=Homo sapiens GN=ZCCHC8 PE=1 SV=2 - [ZCHC8_HUMAN]	5.23	1	3	3	6	1.139	1.057	0.824	1.096	12.27	5.23	4	6	707	78.5	4.87
P62328	Thymosin beta-4 OS=Homo sapiens GN=TMSB4X PE=1 SV=2 - [TYB4_HUMAN]	61.36	2	5	8	203	1.996	1.497	1.302	1.096	425.51	61.36	13	203	44	5.0	5.06
Q14554	Protein disulfide-isomerase A5 OS=Homo sapiens GN=PDIA5 PE=1 SV=1 - [PDIA5_HUMAN	34.87	1	16	16	76	1.186	0.755	0.721	1.097	197.33	34.87	28	76	519	59.6	7.91
O43148	mRNA cap guanine-N7 methyltransferase OS=Homo sapiens GN=RNMT PE=1 SV=1 - [MCES_HUMAN]	16.39	1	7	8	17	0.829	1.084	1.080	1.097	27.93	16.39	10	17	476	54.8	6.61
Q6Y7W6	PERQ amino acid-rich with GYF domain-containing protein 2 OS=Homo sapiens GN=GIGYF2 PE=1 SV=1 - [PERQ2_HUMAN]	14.09	2	14	16	59	0.986	1.232	1.237	1.097	170.10	14.09	24	59	1299	150.0	5.54
Q9P2J8	Zinc finger protein 624 OS=Homo sapiens GN=ZNF624 PE=2 SV=3 - [ZN624_HUMAN]	5.32	96	1	3	7				1.097	8.99	5.32	4	7	865	99.9	8.88
Q9BW62	Katanin p60 ATPase-containing subunit A-like 1 OS=Homo sapiens GN=KATNAL1 PE=1 SV=1 - [KATL1_HUMAN]	10.00	1	4	4	5	0.895	1.082	1.515	1.097	12.42	10.00	4	5	490	55.4	6.74
P42898	Methylenetetrahydrofolate reductase OS=Homo sapiens GN=MTHFR PE=1 SV=3 - [MTHR_HUMAN]	1.68	1	1	1	1	1.026	1.263	1.275	1.097	0.00	1.68	1	1	656	74.5	5.34
Q96J84	Kin of IRRE-like protein 1 OS=Homo sapiens GN=KIRREL PE=1 SV=2 - [KIRR1_HUMAN]	21.14	1	12	12	45	1.178	0.806	0.858	1.098	115.47	21.14	22	45	757	83.5	5.73

P07203	Glutathione peroxidase 1 OS=Homo sapiens GN=GPX1 PE=1 SV=4 - [GPX1_HUMAN]	46.31	1	7	7	60	1.774	0.773	0.698	1.098	135.38	46.31	12	60	203	22.1	6.55
P68371	Tubulin beta-4B chain OS=Homo sapiens GN=TUBB4B PE=1 SV=1 - [TBB4B_HUMAN]	69.66	1	2	23	1186	1.438	1.267	0.935	1.098	2598.17	69.66	41	1186	445	49.8	4.89
Q13439	Golgin subfamily A member 4 OS=Homo sapiens GN=GOLGA4 PE=1 SV=1 - [GOGA4_HUMAN]	25.43	1	53	56	179	0.789	0.912	0.864	1.098	375.16	25.43	89	179	2230	261.0	5.39
P04040	Catalase OS=Homo sapiens GN=CAT PE=1 SV=3 - [CATA_HUMAN]	46.87	1	24	24	168	0.594	0.991	0.694	1.098	470.80	46.87	43	168	527	59.7	7.39
Q8TC71	Mitochondria-eating protein OS=Homo sapiens GN=SPATA18 PE=1 SV=1 - [MIEAP_HUMAN]	1.49	1	1	1	2	1.171	0.930	0.785	1.098	2.11	1.49	2	2	538	61.1	8.63
Q96DV4	39S ribosomal protein L38, mitochondrial OS=Homo sapiens GN=MRPL38 PE=1 SV=2 - [RM38_HUMAN]	22.63	1	9	9	26	1.024	1.273	1.056	1.098	53.06	22.63	14	26	380	44.6	7.53
O94915	Protein furry homolog-like OS=Homo sapiens GN=FRYL PE=1 SV=2 - [FRYL_HUMAN]	3.75	1	8	9	15	1.110	1.140	1.194	1.098	17.82	3.75	12	15	3013	339.4	5.58
Q96AQ8	Mitochondrial calcium uniporter regulator 1 OS=Homo sapiens GN=MCUR1 PE=1 SV=1 - [MCUR1_HUMAN]	4.74	1	1	2	2	1.052	1.345	1.019	1.099	6.28	4.74	2	2	359	39.7	9.63
O14662	Syntaxin-16 OS=Homo sapiens GN=STX16 PE=1 SV=3 - [STX16_HUMAN]	21.23	1	5	5	15	0.970	0.937	0.960	1.099	42.83	21.23	9	15	325	37.0	6.11

Q12907	Vesicular integral-membrane protein VIP36 OS=Homo sapiens GN=LMAN2 PE=1 SV=1 - [LMAN2_HUMAN]	48.31	1	15	15	121	0.951	0.747	0.708	1.099	327.72	48.31	25	121	356	40.2	6.95
Q9Y4W6	AFG3-like protein 2 OS=Homo sapiens GN=AFG3L2 PE=1 SV=2 - [AFG32_HUMAN]	30.49	1	25	26	81	1.081	0.987	0.786	1.099	167.60	30.49	45	81	797	88.5	8.66
Q9BV38	WD repeat-containing protein 18 OS=Homo sapiens GN=WDR18 PE=1 SV=2 - [WDR18_HUMAN]	18.52	1	8	8	40	0.832	0.835	0.934	1.099	100.43	18.52	14	40	432	47.4	6.70
Q9H9B4	Sideroflexin-1 OS=Homo sapiens GN=SFXN1 PE=1 SV=4 - [SFXN1_HUMAN]	31.99	1	8	9	91	1.082	0.941	0.766	1.099	173.09	31.99	15	91	322	35.6	9.07
Q15746	Myosin light chain kinase, smooth muscle OS=Homo sapiens GN=MYLK PE=1 SV=4 - [MYLK_HUMAN]	36.47	3	64	65	470	1.404	1.593	1.056	1.099	1216.57	36.47	113	470	1914	210.6	6.15
P53778	Mitogen-activated protein kinase 12 OS=Homo sapiens GN=MAPK12 PE=1 SV=3 - [MK12_HUMAN]	8.45	3	2	3	8	0.849	0.669	0.564	1.099	16.00	8.45	5	8	367	41.9	6.39
Q9HCC0	Methylcrotonoyl-CoA carboxylase beta chain, mitochondrial OS=Homo sapiens GN=MCCC2 PE=1 SV=1 - [MCCB_HUMAN]	17.23	1	8	8	34	0.939	1.206	1.124	1.100	115.28	17.23	15	34	563	61.3	7.68
P61165	Transmembrane protein 258 OS=Homo sapiens GN=TMEM258 PE=1 SV=1 - [TM258_HUMAN]	10.13	1	1	1	4	0.825	0.898	0.813	1.100	8.87	10.13	2	4	79	9.1	5.83
Q9NR09	Baculoviral IAP repeat-containing protein 6 OS=Homo sapiens GN=BIRC6 PE=1 SV=2 - [BIRC6_HUMAN]	3.40	1	12	13	23	0.937	0.912	0.947	1.100	46.59	3.40	20	23	4857	529.9	6.05

Q9UJZ1	Stomatin-like protein 2, mitochondrial OS=Homo sapiens GN=STOML2 PE=1 SV=1 - [STML2_HUMAN]	34.83	1	13	13	56	1.002	0.936	0.680	1.100	143.14	34.83	23	56	356	38.5	7.39
Q9NZN5	Rho guanine nucleotide exchange factor 12 OS=Homo sapiens GN=ARHGEF12 PE=1 SV=1 - [ARHGC_HUMAN	17.81	1	22	22	71	1.046	0.999	0.945	1.100	186.19	17.81	33	71	1544	173.1	5.74
Q53F19	Uncharacterized protein C17orf85 OS=Homo sapiens GN=C17orf85 PE=1 SV=2 - [CQ085_HUMAN]	5.65	1	3	3	7	0.973	1.125	1.040	1.100	8.57	5.65	4	7	620	70.5	5.73
Q9H7N4	Splicing factor, arginine/serine-rich 19 OS=Homo sapiens GN=SCAF1 PE=1 SV=3 - [SFR19_HUMAN]	4.42	1	5	5	12	1.215	1.000	1.146	1.100	20.89	4.42	8	12	1312	139.2	9.25
Q14152	Eukaryotic translation initiation factor 3 subunit A OS=Homo sapiens GN=EIF3A PE=1 SV=1 - [EIF3A_HUMAN]	31.04	1	48	49	233	1.006	1.101	0.958	1.100	569.86	31.04	75	233	1382	166.5	6.79
O75937	DnaJ homolog subfamily C member 8 OS=Homo sapiens GN=DNAJC8 PE=1 SV=2 - [DNJC8_HUMAN]	43.08	1	10	10	23	1.138	1.369	0.949	1.100	53.54	43.08	14	23	253	29.8	9.06
Q9UQB8	Brain-specific angiogenesis inhibitor 1-associated protein 2 OS=Homo sapiens GN=BAIAP2 PE=1 SV=1 - [BAIP2_HUMAN]	14.31	1	6	6	17	0.909	1.179	1.225	1.100	46.91	14.31	11	17	552	60.8	8.90
Q7Z591	AT-hook-containing transcription factor OS=Homo sapiens GN=AKNA PE=1 SV=2 - [AKNA_HUMAN]	3.27	1	3	3	11	1.205	1.032	1.138	1.100	2.12	3.27	3	11	1439	155.0	6.32

Q96GD0	Pyridoxal phosphate phosphatase OS=Homo sapiens GN=PDXP PE=1 SV=2 - [PLPP_HUMAN]	12.16	1	3	3	5	1.194	1.009	0.995	1.100	7.17	12.16	4	5	296	31.7	6.55
P09848	Lactase-phlorizin hydrolase OS=Homo sapiens GN=LCT PE=1 SV=3 - [LPH_HUMAN]	1.87	1	3	3	3	2.092	1.320	0.962	1.101	2.06	1.87	3	3	1927	218.4	6.34
O95721	Synaptosomal-associated protein 29 OS=Homo sapiens GN=SNAP29 PE=1 SV=1 - [SNP29_HUMAN]	46.12	1	9	9	23	1.387	1.201	0.961	1.101	54.74	46.12	14	23	258	29.0	5.81
Q12888	Tumor suppressor p53-binding protein 1 OS=Homo sapiens GN=TP53BP1 PE=1 SV=2 - [TP53B_HUMAN]	27.69	1	41	41	170	1.041	1.227	1.076	1.101	404.77	27.69	63	170	1972	213.4	4.70
Q8NCC3	Group XV phospholipase A2 OS=Homo sapiens GN=PLA2G15 PE=1 SV=2 - [PAG15_HUMAN]	7.04	1	2	2	6	0.750	0.848	1.061	1.101	5.87	7.04	3	6	412	46.6	6.73
Q9UKF6	Cleavage and polyadenylation specificity factor subunit 3 OS=Homo sapiens GN=CPSF3 PE=1 SV=1 - [CPSF3_HUMAN]	5.12	1	4	4	14	0.867	0.918	0.973	1.101	19.92	5.12	8	14	684	77.4	5.60
P53350	Serine/threonine-protein kinase PLK1 OS=Homo sapiens GN=PLK1 PE=1 SV=1 - [PLK1_HUMAN]	3.98	1	2	2	3	1.183	0.480	0.989	1.101	4.40	3.98	2	3	603	68.2	8.91
Q8N8R3	Mitochondrial basic amino acids transporter OS=Homo sapiens GN=SLC25A29 PE=2 SV=2 - [MCATL_HUMAN	3.30	1	1	1	2	1.557	1.724	1.358	1.101	3.71	3.30	2	2	303	32.0	8.75
A6NK53	Zinc finger protein 233 OS=Homo sapiens GN=ZNF233 PE=2 SV=3 - [ZN233_HUMAN]	10.75	120	2	4	7	0.577	0.691	1.105	1.101	2.55	10.75	5	7	670	76.8	8.25

Q08043	Alpha-actinin-3 OS=Homo sapiens GN=ACTN3 PE=1 SV=2 - [ACTN3_HUMAN]	16.09	1	2	17	336	2.137	1.605	1.983	1.101	891.21	16.09	29	336	901	103.2	5.52
Q6ZV89	SH2 domain-containing protein 5 OS=Homo sapiens GN=SH2D5 PE=1 SV=2 - [SH2D5_HUMAN]	1.65	1	1	1	1	0.841	0.520	2.291	1.101	2.12	1.65	1	1	423	46.8	8.51
O00214	Galectin-8 OS=Homo sapiens GN=LGALS8 PE=1 SV=4 - [LEG8_HUMAN]	17.35	1	5	5	17	1.010	1.221	1.012	1.101	46.24	17.35	9	17	317	35.8	8.25
Q5JSZ5	Protein PRRC2B OS=Homo sapiens GN=PRRC2B PE=1 SV=2 - [PRC2B_HUMAN	7.49	1	13	16	55	1.130	0.890	1.011	1.102	105.02	7.49	21	55	2229	242.8	8.34
Q9BW66	Cyclin-dependent kinase 2-interacting protein OS=Homo sapiens GN=CINP PE=1 SV=1 - [CINP_HUMAN]	4.25	1	1	1	1	1.171	0.787	0.960	1.102	0.00	4.25	1	1	212	24.3	6.28
P08238	Heat shock protein HSP 90-beta OS=Homo sapiens GN=HSP90AB1 PE=1 SV=4 - [HS90B_HUMAN	59.25	4	29	55	1160	0.868	0.922	1.017	1.102	2946.90	59.25	85	1160	724	83.2	5.03
Q8NFI3	Cytosolic endo-beta-N-acetylglucosaminidase OS=Homo sapiens GN=ENGASE PE=1 SV=1 - [ENASE_HUMAN]	4.17	1	2	2	4	0.558	0.740	1.080	1.102	0.00	4.17	2	4	743	83.9	6.79
Q86TM6	E3 ubiquitin-protein ligase synoviolin OS=Homo sapiens GN=SYVN1 PE=1 SV=2 - [SYVN1_HUMAN]	5.02	1	2	2	5	1.114	0.874	0.700	1.102	10.98	5.02	3	5	617	67.6	6.95
Q9GZU8	Protein FAM192A OS=Homo sapiens GN=FAM192A PE=1 SV=1 - [F192A_HUMAN]	24.02	1	5	6	8	0.855	1.160	1.093	1.102	17.61	24.02	8	8	254	28.9	5.45

O75940	Survival of motor neuron-related-splicing factor 30 OS=Homo sapiens GN=SMNDC1 PE=1 SV=1 - [SPF30_HUMAN]	29.41	1	5	5	20	0.985	0.973	0.781	1.102	38.56	29.41	8	20	238	26.7	7.24
O15371	Eukaryotic translation initiation factor 3 subunit D OS=Homo sapiens GN=EIF3D PE=1 SV=1 - [EIF3D_HUMAN]	22.08	1	12	12	61	1.047	1.011	1.088	1.102	171.89	22.08	19	61	548	63.9	6.05
P05091	Aldehyde dehydrogenase, mitochondrial OS=Homo sapiens GN=ALDH2 PE=1 SV=2 - [ALDH2_HUMAN]	51.45	1	24	27	172	1.426	1.023	1.005	1.102	486.09	51.45	43	172	517	56.3	7.05
Q9Y2W6	Tudor and KH domain-containing protein OS=Homo sapiens GN=TDRKH PE=1 SV=2 - [TDRKH_HUMAN]	3.21	1	1	1	1	2.031	1.116	1.463	1.102	2.72	3.21	1	1	561	62.0	5.02
O60664	Perilipin-3 OS=Homo sapiens GN=PLIN3 PE=1 SV=3 - [PLIN3_HUMAN]	62.21	1	25	25	238	1.094	0.802	1.077	1.103	668.96	62.21	44	238	434	47.0	5.44
Q2VPK5	Cytoplasmic tRNA 2-thiolation protein 2 OS=Homo sapiens GN=CTU2 PE=1 SV=1 - [CTU2_HUMAN]	5.83	1	3	3	4	1.025	0.956	1.072	1.103	4.81	5.83	3	4	515	56.1	6.32
Q15788	Nuclear receptor coactivator 1 OS=Homo sapiens GN=NCOA1 PE=1 SV=3 - [NCOA1_HUMA	1.32	1	1	1	2	2.509	0.964	0.873	1.103	0.00	1.32	1	2	1441	156.7	6.20
P13798	Acylamino-acid-releasing enzyme OS=Homo sapiens GN=APEH PE=1 SV=4 - [ACPH_HUMAN]	27.46	1	15	15	33	1.315	1.128	1.056	1.103	95.45	27.46	22	33	732	81.2	5.48
Q96B54	Zinc finger protein 428 OS=Homo sapiens GN=ZNF428 PE=1 SV=2 - [ZN428_HUMAN]	7.45	1	1	1	1	1.206	0.816	1.175	1.103	3.79	7.45	1	1	188	20.5	4.17

P49720	Proteasome subunit beta type-3 OS=Homo sapiens GN=PSMB3 PE=1 SV=2 - [PSB3_HUMAN]	22.93	1	5	5	17	0.999	0.845	0.763	1.103	43.22	22.93	6	17	205	22.9	6.55
Q9H078	Caseinolytic peptidase B protein homolog OS=Homo sapiens GN=CLPB PE=1 SV=1 - [CLPB_HUMAN]	21.92	1	11	12	32	1.042	1.166	1.102	1.103	94.46	21.92	18	32	707	78.7	9.01
P49207	60S ribosomal protein L34 OS=Homo sapiens GN=RPL34 PE=1 SV=3 - [RL34_HUMAN]	47.01	1	9	9	82	0.934	1.115	1.128	1.103	213.84	47.01	15	82	117	13.3	11.47
Q13905	Rap guanine nucleotide exchange factor 1 OS=Homo sapiens GN=RAPGEF1 PE=1 SV=3 - [RPGF1_HUMAN	2.04	1	2	2	4	1.278	1.434	1.364	1.104	7.30	2.04	4	4	1077	120.5	5.92
Q96D71	RalBP1-associated Eps domain-containing protein 1 OS=Homo sapiens GN=REPS1 PE=1 SV=3 - [REPS1_HUMAN]	10.43	2	8	8	22	0.831	1.041	0.952	1.104	47.94	10.43	11	22	796	86.6	5.69
Q12872	Splicing factor, suppressor of white-apricot homolog OS=Homo sapiens GN=SFSWAP PE=1 SV=3 - [SFSWA_HUMAN]	2.94	1	3	3	6	1.158	1.551	1.356	1.104	10.14	2.94	4	6	951	104.8	8.05
Q16352	Alpha-internexin OS=Homo sapiens GN=INA PE=1 SV=2 - [AINX_HUMAN]	41.68	2	17	21	181	4.186	0.874	0.988	1.104	432.24	41.68	33	181	499	55.4	5.40
P11274	Breakpoint cluster region protein OS=Homo sapiens GN=BCR PE=1 SV=2 - [BCR_HUMAN]	6.69	1	6	8	25	1.090	0.847	1.110	1.104	59.56	6.69	13	25	1271	142.7	7.03

Q8IV20	Laccase domain-containing protein 1 OS=Homo sapiens GN=LACC1 PE=1 SV=1 - [LACC1_HUMAN]	7.91	1	3	3	8	0.736	0.540	1.146	1.104	18.94	7.91	6	8	430	47.7	7.06
O95183	Vesicle-associated membrane protein 5 OS=Homo sapiens GN=VAMP5 PE=1 SV=1 - [VAMP5_HUMAN]	36.21	1	3	4	13	1.578	0.882	0.903	1.104	28.35	36.21	6	13	116	12.8	7.85
P49748	Very long-chain specific acyl-CoA dehydrogenase, mitochondrial OS=Homo sapiens GN=ACADVL PE=1 SV=1 - [ACADV_HUMAN]	36.49	1	25	25	188	1.091	1.375	1.054	1.104	441.74	36.49	43	188	655	70.3	8.75
Q9BVG4	Protein PBDC1 OS=Homo sapiens GN=PBDC1 PE=1 SV=1 - [PBDC1_HUMAN]	21.46	1	5	5	14	1.572	1.122	0.961	1.104	36.05	21.46	8	14	233	26.0	4.79
Q9UHX1	Poly(U)-binding-splicing factor PUF60 OS=Homo sapiens GN=PUF60 PE=1 SV=1 - [PUF60_HUMAN]	28.26	1	15	15	64	0.979	1.167	0.968	1.104	135.58	28.26	27	64	559	59.8	5.29
Q9BW27	Nuclear pore complex protein Nup85 OS=Homo sapiens GN=NUP85 PE=1 SV=1 - [NUP85_HUMAN]	8.84	1	6	7	18	0.994	0.935	0.853	1.104	34.77	8.84	11	18	656	75.0	5.55
Q9UM54	Unconventional myosin-VI OS=Homo sapiens GN=MYO6 PE=1 SV=4 - [MYO6_HUMAN]	24.34	1	30	30	110	0.997	0.955	0.806	1.104	246.99	24.34	50	110	1294	149.6	8.53
P07919	Cytochrome b-c1 complex subunit 6, mitochondrial OS=Homo sapiens GN=UQCRH PE=1 SV=2 - [QCR6_HUMAN]	38.46	1	4	4	51	0.961	1.111	0.699	1.104	169.23	38.46	8	51	91	10.7	4.44

P35244	Replication protein A 14 kDa subunit OS=Homo sapiens GN=RPA3 PE=1 SV=1 - [RFA3_HUMAN]	14.05	1	1	1	2	1.609	1.116	1.009	1.104	7.68	14.05	2	2	121	13.6	5.08
P41226	Ubiquitin-like modifier-activating enzyme 7 OS=Homo sapiens GN=UBA7 PE=1 SV=2 - [UBA7_HUMAN]	14.23	1	11	12	38	1.463	0.898	0.825	1.104	105.12	14.23	19	38	1012	111.6	6.04
Q14353	Guanidinoacetate N-methyltransferase OS=Homo sapiens GN=GAMT PE=1 SV=1 - [GAMT_HUMAN]	12.71	1	2	2	6	1.017	1.091	1.231	1.104	11.25	12.71	3	6	236	26.3	6.14
Q9UKV8	Protein argonaute-2 OS=Homo sapiens GN=AGO2 PE=1 SV=3 - [AGO2_HUMAN]	15.72	1	8	12	27	1.123	1.121	1.028	1.105	55.36	15.72	17	27	859	97.1	9.19
Q15555	Microtubule-associated protein RP/EB family member 2 OS=Homo sapiens GN=MAPRE2 PE=1 SV=1 - [MARE2_HUMAN]	20.80	1	3	6	16	1.159	1.569	1.422	1.105	43.80	20.80	7	16	327	37.0	5.57
P08579	U2 small nuclear ribonucleoprotein B'' OS=Homo sapiens GN=SNRPB2 PE=1 SV=1 - [RU2B_HUMAN]	31.11	1	6	8	25	0.946	1.175	1.183	1.105	62.40	31.11	12	25	225	25.5	9.72
Q7Z4F1	Low-density lipoprotein receptor-related protein 10 OS=Homo sapiens GN=LRP10 PE=1 SV=2 - [LRP10_HUMAN]	4.63	1	2	2	4	0.788	0.801	0.953	1.105	15.38	4.63	3	4	713	76.1	5.52
O75153	Clustered mitochondria protein homolog OS=Homo sapiens GN=CLUH PE=1 SV=2 - [CLU_HUMAN]	7.26	1	9	9	21	0.887	0.987	1.281	1.105	49.61	7.26	13	21	1309	146.6	6.13

Q7Z6B7	SLIT-ROBO Rho GTPase-activating protein 1 OS=Homo sapiens GN=SRGAP1 PE=1 SV=1 - [SRGP1_HUMAN]	11.80	1	8	12	35	0.768	0.948	0.837	1.105	91.94	11.80	19	35	1085	124.2	6.83
Q7KZ85	Transcription elongation factor SPT6 OS=Homo sapiens GN=SUPT6H PE=1 SV=2 - [SPT6H_HUMAN]	10.25	1	14	15	39	1.135	0.991	0.915	1.105	83.24	10.25	21	39	1726	198.9	4.91
P09669	Cytochrome c oxidase subunit 6C OS=Homo sapiens GN=COX6C PE=1 SV=2 - [COX6C_HUMAN]	52.00	1	8	8	48	0.938	1.009	0.803	1.106	67.30	52.00	14	48	75	8.8	10.39
Q9Y259	Choline/ethanolamine kinase OS=Homo sapiens GN=CHKB PE=1 SV=3 - [CHKB_HUMAN]	16.96	1	5	5	17	1.204	1.155	0.849	1.106	33.53	16.96	8	17	395	45.2	5.49
Q92900	Regulator of nonsense transcripts 1 OS=Homo sapiens GN=UPF1 PE=1 SV=2 - [RENT1_HUMAN]	28.08	1	26	26	106	0.989	1.000	1.008	1.106	257.14	28.08	43	106	1129	124.3	6.61
Q5VWW1	Complement C1q-like protein 3 OS=Homo sapiens GN=C1QL3 PE=2 SV=1 - [C1QL3_HUMAN	5.49	1	1	1	1	1.414	0.810	0.774	1.106	0.00	5.49	1	1	255	26.7	6.79
Q8NDT2	Putative RNA-binding protein 15B OS=Homo sapiens GN=RBM15B PE=1 SV=3 - [RB15B_HUMAN]	1.01	1	1	1	2	1.058	2.003	1.319	1.106	4.93	1.01	2	2	890	97.1	9.85
P78536	Disintegrin and metalloproteinase domain-containing protein 17 OS=Homo sapiens GN=ADAM17 PE=1 SV=1 - [ADA17_HUMAN]	9.34	1	7	7	18	1.434	0.787	0.815	1.106	44.41	9.34	12	18	824	93.0	5.76
Q53T59	HCLS1-binding protein 3 OS=Homo sapiens GN=HS1BP3 PE=1 SV=1 - [H1BP3_HUMAN]	23.47	1	7	7	17	1.068	1.268	1.208	1.107	39.19	23.47	10	17	392	42.8	5.01

Q9NSC5	Homer protein homolog 3 OS=Homo sapiens GN=HOMER3 PE=1 SV=2 - [HOME3_HUMAN]	37.40	1	12	13	27	1.028	1.134	1.117	1.107	60.82	37.40	17	27	361	39.8	5.45
Q9Y3V2	RWD domain-containing protein 3 OS=Homo sapiens GN=RWDD3 PE=1 SV=4 - [RWDD3_HUMAN]	3.75	1	1	1	1	0.974	1.664	1.553	1.107	2.78	3.75	1	1	267	30.5	6.18
Q9Y4B6	Protein VPRBP OS=Homo sapiens GN=VPRBP PE=1 SV=3 - [VPRBP_HUMAN]	8.23	1	6	6	13	1.234	1.008	1.090	1.107	17.36	8.23	10	13	1507	168.9	5.06
Q9UKT5	F-box only protein 4 OS=Homo sapiens GN=FBXO4 PE=1 SV=2 - [FBX4_HUMAN]	6.72	1	3	3	5	0.870	0.699	0.858	1.107	7.51	6.72	4	5	387	44.1	6.16
O43281	Embryonal Fyn-associated substrate OS=Homo sapiens GN=EFS PE=2 SV=1 - [EFS_HUMAN]	1.43	1	1	1	1	0.739	0.996	1.471	1.107	1.92	1.43	1	1	561	58.8	5.11
P29728	2'-5'-oligoadenylate synthase 2 OS=Homo sapiens GN=OAS2 PE=1 SV=3 - [OAS2_HUMAN]	4.17	1	2	2	9	1.977	0.755	0.975	1.107	18.09	4.17	3	9	719	82.4	8.25
Q02078	Myocyte-specific enhancer factor 2A OS=Homo sapiens GN=MEF2A PE=1 SV=1 - [MEF2A_HUMAN]	5.52	2	2	2	2	1.286	0.869	0.346	1.107	0.00	5.52	2	2	507	54.8	7.91
A7E2V4	Zinc finger SWIM domain-containing protein 8 OS=Homo sapiens GN=ZSWIM8 PE=1 SV=1 - [ZSWM8_HUMAN]	2.50	1	4	4	6	1.120	0.704	1.135	1.107	12.83	2.50	5	6	1837	197.2	6.80
P51970	NADH dehydrogenase [ubiquinone] 1 alpha subcomplex subunit 8 OS=Homo sapiens GN=NDUFA8 PE=1 SV=3 - [NDUA8_HUMAN]	20.93	1	3	3	12	0.639	0.889	0.821	1.107	20.29	20.93	5	12	172	20.1	7.65

P0CG12	Chromosome transmission fidelity protein 8 homolog isoform 2 OS=Homo sapiens GN=CHTF8 PE=1 SV=1 - [CTF8A_HUMAN]	7.06	1	3	3	3	1.367	1.003	1.499	1.108	6.60	7.06	3	3	524	51.4	12.41
Q9H6Z4	Ran-binding protein 3 OS=Homo sapiens GN=RANBP3 PE=1 SV=1 - [RANB3_HUMAN]	12.17	1	6	6	16	0.847	1.099	0.901	1.108	31.37	12.17	10	16	567	60.2	4.78
O75794	Cell division cycle protein 123 homolog OS=Homo sapiens GN=CDC123 PE=1 SV=1 - [CD123_HUMAN]	5.36	1	2	2	4	0.890	0.859	1.467	1.108	7.52	5.36	3	4	336	39.1	4.81
Q9C0F1	Centrosomal protein of 44 kDa OS=Homo sapiens GN=CEP44 PE=1 SV=2 - [CEP44_HUMAN]	11.54	1	3	3	5	1.011	1.322	1.112	1.108	21.98	11.54	3	5	390	44.1	5.21
Q8IYS8	Putative biorientation of chromosomes in cell division protein 1-like 2 OS=Homo sapiens GN=BOD1L2 PE=5 SV=2 - [BD1L2_HUMAN	10.47	1	1	1	1	0.483	1.069	0.487	1.108	2.40	10.47	1	1	172	18.1	5.33
Q9BW83	Intraflagellar transport protein 27 homolog OS=Homo sapiens GN=IFT27 PE=1 SV=1 - [IFT27_HUMAN]	30.11	1	4	4	9	0.991	0.643	0.863	1.108	22.39	30.11	6	9	186	20.5	5.41
Q9NP72	Ras-related protein Rab-18 OS=Homo sapiens GN=RAB18 PE=1 SV=1 - [RAB18_HUMAN]	43.20	1	8	8	24	0.994	0.748	0.885	1.108	53.24	43.20	15	24	206	23.0	5.24
Q07812	Apoptosis regulator BAX OS=Homo sapiens GN=BAX PE=1 SV=1 - [BAX_HUMAN]	33.33	1	7	7	89	0.986	1.215	1.000	1.108	265.84	33.33	12	89	192	21.2	5.22

O15321	Transmembrane 9 superfamily member 1 OS=Homo sapiens GN=TM9SF1 PE=2 SV=2 - [TM9S1_HUMAN]	10.89	1	7	7	15	1.090	0.714	0.712	1.108	36.32	10.89	9	15	606	68.8	7.17
Q16630	Cleavage and polyadenylation specificity factor subunit 6 OS=Homo sapiens GN=CPSF6 PE=1 SV=2 - [CPSF6_HUMAN]	25.23	1	10	10	48	1.197	1.215	1.012	1.108	117.85	25.23	16	48	551	59.2	7.15
Q8IWC1	MAP7 domain-containing protein 3 OS=Homo sapiens GN=MAP7D3 PE=1 SV=2 - [MA7D3_HUMAN]	4.45	2	2	6	12	0.853	0.999	0.855	1.108	23.37	4.45	6	12	876	98.4	9.32
Q70CQ2	Ubiquitin carboxyl-terminal hydrolase 34 OS=Homo sapiens GN=USP34 PE=1 SV=2 - [UBP34_HUMAN]	1.18	1	4	4	5	0.977	0.885	0.940	1.108	13.27	1.18	4	5	3546	404.0	5.82
Q7Z494	Nephrocystin-3 OS=Homo sapiens GN=NPHP3 PE=1 SV=1 - [NPHP3_HUMAN]	2.18	3	2	3	5	1.672	1.081	1.454	1.108	4.55	2.18	5	5	1330	150.8	6.76
P59768	Guanine nucleotide-binding protein G(I)/G(S)/G(O) subunit gamma-2 OS=Homo sapiens GN=GNG2 PE=1 SV=2 - [GBG2_HUMAN]	54.93	1	4	5	7	1.394	2.865	1.507	1.109	15.70	54.93	5	7	71	7.8	7.99
Q8WYA0	Intraflagellar transport protein 81 homolog OS=Homo sapiens GN=IFT81 PE=1 SV=1 - [IFT81_HUMAN]	9.91	2	6	7	10	1.140	1.357	0.987	1.109	29.53	9.91	7	10	676	79.7	8.82
Q9Y6G9	Cytoplasmic dynein 1 light intermediate chain 1 OS=Homo sapiens GN=DYNC1LI1 PE=1 SV=3 - [DC1L1_HUMAN]	39.58	1	15	16	44	1.270	1.067	1.016	1.109	111.84	39.58	25	44	523	56.5	6.42

Q9Y484	WD repeat domain phosphoinositide-interacting protein 4 OS=Homo sapiens GN=WDR45 PE=2 SV=1 - [WIPI4_HUMAN]	2.50	1	1	1	1	0.690	1.420	1.601	1.110	0.00	2.50	1	1	360	39.8	7.14
Q8IXS0	Protein FAM217A OS=Homo sapiens GN=FAM217A PE=2 SV=2 - [F217A_HUMAN]	3.74	2	1	2	3	1.118	1.653	0.939	1.110	4.39	3.74	3	3	508	57.4	8.44
Q86XP3	ATP-dependent RNA helicase DDX42 OS=Homo sapiens GN=DDX42 PE=1 SV=1 - [DDX42_HUMAN	30.28	1	22	22	59	1.118	1.172	0.979	1.110	139.80	30.28	36	59	938	102.9	7.02
O15127	Secretory carrier-associated membrane protein 2 OS=Homo sapiens GN=SCAMP2 PE=1 SV=2 - [SCAM2_HUMA	14.29	1	4	4	39	0.894	0.934	0.809	1.110	113.98	14.29	7	39	329	36.6	6.10
P08236	Beta-glucuronidase OS=Homo sapiens GN=GUSB PE=1 SV=2 - [BGLR_HUMAN]	11.37	1	10	10	22	1.549	1.638	0.789	1.110	44.42	11.37	13	22	651	74.7	7.02
Q9Y5B9	FACT complex subunit SPT16 OS=Homo sapiens GN=SUPT16H PE=1 SV=1 - [SP16H_HUMAN]	19.01	1	19	20	58	1.025	1.128	0.909	1.110	138.95	19.01	30	58	1047	119.8	5.66
Q9UHI8	A disintegrin and metalloproteinase with thrombospondin motifs 1 OS=Homo sapiens GN=ADAMTS1 PE=1 SV=4 - [ATS1_HUMAN]	6.51	1	6	6	18	2.951	0.408	0.461	1.110	37.46	6.51	9	18	967	105.3	6.83
Q5JTH9	RRP12-like protein OS=Homo sapiens GN=RRP12 PE=1 SV=2 - [RRP12_HUMAN]	12.10	1	13	14	34	0.922	0.959	0.936	1.110	68.86	12.10	20	34	1297	143.6	8.75

P13804	Electron transfer flavoprotein subunit alpha, mitochondrial OS=Homo sapiens GN=ETFA PE=1 SV=1 - [ETFA_HUMAN]	45.65	1	13	13	71	0.987	1.059	0.786	1.110	141.97	45.65	20	71	333	35.1	8.38
Q9H4L5	Oxysterol-binding protein-related protein 3 OS=Homo sapiens GN=OSBPL3 PE=1 SV=1 - [OSBL3_HUMAN]	6.99	1	5	5	11	0.862	1.482	0.931	1.110	27.32	6.99	7	11	887	101.2	6.87
Q8TDN6	Ribosome biogenesis protein BRX1 homolog OS=Homo sapiens GN=BRIX1 PE=1 SV=2 - [BRX1_HUMAN]	12.75	1	5	5	35	0.919	0.907	0.851	1.111	73.27	12.75	7	35	353	41.4	9.92
P54105	Methylosome subunit pICln OS=Homo sapiens GN=CLNS1A PE=1 SV=1 - [ICLN_HUMAN]	23.63	1	3	3	21	0.880	0.873	1.092	1.111	44.33	23.63	5	21	237	26.2	4.11
O95071	E3 ubiquitin-protein ligase UBR5 OS=Homo sapiens GN=UBR5 PE=1 SV=2 - [UBR5_HUMAN]	2.97	1	6	7	10	0.598	0.984	0.943	1.111	16.41	2.97	7	10	2799	309.2	5.85
Q9Y4F1	FERM, RhoGEF and pleckstrin domain-containing protein 1 OS=Homo sapiens GN=FARP1 PE=1 SV=1 - [FARP1_HUMAN]	28.33	1	23	24	101	1.846	1.096	1.092	1.111	255.65	28.33	36	101	1045	118.6	8.15
Q9BSH4	Translational activator of cytochrome c oxidase 1 OS=Homo sapiens GN=TACO1 PE=1 SV=1 - [TACO1_HUMAN]	14.48	1	2	2	4	1.039	0.960	0.621	1.111	14.33	14.48	2	4	297	32.5	8.13
P49407	Beta-arrestin-1 OS=Homo sapiens GN=ARRB1 PE=1 SV=2 - [ARRB1_HUMAN]	17.22	4	7	7	21	1.674	1.264	1.382	1.111	49.53	17.22	11	21	418	47.0	6.20

P60510	Serine/threonine-protein phosphatase 4 catalytic subunit OS=Homo sapiens GN=PPP4C PE=1 SV=1 - [PP4C_HUMAN]	14.01	1	3	3	16	0.925	1.011	1.015	1.111	48.43	14.01	6	16	307	35.1	5.06
Q9Y2W1	Thyroid hormone receptor-associated protein 3 OS=Homo sapiens GN=THRAP3 PE=1 SV=2 - [TR150_HUMAN]	22.20	1	21	21	105	1.189	1.218	1.065	1.111	282.36	22.20	31	105	955	108.6	10.15
P10155	60 kDa SS-A/Ro ribonucleoprotein OS=Homo sapiens GN=TROVE2 PE=1 SV=2 - [RO60_HUMAN]	33.46	1	18	18	62	1.124	0.958	0.920	1.111	158.38	33.46	28	62	538	60.6	8.03
P53814	Smoothelin OS=Homo sapiens GN=SMTN PE=1 SV=7 - [SMTN_HUMAN]	31.19	1	24	24	94	0.930	1.258	2.599	1.112	217.95	31.19	40	94	917	99.0	9.07
Q14139	Ubiquitin conjugation factor E4 A OS=Homo sapiens GN=UBE4A PE=1 SV=2 - [UBE4A_HUMAN]	8.26	1	6	7	35	1.087	1.024	1.202	1.112	94.07	8.26	12	35	1066	122.5	5.24
O75586	Mediator of RNA polymerase II transcription subunit 6 OS=Homo sapiens GN=MED6 PE=1 SV=2 - [MED6_HUMAN]	6.50	1	2	2	3	0.785	1.094	0.833	1.112	6.61	6.50	3	3	246	28.4	8.62
Q9UJY5	ADP-ribosylation factor-binding protein GGA1 OS=Homo sapiens GN=GGA1 PE=1 SV=1 - [GGA1_HUMAN]	8.61	1	4	4	14	0.928	0.979	1.080	1.112	40.07	8.61	6	14	639	70.3	5.29
Q96MT8	Centrosomal protein of 63 kDa OS=Homo sapiens GN=CEP63 PE=1 SV=1 - [CEP63_HUMAN]	2.70	1	2	3	7	0.862	0.561	0.692	1.112	15.38	2.70	3	7	703	81.3	6.13

Q9NPA0	ER membrane protein complex subunit 7 OS=Homo sapiens GN=EMC7 PE=1 SV=1 - [EMC7_HUMAN]	19.42	1	4	4	12	1.136	0.924	0.877	1.112	30.94	19.42	6	12	242	26.5	9.25
P49590	Probable histidine--tRNA ligase, mitochondrial OS=Homo sapiens GN=HARS2 PE=1 SV=1 - [SYHM_HUMAN]	22.33	2	5	12	27	1.457	1.186	1.103	1.112	55.42	22.33	18	27	506	56.9	8.24
Q96L93	Kinesin-like protein KIF16B OS=Homo sapiens GN=KIF16B PE=1 SV=2 - [KI16B_HUMAN]	6.30	2	6	8	10	1.849	0.826	1.155	1.112	23.06	6.30	9	10	1317	151.9	6.16
Q9NP66	High mobility group protein 20A OS=Homo sapiens GN=HMG20A PE=1 SV=1 - [HM20A_HUMA N]	15.85	1	5	5	9	1.062	1.296	1.030	1.112	22.98	15.85	7	9	347	40.1	6.49
Q6NUK1	Calcium-binding mitochondrial carrier protein SCaMC-1 OS=Homo sapiens GN=SLC25A24 PE=1 SV=2 - [SCMC1_HUMA N]	37.11	1	22	22	73	1.494	1.344	0.921	1.112	186.65	37.11	34	73	477	53.3	6.33
Q9UI10	Translation initiation factor eIF-2B subunit delta OS=Homo sapiens GN=EIF2B4 PE=1 SV=2 - [EI2BD_HUMAN	18.93	1	8	8	31	1.119	1.000	1.019	1.112	94.14	18.93	13	31	523	57.5	9.38
Q7Z404	Transmembran e channel-like protein 4 OS=Homo sapiens GN=TMC4 PE=2 SV=3 - [TMC4_HUMAN]	3.23	1	2	2	5	0.966	1.323	1.483	1.113	1.65	3.23	2	5	712	79.2	8.94
Q8IWV7	E3 ubiquitin-protein ligase UBR1 OS=Homo sapiens GN=UBR1 PE=1 SV=1 - [UBR1_HUMAN]	6.00	1	7	7	20	0.984	1.127	1.215	1.113	49.82	6.00	11	20	1749	200.1	6.01
Q8NHV4	Protein NEDD1 OS=Homo sapiens GN=NEDD1 PE=1 SV=1 - [NEDD1_HUMAN]	10.30	1	5	5	12	0.897	1.206	0.785	1.113	33.76	10.30	7	12	660	71.9	7.97

P63218	Guanine nucleotide-binding protein G(I)/G(S)/G(O) subunit gamma-5 OS=Homo sapiens GN=GNG5 PE=1 SV=3 - [GBG5_HUMAN]	23.53	1	2	2	7	1.240	0.717	0.742	1.113	14.08	23.53	3	7	68	7.3	9.85
Q9H469	F-box/LRR-repeat protein 15 OS=Homo sapiens GN=FBXL15 PE=1 SV=2 - [FXL15_HUMAN]	4.33	1	1	1	1	1.049	0.663	1.022	1.113	0.00	4.33	1	1	300	33.0	7.44
P49137	MAP kinase-activated protein kinase 2 OS=Homo sapiens GN=MAPKAPK2 PE=1 SV=1 - [MAPK2_HUMAN]	24.25	3	10	10	19	1.628	1.364	1.014	1.113	45.04	24.25	13	19	400	45.5	8.68
Q15910	Histone-lysine N-methyltransferase EZH2 OS=Homo sapiens GN=EZH2 PE=1 SV=2 - [EZH2_HUMAN]	1.74	2	1	1	2	1.289	0.680	0.861	1.113	0.00	1.74	1	2	746	85.3	7.02
Q9Y2S6	Translation machinery-associated protein 7 OS=Homo sapiens GN=TMA7 PE=1 SV=1 - [TMA7_HUMAN]	53.13	1	4	5	17	0.736	1.353	1.099	1.113	24.82	53.13	7	17	64	7.1	9.99
Q8N3R9	MAGUK p55 subfamily member 5 OS=Homo sapiens GN=MPP5 PE=1 SV=3 - [MPP5_HUMAN]	14.37	1	9	9	18	1.141	1.052	1.243	1.113	44.58	14.37	13	18	675	77.2	6.14
Q15041	ADP-ribosylation factor-like protein 6-interacting protein 1 OS=Homo sapiens GN=ARL6IP1 PE=1 SV=2 - [AR6P1_HUMAN]	7.88	1	1	2	5	1.528	1.063	0.491	1.114	9.65	7.88	3	5	203	23.3	9.32
P22033	Methylmalonyl-CoA mutase, mitochondrial OS=Homo sapiens GN=MUT PE=1 SV=4 - [MUTA_HUMAN]	18.80	1	12	12	30	0.944	1.308	1.189	1.114	82.16	18.80	21	30	750	83.1	6.93

P23368	NAD-dependent malic enzyme, mitochondrial OS=Homo sapiens GN=ME2 PE=1 SV=1 - [MAOM_HUMAN]	19.52	1	9	10	54	0.881	1.542	1.226	1.114	129.62	19.52	17	54	584	65.4	7.61
Q08357	Sodium-dependent phosphate transporter 2 OS=Homo sapiens GN=SLC20A2 PE=1 SV=1 - [S20A2_HUMAN]	5.83	1	3	3	8	1.047	1.103	0.738	1.114	16.42	5.83	4	8	652	70.3	6.54
Q9P2W9	Syntaxin-18 OS=Homo sapiens GN=STX18 PE=1 SV=1 - [STX18_HUMAN]	6.57	1	2	2	5	1.474	1.006	1.232	1.114	13.75	6.57	3	5	335	38.6	5.49
Q12931	Heat shock protein 75 kDa, mitochondrial OS=Homo sapiens GN=TRAP1 PE=1 SV=3 - [TRAP1_HUMAN	30.11	1	19	20	175	0.802	1.147	1.043	1.114	483.14	30.11	34	175	704	80.1	8.21
Q9NR97	Toll-like receptor 8 OS=Homo sapiens GN=TLR8 PE=1 SV=1 - [TLR8_HUMAN]	0.67	1	1	1	1	1.338	1.267	0.970	1.114	1.88	0.67	1	1	1041	119.8	6.65
Q6ZTY8	Putative uncharacterized protein C12orf63 OS=Homo sapiens GN=C12orf63 PE=2 SV=2 - [CL063_HUMAN]	2.76	1	2	3	3	0.271	0.119	0.164	1.114	4.71	2.76	3	3	1197	135.7	8.03
Q96CN5	Leucine-rich repeat-containing protein 45 OS=Homo sapiens GN=LRRC45 PE=1 SV=1 - [LRC45_HUMAN]	5.22	1	3	3	4	1.123	0.773	1.096	1.114	6.24	5.22	3	4	670	75.9	6.23
Q9Y2R5	28S ribosomal protein S17, mitochondrial OS=Homo sapiens GN=MRPS17 PE=2 SV=1 - [RT17_HUMAN]	15.38	1	2	2	4	1.109	0.746	1.074	1.114	10.42	15.38	2	4	130	14.5	9.85

P45973	Chromobox protein homolog 5 OS=Homo sapiens GN=CBX5 PE=1 SV=1 - [CBX5_HUMAN]	42.41	1	8	8	29	1.004	1.086	0.743	1.114	84.92	42.41	12	29	191	22.2	5.86
Q5TFE4	5'-nucleotidase domain-containing protein 1 OS=Homo sapiens GN=NT5DC1 PE=1 SV=1 - [NT5D1_HUMAN]	29.01	1	13	13	28	1.755	1.117	1.135	1.114	64.23	29.01	17	28	455	51.8	6.35
P33908	Mannosyl-oligosaccharide 1,2-alpha-mannosidase IA OS=Homo sapiens GN=MAN1A1 PE=1 SV=3 - [MA1A1_HUMAN]	15.31	2	6	9	27	1.456	0.996	0.884	1.115	70.78	15.31	12	27	653	72.9	6.47
Q9P031	Thyroid transcription factor 1-associated protein 26 OS=Homo sapiens GN=CCDC59 PE=1 SV=2 - [TAP26_HUMAN]	3.73	1	1	1	1	0.910	0.938	2.191	1.115	3.49	3.73	1	1	241	28.7	9.88
Q13144	Translation initiation factor eIF-2B subunit epsilon OS=Homo sapiens GN=EIF2B5 PE=1 SV=3 - [EI2BE_HUMAN]	7.21	1	4	4	20	1.343	1.255	1.084	1.115	48.02	7.21	7	20	721	80.3	5.08
P55084	Trifunctional enzyme subunit beta, mitochondrial OS=Homo sapiens GN=HADHB PE=1 SV=3 - [ECHB_HUMAN]	57.38	1	27	29	201	1.003	1.197	0.956	1.115	444.98	57.38	48	201	474	51.3	9.41
Q9UMR3	T-box transcription factor TBX20 OS=Homo sapiens GN=TBX20 PE=1 SV=4 - [TBX20_HUMAN]	10.29	1	2	2	2	1.062	0.714	1.142	1.115	2.92	10.29	2	2	447	49.2	7.71
Q4KMP7	TBC1 domain family member 10B OS=Homo sapiens GN=TBC1D10B PE=1 SV=3 - [TB10B_HUMAN]	9.03	1	5	5	21	1.090	1.260	1.047	1.115	47.89	9.03	9	21	808	87.1	9.19

Q7Z4L5	Tetratricopeptide repeat protein 21B OS=Homo sapiens GN=TTC21B PE=1 SV=2 - [TT21B_HUMAN	4.79	1	3	6	7	1.098	0.963	0.853	1.115	14.99	4.79	6	7	1316	150.8	6.96
Q14651	Plastin-1 OS=Homo sapiens GN=PLS1 PE=1 SV=2 - [PLSI_HUMAN]	9.22	1	1	6	36	1.179	0.829	1.166	1.116	96.31	9.22	10	36	629	70.2	5.41
Q15397	Pumilio domain-containing protein KIAA0020 OS=Homo sapiens GN=KIAA0020 PE=1 SV=3 - [K0020_HUMAN]	14.51	2	10	10	18	0.922	0.886	0.833	1.116	47.76	14.51	15	18	648	73.5	9.64
Q8IWZ8	SURP and G-patch domain-containing protein 1 OS=Homo sapiens GN=SUGP1 PE=1 SV=2 - [SUGP1_HUMAN]	1.71	1	1	1	1	1.012	0.883	0.963	1.116	2.36	1.71	1	1	645	72.4	7.61
Q16186	Proteasomal ubiquitin receptor ADRM1 OS=Homo sapiens GN=ADRM1 PE=1 SV=2 - [ADRM1_HUMAN]	2.70	1	1	1	3	0.998	1.172	1.043	1.116	2.76	2.70	2	3	407	42.1	5.07
P46199	Translation initiation factor IF-2, mitochondrial OS=Homo sapiens GN=MTIF2 PE=1 SV=2 - [IF2M_HUMAN]	3.44	1	2	3	8	1.069	1.156	1.000	1.116	23.36	3.44	4	8	727	81.3	7.15
Q8NCM8	Cytoplasmic dynein 2 heavy chain 1 OS=Homo sapiens GN=DYNC2H1 PE=1 SV=4 - [DYHC2_HUMAN]	2.65	2	9	12	26	1.024	0.976	1.072	1.116	38.72	2.65	14	26	4307	492.3	6.54
Q9UPU5	Ubiquitin carboxyl-terminal hydrolase 24 OS=Homo sapiens GN=USP24 PE=1 SV=3 - [UBP24_HUMAN]	4.31	1	8	10	26	1.013	1.007	1.216	1.116	73.90	4.31	15	26	2620	294.2	6.14

P62072	Mitochondrial import inner membrane translocase subunit Tim10 OS=Homo sapiens GN=TIMM10 PE=1 SV=1 - [TIM10_HUMAN]	53.33	1	5	5	25	0.898	0.989	0.744	1.116	78.96	53.33	9	25	90	10.3	6.29
P43405	Tyrosine-protein kinase SYK OS=Homo sapiens GN=SYK PE=1 SV=1 - [KSYK_HUMAN]	2.36	1	1	1	1	1.551	1.012	1.030	1.117	1.67	2.36	1	1	635	72.0	8.25
O60826	Coiled-coil domain-containing protein 22 OS=Homo sapiens GN=CCDC22 PE=1 SV=1 - [CCD22_HUMAN]	24.08	1	13	13	45	1.065	0.923	1.037	1.117	112.89	24.08	21	45	627	70.7	6.74
P83111	Serine beta-lactamase-like protein LACTB, mitochondrial OS=Homo sapiens GN=LACTB PE=1 SV=2 - [LACTB_HUMAN]	18.65	1	10	10	55	0.980	0.924	0.913	1.117	135.62	18.65	18	55	547	60.7	8.53
Q96KA5	Cleft lip and palate transmembrane protein 1-like protein OS=Homo sapiens GN=CLPTM1L PE=1 SV=1 - [CLP1L_HUMAN]	13.38	1	5	5	9	0.904	0.629	0.809	1.117	28.38	13.38	7	9	538	62.2	8.56
Q9UBU6	Protein FAM8A1 OS=Homo sapiens GN=FAM8A1 PE=1 SV=1 - [FA8A1_HUMAN]	7.51	1	2	2	4	0.944	1.436	2.192	1.117	9.56	7.51	3	4	413	44.1	7.21
O43920	NADH dehydrogenase [ubiquinone] iron-sulfur protein 5 OS=Homo sapiens GN=NDUFS5 PE=1 SV=3 - [NDUS5_HUMAN]	61.32	1	6	6	26	0.708	0.714	0.717	1.117	61.84	61.32	12	26	106	12.5	9.14
Q92643	GPI-anchor transamidase OS=Homo sapiens GN=PIGK PE=1 SV=2 - [GPI8_HUMAN]	6.84	1	2	2	5	1.235	0.990	0.638	1.117	8.06	6.84	3	5	395	45.2	6.16

P09622	Dihydrolipoyl dehydrogenase, mitochondrial OS=Homo sapiens GN=DLD PE=1 SV=2 - [DLDH_HUMAN]	34.77	1	13	13	132	0.961	1.308	1.120	1.117	307.66	34.77	23	132	509	54.1	7.85
Q96EY8	Cob(I)yrinic acid a,c-diamide adenosyltransferase, mitochondrial OS=Homo sapiens GN=MMAB PE=1 SV=1 - [MMAB_HUMAN	16.80	1	3	3	11	1.036	0.895	1.075	1.117	36.60	16.80	4	11	250	27.4	8.60
Q15759	Mitogen-activated protein kinase 11 OS=Homo sapiens GN=MAPK11 PE=1 SV=2 - [MK11_HUMAN]	6.04	3	1	2	4	1.573	1.047	1.059	1.117	8.83	6.04	3	4	364	41.3	5.83
Q5TC12	ATP synthase mitochondrial F1 complex assembly factor 1 OS=Homo sapiens GN=ATPAF1 PE=1 SV=1 - [ATPF1_HUMAN]	1.83	1	1	1	2	0.874	1.176	0.962	1.118	4.42	1.83	1	2	328	36.4	7.96
Q9Y697	Cysteine desulfurase, mitochondrial OS=Homo sapiens GN=NFS1 PE=1 SV=3 - [NFS1_HUMAN]	14.66	1	7	7	19	1.040	1.158	1.186	1.118	44.11	14.66	12	19	457	50.2	8.31
P08754	Guanine nucleotide-binding protein G(k) subunit alpha OS=Homo sapiens GN=GNAI3 PE=1 SV=3 - [GNAI3_HUMAN]	41.24	4	5	14	104	0.862	0.852	0.621	1.118	270.23	41.24	25	104	354	40.5	5.69
Q96HS1	Serine/threonine-protein phosphatase PGAM5, mitochondrial OS=Homo sapiens GN=PGAM5 PE=1 SV=2 - [PGAM5_HUMAN]	25.95	2	9	9	32	1.021	1.130	0.971	1.118	70.84	25.95	14	32	289	32.0	8.68
Q14240	Eukaryotic initiation factor 4A-II OS=Homo sapiens GN=EIF4A2 PE=1 SV=2 - [IF4A2_HUMAN]	51.84	1	8	21	277	1.041	1.054	1.183	1.118	682.40	51.84	35	277	407	46.4	5.48

Q9UG63	ATP-binding cassette sub-family F member 2 OS=Homo sapiens GN=ABCF2 PE=1 SV=2 - [ABCF2_HUMAN]	27.77	1	15	15	52	0.974	0.883	0.978	1.118	124.86	27.77	26	52	623	71.2	7.37
P54315	Inactive pancreatic lipase-related protein 1 OS=Homo sapiens GN=PNLIPRP1 PE=1 SV=1 - [LIPR1_HUMAN]	3.64	1	1	1	1	2.960	1.821	0.953	1.118	2.83	3.64	1	1	467	51.8	5.73
P28799	Granulins OS=Homo sapiens GN=GRN PE=1 SV=2 - [GRN_HUMAN]	10.62	1	6	6	15	1.024	0.917	1.292	1.118	41.70	10.62	8	15	593	63.5	6.83
Q6P2E9	Enhancer of mRNA-decapping protein 4 OS=Homo sapiens GN=EDC4 PE=1 SV=1 - [EDC4_HUMAN]	13.99	3	16	17	49	0.944	0.967	1.029	1.118	118.23	13.99	30	49	1401	151.6	5.86
Q9HD67	Unconventional myosin-X OS=Homo sapiens GN=MYO10 PE=1 SV=3 - [MYO10_HUMAN]	3.64	1	4	4	13	0.712	0.649	0.504	1.119	16.07	3.64	5	13	2058	237.2	6.21
P20933	N(4)-(beta-N-acetylglucosaminyl)-L-asparaginase OS=Homo sapiens GN=AGA PE=1 SV=2 - [ASPG_HUMAN]	13.01	1	4	4	13	0.529	1.219	1.102	1.119	37.08	13.01	6	13	346	37.2	6.28
Q9H2W6	39S ribosomal protein L46, mitochondrial OS=Homo sapiens GN=MRPL46 PE=1 SV=1 - [RM46_HUMAN]	12.90	1	3	3	12	1.068	1.519	1.175	1.119	38.66	12.90	6	12	279	31.7	7.05
Q9UHR5	SAP30-binding protein OS=Homo sapiens GN=SAP30BP PE=1 SV=1 - [S30BP_HUMAN]	26.62	1	6	6	10	0.922	0.995	0.882	1.119	34.87	26.62	9	10	308	33.8	4.84
P11802	Cyclin-dependent kinase 4 OS=Homo sapiens GN=CDK4 PE=1 SV=2 - [CDK4_HUMAN]	17.16	6	3	4	17	1.214	1.213	1.135	1.119	33.16	17.16	5	17	303	33.7	7.01

Q6IA86	Elongator complex protein 2 OS=Homo sapiens GN=ELP2 PE=1 SV=2 - [ELP2_HUMAN]	0.97	1	1	1	5	0.491	1.033	0.853	1.119	5.76	0.97	2	5	826	92.4	5.96
Q13114	TNF receptor-associated factor 3 OS=Homo sapiens GN=TRAF3 PE=1 SV=2 - [TRAF3_HUMAN	3.52	1	2	2	4	0.912	1.128	1.142	1.119	7.87	3.52	2	4	568	64.4	7.91
P37802	Transgelin-2 OS=Homo sapiens GN=TAGLN2 PE=1 SV=3 - [TAGL2_HUMAN]	85.43	1	20	21	670	**1.584**	0.912	1.021	1.119	1600.81	85.43	36	670	199	22.4	8.25
Q9UGR2	Zinc finger CCCH domain-containing protein 7B OS=Homo sapiens GN=ZC3H7B PE=1 SV=1 - [Z3H7B_HUMAN]	11.18	1	8	9	20	1.153	0.743	0.915	1.119	43.79	11.18	13	20	993	111.5	7.17
O43760	Synaptogyrin-2 OS=Homo sapiens GN=SYNGR2 PE=1 SV=1 - [SNG2_HUMAN]	3.57	1	1	1	1	1.138	0.932	0.850	1.119	2.09	3.57	1	1	224	24.8	4.94
Q86WB0	Nuclear-interacting partner of ALK OS=Homo sapiens GN=ZC3HC1 PE=1 SV=1 - [NIPA_HUMAN]	11.35	1	4	4	8	1.277	0.936	0.686	1.120	8.21	11.35	5	8	502	55.2	5.62
Q2TAY7	WD40 repeat-containing protein SMU1 OS=Homo sapiens GN=SMU1 PE=1 SV=2 - [SMU1_HUMAN]	14.23	1	6	6	24	1.056	1.107	0.792	1.120	42.96	14.23	11	24	513	57.5	7.18
Q92917	G patch domain and KOW motifs-containing protein OS=Homo sapiens GN=GPKOW PE=1 SV=2 - [GPKOW_HUMAN]	13.87	1	6	6	13	1.298	1.255	1.099	1.120	25.67	13.87	8	13	476	52.2	6.15
Q6UN15	Pre-mRNA 3'-end-processing factor FIP1 OS=Homo sapiens GN=FIP1L1 PE=1 SV=1 - [FIP1_HUMAN]	10.27	1	4	4	14	1.048	1.073	1.017	1.120	24.06	10.27	6	14	594	66.5	5.59

Q14767	Latent-transforming growth factor beta-binding protein 2 OS=Homo sapiens GN=LTBP2 PE=1 SV=3 - [LTBP2_HUMAN]	3.19	1	4	4	20	0.452	1.185	1.423	1.120	50.11	3.19	8	20	1821	194.9	5.19
P36021	Monocarboxylate transporter 8 OS=Homo sapiens GN=SLC16A2 PE=1 SV=2 - [MOT8_HUMAN]	2.41	1	1	1	15	2.007	1.025	1.268	1.120	46.19	2.41	2	15	539	59.5	5.59
P57772	Selenocysteine-specific elongation factor OS=Homo sapiens GN=EEFSEC PE=1 SV=4 - [SELB_HUMAN]	5.54	1	2	2	8	1.705	1.355	0.956	1.120	22.84	5.54	4	8	596	65.3	8.35
P11940	Polyadenylate-binding protein 1 OS=Homo sapiens GN=PABPC1 PE=1 SV=2 - [PABP1_HUMAN]	39.62	4	18	27	187	1.058	0.998	0.991	1.120	486.69	39.62	48	187	636	70.6	9.50
O75251	NADH dehydrogenase [ubiquinone] iron-sulfur protein 7, mitochondrial OS=Homo sapiens GN=NDUFS7 PE=1 SV=3 - [NDUS7_HUMAN]	15.02	1	4	4	11	0.639	0.925	0.710	1.120	23.77	15.02	7	11	213	23.5	9.99
Q9H0S4	Probable ATP-dependent RNA helicase DDX47 OS=Homo sapiens GN=DDX47 PE=1 SV=1 - [DDX47_HUMAN	2.64	1	1	1	3	0.943	0.649	0.704	1.120	6.96	2.64	2	3	455	50.6	9.10
Q99623	Prohibitin-2 OS=Homo sapiens GN=PHB2 PE=1 SV=2 - [PHB2_HUMAN]	66.89	1	20	20	125	1.077	1.089	0.843	1.120	337.62	66.89	32	125	299	33.3	9.83
Q86WR0	Coiled-coil domain-containing protein 25 OS=Homo sapiens GN=CCDC25 PE=1 SV=2 - [CCD25_HUMAN]	12.98	1	4	4	4	1.102	1.115	0.962	1.120	4.23	12.98	4	4	208	24.5	6.80

P28062	Proteasome subunit beta type-8 OS=Homo sapiens GN=PSMB8 PE=1 SV=3 - [PSB8_HUMAN]	26.45	1	7	7	29	1.261	1.182	0.922	1.120	81.65	26.45	13	29	276	30.3	7.43
Q9P0M6	Core histone macro-H2A.2 OS=Homo sapiens GN=H2AFY2 PE=1 SV=3 - [H2AW_HUMAN]	26.08	1	10	11	47	1.157	1.125	1.411	1.120	104.16	26.08	21	47	372	40.0	9.69
Q6P6C2	RNA demethylase ALKBH5 OS=Homo sapiens GN=ALKBH5 PE=1 SV=2 - [ALKB5_HUMAN]	5.58	1	2	2	3	0.682	1.740	1.132	1.121	4.97	5.58	3	3	394	44.2	9.09
Q53F39	Metallophosphoesterase 1 OS=Homo sapiens GN=MPPE1 PE=1 SV=2 - [MPPE1_HUMAN]	1.77	1	1	1	2	0.947	0.812	0.926	1.121	2.31	1.77	1	2	396	45.1	7.15
Q9H000	Probable E3 ubiquitin-protein ligase makorin-2 OS=Homo sapiens GN=MKRN2 PE=1 SV=2 - [MKRN2_HUMAN]	19.71	1	8	8	15	0.992	1.015	1.182	1.121	24.94	19.71	11	15	416	46.9	7.61
P05771	Protein kinase C beta type OS=Homo sapiens GN=PRKCB PE=1 SV=4 - [KPCB_HUMAN]	8.64	1	3	6	11	1.017	1.255	0.937	1.121	14.92	8.64	7	11	671	76.8	7.01
P55157	Microsomal triglyceride transfer protein large subunit OS=Homo sapiens GN=MTTP PE=1 SV=1 - [MTP_HUMAN]	0.89	1	1	1	1	1.059	1.020	0.756	1.121	2.38	0.89	1	1	894	99.3	8.41
Q9NRF9	DNA polymerase epsilon subunit 3 OS=Homo sapiens GN=POLE3 PE=1 SV=1 - [DPOE3_HUMAN]	7.48	1	1	1	2	0.947	0.679	0.725	1.121	2.81	7.48	2	2	147	16.8	4.74

Q9UEU0	Veside transport through interaction with t-SNAREs homolog 1B OS=Homo sapiens GN=VTI1B PE=1 SV=3 - [VTI1B_HUMAN	28.02	1	5	5	20	1.034	0.847	1.002	1.121	44.54	28.02	10	20	232	26.7	9.04
O75438	NADH dehydrogenase [ubiquinone] 1 beta subcomplex subunit 1 OS=Homo sapiens GN=NDUFB1 PE=1 SV=1 - [NDUB1_HUMAN]	20.69	1	2	2	11	0.863	1.243	0.840	1.121	26.04	20.69	4	11	58	7.0	8.92
O14979	Heterogeneous nudear ribonudeoprotein D-like OS=Homo sapiens GN=HNRNPDL PE=1 SV=3 - [HNRDL_HUMAN]	22.38	1	10	11	57	1.160	1.076	0.919	1.121	149.87	22.38	16	57	420	46.4	9.57
Q8ND94	LRRN4 C-terminal-like protein OS=Homo sapiens GN=LRRN4CL PE=2 SV=1 - [LRN4L_HUMAN]	6.30	1	2	2	4	1.490	1.172	0.996	1.121	9.34	6.30	3	4	238	25.2	6.51
Q6DT37	Serine/threonine-protein kinase MRCK gamma OS=Homo sapiens GN=CDC42BPG PE=1 SV=2 - [MRCKG_HUMA	5.03	1	3	7	40	0.882	1.314	1.242	1.121	89.73	5.03	7	40	1551	172.4	6.28
Q70UQ0	Inhibitor of nudear factor kappa-B kinase-interacting protein OS=Homo sapiens GN=IKBIP PE=1 SV=1 - [IKIP_HUMAN]	48.29	1	18	19	128	0.825	0.806	0.714	1.121	259.04	48.29	31	128	350	39.3	9.17
Q9NQ48	Leucine zipper transcription factor-like protein 1 OS=Homo sapiens GN=LZTFL1 PE=1 SV=1 - [LZTL1_HUMAN]	39.80	1	12	12	35	0.941	0.997	1.089	1.121	78.22	39.80	19	35	299	34.6	5.36

O43765	Small glutamine-rich tetratricopeptide repeat-containing protein alpha OS=Homo sapiens GN=SGTA PE=1 SV=1 - [SGTA_HUMAN]	13.42	1	4	4	20	0.992	1.182	0.951	1.121	48.88	13.42	8	20	313	34.0	4.87
O60333	Kinesin-like protein KIF1B OS=Homo sapiens GN=KIF1B PE=1 SV=5 - [KIF1B_HUMAN]	7.16	1	4	12	40	0.805	0.883	1.249	1.122	93.36	7.16	17	40	1816	204.3	5.60
Q8TDF6	RAS guanyl-releasing protein 4 OS=Homo sapiens GN=RASGRP4 PE=1 SV=2 - [GRP4_HUMAN]	1.04	1	1	1	4	1.233	0.790	0.914	1.122	9.71	1.04	1	4	673	74.8	8.02
Q9NY27	Serine/threonine-protein phosphatase 4 regulatory subunit 2 OS=Homo sapiens GN=PPP4R2 PE=1 SV=3 - [PP4R2_HUMAN]	20.14	1	6	7	14	0.819	0.802	0.759	1.122	34.86	20.14	8	14	417	46.9	4.54
Q9BXR0	Queuine tRNA-ribosyltransferase OS=Homo sapiens GN=QTRT1 PE=1 SV=3 - [TGT_HUMAN]	10.92	1	4	4	8	0.856	1.140	0.990	1.122	17.11	10.92	6	8	403	44.0	7.23
Q12788	Transducin beta-like protein 3 OS=Homo sapiens GN=TBL3 PE=1 SV=2 - [TBL3_HUMAN]	13.12	1	7	7	11	0.863	0.867	0.894	1.122	35.17	13.12	8	11	808	89.0	6.90
Q13445	Transmembrane emp24 domain-containing protein 1 OS=Homo sapiens GN=TMED1 PE=1 SV=1 - [TMED1_HUMA	9.25	1	2	2	6	0.703	0.447	0.620	1.122	16.14	9.25	3	6	227	25.2	4.48
Q9NPJ6	Mediator of RNA polymerase II transcription subunit 4 OS=Homo sapiens GN=MED4 PE=1 SV=1 - [MED4_HUMAN]	8.15	1	2	2	4	0.948	1.191	1.035	1.123	9.74	8.15	3	4	270	29.7	5.10

Q9BTU6	Phosphatidylinositol 4-kinase type 2-alpha OS=Homo sapiens GN=PI4K2A PE=1 SV=1 - [P4K2A_HUMAN]	20.04	1	9	10	34	0.912	0.900	0.931	1.123	68.42	20.04	15	34	479	54.0	8.29
P62341	Selenoprotein T OS=Homo sapiens GN=SELT PE=2 SV=2 - [SELT_HUMAN]	17.95	1	3	3	13	1.092	0.809	0.816	1.123	36.43	17.95	6	13	195	22.3	8.60
Q969H8	UPF0556 protein C19orf10 OS=Homo sapiens GN=C19orf10 PE=1 SV=1 - [CS010_HUMAN]	24.86	1	6	6	31	0.770	0.787	0.681	1.123	72.44	24.86	10	31	173	18.8	6.68
Q9H477	Ribokinase OS=Homo sapiens GN=RBKS PE=1 SV=1 - [RBSK_HUMAN]	5.59	1	2	2	5	0.849	0.840	0.821	1.123	9.81	5.59	4	5	322	34.1	5.05
Q15287	RNA-binding protein with serine-rich domain 1 OS=Homo sapiens GN=RNPS1 PE=1 SV=1 - [RNPS1_HUMAN]	6.56	1	2	2	6	1.055	1.111	1.046	1.123	14.01	6.56	3	6	305	34.2	11.84
P16389	Potassium voltage-gated channel subfamily A member 2 OS=Homo sapiens GN=KCNA2 PE=1 SV=2 - [KCNA2_HUMAN]	2.61	1	1	1	1	1.499	0.962	1.029	1.123	3.33	2.61	1	1	499	56.7	4.86
Q9UBV7	Beta-1,4-galactosyltransferase 7 OS=Homo sapiens GN=B4GALT7 PE=1 SV=1 - [B4GT7_HUMAN]	3.36	1	1	1	4	1.198	0.973	1.354	1.123	5.39	3.36	2	4	327	37.4	8.98
Q9H8M5	Metal transporter CNNM2 OS=Homo sapiens GN=CNNM2 PE=1 SV=2 - [CNNM2_HUMA	7.77	1	6	6	9	1.196	0.720	0.784	1.123	18.93	7.77	8	9	875	96.6	6.38
P26447	Protein S100-A4 OS=Homo sapiens GN=S100A4 PE=1 SV=1 - [S10A4_HUMAN]	53.47	1	8	8	115	2.986	0.417	0.603	1.123	223.86	53.47	13	115	101	11.7	6.11

P62312	U6 snRNA-associated Sm-like protein LSm6 OS=Homo sapiens GN=LSM6 PE=1 SV=1 - [LSM6_HUMAN]	33.75	1	3	3	8	1.240	1.247	0.847	1.123	17.04	33.75	5	8	80	9.1	9.58
Q9HBL7	Plasminogen receptor (KT) OS=Homo sapiens GN=PLGRKT PE=1 SV=1 - [PLRKT_HUMAN]	15.65	1	2	2	12	1.291	1.014	0.717	1.123	36.16	15.65	4	12	147	17.2	9.58
P59826	BPI fold-containing family B member 3 OS=Homo sapiens GN=BPIFB3 PE=2 SV=2 - [BPIB3_HUMAN]	2.31	1	1	1	2	0.855	0.727	0.652	1.123	1.77	2.31	2	2	476	50.3	6.74
P82673	28S ribosomal protein S35, mitochondrial OS=Homo sapiens GN=MRPS35 PE=1 SV=1 - [RT35_HUMAN]	18.58	1	7	7	12	0.826	1.497	0.996	1.124	20.77	18.58	9	12	323	36.8	8.24
Q8WZ64	Arf-GAP with Rho-GAP domain, ANK repeat and PH domain-containing protein 2 OS=Homo sapiens GN=ARAP2 PE=1 SV=3 - [ARAP2_HUMAN]	0.70	1	1	1	1	1.193	0.726	0.586	1.124	3.10	0.70	1	1	1704	193.3	7.39
Q17RR3	Pancreatic lipase-related protein 3 OS=Homo sapiens GN=PNLIPRP3 PE=2 SV=2 - [LIPR3_HUMAN]	2.57	1	1	1	1	0.719	0.738	1.373	1.124	0.00	2.57	1	1	467	52.2	8.27
Q6UX04	Peptidyl-prolyl cis-trans isomerase CWC27 homolog OS=Homo sapiens GN=CWC27 PE=1 SV=1 - [CWC27_HUMAN]	6.78	1	2	3	7	0.950	1.166	1.197	1.124	20.55	6.78	4	7	472	53.8	5.80
P12111	Collagen alpha-3(VI) chain OS=Homo sapiens GN=COL6A3 PE=1 SV=5 - [CO6A3_HUMAN]	35.76	1	98	98	540	0.808	2.457	1.208	1.124	1390.67	35.76	164	540	3177	343.5	6.68

Q09028	Histone-binding protein RBBP4 OS=Homo sapiens GN=RBBP4 PE=1 SV=3 - [RBBP4_HUMAN]	21.88	1	3	9	35	1.300	1.101	1.034	1.124	105.81	21.88	13	35	425	47.6	4.89
Q13136	Liprin-alpha-1 OS=Homo sapiens GN=PPFIA1 PE=1 SV=1 - [LIPA1_HUMAN]	13.23	1	12	16	42	1.115	1.030	0.857	1.124	102.27	13.23	25	42	1202	135.7	6.29
Q9BXP2	Solute carrier family 12 member 9 OS=Homo sapiens GN=SLC12A9 PE=1 SV=1 - [S12A9_HUMAN]	1.64	1	1	1	4	1.377	0.837	0.768	1.124	14.56	1.64	2	4	914	96.0	8.07
Q8IVL0	Neuron navigator 3 OS=Homo sapiens GN=NAV3 PE=1 SV=3 - [NAV3_HUMAN]	2.14	1	2	3	4	0.933	1.655	0.518	1.124	12.37	2.14	3	4	2385	255.5	8.76
P51531	Probable global transcription activator SNF2L2 OS=Homo sapiens GN=SMARCA2 PE=1 SV=2 - [SMCA2_HUMA	8.11	1	5	13	37	0.948	1.165	0.978	1.124	95.22	8.11	17	37	1590	181.2	7.20
Q5C9Z4	Nucleolar MIF4G domain-containing protein 1 OS=Homo sapiens GN=NOM1 PE=1 SV=1 - [NOM1_HUMAN]	1.51	1	1	1	2	0.560	0.751	0.890	1.124	5.41	1.51	2	2	860	96.2	8.10
Q5NDL2	EGF domain-specific O-linked N-acetylglucosamine transferase OS=Homo sapiens GN=EOGT PE=1 SV=1 - [EOGT_HUMAN]	13.28	1	5	5	17	1.053	1.031	1.220	1.124	32.67	13.28	7	17	527	62.0	7.05
Q9Y266	Nuclear migration protein nudC OS=Homo sapiens GN=NUDC PE=1 SV=1 - [NUDC_HUMAN]	50.76	1	21	22	124	1.050	1.075	1.098	1.125	306.03	50.76	33	124	331	38.2	5.38

Q9UHU1	Putative uncharacterized protein PRO1716 OS=Homo sapiens GN=PRO1716 PE=5 SV=1 - [YK039_HUMAN]	30.23	1	1	1	13	1.583	0.904	1.014	1.125	16.36	30.23	1	13	43	4.9	8.62
Q8N3V7	Synaptopodin OS=Homo sapiens GN=SYNPO PE=1 SV=2 - [SYNPO_HUMAN]	15.72	1	11	11	38	2.106	0.518	0.793	1.125	106.78	15.72	18	38	929	99.4	8.72
Q0JRZ9	FCH domain only protein 2 OS=Homo sapiens GN=FCHO2 PE=1 SV=2 - [FCHO2_HUMAN]	10.62	1	7	8	15	0.788	1.152	1.239	1.125	31.27	10.62	10	15	810	88.9	6.86
Q96QD8	Sodium-coupled neutral amino acid transporter 2 OS=Homo sapiens GN=SLC38A2 PE=1 SV=2 - [S38A2_HUMAN	4.35	1	2	2	5	1.957	0.781	1.668	1.125	26.32	4.35	2	5	506	56.0	8.00
Q2NL98	Vimentin-type intermediate filament-associated coiled-coil protein OS=Homo sapiens GN=VMAC PE=2 SV=1 - [VMAC_HUMAN]	5.33	1	1	1	3	1.452	1.413	1.112	1.125	9.69	5.33	2	3	169	18.3	5.73
Q9NRX2	39S ribosomal protein L17, mitochondrial OS=Homo sapiens GN=MRPL17 PE=1 SV=1 - [RM17_HUMAN]	8.57	1	2	2	2	0.872	0.926	1.164	1.125	3.69	8.57	2	2	175	20.0	10.11
Q96EY1	DnaJ homolog subfamily A member 3, mitochondrial OS=Homo sapiens GN=DNAJA3 PE=1 SV=2 - [DNJA3_HUMAN]	15.83	1	6	6	15	1.013	1.101	0.883	1.126	37.85	15.83	9	15	480	52.5	9.26
O75582	Ribosomal protein S6 kinase alpha-5 OS=Homo sapiens GN=RPS6KA5 PE=1 SV=1 - [KS6A5_HUMAN]	1.62	1	1	2	2	0.770	0.713	0.890	1.126	4.73	1.62	2	2	802	89.8	7.11

O94874	E3 UFM1-protein ligase 1 OS=Homo sapiens GN=UFL1 PE=1 SV=2 - [UFL1_HUMAN]	44.46	2	30	30	92	0.932	0.845	0.819	1.126	221.87	44.46	46	92	794	89.5	6.79
Q3L8U1	Chromodomain-helicase-DNA-binding protein 9 OS=Homo sapiens GN=CHD9 PE=1 SV=2 - [CHD9_HUMAN]	0.41	1	1	1	1	1.046	0.996	1.303	1.126	2.27	0.41	1	1	2897	325.8	7.01
Q7L7V1	Putative pre-mRNA-splicing factor ATP-dependent RNA helicase DHX32 OS=Homo sapiens GN=DHX32 PE=1 SV=1 - [DHX32_HUMAN	0.94	1	1	1	2	0.723	2.315	1.778	1.126	5.52	0.94	1	2	743	84.4	4.97
Q9Y3D0	Mitotic spindle-associated MMXD complex subunit MIP18 OS=Homo sapiens GN=FAM96B PE=1 SV=1 - [MIP18_HUMAN]	30.67	1	3	3	13	0.862	1.494	1.050	1.126	52.75	30.67	3	13	163	17.7	5.19
Q86Y01	E3 ubiquitin-protein ligase DTX1 OS=Homo sapiens GN=DTX1 PE=1 SV=1 - [DTX1_HUMAN]	2.26	1	1	1	2	1.215	0.861	1.125	1.126	2.75	2.26	1	2	620	67.3	9.60
Q92599	Septin-8 OS=Homo sapiens GN=SEPT8 PE=1 SV=4 - [SEPT8_HUMAN]	26.29	1	7	11	43	1.700	0.865	1.147	1.126	101.01	26.29	16	43	483	55.7	6.28
Q9BZZ5	Apoptosis inhibitor 5 OS=Homo sapiens GN=API5 PE=1 SV=3 - [API5_HUMAN]	29.58	1	14	14	54	1.127	1.307	1.001	1.126	141.15	29.58	21	54	524	59.0	7.34
Q14699	Raftlin OS=Homo sapiens GN=RFTN1 PE=1 SV=4 - [RFTN1_HUMAN]	30.28	1	14	14	47	1.494	0.825	1.244	1.126	131.80	30.28	22	47	578	63.1	5.67

Q9UJJ9	N-acetylglucosamine-1-phosphotransferase subunit gamma OS=Homo sapiens GN=GNPTG PE=1 SV=1 - [GNPTG_HUMAN]	5.57	1	2	2	2	1.172	0.993	1.048	1.127	4.38	5.57	2	2	305	34.0	6.95
O60292	Signal-induced proliferation-associated 1-like protein 3 OS=Homo sapiens GN=SIPA1L3 PE=1 SV=3 - [SI1L3_HUMAN]	3.82	1	5	5	9	0.835	0.381	0.510	1.127	20.50	3.82	5	9	1781	194.5	8.32
Q13201	Multimerin-1 OS=Homo sapiens GN=MMRN1 PE=1 SV=3 - [MMRN1_HUMAN]	0.65	1	1	1	1	1.133	0.909	0.776	1.127	2.08	0.65	1	1	1228	138.0	7.93
Q63ZY3	KN motif and ankyrin repeat domain-containing protein 2 OS=Homo sapiens GN=KANK2 PE=1 SV=1 - [KANK2_HUMAN]	40.07	2	28	30	129	1.008	1.923	1.376	1.127	339.70	40.07	47	129	851	91.1	5.63
P51793	H(+)/Cl(-) exchange transporter 4 OS=Homo sapiens GN=CLCN4 PE=1 SV=2 - [CLCN4_HUMAN]	1.45	1	2	2	4	1.027	0.960	0.819	1.127	2.66	1.45	2	4	760	84.9	6.86
Q13148	TAR DNA-binding protein 43 OS=Homo sapiens GN=TARDBP PE=1 SV=1 - [TADBP_HUMAN]	21.26	1	7	7	20	1.105	1.406	1.075	1.127	49.79	21.26	9	20	414	44.7	6.19
P53420	Collagen alpha-4(IV) chain OS=Homo sapiens GN=COL4A4 PE=1 SV=3 - [CO4A4_HUMAN]	2.84	1	2	2	2	1.380	0.636	1.211	1.127	5.75	2.84	2	2	1690	163.9	8.62
P08253	72 kDa type IV collagenase OS=Homo sapiens GN=MMP2 PE=1 SV=2 - [MMP2_HUMAN]	11.97	2	7	7	11	0.515	0.587	0.436	1.127	26.52	11.97	10	11	660	73.8	5.47

Q32P44	Echinoderm microtubule-associated protein-like 3 OS=Homo sapiens GN=EML3 PE=1 SV=1 - [EMAL3_HUMAN]	16.41	1	14	14	42	1.029	1.168	1.045	1.127	98.93	16.41	24	42	896	95.1	7.12
Q9NZ01	Very-long-chain enoyl-CoA reductase OS=Homo sapiens GN=TECR PE=1 SV=1 - [TECR_HUMAN]	14.61	1	4	5	25	1.294	0.886	0.818	1.127	58.86	14.61	10	25	308	36.0	9.45
P82675	28S ribosomal protein S5, mitochondrial OS=Homo sapiens GN=MRPS5 PE=1 SV=2 - [RT05_HUMAN]	20.47	1	8	9	20	0.921	1.366	0.970	1.127	55.24	20.47	12	20	430	48.0	9.92
O75380	NADH dehydrogenase [ubiquinone] iron-sulfur protein 6, mitochondrial OS=Homo sapiens GN=NDUFS6 PE=1 SV=1 - [NDUS6_HUMAN]	48.39	1	4	4	8	0.817	0.952	0.820	1.128	24.59	48.39	5	8	124	13.7	8.28
Q9HC16	DNA dC->dU-editing enzyme APOBEC-3G OS=Homo sapiens GN=APOBEC3G PE=1 SV=1 - [ABC3G_HUMAN]	7.03	1	3	3	8	1.334	0.904	0.419	1.128	19.48	7.03	3	8	384	46.4	8.00
P23258	Tubulin gamma-1 chain OS=Homo sapiens GN=TUBG1 PE=1 SV=2 - [TBG1_HUMAN]	22.17	2	11	11	24	1.130	1.171	0.923	1.128	55.50	22.17	17	24	451	51.1	6.14
O43709	Probable 18S rRNA (guanine-N(7))-methyltransferase OS=Homo sapiens GN=WBSCR22 PE=1 SV=2 - [WBS22_HUMAN]	10.32	1	2	2	2	1.000	0.561	0.662	1.128	8.53	10.32	2	2	281	31.9	8.73
Q06546	GA-binding protein alpha chain OS=Homo sapiens GN=GABPA PE=1 SV=1 - [GABPA_HUMAN]	10.35	1	5	5	15	1.100	1.252	0.915	1.128	32.34	10.35	7	15	454	51.3	4.97

Q9NWH9	SAFB-like transcription modulator OS=Homo sapiens GN=SLTM PE=1 SV=2 - [SLTM_HUMAN]	12.57	1	12	12	22	1.229	1.472	1.100	1.128	46.52	12.57	17	22	1034	117.1	7.87
Q9BT22	Chitobiosyldiphosphodolichol beta-mannosyltransferase OS=Homo sapiens GN=ALG1 PE=1 SV=2 - [ALG1_HUMAN]	4.74	1	2	2	11	1.382	1.059	0.788	1.129	37.12	4.74	3	11	464	52.5	7.23
Q8TDP1	Ribonuclease H2 subunit C OS=Homo sapiens GN=RNASEH2C PE=1 SV=1 - [RNH2C_HUMAN]	6.71	1	1	1	2	0.928	1.385	1.072	1.129	2.59	6.71	2	2	164	17.8	5.03
Q5T653	39S ribosomal protein L2, mitochondrial OS=Homo sapiens GN=MRPL2 PE=1 SV=2 - [RM02_HUMAN]	8.52	1	3	3	8	1.082	1.522	0.967	1.129	18.11	8.52	5	8	305	33.3	11.30
Q9HD20	Manganese-transporting ATPase 13A1 OS=Homo sapiens GN=ATP13A1 PE=1 SV=2 - [AT131_HUMAN]	20.27	1	23	23	86	0.920	0.765	0.763	1.129	203.41	20.27	39	86	1204	132.9	8.13
Q96S66	Chloride channel CLIC-like protein 1 OS=Homo sapiens GN=CLCC1 PE=1 SV=1 - [CLCC1_HUMAN]	25.77	1	14	14	93	0.986	0.747	0.881	1.129	222.72	25.77	21	93	551	62.0	5.55
Q9BWF3	RNA-binding protein 4 OS=Homo sapiens GN=RBM4 PE=1 SV=1 - [RBM4_HUMAN]	33.52	2	10	10	33	1.187	1.052	0.958	1.129	99.12	33.52	19	33	364	40.3	7.08
P30038	Delta-1-pyrroline-5-carboxylate dehydrogenase, mitochondrial OS=Homo sapiens GN=ALDH4A1 PE=1 SV=3 - [AL4A1_HUMAN]	26.29	1	14	14	45	1.005	1.331	1.277	1.129	92.07	26.29	24	45	563	61.7	8.07

Q92878	DNA repair protein RAD50 OS=Homo sapiens GN=RAD50 PE=1 SV=1 - [RAD50_HUMAN]	26.60	1	34	35	95	0.967	1.213	0.917	1.129	221.27	26.60	49	95	1312	153.8	6.89
Q5T3F8	CSC1-like protein 2 OS=Homo sapiens GN=TMEM63B PE=1 SV=1 - [CSCL2_HUMAN]	7.45	1	7	7	11	1.532	0.501	0.661	1.129	21.17	7.45	10	11	832	94.9	7.40
O00754	Lysosomal alpha-mannosidase OS=Homo sapiens GN=MAN2B1 PE=1 SV=3 - [MA2B1_HUMAN]	22.55	1	20	20	67	1.130	1.241	1.336	1.129	153.24	22.55	29	67	1011	113.7	7.28
P19438	Tumor necrosis factor receptor superfamily member 1A OS=Homo sapiens GN=TNFRSF1A PE=1 SV=1 - [TNR1A_HUMAN]	2.20	1	1	1	2	0.771	0.703	0.661	1.129	7.69	2.20	1	2	455	50.5	6.64
Q8NC56	LEM domain-containing protein 2 OS=Homo sapiens GN=LEMD2 PE=1 SV=1 - [LEMD2_HUMAN]	25.05	1	12	12	43	1.272	1.085	1.040	1.129	109.13	25.05	19	43	503	56.9	9.00
Q9BQG0	Myb-binding protein 1A OS=Homo sapiens GN=MYBBP1A PE=1 SV=2 - [MBB1A_HUMAN]	20.93	1	25	25	72	0.731	0.763	0.770	1.129	223.04	20.93	38	72	1328	148.8	9.28
Q9NR50	Translation initiation factor eIF-2B subunit gamma OS=Homo sapiens GN=EIF2B3 PE=1 SV=1 - [EI2BG_HUMAN]	20.13	1	10	10	21	1.073	0.707	0.799	1.129	31.46	20.13	16	21	452	50.2	6.47
O60486	Plexin-C1 OS=Homo sapiens GN=PLXNC1 PE=1 SV=1 - [PLXC1_HUMAN]	0.77	1	1	1	1	5.746	1.186	0.822	1.130	0.00	0.77	1	1	1568	175.6	7.61
Q9BUV8	Uncharacterized protein C20orf24 OS=Homo sapiens GN=C20orf24 PE=2 SV=1 - [CT024_HUMAN]	11.68	1	1	1	3	0.919	0.722	0.801	1.130	3.53	11.68	2	3	137	15.5	5.20

P55072	Transitional endoplasmic reticulum ATPase OS=Homo sapiens GN=VCP PE=1 SV=4 - [TERA_HUMAN]	57.69	4	47	47	778	1.142	0.941	1.071	1.130	1887.06	57.69	81	778	806	89.3	5.26
O95372	Acyl-protein thioesterase 2 OS=Homo sapiens GN=LYPLA2 PE=1 SV=1 - [LYPA2_HUMAN]	36.80	1	8	8	33	1.015	1.114	1.469	1.130	55.08	36.80	16	33	231	24.7	7.23
O43159	Ribosomal RNA-processing protein 8 OS=Homo sapiens GN=RRP8 PE=1 SV=2 - [RRP8_HUMAN]	13.82	1	5	5	13	0.864	0.970	0.785	1.130	37.36	13.82	7	13	456	50.7	9.42
O95352	Ubiquitin-like modifier-activating enzyme ATG7 OS=Homo sapiens GN=ATG7 PE=1 SV=1 - [ATG7_HUMAN]	19.49	1	12	12	29	1.027	1.097	1.061	1.130	73.71	19.49	18	29	703	77.9	6.24
Q13137	Calcium-binding and coiled-coil domain-containing protein 2 OS=Homo sapiens GN=CALCOCO2 PE=1 SV=1 - [CACO2_HUMAN]	5.16	1	2	2	8	1.336	1.059	1.118	1.130	14.70	5.16	4	8	446	52.2	5.02
Q92600	Cell differentiation protein RCD1 homolog OS=Homo sapiens GN=RQCD1 PE=1 SV=1 - [RCD1_HUMAN]	11.71	1	4	4	4	0.916	1.312	1.295	1.130	5.83	11.71	4	4	299	33.6	8.03
Q9Y6K9	NF-kappa-B essential modulator OS=Homo sapiens GN=IKBKG PE=1 SV=2 - [NEMO_HUMAN]	34.37	1	14	14	61	1.069	1.013	1.111	1.130	160.59	34.37	25	61	419	48.2	5.71
O76064	E3 ubiquitin-protein ligase RNF8 OS=Homo sapiens GN=RNF8 PE=1 SV=1 - [RNF8_HUMAN]	2.27	1	1	1	1	1.167	0.851	2.017	1.130	1.94	2.27	1	1	485	55.5	7.33

P39687	Acidic leucine-rich nuclear phosphoprotein 32 family member A OS=Homo sapiens GN=ANP32A PE=1 SV=1 - [AN32A_HUMAN]	45.38	3	6	12	68	0.872	1.507	1.025	1.130	138.26	45.38	17	68	249	28.6	4.09
Q9BRQ6	MICOS complex subunit MIC25 OS=Homo sapiens GN=CHCHD6 PE=1 SV=1 - [MIC25_HUMAN]	27.66	1	7	7	13	0.627	1.288	1.259	1.130	38.50	27.66	10	13	235	26.4	8.85
Q13310	Polyadenylate-binding protein 4 OS=Homo sapiens GN=PABPC4 PE=1 SV=1 - [PABP4_HUMAN]	33.54	3	13	22	114	1.043	1.003	0.953	1.131	273.88	33.54	37	114	644	70.7	9.26
P78346	Ribonuclease P protein subunit p30 OS=Homo sapiens GN=RPP30 PE=1 SV=1 - [RPP30_HUMAN]	5.60	1	1	1	1	0.874	0.750	0.793	1.131	2.07	5.60	1	1	268	29.3	8.91
P57723	Poly(rC)-binding protein 4 OS=Homo sapiens GN=PCBP4 PE=2 SV=1 - [PCBP4_HUMAN]	4.71	1	1	2	10	0.642	0.689	0.664	1.131	19.35	4.71	3	10	403	41.5	8.16
Q7RTY7	Ovochymase-1 OS=Homo sapiens GN=OVCH1 PE=2 SV=2 - [OVCH1_HUMAN]	1.15	1	1	1	1	0.576	0.612	0.753	1.131	0.00	1.15	1	1	1134	125.0	8.32
Q8NEM8	Cytosolic carboxypeptidase 3 OS=Homo sapiens GN=AGBL3 PE=2 SV=2 - [CBPC3_HUMAN]	2.10	1	1	2	2	1.456	1.688	1.073	1.131	1.66	2.10	2	2	1001	115.9	8.79
Q7L0Y3	Mitochondrial ribonuclease P protein 1 OS=Homo sapiens GN=TRMT10C PE=1 SV=2 - [MRRP1_HUMAN]	25.31	1	9	9	26	0.912	1.251	0.988	1.131	52.35	25.31	13	26	403	47.3	9.36
Q12849	G-rich sequence factor 1 OS=Homo sapiens GN=GRSF1 PE=1 SV=3 - [GRSF1_HUMAN	20.42	1	6	6	20	0.803	1.098	1.252	1.131	59.40	20.42	9	20	480	53.1	6.19

Q8IX12	Cell division cycle and apoptosis regulator protein 1 OS=Homo sapiens GN=CCAR1 PE=1 SV=2 - [CCAR1_HUMAN]	12.78	1	14	14	28	1.027	1.185	0.857	1.132	53.27	12.78	19	28	1150	132.7	5.76
P20396	Pro-thyrotropin-releasing hormone OS=Homo sapiens GN=TRH PE=1 SV=1 - [TRH_HUMAN]	8.68	1	1	1	1	1.291	1.022	0.866	1.132	0.00	8.68	1	1	242	27.4	5.45
Q6ZRH7	Cation channel sperm-associated protein subunit gamma OS=Homo sapiens GN=CATSPERG PE=2 SV=3 - [CTSRG_HUMAN]	0.60	1	1	1	1	1.556	0.725	0.854	1.132	1.75	0.60	1	1	1159	132.9	6.37
Q8TBC5	Zinc finger and SCAN domain-containing protein 18 OS=Homo sapiens GN=ZSCAN18 PE=2 SV=2 - [ZSC18_HUMAN]	3.53	1	2	2	3	1.166	1.393	1.056	1.132	7.01	3.53	3	3	510	54.8	4.82
Q8IUR5	Transmembrane and TPR repeat-containing protein 1 OS=Homo sapiens GN=TMTC1 PE=1 SV=3 - [TMTC1_HUMA	1.93	1	2	2	5	2.642	0.562	0.586	1.132	15.01	1.93	4	5	882	98.8	8.84
P34741	Syndecan-2 OS=Homo sapiens GN=SDC2 PE=1 SV=2 - [SDC2_HUMAN]	11.94	1	2	2	12	1.717	0.676	1.174	1.132	20.97	11.94	3	12	201	22.1	4.86
Q9H3H9	Transcription elongation factor A protein-like 2 OS=Homo sapiens GN=TCEAL2 PE=2 SV=1 - [TCAL2_HUMAN]	13.66	2	2	2	3	0.857	1.628	1.968	1.132	5.15	13.66	3	3	227	25.8	5.94
Q6NYC1	Bifunctional arginine demethylase and lysyl-hydroxylase JMJD6 OS=Homo sapiens GN=JMJD6 PE=1 SV=1 - [JMJD6_HUMAN]	8.44	1	3	3	4	1.105	0.848	1.237	1.132	10.29	8.44	4	4	403	46.4	8.69

P48307	Tissue factor pathway inhibitor 2 OS=Homo sapiens GN=TFPI2 PE=1 SV=1 - [TFPI2_HUMAN]	11.06	1	2	2	9	2.580	0.646	0.595	1.132	18.39	11.06	4	9	235	26.9	8.53
Q9H013	Disintegrin and metalloproteinase domain-containing protein 19 OS=Homo sapiens GN=ADAM19 PE=1 SV=3 - [ADA19_HUMAN]	1.05	1	1	1	1	0.562	0.928	1.951	1.132	1.78	1.05	1	1	955	104.9	8.35
Q8WUD6	Cholinephosphotransferase 1 OS=Homo sapiens GN=CHPT1 PE=1 SV=1 - [CHPT1_HUMAN]	2.96	1	1	1	2	1.049	0.858	1.316	1.132	5.30	2.96	1	2	406	45.1	6.92
Q9P2Y5	UV radiation resistance-associated gene protein OS=Homo sapiens GN=UVRAG PE=1 SV=1 - [UVRAG_HUMAN]	1.00	1	1	1	1	1.737	0.644	0.857	1.132	2.16	1.00	1	1	699	78.1	8.29
Q9UKA4	A-kinase anchor protein 11 OS=Homo sapiens GN=AKAP11 PE=1 SV=1 - [AKA11_HUMAN]	2.26	1	4	4	8	0.626	0.784	0.927	1.132	18.00	2.26	5	8	1901	210.4	5.39
Q8WXD9	Caskin-1 OS=Homo sapiens GN=CASKIN1 PE=1 SV=1 - [CSKI1_HUMAN]	0.56	1	1	1	2	1.065	1.095	0.983	1.132	5.17	0.56	1	2	1431	149.7	9.14
Q7Z5R6	Amyloid beta A4 precursor protein-binding family B member 1-interacting protein OS=Homo sapiens GN=APBB1IP PE=1 SV=1 - [AB1IP_HUMAN]	5.86	1	3	3	4	0.698	0.987	0.817	1.133	9.16	5.86	3	4	666	73.1	5.59
Q8N1G2	Cap-specific mRNA (nucleoside-2'-O-)-methyltransferase 1 OS=Homo sapiens GN=CMTR1 PE=1 SV=1 - [CMTR1_HUMA	10.54	1	7	9	20	1.088	1.184	1.070	1.133	44.28	10.54	13	20	835	95.3	7.05

P08237	ATP-dependent 6-phosphofructokinase, muscle type OS=Homo sapiens GN=PFKM PE=1 SV=2 - [PFKAM_HUMAN]	28.46	2	17	19	60	1.292	1.003	0.908	1.133	167.81	28.46	28	60	780	85.1	7.99
Q01546	Keratin, type II cytoskeletal 2 oral OS=Homo sapiens GN=KRT76 PE=1 SV=2 - [K22O_HUMAN]	8.15	2	1	7	94	1.351	1.098	1.150	1.133	184.58	8.15	10	94	638	65.8	8.12
Q6UXH9	Inactive serine protease PAMR1 OS=Homo sapiens GN=PAMR1 PE=1 SV=1 - [PAMR1_HUMAN]	1.53	1	1	1	1	0.971	0.779	1.248	1.134	2.27	1.53	1	1	720	80.1	7.46
O95168	NADH dehydrogenase [ubiquinone] 1 beta subcomplex subunit 4 OS=Homo sapiens GN=NDUFB4 PE=1 SV=3 - [NDUB4_HUMAN]	43.41	1	5	6	12	0.723	0.980	0.716	1.134	33.73	43.41	8	12	129	15.2	9.85
Q9H808	Transducin-like enhancer protein 6 OS=Homo sapiens GN=TLE6 PE=2 SV=2 - [TLE6_HUMAN]	2.10	1	1	1	1	0.580	0.695	0.938	1.134	1.93	2.10	1	1	572	63.4	6.15
P16499	Rod cGMP-specific 3',5'-cyclic phosphodiesterase subunit alpha OS=Homo sapiens GN=PDE6A PE=1 SV=4 - [PDE6A_HUMAN]	0.81	1	1	1	2	1.325	0.919	0.955	1.134	2.93	0.81	1	2	860	99.5	5.72
Q8IZ69	tRNA (uracil-5-)-methyltransferase homolog A OS=Homo sapiens GN=TRMT2A PE=1 SV=2 - [TRM2A_HUMAN]	4.80	1	2	2	3	0.925	0.892	0.861	1.134	9.26	4.80	3	3	625	68.7	7.94
Q2TAL8	Glutamine-rich protein 1 OS=Homo sapiens GN=QRICH1 PE=1 SV=1 - [QRIC1_HUMAN]	5.80	1	4	5	17	1.045	1.131	0.941	1.134	32.06	5.80	8	17	776	86.4	5.87

P26885	Peptidyl-prolyl cis-trans isomerase FKBP2 OS=Homo sapiens GN=FKBP2 PE=1 SV=2 - [FKBP2_HUMAN]	50.00	1	11	11	66	1.124	0.989	0.976	1.134	146.99	50.00	17	66	142	15.6	9.13
B7ZW38	Heterogeneous nuclear ribonucleoprotein C-like 3 OS=Homo sapiens GN=HNRNPCL3 PE=2 SV=1 - [HNRC3_HUMAN]	12.97	3	1	7	62	0.835	1.245	0.991	1.135	132.86	12.97	12	62	293	32.0	5.68
Q9Y530	O-acetyl-ADP-ribose deacetylase 1 OS=Homo sapiens GN=OARD1 PE=1 SV=2 - [OARD1_HUMAN]	17.11	1	2	3	6	1.495	1.668	1.021	1.135	12.84	17.11	4	6	152	17.0	8.31
P61009	Signal peptidase complex subunit 3 OS=Homo sapiens GN=SPCS3 PE=1 SV=1 - [SPCS3_HUMAN	28.33	1	6	6	17	0.650	0.669	0.831	1.135	31.71	28.33	9	17	180	20.3	8.62
A6NCF6	Putative MAGE domain-containing protein MAGEA13P OS=Homo sapiens GN=MAGEA13P PE=5 SV=1 - [MA13P_HUMAN]	2.93	1	1	1	1	1.122	0.615	0.518	1.136	2.83	2.93	1	1	341	37.9	6.37
Q8WUJ1	Neuferricin OS=Homo sapiens GN=CYB5D2 PE=2 SV=1 - [NEUFC_HUMAN]	6.06	1	1	1	2	1.045	1.032	2.070	1.136	4.69	6.06	2	2	264	28.7	8.40
P24752	Acetyl-CoA acetyltransferase, mitochondrial OS=Homo sapiens GN=ACAT1 PE=1 SV=1 - [THIL_HUMAN]	36.07	1	18	18	144	1.112	1.616	1.182	1.136	348.43	36.07	31	144	427	45.2	8.85
Q05519	Serine/arginine-rich splicing factor 11 OS=Homo sapiens GN=SRSF11 PE=1 SV=1 - [SRS11_HUMAN]	16.53	1	7	7	23	1.008	1.143	0.980	1.136	46.65	16.53	10	23	484	53.5	10.52

P10636	Microtubule-associated protein tau OS=Homo sapiens GN=MAPT PE=1 SV=5 - [TAU_HUMAN]	7.39	1	4	4	5	1.284	1.503	0.612	1.136	9.73	7.39	4	5	758	78.9	6.71
O96008	Mitochondrial import receptor subunit TOM40 homolog OS=Homo sapiens GN=TOMM40 PE=1 SV=1 - [TOM40_HUMA	16.62	1	5	5	31	1.135	0.775	0.623	1.136	69.72	16.62	10	31	361	37.9	7.25
Q9H0W9	Ester hydrolase C11orf54 OS=Homo sapiens GN=C11orf54 PE=1 SV=1 - [CK054_HUMAN]	20.95	1	6	6	14	1.514	1.178	0.913	1.137	27.86	20.95	9	14	315	35.1	6.70
Q9Y3B2	Exosome complex component CSL4 OS=Homo sapiens GN=EXOSC1 PE=1 SV=1 - [EXOS1_HUMA N]	8.21	1	1	1	1	1.050	0.960	0.913	1.137	4.65	8.21	1	1	195	21.4	8.24
Q9H488	GDP-fucose protein O-fucosyltransfer ase 1 OS=Homo sapiens GN=POFUT1 PE=1 SV=1 - [OFUT1_HUMA	27.58	1	7	7	56	1.212	0.975	1.023	1.137	132.52	27.58	13	56	388	43.9	8.53
O94903	Proline synthase co-transcribed bacterial homolog protein OS=Homo sapiens GN=PROSC PE=1 SV=1 - [PROSC_HUMA	44.73	1	10	10	43	1.071	1.028	1.025	1.137	100.77	44.73	17	43	275	30.3	7.50
Q9Y2X3	Nucleolar protein 58 OS=Homo sapiens GN=NOP58 PE=1 SV=1 - [NOP58_HUMA N]	37.81	1	16	16	58	0.843	1.042	0.846	1.137	169.93	37.81	25	58	529	59.5	8.92
Q9H3K6	BolA-like protein 2 OS=Homo sapiens GN=BOLA2 PE=1 SV=1 - [BOLA2_HUMAN]	80.23	1	6	6	16	0.835	0.906	1.360	1.138	42.98	80.23	9	16	86	10.1	6.52

P51784	Ubiquitin carboxyl-terminal hydrolase 11 OS=Homo sapiens GN=USP11 PE=1 SV=3 - [UBP11_HUMAN]	6.65	1	6	7	16	0.955	0.831	0.985	1.138	47.81	6.65	9	16	963	109.7	5.45
P57740	Nuclear pore complex protein Nup107 OS=Homo sapiens GN=NUP107 PE=1 SV=1 - [NU107_HUMAN	17.41	1	12	12	29	0.971	1.079	0.869	1.138	68.16	17.41	17	29	925	106.3	5.43
Q9NY12	H/ACA ribonucleoprotein complex subunit 1 OS=Homo sapiens GN=GAR1 PE=1 SV=1 - [GAR1_HUMAN]	36.41	1	8	8	17	1.116	0.973	0.775	1.138	27.03	36.41	10	17	217	22.3	10.92
Q08752	Peptidyl-prolyl cis-trans isomerase D OS=Homo sapiens GN=PPID PE=1 SV=3 - [PPID_HUMAN]	50.00	1	17	18	38	0.915	1.206	1.081	1.138	77.10	50.00	25	38	370	40.7	7.21
O60518	Ran-binding protein 6 OS=Homo sapiens GN=RANBP6 PE=1 SV=2 - [RNBP6_HUMAN]	4.52	2	5	6	15	1.115	1.007	1.406	1.138	34.08	4.52	7	15	1105	124.6	5.01
Q16082	Heat shock protein beta-2 OS=Homo sapiens GN=HSPB2 PE=1 SV=2 - [HSPB2_HUMAN]	24.73	1	3	3	5	0.848	0.520	0.784	1.138	16.64	24.73	5	5	182	20.2	5.17
Q9NVH0	Exonuclease 3'-5' domain-containing protein 2 OS=Homo sapiens GN=EXD2 PE=1 SV=2 - [EXD2_HUMAN]	9.18	1	6	6	15	0.843	0.916	1.091	1.138	35.09	9.18	9	15	621	70.3	8.32
Q92552	28S ribosomal protein S27, mitochondrial OS=Homo sapiens GN=MRPS27 PE=1 SV=3 - [RT27_HUMAN]	6.76	1	3	3	11	0.617	0.770	0.675	1.138	24.14	6.76	6	11	414	47.6	6.18
Q9Y519	Transmembrane protein 184B OS=Homo sapiens GN=TMEM184B PE=1 SV=2 - [T184B_HUMAN]	1.72	1	1	1	2	1.003	0.383	0.535	1.138	4.50	1.72	2	2	407	45.5	6.89

P31942	Heterogeneous nudear ribonudeoprotein H3 OS=Homo sapiens GN=HNRNPH3 PE=1 SV=2 - [HNRH3_HUMAN]	35.55	2	7	9	84	1.186	1.104	1.054	1.138	248.08	35.55	15	84	346	36.9	6.87
Q9UPN4	Centrosomal protein of 131 kDa OS=Homo sapiens GN=CEP131 PE=1 SV=3 - [CP131_HUMAN]	4.43	1	4	5	16	0.836	1.625	1.027	1.139	40.96	4.43	6	16	1083	122.1	8.69
Q86X76	Nitrilase homolog 1 OS=Homo sapiens GN=NIT1 PE=1 SV=2 - [NIT1_HUMAN]	8.56	1	3	3	8	1.125	1.110	1.046	1.139	15.93	8.56	5	8	327	35.9	7.74
Q70EL1	Inactive ubiquitin carboxyl-terminal hydrolase 54 OS=Homo sapiens GN=USP54 PE=1 SV=4 - [UBP54_HUMAN]	1.66	1	2	2	2	0.956	0.834	1.043	1.139	4.54	1.66	2	2	1684	187.3	7.28
Q9H9P8	L-2-hydroxyglutarate dehydrogenase, mitochondrial OS=Homo sapiens GN=L2HGDH PE=1 SV=3 - [L2HDH_HUMAN]	4.32	1	2	2	3	0.932	1.309	0.946	1.139	7.08	4.32	3	3	463	50.3	8.15
Q9NYL2	Mitogen-activated protein kinase kinase kinase MLT OS=Homo sapiens GN=ZAK PE=1 SV=3 - [MLTK_HUMAN]	11.25	1	10	11	24	1.180	1.013	1.775	1.139	55.06	11.25	17	24	800	91.1	7.87
Q86Y39	NADH dehydrogenase [ubiquinone] 1 alpha subcomplex subunit 11 OS=Homo sapiens GN=NDUFA11 PE=1 SV=3 - [NDUAB_HUMAN]	19.86	1	2	2	4	0.740	0.591	1.349	1.139	11.09	19.86	3	4	141	14.8	8.72
P16070	CD44 antigen OS=Homo sapiens GN=CD44 PE=1 SV=3 - [CD44_HUMAN]	16.58	1	15	15	374	1.773	0.744	1.041	1.139	785.22	16.58	26	374	742	81.5	5.33

Q08379	Golgin subfamily A member 2 OS=Homo sapiens GN=GOLGA2 PE=1 SV=3 - [GOGA2_HUMAN]	24.65	2	22	23	73	0.997	0.956	1.001	1.139	150.83	24.65	36	73	1002	113.0	5.02
Q9C0A0	Contactin-associated protein-like 4 OS=Homo sapiens GN=CNTNAP4 PE=1 SV=3 - [CNTP4_HUMAN]	2.37	1	2	3	19	1.364	0.714	1.074	1.139	30.60	2.37	4	19	1308	145.2	6.68
Q9H0U4	Ras-related protein Rab-1B OS=Homo sapiens GN=RAB1B PE=1 SV=1 - [RAB1B_HUMAN]	81.09	3	7	17	114	1.295	1.178	1.039	1.139	277.26	81.09	27	114	201	22.2	5.73
P52948	Nuclear pore complex protein Nup98-Nup96 OS=Homo sapiens GN=NUP98 PE=1 SV=4 - [NUP98_HUMAN	17.34	1	28	28	88	1.156	1.130	0.842	1.139	190.42	17.34	48	88	1817	197.5	6.40
Q8WYQ9	Zinc finger CCHC domain-containing protein 14 OS=Homo sapiens GN=ZCCHC14 PE=2 SV=1 - [ZCH14_HUMAN]	0.74	1	1	1	1	1.249	1.324	1.214	1.140	2.02	0.74	1	1	949	100.0	8.18
Q9Y2U8	Inner nuclear membrane protein Man1 OS=Homo sapiens GN=LEMD3 PE=1 SV=2 - [MAN1_HUMAN]	10.76	1	6	7	11	1.080	0.882	0.969	1.140	24.16	10.76	9	11	911	99.9	7.55
Q9H9F9	Actin-related protein 5 OS=Homo sapiens GN=ACTR5 PE=1 SV=2 - [ARP5_HUMAN]	1.15	1	1	1	2	0.863	0.863	0.659	1.140	4.60	1.15	1	2	607	68.3	5.27
Q86T26	Transmembrane protease serine 11B OS=Homo sapiens GN=TMPRSS11B PE=2 SV=3 - [TM11B_HUMA	1.44	1	1	1	1	1.149	1.192	1.004	1.140	2.28	1.44	1	1	416	46.3	8.85
P36542	ATP synthase subunit gamma, mitochondrial OS=Homo sapiens GN=ATP5C1 PE=1 SV=1 - [ATPG_HUMAN]	29.87	1	11	12	66	1.274	0.892	0.686	1.140	166.70	29.87	19	66	298	33.0	9.22

Q9H2A2	Aldehyde dehydrogenase family 8 member A1 OS=Homo sapiens GN=ALDH8A1 PE=1 SV=1 - [AL8A1_HUMAN]	2.87	1	1	2	4	1.219	0.736	2.545	1.140	8.72	2.87	2	4	487	53.4	7.14
Q8IWD4	Coiled-coil domain-containing protein 117 OS=Homo sapiens GN=CCDC117 PE=1 SV=1 - [CC117_HUMAN]	3.94	1	1	1	3	0.847	0.888	0.621	1.140	6.87	3.94	2	3	279	30.5	5.07
Q15428	Splicing factor 3A subunit 2 OS=Homo sapiens GN=SF3A2 PE=1 SV=2 - [SF3A2_HUMAN]	5.82	1	3	3	11	1.256	1.147	1.109	1.140	30.67	5.82	5	11	464	49.2	9.64
Q13423	NAD(P) transhydrogenase, mitochondrial OS=Homo sapiens GN=NNT PE=1 SV=3 - [NNTM_HUMAN]	34.07	1	39	40	132	1.030	1.191	0.868	1.141	312.16	34.07	61	132	1086	113.8	8.09
O00746	Nucleoside diphosphate kinase, mitochondrial OS=Homo sapiens GN=NME4 PE=1 SV=1 - [NDKM_HUMAN]	20.32	1	4	4	6	0.758	1.085	1.050	1.141	14.21	20.32	6	6	187	20.6	10.29
O00267	Transcription elongation factor SPT5 OS=Homo sapiens GN=SUPT5H PE=1 SV=1 - [SPT5H_HUMAN]	14.90	1	13	13	39	1.120	1.056	1.019	1.141	96.37	14.90	22	39	1087	120.9	5.06
P15153	Ras-related C3 botulinum toxin substrate 2 OS=Homo sapiens GN=RAC2 PE=1 SV=1 - [RAC2_HUMAN]	24.48	2	1	6	64	0.916	0.844	0.598	1.141	149.61	24.48	10	64	192	21.4	7.61
P37235	Hippocalcin-like protein 1 OS=Homo sapiens GN=HPCAL1 PE=1 SV=3 - [HPCL1_HUMAN]	36.79	3	7	7	40	1.329	0.594	0.692	1.141	86.68	36.79	13	40	193	22.3	5.35

Q8NCU8	Uncharacterized protein encoded by LINC00116 OS=Homo sapiens GN=LINC00116 PE=1 SV=1 - [YB039_HUMAN	4.35	1	1	1	4	0.860	0.808	0.517	1.141	8.30	4.35	1	4	138	15.6	11.19
P24928	DNA-directed RNA polymerase II subunit RPB1 OS=Homo sapiens GN=POLR2A PE=1 SV=2 - [RPB1_HUMAN]	13.45	1	25	25	47	0.935	1.191	0.937	1.141	100.96	13.45	35	47	1970	217.0	7.37
Q969T7	7-methylguanosine phosphate-specific 5'-nucleotidase OS=Homo sapiens GN=NT5C3B PE=1 SV=4 - [5NT3B_HUMAN]	9.67	1	3	3	12	1.124	1.052	1.106	1.141	24.52	9.67	6	12	300	34.4	6.38
Q9P258	Protein RCC2 OS=Homo sapiens GN=RCC2 PE=1 SV=2 - [RCC2_HUMAN]	6.51	1	4	4	14	0.850	1.324	0.851	1.141	33.91	6.51	8	14	522	56.0	8.78
P31146	Coronin-1A OS=Homo sapiens GN=CORO1A PE=1 SV=4 - [COR1A_HUMAN]	4.99	1	1	2	3	0.774	1.843	1.693	1.141	5.45	4.99	2	3	461	51.0	6.68
Q9Y3L3	SH3 domain-binding protein 1 OS=Homo sapiens GN=SH3BP1 PE=1 SV=3 - [3BP1_HUMAN]	16.55	1	11	11	30	0.705	0.809	1.120	1.141	74.64	16.55	14	30	701	75.7	6.77
Q86UX6	Serine/threonine-protein kinase 32C OS=Homo sapiens GN=STK32C PE=1 SV=1 - [ST32C_HUMAN	4.32	1	1	1	1	1.240	1.180	1.329	1.142	0.00	4.32	1	1	486	55.0	6.61
Q8IY33	MICAL-like protein 2 OS=Homo sapiens GN=MICALL2 PE=1 SV=1 - [MILK2_HUMAN]	23.56	2	17	17	42	1.959	1.041	1.655	1.142	97.57	23.56	24	42	904	97.4	9.57
Q5T3I0	G patch domain-containing protein 4 OS=Homo sapiens GN=GPATCH4 PE=1 SV=2 - [GPTC4_HUMAN	5.61	1	2	2	3	0.601	0.868	0.991	1.142	3.90	5.61	3	3	446	50.4	9.63

P62877	E3 ubiquitin-protein ligase RBX1 OS=Homo sapiens GN=RBX1 PE=1 SV=1 - [RBX1_HUMAN]	12.96	1	2	2	4	3.115	1.565	1.346	1.142	8.50	12.96	3	4	108	12.3	6.96
Q9UGH3	Solute carrier family 23 member 2 OS=Homo sapiens GN=SLC23A2 PE=1 SV=1 - [S23A2_HUMAN]	6.31	1	2	3	3	3.804		0.355	1.142	9.09	6.31	3	3	650	70.3	7.68
Q9UQ88	Cyclin-dependent kinase 11A OS=Homo sapiens GN=CDK11A PE=1 SV=4 - [CD11A_HUMAN]	4.60	2	4	4	22	0.925	1.224	0.951	1.142	35.00	4.60	6	22	783	91.3	5.36
Q9BW92	Threonine--tRNA ligase, mitochondrial OS=Homo sapiens GN=TARS2 PE=1 SV=1 - [SYTM_HUMAN]	5.85	1	5	5	8	0.869	1.758	1.277	1.142	21.44	5.85	7	8	718	81.0	7.30
Q9Y3Y2	Chromatin target of PRMT1 protein OS=Homo sapiens GN=CHTOP PE=1 SV=2 - [CHTOP_HUMAN]	21.37	1	5	5	34	1.231	1.323	1.177	1.143	54.16	21.37	9	34	248	26.4	12.23
Q9BQ70	Transcription factor 25 OS=Homo sapiens GN=TCF25 PE=1 SV=1 - [TCF25_HUMAN]	14.79	1	8	8	23	1.033	1.026	1.053	1.143	43.93	14.79	14	23	676	76.6	6.35
Q9Y262	Eukaryotic translation initiation factor 3 subunit L OS=Homo sapiens GN=EIF3L PE=1 SV=1 - [EIF3L_HUMAN]	43.44	1	24	24	88	0.823	0.757	0.821	1.143	204.68	43.44	43	88	564	66.7	6.34
P35869	Aryl hydrocarbon receptor OS=Homo sapiens GN=AHR PE=1 SV=2 - [AHR_HUMAN]	1.30	1	1	1	2	0.577	0.892	0.261	1.143	0.00	1.30	2	2	848	96.1	6.38

O14672	Disintegrin and metalloproteinase domain-containing protein 10 OS=Homo sapiens GN=ADAM10 PE=1 SV=1 - [ADA10_HUMAN]	20.32	1	13	13	42	1.160	0.551	0.782	1.143	101.66	20.32	20	42	748	84.1	7.77
Q9NSA3	Beta-catenin-interacting protein 1 OS=Homo sapiens GN=CTNNBIP1 PE=1 SV=1 - [CNBP1_HUMAN]	18.52	1	2	2	7	1.123	0.834	1.324	1.143	19.99	18.52	4	7	81	9.2	5.41
P35241	Radixin OS=Homo sapiens GN=RDX PE=1 SV=1 - [RADI_HUMAN]	53.34	1	25	42	343	1.049	1.054	0.906	1.143	771.25	53.34	68	343	583	68.5	6.37
Q8TF42	Ubiquitin-associated and SH3 domain-containing protein B OS=Homo sapiens GN=UBASH3B PE=1 SV=2 - [UBS3B_HUMAN]	5.39	1	3	3	12	0.843	0.983	0.991	1.143	24.91	5.39	6	12	649	72.6	6.93
O95140	Mitofusin-2 OS=Homo sapiens GN=MFN2 PE=1 SV=3 - [MFN2_HUMAN]	12.29	1	7	7	12	0.944	0.952	1.028	1.144	26.11	12.29	10	12	757	86.3	6.98
Q8NBN7	Retinol dehydrogenase 13 OS=Homo sapiens GN=RDH13 PE=1 SV=2 - [RDH13_HUMAN]	3.02	1	1	1	3	0.818	1.245	1.207	1.144	6.72	3.02	2	3	331	35.9	8.10
Q9ULH0	Kinase D-interacting substrate of 220 kDa OS=Homo sapiens GN=KIDINS220 PE=1 SV=3 - [KDIS_HUMAN]	6.27	1	8	8	16	1.080	0.860	1.079	1.144	35.09	6.27	13	16	1771	196.4	6.62
Q9GZL7	Ribosome biogenesis protein WDR12 OS=Homo sapiens GN=WDR12 PE=1 SV=2 - [WDR12_HUMAN]	9.22	1	4	4	4	0.984	1.332	0.913	1.144	7.97	9.22	4	4	423	47.7	5.90
Q15459	Splicing factor 3A subunit 1 OS=Homo sapiens GN=SF3A1 PE=1 SV=1 - [SF3A1_HUMAN]	37.96	2	29	30	105	1.123	1.257	0.891	1.144	250.62	37.96	45	105	793	88.8	5.22

Q9Y2X7	ARF GTPase-activating protein GIT1 OS=Homo sapiens GN=GIT1 PE=1 SV=2 - [GIT1_HUMAN]	20.37	1	11	14	42	1.204	1.189	1.074	1.144	99.64	20.37	22	42	761	84.3	6.80
Q9Y223	Bifunctional UDP-N-acetylglucosamine 2-epimerase/N-acetylmannosamine kinase OS=Homo sapiens GN=GNE PE=1 SV=1 - [GLCNE_HUMAN]	8.45	1	5	5	11	1.025	0.890	1.248	1.144	25.27	8.45	8	11	722	79.2	6.80
Q9UKG9	Peroxisomal carnitine O-octanoyltransferase OS=Homo sapiens GN=CROT PE=1 SV=2 - [OCTC_HUMAN]	15.03	1	10	10	23	0.754	1.144	1.255	1.144	48.06	15.03	16	23	612	70.1	7.08
Q9UHR4	Brain-specific angiogenesis inhibitor 1-associated protein 2-like protein 1 OS=Homo sapiens GN=BAIAP2L1 PE=1 SV=2 - [BI2L1_HUMAN]	10.96	1	4	5	9	0.822	0.890	0.929	1.144	14.75	10.96	8	9	511	56.8	8.68
O43353	Receptor-interacting serine/threonine-protein kinase 2 OS=Homo sapiens GN=RIPK2 PE=1 SV=2 - [RIPK2_HUMAN	17.96	1	8	8	12	0.864	0.731	0.609	1.144	35.30	17.96	9	12	540	61.2	7.09
Q9H7B2	Ribosome production factor 2 homolog OS=Homo sapiens GN=RPF2 PE=1 SV=2 - [RPF2_HUMAN]	18.30	1	9	9	17	0.796	1.120	0.980	1.144	34.29	18.30	11	17	306	35.6	9.99
P46926	Glucosamine-6-phosphate isomerase 1 OS=Homo sapiens GN=GNPDA1 PE=1 SV=1 - [GNPI1_HUMAN]	26.99	1	6	8	24	0.789	0.880	0.926	1.145	43.68	26.99	13	24	289	32.6	6.92

Q9UG01	Intraflagellar transport protein 172 homolog OS=Homo sapiens GN=IFT172 PE=1 SV=2 - [IF172_HUMAN]	1.89	1	2	2	2	0.464	0.897	1.052	1.145	8.13	1.89	2	2	1749	197.5	6.13
O75792	Ribonuclease H2 subunit A OS=Homo sapiens GN=RNASEH2A PE=1 SV=2 - [RNH2A_HUMAN]	14.38	1	4	4	11	0.840	1.363	1.055	1.145	32.96	14.38	4	11	299	33.4	5.25
P55774	C-C motif chemokine 18 OS=Homo sapiens GN=CCL18 PE=1 SV=1 - [CCL18_HUMAN]	12.36	1	1	1	2	2.034	1.502	0.913	1.145	5.66	12.36	1	2	89	9.8	8.75
Q8TC12	Retinol dehydrogenase 11 OS=Homo sapiens GN=RDH11 PE=1 SV=2 - [RDH11_HUMAN]	16.98	1	4	4	15	1.417	0.887	0.804	1.145	27.33	16.98	7	15	318	35.4	8.82
Q96M96	FYVE, RhoGEF and PH domain-containing protein 4 OS=Homo sapiens GN=FGD4 PE=1 SV=2 - [FGD4_HUMAN]	26.37	1	16	16	38	0.926	0.867	1.309	1.145	93.11	26.37	25	38	766	86.6	6.13
Q86T90	Uncharacterized protein KIAA1328 OS=Homo sapiens GN=KIAA1328 PE=1 SV=2 - [K1328_HUMAN]	5.20	2	3	4	15	0.850	0.639	0.873	1.145	24.75	5.20	4	15	577	65.3	8.09
Q9H3Q1	Cdc42 effector protein 4 OS=Homo sapiens GN=CDC42EP4 PE=1 SV=1 - [BORG4_HUMAN]	12.08	1	4	4	10	1.184	1.353	1.157	1.146	15.72	12.08	7	10	356	38.0	5.19
O95182	NADH dehydrogenase [ubiquinone] 1 alpha subcomplex subunit 7 OS=Homo sapiens GN=NDUFA7 PE=1 SV=3 - [NDUA7_HUMAN]	72.57	1	9	9	25	0.728	1.077	0.812	1.146	64.16	72.57	16	25	113	12.5	10.18

P18846	Cyclic AMP-dependent transcription factor ATF-1 OS=Homo sapiens GN=ATF1 PE=1 SV=2 - [ATF1_HUMAN]	5.54	1	1	2	5	0.898	0.956	0.752	1.146	8.90	5.54	3	5	271	29.2	8.37
O75410	Transforming acidic coiled-coil-containing protein 1 OS=Homo sapiens GN=TACC1 PE=1 SV=2 - [TACC1_HUMAN]	16.40	1	10	12	34	1.103	1.524	1.271	1.147	90.03	16.40	18	34	805	87.7	4.88
Q9HDC9	Adipocyte plasma membrane-associated protein OS=Homo sapiens GN=APMAP PE=1 SV=2 - [APMAP_HUMAN]	55.53	1	19	20	131	0.992	0.950	0.819	1.147	295.99	55.53	32	131	416	46.5	6.16
P30305	M-phase inducer phosphatase 2 OS=Homo sapiens GN=CDC25B PE=1 SV=2 - [MPIP2_HUMAN]	1.38	1	1	1	1	0.848	0.350	1.682	1.147	1.62	1.38	1	1	580	64.9	6.40
Q8N2F6	Armadillo repeat-containing protein 10 OS=Homo sapiens GN=ARMC10 PE=1 SV=1 - [ARM10_HUMA	15.74	1	5	5	9	0.724	1.736	0.861	1.147	11.93	15.74	9	9	343	37.5	6.61
Q9NZM3	Intersectin-2 OS=Homo sapiens GN=ITSN2 PE=1 SV=3 - [ITSN2_HUMAN]	9.07	2	12	15	27	1.070	1.516	1.184	1.147	63.84	9.07	21	27	1697	193.3	8.12
P21796	Voltage-dependent anion-selective channel protein 1 OS=Homo sapiens GN=VDAC1 PE=1 SV=2 - [VDAC1_HUMAN	71.73	1	14	16	178	0.888	0.822	0.775	1.147	532.71	71.73	28	178	283	30.8	8.54
Q9NX24	H/ACA ribonucleoprotein complex subunit 2 OS=Homo sapiens GN=NHP2 PE=1 SV=1 - [NHP2_HUMAN]	37.91	1	4	4	23	1.092	1.117	1.017	1.147	61.05	37.91	7	23	153	17.2	8.22

Q9Y238	Deleted in lung and esophageal cancer protein 1 OS=Homo sapiens GN=DLEC1 PE=2 SV=2 - [DLEC1_HUMAN	0.63	1	1	1	1	1.186	1.286	1.219	1.147	0.00	0.63	1	1	1755	195.6	6.33
Q01196	Runt-related transcription factor 1 OS=Homo sapiens GN=RUNX1 PE=1 SV=3 - [RUNX1_HUMAN]	6.40	1	2	2	3	0.743	1.102	1.145	1.147	8.74	6.40	3	3	453	48.7	9.35
Q8N612	FTS and Hook-interacting protein OS=Homo sapiens GN=FAM160A2 PE=1 SV=3 - [F16A2_HUMAN]	2.26	1	1	1	1	0.859	0.967	1.046	1.147	6.16	2.26	1	1	972	105.5	6.77
Q9P0P0	E3 ubiquitin-protein ligase RNF181 OS=Homo sapiens GN=RNF181 PE=1 SV=1 - [RN181_HUMAN]	11.11	1	2	2	5	0.535	1.004	1.233	1.147	10.69	11.11	3	5	153	17.9	5.06
Q9H9H4	Vacuolar protein sorting-associated protein 37B OS=Homo sapiens GN=VPS37B PE=1 SV=1 - [VP37B_HUMAN]	9.47	1	2	2	2	0.822	1.551	0.934	1.147	0.00	9.47	2	2	285	31.3	7.34
Q9BX69	Caspase recruitment domain-containing protein 6 OS=Homo sapiens GN=CARD6 PE=1 SV=2 - [CARD6_HUMAN]	1.16	1	1	1	2	0.333	1.479	0.882	1.147	6.35	1.16	1	2	1037	116.4	6.37
Q8NAF0	Zinc finger protein 579 OS=Homo sapiens GN=ZNF579 PE=1 SV=2 - [ZN579_HUMAN]	1.60	1	1	1	1	1.086	0.925	1.143	1.147	1.80	1.60	1	1	562	60.5	8.69
Q8TAM2	Tetratricopeptide repeat protein 8 OS=Homo sapiens GN=TTC8 PE=1 SV=2 - [TTC8_HUMAN]	1.29	1	1	1	1	0.923	1.366	1.357	1.148	0.00	1.29	1	1	541	61.5	6.80

P51948	CDK-activating kinase assembly factor MAT1 OS=Homo sapiens GN=MNAT1 PE=1 SV=1 - [MAT1_HUMAN]	10.36	1	4	4	11	1.050	1.291	1.013	1.148	9.98	10.36	5	11	309	35.8	6.09
O96000	NADH dehydrogenase [ubiquinone] 1 beta subcomplex subunit 10 OS=Homo sapiens GN=NDUFB10 PE=1 SV=3 - [NDUBA_HUMAN]	34.88	1	6	6	21	0.928	1.070	0.977	1.148	48.71	34.88	10	21	172	20.8	8.48
Q96QU8	Exportin-6 OS=Homo sapiens GN=XPO6 PE=1 SV=1 - [XPO6_HUMAN]	3.29	1	4	4	9	1.170	0.663	0.671	1.148	17.12	3.29	6	9	1125	128.8	6.35
O95881	Thioredoxin domain-containing protein 12 OS=Homo sapiens GN=TXNDC12 PE=1 SV=1 - [TXD12_HUMAN]	40.12	1	6	6	67	0.944	0.827	0.799	1.148	135.21	40.12	11	67	172	19.2	5.40
Q99613	Eukaryotic translation initiation factor 3 subunit C OS=Homo sapiens GN=EIF3C PE=1 SV=1 - [EIF3C_HUMAN]	26.94	3	26	26	178	0.846	0.940	0.970	1.148	344.39	26.94	42	178	913	105.3	5.68
Q2M2I5	Keratin, type I cytoskeletal 24 OS=Homo sapiens GN=KRT24 PE=1 SV=1 - [K1C24_HUMAN]	16.95	5	3	5	22	1.507	1.240	1.439	1.148	34.05	16.95	7	22	525	55.1	4.96
P30084	Enoyl-CoA hydratase, mitochondrial OS=Homo sapiens GN=ECHS1 PE=1 SV=4 - [ECHM_HUMAN]	52.41	1	17	17	73	1.068	1.260	1.131	1.148	169.90	52.41	28	73	290	31.4	8.07
P36915	Guanine nucleotide-binding protein-like 1 OS=Homo sapiens GN=GNL1 PE=1 SV=2 - [GNL1_HUMAN]	18.78	1	11	11	40	1.088	1.089	0.999	1.149	109.83	18.78	18	40	607	68.6	5.80

O43293	Death-associated protein kinase 3 OS=Homo sapiens GN=DAPK3 PE=1 SV=1 - [DAPK3_HUMAN	31.06	2	11	12	94	1.615	0.797	1.488	1.149	260.84	31.06	22	94	454	52.5	6.89
P16519	Neuroendocrine convertase 2 OS=Homo sapiens GN=PCSK2 PE=2 SV=2 - [NEC2_HUMAN]	7.05	1	3	3	3	1.234	1.143	0.960	1.149	4.91	7.05	3	3	638	70.5	6.49
P51398	28S ribosomal protein S29, mitochondrial OS=Homo sapiens GN=DAP3 PE=1 SV=1 - [RT29_HUMAN]	24.12	1	9	9	19	0.787	1.012	0.884	1.149	46.83	24.12	11	19	398	45.5	8.88
Q13405	39S ribosomal protein L49, mitochondrial OS=Homo sapiens GN=MRPL49 PE=1 SV=1 - [RM49_HUMAN]	36.75	1	6	6	17	0.993	1.367	1.141	1.149	42.14	36.75	8	17	166	19.2	9.45
Q14CX7	N-alpha-acetyltransferase 25, NatB auxiliary subunit OS=Homo sapiens GN=NAA25 PE=1 SV=1 - [NAA25_HUMAN]	5.86	1	7	7	14	1.014	1.105	1.088	1.149	23.71	5.86	11	14	972	112.2	6.64
Q9NYJ8	TGF-beta-activated kinase 1 and MAP3K7-binding protein 2 OS=Homo sapiens GN=TAB2 PE=1 SV=1 - [TAB2_HUMAN]	6.20	1	3	4	6	0.757	1.225	1.146	1.149	7.98	6.20	5	6	693	76.4	8.54
P0CAP2	DNA-directed RNA polymerase II subunit GRINL1A OS=Homo sapiens GN=POLR2M PE=1 SV=1 - [GRL1A_HUMAN]	2.99	2	1	1	1	0.958	1.217	1.121	1.150	1.81	2.99	1	1	368	41.7	6.39
Q13011	Delta(3,5)-Delta(2,4)-dienoyl-CoA isomerase, mitochondrial OS=Homo sapiens GN=ECH1 PE=1 SV=2 - [ECH1_HUMAN]	58.23	1	15	15	70	1.268	1.249	1.211	1.150	200.08	58.23	21	70	328	35.8	8.00

Q16610	Extracellular matrix protein 1 OS=Homo sapiens GN=ECM1 PE=1 SV=2 - [ECM1_HUMAN]	17.59	1	8	8	20	1.326	0.884	1.003	1.150	52.17	17.59	13	20	540	60.6	6.71
A4FU49	SH3 domain-containing protein 21 OS=Homo sapiens GN=SH3D21 PE=2 SV=2 - [SH321_HUMAN]	3.91	1	2	2	2	1.314	1.160	0.894	1.150	4.09	3.91	2	2	640	70.5	5.76
Q9UI26	Importin-11 OS=Homo sapiens GN=IPO11 PE=1 SV=1 - [IPO11_HUMAN]	2.05	1	2	2	4	0.909	1.162	1.072	1.150	9.60	2.05	3	4	975	112.5	5.25
A0JNW5	UHRF1-binding protein 1-like OS=Homo sapiens GN=UHRF1BP1L PE=1 SV=2 - [UH1BL_HUMAN]	7.86	1	9	9	15	1.108	0.935	0.821	1.150	33.05	7.86	13	15	1464	164.1	6.32
O14827	Ras-specific guanine nucleotide-releasing factor 2 OS=Homo sapiens GN=RASGRF2 PE=1 SV=2 - [RGRF2_HUMAN	2.10	1	3	3	3	1.484	1.406	1.131	1.151	7.59	2.10	3	3	1237	140.7	7.53
P60604	Ubiquitin-conjugating enzyme E2 G2 OS=Homo sapiens GN=UBE2G2 PE=1 SV=1 - [UB2G2_HUMAN]	41.82	1	4	4	7	1.067	0.844	0.911	1.151	20.20	41.82	5	7	165	18.6	4.70
Q9UHD1	Cysteine and histidine-rich domain-containing protein 1 OS=Homo sapiens GN=CHORDC1 PE=1 SV=2 - [CHRD1_HUMAN]	32.53	1	8	8	11	0.767	0.841	1.138	1.151	29.71	32.53	11	11	332	37.5	7.87
Q6QNY1	Biogenesis of lysosome-related organelles complex 1 subunit 2 OS=Homo sapiens GN=BLOC1S2 PE=1 SV=1 - [BL1S2_HUMAN]	4.23	1	1	1	1	0.930	1.119	1.342	1.151	2.23	4.23	1	1	142	16.0	4.86

P41219	Peripherin OS=Homo sapiens GN=PRPH PE=1 SV=2 - [PERI_HUMAN]	14.26	2	2	9	411	1.371	0.992	1.233	1.151	1004.59	14.26	15	411	470	53.6	5.47
Q9UGQ2	Calcium channel flower homolog OS=Homo sapiens GN=CACFD1 PE=2 SV=1 - [FLOWR_HUMAN]	5.23	1	1	1	1	1.082	1.366	0.952	1.151	0.00	5.23	1	1	172	18.5	5.44
Q9UL15	BAG family molecular chaperone regulator 5 OS=Homo sapiens GN=BAG5 PE=1 SV=1 - [BAG5_HUMAN]	13.65	2	5	6	22	1.016	0.841	1.030	1.151	54.60	13.65	10	22	447	51.2	6.05
Q3LXA3	Bifunctional ATP-dependent dihydroxyacetone kinase/FAD-AMP lyase (cyclizing) OS=Homo sapiens GN=DAK PE=1 SV=2 - [DHAK_HUMAN]	18.26	1	8	8	42	1.037	1.136	1.191	1.151	111.69	18.26	14	42	575	58.9	7.49
Q9H1I8	Activating signal cointegrator 1 complex subunit 2 OS=Homo sapiens GN=ASCC2 PE=1 SV=3 - [ASCC2_HUMAN	13.74	1	9	9	23	1.119	0.940	1.140	1.152	67.75	13.74	13	23	757	86.3	5.16
Q8NCX0	Coiled-coil domain-containing protein 150 OS=Homo sapiens GN=CCDC150 PE=2 SV=2 - [CC150_HUMAN]	4.45	2	4	6	7	1.332	1.012	1.871	1.152	12.78	4.45	6	7	1101	128.7	7.02
P0DMQ5	Putative transmembrane protein INAFM2 OS=Homo sapiens GN=INAFM2 PE=2 SV=1 - [INAM2_HUMAN]	7.19	1	1	1	2	1.256	1.186	0.982	1.152	2.25	7.19	1	2	153	15.4	9.31
P49585	Choline-phosphate cytidylyltransferase A OS=Homo sapiens GN=PCYT1A PE=1 SV=2 - [PCY1A_HUMAN]	59.13	2	18	18	67	0.929	1.024	1.165	1.152	176.95	59.13	30	67	367	41.7	7.25

P98170	E3 ubiquitin-protein ligase XIAP OS=Homo sapiens GN=XIAP PE=1 SV=2 - [XIAP_HUMAN]	11.87	1	5	5	16	0.890	1.220	1.489	1.152	42.11	11.87	8	16	497	56.6	6.65
Q13188	Serine/threonine-protein kinase 3 OS=Homo sapiens GN=STK3 PE=1 SV=2 - [STK3_HUMAN]	16.29	1	5	7	21	0.951	1.105	1.045	1.152	51.73	16.29	9	21	491	56.3	5.24
P34931	Heat shock 70 kDa protein 1-like OS=Homo sapiens GN=HSPA1L PE=1 SV=2 - [HS71L_HUMAN]	31.36	1	1	19	587	0.763	0.761	0.776	1.152	1288.25	31.36	35	587	641	70.3	6.02
O76080	AN1-type zinc finger protein 5 OS=Homo sapiens GN=ZFAND5 PE=1 SV=1 - [ZFAN5_HUMAN]	4.23	1	1	1	1	0.979		0.923	1.152	2.18	4.23	1	1	213	23.1	8.51
P11597	Cholesteryl ester transfer protein OS=Homo sapiens GN=CETP PE=1 SV=2 - [CETP_HUMAN]	3.04	1	1	1	1	1.127	1.065	1.153	1.152	2.54	3.04	1	1	493	54.7	6.09
Q8IYQ7	Threonine synthase-like 1 OS=Homo sapiens GN=THNSL1 PE=1 SV=2 - [THNS1_HUMAN]	3.50	1	3	3	8	0.964	1.475	1.183	1.152	11.06	3.50	5	8	743	83.0	7.12
Q9Y618	Nuclear receptor corepressor 2 OS=Homo sapiens GN=NCOR2 PE=1 SV=2 - [NCOR2_HUMA	2.02	1	4	5	9	0.906	1.137	1.112	1.153	21.87	2.02	8	9	2525	274.6	7.59
P10515	Dihydrolipoyllysine-residue acetyltransferase component of pyruvate dehydrogenase complex, mitochondrial OS=Homo sapiens GN=DLAT PE=1 SV=3 - [ODP2_HUMAN]	24.11	1	12	12	47	1.196	1.094	0.886	1.153	95.30	24.11	21	47	647	69.0	7.84
P11441	Ubiquitin-like protein 4A OS=Homo sapiens GN=UBL4A PE=1 SV=1 - [UBL4A_HUMAN]	33.12	1	6	6	25	1.216	1.278	1.151	1.153	47.09	33.12	7	25	157	17.8	8.66

Q9BUJ2	Heterogeneous nuclear ribonucleoprotein U-like protein 1 OS=Homo sapiens GN=HNRNPUL1 PE=1 SV=2 - [HNRL1_HUMAN	24.42	1	18	18	67	1.142	1.430	1.078	1.153	187.65	24.42	29	67	856	95.7	6.92
Q03013	Glutathione S-transferase Mu 4 OS=Homo sapiens GN=GSTM4 PE=1 SV=3 - [GSTM4_HUMAN]	55.05	1	3	14	114	2.615	0.786	1.095	1.153	313.27	55.05	23	114	218	25.5	5.90
P56945	Breast cancer anti-estrogen resistance protein 1 OS=Homo sapiens GN=BCAR1 PE=1 SV=2 - [BCAR1_HUMAN]	21.15	1	12	13	36	0.969	0.753	1.228	1.154	118.88	21.15	20	36	870	93.3	5.67
Q9NXF1	Testis-expressed sequence 10 protein OS=Homo sapiens GN=TEX10 PE=1 SV=2 - [TEX10_HUMAN	10.44	1	8	9	14	1.019	0.955	0.834	1.154	30.92	10.44	12	14	929	105.6	9.36
Q9P127	Leucine zipper protein 4 OS=Homo sapiens GN=LUZP4 PE=2 SV=1 - [LUZP4_HUMAN]	2.88	1	1	1	1	1.035	0.920	1.558	1.154	3.61	2.88	1	1	313	35.9	9.45
Q8N6H7	ADP-ribosylation factor GTPase-activating protein 2 OS=Homo sapiens GN=ARFGAP2 PE=1 SV=1 - [ARFG2_HUMAN]	23.03	1	11	11	30	0.905	1.070	1.114	1.154	60.69	23.03	17	30	521	56.7	7.99
Q9BY43	Charged multivesicular body protein 4a OS=Homo sapiens GN=CHMP4A PE=1 SV=3 - [CHM4A_HUMAN]	31.98	1	6	7	23	0.838	1.107	1.192	1.154	62.07	31.98	10	23	222	25.1	4.70
Q8NBP0	Tetratricopeptide repeat protein 13 OS=Homo sapiens GN=TTC13 PE=2 SV=3 - [TTC13_HUMAN	6.98	1	6	6	11	1.115	1.093	0.851	1.154	21.39	6.98	8	11	860	96.8	7.01

Q9H6R7	WD repeat-containing protein C2orf44 OS=Homo sapiens GN=C2orf44 PE=1 SV=1 - [CB044_HUMAN]	0.97	1	1	1	3	0.650	0.877	1.160	1.154	6.46	0.97	2	3	721	79.1	6.70
Q13620	Cullin-4B OS=Homo sapiens GN=CUL4B PE=1 SV=4 - [CUL4B_HUMAN]	23.88	1	13	24	69	1.026	1.207	1.019	1.154	116.32	23.88	37	69	913	103.9	7.37
Q9UQ35	Serine/arginine repetitive matrix protein 2 OS=Homo sapiens GN=SRRM2 PE=1 SV=2 - [SRRM2_HUMAN]	12.68	1	26	26	78	0.924	1.162	0.976	1.154	170.71	12.68	40	78	2752	299.4	12.06
Q9Y2X9	Zinc finger protein 281 OS=Homo sapiens GN=ZNF281 PE=1 SV=1 - [ZN281_HUMAN]	4.92	1	2	2	2	1.780	1.338	0.899	1.154	0.00	4.92	2	2	895	96.9	8.48
Q12857	Nuclear factor 1 A-type OS=Homo sapiens GN=NFIA PE=1 SV=2 - [NFIA_HUMAN]	6.29	1	2	3	4	0.626	2.471	1.719	1.154	8.23	6.29	3	4	509	55.9	8.44
Q9UMS4	Pre-mRNA-processing factor 19 OS=Homo sapiens GN=PRPF19 PE=1 SV=1 - [PRP19_HUMAN]	17.86	1	9	9	50	1.151	1.166	0.951	1.154	108.37	17.86	15	50	504	55.1	6.61
Q92623	Tetratricopeptide repeat protein 9A OS=Homo sapiens GN=TTC9 PE=2 SV=3 - [TTC9A_HUMAN	8.11	1	2	2	2	1.827	2.082	2.272	1.154	5.33	8.11	2	2	222	24.4	8.98
P34947	G protein-coupled receptor kinase 5 OS=Homo sapiens GN=GRK5 PE=1 SV=1 - [GRK5_HUMAN]	15.76	1	7	7	10	1.630	0.846	0.615	1.154	17.94	15.76	9	10	590	67.7	8.10
Q14728	Major facilitator superfamily domain-containing protein 10 OS=Homo sapiens GN=MFSD10 PE=2 SV=1 - [MFS10_HUMA	4.40	1	2	2	8	0.963	0.902	0.899	1.154	15.76	4.40	3	8	455	48.3	9.60

Q96A32	Myosin regulatory light chain 2, skeletal muscle isoform OS=Homo sapiens GN=MYLPF PE=2 SV=1 - [MLRS_HUMAN]	13.61	1	2	2	5	1.184	1.204	1.332	1.155	7.96	13.61	3	5	169	19.0	5.01
Q9NPH0	Lysophosphatidic acid phosphatase type 6 OS=Homo sapiens GN=ACP6 PE=1 SV=2 - [PPA6_HUMAN]	3.50	1	1	1	7	0.945	1.618	1.176	1.155	11.41	3.50	2	7	428	48.8	6.47
O95831	Apoptosis-inducing factor 1, mitochondrial OS=Homo sapiens GN=AIFM1 PE=1 SV=1 - [AIFM1_HUMAN	30.51	1	19	19	49	1.024	1.255	0.895	1.155	120.87	30.51	25	49	613	66.9	8.95
Q658P3	Metalloreductase STEAP3 OS=Homo sapiens GN=STEAP3 PE=1 SV=2 - [STEA3_HUMAN]	7.38	1	3	3	6	1.572	0.719	1.316	1.155	23.29	7.38	4	6	488	54.6	8.60
Q02040	A-kinase anchor protein 17A OS=Homo sapiens GN=AKAP17A PE=1 SV=2 - [AK17A_HUMAN]	4.75	1	3	3	10	0.793	0.929	0.974	1.155	20.05	4.75	6	10	695	80.7	9.73
O75152	Zinc finger CCCH domain-containing protein 11A OS=Homo sapiens GN=ZC3H11A PE=1 SV=3 - [ZC11A_HUMAN]	13.95	1	9	10	31	1.077	1.057	1.024	1.155	90.84	13.95	12	31	810	89.1	8.37
P26599	Polypyrimidine tract-binding protein 1 OS=Homo sapiens GN=PTBP1 PE=1 SV=1 - [PTBP1_HUMAN]	32.39	1	8	11	67	1.144	1.043	0.888	1.155	193.97	32.39	16	67	531	57.2	9.17
P62753	40S ribosomal protein S6 OS=Homo sapiens GN=RPS6 PE=1 SV=1 - [RS6_HUMAN]	38.15	1	15	16	124	0.839	1.151	1.034	1.155	338.99	38.15	26	124	249	28.7	10.84

Q9H9A7	RecQ-mediated genome instability protein 1 OS=Homo sapiens GN=RMI1 PE=1 SV=3 - [RMI1_HUMAN]	3.04	1	2	2	2	1.729	0.437	0.478	1.155	2.24	3.04	2	2	625	70.1	4.96
Q17R98	Zinc finger protein 827 OS=Homo sapiens GN=ZNF827 PE=2 SV=1 - [ZN827_HUMAN]	1.94	1	1	1	1	2.172	0.990	1.445	1.155	2.13	1.94	1	1	1081	119.1	6.89
P62269	40S ribosomal protein S18 OS=Homo sapiens GN=RPS18 PE=1 SV=3 - [RS18_HUMAN]	49.34	1	12	12	59	0.865	1.042	0.983	1.155	125.48	49.34	16	59	152	17.7	10.99
P53597	Succinyl-CoA ligase [ADP/GDP-forming] subunit alpha, mitochondrial OS=Homo sapiens GN=SUCLG1 PE=1 SV=4 - [SUCA_HUMAN]	20.81	1	8	8	55	1.107	1.184	1.113	1.155	144.51	20.81	16	55	346	36.2	8.79
P78527	DNA-dependent protein kinase catalytic subunit OS=Homo sapiens GN=PRKDC PE=1 SV=3 - [PRKDC_HUMAN	27.74	1	109	110	417	1.100	1.060	0.824	1.155	983.65	27.74	175	417	4128	468.8	7.12
Q96SI9	Spermatid perinuclear RNA-binding protein OS=Homo sapiens GN=STRBP PE=1 SV=1 - [STRBP_HUMAN]	10.42	1	2	7	42	0.980	0.749	1.071	1.155	102.43	10.42	11	42	672	73.6	8.72
Q8TD22	Sideroflexin-5 OS=Homo sapiens GN=SFXN5 PE=2 SV=1 - [SFXN5_HUMAN]	2.94	1	1	1	1	1.238	1.121	0.766	1.156	0.00	2.94	1	1	340	37.1	9.33
Q8WXD5	Gem-associated protein 6 OS=Homo sapiens GN=GEMIN6 PE=1 SV=1 - [GEMI6_HUMAN]	5.99	1	1	1	4	0.505	1.488	0.943	1.156	13.70	5.99	1	4	167	18.8	5.12
O14684	Prostaglandin E synthase OS=Homo sapiens GN=PTGES PE=1 SV=2 - [PTGES_HUMAN]	7.24	1	2	2	8	2.418	0.475	0.582	1.156	19.73	7.24	4	8	152	17.1	9.50

Q8N3C0	Activating signal cointegrator 1 complex subunit 3 OS=Homo sapiens GN=ASCC3 PE=1 SV=3 - [ASCC3_HUMAN	15.67	1	36	36	73	1.086	0.748	1.042	1.156	157.26	15.67	52	73	2202	251.3	7.09
Q15714	TSC22 domain family protein 1 OS=Homo sapiens GN=TSC22D1 PE=1 SV=3 - [T22D1_HUMAN]	3.82	1	2	5	27	0.714	1.121	1.314	1.156	56.78	3.82	7	27	1073	109.6	5.64
Q8NC96	Adaptin ear-binding coat-associated protein 1 OS=Homo sapiens GN=NECAP1 PE=1 SV=2 - [NECP1_HUMAN]	25.09	1	5	6	12	0.973	0.725	0.866	1.156	30.58	25.09	7	12	275	29.7	6.80
Q6Y1H2	Very-long-chain (3R)-3-hydroxyacyl-CoA dehydratase 2 OS=Homo sapiens GN=PTPLB PE=1 SV=1 - [HACD2_HUMAN]	4.33	1	3	3	5	1.005	1.002	0.763	1.156	14.41	4.33	3	5	254	28.4	9.55
Q5T5U3	Rho GTPase-activating protein 21 OS=Homo sapiens GN=ARHGAP21 PE=1 SV=1 - [RHG21_HUMAN]	3.12	1	4	6	10	1.095	1.105	1.108	1.156	17.89	3.12	7	10	1957	217.2	7.80
P62979	Ubiquitin-40S ribosomal protein S27a OS=Homo sapiens GN=RPS27A PE=1 SV=2 - [RS27A_HUMAN]	70.51	3	4	17	459	2.701	2.096	1.026	1.156	1095.71	70.51	27	459	156	18.0	9.64
Q13093	Platelet-activating factor acetylhydrolase OS=Homo sapiens GN=PLA2G7 PE=1 SV=1 - [PAFA_HUMAN]	4.54	1	1	2	2	1.022	0.822	1.204	1.156	4.31	4.54	2	2	441	50.0	7.56
Q7RTV2	Glutathione S-transferase A5 OS=Homo sapiens GN=GSTA5 PE=1 SV=1 - [GSTA5_HUMAN]	8.11	1	2	2	2	1.123	1.858	1.470	1.156	4.36	8.11	2	2	222	25.7	8.03

Q8IUH3	RNA-binding protein 45 OS=Homo sapiens GN=RBM45 PE=2 SV=1 - [RBM45_HUMAN]	2.31	1	1	1	5	1.726	2.016	0.917	1.157	4.21	2.31	1	5	476	53.5	7.17
Q9HCE1	Putative helicase MOV-10 OS=Homo sapiens GN=MOV10 PE=1 SV=2 - [MOV10_HUMAN]	17.35	1	15	15	41	1.009	1.359	1.012	1.157	97.38	17.35	23	41	1003	113.6	8.82
Q5T440	Putative transferase CAF17, mitochondrial OS=Homo sapiens GN=IBA57 PE=1 SV=1 - [CAF17_HUMAN]	10.11	1	3	3	12	1.193	0.935	1.089	1.157	23.08	10.11	6	12	356	38.1	9.83
A1L4H1	Soluble scavenger receptor cysteine-rich domain-containing protein SSC5D OS=Homo sapiens GN=SSC5D PE=2 SV=3 - [SRCRL_HUMAN	1.53	1	1	1	1	0.939	1.545	0.769	1.157	0.00	1.53	1	1	1573	165.6	6.13
Q12800	Alpha-globin transcription factor CP2 OS=Homo sapiens GN=TFCP2 PE=1 SV=2 - [TFCP2_HUMAN]	12.55	1	3	4	8	0.809	1.185	1.129	1.157	20.78	12.55	6	8	502	57.2	5.80
Q5UIP0	Telomere-associated protein RIF1 OS=Homo sapiens GN=RIF1 PE=1 SV=2 - [RIF1_HUMAN]	2.67	1	5	5	5		1.121	1.354	1.157	7.67	2.67	5	5	2472	274.3	5.52
Q92667	A-kinase anchor protein 1, mitochondrial OS=Homo sapiens GN=AKAP1 PE=1 SV=1 - [AKAP1_HUMAN	3.54	1	2	2	5	0.202	2.166	4.760	1.158	2.54	3.54	2	5	903	97.3	4.94
Q9Y606	tRNA pseudouridine synthase A, mitochondrial OS=Homo sapiens GN=PUS1 PE=1 SV=3 - [TRUA_HUMAN]	18.27	1	6	6	17	0.763	0.872	0.787	1.158	39.27	18.27	10	17	427	47.4	8.41

P62745	Rho-related GTP-binding protein RhoB OS=Homo sapiens GN=RHOB PE=1 SV=1 - [RHOB_HUMAN]	38.27	1	3	4	16	1.038	1.231	1.242	1.158	53.73	38.27	6	16	196	22.1	5.24
Q6NT04	Tigger transposable element-derived protein 7 OS=Homo sapiens GN=TIGD7 PE=2 SV=1 - [TIGD7_HUMAN]	3.28	1	2	2	3	0.980	1.317	0.492	1.158	3.88	3.28	2	3	549	63.2	8.75
Q9Y2G2	Caspase recruitment domain-containing protein 8 OS=Homo sapiens GN=CARD8 PE=1 SV=1 - [CARD8_HUMAN]	3.25	1	1	1	1	0.823	0.472	0.544	1.158	3.34	3.25	1	1	431	48.9	5.22
P46976	Glycogenin-1 OS=Homo sapiens GN=GYG1 PE=1 SV=4 - [GLYG_HUMAN]	17.71	1	7	7	32	1.085	1.022	0.970	1.158	87.24	17.71	10	32	350	39.4	5.53
Q92665	28S ribosomal protein S31, mitochondrial OS=Homo sapiens GN=MRPS31 PE=1 SV=3 - [RT31_HUMAN]	19.24	1	7	7	20	0.929	1.155	1.087	1.158	48.85	19.24	9	20	395	45.3	9.29
A6NKF9	Putative Golgi pH regulator C OS=Homo sapiens GN=GPR89C PE=5 SV=2 - [GPHRC_HUMAN]	3.75	2	1	1	6	0.784	0.726	1.026	1.159	10.65	3.75	2	6	320	36.6	9.32
Q15014	Mortality factor 4-like protein 2 OS=Homo sapiens GN=MORF4L2 PE=1 SV=1 - [MO4L2_HUMAN]	7.29	1	2	2	5	0.804	1.108	1.119	1.159	10.53	7.29	3	5	288	32.3	9.72
Q9Y6F1	Poly [ADP-ribose] polymerase 3 OS=Homo sapiens GN=PARP3 PE=1 SV=3 - [PARP3_HUMAN]	2.06	1	1	1	1	1.126	0.657	0.985	1.159	0.00	2.06	1	1	533	60.0	6.74
Q96T37	Putative RNA-binding protein 15 OS=Homo sapiens GN=RBM15 PE=1 SV=2 - [RBM15_HUMAN]	13.00	1	10	10	17	1.193	0.999	1.199	1.159	45.21	13.00	13	17	977	107.1	10.08

O14519	Cyclin-dependent kinase 2-associated protein 1 OS=Homo sapiens GN=CDK2AP1 PE=1 SV=1 - [CDKA1_HUMAN]	20.00	2	2	2	3	1.461	0.959	0.741	1.159	5.77	20.00	2	3	115	12.4	9.38
Q10471	Polypeptide N-acetylgalactosaminyltransferase 2 OS=Homo sapiens GN=GALNT2 PE=1 SV=1 - [GALT2_HUMAN]	26.97	1	17	19	63	0.854	0.860	0.923	1.159	143.36	26.97	30	63	571	64.7	8.35
Q9H845	Acyl-CoA dehydrogenase family member 9, mitochondrial OS=Homo sapiens GN=ACAD9 PE=1 SV=1 - [ACAD9_HUMAN	26.89	1	16	16	51	1.142	1.019	0.902	1.159	121.53	26.89	25	51	621	68.7	7.96
P46379	Large proline-rich protein BAG6 OS=Homo sapiens GN=BAG6 PE=1 SV=2 - [BAG6_HUMAN]	20.58	1	16	16	59	0.992	1.099	1.124	1.159	163.62	20.58	25	59	1132	119.3	5.60
Q12906	Interleukin enhancer-binding factor 3 OS=Homo sapiens GN=ILF3 PE=1 SV=3 - [ILF3_HUMAN]	35.57	1	26	31	296	1.118	1.112	0.974	1.159	705.95	35.57	55	296	894	95.3	8.76
Q96CW5	Gamma-tubulin complex component 3 OS=Homo sapiens GN=TUBGCP3 PE=1 SV=2 - [GCP3_HUMAN]	9.37	1	7	7	20	1.158	1.169	0.883	1.159	44.69	9.37	12	20	907	103.5	8.12
Q99576	TSC22 domain family protein 3 OS=Homo sapiens GN=TSC22D3 PE=1 SV=2 - [T22D3_HUMAN]	19.40	1	2	3	11	1.056	1.182	1.102	1.160	23.26	19.40	5	11	134	14.8	4.54
O95479	GDH/6PGL endoplasmic bifunctional protein OS=Homo sapiens GN=H6PD PE=1 SV=2 - [G6PE_HUMAN]	22.38	1	17	17	64	0.847	1.007	0.853	1.160	154.63	22.38	29	64	791	88.8	7.30

P52272	Heterogeneous nudear ribonudeoprotein M OS=Homo sapiens GN=HNRNPM PE=1 SV=3 - [HNRPM_HUMA	45.34	1	35	35	254	1.198	1.169	1.007	1.160	647.46	45.34	55	254	730	77.5	8.70
Q9P225	Dynein heavy chain 2, axonemal OS=Homo sapiens GN=DNAH2 PE=2 SV=3 - [DYH2_HUMAN]	0.79	1	1	3	5	2.519	4.218	2.566	1.160	4.12	0.79	3	5	4427	507.4	6.37
Q8NFS9	N-acetyllactosaminide beta-1,6-N-acetylglucosaminyl-transferase, isoform C OS=Homo sapiens GN=GCNT2 PE=2 SV=2 -	4.48	1	1	1	1	0.967	0.777	1.580	1.161	3.16	4.48	1	1	402	46.5	8.16
Q9BTL3	RNMT-activating mini protein OS=Homo sapiens GN=FAM103A1 PE=1 SV=1 - [RAM_HUMAN]	25.42	1	3	3	11	0.925	1.300	1.308	1.161	37.14	25.42	5	11	118	14.4	8.94
Q8IUC6	TIR domain-containing adapter molecule 1 OS=Homo sapiens GN=TICAM1 PE=1 SV=1 - [TCAM1_HUMA	1.26	1	1	1	6	0.674	1.525	1.819	1.161	11.56	1.26	2	6	712	76.4	5.41
P54725	UV excision repair protein RAD23 homolog A OS=Homo sapiens GN=RAD23A PE=1 SV=1 - [RD23A_HUMAN]	23.42	1	6	9	47	1.249	1.110	1.357	1.161	113.93	23.42	16	47	363	39.6	4.58
Q9P2Y4	Zinc finger protein 219 OS=Homo sapiens GN=ZNF219 PE=1 SV=2 - [ZN219_HUMAN]	1.39	1	1	1	1	1.313	1.153	1.137	1.161	0.00	1.39	1	1	722	76.8	9.39
Q96LB3	Intraflagellar transport protein 74 homolog OS=Homo sapiens GN=IFT74 PE=1 SV=1 - [IFT74_HUMAN]	10.50	1	6	6	9	0.828	1.347	1.134	1.161	22.87	10.50	7	9	600	69.2	6.00

Q969X6	Cirhin OS=Homo sapiens GN=CIRH1A PE=1 SV=1 - [CIR1A_HUMAN]	3.06	1	2	2	3	0.747	1.194	0.766	1.161	9.55	3.06	2	3	686	76.8	8.85
P43155	Carnitine O-acetyltransfera se OS=Homo sapiens GN=CRAT PE=1 SV=5 - [CACP_HUMAN]	27.00	1	17	17	46	0.958	1.238	1.020	1.161	105.28	27.00	25	46	626	70.8	8.44
O95171	Sciellin OS=Homo sapiens GN=SCEL PE=1 SV=2 - [SCEL_HUMAN]	1.31	1	1	1	1	1.206	0.814	0.832	1.161	1.88	1.31	1	1	688	77.5	9.38
P51991	Heterogeneous nuclear ribonucleoprot ein A3 OS=Homo sapiens GN=HNRNPA3 PE=1 SV=2 - [ROA3_HUMAN]	45.77	1	20	22	177	1.075	1.057	0.894	1.161	454.75	45.77	33	177	378	39.6	9.01
P00367	Glutamate dehydrogenase 1, mitochondrial OS=Homo sapiens GN=GLUD1 PE=1 SV=2 - [DHE3_HUMAN]	45.88	2	26	27	296	1.241	1.212	1.053	1.162	738.85	45.88	47	296	558	61.4	7.80
Q12873	Chromodomain-helicase-DNA-binding protein 3 OS=Homo sapiens GN=CHD3 PE=1 SV=3 - [CHD3_HUMAN]	4.80	1	3	8	25	0.886	1.755	0.871	1.162	53.25	4.80	15	25	2000	226.4	7.30
O14530	Thioredoxin domain-containing protein 9 OS=Homo sapiens GN=TXNDC9 PE=1 SV=2 - [TXND9_HUMAN]	13.27	1	2	4	9	0.966	0.894	1.092	1.162	15.69	13.27	5	9	226	26.5	5.88
Q9NXG2	THUMP domain-containing protein 1 OS=Homo sapiens GN=THUMPD1 PE=1 SV=2 - [THUM1_HUMA N]	15.01	1	6	6	17	1.111	1.305	0.915	1.162	30.66	15.01	9	17	353	39.3	7.88
P51810	G-protein coupled receptor 143 OS=Homo sapiens GN=GPR143 PE=1 SV=2 - [GP143_HUMAN]	3.22	1	1	1	2	0.754	0.895	0.867	1.162	4.72	3.22	1	2	404	43.8	7.58

Q96RP9	Elongation factor G, mitochondrial OS=Homo sapiens GN=GFM1 PE=1 SV=2 - [EFGM_HUMAN]	17.04	1	13	13	28	0.879	0.812	0.927	1.163	68.36	17.04	18	28	751	83.4	7.01
Q03169	Tumor necrosis factor alpha-induced protein 2 OS=Homo sapiens GN=TNFAIP2 PE=1 SV=2 - [TNAP2_HUMAN	17.74	1	9	9	19	1.064	0.800	0.705	1.163	51.23	17.74	12	19	654	72.6	6.46
Q6V0I7	Protocadherin Fat 4 OS=Homo sapiens GN=FAT4 PE=1 SV=2 - [FAT4_HUMAN]	0.68	1	2	2	5	2.727	0.949	0.941	1.163	7.11	0.68	3	5	4981	542.4	4.94
P30711	Glutathione S-transferase theta-1 OS=Homo sapiens GN=GSTT1 PE=1 SV=4 - [GSTT1_HUMAN	42.50	1	9	11	30	1.349	1.127	1.046	1.163	61.55	42.50	16	30	240	27.3	7.49
O00635	E3 ubiquitin-protein ligase TRIM38 OS=Homo sapiens GN=TRIM38 PE=1 SV=1 - [TRI38_HUMAN]	6.67	1	3	3	8	1.407	1.408	1.218	1.163	17.09	6.67	4	8	465	53.4	7.02
P82933	28S ribosomal protein S9, mitochondrial OS=Homo sapiens GN=MRPS9 PE=1 SV=2 - [RT09_HUMAN]	22.73	1	7	7	22	0.797	1.214	0.957	1.163	89.37	22.73	9	22	396	45.8	9.51
O14786	Neuropilin-1 OS=Homo sapiens GN=NRP1 PE=1 SV=3 - [NRP1_HUMAN]	3.14	1	2	2	3	0.696	1.480	2.069	1.164	2.20	3.14	2	3	923	103.1	5.88
Q9H7V2	Synapse differentiation-inducing gene protein 1 OS=Homo sapiens GN=SYNDIG1 PE=2 SV=1 - [SYNG1_HUMAN]	8.91	1	1	2	4	1.198	0.604	0.733	1.164	1.61	8.91	2	4	258	28.5	4.88
O94927	HAUS augmin-like complex subunit 5 OS=Homo sapiens GN=HAUS5 PE=1 SV=2 - [HAUS5_HUMAN]	1.74	1	1	1	1	0.720	1.043	0.880	1.164	2.80	1.74	1	1	633	71.6	8.51

P07954	Fumarate hydratase, mitochondrial OS=Homo sapiens GN=FH PE=1 SV=3 - [FUMH_HUMAN]	50.20	3	24	25	125	0.945	1.334	1.059	1.164	337.69	50.20	37	125	510	54.6	8.76
O15439	Multidrug resistance-associated protein 4 OS=Homo sapiens GN=ABCC4 PE=1 SV=3 - [MRP4_HUMAN]	19.77	1	26	26	77	2.639	0.438	0.824	1.164	201.44	19.77	37	77	1325	149.4	8.19
Q9UBX3	Mitochondrial dicarboxylate carrier OS=Homo sapiens GN=SLC25A10 PE=1 SV=2 - [DIC_HUMAN]	6.62	1	2	2	5	1.029	1.094	1.230	1.164	13.21	6.62	3	5	287	31.3	9.54
Q9Y2I8	WD repeat-containing protein 37 OS=Homo sapiens GN=WDR37 PE=1 SV=2 - [WDR37_HUMAN]	4.45	1	2	2	3	1.160	1.008	1.055	1.164	3.57	4.45	3	3	494	54.6	7.23
Q9NW13	RNA-binding protein 28 OS=Homo sapiens GN=RBM28 PE=1 SV=3 - [RBM28_HUMAN]	8.56	1	6	7	13	0.999	0.951	0.915	1.165	29.64	8.56	9	13	759	85.7	9.22
Q9BQC6	Ribosomal protein 63, mitochondrial OS=Homo sapiens GN=MRPL57 PE=1 SV=1 - [RT63_HUMAN]	6.86	1	1	1	9	0.996	1.114	0.995	1.165	19.42	6.86	2	9	102	12.3	11.44
Q14320	Protein FAM50A OS=Homo sapiens GN=FAM50A PE=1 SV=2 - [FA50A_HUMAN]	25.37	2	6	8	26	1.203	1.182	1.448	1.165	51.43	25.37	13	26	339	40.2	6.83
P38919	Eukaryotic initiation factor 4A-III OS=Homo sapiens GN=EIF4A3 PE=1 SV=4 - [IF4A3_HUMAN]	34.55	1	13	17	76	1.153	1.201	0.927	1.165	194.94	34.55	28	76	411	46.8	6.73
Q6P3W7	SCY1-like protein 2 OS=Homo sapiens GN=SCYL2 PE=1 SV=1 - [SCYL2_HUMAN]	10.76	1	8	8	35	1.749	1.054	1.002	1.165	65.61	10.76	14	35	929	103.6	8.22

P19367	Hexokinase-1 OS=Homo sapiens GN=HK1 PE=1 SV=3 - [HXK1_HUMAN]	42.31	1	34	38	175	1.233	1.218	1.157	1.165	431.55	42.31	62	175	917	102.4	6.80
Q96TA2	ATP-dependent zinc metalloprotease YME1L1 OS=Homo sapiens GN=YME1L1 PE=1 SV=2 - [YMEL1_HUMAN]	14.88	1	10	10	20	0.884	0.867	0.836	1.165	35.33	14.88	15	20	773	86.4	8.76
O95298	NADH dehydrogenase [ubiquinone] 1 subunit C2 OS=Homo sapiens GN=NDUFC2 PE=1 SV=1 - [NDUC2_HUMAN]	30.25	2	4	4	19	0.770	0.982	0.753	1.165	45.45	30.25	6	19	119	14.2	8.98
Q9BVC4	Target of rapamycin complex subunit LST8 OS=Homo sapiens GN=MLST8 PE=1 SV=1 - [LST8_HUMAN]	9.20	1	3	3	7	0.908	0.971	1.271	1.165	16.81	9.20	5	7	326	35.9	5.86
Q9Y2L1	Exosome complex exonuclease RRP44 OS=Homo sapiens GN=DIS3 PE=1 SV=2 - [RRP44_HUMAN]	10.65	1	10	10	22	1.084	1.267	1.039	1.166	46.01	10.65	16	22	958	108.9	7.14
Q96DI7	U5 small nuclear ribonucleoprotein 40 kDa protein OS=Homo sapiens GN=SNRNP40 PE=1 SV=1 - [SNR40_HUMAN	16.53	1	6	6	8	1.001	1.375	1.081	1.166	16.80	16.53	8	8	357	39.3	8.10
Q96K19	E3 ubiquitin-protein ligase RNF170 OS=Homo sapiens GN=RNF170 PE=1 SV=2 - [RN170_HUMAN]	12.79	1	3	3	16	1.109	0.852	0.590	1.166	38.10	12.79	5	16	258	29.8	5.58
Q9H089	Large subunit GTPase 1 homolog OS=Homo sapiens GN=LSG1 PE=1 SV=2 - [LSG1_HUMAN]	10.33	1	7	7	12	1.278	1.046	0.973	1.166	22.03	10.33	10	12	658	75.2	6.38

Q8WX93	Palladin OS=Homo sapiens GN=PALLD PE=1 SV=3 - [PALLD_HUMAN]	28.05	1	27	28	368	1.298	1.099	1.485	1.167	943.54	28.05	48	368	1383	150.5	7.09
Q6DKK2	Tetratricopeptide repeat protein 19, mitochondrial OS=Homo sapiens GN=TTC19 PE=1 SV=4 - [TTC19_HUMAN]	7.63	1	3	3	10	1.221	0.853	0.502	1.167	23.68	7.63	5	10	380	42.4	5.77
Q9Y2G4	Ankyrin repeat domain-containing protein 6 OS=Homo sapiens GN=ANKRD6 PE=1 SV=3 - [ANKR6_HUMAN]	1.79	1	1	1	1	0.809	1.040	1.148	1.167	0.00	1.79	1	1	727	79.9	9.32
P61978	Heterogeneous nuclear ribonucleoprotein K OS=Homo sapiens GN=HNRNPK PE=1 SV=1 - [HNRPK_HUMAN	63.07	1	26	26	271	1.081	1.281	1.039	1.168	612.90	63.07	44	271	463	50.9	5.54
Q13952	Nuclear transcription factor Y subunit gamma OS=Homo sapiens GN=NFYC PE=1 SV=3 - [NFYC_HUMAN]	4.59	1	2	2	4	1.001	1.409	0.853	1.168	8.94	4.59	4	4	458	50.3	6.10
Q8WVC0	RNA polymerase-associated protein LEO1 OS=Homo sapiens GN=LEO1 PE=1 SV=1 - [LEO1_HUMAN]	1.35	1	1	1	1	1.406	1.023	1.081	1.168	0.00	1.35	1	1	666	75.4	4.51
Q96BX8	MOB kinase activator 3A OS=Homo sapiens GN=MOB3A PE=1 SV=1 - [MOB3A_HUMAN]	7.83	1	2	2	3	0.611	1.340	1.379	1.168	2.11	7.83	3	3	217	25.4	8.63
P48507	Glutamate--cysteine ligase regulatory subunit OS=Homo sapiens GN=GCLM PE=1 SV=1 - [GSH0_HUMAN]	32.12	1	8	8	58	1.093	0.412	0.935	1.168	115.39	32.12	15	58	274	30.7	6.02

P01889	HLA class I histocompatibility antigen, B-7 alpha chain OS=Homo sapiens GN=HLA-B PE=1 SV=3 - [1B07_HUMAN]	38.95	2	2	13	90	44.062		2.387	1.169	242.11	38.95	21	90	362	40.4	5.85
O00257	E3 SUMO-protein ligase CBX4 OS=Homo sapiens GN=CBX4 PE=1 SV=3 - [CBX4_HUMAN]	1.61	1	1	1	2	1.479	1.661	1.019	1.169	2.60	1.61	2	2	560	61.3	9.36
Q96A35	39S ribosomal protein L24, mitochondrial OS=Homo sapiens GN=MRPL24 PE=1 SV=1 - [RM24_HUMAN]	33.33	1	6	6	16	0.977	1.228	0.874	1.169	38.40	33.33	9	16	216	24.9	9.29
Q96RQ1	Endoplasmic reticulum-Golgi intermediate compartment protein 2 OS=Homo sapiens GN=ERGIC2 PE=1 SV=2 - [ERGI2_HUMAN]	13.79	1	5	5	10	0.909	0.808	0.729	1.169	16.34	13.79	7	10	377	42.5	6.77
Q8WVC6	Dephospho-CoA kinase domain-containing protein OS=Homo sapiens GN=DCAKD PE=1 SV=1 - [DCAKD_HUMAN]	17.32	1	3	3	5	0.850	1.056	0.811	1.169	12.14	17.32	5	5	231	26.5	9.58
Q92614	Unconventional myosin-XVIIIa OS=Homo sapiens GN=MYO18A PE=1 SV=3 - [MY18A_HUMAN]	17.04	4	30	34	101	1.475	1.288	1.116	1.170	221.40	17.04	50	101	2054	233.0	6.30
O60508	Pre-mRNA-processing factor 17 OS=Homo sapiens GN=CDC40 PE=1 SV=1 - [PRP17_HUMAN]	1.73	1	1	1	1	1.206	1.456	1.159	1.170	2.85	1.73	1	1	579	65.5	7.06
Q99972	Myocilin OS=Homo sapiens GN=MYOC PE=1 SV=2 - [MYOC_HUMAN]	1.98	1	1	1	1	1.077	0.785	1.313	1.170	0.00	1.98	1	1	504	56.9	5.54

Q5TC84	Opioid growth factor receptor-like protein 1 OS=Homo sapiens GN=OGFRL1 PE=2 SV=1 - [OGRL1_HUMAN]	9.98	1	4	4	6	1.377	0.627	1.198	1.170	11.32	9.98	6	6	451	51.2	6.13
Q13596	Sorting nexin-1 OS=Homo sapiens GN=SNX1 PE=1 SV=3 - [SNX1_HUMAN]	44.06	1	21	23	83	1.119	0.918	0.955	1.170	239.09	44.06	36	83	522	59.0	5.15
Q8WW12	PEST proteolytic signal-containing nuclear protein OS=Homo sapiens GN=PCNP PE=1 SV=2 - [PCNP_HUMAN]	40.45	1	6	6	35	1.260	1.480	0.870	1.170	87.52	40.45	12	35	178	18.9	7.49
P49770	Translation initiation factor eIF-2B subunit beta OS=Homo sapiens GN=EIF2B2 PE=1 SV=3 - [EI2BB_HUMAN	31.62	1	10	10	22	1.070	0.939	1.006	1.170	50.49	31.62	15	22	351	39.0	6.16
O00629	Importin subunit alpha-3 OS=Homo sapiens GN=KPNA4 PE=1 SV=1 - [IMA3_HUMAN]	18.62	1	4	9	48	0.927	1.163	1.359	1.170	100.38	18.62	14	48	521	57.9	4.96
Q7Z478	ATP-dependent RNA helicase DHX29 OS=Homo sapiens GN=DHX29 PE=1 SV=2 - [DHX29_HUMAN	14.32	1	18	18	47	0.937	0.998	1.060	1.170	126.33	14.32	23	47	1369	155.1	8.09
P16278	Beta-galactosidase OS=Homo sapiens GN=GLB1 PE=1 SV=2 - [BGAL_HUMAN]	26.00	1	12	13	67	0.909	0.943	0.976	1.170	172.37	26.00	21	67	677	76.0	6.57
Q9Y676	28S ribosomal protein S18b, mitochondrial OS=Homo sapiens GN=MRPS18B PE=1 SV=1 - [RT18B_HUMAN]	33.72	1	5	5	39	0.898	1.078	0.891	1.171	140.13	33.72	8	39	258	29.4	9.38
P42166	Lamina-associated polypeptide 2, isoform alpha OS=Homo sapiens GN=TMPO PE=1 SV=2 - [LAP2A_HUMAN]	22.05	3	6	14	71	0.954	0.993	0.755	1.171	143.22	22.05	22	71	694	75.4	7.66

Q9NYI0	PH and SEC7 domain-containing protein 3 OS=Homo sapiens GN=PSD3 PE=1 SV=2 - [PSD3_HUMAN]	8.30	1	8	8	15	2.141	0.698	1.276	1.171	34.66	8.30	10	15	1048	116.0	5.99
P09651	Heterogeneous nudear ribonudeoprotein A1 OS=Homo sapiens GN=HNRNPA1 PE=1 SV=5 - [ROA1_HUMAN]	50.00	2	19	23	499	1.040	1.197	0.892	1.171	1315.40	50.00	41	499	372	38.7	9.13
Q9UHQ9	NADH-cytochrome b5 reductase 1 OS=Homo sapiens GN=CYB5R1 PE=1 SV=1 - [NB5R1_HUMAN]	35.08	1	13	13	67	1.149	0.653	0.789	1.171	134.19	35.08	24	67	305	34.1	9.38
P82930	28S ribosomal protein S34, mitochondrial OS=Homo sapiens GN=MRPS34 PE=1 SV=2 - [RT34_HUMAN]	25.69	1	4	5	17	0.811	1.404	1.100	1.171	53.48	25.69	7	17	218	25.6	9.98
Q5H9F3	BCL-6 corepressor-like protein 1 OS=Homo sapiens GN=BCORL1 PE=1 SV=1 - [BCORL_HUMAN]	1.17	1	2	2	2	1.165	1.111	1.206	1.171	4.41	1.17	2	2	1711	182.4	7.24
Q9UKN7	Unconventional myosin-XV OS=Homo sapiens GN=MYO15A PE=1 SV=2 - [MYO15_HUMAN]	0.68	1	1	2	23	1.081	0.984	1.438	1.171	23.26	0.68	3	23	3530	395.0	9.17
Q8NDV7	Trinudeotide repeat-containing gene 6A protein OS=Homo sapiens GN=TNRC6A PE=1 SV=2 - [TNR6A_HUMAN	2.04	2	3	3	8	0.875	0.987	1.155	1.171	6.85	2.04	5	8	1962	210.2	7.01
Q9UBB6	Neurochondrin OS=Homo sapiens GN=NCDN PE=1 SV=1 - [NCDN_HUMAN]	3.70	1	3	3	7	0.886	0.921	1.052	1.171	19.08	3.70	5	7	729	78.8	5.48
Q9NWB6	Arginine and glutamate-rich protein 1 OS=Homo sapiens GN=ARGLU1 PE=1 SV=1 - [ARGL1_HUMAN]	16.85	1	6	7	23	1.040	1.216	1.022	1.171	47.01	16.85	11	23	273	33.2	10.35

P05106	Integrin beta-3 OS=Homo sapiens GN=ITGB3 PE=1 SV=2 - [ITB3_HUMAN]	17.39	1	13	14	65	1.682	0.614	0.689	1.172	173.14	17.39	24	65	788	87.0	5.24
P49459	Ubiquitin-conjugating enzyme E2 A OS=Homo sapiens GN=UBE2A PE=1 SV=2 - [UBE2A_HUMAN]	17.76	1	2	2	11	0.990	1.001	1.116	1.172	24.41	17.76	4	11	152	17.3	5.15
Q9C0G6	Dynein heavy chain 6, axonemal OS=Homo sapiens GN=DNAH6 PE=1 SV=3 - [DYH6_HUMAN]	1.18	1	4	5	8	1.170	0.380	0.718	1.172	11.47	1.18	5	8	4158	475.7	6.00
Q96GA3	Protein LTV1 homolog OS=Homo sapiens GN=LTV1 PE=1 SV=1 - [LTV1_HUMAN]	2.11	1	1	1	2	0.731	1.434	0.718	1.172	2.53	2.11	2	2	475	54.8	4.91
P51572	B-cell receptor-associated protein 31 OS=Homo sapiens GN=BCAP31 PE=1 SV=3 - [BAP31_HUMAN]	47.15	1	17	17	202	1.180	1.083	0.988	1.172	444.58	47.15	28	202	246	28.0	8.44
Q4G0J3	La-related protein 7 OS=Homo sapiens GN=LARP7 PE=1 SV=1 - [LARP7_HUMAN]	19.59	1	11	11	41	1.111	1.377	1.138	1.172	99.48	19.59	19	41	582	66.9	9.55
Q8WZ82	Ovarian cancer-associated gene 2 protein OS=Homo sapiens GN=OVCA2 PE=1 SV=1 - [OVCA2_HUMAN]	15.42	1	2	2	11	1.130	1.035	1.055	1.172	35.35	15.42	3	11	227	24.4	6.89
Q9UK10	Zinc finger protein 225 OS=Homo sapiens GN=ZNF225 PE=2 SV=2 - [ZN225_HUMAN]	3.26	11	1	2	3	1.049	2.252	5.682	1.173	2.55	3.26	3	3	706	82.4	8.91
O00567	Nucleolar protein 56 OS=Homo sapiens GN=NOP56 PE=1 SV=4 - [NOP56_HUMAN]	45.29	1	21	21	71	0.915	1.015	0.940	1.173	190.41	45.29	36	71	594	66.0	9.19

Q99717	Mothers against decapentaplegic homolog 5 OS=Homo sapiens GN=SMAD5 PE=1 SV=1 - [SMAD5_HUMA	7.10	2	3	3	7	1.337	1.676	1.124	1.173	9.65	7.10	4	7	465	52.2	7.71
Q8WY36	HMG box transcription factor BBX OS=Homo sapiens GN=BBX PE=1 SV=1 - [BBX_HUMAN]	4.46	3	3	4	7	0.939	1.340	1.048	1.173	11.14	4.46	5	7	941	105.1	8.79
Q96SB3	Neurabin-2 OS=Homo sapiens GN=PPP1R9B PE=1 SV=2 - [NEB2_HUMAN]	18.65	1	13	14	50	0.988	1.016	0.970	1.173	114.32	18.65	24	50	815	89.1	4.97
O75575	DNA-directed RNA polymerase III subunit RPC9 OS=Homo sapiens GN=CRCP PE=1 SV=1 - [RPC9_HUMAN]	4.73	1	1	1	3	0.816	1.265	1.110	1.173	3.47	4.73	2	3	148	16.9	5.39
Q9NYF8	Bcl-2-associated transcription factor 1 OS=Homo sapiens GN=BCLAF1 PE=1 SV=2 - [BCLF1_HUMAN	23.70	1	25	25	80	1.088	1.399	0.984	1.173	180.25	23.70	36	80	920	106.1	9.98
P61289	Proteasome activator complex subunit 3 OS=Homo sapiens GN=PSME3 PE=1 SV=1 - [PSME3_HUMAN]	42.13	1	10	10	56	0.916	0.815	0.822	1.174	151.75	42.13	17	56	254	29.5	5.95
Q7L273	BTB/POZ domain-containing protein KCTD9 OS=Homo sapiens GN=KCTD9 PE=2 SV=1 - [KCTD9_HUMAN]	5.14	1	2	2	3	1.103	1.027	1.061	1.174	6.22	5.14	3	3	389	42.5	6.37
O75970	Multiple PDZ domain protein OS=Homo sapiens GN=MPDZ PE=1 SV=2 - [MPDZ_HUMAN]	5.31	1	9	9	31	1.231	1.118	1.163	1.174	51.91	5.31	14	31	2070	221.5	5.06
P30260	Cell division cycle protein 27 homolog OS=Homo sapiens GN=CDC27 PE=1 SV=2 - [CDC27_HUMAN]	10.32	1	7	7	14	1.078	0.835	0.839	1.174	24.52	10.32	9	14	824	91.8	7.02

Q9P270	SLAIN motif-containing protein 2 OS=Homo sapiens GN=SLAIN2 PE=1 SV=2 - [SLAI2_HUMAN]	15.66	1	9	9	18	1.149	1.065	1.108	1.174	37.76	15.66	15	18	581	62.5	9.45
Q9HCG8	Pre-mRNA-splicing factor CWC22 homolog OS=Homo sapiens GN=CWC22 PE=1 SV=3 - [CWC22_HUMAN]	4.52	1	3	3	6	1.009	1.385	0.966	1.174	8.90	4.52	5	6	908	105.4	7.03
Q5Y7A7	HLA class II histocompatibility antigen, DRB1-13 beta chain OS=Homo sapiens GN=HLA-DRB1 PE=1 SV=1 - [2B1D_HUMAN]	2.63	1	1	1	1	0.880	0.634	0.658	1.174	2.14	2.63	1	1	266	30.0	6.99
Q9BX59	Tapasin-related protein OS=Homo sapiens GN=TAPBPL PE=1 SV=2 - [TPSNR_HUMAN]	5.77	1	2	2	18	1.390	0.832	0.722	1.174	42.49	5.77	4	18	468	50.2	5.26
O75953	DnaJ homolog subfamily B member 5 OS=Homo sapiens GN=DNAJB5 PE=1 SV=1 - [DNJB5_HUMAN]	2.59	1	1	1	2	0.838	1.572	2.033	1.174	7.60	2.59	2	2	348	39.1	9.04
P15923	Transcription factor E2-alpha OS=Homo sapiens GN=TCF3 PE=1 SV=1 - [TFE2_HUMAN]	1.22	1	1	1	1	1.082	1.023	1.080	1.174	0.00	1.22	1	1	654	67.6	6.47
P15289	Arylsulfatase A OS=Homo sapiens GN=ARSA PE=1 SV=3 - [ARSA_HUMAN]	17.55	1	7	7	30	0.771	1.086	1.176	1.174	91.89	17.55	12	30	507	53.6	6.07
Q8IXM2	Chromatin complexes subunit BAP18 OS=Homo sapiens GN=BAP18 PE=1 SV=1 - [BAP18_HUMAN]	26.74	1	3	3	12	0.896	1.438	1.138	1.174	30.03	26.74	5	12	172	17.9	7.33
Q9NXH8	Torsin-4A OS=Homo sapiens GN=TOR4A PE=1 SV=2 - [TOR4A_HUMAN]	21.99	1	7	8	13	1.164	0.897	0.744	1.174	35.77	21.99	12	13	423	46.9	9.94

Q8WWY3	U4/U6 small nuclear ribonucleoprotein Prp31 OS=Homo sapiens GN=PRPF31 PE=1 SV=2 - [PRP31_HUMAN]	14.83	1	7	8	22	1.235	1.267	0.910	1.175	51.18	14.83	10	22	499	55.4	5.78
P83881	60S ribosomal protein L36a OS=Homo sapiens GN=RPL36A PE=1 SV=2 - [RL36A_HUMAN]	40.57	1	2	8	40	0.872	1.410	0.936	1.175	101.72	40.57	14	40	106	12.4	10.58
Q9NZQ3	NCK-interacting protein with SH3 domain OS=Homo sapiens GN=NCKIPSD PE=1 SV=1 - [SPN90_HUMAN]	11.63	1	7	7	18	1.280	0.849	1.066	1.175	44.03	11.63	10	18	722	78.9	6.38
O14920	Inhibitor of nuclear factor kappa-B kinase subunit beta OS=Homo sapiens GN=IKBKB PE=1 SV=1 - [IKKB_HUMAN]	25.13	1	14	14	45	1.278	1.307	1.358	1.175	114.35	25.13	21	45	756	86.5	5.78
Q8IYL3	UPF0688 protein C1orf174 OS=Homo sapiens GN=C1orf174 PE=1 SV=2 - [CA174_HUMAN]	8.64	1	2	2	3	1.204	1.768	0.924	1.175	5.94	8.64	3	3	243	26.0	6.90
O76021	Ribosomal L1 domain-containing protein 1 OS=Homo sapiens GN=RSL1D1 PE=1 SV=3 - [RL1D1_HUMAN]	33.47	1	23	24	65	1.097	1.008	0.836	1.175	157.95	33.47	32	65	490	54.9	10.13
P13861	cAMP-dependent protein kinase type II-alpha regulatory subunit OS=Homo sapiens GN=PRKAR2A PE=1 SV=2 - [KAP2_HUMAN]	58.17	1	16	19	76	1.038	1.113	1.002	1.175	193.89	58.17	33	76	404	45.5	5.07
O14936	Peripheral plasma membrane protein CASK OS=Homo sapiens GN=CASK PE=1 SV=3 - [CSKP_HUMAN]	15.23	1	13	13	34	1.867	0.987	0.980	1.176	71.40	15.23	21	34	926	105.1	6.43

Q16718	NADH dehydrogenase [ubiquinone] 1 alpha subcomplex subunit 5 OS=Homo sapiens GN=NDUFA5 PE=1 SV=3 - [NDUA5_HUMAN]	66.38	1	5	5	26	0.608	1.070	0.817	1.176	69.64	66.38	9	26	116	13.5	5.99
Q9UDT6	CAP-Gly domain-containing linker protein 2 OS=Homo sapiens GN=CLIP2 PE=1 SV=1 - [CLIP2_HUMAN]	35.66	3	35	39	127	1.056	0.990	1.085	1.176	300.55	35.66	59	127	1046	115.8	6.73
Q15906	Vacuolar protein sorting-associated protein 72 homolog OS=Homo sapiens GN=VPS72 PE=1 SV=1 - [VPS72_HUMAN]	9.89	1	3	3	7	1.498	0.757	1.296	1.176	17.38	9.89	4	7	364	40.6	6.48
Q9HB07	UPF0160 protein MYG1, mitochondrial OS=Homo sapiens GN=C12orf10 PE=1 SV=2 - [MYG1_HUMAN]	23.40	1	8	8	21	1.181	1.342	1.062	1.176	52.99	23.40	13	21	376	42.4	6.67
P83369	U7 snRNA-associated Sm-like protein LSm11 OS=Homo sapiens GN=LSM11 PE=1 SV=2 - [LSM11_HUMAN]	4.44	1	1	1	1	1.428	0.923	0.870	1.176	0.00	4.44	1	1	360	39.5	10.99
Q96GQ7	Probable ATP-dependent RNA helicase DDX27 OS=Homo sapiens GN=DDX27 PE=1 SV=2 - [DDX27_HUMAN	14.20	1	12	12	29	0.974	1.096	0.792	1.177	54.49	14.20	16	29	796	89.8	9.28
P49189	4-trimethylamino butyraldehyde dehydrogenase OS=Homo sapiens GN=ALDH9A1 PE=1 SV=3 - [AL9A1_HUMAN]	45.75	1	20	20	111	1.104	1.020	0.915	1.177	211.26	45.75	30	111	494	53.8	5.87
Q16762	Thiosulfate sulfurtransferase OS=Homo sapiens GN=TST PE=1 SV=4 - [THTR_HUMAN]	32.66	1	8	9	29	1.278	1.300	1.136	1.177	65.71	32.66	15	29	297	33.4	7.25

Q8TCB0	Interferon-induced protein 44 OS=Homo sapiens GN=IFI44 PE=2 SV=2 - [IFI44_HUMAN]	2.70	1	1	2	6	0.808	0.875	0.888	1.177	14.26	2.70	2	6	444	50.5	6.89
Q9UKM9	RNA-binding protein Raly OS=Homo sapiens GN=RALY PE=1 SV=1 - [RALY_HUMAN]	32.68	1	12	12	41	1.386	1.294	0.961	1.177	104.17	32.68	21	41	306	32.4	9.17
Q96FZ2	Embryonic stem cell-specific 5-hydroxymethylcytosine-binding protein OS=Homo sapiens GN=HMCES PE=1 SV=1 - [HMCES_HUMAN]	8.76	1	2	2	3	1.095	1.094	1.097	1.177	9.02	8.76	3	3	354	40.5	8.15
Q86V85	Integral membrane protein GPR180 OS=Homo sapiens GN=GPR180 PE=2 SV=1 - [GP180_HUMAN]	3.41	1	1	1	2	1.076	0.845	0.603	1.177	5.87	3.41	2	2	440	49.4	7.39
Q9NX14	NADH dehydrogenase [ubiquinone] 1 beta subcomplex subunit 11, mitochondrial OS=Homo sapiens GN=NDUFB11 PE=1 SV=1 - [NDUBB_HUMAN]	18.30	1	2	2	5	1.079	0.450	0.262	1.178	17.60	18.30	3	5	153	17.3	5.22
Q8NEZ5	F-box only protein 22 OS=Homo sapiens GN=FBXO22 PE=1 SV=1 - [FBX22_HUMAN]	7.20	1	3	3	9	1.213	0.702	1.016	1.178	21.90	7.20	5	9	403	44.5	7.03
Q9ULK4	Mediator of RNA polymerase II transcription subunit 23 OS=Homo sapiens GN=MED23 PE=1 SV=2 - [MED23_HUMAN]	4.90	1	5	5	7	1.191	1.118	1.126	1.178	7.57	4.90	5	7	1368	156.4	7.40
P11142	Heat shock cognate 71 kDa protein OS=Homo sapiens GN=HSPA8 PE=1 SV=1 - [HSP7C_HUMAN]	69.04	1	32	51	1967	1.033	0.994	1.117	1.178	4797.12	69.04	91	1967	646	70.9	5.52

Q9BU76	Multiple myeloma tumor-associated protein 2 OS=Homo sapiens GN=MMTAG2 PE=1 SV=1 - [MMTA2_HUMAN]	3.04	1	1	1	2	1.002	1.239	1.098	1.178	0.00	3.04	2	2	263	29.4	10.02
P12956	X-ray repair cross-complementing protein 6 OS=Homo sapiens GN=XRCC6 PE=1 SV=2 - [XRCC6_HUMAN]	50.25	1	30	31	226	1.149	1.164	0.902	1.178	586.73	50.25	51	226	609	69.8	6.64
Q6L8Q7	2',5'-phosphodiesterase 12 OS=Homo sapiens GN=PDE12 PE=1 SV=2 - [PDE12_HUMAN]	16.58	1	8	9	27	1.007	1.119	1.292	1.178	53.12	16.58	15	27	609	67.3	6.57
P04222	HLA class I histocompatibility antigen, Cw-3 alpha chain OS=Homo sapiens GN=HLA-C PE=1 SV=2 - [1C03_HUMAN]	35.79	1	2	10	90	2.390	0.556	0.826	1.178	223.61	35.79	17	90	366	40.8	6.39
O60870	DNA/RNA-binding protein KIN17 OS=Homo sapiens GN=KIN PE=1 SV=2 - [KIN17_HUMAN]	3.56	1	1	2	2	0.836	0.973	0.910	1.178	3.92	3.56	2	2	393	45.3	8.95
Q9UPQ9	Trinucleotide repeat-containing gene 6B protein OS=Homo sapiens GN=TNRC6B PE=1 SV=4 - [TNR6B_HUMAN	2.56	1	4	4	9	0.991	1.077	0.799	1.178	15.96	2.56	6	9	1833	193.9	6.76
Q2T9J0	Peroxisomal leader peptide-processing protease OS=Homo sapiens GN=TYSND1 PE=1 SV=3 - [TYSD1_HUMAN]	3.18	1	1	1	2	1.074	1.079	1.143	1.178	6.78	3.18	2	2	566	59.3	6.20
P62136	Serine/threonine-protein phosphatase PP1-alpha catalytic subunit OS=Homo sapiens GN=PPP1CA PE=1 SV=1 - [PP1A_HUMAN]	41.52	1	2	12	62	0.978	1.155	0.979	1.178	173.71	41.52	20	62	330	37.5	6.33

Q16795	NADH dehydrogenase [ubiquinone] 1 alpha subcomplex subunit 9, mitochondrial OS=Homo sapiens GN=NDUFA9 PE=1 SV=2 - [NDUA9_HUMAN]	26.53	1	8	10	23	0.661	1.030	0.777	1.178	61.22	26.53	14	23	377	42.5	9.80
Q13451	Peptidyl-prolyl cis-trans isomerase FKBP5 OS=Homo sapiens GN=FKBP5 PE=1 SV=2 - [FKBP5_HUMAN]	21.01	1	8	9	29	0.650	1.139	1.087	1.178	78.38	21.01	12	29	457	51.2	5.90
Q8N5M4	Tetratricopeptide repeat protein 9C OS=Homo sapiens GN=TTC9C PE=1 SV=1 - [TTC9C_HUMAN	17.54	1	3	3	11	1.153	1.476	1.356	1.178	20.57	17.54	5	11	171	20.0	8.92
Q6PJI9	WD repeat-containing protein 59 OS=Homo sapiens GN=WDR59 PE=1 SV=2 - [WDR59_HUMAN]	4.83	1	3	4	5	1.114	1.551	1.331	1.178	8.73	4.83	4	5	974	109.7	7.91
Q17RY0	Cytoplasmic polyadenylation element-binding protein 4 OS=Homo sapiens GN=CPEB4 PE=1 SV=1 - [CPEB4_HUMAN]	4.25	1	2	2	5	1.225	0.742	1.039	1.179	7.54	4.25	3	5	729	80.1	7.18
P62917	60S ribosomal protein L8 OS=Homo sapiens GN=RPL8 PE=1 SV=2 - [RL8_HUMAN]	44.36	1	10	11	60	1.299	1.360	1.328	1.179	146.80	44.36	17	60	257	28.0	11.03
Q9ULI1	NACHT and WD repeat domain-containing protein 2 OS=Homo sapiens GN=NWD2 PE=2 SV=3 - [NWD2_HUMAN]	0.86	1	1	1	2	0.656	0.594	2.038	1.179	5.35	0.86	1	2	1742	197.3	6.24
P16435	NADPH--cytochrome P450 reductase OS=Homo sapiens GN=POR PE=1 SV=2 - [NCPR_HUMAN]	30.13	1	16	16	43	1.057	0.755	0.897	1.179	126.74	30.13	25	43	677	76.6	5.58

Q5JTZ9	Alanine--tRNA ligase, mitochondrial OS=Homo sapiens GN=AARS2 PE=1 SV=1 - [SYAM_HUMAN]	10.05	2	9	9	39	1.021	1.304	1.101	1.179	70.18	10.05	17	39	985	107.3	6.27
Q9NZ32	Actin-related protein 10 OS=Homo sapiens GN=ACTR10 PE=1 SV=1 - [ARP10_HUMAN]	24.46	1	6	7	16	1.462	1.254	1.055	1.179	46.86	24.46	10	16	417	46.3	7.37
Q7Z3D6	UPF0317 protein C14orf159, mitochondrial OS=Homo sapiens GN=C14orf159 PE=1 SV=2 - [CN159_HUMAN]	7.31	3	5	5	14	0.869	1.876	1.100	1.179	29.33	7.31	6	14	616	66.4	6.79
Q969E2	Secretory carrier-associated membrane protein 4 OS=Homo sapiens GN=SCAMP4 PE=2 SV=1 - [SCAM4_HUMA	4.80	1	1	1	8	1.196	1.125	1.125	1.179	16.64	4.80	2	8	229	25.7	8.82
P38117	Electron transfer flavoprotein subunit beta OS=Homo sapiens GN=ETFB PE=1 SV=3 - [ETFB_HUMAN]	54.51	1	15	15	118	0.991	0.994	0.960	1.180	261.20	54.51	27	118	255	27.8	8.10
Q7RTV3	Zinc finger protein 367 OS=Homo sapiens GN=ZNF367 PE=1 SV=1 - [ZN367_HUMAN]	10.00	1	1	1	1				1.180	2.00	10.00	1	1	350	38.4	7.93
Q96BQ5	Coiled-coil domain-containing protein 127 OS=Homo sapiens GN=CCDC127 PE=1 SV=1 - [CC127_HUMAN]	18.08	8	4	5	14	1.047	1.109	0.904	1.180	31.14	18.08	8	14	260	30.8	9.20
Q6DJT9	Zinc finger protein PLAG1 OS=Homo sapiens GN=PLAG1 PE=1 SV=1 - [PLAG1_HUMAN]	2.20	1	1	1	1	0.691			1.180	1.94	2.20	1	1	500	55.9	8.56

Q9Y6R4	Mitogen-activated protein kinase kinase kinase 4 OS=Homo sapiens GN=MAP3K4 PE=1 SV=2 - [M3K4_HUMAN]	4.60	1	7	10	16	0.937	1.094	1.026	1.180	33.67	4.60	13	16	1608	181.6	6.32
Q96L34	MAP/microtubule affinity-regulating kinase 4 OS=Homo sapiens GN=MARK4 PE=1 SV=1 - [MARK4_HUMAN]	6.52	1	2	3	4	1.549	0.730	1.009	1.180	6.18	6.52	3	4	752	82.5	9.66
Q9NZL9	Methionine adenosyltransferase 2 subunit beta OS=Homo sapiens GN=MAT2B PE=1 SV=1 - [MAT2B_HUMA	41.92	1	13	13	50	1.254	1.174	1.072	1.180	96.60	41.92	20	50	334	37.5	7.36
Q96FV9	THO complex subunit 1 OS=Homo sapiens GN=THOC1 PE=1 SV=1 - [THOC1_HUMAN]	7.46	1	4	4	8	0.948	1.286	1.206	1.180	25.03	7.46	6	8	657	75.6	4.98
Q8N1W1	Rho guanine nucleotide exchange factor 28 OS=Homo sapiens GN=ARHGEF28 PE=1 SV=3 - [ARG28_HUMAN	3.46	1	6	6	13	1.155	0.862	1.137	1.181	26.72	3.46	10	13	1705	191.8	6.04
P22626	Heterogeneous nuclear ribonucleoproteins A2/B1 OS=Homo sapiens GN=HNRNPA2B1 PE=1 SV=2 - [ROA2_HUMAN]	65.44	1	24	28	559	1.139	1.296	0.979	1.181	1573.89	65.44	51	559	353	37.4	8.95
Q9H251	Cadherin-23 OS=Homo sapiens GN=CDH23 PE=1 SV=2 - [CAD23_HUMAN]	1.01	1	2	3	3	0.913	0.949	1.390	1.181	2.54	1.01	3	3	3354	369.3	4.67
P0C221	Coiled-coil domain-containing protein 175 OS=Homo sapiens GN=CCDC175 PE=2 SV=2 - [CC175_HUMAN]	1.51	2	2	2	3	1.630	0.565	0.666	1.181	8.08	1.51	2	3	793	93.6	6.71

Q9NRC6	Spectrin beta chain, non-erythrocytic 5 OS=Homo sapiens GN=SPTBN5 PE=1 SV=2 - [SPTN5_HUMAN]	1.20	1	4	4	4	0.969	0.955	1.024	1.181	8.30	1.20	4	4	3674	416.5	6.65
Q96ST3	Paired amphipathic helix protein Sin3a OS=Homo sapiens GN=SIN3A PE=1 SV=2 - [SIN3A_HUMAN]	9.51	1	12	12	25	1.339	1.314	0.898	1.181	56.37	9.51	17	25	1273	145.1	7.25
O43426	Synaptojanin-1 OS=Homo sapiens GN=SYNJ1 PE=1 SV=2 - [SYNJ1_HUMAN]	4.32	1	6	6	25	1.095	1.080	1.042	1.181	52.25	4.32	10	25	1573	173.0	7.42
Q14814	Myocyte-specific enhancer factor 2D OS=Homo sapiens GN=MEF2D PE=1 SV=1 - [MEF2D_HUMAN]	17.66	1	5	5	11	1.079	1.346	1.108	1.181	22.70	17.66	9	11	521	55.9	7.88
Q96J88	Epithelial-stromal interaction protein 1 OS=Homo sapiens GN=EPSTI1 PE=2 SV=2 - [ESIP1_HUMAN]	5.97	1	2	2	3	1.820	1.021	0.955	1.181	2.35	5.97	3	3	318	36.8	9.89
Q00613	Heat shock factor protein 1 OS=Homo sapiens GN=HSF1 PE=1 SV=1 - [HSF1_HUMAN]	6.81	1	2	2	4	1.297	1.702	1.443	1.182	10.04	6.81	3	4	529	57.2	5.19
P82650	28S ribosomal protein S22, mitochondrial OS=Homo sapiens GN=MRPS22 PE=1 SV=1 - [RT22_HUMAN]	26.11	1	8	8	25	0.832	1.192	0.922	1.182	61.54	26.11	14	25	360	41.3	7.90
O75828	Carbonyl reductase [NADPH] 3 OS=Homo sapiens GN=CBR3 PE=1 SV=3 - [CBR3_HUMAN]	45.13	1	9	12	71	1.436	1.316	1.719	1.182	155.31	45.13	19	71	277	30.8	6.18

O75448	Mediator of RNA polymerase II transcription subunit 24 OS=Homo sapiens GN=MED24 PE=1 SV=1 - [MED24_HUMAN]	3.44	1	3	3	3	1.236	1.219	0.905	1.182	6.99	3.44	3	3	989	110.2	6.95
Q9NY61	Protein AATF OS=Homo sapiens GN=AATF PE=1 SV=1 - [AATF_HUMAN]	4.82	1	2	2	2	0.944	1.113	1.255	1.182	6.07	4.82	2	2	560	63.1	4.94
Q96S15	WD repeat-containing protein 24 OS=Homo sapiens GN=WDR24 PE=1 SV=1 - [WDR24_HUMAN]	1.63	1	1	1	1	1.424	0.863	0.645	1.182	3.01	1.63	1	1	920	101.9	6.58
Q8WXG9	G-protein coupled receptor 98 OS=Homo sapiens GN=GPR98 PE=1 SV=2 - [GPR98_HUMAN]	0.65	1	2	3	3	1.469	0.827	0.737	1.182	3.44	0.65	3	3	6306	692.6	4.64
P42773	Cyclin-dependent kinase 4 inhibitor C OS=Homo sapiens GN=CDKN2C PE=1 SV=1 - [CDN2C_HUMAN]	14.88	1	3	3	8	0.822	2.454	0.979	1.183	13.63	14.88	4	8	168	18.1	6.52
P63173	60S ribosomal protein L38 OS=Homo sapiens GN=RPL38 PE=1 SV=2 - [RL38_HUMAN]	52.86	1	4	5	19	1.208	0.920	0.901	1.183	41.96	52.86	8	19	70	8.2	10.10
P05231	Interleukin-6 OS=Homo sapiens GN=IL6 PE=1 SV=1 - [IL6_HUMAN]	3.30	1	1	1	1	1.872	0.442	1.159	1.183	1.91	3.30	1	1	212	23.7	6.57
Q13938	Calcyphosin OS=Homo sapiens GN=CAPS PE=1 SV=1 - [CAYP1_HUMAN]	9.52	1	2	2	12	1.310	1.931	0.431	1.183	27.12	9.52	2	12	189	21.0	4.89
Q8WVQ1	Soluble calcium-activated nucleotidase 1 OS=Homo sapiens GN=CANT1 PE=1 SV=1 - [CANT1_HUMAN]	3.49	1	1	1	3	0.628	0.945	1.083	1.183	9.36	3.49	1	3	401	44.8	6.09

Q24JP5	Transmembrane protein 132A OS=Homo sapiens GN=TMEM132A PE=2 SV=1 - [T132A_HUMAN]	3.52	1	2	2	5	0.982	1.001	1.153	1.183	16.02	3.52	3	5	1023	110.0	5.62
Q6NZ67	Mitotic-spindle organizing protein 2B OS=Homo sapiens GN=MZT2B PE=1 SV=1 - [MZT2B_HUMAN]	8.86	1	1	1	5	0.997	0.986	1.149	1.183	17.67	8.86	2	5	158	16.2	10.15
Q96NA2	Rab-interacting lysosomal protein OS=Homo sapiens GN=RILP PE=1 SV=1 - [RILP_HUMAN]	2.24	1	1	1	1	1.093	0.994	0.887	1.183	1.96	2.24	1	1	401	44.2	5.59
Q9Y2I7	1-phosphatidylinositol 3-phosphate 5-kinase OS=Homo sapiens GN=PIKFYVE PE=1 SV=3 - [FYV1_HUMAN]	1.00	1	1	2	3	1.638	0.722	0.862	1.183	0.00	1.00	3	3	2098	237.0	6.70
Q7RTP6	Protein-methionine sulfoxide oxidase MICAL3 OS=Homo sapiens GN=MICAL3 PE=1 SV=2 - [MICA3_HUMAN]	6.99	1	12	13	24	0.939	1.381	1.303	1.183	37.86	6.99	17	24	2002	224.2	5.55
Q6P1M0	Long-chain fatty acid transport protein 4 OS=Homo sapiens GN=SLC27A4 PE=1 SV=1 - [S27A4_HUMAN]	11.20	1	6	6	18	1.079	0.966	1.009	1.184	55.44	11.20	9	18	643	72.0	8.47
P11117	Lysosomal acid phosphatase OS=Homo sapiens GN=ACP2 PE=1 SV=3 - [PPAL_HUMAN]	7.33	1	2	2	21	0.997	1.119	0.968	1.184	63.40	7.33	3	21	423	48.3	6.74
Q16836	Hydroxyacyl-coenzyme A dehydrogenase, mitochondrial OS=Homo sapiens GN=HADH PE=1 SV=3 - [HCDH_HUMAN]	66.24	1	17	18	78	0.944	1.471	1.061	1.184	176.71	66.24	26	78	314	34.3	8.85

P29372	DNA-3-methyladenine glycosylase OS=Homo sapiens GN=MPG PE=1 SV=3 - [3MG_HUMAN]	21.81	1	5	5	13	1.216	1.159	1.322	1.184	30.94	21.81	9	13	298	32.8	9.57
Q13503	Mediator of RNA polymerase II transcription subunit 21 OS=Homo sapiens GN=MED21 PE=1 SV=1 - [MED21_HUMAN]	12.50	1	1	1	2	1.031	0.758	0.596	1.184	9.96	12.50	2	2	144	15.6	4.45
Q7Z6M1	Rab9 effector protein with kelch motifs OS=Homo sapiens GN=RABEPK PE=1 SV=1 - [RABEK_HUMAN]	5.65	1	2	2	4	0.713	1.571	0.818	1.184	11.76	5.65	3	4	372	40.5	6.25
O75391	Sperm-associated antigen 7 OS=Homo sapiens GN=SPAG7 PE=1 SV=2 - [SPAG7_HUMAN]	12.78	1	4	4	8	0.995	1.088	1.203	1.184	14.63	12.78	7	8	227	26.0	7.91
Q9UKV5	E3 ubiquitin-protein ligase AMFR OS=Homo sapiens GN=AMFR PE=1 SV=2 - [AMFR_HUMAN]	9.95	1	4	4	12	1.021	0.893	0.878	1.184	28.67	9.95	7	12	643	72.9	6.39
Q5T9G4	Armadillo repeat-containing protein 12 OS=Homo sapiens GN=ARMC12 PE=2 SV=1 - [ARM12_HUMA	2.06	1	1	1	1	1.389	0.963	0.892	1.185	2.09	2.06	1	1	340	38.6	7.88
Q8IXT5	RNA-binding protein 12B OS=Homo sapiens GN=RBM12B PE=1 SV=2 - [RB12B_HUMAN]	5.09	1	5	5	11	0.967	1.338	0.977	1.185	13.43	5.09	6	11	1001	118.0	6.81
Q969Y2	tRNA modification GTPase GTPBP3, mitochondrial OS=Homo sapiens GN=GTPBP3 PE=1 SV=2 - [GTPB3_HUMAN]	3.05	1	1	1	1	0.329	0.751	1.230	1.185	0.00	3.05	1	1	492	52.0	6.48

Q5PRF9	Protein Smaug homolog 2 OS=Homo sapiens GN=SAMD4B PE=1 SV=1 - [SMAG2_HUMAN]	3.31	1	3	3	4	1.210	0.948	1.186	1.185	4.76	3.31	3	4	694	75.4	6.83
Q9Y6X9	MORC family CW-type zinc finger protein 2 OS=Homo sapiens GN=MORC2 PE=1 SV=2 - [MORC2_HUMAN]	2.42	1	2	3	3	0.656	0.881	1.094	1.185	5.24	2.42	3	3	1032	117.7	8.38
Q9Y4X5	E3 ubiquitin-protein ligase ARIH1 OS=Homo sapiens GN=ARIH1 PE=1 SV=2 - [ARI1_HUMAN]	12.21	1	6	6	23	1.064	1.113	1.080	1.185	55.79	12.21	12	23	557	64.1	5.08
Q96KR1	Zinc finger RNA-binding protein OS=Homo sapiens GN=ZFR PE=1 SV=2 - [ZFR_HUMAN]	20.30	1	19	19	52	0.986	1.136	0.980	1.185	118.04	20.30	29	52	1074	116.9	9.04
O94817	Ubiquitin-like protein ATG12 OS=Homo sapiens GN=ATG12 PE=1 SV=1 - [ATG12_HUMAN]	6.43	1	1	1	6	1.151	1.353	1.221	1.185	15.49	6.43	2	6	140	15.1	5.10
Q9H0F6	Sharpin OS=Homo sapiens GN=SHARPIN PE=1 SV=1 - [SHRPN_HUMAN]	6.98	1	2	2	3	1.006	1.050	1.254	1.185	4.22	6.98	3	3	387	39.9	5.80
Q8IY21	Probable ATP-dependent RNA helicase DDX60 OS=Homo sapiens GN=DDX60 PE=1 SV=3 - [DDX60_HUMAN	0.53	1	1	1	1	1.265	1.171	1.415	1.186	1.75	0.53	1	1	1712	197.7	7.59
Q96EV2	RNA-binding protein 33 OS=Homo sapiens GN=RBM33 PE=1 SV=3 - [RBM33_HUMAN]	4.36	1	3	4	15	1.068	1.225	0.857	1.186	29.04	4.36	6	15	1170	129.9	6.93
P13611	Versican core protein OS=Homo sapiens GN=VCAN PE=1 SV=3 - [CSPG2_HUMAN]	0.82	1	2	2	11	1.666	0.866	1.416	1.186	9.74	0.82	2	11	3396	372.6	4.51

Q5T601	Probable G-protein coupled receptor 110 OS=Homo sapiens GN=GPR110 PE=2 SV=2 - [GP110_HUMAN	1.87	1	1	1	1	0.976	1.481	1.404	1.186	2.92	1.87	1	1	910	101.3	8.62
Q8WX92	Negative elongation factor B OS=Homo sapiens GN=NELFB PE=1 SV=1 - [NELFB_HUMAN]	17.07	1	9	11	28	1.225	1.459	1.003	1.186	54.87	17.07	19	28	580	65.7	6.13
O75113	NEDD4-binding protein 1 OS=Homo sapiens GN=N4BP1 PE=1 SV=4 - [N4BP1_HUMAN]	5.69	1	4	4	9	1.058	1.076	1.243	1.186	14.78	5.69	5	9	896	100.3	5.36
P82979	SAP domain-containing ribonucleoprotein OS=Homo sapiens GN=SARNP PE=1 SV=3 - [SARNP_HUMAN]	29.05	1	5	5	25	1.129	1.275	1.027	1.186	58.76	29.05	10	25	210	23.7	6.42
P35754	Glutaredoxin-1 OS=Homo sapiens GN=GLRX PE=1 SV=2 - [GLRX1_HUMAN]	66.04	1	6	6	89	1.705	0.455	0.840	1.186	220.23	66.04	11	89	106	11.8	8.09
Q01081	Splicing factor U2AF 35 kDa subunit OS=Homo sapiens GN=U2AF1 PE=1 SV=3 - [U2AF1_HUMAN]	35.42	2	5	5	29	1.063	1.241	1.079	1.187	62.02	35.42	8	29	240	27.9	8.81
Q676U5	Autophagy-related protein 16-1 OS=Homo sapiens GN=ATG16L1 PE=1 SV=2 - [A16L1_HUMAN]	9.06	1	7	7	15	1.002	0.988	1.173	1.187	26.50	9.06	11	15	607	68.2	6.64
A0JLT2	Mediator of RNA polymerase II transcription subunit 19 OS=Homo sapiens GN=MED19 PE=1 SV=2 - [MED19_HUMAN]	15.16	1	2	2	10	0.875	1.231	1.332	1.187	6.36	15.16	3	10	244	26.3	9.82
Q6UXN9	WD repeat-containing protein 82 OS=Homo sapiens GN=WDR82 PE=1 SV=1 - [WDR82_HUMAN]	20.45	1	6	6	17	1.103	1.307	0.874	1.187	38.57	20.45	9	17	313	35.1	7.69

P51153	Ras-related protein Rab-13 OS=Homo sapiens GN=RAB13 PE=1 SV=1 - [RAB13_HUMAN]	55.67	1	10	13	76	1.357	1.306	0.815	1.187	163.01	55.67	20	76	203	22.8	9.19
Q99941	Cyclic AMP-dependent transcription factor ATF-6 beta OS=Homo sapiens GN=ATF6B PE=1 SV=2 - [ATF6B_HUMAN	1.71	1	1	1	2	0.819	1.098	1.180	1.187	4.36	1.71	2	2	703	76.7	6.27
O95672	Endothelin-converting enzyme-like 1 OS=Homo sapiens GN=ECEL1 PE=1 SV=3 - [ECEL1_HUMAN]	1.42	1	1	1	2	1.320	1.018	1.222	1.187	5.73	1.42	1	2	775	87.7	7.03
P12882	Myosin-1 OS=Homo sapiens GN=MYH1 PE=1 SV=3 - [MYH1_HUMAN]	6.81	2	3	13	87	0.995	1.017	1.039	1.187	171.30	6.81	16	87	1939	223.0	5.74
Q9H583	HEAT repeat-containing protein 1 OS=Homo sapiens GN=HEATR1 PE=1 SV=3 - [HEAT1_HUMAN]	5.13	1	10	10	23	0.804	0.862	0.838	1.187	43.37	5.13	16	23	2144	242.2	6.54
P16949	Stathmin OS=Homo sapiens GN=STMN1 PE=1 SV=3 - [STMN1_HUMAN]	48.99	4	11	12	85	1.378	1.698	0.915	1.188	179.54	48.99	18	85	149	17.3	5.97
O75616	GTPase Era, mitochondrial OS=Homo sapiens GN=ERAL1 PE=1 SV=2 - [ERAL1_HUMAN]	6.41	1	2	2	5	1.264	0.877	1.059	1.188	2.11	6.41	2	5	437	48.3	8.84
Q96JM3	Chromosome alignment-maintaining phosphoprotein 1 OS=Homo sapiens GN=CHAMP1 PE=1 SV=2 - [CHAP1_HUMAN]	13.42	1	10	10	17	1.133	1.344	1.048	1.188	28.87	13.42	15	17	812	89.0	8.44
Q13685	Angio-associated migratory cell protein OS=Homo sapiens GN=AAMP PE=1 SV=2 - [AAMP_HUMAN]	10.14	1	4	4	12	0.853	1.205	1.119	1.188	23.99	10.14	5	12	434	46.7	4.42

P69849	Nodal modulator 3 OS=Homo sapiens GN=NOMO3 PE=2 SV=2 - [NOMO3_HUMAN]	19.31	2	2	20	73	0.973	0.746	0.688	1.188	185.42	19.31	32	73	1222	134.0	5.67
Q92797	Symplekin OS=Homo sapiens GN=SYMPK PE=1 SV=2 - [SYMPK_HUMAN]	9.11	1	8	8	20	1.009	1.301	1.136	1.188	46.06	9.11	13	20	1274	141.1	6.13
Q8WU79	Stromal membrane-associated protein 2 OS=Homo sapiens GN=SMAP2 PE=1 SV=1 - [SMAP2_HUMAN]	13.52	1	5	5	15	1.134	0.969	0.952	1.189	38.45	13.52	7	15	429	46.8	8.87
Q6YN16	Hydroxysteroid dehydrogenase-like protein 2 OS=Homo sapiens GN=HSDL2 PE=1 SV=1 - [HSDL2_HUMAN]	35.41	1	12	12	54	0.836	1.174	1.076	1.189	178.42	35.41	20	54	418	45.4	7.99
Q9NR28	Diablo homolog, mitochondrial OS=Homo sapiens GN=DIABLO PE=1 SV=1 - [DBLOH_HUMAN]	28.45	1	6	6	28	1.108	1.177	1.133	1.189	70.96	28.45	11	28	239	27.1	5.90
Q92621	Nuclear pore complex protein Nup205 OS=Homo sapiens GN=NUP205 PE=1 SV=3 - [NU205_HUMAN	8.50	1	18	18	40	1.115	1.025	0.887	1.189	93.03	8.50	29	40	2012	227.8	6.19
Q9Y2D4	Exocyst complex component 6B OS=Homo sapiens GN=EXOC6B PE=1 SV=3 - [EXC6B_HUMAN]	20.22	2	14	16	43	1.244	0.955	1.067	1.189	107.07	20.22	22	43	811	94.1	6.46
Q9NQG7	Hermansky-Pudlak syndrome 4 protein OS=Homo sapiens GN=HPS4 PE=1 SV=2 - [HPS4_HUMAN]	2.54	1	1	1	1	1.021	0.814	1.095	1.189	0.00	2.54	1	1	708	76.9	5.45
Q9HAT2	Sialate O-acetylesterase OS=Homo sapiens GN=SIAE PE=1 SV=1 - [SIAE_HUMAN]	10.52	1	6	6	18	0.794	0.978	0.997	1.190	41.32	10.52	11	18	523	58.3	7.33

O43663	Protein regulator of cytokinesis 1 OS=Homo sapiens GN=PRC1 PE=1 SV=2 - [PRC1_HUMAN]	6.77	3	3	6	12	1.062	1.007	0.926	1.190	20.92	6.77	6	12	620	71.6	6.68
Q9Y692	Glucocorticoid modulatory element-binding protein 1 OS=Homo sapiens GN=GMEB1 PE=1 SV=2 - [GMEB1_HUMAN]	2.62	2	2	3	9	1.924	1.456	1.018	1.190	22.87	2.62	3	9	573	62.6	4.83
Q6KC79	Nipped-B-like protein OS=Homo sapiens GN=NIPBL PE=1 SV=2 - [NIPBL_HUMAN]	2.03	1	6	6	7	1.246	1.362	1.123	1.190	6.40	2.03	7	7	2804	315.9	7.91
Q8N9N8	Probable RNA-binding protein EIF1AD OS=Homo sapiens GN=EIF1AD PE=1 SV=1 - [EIF1A_HUMAN]	10.30	1	1	1	5	1.332	0.902	1.114	1.190	15.20	10.30	2	5	165	19.0	5.21
Q8N4H5	Mitochondrial import receptor subunit TOM5 homolog OS=Homo sapiens GN=TOMM5 PE=1 SV=1 - [TOM5_HUMAN	41.18	1	3	3	10	1.064	0.740	0.544	1.190	21.19	41.18	5	10	51	6.0	9.70
Q9BSH5	Haloacid dehalogenase-like hydrolase domain-containing protein 3 OS=Homo sapiens GN=HDHD3 PE=1 SV=1 - [HDHD3_HUMAN]	22.31	1	4	4	9	0.794	1.422	1.079	1.190	21.32	22.31	6	9	251	28.0	6.71
Q96I25	Splicing factor 45 OS=Homo sapiens GN=RBM17 PE=1 SV=1 - [SPF45_HUMAN]	10.47	1	4	5	28	1.017	1.292	1.084	1.190	58.98	10.47	8	28	401	44.9	5.97
Q7Z5N4	Protein sidekick-1 OS=Homo sapiens GN=SDK1 PE=1 SV=3 - [SDK1_HUMAN]	3.21	1	4	4	10	1.286	0.720	0.511	1.191	28.70	3.21	5	10	2213	242.0	6.39

P46087	Probable 28S rRNA (cytosine(4447)-C(5))-methyltransferase OS=Homo sapiens GN=NOP2 PE=1 SV=2 - [NOP2_HUMAN]	23.89	1	17	17	44	0.853	0.961	0.911	1.191	105.35	23.89	24	44	812	89.2	9.23
O95834	Echinoderm microtubule-associated protein-like 2 OS=Homo sapiens GN=EML2 PE=1 SV=1 - [EMAL2_HUMAN]	16.02	1	10	10	50	1.620	0.728	0.822	1.191	109.65	16.02	17	50	649	70.6	6.32
O75964	ATP synthase subunit g, mitochondrial OS=Homo sapiens GN=ATP5L PE=1 SV=3 - [ATP5L_HUMAN]	51.46	2	5	6	20	1.049	0.943	0.698	1.191	51.04	51.46	9	20	103	11.4	9.64
Q7Z6B0	Coiled-coil domain-containing protein 91 OS=Homo sapiens GN=CCDC91 PE=1 SV=2 - [CCD91_HUMAN]	11.56	1	6	7	18	1.056	1.493	1.291	1.192	37.26	11.56	9	18	441	49.9	5.07
Q14694	Ubiquitin carboxyl-terminal hydrolase 10 OS=Homo sapiens GN=USP10 PE=1 SV=2 - [UBP10_HUMAN]	14.04	1	11	11	31	1.148	1.120	1.015	1.192	63.38	14.04	17	31	798	87.1	5.31
Q9P2K6	Kelch-like protein 42 OS=Homo sapiens GN=KLHL42 PE=1 SV=2 - [KLH42_HUMAN]	2.97	1	2	2	3	1.204	1.329	1.093	1.192	6.12	2.97	2	3	505	56.8	5.74
Q8TB52	F-box only protein 30 OS=Homo sapiens GN=FBXO30 PE=1 SV=3 - [FBX30_HUMAN]	3.09	1	3	3	5	1.006	1.250	1.073	1.192	8.75	3.09	4	5	745	82.3	5.40
O76071	Probable cytosolic iron-sulfur protein assembly protein CIAO1 OS=Homo sapiens GN=CIAO1 PE=1 SV=1 - [CIAO1_HUMAN]	5.31	1	2	2	2	1.293	0.914	0.919	1.192	4.19	5.31	2	2	339	37.8	4.97

Q4U2R6	39S ribosomal protein L51, mitochondrial OS=Homo sapiens GN=MRPL51 PE=1 SV=1 - [RM51_HUMAN]	28.13	1	4	4	9	0.914	1.316	1.024	1.192	17.85	28.13	6	9	128	15.1	11.27
A1X283	SH3 and PX domain-containing protein 2B OS=Homo sapiens GN=SH3PXD2B PE=1 SV=3 - [SPD2B_HUMAN]	22.39	1	20	21	75	1.242	1.380	1.245	1.192	158.87	22.39	34	75	911	101.5	8.69
Q99685	Monoglyceride lipase OS=Homo sapiens GN=MGLL PE=1 SV=2 - [MGLL_HUMAN]	22.11	1	6	6	21	1.479	1.636	1.271	1.193	39.07	22.11	9	21	303	33.2	6.99
P07900	Heat shock protein HSP 90-alpha OS=Homo sapiens GN=HSP90AA1 PE=1 SV=5 - [HS90A_HUMAN]	59.15	4	32	55	1078	0.969	1.113	1.087	1.193	2744.65	59.15	87	1078	732	84.6	5.02
Q9UNX4	WD repeat-containing protein 3 OS=Homo sapiens GN=WDR3 PE=1 SV=1 - [WDR3_HUMAN]	4.98	1	4	4	6	0.836	0.959	0.831	1.193	8.20	4.98	5	6	943	106.0	6.64
Q14978	Nucleolar and coiled-body phosphoprotein 1 OS=Homo sapiens GN=NOLC1 PE=1 SV=2 - [NOLC1_HUMAN]	21.46	1	19	19	64	0.908	1.045	0.857	1.193	87.36	21.46	31	64	699	73.6	9.47
O43166	Signal-induced proliferation-associated 1-like protein 1 OS=Homo sapiens GN=SIPA1L1 PE=1 SV=4 - [SI1L1_HUMAN]	4.82	1	7	7	18	0.934	0.806	0.876	1.193	51.45	4.82	13	18	1804	199.9	8.19
Q15007	Pre-mRNA-splicing regulator WTAP OS=Homo sapiens GN=WTAP PE=1 SV=2 - [FL2D_HUMAN]	3.79	1	2	2	2	0.963	1.360	1.345	1.194	4.86	3.79	2	2	396	44.2	5.19

Q9BX66	Sorbin and SH3 domain-containing protein 1 OS=Homo sapiens GN=SORBS1 PE=1 SV=3 - [SRBS1_HUMAN]	15.25	1	14	15	27	1.098	4.941	0.935	1.194	75.41	15.25	22	27	1292	142.4	6.84
Q9BZ29	Dedicator of cytokinesis protein 9 OS=Homo sapiens GN=DOCK9 PE=1 SV=2 - [DOCK9_HUMAN]	1.26	1	3	3	5	1.051	0.932	0.919	1.194	4.36	1.26	3	5	2069	236.3	7.49
O60869	Endothelial differentiation-related factor 1 OS=Homo sapiens GN=EDF1 PE=1 SV=1 - [EDF1_HUMAN]	39.86	1	7	7	41	1.046	1.129	1.082	1.194	75.18	39.86	12	41	148	16.4	9.95
Q96JN8	Neuralized-like protein 4 OS=Homo sapiens GN=NEURL4 PE=1 SV=2 - [NEUL4_HUMAN]	0.96	1	2	2	3	0.791	0.220	0.406	1.194	4.29	0.96	2	3	1562	166.8	5.86
Q9HAU0	Pleckstrin homology domain-containing family A member 5 OS=Homo sapiens GN=PLEKHA5 PE=1 SV=1 - [PKHA5_HUMAN]	25.36	1	20	21	75	1.641	1.409	0.868	1.194	185.01	25.36	33	75	1116	127.4	7.53
P52815	39S ribosomal protein L12, mitochondrial OS=Homo sapiens GN=MRPL12 PE=1 SV=2 - [RM12_HUMAN]	23.74	1	6	6	27	1.183	1.255	1.113	1.195	48.22	23.74	11	27	198	21.3	8.87
O00472	RNA polymerase II elongation factor ELL2 OS=Homo sapiens GN=ELL2 PE=1 SV=2 - [ELL2_HUMAN]	2.03	1	1	1	9	0.765	0.479	0.577	1.195	11.92	2.03	1	9	640	72.3	9.00
Q29RF7	Sister chromatid cohesion protein PDS5 homolog A OS=Homo sapiens GN=PDS5A PE=1 SV=1 - [PDS5A_HUMAN	8.30	1	10	11	15	1.141	1.233	1.136	1.195	29.09	8.30	12	15	1337	150.7	7.91

Q9BXJ1	Complement C1q tumor necrosis factor-related protein 1 OS=Homo sapiens GN=C1QTNF1 PE=1 SV=1 - [C1QT1_HUMAN]	4.63	1	1	1	3	0.802	1.292	0.445	1.195	12.05	4.63	2	3	281	31.7	6.90
Q9NX40	OCIA domain-containing protein 1 OS=Homo sapiens GN=OCIAD1 PE=1 SV=1 - [OCAD1_HUMAN]	42.04	1	12	12	39	1.305	1.029	0.823	1.195	104.14	42.04	18	39	245	27.6	7.49
O75264	Small integral membrane protein 24 OS=Homo sapiens GN=SMIM24 PE=2 SV=2 - [SIM24_HUMAN]	10.00	1	1	1	2	1.393	0.960	1.263	1.195	4.51	10.00	1	2	130	15.0	5.29
P32418	Sodium/calcium exchanger 1 OS=Homo sapiens GN=SLC8A1 PE=1 SV=3 - [NAC1_HUMAN]	5.96	1	4	4	16	0.845	0.774	0.628	1.196	28.49	5.96	8	16	973	108.5	5.00
Q14149	MORC family CW-type zinc finger protein 3 OS=Homo sapiens GN=MORC3 PE=1 SV=3 - [MORC3_HUMAN]	2.66	1	2	2	2	1.585	0.794	1.076	1.196	4.70	2.66	2	2	939	107.0	5.60
Q96CB8	Integrator complex subunit 12 OS=Homo sapiens GN=INTS12 PE=1 SV=1 - [INT12_HUMAN]	10.17	1	3	4	7	1.262	1.548	1.185	1.196	12.94	10.17	6	7	462	48.8	9.69
P43378	Tyrosine-protein phosphatase non-receptor type 9 OS=Homo sapiens GN=PTPN9 PE=1 SV=1 - [PTN9_HUMAN]	7.25	1	4	4	13	1.346	0.988	0.882	1.196	33.91	7.25	8	13	593	68.0	8.02
Q9ULC6	Protein-arginine deiminase type-1 OS=Homo sapiens GN=PADI1 PE=1 SV=2 - [PADI1_HUMAN	3.47	1	1	1	1	2.478	2.479	1.630	1.196	2.90	3.47	1	1	663	74.6	6.49

Q00839	Heterogeneous nuclear ribonucleoprotein U OS=Homo sapiens GN=HNRNPU PE=1 SV=6 - [HNRPU_HUMAN	34.55	1	34	34	321	1.157	1.212	0.922	1.196	817.39	34.55	44	321	825	90.5	6.00
Q99608	Necdin OS=Homo sapiens GN=NDN PE=2 SV=1 - [NECD_HUMAN]	3.12	1	1	1	4	0.395	0.485	0.364	1.196	7.60	3.12	2	4	321	36.1	8.78
Q8IVB4	Sodium/hydrogen exchanger 9 OS=Homo sapiens GN=SLC9A9 PE=1 SV=1 - [SL9A9_HUMAN]	6.51	1	2	2	5	1.462	1.228	0.828	1.197	13.00	6.51	3	5	645	72.5	6.21
Q5JTD0	Tight junction-associated protein 1 OS=Homo sapiens GN=TJAP1 PE=1 SV=1 - [TJAP1_HUMAN]	10.77	1	5	6	10	0.918	1.134	1.123	1.197	15.53	10.77	8	10	557	61.8	5.97
P09016	Homeobox protein Hox-D4 OS=Homo sapiens GN=HOXD4 PE=2 SV=3 - [HXD4_HUMAN]	6.27	1	1	1	3	3.275	1.518	1.257	1.197	0.00	6.27	1	3	255	27.9	9.39
Q8NDW8	Tetratricopeptide repeat protein 21A OS=Homo sapiens GN=TTC21A PE=2 SV=3 - [TT21A_HUMAN]	1.67	1	1	2	3	1.074	0.653	1.192	1.197	1.85	1.67	2	3	1320	150.8	7.24
Q9NZR1	Tropomodulin-2 OS=Homo sapiens GN=TMOD2 PE=1 SV=1 - [TMOD2_HUMAN]	23.08	1	7	8	23	1.387	0.751	1.051	1.197	44.36	23.08	11	23	351	39.6	5.27
Q86VM9	Zinc finger CCCH domain-containing protein 18 OS=Homo sapiens GN=ZC3H18 PE=1 SV=2 - [ZCH18_HUMAN]	11.54	2	9	10	26	1.097	1.414	0.969	1.197	56.91	11.54	16	26	953	106.3	8.32
P51798	H(+)/Cl(-) exchange transporter 7 OS=Homo sapiens GN=CLCN7 PE=1 SV=2 - [CLCN7_HUMAN]	7.08	1	5	5	25	0.803	0.710	0.818	1.197	76.22	7.08	8	25	805	88.6	8.43

Q9NZN8	CCR4-NOT transcription complex subunit 2 OS=Homo sapiens GN=CNOT2 PE=1 SV=1 - [CNOT2_HUMAN]	9.63	1	4	4	11	0.715	0.917	1.056	1.197	31.48	9.63	6	11	540	59.7	7.66
O43298	Zinc finger and BTB domain-containing protein 43 OS=Homo sapiens GN=ZBTB43 PE=1 SV=1 - [ZBT43_HUMAN]	3.21	1	1	1	1	1.344	1.182	1.077	1.198	0.00	3.21	1	1	467	52.6	5.76
Q58DX5	Inactive N-acetylated-alpha-linked acidic dipeptidase-like protein 2 OS=Homo sapiens GN=NAALADL2 PE=1 SV=3 - [NADL2_HUMAN	7.17	1	5	5	11	0.807	0.712	0.854	1.198	23.89	7.17	8	11	795	88.6	6.15
P42330	Aldo-keto reductase family 1 member C3 OS=Homo sapiens GN=AKR1C3 PE=1 SV=4 - [AK1C3_HUMAN	50.46	3	5	16	154	0.843	0.682	0.971	1.198	326.02	50.46	24	154	323	36.8	7.94
P14678	Small nuclear ribonucleoprotein-associated proteins B and B' OS=Homo sapiens GN=SNRPB PE=1 SV=2 - [RSMB_HUMAN]	15.00	2	4	4	17	1.042	1.217	1.152	1.199	43.97	15.00	7	17	240	24.6	11.19
Q6ZPD9	Probable C-mannosyltransferase DPY19L3 OS=Homo sapiens GN=DPY19L3 PE=2 SV=1 - [D19L3_HUMAN]	1.54	1	1	1	1	1.880	1.462	1.112	1.199	2.60	1.54	1	1	716	83.1	8.72
Q9H8M9	Protein eva-1 homolog A OS=Homo sapiens GN=EVA1A PE=2 SV=1 - [EVA1A_HUMAN]	7.24	1	1	1	4	0.599	0.811	1.534	1.199	7.75	7.24	2	4	152	17.5	6.93
Q08AE8	Protein spire homolog 1 OS=Homo sapiens GN=SPIRE1 PE=1 SV=3 - [SPIR1_HUMAN]	5.95	3	2	4	14	0.449	0.969	1.477	1.199	26.84	5.95	5	14	756	85.5	8.62

Q9Y3E2	BolA-like protein 1 OS=Homo sapiens GN=BOLA1 PE=1 SV=1 - [BOLA1_HUMAN]	16.06	1	2	2	6	1.122	1.436	1.284	1.199	13.53	16.06	3	6	137	14.3	7.96
Q5T6F2	Ubiquitin-associated protein 2 OS=Homo sapiens GN=UBAP2 PE=1 SV=1 - [UBAP2_HUMAN]	10.01	1	9	10	23	0.960	1.144	1.047	1.200	49.46	10.01	15	23	1119	117.0	7.34
P61587	Rho-related GTP-binding protein RhoE OS=Homo sapiens GN=RND3 PE=1 SV=1 - [RND3_HUMAN]	32.38	1	7	7	20	1.067	0.314	0.508	1.200	54.59	32.38	12	20	244	27.4	8.48
Q9Y5W7	Sorting nexin-14 OS=Homo sapiens GN=SNX14 PE=1 SV=3 - [SNX14_HUMAN]	0.95	1	1	1	1	1.403	1.391	1.007	1.200	0.00	0.95	1	1	946	110.1	6.70
Q9NQ50	39S ribosomal protein L40, mitochondrial OS=Homo sapiens GN=MRPL40 PE=1 SV=1 - [RM40_HUMAN]	15.05	1	3	3	13	0.972	1.334	0.970	1.200	33.67	15.05	6	13	206	24.5	9.63
Q9UHD2	Serine/threonine-protein kinase TBK1 OS=Homo sapiens GN=TBK1 PE=1 SV=1 - [TBK1_HUMAN]	24.55	1	14	15	44	1.061	1.010	0.879	1.200	110.77	24.55	24	44	729	83.6	6.79
A6NKD9	Coiled-coil domain-containing protein 85C OS=Homo sapiens GN=CCDC85C PE=1 SV=1 - [CC85C_HUMAN]	5.25	1	2	2	6	1.341	1.849	1.033	1.200	13.52	5.25	4	6	419	45.2	6.96
A6NHR9	Structural maintenance of chromosomes flexible hinge domain-containing protein 1 OS=Homo sapiens GN=SMCHD1 PE=1 SV=2 - [SMHD1_HUMA	12.57	1	22	23	42	1.188	1.253	1.009	1.200	83.82	12.57	31	42	2005	226.2	7.30

Q9H2P0	Activity-dependent neuroprotector homeobox protein OS=Homo sapiens GN=ADNP PE=1 SV=1 - [ADNP_HUMAN]	9.80	1	10	10	25	1.110	1.387	1.260	1.200	44.60	9.80	14	25	1102	123.5	7.34
Q86V81	THO complex subunit 4 OS=Homo sapiens GN=ALYREF PE=1 SV=3 - [THOC4_HUMAN]	47.86	1	8	8	31	1.250	1.823	0.955	1.201	108.87	47.86	11	31	257	26.9	11.15
Q04656	Copper-transporting ATPase 1 OS=Homo sapiens GN=ATP7A PE=1 SV=3 - [ATP7A_HUMAN]	4.13	3	6	7	15	1.359	1.139	1.077	1.201	23.61	4.13	11	15	1500	163.3	6.24
Q13435	Splicing factor 3B subunit 2 OS=Homo sapiens GN=SF3B2 PE=1 SV=2 - [SF3B2_HUMAN]	37.21	1	28	29	142	1.082	1.363	1.115	1.201	316.96	37.21	49	142	895	100.2	5.67
Q92736	Ryanodine receptor 2 OS=Homo sapiens GN=RYR2 PE=1 SV=3 - [RYR2_HUMAN]	1.91	1	5	7	9	0.641	1.059	0.791	1.201	15.52	1.91	7	9	4967	564.2	6.07
Q96C86	m7GpppX diphosphatase OS=Homo sapiens GN=DCPS PE=1 SV=2 - [DCPS_HUMAN]	32.64	1	7	7	18	1.460	0.994	0.885	1.201	47.42	32.64	10	18	337	38.6	6.38
Q8IWZ3	Ankyrin repeat and KH domain-containing protein 1 OS=Homo sapiens GN=ANKHD1 PE=1 SV=1 - [ANKH1_HUMAN]	3.70	1	4	8	13	0.849	0.738	0.783	1.201	40.48	3.70	10	13	2542	269.3	5.73
P14866	Heterogeneous nuclear ribonucleoprotein L OS=Homo sapiens GN=HNRNPL PE=1 SV=2 - [HNRPL_HUMAN	31.24	1	13	14	117	1.190	1.290	1.044	1.201	305.99	31.24	25	117	589	64.1	8.22
Q9UBE0	SUMO-activating enzyme subunit 1 OS=Homo sapiens GN=SAE1 PE=1 SV=1 - [SAE1_HUMAN]	39.88	1	13	13	56	1.012	0.941	0.856	1.202	155.31	39.88	19	56	346	38.4	5.30

Q9UBU9	Nuclear RNA export factor 1 OS=Homo sapiens GN=NXF1 PE=1 SV=1 - [NXF1_HUMAN]	5.98	1	3	4	7	1.355	1.068	0.978	1.202	8.32	5.98	6	7	619	70.1	8.51
Q9BZE4	Nucleolar GTP-binding protein 1 OS=Homo sapiens GN=GTPBP4 PE=1 SV=3 - [NOG1_HUMAN]	19.09	1	12	12	27	0.832	0.823	0.698	1.202	62.03	19.09	17	27	634	73.9	9.50
Q8ND24	RING finger protein 214 OS=Homo sapiens GN=RNF214 PE=1 SV=2 - [RN214_HUMAN]	18.21	2	11	12	80	1.219	1.033	0.936	1.202	179.55	18.21	18	80	703	77.6	6.95
Q14166	Tubulin--tyrosine ligase-like protein 12 OS=Homo sapiens GN=TTLL12 PE=1 SV=2 - [TTL12_HUMAN]	28.26	1	12	12	41	1.126	0.815	0.771	1.202	102.31	28.26	22	41	644	74.4	5.53
P54259	Atrophin-1 OS=Homo sapiens GN=ATN1 PE=1 SV=3 - [ATN1_HUMAN]	2.10	1	3	3	5	1.245	1.282	1.152	1.202	4.70	2.10	4	5	1190	125.3	8.98
Q16698	2,4-dienoyl-CoA reductase, mitochondrial OS=Homo sapiens GN=DECR1 PE=1 SV=1 - [DECR_HUMAN]	42.09	1	14	14	123	1.132	1.123	1.042	1.202	278.49	42.09	22	123	335	36.0	9.28
P27707	Deoxycytidine kinase OS=Homo sapiens GN=DCK PE=1 SV=1 - [DCK_HUMAN]	14.62	1	3	3	4	1.446	1.022	1.070	1.202	12.47	14.62	4	4	260	30.5	5.21
Q9UEE9	Craniofacial development protein 1 OS=Homo sapiens GN=CFDP1 PE=1 SV=1 - [CFDP1_HUMAN]	17.73	1	4	4	12	0.916	1.325	0.984	1.202	30.32	17.73	5	12	299	33.6	4.81
Q9Y2Q9	28S ribosomal protein S28, mitochondrial OS=Homo sapiens GN=MRPS28 PE=1 SV=1 - [RT28_HUMAN]	9.09	1	2	2	5	1.102	1.247	0.956	1.202	14.36	9.09	3	5	187	20.8	9.10

P18583	Protein SON OS=Homo sapiens GN=SON PE=1 SV=4 - [SON_HUMAN]	9.48	1	17	17	36	1.170	1.101	1.073	1.202	70.67	9.48	26	36	2426	263.7	5.64
Q8N9V3	WD repeat, SAM and U-box domain-containing protein 1 OS=Homo sapiens GN=WDSUB1 PE=1 SV=3 - [WSDU1_HUMAN]	3.15	1	1	1	1	2.015	3.096	1.867	1.203	2.71	3.15	1	1	476	52.8	6.37
Q8N1G1	RNA exonuclease 1 homolog OS=Homo sapiens GN=REXO1 PE=1 SV=3 - [REXO1_HUMAN]	0.82	1	1	1	1	0.627	0.993	0.740	1.203	3.15	0.82	1	1	1221	131.4	8.95
Q9Y614	Actin-like protein 7B OS=Homo sapiens GN=ACTL7B PE=2 SV=1 - [ACL7B_HUMAN]	1.93	1	1	1	1	0.649	1.098	1.392	1.203	2.12	1.93	1	1	415	45.2	5.49
Q96P50	Arf-GAP with coiled-coil, ANK repeat and PH domain-containing protein 3 OS=Homo sapiens GN=ACAP3 PE=1 SV=2 - [ACAP3_HUMAN]	1.20	1	1	1	2	0.822	0.916	0.827	1.203	6.79	1.20	2	2	834	92.4	5.99
O95396	Adenylyltransferase and sulfurtransferase MOCS3 OS=Homo sapiens GN=MOCS3 PE=1 SV=1 - [MOCS3_HUMAN]	8.70	1	4	4	9	1.156	1.087	1.260	1.203	12.51	8.70	7	9	460	49.6	6.21
Q6ZSJ8	Uncharacterized protein C1orf122 OS=Homo sapiens GN=C1orf122 PE=2 SV=2 - [CA122_HUMAN]	22.73	1	2	2	4	0.757	0.881	1.166	1.203	7.78	22.73	4	4	110	11.5	6.73
Q9BXV9	Uncharacterized protein C14orf142 OS=Homo sapiens GN=C14orf142 PE=1 SV=2 - [CN142_HUMAN]	27.00	1	2	2	7	0.921	0.983	1.112	1.203	21.96	27.00	4	7	100	10.9	4.27

O00204	Sulfotransferase family cytosolic 2B member 1 OS=Homo sapiens GN=SULT2B1 PE=1 SV=2 - [ST2B1_HUMAN	1.64	1	1	1	2	1.434	0.959	0.748	1.203	3.90	1.64	1	2	365	41.3	5.40
Q02127	Dihydroorotate dehydrogenase (quinone), mitochondrial OS=Homo sapiens GN=DHODH PE=1 SV=3 - [PYRD_HUMAN]	13.42	1	4	4	5	0.914	0.995	0.891	1.203	16.90	13.42	5	5	395	42.8	9.67
Q9NVI7	ATPase family AAA domain-containing protein 3A OS=Homo sapiens GN=ATAD3A PE=1 SV=2 - [ATD3A_HUMAN]	44.48	2	9	25	99	1.071	0.962	0.938	1.204	270.36	44.48	38	99	634	71.3	8.98
Q16666	Gamma-interferon-inducible protein 16 OS=Homo sapiens GN=IFI16 PE=1 SV=3 - [IF16_HUMAN]	34.39	1	19	23	69	0.953	0.899	0.729	1.204	197.67	34.39	35	69	785	88.2	9.28
O60879	Protein diaphanous homolog 2 OS=Homo sapiens GN=DIAPH2 PE=1 SV=1 - [DIAP2_HUMAN]	17.35	1	19	20	53	1.088	1.302	1.330	1.204	121.65	17.35	31	53	1101	125.5	6.58
Q8TBY8	Polyamine-modulated factor 1-binding protein 1 OS=Homo sapiens GN=PMFBP1 PE=2 SV=1 - [PMFBP_HUMAN]	4.89	1	3	4	6	1.173	1.035	0.955	1.204	8.83	4.89	4	6	1022	119.0	6.29
Q9NZC7	WW domain-containing oxidoreductase OS=Homo sapiens GN=WWOX PE=1 SV=1 - [WWOX_HUMAN]	3.62	1	1	1	3	1.705	0.947	1.060	1.205	7.46	3.62	2	3	414	46.6	7.17
Q8N568	Serine/threonine-protein kinase DCLK2 OS=Homo sapiens GN=DCLK2 PE=2 SV=4 - [DCLK2_HUMAN]	1.70	1	1	1	1	0.805	1.143	2.020	1.205	3.77	1.70	1	1	766	83.6	8.34

Q9NRG7	Epimerase family protein SDR39U1 OS=Homo sapiens GN=SDR39U1 PE=1 SV=2 - [D39U1_HUMAN]	7.84	1	3	3	7	1.040	1.210	0.962	1.205	11.54	7.84	5	7	319	34.7	9.77
P48995	Short transient receptor potential channel 1 OS=Homo sapiens GN=TRPC1 PE=1 SV=1 - [TRPC1_HUMAN	3.03	1	1	2	4		1.628	1.676	1.205	5.80	3.03	3	4	793	91.2	8.05
Q6ZVH7	Espin-like protein OS=Homo sapiens GN=ESPNL PE=2 SV=3 - [ESPNL_HUMAN]	5.97	1	2	3	6	1.053	0.779	0.932	1.205	9.48	5.97	4	6	1005	108.1	6.47
Q7RTV5	Thioredoxin-like protein AAED1 OS=Homo sapiens GN=AAED1 PE=2 SV=1 - [AAED1_HUMAN]	6.64	1	1	1	1	0.498	0.745	1.237	1.205	4.86	6.64	1	1	226	24.8	8.91
Q9HC52	Chromobox protein homolog 8 OS=Homo sapiens GN=CBX8 PE=1 SV=3 - [CBX8_HUMAN]	2.57	1	1	1	4	1.357	1.415	0.978	1.205	7.26	2.57	2	4	389	43.4	9.91
Q9UH99	SUN domain-containing protein 2 OS=Homo sapiens GN=SUN2 PE=1 SV=3 - [SUN2_HUMAN]	28.03	1	14	15	35	1.371	1.847	1.261	1.206	119.03	28.03	22	35	717	80.3	6.73
Q9BX79	Stimulated by retinoic acid gene 6 protein homolog OS=Homo sapiens GN=STRA6 PE=1 SV=1 - [STRA6_HUMAN]	2.85	1	1	1	1	1.427	1.096	1.587	1.206	2.04	2.85	1	1	667	73.5	8.98
Q96F46	Interleukin-17 receptor A OS=Homo sapiens GN=IL17RA PE=1 SV=2 - [I17RA_HUMAN]	2.31	1	2	2	5	1.037	1.440	1.235	1.206	15.19	2.31	3	5	866	96.1	5.21
P12821	Angiotensin-converting enzyme OS=Homo sapiens GN=ACE PE=1 SV=1 - [ACE_HUMAN]	4.52	2	5	6	18	1.573	0.764	0.342	1.206	30.55	4.52	8	18	1306	149.6	6.39

Q71RC2	La-related protein 4 OS=Homo sapiens GN=LARP4 PE=1 SV=3 - [LARP4_HUMAN]	14.36	1	8	8	16	0.796	0.689	0.925	1.206	36.47	14.36	10	16	724	80.5	6.61
P35232	Prohibitin OS=Homo sapiens GN=PHB PE=1 SV=1 - [PHB_HUMAN]	47.43	1	15	15	129	1.105	1.133	0.880	1.207	287.74	47.43	28	129	272	29.8	5.76
Q86X95	Corepressor interacting with RBPJ 1 OS=Homo sapiens GN=CIR1 PE=1 SV=1 - [CIR1_HUMAN]	2.00	1	1	1	1	0.885	0.910	1.174	1.207	0.00	2.00	1	1	450	52.3	9.86
P48634	Protein PRRC2A OS=Homo sapiens GN=PRRC2A PE=1 SV=3 - [PRC2A_HUMAN]	15.30	1	22	26	71	1.080	1.404	1.087	1.208	175.06	15.30	36	71	2157	228.7	9.45
P42167	Lamina-associated polypeptide 2, isoforms beta/gamma OS=Homo sapiens GN=TMPO PE=1 SV=2 - [LAP2B_HUMAN]	39.65	3	7	15	81	1.304	1.625	0.942	1.208	193.68	39.65	24	81	454	50.6	9.38
P57679	Ellis-van Creveld syndrome protein OS=Homo sapiens GN=EVC PE=1 SV=1 - [EVC_HUMAN]	3.53	1	2	2	5	2.472	1.471	1.375	1.208	10.13	3.53	2	5	992	111.9	6.73
Q9Y6M5	Zinc transporter 1 OS=Homo sapiens GN=SLC30A1 PE=1 SV=3 - [ZNT1_HUMAN]	10.45	1	5	5	8	1.179	0.842	0.767	1.208	22.49	10.45	8	8	507	55.3	6.48
Q01581	Hydroxymethylglutaryl-CoA synthase, cytoplasmic OS=Homo sapiens GN=HMGCS1 PE=1 SV=2 - [HMCS1_HUMAN]	4.04	1	2	2	4	1.244	0.554	1.265	1.208	9.88	4.04	3	4	520	57.3	5.41
P09132	Signal recognition particle 19 kDa protein OS=Homo sapiens GN=SRP19 PE=1 SV=3 - [SRP19_HUMAN]	20.14	1	3	3	16	0.775	0.795	1.010	1.208	47.21	20.14	5	16	144	16.1	9.85

Q9UIS9	Methyl-CpG-binding domain protein 1 OS=Homo sapiens GN=MBD1 PE=1 SV=2 - [MBD1_HUMAN]	7.60	1	2	2	2	0.921	1.509	1.345	1.208	3.27	7.60	2	2	605	66.6	9.04
O60930	Ribonuclease H1 OS=Homo sapiens GN=RNASEH1 PE=1 SV=2 - [RNH1_HUMAN]	6.99	1	1	2	4	0.792	0.820	1.078	1.208	4.16	6.99	3	4	286	32.0	9.16
Q9UHI6	Probable ATP-dependent RNA helicase DDX20 OS=Homo sapiens GN=DDX20 PE=1 SV=2 - [DDX20_HUMAN	7.65	1	5	5	11	0.950	0.779	0.834	1.209	16.82	7.65	9	11	824	92.2	6.95
Q6P4F2	Adrenodoxin-like protein, mitochondrial OS=Homo sapiens GN=FDX1L PE=1 SV=1 - [ADXL_HUMAN]	6.01	1	1	1	2	1.191	1.016	1.425	1.209	2.22	6.01	2	2	183	19.5	5.38
P07099	Epoxide hydrolase 1 OS=Homo sapiens GN=EPHX1 PE=1 SV=1 - [HYEP_HUMAN]	40.44	1	21	21	149	1.282	0.439	0.533	1.209	480.06	40.44	36	149	455	52.9	7.25
Q13546	Receptor-interacting serine/threonine-protein kinase 1 OS=Homo sapiens GN=RIPK1 PE=1 SV=3 - [RIPK1_HUMAN	9.54	1	5	5	12	1.134	1.073	1.191	1.210	23.77	9.54	7	12	671	75.9	6.33
O14776	Transcription elongation regulator 1 OS=Homo sapiens GN=TCERG1 PE=1 SV=2 - [TCRG1_HUMAN]	20.58	2	26	27	68	1.189	1.204	0.931	1.210	142.65	20.58	42	68	1098	123.8	8.65
Q8NBM4	Ubiquitin-associated domain-containing protein 2 OS=Homo sapiens GN=UBAC2 PE=2 SV=1 - [UBAC2_HUMAN]	2.33	1	1	1	2	0.741	0.756	0.670	1.210	1.97	2.33	2	2	344	38.9	9.01
O00151	PDZ and LIM domain protein 1 OS=Homo sapiens GN=PDLIM1 PE=1 SV=4 - [PDLI1_HUMAN]	73.56	1	20	21	351	2.426	0.730	1.352	1.210	928.27	73.56	36	351	329	36.0	7.02

O43172	U4/U6 small nudear ribonudeoprotein Prp4 OS=Homo sapiens GN=PRPF4 PE=1 SV=2 - [PRP4_HUMAN]	23.56	1	9	9	28	0.930	1.066	0.874	1.210	86.05	23.56	15	28	522	58.4	7.42
Q1KMD3	Heterogeneous nudear ribonudeoprotein U-like protein 2 OS=Homo sapiens GN=HNRNPUL2 PE=1 SV=1 - [HNRL2_HUMAN	40.03	1	31	31	95	1.403	1.501	1.141	1.210	236.57	40.03	46	95	747	85.1	4.91
Q92945	Far upstream element-binding protein 2 OS=Homo sapiens GN=KHSRP PE=1 SV=4 - [FUBP2_HUMAN]	46.13	1	24	28	143	1.160	1.301	1.167	1.210	360.80	46.13	45	143	711	73.1	7.30
P20962	Parathymosin OS=Homo sapiens GN=PTMS PE=1 SV=2 - [PTMS_HUMAN]	22.55	1	2	2	50	1.434	1.811	1.189	1.210	152.54	22.55	3	50	102	11.5	4.16
Q6PI48	Aspartate--tRNA ligase, mitochondrial OS=Homo sapiens GN=DARS2 PE=1 SV=1 - [SYDM_HUMAN]	11.32	1	7	8	17	0.982	1.722	1.073	1.211	34.90	11.32	11	17	645	73.5	8.02
Q9ULX6	A-kinase anchor protein 8-like OS=Homo sapiens GN=AKAP8L PE=1 SV=3 - [AKP8L_HUMAN]	11.15	1	6	6	9	1.188	0.961	1.273	1.211	22.59	11.15	7	9	646	71.6	5.07
P17480	Nudeolar transcription factor 1 OS=Homo sapiens GN=UBTF PE=1 SV=1 - [UBF1_HUMAN]	33.90	1	28	28	112	0.942	1.211	0.860	1.211	232.95	33.90	42	112	764	89.4	5.81
P18859	ATP synthase-coupling factor 6, mitochondrial OS=Homo sapiens GN=ATP5J PE=1 SV=1 - [ATP5J_HUMAN	55.56	1	7	7	62	1.051	1.040	0.805	1.211	236.25	55.56	10	62	108	12.6	9.52
Q8IYM9	E3 ubiquitin-protein ligase TRIM22 OS=Homo sapiens GN=TRIM22 PE=1 SV=1 - [TRI22_HUMAN]	14.06	1	6	6	26	1.117	0.760	0.993	1.212	43.06	14.06	9	26	498	56.9	7.72

Q14690	Protein RRP5 homolog OS=Homo sapiens GN=PDCD11 PE=1 SV=3 - [RRP5_HUMAN]	14.64	1	24	24	54	0.963	0.831	0.771	1.212	108.75	14.64	33	54	1871	208.6	8.87
Q9HAK2	Transcription factor COE2 OS=Homo sapiens GN=EBF2 PE=1 SV=4 - [COE2_HUMAN]	3.65	1	1	1	3	2.630	2.125	1.649	1.212	0.00	3.65	1	3	575	62.6	9.06
Q5TDH0	Protein DDI1 homolog 2 OS=Homo sapiens GN=DDI2 PE=1 SV=1 - [DDI2_HUMAN]	23.31	1	7	8	26	1.065	0.939	0.936	1.212	60.53	23.31	12	26	399	44.5	5.05
Q9NQZ5	StAR-related lipid transfer protein 7, mitochondrial OS=Homo sapiens GN=STARD7 PE=1 SV=2 - [STAR7_HUMAN]	4.05	1	2	2	3	0.814	0.744	1.413	1.212	7.79	4.05	2	3	370	43.1	8.95
O75150	E3 ubiquitin-protein ligase BRE1B OS=Homo sapiens GN=RNF40 PE=1 SV=4 - [BRE1B_HUMAN]	17.68	2	13	17	40	1.022	1.256	0.989	1.212	97.39	17.68	22	40	1001	113.6	6.23
Q9BSV6	tRNA-splicing endonuclease subunit Sen34 OS=Homo sapiens GN=TSEN34 PE=1 SV=1 - [SEN34_HUMAN]	2.26	1	1	1	2	1.255	0.958	1.148	1.212	3.13	2.26	1	2	310	33.6	8.43
Q6VAB6	Kinase suppressor of Ras 2 OS=Homo sapiens GN=KSR2 PE=1 SV=2 - [KSR2_HUMAN]	1.37	1	1	1	1	1.015	0.969	0.996	1.213	0.00	1.37	1	1	950	107.6	8.69
O76024	Wolframin OS=Homo sapiens GN=WFS1 PE=1 SV=2 - [WFS1_HUMAN]	20.56	1	17	17	40	0.916	0.850	0.948	1.213	96.73	20.56	22	40	890	100.2	8.05
O43143	Putative pre-mRNA-splicing factor ATP-dependent RNA helicase DHX15 OS=Homo sapiens GN=DHX15 PE=1 SV=2 - [DHX15_HUMAN	31.57	2	23	24	76	1.134	1.143	0.941	1.213	196.41	31.57	37	76	795	90.9	7.46

Q9NR77	Peroxisomal membrane protein 2 OS=Homo sapiens GN=PXMP2 PE=1 SV=3 - [PXMP2_HUMAN]	5.13	1	1	1	2	1.070	0.878	0.922	1.213	4.46	5.13	2	2	195	22.2	10.58
Q53GL7	Poly [ADP-ribose] polymerase 10 OS=Homo sapiens GN=PARP10 PE=1 SV=2 - [PAR10_HUMAN]	9.66	1	6	6	28	1.797	1.226	1.126	1.213	58.09	9.66	11	28	1025	109.9	4.97
Q9H1C3	Glycosyltransferase 8 domain-containing protein 2 OS=Homo sapiens GN=GLT8D2 PE=2 SV=1 - [GL8D2_HUMAN]	4.87	1	2	2	3	1.050	1.008	0.841	1.213	9.83	4.87	2	3	349	40.0	7.06
Q9BVM4	Gamma-glutamylaminecyclotransferase OS=Homo sapiens GN=GGACT PE=1 SV=2 - [GGACT_HUMAN]	12.42	1	1	2	6	0.899	0.996	0.957	1.213	13.58	12.42	3	6	153	17.3	6.87
O15239	NADH dehydrogenase [ubiquinone] 1 alpha subcomplex subunit 1 OS=Homo sapiens GN=NDUFA1 PE=1 SV=1 - [NDUA1_HUMAN]	8.57	1	1	1	2	0.665	1.214	0.727	1.213	3.86	8.57	1	2	70	8.1	8.84
Q2KJY2	Kinesin-like protein KIF26B OS=Homo sapiens GN=KIF26B PE=2 SV=1 - [KI26B_HUMAN]	2.85	1	3	3	4	3.077	1.014	0.947	1.213	6.93	2.85	4	4	2108	223.7	8.44
Q96BP3	Peptidylprolyl isomerase domain and WD repeat-containing protein 1 OS=Homo sapiens GN=PPWD1 PE=1 SV=1 - [PPWD1_HUMAN]	2.79	1	2	2	5	1.048	1.613	1.109	1.213	13.09	2.79	3	5	646	73.5	7.15
Q9BXW7	Cat eye syndrome critical region protein 5 OS=Homo sapiens GN=CECR5 PE=1 SV=1 - [CECR5_HUMAN]	15.37	1	6	6	17	0.939	1.181	0.927	1.213	51.84	15.37	8	17	423	46.3	8.13

Q86UQ4	ATP-binding cassette sub-family A member 13 OS=Homo sapiens GN=ABCA13 PE=2 SV=3 - [ABCAD_HUMAN]	0.97	4	2	5	11	0.904	1.054	0.934	1.214	14.22	0.97	5	11	5058	575.8	6.46
Q8N3U4	Cohesin subunit SA-2 OS=Homo sapiens GN=STAG2 PE=1 SV=3 - [STAG2_HUMAN]	0.73	1	1	1	1	0.988	1.217	0.800	1.214	1.78	0.73	1	1	1231	141.2	5.43
Q8N3E9	1-phosphatidylinositol 4,5-bisphosphate phosphodiesterase delta-3 OS=Homo sapiens GN=PLCD3 PE=1 SV=3 - [PLCD3_HUMAN]	14.45	2	8	8	15	1.786	0.807	1.761	1.214	38.84	14.45	12	15	789	89.2	6.98
P07305	Histone H1.0 OS=Homo sapiens GN=H1F0 PE=1 SV=3 - [H10_HUMAN]	26.80	1	7	7	22	1.315	4.396	1.749	1.214	44.96	26.80	9	22	194	20.9	10.84
Q9NX63	MICOS complex subunit MIC19 OS=Homo sapiens GN=CHCHD3 PE=1 SV=1 - [MIC19_HUMAN]	41.85	1	12	12	70	1.013	0.972	0.729	1.214	198.82	41.85	19	70	227	26.1	8.28
Q13428	Treacle protein OS=Homo sapiens GN=TCOF1 PE=1 SV=3 - [TCOF_HUMAN]	27.76	1	36	37	145	0.881	1.328	1.097	1.214	375.55	27.76	58	145	1488	152.0	9.04
Q9NPL8	Complex I assembly factor TIMMDC1, mitochondrial OS=Homo sapiens GN=TIMMDC1 PE=1 SV=2 - [TIDC1_HUMAN]	15.79	1	4	4	14	0.949	0.656	0.898	1.214	35.58	15.79	7	14	285	32.2	8.50
O00533	Neural cell adhesion molecule L1-like protein OS=Homo sapiens GN=CHL1 PE=1 SV=4 - [NCHL1_HUMAN]	0.99	1	1	1	2	3.102	0.839	2.384	1.214	2.44	0.99	1	2	1208	135.0	5.76
P51610	Host cell factor 1 OS=Homo sapiens GN=HCFC1 PE=1 SV=2 - [HCFC1_HUMAN]	6.58	1	12	12	36	1.248	1.371	1.036	1.214	73.70	6.58	20	36	2035	208.6	7.46

Q7Z7M9	Polypeptide N-acetylgalactosaminyltransferase 5 OS=Homo sapiens GN=GALNT5 PE=1 SV=1 - [GALT5_HUMAN]	8.09	1	7	8	23	0.979	0.530	0.574	1.214	40.89	8.09	13	23	940	106.2	9.47
P49756	RNA-binding protein 25 OS=Homo sapiens GN=RBM25 PE=1 SV=3 - [RBM25_HUMAN]	21.71	1	13	14	43	1.163	1.264	1.025	1.214	96.87	21.71	21	43	843	100.1	6.32
Q9BS26	Endoplasmic reticulum resident protein 44 OS=Homo sapiens GN=ERP44 PE=1 SV=1 - [ERP44_HUMAN	54.68	1	19	21	175	0.940	0.746	0.649	1.215	450.12	54.68	32	175	406	46.9	5.26
Q92859	Neogenin OS=Homo sapiens GN=NEO1 PE=1 SV=2 - [NEO1_HUMAN]	6.78	1	6	6	19	1.585	0.760	1.143	1.215	47.29	6.78	8	19	1461	159.9	6.54
P29084	Transcription initiation factor IIE subunit beta OS=Homo sapiens GN=GTF2E2 PE=1 SV=1 - [T2EB_HUMAN]	10.31	1	3	3	6	1.096	0.891	0.814	1.215	15.30	10.31	5	6	291	33.0	9.66
Q7Z417	Nuclear fragile X mental retardation-interacting protein 2 OS=Homo sapiens GN=NUFIP2 PE=1 SV=1 - [NUFP2_HUMAN]	32.23	1	20	20	66	0.985	0.875	0.927	1.215	150.04	32.23	29	66	695	76.1	8.70
Q8N7H5	RNA polymerase II-associated factor 1 homolog OS=Homo sapiens GN=PAF1 PE=1 SV=2 - [PAF1_HUMAN]	7.53	1	4	4	12	0.956	1.017	1.011	1.215	29.37	7.53	6	12	531	59.9	4.63
Q9BWH2	FUN14 domain-containing protein 2 OS=Homo sapiens GN=FUNDC2 PE=1 SV=2 - [FUND2_HUMAN]	17.99	1	3	3	12	0.765	0.540	0.469	1.216	34.05	17.99	5	12	189	20.7	9.73

P63096	Guanine nucleotide-binding protein G(i) subunit alpha-1 OS=Homo sapiens GN=GNAI1 PE=1 SV=2 - [GNAI1_HUMAN]	40.11	4	5	14	99	2.283	0.780	0.856	1.216	248.26	40.11	26	99	354	40.3	5.97
P19338	Nucleolin OS=Homo sapiens GN=NCL PE=1 SV=3 - [NUCL_HUMAN]	48.03	1	47	48	510	0.910	1.132	0.931	1.217	1268.29	48.03	78	510	710	76.6	4.70
Q75T13	GPI inositol-deacylase OS=Homo sapiens GN=PGAP1 PE=1 SV=1 - [PGAP1_HUMAN]	3.15	1	2	2	4	1.437	1.568	2.194	1.217	2.07	3.15	2	4	922	105.3	9.01
Q99633	Pre-mRNA-splicing factor 18 OS=Homo sapiens GN=PRPF18 PE=1 SV=1 - [PRP18_HUMAN]	2.34	1	1	1	1	0.939	1.031	0.836	1.217	2.78	2.34	1	1	342	39.8	8.15
Q99615	DnaJ homolog subfamily C member 7 OS=Homo sapiens GN=DNAJC7 PE=1 SV=2 - [DNJC7_HUMAN]	19.43	1	10	10	29	0.963	0.879	1.050	1.217	63.45	19.43	16	29	494	56.4	6.96
O14976	Cyclin-G-associated kinase OS=Homo sapiens GN=GAK PE=1 SV=2 - [GAK_HUMAN]	10.37	1	14	14	56	0.982	1.016	1.006	1.217	122.25	10.37	21	56	1311	143.1	5.73
Q9NVK5	FGFR1 oncogene partner 2 OS=Homo sapiens GN=FGFR1OP2 PE=2 SV=1 - [FGOP2_HUMAN]	6.32	1	2	2	6	1.207	0.830	1.418	1.217	15.76	6.32	2	6	253	29.4	6.00
P50750	Cyclin-dependent kinase 9 OS=Homo sapiens GN=CDK9 PE=1 SV=3 - [CDK9_HUMAN]	22.85	9	6	9	32	1.117	1.053	0.905	1.217	74.12	22.85	12	32	372	42.8	8.79
Q8N8N7	Prostaglandin reductase 2 OS=Homo sapiens GN=PTGR2 PE=1 SV=1 - [PTGR2_HUMAN]	10.54	1	3	3	6	0.959	0.939	0.875	1.218	13.40	10.54	4	6	351	38.5	5.41

Q13485	Mothers against decapentaplegic homolog 4 OS=Homo sapiens GN=SMAD4 PE=1 SV=1 - [SMAD4_HUMA	8.51	1	4	4	24	1.062	1.080	1.054	1.218	38.82	8.51	7	24	552	60.4	6.99
Q9NTN3	UDP-glucuronic acid/UDP-N-acetylgalactosamine transporter OS=Homo sapiens GN=SLC35D1 PE=1 SV=1 - [S35D1_HUMAN]	2.25	1	1	1	3	2.899	0.664	0.604	1.218	1.89	2.25	2	3	355	39.2	8.97
Q9NXW2	DnaJ homolog subfamily B member 12 OS=Homo sapiens GN=DNAJB12 PE=1 SV=4 - [DJB12_HUMAN]	26.40	1	8	9	19	1.186	0.772	0.835	1.218	55.01	26.40	13	19	375	41.8	8.53
Q6ULP2	Aftiphilin OS=Homo sapiens GN=AFTPH PE=1 SV=2 - [AFTIN_HUMAN]	4.48	1	4	4	12	1.048	1.216	1.135	1.218	32.04	4.48	6	12	937	102.1	4.54
Q96NL6	Sodium channel and clathrin linker 1 OS=Homo sapiens GN=SCLT1 PE=1 SV=2 - [SCLT1_HUMAN]	4.65	1	2	3	3	0.958	1.272	1.515	1.218	3.03	4.65	3	3	688	80.9	6.07
Q7Z2K8	G protein-regulated inducer of neurite outgrowth 1 OS=Homo sapiens GN=GPRIN1 PE=2 SV=2 - [GRIN1_HUMAN]	3.67	1	3	3	9	1.239	0.729	1.263	1.218	20.54	3.67	3	9	1008	102.3	8.06
Q13190	Syntaxin-5 OS=Homo sapiens GN=STX5 PE=1 SV=2 - [STX5_HUMAN]	23.38	1	7	7	22	1.133	1.204	1.113	1.218	59.52	23.38	14	22	355	39.6	9.16
Q16637	Survival motor neuron protein OS=Homo sapiens GN=SMN1 PE=1 SV=1 - [SMN_HUMAN]	6.80	1	3	3	7	0.907	1.022	0.900	1.219	2.25	6.80	5	7	294	31.8	6.55
Q9BTE6	Alanyl-tRNA editing protein Aarsd1 OS=Homo sapiens GN=AARSD1 PE=1 SV=2 - [AASD1_HUMAN]	18.93	1	3	5	9	0.916	1.216	1.018	1.219	20.11	18.93	7	9	412	45.5	6.42

Q92541	RNA polymerase-associated protein RTF1 homolog OS=Homo sapiens GN=RTF1 PE=1 SV=4 - [RTF1_HUMAN]	19.86	1	13	14	32	1.140	1.154	1.046	1.219	72.57	19.86	18	32	710	80.3	8.15
Q969Q5	Ras-related protein Rab-24 OS=Homo sapiens GN=RAB24 PE=1 SV=1 - [RAB24_HUMAN]	16.26	1	3	3	17	1.157	0.999	1.075	1.219	47.18	16.26	5	17	203	23.1	6.23
Q8N201	Integrator complex subunit 1 OS=Homo sapiens GN=INTS1 PE=1 SV=2 - [INT1_HUMAN]	4.11	1	6	6	24	1.082	1.398	1.107	1.219	52.88	4.11	12	24	2190	244.1	6.13
P15692	Vascular endothelial growth factor A OS=Homo sapiens GN=VEGFA PE=1 SV=2 - [VEGFA_HUMAN]	3.02	1	1	1	2	0.646	0.465	0.439	1.219	5.68	3.02	1	2	232	27.0	8.91
P32189	Glycerol kinase OS=Homo sapiens GN=GK PE=1 SV=3 - [GLPK_HUMAN]	19.50	3	11	11	37	1.198	1.153	0.364	1.219	97.37	19.50	17	37	559	61.2	6.54
Q9NVS2	28S ribosomal protein S18a, mitochondrial OS=Homo sapiens GN=MRPS18A PE=1 SV=1 - [RT18A_HUMAN]	8.67	1	2	2	4	0.953	1.316	1.202	1.219	7.45	8.67	3	4	196	22.2	10.33
Q96K80	Zinc finger CCCH domain-containing protein 10 OS=Homo sapiens GN=ZC3H10 PE=1 SV=1 - [ZC3HA_HUMAN]	9.22	1	2	3	6	0.773	1.017	0.941	1.219	11.57	9.22	3	6	434	46.0	7.64
Q16650	T-box brain protein 1 OS=Homo sapiens GN=TBR1 PE=1 SV=1 - [TBR1_HUMAN]	2.20	1	1	1	1	2.611	0.795	1.459	1.219	2.85	2.20	1	1	682	74.0	7.33
Q9BYD6	39S ribosomal protein L1, mitochondrial OS=Homo sapiens GN=MRPL1 PE=1 SV=2 - [RM01_HUMAN]	20.00	1	7	7	14	0.969	1.480	1.011	1.220	31.18	20.00	9	14	325	36.9	8.78

O94913	Pre-mRNA deavage complex 2 protein Pcf11 OS=Homo sapiens GN=PCF11 PE=1 SV=3 - [PCF11_HUMAN]	2.38	1	3	4	6	1.314	0.724	0.945	1.220	11.31	2.38	5	6	1555	172.9	8.48
Q9BPU6	Dihydropyrimidinase-related protein 5 OS=Homo sapiens GN=DPYSL5 PE=1 SV=1 - [DPYL5_HUMAN]	1.24	1	1	1	3	1.307	2.038	0.853	1.220	6.27	1.24	2	3	564	61.4	7.20
Q00653	Nudear factor NF-kappa-B p100 subunit OS=Homo sapiens GN=NFKB2 PE=1 SV=4 - [NFKB2_HUMAN]	20.78	1	15	16	36	1.458	1.042	1.472	1.220	91.99	20.78	22	36	900	96.7	6.25
O75449	Katanin p60 ATPase-containing subunit A1 OS=Homo sapiens GN=KATNA1 PE=1 SV=1 - [KTNA1_HUMAN]	8.76	1	4	4	5	0.846	0.963	0.951	1.220	12.21	8.76	5	5	491	55.9	6.90
Q96M93	Adenosine deaminase domain-containing protein 1 OS=Homo sapiens GN=ADAD1 PE=2 SV=1 - [ADAD1_HUMAN]	1.56	1	1	1	1	2.507	2.453	2.488	1.220	0.00	1.56	1	1	576	64.4	9.01
Q8WY91	THAP domain-containing protein 4 OS=Homo sapiens GN=THAP4 PE=1 SV=2 - [THAP4_HUMAN]	1.04	1	1	1	1	1.136	1.753	0.964	1.220	0.00	1.04	1	1	577	62.9	9.28
Q9Y3C1	Nudeolar protein 16 OS=Homo sapiens GN=NOP16 PE=1 SV=2 - [NOP16_HUMAN]	32.58	1	6	6	28	0.610	0.817	0.795	1.220	67.59	32.58	11	28	178	21.2	9.94
Q9UHN6	Transmembrane protein 2 OS=Homo sapiens GN=TMEM2 PE=1 SV=1 - [TMEM2_HUMAN]	5.13	1	7	7	12	0.872	0.690	1.098	1.220	21.62	5.13	11	12	1383	154.3	8.15

O75643	U5 small nudear ribonudeoprotein 200 kDa helicase OS=Homo sapiens GN=SNRNP200 PE=1 SV=2 - [U520_HUMAN]	25.66	1	49	49	143	1.134	1.072	0.929	1.220	341.84	25.66	75	143	2136	244.4	6.06
Q6PJG6	BRCA1-associated ATM activator 1 OS=Homo sapiens GN=BRAT1 PE=1 SV=2 - [BRAT1_HUMAN]	0.97	1	1	1	1	0.798	1.272	1.377	1.220	2.60	0.97	1	1	821	88.1	5.27
Q8ND90	Paraneoplastic antigen Ma1 OS=Homo sapiens GN=PNMA1 PE=1 SV=2 - [PNMA1_HUMAN]	7.08	1	2	2	4	1.768	1.601	1.938	1.220	4.56	7.08	4	4	353	39.7	4.83
P13667	Protein disulfide-isomerase A4 OS=Homo sapiens GN=PDIA4 PE=1 SV=2 - [PDIA4_HUMAN	62.33	2	47	49	484	0.933	1.153	1.042	1.220	1226.84	62.33	79	484	645	72.9	5.07
P31943	Heterogeneous nudear ribonudeoprotein H OS=Homo sapiens GN=HNRNPH1 PE=1 SV=4 - [HNRH1_HUMAN	52.34	1	7	16	196	1.099	1.416	1.132	1.221	591.28	52.34	27	196	449	49.2	6.30
Q5UCC4	ER membrane protein complex subunit 10 OS=Homo sapiens GN=EMC10 PE=1 SV=1 - [EMC10_HUMA	23.66	1	5	5	20	1.044	1.128	1.126	1.221	52.06	23.66	9	20	262	27.3	6.13
Q13523	Serine/threonine-protein kinase PRP4 homolog OS=Homo sapiens GN=PRPF4B PE=1 SV=3 - [PRP4B_HUMAN	12.21	1	10	11	26	0.981	1.301	0.996	1.221	66.24	12.21	16	26	1007	116.9	10.26
Q14839	Chromodomain-helicase-DNA-binding protein 4 OS=Homo sapiens GN=CHD4 PE=1 SV=2 - [CHD4_HUMAN]	10.20	1	12	16	49	0.987	1.172	0.802	1.221	108.08	10.20	26	49	1912	217.9	5.86

P11166	Solute carrier family 2, facilitated glucose transporter member 1 OS=Homo sapiens GN=SLC2A1 PE=1 SV=2 - [GTR1_HUMAN]	10.77	1	6	6	26	2.125	0.372	1.069	1.222	61.03	10.77	9	26	492	54.0	8.72
P52298	Nuclear cap-binding protein subunit 2 OS=Homo sapiens GN=NCBP2 PE=1 SV=1 - [NCBP2_HUMAN]	12.18	1	2	2	5	1.166	0.998	0.770	1.222	9.44	12.18	4	5	156	18.0	8.21
Q8IYG6	Leucine-rich repeat-containing protein 56 OS=Homo sapiens GN=LRRC56 PE=2 SV=1 - [LRC56_HUMAN]	1.11	2	1	1	1	0.570	0.761	0.627	1.222	1.85	1.11	1	1	542	58.7	7.90
Q9NPF4	Probable tRNA N6-adenosine threonylcarbamoyltransferase OS=Homo sapiens GN=OSGEP PE=1 SV=1 - [OSGEP_HUMAN]	13.13	1	4	4	11	1.136	1.327	1.022	1.222	18.62	13.13	7	11	335	36.4	6.35
Q92759	General transcription factor IIH subunit 4 OS=Homo sapiens GN=GTF2H4 PE=2 SV=1 - [TF2H4_HUMAN]	1.52	1	1	1	3	0.979	0.984	0.784	1.222	6.76	1.52	2	3	462	52.2	9.04
A8MUK1	Ubiquitin carboxyl-terminal hydrolase 17-like protein 5 OS=Homo sapiens GN=USP17L5 PE=3 SV=2 - [U17L5_HUMAN]	4.34	15	2	2	19	1.267	0.912	1.212	1.222	36.93	4.34	3	19	530	59.6	8.07
O14802	DNA-directed RNA polymerase III subunit RPC1 OS=Homo sapiens GN=POLR3A PE=1 SV=2 - [RPC1_HUMAN]	4.46	1	5	5	6	0.893	1.122	1.036	1.222	15.64	4.46	6	6	1390	155.5	8.48

Q9BQ95	Evolutionarily conserved signaling intermediate in Toll pathway, mitochondrial OS=Homo sapiens GN=ECSIT PE=1 SV=1 - [ECSIT_HUMAN]	8.35	1	2	2	9	1.210	1.023	0.658	1.223	17.45	8.35	3	9	431	49.1	6.29
Q08AD1	Calmodulin-regulated spectrin-associated protein 2 OS=Homo sapiens GN=CAMSAP2 PE=1 SV=3 - [CAMP2_HUMAN]	10.54	1	12	13	23	1.007	0.866	1.201	1.223	40.15	10.54	18	23	1489	168.0	6.80
Q8TCD1	UPF0729 protein C18orf32 OS=Homo sapiens GN=C18orf32 PE=2 SV=1 - [CR032_HUMAN]	30.26	1	5	5	25	1.578	1.202	0.846	1.223	52.09	30.26	7	25	76	8.7	9.13
P30049	ATP synthase subunit delta, mitochondrial OS=Homo sapiens GN=ATP5D PE=1 SV=2 - [ATPD_HUMAN]	17.26	1	2	3	89	1.064	1.054	0.821	1.223	301.16	17.26	5	89	168	17.5	5.49
P98179	Putative RNA-binding protein 3 OS=Homo sapiens GN=RBM3 PE=1 SV=1 - [RBM3_HUMAN]	43.95	1	4	4	31	1.465	1.390	1.457	1.224	67.54	43.95	5	31	157	17.2	8.91
Q9H7F0	Probable cation-transporting ATPase 13A3 OS=Homo sapiens GN=ATP13A3 PE=1 SV=4 - [AT133_HUMAN	0.90	1	1	1	4	1.210	0.586	0.714	1.224	7.16	0.90	2	4	1226	138.0	6.64
Q9H0U6	39S ribosomal protein L18, mitochondrial OS=Homo sapiens GN=MRPL18 PE=1 SV=1 - [RM18_HUMAN]	17.78	1	3	3	8	0.833	1.001	0.885	1.224	10.59	17.78	5	8	180	20.6	9.54
Q6NXT4	Zinc transporter 6 OS=Homo sapiens GN=SLC30A6 PE=1 SV=2 - [ZNT6_HUMAN]	11.71	1	3	3	6	0.848	0.748	0.986	1.224	13.73	11.71	5	6	461	51.1	9.16

Q8IV50	LysM and putative peptidoglycan-binding domain-containing protein 2 OS=Homo sapiens GN=LYSMD2 PE=1 SV=1 - [LYSM2_HUMAN]	7.44	1	1	2	3	0.988	0.889	0.945	1.224	1.75	7.44	2	3	215	23.4	5.35
P20290	Transcription factor BTF3 OS=Homo sapiens GN=BTF3 PE=1 SV=1 - [BTF3_HUMAN]	36.89	1	6	6	22	0.899	1.084	1.423	1.224	61.32	36.89	11	22	206	22.2	9.38
Q96RY7	Intraflagellar transport protein 140 homolog OS=Homo sapiens GN=IFT140 PE=1 SV=1 - [IF140_HUMAN]	3.49	1	3	3	5	1.135	1.116	0.939	1.224	9.15	3.49	3	5	1462	165.1	6.04
O95104	Splicing factor, arginine/serine-rich 15 OS=Homo sapiens GN=SCAF4 PE=1 SV=3 - [SFR15_HUMAN]	3.40	1	3	3	7	1.199	1.926	1.301	1.224	15.21	3.40	4	7	1147	125.8	9.55
P63279	SUMO-conjugating enzyme UBC9 OS=Homo sapiens GN=UBE2I PE=1 SV=1 - [UBC9_HUMAN]	34.81	1	7	7	33	1.225	1.509	0.957	1.225	67.44	34.81	11	33	158	18.0	8.66
P30040	Endoplasmic reticulum resident protein 29 OS=Homo sapiens GN=ERP29 PE=1 SV=4 - [ERP29_HUMAN	54.79	1	14	14	107	1.046	1.096	0.808	1.225	353.54	54.79	20	107	261	29.0	7.31
Q9P2I0	Cleavage and polyadenylation specificity factor subunit 2 OS=Homo sapiens GN=CPSF2 PE=1 SV=2 - [CPSF2_HUMAN]	3.32	1	2	3	7	0.934	0.775	0.989	1.225	8.53	3.32	3	7	782	88.4	5.11
Q99543	DnaJ homolog subfamily C member 2 OS=Homo sapiens GN=DNAJC2 PE=1 SV=4 - [DNJC2_HUMAN]	14.98	1	11	11	28	0.924	1.146	1.058	1.225	68.18	14.98	19	28	621	72.0	8.70

Q6P087	RNA pseudouridylate synthase domain-containing protein 3 OS=Homo sapiens GN=RPUSD3 PE=1 SV=3 - [RUSD3_HUMAN]	2.28	1	1	1	4	0.624	0.947	1.004	1.225	6.86	2.28	2	4	351	38.4	10.32
Q99988	Growth/differentiation factor 15 OS=Homo sapiens GN=GDF15 PE=1 SV=3 - [GDF15_HUMAN]	15.91	1	2	2	3	1.457	0.783	1.591	1.226	0.00	15.91	2	3	308	34.1	9.66
P26440	Isovaleryl-CoA dehydrogenase, mitochondrial OS=Homo sapiens GN=IVD PE=1 SV=1 - [IVD_HUMAN]	26.00	1	12	12	62	1.050	1.241	1.076	1.226	127.14	26.00	21	62	423	46.3	8.19
Q69YN4	Protein virilizer homolog OS=Homo sapiens GN=KIAA1429 PE=1 SV=2 - [VIR_HUMAN]	1.21	1	2	2	3	1.108	1.075	0.769	1.226	8.53	1.21	3	3	1812	201.9	5.01
P22695	Cytochrome b-c1 complex subunit 2, mitochondrial OS=Homo sapiens GN=UQCRC2 PE=1 SV=3 - [QCR2_HUMAN]	49.45	1	18	18	69	1.052	1.138	0.782	1.226	203.42	49.45	31	69	453	48.4	8.63
P06576	ATP synthase subunit beta, mitochondrial OS=Homo sapiens GN=ATP5B PE=1 SV=3 - [ATPB_HUMAN]	69.00	1	27	27	707	1.201	1.152	0.952	1.226	1731.47	69.00	44	707	529	56.5	5.40
P16455	Methylated-DNA--protein-cysteine methyltransferase OS=Homo sapiens GN=MGMT PE=1 SV=1 - [MGMT_HUMAN]	15.94	1	3	3	12	1.613	1.008	0.816	1.227	35.32	15.94	5	12	207	21.6	8.10
Q96MK2	Protein FAM65C OS=Homo sapiens GN=FAM65C PE=2 SV=4 - [FA65C_HUMAN]	3.81	1	3	3	8	1.462	0.798	1.043	1.227	19.22	3.81	4	8	946	105.2	7.43

Q07955	Serine/arginine-rich splicing factor 1 OS=Homo sapiens GN=SRSF1 PE=1 SV=2 - [SRSF1_HUMAN]	42.34	1	14	14	103	1.063	1.268	1.031	1.227	241.36	42.34	24	103	248	27.7	10.36
P08574	Cytochrome c1, heme protein, mitochondrial OS=Homo sapiens GN=CYC1 PE=1 SV=3 - [CY1_HUMAN]	18.77	1	5	5	18	0.979	1.007	0.646	1.227	48.97	18.77	7	18	325	35.4	9.00
Q96MP8	BTB/POZ domain-containing protein KCTD7 OS=Homo sapiens GN=KCTD7 PE=1 SV=1 - [KCTD7_HUMAN]	3.11	1	1	1	2	1.315	1.288	1.346	1.228	3.56	3.11	2	2	289	33.1	5.91
O43290	U4/U6.U5 tri-snRNP-associated protein 1 OS=Homo sapiens GN=SART1 PE=1 SV=1 - [SNUT1_HUMAN]	19.88	2	13	13	54	1.066	1.282	0.941	1.228	146.33	19.88	21	54	800	90.2	6.13
Q9BWJ5	Splicing factor 3B subunit 5 OS=Homo sapiens GN=SF3B5 PE=1 SV=1 - [SF3B5_HUMAN]	32.56	1	3	3	8	1.248	1.419	0.978	1.228	16.32	32.56	5	8	86	10.1	6.35
Q9BYD1	39S ribosomal protein L13, mitochondrial OS=Homo sapiens GN=MRPL13 PE=1 SV=1 - [RM13_HUMAN]	28.09	1	4	4	15	1.151	1.621	1.115	1.228	32.31	28.09	7	15	178	20.7	9.16
Q9UHY7	Enolase-phosphatase E1 OS=Homo sapiens GN=ENOPH1 PE=1 SV=1 - [ENOPH_HUMAN]	13.79	1	3	3	5	1.439	1.454	1.221	1.229	11.71	13.79	5	5	261	28.9	4.78
P22003	Bone morphogenetic protein 5 OS=Homo sapiens GN=BMP5 PE=2 SV=1 - [BMP5_HUMAN]	3.96	1	1	1	1	1.648	0.943	0.660	1.229	0.00	3.96	1	1	454	51.7	8.88
O75592	E3 ubiquitin-protein ligase MYCBP2 OS=Homo sapiens GN=MYCBP2 PE=1 SV=3 - [MYCB2_HUMAN]	2.00	1	9	9	18	1.055	0.974	0.951	1.229	35.22	2.00	12	18	4640	509.8	7.03

P27694	Replication protein A 70 kDa DNA-binding subunit OS=Homo sapiens GN=RPA1 PE=1 SV=2 - [RFA1_HUMAN]	19.48	1	8	8	29	1.526	1.492	1.245	1.229	54.19	19.48	14	29	616	68.1	7.21
Q9BYN8	28S ribosomal protein S26, mitochondrial OS=Homo sapiens GN=MRPS26 PE=1 SV=1 - [RT26_HUMAN]	28.78	1	6	6	17	0.981	1.397	1.076	1.229	40.37	28.78	9	17	205	24.2	10.39
Q6ZRP7	Sulfhydryl oxidase 2 OS=Homo sapiens GN=QSOX2 PE=1 SV=3 - [QSOX2_HUMAN]	7.88	1	5	5	12	0.912	0.861	0.766	1.230	29.87	7.88	7	12	698	77.5	7.72
Q9Y5A9	YTH domain-containing family protein 2 OS=Homo sapiens GN=YTHDF2 PE=1 SV=2 - [YTHD2_HUMAN]	12.61	1	6	6	19	0.843	0.956	0.980	1.230	21.76	12.61	8	19	579	62.3	8.79
O75208	Ubiquinone biosynthesis protein COQ9, mitochondrial OS=Homo sapiens GN=COQ9 PE=1 SV=1 - [COQ9_HUMAN]	10.38	1	3	3	7	1.024	2.153	1.002	1.230	11.49	10.38	5	7	318	35.5	5.94
Q9BQ69	O-acetyl-ADP-ribose deacetylase MACROD1 OS=Homo sapiens GN=MACROD1 PE=1 SV=2 - [MACD1_HUMAN]	13.23	1	3	3	3	1.117	1.397	0.985	1.230	9.46	13.23	3	3	325	35.5	9.51
Q12824	SWI/SNF-related matrix-associated actin-dependent regulator of chromatin subfamily B member 1 OS=Homo sapiens GN=SMARCB1 PE=1 SV=2 - [SNF5_HUMAN]	11.69	1	4	4	11	1.124	1.178	1.073	1.230	24.69	11.69	7	11	385	44.1	6.23
P11277	Spectrin beta chain, erythrocytic OS=Homo sapiens GN=SPTB PE=1 SV=5 - [SPTB1_HUMAN]	4.68	1	2	11	49	1.286	0.395	1.436	1.230	93.94	4.68	17	49	2137	246.3	5.27

P35658	Nudear pore complex protein Nup214 OS=Homo sapiens GN=NUP214 PE=1 SV=2 - [NU214_HUMAN	12.01	3	19	21	72	1.150	1.251	0.972	1.230	158.27	12.01	34	72	2090	213.5	7.47
P78345	Ribonudease P protein subunit p38 OS=Homo sapiens GN=RPP38 PE=1 SV=2 - [RPP38_HUMAN]	4.59	1	1	2	3	0.744	0.796	0.714	1.231	4.41	4.59	2	3	283	31.8	9.92
Q8WY22	BRI3-binding protein OS=Homo sapiens GN=BRI3BP PE=1 SV=1 - [BRI3B_HUMAN]	9.56	1	2	2	7	1.250	0.646	0.642	1.231	25.02	9.56	3	7	251	27.8	9.44
Q8IYU8	Calcium uptake protein 2, mitochondrial OS=Homo sapiens GN=MICU2 PE=1 SV=2 - [MICU2_HUMAN]	10.37	1	3	3	6	0.758	0.641	0.697	1.231	18.47	10.37	4	6	434	49.6	9.09
Q9Y2R4	Probable ATP-dependent RNA helicase DDX52 OS=Homo sapiens GN=DDX52 PE=1 SV=3 - [DDX52_HUMAN	6.34	1	3	4	5	0.762	0.640	0.865	1.231	9.18	6.34	5	5	599	67.5	9.67
Q9NUU7	ATP-dependent RNA helicase DDX19A OS=Homo sapiens GN=DDX19A PE=1 SV=1 - [DD19A_HUMAN	25.94	2	13	13	92	1.076	1.172	1.069	1.231	187.82	25.94	21	92	478	53.9	6.58
Q75N03	E3 ubiquitin-protein ligase Hakai OS=Homo sapiens GN=CBLL1 PE=1 SV=1 - [HAKAI_HUMAN]	10.39	1	3	3	6	0.837	1.078	1.168	1.231	13.21	10.39	4	6	491	54.5	8.29
Q9Y315	Deoxyribose-phosphate aldolase OS=Homo sapiens GN=DERA PE=1 SV=2 - [DEOC_HUMAN]	25.16	1	7	7	19	1.287	0.820	0.448	1.232	44.01	25.16	10	19	318	35.2	8.94
P14316	Interferon regulatory factor 2 OS=Homo sapiens GN=IRF2 PE=1 SV=2 - [IRF2_HUMAN]	5.16	1	1	1	1	1.166	0.868	0.619	1.232	2.02	5.16	1	1	349	39.3	7.52

Q8NFH5	Nucleoporin NUP53 OS=Homo sapiens GN=NUP35 PE=1 SV=1 - [NUP53_HUMAN]	19.94	1	5	5	16	1.115	1.558	1.046	1.232	46.03	19.94	8	16	326	34.8	9.09
Q9BVL4	Selenoprotein O OS=Homo sapiens GN=SELO PE=2 SV=3 - [SELO_HUMAN]	9.72	1	5	5	16	1.176	1.208	1.365	1.232	36.51	9.72	7	16	669	73.4	5.97
P07992	DNA excision repair protein ERCC-1 OS=Homo sapiens GN=ERCC1 PE=1 SV=1 - [ERCC1_HUMAN]	5.72	1	2	2	6	0.944	1.194	0.780	1.232	9.41	5.72	3	6	297	32.5	6.25
Q01844	RNA-binding protein EWS OS=Homo sapiens GN=EWSR1 PE=1 SV=1 - [EWS_HUMAN]	9.91	1	5	5	28	1.121	1.621	1.042	1.232	74.57	9.91	9	28	656	68.4	9.33
Q96E11	Ribosome-recycling factor, mitochondrial OS=Homo sapiens GN=MRRF PE=1 SV=1 - [RRFM_HUMAN]	18.32	1	4	4	14	1.858	1.338	0.925	1.232	32.88	18.32	7	14	262	29.3	9.79
Q15717	ELAV-like protein 1 OS=Homo sapiens GN=ELAVL1 PE=1 SV=2 - [ELAV1_HUMAN]	33.13	2	9	10	43	1.059	1.300	1.061	1.232	99.16	33.13	19	43	326	36.1	9.17
Q9BRT6	Protein LLP homolog OS=Homo sapiens GN=LLPH PE=2 SV=1 - [LLPH_HUMAN]	12.40	1	1	1	17	1.007	1.221	1.073	1.232	54.28	12.40	2	17	129	15.2	10.37
O43837	Isocitrate dehydrogenase [NAD] subunit beta, mitochondrial OS=Homo sapiens GN=IDH3B PE=1 SV=2 - [IDH3B_HUMAN]	24.16	1	7	7	20	1.315	1.417	1.013	1.232	44.28	24.16	11	20	385	42.2	8.46
A5D6W6	Fat storage-inducing transmembrane protein 1 OS=Homo sapiens GN=FITM1 PE=1 SV=1 - [FITM1_HUMAN]	12.33	1	2	2	2	2.296	0.804	0.879	1.233	2.70	12.33	2	2	292	32.2	9.79

Q7Z7K0	COX assembly mitochondrial protein homolog OS=Homo sapiens GN=CMC1 PE=1 SV=1 - [COXM1_HUMAN]	33.96	1	4	4	5	1.609	1.209	1.290	1.233	11.75	33.96	4	5	106	12.5	8.63
O43390	Heterogeneous nudear ribonudeoprotein R OS=Homo sapiens GN=HNRNPR PE=1 SV=1 - [HNRPR_HUMAN	43.76	2	19	27	124	1.082	1.390	0.986	1.233	311.18	43.76	45	124	633	70.9	8.13
O95777	U6 snRNA-associated Sm-like protein LSm8 OS=Homo sapiens GN=LSM8 PE=1 SV=3 - [LSM8_HUMAN]	59.38	1	4	4	10	0.909	1.094	0.715	1.233	27.39	59.38	8	10	96	10.4	4.48
Q96EY7	Pentatricopeptide repeat domain-containing protein 3, mitochondrial OS=Homo sapiens GN=PTCD3 PE=1 SV=3 - [PTCD3_HUMAN	14.22	1	8	8	29	1.037	1.347	0.889	1.233	60.44	14.22	14	29	689	78.5	6.42
A2RUR9	Coiled-coil domain-containing protein 144A OS=Homo sapiens GN=CCDC144A PE=1 SV=1 - [C144A_HUMAN]	1.89	2	2	4	5	1.051	0.511	0.867	1.234	10.20	1.89	4	5	1427	165.0	5.36
Q00978	Interferon regulatory factor 9 OS=Homo sapiens GN=IRF9 PE=1 SV=1 - [IRF9_HUMAN]	7.38	1	3	3	6	2.033	1.579	2.057	1.234	10.45	7.38	4	6	393	43.7	5.77
P78562	Phosphate-regulating neutral endopeptidase OS=Homo sapiens GN=PHEX PE=1 SV=1 - [PHEX_HUMAN]	0.80	1	1	1	1	0.795	1.085	1.220	1.234	0.00	0.80	1	1	749	86.4	8.76
Q9P013	Spliceosome-associated protein CWC15 homolog OS=Homo sapiens GN=CWC15 PE=1 SV=2 - [CWC15_HUMAN]	8.30	1	3	3	9	1.126	1.263	1.059	1.234	20.91	8.30	4	9	229	26.6	5.71

O75533	Splicing factor 3B subunit 1 OS=Homo sapiens GN=SF3B1 PE=1 SV=3 - [SF3B1_HUMAN]	31.29	1	37	37	124	1.070	1.273	1.105	1.234	367.52	31.29	53	124	1304	145.7	7.09
Q9NPF5	DNA methyltransferase 1-associated protein 1 OS=Homo sapiens GN=DMAP1 PE=1 SV=1 - [DMAP1_HUMA	7.49	1	3	3	5	1.114	1.761	1.011	1.235	17.75	7.49	4	5	467	53.0	9.50
P49662	Caspase-4 OS=Homo sapiens GN=CASP4 PE=1 SV=1 - [CASP4_HUMAN]	19.36	2	8	8	27	0.723	0.867	0.696	1.235	90.17	19.36	14	27	377	43.2	6.00
Q9NX61	Transmembrane protein 161A OS=Homo sapiens GN=TMEM161A PE=2 SV=1 - [T161A_HUMAN]	3.13	1	1	1	6	1.049	0.815	0.893	1.235	8.94	3.13	2	6	479	53.6	8.44
Q96RJ0	Trace amine-associated receptor 1 OS=Homo sapiens GN=TAAR1 PE=1 SV=1 - [TAAR1_HUMAN]	2.36	1	1	1	1	1.323	1.086	1.010	1.235	2.19	2.36	1	1	339	39.1	8.70
P10809	60 kDa heat shock protein, mitochondrial OS=Homo sapiens GN=HSPD1 PE=1 SV=2 - [CH60_HUMAN]	81.68	1	46	46	696	0.950	1.243	0.908	1.235	2024.67	81.68	77	696	573	61.0	5.87
P51553	Isocitrate dehydrogenase [NAD] subunit gamma, mitochondrial OS=Homo sapiens GN=IDH3G PE=1 SV=1 - [IDH3G_HUMAN]	15.52	1	4	4	12	1.160	1.115	0.898	1.235	27.14	15.52	6	12	393	42.8	8.50
Q9UKK6	NTF2-related export protein 1 OS=Homo sapiens GN=NXT1 PE=1 SV=1 - [NXT1_HUMAN]	5.71	2	1	1	1	0.957	1.127	1.602	1.235	0.00	5.71	1	1	140	15.8	5.03
Q96RU2	Ubiquitin carboxyl-terminal hydrolase 28 OS=Homo sapiens GN=USP28 PE=1 SV=1 - [UBP28_HUMAN]	1.39	1	1	1	1	1.859	1.944	1.332	1.235	2.20	1.39	1	1	1077	122.4	5.20

Q9NNW7	Thioredoxin reductase 2, mitochondrial OS=Homo sapiens GN=TXNRD2 PE=1 SV=3 - [TRXR2_HUMAN]	8.21	1	5	5	32	1.326	1.260	1.183	1.235	76.31	8.21	9	32	524	56.5	7.50
Q9BW91	ADP-ribose pyrophosphatase, mitochondrial OS=Homo sapiens GN=NUDT9 PE=1 SV=1 - [NUDT9_HUMAN]	21.71	1	7	7	25	1.168	1.097	1.038	1.236	60.31	21.71	14	25	350	39.1	8.22
P80723	Brain acid soluble protein 1 OS=Homo sapiens GN=BASP1 PE=1 SV=2 - [BASP1_HUMAN]	95.59	1	24	24	250	3.786	0.917	0.445	1.236	745.66	95.59	34	250	227	22.7	4.63
Q15424	Scaffold attachment factor B1 OS=Homo sapiens GN=SAFB PE=1 SV=4 - [SAFB1_HUMAN	17.05	1	9	16	43	0.945	1.180	0.915	1.236	102.54	17.05	23	43	915	102.6	5.47
P50749	Ras association domain-containing protein 2 OS=Homo sapiens GN=RASSF2 PE=1 SV=1 - [RASF2_HUMAN]	17.18	1	5	5	20	1.057	0.806	1.020	1.236	44.31	17.18	8	20	326	37.8	8.84
Q06203	Amidophosphoribosyltransferase OS=Homo sapiens GN=PPAT PE=1 SV=1 - [PUR1_HUMAN]	16.63	1	7	7	19	0.589	1.030	0.694	1.236	33.81	16.63	12	19	517	57.4	6.76
Q99575	Ribonucleases P/MRP protein subunit POP1 OS=Homo sapiens GN=POP1 PE=1 SV=2 - [POP1_HUMAN]	12.89	1	9	9	21	0.953	1.042	1.188	1.236	59.55	12.89	12	21	1024	114.6	9.22
Q96RN5	Mediator of RNA polymerase II transcription subunit 15 OS=Homo sapiens GN=MED15 PE=1 SV=2 - [MED15_HUMAN]	6.60	1	4	4	11	1.137	1.034	1.089	1.236	21.54	6.60	6	11	788	86.7	9.42

Q16539	Mitogen-activated protein kinase 14 OS=Homo sapiens GN=MAPK14 PE=1 SV=3 - [MK14_HUMAN]	33.89	3	10	11	37	1.199	1.530	1.282	1.236	91.91	33.89	18	37	360	41.3	5.78
Q9P291	Armadillo repeat-containing X-linked protein 1 OS=Homo sapiens GN=ARMCX1 PE=1 SV=1 - [ARMX1_HUMA	3.09	1	2	2	6	1.158	0.822	1.031	1.237	12.28	3.09	3	6	453	49.1	9.22
Q9P2H5	Ubiquitin carboxyl-terminal hydrolase 35 OS=Homo sapiens GN=USP35 PE=1 SV=3 - [UBP35_HUMAN]	2.26	1	1	2	4	1.514	0.942	0.962	1.237	0.00	2.26	2	4	1018	113.3	5.57
O75880	Protein SCO1 homolog, mitochondrial OS=Homo sapiens GN=SCO1 PE=1 SV=1 - [SCO1_HUMAN]	19.93	1	4	4	9	0.842	1.005	1.399	1.237	17.99	19.93	5	9	301	33.8	8.88
Q96P47	Arf-GAP with GTPase, ANK repeat and PH domain-containing protein 3 OS=Homo sapiens GN=AGAP3 PE=1 SV=2 - [AGAP3_HUMAN]	5.14	2	2	5	8	0.975	1.165	1.576	1.237	15.20	5.14	6	8	875	95.0	7.97
A6NCW7	Inactive ubiquitin carboxyl-terminal hydrolase 17-like protein 4 OS=Homo sapiens GN=USP17L4 PE=3 SV=3 - [U17L4_HUMAN]	4.34	4	1	2	3	1.145	0.885	1.236	1.237	4.78	4.34	2	3	530	59.6	7.42
Q6IMI4	Sulfotransferase 6B1 OS=Homo sapiens GN=SULT6B1 PE=2 SV=2 - [ST6B1_HUMAN	2.64	1	1	1	3	0.945	1.533	1.869	1.238	6.88	2.64	1	3	303	34.9	6.46
Q92979	Ribosomal RNA small subunit methyltransferase NEP1 OS=Homo sapiens GN=EMG1 PE=1 SV=4 - [NEP1_HUMAN]	4.51	1	1	1	4	0.963	0.673	0.807	1.238	5.09	4.51	2	4	244	26.7	9.17

P22087	rRNA 2'-O-methyltransferase fibrillarin OS=Homo sapiens GN=FBL PE=1 SV=2 - [FBRL_HUMAN]	35.20	1	12	13	48	1.111	1.315	1.022	1.238	105.54	35.20	21	48	321	33.8	10.18
Q96T17	MAP7 domain-containing protein 2 OS=Homo sapiens GN=MAP7D2 PE=1 SV=2 - [MA7D2_HUMAN]	6.15	1	3	3	6	1.488	0.640	1.080	1.238	13.85	6.15	3	6	732	81.9	8.84
O14879	Interferon-induced protein with tetratricopeptide repeats 3 OS=Homo sapiens GN=IFIT3 PE=1 SV=1 - [IFIT3_HUMAN]	31.84	2	14	15	41	5.915	1.176	1.925	1.238	105.95	31.84	22	41	490	55.9	5.20
Q8N392	Rho GTPase-activating protein 18 OS=Homo sapiens GN=ARHGAP18 PE=1 SV=3 - [RHG18_HUMAN]	18.10	1	10	11	30	1.459	0.932	1.248	1.238	75.97	18.10	18	30	663	74.9	6.44
Q14527	Helicase-like transcription factor OS=Homo sapiens GN=HLTF PE=1 SV=2 - [HLTF_HUMAN]	2.08	1	3	3	6	0.877	1.210	1.165	1.238	10.44	2.08	4	6	1009	113.9	8.60
O75175	CCR4-NOT transcription complex subunit 3 OS=Homo sapiens GN=CNOT3 PE=1 SV=1 - [CNOT3_HUMAN]	12.08	1	10	10	18	0.903	1.206	1.020	1.239	37.28	12.08	13	18	753	81.8	6.20
Q4L180	Filamin A-interacting protein 1-like OS=Homo sapiens GN=FILIP1L PE=1 SV=2 - [FIL1L_HUMAN]	39.30	2	45	45	159	2.263	0.486	1.482	1.239	358.19	39.30	62	159	1135	130.3	6.57
O60235	Transmembrane protease serine 11D OS=Homo sapiens GN=TMPRSS11D PE=1 SV=1 - [TM11D_HUMAN]	3.11	1	1	1	1	0.899	0.205	0.325	1.239	2.02	3.11	1	1	418	46.2	8.38

P25705	ATP synthase subunit alpha, mitochondrial OS=Homo sapiens GN=ATP5A1 PE=1 SV=1 - [ATPA_HUMAN]	58.23	1	36	37	546	1.170	1.055	0.891	1.240	1357.11	58.23	64	546	553	59.7	9.13
P29622	Kallistatin OS=Homo sapiens GN=SERPINA4 PE=1 SV=3 - [KAIN_HUMAN]	2.81	1	1	1	1	1.064	1.606	1.363	1.240	2.63	2.81	1	1	427	48.5	7.75
Q93009	Ubiquitin carboxyl-terminal hydrolase 7 OS=Homo sapiens GN=USP7 PE=1 SV=2 - [UBP7_HUMAN]	16.88	1	16	16	50	1.038	1.099	1.039	1.240	115.59	16.88	23	50	1102	128.2	5.55
Q96CS2	HAUS augmin-like complex subunit 1 OS=Homo sapiens GN=HAUS1 PE=1 SV=1 - [HAUS1_HUMAN]	3.96	1	1	1	2	1.093	0.906	1.056	1.240	6.65	3.96	1	2	278	31.8	5.53
O75351	Vacuolar protein sorting-associated protein 4B OS=Homo sapiens GN=VPS4B PE=1 SV=2 - [VPS4B_HUMAN]	35.81	1	11	17	60	1.104	1.084	1.037	1.240	140.51	35.81	27	60	444	49.3	7.23
Q7Z434	Mitochondrial antiviral-signaling protein OS=Homo sapiens GN=MAVS PE=1 SV=2 - [MAVS_HUMAN]	23.52	1	9	9	31	1.099	1.050	1.044	1.240	69.58	23.52	13	31	540	56.5	5.52
Q92733	Proline-rich protein PRCC OS=Homo sapiens GN=PRCC PE=1 SV=1 - [PRCC_HUMAN]	9.16	1	3	3	5	0.903	0.908	0.603	1.240	9.55	9.16	4	5	491	52.4	5.10
P43121	Cell surface glycoprotein MUC18 OS=Homo sapiens GN=MCAM PE=1 SV=2 - [MUC18_HUMAN]	22.29	1	12	12	43	2.642	1.028	1.084	1.240	99.68	22.29	21	43	646	71.6	5.76
Q6P587	Acylpyruvase FAHD1, mitochondrial OS=Homo sapiens GN=FAHD1 PE=1 SV=2 - [FAHD1_HUMAN]	26.34	1	6	6	18	1.323	1.443	1.120	1.240	43.09	26.34	9	18	224	24.8	7.39

Q96L91	E1A-binding protein p400 OS=Homo sapiens GN=EP400 PE=1 SV=4 - [EP400_HUMAN]	0.95	1	2	2	7	1.910	1.133	0.932	1.240	6.74	0.95	3	7	3159	343.3	9.19
P49711	Transcriptional repressor CTCF OS=Homo sapiens GN=CTCF PE=1 SV=1 - [CTCF_HUMAN]	5.91	2	5	5	8	1.004	1.097	0.866	1.241	4.73	5.91	5	8	727	82.7	6.96
P18887	DNA repair protein XRCC1 OS=Homo sapiens GN=XRCC1 PE=1 SV=2 - [XRCC1_HUMAN]	19.91	1	10	10	24	1.120	1.267	1.065	1.241	44.72	19.91	16	24	633	69.4	6.39
Q1X8D7	Leucine-rich repeat-containing protein 36 OS=Homo sapiens GN=LRRC36 PE=2 SV=2 - [LRC36_HUMAN]	1.46	1	1	1	1	1.420	0.881	1.017	1.241	3.01	1.46	1	1	754	83.8	7.15
Q9ULE0	Protein WWC3 OS=Homo sapiens GN=WWC3 PE=1 SV=3 - [WWC3_HUMA N]	4.76	2	2	6	13	1.086	0.642	0.747	1.241	12.93	4.76	7	13	1092	122.6	6.37
Q5VTL8	Pre-mRNA-splicing factor 38B OS=Homo sapiens GN=PRPF38B PE=1 SV=1 - [PR38B_HUMAN]	4.95	1	4	4	7	1.094	0.997	0.962	1.241	11.72	4.95	5	7	546	64.4	10.54
Q9H4Z3	Phosphorylated CTD-interacting factor 1 OS=Homo sapiens GN=PCIF1 PE=1 SV=1 - [PCIF1_HUMAN]	1.28	1	1	1	1	1.165	1.166	1.113	1.241	1.81	1.28	1	1	704	80.6	7.42
Q96IK1	Biorientation of chromosomes in cell division protein 1 OS=Homo sapiens GN=BOD1 PE=1 SV=2 - [BOD1_HUMAN]	18.38	1	2	3	5	1.019	1.716	2.499	1.242	14.27	18.38	4	5	185	19.2	6.33
Q9NVA1	Ubiquinol-cytochrome-c reductase complex assembly factor 1 OS=Homo sapiens GN=UQCC1 PE=1 SV=3 - [UQCC1_HUMA N]	12.37	1	3	3	11	1.057	1.124	1.110	1.242	28.85	12.37	4	11	299	34.6	8.91

O43679	LIM domain-binding protein 2 OS=Homo sapiens GN=LDB2 PE=1 SV=1 - [LDB2_HUMAN]	2.68	2	1	1	2	1.442	1.516	1.192	1.242	2.56	2.68	1	2	373	42.8	6.93
Q13018	Secretory phospholipase A2 receptor OS=Homo sapiens GN=PLA2R1 PE=1 SV=2 - [PLA2R_HUMAN]	0.89	1	1	2	3	0.633	0.746	1.269	1.242	9.32	0.89	2	3	1463	168.5	6.11
P11498	Pyruvate carboxylase, mitochondrial OS=Homo sapiens GN=PC PE=1 SV=2 - [PYC_HUMAN]	29.37	1	28	28	75	0.969	1.553	1.059	1.242	190.01	29.37	43	75	1178	129.6	6.84
Q9NV06	DDB1- and CUL4-associated factor 13 OS=Homo sapiens GN=DCAF13 PE=1 SV=2 - [DCA13_HUMAN	9.44	1	5	5	10	1.052	1.067	1.264	1.243	17.00	9.44	9	10	445	51.4	9.19
Q9UQR1	Zinc finger protein 148 OS=Homo sapiens GN=ZNF148 PE=1 SV=2 - [ZN148_HUMAN]	2.14	3	1	2	4	1.133	1.189	1.003	1.243	4.60	2.14	3	4	794	88.9	6.48
Q9UNA4	DNA polymerase iota OS=Homo sapiens GN=POLI PE=1 SV=3 - [POLI_HUMAN]	2.03	1	1	2	2	1.062	0.978	1.320	1.243	5.16	2.03	2	2	740	83.0	6.07
Q9UQE7	Structural maintenance of chromosomes protein 3 OS=Homo sapiens GN=SMC3 PE=1 SV=2 - [SMC3_HUMAN]	40.26	1	46	48	134	0.970	1.539	1.010	1.243	331.64	40.26	68	134	1217	141.5	7.18
O43598	2'-deoxynucleoside 5'-phosphate N-hydrolase 1 OS=Homo sapiens GN=DNPH1 PE=1 SV=1 - [DNPH1_HUMAN]	36.78	1	3	3	15	0.745	0.802	0.837	1.243	45.60	36.78	4	15	174	19.1	5.05
Q96HD1	Cysteine-rich with EGF-like domain protein 1 OS=Homo sapiens GN=CRELD1 PE=1 SV=3 - [CREL1_HUMAN]	21.19	1	8	8	28	0.799	1.055	1.015	1.243	81.06	21.19	14	28	420	45.4	4.87

Q13547	Histone deacetylase 1 OS=Homo sapiens GN=HDAC1 PE=1 SV=1 - [HDAC1_HUMAN]	17.63	1	3	7	24	1.238	1.510	0.788	1.243	34.02	17.63	11	24	482	55.1	5.48
Q86TS9	39S ribosomal protein L52, mitochondrial OS=Homo sapiens GN=MRPL52 PE=2 SV=2 - [RM52_HUMAN]	7.32	1	1	1	2	1.161	1.791	1.021	1.244	0.00	7.32	1	2	123	13.7	10.26
P36954	DNA-directed RNA polymerase II subunit RPB9 OS=Homo sapiens GN=POLR2I PE=1 SV=1 - [RPB9_HUMAN]	8.80	1	1	1	4	1.304	1.168	1.024	1.244	7.96	8.80	2	4	125	14.5	5.14
P17152	Transmembrane protein 11, mitochondrial OS=Homo sapiens GN=TMEM11 PE=1 SV=1 - [TMM11_HUMAN]	4.17	1	1	1	3	1.144	0.829	0.866	1.244	3.89	4.17	2	3	192	21.5	7.36
Q562E7	WD repeat-containing protein 81 OS=Homo sapiens GN=WDR81 PE=1 SV=2 - [WDR81_HUMAN]	3.30	1	4	4	5	1.429	1.680	1.257	1.244	9.18	3.30	5	5	1941	211.6	5.58
O15067	Phosphoribosylformylglycinamidine synthase OS=Homo sapiens GN=PFAS PE=1 SV=4 - [PUR4_HUMAN]	18.01	1	17	18	61	1.059	1.227	0.909	1.244	155.46	18.01	30	61	1338	144.6	5.76
Q9UKV3	Apoptotic chromatin condensation inducer in the nucleus OS=Homo sapiens GN=ACIN1 PE=1 SV=2 - [ACINU_HUMAN]	16.48	1	21	21	63	1.310	1.464	1.139	1.244	159.69	16.48	33	63	1341	151.8	6.43
Q6RW13	Type-1 angiotensin II receptor-associated protein OS=Homo sapiens GN=AGTRAP PE=1 SV=1 - [ATRAP_HUMAN]	10.69	1	1	1	2	1.509	0.839	0.935	1.244	7.25	10.69	2	2	159	17.4	6.14

Q9Y2W2	WW domain-binding protein 11 OS=Homo sapiens GN=WBP11 PE=1 SV=1 - [WBP11_HUMAN]	17.94	1	12	12	37	1.134	1.371	1.042	1.244	90.03	17.94	15	37	641	70.0	8.38
P09001	39S ribosomal protein L3, mitochondrial OS=Homo sapiens GN=MRPL3 PE=1 SV=1 - [RM03_HUMAN]	7.47	1	2	2	5	1.043	1.471	1.091	1.244	13.67	7.47	4	5	348	38.6	9.48
Q16540	39S ribosomal protein L23, mitochondrial OS=Homo sapiens GN=MRPL23 PE=1 SV=1 - [RM23_HUMAN]	9.80	1	1	1	3	0.849	1.067	0.920	1.244	9.61	9.80	2	3	153	17.8	9.69
A8MVJ9	Putative UPF0609 protein C4orf27-like OS=Homo sapiens PE=3 SV=1 - [YI028_HUMAN]	4.32	1	1	2	3	0.751	2.547	2.126	1.244	6.43	4.32	3	3	347	39.7	8.10
Q9Y244	Proteasome maturation protein OS=Homo sapiens GN=POMP PE=1 SV=1 - [POMP_HUMAN]	20.57	1	3	3	9	0.884	0.728	0.789	1.245	19.65	20.57	5	9	141	15.8	5.11
P30154	Serine/threonine-protein phosphatase 2A 65 kDa regulatory subunit A beta isoform OS=Homo sapiens GN=PPP2R1B PE=1 SV=3 - [2AAB_HUMAN]	12.31	1	2	7	31	0.734	1.091	1.137	1.245	78.57	12.31	11	31	601	66.2	4.94
Q03188	Centromere protein C OS=Homo sapiens GN=CENPC PE=1 SV=2 - [CENPC_HUMAN]	0.74	1	1	1	1	0.754	1.580	1.173	1.245	2.19	0.74	1	1	943	106.8	9.39
Q6GYQ0	Ral GTPase-activating protein subunit alpha-1 OS=Homo sapiens GN=RALGAPA1 PE=1 SV=1 - [RGPA1_HUMAN]	1.72	1	3	3	4	1.065	1.227	1.055	1.245	9.00	1.72	4	4	2036	229.7	6.19

Q9BQG2	Peroxisomal NADH pyrophosphatase NUDT12 OS=Homo sapiens GN=NUDT12 PE=1 SV=1 - [NUD12_HUMAN]	1.73	1	1	1	2	1.303	1.379	0.741	1.245	2.12	1.73	2	2	462	52.0	6.83
P42285	Superkiller viralicidic activity 2-like 2 OS=Homo sapiens GN=SKIV2L2 PE=1 SV=3 - [SK2L2_HUMAN]	13.24	1	14	14	39	0.980	1.126	0.850	1.245	85.30	13.24	20	39	1042	117.7	6.52
Q9Y483	Metal-response element-binding transcription factor 2 OS=Homo sapiens GN=MTF2 PE=1 SV=2 - [MTF2_HUMAN]	1.35	1	1	1	1	1.560	0.191	0.510	1.245	2.12	1.35	1	1	593	67.1	8.72
O60524	Nuclear export mediator factor NEMF OS=Homo sapiens GN=NEMF PE=1 SV=4 - [NEMF_HUMAN]	12.64	1	14	14	58	1.005	0.996	1.012	1.246	122.28	12.64	20	58	1076	122.9	6.35
P61326	Protein mago nashi homolog OS=Homo sapiens GN=MAGOH PE=1 SV=1 - [MGN_HUMAN]	20.55	2	3	3	7	1.108	1.046	0.779	1.246	16.71	20.55	3	7	146	17.2	6.11
Q9Y548	Protein YIPF1 OS=Homo sapiens GN=YIPF1 PE=2 SV=1 - [YIPF1_HUMAN]	2.94	1	1	1	2	1.505	0.818	0.901	1.246	4.80	2.94	2	2	306	34.3	5.31
Q8IXI1	Mitochondrial Rho GTPase 2 OS=Homo sapiens GN=RHOT2 PE=1 SV=2 - [MIRO2_HUMAN]	7.61	1	2	4	5	0.780	0.789	0.668	1.246	14.98	7.61	4	5	618	68.1	5.86
Q9UJX3	Anaphase-promoting complex subunit 7 OS=Homo sapiens GN=ANAPC7 PE=1 SV=4 - [APC7_HUMAN]	11.35	1	6	6	11	1.127	1.055	1.172	1.246	31.43	11.35	9	11	599	66.8	5.64
Q9UK59	Lariat debranching enzyme OS=Homo sapiens GN=DBR1 PE=1 SV=2 - [DBR1_HUMAN]	2.02	1	1	1	4	1.611	1.302	1.099	1.246	5.25	2.02	2	4	544	61.5	5.47

Q8WXE0	Caskin-2 OS=Homo sapiens GN=CASKIN2 PE=1 SV=2 - [CSKI2_HUMAN]	5.16	1	3	4	12	0.902	1.146	1.373	1.247	47.27	5.16	6	12	1202	126.7	7.09
Q9H147	Deoxynucleotidyltransferase terminal-interacting protein 1 OS=Homo sapiens GN=DNTTIP1 PE=1 SV=2 - [TDIF1_HUMAN]	6.08	1	2	2	3	1.224	1.425	1.135	1.247	2.29	6.08	2	3	329	37.0	8.97
Q9Y6D9	Mitotic spindle assembly checkpoint protein MAD1 OS=Homo sapiens GN=MAD1L1 PE=1 SV=2 - [MD1L1_HUMAN]	19.36	1	12	12	28	1.204	1.251	1.046	1.247	71.12	19.36	17	28	718	83.0	5.92
Q96D96	Voltage-gated hydrogen channel 1 OS=Homo sapiens GN=HVCN1 PE=1 SV=1 - [HVCN1_HUMAN]	5.49	1	1	1	2	0.890	1.109	1.419	1.247	5.44	5.49	2	2	273	31.7	6.80
P51570	Galactokinase OS=Homo sapiens GN=GALK1 PE=1 SV=1 - [GALK1_HUMAN]	35.46	1	12	12	48	1.225	0.918	0.998	1.247	141.63	35.46	21	48	392	42.2	6.46
O75027	ATP-binding cassette sub-family B member 7, mitochondrial OS=Homo sapiens GN=ABCB7 PE=1 SV=2 - [ABCB7_HUMAN]	5.05	1	4	4	5	0.764	1.358	1.002	1.247	9.87	5.05	4	5	752	82.6	9.33
Q9NUW8	Tyrosyl-DNA phosphodiesterase 1 OS=Homo sapiens GN=TDP1 PE=1 SV=2 - [TYDP1_HUMAN]	3.78	1	2	2	3	0.865	1.675	1.420	1.247	3.40	3.78	3	3	608	68.4	7.65
O43674	NADH dehydrogenase [ubiquinone] 1 beta subcomplex subunit 5, mitochondrial OS=Homo sapiens GN=NDUFB5 PE=1 SV=1 - [NDUB5_HUMAN]	23.28	1	5	5	15	0.828	0.846	0.780	1.247	30.36	23.28	8	15	189	21.7	9.63

Q86X10	Ral GTPase-activating protein subunit beta OS=Homo sapiens GN=RALGAPB PE=1 SV=1 - [RLGPB_HUMAN	1.41	1	3	3	6	1.090	1.302	1.051	1.248	12.18	1.41	3	6	1494	166.7	6.79
Q9H0U3	Magnesium transporter protein 1 OS=Homo sapiens GN=MAGT1 PE=1 SV=1 - [MAGT1_HUMAN]	15.52	1	6	7	20	1.004	1.019	0.753	1.248	47.47	15.52	11	20	335	38.0	9.63
Q9Y4B5	Microtubule cross-linking factor 1 OS=Homo sapiens GN=MTCL1 PE=1 SV=5 - [MTCL1_HUMAN]	12.86	1	17	20	36	1.040	1.221	1.256	1.248	80.91	12.86	29	36	1905	209.4	6.43
Q96CB9	5-methylcytosine rRNA methyltransferase NSUN4 OS=Homo sapiens GN=NSUN4 PE=1 SV=2 - [NSUN4_HUMAN]	5.73	1	2	2	4	1.019	1.078	0.858	1.248	10.80	5.73	4	4	384	43.1	8.18
P23508	Colorectal mutant cancer protein OS=Homo sapiens GN=MCC PE=1 SV=2 - [CRCM_HUMAN]	9.89	1	7	7	15	1.276	1.225	0.756	1.248	32.48	9.89	10	15	829	93.0	5.52
P55265	Double-stranded RNA-specific adenosine deaminase OS=Homo sapiens GN=ADAR PE=1 SV=4 - [DSRAD_HUMAN]	23.65	1	29	29	148	1.417	1.053	1.054	1.248	351.32	23.65	48	148	1226	136.0	8.65
P83916	Chromobox protein homolog 1 OS=Homo sapiens GN=CBX1 PE=1 SV=1 - [CBX1_HUMAN]	41.08	1	4	5	19	1.350	1.159	0.762	1.248	40.31	41.08	7	19	185	21.4	4.93
Q7L2H7	Eukaryotic translation initiation factor 3 subunit M OS=Homo sapiens GN=EIF3M PE=1 SV=1 - [EIF3M_HUMAN]	7.49	1	2	2	8	0.785	0.861	0.795	1.248	24.88	7.49	3	8	374	42.5	5.63

Q9P2G1	Ankyrin repeat and IBR domain-containing protein 1 OS=Homo sapiens GN=ANKIB1 PE=1 SV=3 - [AKIB1_HUMAN	5.51	1	5	5	7	2.083	1.336	1.237	1.248	20.54	5.51	6	7	1089	121.9	5.11
Q13610	Periodic tryptophan protein 1 homolog OS=Homo sapiens GN=PWP1 PE=1 SV=1 - [PWP1_HUMAN]	9.78	1	3	4	8	0.510	0.893	1.003	1.248	16.94	9.78	5	8	501	55.8	4.77
Q15172	Serine/threonine-protein phosphatase 2A 56 kDa regulatory subunit alpha isoform OS=Homo sapiens GN=PPP2R5A PE=1 SV=1 - [2A5A_HUMAN]	15.02	1	5	7	16	1.266	1.103	1.451	1.248	45.28	15.02	11	16	486	56.2	6.71
Q2QGD7	Zinc finger protein ZXDC OS=Homo sapiens GN=ZXDC PE=1 SV=2 - [ZXDC_HUMAN]	1.86	1	2	2	5	1.364	0.919	1.098	1.249	11.57	1.86	2	5	858	89.9	8.13
Q9UJS0	Calcium-binding mitochondrial carrier protein Aralar2 OS=Homo sapiens GN=SLC25A13 PE=1 SV=2 - [CMC2_HUMAN]	22.96	1	8	12	32	1.320	0.947	0.839	1.249	70.04	22.96	17	32	675	74.1	8.62
Q09328	Alpha-1,6-mannosylglycoprotein 6-beta-N-acetylglucosaminyltransferase A OS=Homo sapiens GN=MGAT5 PE=1 SV=1 - [MGT5A_HUMAN]	14.71	1	10	10	23	2.020	0.504	0.998	1.249	40.28	14.71	15	23	741	84.5	8.12
Q99986	Serine/threonine-protein kinase VRK1 OS=Homo sapiens GN=VRK1 PE=1 SV=1 - [VRK1_HUMAN]	7.07	1	3	3	4	1.216	1.382	0.938	1.249	12.37	7.07	4	4	396	45.4	8.91

Q969U6	F-box/WD repeat-containing protein 5 OS=Homo sapiens GN=FBXW5 PE=1 SV=1 - [FBXW5_HUMAN]	2.65	1	1	1	1	0.832	0.779	1.617	1.249	0.00	2.65	1	1	566	63.9	6.33
Q9BRG1	Vacuolar protein-sorting-associated protein 25 OS=Homo sapiens GN=VPS25 PE=1 SV=1 - [VPS25_HUMAN]	25.00	1	5	5	18	0.950	1.116	1.002	1.249	31.73	25.00	7	18	176	20.7	6.34
Q12972	Nuclear inhibitor of protein phosphatase 1 OS=Homo sapiens GN=PPP1R8 PE=1 SV=2 - [PP1R8_HUMAN]	33.33	1	9	9	22	0.966	1.131	1.017	1.249	58.99	33.33	15	22	351	38.5	7.37
Q9Y5L4	Mitochondrial import inner membrane translocase subunit Tim13 OS=Homo sapiens GN=TIMM13 PE=1 SV=1 - [TIM13_HUMAN]	27.37	1	2	2	12	0.932	1.108	0.747	1.249	16.63	27.37	3	12	95	10.5	8.18
P10253	Lysosomal alpha-glucosidase OS=Homo sapiens GN=GAA PE=1 SV=4 - [LYAG_HUMAN]	24.05	1	17	17	82	0.913	1.066	0.884	1.249	213.76	24.05	27	82	952	105.3	6.00
Q8NBF6	Late secretory pathway protein AVL9 homolog OS=Homo sapiens GN=AVL9 PE=1 SV=1 - [AVL9_HUMAN]	8.02	1	5	5	10	0.924	0.908	0.869	1.249	18.93	8.02	8	10	648	71.9	6.21
O43861	Probable phospholipid-transporting ATPase IIB OS=Homo sapiens GN=ATP9B PE=2 SV=4 - [ATP9B_HUMAN]	5.41	1	4	4	12	1.230	0.902	1.120	1.250	21.78	5.41	7	12	1147	129.2	7.61
Q9GZT3	SRA stem-loop-interacting RNA-binding protein, mitochondrial OS=Homo sapiens GN=SLIRP PE=1 SV=1 - [SLIRP_HUMAN]	52.29	1	5	5	21	0.903	0.813	1.119	1.250	49.92	52.29	9	21	109	12.3	10.24

Q9UBH6	Xenotropic and polytropic retrovirus receptor 1 OS=Homo sapiens GN=XPR1 PE=1 SV=1 - [XPR1_HUMAN]	5.75	1	3	3	7	1.193	0.792	0.969	1.250	12.45	5.75	5	7	696	81.5	8.44
Q9HAN9	Nicotinamide mononucleotide adenylyltransferase 1 OS=Homo sapiens GN=NMNAT1 PE=1 SV=1 - [NMNA1_HUMA	12.90	1	3	4	10	0.946	1.252	0.910	1.250	14.80	12.90	6	10	279	31.9	8.87
Q92620	Pre-mRNA-splicing factor ATP-dependent RNA helicase PRP16 OS=Homo sapiens GN=DHX38 PE=1 SV=2 - [PRP16_HUMAN	6.03	1	6	6	15	1.000	1.029	1.006	1.250	20.81	6.03	10	15	1227	140.4	6.54
P82664	28S ribosomal protein S10, mitochondrial OS=Homo sapiens GN=MRPS10 PE=1 SV=2 - [RT10_HUMAN]	14.93	1	2	2	6	0.729	1.556	1.141	1.250	19.02	14.93	3	6	201	23.0	8.00
Q9NR30	Nucleolar RNA helicase 2 OS=Homo sapiens GN=DDX21 PE=1 SV=5 - [DDX21_HUMAN]	23.88	1	15	18	55	0.724	0.751	0.726	1.251	120.09	23.88	28	55	783	87.3	9.28
O00264	Membrane-associated progesterone receptor component 1 OS=Homo sapiens GN=PGRMC1 PE=1 SV=3 - [PGRC1_HUMAN]	52.31	1	9	11	68	1.031	0.891	1.054	1.251	172.72	52.31	18	68	195	21.7	4.70
P48047	ATP synthase subunit O, mitochondrial OS=Homo sapiens GN=ATP5O PE=1 SV=1 - [ATPO_HUMAN]	46.48	3	10	10	54	1.254	1.158	0.920	1.251	111.67	46.48	16	54	213	23.3	9.96
Q13243	Serine/arginine-rich splicing factor 5 OS=Homo sapiens GN=SRSF5 PE=1 SV=1 - [SRSF5_HUMAN]	13.97	1	2	3	22	1.132	1.622	1.156	1.251	53.98	13.97	6	22	272	31.2	11.59

P51159	Ras-related protein Rab-27A OS=Homo sapiens GN=RAB27A PE=1 SV=3 - [RB27A_HUMAN]	10.86	1	2	2	7	1.129	1.514	0.837	1.251	12.39	10.86	4	7	221	24.9	5.22
Q12769	Nuclear pore complex protein Nup160 OS=Homo sapiens GN=NUP160 PE=1 SV=3 - [NU160_HUMAN	2.79	1	4	4	9	0.913	0.982	1.006	1.251	13.26	2.79	7	9	1436	162.0	5.50
P14778	Interleukin-1 receptor type 1 OS=Homo sapiens GN=IL1R1 PE=1 SV=1 - [IL1R1_HUMAN]	1.58	1	1	1	3	0.943	1.003	1.686	1.252	4.87	1.58	2	3	569	65.4	7.83
P08107	Heat shock 70 kDa protein 1A/1B OS=Homo sapiens GN=HSPA1A PE=1 SV=5 - [HSP71_HUMAN]	58.81	2	20	36	824	1.405	1.291	1.192	1.252	1808.32	58.81	65	824	641	70.0	5.66
P13010	X-ray repair cross-complementing protein 5 OS=Homo sapiens GN=XRCC5 PE=1 SV=3 - [XRCC5_HUMAN]	54.37	1	33	34	184	1.171	1.116	0.878	1.253	457.18	54.37	55	184	732	82.7	5.81
Q8NFD5	AT-rich interactive domain-containing protein 1B OS=Homo sapiens GN=ARID1B PE=1 SV=2 - [ARI1B_HUMAN]	6.89	1	10	11	30	1.035	1.521	1.249	1.253	86.91	6.89	15	30	2236	236.0	6.73
O75865	Trafficking protein particle complex subunit 6A OS=Homo sapiens GN=TRAPPC6A PE=1 SV=2 - [TPC6A_HUMAN]	9.43	1	2	2	4	0.957	1.101	1.268	1.253	8.10	9.43	3	4	159	17.6	5.26
P55082	Microfibril-associated glycoprotein 3 OS=Homo sapiens GN=MFAP3 PE=2 SV=1 - [MFAP3_HUMAN]	1.66	2	1	1	1	1.053	0.724	0.818	1.254	1.64	1.66	1	1	362	40.1	5.00

P17096	High mobility group protein HMG-I/HMG-Y OS=Homo sapiens GN=HMGA1 PE=1 SV=3 - [HMGA1_HUMAN]	38.32	1	6	6	38	0.882	2.539	3.226	1.254	86.59	38.32	11	38	107	11.7	10.32
Q8WXF1	Paraspeckle component 1 OS=Homo sapiens GN=PSPC1 PE=1 SV=1 - [PSPC1_HUMAN]	23.14	1	13	14	83	1.088	1.314	0.988	1.254	184.16	23.14	26	83	523	58.7	6.67
O75155	Cullin-associated NEDD8-dissociated protein 2 OS=Homo sapiens GN=CAND2 PE=1 SV=3 - [CAND2_HUMAN	6.39	1	3	8	24	0.898	1.924	1.765	1.254	44.53	6.39	12	24	1236	135.2	5.68
Q86UU0	B-cell CLL/lymphoma 9-like protein OS=Homo sapiens GN=BCL9L PE=1 SV=1 - [BCL9L_HUMAN]	7.20	1	9	10	24	0.819	1.143	0.962	1.254	47.24	7.20	14	24	1499	157.0	8.63
Q13123	Protein Red OS=Homo sapiens GN=IK PE=1 SV=3 - [RED_HUMAN]	20.65	1	11	11	29	1.225	1.353	1.131	1.254	62.58	20.65	13	29	557	65.6	6.64
Q9UBT2	SUMO-activating enzyme subunit 2 OS=Homo sapiens GN=UBA2 PE=1 SV=2 - [SAE2_HUMAN]	35.16	1	19	19	59	1.023	1.071	0.893	1.254	154.63	35.16	31	59	640	71.2	5.29
Q96AE4	Far upstream element-binding protein 1 OS=Homo sapiens GN=FUBP1 PE=1 SV=3 - [FUBP1_HUMAN]	38.20	1	19	24	111	1.118	1.456	0.941	1.255	237.98	38.20	37	111	644	67.5	7.61
Q9NYB0	Telomeric repeat-binding factor 2-interacting protein 1 OS=Homo sapiens GN=TERF2IP PE=1 SV=1 - [TE2IP_HUMAN	26.82	1	9	9	16	0.973	1.327	1.028	1.255	27.77	26.82	11	16	399	44.2	4.73
Q9H9S4	Calcium-binding protein 39-like OS=Homo sapiens GN=CAB39L PE=1 SV=3 - [CB39L_HUMAN]	21.07	1	6	7	23	1.248	0.881	1.089	1.255	35.40	21.07	10	23	337	39.1	8.40

Q5W0Q7	SUMO-specific isopeptidase USPL1 OS=Homo sapiens GN=USPL1 PE=1 SV=1 - [USPL1_HUMAN]	1.37	1	1	1	1	0.841		0.858	1.255	3.27	1.37	1	1	1092	120.4	6.19
Q8IVS2	Malonyl-CoA-acyl carrier protein transacylase, mitochondrial OS=Homo sapiens GN=MCAT PE=1 SV=2 - [FABD_HUMAN]	16.67	1	4	4	10	1.447	1.380	1.269	1.255	21.24	16.67	5	10	390	42.9	8.72
Q8N5A5	Zinc finger CCCH-type with G patch domain-containing protein OS=Homo sapiens GN=ZGPAT PE=1 SV=3 - [ZGPAT_HUMAN	1.69	1	1	1	1	1.015	1.040	0.603	1.256	0.00	1.69	1	1	531	57.3	5.43
P17612	cAMP-dependent protein kinase catalytic subunit alpha OS=Homo sapiens GN=PRKACA PE=1 SV=2 - [KAPCA_HUMAN]	22.51	2	3	13	37	1.757	1.355	1.202	1.256	83.81	22.51	18	37	351	40.6	8.79
Q2M3C7	A-kinase anchor protein SPHKAP OS=Homo sapiens GN=SPHKAP PE=1 SV=1 - [SPKAP_HUMAN]	0.47	1	1	1	1	2.259	0.434	1.218	1.256	2.44	0.47	1	1	1700	186.3	5.14
Q8N878	FERM domain-containing protein 1 OS=Homo sapiens GN=FRMD1 PE=2 SV=2 - [FRMD1_HUMA N]	4.92	1	2	3	4	0.327	0.971	0.931	1.256	8.41	4.92	3	4	549	62.5	8.21
P28290	Sperm-specific antigen 2 OS=Homo sapiens GN=SSFA2 PE=1 SV=3 - [SSFA2_HUMAN]	13.11	1	14	14	42	1.925	0.894	0.693	1.256	98.30	13.11	20	42	1259	138.3	5.19
Q9HCN8	Stromal cell-derived factor 2-like protein 1 OS=Homo sapiens GN=SDF2L1 PE=1 SV=2 - [SDF2L_HUMAN]	12.67	1	2	2	3	0.948	1.012	1.134	1.257	6.62	12.67	3	3	221	23.6	7.03

Q13217	DnaJ homolog subfamily C member 3 OS=Homo sapiens GN=DNAJC3 PE=1 SV=1 - [DNJC3_HUMAN]	44.25	1	21	22	94	1.134	0.904	0.880	1.257	155.59	44.25	33	94	504	57.5	6.15
Q03111	Protein ENL OS=Homo sapiens GN=MLLT1 PE=1 SV=2 - [ENL_HUMAN]	6.44	1	3	3	8	0.863	1.526	1.189	1.257	20.15	6.44	5	8	559	62.0	8.59
Q9BXJ8	Transmembrane protein 120A OS=Homo sapiens GN=TMEM120A PE=2 SV=1 - [T120A_HUMAN]	4.96	1	2	2	3	1.527	0.927	0.977	1.257	2.36	4.96	3	3	343	40.6	8.98
O43819	Protein SCO2 homolog, mitochondrial OS=Homo sapiens GN=SCO2 PE=1 SV=3 - [SCO2_HUMAN]	6.39	1	2	2	7	1.337	0.961	0.981	1.257	17.72	6.39	4	7	266	29.8	8.85
O00623	Peroxisome assembly protein 12 OS=Homo sapiens GN=PEX12 PE=1 SV=1 - [PEX12_HUMAN]	4.74	1	2	2	4	0.787	1.175	1.074	1.257	4.33	4.74	3	4	359	40.8	9.11
Q7Z553	MAM domain-containing glycosylphosphatidylinositol anchor protein 2 OS=Homo sapiens GN=MDGA2 PE=1 SV=2 - [MDGA2_HUMAN]	1.67	1	1	1	1	1.272	1.772	1.140	1.258	2.83	1.67	1	1	956	107.4	7.20
Q8N302	Angiogenic factor with G patch and FHA domains 1 OS=Homo sapiens GN=AGGF1 PE=1 SV=2 - [AGGF1_HUMAN]	1.54	1	1	1	2	0.968	0.790	0.640	1.258	6.38	1.54	1	2	714	80.9	5.44
Q7Z5J4	Retinoic acid-induced protein 1 OS=Homo sapiens GN=RAI1 PE=1 SV=2 - [RAI1_HUMAN]	1.52	1	3	3	4	1.236	1.330	1.097	1.258	5.44	1.52	4	4	1906	203.2	8.79
P05165	Propionyl-CoA carboxylase alpha chain, mitochondrial OS=Homo sapiens GN=PCCA PE=1 SV=4 - [PCCA_HUMAN]	23.76	1	16	16	50	1.041	1.382	1.262	1.258	139.59	23.76	24	50	728	80.0	7.52

Q99650	Oncostatin-M-specific receptor subunit beta OS=Homo sapiens GN=OSMR PE=1 SV=1 - [OSMR_HUMAN	1.33	1	1	1	2	1.010	1.362	1.838	1.258	0.00	1.33	1	2	979	110.4	5.82
P40937	Replication factor C subunit 5 OS=Homo sapiens GN=RFC5 PE=1 SV=1 - [RFC5_HUMAN]	12.35	1	2	3	7	0.923	1.232	0.753	1.259	16.37	12.35	4	7	340	38.5	7.20
O15018	PDZ domain-containing protein 2 OS=Homo sapiens GN=PDZD2 PE=1 SV=4 - [PDZD2_HUMAN]	0.63	3	2	2	2	1.042	1.149	1.743	1.259	2.67	0.63	2	2	2839	301.5	7.43
Q8TCB7	Methyltransferase-like protein 6 OS=Homo sapiens GN=METTL6 PE=2 SV=2 - [METL6_HUMAN]	2.11	1	1	1	1	1.323	1.274	1.765	1.259	1.62	2.11	1	1	284	33.2	6.19
Q6P4A7	Sideroflexin-4 OS=Homo sapiens GN=SFXN4 PE=1 SV=1 - [SFXN4_HUMAN]	4.15	1	1	1	2	0.891	1.329	1.195	1.259	3.71	4.15	2	2	337	38.0	9.19
Q9BUQ8	Probable ATP-dependent RNA helicase DDX23 OS=Homo sapiens GN=DDX23 PE=1 SV=3 - [DDX23_HUMAN	18.78	1	14	15	38	1.112	1.005	0.887	1.260	99.36	18.78	22	38	820	95.5	9.55
O60232	Sjoegren syndrome/scleroderma autoantigen 1 OS=Homo sapiens GN=SSSCA1 PE=1 SV=1 - [SSA27_HUMAN]	7.54	1	2	2	5	0.760	1.134	1.178	1.260	10.51	7.54	3	5	199	21.5	5.24
Q86YA3	Protein ZGRF1 OS=Homo sapiens GN=ZGRF1 PE=2 SV=3 - [ZGRF1_HUMAN]	1.85	1	2	3	5	1.678	0.958	0.843	1.260	0.00	1.85	3	5	2104	236.5	6.16
P18754	Regulator of chromosome condensation OS=Homo sapiens GN=RCC1 PE=1 SV=1 - [RCC1_HUMAN]	22.33	1	8	8	43	1.105	1.228	0.885	1.260	128.21	22.33	13	43	421	44.9	7.52

Q13506	NGFI-A-binding protein 1 OS=Homo sapiens GN=NAB1 PE=1 SV=2 - [NAB1_HUMAN]	4.52	1	2	2	5	0.824	0.826	0.954	1.260	8.03	4.52	3	5	487	54.4	6.70
P24386	Rab proteins geranylgeranyltransferase component A 1 OS=Homo sapiens GN=CHM PE=1 SV=3 - [RAE1_HUMAN]	4.29	2	2	2	4	0.995	1.059	1.357	1.260	12.81	4.29	3	4	653	73.4	4.75
Q92738	USP6 N-terminal-like protein OS=Homo sapiens GN=USP6NL PE=1 SV=3 - [US6NL_HUMAN]	1.33	1	1	1	3	1.522	1.995	1.656	1.260	4.73	1.33	2	3	828	94.0	9.07
Q9Y3Z3	Deoxynucleoside triphosphate triphosphohydrolase SAMHD1 OS=Homo sapiens GN=SAMHD1 PE=1 SV=2 - [SAMH1_HUMAN]	53.04	1	28	29	97	3.493	1.766	1.034	1.260	245.64	53.04	43	97	626	72.2	7.14
P17301	Integrin alpha-2 OS=Homo sapiens GN=ITGA2 PE=1 SV=1 - [ITA2_HUMAN]	24.64	1	23	24	83	1.193	0.850	0.682	1.260	210.84	24.64	34	83	1181	129.2	5.31
Q8IZQ1	WD repeat and FYVE domain-containing protein 3 OS=Homo sapiens GN=WDFY3 PE=1 SV=2 - [WDFY3_HUMA	1.73	1	4	5	6	1.089	0.852	1.124	1.260	11.23	1.73	6	6	3526	395.0	6.76
Q9P015	39S ribosomal protein L15, mitochondrial OS=Homo sapiens GN=MRPL15 PE=1 SV=1 - [RM15_HUMAN]	30.74	1	7	8	14	1.090	1.597	0.943	1.261	27.94	30.74	12	14	296	33.4	10.01
Q9NUP1	Biogenesis of lysosome-related organelles complex 1 subunit 4 OS=Homo sapiens GN=BLOC1S4 PE=1 SV=1 - [BL1S4_HUMAN]	33.18	1	4	6	12	1.187	0.957	1.009	1.261	28.37	33.18	9	12	217	23.3	4.98

Q9HCD5	Nudear receptor coactivator 5 OS=Homo sapiens GN=NCOA5 PE=1 SV=2 - [NCOA5_HUMA	10.19	1	6	6	18	0.899	0.984	1.087	1.261	46.20	10.19	6	18	579	65.5	9.60
O43497	Voltage-dependent T-type calcium channel subunit alpha-1G OS=Homo sapiens GN=CACNA1G PE=2 SV=3 - [CAC1G_HUMAN]	0.97	1	2	2	2	1.194	0.948	0.876	1.262	5.50	0.97	2	2	2377	262.3	6.60
Q9UHL9	General transcription factor II-I repeat domain-containing protein 1 OS=Homo sapiens GN=GTF2IRD1 PE=1 SV=1 - [GT2D1_HUMAN]	0.83	1	1	1	2	0.965	0.926	1.130	1.262	3.93	0.83	1	2	959	106.0	6.87
Q9H0L4	Cleavage stimulation factor subunit 2 tau variant OS=Homo sapiens GN=CSTF2T PE=1 SV=1 - [CSTFT_HUMAN]	12.34	1	2	7	11	0.900	1.457	1.017	1.262	11.89	12.34	9	11	616	64.4	7.25
Q7Z3T8	Zinc finger FYVE domain-containing protein 16 OS=Homo sapiens GN=ZFYVE16 PE=1 SV=3 - [ZFY16_HUMAN]	4.61	1	8	8	10	1.099	0.732	0.803	1.262	16.37	4.61	9	10	1539	168.8	4.82
Q96PE7	Methylmalonyl-CoA epimerase, mitochondrial OS=Homo sapiens GN=MCEE PE=1 SV=1 - [MCEE_HUMAN]	13.07	1	2	2	6	1.008	1.265	0.964	1.262	4.71	13.07	4	6	176	18.7	9.09
P28340	DNA polymerase delta catalytic subunit OS=Homo sapiens GN=POLD1 PE=1 SV=2 - [DPOD1_HUMA	4.43	1	4	5	14	0.953	2.802	1.242	1.263	19.02	4.43	7	14	1107	123.6	7.03
Q9UKJ3	G patch domain-containing protein 8 OS=Homo sapiens GN=GPATCH8 PE=1 SV=2 - [GPTC8_HUMAN	2.20	1	3	3	5	0.953	1.421	1.097	1.263	7.16	2.20	4	5	1502	164.1	8.66

P29401	Transketolase OS=Homo sapiens GN=TKT PE=1 SV=3 - [TKT_HUMAN]	53.29	1	33	34	451	1.552	1.012	1.052	1.263	1010.11	53.29	61	451	623	67.8	7.66
Q07817	Bcl-2-like protein 1 OS=Homo sapiens GN=BCL2L1 PE=1 SV=1 - [B2CL1_HUMAN]	3.86	1	1	1	9	1.037	1.220	0.806	1.263	26.81	3.86	1	9	233	26.0	4.93
Q96L92	Sorting nexin-27 OS=Homo sapiens GN=SNX27 PE=1 SV=2 - [SNX27_HUMAN]	16.82	1	7	7	21	1.238	0.911	1.069	1.263	46.70	16.82	12	21	541	61.2	6.49
Q9UBD0	Heat shock transcription factor, X-linked OS=Homo sapiens GN=HSFX1 PE=1 SV=1 - [HSFX1_HUMAN]	1.89	1	1	1	1	0.832	0.678	0.959	1.263	1.95	1.89	1	1	423	46.7	7.11
Q9H9J2	39S ribosomal protein L44, mitochondrial OS=Homo sapiens GN=MRPL44 PE=1 SV=1 - [RM44_HUMAN]	26.51	1	9	9	22	1.069	1.113	0.964	1.263	55.80	26.51	12	22	332	37.5	8.40
O00566	U3 small nucleolar ribonucleoprotein protein MPP10 OS=Homo sapiens GN=MPHOSPH10 PE=1 SV=2 - [MPP10_HUMAN]	5.58	1	3	4	20	0.930	1.093	0.998	1.264	59.75	5.58	5	20	681	78.8	4.86
Q9C0D5	Protein TANC1 OS=Homo sapiens GN=TANC1 PE=1 SV=3 - [TANC1_HUMAN]	5.37	1	8	9	15	1.158	0.903	1.062	1.264	22.33	5.37	11	15	1861	202.1	8.32
P19971	Thymidine phosphorylase OS=Homo sapiens GN=TYMP PE=1 SV=2 - [TYPH_HUMAN]	29.46	1	11	11	48	3.954	1.095	0.753	1.264	129.88	29.46	20	48	482	49.9	5.53
Q9Y3C4	EKC/KEOPS complex subunit TPRKB OS=Homo sapiens GN=TPRKB PE=1 SV=1 - [TPRKB_HUMAN]	28.57	1	4	4	7	1.053	1.065	0.847	1.264	18.72	28.57	6	7	175	19.6	6.79

Q7Z3B4	Nudeoporin p54 OS=Homo sapiens GN=NUP54 PE=1 SV=2 - [NUP54_HUMAN]	29.39	1	13	13	29	1.193	1.231	1.088	1.264	78.06	29.39	19	29	507	55.4	7.02
Q8IZ83	Aldehyde dehydrogenase family 16 member A1 OS=Homo sapiens GN=ALDH16A1 PE=1 SV=2 - [A16A1_HUMAN]	19.45	1	14	14	45	1.588	0.696	0.621	1.264	109.96	19.45	27	45	802	85.1	6.79
Q96PK6	RNA-binding protein 14 OS=Homo sapiens GN=RBM14 PE=1 SV=2 - [RBM14_HUMAN]	25.71	1	22	22	88	1.212	1.197	1.061	1.264	173.11	25.71	38	88	669	69.4	9.67
Q8NHR7	Uncharacterized protein C15orf43 OS=Homo sapiens GN=C15orf43 PE=2 SV=1 - [CO043_HUMAN]	7.27	1	1	1	1	0.728	1.161	1.247	1.264	0.00	7.27	1	1	220	25.3	7.59
Q9H8L6	Multimerin-2 OS=Homo sapiens GN=MMRN2 PE=1 SV=2 - [MMRN2_HUMAN]	2.32	1	2	2	2	0.695	0.953	1.376	1.265	2.34	2.32	2	2	949	104.3	5.86
P49750	YLP motif-containing protein 1 OS=Homo sapiens GN=YLPM1 PE=1 SV=3 - [YLPM1_HUMAN]	13.99	2	22	23	88	1.181	1.464	1.165	1.265	205.91	13.99	35	88	1951	219.8	6.57
Q9NRA8	Eukaryotic translation initiation factor 4E transporter OS=Homo sapiens GN=EIF4ENIF1 PE=1 SV=2 - [4ET_HUMAN]	3.96	2	4	4	7	1.133	1.527	1.323	1.265	13.84	3.96	5	7	985	108.1	8.32
Q8NCS7	Choline transporter-like protein 5 OS=Homo sapiens GN=SLC44A5 PE=2 SV=4 - [CTL5_HUMAN]	2.92	1	1	1	1	1.808	1.114	1.179	1.266	0.00	2.92	1	1	719	81.6	8.18
Q13257	Mitotic spindle assembly checkpoint protein MAD2A OS=Homo sapiens GN=MAD2L1 PE=1 SV=1 - [MD2L1_HUMAN]	3.41	1	1	1	9	1.007	1.036	0.660	1.266	20.48	3.41	2	9	205	23.5	5.08

O43670	BUB3-interacting and GLEBS motif-containing protein ZNF207 OS=Homo sapiens GN=ZNF207 PE=1 SV=1 - [ZN207_HUMAN]	11.30	1	4	4	53	1.028	1.182	0.890	1.266	121.23	11.30	7	53	478	50.7	9.10
Q9BZD4	Kinetochore protein Nuf2 OS=Homo sapiens GN=NUF2 PE=1 SV=2 - [NUF2_HUMAN]	4.53	1	2	3	5	0.856	1.070	0.567	1.266	10.74	4.53	3	5	464	54.3	8.27
O94906	Pre-mRNA-processing factor 6 OS=Homo sapiens GN=PRPF6 PE=1 SV=1 - [PRP6_HUMAN]	19.02	1	19	19	66	1.140	1.174	0.964	1.266	141.87	19.02	30	66	941	106.9	8.25
Q9UNX9	ATP-sensitive inward rectifier potassium channel 14 OS=Homo sapiens GN=KCNJ14 PE=1 SV=1 - [KCJ14_HUMAN]	4.13	1	1	1	1	0.698	1.208	1.498	1.267	0.00	4.13	1	1	436	47.8	5.41
Q9BUH6	Uncharacterized protein C9orf142 OS=Homo sapiens GN=C9orf142 PE=1 SV=2 - [CI142_HUMAN]	34.80	1	8	8	16	0.982	1.379	1.313	1.267	42.25	34.80	13	16	204	21.6	5.48
Q9UKC9	F-box/LRR-repeat protein 2 OS=Homo sapiens GN=FBXL2 PE=1 SV=3 - [FBXL2_HUMAN]	5.20	1	2	3	5	1.288	1.577	1.550	1.267	8.11	5.20	4	5	423	47.0	7.40
Q6P1J9	Parafibromin OS=Homo sapiens GN=CDC73 PE=1 SV=1 - [CDC73_HUMAN]	13.75	1	7	7	27	1.007	1.151	1.000	1.267	47.38	13.75	11	27	531	60.5	9.61
Q00059	Transcription factor A, mitochondrial OS=Homo sapiens GN=TFAM PE=1 SV=1 - [TFAM_HUMAN]	41.06	1	13	13	37	0.930	1.057	0.851	1.267	72.59	41.06	21	37	246	29.1	9.72
P56381	ATP synthase subunit epsilon, mitochondrial OS=Homo sapiens GN=ATP5E PE=1 SV=2 - [ATP5E_HUMAN	45.10	2	3	3	5	1.341	1.358	0.852	1.267	12.30	45.10	4	5	51	5.8	9.92

O94905	Erlin-2 OS=Homo sapiens GN=ERLIN2 PE=1 SV=1 - [ERLN2_HUMAN]	41.30	1	12	18	106	1.058	0.754	0.662	1.268	271.14	41.30	29	106	339	37.8	5.62
Q8N4T8	Carbonyl reductase family member 4 OS=Homo sapiens GN=CBR4 PE=1 SV=3 - [CBR4_HUMAN]	3.38	1	1	1	2	1.899	1.093	0.862	1.268	1.92	3.38	2	2	237	25.3	9.33
Q6KF10	Growth/differentiation factor 6 OS=Homo sapiens GN=GDF6 PE=1 SV=1 - [GDF6_HUMAN]	4.18	1	1	1	1	1.398	1.675	1.177	1.268	2.58	4.18	1	1	455	50.6	8.88
Q969Z0	Protein TBRG4 OS=Homo sapiens GN=TBRG4 PE=1 SV=1 - [TBRG4_HUMAN]	13.63	1	8	8	16	0.978	1.007	1.036	1.268	31.70	13.63	12	16	631	70.7	7.42
Q99836	Myeloid differentiation primary response protein MyD88 OS=Homo sapiens GN=MYD88 PE=1 SV=1 - [MYD88_HUMAN]	22.64	1	5	5	12	1.238	1.078	1.162	1.268	26.17	22.64	8	12	296	33.2	6.15
Q01130	Serine/arginine-rich splicing factor 2 OS=Homo sapiens GN=SRSF2 PE=1 SV=4 - [SRSF2_HUMAN]	14.48	2	3	3	32	1.024	1.187	1.008	1.268	62.65	14.48	6	32	221	25.5	11.85
P59045	NACHT, LRR and PYD domains-containing protein 11 OS=Homo sapiens GN=NLRP11 PE=2 SV=2 - [NAL11_HUMAN]	2.42	1	1	2	2	1.018	1.509	1.621	1.268	2.17	2.42	2	2	1033	117.7	7.71
Q14865	AT-rich interactive domain-containing protein 5B OS=Homo sapiens GN=ARID5B PE=1 SV=3 - [ARI5B_HUMAN]	5.47	1	6	6	6	1.334	1.440	1.012	1.268	10.81	5.47	6	6	1188	132.3	8.72
P61769	Beta-2-microglobulin OS=Homo sapiens GN=B2M PE=1 SV=1 - [B2MG_HUMAN]	43.70	1	5	5	80	2.265	0.872	0.998	1.269	189.32	43.70	8	80	119	13.7	6.52

Q9NVM9	Protein asunder homolog OS=Homo sapiens GN=ASUN PE=1 SV=2 - [ASUN_HUMAN]	7.22	1	5	5	12	1.207	1.068	0.890	1.269	21.66	7.22	8	12	706	80.2	6.70
Q6ZMU5	Tripartite motif-containing protein 72 OS=Homo sapiens GN=TRIM72 PE=1 SV=2 - [TRI72_HUMAN]	3.77	1	1	1	1	1.290	0.712	1.955	1.269	2.61	3.77	1	1	477	52.7	6.48
O75382	Tripartite motif-containing protein 3 OS=Homo sapiens GN=TRIM3 PE=1 SV=2 - [TRIM3_HUMAN]	4.97	1	3	3	6	1.384	1.299	1.261	1.270	13.55	4.97	5	6	744	80.8	7.83
O94776	Metastasis-associated protein MTA2 OS=Homo sapiens GN=MTA2 PE=1 SV=1 - [MTA2_HUMAN]	18.56	1	8	12	33	1.036	1.170	0.937	1.270	61.58	18.56	21	33	668	75.0	9.66
Q96F25	UDP-N-acetylglucosamine transferase subunit ALG14 homolog OS=Homo sapiens GN=ALG14 PE=2 SV=1 - [ALG14_HUMAN]	5.09	1	1	1	2	1.132	1.270	0.895	1.270	5.93	5.09	1	2	216	24.1	8.94
O95347	Structural maintenance of chromosomes protein 2 OS=Homo sapiens GN=SMC2 PE=1 SV=2 - [SMC2_HUMAN]	17.13	1	20	20	50	1.154	1.635	0.777	1.270	128.74	17.13	31	50	1197	135.6	8.43
Q9NXV6	CDKN2A-interacting protein OS=Homo sapiens GN=CDKN2AIP PE=1 SV=3 - [CARF_HUMAN]	14.48	1	6	6	14	1.171	1.837	1.164	1.271	25.36	14.48	10	14	580	61.1	9.01
P78406	mRNA export factor OS=Homo sapiens GN=RAE1 PE=1 SV=1 - [RAE1L_HUMAN]	51.90	1	16	17	51	1.103	1.050	1.033	1.271	124.47	51.90	28	51	368	40.9	7.83

O14949	Cytochrome b-c1 complex subunit 8 OS=Homo sapiens GN=UQCRQ PE=1 SV=4 - [QCR8_HUMAN]	37.80	1	3	3	26	1.155	1.167	0.851	1.271	69.06	37.80	6	26	82	9.9	10.08
Q15365	Poly(rC)-binding protein 1 OS=Homo sapiens GN=PCBP1 PE=1 SV=2 - [PCBP1_HUMAN]	41.57	1	7	13	153	1.086	1.076	0.986	1.271	419.29	41.57	24	153	356	37.5	7.09
P52735	Guanine nucleotide exchange factor VAV2 OS=Homo sapiens GN=VAV2 PE=1 SV=2 - [VAV2_HUMAN]	9.79	1	7	8	25	1.346	0.849	1.113	1.271	63.21	9.79	13	25	878	101.2	7.08
Q9BPZ7	Target of rapamycin complex 2 subunit MAPKAP1 OS=Homo sapiens GN=MAPKAP1 PE=1 SV=2 - [SIN1_HUMAN]	2.87	1	1	2	3	1.422	1.246	1.381	1.272	4.69	2.87	2	3	522	59.1	7.55
Q6UWP2	Dehydrogenase/reductase SDR family member 11 OS=Homo sapiens GN=DHRS11 PE=1 SV=1 - [DHR11_HUMAN]	4.62	1	1	1	1	1.077	1.187	1.136	1.272	2.81	4.62	1	1	260	28.3	6.64
A0A087WVF3	TBC1 domain family member 3D OS=Homo sapiens GN=TBC1D3D PE=2 SV=1 - [TBC3D_HUMAN]	3.28	10	1	1	1	0.584	0.526	1.061	1.272	0.00	3.28	1	1	549	62.1	9.01
Q5JU69	Torsin-2A OS=Homo sapiens GN=TOR2A PE=2 SV=1 - [TOR2A_HUMAN]	9.97	1	1	1	1				1.273	0.00	9.97	1	1	321	35.7	7.97
Q99417	C-Myc-binding protein OS=Homo sapiens GN=MYCBP PE=1 SV=3 - [MYCBP_HUMAN]	52.43	1	5	5	27	1.220	1.061	0.977	1.273	66.92	52.43	8	27	103	12.0	5.91
P03928	ATP synthase protein 8 OS=Homo sapiens GN=MT-ATP8 PE=1 SV=1 - [ATP8_HUMAN]	72.06	1	4	4	10	1.019	0.919	0.923	1.273	4.50	72.06	6	10	68	8.0	9.91

Q9NYB9	Abl interactor 2 OS=Homo sapiens GN=ABI2 PE=1 SV=1 - [ABI2_HUMAN]	18.91	1	5	8	22	1.173	1.259	1.425	1.273	44.03	18.91	13	22	513	55.6	6.16
Q69YN2	CWF19-like protein 1 OS=Homo sapiens GN=CWF19L1 PE=1 SV=2 - [C19L1_HUMAN]	3.35	1	2	2	3	0.577	1.141	1.093	1.273	5.29	3.35	2	3	538	60.6	7.24
Q8WUM0	Nuclear pore complex protein Nup133 OS=Homo sapiens GN=NUP133 PE=1 SV=2 - [NU133_HUMAN	12.37	1	14	14	44	1.130	1.081	0.965	1.273	100.17	12.37	25	44	1156	128.9	5.10
P06396	Gelsolin OS=Homo sapiens GN=GSN PE=1 SV=1 - [GELS_HUMAN]	51.79	1	31	31	413	1.410	1.371	1.548	1.274	1111.53	51.79	48	413	782	85.6	6.28
O15155	BET1 homolog OS=Homo sapiens GN=BET1 PE=1 SV=1 - [BET1_HUMAN]	15.25	1	2	2	4	0.813	2.195	1.408	1.274	12.81	15.25	3	4	118	13.3	9.06
P31930	Cytochrome b-c1 complex subunit 1, mitochondrial OS=Homo sapiens GN=UQCRC1 PE=1 SV=3 - [QCR1_HUMAN]	44.17	1	15	16	82	1.057	1.186	0.818	1.274	203.80	44.17	25	82	480	52.6	6.37
O14646	Chromodomain-helicase-DNA-binding protein 1 OS=Homo sapiens GN=CHD1 PE=1 SV=2 - [CHD1_HUMAN]	3.63	1	2	4	4	0.825	1.088	1.599	1.274	9.50	3.63	4	4	1710	196.6	7.14
Q96EK9	Protein KTI12 homolog OS=Homo sapiens GN=KTI12 PE=1 SV=1 - [KTI12_HUMAN]	12.43	1	4	4	8	0.808	0.839	1.021	1.274	17.99	12.43	5	8	354	38.6	6.95
Q01831	DNA repair protein complementing XP-C cells OS=Homo sapiens GN=XPC PE=1 SV=4 - [XPC_HUMAN]	7.45	1	7	7	15	1.136	1.435	0.873	1.274	33.31	7.45	10	15	940	105.9	8.90

P35249	Replication factor C subunit 4 OS=Homo sapiens GN=RFC4 PE=1 SV=2 - [RFC4_HUMAN]	17.63	1	5	5	8	1.348	1.397	1.003	1.274	14.17	17.63	7	8	363	39.7	8.02
Q9H307	Pinin OS=Homo sapiens GN=PNN PE=1 SV=4 - [PININ_HUMAN]	21.90	2	16	18	59	1.192	1.102	1.035	1.274	141.47	21.90	27	59	717	81.6	7.14
Q8IYX3	Coiled-coil domain-containing protein 116 OS=Homo sapiens GN=CCDC116 PE=2 SV=2 - [CC116_HUMAN]	3.50	1	1	2	2	1.015	0.514	1.351	1.275	2.02	3.50	2	2	515	56.9	9.64
Q9Y240	C-type lectin domain family 11 member A OS=Homo sapiens GN=CLEC11A PE=1 SV=1 - [CLC11_HUMAN]	8.05	1	2	2	8	0.571	0.460	0.526	1.275	19.67	8.05	4	8	323	35.7	5.16
Q49AR2	UPF0489 protein C5orf22 OS=Homo sapiens GN=C5orf22 PE=1 SV=2 - [CE022_HUMAN]	1.58	1	1	1	2	0.925	0.819	1.121	1.275	2.28	1.58	1	2	442	49.9	4.78
Q07820	Induced myeloid leukemia cell differentiation protein Mcl-1 OS=Homo sapiens GN=MCL1 PE=1 SV=3 - [MCL1_HUMAN]	5.71	1	1	1	4	0.958	1.000	0.838	1.276	17.13	5.71	1	4	350	37.3	5.66
Q96B26	Exosome complex component RRP43 OS=Homo sapiens GN=EXOSC8 PE=1 SV=1 - [EXOS8_HUMAN]	2.90	1	1	1	2	1.331	1.416	0.868	1.276	2.86	2.90	1	2	276	30.0	5.30
Q99719	Septin-5 OS=Homo sapiens GN=SEPT5 PE=1 SV=1 - [SEPT5_HUMAN]	1.90	1	1	1	2	1.460	0.890	1.225	1.276	3.61	1.90	1	2	369	42.7	6.67
Q7LBC6	Lysine-specific demethylase 3B OS=Homo sapiens GN=KDM3B PE=1 SV=2 - [KDM3B_HUMAN]	6.47	1	7	8	15	1.310	0.966	1.093	1.276	30.59	6.47	12	15	1761	191.5	7.18

Q5D0E6	DALR anticodon-binding domain-containing protein 3 OS=Homo sapiens GN=DALRD3 PE=2 SV=2 - [DALD3_HUMAN]	1.47	1	1	1	1	0.659	1.322	1.220	1.276	2.48	1.47	1	1	543	59.3	7.42
Q9BRR6	ADP-dependent glucokinase OS=Homo sapiens GN=ADPGK PE=1 SV=1 - [ADPGK_HUMAN]	15.09	1	5	5	14	1.155	0.737	0.771	1.276	31.93	15.09	9	14	497	54.1	6.20
Q8IY37	Probable ATP-dependent RNA helicase DHX37 OS=Homo sapiens GN=DHX37 PE=1 SV=1 - [DHX37_HUMAN	2.59	1	2	3	5	0.949	1.424	1.443	1.276	13.55	2.59	4	5	1157	129.5	8.10
Q9BRP4	Proteasomal ATPase-associated factor 1 OS=Homo sapiens GN=PAAF1 PE=1 SV=2 - [PAAF1_HUMAN	8.16	1	3	3	6	1.003	1.079	1.160	1.276	8.37	8.16	4	6	392	42.2	6.32
P23276	Kell blood group glycoprotein OS=Homo sapiens GN=KEL PE=1 SV=2 - [KELL_HUMAN]	3.69	1	2	2	2	0.825	1.473	2.049	1.276	0.00	3.69	2	2	732	82.8	7.90
Q13901	Nuclear nucleic acid-binding protein C1D OS=Homo sapiens GN=C1D PE=1 SV=1 - [C1D_HUMAN]	9.22	2	1	2	3	0.730	1.186	1.314	1.277	5.32	9.22	3	3	141	16.0	9.03
P28370	Probable global transcription activator SNF2L1 OS=Homo sapiens GN=SMARCA1 PE=1 SV=2 - [SMCA1_HUMA	14.80	1	13	17	41	1.057	1.115	1.052	1.277	80.35	14.80	24	41	1054	122.5	8.09
Q01638	Interleukin-1 receptor-like 1 OS=Homo sapiens GN=IL1RL1 PE=1 SV=4 - [ILRL1_HUMAN]	1.08	1	1	1	1	1.132	0.978	1.410	1.277	2.05	1.08	1	1	556	63.3	8.35

Q9H479	Fructosamine-3-kinase OS=Homo sapiens GN=FN3K PE=1 SV=1 - [FN3K_HUMAN]	7.12	1	2	2	5	1.111	1.895	1.110	1.277	15.56	7.12	2	5	309	35.1	7.55
Q9Y2R0	Cytochrome c oxidase assembly factor 3 homolog, mitochondrial OS=Homo sapiens GN=COA3 PE=1 SV=1 - [COA3_HUMAN]	27.36	1	3	3	7	0.943	0.935	0.791	1.277	22.30	27.36	5	7	106	11.7	9.60
Q92922	SWI/SNF complex subunit SMARCC1 OS=Homo sapiens GN=SMARCC1 PE=1 SV=3 - [SMRC1_HUMAN]	13.21	1	9	15	33	1.093	1.274	0.891	1.277	83.95	13.21	22	33	1105	122.8	5.76
Q9Y3M8	StAR-related lipid transfer protein 13 OS=Homo sapiens GN=STARD13 PE=1 SV=2 - [STA13_HUMAN]	7.10	1	6	8	25	1.677	0.658	1.061	1.277	65.83	7.10	13	25	1113	124.9	7.02
Q99459	Cell division cycle 5-like protein OS=Homo sapiens GN=CDC5L PE=1 SV=2 - [CDC5L_HUMAN]	36.66	1	21	22	47	1.147	1.233	1.023	1.277	124.63	36.66	30	47	802	92.2	8.18
Q4KMZ1	IQ domain-containing protein C OS=Homo sapiens GN=IQCC PE=2 SV=2 - [IQCC_HUMAN]	1.72	1	1	1	10	1.696	1.972	1.548	1.278	19.17	1.72	2	10	466	53.0	7.99
Q8NFH3	Nucleoporin Nup43 OS=Homo sapiens GN=NUP43 PE=1 SV=1 - [NUP43_HUMAN]	19.74	1	6	6	20	1.161	0.948	0.886	1.279	39.85	19.74	12	20	380	42.1	5.63
P00533	Epidermal growth factor receptor OS=Homo sapiens GN=EGFR PE=1 SV=2 - [EGFR_HUMAN]	19.26	1	20	22	64	1.269	0.864	1.030	1.279	160.03	19.26	34	64	1210	134.2	6.68

O14735	CDP-diacylglycerol--inositol 3-phosphatidyltransferase OS=Homo sapiens GN=CDIPT PE=1 SV=1 - [CDIPT_HUMAN]	9.86	1	2	2	7	0.980	0.754	0.748	1.279	10.94	9.86	4	7	213	23.5	8.03
Q9C0E8	Protein lunapark OS=Homo sapiens GN=LNP PE=1 SV=2 - [LNP_HUMAN]	23.36	1	8	8	25	1.311	1.277	1.310	1.279	65.42	23.36	14	25	428	47.7	5.11
Q06787	Fragile X mental retardation protein 1 OS=Homo sapiens GN=FMR1 PE=1 SV=1 - [FMR1_HUMAN]	24.68	1	11	15	44	1.352	1.372	1.093	1.279	96.98	24.68	21	44	632	71.1	7.42
P21926	CD9 antigen OS=Homo sapiens GN=CD9 PE=1 SV=4 - [CD9_HUMAN]	13.60	1	5	5	22	3.916	1.350	2.138	1.279	42.81	13.60	7	22	228	25.4	7.15
Q86YV5	Tyrosine-protein kinase SgK223 OS=Homo sapiens GN=SGK223 PE=1 SV=4 - [SG223_HUMAN]	0.92	1	1	1	1	1.011	1.999	1.458	1.280	3.22	0.92	1	1	1406	149.5	7.20
P09874	Poly [ADP-ribose] polymerase 1 OS=Homo sapiens GN=PARP1 PE=1 SV=4 - [PARP1_HUMAN]	38.56	1	37	37	109	1.231	1.564	1.149	1.280	268.09	38.56	56	109	1014	113.0	8.88
Q969S8	Histone deacetylase 10 OS=Homo sapiens GN=HDAC10 PE=1 SV=1 - [HDA10_HUMAN]	1.20	1	1	1	4	1.273	1.271	1.205	1.280	15.53	1.20	2	4	669	71.4	5.74
Q9NQX3	Gephyrin OS=Homo sapiens GN=GPHN PE=1 SV=1 - [GEPH_HUMAN]	14.27	1	8	8	21	1.168	1.051	1.015	1.280	45.27	14.27	13	21	736	79.7	5.43
Q6ZSZ5	Rho guanine nucleotide exchange factor 18 OS=Homo sapiens GN=ARHGEF18 PE=1 SV=3 - [ARHGI_HUMAN	2.64	1	3	4	8	1.290	1.047	1.119	1.280	14.61	2.64	4	8	1173	130.7	7.08

Q8TCC3	39S ribosomal protein L30, mitochondrial OS=Homo sapiens GN=MRPL30 PE=1 SV=1 - [RM30_HUMAN]	10.56	1	1	1	1	1.531	1.619	1.213	1.280	2.20	10.56	1	1	161	18.5	9.99
Q96EX3	WD repeat-containing protein 34 OS=Homo sapiens GN=WDR34 PE=1 SV=2 - [WDR34_HUMAN]	1.87	1	1	1	1	1.543	1.131	0.826	1.280	0.00	1.87	1	1	536	57.8	6.64
O43318	Mitogen-activated protein kinase kinase kinase 7 OS=Homo sapiens GN=MAP3K7 PE=1 SV=1 - [M3K7_HUMAN]	10.56	1	6	6	17	0.970	1.219	1.317	1.281	32.08	10.56	11	17	606	67.2	7.11
Q9Y320	Thioredoxin-related transmembrane protein 2 OS=Homo sapiens GN=TMX2 PE=1 SV=1 - [TMX2_HUMAN]	6.08	1	2	2	10	1.086	0.579	0.768	1.281	26.51	6.08	4	10	296	34.0	8.69
P33076	MHC class II transactivator OS=Homo sapiens GN=CIITA PE=1 SV=3 - [C2TA_HUMAN]	1.33	1	1	1	1			0.598	1.281	2.22	1.33	1	1	1130	123.4	5.47
Q709F0	Acyl-CoA dehydrogenase family member 11 OS=Homo sapiens GN=ACAD11 PE=1 SV=2 - [ACD11_HUMAN]	10.38	1	6	6	13	1.012	1.326	1.497	1.281	30.45	10.38	8	13	780	87.2	8.12
O60308	Centrosomal protein of 104 kDa OS=Homo sapiens GN=CEP104 PE=1 SV=1 - [CE104_HUMAN]	3.46	1	2	3	4	1.609	0.686	1.555	1.281	4.54	3.46	3	4	925	104.4	7.62
Q9Y2U5	Mitogen-activated protein kinase kinase kinase 2 OS=Homo sapiens GN=MAP3K2 PE=1 SV=2 - [M3K2_HUMAN]	6.79	1	3	4	9	0.939	0.722	0.926	1.281	17.00	6.79	6	9	619	69.7	8.00
Q9Y5G8	Protocadherin gamma-A5 OS=Homo sapiens GN=PCDHGA5 PE=2 SV=1 - [PCDG5_HUMAN]	4.83	13	1	3	5				1.282	11.04	4.83	4	5	931	100.9	4.87

Q9BUT1	3-hydroxybutyrate dehydrogenase type 2 OS=Homo sapiens GN=BDH2 PE=1 SV=2 - [BDH2_HUMAN]	28.57	1	7	8	36	1.642	0.847	0.685	1.282	79.80	28.57	13	36	245	26.7	7.65
Q9HBF4	Zinc finger FYVE domain-containing protein 1 OS=Homo sapiens GN=ZFYVE1 PE=1 SV=1 - [ZFYV1_HUMAN]	2.96	1	2	2	2	0.522		0.846	1.282	5.89	2.96	2	2	777	87.1	7.42
Q13444	Disintegrin and metalloproteinase domain-containing protein 15 OS=Homo sapiens GN=ADAM15 PE=1 SV=4 - [ADA15_HUMAN]	4.63	1	4	4	6	0.878	0.714	0.904	1.282	12.57	4.63	5	6	863	92.9	6.73
Q9BXI6	TBC1 domain family member 10A OS=Homo sapiens GN=TBC1D10A PE=1 SV=1 - [TB10A_HUMAN]	23.62	1	9	9	30	1.650	0.966	1.114	1.282	48.65	23.62	15	30	508	57.1	8.44
Q969G3	SWI/SNF-related matrix-associated actin-dependent regulator of chromatin subfamily E member 1 OS=Homo sapiens GN=SMARCE1 PE=1 SV=2 - [SMCE1_HUMA	18.73	1	5	6	18	1.080	1.255	1.041	1.283	52.27	18.73	8	18	411	46.6	4.88
Q9UPA5	Protein bassoon OS=Homo sapiens GN=BSN PE=2 SV=4 - [BSN_HUMAN]	0.33	1	1	2	2	0.982	1.155	1.533	1.283	2.72	0.33	2	2	3926	416.2	7.55
O14497	AT-rich interactive domain-containing protein 1A OS=Homo sapiens GN=ARID1A PE=1 SV=3 - [ARI1A_HUMAN]	4.29	1	7	8	19	0.919	1.303	1.026	1.284	38.61	4.29	12	19	2285	241.9	6.70

P21912	Succinate dehydrogenase [ubiquinone] iron-sulfur subunit, mitochondrial OS=Homo sapiens GN=SDHB PE=1 SV=3 - [SDHB_HUMAN]	42.50	1	13	15	36	0.830	1.330	0.914	1.284	78.41	42.50	20	36	280	31.6	8.76
Q99523	Sortilin OS=Homo sapiens GN=SORT1 PE=1 SV=3 - [SORT_HUMAN]	6.38	1	5	5	13	1.101	0.673	1.015	1.284	23.90	6.38	9	13	831	92.0	5.74
Q7L4S7	Protein ARMCX6 OS=Homo sapiens GN=ARMCX6 PE=2 SV=1 - [ARMX6_HUMAN]	3.00	1	1	1	3	1.745	1.238	1.368	1.284	4.26	3.00	2	3	300	33.0	4.78
Q7L2E3	Putative ATP-dependent RNA helicase DHX30 OS=Homo sapiens GN=DHX30 PE=1 SV=1 - [DHX30_HUMAN	14.66	1	16	18	49	1.047	0.903	0.931	1.284	100.61	14.66	29	49	1194	133.9	8.78
Q9NVU7	Protein SDA1 homolog OS=Homo sapiens GN=SDAD1 PE=1 SV=3 - [SDA1_HUMAN]	6.40	1	5	5	7	0.637	0.775	0.696	1.284	14.26	6.40	6	7	687	79.8	9.25
O60336	Mitogen-activated protein kinase-binding protein 1 OS=Homo sapiens GN=MAPKBP1 PE=1 SV=4 - [MABP1_HUMAN]	1.39	1	2	3	5	0.547	1.340	1.354	1.285	9.90	1.39	4	5	1514	163.7	6.76
Q9NTX5	Ethylmalonyl-CoA decarboxylase OS=Homo sapiens GN=ECHDC1 PE=1 SV=2 - [ECHD1_HUMAN]	38.11	1	10	10	31	0.856	0.972	0.870	1.285	75.90	38.11	16	31	307	33.7	8.21
P06400	Retinoblastoma-associated protein OS=Homo sapiens GN=RB1 PE=1 SV=2 - [RB_HUMAN]	6.79	1	6	7	11	1.530	1.198	0.978	1.285	23.58	6.79	9	11	928	106.1	7.94
Q15029	116 kDa U5 small nuclear ribonucleoprotein component OS=Homo sapiens GN=EFTUD2 PE=1 SV=1 - [U5S1_HUMAN]	29.84	1	24	25	156	1.094	1.154	0.897	1.285	348.05	29.84	41	156	972	109.4	5.00

Q5VT52	Regulation of nuclear pre-mRNA domain-containing protein 2 OS=Homo sapiens GN=RPRD2 PE=1 SV=1 - [RPRD2_HUMAN]	6.02	1	6	6	10	1.041	1.529	1.113	1.285	21.54	6.02	7	10	1461	155.9	7.42
Q9Y2P8	RNA 3'-terminal phosphate cyclase-like protein OS=Homo sapiens GN=RCL1 PE=1 SV=3 - [RCL1_HUMAN]	6.43	1	3	3	4	0.836	0.743	0.603	1.285	8.61	6.43	4	4	373	40.8	9.26
Q6ZP01	RNA-binding protein 44 OS=Homo sapiens GN=RBM44 PE=2 SV=2 - [RBM44_HUMAN]	1.24	1	1	1	2	0.198	1.163	2.089	1.286	2.89	1.24	1	2	1051	117.9	5.72
O95785	Protein Wiz OS=Homo sapiens GN=WIZ PE=1 SV=2 - [WIZ_HUMAN]	1.51	1	1	2	2	1.083	1.981	1.192	1.286	5.15	1.51	2	2	1651	178.6	6.86
Q6P6B7	Ankyrin repeat domain-containing protein 16 OS=Homo sapiens GN=ANKRD16 PE=1 SV=1 - [ANR16_HUMAN]	3.05	1	1	1	3	1.841	0.984	0.799	1.286	6.30	3.05	2	3	361	39.3	7.25
Q86U90	YrdC domain-containing protein, mitochondrial OS=Homo sapiens GN=YRDC PE=1 SV=1 - [YRDC_HUMAN]	10.75	1	2	2	4	1.401	1.709	1.143	1.286	13.44	10.75	3	4	279	29.3	8.57
O14925	Mitochondrial import inner membrane translocase subunit Tim23 OS=Homo sapiens GN=TIMM23 PE=1 SV=1 - [TIM23_HUMAN]	5.26	2	1	1	5	1.107	0.761	0.851	1.286	5.58	5.26	2	5	209	21.9	8.60
Q01433	AMP deaminase 2 OS=Homo sapiens GN=AMPD2 PE=1 SV=2 - [AMPD2_HUMA	24.46	1	22	22	71	0.790	0.842	0.892	1.287	191.20	24.46	37	71	879	100.6	6.93

Q96G46	tRNA-dihydrouridine(47) synthase [NAD(P)(+)]-like OS=Homo sapiens GN=DUS3L PE=1 SV=2 - [DUS3L_HUMAN]	6.77	1	3	3	4	0.955	0.923	1.086	1.287	12.65	6.77	4	4	650	72.5	8.05
Q96CP2	FLYWCH family member 2 OS=Homo sapiens GN=FLYWCH2 PE=1 SV=1 - [FWCH2_HUMAN]	30.00	1	3	3	18	1.273	0.903	0.894	1.287	42.87	30.00	6	18	140	14.6	8.46
Q9BUA3	Uncharacterized protein C11orf84 OS=Homo sapiens GN=C11orf84 PE=1 SV=3 - [CK084_HUMAN]	7.61	1	2	2	5	1.377	1.125	1.283	1.287	13.27	7.61	3	5	381	41.0	5.01
Q12986	Transcriptional repressor NF-X1 OS=Homo sapiens GN=NFX1 PE=1 SV=2 - [NFX1_HUMAN]	1.88	1	2	2	3	0.836	1.042	0.918	1.287	8.12	1.88	2	3	1120	124.3	8.24
Q7Z3K3	Pogo transposable element with ZNF domain OS=Homo sapiens GN=POGZ PE=1 SV=2 - [POGZ_HUMAN]	3.40	1	4	4	10	1.134	1.389	1.178	1.288	19.21	3.40	6	10	1410	155.2	7.40
Q8N5C6	S1 RNA-binding domain-containing protein 1 OS=Homo sapiens GN=SRBD1 PE=1 SV=2 - [SRBD1_HUMAN]	0.90	1	1	1	1	1.190	1.234	1.326	1.288	2.40	0.90	1	1	995	111.7	8.72
Q9BQ61	Uncharacterized protein C19orf43 OS=Homo sapiens GN=C19orf43 PE=1 SV=1 - [CS043_HUMAN]	39.20	1	6	6	18	1.156	1.118	0.913	1.288	43.27	39.20	7	18	176	18.4	9.44
Q96LM1	Putative uncharacterized protein encoded by LINC00615 OS=Homo sapiens GN=LINC00615 PE=5 SV=1 - [CL037_HUMAN	10.61	1	1	1	1	2.148	2.586	2.427	1.289	0.00	10.61	1	1	132	14.5	9.91

Q8IZX4	Transcription initiation factor TFIID subunit 1-like OS=Homo sapiens GN=TAF1L PE=1 SV=1 - [TAF1L_HUMAN]	0.38	1	1	1	1	1.030	1.284	1.172	1.289	1.98	0.38	1	1	1826	207.2	5.40
O95602	DNA-directed RNA polymerase I subunit RPA1 OS=Homo sapiens GN=POLR1A PE=1 SV=2 - [RPA1_HUMAN]	3.55	1	6	6	16	0.949	1.123	1.219	1.289	32.67	3.55	8	16	1720	194.7	7.03
Q7Z3E2	Coiled-coil domain-containing protein 186 OS=Homo sapiens GN=CCDC186 PE=1 SV=2 - [CC186_HUMAN]	4.57	1	2	3	6	1.192	0.872	1.895	1.289	15.27	4.57	5	6	898	103.6	6.27
P43243	Matrin-3 OS=Homo sapiens GN=MATR3 PE=1 SV=2 - [MATR3_HUMAN]	34.12	1	28	28	169	1.125	1.151	1.143	1.289	432.23	34.12	43	169	847	94.6	6.25
Q9UER7	Death domain-associated protein 6 OS=Homo sapiens GN=DAXX PE=1 SV=2 - [DAXX_HUMAN]	6.62	1	4	4	5	1.030	1.049	0.940	1.289	15.47	6.62	5	5	740	81.3	4.87
Q9BVJ7	Dual specificity protein phosphatase 23 OS=Homo sapiens GN=DUSP23 PE=1 SV=1 - [DUS23_HUMAN]	22.00	1	3	3	6	1.035	1.149	1.115	1.290	17.57	22.00	5	6	150	16.6	8.21
P49792	E3 SUMO-protein ligase RanBP2 OS=Homo sapiens GN=RANBP2 PE=1 SV=2 - [RBP2_HUMAN]	21.12	5	33	63	164	1.163	1.280	0.948	1.290	383.59	21.12	88	164	3224	358.0	6.20
Q7L2J0	7SK snRNA methylphosphate capping enzyme OS=Homo sapiens GN=MEPCE PE=1 SV=1 - [MEPCE_HUMAN]	8.27	1	5	5	9	0.834	1.298	1.201	1.290	20.17	8.27	7	9	689	74.3	9.57

Q14498	RNA-binding protein 39 OS=Homo sapiens GN=RBM39 PE=1 SV=2 - [RBM39_HUMAN]	25.28	1	12	12	36	1.109	1.326	0.958	1.290	89.15	25.28	17	36	530	59.3	10.10
O00541	Pescadillo homolog OS=Homo sapiens GN=PES1 PE=1 SV=1 - [PESC_HUMAN]	21.09	1	12	13	30	0.712	0.820	0.694	1.290	71.97	21.09	19	30	588	68.0	7.33
P23786	Carnitine O-palmitoyltransferase 2, mitochondrial OS=Homo sapiens GN=CPT2 PE=1 SV=2 - [CPT2_HUMAN]	34.35	1	23	23	50	1.275	1.043	0.763	1.291	123.69	34.35	32	50	658	73.7	8.18
Q8NEB9	Phosphatidylinositol 3-kinase catalytic subunit type 3 OS=Homo sapiens GN=PIK3C3 PE=1 SV=1 - [PK3C3_HUMAN]	8.68	1	7	7	20	0.965	0.857	0.931	1.291	49.41	8.68	13	20	887	101.5	6.81
A6NGB9	WAS/WASL-interacting protein family member 3 OS=Homo sapiens GN=WIPF3 PE=2 SV=4 - [WIPF3_HUMAN]	1.45	1	1	1	4	1.513	1.245	1.948	1.291	9.10	1.45	1	4	483	49.4	10.15
Q9UPT6	C-Jun-amino-terminal kinase-interacting protein 3 OS=Homo sapiens GN=MAPK8IP3 PE=1 SV=3 - [JIP3_HUMAN]	6.36	1	4	5	7	0.955	1.135	1.062	1.292	15.83	6.36	6	7	1336	147.4	5.40
P16219	Short-chain specific acyl-CoA dehydrogenase, mitochondrial OS=Homo sapiens GN=ACADS PE=1 SV=1 - [ACADS_HUMAN]	24.27	1	9	9	17	1.050	1.373	0.875	1.292	35.75	24.27	13	17	412	44.3	7.99
P24723	Protein kinase C eta type OS=Homo sapiens GN=PRKCH PE=1 SV=4 - [KPCL_HUMAN]	3.81	1	1	2	5	1.601	2.007	1.297	1.293	8.16	3.81	2	5	683	77.8	7.77

Q7Z2W4	Zinc finger CCCH-type antiviral protein 1 OS=Homo sapiens GN=ZC3HAV1 PE=1 SV=3 - [ZCCHV_HUMAN	19.51	1	13	13	47	1.320	1.045	1.185	1.293	137.89	19.51	22	47	902	101.4	8.40
Q9BVJ6	U3 small nucleolar RNA-associated protein 14 homolog A OS=Homo sapiens GN=UTP14A PE=1 SV=1 - [UT14A_HUMAN]	3.11	1	2	2	7	0.779	0.994	1.005	1.293	21.11	3.11	3	7	771	87.9	7.87
Q15233	Non-POU domain-containing octamer-binding protein OS=Homo sapiens GN=NONO PE=1 SV=4 - [NONO_HUMAN]	55.41	1	21	23	176	1.163	1.243	1.056	1.293	487.91	55.41	41	176	471	54.2	8.95
P55081	Microfibrillar-associated protein 1 OS=Homo sapiens GN=MFAP1 PE=1 SV=2 - [MFAP1_HUMA N]	23.23	1	6	8	15	1.376	1.429	1.124	1.293	50.62	23.23	11	15	439	51.9	4.98
Q8IYT3	Coiled-coil domain-containing protein 170 OS=Homo sapiens GN=CCDC170 PE=2 SV=3 - [CC170_HUMAN]	2.10	1	1	2	4	0.992	1.490	1.283	1.293	8.38	2.10	2	4	715	82.2	6.64
Q08211	ATP-dependent RNA helicase A OS=Homo sapiens GN=DHX9 PE=1 SV=4 - [DHX9_HUMAN]	34.96	1	39	42	197	1.149	1.148	0.960	1.293	553.04	34.96	61	197	1270	140.9	6.84
O75167	Phosphatase and actin regulator 2 OS=Homo sapiens GN=PHACTR2 PE=1 SV=2 - [PHAR2_HUMAN]	7.10	1	3	4	5	2.347	1.605	1.262	1.293	10.28	7.10	5	5	634	69.7	8.16
O14832	Phytanoyl-CoA dioxygenase, peroxisomal OS=Homo sapiens GN=PHYH PE=1 SV=1 - [PAHX_HUMAN]	5.92	1	3	3	6	0.996	1.163	1.141	1.293	12.30	5.92	4	6	338	38.5	8.47

Q15061	WD repeat-containing protein 43 OS=Homo sapiens GN=WDR43 PE=1 SV=3 - [WDR43_HUMAN]	13.59	1	5	6	16	0.846	1.039	0.949	1.294	35.50	13.59	7	16	677	74.8	5.57
Q5VYS8	Terminal uridylyltransferase 7 OS=Homo sapiens GN=ZCCHC6 PE=1 SV=1 - [TUT7_HUMAN]	2.94	2	4	5	10	1.095	0.776	0.955	1.294	18.53	2.94	8	10	1495	171.1	6.83
Q9HCK8	Chromodomain-helicase-DNA-binding protein 8 OS=Homo sapiens GN=CHD8 PE=1 SV=5 - [CHD8_HUMAN]	1.63	1	3	3	4	1.215	1.286	1.110	1.294	4.76	1.63	3	4	2581	290.3	6.47
P60880	Synaptosomal-associated protein 25 OS=Homo sapiens GN=SNAP25 PE=1 SV=1 - [SNP25_HUMAN]	6.80	1	1	1	2	1.867	1.963	0.988	1.294	5.49	6.80	2	2	206	23.3	4.77
B7Z6K7	Putative uncharacterized zinc finger protein 814 OS=Homo sapiens GN=ZNF814 PE=2 SV=2 - [ZN814_HUMAN]	2.57	11	1	2	3	1.373	1.301	1.147	1.294	5.25	2.57	3	3	855	97.3	8.85
P20700	Lamin-B1 OS=Homo sapiens GN=LMNB1 PE=1 SV=2 - [LMNB1_HUMAN]	72.70	1	36	43	342	1.128	1.538	0.793	1.294	796.87	72.70	72	342	586	66.4	5.16
P09455	Retinol-binding protein 1 OS=Homo sapiens GN=RBP1 PE=1 SV=2 - [RET1_HUMAN]	53.33	1	6	6	14	0.869	3.984	1.146	1.294	37.28	53.33	9	14	135	15.8	5.11
Q86UE8	Serine/threonine-protein kinase tousled-like 2 OS=Homo sapiens GN=TLK2 PE=1 SV=2 - [TLK2_HUMAN]	6.99	1	4	5	6	0.880	0.821	1.024	1.294	9.28	6.99	5	6	772	87.6	8.41
P61604	10 kDa heat shock protein, mitochondrial OS=Homo sapiens GN=HSPE1 PE=1 SV=2 - [CH10_HUMAN]	63.73	1	9	9	122	1.012	1.676	1.054	1.295	198.06	63.73	16	122	102	10.9	8.92

Q14241	Transcription elongation factor B polypeptide 3 OS=Homo sapiens GN=TCEB3 PE=1 SV=2 - [ELOA1_HUMAN]	8.40	1	7	7	23	0.998	1.063	0.862	1.295	38.74	8.40	12	23	798	89.9	9.57
O43741	5'-AMP-activated protein kinase subunit beta-2 OS=Homo sapiens GN=PRKAB2 PE=1 SV=1 - [AAKB2_HUMAN]	9.19	1	2	2	7	0.727	0.709	1.140	1.295	14.60	9.19	3	7	272	30.3	6.46
Q05209	Tyrosine-protein phosphatase non-receptor type 12 OS=Homo sapiens GN=PTPN12 PE=1 SV=3 - [PTN12_HUMAN]	20.26	1	13	13	46	1.119	0.957	1.138	1.295	93.33	20.26	22	46	780	88.1	5.62
Q92522	Histone H1x OS=Homo sapiens GN=H1FX PE=1 SV=1 - [H1X_HUMAN]	40.85	1	11	11	55	1.841	2.369	0.971	1.295	134.55	40.85	20	55	213	22.5	10.76
Q8TA86	Retinitis pigmentosa 9 protein OS=Homo sapiens GN=RP9 PE=1 SV=2 - [RP9_HUMAN]	4.98	1	1	1	2	1.012	1.487	0.954	1.295	2.77	4.98	1	2	221	26.1	9.79
Q9C0E4	Glutamate receptor-interacting protein 2 OS=Homo sapiens GN=GRIP2 PE=1 SV=3 - [GRIP2_HUMAN]	1.44	1	1	1	1	0.537	0.606	1.090	1.295	0.00	1.44	1	1	1043	112.4	6.49
P19404	NADH dehydrogenase [ubiquinone] flavoprotein 2, mitochondrial OS=Homo sapiens GN=NDUFV2 PE=1 SV=2 - [NDUV2_HUMAN]	22.49	2	5	5	36	0.753	1.212	0.840	1.296	78.37	22.49	8	36	249	27.4	8.06
Q2M218	Zinc finger protein 630 OS=Homo sapiens GN=ZNF630 PE=2 SV=1 - [ZN630_HUMAN]	1.22	1	1	1	1	1.121	1.861	1.468	1.296	0.00	1.22	1	1	657	76.0	8.78

Q9BYG3	MKI67 FHA domain-interacting nucleolar phosphoprotein OS=Homo sapiens GN=NIFK PE=1 SV=1 - [MK67I_HUMAN]	23.21	1	6	6	13	0.831	0.841	0.764	1.296	21.76	23.21	9	13	293	34.2	9.88
Q9H8H0	Nucleolar protein 11 OS=Homo sapiens GN=NOL11 PE=1 SV=1 - [NOL11_HUMAN]	5.01	1	4	4	5	0.852	1.099	0.911	1.297	6.71	5.01	4	5	719	81.1	6.07
Q9BYD2	39S ribosomal protein L9, mitochondrial OS=Homo sapiens GN=MRPL9 PE=1 SV=2 - [RM09_HUMAN]	16.48	1	4	4	9	0.949	0.974	1.032	1.298	25.90	16.48	6	9	267	30.2	10.08
Q3KRA6	UPF0538 protein C2orf76 OS=Homo sapiens GN=C2orf76 PE=1 SV=3 - [CB076_HUMAN]	10.32	1	1	1	2	0.873	0.789	0.658	1.298	8.08	10.32	2	2	126	14.6	6.93
O75385	Serine/threonine-protein kinase ULK1 OS=Homo sapiens GN=ULK1 PE=1 SV=2 - [ULK1_HUMAN]	0.86	1	1	1	1	0.593	0.935	0.932	1.299	0.00	0.86	1	1	1050	112.6	8.79
Q8TB72	Pumilio homolog 2 OS=Homo sapiens GN=PUM2 PE=1 SV=2 - [PUM2_HUMAN]	6.00	2	3	7	17	0.987	1.497	0.708	1.299	36.15	6.00	9	17	1066	114.1	7.08
P62263	40S ribosomal protein S14 OS=Homo sapiens GN=RPS14 PE=1 SV=3 - [RS14_HUMAN]	47.02	1	11	11	71	1.000	0.992	1.125	1.299	171.90	47.02	21	71	151	16.3	10.05
O95299	NADH dehydrogenase [ubiquinone] 1 alpha subcomplex subunit 10, mitochondrial OS=Homo sapiens GN=NDUFA10 PE=1 SV=1 - [NDUAA_HUMAN]	30.70	2	9	9	17	1.018	0.897	1.104	1.300	44.42	30.70	13	17	355	40.7	8.48

Q9P2D1	Chromodomain-helicase-DNA-binding protein 7 OS=Homo sapiens GN=CHD7 PE=1 SV=3 - [CHD7_HUMAN]	1.33	1	3	4	6	0.661	0.554	0.728	1.300	6.47	1.33	4	6	2997	335.7	6.34
Q9BY77	Polymerase delta-interacting protein 3 OS=Homo sapiens GN=POLDIP3 PE=1 SV=2 - [PDIP3_HUMAN	18.29	1	7	7	17	1.063	1.051	0.985	1.300	41.18	18.29	11	17	421	46.1	9.99
Q7Z3D4	LysM and putative peptidoglycan-binding domain-containing protein 3 OS=Homo sapiens GN=LYSMD3 PE=1 SV=2 - [LYSM3_HUMAN]	3.59	1	1	1	1	0.612	1.015	1.501	1.300	2.99	3.59	1	1	306	34.5	5.97
P19823	Inter-alpha-trypsin inhibitor heavy chain H2 OS=Homo sapiens GN=ITIH2 PE=1 SV=2 - [ITIH2_HUMAN]	3.07	1	2	3	10	0.853	0.599	0.992	1.301	20.56	3.07	4	10	946	106.4	6.86
Q9Y512	Sorting and assembly machinery component 50 homolog OS=Homo sapiens GN=SAMM50 PE=1 SV=3 - [SAM50_HUMA N]	21.32	1	10	10	19	1.026	0.900	1.012	1.301	42.92	21.32	13	19	469	51.9	6.90
Q9UL03	Integrator complex subunit 6 OS=Homo sapiens GN=INTS6 PE=1 SV=1 - [INT6_HUMAN]	2.14	1	2	2	4	1.040	0.984	1.002	1.301	6.87	2.14	3	4	887	100.3	8.62
Q5W041	Armadillo repeat-containing protein 3 OS=Homo sapiens GN=ARMC3 PE=2 SV=2 - [ARMC3_HUMA	0.69	1	1	1	1	1.291	0.787	1.289	1.301	1.89	0.69	1	1	872	96.3	6.21
O95758	Polypyrimidine tract-binding protein 3 OS=Homo sapiens GN=PTBP3 PE=1 SV=2 - [PTBP3_HUMAN]	7.97	1	2	5	33	1.092	1.057	1.110	1.302	92.68	7.97	7	33	552	59.7	9.04

O00478	Butyrophilin subfamily 3 member A3 OS=Homo sapiens GN=BTN3A3 PE=1 SV=1 - [BT3A3_HUMAN]	8.05	3	4	5	13	3.191	0.997	0.722	1.302	29.59	8.05	6	13	584	65.0	5.53
Q8NEJ9	Neuroguidin OS=Homo sapiens GN=NGDN PE=1 SV=1 - [NGDN_HUMAN]	13.97	1	4	4	15	0.771	0.655	0.789	1.302	35.30	13.97	7	15	315	35.9	9.57
Q9H0A0	N-acetyltransferase 10 OS=Homo sapiens GN=NAT10 PE=1 SV=2 - [NAT10_HUMAN	16.20	2	14	15	38	0.849	0.827	0.722	1.302	81.85	16.20	24	38	1025	115.7	8.27
Q8WUZ0	B-cell CLL/lymphoma 7 protein family member C OS=Homo sapiens GN=BCL7C PE=1 SV=3 - [BCL7C_HUMAN]	10.14	1	1	2	9	1.157	2.464	1.546	1.302	18.21	10.14	4	9	217	23.5	5.20
Q96LD8	Sentrin-specific protease 8 OS=Homo sapiens GN=SENP8 PE=1 SV=1 - [SENP8_HUMAN]	3.30	1	1	1	4	0.822	0.981	0.902	1.302	9.26	3.30	2	4	212	24.1	5.97
P51808	Dynein light chain Tctex-type 3 OS=Homo sapiens GN=DYNLT3 PE=1 SV=1 - [DYLT3_HUMAN	14.66	1	1	1	7	1.734	0.886	1.057	1.303	22.00	14.66	2	7	116	13.1	5.66
P51003	Poly(A) polymerase alpha OS=Homo sapiens GN=PAPOLA PE=1 SV=4 - [PAPOA_HUMA N]	3.89	2	3	4	7	1.046	0.882	0.850	1.303	16.08	3.89	4	7	745	82.8	7.37
Q15345	Leucine-rich repeat-containing protein 41 OS=Homo sapiens GN=LRRC41 PE=1 SV=3 - [LRC41_HUMAN]	4.43	1	2	2	2	1.214	1.767	1.237	1.303	9.55	4.43	2	2	812	88.6	8.38
Q14151	Scaffold attachment factor B2 OS=Homo sapiens GN=SAFB2 PE=1 SV=1 - [SAFB2_HUMAN	16.79	1	10	17	47	1.137	1.503	1.019	1.303	100.52	16.79	27	47	953	107.4	6.16

Q9BWS9	Chitinase domain-containing protein 1 OS=Homo sapiens GN=CHID1 PE=1 SV=1 - [CHID1_HUMAN]	22.14	1	9	9	27	0.953	0.960	0.832	1.303	57.34	22.14	14	27	393	44.9	8.63
Q96QD9	UAP56-interacting factor OS=Homo sapiens GN=FYTTD1 PE=1 SV=3 - [UIF_HUMAN]	8.49	1	3	3	6	1.315	1.133	1.271	1.303	9.76	8.49	4	6	318	35.8	11.78
Q14103	Heterogeneous nuclear ribonucleoprotein D0 OS=Homo sapiens GN=HNRNPD PE=1 SV=1 - [HNRPD_HUMAN]	39.72	1	16	17	114	1.111	1.399	1.085	1.304	311.39	39.72	27	114	355	38.4	7.81
Q9NRY2	SOSS complex subunit C OS=Homo sapiens GN=INIP PE=1 SV=1 - [SOSSC_HUMAN]	10.58	1	1	1	3	1.476	1.416	0.667	1.305	2.20	10.58	2	3	104	11.4	9.25
Q03252	Lamin-B2 OS=Homo sapiens GN=LMNB2 PE=1 SV=3 - [LMNB2_HUMAN]	53.50	1	36	44	285	1.144	1.366	1.019	1.305	638.21	53.50	72	285	600	67.6	5.35
P56277	Cx9C motif-containing protein 4 OS=Homo sapiens GN=CMC4 PE=1 SV=1 - [CMC4_HUMAN]	10.29	1	1	1	2	1.271	1.322	1.263	1.305	4.16	10.29	1	2	68	7.7	8.18
Q6P161	39S ribosomal protein L54, mitochondrial OS=Homo sapiens GN=MRPL54 PE=1 SV=1 - [RM54_HUMAN]	7.25	1	1	1	2	0.923	1.661	1.030	1.306	2.85	7.25	2	2	138	15.8	9.60
P52655	Transcription initiation factor IIA subunit 1 OS=Homo sapiens GN=GTF2A1 PE=1 SV=1 - [TF2AA_HUMAN]	7.45	1	2	2	4	0.830	1.289	0.548	1.306	10.97	7.45	4	4	376	41.5	4.55
O75376	Nuclear receptor corepressor 1 OS=Homo sapiens GN=NCOR1 PE=1 SV=2 - [NCOR1_HUMA	5.33	2	11	12	21	0.985	1.134	1.009	1.306	53.73	5.33	14	21	2440	270.0	7.11

P00491	Purine nudeoside phosphorylase OS=Homo sapiens GN=PNP PE=1 SV=2 - [PNPH_HUMAN]	35.29	1	7	8	25	0.921	0.989	1.061	1.306	57.73	35.29	12	25	289	32.1	6.95
Q6ZWT7	Lysophospholipid acyltransferase 2 OS=Homo sapiens GN=MBOAT2 PE=2 SV=2 - [MBOA2_HUMAN]	5.19	1	3	3	5	1.065	1.900	1.522	1.306	10.30	5.19	4	5	520	59.5	8.03
A1XBS5	Protein FAM92A1 OS=Homo sapiens GN=FAM92A1 PE=2 SV=2 - [F92A1_HUMAN]	4.84	1	2	2	2	1.333	0.860	0.510	1.307	3.36	4.84	2	2	289	33.4	6.21
Q9UFW8	CGG triplet repeat-binding protein 1 OS=Homo sapiens GN=CGGBP1 PE=1 SV=2 - [CGBP1_HUMAN]	5.39	1	1	1	3	1.006	1.219	1.170	1.307	0.00	5.39	1	3	167	18.8	8.95
P78524	Suppression of tumorigenicity 5 protein OS=Homo sapiens GN=ST5 PE=1 SV=3 - [ST5_HUMAN]	6.24	1	6	6	21	1.154	0.935	1.040	1.307	44.95	6.24	9	21	1137	126.4	9.25
P17010	Zinc finger X-chromosomal protein OS=Homo sapiens GN=ZFX PE=2 SV=2 - [ZFX_HUMAN]	4.22	3	3	3	9	1.078	1.612	0.964	1.307	4.67	4.22	4	9	805	90.5	6.14
Q9BYN0	Sulfiredoxin-1 OS=Homo sapiens GN=SRXN1 PE=1 SV=2 - [SRXN1_HUMAN]	58.39	1	6	6	12	1.007	0.471	1.191	1.307	41.34	58.39	10	12	137	14.3	8.19
P49411	Elongation factor Tu, mitochondrial OS=Homo sapiens GN=TUFM PE=1 SV=2 - [EFTU_HUMAN]	63.05	1	26	26	207	1.185	1.228	1.007	1.307	634.20	63.05	42	207	452	49.5	7.61
Q9BZI7	Regulator of nonsense transcripts 3B OS=Homo sapiens GN=UPF3B PE=1 SV=1 - [REN3B_HUMAN]	14.91	1	7	7	15	1.107	1.340	1.093	1.308	35.81	14.91	8	15	483	57.7	9.48

Q9NVH2	Integrator complex subunit 7 OS=Homo sapiens GN=INTS7 PE=1 SV=1 - [INT7_HUMAN]	3.85	1	3	3	17	1.283	1.084	0.936	1.308	17.21	3.85	5	17	962	106.8	8.02
P06746	DNA polymerase beta OS=Homo sapiens GN=POLB PE=1 SV=3 - [DPOLB_HUMAN	11.34	1	4	4	18	0.984	1.554	1.497	1.308	34.90	11.34	6	18	335	38.2	8.95
Q8TDR0	TRAF3-interacting protein 1 OS=Homo sapiens GN=TRAF3IP1 PE=1 SV=1 - [MIPT3_HUMAN]	4.49	1	3	4	5	1.403	1.731	1.216	1.308	5.16	4.49	4	5	691	78.6	7.93
O75674	TOM1-like protein 1 OS=Homo sapiens GN=TOM1L1 PE=1 SV=2 - [TM1L1_HUMAN]	3.36	1	1	2	3	1.366	1.006	1.314	1.309	6.38	3.36	3	3	476	53.0	5.24
P98198	Phospholipid-transporting ATPase ID OS=Homo sapiens GN=ATP8B2 PE=1 SV=2 - [AT8B2_HUMAN]	3.47	1	3	3	4	0.808	0.628	1.061	1.309	7.67	3.47	4	4	1209	137.4	7.01
P24539	ATP synthase F(0) complex subunit B1, mitochondrial OS=Homo sapiens GN=ATP5F1 PE=1 SV=2 - [AT5F1_HUMAN]	39.06	1	14	14	86	1.222	1.060	0.942	1.309	201.50	39.06	23	86	256	28.9	9.36
Q9UBW7	Zinc finger MYM-type protein 2 OS=Homo sapiens GN=ZMYM2 PE=1 SV=1 - [ZMYM2_HUMA N]	1.16	1	1	1	2	1.514	1.808	1.425	1.309	4.49	1.16	2	2	1377	154.8	6.34
P39210	Protein Mpv17 OS=Homo sapiens GN=MPV17 PE=1 SV=1 - [MPV17_HUMA N]	4.55	1	1	1	3	1.192	1.210	0.754	1.309	2.13	4.55	2	3	176	19.7	9.47
Q9Y314	Nitric oxide synthase-interacting protein OS=Homo sapiens GN=NOSIP PE=1 SV=1 - [NOSIP_HUMAN]	24.25	1	7	7	15	1.016	1.235	1.124	1.309	36.92	24.25	10	15	301	33.2	8.82

Q92841	Probable ATP-dependent RNA helicase DDX17 OS=Homo sapiens GN=DDX17 PE=1 SV=2 - [DDX17_HUMAN	45.54	1	28	35	239	1.156	1.394	1.102	1.309	540.15	45.54	58	239	729	80.2	8.27
Q8NHQ8	Ras association domain-containing protein 8 OS=Homo sapiens GN=RASSF8 PE=1 SV=2 - [RASF8_HUMAN]	15.51	1	6	7	11	1.237	1.097	1.136	1.309	21.95	15.51	9	11	419	48.3	6.49
Q9BRD0	BUD13 homolog OS=Homo sapiens GN=BUD13 PE=1 SV=1 - [BUD13_HUMAN]	1.62	1	1	1	1	0.888	1.299	1.132	1.310	0.00	1.62	1	1	619	70.5	9.86
P25440	Bromodomain-containing protein 2 OS=Homo sapiens GN=BRD2 PE=1 SV=2 - [BRD2_HUMAN]	5.12	1	4	4	10	0.992	1.168	0.831	1.310	19.55	5.12	7	10	801	88.0	9.09
Q12789	General transcription factor 3C polypeptide 1 OS=Homo sapiens GN=GTF3C1 PE=1 SV=4 - [TF3C1_HUMAN]	2.47	1	5	5	8	0.732	1.082	0.994	1.310	15.22	2.47	6	8	2109	238.7	7.30
Q99704	Docking protein 1 OS=Homo sapiens GN=DOK1 PE=1 SV=1 - [DOK1_HUMAN]	6.24	1	2	2	6	0.572	1.165	0.711	1.310	22.06	6.24	3	6	481	52.4	6.47
Q7L775	EPM2A-interacting protein 1 OS=Homo sapiens GN=EPM2AIP1 PE=1 SV=1 - [EPMIP_HUMAN]	15.49	1	6	8	22	1.145	1.093	1.111	1.310	58.43	15.49	12	22	607	70.3	6.11
Q01804	OTU domain-containing protein 4 OS=Homo sapiens GN=OTUD4 PE=1 SV=4 - [OTUD4_HUMA N]	6.91	1	5	5	12	1.009	1.227	1.369	1.310	25.54	6.91	8	12	1114	124.0	6.71

Q96HR9	Receptor expression-enhancing protein 6 OS=Homo sapiens GN=REEP6 PE=1 SV=1 - [REEP6_HUMAN]	5.98	1	1	1	2	0.787	2.331	0.546	1.310	2.35	5.98	2	2	184	20.7	8.56
Q92539	Phosphatidate phosphatase LPIN2 OS=Homo sapiens GN=LPIN2 PE=1 SV=1 - [LPIN2_HUMAN]	1.56	1	1	2	3	0.862	0.355	0.824	1.310	6.46	1.56	2	3	896	99.3	5.33
P57737	Coronin-7 OS=Homo sapiens GN=CORO7 PE=1 SV=2 - [CORO7_HUMAN]	8.86	1	7	7	9	1.542	0.866	0.944	1.311	24.60	8.86	9	9	925	100.5	5.80
Q5RKV6	Exosome complex component MTR3 OS=Homo sapiens GN=EXOSC6 PE=1 SV=1 - [EXOS6_HUMAN]	30.15	1	6	6	16	1.383	1.332	0.954	1.311	39.97	30.15	10	16	272	28.2	6.28
Q86WA6	Valacyclovir hydrolase OS=Homo sapiens GN=BPHL PE=1 SV=1 - [BPHL_HUMAN]	20.27	1	7	7	11	1.587	1.544	1.303	1.311	27.70	20.27	8	11	291	32.5	9.14
Q9P2N5	RNA-binding protein 27 OS=Homo sapiens GN=RBM27 PE=1 SV=2 - [RBM27_HUMAN]	5.57	1	5	5	11	1.167	1.543	1.043	1.311	23.04	5.57	6	11	1060	118.6	9.19
Q15031	Probable leucine--tRNA ligase, mitochondrial OS=Homo sapiens GN=LARS2 PE=1 SV=2 - [SYLM_HUMAN]	6.98	1	6	6	14	1.106	1.061	0.996	1.311	11.40	6.98	11	14	903	101.9	8.22
P37198	Nuclear pore glycoprotein p62 OS=Homo sapiens GN=NUP62 PE=1 SV=3 - [NUP62_HUMAN]	16.86	1	8	8	78	1.349	1.460	0.939	1.311	258.44	16.86	15	78	522	53.2	5.31
O00534	von Willebrand factor A domain-containing protein 5A OS=Homo sapiens GN=VWA5A PE=2 SV=2 - [VMA5A_HUMA	22.39	1	16	16	38	1.915	0.684	0.572	1.311	93.08	22.39	27	38	786	86.4	6.58

Q6ZMC9	Sialic acid-binding Ig-like lectin 15 OS=Homo sapiens GN=SIGLEC15 PE=1 SV=1 - [SIG15_HUMAN]	1.83	1	1	1	1	0.967	1.472	1.006	1.312	2.28	1.83	1	1	328	35.6	8.51
Q16594	Transcription initiation factor TFIID subunit 9 OS=Homo sapiens GN=TAF9 PE=1 SV=1 - [TAF9_HUMAN]	10.61	2	3	3	5	1.073	0.793	1.129	1.312	6.58	10.61	4	5	264	29.0	8.66
Q9BPZ3	Polyadenylate-binding protein-interacting protein 2 OS=Homo sapiens GN=PAIP2 PE=1 SV=1 - [PAIP2_HUMAN]	11.02	1	1	1	2	1.249	0.708	0.691	1.312	8.20	11.02	2	2	127	15.0	4.12
Q14669	E3 ubiquitin-protein ligase TRIP12 OS=Homo sapiens GN=TRIP12 PE=1 SV=1 - [TRIPC_HUMAN]	7.13	1	12	12	33	0.830	0.994	0.952	1.313	82.76	7.13	20	33	1992	220.3	8.48
Q12840	Kinesin heavy chain isoform 5A OS=Homo sapiens GN=KIF5A PE=1 SV=2 - [KIF5A_HUMAN]	10.08	1	2	9	39	1.230	1.016	0.972	1.314	108.81	10.08	14	39	1032	117.3	5.90
P98164	Low-density lipoprotein receptor-related protein 2 OS=Homo sapiens GN=LRP2 PE=1 SV=3 - [LRP2_HUMAN]	0.39	1	1	2	2	0.834	0.757	1.152	1.314	5.58	0.39	2	2	4655	521.6	5.08
Q9BVC6	Transmembrane protein 109 OS=Homo sapiens GN=TMEM109 PE=1 SV=1 - [TM109_HUMAN]	9.47	1	5	5	23	1.374	1.431	1.702	1.314	60.09	9.47	9	23	243	26.2	10.48
P55209	Nucleosome assembly protein 1-like 1 OS=Homo sapiens GN=NAP1L1 PE=1 SV=1 - [NP1L1_HUMAN]	39.90	1	9	11	198	0.876	1.004	1.033	1.314	577.01	39.90	16	198	391	45.3	4.46
Q15773	Myeloid leukemia factor 2 OS=Homo sapiens GN=MLF2 PE=1 SV=1 - [MLF2_HUMAN]	4.84	1	1	1	2	0.825	1.055	1.313	1.315	3.57	4.84	2	2	248	28.1	6.90

Q96AP7	Endothelial cell-selective adhesion molecule OS=Homo sapiens GN=ESAM PE=1 SV=1 - [ESAM_HUMAN]	2.82	1	1	1	1	0.960	1.541	1.851	1.315	0.00	2.82	1	1	390	41.2	9.32
Q5VSP4	Putative lipocalin 1-like protein 1 OS=Homo sapiens GN=LCN1P1 PE=5 SV=1 - [LC1L1_HUMAN]	6.79	2	1	1	2	0.148	1.242	1.052	1.315	0.00	6.79	2	2	162	17.9	5.00
Q6ZR62	Zinc finger CCHC domain-containing protein 16 OS=Homo sapiens GN=ZCCHC16 PE=2 SV=2 - [ZCH16_HUMAN]	3.23	1	1	1	1	0.932	1.007	0.898	1.315	3.03	3.23	1	1	310	34.7	5.80
Q8WUY8	N-acetyltransferase 14 OS=Homo sapiens GN=NAT14 PE=1 SV=1 - [NAT14_HUMAN	16.02	1	3	3	4	1.395	0.958	0.803	1.315	4.58	16.02	4	4	206	21.6	10.74
Q14957	Glutamate receptor ionotropic, NMDA 2C OS=Homo sapiens GN=GRIN2C PE=1 SV=3 - [NMDE3_HUMA N]	0.89	1	1	1	1	0.746	1.334	0.891	1.315	0.00	0.89	1	1	1233	134.1	8.48
O43464	Serine protease HTRA2, mitochondrial OS=Homo sapiens GN=HTRA2 PE=1 SV=2 - [HTRA2_HUMAN	27.29	2	8	8	22	1.050	1.353	1.157	1.316	61.68	27.29	13	22	458	48.8	10.07
P52597	Heterogeneous nuclear ribonucleoprotein F OS=Homo sapiens GN=HNRNPF PE=1 SV=3 - [HNRPF_HUMAN	44.34	1	11	13	116	1.107	1.520	1.034	1.316	334.62	44.34	21	116	415	45.6	5.58
Q92504	Zinc transporter SLC39A7 OS=Homo sapiens GN=SLC39A7 PE=1 SV=2 - [S39A7_HUMAN	2.99	1	2	2	18	1.235	1.109	0.861	1.316	32.78	2.99	3	18	469	50.1	6.87

O75306	NADH dehydrogenase [ubiquinone] iron-sulfur protein 2, mitochondrial OS=Homo sapiens GN=NDUFS2 PE=1 SV=2 - [NDUS2_HUMAN]	28.29	1	10	10	38	0.820	1.299	0.965	1.316	113.14	28.29	14	38	463	52.5	7.55
Q9Y6N7	Roundabout homolog 1 OS=Homo sapiens GN=ROBO1 PE=1 SV=1 - [ROBO1_HUMAN]	8.84	1	10	10	23	1.553	0.904	1.005	1.316	26.42	8.84	14	23	1651	180.8	6.04
Q8N6R0	Methyltransferase-like protein 13 OS=Homo sapiens GN=METTL13 PE=1 SV=1 - [MET13_HUMAN]	3.86	1	3	3	7	0.801	0.802	1.022	1.316	16.02	3.86	4	7	699	78.7	6.73
B7ZAP0	Rab GTPase-activating protein 1-like, isoform 10 OS=Homo sapiens GN=RABGAP1L PE=1 SV=1 - [RBG10_HUMAN]	14.62	1	1	3	4	2.224	5.234	6.990	1.317	6.88	14.62	4	4	253	29.0	5.33
Q9BXL7	Caspase recruitment domain-containing protein 11 OS=Homo sapiens GN=CARD11 PE=1 SV=3 - [CAR11_HUMAN]	0.69	1	1	1	1	1.838	2.766	1.965	1.317	2.49	0.69	1	1	1154	133.2	6.09
O94760	N(G),N(G)-dimethylarginine dimethylaminohydrolase 1 OS=Homo sapiens GN=DDAH1 PE=1 SV=3 - [DDAH1_HUMAN]	56.14	1	10	11	52	1.185	1.109	0.755	1.317	141.46	56.14	19	52	285	31.1	5.81
Q8TAQ2	SWI/SNF complex subunit SMARCC2 OS=Homo sapiens GN=SMARCC2 PE=1 SV=1 - [SMRC2_HUMAN]	17.05	1	12	18	62	1.268	1.612	1.322	1.317	157.87	17.05	26	62	1214	132.8	5.69
P19419	ETS domain-containing protein Elk-1 OS=Homo sapiens GN=ELK1 PE=1 SV=2 - [ELK1_HUMAN]	5.61	1	2	2	6	0.578	0.967	1.020	1.318	11.90	5.61	3	6	428	44.9	6.89

Q9Y3T6	R3H and coiled-coil domain-containing protein 1 OS=Homo sapiens GN=R3HCC1 PE=1 SV=2 - [R3HC1_HUMAN]	11.46	1	3	3	6	1.373	1.428	1.353	1.318	5.43	11.46	4	6	480	53.2	6.27
Q9BVL2	Nucleoporin p58/p45 OS=Homo sapiens GN=NUPL1 PE=1 SV=1 - [NUPL1_HUMAN]	6.34	1	5	5	14	1.141	1.064	0.921	1.318	18.45	6.34	7	14	599	60.9	9.33
A8MW92	PHD finger protein 20-like protein 1 OS=Homo sapiens GN=PHF20L1 PE=1 SV=2 - [P20L1_HUMAN]	1.97	1	2	2	2	1.988	1.838	0.619	1.318	4.89	1.97	2	2	1017	114.9	6.83
Q14393	Growth arrest-specific protein 6 OS=Homo sapiens GN=GAS6 PE=1 SV=2 - [GAS6_HUMAN]	6.10	1	4	4	10	0.681	0.931	0.669	1.318	27.53	6.10	6	10	721	79.6	6.21
P10109	Adrenodoxin, mitochondrial OS=Homo sapiens GN=FDX1 PE=1 SV=1 - [ADX_HUMAN]	27.72	1	4	4	6	0.731	1.042	1.346	1.318	15.17	27.72	5	6	184	19.4	5.83
P12270	Nucleoprotein TPR OS=Homo sapiens GN=TPR PE=1 SV=3 - [TPR_HUMAN]	38.21	2	84	89	320	1.166	1.253	0.956	1.318	742.87	38.21	136	320	2363	267.1	5.02
Q8NEV4	Myosin-IIIa OS=Homo sapiens GN=MYO3A PE=2 SV=2 - [MYO3A_HUMAN]	0.93	1	2	2	2	2.060	0.215	0.339	1.319	2.27	0.93	2	2	1616	186.1	8.91
Q9H9T3	Elongator complex protein 3 OS=Homo sapiens GN=ELP3 PE=1 SV=2 - [ELP3_HUMAN]	5.30	1	3	3	10	0.598	1.161	1.095	1.319	18.78	5.30	5	10	547	62.2	8.88
Q0D2K2	Kelch-like protein 30 OS=Homo sapiens GN=KLHL30 PE=2 SV=3 - [KLH30_HUMAN]	4.50	1	1	1	2	2.666	1.089	0.960	1.320	5.08	4.50	1	2	578	63.9	5.94
Q8IVL5	Prolyl 3-hydroxylase 2 OS=Homo sapiens GN=LEPREL1 PE=1 SV=1 - [P3H2_HUMAN]	29.10	1	14	16	54	1.756	0.447	0.809	1.320	146.30	29.10	26	54	708	80.9	5.71

Q9Y605	MORF4 family-associated protein 1 OS=Homo sapiens GN=MRFAP1 PE=1 SV=1 - [MOFA1_HUMAN]	25.20	1	2	2	8	0.766	0.868	1.025	1.320	16.35	25.20	4	8	127	14.6	4.70
O75914	Serine/threonine-protein kinase PAK 3 OS=Homo sapiens GN=PAK3 PE=1 SV=2 - [PAK3_HUMAN]	9.66	1	1	5	33	0.748	1.823	2.085	1.321	73.58	9.66	9	33	559	62.3	5.45
P17844	Probable ATP-dependent RNA helicase DDX5 OS=Homo sapiens GN=DDX5 PE=1 SV=1 - [DDX5_HUMAN]	39.58	1	23	30	204	1.159	1.073	0.884	1.321	448.38	39.58	46	204	614	69.1	8.92
O60476	Mannosyl-oligosaccharide 1,2-alpha-mannosidase IB OS=Homo sapiens GN=MAN1A2 PE=1 SV=1 - [MA1A2_HUMAN]	7.02	1	4	5	9	0.887	1.338	1.049	1.321	21.12	7.02	6	9	641	73.0	7.61
Q8NB16	Mixed lineage kinase domain-like protein OS=Homo sapiens GN=MLKL PE=1 SV=1 - [MLKL_HUMAN]	7.86	1	5	5	7	1.091	0.902	1.012	1.321	11.17	7.86	6	7	471	54.4	8.82
P31153	S-adenosylmethionine synthase isoform type-2 OS=Homo sapiens GN=MAT2A PE=1 SV=1 - [METK2_HUMAN]	36.20	2	14	14	52	0.960	0.849	0.918	1.321	138.62	36.20	23	52	395	43.6	6.48
Q9BUL5	PHD finger protein 23 OS=Homo sapiens GN=PHF23 PE=1 SV=1 - [PHF23_HUMAN]	6.20	1	2	2	6	1.021	1.825	1.316	1.321	14.69	6.20	4	6	403	43.8	5.78
Q9BVV7	Mitochondrial import inner membrane translocase subunit Tim21 OS=Homo sapiens GN=TIMM21 PE=1 SV=1 - [TIM21_HUMAN]	25.40	1	7	7	14	1.144	0.924	0.738	1.321	31.22	25.40	12	14	248	28.2	9.70

Q8N108	Mesoderm induction early response protein 1 OS=Homo sapiens GN=MIER1 PE=1 SV=2 - [MIER1_HUMAN]	1.76	1	1	1	1	1.367	1.016	1.362	1.322	0.00	1.76	1	1	512	57.9	4.42
Q13573	SNW domain-containing protein 1 OS=Homo sapiens GN=SNW1 PE=1 SV=1 - [SNW1_HUMAN]	19.03	1	10	10	41	1.173	1.532	0.976	1.322	74.21	19.03	18	41	536	61.5	9.52
O75676	Ribosomal protein S6 kinase alpha-4 OS=Homo sapiens GN=RPS6KA4 PE=1 SV=1 - [KS6A4_HUMAN]	3.11	1	2	2	4	1.121	1.429	1.291	1.322	9.59	3.11	3	4	772	85.6	8.28
P50747	Biotin--protein ligase OS=Homo sapiens GN=HLCS PE=1 SV=1 - [BPL1_HUMAN]	4.96	1	3	3	9	1.372	1.207	1.179	1.322	13.49	4.96	6	9	726	80.7	5.62
Q13084	39S ribosomal protein L28, mitochondrial OS=Homo sapiens GN=MRPL28 PE=1 SV=4 - [RM28_HUMAN]	21.48	1	4	4	5	1.376	1.144	0.956	1.323	15.66	21.48	4	5	256	30.1	8.29
Q03519	Antigen peptide transporter 2 OS=Homo sapiens GN=TAP2 PE=1 SV=1 - [TAP2_HUMAN]	12.83	1	8	8	14	2.232	0.766	0.921	1.323	33.46	12.83	11	14	686	75.6	8.02
P18858	DNA ligase 1 OS=Homo sapiens GN=LIG1 PE=1 SV=1 - [DNLI1_HUMAN]	2.07	1	2	2	2	1.082	0.685	0.765	1.323	4.12	2.07	2	2	919	101.7	5.62
P17275	Transcription factor jun-B OS=Homo sapiens GN=JUNB PE=1 SV=1 - [JUNB_HUMAN]	7.20	1	2	2	5	0.875	0.999	0.878	1.324	4.64	7.20	4	5	347	35.9	9.22
Q86Y79	Probable peptidyl-tRNA hydrolase OS=Homo sapiens GN=PTRH1 PE=1 SV=1 - [PTH_HUMAN]	4.67	1	1	1	2	1.488	1.287	1.042	1.324	9.05	4.67	2	2	214	22.9	10.56

P98175	RNA-binding protein 10 OS=Homo sapiens GN=RBM10 PE=1 SV=3 - [RBM10_HUMAN]	16.34	1	13	15	30	1.288	1.039	1.011	1.324	68.51	16.34	22	30	930	103.5	5.97
O15116	U6 snRNA-associated Sm-like protein LSm1 OS=Homo sapiens GN=LSM1 PE=1 SV=1 - [LSM1_HUMAN]	9.77	1	2	2	2	1.671	1.145	1.325	1.324	4.50	9.77	2	2	133	15.2	5.22
Q9BYX4	Interferon-induced helicase C domain-containing protein 1 OS=Homo sapiens GN=IFIH1 PE=1 SV=3 - [IFIH1_HUMAN]	1.46	1	1	1	1	1.288	1.049	1.213	1.325	2.85	1.46	1	1	1025	116.6	5.52
O00422	Histone deacetylase complex subunit SAP18 OS=Homo sapiens GN=SAP18 PE=1 SV=1 - [SAP18_HUMAN]	50.98	1	8	8	38	0.960	1.278	0.838	1.325	86.55	50.98	13	38	153	17.5	9.35
Q13636	Ras-related protein Rab-31 OS=Homo sapiens GN=RAB31 PE=1 SV=1 - [RAB31_HUMAN]	21.65	1	3	4	13	0.750	0.685	0.878	1.325	32.47	21.65	7	13	194	21.6	7.06
P35226	Polycomb complex protein BMI-1 OS=Homo sapiens GN=BMI1 PE=1 SV=2 - [BMI1_HUMAN]	5.21	1	1	1	1	0.766	1.457	1.608	1.325	3.41	5.21	1	1	326	36.9	8.63
P29590	Protein PML OS=Homo sapiens GN=PML PE=1 SV=3 - [PML_HUMAN]	26.64	1	23	23	98	1.729	1.009	1.034	1.325	214.52	26.64	39	98	882	97.5	6.21
P51532	Transcription activator BRG1 OS=Homo sapiens GN=SMARCA4 PE=1 SV=2 - [SMCA4_HUMAN]	10.44	1	9	17	50	1.326	1.294	0.929	1.326	136.13	10.44	25	50	1647	184.5	7.88
P0C7P3	Schlafen family member 14 OS=Homo sapiens GN=SLFN14 PE=2 SV=2 - [SLN14_HUMAN]	0.99	1	1	1	1	0.755	0.798	0.977	1.326	0.00	0.99	1	1	912	103.8	8.24

P37837	Transaldolase OS=Homo sapiens GN=TALDO1 PE=1 SV=2 - [TALDO_HUMAN]	52.52	1	24	24	512	1.392	0.632	1.052	1.326	1287.85	52.52	41	512	337	37.5	6.81
Q8WZA1	Protein O-linked-mannose beta-1,2-N-acetylglucosaminyltransferase 1 OS=Homo sapiens GN=POMGNT1 PE=1 SV=2 - [PMGT1_HUMAN]	1.36	1	1	1	2	0.637	1.855	1.230	1.326	4.43	1.36	2	2	660	75.2	6.83
Q7L8L6	FAST kinase domain-containing protein 5 OS=Homo sapiens GN=FASTKD5 PE=1 SV=1 - [FAKD5_HUMAN]	2.62	1	2	2	5	0.960	0.918	0.799	1.326	10.20	2.62	4	5	764	86.5	8.13
P15621	Zinc finger protein 44 OS=Homo sapiens GN=ZNF44 PE=2 SV=3 - [ZNF44_HUMAN]	6.79	28	1	2	3	0.824	0.735	1.208	1.327	2.55	6.79	2	3	663	76.5	8.68
Q9Y394	Dehydrogenase/reductase SDR family member 7 OS=Homo sapiens GN=DHRS7 PE=1 SV=1 - [DHRS7_HUMAN]	28.61	1	10	10	54	1.336	1.007	1.036	1.327	110.16	28.61	17	54	339	38.3	8.32
Q8WVV9	Heterogeneous nuclear ribonucleoprotein L-like OS=Homo sapiens GN=HNRNPLL PE=1 SV=1 - [HNRLL_HUMAN]	22.14	1	9	10	45	1.132	1.266	1.066	1.327	105.41	22.14	18	45	542	60.0	7.72
Q9NP92	28S ribosomal protein S30, mitochondrial OS=Homo sapiens GN=MRPS30 PE=1 SV=2 - [RT30_HUMAN]	5.92	1	2	2	5	1.277	1.343	1.141	1.327	8.18	5.92	3	5	439	50.3	7.97
O95563	Mitochondrial pyruvate carrier 2 OS=Homo sapiens GN=MPC2 PE=1 SV=1 - [MPC2_HUMAN]	9.45	1	1	1	2	1.367	0.874	0.770	1.328	0.00	9.45	2	2	127	14.3	10.43
Q8N983	39S ribosomal protein L43, mitochondrial OS=Homo sapiens GN=MRPL43 PE=1 SV=1 - [RM43_HUMAN]	13.49	1	3	3	19	0.870	1.242	1.021	1.328	37.23	13.49	4	19	215	23.4	8.65

Q8TDM6	Disks large homolog 5 OS=Homo sapiens GN=DLG5 PE=1 SV=4 - [DLG5_HUMAN]	3.60	1	7	7	16	0.877	0.930	1.155	1.328	40.55	3.60	9	16	1919	213.7	7.42
Q68CQ4	Digestive organ expansion factor homolog OS=Homo sapiens GN=DIEXF PE=1 SV=2 - [DIEXF_HUMAN]	3.84	1	3	3	6	0.875	0.917	1.084	1.328	5.33	3.84	5	6	756	87.0	5.88
P07311	Acylphosphatase-1 OS=Homo sapiens GN=ACYP1 PE=1 SV=2 - [ACYP1_HUMAN]	39.39	1	4	4	8	1.239	1.910	1.140	1.328	21.97	39.39	5	8	99	11.3	9.31
Q9BYC8	39S ribosomal protein L32, mitochondrial OS=Homo sapiens GN=MRPL32 PE=1 SV=1 - [RM32_HUMAN]	12.23	1	1	1	4	1.345	1.614	0.894	1.328	15.34	12.23	2	4	188	21.4	9.73
B2RTY4	Unconventional myosin-IXa OS=Homo sapiens GN=MYO9A PE=1 SV=2 - [MYO9A_HUMAN]	2.43	1	3	4	4	1.563	1.361	1.079	1.328	0.00	2.43	4	4	2548	292.5	8.88
O95466	Formin-like protein 1 OS=Homo sapiens GN=FMNL1 PE=1 SV=3 - [FMNL_HUMAN]	4.64	1	4	5	6	1.570	0.640	0.510	1.329	8.27	4.64	6	6	1100	121.8	5.72
O75746	Calcium-binding mitochondrial carrier protein Aralar1 OS=Homo sapiens GN=SLC25A12 PE=1 SV=2 - [CMC1_HUMAN]	24.34	1	10	14	54	1.410	1.200	1.106	1.329	123.69	24.34	22	54	678	74.7	8.38
Q9UPT8	Zinc finger CCCH domain-containing protein 4 OS=Homo sapiens GN=ZC3H4 PE=1 SV=3 - [ZC3H4_HUMAN]	7.98	1	7	7	27	1.238	1.661	1.117	1.329	63.81	7.98	10	27	1303	140.2	6.27
P38159	RNA-binding motif protein, X chromosome OS=Homo sapiens GN=RBMX PE=1 SV=3 - [RBMX_HUMAN]	31.20	2	5	13	75	1.304	1.769	1.336	1.329	174.14	31.20	21	75	391	42.3	10.05

Q02790	Peptidyl-prolyl cis-trans isomerase FKBP4 OS=Homo sapiens GN=FKBP4 PE=1 SV=3 - [FKBP4_HUMAN]	34.86	1	14	15	52	0.941	0.828	0.987	1.330	137.36	34.86	22	52	459	51.8	5.43
Q9UKD1	Glucocorticoid modulatory element-binding protein 2 OS=Homo sapiens GN=GMEB2 PE=1 SV=1 - [GMEB2_HUMAN]	1.32	1	1	1	3	1.042	1.032	1.108	1.330	6.05	1.32	2	3	530	56.4	5.60
Q8NF37	Lysophosphatidylcholine acyltransferase 1 OS=Homo sapiens GN=LPCAT1 PE=1 SV=2 - [PCAT1_HUMAN]	2.62	1	1	2	5	1.092	0.621	0.476	1.331	13.14	2.62	3	5	534	59.1	6.02
Q99558	Mitogen-activated protein kinase kinase kinase 14 OS=Homo sapiens GN=MAP3K14 PE=1 SV=2 - [M3K14_HUMAN]	0.95	1	1	1	1	1.065	1.050	0.766	1.331	2.33	0.95	1	1	947	104.0	7.72
A4D1P6	WD repeat-containing protein 91 OS=Homo sapiens GN=WDR91 PE=1 SV=2 - [WDR91_HUMAN]	3.61	1	2	3	4	1.891	1.538	1.078	1.331	10.21	3.61	4	4	747	83.3	6.58
Q86U44	N6-adenosine-methyltransferase 70 kDa subunit OS=Homo sapiens GN=METTL3 PE=1 SV=2 - [MTA70_HUMAN]	6.03	1	3	3	5	1.599	1.567	1.267	1.331	9.88	6.03	4	5	580	64.4	6.42
Q5VTR2	E3 ubiquitin-protein ligase BRE1A OS=Homo sapiens GN=RNF20 PE=1 SV=2 - [BRE1A_HUMAN]	7.59	1	4	7	22	1.233	1.309	1.227	1.331	52.62	7.59	10	22	975	113.6	5.94
Q15637	Splicing factor 1 OS=Homo sapiens GN=SF1 PE=1 SV=4 - [SF01_HUMAN]	22.38	1	11	12	73	1.117	1.261	1.240	1.332	186.45	22.38	18	73	639	68.3	8.98

Q13769	THO complex subunit 5 homolog OS=Homo sapiens GN=THOC5 PE=1 SV=2 - [THOC5_HUMAN]	7.03	1	5	5	15	1.012	1.191	0.772	1.332	30.21	7.03	8	15	683	78.5	6.87
P41002	Cyclin-F OS=Homo sapiens GN=CCNF PE=1 SV=2 - [CCNF_HUMAN]	1.53	1	1	1	1	0.889	3.214	6.256	1.332	0.00	1.53	1	1	786	87.6	6.33
Q9H9V4	RING finger protein 122 OS=Homo sapiens GN=RNF122 PE=2 SV=2 - [RN122_HUMAN]	12.26	1	1	1	2	1.808	3.655	1.284	1.332	0.00	12.26	1	2	155	17.5	7.88
P56715	Oxygen-regulated protein 1 OS=Homo sapiens GN=RP1 PE=1 SV=1 - [RP1_HUMAN]	1.86	1	3	3	3	1.138	0.838	1.013	1.332	4.46	1.86	3	3	2156	240.5	5.80
Q6PJ69	Tripartite motif-containing protein 65 OS=Homo sapiens GN=TRIM65 PE=1 SV=3 - [TRI65_HUMAN]	3.87	1	2	2	5	1.247	1.066	1.104	1.333	10.02	3.87	4	5	517	57.3	6.70
O43818	U3 small nucleolar RNA-interacting protein 2 OS=Homo sapiens GN=RRP9 PE=1 SV=1 - [U3IP2_HUMAN]	6.53	1	3	3	4	1.029	0.955	0.937	1.333	6.35	6.53	3	4	475	51.8	7.85
Q9BSJ5	Uncharacterized protein C17orf80 OS=Homo sapiens GN=C17orf80 PE=2 SV=2 - [CQ080_HUMAN]	9.20	1	4	4	6	0.927	1.821	1.535	1.333	8.37	9.20	5	6	609	67.3	9.16
Q9HCH0	Nck-associated protein 5-like OS=Homo sapiens GN=NCKAP5L PE=1 SV=2 - [NCK5L_HUMAN]	1.95	1	2	2	3	1.399	2.278	2.341	1.334	3.83	1.95	3	3	1330	138.9	8.13
Q9H3P2	Negative elongation factor A OS=Homo sapiens GN=NELFA PE=1 SV=3 - [NELFA_HUMAN]	12.31	1	5	5	14	1.569	1.386	1.206	1.334	40.67	12.31	7	14	528	57.2	9.03

Q13315	Serine-protein kinase ATM OS=Homo sapiens GN=ATM PE=1 SV=4 - [ATM_HUMAN]	3.53	1	9	10	22	1.046	1.373	1.228	1.334	44.92	3.53	13	22	3056	350.5	6.81
Q5T200	Zinc finger CCCH domain-containing protein 13 OS=Homo sapiens GN=ZC3H13 PE=1 SV=1 - [ZC3HD_HUMAN]	3.48	1	5	6	9	1.183	1.253	1.140	1.334	22.83	3.48	8	9	1668	196.5	9.42
P81605	Dermcidin OS=Homo sapiens GN=DCD PE=1 SV=2 - [DCD_HUMAN]	6.36	1	1	1	1	0.823	1.887	1.022	1.335	2.03	6.36	1	1	110	11.3	6.54
Q86V25	Vasohibin-2 OS=Homo sapiens GN=VASH2 PE=1 SV=2 - [VASH2_HUMAN]	2.82	1	1	1	1	2.603	0.544	1.043	1.335	1.71	2.82	1	1	355	40.4	10.01
Q8NCN5	Pyruvate dehydrogenase phosphatase regulatory subunit, mitochondrial OS=Homo sapiens GN=PDPR PE=1 SV=2 - [PDPR_HUMAN]	13.08	1	10	10	15	1.146	1.175	0.997	1.336	31.71	13.08	12	15	879	99.3	6.35
Q96I99	Succinyl-CoA ligase [GDP-forming] subunit beta, mitochondrial OS=Homo sapiens GN=SUCLG2 PE=1 SV=2 - [SUCB2_HUMAN]	46.30	1	20	20	84	1.123	1.041	0.928	1.336	193.61	46.30	38	84	432	46.5	6.39
O60290	Zinc finger protein 862 OS=Homo sapiens GN=ZNF862 PE=2 SV=2 - [ZN862_HUMAN]	0.51	1	1	1	1	1.036	1.158	0.862	1.336	0.00	0.51	1	1	1169	131.6	7.23
Q9P2V4	Leucine-rich repeat, immunoglobulin-like domain and transmembrane domain-containing protein 1 OS=Homo sapiens GN=LRIT1 PE=2 SV=1 - [LRIT1_HUMAN]	6.58	1	1	2	2	0.911	0.649	1.093	1.336	2.83	6.58	2	2	623	68.0	8.54

B9ZVM9	Putative t-complex protein 10A homolog 2 OS=Homo sapiens GN=TCP10L2 PE=5 SV=2 - [TCP2L_HUMAN	4.25	1	1	1	1		3.581	2.025	1.337	0.00	4.25	1	1	353	38.3	7.66
P33121	Long-chain-fatty-acid--CoA ligase 1 OS=Homo sapiens GN=ACSL1 PE=1 SV=1 - [ACSL1_HUMAN]	28.37	2	17	19	46	0.906	0.979	0.807	1.337	94.40	28.37	29	46	698	77.9	7.15
Q2M1Z3	Rho GTPase-activating protein 31 OS=Homo sapiens GN=ARHGAP31 PE=1 SV=2 - [RHG31_HUMAN]	3.12	1	3	3	4	0.697	1.127	1.100	1.337	7.52	3.12	4	4	1444	156.9	5.76
P07199	Major centromere autoantigen B OS=Homo sapiens GN=CENPB PE=1 SV=2 - [CENPB_HUMAN]	2.00	1	1	1	1	1.285	1.618	1.331	1.337	2.66	2.00	1	1	599	65.1	4.55
Q6W0C5	Developmental pluripotency-associated protein 3 OS=Homo sapiens GN=DPPA3 PE=2 SV=1 - [DPPA3_HUMAN]	4.40	1	1	1	1	1.055	0.662	0.690	1.338	1.88	4.40	1	1	159	17.8	8.63
Q8ND30	Liprin-beta-2 OS=Homo sapiens GN=PPFIBP2 PE=1 SV=3 - [LIPB2_HUMAN]	5.25	1	2	4	8	1.434	0.953	0.931	1.338	19.85	5.25	5	8	876	98.5	6.27
Q8TB40	Abhydrolase domain-containing protein 4 OS=Homo sapiens GN=ABHD4 PE=2 SV=1 - [ABHD4_HUMAN]	2.63	1	1	1	2	0.583	0.945	1.663	1.338	5.91	2.63	2	2	342	38.8	7.59
Q9UBT7	Alpha-catulin OS=Homo sapiens GN=CTNNAL1 PE=1 SV=2 - [CTNL1_HUMAN]	4.22	1	3	3	4	1.108	1.214	1.153	1.338	6.78	4.22	4	4	734	81.8	6.64
Q6PJT7	Zinc finger CCCH domain-containing protein 14 OS=Homo sapiens GN=ZC3H14 PE=1 SV=1 - [ZC3HE_HUMAN]	19.02	1	11	11	28	1.078	1.345	1.003	1.338	65.81	19.02	18	28	736	82.8	7.31

Q13895	Bystin OS=Homo sapiens GN=BYSL PE=1 SV=3 - [BYST_HUMAN]	18.08	1	9	9	17	0.741	0.974	0.906	1.339	39.64	18.08	12	17	437	49.6	8.12
P60468	Protein transport protein Sec61 subunit beta OS=Homo sapiens GN=SEC61B PE=1 SV=2 - [SC61B_HUMAN]	21.88	1	2	2	62	0.769	1.008	1.019	1.339	132.25	21.88	4	62	96	10.0	11.56
P40425	Pre-B-cell leukemia transcription factor 2 OS=Homo sapiens GN=PBX2 PE=1 SV=2 - [PBX2_HUMAN]	6.05	1	1	2	3	0.899	1.038	0.900	1.340	6.69	6.05	2	3	430	45.9	7.56
O43660	Pleiotropic regulator 1 OS=Homo sapiens GN=PLRG1 PE=1 SV=1 - [PLRG1_HUMAN]	14.01	1	4	4	10	1.067	1.135	0.910	1.340	28.64	14.01	5	10	514	57.2	9.17
Q15022	Polycomb protein SUZ12 OS=Homo sapiens GN=SUZ12 PE=1 SV=3 - [SUZ12_HUMAN]	1.35	1	1	1	3	0.921	1.252	0.992	1.340	4.76	1.35	2	3	739	83.0	8.81
P52434	DNA-directed RNA polymerases I, II, and III subunit RPABC3 OS=Homo sapiens GN=POLR2H PE=1 SV=4 - [RPAB3_HUMAN]	15.33	1	2	2	7	1.715	1.845	1.495	1.340	14.57	15.33	3	7	150	17.1	4.68
Q9NS73	MAP3K12-binding inhibitory protein 1 OS=Homo sapiens GN=MBIP PE=1 SV=2 - [MBIP1_HUMAN]	4.36	1	1	2	4	1.086	1.351	1.273	1.341	5.55	4.36	3	4	344	39.3	7.24
P52209	6-phosphogluconate dehydrogenase, decarboxylating OS=Homo sapiens GN=PGD PE=1 SV=3 - [6PGD_HUMAN]	55.69	1	29	31	372	1.546	0.546	0.862	1.341	946.62	55.69	50	372	483	53.1	7.23

Q00403	Transcription initiation factor IIB OS=Homo sapiens GN=GTF2B PE=1 SV=1 - [TF2B_HUMAN]	22.47	1	5	6	13	1.203	1.398	1.109	1.341	32.34	22.47	9	13	316	34.8	8.35
Q03701	CCAAT/enhancer-binding protein zeta OS=Homo sapiens GN=CEBPZ PE=1 SV=3 - [CEBPZ_HUMAN]	6.36	1	6	6	14	0.802	0.923	0.848	1.341	29.85	6.36	10	14	1054	120.9	5.94
Q7Z5L9	Interferon regulatory factor 2-binding protein 2 OS=Homo sapiens GN=IRF2BP2 PE=1 SV=2 - [I2BP2_HUMAN]	17.38	1	7	8	23	1.122	1.493	0.860	1.341	41.78	17.38	10	23	587	61.0	8.69
P27487	Dipeptidyl peptidase 4 OS=Homo sapiens GN=DPP4 PE=1 SV=2 - [DPP4_HUMAN]	38.12	1	30	30	132	2.968	0.563	0.697	1.341	310.10	38.12	46	132	766	88.2	6.04
P36508	Zinc finger protein 76 OS=Homo sapiens GN=ZNF76 PE=2 SV=2 - [ZNF76_HUMAN]	1.58	2	1	1	4	1.014	1.129	1.147	1.341	5.32	1.58	2	4	570	61.8	6.05
Q70EK8	Inactive ubiquitin carboxyl-terminal hydrolase 53 OS=Homo sapiens GN=USP53 PE=2 SV=2 - [UBP53_HUMAN]	1.30	1	1	1	1	1.519	1.062	1.268	1.342	2.25	1.30	1	1	1073	120.7	7.59
Q9BRS2	Serine/threonine-protein kinase RIO1 OS=Homo sapiens GN=RIOK1 PE=1 SV=2 - [RIOK1_HUMAN]	3.87	1	2	2	5	0.825	0.864	0.911	1.342	10.87	3.87	3	5	568	65.5	6.19
P62306	Small nuclear ribonucleoprotein F OS=Homo sapiens GN=SNRPF PE=1 SV=1 - [RUXF_HUMAN]	9.30	1	1	1	1	1.054	0.915	1.116	1.342	2.18	9.30	1	1	86	9.7	4.67

P36551	Oxygen-dependent coproporphyrinogen-III oxidase, mitochondrial OS=Homo sapiens GN=CPOX PE=1 SV=3 - [HEM6_HUMAN]	25.11	1	10	10	24	1.041	1.328	1.173	1.343	64.76	25.11	17	24	454	50.1	8.25
O94868	FCH and double SH3 domains protein 2 OS=Homo sapiens GN=FCHSD2 PE=1 SV=3 - [FCSD2_HUMAN	2.84	1	2	3	8	0.863	0.964	1.429	1.343	11.83	2.84	4	8	740	84.2	5.77
Q8TBM8	DnaJ homolog subfamily B member 14 OS=Homo sapiens GN=DNAJB14 PE=1 SV=1 - [DJB14_HUMAN]	3.69	1	2	2	3	1.005	0.877	1.026	1.344	4.34	3.69	3	3	379	42.5	8.59
O15160	DNA-directed RNA polymerases I and III subunit RPAC1 OS=Homo sapiens GN=POLR1C PE=1 SV=1 - [RPAC1_HUMAN]	26.59	1	7	7	21	0.906	0.914	1.038	1.344	49.46	26.59	10	21	346	39.2	5.50
Q96P20	NACHT, LRR and PYD domains-containing protein 3 OS=Homo sapiens GN=NLRP3 PE=1 SV=3 - [NALP3_HUMAN]	1.06	1	1	1	1	2.958	0.897	1.124	1.344	0.00	1.06	1	1	1036	118.1	6.65
Q9UBI9	Headcase protein homolog OS=Homo sapiens GN=HECA PE=1 SV=1 - [HDC_HUMAN]	1.84	1	1	1	1	0.638	1.882	1.365	1.344	0.00	1.84	1	1	543	58.8	8.54
Q86WV6	Stimulator of interferon genes protein OS=Homo sapiens GN=TMEM173 PE=1 SV=1 - [STING_HUMAN]	28.23	1	7	7	20	1.743	1.466	1.158	1.345	46.12	28.23	11	20	379	42.2	7.05
O14718	Visual pigment-like receptor peropsin OS=Homo sapiens GN=RRH PE=1 SV=1 - [OPSX_HUMAN]	2.97	1	1	1	1	1.160			1.345	2.90	2.97	1	1	337	37.4	8.48

Q49MG5	Microtubule-associated protein 9 OS=Homo sapiens GN=MAP9 PE=1 SV=3 - [MAP9_HUMAN]	1.39	1	1	1	2	1.070	1.370	1.365	1.345	3.02	1.39	2	2	647	74.2	7.80
Q9C098	Serine/threonine-protein kinase DCLK3 OS=Homo sapiens GN=DCLK3 PE=1 SV=2 - [DCLK3_HUMAN]	4.01	1	2	3	4	1.270	1.118	1.219	1.346	9.68	4.01	3	4	648	73.8	8.84
Q96AQ6	Pre-B-cell leukemia transcription factor-interacting protein 1 OS=Homo sapiens GN=PBXIP1 PE=1 SV=1 - [PBIP1_HUMAN]	31.33	1	21	21	74	1.412	1.355	1.501	1.346	166.75	31.33	35	74	731	80.6	5.33
Q96M86	Dynein heavy chain domain-containing protein 1 OS=Homo sapiens GN=DNHD1 PE=2 SV=2 - [DNHD1_HUMAN]	1.05	1	4	5	8	1.052	0.867	1.146	1.346	13.06	1.05	6	8	4753	533.3	6.71
P56182	Ribosomal RNA processing protein 1 homolog A OS=Homo sapiens GN=RRP1 PE=1 SV=1 - [RRP1_HUMAN]	2.60	1	1	1	5	0.808	1.116	1.061	1.347	11.35	2.60	2	5	461	52.8	9.33
Q9UI30	Multifunctional methyltransferase subunit TRM112-like protein OS=Homo sapiens GN=TRMT112 PE=1 SV=1 - [TR112_HUMAN]	39.20	1	4	4	7	0.683	0.961	1.156	1.347	20.22	39.20	6	7	125	14.2	5.26
Q99567	Nuclear pore complex protein Nup88 OS=Homo sapiens GN=NUP88 PE=1 SV=2 - [NUP88_HUMAN]	12.55	1	8	8	19	1.110	1.133	1.011	1.347	47.98	12.55	11	19	741	83.5	5.69
Q9Y6G3	39S ribosomal protein L42, mitochondrial OS=Homo sapiens GN=MRPL42 PE=1 SV=1 - [RM42_HUMAN]	21.13	1	3	3	9	1.096	1.229	1.631	1.348	22.78	21.13	4	9	142	16.7	8.35

O00625	Pirin OS=Homo sapiens GN=PIR PE=1 SV=1 - [PIR_HUMAN]	16.55	1	4	4	6	1.375	0.512	0.727	1.348	11.70	16.55	4	6	290	32.1	6.92
Q96N95	Zinc finger protein 396 OS=Homo sapiens GN=ZNF396 PE=1 SV=2 - [ZN396_HUMAN]	2.39	1	1	1	1	0.700	0.670	0.820	1.348	2.43	2.39	1	1	335	38.6	8.05
Q63HQ2	Pikachurin OS=Homo sapiens GN=EGFLAM PE=1 SV=2 - [EGFLA_HUMAN]	1.38	1	1	1	2	1.985	0.548	1.098	1.348	0.00	1.38	1	2	1017	111.2	7.40
P29558	RNA-binding motif, single-stranded-interacting protein 1 OS=Homo sapiens GN=RBMS1 PE=1 SV=3 - [RBMS1_HUMAN]	17.24	1	3	7	23	1.259	0.836	1.017	1.348	61.07	17.24	11	23	406	44.5	8.79
Q6P1L8	39S ribosomal protein L14, mitochondrial OS=Homo sapiens GN=MRPL14 PE=1 SV=1 - [RM14_HUMAN]	22.76	1	2	2	6	1.179	1.011	1.079	1.348	18.26	22.76	3	6	145	15.9	10.24
Q7Z333	Probable helicase senataxin OS=Homo sapiens GN=SETX PE=1 SV=4 - [SETX_HUMAN]	1.87	2	3	5	10	0.772	1.034	0.891	1.349	8.44	1.87	5	10	2677	302.7	7.17
P23246	Splicing factor, proline- and glutamine-rich OS=Homo sapiens GN=SFPQ PE=1 SV=2 - [SFPQ_HUMAN]	38.76	1	26	27	192	1.100	1.303	1.042	1.349	446.41	38.76	44	192	707	76.1	9.44
Q8WUI4	Histone deacetylase 7 OS=Homo sapiens GN=HDAC7 PE=1 SV=2 - [HDAC7_HUMAN]	4.10	1	4	4	25	0.744	0.956	1.020	1.349	47.82	4.10	7	25	952	102.9	7.58
O95926	Pre-mRNA-splicing factor SYF2 OS=Homo sapiens GN=SYF2 PE=1 SV=1 - [SYF2_HUMAN]	15.23	1	3	3	5	1.056	1.108	0.953	1.350	2.48	15.23	4	5	243	28.7	8.40

Q9HAV4	Exportin-5 OS=Homo sapiens GN=XPO5 PE=1 SV=1 - [XPO5_HUMAN]	4.15	1	5	5	12	1.015	0.856	0.873	1.350	25.22	4.15	8	12	1204	136.2	5.80
Q5K4L6	Long-chain fatty acid transport protein 3 OS=Homo sapiens GN=SLC27A3 PE=2 SV=3 - [S27A3_HUMAN]	6.03	1	3	3	5	1.389	1.332	0.815	1.350	18.08	6.03	4	5	730	78.6	7.49
Q9NTZ6	RNA-binding protein 12 OS=Homo sapiens GN=RBM12 PE=1 SV=1 - [RBM12_HUMAN]	11.59	1	9	9	33	1.203	1.232	1.013	1.350	76.91	11.59	16	33	932	97.3	8.63
Q6R327	Rapamycin-insensitive companion of mTOR OS=Homo sapiens GN=RICTOR PE=1 SV=1 - [RICTR_HUMAN]	2.11	1	3	3	3	1.009	0.968	0.781	1.350	7.52	2.11	3	3	1708	192.1	7.47
Q92754	Transcription factor AP-2 gamma OS=Homo sapiens GN=TFAP2C PE=1 SV=1 - [AP2C_HUMAN]	2.89	1	1	2	8	3.748	1.423	1.144	1.350	18.08	2.89	2	8	450	49.1	7.83
P00390	Glutathione reductase, mitochondrial OS=Homo sapiens GN=GSR PE=1 SV=2 - [GSHR_HUMAN]	32.76	1	11	12	56	1.721	0.659	0.826	1.350	139.57	32.76	19	56	522	56.2	8.50
O43181	NADH dehydrogenase [ubiquinone] iron-sulfur protein 4, mitochondrial OS=Homo sapiens GN=NDUFS4 PE=1 SV=1 - [NDUS4_HUMAN]	32.00	1	5	5	30	0.671	1.038	0.817	1.351	86.28	32.00	8	30	175	20.1	10.30
O43303	Centriolar coiled-coil protein of 110 kDa OS=Homo sapiens GN=CCP110 PE=1 SV=3 - [CP110_HUMAN]	5.04	1	3	4	8	1.064	1.262	1.007	1.351	18.43	5.04	6	8	1012	113.4	8.65

Q5T3U5	Multidrug resistance-associated protein 7 OS=Homo sapiens GN=ABCC10 PE=1 SV=1 - [MRP7_HUMAN]	0.47	1	1	1	2	0.938	1.020	0.932	1.351	3.68	0.47	1	2	1492	161.5	7.08
A2RUS2	DENN domain-containing protein 3 OS=Homo sapiens GN=DENND3 PE=1 SV=2 - [DEND3_HUMAN]	3.67	1	5	5	13	1.484	0.931	1.736	1.352	26.46	3.67	7	13	1198	135.8	7.06
P04920	Anion exchange protein 2 OS=Homo sapiens GN=SLC4A2 PE=1 SV=4 - [B3A2_HUMAN]	0.73	1	1	1	3	2.355	0.822	1.313	1.352	0.00	0.73	2	3	1241	136.9	6.29
Q6V9R5	Zinc finger protein 562 OS=Homo sapiens GN=ZNF562 PE=2 SV=2 - [ZN562_HUMAN]	2.11	2	1	1	1	1.617	0.749	0.821	1.352	1.84	2.11	1	1	426	48.5	8.41
Q9NRK6	ATP-binding cassette sub-family B member 10, mitochondrial OS=Homo sapiens GN=ABCB10 PE=1 SV=2 - [ABCBA_HUMAN]	2.17	1	2	2	2	0.978	2.050	1.333	1.353	4.49	2.17	2	2	738	79.1	9.85
Q16629	Serine/arginine-rich splicing factor 7 OS=Homo sapiens GN=SRSF7 PE=1 SV=1 - [SRSF7_HUMAN]	19.75	1	4	5	45	1.275	1.229	1.012	1.353	103.14	19.75	10	45	238	27.4	11.82
P11387	DNA topoisomerase 1 OS=Homo sapiens GN=TOP1 PE=1 SV=2 - [TOP1_HUMAN]	34.51	1	23	26	67	0.993	1.123	0.817	1.353	133.67	34.51	39	67	765	90.7	9.31
Q9HBU6	Ethanolamine kinase 1 OS=Homo sapiens GN=ETNK1 PE=1 SV=1 - [EKI1_HUMAN]	2.65	1	1	1	4	0.675	0.887	1.153	1.353	13.97	2.65	2	4	452	50.9	6.54
Q9UHL4	Dipeptidyl peptidase 2 OS=Homo sapiens GN=DPP7 PE=1 SV=3 - [DPP2_HUMAN]	26.02	1	11	11	54	1.109	1.056	0.879	1.353	144.70	26.02	19	54	492	54.3	6.32

Q9NZN3	EH domain-containing protein 3 OS=Homo sapiens GN=EHD3 PE=1 SV=2 - [EHD3_HUMAN]	26.17	2	6	20	117	0.989	1.068	1.208	1.353	251.25	26.17	30	117	535	60.8	6.57
Q8N4T4	Rho guanine nucleotide exchange factor 39 OS=Homo sapiens GN=ARHGEF39 PE=2 SV=1 - [ARG39_HUMAN	8.96	1	1	1	1	0.936	0.593		1.354	2.82	8.96	1	1	335	38.3	9.64
Q9BTL4	Immediate early response gene 2 protein OS=Homo sapiens GN=IER2 PE=1 SV=1 - [IER2_HUMAN]	7.62	1	1	1	1	4.977	2.795	1.572	1.354	2.32	7.62	1	1	223	24.2	6.92
Q9BUR5	Apolipoprotein O OS=Homo sapiens GN=APOO PE=1 SV=1 - [APOO_HUMAN]	18.18	1	3	3	6	1.048	1.088	0.779	1.354	13.90	18.18	5	6	198	22.3	9.13
O96013	Serine/threonine-protein kinase PAK 4 OS=Homo sapiens GN=PAK4 PE=1 SV=1 - [PAK4_HUMAN]	3.89	2	2	3	3	1.278	1.793	0.999	1.355	7.30	3.89	3	3	591	64.0	9.73
Q9Y282	Endoplasmic reticulum-Golgi intermediate compartment protein 3 OS=Homo sapiens GN=ERGIC3 PE=1 SV=1 - [ERGI3_HUMAN]	19.58	1	7	8	26	0.952	0.848	0.884	1.355	74.69	19.58	10	26	383	43.2	6.06
A6NGH7	Coiled-coil domain-containing protein 160 OS=Homo sapiens GN=CCDC160 PE=3 SV=3 - [CC160_HUMAN]	4.62	2	1	2	3	0.824	1.395	0.844	1.356	7.44	4.62	2	3	325	38.3	5.41
Q14644	Ras GTPase-activating protein 3 OS=Homo sapiens GN=RASA3 PE=1 SV=3 - [RASA3_HUMAN]	15.47	1	13	14	26	1.511	0.779	0.927	1.356	54.91	15.47	20	26	834	95.6	7.15

Q9Y4W2	Ribosomal biogenesis protein LAS1L OS=Homo sapiens GN=LAS1L PE=1 SV=2 - [LAS1L_HUMAN]	5.18	1	3	3	7	0.654	1.292	0.936	1.356	24.89	5.18	5	7	734	83.0	4.73
Q2M3G4	Protein Shroom1 OS=Homo sapiens GN=SHROOM1 PE=1 SV=1 - [SHRM1_HUMAN]	0.94	1	1	1	1	0.900	0.586	0.780	1.357	2.19	0.94	1	1	852	90.7	6.15
Q96MU7	YTH domain-containing protein 1 OS=Homo sapiens GN=YTHDC1 PE=1 SV=3 - [YTDC1_HUMAN]	7.84	1	6	6	18	1.268	1.620	1.131	1.357	29.32	7.84	10	18	727	84.6	6.23
P14927	Cytochrome b-c1 complex subunit 7 OS=Homo sapiens GN=UQCRB PE=1 SV=2 - [QCR7_HUMAN]	66.67	1	8	8	23	1.072	1.303	0.817	1.357	52.58	66.67	13	23	111	13.5	8.78
Q8N5G0	Small integral membrane protein 20 OS=Homo sapiens GN=SMIM20 PE=1 SV=2 - [SMI20_HUMAN]	13.10	1	2	2	17	1.141	1.148	0.930	1.357	36.16	13.10	3	17	168	18.4	10.99
P37108	Signal recognition particle 14 kDa protein OS=Homo sapiens GN=SRP14 PE=1 SV=2 - [SRP14_HUMAN]	44.12	1	7	7	28	1.092	1.354	0.982	1.357	59.62	44.12	12	28	136	14.6	10.04
Q9P0V3	SH3 domain-binding protein 4 OS=Homo sapiens GN=SH3BP4 PE=1 SV=1 - [SH3B4_HUMAN]	9.24	1	8	8	17	1.245	0.762	0.976	1.357	38.28	9.24	13	17	963	107.4	7.71
O14807	Ras-related protein M-Ras OS=Homo sapiens GN=MRAS PE=1 SV=2 - [RASM_HUMAN]	3.37	1	1	1	2	1.325	0.363	0.321	1.358	5.28	3.37	2	2	208	23.8	8.79
Q02318	Sterol 26-hydroxylase, mitochondrial OS=Homo sapiens GN=CYP27A1 PE=1 SV=1 - [CP27A_HUMAN]	9.79	1	5	5	9	1.200	0.985	0.920	1.358	14.07	9.79	8	9	531	60.2	8.90

Q13825	Methylglutaconyl-CoA hydratase, mitochondrial OS=Homo sapiens GN=AUH PE=1 SV=1 - [AUHM_HUMAN]	15.93	1	4	5	8	1.219	1.641	0.921	1.358	20.58	15.93	6	8	339	35.6	9.48
Q6UXH1	Cysteine-rich with EGF-like domain protein 2 OS=Homo sapiens GN=CRELD2 PE=1 SV=1 - [CREL2_HUMAN]	8.50	1	3	3	8	0.596	0.986	0.805	1.358	24.41	8.50	6	8	353	38.2	4.59
Q14444	Caprin-1 OS=Homo sapiens GN=CAPRIN1 PE=1 SV=2 - [CAPR1_HUMAN]	21.72	1	16	16	111	0.993	1.191	1.168	1.359	237.03	21.72	26	111	709	78.3	5.25
Q5BKZ1	DBIRD complex subunit ZNF326 OS=Homo sapiens GN=ZNF326 PE=1 SV=2 - [ZN326_HUMAN]	15.12	1	8	8	25	1.422	1.125	1.012	1.360	52.87	15.12	12	25	582	65.6	5.15
P55789	FAD-linked sulfhydryl oxidase ALR OS=Homo sapiens GN=GFER PE=1 SV=2 - [ALR_HUMAN]	12.68	1	2	2	3	0.683	1.025	0.871	1.360	12.21	12.68	2	3	205	23.4	7.62
Q86VF7	Nebulin-related-anchoring protein OS=Homo sapiens GN=NRAP PE=2 SV=2 - [NRAP_HUMAN]	1.45	1	2	2	2	1.051	0.937	1.342	1.360	2.77	1.45	2	2	1730	197.0	9.20
O00221	NF-kappa-B inhibitor epsilon OS=Homo sapiens GN=NFKBIE PE=1 SV=3 - [IKBE_HUMAN]	2.40	1	1	1	2	1.518	1.305	1.696	1.360	6.93	2.40	2	2	500	52.8	6.68
Q03989	AT-rich interactive domain-containing protein 5A OS=Homo sapiens GN=ARID5A PE=1 SV=2 - [ARI5A_HUMAN]	2.53	1	1	1	1	4.731	1.992	1.183	1.360	0.00	2.53	1	1	594	64.0	9.25
O94988	Protein FAM13A OS=Homo sapiens GN=FAM13A PE=1 SV=2 - [FA13A_HUMAN]	1.47	1	1	2	6	0.951	1.029	0.800	1.361	12.24	1.47	2	6	1023	116.9	6.48

Q96B97	SH3 domain-containing kinase-binding protein 1 OS=Homo sapiens GN=SH3KBP1 PE=1 SV=2 - [SH3K1_HUMAN]	19.25	1	12	12	37	1.049	0.435	0.594	1.362	82.28	19.25	20	37	665	73.1	6.62
Q9BRF8	Serine/threonine-protein phosphatase CPPED1 OS=Homo sapiens GN=CPPED1 PE=1 SV=3 - [CPPED_HUMAN]	35.99	1	9	9	43	2.146	0.667	1.125	1.362	100.69	35.99	17	43	314	35.5	6.20
O95400	CD2 antigen cytoplasmic tail-binding protein 2 OS=Homo sapiens GN=CD2BP2 PE=1 SV=1 - [CD2B2_HUMAN]	16.72	1	6	6	11	1.330	1.294	1.189	1.362	21.16	16.72	9	11	341	37.6	4.61
Q9Y5Q9	General transcription factor 3C polypeptide 3 OS=Homo sapiens GN=GTF3C3 PE=1 SV=1 - [TF3C3_HUMAN]	3.05	1	2	2	6	1.088	1.125	0.961	1.362	8.52	3.05	3	6	886	101.2	5.07
Q14966	Zinc finger protein 638 OS=Homo sapiens GN=ZNF638 PE=1 SV=2 - [ZN638_HUMAN]	3.39	1	6	6	16	1.148	1.364	0.960	1.363	25.08	3.39	9	16	1978	220.5	6.38
Q13283	Ras GTPase-activating protein-binding protein 1 OS=Homo sapiens GN=G3BP1 PE=1 SV=1 - [G3BP1_HUMAN]	40.77	1	14	16	117	0.943	0.876	0.915	1.363	296.82	40.77	26	117	466	52.1	5.52
Q5VWQ8	Disabled homolog 2-interacting protein OS=Homo sapiens GN=DAB2IP PE=1 SV=2 - [DAB2P_HUMAN]	8.24	2	8	11	16	2.657	0.918	1.052	1.363	27.35	8.24	14	16	1189	131.5	8.72
Q5MNZ9	WD repeat domain phosphoinositide-interacting protein 1 OS=Homo sapiens GN=WIPI1 PE=1 SV=3 - [WIPI1_HUMAN]	3.81	1	2	2	3	1.088	0.705	0.752	1.363	7.41	3.81	2	3	446	48.6	6.60

O60885	Bromodomain-containing protein 4 OS=Homo sapiens GN=BRD4 PE=1 SV=2 - [BRD4_HUMAN]	4.48	1	4	5	13	1.822	1.345	0.982	1.363	17.02	4.48	7	13	1362	152.1	9.19
P48735	Isocitrate dehydrogenase [NADP], mitochondrial OS=Homo sapiens GN=IDH2 PE=1 SV=2 - [IDHP_HUMAN]	45.58	1	19	19	107	0.888	1.477	0.952	1.363	234.23	45.58	32	107	452	50.9	8.69
P08183	Multidrug resistance protein 1 OS=Homo sapiens GN=ABCB1 PE=1 SV=3 - [MDR1_HUMAN]	1.48	1	2	2	2	0.851	0.967	1.696	1.364	2.21	1.48	2	2	1280	141.4	9.00
Q9NTJ4	Alpha-mannosidase 2C1 OS=Homo sapiens GN=MAN2C1 PE=1 SV=1 - [MA2C1_HUMAN]	3.27	1	5	5	8	1.268	1.217	1.204	1.364	7.08	3.27	7	8	1040	115.8	6.57
P19387	DNA-directed RNA polymerase II subunit RPB3 OS=Homo sapiens GN=POLR2C PE=1 SV=2 - [RPB3_HUMAN]	22.55	1	4	4	6	1.402	1.491	0.940	1.364	10.07	22.55	6	6	275	31.4	4.92
P49959	Double-strand break repair protein MRE11A OS=Homo sapiens GN=MRE11A PE=1 SV=3 - [MRE11_HUMAN]	19.49	1	14	14	37	0.895	1.320	0.878	1.365	61.54	19.49	22	37	708	80.5	5.90
Q02410	Amyloid beta A4 precursor protein-binding family A member 1 OS=Homo sapiens GN=APBA1 PE=1 SV=3 - [APBA1_HUMAN]	5.62	1	4	4	13	1.597	0.707	1.069	1.366	25.93	5.62	6	13	837	92.8	4.93
Q9NQT8	Kinesin-like protein KIF13B OS=Homo sapiens GN=KIF13B PE=1 SV=2 - [KI13B_HUMAN]	8.54	1	10	16	40	1.462	1.340	1.251	1.366	107.71	8.54	24	40	1826	202.7	5.88

O60216	Double-strand-break repair protein rad21 homolog OS=Homo sapiens GN=RAD21 PE=1 SV=2 - [RAD21_HUMAN]	9.67	1	4	4	13	1.257	1.032	0.788	1.366	36.03	9.67	7	13	631	71.6	4.65
Q96HJ3	Coiled-coil domain-containing protein 34 OS=Homo sapiens GN=CCDC34 PE=2 SV=2 - [CCD34_HUMAN]	2.41	1	1	1	2	1.886	0.744	0.679	1.366	0.00	2.41	1	2	373	43.2	7.40
Q9GZR7	ATP-dependent RNA helicase DDX24 OS=Homo sapiens GN=DDX24 PE=1 SV=1 - [DDX24_HUMAN	21.65	2	16	17	38	0.930	0.866	0.915	1.366	100.78	21.65	22	38	859	96.3	9.06
Q496A3	Spermatogenesis-associated serine-rich protein 1 OS=Homo sapiens GN=SPATS1 PE=2 SV=2 - [SPAS1_HUMAN]	2.33	1	1	1	2	0.616	0.549	1.180	1.366	4.79	2.33	1	2	300	33.7	7.46
Q7Z6K5	Arpin OS=Homo sapiens GN=ARPIN PE=1 SV=1 - [ARPIN_HUMAN]	23.01	1	3	4	6	1.398	1.327	1.083	1.366	14.59	23.01	5	6	226	24.9	5.83
Q8N131	Porimin OS=Homo sapiens GN=TMEM123 PE=1 SV=1 - [PORIM_HUMAN]	4.33	1	1	1	1	0.935	1.133	1.294	1.367	0.00	4.33	1	1	208	21.5	8.70
Q96JI7	Spatacsin OS=Homo sapiens GN=SPG11 PE=1 SV=3 - [SPTCS_HUMAN]	0.82	1	2	2	4	0.854	1.457	1.923	1.368	7.40	0.82	2	4	2443	278.7	5.97
O95232	Luc7-like protein 3 OS=Homo sapiens GN=LUC7L3 PE=1 SV=2 - [LC7L3_HUMAN]	14.81	1	5	6	11	0.928	0.804	0.657	1.368	28.12	14.81	10	11	432	51.4	9.79
O94766	Galactosylgalactosylxylosylprotein 3-beta-glucuronosyltransferase 3 OS=Homo sapiens GN=B3GAT3 PE=1 SV=2 - [B3GA3_HUMAN]	13.73	1	3	3	8	0.858	0.861	0.992	1.368	22.68	13.73	5	8	335	37.1	8.27

Q08945	FACT complex subunit SSRP1 OS=Homo sapiens GN=SSRP1 PE=1 SV=1 - [SSRP1_HUMAN]	17.21	1	14	14	37	1.025	1.361	0.891	1.368	79.72	17.21	20	37	709	81.0	6.87
Q9ULW3	Activator of basal transcription 1 OS=Homo sapiens GN=ABT1 PE=1 SV=1 - [ABT1_HUMAN]	2.57	1	1	1	2	0.852	1.086	1.284	1.369	2.04	2.57	2	2	272	31.1	9.88
P62875	DNA-directed RNA polymerases I, II, and III subunit RPABC5 OS=Homo sapiens GN=POLR2L PE=1 SV=1 - [RPAB5_HUMAN]	29.85	1	2	2	6	1.114	1.145	1.028	1.369	17.17	29.85	4	6	67	7.6	7.77
Q15154	Pericentriolar material 1 protein OS=Homo sapiens GN=PCM1 PE=1 SV=4 - [PCM1_HUMAN]	7.02	1	11	12	25	0.908	1.501	1.151	1.369	60.70	7.02	17	25	2024	228.4	5.02
Q13393	Phospholipase D1 OS=Homo sapiens GN=PLD1 PE=1 SV=1 - [PLD1_HUMAN]	6.33	1	8	8	12	1.670	1.061	0.864	1.370	27.66	6.33	11	12	1074	124.1	8.78
Q9NW64	Pre-mRNA-splicing factor RBM22 OS=Homo sapiens GN=RBM22 PE=1 SV=1 - [RBM22_HUMAN]	14.76	1	5	6	13	1.310	0.963	1.152	1.370	18.88	14.76	8	13	420	46.9	8.54
O95707	Ribonuclease P protein subunit p29 OS=Homo sapiens GN=POP4 PE=1 SV=2 - [RPP29_HUMAN]	4.55	1	1	1	3	0.915	0.831	0.752	1.370	2.46	4.55	2	3	220	25.4	10.07
P01213	Proenkephalin-B OS=Homo sapiens GN=PDYN PE=1 SV=1 - [PDYN_HUMAN]	3.54	1	1	1	1	1.019	1.299	1.257	1.370	1.97	3.54	1	1	254	28.4	6.30
Q96J01	THO complex subunit 3 OS=Homo sapiens GN=THOC3 PE=1 SV=1 - [THOC3_HUMAN]	2.56	1	1	1	1	0.958	1.378	0.911	1.371	2.54	2.56	1	1	351	38.7	6.09

Q9BRP1	Programmed cell death protein 2-like OS=Homo sapiens GN=PDCD2L PE=1 SV=1 - [PDD2L_HUMAN]	5.03	1	1	1	1		0.966		1.371	2.96	5.03	1	1	358	39.4	4.86
P36269	Gamma-glutamyltransferase 5 OS=Homo sapiens GN=GGT5 PE=1 SV=2 - [GGT5_HUMAN]	17.92	1	9	9	28	1.364	0.616	0.355	1.371	74.07	17.92	16	28	586	62.2	7.55
O43395	U4/U6 small nuclear ribonucleoprotein Prp3 OS=Homo sapiens GN=PRPF3 PE=1 SV=2 - [PRPF3_HUMAN]	20.20	1	12	12	21	1.082	1.241	1.068	1.371	34.63	20.20	18	21	683	77.5	9.50
Q8N111	Cell cycle exit and neuronal differentiation protein 1 OS=Homo sapiens GN=CEND1 PE=2 SV=1 - [CEND_HUMAN]	15.44	1	2	2	2	1.692	2.321	1.166	1.371	4.76	15.44	2	2	149	14.9	9.20
Q6JQN1	Acyl-CoA dehydrogenase family member 10 OS=Homo sapiens GN=ACAD10 PE=2 SV=1 - [ACD10_HUMAN]	5.67	1	6	6	10	0.954	1.689	1.301	1.371	17.58	5.67	8	10	1059	118.8	8.06
Q8N2G8	GH3 domain-containing protein OS=Homo sapiens GN=GHDC PE=1 SV=2 - [GHDC_HUMAN]	8.87	1	5	6	14	1.095	1.067	0.867	1.372	33.54	8.87	10	14	530	57.5	7.88
Q9BXS9	Solute carrier family 26 member 6 OS=Homo sapiens GN=SLC26A6 PE=1 SV=1 - [S26A6_HUMAN]	0.92	1	1	1	2	1.627	1.436	0.899	1.372	4.67	0.92	1	2	759	82.9	8.37
O43933	Peroxisome biogenesis factor 1 OS=Homo sapiens GN=PEX1 PE=1 SV=1 - [PEX1_HUMAN]	2.10	1	2	3	8	1.003	1.124	1.191	1.373	20.16	2.10	4	8	1283	142.8	6.33

Q9H6R4	Nucleolar protein 6 OS=Homo sapiens GN=NOL6 PE=1 SV=2 - [NOL6_HUMAN]	6.72	1	7	8	13	0.813	0.806	0.771	1.373	20.90	6.72	10	13	1146	127.5	7.64
Q96I59	Probable asparagine--tRNA ligase, mitochondrial OS=Homo sapiens GN=NARS2 PE=1 SV=3 - [SYNM_HUMAN]	3.98	1	2	2	3	0.848	1.228	1.185	1.374	5.33	3.98	2	3	477	54.1	7.24
Q12851	Mitogen-activated protein kinase kinase kinase kinase 2 OS=Homo sapiens GN=MAP4K2 PE=1 SV=2 - [M4K2_HUMAN]	2.20	1	1	2	2	1.264	0.756	0.782	1.374	4.63	2.20	2	2	820	91.5	6.34
Q5T9A4	ATPase family AAA domain-containing protein 3B OS=Homo sapiens GN=ATAD3B PE=1 SV=1 - [ATD3B_HUMAN]	27.47	1	1	17	78	1.440	1.430	0.786	1.374	200.81	27.47	26	78	648	72.5	9.20
Q9Y5J9	Mitochondrial import inner membrane translocase subunit Tim8 B OS=Homo sapiens GN=TIMM8B PE=1 SV=1 - [TIM8B_HUMAN]	40.96	1	4	4	7	0.720	0.788	0.845	1.374	12.61	40.96	5	7	83	9.3	5.12
Q9Y2H5	Pleckstrin homology domain-containing family A member 6 OS=Homo sapiens GN=PLEKHA6 PE=1 SV=4 - [PKHA6_HUMAN]	6.20	1	6	8	11	1.317	0.460	1.065	1.375	12.89	6.20	9	11	1048	117.1	9.10
P55769	NHP2-like protein 1 OS=Homo sapiens GN=NHP2L1 PE=1 SV=3 - [NH2L1_HUMAN]	39.84	1	3	3	10	0.931	0.966	0.972	1.375	27.03	39.84	5	10	128	14.2	8.46
A6NGC4	TLC domain-containing protein 2 OS=Homo sapiens GN=TLCD2 PE=2 SV=3 - [TLCD2_HUMAN]	3.79	1	1	1	4	0.408	0.868	0.584	1.376	11.86	3.79	2	4	264	28.7	10.10

Q9Y3A4	Ribosomal RNA-processing protein 7 homolog A OS=Homo sapiens GN=RRP7A PE=1 SV=2 - [RRP7A_HUMAN]	11.07	2	3	3	5	1.213	1.081	1.030	1.376	10.64	11.07	5	5	280	32.3	9.58
C9JR72	Kelch repeat and BTB domain-containing protein 13 OS=Homo sapiens GN=KBTBD13 PE=1 SV=1 - [KBTBD_HUMAN	4.80	1	1	1	3	1.268	1.776	1.658	1.376	0.00	4.80	1	3	458	49.5	5.71
Q96F85	CB1 cannabinoid receptor-interacting protein 1 OS=Homo sapiens GN=CNRIP1 PE=1 SV=1 - [CNRP1_HUMAN]	45.73	1	7	7	14	1.611	1.026	0.850	1.376	37.45	45.73	9	14	164	18.6	7.94
P84243	Histone H3.3 OS=Homo sapiens GN=H3F3A PE=1 SV=2 - [H33_HUMAN]	59.56	2	2	10	142		3.217	0.612	1.377	270.79	59.56	13	142	136	15.3	11.27
O14874	[3-methyl-2-oxobutanoate dehydrogenase [lipoamide]] kinase, mitochondrial OS=Homo sapiens GN=BCKDK PE=1 SV=2 - [BCKD_HUMAN]	13.83	1	4	4	14	1.434	1.591	1.636	1.377	22.61	13.83	8	14	412	46.3	8.82
Q13057	Bifunctional coenzyme A synthase OS=Homo sapiens GN=COASY PE=1 SV=4 - [COASY_HUMA N]	7.45	1	4	4	9	1.086	0.984	0.929	1.377	20.16	7.45	6	9	564	62.3	6.99
Q9GZS1	DNA-directed RNA polymerase I subunit RPA49 OS=Homo sapiens GN=POLR1E PE=1 SV=2 - [RPA49_HUMAN]	1.66	1	1	1	2	0.825	0.884	1.167	1.378	2.16	1.66	2	2	481	53.9	8.56
Q8WUA2	Peptidyl-prolyl cis-trans isomerase-like 4 OS=Homo sapiens GN=PPIL4 PE=1 SV=1 - [PPIL4_HUMAN]	5.28	1	2	2	6	0.987	1.491	1.184	1.378	19.12	5.28	4	6	492	57.2	5.92

Q9BYB4	Guanine nucleotide-binding protein subunit beta-like protein 1 OS=Homo sapiens GN=GNB1L PE=1 SV=2 - [GNB1L_HUMAN]	2.75	1	1	1	1	0.913	0.591	0.688	1.379	0.00	2.75	1	1	327	35.6	7.97
Q5SNV9	Uncharacterized protein C1orf167 OS=Homo sapiens GN=C1orf167 PE=2 SV=2 - [CA167_HUMAN]	0.75	1	1	1	1	1.201	0.316	0.440	1.379	1.64	0.75	1	1	1468	162.3	10.70
P51522	Zinc finger protein 83 OS=Homo sapiens GN=ZNF83 PE=2 SV=3 - [ZNF83_HUMAN]	15.89	94	1	2	3	1.838	2.833	1.742	1.379	2.61	15.89	2	3	516	59.7	9.22
Q8IX01	SURP and G-patch domain-containing protein 2 OS=Homo sapiens GN=SUGP2 PE=1 SV=2 - [SUGP2_HUMAN]	10.91	1	10	10	21	1.156	1.131	0.909	1.380	39.40	10.91	16	21	1082	120.1	7.28
Q9C004	Protein sprouty homolog 4 OS=Homo sapiens GN=SPRY4 PE=1 SV=2 - [SPY4_HUMAN]	7.36	1	2	2	4	1.202	0.691	1.691	1.380	8.61	7.36	3	4	299	32.5	7.88
Q96EL3	39S ribosomal protein L53, mitochondrial OS=Homo sapiens GN=MRPL53 PE=1 SV=1 - [RM53_HUMAN]	24.11	1	2	2	4	1.147	0.818	1.211	1.381	10.28	24.11	4	4	112	12.1	8.76
Q9Y2H6	Fibronectin type-III domain-containing protein 3A OS=Homo sapiens GN=FNDC3A PE=1 SV=4 - [FND3A_HUMAN	10.60	1	12	12	39	1.558	1.065	0.595	1.383	97.91	10.60	21	39	1198	131.8	6.71
Q92879	CUGBP Elav-like family member 1 OS=Homo sapiens GN=CELF1 PE=1 SV=2 - [CELF1_HUMAN]	8.44	1	3	4	11	1.639	1.217	0.952	1.383	18.96	8.44	6	11	486	52.0	8.46

P05166	Propionyl-CoA carboxylase beta chain, mitochondrial OS=Homo sapiens GN=PCCB PE=1 SV=3 - [PCCB_HUMAN]	17.44	1	7	7	24	1.106	1.476	1.347	1.384	75.44	17.44	14	24	539	58.2	7.64
Q9H974	Queuine tRNA-ribosyltransferase subunit QTRTD1 OS=Homo sapiens GN=QTRTD1 PE=1 SV=1 - [QTRD1_HUMAN]	6.27	1	2	2	5	1.056	1.374	1.103	1.384	12.26	6.27	4	5	415	46.7	6.81
P23219	Prostaglandin G/H synthase 1 OS=Homo sapiens GN=PTGS1 PE=1 SV=2 - [PGH1_HUMAN]	25.04	1	12	12	50	1.432	1.099	0.927	1.384	135.22	25.04	20	50	599	68.6	7.23
O75400	Pre-mRNA-processing factor 40 homolog A OS=Homo sapiens GN=PRPF40A PE=1 SV=2 - [PR40A_HUMAN]	20.06	2	21	21	53	1.018	1.206	0.888	1.385	132.84	20.06	28	53	957	108.7	7.56
O75694	Nuclear pore complex protein Nup155 OS=Homo sapiens GN=NUP155 PE=1 SV=1 - [NU155_HUMAN	12.15	1	12	12	43	1.045	0.993	0.894	1.386	105.43	12.15	18	43	1391	155.1	6.16
Q14137	Ribosome biogenesis protein BOP1 OS=Homo sapiens GN=BOP1 PE=1 SV=2 - [BOP1_HUMAN]	12.20	1	9	9	20	0.831	1.036	0.915	1.386	49.46	12.20	15	20	746	83.6	6.19
O75367	Core histone macro-H2A.1 OS=Homo sapiens GN=H2AFY PE=1 SV=4 - [H2AY_HUMAN]	48.12	1	17	18	76	1.134	1.404	1.091	1.386	196.26	48.12	27	76	372	39.6	9.79
O96028	Histone-lysine N-methyltransferase NSD2 OS=Homo sapiens GN=WHSC1 PE=1 SV=1 - [NSD2_HUMAN]	0.51	3	1	1	1	0.477	1.801	1.177	1.387	0.00	0.51	1	1	1365	152.2	8.69

Q92889	DNA repair endonuclease XPF OS=Homo sapiens GN=ERCC4 PE=1 SV=3 - [XPF_HUMAN]	1.31	1	1	1	9	1.157	1.147	1.091	1.387	18.89	1.31	2	9	916	104.4	6.93
Q14517	Protocadherin Fat 1 OS=Homo sapiens GN=FAT1 PE=1 SV=2 - [FAT1_HUMAN]	6.12	1	20	20	32	1.265	0.582	0.779	1.387	69.06	6.12	25	32	4588	506.0	5.00
Q13232	Nucleoside diphosphate kinase 3 OS=Homo sapiens GN=NME3 PE=1 SV=2 - [NDK3_HUMAN]	40.83	1	6	6	19	1.627	0.847	0.554	1.387	33.27	40.83	9	19	169	19.0	7.84
O43823	A-kinase anchor protein 8 OS=Homo sapiens GN=AKAP8 PE=1 SV=1 - [AKAP8_HUMAN]	6.79	1	5	5	9	1.439	1.471	1.197	1.387	19.13	6.79	7	9	692	76.1	5.15
Q9HCN4	GPN-loop GTPase 1 OS=Homo sapiens GN=GPN1 PE=1 SV=1 - [GPN1_HUMAN]	6.68	1	2	2	5	0.962	1.083	0.874	1.388	12.25	6.68	3	5	374	41.7	4.92
P32239	Gastrin/cholecystokinin type B receptor OS=Homo sapiens GN=CCKBR PE=1 SV=1 - [GASR_HUMAN]	5.59	1	1	1	1	3.767	1.510	3.514	1.388	2.46	5.59	1	1	447	48.4	9.96
Q96C23	Aldose 1-epimerase OS=Homo sapiens GN=GALM PE=1 SV=1 - [GALM_HUMAN]	26.32	1	7	7	22	1.543	0.853	0.647	1.388	47.15	26.32	9	22	342	37.7	6.65
Q9BVA0	Katanin p80 WD40 repeat-containing subunit B1 OS=Homo sapiens GN=KATNB1 PE=1 SV=1 - [KTNB1_HUMAN]	5.19	1	2	2	9	1.913	1.173	1.038	1.388	41.63	5.19	2	9	655	72.3	7.56
Q9UFE4	Coiled-coil domain-containing protein 39 OS=Homo sapiens GN=CCDC39 PE=2 SV=3 - [CCD39_HUMAN]	3.08	1	1	2	5		3.753	5.616	1.389	0.00	3.08	2	5	941	109.8	6.44

Q6PD62	RNA polymerase-associated protein CTR9 homolog OS=Homo sapiens GN=CTR9 PE=1 SV=1 - [CTR9_HUMAN]	5.03	1	3	6	11	1.095	0.857	0.960	1.389	33.80	5.03	7	11	1173	133.4	6.77
Q96HJ9	UPF0562 protein C7orf55 OS=Homo sapiens GN=C7orf55 PE=1 SV=2 - [CG055_HUMAN]	28.32	1	3	3	5	1.880	1.329	1.312	1.389	9.95	28.32	4	5	113	12.7	10.07
Q09161	Nuclear cap-binding protein subunit 1 OS=Homo sapiens GN=NCBP1 PE=1 SV=1 - [NCBP1_HUMAN]	6.58	1	5	6	27	1.261	1.111	0.837	1.389	63.24	6.58	8	27	790	91.8	6.43
Q9UPY3	Endoribonuclease Dicer OS=Homo sapiens GN=DICER1 PE=1 SV=3 - [DICER_HUMAN]	2.13	1	3	3	5	0.795	1.178	1.247	1.389	5.87	2.13	5	5	1922	218.5	5.68
Q13330	Metastasis-associated protein MTA1 OS=Homo sapiens GN=MTA1 PE=1 SV=2 - [MTA1_HUMAN]	16.50	1	7	10	21	0.973	1.418	1.090	1.390	39.62	16.50	14	21	715	80.7	9.26
Q13426	DNA repair protein XRCC4 OS=Homo sapiens GN=XRCC4 PE=1 SV=2 - [XRCC4_HUMAN]	5.36	1	2	2	2	1.181	1.896	1.659	1.390	2.25	5.36	2	2	336	38.3	4.98
A4D1F6	Leucine-rich repeat and death domain-containing protein 1 OS=Homo sapiens GN=LRRD1 PE=2 SV=2 - [LRRD1_HUMAN]	3.49	1	2	2	14	2.368	1.298	0.995	1.390	0.00	3.49	2	14	860	98.0	7.46
Q99643	Succinate dehydrogenase cytochrome b560 subunit, mitochondrial OS=Homo sapiens GN=SDHC PE=1 SV=1 - [C560_HUMAN]	9.47	1	1	1	1	2.758	1.975	1.452	1.392	2.64	9.47	1	1	169	18.6	9.69

Q8WVM7	Cohesin subunit SA-1 OS=Homo sapiens GN=STAG1 PE=1 SV=3 - [STAG1_HUMAN]	4.21	1	5	7	9	1.756	0.768	0.741	1.392	17.19	4.21	8	9	1258	144.3	5.59
O00712	Nudear factor 1 B-type OS=Homo sapiens GN=NFIB PE=1 SV=2 - [NFIB_HUMAN]	5.71	1	1	2	3	0.760	0.832	1.076	1.392	8.69	5.71	2	3	420	47.4	8.87
Q96NY7	Chloride intracellular channel protein 6 OS=Homo sapiens GN=CLIC6 PE=2 SV=3 - [CLIC6_HUMAN]	8.81	1	2	5	6	1.420	0.599	1.564	1.392	16.20	8.81	6	6	704	73.0	4.37
O14548	Cytochrome c oxidase subunit 7A-related protein, mitochondrial OS=Homo sapiens GN=COX7A2L PE=1 SV=2 - [COX7R_HUMAN]	33.33	1	4	4	6	1.648	0.980	0.799	1.393	16.78	33.33	5	6	114	12.6	9.42
Q9Y6M7	Sodium bicarbonate cotransporter 3 OS=Homo sapiens GN=SLC4A7 PE=1 SV=2 - [S4A7_HUMAN]	9.56	1	7	7	14	0.885	0.453	0.600	1.393	29.19	9.56	9	14	1214	136.0	6.71
Q9UQ90	Paraplegin OS=Homo sapiens GN=SPG7 PE=1 SV=2 - [SPG7_HUMAN]	6.04	1	3	3	8	1.065	1.096	0.972	1.393	23.29	6.04	4	8	795	88.2	8.69
Q9NUT2	ATP-binding cassette sub-family B member 8, mitochondrial OS=Homo sapiens GN=ABCB8 PE=1 SV=3 - [ABCB8_HUMAN]	4.63	1	3	3	5	1.560	1.629	1.415	1.393	5.16	4.63	4	5	735	79.9	8.98
Q8N9T8	Protein KRI1 homolog OS=Homo sapiens GN=KRI1 PE=1 SV=3 - [KRI1_HUMAN]	7.54	1	5	6	17	1.088	0.893	0.913	1.393	24.53	7.54	8	17	703	82.5	5.14
Q8WZ74	Cortactin-binding protein 2 OS=Homo sapiens GN=CTTNBP2 PE=1 SV=1 - [CTTB2_HUMAN]	1.92	2	1	4	5	1.156	2.390	1.611	1.393	6.69	1.92	4	5	1663	180.9	7.93

O43676	NADH dehydrogenase [ubiquinone] 1 beta subcomplex subunit 3 OS=Homo sapiens GN=NDUFB3 PE=1 SV=3 - [NDUB3_HUMAN]	31.63	1	4	4	10	0.685	1.218	1.031	1.393	22.73	31.63	6	10	98	11.4	9.20
P13500	C-C motif chemokine 2 OS=Homo sapiens GN=CCL2 PE=1 SV=1 - [CCL2_HUMAN]	17.17	3	2	2	2	0.903	0.299	0.544	1.393	2.87	17.17	2	2	99	11.0	9.25
Q10570	Cleavage and polyadenylation specificity factor subunit 1 OS=Homo sapiens GN=CPSF1 PE=1 SV=2 - [CPSF1_HUMAN]	9.22	1	11	11	27	1.118	1.372	1.127	1.393	68.00	9.22	17	27	1443	160.8	6.40
Q14722	Voltage-gated potassium channel subunit beta-1 OS=Homo sapiens GN=KCNAB1 PE=2 SV=1 - [KCAB1_HUMAN]	3.10	1	1	2	2	3.186	2.062	1.064	1.394	2.09	3.10	2	2	419	46.5	8.94
P39060	Collagen alpha-1(XVIII) chain OS=Homo sapiens GN=COL18A1 PE=1 SV=5 - [COIA1_HUMAN]	12.71	1	17	17	42	1.465	0.817	0.725	1.394	94.11	12.71	25	42	1754	178.1	6.01
Q5H8A4	GPI ethanolamine phosphate transferase 2 OS=Homo sapiens GN=PIGG PE=1 SV=1 - [PIGG_HUMAN]	3.66	1	2	3	4	1.084	0.970	1.052	1.394	6.96	3.66	4	4	983	108.1	7.15
Q9BZL6	Serine/threonine-protein kinase D2 OS=Homo sapiens GN=PRKD2 PE=1 SV=2 - [KPCD2_HUMAN	6.95	2	3	5	14	1.039	0.747	1.372	1.395	35.12	6.95	8	14	878	96.7	6.84
Q16719	Kynureninase OS=Homo sapiens GN=KYNU PE=1 SV=1 - [KYNU_HUMAN]	18.49	1	7	8	14	7.489	0.557	0.482	1.395	27.26	18.49	11	14	465	52.3	7.03

Q8NDD1	Uncharacterized protein C1orf131 OS=Homo sapiens GN=C1orf131 PE=1 SV=3 - [CA131_HUMAN]	5.78	8	1	2	6	0.963	0.913	0.911	1.395	15.76	5.78	2	6	294	32.7	9.92
Q9NTJ3	Structural maintenance of chromosomes protein 4 OS=Homo sapiens GN=SMC4 PE=1 SV=2 - [SMC4_HUMAN]	6.60	1	8	8	20	1.335	1.298	0.745	1.395	42.54	6.60	13	20	1288	147.1	6.79
P17676	CCAAT/enhancer-binding protein beta OS=Homo sapiens GN=CEBPB PE=1 SV=2 - [CEBPB_HUMAN]	11.30	1	4	4	14	1.156	1.317	1.096	1.395	24.23	11.30	5	14	345	36.1	8.31
Q99549	M-phase phosphoprotein 8 OS=Homo sapiens GN=MPHOSPH8 PE=1 SV=2 - [MPP8_HUMAN]	6.51	1	5	5	7	1.072	1.156	1.065	1.396	12.40	6.51	5	7	860	97.1	6.06
P09601	Heme oxygenase 1 OS=Homo sapiens GN=HMOX1 PE=1 SV=1 - [HMOX1_HUMAN]	32.29	1	10	10	28	0.480	1.389	1.122	1.396	74.37	32.29	13	28	288	32.8	8.25
Q92796	Disks large homolog 3 OS=Homo sapiens GN=DLG3 PE=1 SV=2 - [DLG3_HUMAN]	4.16	1	2	4	10	1.465	1.555	1.181	1.396	16.25	4.16	4	10	817	90.3	7.03
Q8N4C6	Ninein OS=Homo sapiens GN=NIN PE=1 SV=4 - [NIN_HUMAN]	1.82	1	2	5	32	1.574	0.819	1.463	1.397	61.60	1.82	6	32	2090	243.1	5.03
Q9P1T7	MyoD family inhibitor domain-containing protein OS=Homo sapiens GN=MDFIC PE=1 SV=2 - [MDFIC_HUMAN]	6.50	1	1	1	4	1.763	0.814	0.617	1.398	12.12	6.50	2	4	246	25.8	5.27
P61244	Protein max OS=Homo sapiens GN=MAX PE=1 SV=1 - [MAX_HUMAN]	6.25	1	1	1	2	0.916	1.150	0.959	1.398	2.42	6.25	2	2	160	18.3	6.30

Q14684	Ribosomal RNA processing protein 1 homolog B OS=Homo sapiens GN=RRP1B PE=1 SV=3 - [RRP1B_HUMAN]	15.70	1	10	10	17	0.922	1.112	0.885	1.399	25.96	15.70	13	17	758	84.4	9.76
Q9BX40	Protein LSM14 homolog B OS=Homo sapiens GN=LSM14B PE=1 SV=1 - [LS14B_HUMAN]	7.53	1	2	3	9	1.238	1.877	1.121	1.399	21.19	7.53	4	9	385	42.0	9.69
Q7Z7A4	PX domain-containing protein kinase-like protein OS=Homo sapiens GN=PXK PE=1 SV=1 - [PXK_HUMAN]	9.69	1	3	4	5	0.924	0.672	2.851	1.399	4.97	9.69	4	5	578	64.9	9.35
Q9Y3T9	Nucleolar complex protein 2 homolog OS=Homo sapiens GN=NOC2L PE=1 SV=4 - [NOC2L_HUMAN	4.01	1	4	4	7	0.689	0.862	0.800	1.400	15.01	4.01	6	7	749	84.9	5.62
Q06265	Exosome complex component RRP45 OS=Homo sapiens GN=EXOSC9 PE=1 SV=3 - [EXOS9_HUMAN]	4.78	1	2	2	6	1.080	0.931	0.882	1.400	15.84	4.78	4	6	439	48.9	5.29
P50453	Serpin B9 OS=Homo sapiens GN=SERPINB9 PE=1 SV=1 - [SPB9_HUMAN]	53.72	1	17	17	52	1.631	0.620	0.655	1.401	128.15	53.72	27	52	376	42.4	5.86
Q6UWH4	Protein FAM198B OS=Homo sapiens GN=FAM198B PE=2 SV=1 - [F198B_HUMAN]	8.09	1	4	4	7	0.487	1.034	0.607	1.401	21.43	8.09	7	7	519	57.5	9.70
Q9NXJ5	Pyroglutamyl-peptidase 1 OS=Homo sapiens GN=PGPEP1 PE=1 SV=1 - [PGPI_HUMAN]	7.66	1	1	1	3	0.886	1.038	1.408	1.401	8.24	7.66	2	3	209	23.1	5.90
Q15291	Retinoblastoma-binding protein 5 OS=Homo sapiens GN=RBBP5 PE=1 SV=2 - [RBBP5_HUMAN]	9.29	1	3	3	5	1.052	1.149	1.007	1.401	15.16	9.29	4	5	538	59.1	5.10

P84103	Serine/arginine-rich splicing factor 3 OS=Homo sapiens GN=SRSF3 PE=1 SV=1 - [SRSF3_HUMAN]	34.76	1	8	9	94	1.227	1.558	1.084	1.401	173.33	34.76	18	94	164	19.3	11.65
Q9BYG4	Partitioning defective 6 homolog gamma OS=Homo sapiens GN=PARD6G PE=1 SV=1 - [PAR6G_HUMAN]	4.52	2	1	2	6	3.117	1.049	1.624	1.401	13.02	4.52	4	6	376	40.9	8.22
Q9BST9	Rhotekin OS=Homo sapiens GN=RTKN PE=1 SV=2 - [RTKN_HUMAN]	4.09	1	2	2	2	1.299	1.025	1.191	1.401	5.76	4.09	2	2	563	62.6	7.44
Q7RTV0	PHD finger-like domain-containing protein 5A OS=Homo sapiens GN=PHF5A PE=1 SV=1 - [PHF5A_HUMAN]	21.82	1	3	3	9	1.122	1.727	1.072	1.401	10.34	21.82	6	9	110	12.4	8.41
Q15057	Arf-GAP with coiled-coil, ANK repeat and PH domain-containing protein 2 OS=Homo sapiens GN=ACAP2 PE=1 SV=3 - [ACAP2_HUMAN]	11.83	1	10	10	30	1.280	1.081	0.950	1.401	80.27	11.83	14	30	778	88.0	6.80
Q8IZA0	Dyslexia-associated protein KIAA0319-like protein OS=Homo sapiens GN=KIAA0319L PE=1 SV=2 - [K319L_HUMAN]	3.91	1	3	3	10	1.390	1.092	1.127	1.402	16.54	3.91	6	10	1049	115.6	6.05
Q9C0A1	Zinc finger homeobox protein 2 OS=Homo sapiens GN=ZFHX2 PE=2 SV=3 - [ZFHX2_HUMAN]	0.78	1	1	1	2	0.722	3.910	3.071	1.402	0.00	0.78	1	2	2572	274.0	5.88
Q8IXK0	Polyhomeotic-like protein 2 OS=Homo sapiens GN=PHC2 PE=1 SV=1 - [PHC2_HUMAN]	3.03	1	3	3	6	1.006	1.308	0.919	1.402	11.68	3.03	4	6	858	90.7	8.69

Q9H8H2	Probable ATP-dependent RNA helicase DDX31 OS=Homo sapiens GN=DDX31 PE=1 SV=2 - [DDX31_HUMAN	7.17	1	6	6	12	0.760	1.011	0.922	1.403	18.66	7.17	10	12	851	94.0	9.99
P58872	Rhomboid-related protein 3 OS=Homo sapiens GN=RHBDL3 PE=2 SV=1 - [RHBL3_HUMAN]	1.98	1	1	1	1	0.668	1.118	1.317	1.403	0.00	1.98	1	1	404	45.2	7.61
Q9BUI4	DNA-directed RNA polymerase III subunit RPC3 OS=Homo sapiens GN=POLR3C PE=1 SV=1 - [RPC3_HUMAN]	7.87	1	3	3	5	0.948	1.523	1.349	1.403	10.83	7.87	4	5	534	60.6	7.31
Q86YP4	Transcriptional repressor p66-alpha OS=Homo sapiens GN=GATAD2A PE=1 SV=1 - [P66A_HUMAN]	21.01	1	9	10	35	1.008	1.090	0.975	1.403	65.61	21.01	17	35	633	68.0	9.94
Q8IYW2	Protein CFAP46 OS=Homo sapiens GN=CFAP46 PE=2 SV=3 - [CFA46_HUMAN]	0.66	1	1	1	1	1.172	2.357	1.836	1.403	0.00	0.66	1	1	2715	303.3	7.36
Q9Y5J1	U3 small nucleolar RNA-associated protein 18 homolog OS=Homo sapiens GN=UTP18 PE=1 SV=3 - [UTP18_HUMAN]	7.37	1	3	3	13	0.726	0.836	0.870	1.403	24.64	7.37	5	13	556	62.0	8.76
Q9ULH1	Arf-GAP with SH3 domain, ANK repeat and PH domain-containing protein 1 OS=Homo sapiens GN=ASAP1 PE=1 SV=4 - [ASAP1_HUMAN]	25.16	1	21	24	88	1.824	0.618	0.961	1.404	242.30	25.16	36	88	1129	125.4	7.31
Q9H0K1	Serine/threonine-protein kinase SIK2 OS=Homo sapiens GN=SIK2 PE=1 SV=1 - [SIK2_HUMAN]	3.02	1	3	3	5	0.864	1.727	1.297	1.404	14.81	3.02	4	5	926	103.8	6.02

Q96CM3	RNA pseudouridylate synthase domain-containing protein 4 OS=Homo sapiens GN=RPUSD4 PE=2 SV=1 - [RUSD4_HUMAN]	2.92	1	1	1	1	0.759	0.981	1.447	1.404	2.09	2.92	1	1	377	42.2	9.88
Q8IZD4	mRNA-decapping enzyme 1B OS=Homo sapiens GN=DCP1B PE=1 SV=2 - [DCP1B_HUMAN]	6.65	1	3	3	10	1.167	1.369	0.959	1.404	24.30	6.65	5	10	617	67.7	8.56
Q15165	Serum paraoxonase/arylesterase 2 OS=Homo sapiens GN=PON2 PE=1 SV=3 - [PON2_HUMAN]	22.60	1	5	5	9	0.803	0.853	0.759	1.406	21.54	22.60	8	9	354	39.4	5.60
P48051	G protein-activated inward rectifier potassium channel 2 OS=Homo sapiens GN=KCNJ6 PE=1 SV=1 - [KCNJ6_HUMAN]	1.89	1	1	1	2	5.773	0.923	1.257	1.406	1.81	1.89	2	2	423	48.4	5.38
Q9NZJ6	Hexaprenyldihydroxybenzoate methyltransferase, mitochondrial OS=Homo sapiens GN=COQ3 PE=1 SV=3 - [COQ3_HUMAN]	6.50	1	2	2	7	1.335	1.439	2.453	1.406	3.19	6.50	2	7	369	41.0	7.42
Q9Y613	FH1/FH2 domain-containing protein 1 OS=Homo sapiens GN=FHOD1 PE=1 SV=3 - [FHOD1_HUMAN]	6.10	1	7	7	12	1.035	0.892	0.783	1.407	37.59	6.10	10	12	1164	126.5	6.39
Q9P0U4	CXXC-type zinc finger protein 1 OS=Homo sapiens GN=CXXC1 PE=1 SV=2 - [CXXC1_HUMAN]	3.51	1	2	2	12	0.923	1.411	0.989	1.407	32.28	3.51	4	12	656	75.7	8.24
Q9NQG5	Regulation of nuclear pre-mRNA domain-containing protein 1B OS=Homo sapiens GN=RPRD1B PE=1 SV=1 - [RPR1B_HUMAN]	31.90	3	8	10	33	1.189	1.346	0.850	1.407	85.63	31.90	15	33	326	36.9	5.97

Q99496	E3 ubiquitin-protein ligase RING2 OS=Homo sapiens GN=RNF2 PE=1 SV=1 - [RING2_HUMAN]	6.25	1	2	2	5	0.907	1.128	0.890	1.407	11.35	6.25	4	5	336	37.6	6.84
Q9H5X1	MIP18 family protein FAM96A OS=Homo sapiens GN=FAM96A PE=1 SV=1 - [FA96A_HUMAN]	12.50	1	2	2	10	1.165	0.918	0.878	1.408	21.42	12.50	4	10	160	18.3	4.88
Q8NFU7	Methylcytosine dioxygenase TET1 OS=Homo sapiens GN=TET1 PE=1 SV=2 - [TET1_HUMAN]	1.54	1	2	2	4	0.934	1.050	0.955	1.408	2.74	1.54	2	4	2136	235.2	8.24
Q8IX05	CD302 antigen OS=Homo sapiens GN=CD302 PE=1 SV=1 - [CD302_HUMAN]	11.64	1	2	2	3	1.078	0.589	0.410	1.409	6.58	11.64	2	3	232	26.2	4.61
Q6Y288	Beta-1,3-glucosyltransferase OS=Homo sapiens GN=B3GALTL PE=1 SV=2 - [B3GLT_HUMAN]	3.01	1	1	2	3	1.229	1.690	1.876	1.410	4.67	3.01	3	3	498	56.5	7.50
Q5VZL5	Zinc finger MYM-type protein 4 OS=Homo sapiens GN=ZMYM4 PE=1 SV=1 - [ZMYM4_HUMAN]	5.36	1	4	4	5	1.598	1.471	1.237	1.410	5.44	5.36	4	5	1548	172.7	6.84
Q8N5G2	Macoilin OS=Homo sapiens GN=TMEM57 PE=1 SV=1 - [MACOI_HUMAN]	5.57	1	3	4	17	1.077	1.030	1.437	1.410	36.23	5.57	7	17	664	76.1	9.07
O95425	Supervillin OS=Homo sapiens GN=SVIL PE=1 SV=2 - [SVIL_HUMAN]	26.33	1	48	48	160	1.491	2.158	1.444	1.410	430.91	26.33	74	160	2214	247.6	6.98
O95139	NADH dehydrogenase [ubiquinone] 1 beta subcomplex subunit 6 OS=Homo sapiens GN=NDUFB6 PE=1 SV=3 - [NDUB6_HUMAN]	39.84	1	6	6	17	1.253	1.167	0.963	1.411	30.67	39.84	8	17	128	15.5	9.63

Q8IZQ5	Selenoprotein H OS=Homo sapiens GN=SELH PE=1 SV=2 - [SELH_HUMAN]	15.57	1	2	2	5	1.717	0.990	0.686	1.411	12.26	15.57	3	5	122	13.4	9.74
Q9H0C8	Integrin-linked kinase-associated serine/threonine phosphatase 2C OS=Homo sapiens GN=ILKAP PE=1 SV=1 - [ILKAP_HUMAN]	16.33	1	6	6	14	1.277	1.083	0.702	1.411	35.70	16.33	8	14	392	42.9	7.09
P23527	Histone H2B type 1-O OS=Homo sapiens GN=HIST1H2BO PE=1 SV=3 - [H2B1O_HUMAN]	67.46	7	2	13	353	1.661	1.725	1.031	1.412	745.67	67.46	23	353	126	13.9	10.32
Q8N9N7	Leucine-rich repeat-containing protein 57 OS=Homo sapiens GN=LRRC57 PE=1 SV=1 - [LRC57_HUMAN]	17.99	1	5	5	9	1.041	1.137	1.127	1.412	19.18	17.99	7	9	239	26.7	8.43
Q9BYX2	TBC1 domain family member 2A OS=Homo sapiens GN=TBC1D2 PE=1 SV=3 - [TBD2A_HUMAN]	8.84	2	6	8	31	1.748	0.706	1.064	1.412	74.75	8.84	11	31	928	105.3	6.58
Q16875	6-phosphofructo-2-kinase/fructose-2,6-bisphosphatase 3 OS=Homo sapiens GN=PFKFB3 PE=1 SV=1 - [F263_HUMAN]	1.92	1	1	1	1	1.182	1.056	1.539	1.413	2.13	1.92	1	1	520	59.6	8.21
Q9H5Q4	Dimethyladenosine transferase 2, mitochondrial OS=Homo sapiens GN=TFB2M PE=1 SV=1 - [TFB2M_HUMA	5.81	1	2	2	6	0.686	0.938	1.009	1.413	15.93	5.81	2	6	396	45.3	9.19
Q5TCY1	Tau-tubulin kinase 1 OS=Homo sapiens GN=TTBK1 PE=1 SV=2 - [TTBK1_HUMAN]	0.98	1	1	1	2	0.658	0.724	1.331	1.413	0.00	0.98	1	2	1321	142.6	5.60

Q9H6K4	Optic atrophy 3 protein OS=Homo sapiens GN=OPA3 PE=1 SV=1 - [OPA3_HUMAN]	9.50	1	2	2	4	1.515	0.888	1.015	1.413	2.14	9.50	3	4	179	20.0	8.91
Q52LJ0	Protein FAM98B OS=Homo sapiens GN=FAM98B PE=1 SV=1 - [FA98B_HUMAN]	31.21	1	7	10	33	1.161	1.346	1.258	1.414	64.40	31.21	14	33	330	37.2	6.29
Q9BXP5	Serrate RNA effector molecule homolog OS=Homo sapiens GN=SRRT PE=1 SV=1 - [SRRT_HUMAN]	20.32	2	20	20	77	1.199	1.220	1.072	1.414	167.42	20.32	27	77	876	100.6	5.96
O00488	Zinc finger protein 593 OS=Homo sapiens GN=ZNF593 PE=1 SV=2 - [ZN593_HUMAN]	10.45	1	1	1	4	0.294	0.840	0.585	1.414	18.31	10.45	1	4	134	15.2	9.82
Q8N163	Cell cycle and apoptosis regulator protein 2 OS=Homo sapiens GN=CCAR2 PE=1 SV=2 - [CCAR2_HUMAN]	13.98	1	11	13	25	1.576	1.208	0.973	1.415	52.93	13.98	20	25	923	102.8	5.22
Q05048	Cleavage stimulation factor subunit 1 OS=Homo sapiens GN=CSTF1 PE=1 SV=1 - [CSTF1_HUMAN]	11.37	1	3	3	12	0.721	1.317	1.366	1.415	40.77	11.37	6	12	431	48.3	6.58
O95478	Ribosome biogenesis protein NSA2 homolog OS=Homo sapiens GN=NSA2 PE=1 SV=1 - [NSA2_HUMAN]	10.77	1	3	3	6	0.616	0.730	0.666	1.416	11.33	10.77	3	6	260	30.0	10.27
Q15020	Squamous cell carcinoma antigen recognized by T-cells 3 OS=Homo sapiens GN=SART3 PE=1 SV=1 - [SART3_HUMAN]	16.10	1	13	14	42	0.975	1.137	0.858	1.416	90.80	16.10	21	42	963	109.9	5.57

Q9Y383	Putative RNA-binding protein Luc7-like 2 OS=Homo sapiens GN=LUC7L2 PE=1 SV=2 - [LC7L2_HUMAN]	26.79	1	6	10	67	1.052	1.300	1.102	1.418	185.29	26.79	18	67	392	46.5	10.01
Q6UW63	KDEL motif-containing protein 1 OS=Homo sapiens GN=KDELC1 PE=1 SV=1 - [KDEL1_HUMAN]	7.77	1	3	4	7	1.934	1.067	1.458	1.419	10.99	7.77	5	7	502	58.0	7.71
Q6PL18	ATPase family AAA domain-containing protein 2 OS=Homo sapiens GN=ATAD2 PE=1 SV=1 - [ATAD2_HUMAN]	2.23	1	2	2	4	1.078	1.351	0.938	1.419	8.00	2.23	2	4	1390	158.5	6.32
Q13228	Selenium-binding protein 1 OS=Homo sapiens GN=SELENBP1 PE=1 SV=2 - [SBP1_HUMAN]	43.22	1	18	18	64	1.823	1.254	0.923	1.419	173.28	43.22	29	64	472	52.4	6.37
Q8TAV0	Protein FAM76A OS=Homo sapiens GN=FAM76A PE=2 SV=1 - [FA76A_HUMAN]	2.28	1	1	1	1	1.698	1.364	1.179	1.420	2.72	2.28	1	1	307	35.0	9.19
Q9BUP3	Oxidoreductase HTATIP2 OS=Homo sapiens GN=HTATIP2 PE=1 SV=2 - [HTAI2_HUMAN]	42.56	1	11	11	18	1.371	0.825	0.723	1.421	38.09	42.56	15	18	242	27.0	8.38
P35637	RNA-binding protein FUS OS=Homo sapiens GN=FUS PE=1 SV=1 - [FUS_HUMAN]	20.15	1	9	12	87	1.204	0.908	0.858	1.422	193.98	20.15	19	87	526	53.4	9.36
Q13263	Transcription intermediary factor 1-beta OS=Homo sapiens GN=TRIM28 PE=1 SV=5 - [TIF1B_HUMAN]	23.35	1	21	21	182	1.168	1.106	0.870	1.422	455.13	23.35	38	182	835	88.5	5.77
Q6IC98	GRAM domain-containing protein 4 OS=Homo sapiens GN=GRAMD4 PE=1 SV=1 - [GRAM4_HUMAN]	1.73	1	1	1	1	2.271	1.141	0.279	1.422	2.39	1.73	1	1	578	66.4	8.84

Q8ND76	Cyclin-Y OS=Homo sapiens GN=CCNY PE=1 SV=2 - [CCNY_HUMAN]	4.99	1	1	2	4	3.512	2.306	1.373	1.422	9.93	4.99	3	4	341	39.3	7.20
Q9Y2I1	Nischarin OS=Homo sapiens GN=NISCH PE=1 SV=3 - [NISCH_HUMAN]	1.20	1	2	2	5	1.280	1.153	0.983	1.423	10.99	1.20	2	5	1504	166.5	5.14
Q9NQ55	Suppressor of SWI4 1 homolog OS=Homo sapiens GN=PPAN PE=1 SV=1 - [SSF1_HUMAN]	3.38	1	2	2	5	0.988	0.931	0.659	1.424	11.93	3.38	3	5	473	53.2	10.13
Q96GC9	Vacuole membrane protein 1 OS=Homo sapiens GN=VMP1 PE=1 SV=1 - [VMP1_HUMAN]	8.87	1	1	2	4			0.525	1.424	11.29	8.87	2	4	406	46.2	6.95
O14578	Citron Rho-interacting kinase OS=Homo sapiens GN=CIT PE=1 SV=2 - [CTRO_HUMAN]	4.14	1	5	8	24	0.933	1.489	1.522	1.424	51.98	4.14	9	24	2027	231.3	6.57
O14939	Phospholipase D2 OS=Homo sapiens GN=PLD2 PE=1 SV=2 - [PLD2_HUMAN]	5.04	1	4	4	7	1.581	1.208	1.280	1.425	19.19	5.04	5	7	933	105.9	7.64
Q86U38	Nucleolar protein 9 OS=Homo sapiens GN=NOP9 PE=1 SV=1 - [NOP9_HUMAN]	5.35	1	2	3	9	0.791	0.519	0.695	1.425	26.00	5.35	4	9	636	69.4	7.28
P07910	Heterogeneous nuclear ribonucleoproteins C1/C2 OS=Homo sapiens GN=HNRNPC PE=1 SV=4 - [HNRPC_HUMAN]	36.27	7	11	17	159	1.275	1.441	1.009	1.425	376.80	36.27	27	159	306	33.6	5.08
A6NCK2	Tripartite motif-containing protein 43B OS=Homo sapiens GN=TRIM43B PE=3 SV=3 - [TR43B_HUMAN]	3.36	1	1	1	1	1.247	2.081	1.446	1.426	2.96	3.36	1	1	446	52.3	7.85

Q9BRP8	Partner of Y14 and mago OS=Homo sapiens GN=WIBG PE=1 SV=1 - [WIBG_HUMAN]	62.25	1	9	9	21	1.257	1.109	1.157	1.426	57.92	62.25	11	21	204	22.6	9.45
Q14914	Prostaglandin reductase 1 OS=Homo sapiens GN=PTGR1 PE=1 SV=2 - [PTGR1_HUMAN]	44.38	1	17	17	92	2.439	0.536	0.686	1.426	233.60	44.38	28	92	329	35.8	8.29
Q9Y291	28S ribosomal protein S33, mitochondrial OS=Homo sapiens GN=MRPS33 PE=1 SV=1 - [RT33_HUMAN]	11.32	1	1	1	2	0.767	1.612	1.284	1.427	2.80	11.32	2	2	106	12.6	10.11
P09234	U1 small nuclear ribonucleoprot ein C OS=Homo sapiens GN=SNRPC PE=1 SV=1 -	5.66	1	1	1	4	1.107	1.251	0.988	1.427	12.83	5.66	2	4	159	17.4	9.67
Q9UBR2	Cathepsin Z OS=Homo sapiens GN=CTSZ PE=1 SV=1 - [CATZ_HUMAN]	20.13	1	6	6	30	1.278	1.206	1.774	1.427	58.23	20.13	10	30	303	33.8	7.11
Q13077	TNF receptor-associated factor 1 OS=Homo sapiens GN=TRAF1 PE=1 SV=1 - [TRAF1_HUMAN	2.64	1	1	1	2	1.089	0.634	1.293	1.428	1.64	2.64	2	2	416	46.1	6.11
Q13835	Plakophilin-1 OS=Homo sapiens GN=PKP1 PE=1 SV=2 - [PKP1_HUMAN]	1.74	1	1	1	1	1.384	0.953	0.980	1.428	0.00	1.74	1	1	747	82.8	9.13
Q9H814	Phosphorylated adapter RNA export protein OS=Homo sapiens GN=PHAX PE=1 SV=1 - [PHAX_HUMAN]	7.87	1	3	3	6	0.999	1.242	1.130	1.430	19.06	7.87	5	6	394	44.4	5.40
Q9NQ29	Putative RNA-binding protein Luc7-like 1 OS=Homo sapiens GN=LUC7L PE=1 SV=1 - [LUC7L_HUMAN]	18.33	1	2	6	20	1.018	1.410	0.804	1.430	58.51	18.33	9	20	371	43.7	9.92

Q9BW30	Tubulin polymerization-promoting protein family member 3 OS=Homo sapiens GN=TPPP3 PE=1 SV=1 - [TPPP3_HUMAN]	12.50	1	2	2	4	1.641	9.479	1.566	1.430	8.48	12.50	3	4	176	19.0	9.13
Q12946	Forkhead box protein F1 OS=Homo sapiens GN=FOXF1 PE=1 SV=2 - [FOXF1_HUMAN]	15.57	2	3	3	11	1.038	1.077	1.349	1.431	29.90	15.57	4	11	379	40.1	9.10
O95905	Protein SGT1 OS=Homo sapiens GN=ECD PE=1 SV=1 - [SGT1_HUMAN]	1.24	1	1	1	2	1.187	0.974	1.172	1.431	1.64	1.24	2	2	644	72.7	4.87
O94952	F-box only protein 21 OS=Homo sapiens GN=FBXO21 PE=2 SV=2 - [FBX21_HUMAN]	1.27	1	1	1	1	1.230	2.643	1.947	1.431	0.00	1.27	1	1	628	72.2	6.09
P21399	Cytoplasmic aconitate hydratase OS=Homo sapiens GN=ACO1 PE=1 SV=3 - [ACOC_HUMAN]	32.85	1	27	27	170	2.013	1.342	1.049	1.431	429.99	32.85	43	170	889	98.3	6.68
O15381	Nuclear valosin-containing protein-like OS=Homo sapiens GN=NVL PE=1 SV=1 - [NVL_HUMAN]	1.40	1	1	1	2	0.939	0.963	0.646	1.432	3.33	1.40	2	2	856	95.0	6.48
Q5VV42	Threonylcarbamoyladenosine tRNA methylthiotransferase OS=Homo sapiens GN=CDKAL1 PE=1 SV=1 - [CDKAL_HUMAN]	2.07	1	1	1	4	0.902	0.922	0.874	1.432	9.46	2.07	2	4	579	65.1	7.46
P28065	Proteasome subunit beta type-9 OS=Homo sapiens GN=PSMB9 PE=1 SV=2 - [PSB9_HUMAN]	24.66	1	4	4	12	1.720	0.776	0.624	1.432	33.28	24.66	6	12	219	23.2	5.03
Q96AE7	Tetratricopeptide repeat protein 17 OS=Homo sapiens GN=TTC17 PE=1 SV=1 - [TTC17_HUMAN	4.65	1	5	6	17	1.192	0.976	0.856	1.432	40.92	4.65	8	17	1141	129.5	6.58

Q8NA47	Coiled-coil domain-containing protein 63 OS=Homo sapiens GN=CCDC63 PE=2 SV=1 - [CCD63_HUMAN]	1.78	1	1	1	2	1.950	0.799	1.501	1.432	0.00	1.78	1	2	563	66.2	9.06
Q96P56	Cation channel sperm-associated protein 2 OS=Homo sapiens GN=CATSPER2 PE=1 SV=2 - [CTSR2_HUMAN]	2.26	1	1	1	2	1.669	0.990	1.561	1.432	4.22	2.26	1	2	530	62.0	7.30
Q9NVP1	ATP-dependent RNA helicase DDX18 OS=Homo sapiens GN=DDX18 PE=1 SV=2 - [DDX18_HUMAN	16.12	1	9	9	24	0.878	0.938	0.865	1.432	62.60	16.12	12	24	670	75.4	9.50
Q96FJ2	Dynein light chain 2, cytoplasmic OS=Homo sapiens GN=DYNLL2 PE=1 SV=1 - [DYL2_HUMAN]	20.22	1	2	3	16	0.972	1.649	1.450	1.433	33.93	20.22	4	16	89	10.3	7.37
Q13625	Apoptosis-stimulating of p53 protein 2 OS=Homo sapiens GN=TP53BP2 PE=1 SV=2 - [ASPP2_HUMAN]	4.88	1	5	5	11	1.005	1.482	1.365	1.435	23.53	4.88	7	11	1128	125.5	6.07
Q9Y624	Junctional adhesion molecule A OS=Homo sapiens GN=F11R PE=1 SV=1 - [JAM1_HUMAN]	6.02	1	2	2	5	1.330	0.655	0.571	1.435	7.72	6.02	4	5	299	32.6	7.90
Q6NSJ2	Pleckstrin homology-like domain family B member 3 OS=Homo sapiens GN=PHLDB3 PE=2 SV=3 - [PHLB3_HUMAN]	6.41	1	4	4	6	0.885	0.884	0.871	1.436	11.76	6.41	4	6	640	71.9	6.57
Q9UDR5	Alpha-aminoadipic semialdehyde synthase, mitochondrial OS=Homo sapiens GN=AASS PE=1 SV=1 - [AASS_HUMAN]	12.85	1	12	12	34	1.177	1.127	0.677	1.437	55.10	12.85	18	34	926	102.1	6.64

Q9UK61	Protein FAM208A OS=Homo sapiens GN=FAM208A PE=1 SV=3 - [F208A_HUMAN]	4.13	1	7	7	9	0.888	1.590	0.938	1.437	11.64	4.13	9	9	1670	188.9	5.80
Q8NBL1	Protein O-glucosyltransferase 1 OS=Homo sapiens GN=POGLUT1 PE=1 SV=1 - [PGLT1_HUMAN]	26.02	1	12	12	41	1.149	0.869	0.926	1.437	71.48	26.02	19	41	392	46.2	8.72
O75151	Lysine-specific demethylase PHF2 OS=Homo sapiens GN=PHF2 PE=1 SV=4 - [PHF2_HUMAN]	5.02	1	5	5	9	1.065	1.503	1.087	1.437	12.14	5.02	7	9	1096	120.7	9.17
Q6P4R8	Nuclear factor related to kappa-B-binding protein OS=Homo sapiens GN=NFRKB PE=1 SV=2 - [NFRKB_HUMAN]	1.23	1	1	1	1	1.157	1.290	1.074	1.438	0.00	1.23	1	1	1299	138.9	9.25
Q8N283	Ankyrin repeat domain-containing protein 35 OS=Homo sapiens GN=ANKRD35 PE=2 SV=2 - [ANR35_HUMAN]	5.29	2	4	5	6	1.649	2.204	1.390	1.438	12.97	5.29	6	6	1001	109.9	6.02
Q96T60	Bifunctional polynucleotide phosphatase/kinase OS=Homo sapiens GN=PNKP PE=1 SV=1 - [PNKP_HUMAN]	8.06	1	3	4	15	1.157	1.409	1.160	1.438	33.37	8.06	5	15	521	57.0	8.46
Q8TCG2	Phosphatidylinositol 4-kinase type 2-beta OS=Homo sapiens GN=PI4K2B PE=1 SV=1 - [P4K2B_HUMAN]	3.12	1	1	2	3	1.158	0.840	1.193	1.439	0.00	3.12	2	3	481	54.7	5.97
Q9UHA3	Probable ribosome biogenesis protein RLP24 OS=Homo sapiens GN=RSL24D1 PE=1 SV=1 - [RLP24_HUMAN]	4.29	1	1	1	3	0.733	0.953	1.204	1.440	5.51	4.29	2	3	163	19.6	9.98

Q9Y2B9	cAMP-dependent protein kinase inhibitor gamma OS=Homo sapiens GN=PKIG PE=2 SV=1 - [IPKG_HUMAN]	21.05	1	1	1	11	1.469	2.420	1.819	1.440	29.25	21.05	2	11	76	7.9	4.25
Q96SK2	Transmembrane protein 209 OS=Homo sapiens GN=TMEM209 PE=1 SV=2 - [TM209_HUMAN]	5.88	1	3	3	6	1.293	1.134	0.856	1.441	15.75	5.88	4	6	561	62.9	8.63
P62081	40S ribosomal protein S7 OS=Homo sapiens GN=RPS7 PE=1 SV=1 - [RS7_HUMAN]	59.79	1	12	12	51	1.227	1.368	1.042	1.441	100.50	59.79	16	51	194	22.1	10.10
Q8N142	Adenylosuccinate synthetase isozyme 1 OS=Homo sapiens GN=ADSSL1 PE=2 SV=1 - [PURA1_HUMAN]	7.66	1	2	3	10	2.185	1.166	1.283	1.441	26.27	7.66	5	10	457	50.2	8.59
Q9BQE9	B-cell CLL/lymphoma 7 protein family member B OS=Homo sapiens GN=BCL7B PE=1 SV=1 - [BCL7B_HUMAN]	5.45	2	1	2	8	0.853	1.288	1.360	1.441	15.12	5.45	4	8	202	22.2	4.75
O60573	Eukaryotic translation initiation factor 4E type 2 OS=Homo sapiens GN=EIF4E2 PE=1 SV=1 - [IF4E2_HUMAN]	6.94	1	1	1	2	1.032	1.260	0.890	1.442	5.60	6.94	1	2	245	28.3	8.88
Q7Z4H7	HAUS augmin-like complex subunit 6 OS=Homo sapiens GN=HAUS6 PE=1 SV=2 - [HAUS6_HUMAN]	3.56	1	1	3	3	1.192	1.190	0.870	1.442	5.75	3.56	3	3	955	108.6	6.47
Q01780	Exosome component 10 OS=Homo sapiens GN=EXOSC10 PE=1 SV=2 - [EXOSX_HUMAN]	11.07	1	9	9	24	0.919	1.090	0.944	1.443	47.03	11.07	13	24	885	100.8	8.46
Q9Y4G6	Talin-2 OS=Homo sapiens GN=TLN2 PE=1 SV=4 - [TLN2_HUMAN]	31.39	3	48	70	356	1.496	1.012	0.978	1.443	845.38	31.39	112	356	2542	271.4	5.57

Q16740	ATP-dependent Clp protease proteolytic subunit, mitochondrial OS=Homo sapiens GN=CLPP PE=1 SV=1 - [CLPP_HUMAN]	36.82	1	8	8	24	1.209	1.082	1.161	1.443	69.11	36.82	12	24	277	30.2	8.09
Q14CN4	Keratin, type II cytoskeletal 72 OS=Homo sapiens GN=KRT72 PE=1 SV=2 - [K2C72_HUMAN]	18.00	2	3	6	12	1.192	1.940	1.717	1.444	24.31	18.00	7	12	511	55.8	6.89
Q8N4X5	Actin filament-associated protein 1-like 2 OS=Homo sapiens GN=AFAP1L2 PE=1 SV=1 - [AF1L2_HUMAN]	2.81	1	2	2	2	0.783	0.778	1.063	1.444	7.50	2.81	2	2	818	91.2	5.31
Q9H0D6	5'-3' exoribonuclease 2 OS=Homo sapiens GN=XRN2 PE=1 SV=1 - [XRN2_HUMAN]	17.68	1	15	15	48	1.504	1.280	0.859	1.444	130.67	17.68	24	48	950	108.5	7.47
O43684	Mitotic checkpoint protein BUB3 OS=Homo sapiens GN=BUB3 PE=1 SV=1 - [BUB3_HUMAN]	24.70	1	7	7	31	0.971	0.850	0.687	1.445	82.30	24.70	13	31	328	37.1	6.84
P48960	CD97 antigen OS=Homo sapiens GN=CD97 PE=1 SV=4 - [CD97_HUMAN]	10.54	2	6	6	17	1.851	1.242	1.314	1.445	33.04	10.54	10	17	835	91.8	6.87
Q9H7L9	Sin3 histone deacetylase corepressor complex component SDS3 OS=Homo sapiens GN=SUDS3 PE=1 SV=2 - [SDS3_HUMAN]	7.01	1	2	2	5	0.583	2.309	1.243	1.445	14.81	7.01	2	5	328	38.1	5.66
Q5T8P6	RNA-binding protein 26 OS=Homo sapiens GN=RBM26 PE=1 SV=3 - [RBM26_HUMAN]	6.95	1	7	7	11	1.156	1.173	0.986	1.446	28.30	6.95	9	11	1007	113.5	9.16

Q8IW45	ATP-dependent (S)-NAD(P)H-hydrate dehydratase OS=Homo sapiens GN=CARKD PE=1 SV=1 - [NNRD_HUMAN]	23.05	1	6	6	20	1.482	1.209	1.241	1.446	47.87	23.05	9	20	347	36.6	8.06
Q5KU26	Collectin-12 OS=Homo sapiens GN=COLEC12 PE=1 SV=3 - [COL12_HUMAN]	11.05	1	6	6	9	0.918	1.440	1.267	1.446	16.38	11.05	9	9	742	81.5	5.69
Q9Y221	60S ribosome subunit biogenesis protein NIP7 homolog OS=Homo sapiens GN=NIP7 PE=1 SV=1 - [NIP7_HUMAN]	7.78	1	1	1	2	0.912	1.001	0.688	1.446	0.00	7.78	1	2	180	20.4	8.51
Q14691	DNA replication complex GINS protein PSF1 OS=Homo sapiens GN=GINS1 PE=1 SV=1 - [PSF1_HUMAN]	3.57	1	1	1	1	0.702	1.048	1.781	1.446	3.19	3.57	1	1	196	23.0	7.39
P35250	Replication factor C subunit 2 OS=Homo sapiens GN=RFC2 PE=1 SV=3 - [RFC2_HUMAN]	9.89	1	3	3	4	1.149	1.572	1.146	1.446	7.76	9.89	4	4	354	39.1	6.44
Q12996	Cleavage stimulation factor subunit 3 OS=Homo sapiens GN=CSTF3 PE=1 SV=1 - [CSTF3_HUMAN]	4.32	1	2	3	4	1.236	1.750	1.276	1.447	14.16	4.32	4	4	717	82.9	8.12
O43719	HIV Tat-specific factor 1 OS=Homo sapiens GN=HTATSF1 PE=1 SV=1 - [HTSF1_HUMAN]	8.87	1	6	6	14	1.129	1.043	0.914	1.447	32.20	8.87	7	14	755	85.8	4.40
Q13868	Exosome complex component RRP4 OS=Homo sapiens GN=EXOSC2 PE=1 SV=2 - [EXOS2_HUMAN]	5.80	1	1	1	1	0.966	1.813	2.341	1.448	3.82	5.80	1	1	293	32.8	7.50

Q15262	Receptor-type tyrosine-protein phosphatase kappa OS=Homo sapiens GN=PTPRK PE=1 SV=2 - [PTPRK_HUMAN	11.61	1	11	13	37	1.454	0.972	1.129	1.448	92.43	11.61	19	37	1439	162.0	5.90
Q5EG05	Caspase recruitment domain-containing protein 16 OS=Homo sapiens GN=CARD16 PE=1 SV=1 - [CAR16_HUMAN]	13.20	1	1	3	10	0.766	0.521	0.449	1.449	20.55	13.20	6	10	197	22.6	8.44
P13716	Delta-aminolevulinic acid dehydratase OS=Homo sapiens GN=ALAD PE=1 SV=1 - [HEM2_HUMAN]	29.70	1	8	8	42	1.730	0.874	0.999	1.449	130.57	29.70	14	42	330	36.3	6.79
O00330	Pyruvate dehydrogenase protein X component, mitochondrial OS=Homo sapiens GN=PDHX PE=1 SV=3 - [ODPX_HUMAN]	18.76	1	8	9	37	1.058	0.979	0.952	1.450	85.61	18.76	14	37	501	54.1	8.66
Q9NPG3	Ubinuclein-1 OS=Homo sapiens GN=UBN1 PE=1 SV=2 - [UBN1_HUMAN]	1.94	1	2	2	3	0.944	1.408	1.013	1.450	2.47	1.94	3	3	1134	121.4	9.33
Q8NCS4	Uncharacterized protein ZMYM6NB OS=Homo sapiens GN=ZMYM6NB PE=2 SV=1 - [ZMYNB_HUMAN]	11.69	2	2	2	3	0.813	0.860	0.892	1.450	6.85	11.69	3	3	154	16.9	9.17
Q9NY93	Probable ATP-dependent RNA helicase DDX56 OS=Homo sapiens GN=DDX56 PE=1 SV=1 - [DDX56_HUMAN	6.76	1	3	3	12	1.069	0.860	0.857	1.451	18.16	6.76	6	12	547	61.6	9.26
Q32NB8	CDP-diacylglycerol--glycerol-3-phosphate 3-phosphatidyltransferase, mitochondrial OS=Homo sapiens GN=PGS1 PE=2 SV=1 - [PGPS1_HUMAN]	2.52	1	1	1	2	1.266	1.127	0.936	1.451	3.94	2.52	2	2	556	62.7	8.90

Q9UG56	Phosphatidylserine decarboxylase proenzyme OS=Homo sapiens GN=PISD PE=2 SV=4 - [PISD_HUMAN]	1.71	1	1	1	1	0.925	0.809	1.111	1.451	2.64	1.71	1	1	409	46.6	9.42
Q12933	TNF receptor-associated factor 2 OS=Homo sapiens GN=TRAF2 PE=1 SV=2 - [TRAF2_HUMAN	4.19	1	2	3	6	1.047	1.115	1.247	1.452	13.18	4.19	3	6	501	55.8	7.53
Q6ZUT9	DENN domain-containing protein 5B OS=Homo sapiens GN=DENND5B PE=1 SV=2 - [DEN5B_HUMAN]	1.26	1	1	1	1	1.535	0.782	0.930	1.452	2.74	1.26	1	1	1274	144.9	6.73
Q5SYE7	NHS-like protein 1 OS=Homo sapiens GN=NHSL1 PE=1 SV=2 - [NHSL1_HUMAN]	4.35	1	5	5	7	1.149	1.264	1.002	1.452	6.34	4.35	7	7	1610	170.6	6.96
Q08170	Serine/arginine-rich splicing factor 4 OS=Homo sapiens GN=SRSF4 PE=1 SV=2 - [SRSF4_HUMAN]	17.61	1	7	11	44	0.911	1.329	0.951	1.453	95.93	17.61	17	44	494	56.6	11.52
Q3SY00	Testis-specific protein 10-interacting protein OS=Homo sapiens GN=TSGA10IP PE=2 SV=2 - [T10IP_HUMAN]	6.65	1	1	2	2	1.630	0.808	1.649	1.453	5.16	6.65	2	2	556	62.3	10.05
Q5T2E6	UPF0668 protein C10orf76 OS=Homo sapiens GN=C10orf76 PE=2 SV=1 - [CJ076_HUMAN]	2.76	1	2	2	6	1.342	0.809	0.965	1.454	17.31	2.76	3	6	689	78.7	6.60
P62805	Histone H4 OS=Homo sapiens GN=HIST1H4A PE=1 SV=2 - [H4_HUMAN]	54.37	1	9	9	486	1.376	2.014	1.005	1.454	938.11	54.37	18	486	103	11.4	11.36
O14773	Tripeptidyl-peptidase 1 OS=Homo sapiens GN=TPP1 PE=1 SV=2 - [TPP1_HUMAN]	9.41	1	4	4	28	1.266	1.276	1.086	1.454	92.95	9.41	7	28	563	61.2	6.48

P43628	Killer cell immunoglobulin-like receptor 2DL3 OS=Homo sapiens GN=KIR2DL3 PE=1 SV=1 - [KI2L3_HUMAN]	4.99	2	1	1	1	1.233	1.258	1.819	1.455	0.00	4.99	1	1	341	37.9	6.71
Q8NFH4	Nucleoporin Nup37 OS=Homo sapiens GN=NUP37 PE=1 SV=1 - [NUP37_HUMAN]	3.99	1	1	1	3	1.231	1.131	0.792	1.455	5.49	3.99	2	3	326	36.7	5.92
Q06330	Recombining binding protein suppressor of hairless OS=Homo sapiens GN=RBPJ PE=1 SV=3 - [SUH_HUMAN]	6.40	2	3	3	3	1.380	1.350	1.181	1.455	7.32	6.40	3	3	500	55.6	7.18
Q9NQC1	Protein Jade-2 OS=Homo sapiens GN=JADE2 PE=1 SV=2 - [JADE2_HUMAN]	2.53	1	1	1	1	1.252	1.277	1.266	1.456	0.00	2.53	1	1	790	87.4	5.26
Q9Y6Q3	Zinc finger protein 37 homolog OS=Homo sapiens GN=ZFP37 PE=2 SV=3 - [ZFP37_HUMAN]	5.71	1	3	3	3	1.340	1.854	1.604	1.458	5.42	5.71	3	3	630	71.2	9.09
O15213	WD repeat-containing protein 46 OS=Homo sapiens GN=WDR46 PE=1 SV=3 - [WDR46_HUMAN]	5.57	1	2	2	2	0.777	1.273	1.958	1.458	6.66	5.57	2	2	610	68.0	9.67
Q9Y5Q8	General transcription factor 3C polypeptide 5 OS=Homo sapiens GN=GTF3C5 PE=1 SV=2 - [TF3C5_HUMAN]	5.01	1	2	2	3	1.258	0.783	0.964	1.458	6.44	5.01	3	3	519	59.5	6.90
Q5EBL8	PDZ domain-containing protein 11 OS=Homo sapiens GN=PDZD11 PE=1 SV=2 - [PDZ11_HUMAN]	22.86	1	2	3	9	1.455	1.416	1.522	1.459	23.19	22.86	4	9	140	16.1	7.21
O43306	Adenylate cyclase type 6 OS=Homo sapiens GN=ADCY6 PE=1 SV=2 - [ADCY6_HUMAN]	3.00	1	2	3	5	1.063	0.724	0.995	1.459	17.39	3.00	4	5	1168	130.5	8.22

P13497	Bone morphogenetic protein 1 OS=Homo sapiens GN=BMP1 PE=1 SV=2 - [BMP1_HUMAN]	1.62	1	1	1	2	0.953	1.041	1.929	1.459	5.04	1.62	2	2	986	111.2	6.90
P26358	DNA (cytosine-5)-methyltransferase 1 OS=Homo sapiens GN=DNMT1 PE=1 SV=2 - [DNMT1_HUMA	5.69	1	8	8	12	0.881	1.041	0.738	1.459	21.65	5.69	10	12	1616	183.0	7.75
Q9NUL5	UPF0515 protein C19orf66 OS=Homo sapiens GN=C19orf66 PE=1 SV=2 - [CS066_HUMAN]	13.75	1	4	4	18	1.813	1.086	1.101	1.459	40.52	13.75	8	18	291	33.1	7.25
Q9H4I0	Double-strand-break repair protein rad21-like protein 1 OS=Homo sapiens GN=RAD21L1 PE=2 SV=3 - [RD21L_HUMAN]	8.45	1	2	2	2	1.248	0.753	0.796	1.460	5.20	8.45	2	2	556	63.3	5.16
Q8IU81	Interferon regulatory factor 2-binding protein 1 OS=Homo sapiens GN=IRF2BP1 PE=1 SV=1 - [I2BP1_HUMAN]	16.10	1	6	6	20	1.289	1.236	0.975	1.461	37.84	16.10	10	20	584	61.6	8.18
P16157	Ankyrin-1 OS=Homo sapiens GN=ANK1 PE=1 SV=3 - [ANK1_HUMAN]	0.80	1	1	1	1	1.894	0.798	1.073	1.461	0.00	0.80	1	1	1881	206.1	6.01
Q9NPD3	Exosome complex component RRP41 OS=Homo sapiens GN=EXOSC4 PE=1 SV=3 - [EXOS4_HUMAN]	7.35	1	2	2	5	1.218	0.956	0.844	1.461	3.92	7.35	3	5	245	26.4	6.52
O14828	Secretory carrier-associated membrane protein 3 OS=Homo sapiens GN=SCAMP3 PE=1 SV=3 - [SCAM3_HUMA	27.67	1	7	7	43	1.259	1.075	0.930	1.462	128.85	27.67	11	43	347	38.3	7.64

Q9UNW9	RNA-binding protein Nova-2 OS=Homo sapiens GN=NOVA2 PE=1 SV=1 - [NOVA2_HUMAN]	3.86	1	1	2	3	1.186	1.873	1.168	1.462	4.43	3.86	3	3	492	49.0	8.16
Q5MNV8	F-box only protein 47 OS=Homo sapiens GN=FBXO47 PE=2 SV=2 - [FBX47_HUMAN]	2.88	1	1	1	1	1.840	1.196	1.900	1.462	0.00	2.88	1	1	452	51.9	8.35
Q13823	Nucleolar GTP-binding protein 2 OS=Homo sapiens GN=GNL2 PE=1 SV=1 - [NOG2_HUMAN]	2.19	1	2	2	2	0.507	0.873	0.873	1.463	2.07	2.19	2	2	731	83.6	9.25
Q9Y5U8	Mitochondrial pyruvate carrier 1 OS=Homo sapiens GN=MPC1 PE=1 SV=1 - [MPC1_HUMAN]	6.42	1	1	1	1	2.538	1.042	0.979	1.463	2.11	6.42	1	1	109	12.3	9.61
O95873	Uncharacterized protein C6orf47 OS=Homo sapiens GN=C6orf47 PE=2 SV=2 - [CF047_HUMAN]	4.42	1	1	1	1	1.686	0.957	1.057	1.464	3.09	4.42	1	1	294	31.7	6.37
Q7L4I2	Arginine/serine-rich coiled-coil protein 2 OS=Homo sapiens GN=RSRC2 PE=1 SV=1 - [RSRC2_HUMAN]	5.76	1	2	2	5	1.026	1.244	1.301	1.465	14.47	5.76	3	5	434	50.5	11.33
Q96AX2	Ras-related protein Rab-37 OS=Homo sapiens GN=RAB37 PE=1 SV=3 - [RAB37_HUMAN]	10.76	3	1	3	23	4.026	1.163	0.908	1.466	48.85	10.76	4	23	223	24.8	6.35
Q9BY12	S phase cyclin A-associated protein in the endoplasmic reticulum OS=Homo sapiens GN=SCAPER PE=1 SV=2 - [SCAPE_HUMAN]	4.29	1	3	4	6	1.196	2.052	0.721	1.466	4.61	4.29	4	6	1400	158.2	7.44
Q53GS9	U4/U6.U5 tri-snRNP-associated protein 2 OS=Homo sapiens GN=USP39 PE=1 SV=2 - [SNUT2_HUMAN]	4.42	1	3	4	5	0.905	0.992	1.100	1.466	11.01	4.42	5	5	565	65.3	8.91

Q14331	Protein FRG1 OS=Homo sapiens GN=FRG1 PE=1 SV=1 - [FRG1_HUMAN]	8.91	2	2	2	3	0.888	1.046	1.034	1.467	7.46	8.91	3	3	258	29.2	9.01
Q9BY42	Protein RTF2 homolog OS=Homo sapiens GN=RTFDC1 PE=1 SV=3 - [RTF2_HUMAN]	20.59	1	5	5	14	0.949	0.983	1.252	1.467	28.61	20.59	8	14	306	33.9	8.59
Q96S44	TP53-regulating kinase OS=Homo sapiens GN=TP53RK PE=1 SV=2 - [PRPK_HUMAN]	7.51	1	2	2	4	0.996	0.872	1.097	1.467	8.09	7.51	3	4	253	28.1	9.54
Q7Z2K6	Endoplasmic reticulum metallopeptidase 1 OS=Homo sapiens GN=ERMP1 PE=1 SV=2 - [ERMP1_HUMAN]	5.75	1	5	5	17	1.149	1.304	0.882	1.468	33.80	5.75	9	17	904	100.2	7.52
O15438	Canalicular multispecific organic anion transporter 2 OS=Homo sapiens GN=ABCC3 PE=1 SV=3 - [MRP3_HUMAN]	12.18	1	12	14	43	2.132	0.776	0.754	1.468	108.52	12.18	22	43	1527	169.2	7.20
Q96RT7	Gamma-tubulin complex component 6 OS=Homo sapiens GN=TUBGCP6 PE=1 SV=3 - [GCP6_HUMAN]	3.74	1	4	4	8	1.393	1.522	1.945	1.468	19.23	3.74	6	8	1819	200.4	6.32
P51965	Ubiquitin-conjugating enzyme E2 E1 OS=Homo sapiens GN=UBE2E1 PE=1 SV=1 - [UB2E1_HUMAN]	17.10	2	2	3	3	1.441	1.910	1.926	1.468	4.70	17.10	3	3	193	21.4	8.53
Q13185	Chromobox protein homolog 3 OS=Homo sapiens GN=CBX3 PE=1 SV=4 - [CBX3_HUMAN]	43.72	1	8	9	50	1.455	1.511	0.952	1.469	136.11	43.72	14	50	183	20.8	5.33
P23921	Ribonucleoside-diphosphate reductase large subunit OS=Homo sapiens GN=RRM1 PE=1 SV=1 - [RIR1_HUMAN]	20.83	1	11	11	20	0.942	1.025	0.744	1.470	50.80	20.83	15	20	792	90.0	7.15

P15121	Aldose reductase OS=Homo sapiens GN=AKR1B1 PE=1 SV=3 - [ALDR_HUMAN]	61.71	2	19	20	229	2.106	0.540	0.940	1.470	492.62	61.71	33	229	316	35.8	6.98
Q9NZM5	Glioma tumor suppressor candidate region gene 2 protein OS=Homo sapiens GN=GLTSCR2 PE=1 SV=2 - [GSCR2_HUMAN	11.51	1	4	4	9	0.838	0.842	0.823	1.471	14.14	11.51	7	9	478	54.4	10.32
Q9NQT4	Exosome complex component RRP46 OS=Homo sapiens GN=EXOSC5 PE=1 SV=1 - [EXOS5_HUMAN]	23.40	1	3	3	4	1.050	1.106	0.719	1.471	5.78	23.40	3	4	235	25.2	7.59
Q9BZK7	F-box-like/WD repeat-containing protein TBL1XR1 OS=Homo sapiens GN=TBL1XR1 PE=1 SV=1 - [TBL1R_HUMAN]	9.14	3	4	4	15	0.867	1.135	0.787	1.471	37.61	9.14	6	15	514	55.6	5.55
Q96RD7	Pannexin-1 OS=Homo sapiens GN=PANX1 PE=1 SV=4 - [PANX1_HUMAN]	6.81	1	2	3	7	1.284	1.343	0.719	1.471	15.90	6.81	4	7	426	48.0	6.01
Q92598	Heat shock protein 105 kDa OS=Homo sapiens GN=HSPH1 PE=1 SV=1 - [HS105_HUMAN]	30.89	2	21	26	111	1.053	1.122	1.046	1.471	268.91	30.89	40	111	858	96.8	5.39
Q8IY85	EF-hand calcium-binding domain-containing protein 13 OS=Homo sapiens GN=EFCAB13 PE=1 SV=2 - [EFC13_HUMAN]	1.03	1	1	1	1	0.665	0.910	0.685	1.472	2.45	1.03	1	1	973	110.1	6.57
O14745	Na(+)/H(+) exchange regulatory cofactor NHE-RF1 OS=Homo sapiens GN=SLC9A3R1 PE=1 SV=4 - [NHRF1_HUMAN]	13.97	1	5	5	16	1.103	1.354	1.248	1.472	33.61	13.97	6	16	358	38.8	5.77

P51811	Membrane transport protein XK OS=Homo sapiens GN=XK PE=1 SV=5 - [XK_HUMAN]	3.60	1	1	1	1	1.866	1.738	1.424	1.472	0.00	3.60	1	1	444	50.9	8.25
P13747	HLA class I histocompatibility antigen, alpha chain E OS=Homo sapiens GN=HLA-E PE=1 SV=3 - [HLAE_HUMAN]	20.95	1	3	5	31	1.296	1.160	0.695	1.472	86.28	20.95	8	31	358	40.1	5.95
P48651	Phosphatidylserine synthase 1 OS=Homo sapiens GN=PTDSS1 PE=1 SV=1 - [PTSS1_HUMAN]	4.23	1	2	2	5	1.823	0.930	0.954	1.472	11.23	4.23	3	5	473	55.5	8.43
Q8NG31	Protein CASC5 OS=Homo sapiens GN=CASC5 PE=1 SV=3 - [CASC5_HUMAN]	2.69	1	2	4	23	2.461	1.924	1.095	1.472	32.07	2.69	5	23	2342	265.2	5.47
Q14980	Nuclear mitotic apparatus protein 1 OS=Homo sapiens GN=NUMA1 PE=1 SV=2 - [NUMA1_HUMAN]	35.22	1	66	68	227	1.605	1.872	0.916	1.473	580.35	35.22	104	227	2115	238.1	5.78
Q8NE18	Putative methyltransferase NSUN7 OS=Homo sapiens GN=NSUN7 PE=2 SV=4 - [NSUN7_HUMAN]	1.53	1	1	1	1	0.643	0.713	0.732	1.473	2.31	1.53	1	1	718	81.0	8.75
Q6ZMZ3	Nesprin-3 OS=Homo sapiens GN=SYNE3 PE=1 SV=2 - [SYNE3_HUMAN]	13.23	2	12	13	49	1.069	0.618	0.851	1.473	132.13	13.23	18	49	975	112.1	6.23
Q6P2C8	Mediator of RNA polymerase II transcription subunit 27 OS=Homo sapiens GN=MED27 PE=1 SV=1 - [MED27_HUMAN]	3.22	1	1	1	2	0.898	1.080	1.025	1.473	5.67	3.22	2	2	311	35.4	9.31
O60636	Tetraspanin-2 OS=Homo sapiens GN=TSPAN2 PE=2 SV=2 - [TSN2_HUMAN]	8.14	1	2	2	3	8.312	0.226	0.596	1.474	7.78	8.14	3	3	221	24.1	7.77

Q8N806	Putative E3 ubiquitin-protein ligase UBR7 OS=Homo sapiens GN=UBR7 PE=1 SV=2 - [UBR7_HUMAN]	4.47	1	2	2	13	0.874	1.079	0.925	1.474	29.35	4.47	3	13	425	48.0	4.81
O60306	Intron-binding protein aquarius OS=Homo sapiens GN=AQR PE=1 SV=4 - [AQR_HUMAN]	4.31	1	6	8	25	1.106	1.306	0.983	1.474	57.67	4.31	11	25	1485	171.2	6.37
P16401	Histone H1.5 OS=Homo sapiens GN=HIST1H1B PE=1 SV=3 - [H15_HUMAN]	42.04	1	13	20	239	1.325	1.801	0.798	1.475	451.93	42.04	30	239	226	22.6	10.92
Q96Q89	Kinesin-like protein KIF20B OS=Homo sapiens GN=KIF20B PE=1 SV=3 - [KI20B_HUMAN]	2.47	1	3	5	12	1.125	2.135	1.987	1.476	21.66	2.47	5	12	1820	210.5	5.67
Q9P016	Thymocyte nudear protein 1 OS=Homo sapiens GN=THYN1 PE=1 SV=1 - [THYN1_HUMAN]	29.33	1	7	7	23	1.234	2.158	1.149	1.476	50.27	29.33	9	23	225	25.7	9.25
O15120	1-acyl-sn-glycerol-3-phosphate acyltransferase beta OS=Homo sapiens GN=AGPAT2 PE=1 SV=1 - [PLCB_HUMAN]	5.04	1	1	1	2	1.275	0.608	0.988	1.477	9.57	5.04	1	2	278	30.9	9.01
Q17R31	Putative deoxyribonudease TATDN3 OS=Homo sapiens GN=TATDN3 PE=1 SV=1 - [TATD3_HUMAN]	4.38	1	2	2	6	1.093	0.964	0.993	1.478	10.39	4.38	3	6	274	30.3	7.15
P43490	Nicotinamide phosphoribosyltransferase OS=Homo sapiens GN=NAMPT PE=1 SV=1 - [NAMPT_HUMAN]	48.27	1	23	23	192	1.260	1.258	1.034	1.478	507.48	48.27	36	192	491	55.5	7.15
Q96FB5	Protein RRNAD1 OS=Homo sapiens GN=RRNAD1 PE=2 SV=2 - [RRNAD_HUMAN]	1.47	1	1	1	1	0.889	1.179	2.941	1.480	2.13	1.47	1	1	475	52.9	7.97

Q96EY5	Multivesicular body subunit 12A OS=Homo sapiens GN=MVB12A PE=1 SV=1 - [MB12A_HUMAN]	10.99	1	2	2	2	1.181	0.625	1.093	1.480	5.86	10.99	2	2	273	28.8	8.91
O43759	Synaptogyrin-1 OS=Homo sapiens GN=SYNGR1 PE=1 SV=2 - [SNG1_HUMAN]	10.30	1	2	2	10	1.645	0.509	0.669	1.480	18.12	10.30	4	10	233	25.4	4.68
Q9NUQ3	Gamma-taxilin OS=Homo sapiens GN=TXLNG PE=1 SV=2 - [TXLNG_HUMAN]	13.83	2	4	8	17	0.740	1.513	1.122	1.480	35.15	13.83	11	17	528	60.5	7.52
O76041	Nebulette OS=Homo sapiens GN=NEBL PE=1 SV=1 - [NEBL_HUMAN]	2.07	1	2	2	2	1.202	0.481	0.643	1.480	0.00	2.07	2	2	1014	116.4	7.99
Q86UA1	Pre-mRNA-processing factor 39 OS=Homo sapiens GN=PRPF39 PE=1 SV=3 - [PRP39_HUMAN]	3.89	1	2	2	4	0.841	1.059	1.230	1.481	7.97	3.89	2	4	669	78.4	5.40
Q5VZ46	Uncharacterized protein KIAA1614 OS=Homo sapiens GN=KIAA1614 PE=2 SV=3 - [K1614_HUMAN]	6.30	1	4	4	8	0.785	0.576	1.331	1.482	2.02	6.30	4	8	1190	126.5	8.82
Q96SN8	CDK5 regulatory subunit-associated protein 2 OS=Homo sapiens GN=CDK5RAP2 PE=1 SV=5 - [CK5P2_HUMAN]	2.27	3	2	6	13	0.752	0.916	1.048	1.483	16.06	2.27	6	13	1893	214.9	5.58
Q9H3Z7	Abhydrolase domain-containing protein 16B OS=Homo sapiens GN=ABHD16B PE=2 SV=1 - [ABHGB_HUMAN]	1.49	1	1	1	1	1.358	2.213	0.990	1.483	2.44	1.49	1	1	469	52.5	8.57
P29083	General transcription factor IIE subunit 1 OS=Homo sapiens GN=GTF2E1 PE=1 SV=2 - [T2EA_HUMAN]	8.43	1	3	4	5	0.561	0.888	0.648	1.483	14.38	8.43	4	5	439	49.4	4.82

Q14683	Structural maintenance of chromosomes protein 1A OS=Homo sapiens GN=SMC1A PE=1 SV=2 - [SMC1A_HUMA	33.50	3	40	41	119	1.293	1.394	0.906	1.483	278.09	33.50	62	119	1233	143.1	7.64
Q9Y4C8	Probable RNA-binding protein 19 OS=Homo sapiens GN=RBM19 PE=1 SV=3 - [RBM19_HUMAN]	8.65	1	5	6	6	1.572	1.339	0.996	1.484	14.05	8.65	6	6	960	107.3	6.54
Q9UHV7	Mediator of RNA polymerase II transcription subunit 13 OS=Homo sapiens GN=MED13 PE=1 SV=3 - [MED13_HUMAN]	0.46	1	1	1	2	1.176	1.404	1.510	1.484	6.71	0.46	1	2	2174	239.1	5.64
Q9UKX7	Nuclear pore complex protein Nup50 OS=Homo sapiens GN=NUP50 PE=1 SV=2 - [NUP50_HUMAN]	19.87	1	7	7	17	0.972	1.029	0.762	1.485	48.64	19.87	11	17	468	50.1	7.06
Q6IPR1	LYR motif-containing protein 5 OS=Homo sapiens GN=LYRM5 PE=2 SV=2 - [LYRM5_HUMAN]	10.00	1	1	1	2	2.182	0.988	1.049	1.485	4.41	10.00	1	2	90	10.9	9.92
Q9Y566	SH3 and multiple ankyrin repeat domains protein 1 OS=Homo sapiens GN=SHANK1 PE=1 SV=2 - [SHAN1_HUMAN	3.33	1	3	3	4	1.226	0.651	1.143	1.486	7.05	3.33	3	4	2161	224.8	8.15
Q9Y3E0	Vesicle transport protein GOT1B OS=Homo sapiens GN=GOLT1B PE=1 SV=1 - [GOT1B_HUMA	9.42	1	1	1	3	2.387	1.754	1.294	1.486	7.48	9.42	1	3	138	15.4	10.36
Q8WYP5	Protein ELYS OS=Homo sapiens GN=AHCTF1 PE=1 SV=3 - [ELYS_HUMAN]	3.80	1	7	8	12	1.198	1.217	1.047	1.486	27.16	3.80	9	12	2266	252.3	6.60

Q7L9B9	Endonuclease/exonuclease/phosphatase family domain-containing protein 1 OS=Homo sapiens GN=EEPD1 PE=1 SV=2 - [EEPD1_HUMAN]	16.52	1	8	8	18	1.548	0.742	1.485	1.487	49.37	16.52	12	18	569	62.4	8.41
O95391	Pre-mRNA-splicing factor SLU7 OS=Homo sapiens GN=SLU7 PE=1 SV=2 - [SLU7_HUMAN]	1.19	1	1	1	1	1.338	1.370	1.063	1.490	3.01	1.19	1	1	586	68.3	7.14
P22570	NADPH:adrenodoxin oxidoreductase, mitochondrial OS=Homo sapiens GN=FDXR PE=1 SV=3 - [ADRO_HUMAN]	37.68	1	18	18	46	1.174	1.313	1.050	1.490	113.46	37.68	30	46	491	53.8	8.44
O75525	KH domain-containing, RNA-binding, signal transduction-associated protein 3 OS=Homo sapiens GN=KHDRBS3 PE=1 SV=1 - [KHDR3_HUMAN]	17.63	1	5	7	18	1.006	0.916	1.226	1.492	46.49	17.63	7	18	346	38.8	7.61
Q96N53	Putative uncharacterized protein encoded by LINC00167 OS=Homo sapiens GN=LINC00167 PE=5 SV=1 - [CK037_HUMAN	11.56	1	1	1	1	2.795	1.326	1.399	1.492	2.50	11.56	1	1	147	15.4	11.85
Q9BT09	Protein canopy homolog 3 OS=Homo sapiens GN=CNPY3 PE=1 SV=1 - [CNPY3_HUMAN]	34.17	1	8	8	29	0.970	1.226	0.869	1.493	77.49	34.17	14	29	278	30.7	5.49
Q9NX02	NACHT, LRR and PYD domains-containing protein 2 OS=Homo sapiens GN=NLRP2 PE=1 SV=1 - [NALP2_HUMAN]	0.85	1	1	1	1	0.278	0.329	1.464	1.493	0.00	0.85	1	1	1062	120.4	6.07

Q9P2R6	Arginine-glutamic acid dipeptide repeats protein OS=Homo sapiens GN=RERE PE=1 SV=2 - [RERE_HUMAN]	0.51	1	1	1	1	0.717	1.744		1.493	2.20	0.51	1	1	1566	172.3	7.69
Q15526	Surfeit locus protein 1 OS=Homo sapiens GN=SURF1 PE=1 SV=1 - [SURF1_HUMAN]	7.00	1	2	2	3	2.154	1.961	2.164	1.493	3.91	7.00	3	3	300	33.3	9.60
Q16236	Nudear factor erythroid 2-related factor 2 OS=Homo sapiens GN=NFE2L2 PE=1 SV=3 - [NF2L2_HUMAN]	0.99	1	1	1	1	0.971	0.684	0.885	1.494	1.75	0.99	1	1	605	67.8	4.78
Q9C005	Protein dpy-30 homolog OS=Homo sapiens GN=DPY30 PE=1 SV=1 - [DPY30_HUMAN]	17.17	2	3	3	7	2.365	2.070	1.296	1.495	5.77	17.17	5	7	99	11.2	4.88
O94887	FERM, RhoGEF and pleckstrin domain-containing protein 2 OS=Homo sapiens GN=FARP2 PE=1 SV=3 - [FARP2_HUMAN]	5.60	1	3	4	6	0.872	1.633	1.338	1.496	8.77	5.60	4	6	1054	119.8	8.79
P31269	Homeobox protein Hox-A9 OS=Homo sapiens GN=HOXA9 PE=1 SV=4 - [HXA9_HUMAN]	4.41	2	1	1	1	1.948	0.975	1.084	1.496	0.00	4.41	1	1	272	30.2	8.62
Q15427	Splicing factor 3B subunit 4 OS=Homo sapiens GN=SF3B4 PE=1 SV=1 - [SF3B4_HUMAN]	15.57	1	4	4	15	1.092	1.251	1.018	1.497	44.95	15.57	5	15	424	44.4	8.56
O00268	Transcription initiation factor TFIID subunit 4 OS=Homo sapiens GN=TAF4 PE=1 SV=2 - [TAF4_HUMAN]	5.81	1	3	3	6	1.506	1.334	1.228	1.498	7.10	5.81	4	6	1085	110.0	9.94

P50150	Guanine nucleotide-binding protein G(I)/G(S)/G(O) subunit gamma-4 OS=Homo sapiens GN=GNG4 PE=1 SV=1 - [GBG4_HUMAN]	21.33	1	1	1	1	2.469	0.807	0.741	1.498	1.79	21.33	1	1	75	8.4	7.08
Q8WYL5	Protein phosphatase Slingshot homolog 1 OS=Homo sapiens GN=SSH1 PE=1 SV=2 - [SSH1_HUMAN]	10.49	1	7	8	10	0.927	0.934	1.262	1.498	25.75	10.49	8	10	1049	115.4	6.25
Q9H446	RWD domain-containing protein 1 OS=Homo sapiens GN=RWDD1 PE=1 SV=1 - [RWDD1_HUMAN]	4.53	1	2	2	2	0.722	0.959	0.943	1.498	0.00	4.53	2	2	243	27.9	4.20
Q9Y679	Ancient ubiquitous protein 1 OS=Homo sapiens GN=AUP1 PE=1 SV=1 - [AUP1_HUMAN]	12.82	1	5	5	11	1.265	1.243	0.725	1.498	31.97	12.82	6	11	476	53.0	8.09
Q7Z7F0	UPF0469 protein KIAA0907 OS=Homo sapiens GN=KIAA0907 PE=1 SV=1 - [K0907_HUMAN]	4.72	1	1	2	2	1.086	1.714	1.209	1.498	3.21	4.72	2	2	614	64.8	8.73
Q5VST6	Alpha/beta hydrolase domain-containing protein 17B OS=Homo sapiens GN=ABHD17B PE=2 SV=1 - [AB17B_HUMAN]	2.78	1	1	1	1	0.836	1.210	1.099	1.499	1.82	2.78	1	1	288	32.2	6.29
P00374	Dihydrofolate reductase OS=Homo sapiens GN=DHFR PE=1 SV=2 - [DYR_HUMAN]	7.49	1	1	1	3	1.291	1.092	0.958	1.499	7.59	7.49	2	3	187	21.4	7.42
Q9Y333	U6 snRNA-associated Sm-like protein LSm2 OS=Homo sapiens GN=LSM2 PE=1 SV=1 - [LSM2_HUMAN]	27.37	1	4	4	7	1.121	1.622	1.415	1.499	19.01	27.37	6	7	95	10.8	6.52

Q13671	Ras and Rab interactor 1 OS=Homo sapiens GN=RIN1 PE=1 SV=4 - [RIN1_HUMAN]	9.83	1	5	5	9	0.837	1.273	1.033	1.503	23.20	9.83	7	9	783	84.0	8.02
P35269	General transcription factor IIF subunit 1 OS=Homo sapiens GN=GTF2F1 PE=1 SV=2 - [T2FA_HUMAN]	17.60	1	10	10	17	1.505	0.890	0.766	1.503	40.12	17.60	10	17	517	58.2	7.49
Q96I15	Selenocysteine lyase OS=Homo sapiens GN=SCLY PE=1 SV=4 - [SCLY_HUMAN]	22.02	1	7	7	26	1.069	0.843	1.058	1.503	62.25	22.02	10	26	445	48.1	7.12
Q9P0P8	Uncharacterized protein C6orf203 OS=Homo sapiens GN=C6orf203 PE=1 SV=1 - [CF203_HUMAN]	9.17	1	1	1	1	1.150	1.671	1.107	1.505	5.68	9.17	1	1	240	27.9	9.29
P61218	DNA-directed RNA polymerases I, II, and III subunit RPABC2 OS=Homo sapiens GN=POLR2F PE=1 SV=1 - [RPAB2_HUMAN]	6.30	1	1	1	1	0.981	1.318	1.074	1.505	1.93	6.30	1	1	127	14.5	4.22
O60264	SWI/SNF-related matrix-associated actin-dependent regulator of chromatin subfamily A member 5 OS=Homo sapiens GN=SMARCA5 PE=1 SV=1 - [SMCA5_HUMA	14.45	1	11	15	51	1.097	1.368	1.024	1.505	114.44	14.45	24	51	1052	121.8	8.09
Q93074	Mediator of RNA polymerase II transcription subunit 12 OS=Homo sapiens GN=MED12 PE=1 SV=4 - [MED12_HUMAN]	1.24	1	2	2	13	1.369	1.135	0.812	1.506	40.59	1.24	2	13	2177	242.9	7.05

Q96CM8	Acyl-CoA synthetase family member 2, mitochondrial OS=Homo sapiens GN=ACSF2 PE=1 SV=2 - [ACSF2_HUMAN	14.15	1	7	7	21	1.050	0.761	0.970	1.506	63.79	14.15	10	21	615	68.1	7.55
Q15274	Nicotinate-nucleotide pyrophosphorylase [carboxylating] OS=Homo sapiens GN=QPRT PE=1 SV=3 - [NADC_HUMAN]	17.51	1	5	5	16	0.947	1.106	0.530	1.507	28.56	17.51	8	16	297	30.8	6.21
Q9BZX2	Uridine-cytidine kinase 2 OS=Homo sapiens GN=UCK2 PE=1 SV=1 - [UCK2_HUMAN]	17.62	2	5	5	13	0.899	0.617	0.845	1.507	32.35	17.62	7	13	261	29.3	6.70
Q86UK5	Limbin OS=Homo sapiens GN=EVC2 PE=1 SV=1 - [LBN_HUMAN]	3.67	1	3	4	5	1.600	1.257	0.605	1.507	10.45	3.67	4	5	1308	147.9	6.96
Q96G21	U3 small nucleolar ribonucleoprotein protein IMP4 OS=Homo sapiens GN=IMP4 PE=1 SV=1 - [IMP4_HUMAN]	12.03	1	2	2	11	0.975	0.929	0.623	1.508	41.29	12.03	4	11	291	33.7	9.47
Q5SXM8	DNL-type zinc finger protein OS=Homo sapiens GN=DNLZ PE=1 SV=1 - [DNLZ_HUMAN]	13.48	1	2	2	4	1.073	1.419	1.258	1.509	11.90	13.48	4	4	178	19.2	9.80
Q9BRT2	Ubiquinol-cytochrome-c reductase complex assembly factor 2 OS=Homo sapiens GN=UQCC2 PE=1 SV=1 - [UQCC2_HUMAN]	21.43	1	3	3	5	1.159	1.410	0.908	1.510	18.09	21.43	4	5	126	14.9	7.37
Q9BSI4	TERF1-interacting nuclear factor 2 OS=Homo sapiens GN=TINF2 PE=1 SV=1 - [TINF2_HUMAN]	5.99	1	2	2	4	1.117	0.701	0.776	1.510	2.35	5.99	2	4	451	50.0	7.96

Q9ULX3	RNA-binding protein NOB1 OS=Homo sapiens GN=NOB1 PE=1 SV=1 - [NOB1_HUMAN]	4.61	1	2	2	3	1.124	0.971	0.828	1.511	8.32	4.61	3	3	412	46.6	7.18
P52292	Importin subunit alpha-1 OS=Homo sapiens GN=KPNA2 PE=1 SV=1 - [IMA1_HUMAN]	17.20	1	9	9	40	0.834	0.784	0.768	1.512	119.39	17.20	14	40	529	57.8	5.40
Q9H628	Ras-related and estrogen-regulated growth inhibitor-like protein OS=Homo sapiens GN=RERGL PE=2 SV=1 - [RERGL_HUMAN]	6.83	1	1	1	2	1.178	0.563		1.513	5.10	6.83	1	2	205	23.8	9.51
Q9HC98	Serine/threonine-protein kinase Nek6 OS=Homo sapiens GN=NEK6 PE=1 SV=2 - [NEK6_HUMAN]	11.50	1	2	4	16	1.328	1.223	1.044	1.513	40.64	11.50	6	16	313	35.7	8.03
Q01201	Transcription factor RelB OS=Homo sapiens GN=RELB PE=1 SV=2 - [RELB_HUMAN]	6.56	1	2	3	4	1.404	1.146	1.574	1.514	5.78	6.56	4	4	579	62.1	6.24
Q6NUN0	Acyl-coenzyme A synthetase ACSM5, mitochondrial OS=Homo sapiens GN=ACSM5 PE=2 SV=2 - [ACSM5_HUMAN]	3.11	1	1	1	2	1.390	0.987	0.933	1.514	2.79	3.11	1	2	579	64.7	8.40
Q14031	Collagen alpha-6(IV) chain OS=Homo sapiens GN=COL4A6 PE=1 SV=3 - [CO4A6_HUMAN]	3.19	1	3	3	3	1.393	1.532	1.200	1.515	6.08	3.19	3	3	1691	163.7	9.20
Q96ME7	Zinc finger protein 512 OS=Homo sapiens GN=ZNF512 PE=1 SV=2 - [ZN512_HUMAN]	5.11	1	3	3	6	1.522	1.095	1.236	1.516	11.72	5.11	4	6	567	64.6	9.76
O95674	Phosphatidate cytidylyltransferase 2 OS=Homo sapiens GN=CDS2 PE=1 SV=1 - [CDS2_HUMAN]	9.89	1	2	3	10	1.558	0.641	0.569	1.517	30.92	9.89	5	10	445	51.4	7.09

P24941	Cyclin-dependent kinase 2 OS=Homo sapiens GN=CDK2 PE=1 SV=2 - [CDK2_HUMAN]	23.15	8	4	7	19	1.132	1.031	1.010	1.517	32.05	23.15	10	19	298	33.9	8.68
Q9BQ67	Glutamate-rich WD repeat-containing protein 1 OS=Homo sapiens GN=GRWD1 PE=1 SV=1 - [GRWD1_HUMAN]	10.31	1	4	4	6	0.828	1.156	0.986	1.518	14.81	10.31	6	6	446	49.4	4.92
Q8WXW3	Progesterone-induced-blocking factor 1 OS=Homo sapiens GN=PIBF1 PE=1 SV=2 - [PIBF1_HUMAN]	3.96	1	2	4	5	0.819	1.378	1.233	1.518	9.13	3.96	4	5	757	89.7	6.02
Q9Y3B9	RRP15-like protein OS=Homo sapiens GN=RRP15 PE=1 SV=2 - [RRP15_HUMAN]	8.16	1	3	3	7	0.621	0.830	0.808	1.518	13.99	8.16	4	7	282	31.5	5.52
O75934	Pre-mRNA-splicing factor SPF27 OS=Homo sapiens GN=BCAS2 PE=1 SV=1 - [SPF27_HUMAN]	24.89	1	6	6	17	1.087	1.408	1.046	1.518	40.31	24.89	9	17	225	26.1	5.66
Q16831	Uridine phosphorylase 1 OS=Homo sapiens GN=UPP1 PE=1 SV=1 - [UPP1_HUMAN]	9.68	1	3	3	5	1.357	0.670	1.097	1.518	13.37	9.68	3	5	310	33.9	7.88
Q9H706	GRB2-associated and regulator of MAPK protein OS=Homo sapiens GN=GAREM PE=1 SV=2 - [GAREM_HUMAN]	0.91	1	1	1	1	1.270	1.120	1.423	1.520	1.69	0.91	1	1	876	97.1	6.77
P31689	DnaJ homolog subfamily A member 1 OS=Homo sapiens GN=DNAJA1 PE=1 SV=2 - [DNJA1_HUMAN]	39.55	3	14	16	70	1.167	1.112	1.006	1.520	185.43	39.55	26	70	397	44.8	7.08
P02787	Serotransferrin OS=Homo sapiens GN=TF PE=1 SV=3 - [TRFE_HUMAN]	2.01	1	1	1	3	1.474	0.543	0.683	1.521	7.34	2.01	1	3	698	77.0	7.12

P52926	High mobility group protein HMGI-C OS=Homo sapiens GN=HMGA2 PE=1 SV=1 - [HMGA2_HUMAN]	41.28	1	4	4	26	1.213	3.180	10.678	1.521	69.20	41.28	6	26	109	11.8	10.62
Q06587	E3 ubiquitin-protein ligase RING1 OS=Homo sapiens GN=RING1 PE=1 SV=2 - [RING1_HUMAN]	14.29	1	4	4	13	1.098	1.139	1.190	1.523	33.05	14.29	7	13	406	42.4	5.62
Q13595	Transformer-2 protein homolog alpha OS=Homo sapiens GN=TRA2A PE=1 SV=1 - [TRA2A_HUMAN]	26.60	1	4	6	21	1.136	1.290	0.848	1.523	52.61	26.60	8	21	282	32.7	11.27
Q12894	Interferon-related developmental regulator 2 OS=Homo sapiens GN=IFRD2 PE=1 SV=3 - [IFRD2_HUMAN]	4.15	1	2	2	2	0.454	1.369	1.237	1.524	0.00	4.15	2	2	506	54.8	7.94
Q15326	Zinc finger MYND domain-containing protein 11 OS=Homo sapiens GN=ZMYND11 PE=1 SV=2 - [ZMY11_HUMAN]	2.33	1	1	2	3	1.269	1.586	1.174	1.525	8.09	2.33	2	3	602	70.9	8.53
P52951	Homeobox protein GBX-2 OS=Homo sapiens GN=GBX2 PE=2 SV=3 - [GBX2_HUMAN]	10.06	1	2	2	2	1.847	1.991	2.016	1.525	4.73	10.06	2	2	348	37.3	8.38
Q9NXG6	Transmembrane prolyl 4-hydroxylase OS=Homo sapiens GN=P4HTM PE=1 SV=2 - [P4HTM_HUMAN]	12.55	1	4	4	18	1.431	0.986	0.970	1.526	53.59	12.55	6	18	502	56.6	6.06
O00479	High mobility group nucleosome-binding domain-containing protein 4 OS=Homo sapiens GN=HMGN4 PE=1 SV=3 - [HMGN4_HUMAN]	47.78	1	4	4	19	1.742	2.047	1.491	1.527	20.67	47.78	5	19	90	9.5	10.48

Q9Y6N5	Sulfide:quinone oxidoreductase, mitochondrial OS=Homo sapiens GN=SQRDL PE=1 SV=1 - [SQRD_HUMAN]	57.11	1	28	28	304	1.694	0.745	0.910	1.527	853.02	57.11	47	304	450	49.9	9.11
Q9NRP4	Succinate dehydrogenase assembly factor 3, mitochondrial OS=Homo sapiens GN=ACN9 PE=2 SV=1 - [SDHF3_HUMAN	4.80	1	1	1	1	0.822	1.163	1.080	1.528	0.00	4.80	1	1	125	14.6	9.07
Q9H1B7	Interferon regulatory factor 2-binding protein-like OS=Homo sapiens GN=IRF2BPL PE=1 SV=1 - [I2BPL_HUMAN]	14.95	1	7	8	22	1.748	2.047	1.388	1.529	69.04	14.95	12	22	796	82.6	8.24
P04179	Superoxide dismutase [Mn], mitochondrial OS=Homo sapiens GN=SOD2 PE=1 SV=2 - [SODM_HUMAN	36.94	1	12	12	325	1.643	2.262	1.684	1.529	730.29	36.94	21	325	222	24.7	8.25
Q5M775	Cytospin-B OS=Homo sapiens GN=SPECC1 PE=1 SV=1 - [CYTSB_HUMAN]	10.39	1	10	10	20	2.135	0.931	0.711	1.529	58.07	10.39	13	20	1068	118.5	6.70
Q8IZM8	Zinc finger protein 654 OS=Homo sapiens GN=ZNF654 PE=2 SV=3 - [ZN654_HUMAN]	2.07	1	1	1	2	0.906	1.628	1.840	1.529	2.71	2.07	1	2	581	65.8	5.58
P39748	Flap endonuclease 1 OS=Homo sapiens GN=FEN1 PE=1 SV=1 - [FEN1_HUMAN]	24.47	1	8	8	25	1.548	1.266	0.808	1.530	61.14	24.47	11	25	380	42.6	8.62
P26583	High mobility group protein B2 OS=Homo sapiens GN=HMGB2 PE=1 SV=2 - [HMGB2_HUMAN]	40.67	1	11	15	43	1.521	3.726	0.709	1.531	90.09	40.67	21	43	209	24.0	7.81
Q6P3W2	DnaJ homolog subfamily C member 24 OS=Homo sapiens GN=DNAJC24 PE=1 SV=1 - [DJC24_HUMAN]	11.49	1	1	2	2		1.218	2.542	1.532	2.20	11.49	2	2	148	17.0	4.65

Q01658	Protein Dr1 OS=Homo sapiens GN=DR1 PE=1 SV=1 - [NC2B_HUMAN]	21.59	1	4	4	26	1.080	1.300	1.486	1.532	78.46	21.59	6	26	176	19.4	4.75
Q4G0U5	Cilia- and flagella-associated protein 221 OS=Homo sapiens GN=CFAP221 PE=1 SV=2 - [PCDP1_HUMAN	1.43	1	1	1	1	1.326	1.131	0.995	1.533	2.26	1.43	1	1	840	96.8	8.62
Q9HAC7	Succinate--hydroxymethylglutarate CoA-transferase OS=Homo sapiens GN=SUGCT PE=1 SV=2 - [SUCHY_HUMAN]	4.49	1	2	2	2	1.118	1.114	1.046	1.535	2.23	4.49	2	2	445	48.4	8.35
O43196	MutS protein homolog 5 OS=Homo sapiens GN=MSH5 PE=1 SV=1 - [MSH5_HUMAN]	2.52	1	1	2	3	0.661	2.951	2.212	1.535	2.32	2.52	2	3	834	92.8	6.37
Q02252	Methylmalonate-semialdehyde dehydrogenase [acylating], mitochondrial OS=Homo sapiens GN=ALDH6A1 PE=1 SV=2 - [MMSA_HUMAN]	25.98	1	13	14	37	0.837	1.292	1.024	1.537	91.47	25.98	22	37	535	57.8	8.50
Q8WXX5	DnaJ homolog subfamily C member 9 OS=Homo sapiens GN=DNAJC9 PE=1 SV=1 - [DNJC9_HUMAN]	15.00	1	4	4	9	1.103	1.496	1.038	1.539	23.24	15.00	6	9	260	29.9	5.73
Q9Y6T7	Diacylglycerol kinase beta OS=Homo sapiens GN=DGKB PE=2 SV=2 - [DGKB_HUMAN]	1.99	1	1	1	1	1.105	0.466	0.746	1.540	0.00	1.99	1	1	804	90.5	7.85
Q99729	Heterogeneous nuclear ribonucleoprotein A/B OS=Homo sapiens GN=HNRNPAB PE=1 SV=2 - [ROAA_HUMAN]	26.81	1	10	11	61	0.978	1.124	0.900	1.540	147.36	26.81	17	61	332	36.2	8.21

Q6PCB7	Long-chain fatty acid transport protein 1 OS=Homo sapiens GN=SLC27A1 PE=2 SV=1 - [S27A1_HUMAN]	4.95	1	3	3	5	1.404	1.078	1.041	1.541	6.49	4.95	5	5	646	71.1	8.53
O14981	TATA-binding protein-associated factor 172 OS=Homo sapiens GN=BTAF1 PE=1 SV=2 - [BTAF1_HUMAN	3.52	1	4	5	10	0.813	1.216	1.226	1.542	27.05	3.52	6	10	1849	206.8	6.52
Q8IZT9	Protein FAM9C OS=Homo sapiens GN=FAM9C PE=2 SV=1 - [FAM9C_HUMAN]	4.22	2	1	1	5	1.448	0.864	1.236	1.542	8.25	4.22	1	5	166	19.2	5.24
P52701	DNA mismatch repair protein Msh6 OS=Homo sapiens GN=MSH6 PE=1 SV=2 - [MSH6_HUMAN]	11.47	1	13	13	32	1.071	1.252	1.036	1.542	64.11	11.47	21	32	1360	152.7	6.90
O75818	Ribonuclease P protein subunit p40 OS=Homo sapiens GN=RPP40 PE=1 SV=3 - [RPP40_HUMAN]	7.44	1	3	3	8	0.883	1.171	1.525	1.543	25.22	7.44	5	8	363	41.8	6.67
P19388	DNA-directed RNA polymerases I, II, and III subunit RPABC1 OS=Homo sapiens GN=POLR2E PE=1 SV=4 - [RPAB1_HUMAN]	25.71	1	5	5	16	0.806	1.035	0.895	1.543	41.75	25.71	8	16	210	24.5	5.95
Q9H313	Protein tweety homolog 1 OS=Homo sapiens GN=TTYH1 PE=2 SV=1 - [TTYH1_HUMAN]	3.11	1	1	1	1	0.996	2.263	3.091	1.543	0.00	3.11	1	1	450	49.0	4.97
O75340	Programmed cell death protein 6 OS=Homo sapiens GN=PDCD6 PE=1 SV=1 - [PDCD6_HUMAN	16.23	1	4	4	21	1.919	1.514	1.394	1.544	40.04	16.23	8	21	191	21.9	5.40
P78560	Death domain-containing protein CRADD OS=Homo sapiens GN=CRADD PE=1 SV=1 - [CRADD_HUMAN]	34.67	1	6	6	13	0.983	0.807	1.099	1.546	35.03	34.67	9	13	199	22.7	6.80

Q9NRX1	RNA-binding protein PNO1 OS=Homo sapiens GN=PNO1 PE=1 SV=1 - [PNO1_HUMAN]	13.89	1	4	4	8	0.882	1.417	1.171	1.546	18.10	13.89	4	8	252	27.9	9.73
Q14164	Inhibitor of nudear factor kappa-B kinase subunit epsilon OS=Homo sapiens GN=IKBKE PE=1 SV=1 - [IKKE_HUMAN]	3.35	1	1	2	6	1.262	1.203	1.413	1.547	3.79	3.35	4	6	716	80.4	7.84
Q9Y5G0	Protocadherin gamma-B5 OS=Homo sapiens GN=PCDHGB5 PE=2 SV=1 - [PCDGH_HUMAN]	3.47	26	2	3	5	0.728	0.876	1.578	1.549	12.15	3.47	3	5	923	99.8	5.05
Q9NTI5	Sister chromatid cohesion protein PDS5 homolog B OS=Homo sapiens GN=PDS5B PE=1 SV=1 - [PDS5B_HUMAN	14.37	1	17	18	34	1.350	1.467	0.752	1.551	69.02	14.37	24	34	1447	164.6	8.47
Q96PZ0	Pseudouridylat e synthase 7 homolog OS=Homo sapiens GN=PUS7 PE=1 SV=2 - [PUS7_HUMAN]	6.96	1	4	4	6	0.949	0.882	0.980	1.551	15.38	6.96	6	6	661	75.0	6.37
Q9UL42	Paraneoplastic antigen Ma2 OS=Homo sapiens GN=PNMA2 PE=1 SV=2 - [PNMA2_HUMA N]	14.29	1	5	6	20	2.024	0.267	0.327	1.551	35.90	14.29	10	20	364	41.5	4.86
Q5T8A7	Protein phosphatase 1 regulatory subunit 26 OS=Homo sapiens GN=PPP1R26 PE=1 SV=1 - [PPR26_HUMAN]	1.65	2	2	2	3	2.137	1.518	1.309	1.552	1.89	1.65	2	3	1209	127.3	8.59
P16402	Histone H1.3 OS=Homo sapiens GN=HIST1H1D PE=1 SV=2 - [H13_HUMAN]	39.37	2	4	17	143	0.682	6.488	3.465	1.555	297.09	39.37	26	143	221	22.3	11.02
P01024	Complement C3 OS=Homo sapiens GN=C3 PE=1 SV=2 - [CO3_HUMAN]	16.12	1	21	21	44	2.215	1.244	0.918	1.555	104.84	16.12	29	44	1663	187.0	6.40

O95361	Tripartite motif-containing protein 16 OS=Homo sapiens GN=TRIM16 PE=1 SV=3 - [TRI16_HUMAN]	8.16	2	3	3	11	1.629	1.103	1.824	1.555	31.65	8.16	5	11	564	63.9	5.49
Q9HAU8	Arginyl aminopeptidase-like 1 OS=Homo sapiens GN=RNPEPL1 PE=2 SV=2 - [RNPL1_HUMAN]	1.42	1	1	1	1	1.055	1.418	0.943	1.556	2.41	1.42	1	1	494	55.5	4.91
Q8NFP7	Diphosphoinositol polyphosphate phosphohydrolase 3-alpha OS=Homo sapiens GN=NUDT10 PE=1 SV=1 - [NUD10_HUMAN]	41.46	2	2	6	27	0.870	1.018	1.266	1.556	80.47	41.46	7	27	164	18.5	5.68
Q9Y5M1	Uncharacterized protein FAM215A OS=Homo sapiens GN=FAM215A PE=2 SV=2 - [F215A_HUMAN]	14.91	1	1	1	1	2.066	1.395	1.632	1.557	0.00	14.91	1	1	114	12.0	7.87
Q9BTC0	Death-inducer obliterator 1 OS=Homo sapiens GN=DIDO1 PE=1 SV=5 - [DIDO1_HUMAN]	6.43	2	11	12	33	1.316	1.736	1.099	1.557	63.63	6.43	18	33	2240	243.7	7.88
Q53S58	Transmembrane protein 177 OS=Homo sapiens GN=TMEM177 PE=2 SV=1 - [TM177_HUMAN]	2.89	1	1	1	2	1.312	0.713	0.620	1.558	1.68	2.89	1	2	311	33.7	9.61
Q7Z6E9	E3 ubiquitin-protein ligase RBBP6 OS=Homo sapiens GN=RBBP6 PE=1 SV=1 - [RBBP6_HUMAN]	1.40	1	2	2	5	1.289	0.469	1.106	1.561	12.83	1.40	2	5	1792	201.4	9.64
Q6ZN55	Zinc finger protein 574 OS=Homo sapiens GN=ZNF574 PE=1 SV=2 - [ZN574_HUMAN]	1.67	1	1	2	3	1.365	1.283	1.089	1.561	6.50	1.67	2	3	896	98.8	8.07
Q8TCG1	Protein CIP2A OS=Homo sapiens GN=KIAA1524 PE=1 SV=2 - [CIP2A_HUMAN]	5.19	1	4	4	6	3.170	0.922	0.979	1.561	7.49	5.19	4	6	905	102.1	6.23

Q9H8H3	Methyltransferase-like protein 7A OS=Homo sapiens GN=METTL7A PE=1 SV=1 - [MET7A_HUMAN]	17.21	1	3	3	6	0.764	2.763	0.461	1.562	17.99	17.21	5	6	244	28.3	8.38
Q86UL3	Glycerol-3-phosphate acyltransferase 4 OS=Homo sapiens GN=AGPAT6 PE=1 SV=1 - [GPAT4_HUMAN]	5.48	1	2	2	5	3.481	1.551	1.402	1.564	5.72	5.48	2	5	456	52.0	9.19
Q9UKU7	Isobutyryl-CoA dehydrogenase, mitochondrial OS=Homo sapiens GN=ACAD8 PE=1 SV=1 - [ACAD8_HUMAN]	12.29	1	5	5	16	1.379	1.512	1.007	1.564	36.11	12.29	8	16	415	45.0	7.85
Q8TED0	U3 small nucleolar RNA-associated protein 15 homolog OS=Homo sapiens GN=UTP15 PE=1 SV=3 - [UTP15_HUMAN]	9.65	1	4	5	12	0.874	0.701	0.882	1.565	29.24	9.65	6	12	518	58.4	9.11
O95199	RCC1 and BTB domain-containing protein 2 OS=Homo sapiens GN=RCBTB2 PE=2 SV=1 - [RCBT2_HUMAN]	3.09	1	1	1	1	1.415	1.870	4.193	1.566	0.00	3.09	1	1	551	60.3	5.49
P0C0S5	Histone H2A.Z OS=Homo sapiens GN=H2AFZ PE=1 SV=2 - [H2AZ_HUMAN]	35.94	2	3	6	49	1.780	2.719	0.933	1.566	128.55	35.94	10	49	128	13.5	10.58
Q8NA72	Centrosomal protein POC5 OS=Homo sapiens GN=POC5 PE=1 SV=2 - [POC5_HUMAN]	2.43	1	1	1	1	1.345	0.972	0.857	1.566	3.47	2.43	1	1	575	63.3	7.42
P09429	High mobility group protein B1 OS=Homo sapiens GN=HMGB1 PE=1 SV=3 - [HMGB1_HUMAN]	43.26	2	11	16	118	1.762	2.099	0.945	1.567	207.88	43.26	26	118	215	24.9	5.74
Q15025	TNFAIP3-interacting protein 1 OS=Homo sapiens GN=TNIP1 PE=1 SV=2 - [TNIP1_HUMAN]	1.73	1	1	1	2	1.442	0.636	1.097	1.567	2.28	1.73	2	2	636	71.8	6.62

Q9BSQ5	Malcavernin OS=Homo sapiens GN=CCM2 PE=1 SV=1 - [CCM2_HUMAN]	8.78	1	4	4	9	1.665	1.503	1.070	1.568	17.13	8.78	7	9	444	48.8	5.54
Q8NHY5	Checkpoint protein HUS1B OS=Homo sapiens GN=HUS1B PE=1 SV=2 - [HUS1B_HUMAN]	5.04	1	1	1	1	1.189	0.946	0.829	1.568	2.08	5.04	1	1	278	31.0	9.33
Q9UL45	Biogenesis of lysosome-related organelles complex 1 subunit 6 OS=Homo sapiens GN=BLOC1S6 PE=1 SV=1 - [BL1S6_HUMAN]	3.49	1	1	1	1	1.269	2.097	1.442	1.568	0.00	3.49	1	1	172	19.7	6.40
Q9Y657	Spindlin-1 OS=Homo sapiens GN=SPIN1 PE=1 SV=3 - [SPIN1_HUMAN]	17.56	2	3	4	7	0.897	1.867	1.080	1.568	24.23	17.56	5	7	262	29.6	6.96
P05026	Sodium/potassium-transporting ATPase subunit beta-1 OS=Homo sapiens GN=ATP1B1 PE=1 SV=1 - [AT1B1_HUMAN]	12.54	1	3	3	10	1.851	3.053	1.479	1.569	26.57	12.54	5	10	303	35.0	8.53
Q96KB5	Lymphokine-activated killer T-cell-originated protein kinase OS=Homo sapiens GN=PBK PE=1 SV=3 - [TOPK_HUMAN]	13.35	1	5	5	8	1.196	1.022	0.747	1.570	12.66	13.35	7	8	322	36.1	5.12
P19440	Gamma-glutamyltranspeptidase 1 OS=Homo sapiens GN=GGT1 PE=1 SV=2 - [GGT1_HUMAN]	10.90	5	6	6	12	2.463	0.670	0.716	1.570	27.80	10.90	10	12	569	61.4	7.12
Q5TB80	Centrosomal protein of 162 kDa OS=Homo sapiens GN=CEP162 PE=1 SV=2 - [CE162_HUMAN]	1.07	1	1	1	1	1.304	1.143	1.470	1.571	2.08	1.07	1	1	1403	161.8	5.47
O76038	Secretagogin OS=Homo sapiens GN=SCGN PE=1 SV=2 - [SEGN_HUMAN]	7.61	1	1	1	2	0.843	1.214	0.784	1.571	0.00	7.61	1	2	276	32.0	5.41

P78337	Pituitary homeobox 1 OS=Homo sapiens GN=PITX1 PE=1 SV=2 - [PITX1_HUMAN]	8.60	3	2	2	6	1.878	0.860	1.463	1.572	13.63	8.60	3	6	314	34.1	8.98
P56181	NADH dehydrogenase [ubiquinone] flavoprotein 3, mitochondrial OS=Homo sapiens GN=NDUFV3 PE=1 SV=2 - [NDUV3_HUMAN]	34.26	1	2	2	3	1.045	0.768	0.959	1.573	9.07	34.26	2	3	108	11.9	9.70
P04004	Vitronectin OS=Homo sapiens GN=VTN PE=1 SV=1 - [VTNC_HUMAN]	7.32	1	3	3	11	1.124	0.943	1.228	1.573	27.71	7.32	4	11	478	54.3	5.80
P49321	Nuclear autoantigenic sperm protein OS=Homo sapiens GN=NASP PE=1 SV=2 - [NASP_HUMAN]	37.18	3	24	25	109	1.261	1.503	0.806	1.573	288.94	37.18	39	109	788	85.2	4.30
P46439	Glutathione S-transferase Mu 5 OS=Homo sapiens GN=GSTM5 PE=1 SV=3 - [GSTM5_HUMAN]	66.97	1	9	18	173	1.957	0.747	0.801	1.573	416.65	66.97	31	173	218	25.7	7.39
Q49A26	Putative oxidoreductase GLYR1 OS=Homo sapiens GN=GLYR1 PE=1 SV=3 - [GLYR1_HUMAN]	5.42	1	2	2	3	1.003	0.886	0.900	1.574	4.82	5.42	3	3	553	60.5	9.17
Q30201	Hereditary hemochromatosis protein OS=Homo sapiens GN=HFE PE=1 SV=1 - [HFE_HUMAN]	3.45	1	1	1	4	1.354	1.034	0.899	1.574	10.49	3.45	2	4	348	40.1	6.60
O75907	Diacylglycerol O-acyltransferase 1 OS=Homo sapiens GN=DGAT1 PE=1 SV=2 - [DGAT1_HUMAN]	3.89	1	1	1	2	1.453	1.343	1.074	1.574	4.23	3.89	2	2	488	55.2	9.32
P00750	Tissue-type plasminogen activator OS=Homo sapiens GN=PLAT PE=1 SV=1 - [TPA_HUMAN]	21.00	1	11	11	27	2.347	0.556	1.050	1.575	67.17	21.00	17	27	562	62.9	7.80

P00167	Cytochrome b5 OS=Homo sapiens GN=CYB5A PE=1 SV=2 - [CYB5_HUMAN]	47.01	1	5	5	28	1.651	0.743	0.874	1.575	77.95	47.01	9	28	134	15.3	4.96
Q8NFV4	Alpha/beta hydrolase domain-containing protein 11 OS=Homo sapiens GN=ABHD11 PE=2 SV=1 - [ABHDB_HUMAN]	16.83	1	5	5	10	1.043	1.369	1.445	1.575	12.96	16.83	7	10	315	34.7	9.48
Q8IUW5	RELT-like protein 1 OS=Homo sapiens GN=RELL1 PE=1 SV=1 - [RELL1_HUMAN]	9.23	1	2	2	13	1.695	0.914	0.538	1.576	30.33	9.23	4	13	271	29.3	8.34
Q9Y4B4	Helicase ARIP4 OS=Homo sapiens GN=RAD54L2 PE=1 SV=4 - [ARIP4_HUMAN]	2.86	1	4	4	4	1.167	1.367	0.864	1.577	3.85	2.86	4	4	1467	162.7	6.09
Q8IWT6	Volume-regulated anion channel subunit LRRC8A OS=Homo sapiens GN=LRRC8A PE=1 SV=1 - [LRC8A_HUMAN]	4.07	1	2	3	5	1.625	1.109	1.736	1.577	14.20	4.07	4	5	810	94.1	7.94
O15533	Tapasin OS=Homo sapiens GN=TAPBP PE=1 SV=1 - [TPSN_HUMAN]	16.96	1	4	4	8	1.987	1.037	0.985	1.578	26.62	16.96	6	8	448	47.6	7.15
P12236	ADP/ATP translocase 3 OS=Homo sapiens GN=SLC25A6 PE=1 SV=4 - [ADT3_HUMAN]	51.34	2	4	18	94	2.424	1.219	1.234	1.578	197.18	51.34	29	94	298	32.8	9.74
O60565	Gremlin-1 OS=Homo sapiens GN=GREM1 PE=1 SV=1 - [GREM1_HUMAN]	15.22	1	3	3	8	0.583	5.160	2.912	1.578	12.63	15.22	6	8	184	20.7	9.39
O14525	Astrotactin-1 OS=Homo sapiens GN=ASTN1 PE=2 SV=3 - [ASTN1_HUMAN]	1.69	1	2	2	3	2.547	1.064	4.331	1.579	3.79	1.69	2	3	1302	144.8	5.24

O95429	BAG family molecular chaperone regulator 4 OS=Homo sapiens GN=BAG4 PE=1 SV=1 - [BAG4_HUMAN]	14.44	1	3	4	22	1.453	0.858	1.241	1.580	70.77	14.44	5	22	457	49.6	5.12
P04424	Argininosuccinate lyase OS=Homo sapiens GN=ASL PE=1 SV=4 - [ARLY_HUMAN]	30.39	1	14	14	49	2.020	0.866	0.669	1.581	115.97	30.39	23	49	464	51.6	6.48
P53634	Dipeptidyl peptidase 1 OS=Homo sapiens GN=CTSC PE=1 SV=2 - [CATC_HUMAN]	31.32	1	10	10	69	1.518	0.528	0.680	1.581	247.65	31.32	14	69	463	51.8	6.99
Q05901	Neuronal acetylcholine receptor subunit beta-3 OS=Homo sapiens GN=CHRNB3 PE=2 SV=2 - [ACHB3_HUMAN]	2.84	1	1	1	1	1.236	1.334	1.003	1.581	0.00	2.84	1	1	458	52.7	8.10
Q6P179	Endoplasmic reticulum aminopeptidase 2 OS=Homo sapiens GN=ERAP2 PE=1 SV=2 - [ERAP2_HUMAN]	6.35	1	7	7	12	1.908	1.279	1.619	1.582	20.75	6.35	10	12	960	110.4	6.71
P35568	Insulin receptor substrate 1 OS=Homo sapiens GN=IRS1 PE=1 SV=1 - [IRS1_HUMAN]	10.63	1	9	9	21	1.844	1.633	0.975	1.583	64.11	10.63	12	21	1242	131.5	8.54
O00762	Ubiquitin-conjugating enzyme E2 C OS=Homo sapiens GN=UBE2C PE=1 SV=1 - [UBE2C_HUMAN]	6.15	1	1	1	1	1.004	0.920	0.819	1.583	2.17	6.15	1	1	179	19.6	7.37
Q13939	Calicin OS=Homo sapiens GN=CCIN PE=2 SV=3 - [CALI_HUMAN]	2.21	1	1	1	1	0.984	1.226	1.154	1.583	0.00	2.21	1	1	588	66.5	8.18
P0CW20	LIM and senescent cell antigen-like-containing domain protein 3-like OS=Homo sapiens GN=LIMS3L PE=3 SV=1 - [LIM3L_HUMAN]	25.64	1	2	2	17	0.737	1.059	1.180	1.584	43.26	25.64	4	17	117	13.2	5.49

P13984	General transcription factor IIF subunit 2 OS=Homo sapiens GN=GTF2F2 PE=1 SV=2 - [T2FB_HUMAN]	45.78	1	7	8	27	1.711	1.243	0.940	1.584	56.46	45.78	13	27	249	28.4	9.23
Q9Y4J8	Dystrobrevin alpha OS=Homo sapiens GN=DTNA PE=1 SV=2 - [DTNA_HUMAN]	9.42	1	3	6	12	0.750	3.613	1.141	1.587	26.23	9.42	8	12	743	83.8	6.89
Q96FN4	Copine-2 OS=Homo sapiens GN=CPNE2 PE=1 SV=3 - [CPNE2_HUMAN]	3.28	4	1	2	12	1.117	2.625	1.449	1.588	29.97	3.28	2	12	548	61.2	6.07
P50897	Palmitoyl-protein thioesterase 1 OS=Homo sapiens GN=PPT1 PE=1 SV=1 - [PPT1_HUMAN]	33.33	1	9	9	24	1.580	1.174	0.846	1.588	57.47	33.33	13	24	306	34.2	6.52
Q8TEQ0	Sorting nexin-29 OS=Homo sapiens GN=SNX29 PE=1 SV=3 - [SNX29_HUMAN]	13.90	2	9	10	18	1.302	0.843	0.937	1.589	33.65	13.90	11	18	813	91.2	6.21
Q9UNZ5	Leydig cell tumor 10 kDa protein homolog OS=Homo sapiens GN=C19orf53 PE=1 SV=1 - [L10K_HUMAN]	17.17	1	3	3	3	0.981	1.399	0.843	1.589	2.48	17.17	3	3	99	10.6	11.55
P06280	Alpha-galactosidase A OS=Homo sapiens GN=GLA PE=1 SV=1 - [AGAL_HUMAN]	15.85	1	6	6	22	1.346	0.817	0.967	1.590	54.80	15.85	8	22	429	48.7	5.60
Q4G0M1	Erythroferrone OS=Homo sapiens GN=FAM132B PE=2 SV=2 - [ERFE_HUMAN]	3.95	1	1	1	1	0.768	0.806	0.834	1.590	0.00	3.95	1	1	354	37.3	9.83
Q9UI47	Catenin alpha-3 OS=Homo sapiens GN=CTNNA3 PE=1 SV=2 - [CTNA3_HUMAN]	3.69	1	1	2	2	2.874	0.646	0.989	1.592	5.14	3.69	2	2	895	99.7	6.16

P20585	DNA mismatch repair protein Msh3 OS=Homo sapiens GN=MSH3 PE=1 SV=4 - [MSH3_HUMAN]	0.79	1	1	1	8	1.010	0.832	0.837	1.593	23.47	0.79	1	8	1137	127.3	8.02
Q9H875	PRKR-interacting protein 1 OS=Homo sapiens GN=PRKRIP1 PE=1 SV=1 - [PKRI1_HUMAN]	4.89	1	1	1	4	0.795	1.131	1.214	1.593	10.35	4.89	2	4	184	21.0	9.79
P07451	Carbonic anhydrase 3 OS=Homo sapiens GN=CA3 PE=1 SV=3 - [CAH3_HUMAN]	3.46	1	1	1	1	0.900	1.722	1.210	1.593	2.47	3.46	1	1	260	29.5	7.34
Q96SB8	Structural maintenance of chromosomes protein 6 OS=Homo sapiens GN=SMC6 PE=1 SV=2 - [SMC6_HUMAN]	2.29	1	3	3	7	1.118	1.047	1.063	1.593	11.90	2.29	3	7	1091	126.2	6.99
O95983	Methyl-CpG-binding domain protein 3 OS=Homo sapiens GN=MBD3 PE=1 SV=1 - [MBD3_HUMAN]	8.59	1	2	3	5	0.886	1.554	1.334	1.594	5.56	8.59	4	5	291	32.8	5.34
Q9NU22	Midasin OS=Homo sapiens GN=MDN1 PE=1 SV=2 - [MDN1_HUMAN]	0.55	1	3	4	9	0.824	1.063	1.048	1.594	17.89	0.55	6	9	5596	632.4	5.68
Q8WVX9	Fatty acyl-CoA reductase 1 OS=Homo sapiens GN=FAR1 PE=1 SV=1 - [FACR1_HUMAN]	4.08	1	2	2	4	0.772	0.847	1.113	1.595	6.01	4.08	3	4	515	59.3	9.17
P35270	Sepiapterin reductase OS=Homo sapiens GN=SPR PE=1 SV=1 - [SPRE_HUMAN]	33.72	1	8	8	29	1.670	0.866	1.071	1.596	65.50	33.72	13	29	261	28.0	8.05
O00522	Krev interaction trapped protein 1 OS=Homo sapiens GN=KRIT1 PE=1 SV=2 -	2.17	1	2	2	2	0.929	1.165	1.161	1.596	3.95	2.17	2	2	736	84.3	8.41

Q9Y5J7	Mitochondrial import inner membrane translocase subunit Tim9 OS=Homo sapiens GN=TIMM9 PE=1 SV=1 - [TIM9_HUMAN]	29.21	1	3	3	11	1.252	1.351	1.488	1.597	29.76	29.21	5	11	89	10.4	7.21
P98173	Protein FAM3A OS=Homo sapiens GN=FAM3A PE=1 SV=2 - [FAM3A_HUMAN]	3.48	1	1	1	1	1.249	1.283	1.262	1.597	2.37	3.48	1	1	230	25.1	8.68
Q9Y6R1	Electrogenic sodium bicarbonate cotransporter 1 OS=Homo sapiens GN=SLC4A4 PE=1 SV=1 - [S4A4_HUMAN]	4.91	2	4	5	7	2.067	0.186	0.518	1.597	19.25	4.91	6	7	1079	121.4	6.80
Q8TDI7	Transmembrane channel-like protein 2 OS=Homo sapiens GN=TMC2 PE=2 SV=3 - [TMC2_HUMAN]	0.88	1	1	1	5	0.927	1.303	0.662	1.598	8.24	0.88	1	5	906	102.5	9.47
O43505	Beta-1,4-glucuronyltransferase 1 OS=Homo sapiens GN=B3GNT1 PE=1 SV=1 - [B4GA1_HUMAN]	3.86	1	1	1	5	1.158	1.062	0.900	1.599	10.96	3.86	2	5	415	47.1	7.20
P82970	High mobility group nucleosome-binding domain-containing protein 5 OS=Homo sapiens GN=HMGN5 PE=1 SV=1 - [HMGN5_HUMAN]	3.19	1	1	1	1	0.705	1.286	1.334	1.600	2.72	3.19	1	1	282	31.5	4.55
O00124	UBX domain-containing protein 8 OS=Homo sapiens GN=UBXN8 PE=1 SV=2 - [UBXN8_HUMAN]	6.30	1	1	1	2				1.606	8.57	6.30	1	2	270	30.5	7.78
Q8N655	Uncharacterized protein C10orf12 OS=Homo sapiens GN=C10orf12 PE=1 SV=1 - [CJ012_HUMAN]	2.17	1	3	3	3	1.084	0.603	0.908	1.606	6.08	2.17	3	3	1247	137.1	8.24

P30533	Alpha-2-macroglobulin receptor-associated protein OS=Homo sapiens GN=LRPAP1 PE=1 SV=1 - [AMRP_HUMAN]	48.18	2	25	26	152	1.331	1.133	1.221	1.606	416.54	48.18	38	152	357	41.4	8.78
O75626	PR domain zinc finger protein 1 OS=Homo sapiens GN=PRDM1 PE=1 SV=2 - [PRDM1_HUMAN]	2.18	1	2	2	2	1.211	1.565	1.553	1.609	2.05	2.18	2	2	825	91.7	8.48
P10914	Interferon regulatory factor 1 OS=Homo sapiens GN=IRF1 PE=1 SV=2 - [IRF1_HUMAN]	10.15	1	2	2	4	1.085	1.339	1.073	1.610	5.03	10.15	2	4	325	36.5	5.39
Q9UNQ2	Probable dimethyladenosine transferase OS=Homo sapiens GN=DIMT1 PE=1 SV=1 - [DIM1_HUMAN]	2.24	1	1	1	1	0.722	0.703	0.507	1.610	2.52	2.24	1	1	313	35.2	9.99
O60658	High affinity cAMP-specific and IBMX-insensitive 3',5'-cyclic phosphodiesterase 8A OS=Homo sapiens GN=PDE8A PE=1 SV=2 - [PDE8A_HUMAN]	2.65	1	2	2	3	2.338	1.429	1.147	1.610	2.04	2.65	3	3	829	93.2	6.11
P54284	Voltage-dependent L-type calcium channel subunit beta-3 OS=Homo sapiens GN=CACNB3 PE=1 SV=1 - [CACB3_HUMAN]	14.88	3	4	6	22	1.715	1.477	1.126	1.612	56.83	14.88	9	22	484	54.5	6.35
P15559	NAD(P)H dehydrogenase [quinone] 1 OS=Homo sapiens GN=NQO1 PE=1 SV=1 - [NQO1_HUMAN]	45.26	1	15	15	254	1.792	0.537	0.796	1.612	693.17	45.26	26	254	274	30.8	8.88

P31321	cAMP-dependent protein kinase type I-beta regulatory subunit OS=Homo sapiens GN=PRKAR1B PE=1 SV=4 - [KAP1_HUMAN]	22.05	1	4	7	14	1.981	0.652	0.865	1.612	38.93	22.05	8	14	381	43.0	5.71
Q7Z4T9	Protein MAATS1 OS=Homo sapiens GN=MAATS1 PE=1 SV=2 - [MAAT1_HUMAN]	2.32	1	1	1	1	1.362	0.998	1.194	1.613	2.67	2.32	1	1	603	71.0	7.74
Q5QJE6	Deoxynucleotidyltransferase terminal-interacting protein 2 OS=Homo sapiens GN=DNTTIP2 PE=1 SV=2 - [TDIF2_HUMAN]	8.47	2	4	5	21	0.875	1.184	0.829	1.613	47.32	8.47	8	21	756	84.4	6.16
Q6BCY4	NADH-cytochrome b5 reductase 2 OS=Homo sapiens GN=CYB5R2 PE=1 SV=1 - [NB5R2_HUMAN]	32.97	1	7	8	19	0.823	0.480	0.929	1.613	24.20	32.97	13	19	276	31.4	8.50
P69905	Hemoglobin subunit alpha OS=Homo sapiens GN=HBA1 PE=1 SV=2 - [HBA_HUMAN]	21.83	1	2	2	6	1.415	0.677	0.809	1.614	7.08	21.83	3	6	142	15.2	8.68
O95801	Tetratricopeptide repeat protein 4 OS=Homo sapiens GN=TTC4 PE=1 SV=3 - [TTC4_HUMAN]	2.58	1	1	1	3	0.736	1.129	1.486	1.615	4.10	2.58	2	3	387	44.7	5.60
P22607	Fibroblast growth factor receptor 3 OS=Homo sapiens GN=FGFR3 PE=1 SV=1 - [FGFR3_HUMAN]	6.08	7	1	3	13	1.045	1.043	0.868	1.616	22.65	6.08	4	13	806	87.7	5.90
P38435	Vitamin K-dependent gamma-carboxylase OS=Homo sapiens GN=GGCX PE=1 SV=2 - [VKGC_HUMAN]	9.50	1	7	7	22	1.243	0.782	0.735	1.616	56.13	9.50	12	22	758	87.5	8.02

Q6ZN66	Guanylate-binding protein 6 OS=Homo sapiens GN=GBP6 PE=2 SV=1 - [GBP6_HUMAN]	3.79	1	1	2	2	2.329	0.403	0.857	1.617	2.59	3.79	2	2	633	72.4	6.37
P12004	Proliferating cell nuclear antigen OS=Homo sapiens GN=PCNA PE=1 SV=1 - [PCNA_HUMAN]	40.61	1	9	9	37	1.169	1.131	0.886	1.619	70.43	40.61	14	37	261	28.8	4.69
Q9H0E9	Bromodomain-containing protein 8 OS=Homo sapiens GN=BRD8 PE=1 SV=2 - [BRD8_HUMAN]	2.35	1	2	3	5	1.523	1.107	1.230	1.619	9.86	2.35	3	5	1235	135.3	4.60
P50613	Cyclin-dependent kinase 7 OS=Homo sapiens GN=CDK7 PE=1 SV=1 - [CDK7_HUMAN]	7.23	1	2	3	6	1.327	1.200	1.093	1.619	10.14	7.23	5	6	346	39.0	8.47
Q8NFP4	MAM domain-containing glycosylphosphatidylinositol anchor protein 1 OS=Homo sapiens GN=MDGA1 PE=1 SV=1 - [MDGA1_HUMAN]	1.15	1	1	1	1	0.910		2.514	1.621	0.00	1.15	1	1	955	105.7	8.34
Q96RE7	Nucleus accumbens-associated protein 1 OS=Homo sapiens GN=NACC1 PE=1 SV=1 - [NACC1_HUMAN]	9.11	1	3	3	8	1.034	0.833	0.959	1.621	16.42	9.11	4	8	527	57.2	5.74
Q9UIG0	Tyrosine-protein kinase BAZ1B OS=Homo sapiens GN=BAZ1B PE=1 SV=2 - [BAZ1B_HUMAN]	6.54	1	11	12	36	1.168	1.343	0.899	1.621	80.30	6.54	19	36	1483	170.8	8.48
Q8WUY1	Protein THEM6 OS=Homo sapiens GN=THEM6 PE=1 SV=2 - [THEM6_HUMAN]	14.42	1	3	3	7	1.602	0.829	0.574	1.622	18.12	14.42	4	7	208	23.8	9.55
O14763	Tumor necrosis factor receptor superfamily member 10B OS=Homo sapiens GN=TNFRSF10B PE=1 SV=2 - [TR10B_HUMAN]	3.41	1	1	1	3	1.663	1.583	1.644	1.623	3.15	3.41	2	3	440	47.8	5.55

P52823	Stanniocalcin-1 OS=Homo sapiens GN=STC1 PE=1 SV=1 - [STC1_HUMAN]	5.67	1	1	1	1	2.593	0.695	0.957	1.624	4.77	5.67	1	1	247	27.6	7.99
Q63ZY6	Putative methyltransferase NSUN5C OS=Homo sapiens GN=NSUN5P2 PE=5 SV=2 - [NSN5C_HUMAN]	11.75	2	2	3	4	0.908	1.382	1.372	1.627	9.97	11.75	4	4	315	34.3	8.68
Q13099	Intraflagellar transport protein 88 homolog OS=Homo sapiens GN=IFT88 PE=2 SV=2 - [IFT88_HUMAN]	1.80	1	1	2	2	1.034	2.896	4.823	1.627	2.47	1.80	2	2	833	94.2	6.54
O14526	FCH domain only protein 1 OS=Homo sapiens GN=FCHO1 PE=1 SV=2 - [FCHO1_HUMAN]	3.37	2	1	4	8	2.140	2.142	2.740	1.629	10.47	3.37	4	8	889	96.8	6.96
Q9H0R6	Glutamyl-tRNA(Gln) amidotransferase subunit A, mitochondrial OS=Homo sapiens GN=QRSL1 PE=1 SV=2 - [GATA_HUMAN]	5.49	1	2	2	8	0.658	1.311	1.010	1.630	22.13	5.49	4	8	528	57.4	5.68
Q5T7V8	RAB6-interacting golgin OS=Homo sapiens GN=GORAB PE=1 SV=1 - [GORAB_HUMAN]	4.82	1	2	2	4	1.286	0.973	0.924	1.632	7.50	4.82	2	4	394	45.0	7.08
Q5VUA4	Zinc finger protein 318 OS=Homo sapiens GN=ZNF318 PE=1 SV=2 - [ZN318_HUMAN]	1.10	1	2	2	2	1.291	1.443	1.326	1.634	2.20	1.10	2	2	2279	251.0	7.20
P36955	Pigment epithelium-derived factor OS=Homo sapiens GN=SERPINF1 PE=1 SV=4 - [PEDF_HUMAN]	2.39	1	1	1	4	1.047	2.070	0.728	1.635	9.71	2.39	2	4	418	46.3	6.38
O60938	Keratocan OS=Homo sapiens GN=KERA PE=1 SV=1 - [KERA_HUMAN]	2.56	1	1	1	1	1.526	2.305	1.422	1.635	2.48	2.56	1	1	352	40.5	7.47

P49790	Nudear pore complex protein Nup153 OS=Homo sapiens GN=NUP153 PE=1 SV=2 - [NU153_HUMAN	16.00	1	19	19	45	1.156	1.185	0.988	1.635	107.97	16.00	29	45	1475	153.8	8.73
O95696	Bromodomain-containing protein 1 OS=Homo sapiens GN=BRD1 PE=1 SV=1 - [BRD1_HUMAN]	1.89	4	1	3	3	1.259	1.325	1.738	1.635	2.09	1.89	3	3	1058	119.4	8.60
Q9BX82	Zinc finger protein 471 OS=Homo sapiens GN=ZNF471 PE=2 SV=1 - [ZN471_HUMAN]	6.39	94	1	2	3	0.375	0.751	1.819	1.636	0.00	6.39	2	3	626	73.0	8.56
Q8WV22	Non-structural maintenance of chromosomes element 1 homolog OS=Homo sapiens GN=NSMCE1 PE=1 SV=5 - [NSE1_HUMAN]	9.02	1	3	3	3	0.998	1.117	1.128	1.638	5.86	9.02	3	3	266	30.8	7.47
O00463	TNF receptor-associated factor 5 OS=Homo sapiens GN=TRAF5 PE=1 SV=2 - [TRAF5_HUMAN	2.15	1	1	1	1	1.572	0.915	1.088	1.639	3.18	2.15	1	1	557	64.4	7.43
Q93096	Protein tyrosine phosphatase type IVA 1 OS=Homo sapiens GN=PTP4A1 PE=1 SV=2 - [TP4A1_HUMAN	23.70	1	1	4	18	1.092	0.761	1.103	1.639	25.36	23.70	7	18	173	19.8	8.97
Q15723	ETS-related transcription factor Elf-2 OS=Homo sapiens GN=ELF2 PE=1 SV=2 - [ELF2_HUMAN]	3.20	3	2	2	2	1.092	1.442	1.059	1.640	2.51	3.20	2	2	593	63.9	6.57
P07332	Tyrosine-protein kinase Fes/Fps OS=Homo sapiens GN=FES PE=1 SV=3 - [FES_HUMAN]	2.31	1	1	1	1	1.739	1.811	1.203	1.644	0.00	2.31	1	1	822	93.4	6.73
Q9UDX5	Mitochondrial fission process protein 1 OS=Homo sapiens GN=MTFP1 PE=1 SV=1 - [MTFP1_HUMA N]	7.23	1	1	1	2	1.253	1.216	0.507	1.646	0.00	7.23	2	2	166	18.0	9.31

Q6UXI7	Vitrin OS=Homo sapiens GN=VIT PE=2 SV=1 - [VITRN_HUMAN]	2.21	1	1	1	1	1.460	0.782	0.636	1.646	0.00	2.21	1	1	678	73.9	9.20
Q8WUD4	Coiled-coil domain-containing protein 12 OS=Homo sapiens GN=CCDC12 PE=1 SV=1 - [CCD12_HUMAN]	30.72	1	2	3	3	1.383	3.568	1.022	1.647	8.29	30.72	3	3	166	19.2	7.34
Q13751	Laminin subunit beta-3 OS=Homo sapiens GN=LAMB3 PE=1 SV=1 - [LAMB3_HUMAN]	2.82	1	2	2	3	1.311	1.178	1.654	1.648	0.00	2.82	2	3	1172	129.5	7.21
Q9BTE3	Mini-chromosome maintenance complex-binding protein OS=Homo sapiens GN=MCMBP PE=1 SV=2 - [MCMBP_HUMAN]	10.59	1	6	6	16	0.976	1.482	1.176	1.650	28.32	10.59	11	16	642	72.9	5.87
Q8N6T7	NAD-dependent protein deacetylase sirtuin-6 OS=Homo sapiens GN=SIRT6 PE=1 SV=2 - [SIR6_HUMAN]	6.48	1	2	2	3	0.598	0.863	0.854	1.651	8.08	6.48	2	3	355	39.1	9.19
P20719	Homeobox protein Hox-A5 OS=Homo sapiens GN=HOXA5 PE=1 SV=2 - [HXA5_HUMAN]	9.26	1	1	1	1	2.386	0.571		1.651	2.41	9.26	1	1	270	29.3	9.25
Q7Z6I8	UPF0461 protein C5orf24 OS=Homo sapiens GN=C5orf24 PE=1 SV=1 - [CE024_HUMAN]	5.85	1	1	1	1	2.039	1.457	1.524	1.654	0.00	5.85	1	1	188	20.1	9.85
Q03181	Peroxisome proliferator-activated receptor delta OS=Homo sapiens GN=PPARD PE=1 SV=1 - [PPARD_HUMAN]	1.59	1	1	1	8	1.412	0.973	1.194	1.654	22.00	1.59	2	8	441	49.9	7.59
Q8IXS6	Paralemmin-2 OS=Homo sapiens GN=PALM2 PE=2 SV=3 - [PALM2_HUMAN]	34.30	1	12	12	41	1.198	1.008	1.393	1.655	73.65	34.30	22	41	379	42.2	5.10

Q8NA31	Telomere repeats-binding bouquet formation protein 1 OS=Homo sapiens GN=CCDC79 PE=2 SV=3 - [TERB1_HUMAN	1.65	1	1	1	2		1.623	0.906	1.657	2.89	1.65	1	2	727	83.0	7.46
Q9ULE3	DENN domain-containing protein 2A OS=Homo sapiens GN=DENND2A PE=2 SV=4 - [DEN2A_HUMAN]	17.94	2	13	17	51	2.596	0.886	0.604	1.658	141.61	17.94	23	51	1009	113.8	8.95
Q15599	Na(+)/H(+) exchange regulatory cofactor NHE-RF2 OS=Homo sapiens GN=SLC9A3R2 PE=1 SV=2 - [NHRF2_HUMAN]	43.92	1	13	13	40	1.764	0.692	0.949	1.658	91.17	43.92	22	40	337	37.4	7.93
P43246	DNA mismatch repair protein Msh2 OS=Homo sapiens GN=MSH2 PE=1 SV=1 - [MSH2_HUMAN]	12.31	1	10	11	23	1.326	1.171	0.854	1.659	51.57	12.31	19	23	934	104.7	5.77
Q96HP0	Dedicator of cytokinesis protein 6 OS=Homo sapiens GN=DOCK6 PE=1 SV=3 - [DOCK6_HUMA N]	7.62	1	10	13	33	1.057	1.113	1.362	1.660	96.64	7.62	17	33	2047	229.4	6.74
P07711	Cathepsin L1 OS=Homo sapiens GN=CTSL PE=1 SV=2 - [CATL1_HUMAN]	20.72	2	7	8	28	0.756	0.810	0.801	1.660	74.30	20.72	14	28	333	37.5	5.45
Q9UIL8	PHD finger protein 11 OS=Homo sapiens GN=PHF11 PE=1 SV=3 - [PHF11_HUMAN]	3.63	1	1	1	1	1.288	1.420	1.513	1.660	2.53	3.63	1	1	331	37.6	7.72
P33316	Deoxyuridine 5'-triphosphate nucleotidohydrolase, mitochondrial OS=Homo sapiens GN=DUT PE=1 SV=4 - [DUT_HUMAN]	21.43	1	4	4	33	1.246	1.441	1.077	1.662	91.19	21.43	7	33	252	26.5	9.36

Q6XQN6	Nicotinate phosphoribosyltransferase OS=Homo sapiens GN=NAPRT PE=1 SV=2 - [PNCB_HUMAN]	12.64	1	6	6	18	2.132	1.429	1.664	1.663	38.72	12.64	10	18	538	57.5	5.68
Q9UBP6	tRNA (guanine-N(7)-)-methyltransferase OS=Homo sapiens GN=METTL1 PE=1 SV=1 - [TRMB_HUMAN]	7.61	1	2	2	8	1.320	1.595	1.221	1.663	28.08	7.61	3	8	276	31.5	7.64
Q9NVV4	Poly(A) RNA polymerase, mitochondrial OS=Homo sapiens GN=MTPAP PE=1 SV=1 - [PAPD1_HUMAN]	5.15	1	2	3	6	0.941	1.341	0.883	1.664	13.18	5.15	4	6	582	66.1	9.04
Q8NI35	InaD-like protein OS=Homo sapiens GN=INADL PE=1 SV=3 - [INADL_HUMAN]	1.33	1	2	3	6	1.268	0.693	0.846	1.665	13.31	1.33	3	6	1801	196.2	4.94
Q96EA4	Protein Spindly OS=Homo sapiens GN=SPDL1 PE=1 SV=2 - [SPDLY_HUMAN]	5.12	1	2	3	3	1.103	0.618	0.609	1.665	7.33	5.12	3	3	605	70.1	5.47
Q6UW68	Transmembrane protein 205 OS=Homo sapiens GN=TMEM205 PE=1 SV=1 - [TM205_HUMAN]	15.87	1	2	2	6	1.385	1.460	1.066	1.667	19.70	15.87	3	6	189	21.2	8.62
O75717	WD repeat and HMG-box DNA-binding protein 1 OS=Homo sapiens GN=WDHD1 PE=1 SV=1 - [WDHD1_HUMAN]	1.15	1	1	1	2			1.117	1.672	7.01	1.15	1	2	1129	125.9	5.62
O43824	Putative GTP-binding protein 6 OS=Homo sapiens GN=GTPBP6 PE=2 SV=3 - [GTPB6_HUMAN]	2.13	1	1	1	4	0.924	1.592	1.247	1.673	7.42	2.13	2	4	516	56.8	9.42
P78316	Nucleolar protein 14 OS=Homo sapiens GN=NOP14 PE=1 SV=3 - [NOP14_HUMAN]	7.47	1	6	6	8	0.995	1.635	1.350	1.674	20.92	7.47	6	8	857	97.6	7.58

P40306	Proteasome subunit beta type-10 OS=Homo sapiens GN=PSMB10 PE=1 SV=1 - [PSB10_HUMAN]	33.70	1	6	6	23	2.274	1.136	0.729	1.676	49.21	33.70	9	23	273	28.9	7.81
P37287	Phosphatidylinositol N-acetylglucosaminyltransferase subunit A OS=Homo sapiens GN=PIGA PE=1 SV=1 - [PIGA_HUMAN]	4.75	1	2	2	2	1.827	1.799	2.351	1.676	5.63	4.75	2	2	484	54.1	8.34
P61077	Ubiquitin-conjugating enzyme E2 D3 OS=Homo sapiens GN=UBE2D3 PE=1 SV=1 - [UB2D3_HUMAN]	6.80	1	1	2	17	3.798	1.740	1.861	1.677	31.37	6.80	4	17	147	16.7	7.80
O15211	Ral guanine nucleotide dissociation stimulator-like 2 OS=Homo sapiens GN=RGL2 PE=1 SV=1 - [RGL2_HUMAN]	4.38	1	2	3	7	1.935	2.197	2.474	1.679	8.95	4.38	5	7	777	83.5	6.18
Q9UNY4	Transcription termination factor 2 OS=Homo sapiens GN=TTF2 PE=1 SV=2 - [TTF2_HUMAN]	1.03	1	1	1	2	1.047	0.981	1.087	1.682	5.20	1.03	2	2	1162	129.5	8.37
P33991	DNA replication licensing factor MCM4 OS=Homo sapiens GN=MCM4 PE=1 SV=5 - [MCM4_HUMAN]	8.81	1	7	7	16	1.351	1.332	0.598	1.682	36.44	8.81	10	16	863	96.5	6.74
Q9H0V1	Transmembrane protein 168 OS=Homo sapiens GN=TMEM168 PE=1 SV=2 - [TM168_HUMAN]	2.01	1	1	1	1	1.198	1.198	1.331	1.682	2.65	2.01	1	1	697	79.7	8.03
P30481	HLA class I histocompatibility antigen, B-44 alpha chain OS=Homo sapiens GN=HLA-B PE=1 SV=1 - [1B44_HUMAN]	35.91	2	3	13	83	0.598	1.266	1.276	1.684	184.25	35.91	20	83	362	40.5	6.04

Q9NZ63	Uncharacterized protein C9orf78 OS=Homo sapiens GN=C9orf78 PE=1 SV=1 - [CI078_HUMAN]	2.77	1	1	1	3	1.065	1.502	1.264	1.685	6.91	2.77	2	3	289	33.7	6.74
Q03518	Antigen peptide transporter 1 OS=Homo sapiens GN=TAP1 PE=1 SV=2 - [TAP1_HUMAN]	11.14	1	6	6	12	1.776	0.795	0.865	1.685	41.40	11.14	8	12	808	87.2	8.02
P30459	HLA class I histocompatibility antigen, A-74 alpha chain OS=Homo sapiens GN=HLA-A PE=1 SV=1 - [1A74_HUMAN]	48.22	3	1	16	138	0.975	0.985	1.696	1.686	318.57	48.22	26	138	365	40.9	6.47
Q9UFC0	Leucine-rich repeat and WD repeat-containing protein 1 OS=Homo sapiens GN=LRWD1 PE=1 SV=2 - [LRWD1_HUMAN]	3.55	1	2	2	3	1.107	1.401	0.903	1.687	7.51	3.55	2	3	647	70.8	7.21
Q13490	Baculoviral IAP repeat-containing protein 2 OS=Homo sapiens GN=BIRC2 PE=1 SV=2 - [BIRC2_HUMAN]	6.31	1	3	3	3	1.298	1.151	1.047	1.688	6.03	6.31	3	3	618	69.9	6.70
Q96BZ8	Leukocyte receptor cluster member 1 OS=Homo sapiens GN=LENG1 PE=1 SV=1 - [LENG1_HUMAN]	2.65	1	1	1	2	0.808	0.596	0.573	1.688	4.69	2.65	1	2	264	30.5	9.69
P60033	CD81 antigen OS=Homo sapiens GN=CD81 PE=1 SV=1 - [CD81_HUMAN]	8.47	1	1	1	5	1.393	1.547	1.183	1.691	17.11	8.47	1	5	236	25.8	5.29
Q9NNW5	WD repeat-containing protein 6 OS=Homo sapiens GN=WDR6 PE=1 SV=1 - [WDR6_HUMAN]	0.89	1	1	1	1	0.994	1.659	1.344	1.691	2.19	0.89	1	1	1121	121.6	6.87

Q9H6E4	Coiled-coil domain-containing protein 134 OS=Homo sapiens GN=CCDC134 PE=1 SV=1 - [CC134_HUMAN]	20.52	1	3	3	4	1.519	1.494	1.530	1.692	9.42	20.52	4	4	229	26.5	8.85
Q9HBG6	Intraflagellar transport protein 122 homolog OS=Homo sapiens GN=IFT122 PE=1 SV=2 - [IF122_HUMAN]	1.13	1	1	2	4	4.115	7.157	4.636	1.693	3.85	1.13	2	4	1241	141.7	6.49
Q9BXN1	Asporin OS=Homo sapiens GN=ASPN PE=1 SV=2 - [ASPN_HUMAN]	1.58	1	1	1	1	1.304	1.909	1.256	1.696	1.71	1.58	1	1	380	43.4	7.08
Q7Z7H8	39S ribosomal protein L10, mitochondrial OS=Homo sapiens GN=MRPL10 PE=1 SV=3 - [RM10_HUMAN]	11.49	1	2	2	5	1.154	1.226	1.395	1.698	13.00	11.49	3	5	261	29.3	9.58
Q13370	cGMP-inhibited 3',5'-cyclic phosphodiesterase B OS=Homo sapiens GN=PDE3B PE=1 SV=2 - [PDE3B_HUMAN]	0.72	1	1	1	1	1.012	2.072	3.154	1.699	1.95	0.72	1	1	1112	124.3	5.91
P49418	Amphiphysin OS=Homo sapiens GN=AMPH PE=1 SV=1 - [AMPH_HUMAN]	16.40	1	10	10	24	0.462	0.856	1.361	1.699	68.55	16.40	14	24	695	76.2	4.64
P51688	N-sulphoglucosamine sulphohydrolase OS=Homo sapiens GN=SGSH PE=1 SV=1 - [SPHM_HUMAN]	19.72	1	8	8	25	1.057	0.858	1.065	1.700	59.07	19.72	12	25	502	56.7	6.95
P16220	Cyclic AMP-responsive element-binding protein 1 OS=Homo sapiens GN=CREB1 PE=1 SV=2 - [CREB1_HUMAN]	9.68	1	2	3	8	1.981	2.317	1.556	1.705	13.28	9.68	6	8	341	36.7	5.57
P52789	Hexokinase-2 OS=Homo sapiens GN=HK2 PE=1 SV=2 - [HXK2_HUMAN]	41.77	1	33	37	159	2.334	0.488	0.867	1.705	413.55	41.77	61	159	917	102.3	6.05

O15091	Mitochondrial ribonuclease P protein 3 OS=Homo sapiens GN=KIAA0391 PE=1 SV=2 - [MRRP3_HUMAN]	3.60	1	2	2	3	1.736	1.883	1.395	1.705	10.77	3.60	2	3	583	67.3	8.78
Q96RL1	BRCA1-A complex subunit RAP80 OS=Homo sapiens GN=UIMC1 PE=1 SV=2 - [UIMC1_HUMAN]	0.97	1	1	1	1	1.670	1.373	1.066	1.712	0.00	0.97	1	1	719	79.7	5.45
Q9BWG4	Single-stranded DNA-binding protein 4 OS=Homo sapiens GN=SSBP4 PE=1 SV=1 - [SSBP4_HUMAN]	4.42	1	1	1	1	0.907	2.210	1.311	1.712	4.33	4.42	1	1	385	39.4	6.80
Q11201	CMP-N-acetylneuraminate-beta-galactosamide-alpha-2,3-sialyltransferase 1 OS=Homo sapiens GN=ST3GAL1 PE=2 SV=1 - [SIA4A_HUMAN]	9.71	1	3	3	7	0.930	0.568	0.491	1.712	10.71	9.71	4	7	340	39.1	9.09
A5PL33	Protein KRBA1 OS=Homo sapiens GN=KRBA1 PE=1 SV=3 - [KRBA1_HUMAN]	1.84	1	1	1	2	1.434	1.005	0.798	1.712	4.67	1.84	1	2	1030	107.4	7.83
P43304	Glycerol-3-phosphate dehydrogenase, mitochondrial OS=Homo sapiens GN=GPD2 PE=1 SV=3 - [GPDM_HUMAN]	35.21	1	25	25	66	2.246	0.906	0.757	1.715	174.99	35.21	37	66	727	80.8	7.69
Q9NZQ7	Programmed cell death 1 ligand 1 OS=Homo sapiens GN=CD274 PE=1 SV=1 - [PD1L1_HUMAN]	2.41	1	1	1	2	5.189	0.701	0.629	1.716	1.99	2.41	2	2	290	33.3	7.23
O94813	Slit homolog 2 protein OS=Homo sapiens GN=SLIT2 PE=1 SV=1 - [SLIT2_HUMAN]	2.88	1	2	3	6	2.722	3.067	4.977	1.717	4.65	2.88	3	6	1529	169.8	7.06

Q8IY67	Ribonucleoprotein PTB-binding 1 OS=Homo sapiens GN=RAVER1 PE=1 SV=1 - [RAVR1_HUMAN]	1.82	1	1	1	4	1.454	1.559	0.886	1.718	4.83	1.82	2	4	606	63.8	8.48
Q8NBZ7	UDP-glucuronic acid decarboxylase 1 OS=Homo sapiens GN=UXS1 PE=1 SV=1 - [UXS1_HUMAN]	5.71	1	2	2	3	2.299	1.272	1.618	1.719	3.13	5.71	3	3	420	47.5	8.94
P60059	Protein transport protein Sec61 subunit gamma OS=Homo sapiens GN=SEC61G PE=1 SV=1 - [SC61G_HUMAN]	11.76	1	1	1	1	1.269	2.521	3.107	1.721	0.00	11.76	1	1	68	7.7	9.99
Q96P11	Probable 28S rRNA (cytosine-C(5))-methyltransferase OS=Homo sapiens GN=NSUN5 PE=1 SV=2 - [NSUN5_HUMAN]	4.66	1	1	2	4	0.848	1.083	1.046	1.722	7.66	4.66	4	4	429	46.7	8.62
O15479	Melanoma-associated antigen B2 OS=Homo sapiens GN=MAGEB2 PE=1 SV=3 - [MAGB2_HUMAN]	2.51	1	1	1	1	1.750	0.797	1.181	1.722	2.42	2.51	1	1	319	35.3	8.76
Q52LD8	Raftlin-2 OS=Homo sapiens GN=RFTN2 PE=2 SV=3 - [RFTN2_HUMAN]	2.99	1	1	1	2	1.287	0.387	0.721	1.725	4.81	2.99	2	2	501	55.9	5.83
P06727	Apolipoprotein A-IV OS=Homo sapiens GN=APOA4 PE=1 SV=3 - [APOA4_HUMAN]	4.55	1	1	2	5	1.058	0.915	1.178	1.727	12.61	4.55	3	5	396	45.4	5.38
Q6ZRR7	Leucine-rich repeat-containing protein 9 OS=Homo sapiens GN=LRRC9 PE=2 SV=2 - [LRRC9_HUMAN]	2.20	2	2	3	12	2.288	2.320	1.570	1.729	22.95	2.20	4	12	1453	166.8	7.65
Q5VTB9	E3 ubiquitin-protein ligase RNF220 OS=Homo sapiens GN=RNF220 PE=1 SV=1 - [RN220_HUMAN]	2.47	1	1	1	1	1.496	1.702	1.158	1.730	4.10	2.47	1	1	566	62.7	6.04

Q9P217	Zinc finger SWIM domain-containing protein 5 OS=Homo sapiens GN=ZSWIM5 PE=2 SV=2 - [ZSWM5_HUMAN]	3.38	1	2	2	2	1.024	1.063	1.275	1.731	5.35	3.38	2	2	1185	130.6	7.18
Q8WVM0	Dimethyladenosine transferase 1, mitochondrial OS=Homo sapiens GN=TFB1M PE=1 SV=1 - [TFB1M_HUMA	5.78	1	1	2	3	1.280	0.900	1.215	1.733	2.35	5.78	3	3	346	39.5	9.26
Q8IWI9	MAX gene-associated protein OS=Homo sapiens GN=MGA PE=1 SV=3 - [MGAP_HUMAN]	0.93	1	2	2	2	2.183	0.672	1.202	1.734	3.33	0.93	2	2	3026	331.6	6.79
Q13634	Cadherin-18 OS=Homo sapiens GN=CDH18 PE=2 SV=1 - [CAD18_HUMAN]	6.96	1	3	3	3	0.924	1.243	0.860	1.734	2.62	6.96	3	3	790	88.0	5.14
Q5T9L3	Protein wntless homolog OS=Homo sapiens GN=WLS PE=1 SV=2 - [WLS_HUMAN]	11.28	1	9	9	35	1.510	0.934	0.967	1.736	81.94	11.28	16	35	541	62.2	7.36
Q12797	Aspartyl/asparaginyl beta-hydroxylase OS=Homo sapiens GN=ASPH PE=1 SV=3 - [ASPH_HUMAN]	39.71	1	33	33	333	1.337	0.649	0.855	1.736	854.94	39.71	54	333	758	85.8	5.01
Q09472	Histone acetyltransferase p300 OS=Homo sapiens GN=EP300 PE=1 SV=2 - [EP300_HUMAN]	2.03	3	4	5	6	1.436	1.910	0.865	1.738	10.49	2.03	6	6	2414	264.0	8.50
A6NFQ2	Protein FAM115C OS=Homo sapiens GN=FAM115C PE=2 SV=2 - [F115C_HUMAN]	2.18	1	2	2	5	1.842	1.065	1.639	1.738	10.64	2.18	3	5	919	100.8	7.17
Q96NB3	Zinc finger protein 830 OS=Homo sapiens GN=ZNF830 PE=1 SV=2 - [ZN830_HUMAN]	4.03	1	1	2	3	1.603	1.315	1.453	1.738	5.46	4.03	2	3	372	42.0	5.31

Q96MC5	Uncharacterized protein C16orf45 OS=Homo sapiens GN=C16orf45 PE=2 SV=1 - [CP045_HUMAN]	5.39	1	1	1	5	0.730	1.239	1.130	1.738	20.37	5.39	1	5	204	23.7	6.16
Q8TB37	Iron-sulfur protein NUBPL OS=Homo sapiens GN=NUBPL PE=1 SV=3 - [NUBPL_HUMAN]	5.02	1	2	2	4	1.083	1.880	1.336	1.739	5.80	5.02	3	4	319	34.1	9.04
Q04725	Transducin-like enhancer protein 2 OS=Homo sapiens GN=TLE2 PE=1 SV=2 - [TLE2_HUMAN]	8.08	4	4	4	5	1.160	1.474	1.014	1.740	5.32	8.08	5	5	743	79.8	6.62
Q9BWU0	Kanadaptin OS=Homo sapiens GN=SLC4A1AP PE=1 SV=1 - [NADAP_HUMAN]	9.05	1	5	5	17	1.827	1.366	1.108	1.741	35.65	9.05	8	17	796	88.8	5.19
P05412	Transcription factor AP-1 OS=Homo sapiens GN=JUN PE=1 SV=2 - [JUN_HUMAN]	6.95	1	1	2	7	1.230	0.375	0.678	1.743	26.84	6.95	2	7	331	35.7	8.76
P52657	Transcription initiation factor IIA subunit 2 OS=Homo sapiens GN=GTF2A2 PE=1 SV=1 - [T2AG_HUMAN]	8.26	1	1	1	2	1.011	0.980	0.886	1.745	2.62	8.26	2	2	109	12.4	6.62
P14210	Hepatocyte growth factor OS=Homo sapiens GN=HGF PE=1 SV=2 - [HGF_HUMAN]	3.85	1	3	3	4	4.426	0.968	0.537	1.746	6.40	3.85	4	4	728	83.1	7.88
Q9Y592	Centrosomal protein of 83 kDa OS=Homo sapiens GN=CEP83 PE=1 SV=2 - [CEP83_HUMAN]	4.76	1	2	3	3	0.960	0.969	0.814	1.751	4.45	4.76	3	3	693	82.0	6.28
Q9HCC8	Glycerophosphoinositol inositolphosphodiesterase GDPD2 OS=Homo sapiens GN=GDPD2 PE=2 SV=1 - [GDPD2_HUMAN]	1.86	1	1	1	1	0.774	1.157		1.754	1.90	1.86	1	1	539	61.7	8.41

Q13336	Urea transporter 1 OS=Homo sapiens GN=SLC14A1 PE=1 SV=2 - [UT1_HUMAN]	2.83	1	1	1	2	2.546	2.394	4.934	1.754	2.32	2.83	2	2	389	42.5	7.15
P01579	Interferon gamma OS=Homo sapiens GN=IFNG PE=1 SV=1 - [IFNG_HUMAN]	12.05	1	1	2	20	0.932	3.250	5.421	1.755	31.75	12.05	2	20	166	19.3	9.47
Q96RD6	Pannexin-2 OS=Homo sapiens GN=PANX2 PE=2 SV=2 - [PANX2_HUMAN]	3.84	1	1	1	3		1.644	1.867	1.757	5.85	3.84	1	3	677	74.4	8.02
P16188	HLA class I histocompatibility antigen, A-30 alpha chain OS=Homo sapiens GN=HLA-A PE=1 SV=2 - [1A30_HUMAN]	47.40	3	1	16	151	0.965	0.818	1.307	1.759	353.65	47.40	26	151	365	40.9	6.10
P00352	Retinal dehydrogenase 1 OS=Homo sapiens GN=ALDH1A1 PE=1 SV=2 - [AL1A1_HUMAN]	53.49	1	20	23	205	3.012	0.337	1.264	1.760	452.79	53.49	38	205	501	54.8	6.73
O94923	D-glucuronyl C5-epimerase OS=Homo sapiens GN=GLCE PE=1 SV=3 - [GLCE_HUMAN]	1.30	1	1	1	2	2.271	1.286	0.774	1.761	4.72	1.30	2	2	617	70.1	8.97
Q92529	SHC-transforming protein 3 OS=Homo sapiens GN=SHC3 PE=1 SV=1 - [SHC3_HUMAN]	2.69	1	1	1	1	2.473	0.794	0.425	1.762	0.00	2.69	1	1	594	64.0	8.32
Q8IZR5	CKLF-like MARVEL transmembrane domain-containing protein 4 OS=Homo sapiens GN=CMTM4 PE=1 SV=1 - [CKLF4_HUMAN]	10.26	1	2	2	2	2.011	1.173	3.447	1.766	0.00	10.26	2	2	234	25.8	7.75
P16104	Histone H2AX OS=Homo sapiens GN=H2AFX PE=1 SV=2 - [H2AX_HUMAN]	22.38	2	2	6	81	1.799	2.216	1.046	1.769	182.47	22.38	11	81	143	15.1	10.74

Q5JQS6	Germinal center-associated signaling and motility-like protein OS=Homo sapiens GN=GCSAML PE=2 SV=1 - [GSAML_HUMAN	6.67	1	1	1	1	0.461	0.459	0.876	1.770	2.35	6.67	1	1	135	15.7	6.68
Q9UPT9	Ubiquitin carboxyl-terminal hydrolase 22 OS=Homo sapiens GN=USP22 PE=1 SV=2 - [UBP22_HUMAN]	3.62	2	2	2	6	0.969	1.085	0.932	1.771	7.46	3.62	4	6	525	59.9	8.05
Q9ULR0	Pre-mRNA-splicing factor ISY1 homolog OS=Homo sapiens GN=ISY1 PE=1 SV=3 - [ISY1_HUMAN]	21.40	1	6	6	18	1.081	1.122	1.011	1.776	46.72	21.40	8	18	285	33.0	5.17
O75182	Paired amphipathic helix protein Sin3b OS=Homo sapiens GN=SIN3B PE=1 SV=2 - [SIN3B_HUMAN]	2.24	1	2	2	3	0.701	0.967	0.625	1.777	4.10	2.24	2	3	1162	133.0	6.93
Q8N3X1	Formin-binding protein 4 OS=Homo sapiens GN=FNBP4 PE=1 SV=3 - [FNBP4_HUMAN]	3.74	1	2	3	3	1.076	1.435	1.193	1.777	9.67	3.74	3	3	1017	110.2	4.74
Q969Z3	Mitochondrial amidoxime reducing component 2 OS=Homo sapiens GN=MARC2 PE=1 SV=1 - [MARC2_HUMA N]	7.46	2	3	3	5	1.766	0.760	0.657	1.778	13.96	7.46	4	5	335	38.0	9.16
Q08495	Dematin OS=Homo sapiens GN=DMTN PE=1 SV=3 - [DEMA_HUMAN]	3.46	1	1	1	1	0.310	1.717	0.569	1.780	2.51	3.46	1	1	405	45.5	8.88
Q13356	Peptidyl-prolyl cis-trans isomerase-like 2 OS=Homo sapiens GN=PPIL2 PE=1 SV=1 - [PPIL2_HUMAN]	4.81	1	2	2	4	1.001	1.382	0.847	1.780	7.34	4.81	4	4	520	58.8	8.78

Q9BT25	HAUS augmin-like complex subunit 8 OS=Homo sapiens GN=HAUS8 PE=1 SV=3 - [HAUS8_HUMAN]	5.37	1	1	2	8	1.461	1.099	1.077	1.781	21.26	5.37	2	8	410	44.8	7.06
Q15388	Mitochondrial import receptor subunit TOM20 homolog OS=Homo sapiens GN=TOMM20 PE=1 SV=1 - [TOM20_HUMA	31.03	1	3	3	6		1.769	1.309	1.782	26.54	31.03	3	6	145	16.3	8.60
Q9UKL6	Phosphatidylcholine transfer protein OS=Homo sapiens GN=PCTP PE=1 SV=1 - [PPCT_HUMAN]	4.67	1	1	1	2	1.787	1.242	1.218	1.784	4.37	4.67	2	2	214	24.8	5.78
Q2LD37	Uncharacterized protein KIAA1109 OS=Homo sapiens GN=KIAA1109 PE=1 SV=2 - [K1109_HUMAN]	0.28	1	2	2	2	0.897	0.819		1.785	2.33	0.28	2	2	5005	555.1	6.58
Q7Z7G0	Target of Nesh-SH3 OS=Homo sapiens GN=ABI3BP PE=1 SV=1 - [TARSH_HUMAN]	3.63	1	2	2	2	1.234	0.707	0.754	1.786	1.82	3.63	2	2	1075	118.6	9.44
P33993	DNA replication licensing factor MCM7 OS=Homo sapiens GN=MCM7 PE=1 SV=4 - [MCM7_HUMAN]	18.08	1	11	11	28	1.265	1.173	0.631	1.791	81.54	18.08	16	28	719	81.3	6.46
Q8NEF9	Serum response factor-binding protein 1 OS=Homo sapiens GN=SRFBP1 PE=1 SV=1 - [SRFB1_HUMAN	6.99	1	3	3	7	0.749	1.018	0.883	1.795	15.12	6.99	4	7	429	48.6	9.58
Q7LBR1	Charged multivesicular body protein 1b OS=Homo sapiens GN=CHMP1B PE=1 SV=1 - [CHM1B_HUMAN]	11.56	1	3	3	13	1.789	1.214	0.881	1.796	26.09	11.56	6	13	199	22.1	8.10
P02774	Vitamin D-binding protein OS=Homo sapiens GN=GC PE=1 SV=1 - [VTDB_HUMAN]	7.59	1	2	3	9	1.324	0.636	1.319	1.798	22.80	7.59	5	9	474	52.9	5.54

Q96CN7	Isochorismatase domain-containing protein 1 OS=Homo sapiens GN=ISOC1 PE=1 SV=3 - [ISOC1_HUMAN]	43.62	1	10	10	30	1.970	1.423	0.807	1.802	74.49	43.62	16	30	298	32.2	7.39
O60512	Beta-1,4-galactosyltransferase 3 OS=Homo sapiens GN=B4GALT3 PE=1 SV=2 - [B4GT3_HUMAN]	2.04	1	1	1	1	1.120	1.030	0.856	1.806	3.55	2.04	1	1	393	43.9	9.25
Q9BY78	RING finger protein 26 OS=Homo sapiens GN=RNF26 PE=2 SV=1 - [RNF26_HUMAN]	12.01	1	2	2	2	1.835	0.974	2.214	1.811	5.50	12.01	2	2	433	47.7	8.46
P08603	Complement factor H OS=Homo sapiens GN=CFH PE=1 SV=4 - [CFAH_HUMAN]	0.81	1	1	1	2	1.268	0.892	1.262	1.812	3.44	0.81	2	2	1231	139.0	6.61
Q969I3	Glycine N-acyltransferase-like protein 1 OS=Homo sapiens GN=GLYATL1 PE=1 SV=1 - [GLYL1_HUMAN]	3.64	1	1	1	1	1.712	0.915	1.329	1.820	0.00	3.64	1	1	302	35.1	6.87
O14817	Tetraspanin-4 OS=Homo sapiens GN=TSPAN4 PE=1 SV=1 - [TSN4_HUMAN]	3.36	1	1	1	2	3.373	0.449	0.472	1.833	2.19	3.36	1	2	238	26.1	6.47
P53708	Integrin alpha-8 OS=Homo sapiens GN=ITGA8 PE=1 SV=3 - [ITA8_HUMAN]	5.36	1	5	6	9	1.522	0.740	1.634	1.834	17.14	5.36	8	9	1063	117.4	5.58
Q02539	Histone H1.1 OS=Homo sapiens GN=HIST1H1A PE=1 SV=3 - [H11_HUMAN]	36.74	2	3	12	101	0.978	0.792	0.805	1.838	224.65	36.74	18	101	215	21.8	10.99
P49005	DNA polymerase delta subunit 2 OS=Homo sapiens GN=POLD2 PE=1 SV=1 - [DPOD2_HUMAN]	4.90	1	2	2	4	1.275	1.662	1.493	1.843	0.00	4.90	4	4	469	51.3	5.58

P01892	HLA class I histocompatibility antigen, A-2 alpha chain OS=Homo sapiens GN=HLA-A PE=1 SV=1 - [1A02_HUMAN]	54.52	2	3	22	214	2.791	1.134	1.341	1.846	517.59	54.52	36	214	365	40.9	6.99
P58004	Sestrin-2 OS=Homo sapiens GN=SESN2 PE=1 SV=1 - [SESN2_HUMAN]	3.33	1	1	1	8	0.677	0.941	0.943	1.847	33.01	3.33	1	8	480	54.5	5.90
Q8IUE6	Histone H2A type 2-B OS=Homo sapiens GN=HIST2H2AB PE=1 SV=3 - [H2A2B_HUMAN]	17.69	1	1	3	50	1.750	3.283	1.300	1.847	120.51	17.69	4	50	130	14.0	10.89
Q86SG6	Serine/threonine-protein kinase Nek8 OS=Homo sapiens GN=NEK8 PE=1 SV=1 - [NEK8_HUMAN]	1.59	1	1	1	4	1.249		2.434	1.850	10.38	1.59	1	4	692	74.8	7.81
Q53GL0	Pleckstrin homology domain-containing family O member 1 OS=Homo sapiens GN=PLEKHO1 PE=1 SV=2 - [PKHO1_HUMAN]	1.71	1	1	1	1	1.267	2.663	1.834	1.854	2.42	1.71	1	1	409	46.2	8.82
P20592	Interferon-induced GTP-binding protein Mx2 OS=Homo sapiens GN=MX2 PE=1 SV=1 - [MX2_HUMAN]	16.92	1	9	11	27	8.085	1.125	1.400	1.854	54.96	16.92	15	27	715	82.0	8.76
Q765P7	MTSS1-like protein OS=Homo sapiens GN=MTSS1L PE=1 SV=1 - [MTSSL_HUMAN]	15.66	1	7	8	22	1.853	1.189	1.385	1.855	55.21	15.66	11	22	747	79.9	7.47
O14929	Histone acetyltransferase type B catalytic subunit OS=Homo sapiens GN=HAT1 PE=1 SV=1 - [HAT1_HUMAN]	5.97	1	3	3	5	1.374	1.018	0.740	1.856	8.56	5.97	5	5	419	49.5	5.69

P30486	HLA class I histocompatibility antigen, B-48 alpha chain OS=Homo sapiens GN=HLA-B PE=1 SV=1 - [1B48_HUMAN]	32.87	2	1	11	58	12.087	0.906	2.426	1.860	143.05	32.87	16	58	362	40.3	6.00
Q9P2N2	Rho GTPase-activating protein 28 OS=Homo sapiens GN=ARHGAP28 PE=1 SV=3 - [RHG28_HUMAN]	2.88	1	1	2	4	2.070	0.734	0.960	1.864	13.84	2.88	3	4	729	82.0	7.75
Q6IS24	Putative polypeptide N-acetylgalactosaminyltransferase-like protein 3 OS=Homo sapiens GN=WBSCR17 PE=2 SV=2 - [GLTL3_HUMAN]	4.18	1	1	1	1	3.773	4.740	2.642	1.865	0.00	4.18	1	1	598	67.7	8.90
Q4V9L6	Transmembrane protein 119 OS=Homo sapiens GN=TMEM119 PE=2 SV=1 - [TM119_HUMAN]	12.37	1	4	4	12	1.889	0.462	0.607	1.865	17.30	12.37	7	12	283	29.2	4.58
Q5W0B7	Transmembrane protein 236 OS=Homo sapiens GN=TMEM236 PE=2 SV=1 - [TM236_HUMAN]	4.56	1	1	1	1	1.798	1.038	0.529	1.865	0.00	4.56	1	1	351	39.6	9.26
O15554	Intermediate conductance calcium-activated potassium channel protein 4 OS=Homo sapiens GN=KCNN4 PE=1 SV=1 -	3.98	1	1	1	1	1.388	1.112	0.983	1.871	0.00	3.98	1	1	427	47.7	9.83
Q9UKL0	REST corepressor 1 OS=Homo sapiens GN=RCOR1 PE=1 SV=1 - [RCOR1_HUMAN]	6.64	1	2	2	3	1.545	1.705	1.221	1.873	5.03	6.64	3	3	482	53.0	7.03
Q8NBV4	Probable lipid phosphate phosphatase PPAPDC3 OS=Homo sapiens GN=PPAPDC3 PE=2 SV=1 - [PPAC3_HUMAN]	6.27	1	1	1	8	0.992	1.661	1.248	1.878	19.84	6.27	2	8	271	29.4	9.94

P31152	Mitogen-activated protein kinase 4 OS=Homo sapiens GN=MAPK4 PE=1 SV=2 - [MK04_HUMAN]	4.26	2	1	2	5			3.186	1.878	13.04	4.26	3	5	587	65.9	5.45
P57081	tRNA (guanine-N(7)-)-methyltransferase non-catalytic subunit WDR4 OS=Homo sapiens GN=WDR4 PE=1 SV=2 - [WDR4_HUMAN	1.70	1	1	1	1	1.141	1.448	1.256	1.886	2.30	1.70	1	1	412	45.5	7.11
Q96D09	G-protein coupled receptor-associated sorting protein 2 OS=Homo sapiens GN=GPRASP2 PE=1 SV=1 - [GASP2_HUMAN]	5.13	1	4	4	8	1.280	1.145	1.881	1.886	16.30	5.13	6	8	838	93.7	5.01
Q9UJ14	Gamma-glutamyltransferase 7 OS=Homo sapiens GN=GGT7 PE=1 SV=2 - [GGT7_HUMAN]	8.61	1	3	3	8	1.612	1.451	1.605	1.888	21.77	8.61	4	8	662	70.4	5.10
Q01955	Collagen alpha-3(IV) chain OS=Homo sapiens GN=COL4A3 PE=1 SV=3 - [CO4A3_HUMAN]	2.63	1	2	2	3		1.259	1.089	1.892	10.17	2.63	2	3	1670	161.7	9.16
P55345	Protein arginine N-methyltransferase 2 OS=Homo sapiens GN=PRMT2 PE=1 SV=1 - [ANM2_HUMAN]	1.62	1	1	1	1	0.827	2.140	1.098	1.893	0.00	1.62	1	1	433	49.0	5.17
Q8IWL3	Iron-sulfur cluster co-chaperone protein HscB, mitochondrial OS=Homo sapiens GN=HSCB PE=1 SV=3 - [HSC20_HUMAN]	11.49	1	3	3	8	1.684	1.289	0.841	1.894	23.12	11.49	5	8	235	27.4	7.69
Q9NSI6	Bromodomain and WD repeat-containing protein 1 OS=Homo sapiens GN=BRWD1 PE=1 SV=4 - [BRWD1_HUMA	0.52	1	1	1	1	1.496	1.016	1.411	1.895	2.31	0.52	1	1	2320	262.8	8.46

Q9H222	ATP-binding cassette sub-family G member 5 OS=Homo sapiens GN=ABCG5 PE=1 SV=1 - [ABCG5_HUMAN]	2.00	1	1	1	1	1.710	1.207	1.122	1.896	0.00	2.00	1	1	651	72.5	8.95
Q8N264	Rho GTPase-activating protein 24 OS=Homo sapiens GN=ARHGAP24 PE=1 SV=2 - [RHG24_HUMAN]	1.60	1	1	1	2	0.584	1.295	1.041	1.898	2.91	1.60	2	2	748	84.2	6.67
P45983	Mitogen-activated protein kinase 8 OS=Homo sapiens GN=MAPK8 PE=1 SV=2 - [MK08_HUMAN]	13.35	4	2	6	14	0.593	0.836	0.859	1.900	28.37	13.35	10	14	427	48.3	6.89
O60675	Transcription factor MafK OS=Homo sapiens GN=MAFK PE=1 SV=1 - [MAFK_HUMAN]	4.49	3	1	1	4	0.998	0.948	0.912	1.904	9.03	4.49	2	4	156	17.5	10.10
O15516	Circadian locomoter output cycles protein kaput OS=Homo sapiens GN=CLOCK PE=1 SV=1 - [CLOCK_HUMAN]	2.01	1	1	1	1	1.645	0.790	1.722	1.905	0.00	2.01	1	1	846	95.2	7.01
Q7Z7K6	Centromere protein V OS=Homo sapiens GN=CENPV PE=1 SV=1 - [CENPV_HUMAN]	18.18	1	3	3	6	0.670	2.294	1.029	1.909	30.43	18.18	4	6	275	29.9	9.73
Q6PJW8	Consortin OS=Homo sapiens GN=CNST PE=1 SV=3 - [CNST_HUMAN]	1.52	1	1	1	3	0.953	1.092	1.624	1.909	8.24	1.52	1	3	725	79.5	4.53
P25054	Adenomatous polyposis coli protein OS=Homo sapiens GN=APC PE=1 SV=2 - [APC_HUMAN]	2.46	1	4	5	9	1.965	1.162	0.978	1.909	8.99	2.46	7	9	2843	311.5	7.80
P30405	Peptidyl-prolyl cis-trans isomerase F, mitochondrial OS=Homo sapiens GN=PPIF PE=1 SV=1 - [PPIF_HUMAN]	52.17	2	7	10	42	1.744	0.856	0.696	1.910	89.25	52.17	12	42	207	22.0	9.38

P35125	Ubiquitin carboxyl-terminal hydrolase 6 OS=Homo sapiens GN=USP6 PE=1 SV=2 - [UBP6_HUMAN]	2.49	1	2	2	2	1.281	1.112	2.070	1.910	2.55	2.49	2	2	1406	158.6	7.71
Q96DH6	RNA-binding protein Musashi homolog 2 OS=Homo sapiens GN=MSI2 PE=1 SV=1 - [MSI2H_HUMAN	13.11	1	3	3	4	1.502	1.008	0.899	1.911	11.10	13.11	4	4	328	35.2	8.48
P54652	Heat shock-related 70 kDa protein 2 OS=Homo sapiens GN=HSPA2 PE=1 SV=1 - [HSP72_HUMAN]	56.34	1	19	39	983	2.347	1.396	0.859	1.913	2193.98	56.34	69	983	639	70.0	5.74
Q9H6F5	Coiled-coil domain-containing protein 86 OS=Homo sapiens GN=CCDC86 PE=1 SV=1 - [CCD86_HUMAN]	21.11	1	6	6	11	0.901	0.880	0.810	1.919	22.70	21.11	8	11	360	40.2	10.33
Q569K6	Coiled-coil domain-containing protein 157 OS=Homo sapiens GN=CCDC157 PE=2 SV=3 - [CC157_HUMAN]	2.79	3	1	3	20	1.161	0.974	1.178	1.920	46.23	2.79	4	20	752	83.9	7.15
Q9HCL0	Protocadherin-18 OS=Homo sapiens GN=PCDH18 PE=2 SV=3 - [PCD18_HUMAN]	4.41	1	2	2	3	1.659	0.431	0.310	1.924	5.82	4.41	3	3	1135	126.1	5.15
Q9BXB1	Leucine-rich repeat-containing G-protein coupled receptor 4 OS=Homo sapiens GN=LGR4 PE=1 SV=2 - [LGR4_HUMAN]	1.05	1	1	1	4	3.202	0.225	0.476	1.925	1.62	1.05	1	4	951	104.4	6.19
Q86VD7	Mitochondrial coenzyme A transporter SLC25A42 OS=Homo sapiens GN=SLC25A42 PE=2 SV=2 - [S2542_HUMAN]	3.46	1	1	1	3	1.641	1.341	1.470	1.926	7.57	3.46	2	3	318	35.4	10.07

O00160	Unconventional myosin-If OS=Homo sapiens GN=MYO1F PE=1 SV=3 - [MYO1F_HUMAN]	1.55	1	1	2	6	1.153	0.830	1.307	1.934	9.35	1.55	3	6	1098	124.8	9.11
Q8NFG4	Folliculin OS=Homo sapiens GN=FLCN PE=1 SV=1 - [FLCN_HUMAN]	5.70	1	2	2	4	2.369	1.904	1.425	1.935	3.08	5.70	2	4	579	64.4	6.23
P0CJ79	Zinc finger protein 888 OS=Homo sapiens GN=ZNF888 PE=3 SV=1 - [ZN888_HUMAN]	16.20	122	1	3	7	1.098	0.841	1.871	1.942	4.69	16.20	4	7	500	58.7	9.09
Q9H0E3	Histone deacetylase complex subunit SAP130 OS=Homo sapiens GN=SAP130 PE=1 SV=1 - [SP130_HUMAN]	1.15	1	1	1	3	2.123	1.492	1.327	1.945	6.24	1.15	2	3	1048	110.3	9.83
Q8TBF8	Protein FAM81A OS=Homo sapiens GN=FAM81A PE=2 SV=3 - [FA81A_HUMAN]	3.80	1	2	2	8	3.186	3.022	2.189	1.947	8.96	3.80	2	8	368	42.4	8.92
Q15391	P2Y purinoceptor 14 OS=Homo sapiens GN=P2RY14 PE=1 SV=1 - [P2Y14_HUMAN]	2.07	1	1	1	1	2.074	0.913	1.075	1.955	2.05	2.07	1	1	338	38.9	9.47
P16403	Histone H1.2 OS=Homo sapiens GN=HIST1H1C PE=1 SV=2 - [H12_HUMAN]	38.03	2	3	17	144	2.914	5.997	1.204	1.955	299.82	38.03	25	144	213	21.4	10.93
Q5XPI4	E3 ubiquitin-protein ligase RNF123 OS=Homo sapiens GN=RNF123 PE=1 SV=1 - [RN123_HUMAN]	1.60	1	2	2	7	0.845	1.004	1.119	1.956	12.78	1.60	3	7	1314	148.4	6.74
Q8TBP0	TBC1 domain family member 16 OS=Homo sapiens GN=TBC1D16 PE=2 SV=1 - [TBC16_HUMAN]	1.04	1	1	1	1	1.470	0.748	0.894	1.957	0.00	1.04	1	1	767	86.3	5.82

P04818	Thymidylate synthase OS=Homo sapiens GN=TYMS PE=1 SV=3 - [TYSY_HUMAN]	16.29	1	3	3	15	1.747	0.694	0.488	1.958	45.01	16.29	4	15	313	35.7	7.01
P29466	Caspase-1 OS=Homo sapiens GN=CASP1 PE=1 SV=1 - [CASP1_HUMAN]	29.21	1	8	10	39	1.609	1.043	0.880	1.964	84.28	29.21	18	39	404	45.1	5.91
P06493	Cyclin-dependent kinase 1 OS=Homo sapiens GN=CDK1 PE=1 SV=3 - [CDK1_HUMAN]	25.59	7	6	8	24	1.508	0.863	0.602	1.968	37.06	25.59	13	24	297	34.1	8.40
O75762	Transient receptor potential cation channel subfamily A member 1 OS=Homo sapiens GN=TRPA1 PE=1 SV=3 - [TRPA1_HUMAN]	21.27	1	21	21	76	5.154	0.696	1.090	1.969	163.96	21.27	33	76	1119	127.4	7.12
Q9NPE3	H/ACA ribonucleoprotein complex subunit 3 OS=Homo sapiens GN=NOP10 PE=1 SV=1 - [NOP10_HUMAN]	20.31	1	1	1	2	1.288	1.127	1.226	1.969	7.03	20.31	1	2	64	7.7	9.99
Q8WWQ0	PH-interacting protein OS=Homo sapiens GN=PHIP PE=1 SV=2 - [PHIP_HUMAN]	1.21	1	2	2	4	1.017	1.548	1.018	1.976	6.48	1.21	3	4	1821	206.6	8.85
Q9BZ72	Membrane-associated phosphatidylinositol transfer protein 2 OS=Homo sapiens GN=PITPNM2 PE=1 SV=1 - [PITM2_HUMAN]	1.63	1	2	2	2	1.946	0.574	0.657	1.976	4.59	1.63	2	2	1349	148.8	7.17
Q8TF61	F-box only protein 41 OS=Homo sapiens GN=FBXO41 PE=2 SV=5 - [FBX41_HUMAN]	0.91	1	1	1	2	1.249	2.179	2.930	1.983	2.94	0.91	1	2	875	94.4	8.24

P14174	Macrophage migration inhibitory factor OS=Homo sapiens GN=MIF PE=1 SV=4 - [MIF_HUMAN]	17.39	1	2	2	3	2.782	1.796	0.948	1.988	4.74	17.39	3	3	115	12.5	7.88
P43235	Cathepsin K OS=Homo sapiens GN=CTSK PE=1 SV=1 - [CATK_HUMAN]	7.60	2	5	6	23	6.187	1.076	1.036	1.994	39.53	7.60	9	23	329	36.9	8.48
Q8TDX9	Polycystic kidney disease protein 1-like 1 OS=Homo sapiens GN=PKD1L1 PE=1 SV=1 - [PK1L1_HUMAN]	1.47	1	2	2	3	1.092	1.948	2.079	1.995	7.19	1.47	2	3	2849	315.2	7.06
Q6ZSB9	Zinc finger and BTB domain-containing protein 49 OS=Homo sapiens GN=ZBTB49 PE=2 SV=3 - [ZBT49_HUMAN]	1.70	1	1	1	1	1.279	1.283	0.991	2.011	0.00	1.70	1	1	765	85.0	6.98
O15068	Guanine nucleotide exchange factor DBS OS=Homo sapiens GN=MCF2L PE=1 SV=2 - [MCF2L_HUMAN]	3.61	1	2	2	3	1.281	1.252	0.446	2.013	3.18	3.61	2	3	1137	128.0	6.43
Q05193	Dynamin-1 OS=Homo sapiens GN=DNM1 PE=1 SV=2 - [DYN1_HUMAN]	11.46	1	1	12	49	1.034	1.179	1.013	2.013	90.40	11.46	20	49	864	97.3	7.17
Q9HBZ2	Aryl hydrocarbon receptor nuclear translocator 2 OS=Homo sapiens GN=ARNT2 PE=1 SV=2 - [ARNT2_HUMAN	4.04	1	2	3	3				2.016	4.18	4.04	3	3	717	78.6	6.71
Q8WUU5	GATA zinc finger domain-containing protein 1 OS=Homo sapiens GN=GATAD1 PE=1 SV=1 - [GATD1_HUMAN]	4.83	1	1	1	1	1.112	1.298	1.072	2.018	0.00	4.83	1	1	269	28.7	9.41
P02788	Lactotransferri n OS=Homo sapiens GN=LTF PE=1 SV=6 - [TRFL_HUMAN]	6.06	1	4	4	12	1.251	0.717	1.070	2.029	18.58	6.06	6	12	710	78.1	8.12

P19021	Peptidyl-glycine alpha-amidating monooxygenase OS=Homo sapiens GN=PAM PE=1 SV=2 - [AMD_HUMAN]	2.67	1	2	2	6	0.937	1.537	0.727	2.031	15.57	2.67	3	6	973	108.3	6.42
Q15413	Ryanodine receptor 3 OS=Homo sapiens GN=RYR3 PE=1 SV=3 - [RYR3_HUMAN]	1.33	1	3	5	7	1.319	2.019	2.538	2.036	10.43	1.33	6	7	4870	551.7	5.68
P0C091	FRAS1-related extracellular matrix protein 3 OS=Homo sapiens GN=FREM3 PE=2 SV=2 - [FREM3_HUMAN]	0.56	1	1	1	1	3.284	2.228	2.210	2.049	2.97	0.56	1	1	2139	238.0	5.33
Q9P0L9	Polycystic kidney disease 2-like 1 protein OS=Homo sapiens GN=PKD2L1 PE=1 SV=1 - [PK2L1_HUMAN]	2.86	1	2	3	3	1.300	1.164	1.082	2.050	7.32	2.86	3	3	805	91.9	5.48
Q8TDW0	Volume-regulated anion channel subunit LRRC8C OS=Homo sapiens GN=LRRC8C PE=1 SV=2 - [LRC8C_HUMAN]	9.84	1	5	7	18	2.140	1.386	0.922	2.060	40.11	9.84	10	18	803	92.4	7.62
Q14566	DNA replication licensing factor MCM6 OS=Homo sapiens GN=MCM6 PE=1 SV=1 - [MCM6_HUMAN]	9.01	1	7	8	20	1.259	1.250	0.593	2.061	44.17	9.01	11	20	821	92.8	5.41
Q9H582	Zinc finger protein 644 OS=Homo sapiens GN=ZNF644 PE=1 SV=2 - [ZN644_HUMAN]	0.68	1	1	1	1	9.213	3.958	4.110	2.063	2.10	0.68	1	1	1327	149.5	8.16
Q96RD1	Olfactory receptor 6C1 OS=Homo sapiens GN=OR6C1 PE=2 SV=2 - [OR6C1_HUMAN]	11.86	1	2	2	2	0.101	0.664	1.344	2.064	5.25	11.86	2	2	312	35.6	8.82
Q96D53	AarF domain-containing protein kinase 4 OS=Homo sapiens GN=ADCK4 PE=1 SV=2 - [ADCK4_HUMAN]	1.65	1	1	1	2	1.239	3.164	1.169	2.064	6.73	1.65	1	2	544	60.0	7.23

Q96DF8	Protein DGCR14 OS=Homo sapiens GN=DGCR14 PE=1 SV=1 - [DGC14_HUMAN]	7.14	1	2	2	5	1.747	0.856	1.148	2.065	14.05	7.14	3	5	476	52.5	7.56
A6NI28	Rho GTPase-activating protein 42 OS=Homo sapiens GN=ARHGAP42 PE=1 SV=3 - [RHG42_HUMAN]	3.66	1	2	3	6	1.260	0.617	1.267	2.069	14.80	3.66	5	6	874	98.5	7.99
P10145	Interleukin-8 OS=Homo sapiens GN=CXCL8 PE=1 SV=1 - [IL8_HUMAN]	9.09	1	1	1	2	3.703	0.989	0.432	2.084	0.00	9.09	2	2	99	11.1	8.84
P02768	Serum albumin OS=Homo sapiens GN=ALB PE=1 SV=2 - [ALBU_HUMAN]	7.72	1	6	6	221	1.672	0.657	1.167	2.084	404.63	7.72	10	221	609	69.3	6.28
P10412	Histone H1.4 OS=Homo sapiens GN=HIST1H1E PE=1 SV=2 - [H14_HUMAN]	39.73	2	2	16	144	1.785	3.548	1.074	2.091	306.76	39.73	24	144	219	21.9	11.03
Q92982	Ninjurin-1 OS=Homo sapiens GN=NINJ1 PE=1 SV=2 - [NINJ1_HUMAN]	7.89	1	1	1	4	1.733	1.013	1.356	2.100	8.20	7.89	2	4	152	16.3	6.28
Q96HU8	GTP-binding protein Di-Ras2 OS=Homo sapiens GN=DIRAS2 PE=1 SV=1 - [DIRA2_HUMAN]	5.53	1	1	1	2	2.255	4.577	6.397	2.119	1.64	5.53	2	2	199	22.5	8.76
Q9P0V9	Septin-10 OS=Homo sapiens GN=SEPT10 PE=1 SV=2 - [SEP10_HUMAN]	23.57	1	6	8	17	2.621	1.900	1.800	2.128	42.90	23.57	11	17	454	52.6	6.80
P00749	Urokinase-type plasminogen activator OS=Homo sapiens GN=PLAU PE=1 SV=2 - [UROK_HUMAN]	4.87	1	2	2	5	2.677	0.956	0.469	2.132	7.41	4.87	3	5	431	48.5	8.41
Q9NQW6	Actin-binding protein anillin OS=Homo sapiens GN=ANLN PE=1 SV=2 - [ANLN_HUMAN]	7.38	1	9	9	12	1.285	1.141	0.692	2.140	24.79	7.38	9	12	1124	124.1	8.07

Q3YBM2	Transmembrane protein 176B OS=Homo sapiens GN=TMEM176B PE=2 SV=2 - [T176B_HUMAN]	10.74	1	4	4	40	1.904	0.706	0.313	2.146	89.78	10.74	8	40	270	29.0	8.48
Q96T21	Selenocysteine insertion sequence-binding protein 2 OS=Homo sapiens GN=SECISBP2 PE=1 SV=2 - [SEBP2_HUMAN]	0.82	1	1	1	1	1.543	2.682	1.897	2.146	0.00	0.82	1	1	854	95.4	8.12
Q96QE2	Proton myo-inositol cotransporter OS=Homo sapiens GN=SLC2A13 PE=1 SV=3 - [MYCT_HUMAN]	5.56	1	3	3	3	1.781	0.880	0.455	2.150	11.16	5.56	3	3	648	70.3	5.96
Q8WYH8	Inhibitor of growth protein 5 OS=Homo sapiens GN=ING5 PE=1 SV=1 - [ING5_HUMAN]	3.33	1	1	1	2	1.007	1.133	0.973	2.150	5.34	3.33	2	2	240	27.7	7.61
Q15651	High mobility group nucleosome-binding domain-containing protein 3 OS=Homo sapiens GN=HMGN3 PE=1 SV=2 - [HMGN3_HUMAN]	13.13	1	1	1	8			1.307	2.155	6.81	13.13	1	8	99	10.7	9.66
Q7Z7L8	Uncharacterized protein C11orf96 OS=Homo sapiens GN=C11orf96 PE=1 SV=3 - [CK096_HUMAN]	5.52	1	2	2	8	2.134	1.187	1.625	2.158	14.21	5.52	3	8	435	46.1	9.99
Q6AZY7	Scavenger receptor class A member 3 OS=Homo sapiens GN=SCARA3 PE=2 SV=1 - [SCAR3_HUMAN]	4.79	1	2	2	6	1.030	2.122	1.942	2.161	14.99	4.79	3	6	606	65.1	6.54
Q96FF7	Uncharacterized protein LOC113230 OS=Homo sapiens PE=2 SV=4 - [YS003_HUMAN]	9.13	1	2	2	3	1.077	1.104	1.187	2.168	2.05	9.13	2	3	219	24.0	10.92

P61952	Guanine nudeotide-binding protein G(I)/G(S)/G(O) subunit gamma-11 OS=Homo sapiens GN=GNG11 PE=1 SV=1 - [GBG11_HUMAN]	30.14	2	2	3	8	3.981	0.649	0.777	2.175	15.48	30.14	4	8	73	8.5	5.54
Q8N6K7	Sterile alpha motif domain-containing protein 3 OS=Homo sapiens GN=SAMD3 PE=1 SV=2 - [SAMD3_HUMAN]	2.12	1	1	1	1	1.702	0.490	0.895	2.178	2.71	2.12	1	1	520	61.2	7.09
O14523	C2 domain-containing protein 2-like OS=Homo sapiens GN=C2CD2L PE=1 SV=3 - [C2C2L_HUMAN]	1.42	1	1	1	2	1.709	0.989	1.511	2.180	5.20	1.42	2	2	706	76.1	7.69
Q9UK73	Protein fem-1 homolog B OS=Homo sapiens GN=FEM1B PE=1 SV=1 - [FEM1B_HUMAN]	4.15	1	2	2	6	0.851	1.156	0.787	2.182	15.29	4.15	3	6	627	70.2	6.61
P46013	Antigen KI-67 OS=Homo sapiens GN=MKI67 PE=1 SV=2 - [KI67_HUMAN]	3.53	1	7	7	16	1.179	1.191	0.713	2.195	35.58	3.53	9	16	3256	358.5	9.45
Q9BZJ0	Crooked neck-like protein 1 OS=Homo sapiens GN=CRNKL1 PE=1 SV=4 - [CRNL1_HUMAN]	6.25	1	4	4	5	1.030	1.452	2.351	2.199	11.31	6.25	5	5	848	100.4	8.00
P33992	DNA replication licensing factor MCM5 OS=Homo sapiens GN=MCM5 PE=1 SV=5 - [MCM5_HUMAN]	25.89	1	17	17	55	1.457	1.266	0.670	2.203	121.59	25.89	25	55	734	82.2	8.37
Q9Y2P4	Long-chain fatty acid transport protein 6 OS=Homo sapiens GN=SLC27A6 PE=1 SV=1 - [S27A6_HUMAN]	4.52	1	3	3	3	4.458	1.283	0.537	2.218	7.52	4.52	3	3	619	70.1	8.51

Q15437	Protein transport protein Sec23B OS=Homo sapiens GN=SEC23B PE=1 SV=2 - [SC23B_HUMAN]	3.91	1	2	3	14	1.226	0.571	0.563	2.243	31.37	3.91	4	14	767	86.4	6.89
P25205	DNA replication licensing factor MCM3 OS=Homo sapiens GN=MCM3 PE=1 SV=3 - [MCM3_HUMAN]	12.62	1	10	10	21	1.379	1.334	0.654	2.250	50.56	12.62	16	21	808	90.9	5.77
P22735	Protein-glutamine gamma-glutamyltransferase K OS=Homo sapiens GN=TGM1 PE=1 SV=4 - [TGM1_HUMAN]	1.84	1	1	1	1	1.900	3.041	4.988	2.267	0.00	1.84	1	1	817	89.7	6.04
Q8NCL9	Protein APCDD1-like OS=Homo sapiens GN=APCDD1L PE=2 SV=1 - [APCDL_HUMAN]	4.79	1	2	2	3	0.567	0.913	1.207	2.292	7.04	4.79	3	3	501	55.6	8.85
Q9UJA2	Cardiolipin synthase OS=Homo sapiens GN=CRLS1 PE=2 SV=1 - [CRLS1_HUMAN]	4.32	1	1	1	1	1.688	0.884	1.756	2.304	3.82	4.32	1	1	301	32.6	9.67
Q0VF96	Cingulin-like protein 1 OS=Homo sapiens GN=CGNL1 PE=1 SV=2 - [CGNL1_HUMAN]	1.69	1	1	2	3	0.650	0.659	1.793	2.307	4.28	1.69	2	3	1302	149.0	5.67
Q99856	AT-rich interactive domain-containing protein 3A OS=Homo sapiens GN=ARID3A PE=1 SV=2 - [ARI3A_HUMAN]	1.52	1	1	1	1	2.253	1.503	1.132	2.317	0.00	1.52	1	1	593	62.9	4.91
Q5U5Z8	Cytosolic carboxypeptidase 2 OS=Homo sapiens GN=AGBL2 PE=1 SV=2 - [CBPC2_HUMAN]	2.44	1	1	2	2		1.637		2.322	3.87	2.44	2	2	902	104.1	9.00

P31323	cAMP-dependent protein kinase type II-beta regulatory subunit OS=Homo sapiens GN=PRKAR2B PE=1 SV=3 - [KAP3_HUMAN]	28.95	1	6	9	39	3.149	1.852	1.087	2.339	135.21	28.95	14	39	418	46.3	4.92
P14384	Carboxypeptidase M OS=Homo sapiens GN=CPM PE=1 SV=2 - [CBPM_HUMAN]	7.45	1	3	3	5	0.350	4.760	0.475	2.340	11.75	7.45	4	5	443	50.5	7.36
Q9HCF6	Transient receptor potential cation channel subfamily M member 3 OS=Homo sapiens GN=TRPM3 PE=2 SV=4 - [TRPM3_HUMAN]	2.60	1	3	3	6	3.060	0.393	1.960	2.354	1.84	2.60	3	6	1732	197.4	7.15
P23975	Sodium-dependent noradrenaline transporter OS=Homo sapiens GN=SLC6A2 PE=1 SV=1 - [SC6A2_HUMAN]	1.94	1	1	1	1				2.366	2.43	1.94	1	1	617	69.3	7.47
P02765	Alpha-2-HS-glycoprotein OS=Homo sapiens GN=AHSG PE=1 SV=1 - [FETUA_HUMAN]	7.08	1	3	3	14	1.749	0.526	0.961	2.370	36.83	7.08	3	14	367	39.3	5.72
Q5H9E4	Solute carrier family 25 member 53 OS=Homo sapiens GN=SLC25A53 PE=2 SV=1 - [S2553_HUMAN]	2.93	1	1	1	2				2.370	2.94	2.93	1	2	307	34.5	9.85
Q8IY51	Tigger transposable element-derived protein 4 OS=Homo sapiens GN=TIGD4 PE=2 SV=2 - [TIGD4_HUMAN]	2.93	1	1	1	1	1.739	3.256	2.329	2.374	1.97	2.93	1	1	512	57.4	6.39
Q9BQI5	SH3-containing GRB2-like protein 3-interacting protein 1 OS=Homo sapiens GN=SGIP1 PE=1 SV=2 - [SGIP1_HUMAN]	2.66	1	1	1	6	1.817	1.211	1.165	2.382	16.75	2.66	2	6	828	89.1	8.22

Q7Z5K2	Wings apart-like protein homolog OS=Homo sapiens GN=WAPAL PE=1 SV=1 - [WAPL_HUMAN]	1.34	1	2	2	5	2.584	3.208	1.751	2.385	6.76	1.34	2	5	1190	132.9	5.44
Q52M75	Putative uncharacterized protein encoded by LINC01554 OS=Homo sapiens GN=LINC01554 PE=2 SV=2 - [CE027_HUMAN	14.58	1	1	1	1	1.771	1.967	2.289	2.398	0.00	14.58	1	1	96	10.8	9.88
Q8TF68	Zinc finger protein 384 OS=Homo sapiens GN=ZNF384 PE=1 SV=2 - [ZN384_HUMAN]	4.33	1	1	1	1	1.597	1.173	1.289	2.405	5.21	4.33	1	1	577	63.2	8.95
P37059	Estradiol 17-beta-dehydrogenase 2 OS=Homo sapiens GN=HSD17B2 PE=1 SV=1 - [DHB2_HUMAN]	37.73	1	12	12	55	5.723	0.677	1.561	2.406	157.37	37.73	19	55	387	42.8	8.50
Q8NI17	Interleukin-31 receptor subunit alpha OS=Homo sapiens GN=IL31RA PE=1 SV=1 - [IL31R_HUMAN]	3.28	1	1	2	6	5.201	0.967	1.350	2.408	6.79	3.28	2	6	732	82.9	7.09
Q96HP8	Transmembrane protein 176A OS=Homo sapiens GN=TMEM176A PE=2 SV=1 - [T176A_HUMAN]	4.68	1	2	2	7	0.778	1.004	0.590	2.419	17.40	4.68	2	7	235	26.1	8.56
Q96IZ7	Serine/Arginine-related protein 53 OS=Homo sapiens GN=RSRC1 PE=1 SV=1 - [RSRC1_HUMAN]	3.59	1	1	1	1	1.220	0.560	0.891	2.444	2.47	3.59	1	1	334	38.7	11.08
P48740	Mannan-binding lectin serine protease 1 OS=Homo sapiens GN=MASP1 PE=1 SV=3 - [MASP1_HUMA	8.15	1	4	4	12	2.889	0.817	0.629	2.456	29.00	8.15	7	12	699	79.2	5.49

Q9BX84	Transient receptor potential cation channel subfamily M member 6 OS=Homo sapiens GN=TRPM6 PE=1 SV=2 - [TRPM6_HUMAN]	0.59	1	1	1	1	3.950	3.853	6.066	2.497	2.50	0.59	1	1	2022	231.6	7.77
Q14246	EGF-like module-containing mucin-like hormone receptor-like 1 OS=Homo sapiens GN=EMR1 PE=2 SV=3 - [EMR1_HUMAN]	1.81	1	1	1	1	6.526	4.476	0.702	2.507	0.00	1.81	1	1	886	97.6	6.80
P49736	DNA replication licensing factor MCM2 OS=Homo sapiens GN=MCM2 PE=1 SV=4 - [MCM2_HUMAN]	15.93	1	12	12	27	1.668	1.314	0.657	2.520	62.41	15.93	16	27	904	101.8	5.52
Q71DI3	Histone H3.2 OS=Homo sapiens GN=HIST2H3A PE=1 SV=3 - [H32_HUMAN]	59.56	4	3	11	148	0.861	2.168	0.857	2.531	284.34	59.56	15	148	136	15.4	11.27
Q9BZV3	Interphotoreceptor matrix proteoglycan 2 OS=Homo sapiens GN=IMPG2 PE=1 SV=3 - [IMPG2_HUMAN]	1.53	1	1	1	4	1.721	0.931	1.378	2.535	0.00	1.53	1	4	1241	138.5	4.61
Q9UBF8	Phosphatidylinositol 4-kinase beta OS=Homo sapiens GN=PI4KB PE=1 SV=1 - [PI4KB_HUMAN	4.41	1	2	2	3	1.715	3.672	1.935	2.538	2.85	4.41	2	3	816	91.3	6.25
Q5TAT6	Collagen alpha-1(XIII) chain OS=Homo sapiens GN=COL13A1 PE=1 SV=1 - [CODA1_HUMAN]	2.37	1	1	1	4	1.918	0.650	0.461	2.539	6.26	2.37	2	4	717	69.9	9.17
Q9Y294	Histone chaperone ASF1A OS=Homo sapiens GN=ASF1A PE=1 SV=1 - [ASF1A_HUMAN]	11.27	2	2	2	7	2.345	1.168	1.271	2.541	13.14	11.27	3	7	204	23.0	4.41

P24557	Thromboxane-A synthase OS=Homo sapiens GN=TBXAS1 PE=1 SV=3 - [THAS_HUMAN]	5.63	1	3	3	5	3.573	0.673	0.835	2.546	10.52	5.63	4	5	533	60.5	7.62
Q9Y6X5	Bis(5'-adenosyl)-triphosphatase ENPP4 OS=Homo sapiens GN=ENPP4 PE=1 SV=3 - [ENPP4_HUMAN]	2.43	1	1	1	1	0.902	1.533	2.120	2.560	0.00	2.43	1	1	453	51.6	6.15
Q9C0H9	SRC kinase signaling inhibitor 1 OS=Homo sapiens GN=SRCIN1 PE=1 SV=3 - [SRCN1_HUMAN]	3.41	1	1	2	2			3.715	2.562	4.45	3.41	2	2	1055	112.4	9.29
Q13191	E3 ubiquitin-protein ligase CBL-B OS=Homo sapiens GN=CBLB PE=1 SV=2 - [CBLB_HUMAN]	1.73	1	1	2	2	0.719	2.515	3.815	2.569	3.96	1.73	2	2	982	109.4	7.88
Q9UBL3	Set1/Ash2 histone methyltransferase complex subunit ASH2 OS=Homo sapiens GN=ASH2L PE=1 SV=1 - [ASH2L_HUMAN]	9.87	1	7	7	11	0.790	1.566	1.269	2.574	19.06	9.87	10	11	628	68.7	5.69
Q9Y6K8	Adenylate kinase isoenzyme 5 OS=Homo sapiens GN=AK5 PE=1 SV=2 - [KAD5_HUMAN]	7.12	2	2	4	17	1.474	0.368	0.867	2.581	34.79	7.12	6	17	562	63.3	5.07
Q8WV24	Pleckstrin homology-like domain family A member 1 OS=Homo sapiens GN=PHLDA1 PE=1 SV=4 - [PHLA1_HUMAN]	2.24	1	1	1	6	3.063	1.600	1.551	2.627	15.94	2.24	2	6	401	45.0	9.89
O60488	Long-chain-fatty-acid--CoA ligase 4 OS=Homo sapiens GN=ACSL4 PE=1 SV=2 - [ACSL4_HUMAN]	31.36	1	18	22	81	4.512	0.790	0.768	2.634	214.71	31.36	32	81	711	79.1	8.38

Q96A09	Protein FAM46B OS=Homo sapiens GN=FAM46B PE=2 SV=2 - [FA46B_HUMAN]	2.82	1	1	1	1	9.988	1.287	1.575	2.637	2.73	2.82	1	1	425	46.7	8.15
P48681	Nestin OS=Homo sapiens GN=NES PE=1 SV=2 - [NEST_HUMAN]	35.53	2	51	51	271	1.878	1.087	1.820	2.657	709.69	35.53	90	271	1621	177.3	4.36
Q96KK5	Histone H2A type 1-H OS=Homo sapiens GN=HIST1H2AH PE=1 SV=3 - [H2A1H_HUMAN]	21.09	8	1	5	85	2.975	6.661	2.988	2.663	188.84	21.09	9	85	128	13.9	10.89
Q8WWM9	Cytoglobin OS=Homo sapiens GN=CYGB PE=1 SV=1 - [CYGB_HUMAN]	32.63	1	7	7	33	5.935	0.410	0.515	2.693	76.93	32.63	9	33	190	21.4	6.80
Q8IW75	Serpin A12 OS=Homo sapiens GN=SERPINA12 PE=1 SV=1 - [SPA12_HUMAN]	4.11	1	1	1	1	0.630	6.232	15.974	2.739	0.00	4.11	1	1	414	47.1	9.29
P21980	Protein-glutamine gamma-glutamyltransferase 2 OS=Homo sapiens GN=TGM2 PE=1 SV=2 - [TGM2_HUMAN]	63.32	1	36	36	806	1.854	2.644	2.095	2.774	2142.91	63.32	63	806	687	77.3	5.22
Q2KHR3	Glutamine and serine-rich protein 1 OS=Homo sapiens GN=QSER1 PE=1 SV=3 - [QSER1_HUMAN]	0.63	1	1	1	1	1.744	2.518	1.020	2.805	2.08	0.63	1	1	1735	189.9	7.08
O60262	Guanine nucleotide-binding protein G(I)/G(S)/G(O) subunit gamma-7 OS=Homo sapiens GN=GNG7 PE=1 SV=1 - [GBG7_HUMAN]	47.06	1	2	3	10	0.979	1.204	1.220	2.813	30.56	47.06	3	10	68	7.5	8.51
Q9BXR6	Complement factor H-related protein 5 OS=Homo sapiens GN=CFHR5 PE=1 SV=1 - [FHR5_HUMAN]	2.46	1	1	1	1	3.267	1.034	1.047	2.853	0.00	2.46	1	1	569	64.4	7.06

P15863	Paired box protein Pax-1 OS=Homo sapiens GN=PAX1 PE=1 SV=4 - [PAX1_HUMAN]	2.62	1	1	1	1	2.248	3.397	6.609	2.866	0.00	2.62	1	1	534	55.5	9.86
P59797	Selenoprotein V OS=Homo sapiens GN=SELV PE=2 SV=2 - [SELV_HUMAN]	2.60	1	1	1	1	0.516	1.891	3.082	2.884	0.00	2.60	1	1	346	36.8	9.66
Q15858	Sodium channel protein type 9 subunit alpha OS=Homo sapiens GN=SCN9A PE=1 SV=3 - [SCN9A_HUMAN	0.70	1	1	1	1	0.848	0.689		2.888	2.98	0.70	1	1	1988	226.2	6.93
Q9BQE4	Selenoprotein S OS=Homo sapiens GN=VIMP PE=1 SV=3 - [SELS_HUMAN]	9.52	1	1	2	8	1.987	1.080	2.713	2.947	24.12	9.52	2	8	189	21.2	9.70
Q5JS37	NHL repeat-containing protein 3 OS=Homo sapiens GN=NHLRC3 PE=2 SV=1 - [NHLC3_HUMAN]	3.46	1	1	1	1	2.053	1.213	0.915	2.949	0.00	3.46	1	1	347	38.3	6.43
Q68G74	LIM/homeobox protein Lhx8 OS=Homo sapiens GN=LHX8 PE=2 SV=2 - [LHX8_HUMAN]	4.21	1	1	1	1	2.405	1.671	4.187	2.960	2.10	4.21	1	1	356	39.3	8.22
Q12834	Cell division cycle protein 20 homolog OS=Homo sapiens GN=CDC20 PE=1 SV=2 - [CDC20_HUMAN]	2.40	1	1	1	2	1.470	1.136	0.806	2.979	3.08	2.40	2	2	499	54.7	9.23
Q5TGI0	Failed axon connections homolog OS=Homo sapiens GN=FAXC PE=2 SV=2 - [FAXC_HUMAN]	3.67	1	1	1	1	0.885	0.951	1.224	2.999	3.07	3.67	1	1	409	46.8	5.57
Q96NH3	Protein broad-minded OS=Homo sapiens GN=TBC1D32 PE=2 SV=4 - [BROMI_HUMA N]	0.72	1	1	1	1	4.030	4.234	4.519	3.006	0.00	0.72	1	1	1257	144.7	6.74

P17040	Zinc finger and SCAN domain-containing protein 20 OS=Homo sapiens GN=ZSCAN20 PE=2 SV=3 - [ZSC20_HUMAN]	6.04	120	1	3	5	1.151	0.677	1.872	3.082	4.44	6.04	4	5	1043	117.5	6.43
P23141	Liver carboxylesterase 1 OS=Homo sapiens GN=CES1 PE=1 SV=2 - [EST1_HUMAN]	19.05	2	9	9	30	0.589	1.381	0.663	3.163	64.11	19.05	17	30	567	62.5	6.60
P07477	Trypsin-1 OS=Homo sapiens GN=PRSS1 PE=1 SV=1 - [TRY1_HUMAN]	7.29	2	2	2	8	1.517	1.015	1.227	3.173	10.65	7.29	3	8	247	26.5	6.51
Q9H720	PGAP2-interacting protein OS=Homo sapiens GN=CWH43 PE=2 SV=2 - [PG2IP_HUMAN]	2.43	1	1	1	1	1.868	1.406	1.696	3.183	2.23	2.43	1	1	699	78.5	8.57
Q9ULQ1	Two pore calcium channel protein 1 OS=Homo sapiens GN=TPCN1 PE=1 SV=3 - [TPC1_HUMAN]	2.82	1	1	2	3	1.724	0.733	0.583	3.230	7.90	2.82	3	3	816	94.1	8.27
O15055	Period circadian protein homolog 2 OS=Homo sapiens GN=PER2 PE=1 SV=2 - [PER2_HUMAN]	0.64	1	1	1	1	0.781	3.315	2.809	3.261	0.00	0.64	1	1	1255	136.5	6.47
P05204	Non-histone chromosomal protein HMG-17 OS=Homo sapiens GN=HMGN2 PE=1 SV=3 - [HMGN2_HUMAN]	53.33	1	5	5	42	2.501	5.571	2.357	3.330	119.92	53.33	7	42	90	9.4	9.99
Q9Y581	Insulin-like peptide INSL6 OS=Homo sapiens GN=INSL6 PE=2 SV=2 - [INSL6_HUMAN]	6.57	1	1	1	2	1.442	0.886	0.306	3.414	0.00	6.57	1	2	213	24.8	9.67
Q02241	Kinesin-like protein KIF23 OS=Homo sapiens GN=KIF23 PE=1 SV=3 - [KIF23_HUMAN]	2.29	1	1	2	2	1.356	1.139	0.360	3.452	5.40	2.29	2	2	960	110.0	8.51

Q6UQ28	Placenta-expressed transcript 1 protein OS=Homo sapiens GN=PLET1 PE=2 SV=2 - [PLET1_HUMAN]	7.73	1	1	1	1	0.482	4.435	5.098	3.524	0.00	7.73	1	1	207	23.4	5.12
Q9NRD5	PRKCA-binding protein OS=Homo sapiens GN=PICK1 PE=1 SV=2 - [PICK1_HUMAN]	2.65	1	1	1	2		8.212	7.957	3.539	1.82	2.65	1	2	415	46.6	5.30
P49961	Ectonucleoside triphosphate diphosphohydrolase 1 OS=Homo sapiens GN=ENTPD1 PE=1 SV=1 - [ENTP1_HUMAN]	10.39	1	6	6	15	5.875	0.467	0.576	3.603	24.45	10.39	10	15	510	57.9	6.34
Q5VVW2	GTPase-activating Rap/Ran-GAP domain-like protein 3 OS=Homo sapiens GN=GARNL3 PE=2 SV=2 - [GARL3_HUMAN]	1.48	1	2	3	9	1.446	0.420	0.630	3.673	11.02	1.48	3	9	1013	112.8	7.65
Q6N022	Teneurin-4 OS=Homo sapiens GN=TENM4 PE=1 SV=2 - [TEN4_HUMAN]	0.79	1	1	2	2	1.599	1.472	1.719	3.745	4.19	0.79	2	2	2769	307.8	6.55
Q99683	Mitogen-activated protein kinase kinase kinase 5 OS=Homo sapiens GN=MAP3K5 PE=1 SV=1 - [M3K5_HUMAN]	2.18	1	1	3	5	1.667	0.977	1.999	4.146	6.14	2.18	4	5	1374	154.4	5.78
P55771	Paired box protein Pax-9 OS=Homo sapiens GN=PAX9 PE=1 SV=3 - [PAX9_HUMAN]	2.35	1	1	1	4	1.766	0.488	1.484	4.170	11.27	2.35	1	4	341	36.3	9.32
Q9BRX8	Redox-regulatory protein FAM213A OS=Homo sapiens GN=FAM213A PE=1 SV=3 - [F213A_HUMAN]	15.72	1	3	3	7	4.738	1.958	1.710	4.182	10.33	15.72	4	7	229	25.7	8.84

P31350	Ribonucleoside-diphosphate reductase subunit M2 OS=Homo sapiens GN=RRM2 PE=1 SV=1 - [RIR2_HUMAN]	13.88	1	4	6	15	1.860	0.611	0.505	4.250	35.26	13.88	8	15	389	44.8	5.38
Q14493	Histone RNA hairpin-binding protein OS=Homo sapiens GN=SLBP PE=1 SV=1 - [SLBP_HUMAN]	8.15	1	2	2	3	1.698	1.157	0.976	4.523	7.44	8.15	3	3	270	31.3	7.47
O60303	Uncharacterized protein KIAA0556 OS=Homo sapiens GN=KIAA0556 PE=1 SV=4 - [K0556_HUMAN]	1.05	1	1	1	1	6.954	3.858	2.332	4.558	0.00	1.05	1	1	1618	180.8	5.87
A6NKB5	Pecanex-like protein 2 OS=Homo sapiens GN=PCNXL2 PE=2 SV=3 - [PCX2_HUMAN]	1.12	1	2	2	3		1.570	2.902	4.614	4.70	1.12	2	3	2137	237.1	6.76
Q7Z443	Polycystic kidney disease protein 1-like 3 OS=Homo sapiens GN=PKD1L3 PE=1 SV=1 - [PK1L3_HUMAN]	1.04	1	1	1	2	3.377	1.880	0.873	4.784	0.00	1.04	1	2	1732	195.8	8.48
Q8IWV2	Contactin-4 OS=Homo sapiens GN=CNTN4 PE=1 SV=1 - [CNTN4_HUMAN]	0.97	1	1	1	5	0.864	0.940	1.051	4.803	11.63	0.97	2	5	1026	113.4	7.47
Q969S2	Endonuclease 8-like 2 OS=Homo sapiens GN=NEIL2 PE=1 SV=3 - [NEIL2_HUMAN]	3.92	1	1	1	1	0.760	0.916	0.852	4.841	3.48	3.92	1	1	332	36.8	6.79
O00194	Ras-related protein Rab-27B OS=Homo sapiens GN=RAB27B PE=1 SV=4 - [RB27B_HUMAN]	38.53	1	6	8	17	5.953	0.726	0.618	4.888	34.50	38.53	11	17	218	24.6	5.52
O75829	Leukocyte cell-derived chemotaxin 1 OS=Homo sapiens GN=LECT1 PE=1 SV=1 - [LECT1_HUMAN]	1.80	1	1	1	1	1.233	2.680	4.508	5.455	0.00	1.80	1	1	334	37.1	7.56

Q5TIE3	von Willebrand factor A domain-containing protein 5B1 OS=Homo sapiens GN=VWA5B1 PE=1 SV=2 - [VW5B1_HUMA	0.74	1	1	1	2		0.891	1.343	5.640	4.37	0.74	1	2	1220	133.7	8.15
O00294	Tubby-related protein 1 OS=Homo sapiens GN=TULP1 PE=1 SV=3 - [TULP1_HUMAN]	2.40	1	1	1	2	0.780		5.722	6.813	6.18	2.40	1	2	542	60.6	9.47
P08571	Monocyte differentiation antigen CD14 OS=Homo sapiens GN=CD14 PE=1 SV=2 - [CD14_HUMAN]	2.67	1	1	1	2	7.996	0.605	0.523	6.912	3.47	2.67	1	2	375	40.1	6.23
P61626	Lysozyme C OS=Homo sapiens GN=LYZ PE=1 SV=1 - [LYSC_HUMAN]	19.59	1	3	3	12	0.205	1.059	1.061	9.766	24.55	19.59	5	12	148	16.5	9.16
Q9UPR0	Inactive phospholipase C-like protein 2 OS=Homo sapiens GN=PLCL2 PE=1 SV=2 - [PLCL2_HUMAN]	1.69	1	1	1	1		8.755	6.012		1.66	1.69	1	1	1127	125.8	6.90
Q9Y283	Inversin OS=Homo sapiens GN=INVS PE=1 SV=2 - [INVS_HUMAN]	1.13	1	1	1	1	0.818		5.644		2.11	1.13	1	1	1065	117.8	9.35
A8MVM7	Putative uncharacterize d protein ENSP00000382 790 OS=Homo sapiens PE=5 SV=3 - [YD021_HUMAN]	3.15	1	1	2	3		7.246	3.262		8.82	3.15	2	3	634	73.4	9.79
Q9Y6A5	Transforming acidic coiled-coil-containing protein 3 OS=Homo sapiens GN=TACC3 PE=1 SV=1 - [TACC3_HUMAN]	3.10	1	1	2	2	1.377	3.123	3.175		5.40	3.10	2	2	838	90.3	5.05
Q13698	Voltage-dependent L-type calcium channel subunit alpha-1S OS=Homo sapiens GN=CACNA1S PE=1 SV=4 - [CAC1S_HUMAN]	0.75	1	2	2	3	0.886	1.675	2.701		8.31	0.75	2	3	1873	212.2	6.57

Q9H2J7	Sodium-dependent neutral amino acid transporter B(0)AT2 OS=Homo sapiens GN=SLC6A15 PE=1 SV=1 - [S6A15_HUMAN	3.56	1	2	2	2		1.068	2.604		2.02	3.56	2	2	730	81.8	5.19
Q8N128	Protein FAM177A1 OS=Homo sapiens GN=FAM177A1 PE=1 SV=1 - [F177A_HUMAN]	10.80	1	2	2	3	0.640	1.154	2.575		8.41	10.80	2	3	213	23.7	4.45
P42658	Dipeptidyl aminopeptidase-like protein 6 OS=Homo sapiens GN=DPP6 PE=1 SV=2 - [DPP6_HUMAN]	1.97	1	1	1	1			2.376		0.00	1.97	1	1	865	97.5	6.37
Q13733	Sodium/potassium-transporting ATPase subunit alpha-4 OS=Homo sapiens GN=ATP1A4 PE=1 SV=3 - [AT1A4_HUMAN]	9.82	1	1	9	35			2.281		93.21	9.82	15	35	1029	114.1	6.64
Q5GH70	XK-related protein 9 OS=Homo sapiens GN=XKR9 PE=2 SV=1 - [XKR9_HUMAN]	2.14	1	1	1	1	0.654	4.124	2.252		1.73	2.14	1	1	373	43.4	8.22
Q86VI4	Lysosomal-associated transmembrane protein 4B OS=Homo sapiens GN=LAPTM4B PE=1 SV=1 - [LAP4B_HUMAN]	12.16	1	2	2	4			2.134		6.86	12.16	2	4	370	41.1	8.97
Q96L96	Alpha-protein kinase 3 OS=Homo sapiens GN=ALPK3 PE=2 SV=2 - [ALPK3_HUMAN]	1.05	1	1	1	1			2.077		2.62	1.05	1	1	1907	201.1	7.58
O43364	Homeobox protein Hox-A2 OS=Homo sapiens GN=HOXA2 PE=1 SV=1 - [HXA2_HUMAN]	3.99	1	1	1	1	1.316		2.014		3.04	3.99	1	1	376	41.0	5.81

Q5H9M0	PWWP domain-containing protein MUM1L1 OS=Homo sapiens GN=MUM1L1 PE=2 SV=1 - [MUML1_HUMAN]	2.87	1	1	1	2			1.969		3.12	2.87	1	2	696	79.0	4.97
Q9Y2G7	Zinc finger protein 30 homolog OS=Homo sapiens GN=ZFP30 PE=2 SV=1 - [ZFP30_HUMAN]	2.89	1	1	1	1	0.829		1.926		0.00	2.89	1	1	519	61.5	8.91
Q9UI46	Dynein intermediate chain 1, axonemal OS=Homo sapiens GN=DNAI1 PE=1 SV=1 - [DNAI1_HUMAN]	2.86	1	1	1	1			1.924		2.01	2.86	1	1	699	79.2	6.87
O43155	Leucine-rich repeat transmembrane protein FLRT2 OS=Homo sapiens GN=FLRT2 PE=1 SV=1 - [FLRT2_HUMAN	6.06	1	2	2	3	1.050	0.501	1.710		0.00	6.06	2	3	660	74.0	7.80
Q6P4F1	Alpha-(1,3)-fucosyltransferase 10 OS=Homo sapiens GN=FUT10 PE=2 SV=2 - [FUT10_HUMAN]	1.46	1	1	1	1	0.514		1.684		0.00	1.46	1	1	479	56.1	8.37
Q8TDF5	Neuropilin and tolloid-like protein 1 OS=Homo sapiens GN=NETO1 PE=2 SV=2 - [NETO1_HUMAN]	5.07	1	1	1	3	0.674	1.020	1.650		7.94	5.07	1	3	533	60.2	7.02
O60296	Trafficking kinesin-binding protein 2 OS=Homo sapiens GN=TRAK2 PE=1 SV=2 - [TRAK2_HUMAN]	8.97	1	3	3	3			1.593		4.81	8.97	3	3	914	101.4	5.24
Q96L16	Putative ALMS1-like protein OS=Homo sapiens GN=ALMS1P PE=5 SV=1 - [ALM1L_HUMAN]	7.62	1	2	2	4		2.923	1.529		6.61	7.62	2	4	210	24.6	10.68

P02730	Band 3 anion transport protein OS=Homo sapiens GN=SLC4A1 PE=1 SV=3 - [B3AT_HUMAN]	1.43	1	1	1	1	1.252		1.446		0.00	1.43	1	1	911	101.7	5.19
A6NEQ2	Protein FAM181B OS=Homo sapiens GN=FAM181B PE=2 SV=1 - [F181B_HUMAN]	4.69	1	1	1	1	0.864	0.623	1.393		0.00	4.69	1	1	426	42.6	5.58
Q96FS4	Signal-induced proliferation-associated protein 1 OS=Homo sapiens GN=SIPA1 PE=1 SV=1 - [SIPA1_HUMAN]	1.34	2	1	2	6		1.097	1.350		13.53	1.34	3	6	1042	112.1	6.60
Q6J9G0	Tyrosine-protein kinase STYK1 OS=Homo sapiens GN=STYK1 PE=1 SV=4 - [STYK1_HUMAN]	4.03	1	1	1	1	1.222	1.872	1.344		2.48	4.03	1	1	422	47.5	7.40
Q8N3Z6	Zinc finger CCHC domain-containing protein 7 OS=Homo sapiens GN=ZCCHC7 PE=1 SV=2 - [ZCHC7_HUMAN]	1.66	1	1	1	1	1.685	0.743	1.300		1.62	1.66	1	1	543	63.0	7.33
Q8WXI4	Acyl-coenzyme A thioesterase 11 OS=Homo sapiens GN=ACOT11 PE=1 SV=1 - [ACO11_HUMAN]	1.32	1	1	1	1		0.913	1.225		2.25	1.32	1	1	607	68.4	8.35
P13928	Annexin A8 OS=Homo sapiens GN=ANXA8 PE=1 SV=3 - [ANXA8_HUMAN]	4.59	3	1	2	3	1.648		1.225		6.70	4.59	2	3	327	36.9	5.78
Q9GZU2	Paternally-expressed gene 3 protein OS=Homo sapiens GN=PEG3 PE=1 SV=1 - [PEG3_HUMAN]	5.23	1	4	5	7	0.984	0.866	1.213		17.15	5.23	5	7	1588	180.7	5.48
P59894	Doublecortin domain-containing protein 1 OS=Homo sapiens GN=DCDC1 PE=2 SV=2 - [DCDC1_HUMAN]	2.54	1	1	1	2	0.666	0.977	1.192		3.17	2.54	1	2	354	39.8	9.36

Q7RTU9	Stereocilin OS=Homo sapiens GN=STRC PE=2 SV=1 - [STRC_HUMAN]	3.72	1	1	3	7	1.912	1.890	1.183		8.00	3.72	4	7	1775	192.8	5.41
Q9NVM6	DnaJ homolog subfamily C member 17 OS=Homo sapiens GN=DNAJC17 PE=1 SV=1 - [DJC17_HUMAN]	4.28	1	1	1	2			1.179		4.25	4.28	1	2	304	34.7	8.53
P0C6C1	Ankyrin repeat domain-containing protein 34C OS=Homo sapiens GN=ANKRD34C PE=2 SV=2 - [AN34C_HUMAN]	4.67	1	1	2	4	1.717	0.762	1.178		9.69	4.67	2	4	535	58.2	9.03
Q9H4P4	E3 ubiquitin-protein ligase NRDP1 OS=Homo sapiens GN=RNF41 PE=1 SV=2 - [RNF41_HUMAN]	3.79	1	1	1	2			1.161		4.28	3.79	1	2	317	35.9	6.14
Q96DX4	RING finger and SPRY domain-containing protein 1 OS=Homo sapiens GN=RSPRY1 PE=2 SV=1 - [RSPRY_HUMAN]	5.56	1	1	2	3		1.053	1.155		6.29	5.56	2	3	576	64.1	5.57
Q96FX2	DPH3 homolog OS=Homo sapiens GN=DPH3 PE=1 SV=1 - [DPH3_HUMAN]	25.61	1	1	1	1	0.583	0.885	1.149		0.00	25.61	1	1	82	9.2	4.09
Q9BTC8	Metastasis-associated protein MTA3 OS=Homo sapiens GN=MTA3 PE=1 SV=2 - [MTA3_HUMAN]	10.44	1	2	6	14	1.099	1.389	1.123		25.49	10.44	10	14	594	67.5	8.57
Q9HCJ5	Zinc finger SWIM domain-containing protein 6 OS=Homo sapiens GN=ZSWIM6 PE=1 SV=2 - [ZSWM6_HUMAN]	1.07	1	1	1	1	0.973	0.860	1.098		1.79	1.07	1	1	1215	133.4	7.36

Q5TEZ5	Uncharacterized protein C6orf163 OS=Homo sapiens GN=C6orf163 PE=1 SV=2 - [CF163_HUMAN]	2.43	1	1	1	1	0.884		1.079		2.17	2.43	1	1	329	38.5	6.96
Q92608	Dedicator of cytokinesis protein 2 OS=Homo sapiens GN=DOCK2 PE=1 SV=2 - [DOCK2_HUMAN]	1.37	1	2	2	3	0.173	1.050	1.057		6.54	1.37	2	3	1830	211.8	6.87
Q9NS40	Potassium voltage-gated channel subfamily H member 7 OS=Homo sapiens GN=KCNH7 PE=2 SV=2 - [KCNH7_HUMAN]	1.34	1	1	1	1	1.548	1.049	1.041		0.00	1.34	1	1	1196	134.9	7.65
Q9NS91	E3 ubiquitin-protein ligase RAD18 OS=Homo sapiens GN=RAD18 PE=1 SV=2 - [RAD18_HUMAN]	6.06	1	1	2	2	0.942	1.363	1.017		2.36	6.06	2	2	495	56.2	7.58
Q9NQI0	Probable ATP-dependent RNA helicase DDX4 OS=Homo sapiens GN=DDX4 PE=1 SV=2 - [DDX4_HUMAN]	5.25	1	1	4	18	0.285	0.486	0.985		41.67	5.25	6	18	724	79.3	5.87
A4D1B5	Gamma-secretase-activating protein OS=Homo sapiens GN=GSAP PE=1 SV=2 - [GSAP_HUMAN]	2.34	1	1	2	16	1.232	0.908	0.981		24.88	2.34	2	16	854	97.7	6.86
Q9H095	IQ domain-containing protein G OS=Homo sapiens GN=IQCG PE=1 SV=1 - [IQCG_HUMAN]	9.93	1	4	4	9	0.463		0.979		20.11	9.93	4	9	443	51.9	6.52
Q96M32	Adenylate kinase 7 OS=Homo sapiens GN=AK7 PE=1 SV=3 - [KAD7_HUMAN]	0.83	1	1	1	1			0.975		1.78	0.83	1	1	723	82.6	4.74

Q4VNC0	Probable cation-transporting ATPase 13A5 OS=Homo sapiens GN=ATP13A5 PE=2 SV=1 - [AT135_HUMAN	1.56	1	1	1	1	0.521		0.958	0.00	1.56	1	1	1218	137.2	7.90
Q9BYB0	SH3 and multiple ankyrin repeat domains protein 3 OS=Homo sapiens GN=SHANK3 PE=1 SV=3 - [SHAN3_HUMAN	2.20	1	2	2	8		0.623	0.957	15.04	2.20	2	8	1731	184.6	8.88
Q7Z4R8	UPF0669 protein C6orf120 OS=Homo sapiens GN=C6orf120 PE=1 SV=1 - [CF120_HUMAN]	14.14	1	1	1	7	0.988	2.182	0.927	21.62	14.14	2	7	191	20.8	4.84
Q8N4S9	MARVEL domain-containing protein 2 OS=Homo sapiens GN=MARVELD2 PE=1 SV=2 - [MALD2_HUMAN]	1.79	1	1	1	1			0.924	1.68	1.79	1	1	558	64.1	7.53
P56211	cAMP-regulated phosphoprotein 19 OS=Homo sapiens GN=ARPP19 PE=1 SV=2 - [ARP19_HUMAN	42.86	1	3	4	8			0.905	25.62	42.86	6	8	112	12.3	9.09
Q68DE3	Basic helix-loop-helix domain-containing protein KIAA2018 OS=Homo sapiens GN=KIAA2018 PE=1 SV=3 - [K2018_HUMAN]	1.69	1	2	2	12		0.439	0.905	33.36	1.69	3	12	2245	241.5	7.61
Q8N1I0	Dedicator of cytokinesis protein 4 OS=Homo sapiens GN=DOCK4 PE=1 SV=3 - [DOCK4_HUMA N]	0.66	1	1	1	1	0.571	0.474	0.817	4.40	0.66	1	1	1966	225.1	7.65
Q9NRH2	SNF-related serine/threonine-protein kinase OS=Homo sapiens GN=SNRK PE=1 SV=2 - [SNRK_HUMAN]	1.96	1	1	1	1	1.066	0.689	0.783	2.44	1.96	1	1	765	84.2	7.06

Q96PV0	Ras/Rap GTPase-activating protein SynGAP OS=Homo sapiens GN=SYNGAP1 PE=1 SV=4 - [SYGP1_HUMAN]	1.41	2	1	2	3	1.988	0.203	0.759		8.84	1.41	3	3	1343	148.2	8.98
Q9H0K6	Pseudouridylate synthase 7 homolog-like protein OS=Homo sapiens GN=PUS7L PE=1 SV=1 - [PUS7L_HUMAN]	1.28	1	1	1	1			0.715		0.00	1.28	1	1	701	80.6	7.56
Q9P003	Protein cornichon homolog 4 OS=Homo sapiens GN=CNIH4 PE=1 SV=1 - [CNIH4_HUMAN]	14.39	1	1	1	2			0.713		6.58	14.39	2	2	139	16.1	6.65
P18074	TFIIH basal transcription factor complex helicase XPD subunit OS=Homo sapiens GN=ERCC2 PE=1 SV=1 - [ERCC2_HUMAN]	1.84	1	1	2	2	1.564		0.669		5.93	1.84	2	2	760	86.9	7.15
P49638	Alpha-tocopherol transfer protein OS=Homo sapiens GN=TTPA PE=1 SV=1 - [TTPA_HUMAN]	4.68	1	1	1	1	1.453		0.651		2.06	4.68	1	1	278	31.7	7.66
P05534	HLA class I histocompatibility antigen, A-24 alpha chain OS=Homo sapiens GN=HLA-A PE=1 SV=2 - [1A24_HUMAN]	44.93	2	2	20	167			0.641		405.71	44.93	32	167	365	40.7	6.34
P24522	Growth arrest and DNA damage-inducible protein GADD45 alpha OS=Homo sapiens GN=GADD45A PE=1 SV=1 - [GA45A_HUMAN]	7.27	1	1	1	1	0.903		0.619		2.29	7.27	1	1	165	18.3	4.50
Q96N68	Putative uncharacterized protein C18orf15 OS=Homo sapiens GN=C18orf15 PE=5 SV=1 - [CR015_HUMAN]	4.42	1	1	1	1	1.093	0.668	0.594		3.10	4.42	1	1	181	19.1	7.46

Q5TF21	Protein SOGA3 OS=Homo sapiens GN=SOGA3 PE=2 SV=1 - [SOGA3_HUMAN]	5.17	1	1	2	4	1.391		0.590		7.07	5.17	2	4	947	103.1	6.06
Q9H159	Cadherin-19 OS=Homo sapiens GN=CDH19 PE=2 SV=1 - [CAD19_HUMAN]	1.94	1	1	1	1	1.360	0.249	0.556		2.29	1.94	1	1	772	86.9	4.73
Q9H8M1	Coenzyme Q-binding protein COQ10 homolog B, mitochondrial OS=Homo sapiens GN=COQ10B PE=2 SV=1 - [CQ10B_HUMAN]	3.36	1	1	1	1	2.178	0.597	0.548		2.48	3.36	1	1	238	27.1	9.60
Q9NX18	Succinate dehydrogenase assembly factor 2, mitochondrial OS=Homo sapiens GN=SDHAF2 PE=1 SV=1 - [SDHF2_HUMAN	10.84	1	1	2	6	0.632	1.283	0.527		13.50	10.84	2	6	166	19.6	6.80
P11474	Steroid hormone receptor ERR1 OS=Homo sapiens GN=ESRRA PE=1 SV=3 - [ERR1_HUMAN]	4.96	1	2	2	2			0.502		3.96	4.96	2	2	423	45.5	6.38
Q12805	EGF-containing fibulin-like extracellular matrix protein 1 OS=Homo sapiens GN=EFEMP1 PE=1 SV=2 - [FBLN3_HUMAN]	1.83	1	1	1	1			0.489		2.75	1.83	1	1	493	54.6	5.07
Q9NWQ8	Phosphoprotein associated with glycosphingolipid-enriched microdomains 1 OS=Homo sapiens GN=PAG1 PE=1 SV=2 - [PHAG1_HUMAN	13.66	1	3	3	4	1.060		0.465		15.08	13.66	4	4	432	47.0	4.65
Q9BUL9	Ribonuclease P protein subunit p25 OS=Homo sapiens GN=RPP25 PE=1 SV=1 - [RPP25_HUMAN]	6.53	1	1	1	1	1.165	0.915	0.456		3.69	6.53	1	1	199	20.6	9.61
O75564	Jerky protein homolog OS=Homo sapiens GN=JRK PE=1 SV=2 - [JERKY_HUMAN]	6.35	1	1	1	1	1.086	1.196	0.446		2.53	6.35	1	1	520	57.4	8.15

Q9NWS6	Protein FAM118A OS=Homo sapiens GN=FAM118A PE=1 SV=2 - [F118A_HUMAN]	3.64	1	1	1	1	0.538	1.306	0.299		4.98	3.64	1	1	357	40.2	6.09
Q9BT92	Trichoplein keratin filament-binding protein OS=Homo sapiens GN=TCHP PE=1 SV=1 - [TCHP_HUMAN]	4.42	1	2	2	2		0.190	0.218		4.21	4.42	2	2	498	61.0	6.54
Q96QT6	PHD finger protein 12 OS=Homo sapiens GN=PHF12 PE=1 SV=2 - [PHF12_HUMAN]	2.39	1	1	2	2		0.029	0.142		3.24	2.39	2	2	1004	109.6	7.74
O75344	Inactive peptidyl-prolyl cis-trans isomerase FKBP6 OS=Homo sapiens GN=FKBP6 PE=1 SV=1 - [FKBP6_HUMAN	6.12	1	1	1	2	0.485	0.438			0.00	6.12	1	2	327	37.2	6.89
O15527	N-glycosylase/DNA lyase OS=Homo sapiens GN=OGG1 PE=1 SV=2 - [OGG1_HUMAN]	6.67	1	1	1	1	1.020	0.450			2.30	6.67	1	1	345	38.8	8.60
Q9UI42	Carboxypeptidase A4 OS=Homo sapiens GN=CPA4 PE=1 SV=2 - [CBPA4_HUMAN]	1.66	1	1	1	1		0.469			2.97	1.66	1	1	421	47.3	6.70
P32004	Neural cell adhesion molecule L1 OS=Homo sapiens GN=L1CAM PE=1 SV=2 - [L1CAM_HUMAN]	2.70	1	1	3	3	0.901	0.514			4.06	2.70	3	3	1257	139.9	6.24
Q8IYA7	Homeobox protein Mohawk OS=Homo sapiens GN=MKX PE=2 SV=2 - [MKX_HUMAN]	4.83	1	1	1	2	0.755	0.585			5.32	4.83	1	2	352	39.3	9.64
O43808	Peroxisomal membrane protein PMP34 OS=Homo sapiens GN=SLC25A17 PE=1 SV=1 - [PM34_HUMAN]	3.91	1	1	1	1	0.317	0.622			0.00	3.91	1	1	307	34.5	10.08

Q8WXE1	ATR-interacting protein OS=Homo sapiens GN=ATRIP PE=1 SV=1 - [ATRIP_HUMAN]	1.39	1	1	1	2	0.581	0.627			2.26	1.39	2	2	791	85.8	6.32
O95965	Integrin beta-like protein 1 OS=Homo sapiens GN=ITGBL1 PE=2 SV=1 - [ITGBL_HUMAN]	2.83	1	1	1	1		0.631			2.76	2.83	1	1	494	53.9	5.64
Q9NYQ3	Hydroxyacid oxidase 2 OS=Homo sapiens GN=HAO2 PE=2 SV=1 - [HAOX2_HUMAN]	6.84	1	1	1	1		0.688			0.00	6.84	1	1	351	38.8	7.64
Q6PI98	INO80 complex subunit C OS=Homo sapiens GN=INO80C PE=1 SV=1 - [IN80C_HUMAN]	9.90	1	1	1	3	0.676	0.698			8.81	9.90	1	3	192	20.6	10.02
Q9Y4F4	Protein FAM179B OS=Homo sapiens GN=FAM179B PE=1 SV=4 - [F179B_HUMAN]	0.81	1	1	1	5	0.464	0.721			11.47	0.81	1	5	1720	189.2	8.50
P0C5Y9	Histone H2A-Bbd type 1 OS=Homo sapiens GN=H2AFB1 PE=2 SV=1 - [H2AB1_HUMAN]	16.52	2	1	1	1		0.782			3.63	16.52	1	1	115	12.7	10.67
Q0VD83	Apolipoprotein B receptor OS=Homo sapiens GN=APOBR PE=1 SV=2 - [APOBR_HUMAN]	1.56	1	1	1	1	0.556	0.794			3.45	1.56	1	1	1088	114.8	4.40
Q9H1D9	DNA-directed RNA polymerase III subunit RPC6 OS=Homo sapiens GN=POLR3F PE=1 SV=1 - [RPC6_HUMAN]	4.43	1	1	1	1		0.812			3.13	4.43	1	1	316	35.7	6.11
Q8N1M1	Bestrophin-3 OS=Homo sapiens GN=BEST3 PE=2 SV=1 - [BEST3_HUMAN]	3.74	1	2	2	2		0.825			2.70	3.74	2	2	668	76.1	6.58
Q9P273	Teneurin-3 OS=Homo sapiens GN=TENM3 PE=2 SV=3 - [TEN3_HUMAN]	0.93	1	1	1	3		0.831			6.74	0.93	1	3	2699	300.8	6.42

Q07283	Trichohyalin OS=Homo sapiens GN=TCHH PE=1 SV=2 - [TRHY_HUMAN]	1.70	2	2	5	11	0.675	0.906		24.20	1.70	6	11	1943	253.8	5.78
Q14449	Growth factor receptor-bound protein 14 OS=Homo sapiens GN=GRB14 PE=1 SV=2 - [GRB14_HUMAN]	1.67	1	1	1	1	2.744	0.968		1.87	1.67	1	1	540	60.9	8.29
Q15652	Probable JmjC domain-containing histone demethylation protein 2C OS=Homo sapiens GN=JMJD1C PE=1 SV=2 - [JHD2C_HUMAN]	0.79	1	1	2	6		0.971		12.87	0.79	3	6	2540	284.3	7.87
Q495T6	Membrane metallo-endopeptidase-like 1 OS=Homo sapiens GN=MMEL1 PE=1 SV=2 - [MMEL1_HUMAN]	2.82	1	1	2	10		1.049		16.92	2.82	3	10	779	89.3	5.87
P19022	Cadherin-2 OS=Homo sapiens GN=CDH2 PE=1 SV=4 - [CADH2_HUMAN]	2.65	2	2	2	3	0.829	1.164		7.26	2.65	2	3	906	99.7	4.81
Q96QS1	Tetraspanin-32 OS=Homo sapiens GN=TSPAN32 PE=2 SV=1 - [TSN32_HUMAN]	5.00	1	1	1	1		1.229		2.29	5.00	1	1	320	34.6	8.40
Q8NC67	Neuropilin and tolloid-like protein 2 OS=Homo sapiens GN=NETO2 PE=1 SV=1 - [NETO2_HUMAN]	3.43	1	1	1	2	0.490	1.282		0.00	3.43	2	2	525	59.4	6.81
Q0VFZ6	Coiled-coil domain-containing protein 173 OS=Homo sapiens GN=CCDC173 PE=2 SV=2 - [CC173_HUMAN]	3.08	1	1	2	2	0.699	1.439		3.95	3.08	2	2	552	66.4	8.87
Q14CW9	Ataxin-7-like protein 3 OS=Homo sapiens GN=ATXN7L3 PE=1 SV=1 - [AT7L3_HUMAN]	8.36	1	1	1	1		1.453		2.05	8.36	1	1	347	38.6	7.03

Q9Y2B5	VPS9 domain-containing protein 1 OS=Homo sapiens GN=VPS9D1 PE=2 SV=2 - [VP9D1_HUMAN]	3.01	1	1	1	1	0.404	1.532		1.73	3.01	1	1	631	68.9	7.33
Q14126	Desmoglein-2 OS=Homo sapiens GN=DSG2 PE=1 SV=2 - [DSG2_HUMAN]	1.25	1	1	1	2		1.988		6.04	1.25	1	2	1118	122.2	5.24
Q9UHA7	Interleukin-36 alpha OS=Homo sapiens GN=IL36A PE=1 SV=1 - [IL36A_HUMAN]	15.19	1	1	1	1		2.389		0.00	15.19	1	1	158	17.7	6.30
Q6ZT21	Transmembrane protein with metallophosphoesterase domain OS=Homo sapiens GN=TMPPE PE=2 SV=2 - [TMPPE_HUMA	1.55	1	1	1	1	1.751			0.00	1.55	1	1	453	49.4	6.92
P06276	Cholinesterase OS=Homo sapiens GN=BCHE PE=1 SV=1 - [CHLE_HUMAN]	1.33	1	1	1	1	1.750			3.08	1.33	1	1	602	68.4	7.42
Q6ZQQ2	Spermatogenesis-associated protein 31D1 OS=Homo sapiens GN=SPATA31D1 PE=2 SV=1 - [S31D1_HUMAN]	1.59	1	1	1	1	1.262			0.00	1.59	1	1	1576	175.5	8.85
Q96M69	Leucine-rich repeat and guanylate kinase domain-containing protein OS=Homo sapiens GN=LRGUK PE=2 SV=1 - [LRGUK_HUMAN]	2.79	2	1	3	20	0.865			36.79	2.79	3	20	825	93.6	6.43
Q8N0U8	Vitamin K epoxide reductase complex subunit 1-like protein 1 OS=Homo sapiens GN=VKORC1L1 PE=1 SV=2 - [VKORL_HUMAN]	5.68	1	1	1	1	0.839			3.78	5.68	1	1	176	19.8	9.13

Q86US8	Telomerase-binding protein EST1A OS=Homo sapiens GN=SMG6 PE=1 SV=2 - [EST1A_HUMAN]	1.48	1	1	2	2	0.835		5.17	1.48	2	2	1419	160.4	7.05
Q14565	Meiotic recombination protein DMC1/LIM15 homolog OS=Homo sapiens GN=DMC1 PE=1 SV=2 - [DMC1_HUMAN]	8.82	1	1	1	1	0.814		2.07	8.82	1	1	340	37.7	5.88
Q8N998	Coiled-coil domain-containing protein 89 OS=Homo sapiens GN=CCDC89 PE=2 SV=1 - [CCD89_HUMAN]	5.61	1	2	2	2	0.811		5.13	5.61	2	2	374	43.8	5.36
A6NN14	Zinc finger protein 729 OS=Homo sapiens GN=ZNF729 PE=2 SV=4 - [ZN729_HUMAN]	3.27	1	1	2	4	0.761		7.86	3.27	3	4	1252	144.9	9.50
O15090	Zinc finger protein 536 OS=Homo sapiens GN=ZNF536 PE=1 SV=3 - [ZN536_HUMAN]	1.85	94	1	2	4	0.731		0.00	1.85	2	4	1300	141.3	7.27
Q5VW38	Protein GPR107 OS=Homo sapiens GN=GPR107 PE=1 SV=1 - [GP107_HUMAN]	3.00	1	2	2	5	0.708		13.31	3.00	2	5	600	66.9	7.24
Q9NZI6	Transcription factor CP2-like protein 1 OS=Homo sapiens GN=TFCP2L1 PE=2 SV=1 - [TF2L1_HUMAN]	2.51	1	1	1	1	0.691		2.01	2.51	1	1	479	54.6	7.01
P17081	Rho-related GTP-binding protein RhoQ OS=Homo sapiens GN=RHOQ PE=1 SV=2 - [RHOQ_HUMAN]	12.20	2	1	2	15	0.665		40.98	12.20	3	15	205	22.6	6.32
Q6P2H3	Centrosomal protein of 85 kDa OS=Homo sapiens GN=CEP85 PE=1 SV=1 - [CEP85_HUMAN]	3.41	1	2	3	4	0.575		4.77	3.41	4	4	762	85.6	6.00

Q6NXR4	TELO2-interacting protein 2 OS=Homo sapiens GN=TTI2 PE=1 SV=1 - [TTI2_HUMAN]	5.31	1	2	2	3	0.551		12.14	5.31	3	3	508	56.9	7.09
Q96M91	Cilia- and flagella-associated protein 53 OS=Homo sapiens GN=CFAP53 PE=1 SV=2 - [CFA53_HUMAN	2.92	1	1	1	1	0.508		2.09	2.92	1	1	514	61.8	8.90
O00763	Acetyl-CoA carboxylase 2 OS=Homo sapiens GN=ACACB PE=1 SV=3 - [ACACB_HUMAN]	1.42	1	1	3	4	0.502		8.64	1.42	3	4	2458	276.4	6.49
Q96AT1	Uncharacterized protein KIAA1143 OS=Homo sapiens GN=KIAA1143 PE=1 SV=2 - [K1143_HUMAN]	8.44	1	1	1	1	0.388		3.10	8.44	1	1	154	17.5	6.11
Q96I51	Williams-Beuren syndrome chromosomal region 16 protein OS=Homo sapiens GN=WBSCR16 PE=1 SV=2 - [WBS16_HUMA	2.59	1	1	1	3	0.354		7.98	2.59	2	3	464	50.0	8.40
